# DNA Barcode Reference Library for European Ants: A Roadmap for Phylogeography and Species Discovery

**DOI:** 10.1111/1755-0998.70135

**Published:** 2026-04-20

**Authors:** Mattia Menchetti, Enrico Schifani, Fede García, Sämi Schär, Elisabetta Sbrega, Nikola Balević, Bonnie B. Blaimer, Lech Borowiec, Dario Cioppa, Cecilia Corbella, Vlad Dincă, Vincenzo Gentile, Irakleitos Giotis, Kiko Gómez, José María Gómez Durán, Konstantinos Kalaentzis, Albena Lapeva‐Gjonova, Emiliano Mori, Gerard Talavera, Alberto Tinaut, Francisca Ruano, Sebastian Salata, Eduardo Sequeira, Maria Serracanta, Tomasz Suchan, Paul D. N. Hebert, Leonardo Dapporto, Roger Vila

**Affiliations:** ^1^ Institut de Biologia Evolutiva (CSIC‐Univ. Pompeu Fabra) Barcelona Spain; ^2^ Center for Integrative Biodiversity Discovery Museum für Naturkunde, Leibniz Institute for Evolution and Biodiversity Science Berlin Germany; ^3^ Barcelona Spain; ^4^ Dietikon, Zürich Switzerland; ^5^ Institut Botànic de Barcelona (IBB), CSIC‐CMCNB Barcelona Catalonia Spain; ^6^ Department of Biology University of Montenegro Podgorica Montenegro; ^7^ Department of Biodiversity and Evolutionary Taxonomy, Myrmecological Laboratory University of Wroclaw Wroclaw Poland; ^8^ Department of Biological, Geological and Environmental Sciences University of Bologna Bologna Italy; ^9^ ‘Grigore Antipa’ National Museum of Natural History Bucharest Romania; ^10^ Ecology and Genetics Research Unit University of Oulu Oulu Finland; ^11^ Naples Italy; ^12^ Divison of Bioscience University College London UK; ^13^ Garraf, Barcelona Spain; ^14^ Madrid Spain; ^15^ Department of Genetics, Development & Molecular Biology, School of Biology, Faculty of Science Aristotle University of Thessaloniki Thessaloniki Greece; ^16^ Hydrobiological Station of Rhodes, Hellenic Centre for Marine Research Rhodes Greece; ^17^ Department of Zoology and Anthropology, Faculty of Biology Sofia University Sofia Bulgaria; ^18^ Consiglio Nazionale Delle Ricerche Istituto di Ricerca Sugli Ecosistemi Terrestri Sesto Fiorentino Italy; ^19^ National Biodiversity Future Center Palermo Italy; ^20^ Department of Zoology University of Granada Granada Spain; ^21^ Peral, Lisbon Portugal; ^22^ Facultat de Biociències Universitat Autònoma de Barcelona Barcelona Spain; ^23^ Institute of Geography and Spatial Organization, Polish Academy of Sciences Warsaw Poland; ^24^ Centre for Biodiversity Genomics University of Guelph Guelph Canada; ^25^ ZEN Lab, Department of Biology University of Florence Florence Italy

**Keywords:** checklist, distribution, DNA barcoding, Formicidae, Hymenoptera, non‐native species, taxonomy

## Abstract

DNA barcode reference libraries provide useful tools for specimen identification, highlighting potential new species and detecting introduced ones. Here, we present a comprehensive DNA barcode library for European ants and, in order to tackle the Linnean, Wallacean and Darwinian shortfalls of this group, we provide an updated checklist, distribution data, mitochondrial genetic diversity maps and mitochondrial gene trees. The European ant fauna is here established to include 55 genera and 650 species (587 of which are native), including one species newly recorded for Europe and novel citations for 26 species from 11 countries. Our genetic dataset includes 6530 georeferenced *COI* sequences (62.1% *d* *e novo*) for 506 species (77.8%) across all genera. On average, 12.9 sequences were obtained per species, and 209 species were sequenced for the first time. We generated intra‐ and interspecific genetic distance estimates, 52 genus‐level trees, mitochondrial genetic diversity and specimen maps for 384 species, as well as haplotype networks for 289 species, available in the Atlas V1.0 ‘The Mitochondrial Genetic Diversity Maps of European Ants’. We estimate that 56.3% of European ants are monophyletic with respect to the *COI* gene and can be unambiguously identified by DNA barcoding, though performance varies widely among genera. We observed moderate levels of barcode sharing (19.3%) and of barcode gap presence (47.6%), as well as high levels of intraspecific divergences (up to 17.9%). These findings likely reflect both biological and operational factors and highlight the existence of potential cryptic taxa and the need for taxonomic revisions. The framework presented here aims to facilitate future research, species discovery and conservation of European ants.

## 1 Introduction

A remarkable prevalence of previously unnoticed cryptic species has been revealed in the last decades across many taxonomic groups (Hending 2025), frequently first highlighted by DNA barcoding and later confirmed by integrative taxonomy or multilocus analyses (e.g., Hernández‐Roldán et al. 2016; Garcia‐Porta et al. 2012; Schär et al. 2022). Thus, the discovery of dark diversity and the possibility of updating taxonomy are substantially aided by the development of comprehensive, well‐curated DNA barcode libraries. A complete and stable taxonomic framework is also fundamental for research in a variety of fields, from ecological assessments and macroecological studies to conservation and biosecurity. Moreover, the increasing application of metabarcoding techniques, as well as macroecological studies, depends on the development of comprehensive and curated sequence libraries (Deiner et al. 2017).

Taxon‐wide genetic databases have been produced for a range of organisms in Europe, including butterflies, beetles and true bugs (Hendrich et al. 2015; Dincă et al. 2021; Csabai et al. 2023). The high number of published DNA barcodes can also be used in macrogenetics to study intraspecific variation across space and can be linked to species traits, geological processes and climatic processes (Leigh et al. 2021; Dapporto et al. 2024). For instance, atlases of intraspecific genetic variability have been generated for the butterflies of the Western‐Palaearctic (Dapporto et al. 2022) and for continental Canada and the United States (D'Ercole et al. 2024).

Ants (Hymenoptera: Formicidae) are among the most successful and diverse animal groups (Hölldobler and Wilson 1990), with more than 14,000 species globally described (Bolton 2025) and play a fundamental role in most terrestrial ecosystems (Oberski et al. 2025). While they exhibit the highest biomass levels in equatorial forests, they have a strong ecological impact in temperate forests, agricultural lands and urban environments (Parker and Kronauer 2021). Easily spread by humans, many introduced ant species are notorious for their negative effects on the environment, economy or human health (Wong et al. 2023).

The European ant fauna comprises over 600 species (Janicki et al. 2016), a number that continues to grow each year as new species are discovered and alien species are accidentally introduced. Despite the existence of a checklist for Central and Northern Europe (Seifert 2018), others that also extend to the Mediterranean Basin and adjacent regions (Borowiec 2014; Wang et al. 2023) and multiple country‐level lists (e.g., Bulgaria, Lapeva‐Gjonova and Antonova 2022; Hungary, Csősz et al. 2021; Italy, Schifani 2022; Poland, Czechowski et al. 2012), no review offering a comprehensive perspective for Europe exists. The state of knowledge of European ants varies depending on the genera and species groups, but three shortfalls generally apply: Linnean (taxonomy), Wallacean (distribution patterns) and Darwinian (evolutionary processes). While some groups have been moderately studied with integrative tools (e.g., *Temnothorax*, Csősz et al. 2015; *Messor*, Steiner et al. 2018), the taxonomy of others has not been revised for decades (e.g., *Lepisiota*). This has created an imbalanced situation in which a few groups have been studied using genomic techniques, while for many others, little or no molecular data is available (e.g., *Stigmatomma*). Moreover, cryptic species are widespread in several genera, with rates reaching up to 52% in the case of *Lasius s. str*. or 72% in the *Tetramorium caespitum* group of species (Seifert 2009).

Here we present a DNA barcode library of European ants to tackle the three shortfalls and provide: (a) an updated checklist; (b) updated distribution ranges and mitochondrial genetic diversity maps; (c) mitochondrial gene trees. This is the first curated sequence library for a major taxonomic lineage of Hymenoptera on a continental scale.

## 2 Materials and Methods

The study area included all European countries (Supporting Information 1, Figure S1), excluding Russia, Türkiye, Cyprus and the Macaronesian islands for biogeographic reasons (i.e., faunas strongly influenced either by Asia or African elements). Territories under the jurisdiction of European countries but located in other continents (e.g., Spanish territories in North Africa) were not included.

We compiled a checklist for the European ant fauna using current literature and databases (e.g., AntCat, Bolton 2025 and AntMaps, Janicki et al. 2016) as of December 31st, 2025. All the authors revised the species list obtained.

### 2.1 Specimen Processing

Specimens were selected for cytochrome *c* oxidase subunit I (hereafter, *COI*) sequencing (DNA barcode region, 658 bp) from private and public collections and new field expeditions. Whenever possible, specimens were selected to maximize distributional and habitat coverage for each species within the study area. For example, by selecting specimens from both mountain and lowland areas, as well as islands. In a few exceptional cases, specimens or sequences from outside the study area were included: (1) when specimens were not available from the study area; (2) if the native taxon was described from outside the study area; and (3) in the case of alien species.

The material was morphologically identified by the authors using stereoscopic microscopes with up to 180× magnification (see author contributions and Supporting Information 3). For most of the European territory we used the keys published by Seifert (2018), while for the more biodiverse southern European peninsulas we followed other genus revisions (e.g., Rigato 1999; Seifert 2023; Seifert et al. 2024; Csősz, Taheri, et al. 2025), groups of species revisions (e.g., Csősz et al. 2015; Seifert 2023), regional lists including keys (e.g., Arcos and García 2023; Borowiec and Salata 2022), species redescriptions (e.g., Gusten et al. 2006) and descriptions of new species including keys (e.g., García et al. 2024). When possible, specimens were examined by multiple co‐authors.

We also annotated whether specimens were part of a type series and, whenever possible, if they were collected from *terra typica* (countries, first‐level administrative division or island, depending on the information available) or type locality (within a 20 km radius of the known toponym). Additionally, we indicated whether each record represented a faunistic novelty at the national or regional level.

We obtained *COI* sequences (between 300 and 658 bp of the DNA barcode region) by combining several sources. The de novo sequencing was carried out in multiple institutions and following different protocols. The majority of the samples were sequenced on Sequel II (PacBio) at the Canadian Centre for Biodiversity Genomics, University of Guelph, Canada. Other sequences were generated at the Butterfly Diversity and Evolution Lab (BDEL, IBE), following the protocol by Schär et al. (2020) and using the primers LCO1490/HCO2198 (Vrijenhoek 1994). PCR products from those samples were visualized by gel electrophoresis and sent to Macrogen Europe for Sanger sequencing. Raw sequences were edited and aligned in Geneious Prime 2020.2.4 (Kearse et al. 2012). Electropherograms and sequences were inspected for the presence of double peaks and stop codons, respectively, and sequences showing either feature were excluded from subsequent analyses. For a limited number of specimens, in order to extract barcodes and support related research projects, genome skimming and ultraconserved elements (UCE) data were obtained following the protocols by Branstetter et al. (2017) and sent for sequencing at Rapid Genomics (California). DNA barcodes were then extracted using the PHYLUCE software (Faircloth 2016).

Additionally, we included sequences from previously published studies, retrieved from the databases BOLD (www.boldsystems.org), GenBank (www.ncbi.nlm.nih.gov/genbank) and SRA (www.ncbi.nlm.nih.gov/sra). Only sequences overlapping with the *COI* barcode region and ≥ 300 bp were kept. Together with the sequence data, we also retrieved the available metadata. We converted all coordinates to the World‐Geodetic‐System 1984 (WGS84) and stored them in decimal degrees. When coordinates were not available, if the toponym was given, we obtained the approximate coordinates at a resolution of 0.1°, latitude and longitude. When no information besides the country was available, we kept the sequences without coordinates, and we did not plot the location.

Genomic data from the SRA database were used to extract *COI* sequences using Geneious Prime 2020.2.4 (for whole genomes) and the PHYLUCE software for UCE datasets (Faircloth 2016).

To organize the data for the following analyses and tree generation, sequences were stored in different fasta files partitioned by genus. In cases where previous studies had shown that certain genera were not monophyletic, they were combined and treated as single taxonomic units (i.e., *Aphaenogaster* and *Messor*, *Goniomma* and *Oxyopomyrmex* [Branstetter et al. 2022] and *Strongylognathus* and *Tetramorium* [Ward et al. 2015]). All fasta files were then aligned using MUSCLE in Geneious Prime 2020.2.4. The final dataset was handled using RStudio Version 1.4.1103. Supporting Information 1a and 1b were assembled using a custom script made on R markdown (see Dapporto et al. 2022). For each species, we calculated the uncorrected pairwise genetic distances by using the ‘dist.dna’ function of the *APE* R package (Paradis et al. 2004) using all genetic data available from the study area and surrounding regions (hereafter extended study area; illustrated in SI 1, Figure S1). More specifically, for species with at least three sequences ≥ 600 bp, we calculated the maximum and median intraspecific *p*‐distances. For species with at least one sequence ≥ 600 bp in the extended study area and belonging to genera with more than one species, we calculated the minimum interspecific *p*‐distance and the median of the minimum interspecific *p*‐distances of all the sequences of that species, using congeneric sequences (monotypic genera were not analysed). In addition, we calculated the maximum intraspecific *p*‐distances including samples identified as cf. and with more than one sequence ≥ 300 bp. The number of species presenting a DNA barcode gap was assessed by calculating those species having maximum intraspecific *p*‐distances lower than minimum interspecific *p*‐distances. We also inspected and counted the number of cases involved in DNA barcode sharing (i.e., the same *COI* haplotype is found in multiple species), by counting the number of species with minimum interspecific *p*‐distance equal to zero.

### 2.2 Trees

Trees for each genus—or genus group in cases where multiple genera were treated as a single taxonomic unit—are presented as part of the Atlas included in Supporting Information 1 (pages 42–93). To construct them, we included a minimum of two outgroup taxa (SI 1 Table S1). We inferred maximum likelihood (ML) trees using IQ‐TREE 2.0.3 (Minh et al. 2020) with ultrafast bootstrap (1000 replicates), applying the model selected with ModelFinder (Kalyaanamoorthy et al. 2017) (Supporting Information 2, Table S1). These trees were pruned to retain only sequences identified at the species level in order to be inspected for *COI* monophyly with the program Monophylizer (Mutanen et al. 2016) and the number of monophyletic lineages was quantified with a custom script (provided in the Supporting Information). Single sequences from monotypic genera were considered monophyletic; in contrast, single sequences from non‐monotypic genera were categorized as singletons. Trees were plotted with the *ggtree* R package (Yu et al. 2017) (Figure 2a,c and SI 1, pages 42–93). Sequence labels contain the sequence name (voucher code for *de novo* sequences and GenBank accession number, BOLD process ID or SRA code for retrieved ones), scientific name, geographic provenance (island, if applicable and country) and indication if the sequence was retrieved from previously published datasets. The following information was also added to each ML tree (SI 1, Figure S2): (1) ultrafast bootstrap support (ufBS); (2) colour‐coded European regions; (3) specimen information (*terra typica*, type locality and type specimen); (4) sequence length (bp).

### 2.3 Mitochondrial Genetic Diversity Maps

All maps are presented as part of the Atlas included in Supporting Information 1.

For each species, we generated two maps: one showing its mitochondrial intraspecific genetic variation (hereafter, mitochondrial genetic diversity map) and one with information on the source of each sequence and specimen (hereafter, specimen map). Only data from specimens identified at the species level or as ‘cf.’ were included in these maps. Example figures on how these were generated are in Supporting Information 1 (Figures S3 and S4).

Mitochondrial genetic diversity maps displaying the samples originating from the extended study area (Europe and surrounding areas, SI 1, Figure S1) were generated using the *ggplot2* R package (Wickham et al. 2016) and the scripts available in the *iodatabase* R package (Dapporto et al. 2022). For each species, the p‐distance matrix was subjected to a principal coordinates analysis (PCoA) to obtain a two‐dimensional configuration that was then overlaid on a square with blue, green, yellow and red at the corners, and all shades in‐between. This allowed us to associate the genetic similarities among haplotypes with colour similarity. The visualization of the spatial distribution of genetic differentiation was obtained by plotting specimen records on the mitochondrial genetic diversity maps by grouping them by area squares of the same size, using a variable cell size proportional to the plotted area, while islands were kept separated. Pie sectors (specimens) in the mitochondrial genetic diversity maps were coloured according to their haplotype location within the PCoA configuration, when at least three sequences were available and if the maximum intraspecific genetic *p*‐distance was above zero. Otherwise, pies were coloured in grey. The maximum intraspecific distance (including cf. sequences) was calculated and shown on the mitochondrial genetic diversity maps whenever more than one sequence ≥ 300 bp was available from the extended study area. The number of sequences and the maximum intraspecific distances obtained under a stricter criterion (> 2 identified sequences and ≥ 600 bp) and under a less strict criterion (> 1 cf. sequence and ≥ 300 bp) are provided in the caption of each map.

Haplotype networks were also produced for each species when at least three sequences ≥ 600 bp were available and if at least three haplotypes were found. Haplotype networks were calculated with the randomized minimum spanning tree algorithm (Paradis 2018) using the ‘rmst’ function of the *pegas* R package (Paradis 2010). The mutations among haplotypes were indicated using numbers over connections, and the same colours obtained after the PCoA were assigned to the haplotypes.

Supporting Information 1b presents the same information as Supporting Information 1a, but uses a different palette, tested for perception by people with the most common types of colour blindness (see also Dapporto et al. 2022).

For the specimen maps, we added elevation in the plotted area and the main information regarding the sequences: (1) de novo sequence or retrieved; (2) identified or cf.; (3) type locality, *terra typica*, type series or faunistic novelty.

## 3 Results

### 3.1 Distribution Data

We produced a new checklist of the European ant fauna containing 650 species, 587 of which are native, 60 non‐native (9.2%) and 3 (0.5%) of uncertain status (SI 1, Table S2). Our genetic analysis highlighted and morphological analysis confirmed, the presence of a North African species reported for the first time in Europe, namely *Camponotus spissinodis*, found in Sicily (Italy) and southern Spain.

A total of 26 species (belonging to 15 genera) are reported for the first time from 11 countries (Table 1). The collection data of the vouchers for these new faunistic records is provided in Supporting Information 1 (specimen maps) and Supporting Information 3 (raw data as .txt file), while full information about the identification is available in Supporting Information 4. The gathered data also extended the known distribution of many species. The most notable ones (i.e., new for islands or large areas), which are also highlighted in Supporting Information 1, plotted with diamonds in the maps, include: *Aphaenogaster picena* (new for Sicily), *Camponotus sanctus* (new for Samos), *Messor erwini* (new for Ibiza), *Monomorium monomorium* (new for Sicily), *Plagiolepis grassei* (new for the Italian peninsula) and *Solenopsis fugax* (new for Sicily).

**TABLE 1:** Species recorded for the first time at the country level.

1st record for	Newly recorded species
Albania	*Aphaenogaster illyrica*, *Bothriomyrmex corsicus* , *Leptothorax acervorum* , *Messor ibericus* , *Myrmica schencki* , *Pheidole balcanica*, *Temnothorax nadigi*
Bulgaria	*Lasius precursor*, *Plagiolepis grassei* , *Tetramorium kephalosi*
Croatia	*Cataglyphis italica*
Denmark	*Solenopsis abdita* ^‡^
Greece	*Tapinoma insularis*
Italy	*Camponotus oertzeni* , *Camponotus spissinodis* ^†^, *Lasius bombycina*, *Lasius illyricus*, *Messor structor* , *Solenopsis juliae*
Malta	*Solenopsis lusitanica* , *Tapinoma magnum*, *Tetramorium immigrans*
Montenegro	*Lasius psammophilus* , *Hypoponera punctatissima* ^†^
Portugal	*Hypoponera ergatandria* ^‡^
Romania	*Lasius bombycina*, *Tapinoma glabrella*
Spain	*Camponotus spissinodis* ^†^

*Note:* When the record represents the first for Europe it is marked by †, while introduced non‐native species are indicated by ‡.

### 3.2 Genetic Data

**FIGURE 1:**
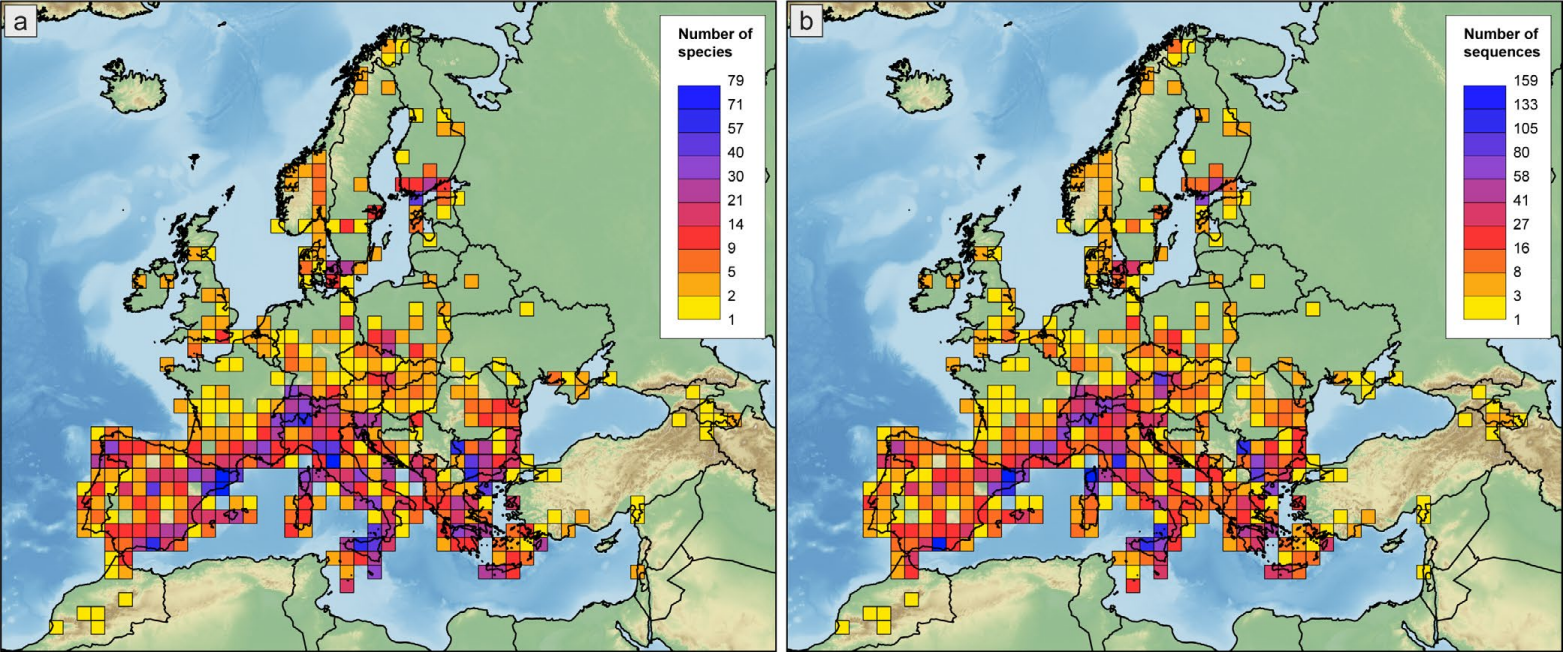
Number of species (a) and *COI* sequences (b) for each 100 × 100 km cell. Southern peninsulas were heavily sampled since they harbour the highest species diversity. Sequences from outside the study area (SI 1, Figure S1) were included only in a few exceptional cases (i.e., when they were not available from the study area, if the native taxon was described from outside the study area and in the case of non‐native species). A small number of sequences belonging to non‐native species from outside the area shown is not represented in the map.

**FIGURE 2:**
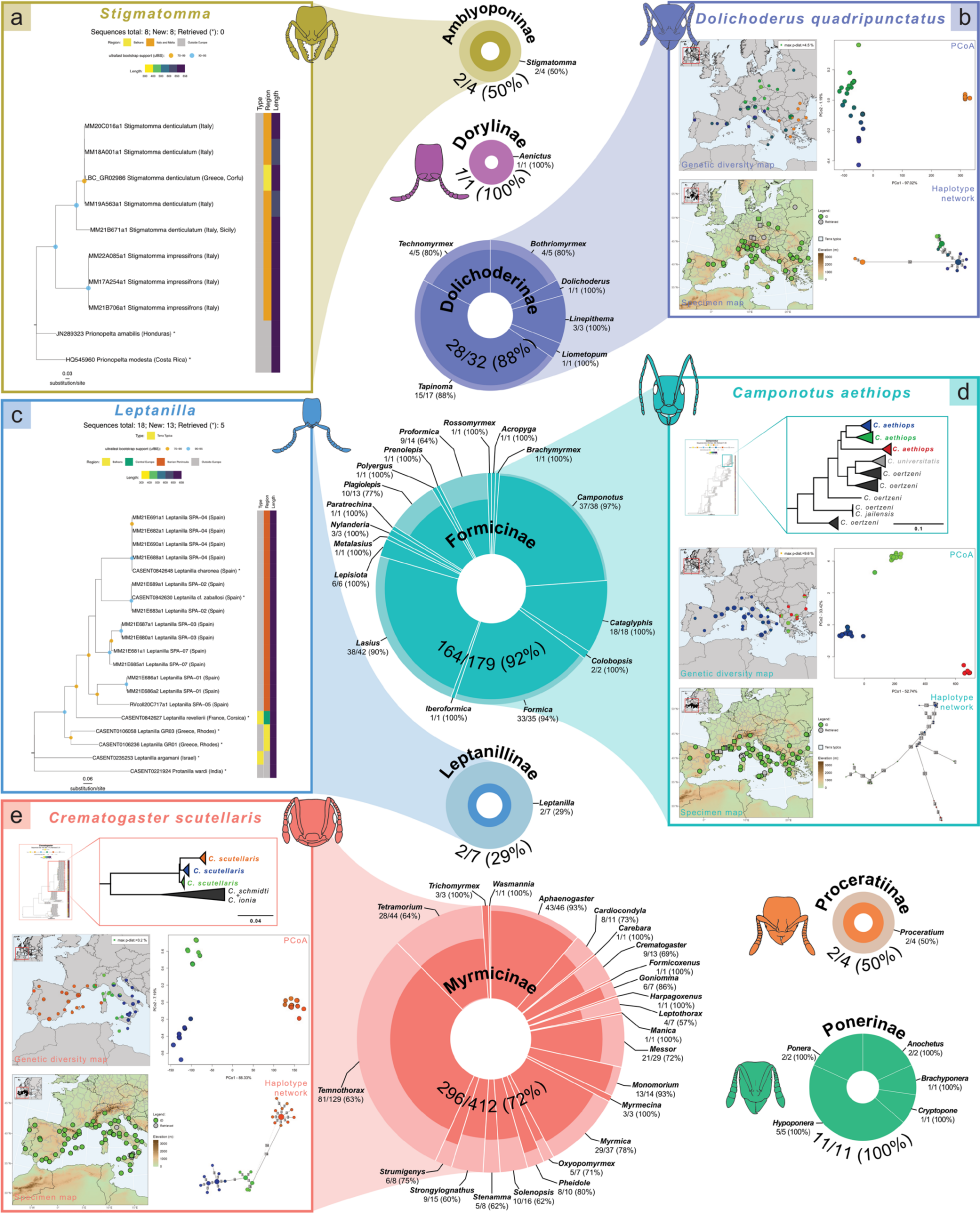
Graphical summary of the taxonomic coverage of the genetic dataset and a selection of results including genus‐level trees (a, c) and mitochondrial genetic diversity maps (b,d,e). Supporting Information 1 includes all 52 trees (pages 42–93) and the maps for 494 species (pages 95–585). Each donut pie corresponds to a subfamily with genus slices proportional to the number of species belonging to that genus. For each slice, the width of the darker areas is proportional to the percentage of species sequenced. Number of described species sequenced, total number of described species and the percentage they represent are indicated at the subfamily and genus level. *COI* trees for the genera *Stigmatomma* (a) and *Leptanilla* (c). Mitochondrial genetic diversity maps of *Dolichoderus quadripunctatus* (b), *Camponotus aethiops* (d) and *Crematogaster scutellaris* (e), with simplified representations of the relationship with the closely related species of the two latter species.

The present DNA barcode library includes records for 506 morphologically determined species, 78% of the European ant species (Figures 1 and 2). It includes a total of 6530 georeferenced sequences with lengths ≥ 300 bp (12.9 sequences per species on average), 5812 of which have ≥ 600 bp (88.6% of the total). From these sequences, 4058 are new (62.1%), while 2472 sequences (37.9%) were retrieved from GenBank (2200 sequences, 33.7%), BOLD (190 sequences, 2.9%) and the SRA database (82 sequences, 1.3%). 95% of specimens sequenced were morphologically identified at the species level (6203 sequences), 4.5% could not be confidently attributed to a species (243 sequences assigned as ‘cf.’ and 54 as ‘group/complex of species’) and 0.5% (31 sequences) were only identified to the genus level. Voucher information (Specimen Code) and GenBank, BOLD and SRA accession numbers are available in the Supporting Information 3 and in the BOLD project ANTEU. The dataset covers all 55 ant genera present in Europe and for 28 of those (50.9%) all European species now have *COI* sequences available (Figure 2). We generated de novo sequences for 452 species (69.5% of the European fauna), and *COI* sequences were provided for the first time for 209 species (32.2%) (SI 1, Table S3). Sequences were retrieved for 297 species (45.7%), of which 30 species (4.6%) were only available as genomic data, from which the *COI* sequence was extracted (SI 1, Table S3).

The full dataset can be found in Supporting Information 1, as an Atlas entitled ‘The Mitochondrial Genetic Diversity Maps of European Ants’. Based on the mitochondrial *COI* data obtained, we generated a total of 52 genus‐level trees (one for each genus or genus group, in cases where multiple genera were treated as a single taxonomic unit) (SI 1, pages 42–93), mitochondrial genetic diversity maps for 384 species, PCoA plots for 328 species, specimen maps for 493 species and haplotype networks for 289 species (SI 1, pages 95–585). Figure 2 provides examples of these trees for the genera *Stigmatomma* (Figure 2a) and *Leptanilla* (Figure 2c), as well as mitochondrial genetic diversity maps, specimen maps, PCoA graphs and haplotype networks for the species *Dolichoderus quadripunctatus* (Figure 2b), *Camponotus aethiops* (Figure 2d) and *Crematogaster scutellaris* (Figure 2e).

We provided and highlighted sequences from *terra typica* in both the genus‐level trees and specimen maps, representing 139 species (530 sequences in total). Among these, 52 species were sampled directly from their type localities (115 sequences). Notably, 31 of these sequences correspond to type specimens from 22 species, including 20 paratypes, 2 holotypes and 2 neotypes.

**FIGURE 3:**
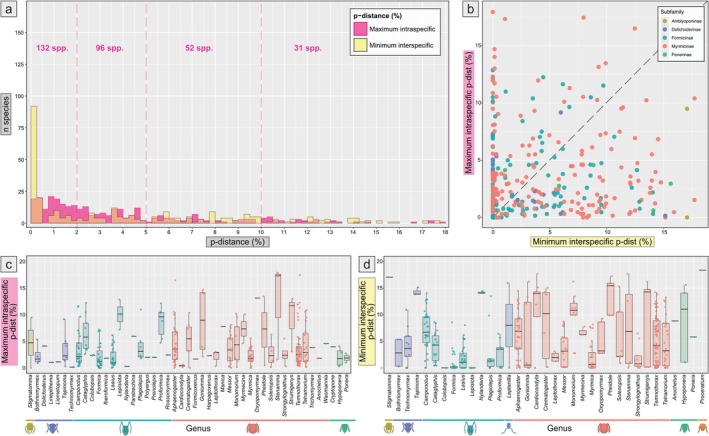
Summary of the maximum intra‐ and minimum interspecific pairwise genetic distances (*p*‐dist) for congeneric sequences. (a) Frequency of pairwise distances (in % substitutions per site, grouped in ranges of 0.25%) illustrating the distribution of maximum intraspecific (magenta) and minimum interspecific (yellow) values among species (311 spp.). The orange portion of the bars highlights the range where intraspecific and interspecific distances overlap. Vertical dashed lines delimit the thresholds of intraspecific *p*‐dist of 2% (conservative estimate of intraspecific variation), 5% (general cutoff for distinguishing between intra‐ and interspecific variation) and 10% (high level of intraspecific variation), while the numbers on top indicate the number of species falling within each cutoff, 132, 96, 52 and 31 species, respectively. (b) Minimum interspecific *p*‐distance versus maximum intraspecific p‐distance for each species (311 spp.), coloured by subfamily. The dashed line indicates the ratio of intraspecific/interspecific distances = 1. Species above this line show no barcode gap (163 spp.). (c) Maximum intraspecific *p*‐dist (%) values in each genus (species represented by a single sequence excluded; 325 spp.), subfamilies are highlighted at the bottom. (d) Minimum interspecific p‐dist (%) values in each genus (monotypic genera excluded; 461 spp.), subfamilies are highlighted at the bottom.

For 325 species (> = 3 sequences ≥ 600 bp from the extended study area), we calculated the maximum intraspecific p‐distance (%) (3.8 mean ± 3.7 SD; 0–17.9 min–max) (Figure 3a,c, SI 2, Figures S2 and S3) and the median intraspecific *p*‐distance (%) (1.9 ± 2.6; 0–17) (SI 2, Figures S1–S3 and S5). For 385, we calculated the max intraspecific *p*‐distance including identification at cf. level and with more than one sequence available (%) (3.8 mean ± 4 SD; 0–24.2 min−max) (SI 2, Figures S3 and S4). For 461 species (≥ 1 sequence ≥ 600 bp from the extended study area and > 1 species per genus) we calculated the minimum interspecific *p*‐distance (%) (5 ± 5; 0–21.7) (Figure 3a,d, SI 2, Figures S2 and S3) and the median of the minimum interspecific *p*‐distances (%) (7 ± 5.6; 0–22.4) (SI 2, Figures S1–S3, S6). Barcode sharing (minimum interspecific *p*‐distance = 0) was found for 89 species (SI 1, Table S3), which corresponds to 19.3% of the 461 species examined. A DNA barcode gap was found in 148 species (47.6% of 311 spp., excluding monotypic genera, Figure 3b).

**FIGURE 4:**
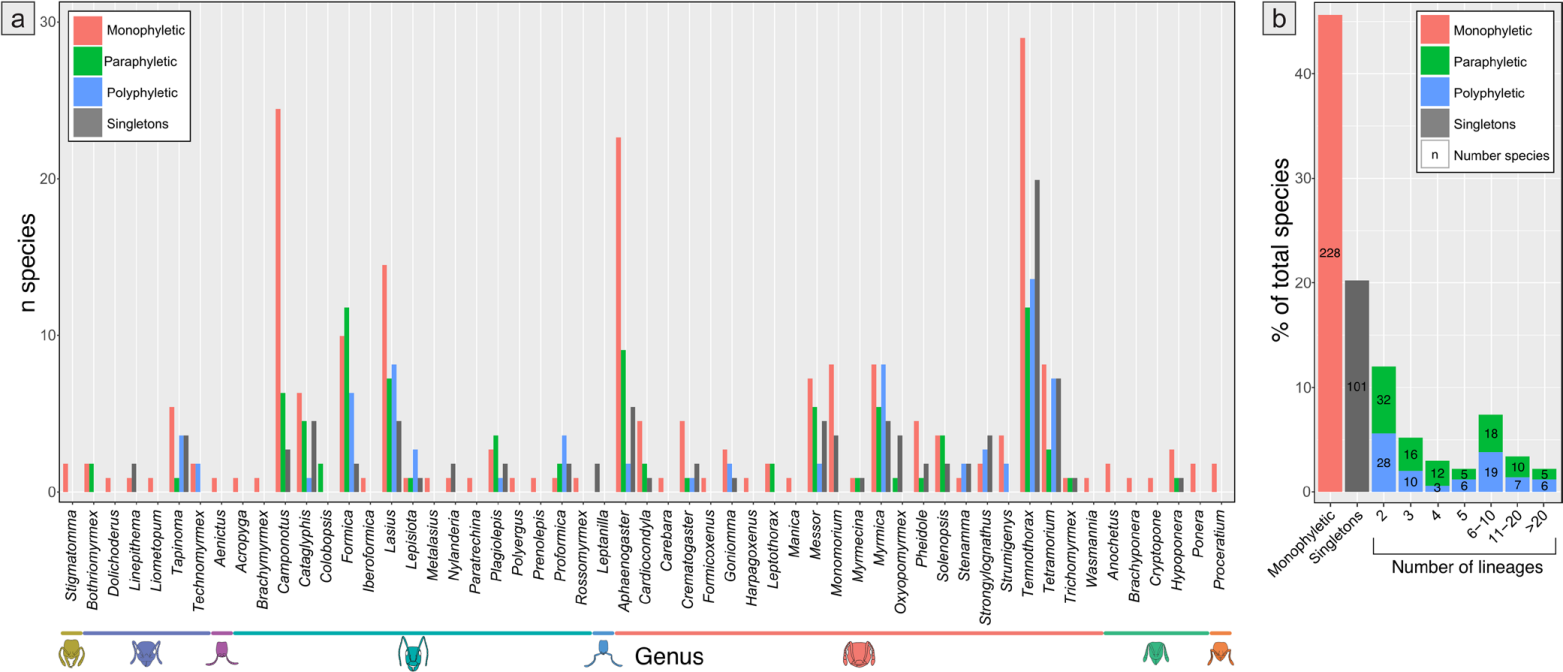
(a) Number of species retrieved as monophyletic (red), paraphyletic (green), polyphyletic (blue) and singletons (grey) in each genus. Horizontal lines at the bottom indicate the subfamilies present in Europe. (b) Proportion of species retrieved as monophyletic, singleton, or non‐monophyletic across lineage‐number bins, with species counts shown inside the bars (*n* = 506 spp.).

Among the 506 species, 101 (20%) were represented by a single sequence (singletons; Figure 4b). Of the remaining 405 species, 228 (56.3%) were retrieved as monophyletic, and 177 (43.7%) were retrieved as non‐monophyletic, out of which 98 (24.2%) were paraphyletic and 79 (19.5%) were polyphyletic (Figure 4b).

## 4 Discussion

### 4.1 The European Ant Fauna

Species checklists are fundamental tools for studying and preserving biodiversity (Droege et al. 1998). The updated checklist of European ants presented here, along with the new faunistic data linked to georeferenced sequences, provides a solid foundation for future research and refining species range delineations.

We report 650 species from Europe, of which 9.2% are introduced. Despite centuries of taxonomic work across the continent, ongoing discoveries and the continuous description or synonymization of new species underscore how far we are from a comprehensive understanding of the diversity and distribution of European ants. Much of the remaining taxonomic work involves cryptic species, where molecular evidence has often provided the first indication of distinct lineages, subsequently validated through morphometrics (Wagner 2017; Menchetti et al. 2023). Molecular tools have also played an important role in higher‐level taxonomy, such as in the case of the recently described genus *Metalasius*, formerly included within *Lasius* (Boudinot et al. 2022).

Species of uncertain taxonomic status, known only from single specimens (e.g., *Crematogaster fuentei* ), were retained in the checklist. We encourage future studies to prioritize these poorly known taxa, along with the highly diverged lineages identified in our mitochondrial genetic maps and phylogenetic trees.

Our extensive field sampling effort, combined with integrative morphological and DNA analysis, allowed us to refine known distributions and uncover new national records—particularly for taxa that are morphologically cryptic or difficult to identify. Notably, we report the presence of *Camponotus spissinodis*, previously considered absent from Europe (Seifert 2019), underscoring the value of intensified surveys along the continent's southern margins. In several cases, we significantly expanded known ranges of species previously thought to have more restricted distributions. For instance, *Messor erwini*, recently described from a single locality in north‐eastern Spain (Orou et al. 2023), is shown here to be widely distributed in the Iberian Peninsula and the Balearic Islands (Ibiza). Other range extensions involve groups needing taxonomic revision, such as *Camponotus oertzeni* and *Cataglyphis italica*. While *C. oertzeni* was known only from the Balkans, we now report it from the Italian peninsula. Conversely, *C. italica* , previously considered endemic to the Italian peninsula, exhibits a trans‐Adriatic distribution.

Morphometric identification is time‐consuming and often restricted to taxa already known at the country level, meaning faunistic inventories may overlook particular species. Many of our newly reported country‐level species required morphometric validation. DNA barcode analysis highlighted a series of incongruences that turned out to be former identification errors. This was the case for the free‐living form of *Messor structor* , reported for the first time in the north of Italy along mountain habitats in the Apennine Mountains, while only clonal males and hybrid workers produced by *M. ibericus* queens are known throughout the country (Juvé et al. 2025).

Similar issues apply to non‐native species. For example, *Hypoponera ergatandria* is reported for the first time in Portugal and *Hypoponera punctatissima* in Montengro. Some historical records of *H. punctatissima* in Europe may belong to *H. ergatandria* , as the two can only be differentiated by morphometric analysis (Seifert 2013).

Ants, as eusocial insects, exhibit distinct castes within colonies—queens, males and workers (Hölldobler and Wilson 1990), with often significant morphological differences. This can result in different castes of the same species being described as different species. In Europe, this issue is particularly evident in the genus *Leptanilla* (Griebenow 2023). Although we were able to assign barcodes to two described species in this genus (Figure 2c), we also sequenced several male specimens that belong to undescribed species or could not be confidently identified. These data offer a basis for linking workers and males and for resolving the parallel taxonomy in this challenging genus.

### 4.2 DNA Barcoding Performance

A series of factors may limit the use of DNA barcoding for reconstructing species population structure and delimiting evolutionary units. The mitochondrial genome is maternally inherited and, thus, it is useful only to reconstruct the matrilineal evolutionary history. Second, since it does not recombine and tends to evolve through genetic sweeps, the patterns observed may be biased (e.g., due to *Wolbachia* infections; Duplouy and Hornett 2018). Third, being based on a single gene, it may be subject to stochasticity or single‐gene effects that may not represent the species history (Toews and Brelsford 2012). For instance, a relatively high incidence of mtDNA introgression across species may lead to conflicting species trees (Paz‐Vinas et al. 2021). However, *COI* is by far the most widely used genetic marker for animal identification, taxonomy and macroecology (Leigh et al. 2021; Dapporto et al. 2019).

Assessing the performance of DNA barcoding for specimen identification in a taxonomically diverse group like ants is inherently challenging. Our results show that DNA barcoding effectiveness largely varies among genera and species groups. High success rates—characterized by species monophyly for *COI*, low barcode sharing and the presence of a barcode gap—were observed in various genera (e.g., *Bothriomyrmex*, *Cardiocondyla*, *Goniomma*, *Hypoponera*, *Ponera, Monomorium*). In contrast, other genera exhibited mixed results depending on the species group (e.g., *Aphaenogaster, Camponotus, Crematogaster, Lasius, Plagiolepis, Tetramorium, Tapinoma*).

Overall, DNA barcoding performance for specimen identification in European ants can be described as moderate, with substantial levels of barcode sharing (19.3% of species), non‐monophyly (43.7%) and absence of a barcode gap (52.4%). The general overlap between intra‐ and interspecific genetic distances and the high level of barcode sharing can be due to a combination of biological (Galtier et al. 2009) (e.g., hybridisation, incomplete lineage sorting) and operational factors (i.e., poor taxonomy, oversplitting or identification errors).

Interspecific hybridization is estimated in more than 18% of the 176 ant species and 14% of the 37 genera present in Central Europe (Yazdi et al. 2012). Low minimum interspecific genetic distances, high barcode sharing and low monophyly in groups known to hybridize are most likely related to this factor (e.g., *Messor* Steiner et al. 2011, *Formica rufa* group, Beresford et al. 2017, *Lasius*, Seifert 1999). Other instances may reflect incomplete lineage sorting, particularly in recently diverged taxa. Some sequence discrepancies are likely to reflect the presence of nuclear mitochondrial DNA segments (NUMTs), although we excluded sequences with stop codons to reduce this issue, which has been reported to be prevalent in some ant genera (e.g., *Atta* [Martins Jr et al. 2007], *Acromyrmex* [Cristiano et al. 2014], *Dorylus* [Kronauer et al. 2010]). Low DNA barcoding performance can also stem from misidentified specimens. Accurate identifications can be particularly difficult in groups such as *Lasius* (e.g., *umbratus* group), *Tetramorium* (e.g., *caespitum* group), *Myrmica* (e.g., *sabuleti* group) and *Formica* (e.g., *rufibarbis* group) (Seifert 2018). Throughout this study, we conducted multiple rounds of verification for dubious identifications using objective morphometric keys whenever feasible and re‐evaluated the genetic outcomes to ensure consistency. Nonetheless, the efficacy of DNA barcoding remains tied to the taxonomic resolution achieved in every group. This limitation is particularly evident in genera like *Lepisiota*, where a taxonomic revision is critically needed (Sharaf et al. 2020), as well as *Temnothorax*, undergoing rapid taxonomic rearrangements due to ongoing species discoveries and synonymizations (Csősz, Taheri, et al. 2025; Csősz, Alicata, et al. 2025).

Finally, at least part of the observed discordance between morphology and DNA barcoding likely reflects the presence of undescribed species. For example, the recently described cryptic species *Messor erwini* (Orou et al. 2023) clusters in a well‐supported monophyletic group in our dataset. The incongruences in other peculiar clades, such as *Stenamma*, may reflect both misidentifications in the retrieved sequences (obtained from a global phylogeny) and genuine new taxa. The highly paraphyletic nature of the *Camponotus aethiops* group underscores the need for a taxonomic revision (Figure 3d, SI 1 Page 49). This pattern may be partly driven by biological factors such as social parasitism. *Camponotus universitatis* , an obligate social parasite of both *C. aethiops* and *C. oertzeni* (Tinaut et al. 1992; Blatrix et al. 2017), is phylogenetically nested within its hosts, suggesting a complex evolutionary history shaped by host–parasite interactions.

### 4.3 Intraspecific Patterns

The intraspecific genetic maps we have generated provide new insights into the mtDNA phylogeographic structure of European ants. A recurring pattern of genetic differentiation between the southern European peninsulas is evident across diverse taxonomic groups, likely indicating the presence of glacial refugia (Dapporto et al. 2024; Hewitt 2000; Taberlet et al. 1998). Several species display divergent mtDNA lineages in different peninsulas (e.g., *Crematogaster scutellaris* in the Iberian and Italian peninsulas), while others exhibit haplotype sharing across peninsulas, like the trans‐Adriatic distribution of a *Camponotus dalmaticus* haplotype. Endemic mtDNA lineages restricted to a single peninsula are common, as seen in *Camponotus ligniperda* in the Balkans, *Stenamma debile* in the Iberian Peninsula and *Camponotus piceus* in the Italian peninsula. Some lineages thought to be endemic to southern Spain (e.g., *Camponotus foreli* ) and Sicily ( *Camponotus nylanderi* ) may represent North African lineages, hypotheses that could be tested with extended sampling into Africa.

We also observed high mtDNA lineage endemicity in Mediterranean islands, both large (e.g., *Aphaenogaster ichnusa* in Sardinia and Corsica or *Camponotus vagus* in Crete) and small ones (e.g., *Aphaenogaster epirotes* in the Ionian islands). However, confirmation of endemicity for some lineages found in the northeastern Aegean and Dodecanese islands (e.g., *Temnothorax balcanicus* and *Camponotus oertzeni* ) will require further sampling to the East, particularly in the Anatolian region. In line with the ‘southern richness and northern purity hypothesis’ (Pontarp et al. 2019), we observed higher haplotype richness in the Mediterranean area than in Central and Northern Europe, which mirrors patterns found in European butterflies (Dincă et al. 2021). Surprisingly, we observed extremely high intraspecific genetic variability even in species with limited distribution ranges. It is the case of *Myrmecina sicula* , restricted to the northwestern tip of Sicily (Schifani et al. 2020), where five haplotypes were found within a 35 km area, with a maximum intraspecific *p*‐dist of 10.1%. High genetic variability and long branches were also found in social parasites, such as *Camponotus universitatis* (max *p*‐dist = 3.9%), the workerless species *Tetramorium atratulum* (max *p*‐dist = 6.9%) and the slave‐maker *Temnothorax muellerianus* (max *p*‐dist = 3.7%). By contrast, we found very few widespread species with low genetic variability (e.g., *Messor ibericus* ).

These contrasting intraspecific mtDNA diversity patterns may be influenced by the differential dispersal capacity of queens. For example, species in the *Aphaenogaster sardoa* group typically lack queen nuptial flights and disperse by nest budding (Baroni‐Urbani 1966). In these taxa, we found high haplotype diversity and intraspecific distances, likely a consequence of their limited dispersal.

Human activity may also contribute to genetic structuring. Native species may have been translocated between regions in Europe, and DNA barcoding can be used to track these events (Comtet et al. 2015) by detecting fragmented or incongruent phylogeographic patterns. In *Pheidole pallidula* , we found one of the highest values of maximum intraspecific *p*‐distances (13.5%) and three clades. The unusual geographic distribution of these clades, highly discordant with expected biogeographic patterns, may reflect human‐mediated introductions facilitated by the tramp species traits of this species (Seifert 2016).

In the case of certain alien species, such as *Hypoponera ergatandria* , the presence of multiple haplotypes throughout their European range points to repeated introduction events underlying the ease with which some ant species are spread and successfully introduced (Menchetti et al. 2024).

Intraspecific genetic patterns found in many species could also be due to mitochondrial selective sweeps. The maternally‐transmitted bacterium *Wolbachia* can influence the reproductive dynamics of its hosts, ants included (Ramalho and Moreau 2020), and its infections have been associated with mtDNA genetic sweeps in other insects (e.g., Gaunet et al. 2019; Ritter et al. 2013).

The data produced in this study offer a significant contribution to the evolving understanding of the biogeography of European ants (Wang et al. 2023). Further investigation using objective methods, more solid taxonomy, single‐species analyses and expanded geographic sampling will be crucial to establish clearer links between the complex interplay of processes determining the high variability of intraspecific genetic patterns observed in ants.

### 4.4 Future Avenues in Ant Exploration

Although ants are one of the most studied insect groups, our study highlights persistent limitations inherent to this group stemming from a still deficient taxonomy. Without a solid taxonomic framework, it remains difficult to reliably assess indexes of mitochondrial genetic diversity, ecological roles and functional traits. By presenting an updated checklist of the European ant fauna and assessing mtDNA genetic patterns for nearly 80% of the fauna, we provide a unique framework for advancing ant research across the continent. The comprehensive dataset here released is designed to support the myrmecological community in species discovery, taxonomic revision and species synonymization within this diverse and ecologically important group.

Preliminary morphological analyses already suggest the presence of putative new species in several genera (e.g., *Goniomma, Stenamma, Messor*) and cases of discordance between mtDNA and morphological identifications underscore the need for further investigation to understand their causes. Resolving these mismatches will require the integration of additional genetic data (multilocus or genomic) alongside morphometric analyses, as proven effective in recent taxonomic revisions (e.g., Csősz, Alicata, et al. 2025; Steiner et al. 2018). The mtDNA genetic maps developed in this study can also guide future sampling strategies, ensuring that representatives of all existing mitochondrial lineages are included in morphological and genomic studies.

Importantly, the utility of this DNA barcode library extends beyond ant taxonomy. Ants play fundamental roles in ecosystems, as ecosystem engineers, predators and prey and are integral to food webs (Oberski et al. 2025). Tools such as metabarcoding can be used to reconstruct trophic interactions and track ecosystem dynamics across space and time (Gorki et al. 2024). However, metabarcoding relies on complete and accurate DNA reference libraries. Similarly, in the context of biological invasions, a continental‐scale barcode library greatly enhances our ability to rapidly identify intercepted or newly introduced species, both within and beyond Europe. Our results identify ant genera in which mitochondrial markers are unreliable, with direct implications for specimen identification, biosurveillance and eDNA‐ and metabarcoding‐based studies. While numerous ant species remain largely unstudied, others have yet to be even discovered or formally described. We hope that the species checklist, DNA barcode library and mitochondrial genetic diversity maps of European ants provided here will become a valuable resource in the collective endeavour to better understand ant diversity and the processes that generate it.

## Author Contributions

M.M., R.V. and L.D. designed the study; M.M., E.S., F.G., S.S., E.S., N.B., B.B.B., L.B., D.C., V.D., V.G., I.G., K.G., J.M.G.D., K.K., A.L.‐G., E.M., G.T., A.T., F.R., S.S., E.S., M.S., T.S., L.D., R.V. contributed sampling; M.M., E.S., F.G., S.S., E.S., N.B., B.B.B., L.B., D.C., V.G., K.G., J.M.G.D., K.K., A.L.‐G., G.T., A.T., F.R., S.S., E.S., R.V. identified specimens, M.M., E.S., C.C., B.B.B. sequenced specimens; M.M., L.D., P.D.N.H., E.S., M.S. performed analyses; M.M. and R.V. wrote a first draft of the manuscript; all authors participated in interpreting the results, in writing and editing the manuscript.

## Funding

Support for this research was provided to MM by ‘la Caixa’ Foundation (ID 100010434, grant LCF/BQ/DR20/11790020) and the European Union's Horizon 2020 research and innovation programme under the Marie Skłodowska‐Curie grant agreement No 101206623, to RV by grants PID2022‐139689NB‐I00 (funded by MICIU/AEI/10.13039/501100011033 and by ERDF, EU) and 2021‐SGR‐00420 (Departament de Recerca i Universitats, Generalitat de Catalunya), to LD by the Horizon project ‘Biodiversity Genomics Europe’(ID: 101059492), to LD and EM by the National Recovery and Resilience Plan (NRRP), Mission 4 Component 2 Investment 1.4 (Project code CN_00000033; Project title ‘National Biodiversity Future Center – NBFC’), to AL‐G by grant No. KP‐06‐N‐51/6 (funded by the National Science Fund of the Republic of Bulgaria), to ES by the ‘Juan de la Cierva’ program (JDC2024‐054485‐I) funded by the MICIU/AEI/10.13039/501100011033 and FSE+, to VD by the Research Council of Finland (Academy Research Fellow, decision nos. 324,988, 328,895 and 352,652), to GT by grant PID2023‐152239NB‐I00 (funded by MICIU/AEI/10.13039/501100011033 and by ERDF, EU) and to PDNH by the New Frontiers In Research Fund (NFRFT‐2020‐00073) and the Canada Foundation for Innovation (MSI 42450).

## Disclosure

Benefit‐Sharing Statement: A research collaboration was developed with scientists, amateur entomologists and nature enthusiasts from the countries that provided the specimens. Collaborators are included as co‐authors or in the acknowledgements based on the quantity and novelty of the specimens they contributed. Benefits from this research arise from the open sharing of data and results through public databases as described above. More broadly, our group is committed to international scientific partnerships.

## Conflicts of Interest

The authors declare no conflicts of interest.

## Supporting information


**Data S1:** supinfo/men70135‐sup‐0001‐Supinfo.zip.

## Data Availability

The Supporting Information and the Atlas V1.0 are on the OSF repository: https://doi.org/10.17605/OSF.IO/SUTWQ. The DNA sequences, the information about collection data, taxonomy, vouchers, GenBank and BOLD accession codes are available in the BOLD dataset DS‐ANTEU and on the OSF repository: https://doi.org/10.17605/OSF.IO/SUTWQ. Checklist is available on the OSF repository: https://doi.org/10.17605/OSF.IO/SUTWQ. Trees are available on the OSF repository: https://doi.org/10.17605/OSF.IO/SUTWQ. R scripts are available on the OSF repository: https://doi.org/10.17605/OSF.IO/SUTWQ.
