# Supplementary material for: DNA Barcode Reference Library for European Ants: A Roadmap for Phylogeography and Species Discovery
Source: Mol Ecol Resour. 2026 Apr 20;26(3):e70135. doi: 10.1111/1755-0998.70135 (PMC13093865; doi:10.1111/1755-0998.70135)
Supplement: Supplementary file 1 — Data S1: supinfo/men70135‐sup‐0001‐Supinfo.zip. [file MEN-26-e70135-s001.zip › men70135-sup-0001-Supinfo/Supporting Information 1b Atlas V1.0_low_res.pdf]

The Mitochondrial Genetic Diversity Maps of European Ants

Supporting Information 1b
Atlas V1.0
(colour-blind accessible version)

See the OSF repository for a higher-resolution version:
10.17605/OSF.IO/SUTWQ

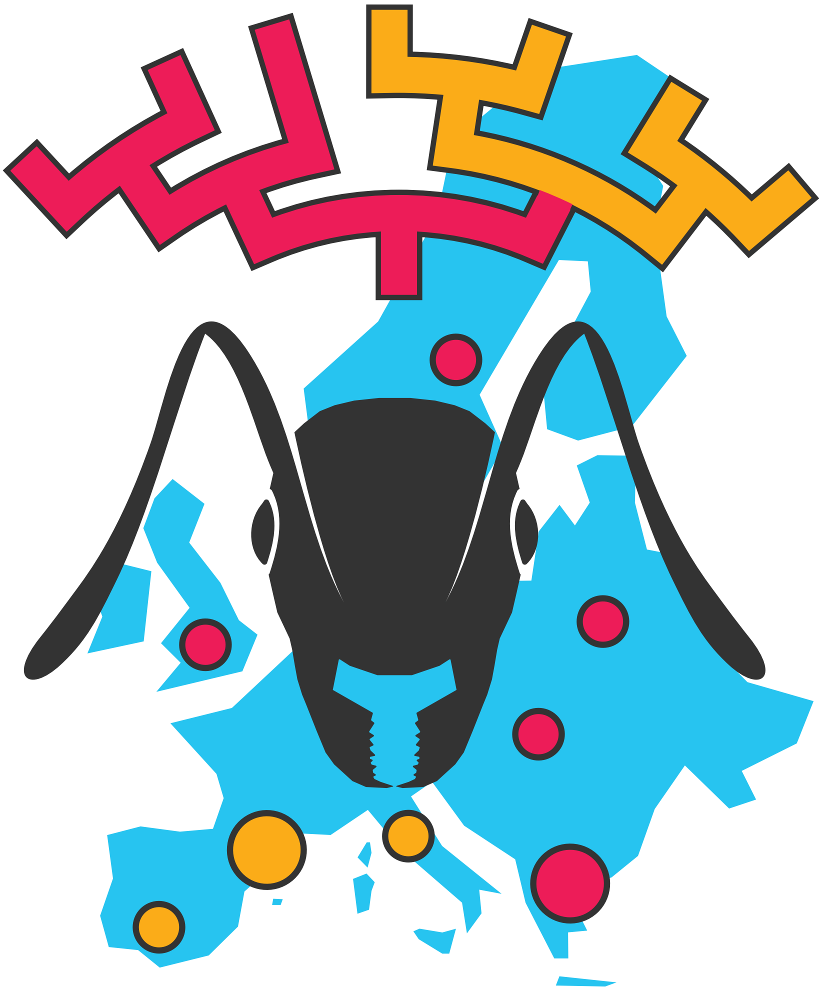

How to cite: Menchetti, M., Schifani, E., García, F., Schär, S., Sbrega, E., Balević, N., Blaimer, B. B., Borowiec, L., Cioppa, D., Corbella, C., Dincă, V., Gentile, V., Giotis, I., Gómez, K., Gómez Durán, J. M., Kalaentzis, K., Lapeva-Gjonova, A., Mori, E., Talavera, G., Tinaut, A., Ruano, F., Salata, S., Sequeira, E., Serracanta, M., Suchan, T., Hebert, P. D. N., Dapporto, L., & Vila, R. (2026). **DNA barcode reference library for European ants: A roadmap for phylogeography and species discovery.** *Molecular Ecology Resources.* DOI: 10.1111/1755-0998.70135.

Table of Contents

Introduction **2**

Interpreting the atlas: **3**
Trees . . . 4
Genetic diversity maps . . . 6

The checklist **8**

The sequence data **22**

The trees **40**

The genetic diversity maps **93**
Acropyga . . . 94
Aenictus . . . 95
Anochetus . . . 96
Aphaenogaster . . . 98
Bothriomyrmex . . . 141
Brachymyrmex . . . 145
Brachyponera . . . 146
Camponotus . . . 147
Cardiocondyla . . . 183
Carebara . . . 191
Cataglyphis . . . 192
Colobopsis . . . 210
Crematogaster . . . 212
Cryptopone . . . 221
Dolichoderus . . . 222
Formica . . . 223
Formicoxenus . . . 256
Goniomma . . . 257
Harpagoxenus . . . 263
Hypoponera . . . 264
Iberoformica . . . 269
Lasius . . . 270
Lepisiota . . . 308
Leptanilla . . . 313
Leptothorax . . . 316
Linepithema . . . 320
Liometopum . . . 321
Manica . . . 322
Messor . . . 323
Metalasius . . . 344
Monomorium . . . 345
Myrmecina . . . 355
Myrmica . . . 358
Nylanderia . . . 386
Oxyopomyrmex . . . 389
Paratrechina . . . 394
Pheidole . . . 395
Plagiolepis . . . 401
Polyergus . . . 411
Ponera . . . 412
Prenolepis . . . 414
Proceratium . . . 415
Proformica . . . 417

Rossomyrmex . . . 426
Solenopsis . . . 427
Stenamma . . . 437
Stigmatomma . . . 442
Strongylognathus . . . 444
Strumigenys . . . 453
Tapinoma . . . 458
Technomyrmex . . . 471
Temnothorax . . . 474
Tetramorium . . . 553
Trichomyrmex . . . 582
Wasmannia . . . 583

Introduction

This atlas contains the information related to the article `DNA barcode reference library for European ants: a roadmap for phylogeography and species discovery`, and includes the checklist, trees, and maps of intraspecific genetic variability of the European ants. This is the colour-blind accessible version; the standard color version is available in Supporting Information 1a. Two initial sections explain how to interpret the trees and genetic maps presented in this file.

Interpreting the atlas:

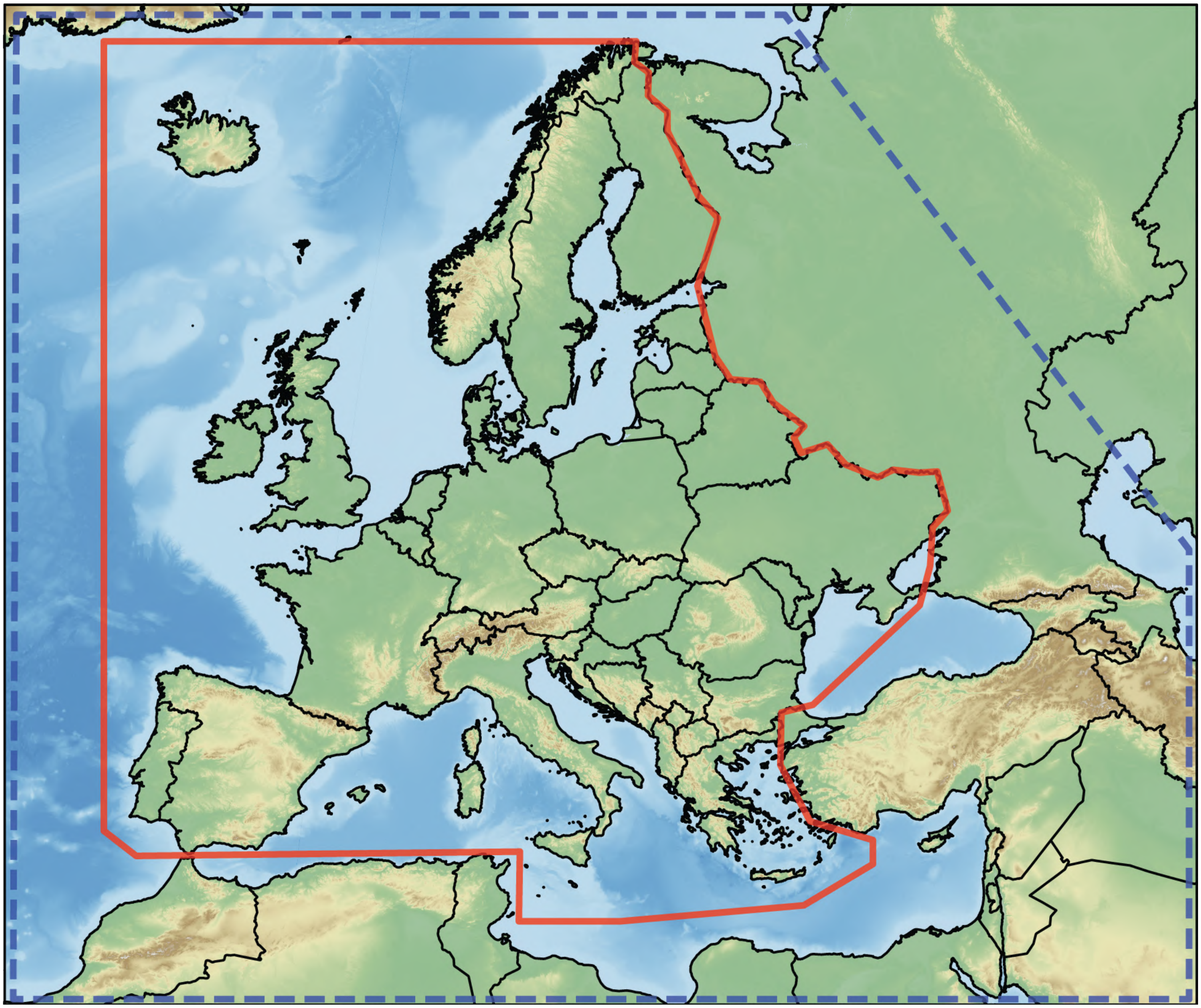

Figure S1: Map showing the study area used for the checklist of European ants and main sampling, outlined in red, as well as the extended study area, outlined with a dashed blue line. In a few exceptional cases, specimens or sequences from outside the study area were included: 1) when specimens were not available from the study area; 2) if the native taxon was described from outside the study area; and 3) in the case of non-native species.

Trees

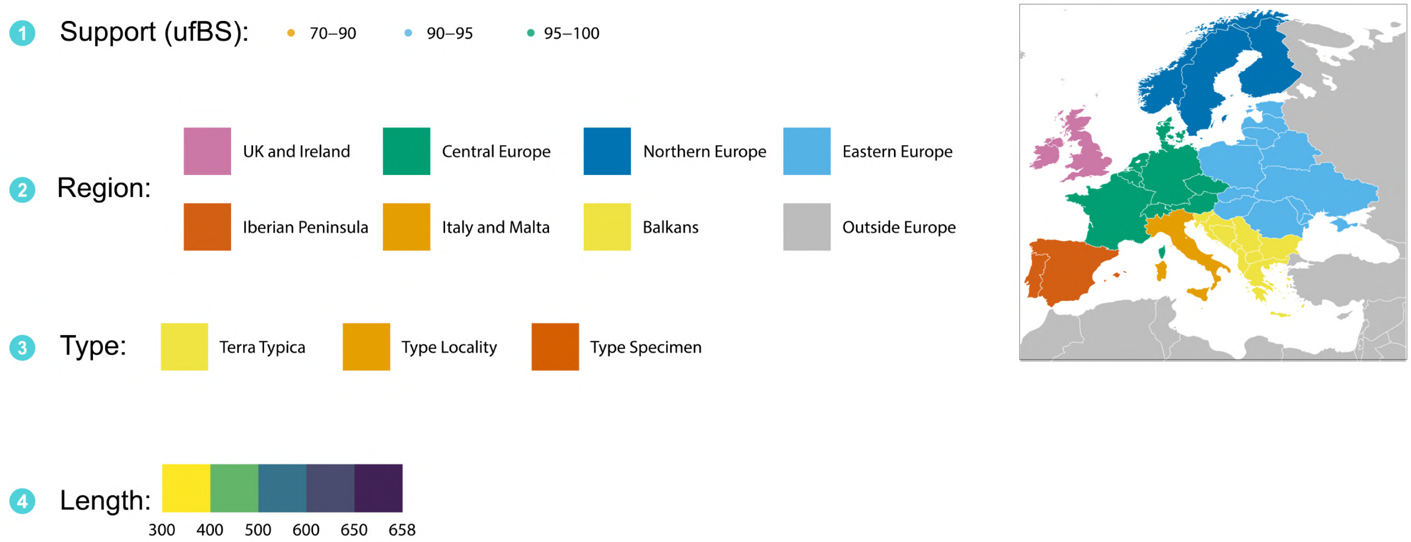

Figure S2: Description of the tree legend. **(1)** Intervals of the ultrafast bootstrap approximation (UFBoot) support values for nodes, note that values below 70 are not shown. Next to each tree there are columns indicating characteristics of the specimens and sequences, as well as results of delimitation methods: **(2)** the region from where the sample was collected, **(3)** specimen feature (if present), and **(4)** coloured-coded sequence lengths (bp).

Table S1: Outgroup species and sequence codes used for each genus tree

	Outgroup 1		Outgroup 2		Outgroup 3		Outgroup 4	
Genus	Species 1	Sequence 1	Species 2	Sequence 2	Species 3	Sequence 3	Species 4	Sequence 4
Acropyga	*Acropyga acutiventris*	MN618336	*Acropyga panamensis*	MK769445	Acropyga sp.	MN134687		
Aenictus	*Lioponera sp. MG02*	JN264391	*Dorylus orientalis*	MF804633	Aenictus sp.	KC685001	Aenictus fergusoni	MF804542
Anochetus	*Phrynoponera transversa*	LN609129	*Anochetus madagascarensis*	EF610749				
Aphaenogaster_Messor	*Stenamma debile*	JQ742645	*Novomessor albisetosus*	JQ742633				
Bothriomyrmex	*Liometopum microcephalum*	KT844617	*Tapinoma magnum*	MT606390				
Brachymyrmex	*Myrmelachista schumanni*	MN134917	*Myrmelachista zeledoni*	MK992402				
Brachyponera	*Ectomomyrmex javanus*	GQ264573	*Anochetus madagascarensis*	EF610749				
Camponotus	*Colobopsis vitrea*	HM914893	*Polyrhachis bihamata*	KM348367				
Cardiocondyla	*Temnothorax angustulus*	MM19A073g1	*Melissotarsus insularis*	KF461544				
Carebara	*Melissotarsus insularis*	KF461544	*Carebara atoma*	GMNGD346-16	Carebara grandidieri	JN283209	Carebara panamensis	MK759252
Cataglyphis	*Formica sanguinea*	MZ609034	*Iberoformica subrufa*	MM19A130b1				
Colobopsis	*Colobopsis_explodens*	MF993260	*Colobopsis vitrea*	HM914893				
Crematogaster	*Cataulacus regularis*	HM418920	*Formicoxenus nitidulus*	MM21F202b1				
Cryptopone	*Brachyponera chinensis*	OM604749	*Euponera antsiraka*	JN287939				
Dolichoderus	*Liometopum microcephalum*	KT844617	*Bothriomyrmex communista*	MM22A046a1				
Formica	*Iberoformica subrufa*	MM19A130b1	*Polyergus rufescens*	MM21D114a1				
Formicoxenus	*Temnothorax angustulus*	MM19A073g1	*Leptothorax acervorum*	LT977583				
Goniomma_Oxyopomyrmex	*Stenamma debile*	JQ742645	*Novomessor albisetosus*	JQ742633				
Harpagoxenus	*Temnothorax angustulus*	MM19A073g1	*Myrmecina americana*	KR926790				
Hypoponera	*Ponera coarctata*	MT606380	*Cryptopone ochracea*	MM16A082a1				
Iberoformica	*Formica sanguinea*	MZ609034	*Polyergus rufescens*	MM21D114a1				
Lasius	*Myrmecocystus sp.*	HQ928785	*Myrmecocystus placodops*	HQ928783				
Lepisiota	*Acropyga acutiventris*	MN618336	*Acropyga panamensis*	MK769445				
Leptanilla	*Protanilla wardi*	CASENT0221924	*Leptanilla argamani*	CASENT0235253				
Leptothorax	*Temnothorax angustulus*	MM19A073g1	*Temnothorax mediterraneus*	MH138470				
Linepithema	*Liometopum microcephalum*	KT844617	*Tapinoma magnum*	MT606390				
Liometopum	*Bothriomyrmex communista*	MM22A046a1	*Tapinoma magnum*	MT606390				
Manica	*Myrmica rubra*	RVcoll19D886a1	*Manica hunteri*	JN291902				
Metalasius	*Lasius myops*	MM21B099b1	*Lasius emarginatus*	MT606326	Nylanderia vividula	KG02007Da1	Prenolepis imparis	MW280444
Monomorium	*Solenopsis invicta*	HQ928647	*Rogeria leptonana*	MK759646				
Myrmecina	*Myrmecina sp.*	GMPNB239-18	*Myrmecina americana*	KR926790				
Myrmica	*Manica rubida*	RVcoll17S262a1	*Manica hunteri*	JN291902				
Nylanderia	*Lasius myops*	MM21B099b1	*Iberoformica subrufa*	MM19A130b1				
Paratrechina	*Lasius myops*	MM21B099b1	*Nylanderia jaegerskioeldi*	MT606361				
Pheidole	*Wasmannia rochai*	MK759603	*Cephalotes atratus*	MK759225				
Plagiolepis	*Acropyga sp.*	MN134687	*Acropyga acutiventris*	MN618336				
Polyergus	*Formica sanguinea*	MZ609034	*Polyergus samurai*	KX664678				
Ponera	*Hypoponera eduardi*	MM21B166d1	*Ponera leae*	JN306827				
Prenolepis	*Lasius myops*	MM21B099b1	*Prenolepis imparis*	MW280444				
Proceratium	*Hypoponera eduardi*	MM21B166d1	*Proceratium_rugigaster*	JN287832				
Proformica	*Formica sanguinea*	MZ609034	*Iberoformica subrufa*	MM19A130b1				
Rossomyrmex	*Iberoformica subrufa*	MM19A130b1	*Cataglyphis cursor*	MM21D542a1				
Solenopsis	*Monomorium carbonarium*	MW562825	*Monomorium pharaonis*	MN619463				
Stenamma	*Novomessor albisetosus*	JQ742633	*Veromessor andrei*	JQ742636				
Stigmatomma	*Prionopelta modesta*	HQ545960	*Prionopelta amabilis*	JN289323				
Strongylognathus_Tetramorium	*Cataulacus regularis*	HM418920	*Melissotarsus insularis*	KF461544				
Strumigenys	*Cephalotes atratus*	MK759225	*Basiceros militaris*	MN134707				
Tapinoma	*Bothriomyrmex corsicus*	MM21D306a1	*Bothriomyrmex communista*	MM22A046a1				
Technomyrmex	*Bothriomyrmex atlantis*	MM21D120a1	*Bothriomyrmex communista*	MM22A046a1				
Temnothorax	*Trichomyrmex destructor*	GU710452	*Leptothorax acervorum*	LT977583				
Trichomyrmex	*Cataulacus regularis*	HM418920	*Melissotarsus insularis*	KF461544				
Wasmannia	*Cephalotes atratus*	MK759225	*Wasmannia rochai*	MK759603				

Genetic diversity maps

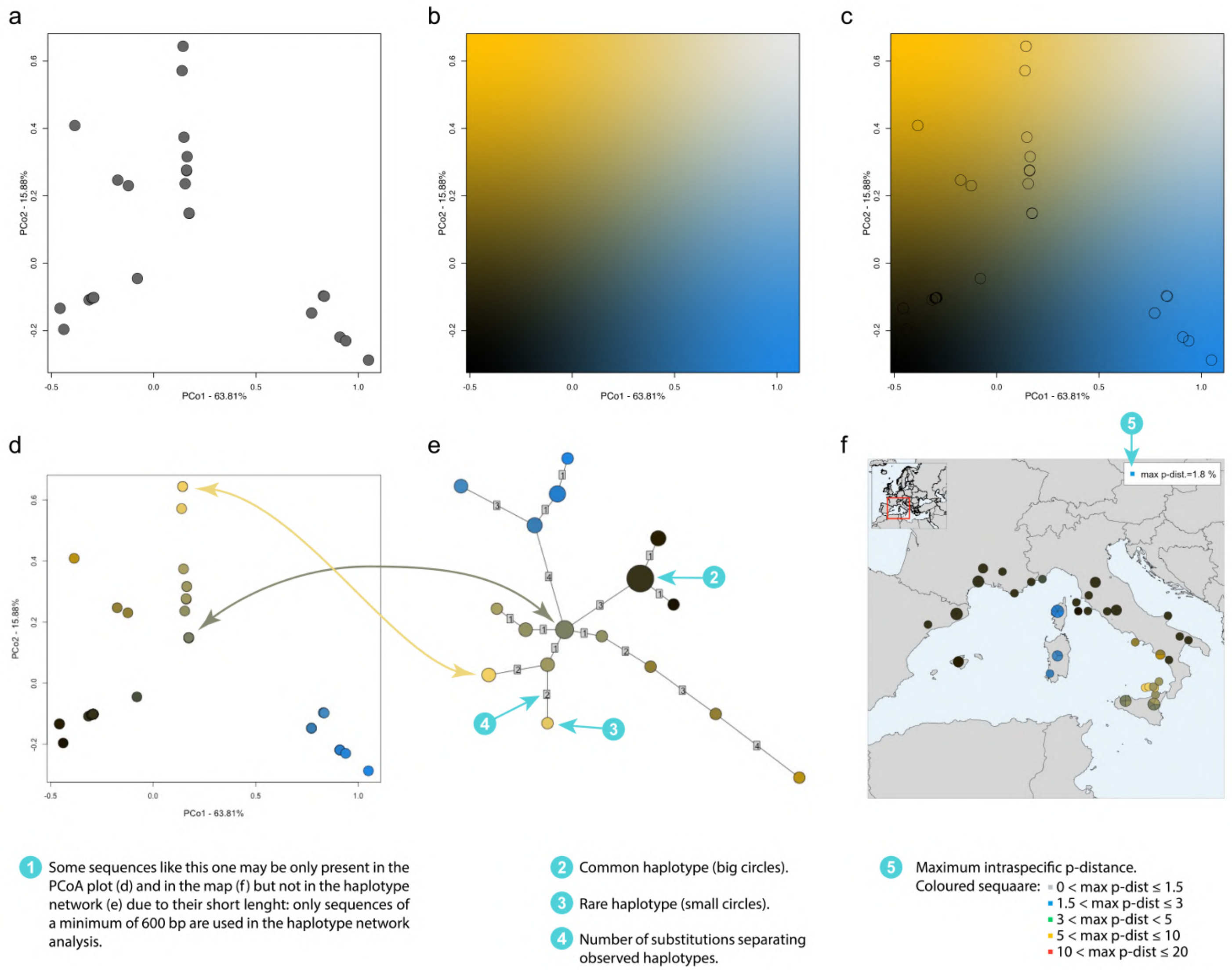

Figure S3: (a, b, c) Steps illustrating the creation of the PCoA plot **(d)**, haplotype network **(e)**, and genetic diversity map **(f)**.

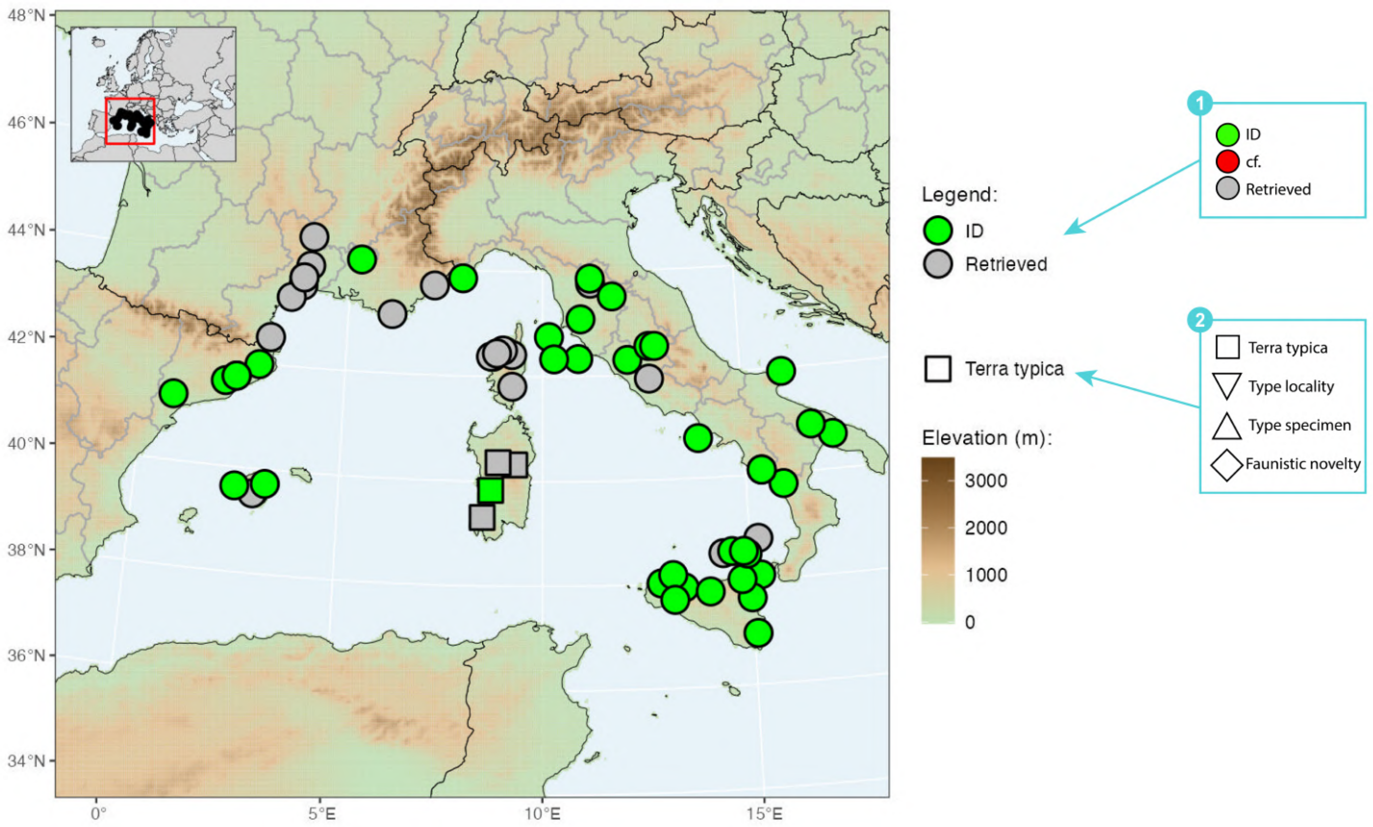

Figure S4: The specimen map shows the localities and features of the sequenced specimens on a map with altitudes as colour background. Symbol colours **(1)** represent the specimen identification (ID = green or cf. = red) or if the sequence was retrieved from a data repository (grey). **(2)** A shape different from a circle indicates if the specimen was collected in the terra typica (square) or type locality (triangle pointing down) of the species, if it is type material (triangle pointing up), or if it represents a faunistic novelty (diamond).

The checklist

Table S2: Checklist of European ant species (compiled up to 31st December 2025). The Introduced column indicates non-native species with established outdoor populations (Y), those restricted to indoor environments (I), and species with a doubtful status regarding their non-native origin in Europe (D).

Subfamily	Species	Author	Introduced
Amblyoponinae	*Stigmatomma denticulatum*	Roger, 1859	
Amblyoponinae	*Stigmatomma emeryi*	Saunders, E., 1890	D
Amblyoponinae	*Stigmatomma gaetulicum*	(Baroni Urbani, 1978)	
Amblyoponinae	*Stigmatomma impressifrons*	Emery, 1869	
Dolichoderinae	*Bothriomyrmex atlantis*	Santschi, 1922	
Dolichoderinae	*Bothriomyrmex communista*	Santschi, 1919	
Dolichoderinae	*Bothriomyrmex corsicus*	Santschi, 1923	
Dolichoderinae	*Bothriomyrmex meridionalis*	(Roger, 1863)	
Dolichoderinae	*Bothriomyrmex modestus*	Radchenko, 1985	
Dolichoderinae	*Dolichoderus quadripunctatus*	(Linnaeus, 1771)	
Dolichoderinae	*Linepithema angulatum*	(Emery, 1894)	I
Dolichoderinae	*Linepithema iniquum*	(Mayr, 1870)	I
Dolichoderinae	*Linepithema humile*	(Mayr, 1868)	Y
Dolichoderinae	*Liometopum microcephalum*	(Panzer, 1798)	
Dolichoderinae	*Tapinoma darioi*	Seifert, D'Eustacchio, Kaufmann, Centorame & Modica, 2017	
Dolichoderinae	*Tapinoma erraticum*	(Latreille, 1798)	
Dolichoderinae	*Tapinoma festae*	Emery, 1925	
Dolichoderinae	*Tapinoma glabrella*	Nylander, 1849	
Dolichoderinae	*Tapinoma hispanicum*	Seifert, Kaufmann & Fraysse, 2024	
Dolichoderinae	*Tapinoma ibericum*	Santschi, 1925	
Dolichoderinae	*Tapinoma insularis*	Seifert, Kaufmann & Fraysse, 2024	
Dolichoderinae	*Tapinoma kinburni*	Karavaiev, 1937	
Dolichoderinae	*Tapinoma madeirense*	Forel, 1895	
Dolichoderinae	*Tapinoma magnum*	Mayr, 1861	
Dolichoderinae	*Tapinoma melanocephalum*	(Fabricius, 1793)	Y
Dolichoderinae	*Tapinoma nigerrimum*	Nylander, 1856	
Dolichoderinae	*Tapinoma pithecorum*	Seifert, 2022	Y
Dolichoderinae	*Tapinoma pygmaeum*	(Dufour, 1857)	
Dolichoderinae	*Tapinoma sessile*	(Say, 1836)	I
Dolichoderinae	*Tapinoma simrothi*	Krausse, 1911	
Dolichoderinae	*Tapinoma subboreale*	Seifert, 2012	
Dolichoderinae	*Technomyrmex albipes*	(Smith, F., 1861)	I
Dolichoderinae	*Technomyrmex difficilis*	Forel, 1892	I
Dolichoderinae	*Technomyrmex pallipes*	(Smith, F., 1876)	I
Dolichoderinae	*Technomyrmex vexatus*	(Santschi, 1919)	Y
Dolichoderinae	*Technomyrmex vitiensis*	Mann, 1921	I
Dorylinae	*Aenictus rhodiensis*	Menozzi, 1936	
Formicinae	*Acropyga paleartica*	Menozzi, 1936	
Formicinae	*Brachymyrmex patagonicus*	Mayr, 1868	Y
Formicinae	*Camponotus aegaeus*	Emery, 1915	
Formicinae	*Camponotus aethiops*	(Latreille, 1798)	
Formicinae	*Camponotus amaurus*	Espadaler, 1997	
Formicinae	*Camponotus atriceps*	(Smith, F., 1858)	I
Formicinae	*Camponotus atricolor*	(Nylander, 1849)	
Formicinae	*Camponotus baldaccii*	Emery, 1908	

Table S2: Checklist of European ant species (compiled up to 31st December 2025). The Introduced column indicates non-native species with established outdoor populations (Y), those restricted to indoor environments (I), and species with a doubtful status regarding their non-native origin in Europe (D). *(continued)*

Subfamily	Species	Author	Introduced
Formicinae	*Camponotus barbaricus*	Emery, 1905	
Formicinae	*Camponotus boghossiani*	Forel, 1911	
Formicinae	*Camponotus candiotes*	Emery, 1894	
Formicinae	*Camponotus cruentatus*	(Latreille, 1802)	
Formicinae	*Camponotus dalmaticus*	(Nylander, 1849)	
Formicinae	*Camponotus fallax*	(Nylander, 1856)	
Formicinae	*Camponotus foreli*	Emery, 1881	
Formicinae	*Camponotus gestroi*	Emery, 1878	
Formicinae	*Camponotus haroi*	Espadaler, 1997	
Formicinae	*Camponotus heidrunvogtae*	Seifert, 2019	
Formicinae	*Camponotus herculeanus*	(Linnaeus, 1758)	
Formicinae	*Camponotus ionius*	Emery, 1920	
Formicinae	*Camponotus jaliensis*	Dalla Torre, 1893	
Formicinae	*Camponotus kiesenwetteri*	(Roger, 1859)	
Formicinae	*Camponotus laconicus*	Emery, 1920	
Formicinae	*Camponotus lateralis*	(Olivier, 1792)	
Formicinae	*Camponotus ligniperda*	(Latreille, 1802)	
Formicinae	*Camponotus micans*	(Nylander, 1856)	
Formicinae	*Camponotus nitidescens*	Forel, 1889	
Formicinae	*Camponotus nylanderi*	Emery, 1921	
Formicinae	*Camponotus oertzeni*	Forel, 1889	
Formicinae	*Camponotus piceus*	(Leach, 1825)	
Formicinae	*Camponotus pilicornis*	(Roger, 1859)	
Formicinae	*Camponotus rebeccae*	Forel, 1913	
Formicinae	*Camponotus ruber*	Emery, 1925	
Formicinae	*Camponotus samius*	Forel, 1889	
Formicinae	*Camponotus sanctus*	Forel, 1904	
Formicinae	*Camponotus spissinodis*	Forel, 1909	
Formicinae	*Camponotus sylvaticus*	(Olivier, 1792)	
Formicinae	*Camponotus tergestinus*	Müller, 1921	
Formicinae	*Camponotus universitatis*	Forel, 1890	
Formicinae	*Camponotus vagus*	(Scopoli, 1763)	
Formicinae	*Cataglyphis cretica*	(Forel, 1910)	
Formicinae	*Cataglyphis cursor*	(Fonscolombe, 1846)	
Formicinae	*Cataglyphis douwesi*	De Haro & Collingwood, 2000	
Formicinae	*Cataglyphis floricola*	Tinaut, 1993	
Formicinae	*Cataglyphis gadeai*	De Haro & Collingwood, 2003	
Formicinae	*Cataglyphis hellenica*	ForeL, 1886	
Formicinae	*Cataglyphis hispanica*	(Emery, 1906)	
Formicinae	*Cataglyphis humeya*	Tinaut, 1991	
Formicinae	*Cataglyphis iberica*	(Emery, 1906)	
Formicinae	*Cataglyphis italica*	(Emery, 1906)	
Formicinae	*Cataglyphis minos*	Borowiec & Salata, 2022	
Formicinae	*Cataglyphis nodus*	(Brullé, 1833)	
Formicinae	*Cataglyphis piliscapa*	(Forel, 1901)	
Formicinae	*Cataglyphis rosenhaueri*	Santschi, 1925	
Formicinae	*Cataglyphis tartessica*	Amor & Ortega, 2014	
Formicinae	*Cataglyphis velox*	Santschi, 1929	

Table S2: Checklist of European ant species (compiled up to 31st December 2025). The Introduced column indicates non-native species with established outdoor populations (Y), those restricted to indoor environments (I), and species with a doubtful status regarding their non-native origin in Europe (D). *(continued)*

Subfamily	Species	Author	Introduced
Formicinae	*Cataglyphis viatica*	(Fabricius, 1787)	
Formicinae	*Cataglyphis viaticoides*	(André, 1881)	
Formicinae	*Colobopsis truncata*	(Spinola, 1808)	
Formicinae	*Colobopsis imitans*	Schifani, Giannetti, Csősz, Castellucci, Luchetti, Castracani, Spotti, Mori & Grasso, 2021	
Formicinae	*Formica aquilonia*	Yarrow, 1955	
Formicinae	*Formica bruni*	Kutter, 1967	
Formicinae	*Formica cinerea*	Mayr, 1853	
Formicinae	*Formica cinereofusca*	Karavaiev, 1929	
Formicinae	*Formica clara*	Forel, 1886	
Formicinae	*Formica corsica*	Seifert, 2002	
Formicinae	*Formica cunicularia*	Latreille, 1798	
Formicinae	*Formica decipiens*	Bondroit, 1918	
Formicinae	*Formica dusmeti*	Emery, 1909	
Formicinae	*Formica exsecta*	Nylander, 1846	
Formicinae	*Formica fennica*	Seifert, 2000	
Formicinae	*Formica foreli*	Bondroit, 1918	
Formicinae	*Formica forsslundi*	Lohmander, 1949	
Formicinae	*Formica frontalis*	Santschi, 1919	
Formicinae	*Formica fusca*	Linnaeus, 1758	
Formicinae	*Formica fuscocinerea*	Forel, 1874	
Formicinae	*Formica gagates*	Latreille, 1798	
Formicinae	*Formica gagatoides*	Ruzsky, 1904	
Formicinae	*Formica gerardi*	Bondroit, 1917	
Formicinae	*Formica helvetica*	Seifert, 2021	
Formicinae	*Formica lemani*	Bondroit, 1917	
Formicinae	*Formica lugubris*	Zetterstedt, 1838	
Formicinae	*Formica paralugubris*	Seifert, 1996	
Formicinae	*Formica picea*	Nylander, 1846	
Formicinae	*Formica polyctena*	Foerster, 1850	
Formicinae	*Formica pratensis*	Retzius, 1783	
Formicinae	*Formica pressilabris*	Nylander, 1846	
Formicinae	*Formica pyrenaea*	Bondroit, 1918	
Formicinae	*Formica rufa*	Linnaeus, 1761	
Formicinae	*Formica rufibarbis*	Fabricius, 1793	
Formicinae	*Formica sanguinea*	Latreille, 1798	
Formicinae	*Formica selysi*	Bondroit, 1918	
Formicinae	*Formica suecica*	Adlerz, 1902	
Formicinae	*Formica truncorum*	Fabricius, 1804	
Formicinae	*Formica uralensis*	Ruzsky, 1895	
Formicinae	*Iberoformica subrufa*	(Roger, 1859)	
Formicinae	*Lasius alienus*	(Foerster, 1850)	
Formicinae	*Lasius austriacus*	Schlick-Steiner, 2003	
Formicinae	*Lasius balcanicus*	Seifert, 1988	
Formicinae	*Lasius balearicus*	Talavera, Espadaler & Vila, 2014	
Formicinae	*Lasius bicornis*	(Foerster, 1850)	
Formicinae	*Lasius bombycinus*	Seifert & Galkowski, 2016	
Formicinae	*Lasius brunneus*	(Latreille, 1798)	

Table S2: Checklist of European ant species (compiled up to 31st December 2025). The Introduced column indicates non-native species with established outdoor populations (Y), those restricted to indoor environments (I), and species with a doubtful status regarding their non-native origin in Europe (D). *(continued)*

Subfamily	Species	Author	Introduced
Formicinae	*Lasius carniolicus*	Mayr, 1861	
Formicinae	*Lasius casevitzi*	Seifert & Galkowski, 2016	
Formicinae	*Lasius cinereus*	Seifert, 1992	
Formicinae	*Lasius citrinus*	Emery, 1922	
Formicinae	*Lasius creticus*	Seifert, 2020	
Formicinae	*Lasius distinguendus*	(Emery, 1916)	
Formicinae	*Lasius emarginatus*	(Olivier, 1792)	
Formicinae	*Lasius flavus*	(Fabricius, 1782)	
Formicinae	*Lasius fuliginosus*	(Latreille, 1798)	
Formicinae	*Lasius grandis*	Forel, 1909	
Formicinae	*Lasius illyricus*	Zimmermann, 1935	
Formicinae	*Lasius jensi*	Seifert, 1982	
Formicinae	*Lasius karpinisi*	Seifert, 1992	
Formicinae	*Lasius kritikos*	Seifert, 2020	
Formicinae	*Lasius lasioides*	(Emery, 1869)	
Formicinae	*Lasius longiceps*	Seifert, 1988	
Formicinae	*Lasius maltaeus*	Seifert, 2020	
Formicinae	*Lasius meridionalis*	(Bondroit, 1920)	
Formicinae	*Lasius mixtus*	(Nylander, 1846)	
Formicinae	*Lasius myops*	Forel, 1894	
Formicinae	*Lasius neglectus*	Van Loon, Boomsma & Andrasfalvy, 1990	Y
Formicinae	*Lasius niger*	(Linnaeus, 1758)	
Formicinae	*Lasius nitidigaster*	Seifert, 1996	
Formicinae	*Lasius paralienus*	Seifert, 1992	
Formicinae	*Lasius piliferus*	Seifert, 1992	
Formicinae	*Lasius platythorax*	Seifert, 1991	
Formicinae	*Lasius precursor*	Seifert, 2020	
Formicinae	*Lasius psammophilus*	Seifert, 1992	
Formicinae	*Lasius rabaudi*	(Bondroit, 1917)	
Formicinae	*Lasius reginae*	Faber, 1967	
Formicinae	*Lasius sabularum*	(Bondroit, 1918)	
Formicinae	*Lasius tapinomoides*	Salata & Borowiec, 2018	
Formicinae	*Lasius turcicus*	Santschi, 1921	
Formicinae	*Lasius umbratus*	(Nylander, 1846)	
Formicinae	*Lasius viehmeyeri*	Emery, 1922	
Formicinae	*Lepisiota capensis*	(Mayr, 1862)	Y
Formicinae	*Lepisiota frauenfeldi*	(Mayr, 1855)	
Formicinae	*Lepisiota melas*	(Emery, 1915)	
Formicinae	*Lepisiota nigra*	(Dalla Torre, 1893)	
Formicinae	*Lepisiota syriaca*	(André, 1881)	Y
Formicinae	*Lepisiota velox*	(Baroni Urbani, 1968)	
Formicinae	*Metalasius myrmidon*	(Mei, 1998)	
Formicinae	*Nylanderia jaegerskioeldi*	(Mayr, 1904)	Y
Formicinae	*Nylanderia flavipes*	(Smith, 1874)	Y
Formicinae	*Nylanderia vividula*	(Nylander, 1846)	Y
Formicinae	*Paratrechina longicornis*	(Latreille, 1802)	Y
Formicinae	*Plagiolepis alluaudi*	Emery, 1894	I
Formicinae	*Plagiolepis ampeloni*	(Faber, 1969)	

Table S2: Checklist of European ant species (compiled up to 31st December 2025). The Introduced column indicates non-native species with established outdoor populations (Y), those restricted to indoor environments (I), and species with a doubtful status regarding their non-native origin in Europe (D). *(continued)*

Subfamily	Species	Author	Introduced
Formicinae	*Plagiolepis delaugerrei*	Casevitz-Weulersse, 2014	
Formicinae	*Plagiolepis grassei*	Le Masne, 1956	
Formicinae	*Plagiolepis invadens*	Seifert, 2020	Y
Formicinae	*Plagiolepis karawajewi*	Radchenko, 1989	
Formicinae	*Plagiolepis pallescens*	Forel, 1889	
Formicinae	*Plagiolepis atlantis*	Santschi, 1920	
Formicinae	*Plagiolepis pyrenaica*	Emery, 1921	
Formicinae	*Plagiolepis pygmaea*	(Latreille, 1798)	
Formicinae	*Plagiolepis schmitzii*	Forel, 1895	
Formicinae	*Plagiolepis taurica*	Santschi, 1920	
Formicinae	*Plagiolepis xene*	Stärcke, 1936	
Formicinae	*Polyergus rufescens*	(Latreille, 1798)	
Formicinae	*Prenolepis nitens*	(Mayr, 1853)	
Formicinae	*Proformica cerdanyensis*	Galkowski, Lebas, Lenoir, Perdereau & Blatrix, 2022	
Formicinae	*Proformica chelmosensis*	Lebas & Galkowski, 2019	
Formicinae	*Proformica christophii*	Lebas, 2025	
Formicinae	*Proformica epinotalis*	Kuznetsov-Ugamsky, 1927	
Formicinae	*Proformica ferreri*	Bondroit, 1918	
Formicinae	*Proformica kobachidzei*	Arnol'di, 1968	
Formicinae	*Proformica korbi*	(Emery, 1909)	
Formicinae	*Proformica lebasi*	Borowiec & Salata, 2022	
Formicinae	*Proformica longipilosa*	Galkowski, Lebas, Wegnez, Lenoir & Blatrix, 2017	
Formicinae	*Proformica longiseta*	Collingwood, 1978	
Formicinae	*Proformica nasuta*	(Nylander, 1856)	
Formicinae	*Proformica oculatissima*	(Forel, 1886)	
Formicinae	*Proformica pilosiscapa*	Dlussky, 1969	
Formicinae	*Proformica striaticeps*	(Forel, 1911)	
Formicinae	*Rossomyrmex minuchae*	Tinaut, 1981	
Leptanillinae	*Leptanilla charonea*	Barandica, López, Martínez & Ortuno, 1994	
Leptanillinae	*Leptanilla doderoi*	Emery, 1915	
Leptanillinae	*Leptanilla ortunoi*	López, Martínez & Barandica, 1994	
Leptanillinae	*Leptanilla plutonia*	López, Martínez & Barandica, 1994	
Leptanillinae	*Leptanilla poggii*	Mei, 1995	
Leptanillinae	*Leptanilla revelierii*	Emery, 1870	
Leptanillinae	*Leptanilla zaballosi*	Barandica, López, Martínez & Ortuno, 1994	
Myrmicinae	*Aphaenogaster aktaci*	Kiran & Tezcan, 2008	
Myrmicinae	*Aphaenogaster asterioni*	Borowiec, Menchetti, Salata, Vila & Zięcina 2024	
Myrmicinae	*Aphaenogaster balcanica*	(Emery, 1898)	
Myrmicinae	*Aphaenogaster balcanicoides*	Boer, 2013	
Myrmicinae	*Aphaenogaster campana*	Emery, 1878	
Myrmicinae	*Aphaenogaster cardenai*	Espadaler, 1981	
Myrmicinae	*Aphaenogaster cecconii*	Emery, 1894	
Myrmicinae	*Aphaenogaster charesi*	Salata & Borowiec, 2016	
Myrmicinae	*Aphaenogaster dulcineae*	Emery, 1924	
Myrmicinae	*Aphaenogaster epirotes*	(Emery, 1895)	
Myrmicinae	*Aphaenogaster festae*	Emery, 1915	
Myrmicinae	*Aphaenogaster fiorii*	Emery, 1915	
Myrmicinae	*Aphaenogaster gemella*	(Roger, 1862)	

Table S2: Checklist of European ant species (compiled up to 31st December 2025). The Introduced column indicates non-native species with established outdoor populations (Y), those restricted to indoor environments (I), and species with a doubtful status regarding their non-native origin in Europe (D). *(continued)*

Subfamily	Species	Author	Introduced
Myrmicinae	*Aphaenogaster gibbosa*	(Latreille, 1798)	
Myrmicinae	*Aphaenogaster graeca*	Schulz, 1994	
Myrmicinae	*Aphaenogaster iberica*	Emery, 1908	
Myrmicinae	*Aphaenogaster ichnusa*	Santschi, 1925	
Myrmicinae	*Aphaenogaster illyrica*	Bračko, Lapeva-Gjonova, Salata, Borowiec & Polak, 2019	
Myrmicinae	*Aphaenogaster inermita*	Bolton, 1995	
Myrmicinae	*Aphaenogaster italica*	Bondroit, 1918	
Myrmicinae	*Aphaenogaster jolantae*	Borowiec & Salata, 2014	
Myrmicinae	*Aphaenogaster karpathica*	Boer, 2013	
Myrmicinae	*Aphaenogaster melitensis*	Santschi, 1933	
Myrmicinae	*Aphaenogaster olympica*	Borowiec & Salata, 2014	
Myrmicinae	*Aphaenogaster ovaticeps*	(Emery, 1898)	
Myrmicinae	*Aphaenogaster pallida*	(Nylander, 1849)	
Myrmicinae	*Aphaenogaster peloponnesiaca*	Salata, Karaman, Kiran, Borowiec 2021	
Myrmicinae	*Aphaenogaster picena*	Baroni Urbani, 1971	
Myrmicinae	*Aphaenogaster rugosoferruginea*	Forel, 1889	
Myrmicinae	*Aphaenogaster sangiorgii*	(Emery, 1901)	
Myrmicinae	*Aphaenogaster sardoa*	Mayr, 1853	
Myrmicinae	*Aphaenogaster semipolita*	(Nylander, 1856)	
Myrmicinae	*Aphaenogaster senilis*	Mayr, 1853	
Myrmicinae	*Aphaenogaster subterraneosplendida*	Emery, 1908	
Myrmicinae	*Aphaenogaster simonellii*	Emery, 1894	
Myrmicinae	*Aphaenogaster spinosa*	Emery, 1878	
Myrmicinae	*Aphaenogaster splendida*	(Roger, 1859)	
Myrmicinae	*Aphaenogaster sporadis*	Santschi, 1933	
Myrmicinae	*Aphaenogaster striativentris*	Forel, 1895	
Myrmicinae	*Aphaenogaster strioloides*	Forel, 1890	
Myrmicinae	*Aphaenogaster subcostata*	Viehmeyer, 1922	
Myrmicinae	*Aphaenogaster subterranea*	(Latreille, 1798)	
Myrmicinae	*Aphaenogaster subterraneoides*	Emery, 1881	
Myrmicinae	*Aphaenogaster tristis*	Borowiec, Menchetti, Salata, Vila & Zięcina 2024	
Myrmicinae	*Aphaenogaster trinacriae*	Alicata & Schifani, 2019	
Myrmicinae	*Aphaenogaster ulibeli*	Gomez & Espadaler, 2018	
Myrmicinae	*Cardiocondyla batesii*	Forel, 1894	
Myrmicinae	*Cardiocondyla bulgarica*	Forel, 1892	
Myrmicinae	*Cardiocondyla dalmatica*	Soudek, 1925	
Myrmicinae	*Cardiocondyla elegans*	Emery, 1869	
Myrmicinae	*Cardiocondyla emeryi*	Forel, 1881	Y
Myrmicinae	*Cardiocondyla mauritanica*	Forel, 1890	D
Myrmicinae	*Cardiocondyla nigra*	Forel, 1905	
Myrmicinae	*Cardiocondyla obscurior*	Wheeler, W.M., 1929	Y
Myrmicinae	*Cardiocondyla sahlbergi*	Forel, 1913	
Myrmicinae	*Cardiocondyla stambuloffii*	Forel, 1913	
Myrmicinae	*Cardiocondyla ulianini*	Emery, 1889	
Myrmicinae	*Carebara oertzeni*	(Forel, 1886)	
Myrmicinae	*Crematogaster auberti*	Emery, 1869	
Myrmicinae	*Crematogaster erectepilosa*	Salata & Borowiec, 2015	

Table S2: Checklist of European ant species (compiled up to 31st December 2025). The Introduced column indicates non-native species with established outdoor populations (Y), those restricted to indoor environments (I), and species with a doubtful status regarding their non-native origin in Europe (D). *(continued)*

Subfamily	Species	Author	Introduced
Myrmicinae	*Crematogaster fuentei*	Menozzi, 1922	
Myrmicinae	*Crematogaster gordani*	Karaman, 2008	
Myrmicinae	*Crematogaster inermis*	Mayr, 1862	Y
Myrmicinae	*Crematogaster ionia*	Forel, 1911	
Myrmicinae	*Crematogaster laestrygon*	Emery, 1869	
Myrmicinae	*Crematogaster lorteti*	Forel, 1910	
Myrmicinae	*Crematogaster montenigrina*	Karaman, 2008	
Myrmicinae	*Crematogaster ourea*	Salata, Ziecina & Borowiec, 2024	
Myrmicinae	*Crematogaster schmidti*	(Mayr, 1853)	
Myrmicinae	*Crematogaster scutellaris*	(Olivier, 1792)	
Myrmicinae	*Crematogaster sordidula*	(Nylander, 1849)	
Myrmicinae	*Formicoxenus nitidulus*	(Nylander, 1846)	
Myrmicinae	*Goniomma baeticum*	Reyes, Espadaler & Rodriguez, 1987	
Myrmicinae	*Goniomma blanci*	(André, 1881)	
Myrmicinae	*Goniomma collingwoodi*	Espadaler, 1997	
Myrmicinae	*Goniomma compressisquama*	Tinaut, 1995	
Myrmicinae	*Goniomma decipiens*	Espadaler, 1997	
Myrmicinae	*Goniomma hispanicum*	(André, 1883)	
Myrmicinae	*Goniomma kugleri*	Espadaler, 1986	
Myrmicinae	*Harpagoxenus sublaevis*	(Nylander, 1849)	
Myrmicinae	*Leptothorax acervorum*	(Fabricius, 1793)	
Myrmicinae	*Leptothorax buschingeri*	Kutter, 1967	
Myrmicinae	*Leptothorax goesswaldi*	Kutter, 1967	
Myrmicinae	*Leptothorax gredleri*	Mayr, 1855	
Myrmicinae	*Leptothorax kutteri*	Buschinger, 1966	
Myrmicinae	*Leptothorax muscorum*	(Nylander, 1846)	
Myrmicinae	*Leptothorax pacis*	(Kutter, 1945)	
Myrmicinae	*Manica rubida*	(Latreille, 1802)	
Myrmicinae	*Messor atanassovii*	Atanassov, 1982	
Myrmicinae	*Messor barbarus*	(Linnaeus, 1767)	
Myrmicinae	*Messor bouvieri*	Bondroit, 1918	
Myrmicinae	*Messor capitatus*	(Latreille, 1798)	
Myrmicinae	*Messor kardamenae*	Salata & Borowiec, 2023	
Myrmicinae	*Messor carpathous*	Menozzi, 1936	
Myrmicinae	*Messor celiae*	Reyes, 1985	
Myrmicinae	*Messor concolor*	Santschi, 1927	
Myrmicinae	*Messor creticus*	Salata & Borowiec, 2019	
Myrmicinae	*Messor danaes*	Salata, Georgiadis, Borowiec, 2023	
Myrmicinae	*Messor erwini*	Orou, Csősz, Arnan, Pol, Arthofe, Schlick-Steiner & Steiner 2023	
Myrmicinae	*Messor hellenius*	Agosti & Collingwood, 1987	
Myrmicinae	*Messor hispanicus*	Santschi, 1919	
Myrmicinae	*Messor ibericus*	Santschi, 1931	
Myrmicinae	*Messor lobicornis*	Forel, 1894	
Myrmicinae	*Messor lusitanicus*	Tinaut, 1985	
Myrmicinae	*Messor marocanus*	Santschi, 1927	

Table S2: Checklist of European ant species (compiled up to 31st December 2025). The Introduced column indicates non-native species with established outdoor populations (Y), those restricted to indoor environments (I), and species with a doubtful status regarding their non-native origin in Europe (D). *(continued)*

Subfamily	Species	Author	Introduced
Myrmicinae	*Messor mcarthuri*	Steiner, Csősz, Markó, Gamisch, Rinnhofer, Folterbauer, Hammerle, Stauffer, Arthofer & Schlick-Steiner, 2018	
Myrmicinae	*Messor minor*	(André, 1883)	
Myrmicinae	*Messor muticus*	(Nylander, 1849)	
Myrmicinae	*Messor oertzeni*	Forel, 1910	
Myrmicinae	*Messor ponticus*	Steiner, Csősz, Markó, Gamisch, Rinnhofer, Folterbauer, Hammerle, Stauffer, Arthofer & Schlick-Steiner, 2018	
Myrmicinae	*Messor sanctus*	Emery, 1921	
Myrmicinae	*Messor sordidus*	Santschi, 1917	
Myrmicinae	*Messor structor*	(Latreille, 1798)	
Myrmicinae	*Messor timidus*	Espadaler, 1997	
Myrmicinae	*Messor varrialei*	Emery, 1921	
Myrmicinae	*Messor veneris*	Salata, Georgiadis, Borowiec, 2023	
Myrmicinae	*Messor wasmanni*	Krausse, 1910	
Myrmicinae	*Monomorium algiricum*	(Bernard, 1955)	
Myrmicinae	*Monomorium andrei*	Saunders, E., 1890	
Myrmicinae	*Monomorium bicolor*	Emery, 1877	Y
Myrmicinae	*Monomorium carbonarium*	(Smith, F., 1858)	Y
Myrmicinae	*Monomorium creticum*	Emery, 1895	
Myrmicinae	*Monomorium exiguum*	Forel, 1894	Y
Myrmicinae	*Monomorium floricola*	(Jerdon, 1851)	Y
Myrmicinae	*Monomorium gallicum*	Seifert 2025	
Myrmicinae	*Monomorium monomorium*	Bolton, 1987	
Myrmicinae	*Monomorium pharaonis*	(Linnaeus, 1758)	I
Myrmicinae	*Monomorium sahlbergi*	Emery, 1898	I
Myrmicinae	*Monomorium salomonis*	(Linnaeus, 1758)	
Myrmicinae	*Monomorium sommieri*	Emery, 1908	
Myrmicinae	*Monomorium subopacum*	(Smith, F., 1858)	
Myrmicinae	*Myrmecina graminicola*	(Latreille, 1802)	
Myrmicinae	*Myrmecina melonii*	Rigato, 1999	
Myrmicinae	*Myrmecina sicula*	André, 1882	
Myrmicinae	*Myrmica aloba*	Forel, 1909	
Myrmicinae	*Myrmica babiensis*	García, Cuesta-Segura & Espadaler, 2024	
Myrmicinae	*Myrmica bergi*	Ruzsky, 1902	
Myrmicinae	*Myrmica bibikoffi*	Kutter, 1963	
Myrmicinae	*Myrmica constricta*	Karavaiev, 1934	
Myrmicinae	*Myrmica curvithorax*	Bondroit, 1920	
Myrmicinae	*Myrmica deplanata*	Emery, 1921	
Myrmicinae	*Myrmica gallienii*	Bondroit, 1920	
Myrmicinae	*Myrmica hellenica*	Finzi, 1926	
Myrmicinae	*Myrmica hirsuta*	Elmes, 1978	
Myrmicinae	*Myrmica karavajevi*	(Arnol'di, 1930)	
Myrmicinae	*Myrmica kozakorum*	Radchenko & Elmes, 2010	
Myrmicinae	*Myrmica laurae*	(Emery, 1907)	
Myrmicinae	*Myrmica lemasnei*	Bernard, 1967	
Myrmicinae	*Myrmica lobicornis*	Nylander, 1846	

Table S2: Checklist of European ant species (compiled up to 31st December 2025). The Introduced column indicates non-native species with established outdoor populations (Y), those restricted to indoor environments (I), and species with a doubtful status regarding their non-native origin in Europe (D). *(continued)*

Subfamily	Species	Author	Introduced
Myrmicinae	*Myrmica lobulicornis*	Nylander, 1857	
Myrmicinae	*Myrmica lonae*	Finzi, 1926	
Myrmicinae	*Myrmica microrubra*	Seifert, 1993	
Myrmicinae	*Myrmica myrmicoxena*	Forel, 1895	
Myrmicinae	*Myrmica obscura*	Finzi, 1926	
Myrmicinae	*Myrmica pelops*	Seifert, 2003	
Myrmicinae	*Myrmica ravasinii*	Finzi, 1923	
Myrmicinae	*Myrmica rubra*	(Linnaeus, 1758)	
Myrmicinae	*Myrmica ruginodis*	Nylander, 1846	
Myrmicinae	*Myrmica rugulosa*	Nylander, 1849	
Myrmicinae	*Myrmica sabuleti*	Meinert, 1861	
Myrmicinae	*Myrmica scabrinodis*	Nylander, 1846	
Myrmicinae	*Myrmica schencki*	Viereck, 1903	
Myrmicinae	*Myrmica siciliana*	Radchenko, Elmes & Alicata, 2006	
Myrmicinae	*Myrmica specioides*	Bondroit, 1918	
Myrmicinae	*Myrmica spinosior*	Santschi, 1931	
Myrmicinae	*Myrmica stangeana*	Ruzsky, 1902	
Myrmicinae	*Myrmica sulcinodis*	Nylander, 1846	
Myrmicinae	*Myrmica tulinae*	Elmes, Radchenko & Aktaç, 2002	
Myrmicinae	*Myrmica vandeli*	Bondroit, 1920	
Myrmicinae	*Myrmica wesmaeli*	Bondroit, 1918	
Myrmicinae	*Myrmica xavieri*	Radchenko, Elmes & Savolainen, 2008	
Myrmicinae	*Oxyopomyrmex arenarium*	Reyes-López 2025	
Myrmicinae	*Oxyopomyrmex krueperi*	Forel, 1911	
Myrmicinae	*Oxyopomyrmex laevibus*	Salata & Borowiec, 2015	
Myrmicinae	*Oxyopomyrmex magnus*	Salata & Borowiec, 2015	
Myrmicinae	*Oxyopomyrmex pallens*	Reyes-López 2025	
Myrmicinae	*Oxyopomyrmex polybotesi*	Salata & Borowiec, 2015	
Myrmicinae	*Oxyopomyrmex saulcyi*	Emery, 1889	
Myrmicinae	*Pheidole anastasii*	Emery, 1896	I
Myrmicinae	*Pheidole balcanica*	Seifert, 2016	
Myrmicinae	*Pheidole bilimeki*	Mayr, 1870	I
Myrmicinae	*Pheidole indica*	Mayr, 1879	Y
Myrmicinae	*Pheidole keftiuensis*	Borowiec & Salata, 2025	D
Myrmicinae	*Pheidole koshewnikovi*	Ruzsky, 1905	
Myrmicinae	*Pheidole megacephala*	(Fabricius, 1793)	Y
Myrmicinae	*Pheidole navigans*	Forel, 1901	I
Myrmicinae	*Pheidole pallidula*	(Nylander, 1849)	
Myrmicinae	*Pheidole punctatissima*	Mayr, 1870	I
Myrmicinae	*Solenopsis abdita*	Thompson, 1989	I
Myrmicinae	*Solenopsis crivellarii*	Menozzi, 1936	
Myrmicinae	*Solenopsis debilior*	Santschi, 1934	
Myrmicinae	*Solenopsis fairchildi*	Wheeler, W.M., 1926	
Myrmicinae	*Solenopsis fugax*	(Latreille, 1798)	
Myrmicinae	*Solenopsis juliae*	(Arakelian, 1991)	
Myrmicinae	*Solenopsis gallica*	Santschi, 1934	
Myrmicinae	*Solenopsis ilinei*	Santschi, 1936	
Myrmicinae	*Solenopsis invicta*	Buren, 1972	Y

Table S2: Checklist of European ant species (compiled up to 31st December 2025). The Introduced column indicates non-native species with established outdoor populations (Y), those restricted to indoor environments (I), and species with a doubtful status regarding their non-native origin in Europe (D). *(continued)*

Subfamily	Species	Author	Introduced
Myrmicinae	*Solenopsis latro*	Forel, 1894	
Myrmicinae	*Solenopsis lusitanica*	Emery, 1915	
Myrmicinae	*Solenopsis oraniensis*	Forel, 1894	
Myrmicinae	*Solenopsis orbula*	Emery, 1875	
Myrmicinae	*Solenopsis santschii*	Forel, 1905	
Myrmicinae	*Solenopsis texana*	Emery, 1895	I
Myrmicinae	*Solenopsis wolfi*	Emery, 1915	
Myrmicinae	*Stenamma debile*	(Foerster, 1850)	
Myrmicinae	*Stenamma petiolatum*	Emery, 1897	
Myrmicinae	*Stenamma punctiventre*	Emery, 1908	
Myrmicinae	*Stenamma sardoum*	Emery, 1915	
Myrmicinae	*Stenamma siculum*	Rigato, 2011	
Myrmicinae	*Stenamma striatulum*	Emery, 1895	
Myrmicinae	*Stenamma westwoodii*	Westwood, 1839	
Myrmicinae	*Stenamma zanoni*	Rigato, 2011	
Myrmicinae	*Strongylognathus afer*	Emery, 1884	
Myrmicinae	*Strongylognathus alpinus*	Wheeler, W.M., 1909	
Myrmicinae	*Strongylognathus arnoldii*	Radchenko, 1985	
Myrmicinae	*Strongylognathus caeciliae*	Forel, 1897	
Myrmicinae	*Strongylognathus chelifer*	Radchenko, 1985	
Myrmicinae	*Strongylognathus christophi*	Emery, 1889	
Myrmicinae	*Strongylognathus destefanii*	Emery, 1915	
Myrmicinae	*Strongylognathus huberi*	Forel, 1874	
Myrmicinae	*Strongylognathus insularis*	Baroni Urbani, 1968	
Myrmicinae	*Strongylognathus italicus*	Finzi, 1924	
Myrmicinae	*Strongylognathus karawajewi*	Pisarski, 1966	
Myrmicinae	*Strongylognathus kratochvili*	Silhavy, 1937	
Myrmicinae	*Strongylognathus pisarskii*	Poldi, 1994	
Myrmicinae	*Strongylognathus silvestrii*	Menozzi, 1936	
Myrmicinae	*Strongylognathus testaceus*	(Schenck, 1852)	
Myrmicinae	*Strumigenys argiola*	(Emery, 1869)	
Myrmicinae	*Strumigenys baudueri*	(Emery, 1875)	
Myrmicinae	*Strumigenys membranifera*	Emery, 1869	Y
Myrmicinae	*Strumigenys perplexa*	(Smith 1876)	Y
Myrmicinae	*Strumigenys rogeri*	Emery, 1890	I
Myrmicinae	*Strumigenys silvestrii*	Emery, 1906	Y
Myrmicinae	*Strumigenys tenuipilis*	Emery, 1915	
Myrmicinae	*Strumigenys tenuissima*	(Brown, 1953)	
Myrmicinae	*Temnothorax adlerzi*	(Douwes, Jessen & Buschinger, 1988)	
Myrmicinae	*Temnothorax aeolius*	(Forel, 1911)	
Myrmicinae	*Temnothorax affinis*	(Mayr, 1855)	
Myrmicinae	*Temnothorax albipennis*	(Curtis, 1854)	
Myrmicinae	*Temnothorax alfacarensis*	Tinaut & Reyes-López, 2020	
Myrmicinae	*Temnothorax algerianus*	(Cagniant, 1968)	
Myrmicinae	*Temnothorax algiricus*	(Forel, 1894)	
Myrmicinae	*Temnothorax alienus*	Schulz, Heinze & Pusch, 2007	
Myrmicinae	*Temnothorax angulinodis*	Csösz, Heinze & Mikó, 2015	
Myrmicinae	*Temnothorax angustifrons*	Csösz, Heinze & Mikó, 2015	

Table S2: Checklist of European ant species (compiled up to 31st December 2025). The Introduced column indicates non-native species with established outdoor populations (Y), those restricted to indoor environments (I), and species with a doubtful status regarding their non-native origin in Europe (D). *(continued)*

Subfamily	Species	Author	Introduced
Myrmicinae	*Temnothorax angustulus*	(Nylander, 1856)	
Myrmicinae	*Temnothorax ansei*	Catarineu, Barberá & Reyes-López, 2017	
Myrmicinae	*Temnothorax antigoni*	(Forel, 1911)	
Myrmicinae	*Temnothorax apenninicus*	Csősz, Schifani, Seifert, Alicata & Prebus, 2024	
Myrmicinae	*Temnothorax arcanus*	(Kutter, 1973)	
Myrmicinae	*Temnothorax ariadnae*	Csösz, Heinze & Mikó, 2015	
Myrmicinae	*Temnothorax arkasi*	Salata & Borowiec, 2022	
Myrmicinae	*Temnothorax atlantis*	(Santschi, 1911)	
Myrmicinae	*Temnothorax aveli*	(Bondroit, 1918)	
Myrmicinae	*Temnothorax baeticus*	(Emery, 1924)	
Myrmicinae	*Temnothorax bejaraniensis*	Reyes-López & Carpintero-Ortega, 2013	
Myrmicinae	*Temnothorax bernardi*	(Espadaler, 1982)	
Myrmicinae	*Temnothorax blascoi*	(Espadaler, 1997)	
Myrmicinae	*Temnothorax brackoi*	Salata & Borowiec, 2019	
Myrmicinae	*Temnothorax bulgaricus*	(Forel, 1892)	
Myrmicinae	*Temnothorax caesari*	(Espadaler, 1997)	
Myrmicinae	*Temnothorax cagnianti*	(Tinaut, 1983)	
Myrmicinae	*Temnothorax clypeatus*	(Mayr, 1853)	
Myrmicinae	*Temnothorax conatensis*	Galkowski & Lebas, 2016	
Myrmicinae	*Temnothorax continentalis*	Galkowski & Cagniant, 2017	
Myrmicinae	*Temnothorax convexus*	(Forel, 1894)	
Myrmicinae	*Temnothorax corsicus*	(Emery, 1895)	
Myrmicinae	*Temnothorax corticalis*	(Schenck, 1852)	
Myrmicinae	*Temnothorax crasecundus*	Seifert & Csősz, 2015	
Myrmicinae	*Temnothorax crassispinus*	(Karavaiev, 1926)	
Myrmicinae	*Temnothorax crassistriatus*	Salata, Borowiec & Trichas, 2018	
Myrmicinae	*Temnothorax crepuscularis*	(Tinaut, 1994)	
Myrmicinae	*Temnothorax cristinae*	(Espadaler, 1997)	
Myrmicinae	*Temnothorax curtisetosus*	Salata & Borowiec, 2015	
Myrmicinae	*Temnothorax curtulus*	(Santschi, 1929)	
Myrmicinae	*Temnothorax daidalosi*	Salata, Borowiec & Trichas, 2018	
Myrmicinae	*Temnothorax dessyi*	(Menozzi, 1936)	
Myrmicinae	*Temnothorax eocho*	(Borowiec & Salata, 2025)	
Myrmicinae	*Temnothorax estel*	Arcos González 2021	
Myrmicinae	*Temnothorax euboeae*	Salata & Borowiec, 2022	
Myrmicinae	*Temnothorax exilis*	(Emery, 1869)	
Myrmicinae	*Temnothorax finzii*	(Menozzi, 1925)	
Myrmicinae	*Temnothorax flavicornis*	(Emery, 1870)	
Myrmicinae	*Temnothorax flavispinus*	(André, 1883)	
Myrmicinae	*Temnothorax fuentei*	(Santschi, 1909)	
Myrmicinae	*Temnothorax gordiagini*	(Ruzsky, 1902)	
Myrmicinae	*Temnothorax gallei*	Csősz, Taheri, Schifani, Reyes-López, Alicata, Báthori & Prebus, 2025	
Myrmicinae	*Temnothorax graecus*	(Forel, 1911)	
Myrmicinae	*Temnothorax gredosi*	(Espadaler & Collingwood, 1982)	
Myrmicinae	*Temnothorax grouvellei*	(Bondroit, 1918)	
Myrmicinae	*Temnothorax helenae*	Csösz, Heinze & Mikó, 2015	
Myrmicinae	*Temnothorax ibericus*	(Menozzi, 1922)	

Table S2: Checklist of European ant species (compiled up to 31st December 2025). The Introduced column indicates non-native species with established outdoor populations (Y), those restricted to indoor environments (I), and species with a doubtful status regarding their non-native origin in Europe (D). *(continued)*

Subfamily	Species	Author	Introduced
Myrmicinae	*Temnothorax ikarosi*	Salata, Borowiec & Trichas, 2018	
Myrmicinae	*Temnothorax incompletus*	Salata, Borowiec & Trichas, 2018	
Myrmicinae	*Temnothorax inquilinus*	Ward, Brady, Fisher & Schultz, 2014	
Myrmicinae	*Temnothorax interruptus*	(Schenck, 1852)	
Myrmicinae	*Temnothorax italicus*	(Consani, 1952)	
Myrmicinae	*Temnothorax jailensis*	(Arnol'di, 1977)	
Myrmicinae	*Temnothorax kemali*	(Santschi, 1934)	
Myrmicinae	*Temnothorax kraussei*	(Emery, 1915)	
Myrmicinae	*Temnothorax kutteri*	(Cagniant, 1973)	
Myrmicinae	*Temnothorax laconicus*	Csősz, Seifert, Müller, Trindl, Schulz. & Heinze, 2013	
Myrmicinae	*Temnothorax laestrygon*	(Santschi, 1931)	
Myrmicinae	*Temnothorax lagrecai*	(Baroni Urbani, 1964)	
Myrmicinae	*Temnothorax leviceps*	(Emery, 1898)	
Myrmicinae	*Temnothorax lichtensteini*	(Bondroit, 1918)	
Myrmicinae	*Temnothorax longispinosus*	(Roger, 1863)	Y
Myrmicinae	*Temnothorax lucidus*	Csösz, Heinze & Mikó, 2015	
Myrmicinae	*Temnothorax luteus*	(Forel, 1874)	
Myrmicinae	*Temnothorax marae*	Alicata, Schifani & Prebus, 2022	
Myrmicinae	*Temnothorax melas*	(Espadaler, Plateaux & Casevitz-Weulersse, 1984)	
Myrmicinae	*Temnothorax minotaurosi*	Salata, Borowiec & Trichas, 2018	
Myrmicinae	*Temnothorax minozzii*	(Santschi, 1922)	
Myrmicinae	*Temnothorax morea*	Csősz, Salata & Borowiec, 2018	
Myrmicinae	*Temnothorax muellerianus*	(Finzi, 1922)	
Myrmicinae	*Temnothorax mytilenes*	Salata, Srodon & Boroweic, 2023	
Myrmicinae	*Temnothorax nadigi*	(Kutter, 1925)	
Myrmicinae	*Temnothorax naeviventris*	(Santschi, 1910)	
Myrmicinae	*Temnothorax nassonovi*	(Ruzsky, 1895)	
Myrmicinae	*Temnothorax nebulosus*	Zięcina, Salata, Borowiec 2025	
Myrmicinae	*Temnothorax niger*	(Forel, 1894)	
Myrmicinae	*Temnothorax nigriceps*	(Mayr, 1855)	
Myrmicinae	*Temnothorax nylanderi*	(Foerster, 1850)	
Myrmicinae	*Temnothorax pardoi*	(Tinaut, 1987)	
Myrmicinae	*Temnothorax parnonensis*	Salata & Borowiec, 2022	
Myrmicinae	*Temnothorax parvulus*	(Schenck, 1852)	
Myrmicinae	*Temnothorax pelagosanus*	(Müller, 1923)	
Myrmicinae	*Temnothorax phaetoni*	Salata, Srodon & Boroweic, 2023	
Myrmicinae	*Temnothorax platycephalus*	(Espadaler, 1997)	
Myrmicinae	*Temnothorax poldii*	Alicata, Schifani & Prebus, 2022	
Myrmicinae	*Temnothorax proteii*	Salata, Borowiec & Trichas, 2018	
Myrmicinae	*Temnothorax racovitzai*	(Bondroit, 1918)	
Myrmicinae	*Temnothorax ravouxi*	(André, 1896)	
Myrmicinae	*Temnothorax recedens*	(Nylander, 1856)	
Myrmicinae	*Temnothorax rogeri*	Emery, 1869	
Myrmicinae	*Temnothorax rottenbergii*	(Emery, 1870)	
Myrmicinae	*Temnothorax rougeti*	(Bondroit, 1918)	
Myrmicinae	*Temnothorax sappho*	Zięcina, Salata, Borowiec 2025	
Myrmicinae	*Temnothorax sardous*	(Santschi, 1909)	
Myrmicinae	*Temnothorax saxatilis*	Schulz, Heinze & Pusch, 2007	

Table S2: Checklist of European ant species (compiled up to 31st December 2025). The Introduced column indicates non-native species with established outdoor populations (Y), those restricted to indoor environments (I), and species with a doubtful status regarding their non-native origin in Europe (D). *(continued)*

Subfamily	Species	Author	Introduced
Myrmicinae	*Temnothorax saxonicus*	(Seifert, 1995)	
Myrmicinae	*Temnothorax schaufussi*	(Forel, 1879)	
Myrmicinae	*Temnothorax semenovi*	(Ruzsky, 1903)	
Myrmicinae	*Temnothorax balcanicus*	(André, 1881)	
Myrmicinae	*Temnothorax siculus*	Schifani, Alicata, Prebus, Csősz, 2025	
Myrmicinae	*Temnothorax smyrnensis*	(Forel, 1911)	
Myrmicinae	*Temnothorax solerii*	(Menozzi, 1936)	
Myrmicinae	*Temnothorax sordidulus*	(Müller, 1923)	
Myrmicinae	*Temnothorax splendidiceps*	(Baroni Urbani, 1968)	
Myrmicinae	*Temnothorax strymonensis*	Csősz, Salata & Borowiec, 2018	
Myrmicinae	*Temnothorax stumperi*	(Kutter, 1950)	
Myrmicinae	*Temnothorax subtilis*	Csösz, Heinze & Mikó, 2015	
Myrmicinae	*Temnothorax tamarae*	(Arnol'di, 1968)	
Myrmicinae	*Temnothorax tergestinus*	(Finzi, 1928)	
Myrmicinae	*Temnothorax triangularis*	Salata & Borowiec, 2019	
Myrmicinae	*Temnothorax tuberum*	(Fabricius, 1775)	
Myrmicinae	*Temnothorax turcicus*	(Santschi, 1934)	
Myrmicinae	*Temnothorax tyndalei*	(Forel, 1909)	
Myrmicinae	*Temnothorax unifasciatus*	(Latreille, 1798)	
Myrmicinae	*Temnothorax universitatis*	(Espadaler, 1997)	
Myrmicinae	*Temnothorax variabilis*	Salata, Borowiec & Trichas, 2018	
Myrmicinae	*Temnothorax vivianoi*	Schifani, Alicata & Prebus, 2022	
Myrmicinae	*Temnothorax volgensis*	(Ruzsky, 1905)	
Myrmicinae	*Temnothorax zaleskyi*	(Sadil, 1953)	
Myrmicinae	*Tetramorium albenae*	Salata, van Delft & Borowiec 2023	
Myrmicinae	*Tetramorium alpestre*	Steiner, Schlick-Steiner & Seifert, 2010	
Myrmicinae	*Tetramorium alternans*	Santschi, 1929	
Myrmicinae	*Tetramorium atratulum*	(Schenck, 1852)	
Myrmicinae	*Tetramorium bicarinatum*	(Nylander, 1846)	Y
Myrmicinae	*Tetramorium biskrense*	Forel, 1904	
Myrmicinae	*Tetramorium brevicorne*	Bondroit, 1918	
Myrmicinae	*Tetramorium breviscapus*	Wagner, Arthofer, Seifert, Muster, Steiner & Schlick-Steiner, 2017	
Myrmicinae	*Tetramorium buschingeri*	(Lapeva-Gjonova, 2017)	
Myrmicinae	*Tetramorium caespitum*	(Linnaeus, 1758)	
Myrmicinae	*Tetramorium caldarium*	(Roger, 1857)	Y
Myrmicinae	*Tetramorium chefketi*	Forel, 1911	
Myrmicinae	*Tetramorium davidi*	Forel, 1911	
Myrmicinae	*Tetramorium diomedeum*	Emery, 1908	
Myrmicinae	*Tetramorium exasperatum*	Emery, 1891	
Myrmicinae	*Tetramorium ferox*	Ruzsky, 1903	
Myrmicinae	*Tetramorium forte*	Forel, 1904	
Myrmicinae	*Tetramorium fusciclava*	Consani & Zangheri, 1952	
Myrmicinae	*Tetramorium galaticum*	Menozzi, 1936	
Myrmicinae	*Tetramorium hippocratis*	Agosti & Collingwood, 1987	
Myrmicinae	*Tetramorium hungaricum*	Röszler, 1935	
Myrmicinae	*Tetramorium immigrans*	Santschi, 1927	Y
Myrmicinae	*Tetramorium impurum*	(Foerster, 1850)	

Table S2: Checklist of European ant species (compiled up to 31st December 2025). The Introduced column indicates non-native species with established outdoor populations (Y), those restricted to indoor environments (I), and species with a doubtful status regarding their non-native origin in Europe (D). *(continued)*

Subfamily	Species	Author	Introduced
Myrmicinae	*Tetramorium indocile*	Santschi, 1927	
Myrmicinae	*Tetramorium inquilinum*	Ward, Brady, Fisher & Schultz, 2014	
Myrmicinae	*Tetramorium insolens*	(Smith, F., 1861)	I
Myrmicinae	*Tetramorium kephalosi*	Salata & Borowiec, 2017	
Myrmicinae	*Tetramorium kutteri*	(Tinaut, 1990)	
Myrmicinae	*Tetramorium lanuginosum*	Mayr, 1870	Y
Myrmicinae	*Tetramorium lucayanum*	Wheeler, W.M., 1905	I
Myrmicinae	*Tetramorium meridionale*	Emery, 1870	
Myrmicinae	*Tetramorium moravicum*	Kratochvíl, 1941	
Myrmicinae	*Tetramorium pacificum*	Mayr, 1870	I
Myrmicinae	*Tetramorium pelagium*	Poldi, 1995	
Myrmicinae	*Tetramorium punctatum*	Santschi, 1927	
Myrmicinae	*Tetramorium punicum*	(Smith, F., 1861)	
Myrmicinae	*Tetramorium rhodium*	Emery, 1924	
Myrmicinae	*Tetramorium sahlbergi*	Finzi, 1936	
Myrmicinae	*Tetramorium sanetrai*	Schulz & Csösz, 2007	
Myrmicinae	*Tetramorium schmidti*	Forel, 1904	
Myrmicinae	*Tetramorium semilaeve*	André, 1883	
Myrmicinae	*Tetramorium splendens*	Ruzsky, 1902	
Myrmicinae	*Tetramorium staerckei*	Kratochvíl, 1944	
Myrmicinae	*Tetramorium sulcinode*	Santschi, 1927	
Myrmicinae	*Trichomyrmex destructor*	(Jerdon, 1851)	Y
Myrmicinae	*Trichomyrmex mayri*	(Forel, 1902)	Y
Myrmicinae	*Trichomyrmex perplexus*	(Radchenko, 1997)	
Myrmicinae	*Wasmannia auropunctata*	(Roger, 1863)	Y
Ponerinae	*Anochetus ghilianii*	(Spinola, 1851)	Y
Ponerinae	*Anochetus mayri*	Emery, 1884	I
Ponerinae	*Brachyponera chinensis*	(Emery, 1895)	Y
Ponerinae	*Cryptopone ochracea*	(Mayr, 1855)	
Ponerinae	*Hypoponera abeillei*	(André, 1881)	
Ponerinae	*Hypoponera eduardi*	(Forel, 1894)	
Ponerinae	*Hypoponera ergatandria*	(Forel, 1893)	Y
Ponerinae	*Hypoponera punctatissima*	(Roger, 1859)	Y
Ponerinae	*Hypoponera ragusai*	(Emery, 1894)	
Ponerinae	*Ponera coarctata*	(Latreille, 1802)	
Ponerinae	*Ponera testacea*	Emery, 1895	
Proceratiinae	*Proceratium algiricum*	Forel, 1899	
Proceratiinae	*Proceratium melinum*	(Roger, 1860)	
Proceratiinae	*Proceratium melitense*	De Andrade, 2003	
Proceratiinae	*Proceratium numidicum*	Santschi, 1912	

The sequence data

Table S3: European species sequenced. For each species the following information is provided: barcoding sharing detected (BS); monophyly, polyphyly or paraphyly detected and singletons (Group); retrieved (R) and/or de novo (N) sequences (Source); number total sequences (All) and only those above 599bp (Long), with distinction between identified - cf. levels; maximum of the intraspecific p-dist applying a strict criterion (MaxIntra) and those with a less strict criterion (MaxIntraCF); median of the intraspecific p-dist (MedIntra); minimum of the interspecific p-dist (MinInter); median of the minimum values of interspecific p-dist obtained for each specimen (MedMinInter).

			Sequences			p-dist				
Taxon	BS	Group	Source	All	Long	MaxIntra	MaxIntraCF	MedIntra	MinInter	MedMinInter
Stigmatomma denticulatum		mono	N	**5-0**	5-0	9.5	9.5	0	17.1	17.2
Stigmatomma emeryi				**0**	0					
Stigmatomma gaetulicum				**0**	0					
Stigmatomma impressifrons		mono	N	**3-0**	3-0	0	0	0	17.1	17.1
Bothriomyrmex atlantis		para	N	**3-0**	3-0	5.3	5.3	5.3	0.3	5
Bothriomyrmex communista		mono	N	**9-0**	9-0	2.1	2.1	1.5	5.4	5.7
Bothriomyrmex corsicus		mono	N	**6-0**	6-0	0.9	0.9	0.5	5.4	5.4
Bothriomyrmex meridionalis		para	N	**6-0**	6-0	1.2	1.2	0.9	0.3	0.6
Bothriomyrmex modestus				**0**	0					
Dolichoderus quadripunctatus		mono	RN	**56-0**	48-0	4.1	4.5	0.5		
Linepithema angulatum		mono	R	**1-0**	0					
Linepithema iniquum		mono	R	**1-0**	1-0					
Linepithema humile		mono	RN	**24-0**	24-0	1.1	1.1	0		
Liometopum microcephalum		mono	RN	**29-0**	29-0	1.1	1.1	0.5		
Tapinoma darioi	Y	poly	R	**34-10**	33-10	4.1	4.3	0.6	0	2.7
Tapinoma erraticum		mono	RN	**23-0**	23-0	2	2	0.5	4.4	4.7
Tapinoma festae		mono	N	**1-0**	1-0				7.8	11.5
Tapinoma glabrella		mono	N	**2-13**	2-13		4.6		4.7	5.2
Tapinoma hispanicum		mono	R	**1-0**	1-0				2.4	5.3
Tapinoma ibericum	Y	poly	RN	**15-4**	15-4	5.1	5.1	1.8	0	2.3
Tapinoma insularis		para	N	**4-1**	4-1	9.2	9.3	8.3	6	6.7
Tapinoma kinburni				**0**	0					
Tapinoma madeirense		mono	RN	**18-1**	18-1	1.4	1.4	0.6	3.7	4
Tapinoma magnum	Y	poly	RN	**70-12**	68-12	4.6	4.6	1.5	0	0.3
Tapinoma melanocephalum		mono	R	**1-0**	1-0					
Tapinoma nigerrimum		poly	RN	**17-3**	15-3	1.5	6.3	0.5	2.2	2.3
Tapinoma pithecorum				**0**	0					
Tapinoma pygmaeum		mono	N	**1-0**	1-0				10.9	12.5
Tapinoma sessile		mono	R	**2-0**	2-0					
Tapinoma simrothi		mono	RN	**4-0**	4-0	0.3	0.3	0.2	6	6
Tapinoma subboreale		mono	RN	**20-0**	20-0	2.3	2.3	1.4	3.7	4.6
Technomyrmex albipes		mono	RN	**6-0**	6-0				13.9	15.2
Technomyrmex difficilis		poly	R	**4-0**	4-0				13.9	17.7
Technomyrmex pallipes		poly	R	**7-0**	7-0					
Technomyrmex vexatus				**0**	0					
Technomyrmex vitiensis		mono	RN	**4-0**	4-0	0.2	0.2	0.1	15.1	15.2

Table S3: European species sequenced. For each species the following information is provided: barcoding sharing detected (BS); monophyly, polyphyly or paraphyly detected and singletons (Group); retrieved (R) and/or de novo (N) sequences (Source); number total sequences (All) and only those above 599bp (Long), with distinction between identified - cf. levels; maximum of the intraspecific p-dist applying a strict criterion (MaxIntra) and those with a less strict criterion (MaxIntraCF); median of the intraspecific p-dist (MedIntra); minimum of the interspecific p-dist (MinInter); median of the minimum values of interspecific p-dist obtained for each specimen (MedMinInter). *(continued)*

			**Sequences**			**p-dist**				
Taxon	BS	Group	Source	All	Long	MaxIntra	MaxIntraCF	MedIntra	MinInter	MedMinInter
Aenictus rhodiensis		mono	N	**1-0**	1-0					
Acropyga paleartica		mono	N	**1-0**	1-0					
Brachymyrmex patagonicus		mono	RN	**3-0**	3-0		0			
Camponotus aegaeus		mono	N	**6-0**	6-0	0.8	0.8	0.5	12.9	13
Camponotus aethiops		mono	RN	**73-0**	72-0	9.6	9.6	2.1	6.7	6.9
Camponotus amaurus	Y	para	N	**3-0**	3-0	1.8	1.8	1.7	0	0.2
Camponotus atriceps		mono	RN	**2-0**	2-0					
Camponotus atricolor		mono	N	**10-0**	10-0	0.9	0.9	0.3	4.3	4.4
Camponotus baldaccii		mono	N	**3-0**	3-0	4.7	4.7	4.7	9.5	10.3
Camponotus barbaricus		mono	N	**11-0**	10-0	6	6	4.6	6.1	7.8
Camponotus boghossiani		para	N	**4-0**	3-0	4.6	4.9	4.3	1.5	1.9
Camponotus candiotes		mono	N	**2-0**	2-0		5.8		7.8	7.9
Camponotus cruentatus		mono	N	**24-0**	24-0	0.5	0.5	0	9.5	9.5
Camponotus dalmaticus		mono	N	**14-0**	14-0	2.1	2.1	1.7	5.7	5.8
Camponotus fallax		mono	RN	**23-0**	23-0	4.1	4.1	1.5	12.7	13
Camponotus foreli	Y	para	RN	**15-0**	15-0	0.9	0.9	0.5	0	0.6
Camponotus gestroi		mono	N	**8-0**	8-0	11.5	11.5	10.6	9.7	10.1
Camponotus haroi				**0**	0					
Camponotus heidrunvogtae		mono	N	**1-0**	1-0				7.8	17.1
Camponotus herculeanus		mono	RN	**24-0**	24-0	1.2	1.2	0.5	6	6.3
Camponotus ionius		mono	N	**10-0**	10-0	2.2	2.2	0.2	14	14.7
Camponotus jaliensis		mono	N	**1-0**	0					
Camponotus kiesenwetteri		mono	N	**7-0**	6-0	3.7	3.7	3.1	8.6	8.7
Camponotus laconicus		mono	N	**3-0**	3-0	0.3	0.3	0.2	4.7	4.9
Camponotus lateralis		mono	RN	**56-0**	55-0	2.6	2.8	1.1	8.3	9
Camponotus ligniperda		mono	RN	**35-0**	35-0	1.8	1.8	0.2	6	6.7
Camponotus micans		para	RN	**8-0**	8-0	7	7	0.2	4.1	6.8
Camponotus nitidescens		para	N	**2-0**	2-0		6.6		2.6	2.8
Camponotus nylanderi		mono	RN	**17-0**	17-0	1.7	1.7	0.2	9.5	9.6
Camponotus oertzeni		para	N	**24-0**	24-0	11.6	11.6	9.2	6.1	8.4
Camponotus piceus		para	RN	**53-0**	53-0	6.5	6.5	0.8	4.3	5
Camponotus pilicornis		mono	RN	**18-0**	18-0	2.6	2.6	0.2	12.2	12.4
Camponotus rebeccae		mono	N	**1-0**	1-0				9.8	16.2
Camponotus ruber		mono	RN	**5-0**	3-0		1.3		1.3	17.5
Camponotus samius		mono	N	**7-0**	7-0	1.1	1.1	0.3	4.7	5.4
Camponotus sanctus		mono	N	**5-0**	5-0	1.1	1.1	0.6	9.5	9.8
Camponotus spissinodis		mono	N	**2-0**	2-0		5.2		10.1	10.5
Camponotus sylvaticus		mono	RN	**15-0**	15-0	0.5	0.5	0.2	6.1	6.3

Table S3: European species sequenced. For each species the following information is provided: barcoding sharing detected (BS); monophyly, polyphyly or paraphyly detected and singletons (Group); retrieved (R) and/or de novo (N) sequences (Source); number total sequences (All) and only those above 599bp (Long), with distinction between identified - cf. levels; maximum of the intraspecific p-dist applying a strict criterion (MaxIntra) and those with a less strict criterion (MaxIntraCF); median of the intraspecific p-dist (MedIntra); minimum of the interspecific p-dist (MinInter); median of the minimum values of interspecific p-dist obtained for each specimen (MedMinInter). *(continued)*

			Sequences			p-dist				
Taxon	BS	Group	Source	All	Long	MaxIntra	MaxIntraCF	MedIntra	MinInter	MedMinInter
Camponotus tergestinus		mono	N	**3-0**	3-0	1.4	1.4	0.8	12.9	13.2
Camponotus universitatis		mono	RN	**5-0**	4-0	3.8	3.9	2.9	6.1	6.6
Camponotus vagus		mono	RN	**54-0**	54-0	7	7	0.5	10.7	11.2
Cataglyphis cretica		para	N	**2-0**	2-0		6.9		5.4	5.8
Cataglyphis cursor		mono	N	**11-0**	11-0	3.7	3.7	3.4	4.9	5.7
Cataglyphis douwesi		mono	N	**1-0**	1-0				0.5	12.2
Cataglyphis floricola		para	R	**15-0**	15-0	3.5	3.5	1.9	1.1	2.2
Cataglyphis gadeai		mono	N	**2-2**	2-2		2.9		2.3	2.6
Cataglyphis hellenica		para	N	**10-1**	10-1	12.3	12.3	8.6	4.4	7.3
Cataglyphis hispanica		mono	N	**8-0**	7-0	10.5	12.9	9.2	8.6	9.2
Cataglyphis humeya		mono	N	**1-0**	1-0				2.6	13.5
Cataglyphis iberica		para	N	**17-2**	17-2	4.3	4.3	2.8	0.5	1.4
Cataglyphis italica		mono	N	**11-0**	11-0	5.8	5.8	4	4.3	4.6
Cataglyphis minos		mono	N	**1-0**	1-0				8.4	12.2
Cataglyphis nodus		mono	N	**14-0**	14-0	2.6	2.6	0.6	6.3	6.3
Cataglyphis piliscapa		mono	N	**21-0**	21-0	7.5	7.5	5.3	4.3	5.1
Cataglyphis rosenhaueri		poly	RN	**3-0**	2-0		1.7		0.5	0.5
Cataglyphis tartessica		mono	N	**1-0**	1-0				1.1	13.2
Cataglyphis velox		para	RN	**8-0**	7-0	8.4	8.4	6.6	2.6	6.6
Cataglyphis viatica		mono	N	**2-0**	2-0		1.2		7	7.2
Cataglyphis viaticoides		mono	N	**1-0**	1-0				6.6	12
Colobopsis truncata	Y	para	RN	**66-0**	63-0	2.3	2.3	0.3	0	0.8
Colobopsis imitans	Y	para	RN	**14-0**	14-0	2.5	2.5	0.2	0	0.2
Formica aquilonia	Y	poly	RN	**11-1**	11-1	1.3	1.3	0	0	0
Formica bruni	Y	mono	N	**1-0**	1-0				0	5.1
Formica cinerea	Y	para	RN	**25-1**	23-1	4	4	1.3	0	0.5
Formica cinereofusca				**0**	0					
Formica clara	Y	poly	RN	**26-4**	21-4	7.2	7.8	0.9	0	0
Formica corsica		para	N	**2-0**	2-0		0.6		0.2	0.2
Formica cunicularia	Y	para	RN	**54-2**	53-2	7	7	3.5	0	0
Formica decipiens	Y	para	N	**8-0**	8-0	2.6	2.6	0.6	0	1.6
Formica dusmeti	Y	poly	N	**3-0**	3-0	0	0	0	0	0
Formica exsecta	Y	para	RN	**33-0**	31-0	1.2	1.3	0.6	0	0.3
Formica fennica	Y	mono	R	**2-0**	2-0		0		0	0
Formica foreli		mono	N	**3-0**	3-0	0	0	0	0.8	0.8
Formica forsslundi		mono	RN	**2-0**	2-0		0		0.2	0.2
Formica frontalis	Y	para	RN	**6-0**	5-0	0.2	0.9	0	0	0

Table S3: European species sequenced. For each species the following information is provided: barcoding sharing detected (BS); monophyly, polyphyly or paraphyly detected and singletons (Group); retrieved (R) and/or de novo (N) sequences (Source); number total sequences (All) and only those above 599bp (Long), with distinction between identified - cf. levels; maximum of the intraspecific p-dist applying a strict criterion (MaxIntra) and those with a less strict criterion (MaxIntraCF); median of the intraspecific p-dist (MedIntra); minimum of the interspecific p-dist (MinInter); median of the minimum values of interspecific p-dist obtained for each specimen (MedMinInter). *(continued)*

			**Sequences**			**p-dist**				
Taxon	BS	Group	Source	All	Long	MaxIntra	MaxIntraCF	MedIntra	MinInter	MedMinInter
Formica fusca	Y	poly	RN	**96-1**	69-1	6.9	7.2	0.3	0	0
Formica fuscocinerea		mono	N	**7-0**	7-0	0	0	0	0.2	0.2
Formica gagates		mono	RN	**35-0**	34-0	4.6	4.6	0	8.6	9.5
Formica gagatoides		mono	RN	**5-0**	5-0	0.3	0.3	0.2	2.6	2.6
Formica gerardi	Y	mono	N	**9-0**	9-0	0.2	0.2	0	0	0
Formica helvetica				**0**	0					
Formica lemani	Y	para	RN	**40-0**	39-0	1.1	1.1	0.2	0	0
Formica lugubris	Y	para	RN	**28-2**	28-2	0.9	0.9	0.2	0	0.2
Formica paralugubris	Y	para	RN	**6-0**	5-0	0.5	0.5	0.2	0	0
Formica picea		mono	RN	**10-0**	10-0	1.4	1.4	0.5	2.6	2.7
Formica polyctena	Y	para	RN	**19-0**	19-0	1.5	1.5	0.2	0	0
Formica pratensis	Y	poly	RN	**28-1**	28-1	2	2	0.8	0	0.2
Formica pressilabris	Y	poly	RN	**15-0**	15-0	3.9	3.9	0.9	0	0.6
Formica pyrenaea	Y	mono	N	**1-0**	1-0				0	4.6
Formica rufa	Y	para	RN	**30-0**	29-0	1.7	1.7	0.2	0	0
Formica rufibarbis	Y	para	RN	**35-5**	26-5	4.6	6.5	0	0	0
Formica sanguinea		mono	RN	**36-0**	35-0	0.8	1.6	0.2	3	3.4
Formica selysi		poly	N	**5-0**	5-0	0.6	0.6	0.3	0.3	0.6
Formica suecica		mono	N	**2-0**	2-0		0		3.2	3.2
Formica truncorum		para	RN	**14-0**	13-0	1.1	1.1	0	0.3	1.1
Formica uralensis		mono	RN	**3-0**	3-0	0.2	0.2	0.2	3.7	3.7
Iberoformica subrufa		mono	RN	**23-0**	22-0	1.8	1.8	1.4		
Lasius alienus	Y	para	RN	**19-0**	18-0	0.9	0.9	0.5	0	0.2
Lasius austriacus		mono	RN	**7-0**	2-0		0.9		6.2	6.4
Lasius balcanicus		mono	N	**1-0**	0					
Lasius balearicus		mono	N	**3-0**	3-0	0.2	0.2	0.2	1.2	1.2
Lasius bicornis		mono	N	**1-0**	1-0				5.6	10.5
Lasius bombycinus		mono	RN	**7-0**	7-0	0.8	0.8	0.3	3.8	3.8
Lasius brunneus		mono	RN	**22-0**	21-0	2	2	0.6	5.4	5.8
Lasius carniolicus		para	N	**6-0**	5-0	0.6	0.8	0.5	1.7	1.7
Lasius casevitzi		mono	RN	**21-0**	20-0	0.8	0.9	0.2	0.9	1.1
Lasius cinereus	Y	poly	RN	**16-0**	16-0	0.9	0.9	0.3	0	0.3
Lasius citrinus	Y	poly	N	**4-0**	4-0	0.6	0.6	0.3	0	0
Lasius creticus	Y	mono	N	**1-0**	1-0				0	8.6
Lasius distinguendus	Y	para	RN	**10-0**	10-0	0.8	0.8	0.5	0	0
Lasius emarginatus	Y	poly	RN	**55-6**	54-6	2.9	3.2	0.5	0	0.3
Lasius flavus		mono	RN	**44-0**	43-0	1.5	1.5	0.3	3.5	3.8
Lasius fuliginosus		mono	RN	**32-0**	32-0	1.7	1.7	0.9	8	8.7

Table S3: European species sequenced. For each species the following information is provided: barcoding sharing detected (BS); monophyly, polyphyly or paraphyly detected and singletons (Group); retrieved (R) and/or de novo (N) sequences (Source); number total sequences (All) and only those above 599bp (Long), with distinction between identified - cf. levels; maximum of the intraspecific p-dist applying a strict criterion (MaxIntra) and those with a less strict criterion (MaxIntraCF); median of the intraspecific p-dist (MedIntra); minimum of the interspecific p-dist (MinInter); median of the minimum values of interspecific p-dist obtained for each specimen (MedMinInter). *(continued)*

			**Sequences**			**p-dist**				
Taxon	BS	Group	Source	All	Long	MaxIntra	MaxIntraCF	MedIntra	MinInter	MedMinInter
Lasius grandis	Y	para	RN	**26-0**	26-0	4.9	4.9	0.3	0	0.2
Lasius illyricus	Y	para	N	**19-1**	19-1	1.7	1.8	0.3	0	0
Lasius jensi		mono	N	**2-1**	2-1		1.2		2.3	2.3
Lasius karpinisi				**0**	0					
Lasius kritikos		mono	N	**2-0**	2-0				0.9	4.7
Lasius lasioides		mono	RN	**30-0**	28-0	5.3	7.8	0.5	5.4	5.6
Lasius longiceps				**0**	0					
Lasius maltaeus		mono	RN	**9-0**	9-0	0.6	0.6	0.3	2.6	2.7
Lasius meridionalis		mono	RN	**11-0**	11-0	1.1	1.1	0.5	2.3	2.4
Lasius mixtus		mono	N	**8-0**	8-0	1.1	1.1	0.8	5.4	5.8
Lasius myops		mono	RN	**42-0**	42-0	4.6	4.6	0.6	3.2	5.1
Lasius neglectus	Y	para	RN	**19-0**	17-0	4.3	4.9	0.2	0	3.8
Lasius niger		mono	RN	**64-0**	62-0	0.8	0.8	0	1.4	1.4
Lasius nitidigaster		poly	RN	**2-0**	2-0		3.2		2.3	2.6
Lasius paralienus	Y	para	RN	**21-0**	21-0	1.8	1.8	1.2	0	1.2
Lasius piliferus	Y	poly	RN	**8-0**	8-0	3.4	3.4	0.3	0	1.2
Lasius platythorax		mono	RN	**31-0**	29-0	0.7	0.7	0.2	1.5	1.5
Lasius precursor	Y	para	RN	**3-0**	3-0	0	0	0	0	0
Lasius psammophilus	Y	para	RN	**23-0**	22-0	3.7	3.7	0.5	0	0.5
Lasius rabaudi				**0**	0					
Lasius reginae		mono	N	**2-0**	2-0		0		1.7	1.7
Lasius sabularum	Y	poly	RN	**10-0**	10-0	6.2	6.2	0.9	0	0
Lasius tapinomoides	Y	mono	N	**1-0**	1-0				0	8.4
Lasius turcicus		para	R	**3-0**	0		2.1			
Lasius umbratus	Y	poly	RN	**14-1**	14-1	0.9	0.9	0.6	0	0
Lasius viehmeyeri				**0**	0					
Lepisiota capensis		mono	R	**3-0**	3-0					
Lepisiota frauenfeldi	Y	poly	RN	**23-1**	22-1	12.9	12.9	0.3	0	0.2
Lepisiota melas	Y	poly	N	**7-1**	7-1	10.1	10.1	8.3	0	0.2
Lepisiota nigra	Y	poly	RN	**10-0**	5-0	7.2	8.5	6.4	0	0.2
Lepisiota syriaca	Y	mono	N	**1-0**	1-0				0	6.2
Lepisiota velox	Y	para	N	**2-0**	2-0		0.2		0	0.1
Metalasius myrmidon		mono	N	**1-0**	1-0					
Nylanderia jaegerskioeldi		mono	RN	**14-0**	14-0	0.3	0.3	0	14.1	14.4
Nylanderia flavipes		mono	R	**1-0**	1-0				14.5	14.7
Nylanderia vividula		mono	N	**1-0**	1-0				14.1	14.4
Paratrechina longicornis		mono	RN	**6-0**	6-0	6	6	5.5		

Table S3: European species sequenced. For each species the following information is provided: barcoding sharing detected (BS); monophyly, polyphyly or paraphyly detected and singletons (Group); retrieved (R) and/or de novo (N) sequences (Source); number total sequences (All) and only those above 599bp (Long), with distinction between identified - cf. levels; maximum of the intraspecific p-dist applying a strict criterion (MaxIntra) and those with a less strict criterion (MaxIntraCF); median of the intraspecific p-dist (MedIntra); minimum of the interspecific p-dist (MinInter); median of the minimum values of interspecific p-dist obtained for each specimen (MedMinInter). *(continued)*

			**Sequences**			**p-dist**				
Taxon	BS	Group	Source	All	Long	MaxIntra	MaxIntraCF	MedIntra	MinInter	MedMinInter
Plagiolepis alluaudi		mono	RN	**2-0**	2-0		0.5		13.5	13.6
Plagiolepis ampeloni				**0**	0					
Plagiolepis delaugerrei	Y	mono	R	**1-0**	1-0				0	3.6
Plagiolepis grassei		mono	RN	**6-0**	4-0	2	2	1.8	1.7	1.8
Plagiolepis invadens				**0**	0					
Plagiolepis karawajewi				**0**	0					
Plagiolepis pallescens		poly	RN	**2-0**	2-0		10.8		1.3	5.3
Plagiolepis atlantis		mono	N	**1-0**	1-0				9.8	13.2
Plagiolepis pyrenaica	Y	para	R	**77-14**	77-13	2.3	3.1	0.3	0	0
Plagiolepis pygmaea		para	RN	**73-0**	73-0	11	11	0.3	1.1	1.6
Plagiolepis schmitzii		para	RN	**15-0**	15-0	5	5	0.5	1.3	1.6
Plagiolepis taurica	Y	para	RN	**56-7**	56-7	3.9	3.9	1.3	0	3
Plagiolepis xene		mono	RN	**23-0**	23-0	1.8	1.8	0.3	1.7	2
Polyergus rufescens		mono	RN	**13-0**	13-0	2	2	1.1		
Prenolepis nitens		mono	N	**14-0**	13-0	2	2	0.2		
Proformica cerdanyensis		poly	RN	**8-0**	4-0	8.8	9.6	8.8	0.2	4.2
Proformica chelmosensis		mono	N	**1-0**	1-0				5.7	8.1
Proformica christophii				**0**	0					
Proformica epinotalis				**0**	0					
Proformica ferreri		para	RN	**8-0**	5-0	4.7	5.7	4.6	3.8	4.2
Proformica kobachidzei				**0**	0					
Proformica korbi		mono	N	**1-0**	1-0				3.5	9.1
Proformica lebasi				**0**	0					
Proformica longipilosa	Y	poly	RN	**25-0**	15-0	12.2	13.5	8.9	0	5.9
Proformica longiseta		poly	RN	**13-0**	6-0	5.5	5.5	3.7	0.3	4.8
Proformica nasuta	Y	para	RN	**30-0**	15-0	10.5	13.5	9	0	4.8
Proformica oculatissima		poly	N	**5-0**	5-0	10.4	10.4	7.9	3.5	4.3
Proformica pilosiscapa				**0**	0					
Proformica striaticeps		mono	N	**2-0**	2-0		1.8		6.4	6.7
Rossomyrmex minuchae		mono	RN	**13-0**	3-0	2.4	4.4	2.4		
Leptanilla charonea	Y	mono	R	**1-0**	1-0				0	15.7
Leptanilla doderoi				**0**	0					
Leptanilla ortunoi				**0**	0					
Leptanilla plutonia				**0**	0					
Leptanilla poggii				**0**	0					
Leptanilla revelierii		mono	R	**1-0**	1-0				16	17.8
Leptanilla zaballosi				**0-1**	0-1					
Aphaenogaster aktaci		mono	RN	**2-0**	2-0		0.6		7.5	7.7

Table S3: European species sequenced. For each species the following information is provided: barcoding sharing detected (BS); monophyly, polyphyly or paraphyly detected and singletons (Group); retrieved (R) and/or de novo (N) sequences (Source); number total sequences (All) and only those above 599bp (Long), with distinction between identified - cf. levels; maximum of the intraspecific p-dist applying a strict criterion (MaxIntra) and those with a less strict criterion (MaxIntraCF); median of the intraspecific p-dist (MedIntra); minimum of the interspecific p-dist (MinInter); median of the minimum values of interspecific p-dist obtained for each specimen (MedMinInter). *(continued)*

			**Sequences**			**p-dist**				
Taxon	BS	Group	Source	All	Long	MaxIntra	MaxIntraCF	MedIntra	MinInter	MedMinInter
Aphaenogaster asterioni		mono	R	**1-0**	1-0				5.8	13
Aphaenogaster balcanica		para	RN	**13-0**	12-0	3.8	3.8	1.5	0.2	0.8
Aphaenogaster balcanicoides				**0**	0					
Aphaenogaster campana		para	RN	**24-0**	13-0	6.3	6.6	1.8	2.6	3.1
Aphaenogaster cardenai		mono	RN	**6-0**	6-0	6.8	6.8	1.4	14.5	14.8
Aphaenogaster cecconii		mono	RN	**4-0**	4-0	1.4	1.4	1.2	9.3	9.4
Aphaenogaster charesi		mono	R	**1-0**	1-0				9.1	12.5
Aphaenogaster dulcineae		mono	RN	**13-0**	13-0	0.6	0.6	0.2	6.9	7.2
Aphaenogaster epirotes		mono	RN	**16-0**	14-0	2.3	2.3	0.8	3.8	4.2
Aphaenogaster festae		mono	RN	**2-0**	2-0		0.6		11.9	12.2
Aphaenogaster fiorii		mono	RN	**2-0**	2-0		0		7	7
Aphaenogaster gemella				**0**	0					
Aphaenogaster gibbosa		para	RN	**30-0**	30-0	10.5	10.5	8.1	7.9	9.7
Aphaenogaster graeca		mono	N	**1-0**	1-0				3.5	11
Aphaenogaster iberica		para	RN	**20-0**	20-0	9.3	9.3	6.7	3.7	5.2
Aphaenogaster ichnusa	Y	para	RN	**63-0**	62-0	1.8	1.8	0.6	0	0.2
Aphaenogaster illyrica		mono	RN	**4-0**	4-0	4.6	4.6	4.1	3.5	3.8
Aphaenogaster inermita		mono	R	**5-0**	1-0		0.6		0.6	15.2
Aphaenogaster italica		mono	N	**1-0**	1-0				11.2	15.4
Aphaenogaster jolantae		mono	N	**1-0**	1-0				3.7	14.7
Aphaenogaster karpathica		mono	N	**1-0**	1-0				1.4	15
Aphaenogaster melitensis				**0**	0					
Aphaenogaster olympica		mono	RN	**2-0**	2-0		0		3.7	3.7
Aphaenogaster ovaticeps		mono	RN	**5-0**	5-0	7.2	7.2	2.1	9.4	9.8
Aphaenogaster pallida		mono	RN	**11-0**	11-0	4.5	4.5	0	6.9	7.6
Aphaenogaster peloponnesiaca		mono	R	**3-0**	3-0	0.5	0.5	0.5	9.1	9.3
Aphaenogaster picena		poly	RN	**21-0**	7-0	1.1	11	0.2	0.3	0.3
Aphaenogaster rugosoferruginea		mono	RN	**4-0**	4-0	3.2	3.2	2.4	12.8	13.2
Aphaenogaster sangiorgii		mono	RN	**4-0**	4-0	4.6	4.6	2.6	7.9	8
Aphaenogaster sardoa		para	RN	**7-0**	7-0	16.5	16.5	2.6	12.5	13
Aphaenogaster semipolita		para	RN	**34-0**	18-0	7.2	7.7	4	5	5.7
Aphaenogaster senilis		mono	RN	**21-0**	17-0	3.6	3.6	2	12.1	12.7
Aphaenogaster subterraneosplendida		mono	RN	**6-0**	6-0	2.5	2.5	2.1	5.8	6.1
Aphaenogaster simonellii		para	RN	**11-0**	11-0	3	3	1.4	1.4	2
Aphaenogaster spinosa		mono	RN	**61-0**	56-0	7.5	7.5	4.4	11.1	11.7
Aphaenogaster splendida		mono	RN	**11-1**	11-1	1.5	1.5	0	13	13.1
Aphaenogaster sporadis		para	RN	**6-0**	6-0	0.6	0.6	0.5	0.5	0.7

Table S3: European species sequenced. For each species the following information is provided: barcoding sharing detected (BS); monophyly, polyphyly or paraphyly detected and singletons (Group); retrieved (R) and/or de novo (N) sequences (Source); number total sequences (All) and only those above 599bp (Long), with distinction between identified - cf. levels; maximum of the intraspecific p-dist applying a strict criterion (MaxIntra) and those with a less strict criterion (MaxIntraCF); median of the intraspecific p-dist (MedIntra); minimum of the interspecific p-dist (MinInter); median of the minimum values of interspecific p-dist obtained for each specimen (MedMinInter). *(continued)*

			**Sequences**			**p-dist**				
Taxon	BS	Group	Source	All	Long	MaxIntra	MaxIntraCF	MedIntra	MinInter	MedMinInter
Aphaenogaster striativentris		mono	R	**3-0**	3-0	3.5	3.5	3.5	9.4	9.8
Aphaenogaster strioloides		mono	RN	**2-0**	2-0		0.2		8.7	8.8
Aphaenogaster subcostata		mono	R	**1-0**	1-0				3.8	11.1
Aphaenogaster subterranea	Y	poly	RN	**75-0**	74-0	10	10	2	0	7.9
Aphaenogaster subterraneoides		mono	N	**4-0**	4-0	1.2	1.2	0.8	7.5	7.6
Aphaenogaster tristis		mono	RN	**9-0**	9-0	3.3	3.3	2.7	5.8	6.4
Aphaenogaster trinacriae		para	RN	**5-0**	5-0	6.6	6.6	0.3	5.8	5.8
Aphaenogaster ulibeli		mono	R	**4-0**	4-0	0	0	0	7.9	7.9
Cardiocondyla batesii		mono	N	**8-0**	8-0	0.5	0.5	0.2	12.1	12.1
Cardiocondyla bulgarica		mono	N	**2-0**	2-0		0		0.9	0.9
Cardiocondyla dalmatica		para	N	**6-0**	6-0	0.2	0.2	0	0.2	0.2
Cardiocondyla elegans		mono	N	**2-0**	2-0		0		0.2	0.2
Cardiocondyla emeryi				**0**	0					
Cardiocondyla mauritanica	Y	para	RN	**16-0**	16-0	0.6	0.6	0.3	0	0.3
Cardiocondyla nigra	Y	mono	N	**1-0**	1-0				0	14
Cardiocondyla obscurior		mono	N	**2-0**	2-0		0		16.2	16.2
Cardiocondyla sahlbergi				**0**	0					
Cardiocondyla stambuloffii		mono	N	**2-0**	2-0		0.2		13.3	13.4
Cardiocondyla ulianini				**0**	0					
Carebara oertzeni		mono	N	**1-0**	1-0					
Crematogaster auberti		mono	RN	**29-0**	28-0	3.2	3.2	0.8	14	15
Crematogaster erectepilosa		mono	N	**1-0**	1-0				11.9	13.7
Crematogaster fuentei				**0**	0					
Crematogaster gordani				**0**	0					
Crematogaster inermis		mono	N	**1-0**	1-0				14.3	16.7
Crematogaster ionia	Y	para	N	**9-1**	9-1	8.1	8.1	6.6	0	0.3
Crematogaster laestrygon		mono	N	**6-0**	6-0	5.5	5.5	5.1	14	14.4
Crematogaster lorteti		mono	N	**6-0**	6-0	0	0	0	15.1	15.2
Crematogaster montenigrina				**0**	0					
Crematogaster ourea				**0**	0					
Crematogaster schmidti	Y	poly	N	**23-0**	23-0	7.3	7.3	2.6	0	0.6
Crematogaster scutellaris		mono	RN	**65-0**	65-0	3.2	3.2	1.5	9.6	10.1
Crematogaster sordidula		mono	RN	**42-0**	42-0	10.4	10.4	6	17.7	18.2
Formicoxenus nitidulus		mono	RN	**5-0**	5-0	1.7	1.7	0.8		
Goniomma baeticum		poly	RN	**5-1**	5-1	14	14	0.5	0.2	6.7
Goniomma blanci		mono	RN	**7-0**	7-0	4	4	3.2	14.3	14.7
Goniomma collingwoodi		mono	RN	**4-0**	4-0	2.1	2.1	1.5	13.8	14.4
Goniomma compressisquama		mono	RN	**2-0**	2-0		0.2		15.1	15.1

Table S3: European species sequenced. For each species the following information is provided: barcoding sharing detected (BS); monophyly, polyphyly or paraphyly detected and singletons (Group); retrieved (R) and/or de novo (N) sequences (Source); number total sequences (All) and only those above 599bp (Long), with distinction between identified - cf. levels; maximum of the intraspecific p-dist applying a strict criterion (MaxIntra) and those with a less strict criterion (MaxIntraCF); median of the intraspecific p-dist (MedIntra); minimum of the interspecific p-dist (MinInter); median of the minimum values of interspecific p-dist obtained for each specimen (MedMinInter). *(continued)*

				Sequences		p-dist				
Taxon	BS	Group	Source	All	Long	MaxIntra	MaxIntraCF	MedIntra	MinInter	MedMinInter
Goniomma decipiens		mono	N	**1-0**	1-0				6.6	14.3
Goniomma hispanicum		poly	RN	**12-0**	11-0	14.7	18.1	0.5	0.2	13.2
Goniomma kugleri				**0**	0					
Harpagoxenus sublaevis		mono	RN	**5-0**	5-0	2.3	2.3	1.4		
Leptothorax acervorum		para	RN	**39-0**	38-0	2.9	2.9	0.5	1.2	2.1
Leptothorax buschingeri				**0**	0					
Leptothorax goesswaldi				**0**	0					
Leptothorax gredleri		mono	RN	**3-0**	3-0	0.3	0.3	0.2	1.2	1.4
Leptothorax kutteri		mono	R	**2-0**	2-0		1.2		3.1	3.2
Leptothorax muscorum		para	RN	**11-0**	11-0	3	3	1.3	2.6	2.9
Leptothorax pacis				**0**	0					
Manica rubida		mono	RN	**37-0**	33-0	7.8	8.3	1.7		
Messor atanassovii		mono	N	**1-0**	1-0				4.9	11.8
Messor barbarus		mono	RN	**23-0**	23-0	6.1	6.1	0.6	8.7	9
Messor bouvieri		poly	RN	**35-0**	34-0	5.4	5.4	2.1	1.5	3.5
Messor capitatus		mono	RN	**42-0**	42-0	5.5	5.5	0.6	7.8	8.5
Messor kardamenae				**0**	0					
Messor carpathous		mono	N	**1-0**	1-0				5.8	12.4
Messor celiae				**0**	0					
Messor concolor				**0**	0					
Messor creticus		mono	N	**1-0**	1-0				5.6	11.6
Messor danaes				**0**	0					
Messor erwini		mono	RN	**8-0**	7-0	0.3	0.3	0.2	8	8
Messor hellenius		para	N	**5-3**	5-3	1.5	1.8	1.2	0.2	0.3
Messor hispanicus		mono	N	**1-0**	1-0				0.2	11.7
Messor ibericus		mono	RN	**56-0**	56-0	0.3	0.3	0	2.8	2.8
Messor lobicornis				**0**	0					
Messor lusitanicus		para	N	**4-0**	4-0	7.5	7.5	3.7	0.2	7.2
Messor marocanus		para	N	**4-0**	4-0	6.9	6.9	3.4	3.5	6.1
Messor mcarthuri		para	RN	**7-0**	7-0	3.5	3.5	0.5	0.2	0.3
Messor minor		para	RN	**18-0**	17-0	3.3	4.1	2	0.2	2
Messor muticus		mono	R	**11-0**	11-0	1.8	1.8	0.8	3.1	3.3
Messor oertzeni		mono	N	**3-0**	3-0	0	0	0	8.3	8.3
Messor ponticus		poly	RN	**9-5**	9-5	1.9	1.9	0.7	0.2	0.3
Messor sanctus		mono	N	**4-0**	4-0	0.8	0.8	0.4	3.7	3.8
Messor sordidus				**0**	0					
Messor structor		mono	RN	**66-1**	66-1	5.5	10.3	3.8	3.1	3.8

Table S3: European species sequenced. For each species the following information is provided: barcoding sharing detected (BS); monophyly, polyphyly or paraphyly detected and singletons (Group); retrieved (R) and/or de novo (N) sequences (Source); number total sequences (All) and only those above 599bp (Long), with distinction between identified - cf. levels; maximum of the intraspecific p-dist applying a strict criterion (MaxIntra) and those with a less strict criterion (MaxIntraCF); median of the intraspecific p-dist (MedIntra); minimum of the interspecific p-dist (MinInter); median of the minimum values of interspecific p-dist obtained for each specimen (MedMinInter). *(continued)*

			**Sequences**			**p-dist**				
Taxon	BS	Group	Source	All	Long	MaxIntra	MaxIntraCF	MedIntra	MinInter	MedMinInter
Messor timidus				**0**	0					
Messor varrialei		mono	N	**1-0**	1-0				0.2	11.4
Messor veneris				**0**	0					
Messor wasmanni		poly	N	**32-2**	32-2	8.1	8.8	0.2	0.2	5
Monomorium algiricum		mono	N	**3-0**	3-0	1.8	1.8	1.8	9.9	9.9
Monomorium andrei		mono	N	**2-0**	2-0		0		12.5	12.5
Monomorium bicolor		mono	R	**1-0**	1-0					
Monomorium carbonarium		mono	RN	**7-0**	6-0	4.4	4.4	4.3	11.3	11.6
Monomorium creticum		mono	N	**3-0**	3-0	6	6	5.8	10.4	12.4
Monomorium exiguum		mono	R	**1-0**	1-0					
Monomorium floricola		mono	R	**4-0**	4-0					
Monomorium gallicum				**0**	0					
Monomorium monomorium		mono	N	**17-0**	17-0	9.5	9.5	2.1	11.3	11.8
Monomorium pharaonis		mono	RN	**8-0**	7-0	10.2	10.8	10.2	13.5	13.8
Monomorium sahlbergi		mono	R	**1-0**	1-0				16.2	16.7
Monomorium salomonis		mono	N	**9-0**	9-0	0.2	0.2	0	2.8	2.8
Monomorium sommieri		mono	N	**1-0**	1-0				2.8	14
Monomorium subopacum		mono	RN	**20-0**	20-0	1.2	1.2	0.9	9.9	10.1
Myrmecina graminicola		mono	RN	**50-0**	50-0	4.6	4.6	0.3	7.8	8.1
Myrmecina melonii		mono	N	**1-0**	1-0				6.3	8.6
Myrmecina sicula		para	N	**5-0**	5-0	10.1	10.1	6.1	6.3	9
Myrmica aloba		poly	RN	**5-0**	5-0	3.8	3.8	2.4	0.3	0.4
Myrmica babiensis				**0**	0					
Myrmica bergi		mono	R	**1-0**	1-0				8.2	10
Myrmica bibikoffi				**0**	0					
Myrmica constricta		poly	RN	**4-0**	3-0	0	1.7	0	0.3	0.3
Myrmica curvithorax		mono	N	**2-0**	2-0		1.1		7.5	8
Myrmica deplanata				**0**	0					
Myrmica gallienii		mono	RN	**4-0**	4-0	0.3	0.3	0.2	7.7	8
Myrmica hellenica	Y	para	N	**5-0**	5-0	1.2	1.2	1.1	0	0.2
Myrmica hirsuta	Y	para	R	**2-0**	2-0		0.2		0	0.1
Myrmica karavajevi		mono	RN	**4-0**	4-0	1.3	1.3	0.8	2	2.6
Myrmica kozakorum				**0**	0					
Myrmica laurae		mono	R	**1-0**	1-0				4	5.7
Myrmica lemasnei		mono	N	**1-0**	1-0				2	8.9
Myrmica lobicornis	Y	para	RN	**13-1**	13-1	3.8	3.8	0.2	0	0
Myrmica lobulicornis	Y	poly	RN	**5-1**	5-1	3.3	3.3	1	0	0.5
Myrmica lonae	Y	poly	RN	**9-1**	9-1	1.8	1.8	0.7	0	0.2

Table S3: European species sequenced. For each species the following information is provided: barcoding sharing detected (BS); monophyly, polyphyly or paraphyly detected and singletons (Group); retrieved (R) and/or de novo (N) sequences (Source); number total sequences (All) and only those above 599bp (Long), with distinction between identified - cf. levels; maximum of the intraspecific p-dist applying a strict criterion (MaxIntra) and those with a less strict criterion (MaxIntraCF); median of the intraspecific p-dist (MedIntra); minimum of the interspecific p-dist (MinInter); median of the minimum values of interspecific p-dist obtained for each specimen (MedMinInter). *(continued)*

			**Sequences**			**p-dist**				
Taxon	BS	Group	Source	All	Long	MaxIntra	MaxIntraCF	MedIntra	MinInter	MedMinInter
Myrmica microrubra	Y	poly	RN	**5-0**	5-0	1.2	1.2	0.4	0	0
Myrmica myrmicoxena				**0**	0					
Myrmica obscura				**0**	0					
Myrmica pelops				**0**	0					
Myrmica ravasinii		mono	N	**1-0**	1-0				5.1	10.6
Myrmica rubra	Y	para	RN	**45-0**	44-0	1.6	1.6	0.6	0	0.5
Myrmica ruginodis		mono	RN	**32-0**	32-0	2.1	2.1	0.2	7.5	8
Myrmica rugulosa		mono	RN	**15-0**	15-0	2.2	2.2	1.8	4	4.8
Myrmica sabuleti	Y	poly	RN	**27-4**	26-4	3.5	3.5	0.6	0	0.2
Myrmica scabrinodis	Y	poly	RN	**70-0**	64-0	5.6	6.6	3	0	0.5
Myrmica schencki		para	RN	**15-0**	15-0	1.4	1.4	0.3	2	2.1
Myrmica siciliana		mono	RN	**2-0**	2-0				2	11.2
Myrmica specioides		poly	N	**16-0**	16-0	2.5	2.5	1.7	0.2	0.4
Myrmica spinosior	Y	poly	RN	**92-1**	81-1	4.1	4.1	2.2	0	0
Myrmica stangeana				**0**	0					
Myrmica sulcinodis		para	RN	**15-0**	15-0	2.3	2.3	1.2	0.8	1.4
Myrmica tulinae		mono	R	**1-0**	1-0					
Myrmica vandeli		mono	RN	**6-0**	6-0	1.2	1.2	0.6	2.5	2.5
Myrmica wesmaeli		mono	RN	**4-0**	4-0	0	0	0	0.5	0.6
Myrmica xavieri		para	RN	**3-0**	3-0	1.6	1.6	1.6	0.8	1.1
Oxyopomyrmex arenarium				**0**	0					
Oxyopomyrmex krueperi		mono	N	**1-0**	1-0				2.7	15.7
Oxyopomyrmex laevibus		mono	N	**1-0**	1-0				2.7	15.2
Oxyopomyrmex magnus		mono	R	**1-0**	1-0				8.6	16.9
Oxyopomyrmex pallens				**0**	0					
Oxyopomyrmex polybotesi		mono	N	**1-0**	1-0				3.2	16.1
Oxyopomyrmex saulcyi		para	RN	**9-0**	8-0	13.1	13.1	11.6	9.3	10.3
Pheidole anastasii		mono	R	**2-0**	1-0					
Pheidole balcanica		mono	N	**9-6**	9-6	7.4	7.4	3.4	9.9	10
Pheidole bilimeki		mono	R	**5-0**	5-0					
Pheidole indica		mono	RN	**13-0**	12-0	0.5	1.5	0.2	15.9	15.9
Pheidole keftiuensis				**0**	0					
Pheidole koshewnikovi				**0-2**	0-2		0.2			
Pheidole megacephala		mono	RN	**4-0**	4-0				15.4	16.9
Pheidole navigans		mono	R	**1-0**	1-0					
Pheidole pallidula		para	RN	**0**	0	13.5	13.5	8	9.9	11
Pheidole punctatissima		mono	N	**1-0**	1-0				17.3	18.1

Table S3: European species sequenced. For each species the following information is provided: barcoding sharing detected (BS); monophyly, polyphyly or paraphyly detected and singletons (Group); retrieved (R) and/or de novo (N) sequences (Source); number total sequences (All) and only those above 599bp (Long), with distinction between identified - cf. levels; maximum of the intraspecific p-dist applying a strict criterion (MaxIntra) and those with a less strict criterion (MaxIntraCF); median of the intraspecific p-dist (MedIntra); minimum of the interspecific p-dist (MinInter); median of the minimum values of interspecific p-dist obtained for each specimen (MedMinInter). *(continued)*

			Sequences			p-dist				
Taxon	BS	Group	Source	All	Long	MaxIntra	MaxIntraCF	MedIntra	MinInter	MedMinInter
Solenopsis abdita		para	RN	**12-1**	10-1		0.9			
Solenopsis crivellarii		mono	N	**1-0**	1-0				2.2	8.2
Solenopsis debilior				**0**	0					
Solenopsis fairchildi				**0**	0					
Solenopsis fugax		para	RN	**28-15**	27-15	6.3	7.8	4.5	3.2	3.5
Solenopsis juliae		mono	N	**7-0**	7-0	1.4	1.4	0.3	2.1	2.4
Solenopsis gallica				**0**	0					
Solenopsis ilinei				**0**	0					
Solenopsis invicta		mono	RN	**7-0**	7-0				15.3	18.7
Solenopsis latro		mono	N	**2-0**	2-0		0.2		10.5	10.6
Solenopsis lusitanica		para	RN	**11-19**	11-17	3.2	8.6	1.5	2.1	2.4
Solenopsis oraniensis				**0**	0					
Solenopsis orbula		mono	RN	**6-0**	6-0	0.2	0.2	0	10.5	10.6
Solenopsis santschii				**0**	0					
Solenopsis texana		para	R	**2-0**	2-0		0		0.9	0.9
Solenopsis wolfi		mono	N	**1-0**	1-0				2.2	8.6
Stenamma debile	Y	poly	RN	**21-0**	19-0	17.9	17.9	1.5	0	0.5
Stenamma petiolatum				**0**	0					
Stenamma punctiventre		mono	R	**1-0**	1-0				21.7	22.4
Stenamma sardoum				**0**	0					
Stenamma siculum				**0**	0					
Stenamma striatulum		mono	RN	**17-0**	17-0	1.5	1.5	0	17.7	17.9
Stenamma westwoodii		poly	N	**3-0**	3-0	17.3	17.3	17	1.1	5.2
Stenamma zanoni		mono	N	**1-0**	1-0				12.6	14.6
Strongylognathus afer				**0**	0					
Strongylognathus alpinus		mono	N	**1-0**	1-0				1.5	11.2
Strongylognathus arnoldii				**0**	0					
Strongylognathus caeciliae		mono	N	**3-0**	3-0	0.3	0.3	0.2	0.8	0.8
Strongylognathus chelifer				**0**	0					
Strongylognathus christophi				**0**	0					
Strongylognathus destefanii	Y	poly	RN	**6-1**	6-1	2.3	2.3	1.5	0	0.3
Strongylognathus huberi		poly	N	**4-1**	4-1	5.5	5.7	5.4	0.3	1.8
Strongylognathus insularis	Y	mono	N	**1-0**	1-0				0	10.4
Strongylognathus italicus				**0**	0					
Strongylognathus karawajewi		mono	N	**1-0**	1-0				6.5	9.4
Strongylognathus kratochvili				**0**	0					
Strongylognathus pisarskii		poly	N	**3-0**	1-0		3.5		0.7	10.9
Strongylognathus silvestrii		mono	N	**1-0**	1-0				2.3	10.9

Table S3: European species sequenced. For each species the following information is provided: barcoding sharing detected (BS); monophyly, polyphyly or paraphyly detected and singletons (Group); retrieved (R) and/or de novo (N) sequences (Source); number total sequences (All) and only those above 599bp (Long), with distinction between identified - cf. levels; maximum of the intraspecific p-dist applying a strict criterion (MaxIntra) and those with a less strict criterion (MaxIntraCF); median of the intraspecific p-dist (MedIntra); minimum of the interspecific p-dist (MinInter); median of the minimum values of interspecific p-dist obtained for each specimen (MedMinInter). *(continued)*

			**Sequences**			**p-dist**				
Taxon	BS	Group	Source	All	Long	MaxIntra	MaxIntraCF	MedIntra	MinInter	MedMinInter
Strongylognathus testaceus		mono	RN	**12-0**	12-0	2.5	2.5	0.5	5.5	5.8
Strumigenys argiola		mono	N	**2-1**	2-1		9.2		14.3	14.4
Strumigenys baudueri		poly	N	**4-1**	4-1	11.7	12.2	8.4	0.2	1.7
Strumigenys membranifera		mono	N	**3-0**	3-0	3.1	3.1	2.6	14.7	14.9
Strumigenys perplexa		mono	RN	**6-0**	6-0		0		16.2	16.2
Strumigenys rogeri		mono	R	**3-0**	3-0					
Strumigenys silvestrii				**0**	0					
Strumigenys tenuipilis		poly	N	**3-0**	3-0	13	13	13	0.2	3.2
Strumigenys tenuissima				**0**	0					
Temnothorax adlerzi				**0**	0					
Temnothorax aeolius				**0**	0					
Temnothorax affinis	Y	mono	RN	**22-0**	19-0	3.9	3.9	1.9	0	1.6
Temnothorax albipennis	Y	poly	N	**4-3**	4-3	12	12	6.8	0	0.8
Temnothorax alfacarensis				**0**	0					
Temnothorax algerianus				**0**	0					
Temnothorax algiricus		poly	N	**8-0**	8-0	3.4	3.4	2.2	2.6	2.7
Temnothorax alienus		mono	RN	**8-0**	8-0	0.8	0.8	0	9.3	9.6
Temnothorax angulinodis		mono	N	**1-0**	1-0				3.6	14.4
Temnothorax angustifrons		mono	N	**1-0**	1-0				6	14
Temnothorax angustulus		mono	RN	**8-0**	6-0	2.9	2.9	0.7	12.1	12.3
Temnothorax ansei				**0**	0					
Temnothorax antigoni		poly	N	**2-0**	2-0		1.8		0.2	0.4
Temnothorax apenninicus				**0**	0					
Temnothorax arcanus				**0**	0					
Temnothorax ariadnae		mono	RN	**4-0**	4-0	1.3	1.3	1.1	4.2	4.9
Temnothorax arkasi				**0**	0					
Temnothorax atlantis	Y	para	N	**4-0**	4-0	0.8	0.8	0.6	0	0.4
Temnothorax aveli	Y	para	N	**4-0**	4-0	0.5	0.5	0.2	0	0.2
Temnothorax baeticus		mono	RN	**3-1**	3-1	0.6	0.6	0.6	12.5	12.5
Temnothorax bejaraniensis				**0**	0					
Temnothorax bernardi		mono	R	**2-0**	2-0		0.5		2.9	3
Temnothorax blascoi		mono	R	**1-0**	1-0				12.2	15.6
Temnothorax brackoi		mono	N	**1-0**	1-0				2.8	14.3
Temnothorax bulgaricus		poly	N	**4-0**	4-0	5.5	5.5	5.1	3.7	3.8
Temnothorax caesari				**0**	0					
Temnothorax cagnianti		mono	R	**1-0**	1-0				11.3	14.5
Temnothorax clypeatus		mono	RN	**5-0**	5-0	2.6	2.6	2.6	13.8	13.9

Table S3: European species sequenced. For each species the following information is provided: barcoding sharing detected (BS); monophyly, polyphyly or paraphyly detected and singletons (Group); retrieved (R) and/or de novo (N) sequences (Source); number total sequences (All) and only those above 599bp (Long), with distinction between identified - cf. levels; maximum of the intraspecific p-dist applying a strict criterion (MaxIntra) and those with a less strict criterion (MaxIntraCF); median of the intraspecific p-dist (MedIntra); minimum of the interspecific p-dist (MinInter); median of the minimum values of interspecific p-dist obtained for each specimen (MedMinInter). *(continued)*

			Sequences			p-dist				
Taxon	BS	Group	Source	All	Long	MaxIntra	MaxIntraCF	MedIntra	MinInter	MedMinInter
Temnothorax conatensis				**0**	0					
Temnothorax continentalis	Y	poly	R	**2-0**	2-0		0.3		0	0.1
Temnothorax convexus		mono	N	**1-0**	1-0				6.9	16.4
Temnothorax corsicus				**0**	0					
Temnothorax corticalis		mono	RN	**4-0**	4-0	1.7	1.7	1.4	10.7	10.7
Temnothorax crasecundus	Y	poly	RN	**7-1**	7-1	2.7	5.5	1.1	0	0.2
Temnothorax crassispinus	Y	poly	RN	**9-0**	9-0	3.6	3.6	2.3	0	0.2
Temnothorax crassistriatus				**0**	0					
Temnothorax crepuscularis		mono	N	**3-0**	3-0	0.3	0.3	0.3	12	12.1
Temnothorax cristinae		mono	R	**1-0**	1-0				6.3	16.9
Temnothorax curtisetosus		mono	N	**1-0**	1-0				1.1	18
Temnothorax curtulus				**0**	0					
Temnothorax daidalosi		mono	N	**1-0**	1-0				6.9	15.9
Temnothorax dessyi		mono	N	**2-0**	2-0		1.8		7.2	7.8
Temnothorax eocho				**0**	0					
Temnothorax estel		mono	N	**4-0**	4-0	0.8	0.8	0.5	7.8	7.9
Temnothorax euboeae				**0**	0					
Temnothorax exilis		poly	N	**9-8**	9-8	12	12.9	10.6	4	6.7
Temnothorax finzii				**0**	0					
Temnothorax flavicornis		mono	RN	**9-0**	9-0	9.6	9.6	1.1	11.4	11.8
Temnothorax flavispinus				**0**	0					
Temnothorax fuentei				**0**	0					
Temnothorax gordiagini		mono	N	**1-0**	1-0				9.3	15.8
Temnothorax gallei		mono	N	**7-0**	7-0	6.7	6.7	0.6	9.2	9.3
Temnothorax graecus		mono	N	**1-0**	1-0				4.2	15
Temnothorax gredosi		mono	RN	**6-0**	6-0	1.1	1.1	0.6	8.8	8.8
Temnothorax grouvellei		poly	N	**2-0**	2-0		15.5		4	9
Temnothorax helenae		mono	RN	**12-0**	12-0	2.1	2.1	1.4	4.2	4.6
Temnothorax ibericus		mono	RN	**2-0**	2-0		0		7.4	7.5
Temnothorax ikarosi				**0**	0					
Temnothorax incompletus				**0**	0					
Temnothorax inquilinus				**0**	0					
Temnothorax interruptus		mono	RN	**5-0**	5-0	1.2	1.2	0	14.2	14.3
Temnothorax italicus	Y	para	N	**4-0**	4-0	0.6	0.6	0.3	0	0
Temnothorax jailensis				**0**	0					
Temnothorax kemali		mono	N	**1-0**	1-0				1.9	14.6
Temnothorax kraussei		mono	N	**1-0**	1-0				1.4	15.8
Temnothorax kutteri		mono	RN	**5-0**	5-0	1.4	1.4	0.2	6.3	6.3

Table S3: European species sequenced. For each species the following information is provided: barcoding sharing detected (BS); monophyly, polyphyly or paraphyly detected and singletons (Group); retrieved (R) and/or de novo (N) sequences (Source); number total sequences (All) and only those above 599bp (Long), with distinction between identified - cf. levels; maximum of the intraspecific p-dist applying a strict criterion (MaxIntra) and those with a less strict criterion (MaxIntraCF); median of the intraspecific p-dist (MedIntra); minimum of the interspecific p-dist (MinInter); median of the minimum values of interspecific p-dist obtained for each specimen (MedMinInter). *(continued)*

			Sequences			p-dist				
Taxon	BS	Group	Source	All	Long	MaxIntra	MaxIntraCF	MedIntra	MinInter	MedMinInter
Temnothorax laconicus		poly	RN	**10-0**	10-0	1.4	1.4	0.2	0.3	0.5
Temnothorax laestrygon		mono	RN	**5-0**	5-0	4.5	4.5	3.2	7	7.5
Temnothorax lagrecai		para	RN	**4-0**	4-0	2	2	1.3	0.5	0.8
Temnothorax leviceps		mono	N	**2-0**	2-0		2		6.7	7
Temnothorax lichtensteini		para	RN	**23-0**	23-0	2.1	2.1	0.6	0.3	0.5
Temnothorax longispinosus		mono	R	**1-0**	1-0					
Temnothorax lucidus		para	R	**6-0**	6-0	4.4	4.4	3.9	3.6	3.8
Temnothorax luteus		mono	N	**6-1**	6-1	0.9	0.9	0.6	9.8	9.8
Temnothorax marae		para	RN	**3-0**	3-0	2.1	2.1	2	0.5	1.1
Temnothorax melas		mono	N	**2-0**	2-0		0		9.3	9.3
Temnothorax minotaurosi				**0**	0					
Temnothorax minozzii				**0**	0					
Temnothorax morea				**0**	0					
Temnothorax muellerianus		para	RN	**6-0**	6-0	3.7	3.7	2.1	1.1	2.9
Temnothorax mytilenes		mono	N	**1-0**	1-0				10.6	15.6
Temnothorax nadigi		mono	N	**3-0**	3-0	1.4	1.4	1.2	4	4.4
Temnothorax naeviventris				**0**	0					
Temnothorax nassonovi		mono	R	**1-0**	1-0					
Temnothorax nebulosus				**0**	0					
Temnothorax niger		mono	N	**6-1**	6-1	1.5	1.5	1.4	9.4	9.5
Temnothorax nigriceps		mono	RN	**7-2**	7-2	3.7	9.4	2.8	7.2	8
Temnothorax nylanderi	Y	poly	RN	**26-0**	26-0	2.5	2.5	0.2	0	0.2
Temnothorax pardoi		poly	RN	**9-0**	9-0	17.5	17.5	10.2	8	13.3
Temnothorax parnonensis				**0**	0					
Temnothorax parvulus		mono	RN	**14-0**	14-0	0.9	0.9	0.2	4.5	4.7
Temnothorax pelagosanus				**0**	0					
Temnothorax phaetoni				**0**	0					
Temnothorax platycephalus		mono	N	**1-0**	1-0				8.3	15.3
Temnothorax poldii		mono	RN	**3-0**	3-0	0.5	0.5	0.5	7.4	7.6
Temnothorax proteii				**0**	0					
Temnothorax racovitzai		poly	N	**10-1**	9-1	5.8	24.2	0.2	7.8	7.8
Temnothorax ravouxi		mono	N	**1-0**	1-0				1.4	16.2
Temnothorax recedens		para	N	**31-1**	31-1	3.2	3.2	1.8	0.2	0.9
Temnothorax rogeri		poly	N	**2-0**	2-0		1.2		0.2	0.5
Temnothorax rottenbergii				**0**	0					
Temnothorax rougeti				**0**	0					
Temnothorax sappho				**0**	0					

Table S3: European species sequenced. For each species the following information is provided: barcoding sharing detected (BS); monophyly, polyphyly or paraphyly detected and singletons (Group); retrieved (R) and/or de novo (N) sequences (Source); number total sequences (All) and only those above 599bp (Long), with distinction between identified - cf. levels; maximum of the intraspecific p-dist applying a strict criterion (MaxIntra) and those with a less strict criterion (MaxIntraCF); median of the intraspecific p-dist (MedIntra); minimum of the interspecific p-dist (MinInter); median of the minimum values of interspecific p-dist obtained for each specimen (MedMinInter). *(continued)*

			Sequences			p-dist				
Taxon	BS	Group	Source	All	Long	MaxIntra	MaxIntraCF	MedIntra	MinInter	MedMinInter
Temnothorax sardous				0	0					
Temnothorax saxatilis				0	0					
Temnothorax saxonicus		poly	N	**2-0**	2-0		8.4		0.5	0.5
Temnothorax schaufussi				0	0					
Temnothorax semenovi				0	0					
Temnothorax balcanicus		para	N	**8-0**	8-0	2	2	1.4	9.2	9.2
Temnothorax siculus		mono	N	**1-0**	1-0				7.8	15.4
Temnothorax smyrnensis				0	0					
Temnothorax solerii		mono	N	**1-0**	1-0				0.2	15
Temnothorax sordidulus		mono	N	**1-0**	1-0				0.5	14.8
Temnothorax splendidiceps				0	0					
Temnothorax strymonensis				0	0					
Temnothorax stumperi				0	0					
Temnothorax subtilis		para	R	**8-0**	8-0	11.3	11.3	1.8	6.8	7
Temnothorax tamarae				0	0					
Temnothorax tergestinus		poly	RN	**5-0**	5-0	9	9	2.4	0.5	0.5
Temnothorax triangularis		mono	N	**1-0**	1-0				0.2	13.5
Temnothorax tuberum		poly	RN	**14-3**	13-3	3.8	10	0.3	0.8	0.9
Temnothorax turcicus		para	N	**3-0**	3-0	2.5	2.5	2.4	0.3	2.4
Temnothorax tyndalei		mono	N	**2-0**	2-0		0		11.5	11.5
Temnothorax unifasciatus	Y	poly	RN	**23-9**	22-9	12.3	12.3	10.2	0	3.5
Temnothorax universitatis				0	0					
Temnothorax variabilis				0	0					
Temnothorax vivianoi		mono	RN	**3-0**	2-0		6.8		9.1	9.1
Temnothorax volgensis				0	0					
Temnothorax zaleskyi				0	0					
Tetramorium albenae				0	0					
Tetramorium alpestre		para	RN	**89-5**	7-5	2.2	4.1	1.5	1.6	2
Tetramorium alternans		mono	N	**1-0**	0					
Tetramorium atratulum		mono	RN	**9-0**	9-0	6.9	6.9	5.4	12.5	13.6
Tetramorium bicarinatum		mono	RN	**8-0**	7-0	1.1	1.3	1.1	8.4	9.2
Tetramorium biskrense		mono	N	**3-0**	3-0	0.3	0.3	0.3	6.3	6.6
Tetramorium brevicorne				0	0					
Tetramorium breviscapus				0	0					
Tetramorium buschingeri				0	0					
Tetramorium caespitum	Y	poly	RN	**225-10**	22-10	6.2	10.6	3.5	0	1.9
Tetramorium caldarium		mono	N	**1-0**	1-0				13.3	16.9
Tetramorium chefketi		mono	RN	**8-0**	5-0	3.5	3.6	3.3	2.9	3.8

Table S3: European species sequenced. For each species the following information is provided: barcoding sharing detected (BS); monophyly, polyphyly or paraphyly detected and singletons (Group); retrieved (R) and/or de novo (N) sequences (Source); number total sequences (All) and only those above 599bp (Long), with distinction between identified - cf. levels; maximum of the intraspecific p-dist applying a strict criterion (MaxIntra) and those with a less strict criterion (MaxIntraCF); median of the intraspecific p-dist (MedIntra); minimum of the interspecific p-dist (MinInter); median of the minimum values of interspecific p-dist obtained for each specimen (MedMinInter). *(continued)*

			**Sequences**			**p-dist**				
Taxon	BS	Group	Source	All	Long	MaxIntra	MaxIntraCF	MedIntra	MinInter	MedMinInter
Tetramorium davidi				**0**	0					
Tetramorium diomedeum	Y	para	RN	**7-0**	7-0	0.9	0.9	0.6	0	0.6
Tetramorium exasperatum		mono	N	**1-0**	1-0				10.1	13.2
Tetramorium ferox		mono	N	**1-0**	1-0				4.7	8.1
Tetramorium forte		poly	RN	**11-0**	10-0	10.6	11.3	0.9	0.3	2.8
Tetramorium fusciclava				**0**	0					
Tetramorium galaticum				**0**	0					
Tetramorium hippocratis				**0**	0					
Tetramorium hungaricum		mono	RN	**26-1**	3-1	2.5	2.8	2.5	3.5	3.5
Tetramorium immigrans	Y	para	RN	**64-9**	17-9	1.4	2.4	0.7	0	0.6
Tetramorium impurum	Y	poly	RN	**74-1**	5-1	9.5	9.5	9	0	0.8
Tetramorium indocile		poly	RN	**8-0**	1-0		4		0.6	6.4
Tetramorium inquilinum		mono	N	**1-0**	0					
Tetramorium insolens		mono	N	**3-0**	3-0	0.2	0.2	0.2	8.4	8.6
Tetramorium kephalosi		poly	N	**9-1**	9-1	2.9	2.9	1.9	1.2	1.2
Tetramorium kutteri		mono	R	**1-0**	1-0				8.2	10.3
Tetramorium lanuginosum		mono	RN	**7-0**	7-0	4.7	4.7	0.2	12.7	13.3
Tetramorium lucayanum				**0**	0					
Tetramorium meridionale		mono	RN	**9-0**	9-0	1.4	1.4	1.2	7	7
Tetramorium moravicum		poly	RN	**9-1**	7-1	8.9	9.7	0.9	2.4	4.1
Tetramorium pacificum		mono	N	**1-0**	1-0				11.5	17.9
Tetramorium pelagium				**0**	0					
Tetramorium punctatum	Y	poly	RN	**4-7**	4-7	10.9	12.4	6	0	5
Tetramorium punicum				**0-1**	0-1					
Tetramorium rhodium		mono	N	**1-0**	1-0				7.8	11.9
Tetramorium sahlbergi				**0**	0					
Tetramorium sanetrai				**0**	0					
Tetramorium schmidti				**0**	0					
Tetramorium semilaeve		poly	RN	**55-2**	54-2	3.8	3.8	1.8	0.3	1.7
Tetramorium splendens				**0**	0					
Tetramorium staerckei		mono	RN	**25-1**	4-1	1.1	2.2	0.9	1.9	2.6
Tetramorium sulcinode				**0**	0					
Trichomyrmex destructor		para	R	**4-0**	4-0					
Trichomyrmex mayri		mono	R	**1-0**	1-0					
Trichomyrmex perplexus		mono	N	**5-0**	5-0	3.8	3.8	0.2		
Wasmannia auropunctata		mono	RN	**12-0**	12-0	1.8	1.8	0		
Anochetus ghilianii		mono	RN	**41-0**	41-0	4.6	4.6	1.5	8.8	11.8

Table S3: European species sequenced. For each species the following information is provided: barcoding sharing detected (BS); monophyly, polyphyly or paraphyly detected and singletons (Group); retrieved (R) and/or de novo (N) sequences (Source); number total sequences (All) and only those above 599bp (Long), with distinction between identified - cf. levels; maximum of the intraspecific p-dist applying a strict criterion (MaxIntra) and those with a less strict criterion (MaxIntraCF); median of the intraspecific p-dist (MedIntra); minimum of the interspecific p-dist (MinInter); median of the minimum values of interspecific p-dist obtained for each specimen (MedMinInter). *(continued)*

			**Sequences**			**p-dist**				
Taxon	BS	Group	Source	All	Long	MaxIntra	MaxIntraCF	MedIntra	MinInter	MedMinInter
Anochetus mayri		mono	RN	**2-0**	2-0				8.8	11.8
Brachyponera chinensis		mono	R	**5-0**	1-0					
Cryptopone ochracea		mono	N	**12-0**	12-0	4	4	0.3		
Hypoponera abeillei		mono	N	**4-0**	4-0	0.6	0.6	0.3	14.1	14.2
Hypoponera eduardi		mono	N	**20-0**	20-0	3	3	0	15.6	16.1
Hypoponera ergatandria		para	RN	**6-0**	6-0	4.1	4.1	3.2	1.1	2.9
Hypoponera punctatissima		mono	N	**5-0**	5-0	0.3	0.3	0.2	1.1	1.1
Hypoponera ragusai		mono	N	**1-0**	1-0				11	18.5
Ponera coarctata		mono	RN	**28-0**	28-0	1	1	0	5.8	6.1
Ponera testacea		mono	N	**15-0**	15-0	2.8	2.8	0.3	5.8	6.2
Proceratium algiricum		mono	N	**2-0**	2-0		11		18.4	18.6
Proceratium melinum		mono	N	**2-0**	2-0		0.3		18.4	18.4
Proceratium melitense				**0**	0					
Proceratium numidicum				**0**	0					

The trees

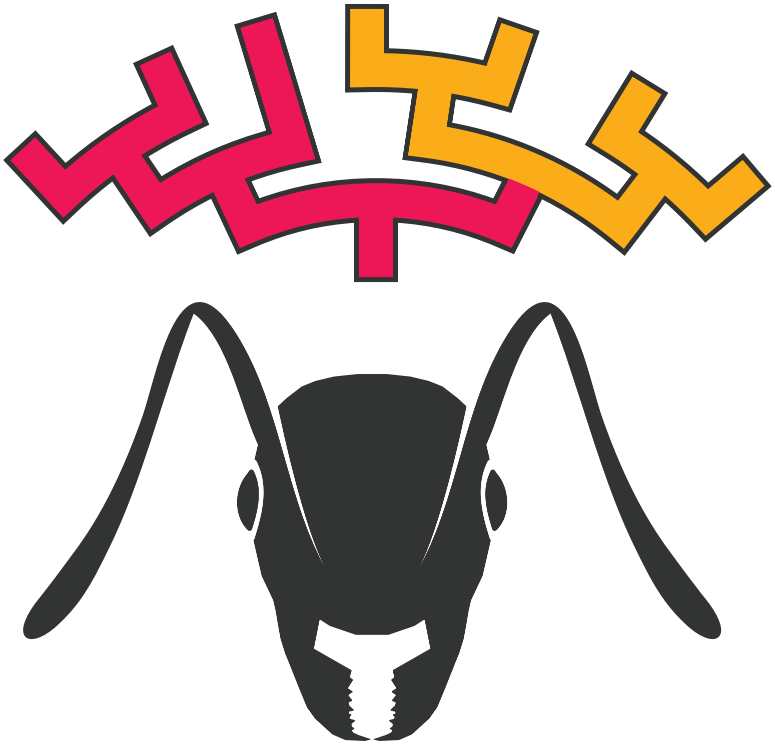

Acropyga

Sequences total: 2; New: 1; Retrieved (*): 1

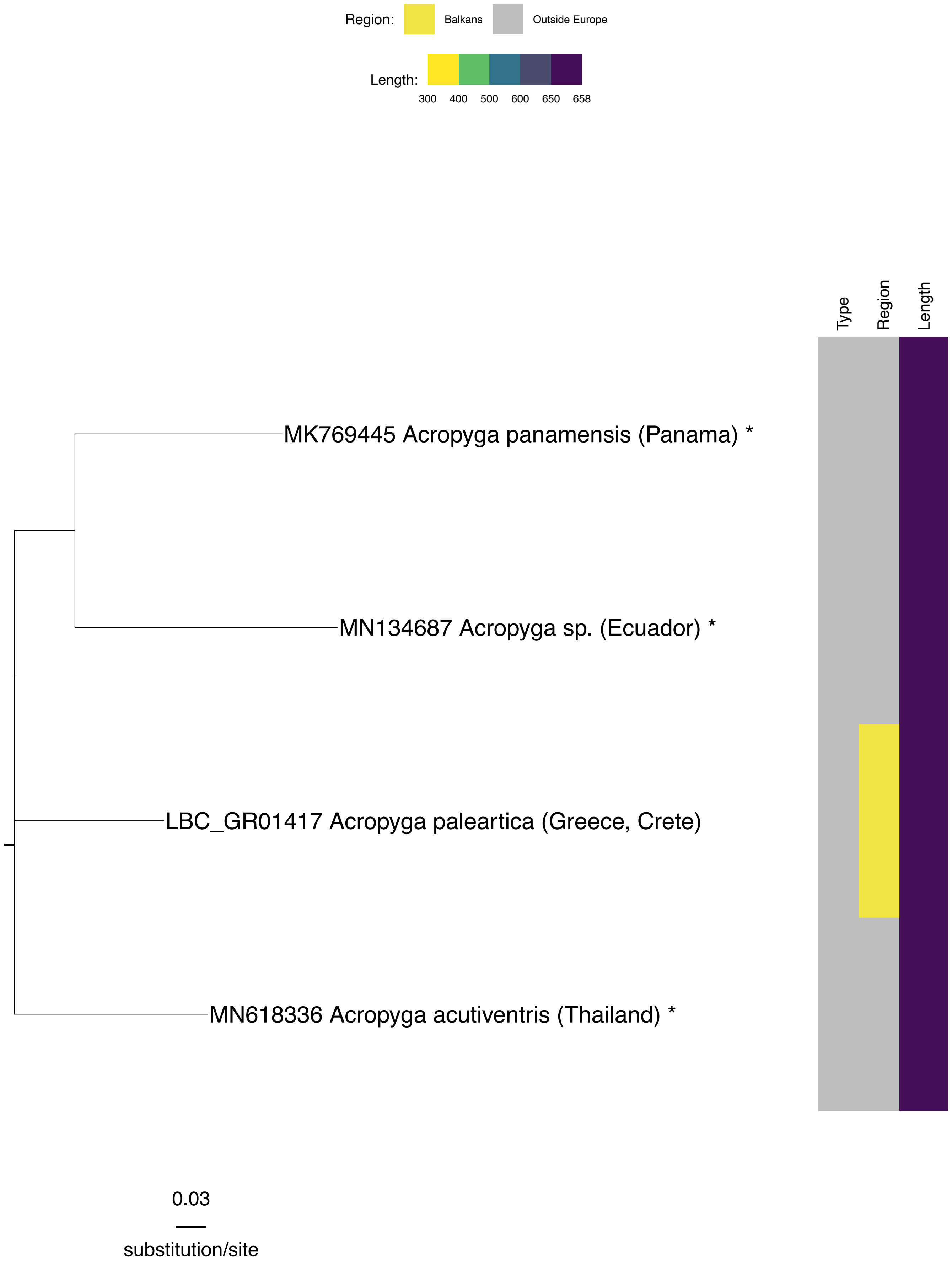

Aenictus

Sequences total: 3; New: 1; Retrieved (*): 2

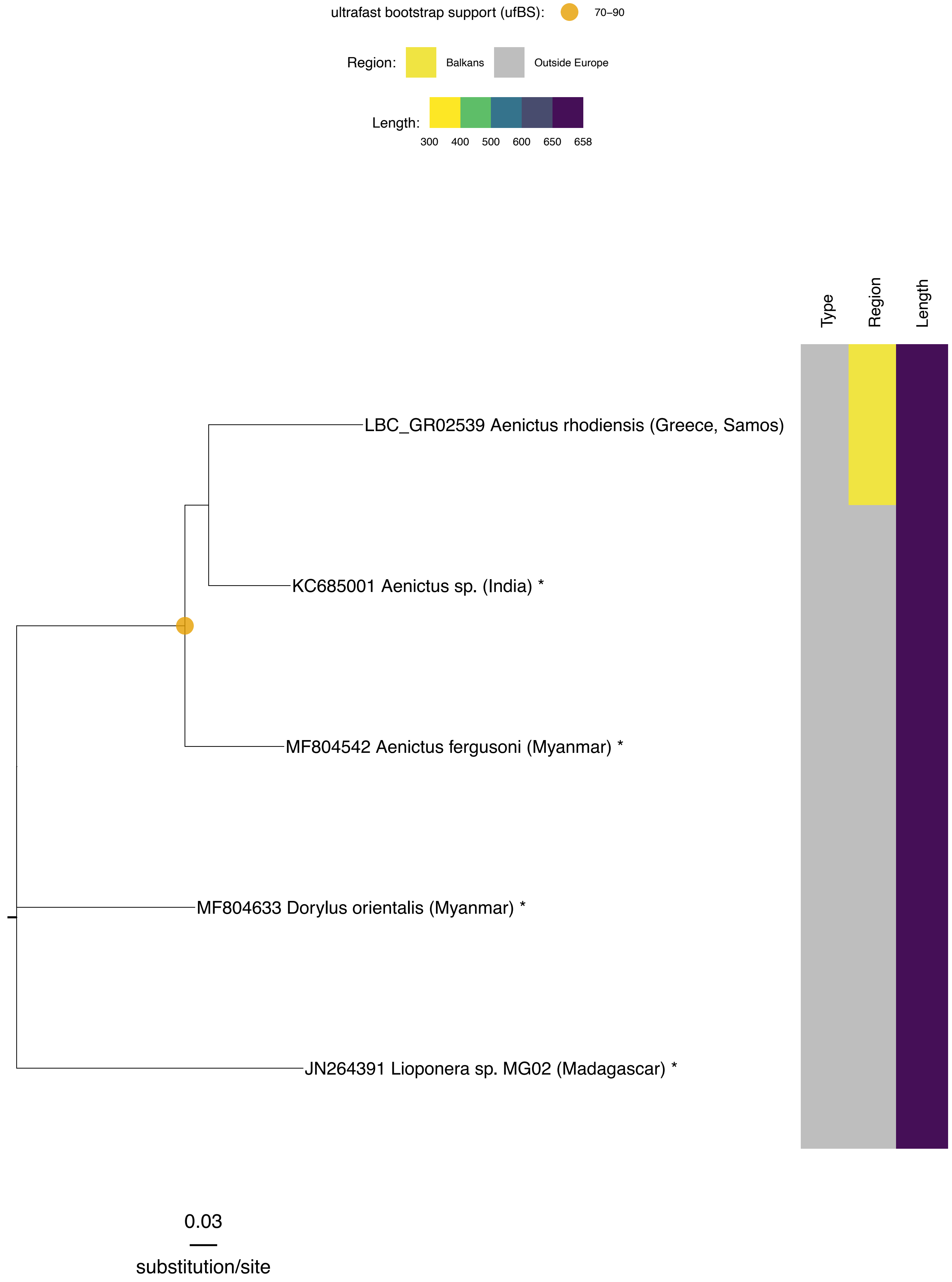

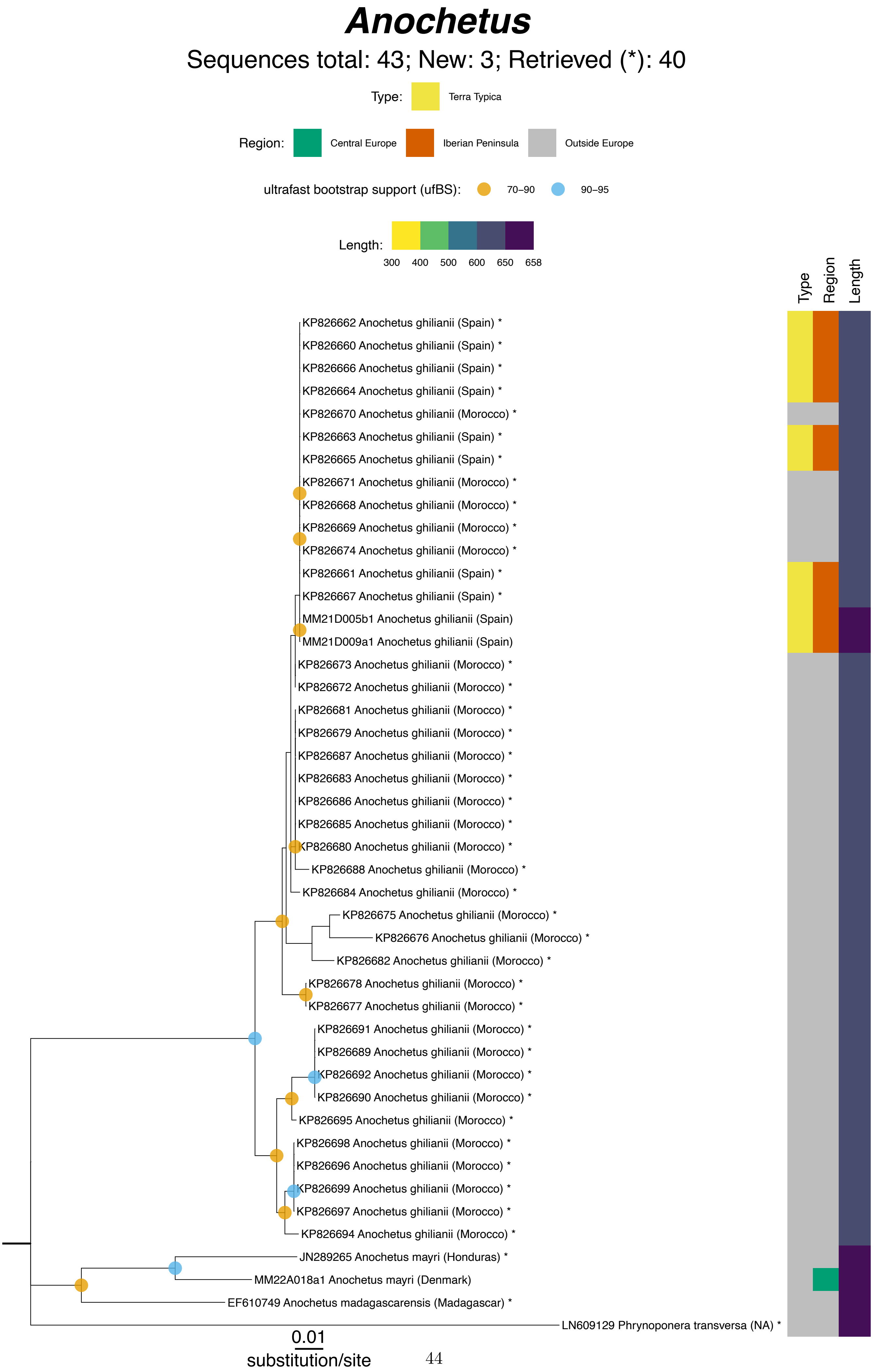
Anochetus
Sequences total: 43; New: 3; Retrieved (*): 40
Type: Terra Typica
Region: Central Europe Iberian Peninsula Outside Europe
ultrafast bootstrap support (ufBS): 70–90 90–95
Length: 300 400 500 600 650 658
Type
Region
Length
KP826662 Anochetus ghilianii (Spain) *
KP826660 Anochetus ghilianii (Spain) *
KP826666 Anochetus ghilianii (Spain) *
KP826664 Anochetus ghilianii (Spain) *
KP826670 Anochetus ghilianii (Morocco) *
KP826663 Anochetus ghilianii (Spain) *
KP826665 Anochetus ghilianii (Spain) *
KP826671 Anochetus ghilianii (Morocco) *
KP826668 Anochetus ghilianii (Morocco) *
KP826669 Anochetus ghilianii (Morocco) *
KP826674 Anochetus ghilianii (Morocco) *
KP826661 Anochetus ghilianii (Spain) *
KP826667 Anochetus ghilianii (Spain) *
MM21D005b1 Anochetus ghilianii (Spain)
MM21D009a1 Anochetus ghilianii (Spain)
KP826673 Anochetus ghilianii (Morocco) *
KP826672 Anochetus ghilianii (Morocco) *
KP826681 Anochetus ghilianii (Morocco) *
KP826679 Anochetus ghilianii (Morocco) *
KP826687 Anochetus ghilianii (Morocco) *
KP826683 Anochetus ghilianii (Morocco) *
KP826686 Anochetus ghilianii (Morocco) *
KP826685 Anochetus ghilianii (Morocco) *
KP826680 Anochetus ghilianii (Morocco) *
KP826688 Anochetus ghilianii (Morocco) *
KP826684 Anochetus ghilianii (Morocco) *
KP826675 Anochetus ghilianii (Morocco) *
KP826676 Anochetus ghilianii (Morocco) *
KP826682 Anochetus ghilianii (Morocco) *
KP826678 Anochetus ghilianii (Morocco) *
KP826677 Anochetus ghilianii (Morocco) *
KP826691 Anochetus ghilianii (Morocco) *
KP826689 Anochetus ghilianii (Morocco) *
KP826692 Anochetus ghilianii (Morocco) *
KP826690 Anochetus ghilianii (Morocco) *
KP826695 Anochetus ghilianii (Morocco) *
KP826698 Anochetus ghilianii (Morocco) *
KP826696 Anochetus ghilianii (Morocco) *
KP826699 Anochetus ghilianii (Morocco) *
KP826697 Anochetus ghilianii (Morocco) *
KP826694 Anochetus ghilianii (Morocco) *
JN289265 Anochetus mayri (Honduras) *
MM22A018a1 Anochetus mayri (Denmark)
EF610749 Anochetus madagascarensis (Madagascar) *
LN609129 Phrynoponera transversa (NA) *
0.01
substitution/site

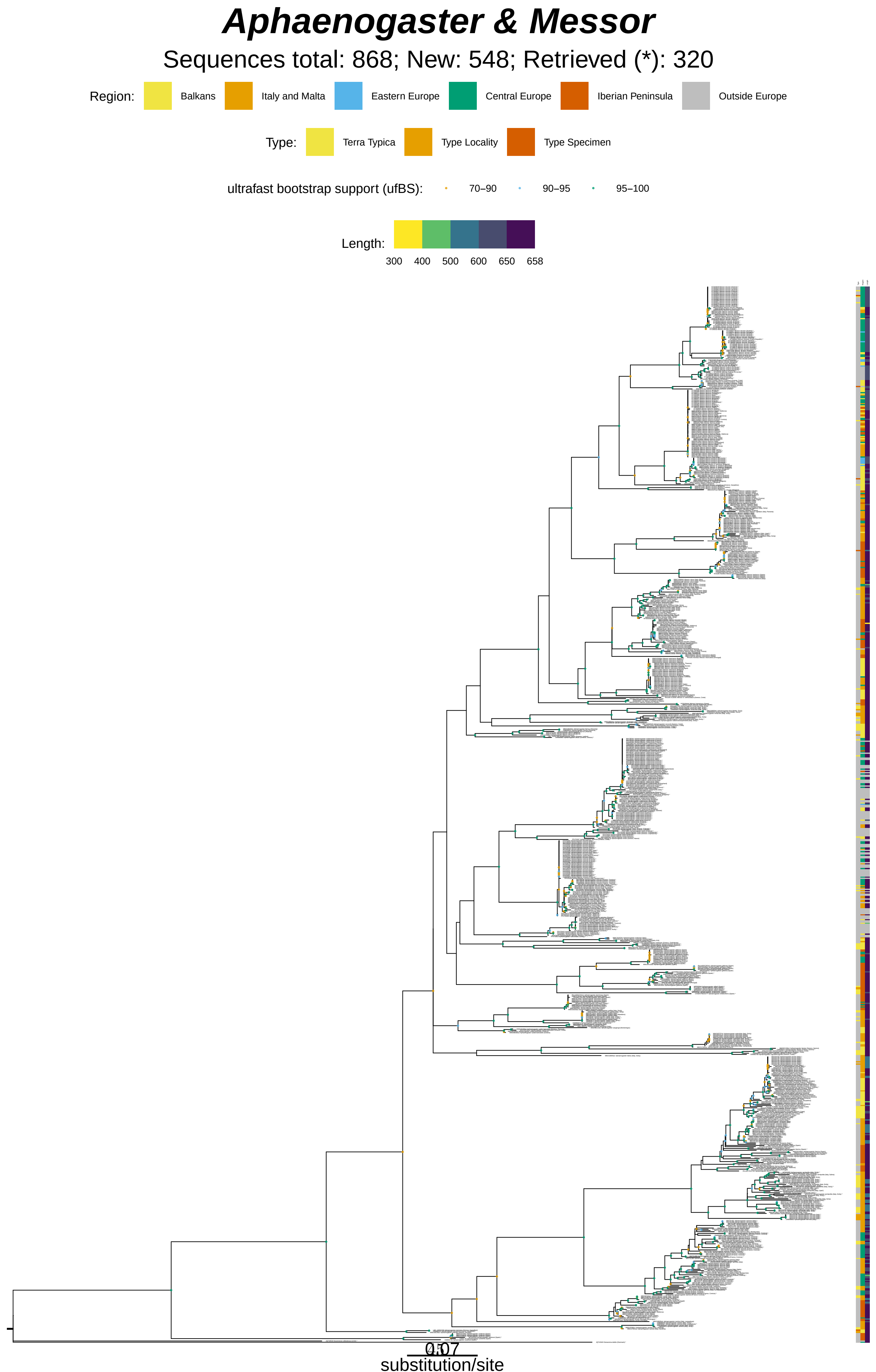
Aphaenogaster & Messor
Sequences total: 868; New: 548; Retrieved (*): 320
Region:
Balkans
Italy and Malta
Eastern Europe
Central Europe
Iberian Peninsula
Outside Europe
Type:
Terra Typica
Type Locality
Type Specimen
ultrafast bootstrap support (ufBS):
70–90
90–95
95–100
Length:
300
400
500
600
650
658
0.07
substitution/site

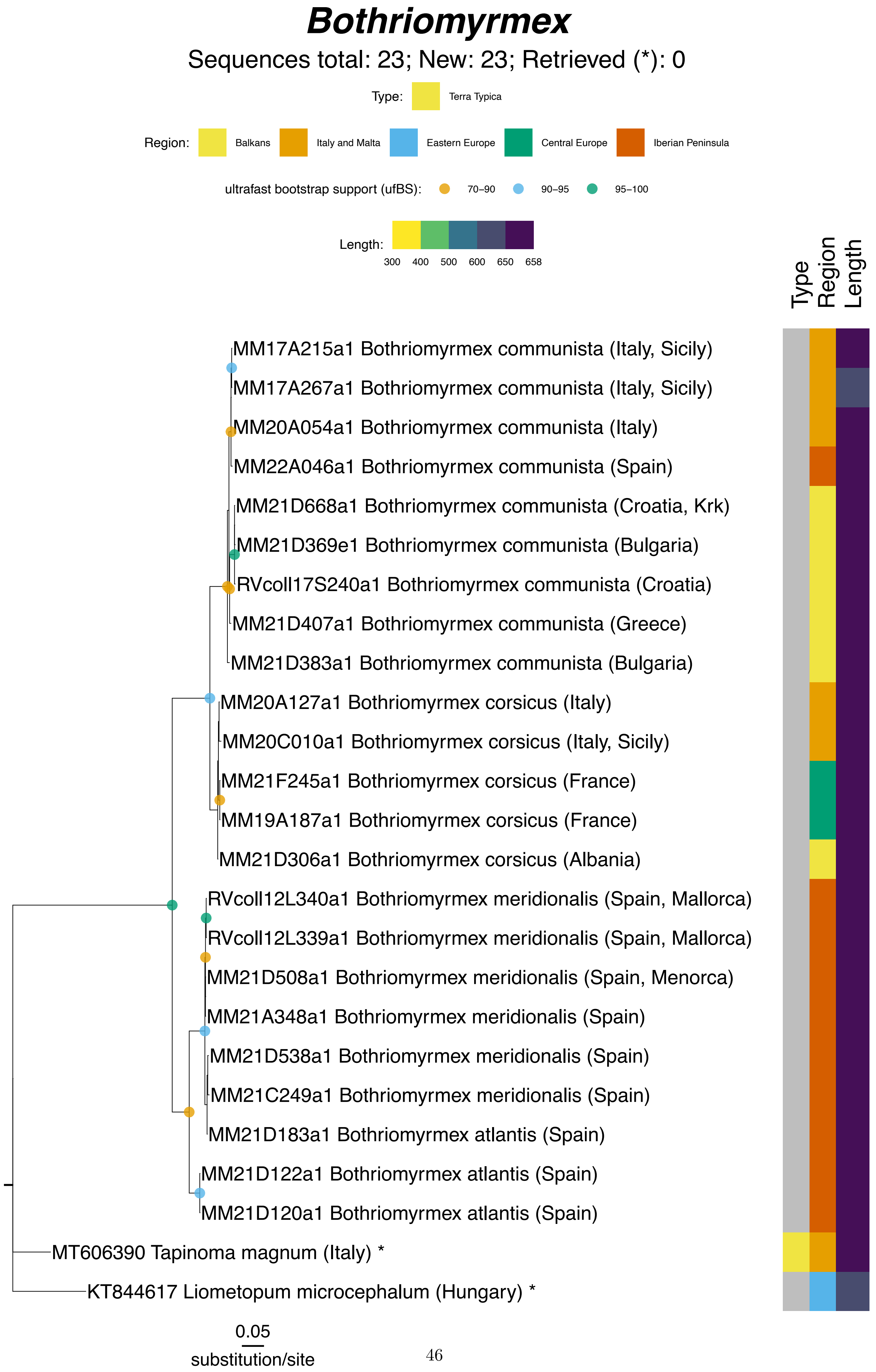

Bothriomyrmex
Sequences total: 23; New: 23; Retrieved (*): 0
Type:
Terra Typica
Region:
Balkans
Italy and Malta
Eastern Europe
Central Europe
Iberian Peninsula
ultrafast bootstrap support (ufBS):
70–90
90–95
95–100
Length:
300
400
500
600
650
658
Type
Region
Length
MM17A215a1 Bothriomyrmex communista (Italy, Sicily)
MM17A267a1 Bothriomyrmex communista (Italy, Sicily)
MM20A054a1 Bothriomyrmex communista (Italy)
MM22A046a1 Bothriomyrmex communista (Spain)
MM21D668a1 Bothriomyrmex communista (Croatia, Krk)
MM21D369e1 Bothriomyrmex communista (Bulgaria)
RVcoll17S240a1 Bothriomyrmex communista (Croatia)
MM21D407a1 Bothriomyrmex communista (Greece)
MM21D383a1 Bothriomyrmex communista (Bulgaria)
MM20A127a1 Bothriomyrmex corsicus (Italy)
MM20C010a1 Bothriomyrmex corsicus (Italy, Sicily)
MM21F245a1 Bothriomyrmex corsicus (France)
MM19A187a1 Bothriomyrmex corsicus (France)
MM21D306a1 Bothriomyrmex corsicus (Albania)
RVcoll12L340a1 Bothriomyrmex meridionalis (Spain, Mallorca)
RVcoll12L339a1 Bothriomyrmex meridionalis (Spain, Mallorca)
MM21D508a1 Bothriomyrmex meridionalis (Spain, Menorca)
MM21A348a1 Bothriomyrmex meridionalis (Spain)
MM21D538a1 Bothriomyrmex meridionalis (Spain)
MM21C249a1 Bothriomyrmex meridionalis (Spain)
MM21D183a1 Bothriomyrmex atlantis (Spain)
MM21D122a1 Bothriomyrmex atlantis (Spain)
MM21D120a1 Bothriomyrmex atlantis (Spain)
MT606390 Tapinoma magnum (Italy) *
KT844617 Liometopum microcephalum (Hungary) *
0.05
substitution/site

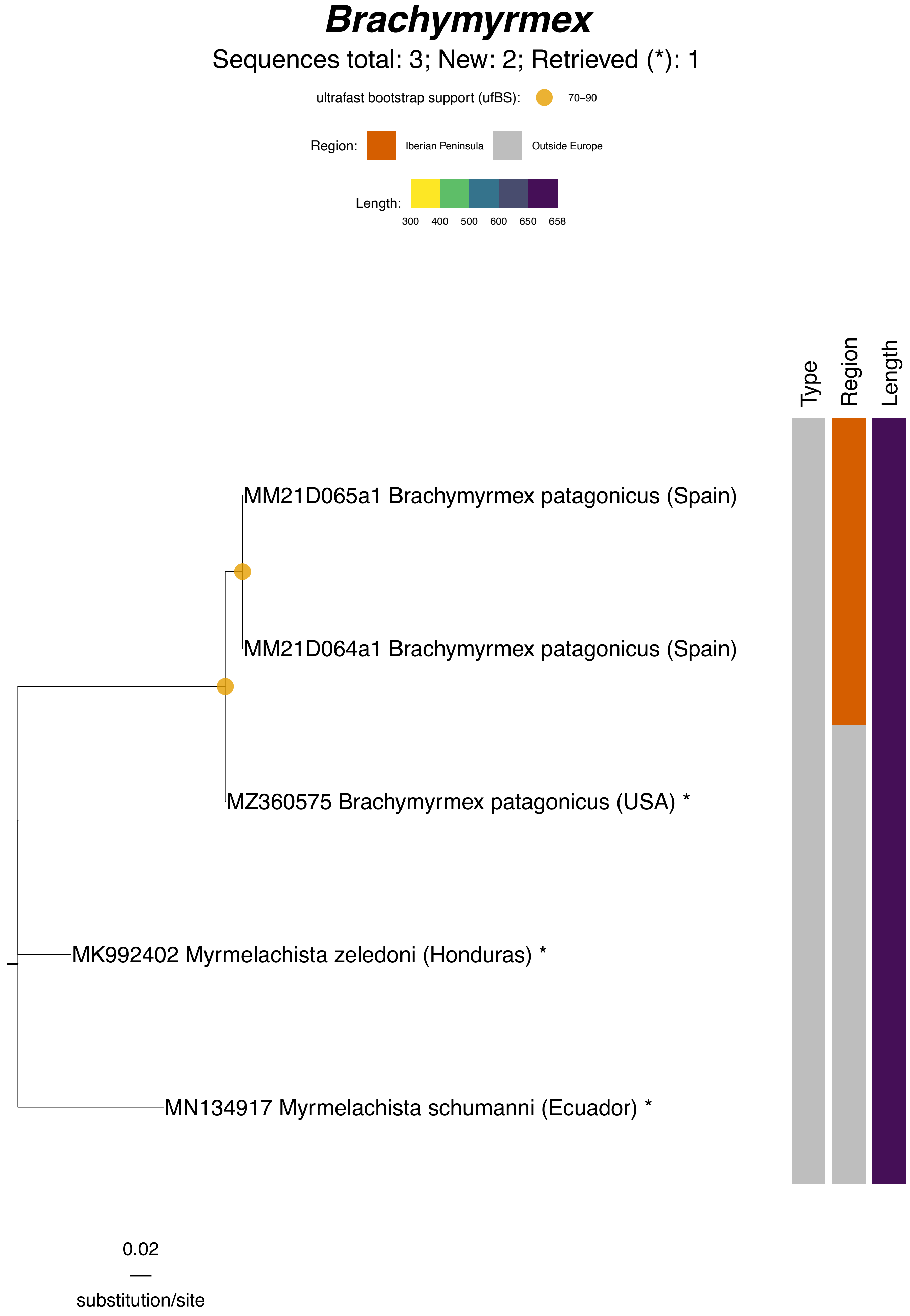
Brachymyrmex
Sequences total: 3; New: 2; Retrieved (*): 1
ultrafast bootstrap support (ufBS): 70–90
Region: Iberian Peninsula Outside Europe
Length: 300 400 500 600 650 658
Type
Region
Length
MM21D065a1 Brachymyrmex patagonicus (Spain)
MM21D064a1 Brachymyrmex patagonicus (Spain)
MZ360575 Brachymyrmex patagonicus (USA) *
MK992402 Myrmelachista zeledoni (Honduras) *
MN134917 Myrmelachista schumanni (Ecuador) *
0.02
substitution/site

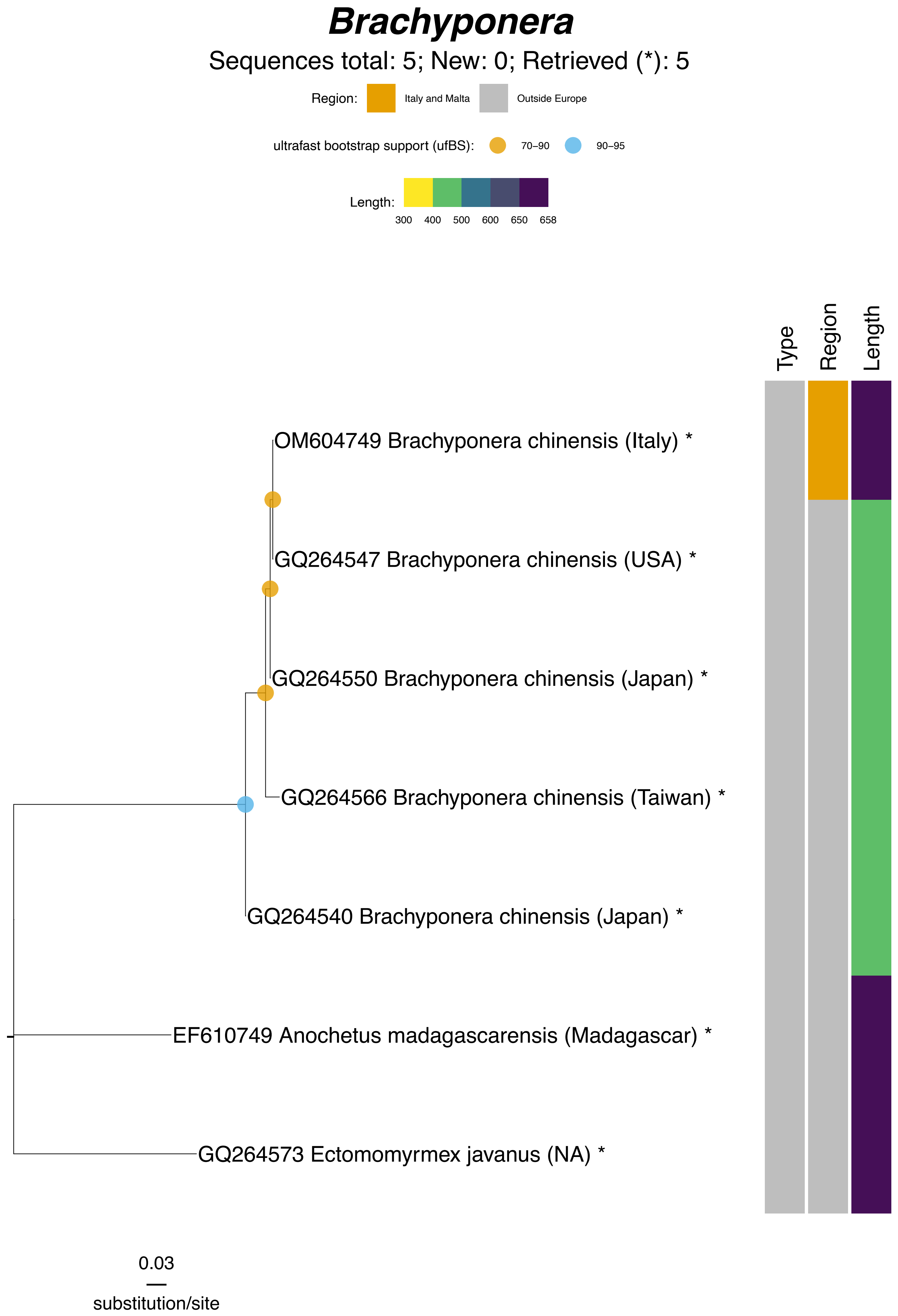

Brachyponera
Sequences total: 5; New: 0; Retrieved (*): 5
Region:
Italy and Malta
Outside Europe
ultrafast bootstrap support (ufBS):
70–90
90–95
Length:
300
400
500
600
650
658
Type
Region
Length
OM604749 Brachyponera chinensis (Italy) *
GQ264547 Brachyponera chinensis (USA) *
GQ264550 Brachyponera chinensis (Japan) *
GQ264566 Brachyponera chinensis (Taiwan) *
GQ264540 Brachyponera chinensis (Japan) *
EF610749 Anochetus madagascarensis (Madagascar) *
GQ264573 Ectomomyrmex javanus (NA) *
0.03
substitution/site

Camponotus

Sequences total: 555; New: 499; Retrieved (*): 56

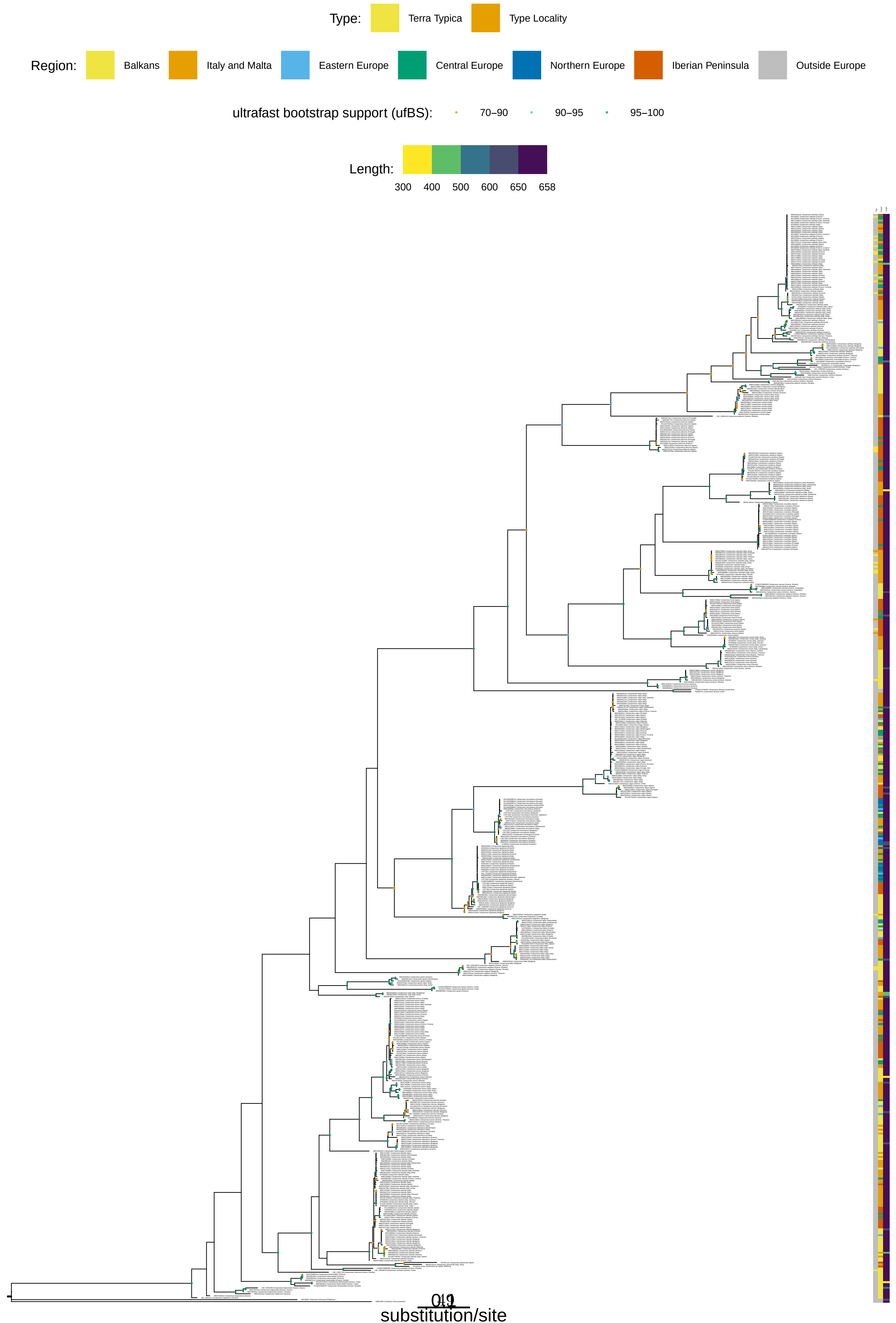

Cardiocondyla

Sequences total: 39; New: 38; Retrieved (*): 1

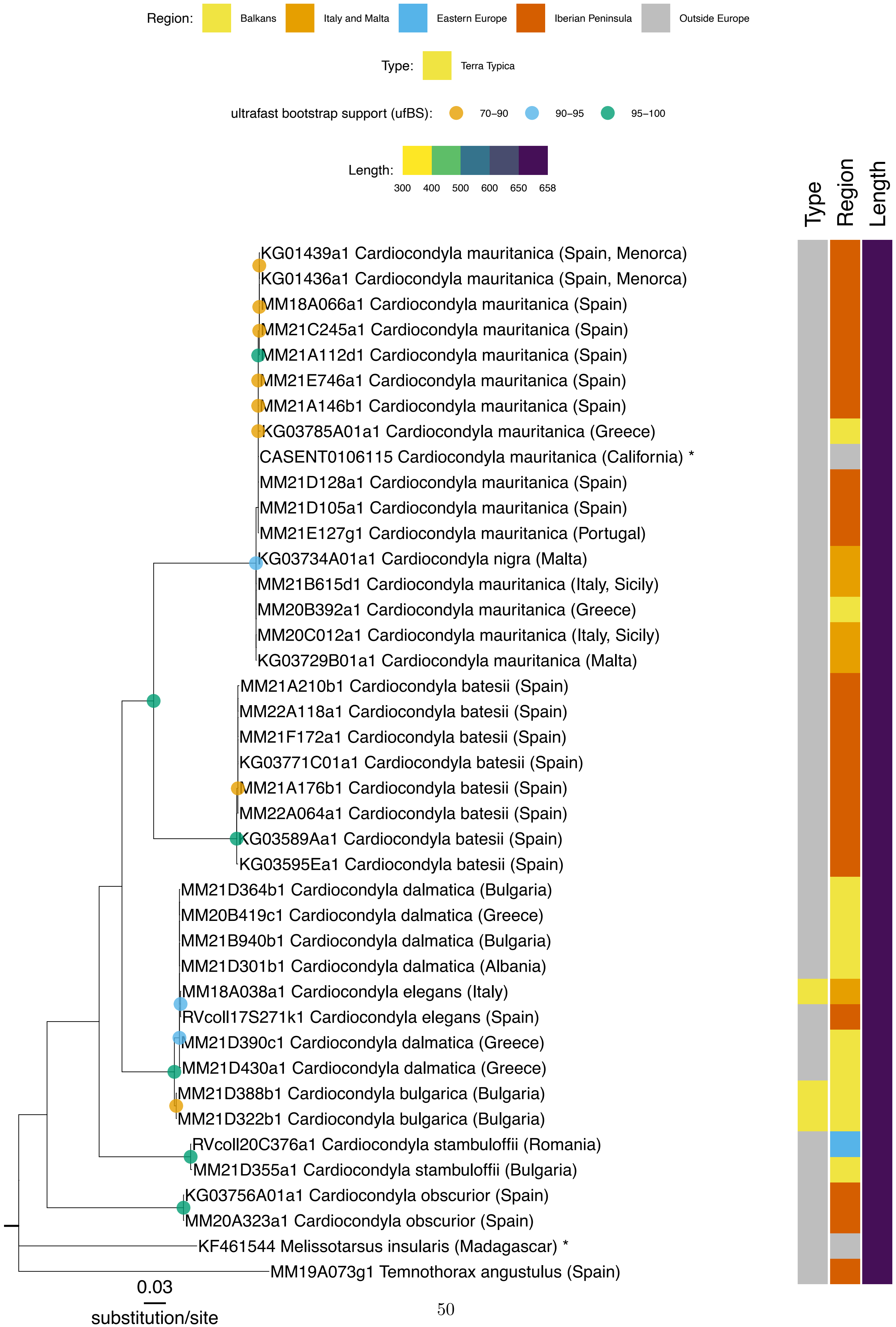

Carebara

Sequences total: 3; New: 1; Retrieved (*): 2

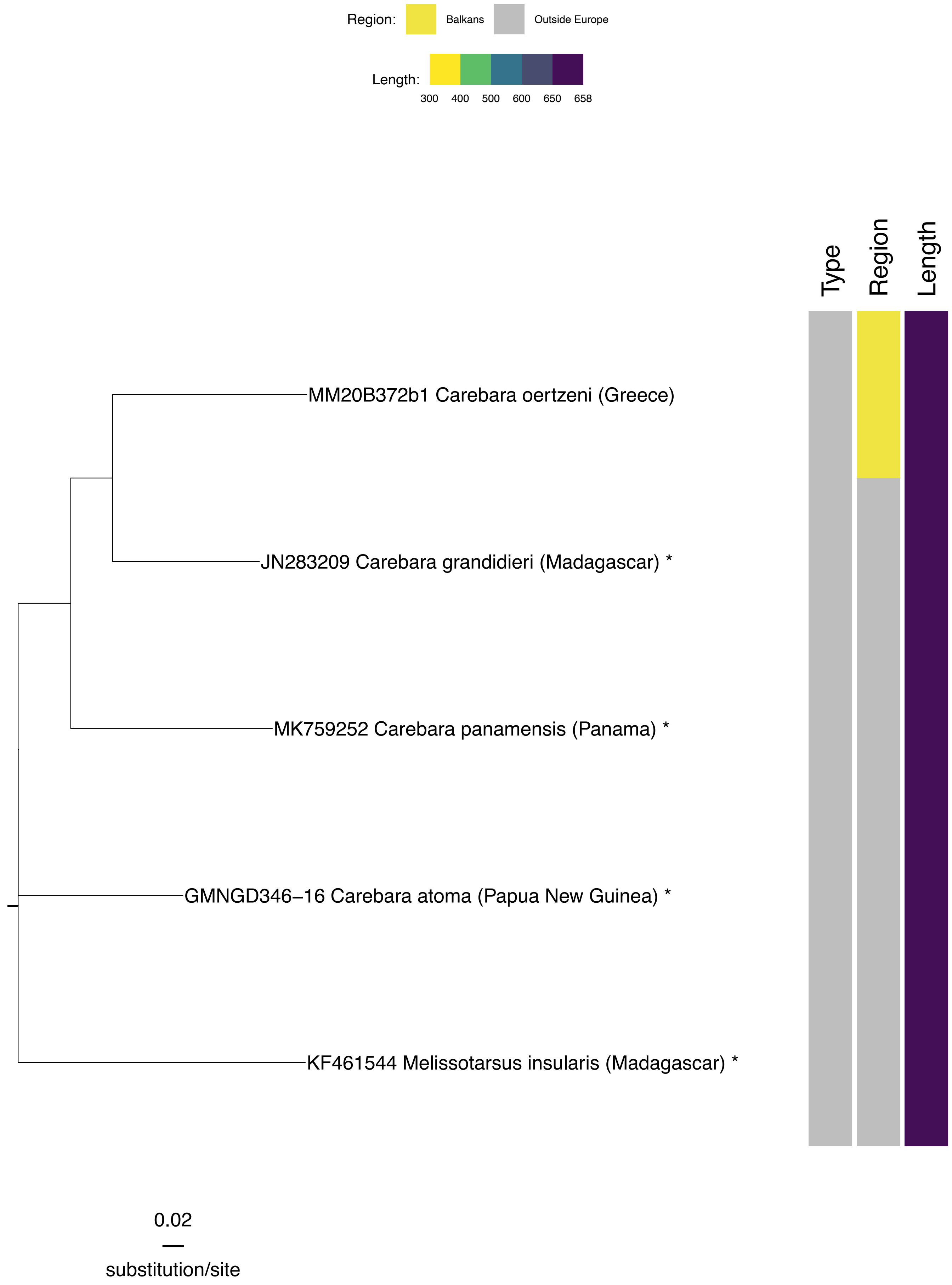

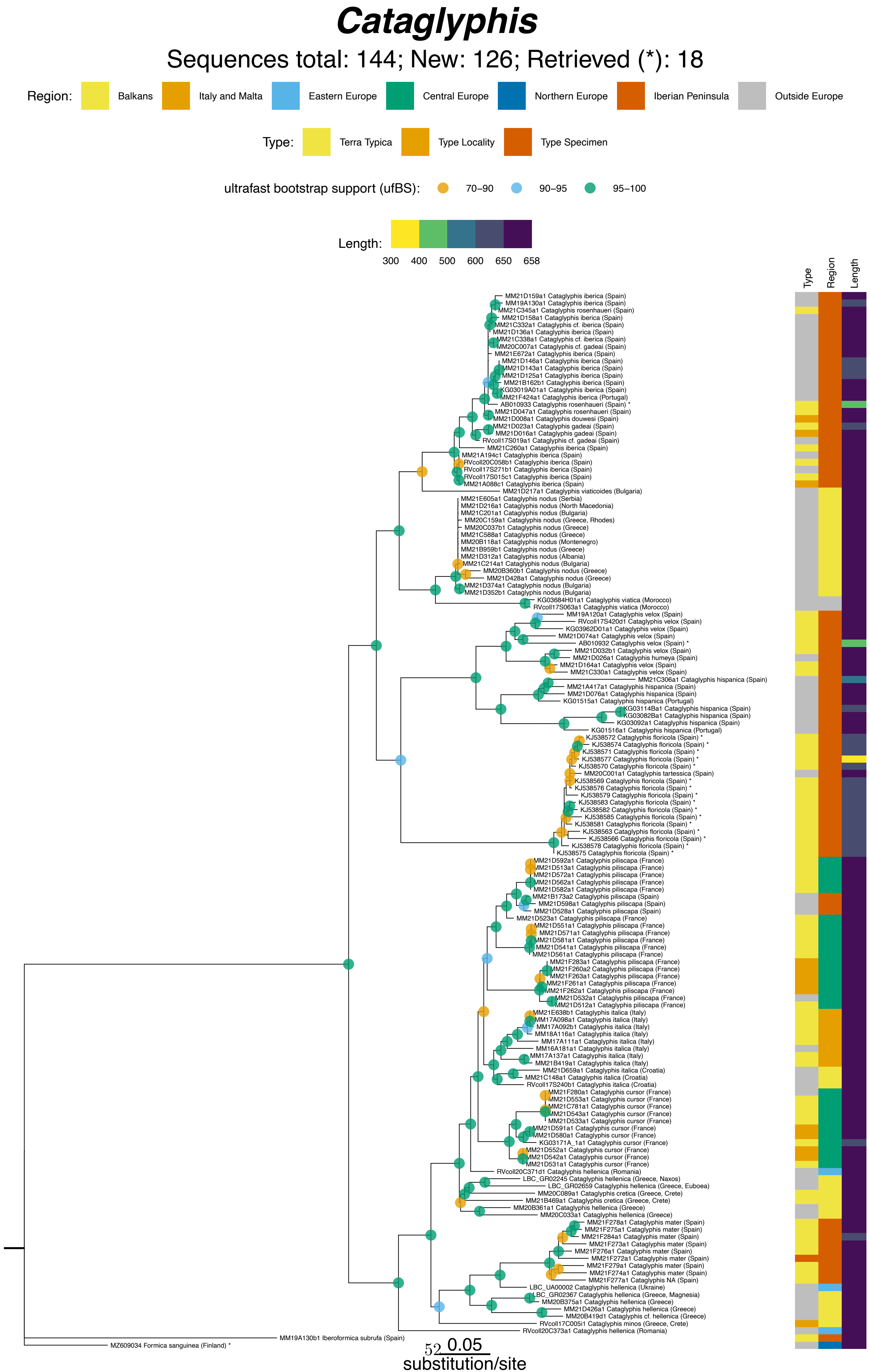

Cataglyphis
Sequences total: 144; New: 126; Retrieved (*): 18
Region: Balkans Italy and Malta Eastern Europe Central Europe Northern Europe Iberian Peninsula Outside Europe
Type: Terra Typica Type Locality Type Specimen
ultrafast bootstrap support (ufBS): 70–90 90–95 95–100
Length: 300 400 500 600 650 658
Type
Region
Length
MM21D159a1 Cataglyphis iberica (Spain)
MM19A130a1 Cataglyphis iberica (Spain)
MM21C345a1 Cataglyphis rosenhaueri (Spain)
MM21D158a1 Cataglyphis iberica (Spain)
MM21C332a1 Cataglyphis cf. iberica (Spain)
MM21D136a1 Cataglyphis iberica (Spain)
MM21C338a1 Cataglyphis cf. iberica (Spain)
MM20C007a1 Cataglyphis cf. gadeai (Spain)
MM21E672a1 Cataglyphis iberica (Spain)
MM21D146a1 Cataglyphis iberica (Spain)
MM21D143a1 Cataglyphis iberica (Spain)
MM21D125a1 Cataglyphis iberica (Spain)
MM21B162b1 Cataglyphis iberica (Spain)
KG03019A01a1 Cataglyphis iberica (Spain)
MM21F424a1 Cataglyphis iberica (Portugal)
AB010933 Cataglyphis rosenhaueri (Spain) *
MM21D047a1 Cataglyphis rosenhaueri (Spain)
MM21D008a1 Cataglyphis douwesi (Spain)
MM21D023a1 Cataglyphis gadeai (Spain)
MM21D016a1 Cataglyphis gadeai (Spain)
RVcoll17S019a1 Cataglyphis cf. gadeai (Spain)
MM21C260a1 Cataglyphis iberica (Spain)
MM21A194c1 Cataglyphis iberica (Spain)
RVcoll20C058b1 Cataglyphis iberica (Spain)
RVcoll17S271b1 Cataglyphis iberica (Spain)
RVcoll17S015c1 Cataglyphis iberica (Spain)
MM21A088c1 Cataglyphis iberica (Spain)
MM21D217a1 Cataglyphis viaticoides (Bulgaria)
MM21E605a1 Cataglyphis nodus (Serbia)
MM21D216a1 Cataglyphis nodus (North Macedonia)
MM21C201a1 Cataglyphis nodus (Bulgaria)
MM20C159a1 Cataglyphis nodus (Greece, Rhodes)
MM20C037b1 Cataglyphis nodus (Greece)
MM21C588a1 Cataglyphis nodus (Greece)
MM20B118a1 Cataglyphis nodus (Montenegro)
MM21B959b1 Cataglyphis nodus (Greece)
MM21D312a1 Cataglyphis nodus (Albania)
MM21C214a1 Cataglyphis nodus (Bulgaria)
MM20B360b1 Cataglyphis nodus (Greece)
MM21D428a1 Cataglyphis nodus (Greece)
MM21D374a1 Cataglyphis nodus (Bulgaria)
MM21D352b1 Cataglyphis nodus (Bulgaria)
KG03684H01a1 Cataglyphis viatica (Morocco)
RVcoll17S063a1 Cataglyphis viatica (Morocco)
MM19A120a1 Cataglyphis velox (Spain)
RVcoll17S420d1 Cataglyphis velox (Spain)
KG03962D01a1 Cataglyphis velox (Spain)
MM21D074a1 Cataglyphis velox (Spain)
AB010932 Cataglyphis velox (Spain) *
MM21D032b1 Cataglyphis velox (Spain)
MM21D026a1 Cataglyphis humeya (Spain)
MM21D164a1 Cataglyphis velox (Spain)
MM21C330a1 Cataglyphis velox (Spain)
MM21C306a1 Cataglyphis hispanica (Spain)
MM21A417a1 Cataglyphis hispanica (Spain)
MM21D076a1 Cataglyphis hispanica (Spain)
KG01515a1 Cataglyphis hispanica (Portugal)
KG03114Ba1 Cataglyphis hispanica (Spain)
KG03082Ba1 Cataglyphis hispanica (Spain)
KG03092a1 Cataglyphis hispanica (Spain)
KG01516a1 Cataglyphis hispanica (Portugal)
KJ538572 Cataglyphis floricola (Spain) *
KJ538574 Cataglyphis floricola (Spain) *
KJ538571 Cataglyphis floricola (Spain) *
KJ538577 Cataglyphis floricola (Spain) *
KJ538570 Cataglyphis floricola (Spain) *
MM20C001a1 Cataglyphis tartessica (Spain)
KJ538569 Cataglyphis floricola (Spain) *
KJ538576 Cataglyphis floricola (Spain) *
KJ538579 Cataglyphis floricola (Spain) *
KJ538583 Cataglyphis floricola (Spain) *
KJ538582 Cataglyphis floricola (Spain) *
KJ538585 Cataglyphis floricola (Spain) *
KJ538581 Cataglyphis floricola (Spain) *
KJ538563 Cataglyphis floricola (Spain) *
KJ538566 Cataglyphis floricola (Spain) *
KJ538578 Cataglyphis floricola (Spain) *
KJ538575 Cataglyphis floricola (Spain) *
MM21D592a1 Cataglyphis piliscapa (France)
MM21D513a1 Cataglyphis piliscapa (France)
MM21D572a1 Cataglyphis piliscapa (France)
MM21D562a1 Cataglyphis piliscapa (France)
MM21D582a1 Cataglyphis piliscapa (France)
MM21B173a2 Cataglyphis piliscapa (Spain)
MM21D598a1 Cataglyphis piliscapa (Spain)
MM21D528a1 Cataglyphis piliscapa (Spain)
MM21D523a1 Cataglyphis piliscapa (France)
MM21D551a1 Cataglyphis piliscapa (France)
MM21D571a1 Cataglyphis piliscapa (France)
MM21D581a1 Cataglyphis piliscapa (France)
MM21D541a1 Cataglyphis piliscapa (France)
MM21D561a1 Cataglyphis piliscapa (France)
MM21F283a1 Cataglyphis piliscapa (France)
MM21F260a2 Cataglyphis piliscapa (France)
MM21F263a1 Cataglyphis piliscapa (France)
MM21F261a1 Cataglyphis piliscapa (France)
MM21F262a1 Cataglyphis piliscapa (France)
MM21D532a1 Cataglyphis piliscapa (France)
MM21D512a1 Cataglyphis piliscapa (France)
MM21E638b1 Cataglyphis italica (Italy)
MM17A098a1 Cataglyphis italica (Italy)
MM17A092b1 Cataglyphis italica (Italy)
MM18A116a1 Cataglyphis italica (Italy)
MM17A111a1 Cataglyphis italica (Italy)
MM16A181a1 Cataglyphis italica (Italy)
MM17A137a1 Cataglyphis italica (Italy)
MM21B419a1 Cataglyphis italica (Italy)
MM21D659a1 Cataglyphis italica (Croatia)
MM21C148a1 Cataglyphis italica (Croatia)
RVcoll17S240b1 Cataglyphis italica (Croatia)
MM21F280a1 Cataglyphis cursor (France)
MM21D553a1 Cataglyphis cursor (France)
MM21C781a1 Cataglyphis cursor (France)
MM21D543a1 Cataglyphis cursor (France)
MM21D533a1 Cataglyphis cursor (France)
MM21D591a1 Cataglyphis cursor (France)
MM21D580a1 Cataglyphis cursor (France)
KG03171A_1a1 Cataglyphis cursor (France)
MM21D552a1 Cataglyphis cursor (France)
MM21D542a1 Cataglyphis cursor (France)
MM21D531a1 Cataglyphis cursor (France)
RVcoll20C371d1 Cataglyphis hellenica (Romania)
LBC_GR02245 Cataglyphis hellenica (Greece, Naxos)
LBC_GR02659 Cataglyphis hellenica (Greece, Euboea)
MM20C089a1 Cataglyphis cretica (Greece, Crete)
MM21B469a1 Cataglyphis cretica (Greece, Crete)
MM20B361a1 Cataglyphis hellenica (Greece)
MM20C033a1 Cataglyphis hellenica (Greece)
MM21F278a1 Cataglyphis mater (Spain)
MM21F275a1 Cataglyphis mater (Spain)
MM21F284a1 Cataglyphis mater (Spain)
MM21F273a1 Cataglyphis mater (Spain)
MM21F276a1 Cataglyphis mater (Spain)
MM21F272a1 Cataglyphis mater (Spain)
MM21F279a1 Cataglyphis mater (Spain)
MM21F274a1 Cataglyphis mater (Spain)
MM21F277a1 Cataglyphis NA (Spain)
LBC_UA00002 Cataglyphis hellenica (Ukraine)
LBC_GR02367 Cataglyphis hellenica (Greece, Magnesia)
MM20B375a1 Cataglyphis hellenica (Greece)
MM21D426a1 Cataglyphis hellenica (Greece)
MM20B419d1 Cataglyphis cf. hellenica (Greece)
RVcoll17C005i1 Cataglyphis minos (Greece, Crete)
RVcoll20C373a1 Cataglyphis hellenica (Romania)
MM19A130b1 Iberoformica subrufa (Spain)
MZ609034 Formica sanguinea (Finland) *
0.05
substitution/site

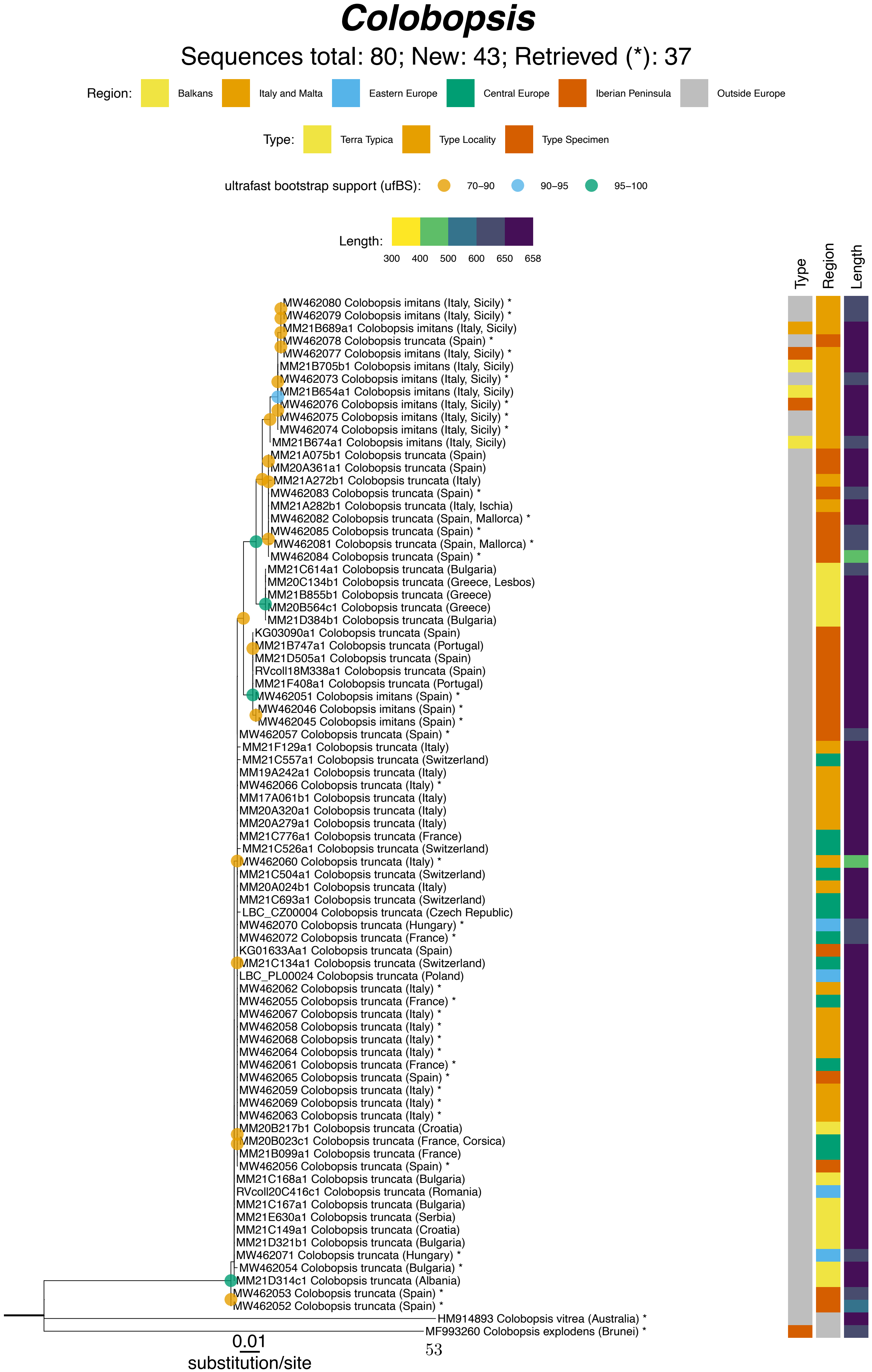
Colobopsis
Sequences total: 80; New: 43; Retrieved (*): 37
Region: Balkans Italy and Malta Eastern Europe Central Europe Iberian Peninsula Outside Europe
Type: Terra Typica Type Locality Type Specimen
ultrafast bootstrap support (ufBS): 70–90 90–95 95–100
Length: 300 400 500 600 650 658
Type
Region
Length
MW462080 Colobopsis imitans (Italy, Sicily) *
MW462079 Colobopsis imitans (Italy, Sicily) *
MM21B689a1 Colobopsis imitans (Italy, Sicily)
MW462078 Colobopsis truncata (Spain) *
MW462077 Colobopsis imitans (Italy, Sicily) *
MM21B705b1 Colobopsis imitans (Italy, Sicily)
MW462073 Colobopsis imitans (Italy, Sicily) *
MM21B654a1 Colobopsis imitans (Italy, Sicily)
MW462076 Colobopsis imitans (Italy, Sicily) *
MW462075 Colobopsis imitans (Italy, Sicily) *
MW462074 Colobopsis imitans (Italy, Sicily) *
MM21B674a1 Colobopsis imitans (Italy, Sicily)
MM21A075b1 Colobopsis truncata (Spain)
MM20A361a1 Colobopsis truncata (Spain)
MM21A272b1 Colobopsis truncata (Italy)
MW462083 Colobopsis truncata (Spain) *
MM21A282b1 Colobopsis truncata (Italy, Ischia)
MW462082 Colobopsis truncata (Spain, Mallorca) *
MW462085 Colobopsis truncata (Spain) *
MW462081 Colobopsis truncata (Spain, Mallorca) *
MW462084 Colobopsis truncata (Spain) *
MM21C614a1 Colobopsis truncata (Bulgaria)
MM20C134b1 Colobopsis truncata (Greece, Lesbos)
MM21B855b1 Colobopsis truncata (Greece)
MM20B564c1 Colobopsis truncata (Greece)
MM21D384b1 Colobopsis truncata (Bulgaria)
KG03090a1 Colobopsis truncata (Spain)
MM21B747a1 Colobopsis truncata (Portugal)
MM21D505a1 Colobopsis truncata (Spain)
RVcoll18M338a1 Colobopsis truncata (Spain)
MM21F408a1 Colobopsis truncata (Portugal)
MW462051 Colobopsis imitans (Spain) *
MW462046 Colobopsis imitans (Spain) *
MW462045 Colobopsis imitans (Spain) *
MW462057 Colobopsis truncata (Spain) *
MM21F129a1 Colobopsis truncata (Italy)
MM21C557a1 Colobopsis truncata (Switzerland)
MM19A242a1 Colobopsis truncata (Italy)
MW462066 Colobopsis truncata (Italy) *
MM17A061b1 Colobopsis truncata (Italy)
MM20A320a1 Colobopsis truncata (Italy)
MM20A279a1 Colobopsis truncata (Italy)
MM21C776a1 Colobopsis truncata (France)
MM21C526a1 Colobopsis truncata (Switzerland)
MW462060 Colobopsis truncata (Italy) *
MM21C504a1 Colobopsis truncata (Switzerland)
MM20A024b1 Colobopsis truncata (Italy)
MM21C693a1 Colobopsis truncata (Switzerland)
LBC_CZ00004 Colobopsis truncata (Czech Republic)
MW462070 Colobopsis truncata (Hungary) *
MW462072 Colobopsis truncata (France) *
KG01633Aa1 Colobopsis truncata (Spain)
MM21C134a1 Colobopsis truncata (Switzerland)
LBC_PL00024 Colobopsis truncata (Poland)
MW462062 Colobopsis truncata (Italy) *
MW462055 Colobopsis truncata (France) *
MW462067 Colobopsis truncata (Italy) *
MW462058 Colobopsis truncata (Italy) *
MW462068 Colobopsis truncata (Italy) *
MW462064 Colobopsis truncata (Italy) *
MW462061 Colobopsis truncata (France) *
MW462065 Colobopsis truncata (Spain) *
MW462059 Colobopsis truncata (Italy) *
MW462069 Colobopsis truncata (Italy) *
MW462063 Colobopsis truncata (Italy) *
MM20B217b1 Colobopsis truncata (Croatia)
MM20B023c1 Colobopsis truncata (France, Corsica)
MM21B099a1 Colobopsis truncata (France)
MW462056 Colobopsis truncata (Spain) *
MM21C168a1 Colobopsis truncata (Bulgaria)
RVcoll20C416c1 Colobopsis truncata (Romania)
MM21C167a1 Colobopsis truncata (Bulgaria)
MM21E630a1 Colobopsis truncata (Serbia)
MM21C149a1 Colobopsis truncata (Croatia)
MM21D321b1 Colobopsis truncata (Bulgaria)
MW462071 Colobopsis truncata (Hungary) *
MW462054 Colobopsis truncata (Bulgaria) *
MM21D314c1 Colobopsis truncata (Albania)
MW462053 Colobopsis truncata (Spain) *
MW462052 Colobopsis truncata (Spain) *
HM914893 Colobopsis vitrea (Australia) *
MF993260 Colobopsis explodens (Brunei) *
0.01
substitution/site

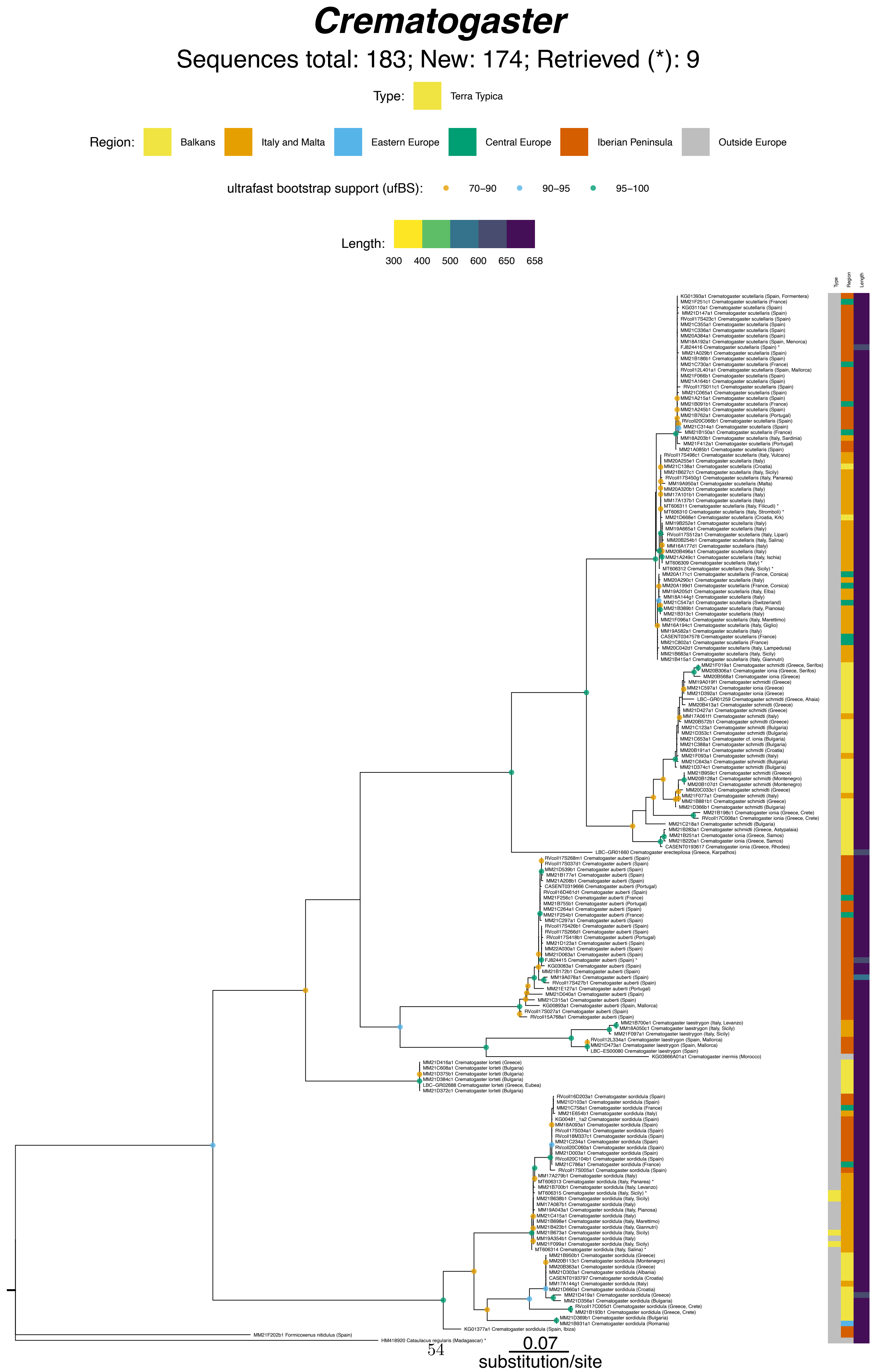
Crematogaster
Sequences total: 183; New: 174; Retrieved (*): 9
Type:
Terra Typica
Region:
Balkans
Italy and Malta
Eastern Europe
Central Europe
Iberian Peninsula
Outside Europe
ultrafast bootstrap support (ufBS):
70–90
90–95
95–100
Length:
300
400
500
600
650
658
Type
Region
Length
0.07
substitution/site

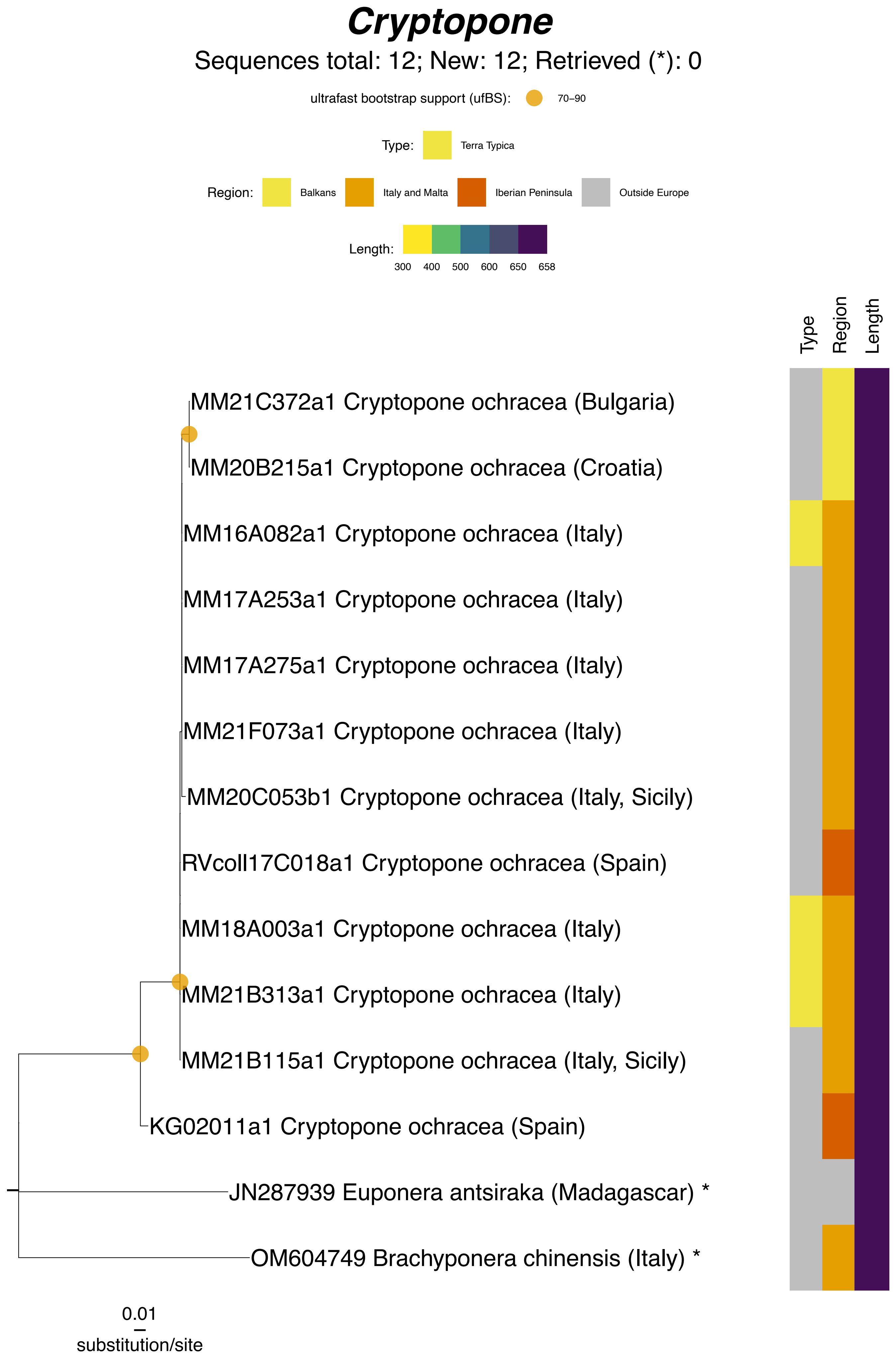

Cryptopone
Sequences total: 12; New: 12; Retrieved (*): 0
ultrafast bootstrap support (ufBS):
70–90
Type:
Terra Typica
Region:
Balkans
Italy and Malta
Iberian Peninsula
Outside Europe
Length:
300
400
500
600
650
658
Type
Region
Length
MM21C372a1 Cryptopone ochracea (Bulgaria)
MM20B215a1 Cryptopone ochracea (Croatia)
MM16A082a1 Cryptopone ochracea (Italy)
MM17A253a1 Cryptopone ochracea (Italy)
MM17A275a1 Cryptopone ochracea (Italy)
MM21F073a1 Cryptopone ochracea (Italy)
MM20C053b1 Cryptopone ochracea (Italy, Sicily)
RVcoll17C018a1 Cryptopone ochracea (Spain)
MM18A003a1 Cryptopone ochracea (Italy)
MM21B313a1 Cryptopone ochracea (Italy)
MM21B115a1 Cryptopone ochracea (Italy, Sicily)
KG02011a1 Cryptopone ochracea (Spain)
JN287939 Euponera antsiraka (Madagascar) *
OM604749 Brachyponera chinensis (Italy) *
0.01
substitution/site

Dolichoderus

Sequences total: 56; New: 38; Retrieved (*): 18

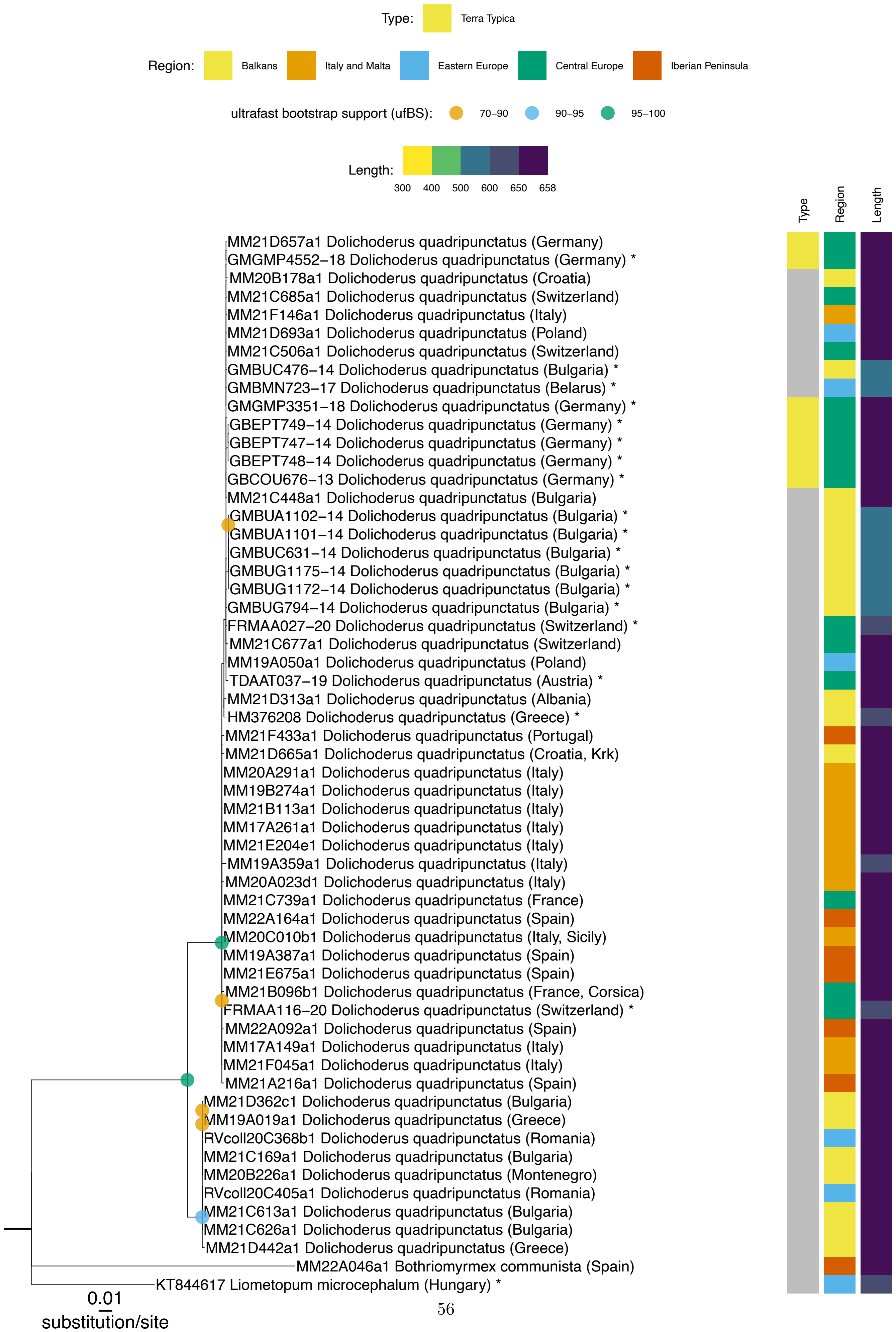

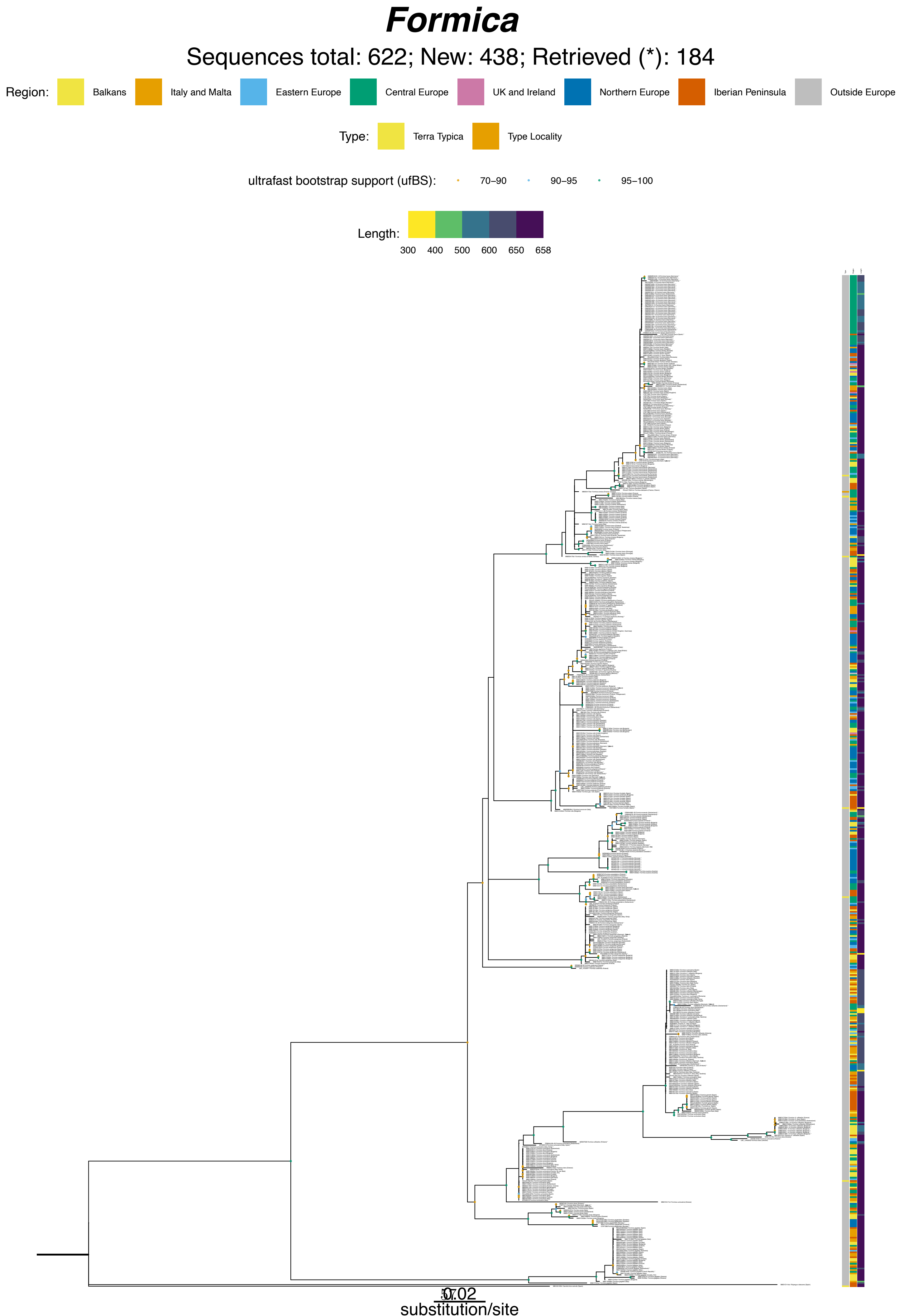
Formica
Sequences total: 622; New: 438; Retrieved (*): 184
Region:
Balkans
Italy and Malta
Eastern Europe
Central Europe
UK and Ireland
Northern Europe
Iberian Peninsula
Outside Europe
Type:
Terra Typica
Type Locality
ultrafast bootstrap support (ufBS):
70–90
90–95
95–100
Length:
300
400
500
600
650
658
0.02
substitution/site

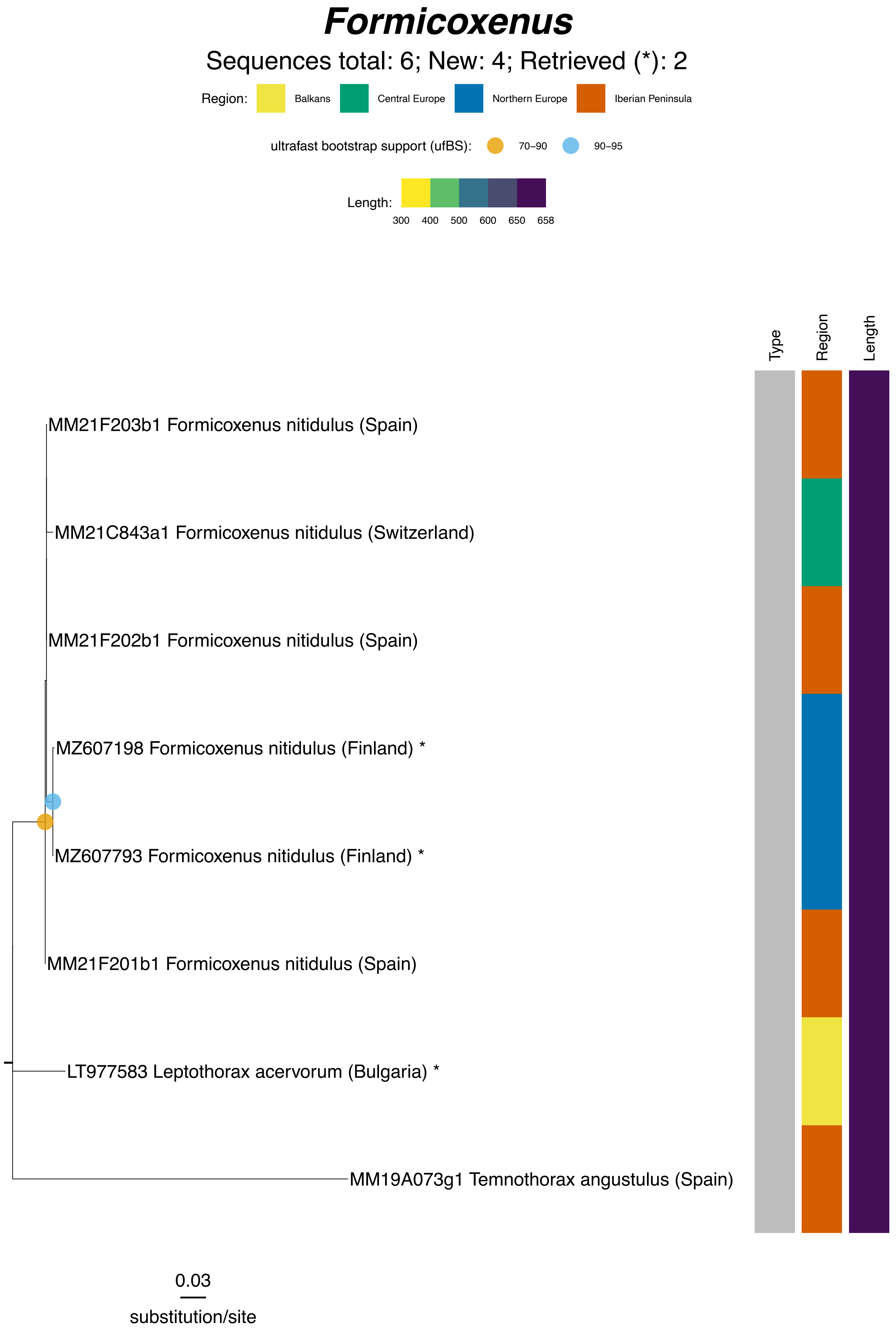
Formicoxenus
Sequences total: 6; New: 4; Retrieved (*): 2
Region:
Balkans
Central Europe
Northern Europe
Iberian Peninsula
ultrafast bootstrap support (ufBS):
70–90
90–95
Length:
300
400
500
600
650
658
Type
Region
Length
MM21F203b1 Formicoxenus nitidulus (Spain)
MM21C843a1 Formicoxenus nitidulus (Switzerland)
MM21F202b1 Formicoxenus nitidulus (Spain)
MZ607198 Formicoxenus nitidulus (Finland) *
MZ607793 Formicoxenus nitidulus (Finland) *
MM21F201b1 Formicoxenus nitidulus (Spain)
LT977583 Leptothorax acervorum (Bulgaria) *
MM19A073g1 Temnothorax angustulus (Spain)
0.03
substitution/site

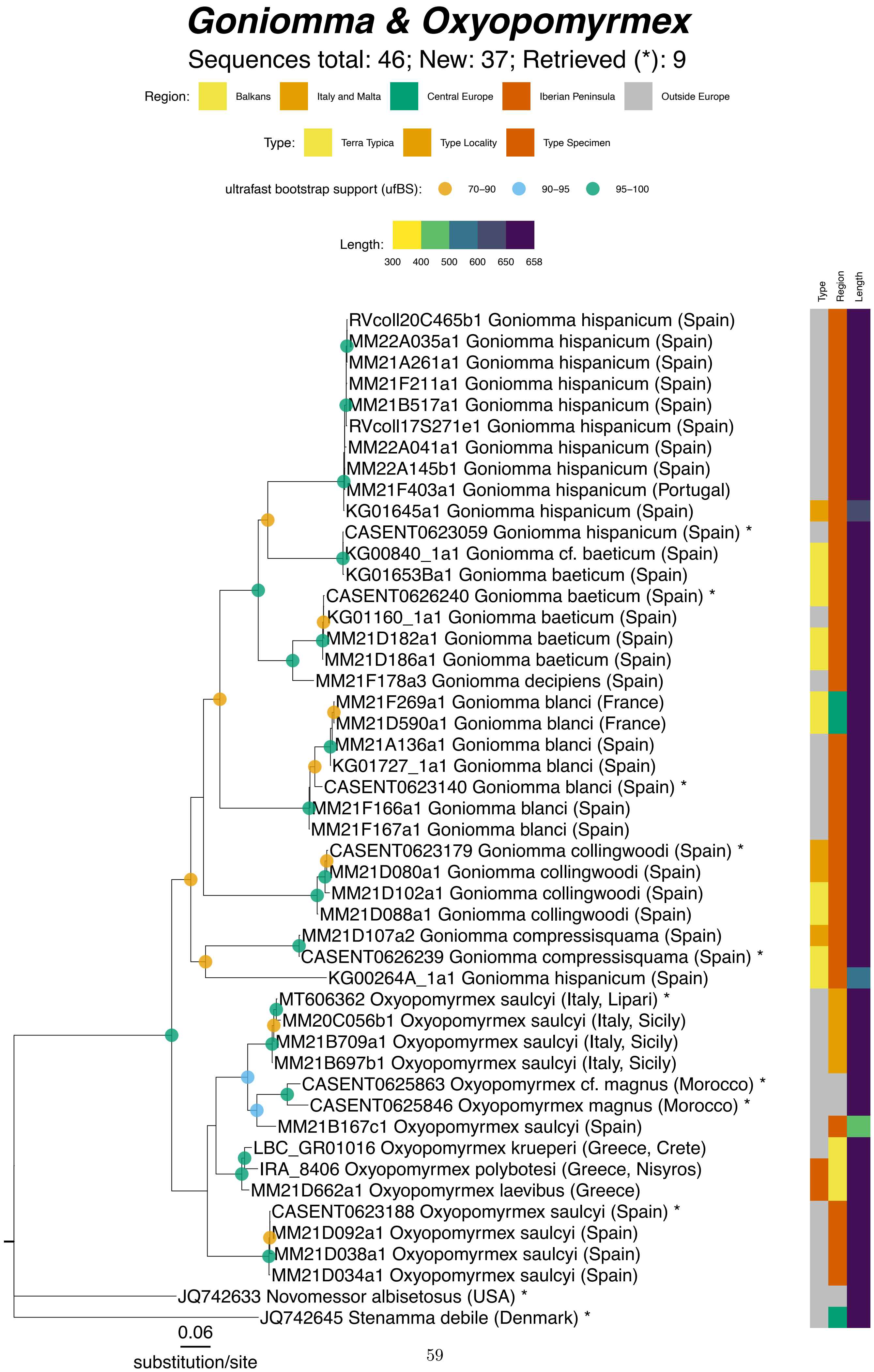
Goniomma & Oxyopomyrmex
Sequences total: 46; New: 37; Retrieved (*): 9
Region:
Balkans
Italy and Malta
Central Europe
Iberian Peninsula
Outside Europe
Type:
Terra Typica
Type Locality
Type Specimen
ultrafast bootstrap support (ufBS):
70–90
90–95
95–100
Length:
300
400
500
600
650
658
Type
Region
Length
RVcoll20C465b1 Goniomma hispanicum (Spain)
MM22A035a1 Goniomma hispanicum (Spain)
MM21A261a1 Goniomma hispanicum (Spain)
MM21F211a1 Goniomma hispanicum (Spain)
MM21B517a1 Goniomma hispanicum (Spain)
RVcoll17S271e1 Goniomma hispanicum (Spain)
MM22A041a1 Goniomma hispanicum (Spain)
MM22A145b1 Goniomma hispanicum (Spain)
MM21F403a1 Goniomma hispanicum (Portugal)
KG01645a1 Goniomma hispanicum (Spain)
CASENT0623059 Goniomma hispanicum (Spain) *
KG00840_1a1 Goniomma cf. baeticum (Spain)
KG01653Ba1 Goniomma baeticum (Spain)
CASENT0626240 Goniomma baeticum (Spain) *
KG01160_1a1 Goniomma baeticum (Spain)
MM21D182a1 Goniomma baeticum (Spain)
MM21D186a1 Goniomma baeticum (Spain)
MM21F178a3 Goniomma decipiens (Spain)
MM21F269a1 Goniomma blanci (France)
MM21D590a1 Goniomma blanci (France)
MM21A136a1 Goniomma blanci (Spain)
KG01727_1a1 Goniomma blanci (Spain)
CASENT0623140 Goniomma blanci (Spain) *
MM21F166a1 Goniomma blanci (Spain)
MM21F167a1 Goniomma blanci (Spain)
CASENT0623179 Goniomma collingwoodi (Spain) *
MM21D080a1 Goniomma collingwoodi (Spain)
MM21D102a1 Goniomma collingwoodi (Spain)
MM21D088a1 Goniomma collingwoodi (Spain)
MM21D107a2 Goniomma compressisquama (Spain)
CASENT0626239 Goniomma compressisquama (Spain) *
KG00264A_1a1 Goniomma hispanicum (Spain)
MT606362 Oxyopomyrmex saulcyi (Italy, Lipari) *
MM20C056b1 Oxyopomyrmex saulcyi (Italy, Sicily)
MM21B709a1 Oxyopomyrmex saulcyi (Italy, Sicily)
MM21B697b1 Oxyopomyrmex saulcyi (Italy, Sicily)
CASENT0625863 Oxyopomyrmex cf. magnus (Morocco) *
CASENT0625846 Oxyopomyrmex magnus (Morocco) *
MM21B167c1 Oxyopomyrmex saulcyi (Spain)
LBC_GR01016 Oxyopomyrmex krueperi (Greece, Crete)
IRA_8406 Oxyopomyrmex polybotesi (Greece, Nisyros)
MM21D662a1 Oxyopomyrmex laevibus (Greece)
CASENT0623188 Oxyopomyrmex saulcyi (Spain) *
MM21D092a1 Oxyopomyrmex saulcyi (Spain)
MM21D038a1 Oxyopomyrmex saulcyi (Spain)
MM21D034a1 Oxyopomyrmex saulcyi (Spain)
JQ742633 Novomessor albisetosus (USA) *
JQ742645 Stenamma debile (Denmark) *
0.06
substitution/site

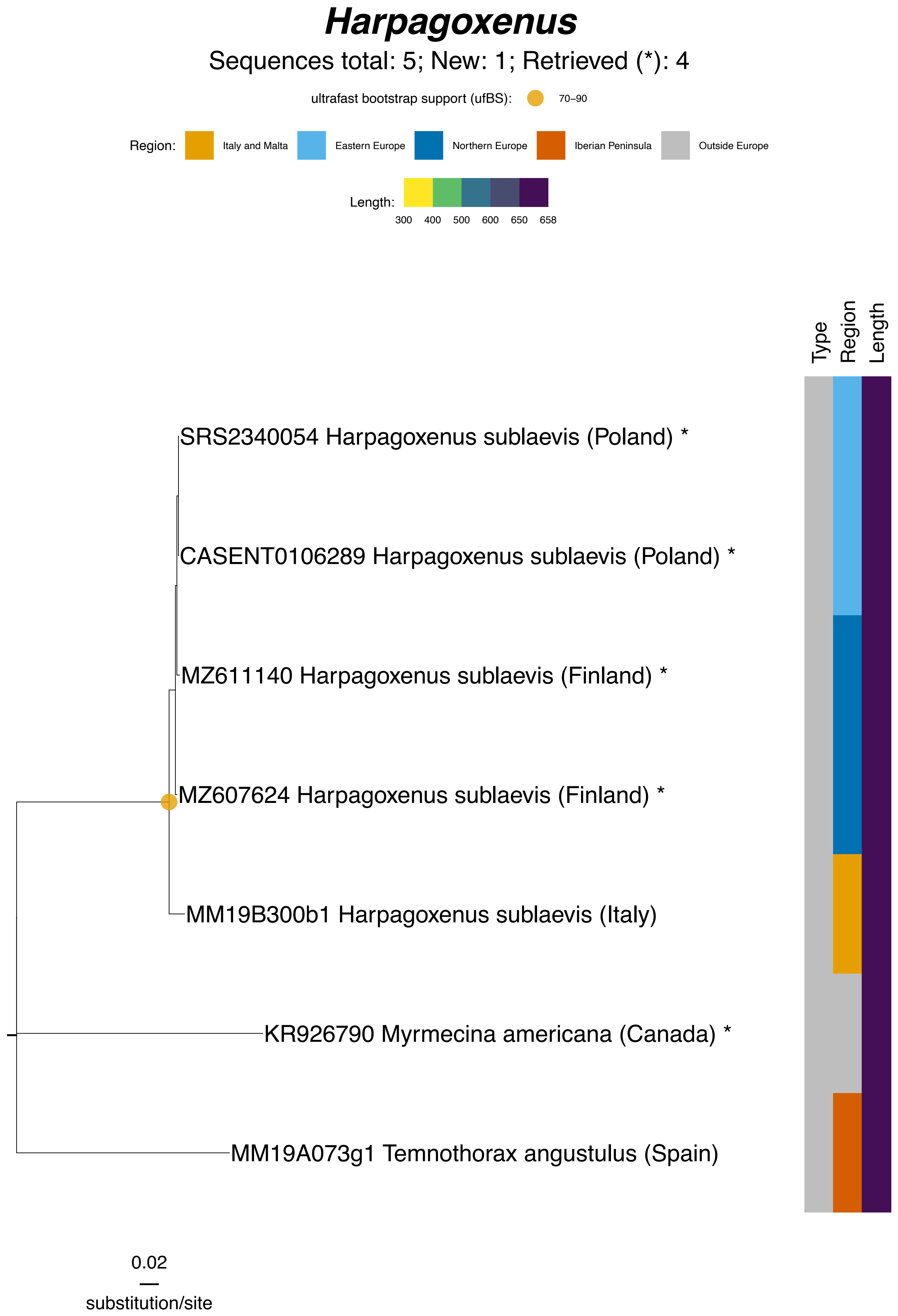

Harpagoxenus
Sequences total: 5; New: 1; Retrieved (*): 4
ultrafast bootstrap support (ufBS):
70–90
Region:
Italy and Malta
Eastern Europe
Northern Europe
Iberian Peninsula
Outside Europe
Length:
300
400
500
600
650
658
Type
Region
Length
SRS2340054 Harpagoxenus sublaevis (Poland) *
CASENT0106289 Harpagoxenus sublaevis (Poland) *
MZ611140 Harpagoxenus sublaevis (Finland) *
MZ607624 Harpagoxenus sublaevis (Finland) *
MM19B300b1 Harpagoxenus sublaevis (Italy)
KR926790 Myrmecina americana (Canada) *
MM19A073g1 Temnothorax angustulus (Spain)
0.02
substitution/site

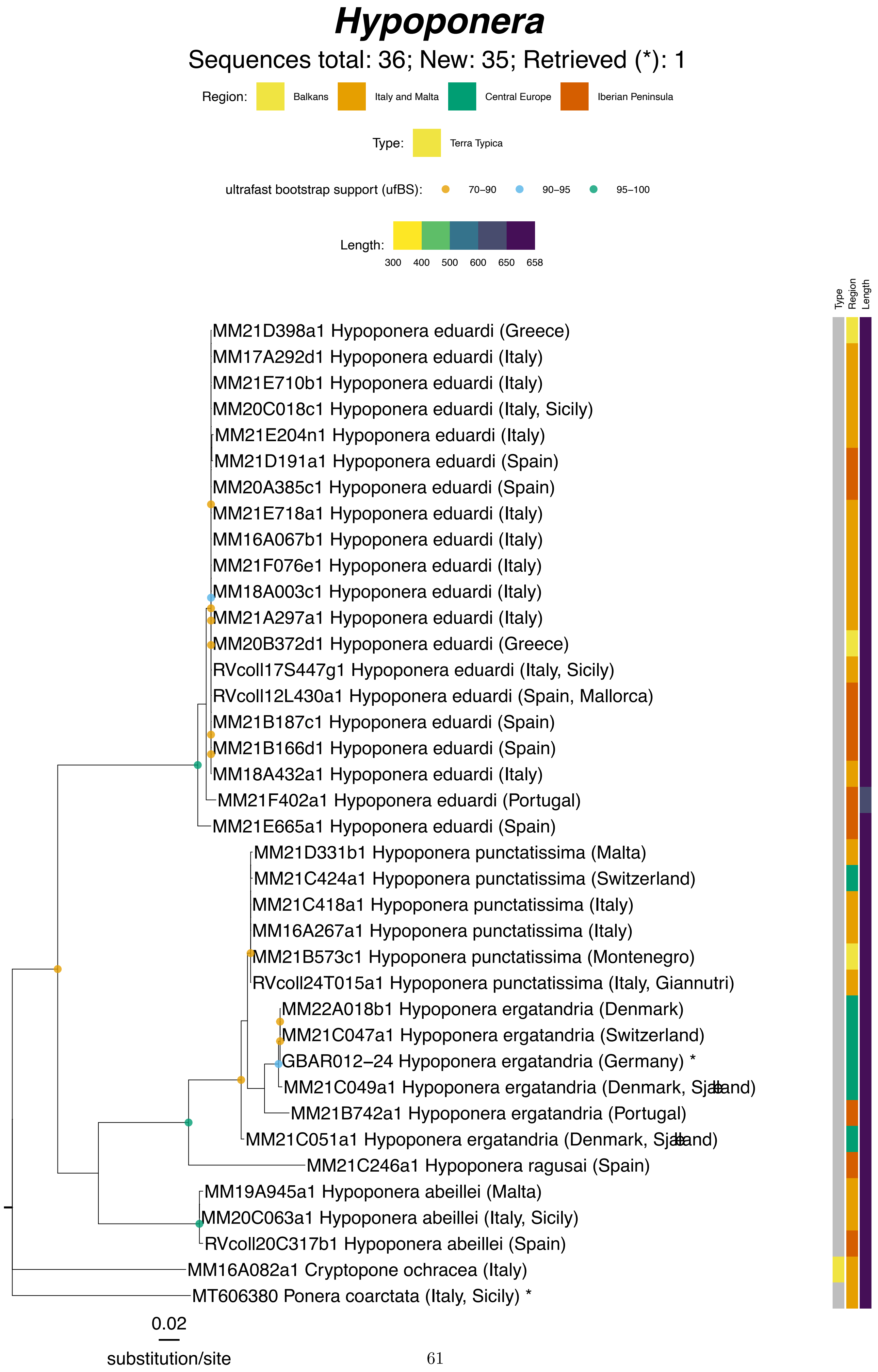

Hypoponera
Sequences total: 36; New: 35; Retrieved (*): 1
Region: Balkans Italy and Malta Central Europe Iberian Peninsula
Type: Terra Typica
ultrafast bootstrap support (ufBS): 70–90 90–95 95–100
Length: 300 400 500 600 650 658
Type Region Length
MM21D398a1 Hypoponera eduardi (Greece)
MM17A292d1 Hypoponera eduardi (Italy)
MM21E710b1 Hypoponera eduardi (Italy)
MM20C018c1 Hypoponera eduardi (Italy, Sicily)
MM21E204n1 Hypoponera eduardi (Italy)
MM21D191a1 Hypoponera eduardi (Spain)
MM20A385c1 Hypoponera eduardi (Spain)
MM21E718a1 Hypoponera eduardi (Italy)
MM16A067b1 Hypoponera eduardi (Italy)
MM21F076e1 Hypoponera eduardi (Italy)
MM18A003c1 Hypoponera eduardi (Italy)
MM21A297a1 Hypoponera eduardi (Italy)
MM20B372d1 Hypoponera eduardi (Greece)
RVcoll17S447g1 Hypoponera eduardi (Italy, Sicily)
RVcoll12L430a1 Hypoponera eduardi (Spain, Mallorca)
MM21B187c1 Hypoponera eduardi (Spain)
MM21B166d1 Hypoponera eduardi (Spain)
MM18A432a1 Hypoponera eduardi (Italy)
MM21F402a1 Hypoponera eduardi (Portugal)
MM21E665a1 Hypoponera eduardi (Spain)
MM21D331b1 Hypoponera punctatissima (Malta)
MM21C424a1 Hypoponera punctatissima (Switzerland)
MM21C418a1 Hypoponera punctatissima (Italy)
MM16A267a1 Hypoponera punctatissima (Italy)
MM21B573c1 Hypoponera punctatissima (Montenegro)
RVcoll24T015a1 Hypoponera punctatissima (Italy, Giannutri)
MM22A018b1 Hypoponera ergatandria (Denmark)
MM21C047a1 Hypoponera ergatandria (Switzerland)
GBAR012–24 Hypoponera ergatandria (Germany) *
MM21C049a1 Hypoponera ergatandria (Denmark, Sjælland)
MM21B742a1 Hypoponera ergatandria (Portugal)
MM21C051a1 Hypoponera ergatandria (Denmark, Sjælland)
MM21C246a1 Hypoponera ragusai (Spain)
MM19A945a1 Hypoponera abeillei (Malta)
MM20C063a1 Hypoponera abeillei (Italy, Sicily)
RVcoll20C317b1 Hypoponera abeillei (Spain)
MM16A082a1 Cryptopone ochracea (Italy)
MT606380 Ponera coarctata (Italy, Sicily) *
0.02
substitution/site

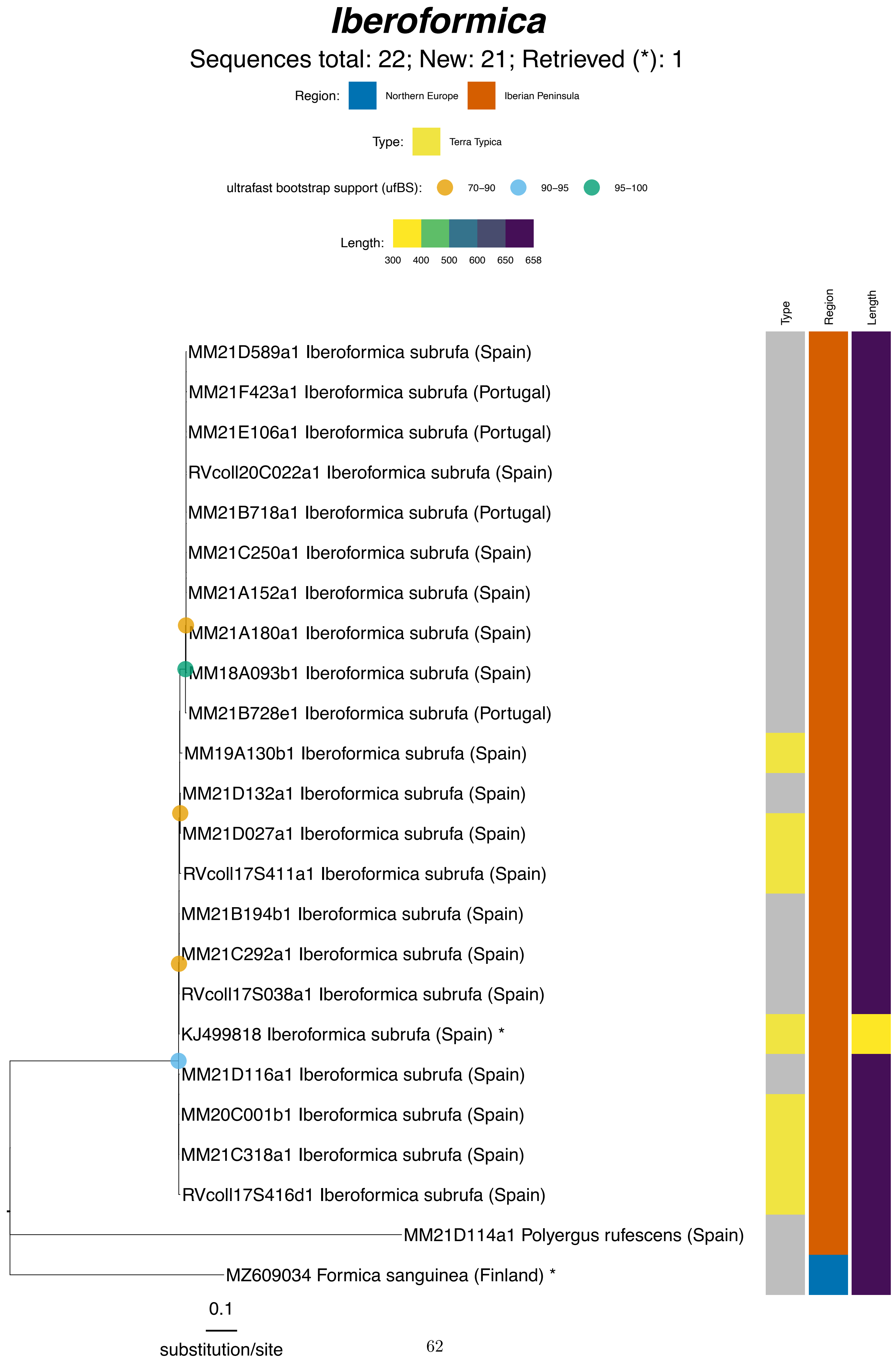
Iberoformica
Sequences total: 22; New: 21; Retrieved (*): 1
Region:
Northern Europe
Iberian Peninsula
Type:
Terra Typica
ultrafast bootstrap support (ufBS):
70−90
90−95
95−100
Length:
300
400
500
600
650
658
Type
Region
Length
MM21D589a1 Iberoformica subrufa (Spain)
MM21F423a1 Iberoformica subrufa (Portugal)
MM21E106a1 Iberoformica subrufa (Portugal)
RVcoll20C022a1 Iberoformica subrufa (Spain)
MM21B718a1 Iberoformica subrufa (Portugal)
MM21C250a1 Iberoformica subrufa (Spain)
MM21A152a1 Iberoformica subrufa (Spain)
MM21A180a1 Iberoformica subrufa (Spain)
MM18A093b1 Iberoformica subrufa (Spain)
MM21B728e1 Iberoformica subrufa (Portugal)
MM19A130b1 Iberoformica subrufa (Spain)
MM21D132a1 Iberoformica subrufa (Spain)
MM21D027a1 Iberoformica subrufa (Spain)
RVcoll17S411a1 Iberoformica subrufa (Spain)
MM21B194b1 Iberoformica subrufa (Spain)
MM21C292a1 Iberoformica subrufa (Spain)
RVcoll17S038a1 Iberoformica subrufa (Spain)
KJ499818 Iberoformica subrufa (Spain) *
MM21D116a1 Iberoformica subrufa (Spain)
MM20C001b1 Iberoformica subrufa (Spain)
MM21C318a1 Iberoformica subrufa (Spain)
RVcoll17S416d1 Iberoformica subrufa (Spain)
MM21D114a1 Polyergus rufescens (Spain)
MZ609034 Formica sanguinea (Finland) *
0.1
substitution/site

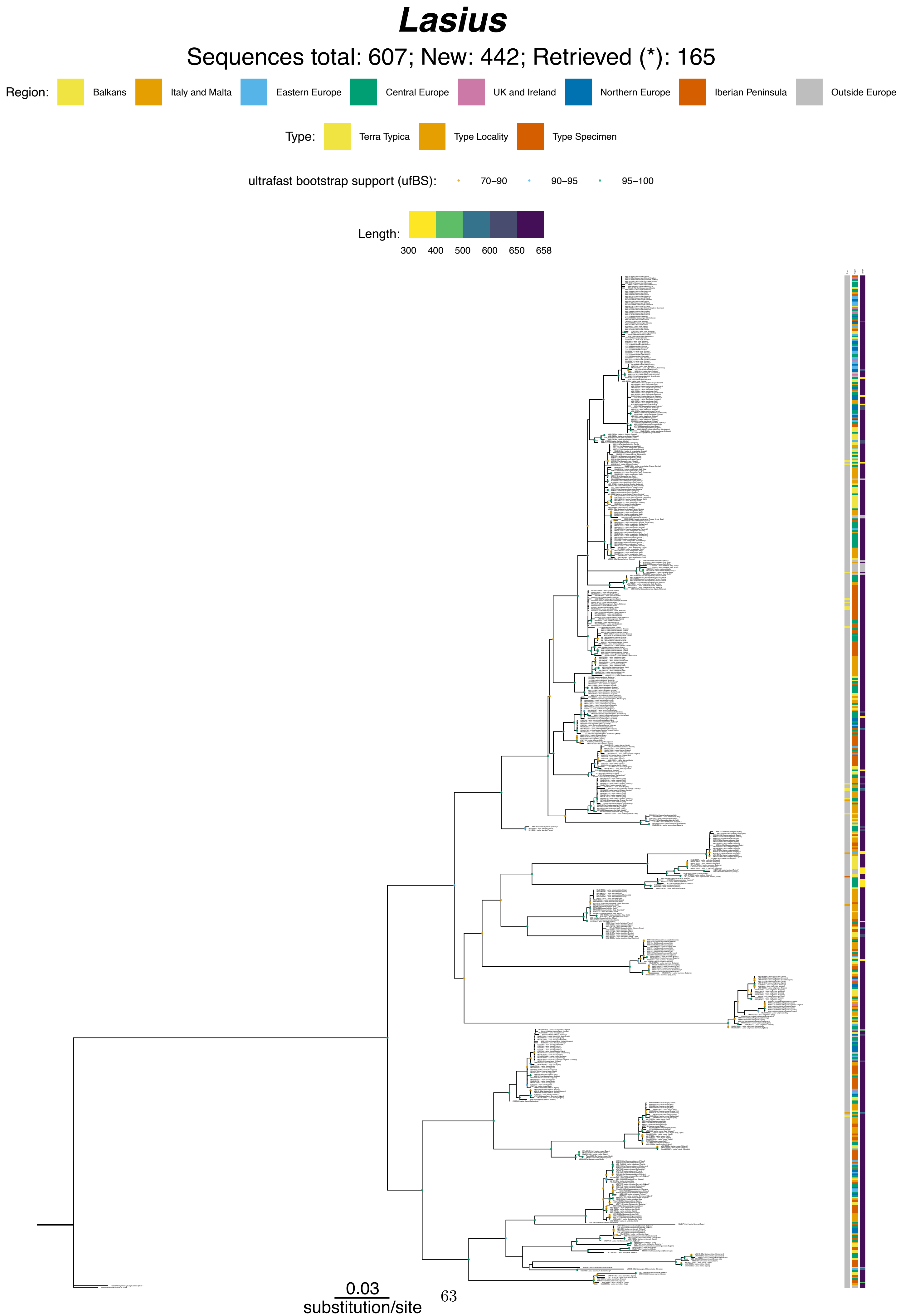

Lasius
Sequences total: 607; New: 442; Retrieved (*): 165
Region: Balkans Italy and Malta Eastern Europe Central Europe UK and Ireland Northern Europe Iberian Peninsula Outside Europe
Type: Terra Typica Type Locality Type Specimen
ultrafast bootstrap support (ufBS): 70–90 90–95 95–100
Length: 300 400 500 600 650 658
0.03
substitution/site

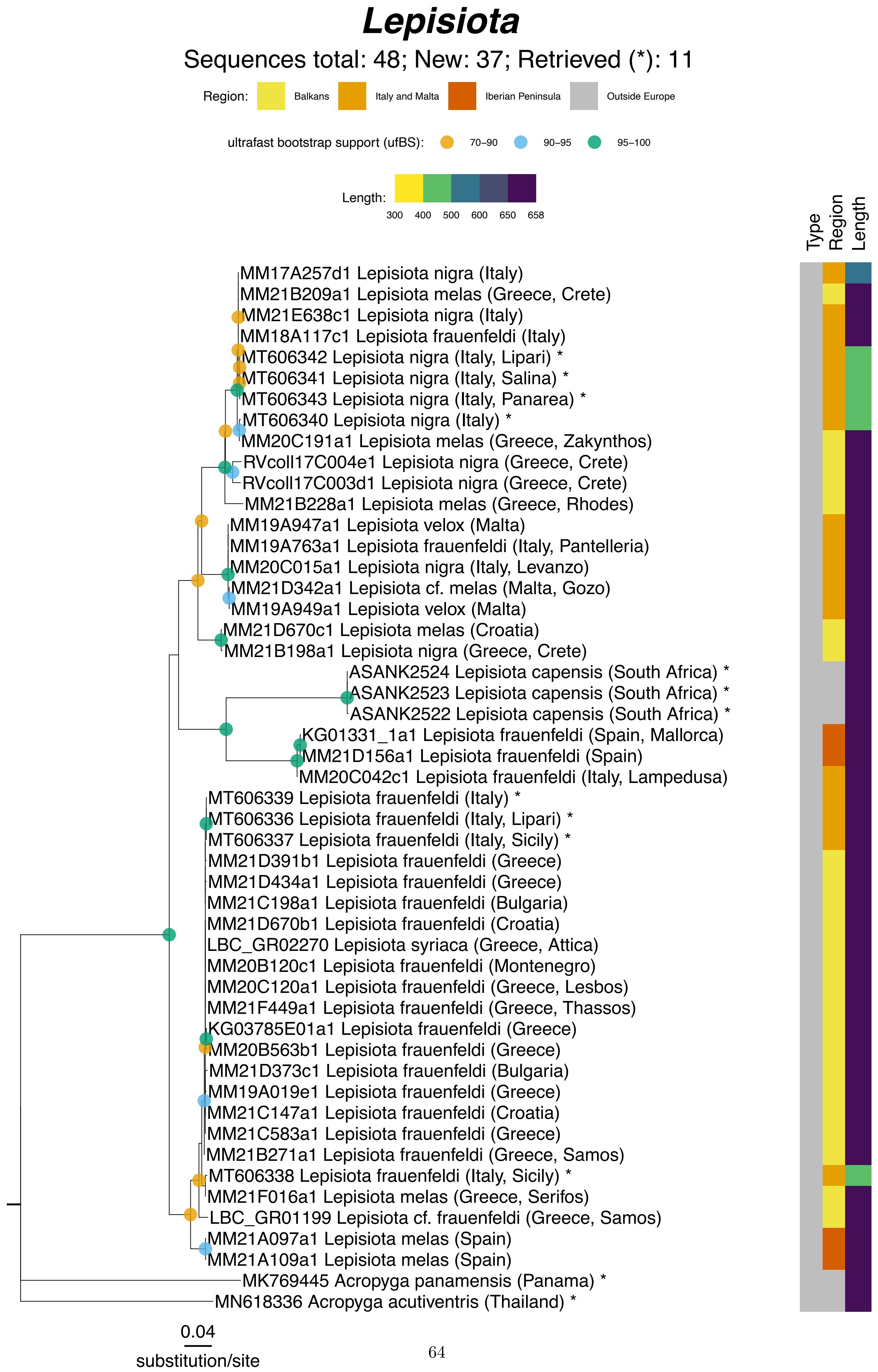

Lepisiota
Sequences total: 48; New: 37; Retrieved (*): 11
Region:
Balkans
Italy and Malta
Iberian Peninsula
Outside Europe
ultrafast bootstrap support (ufBS):
70–90
90–95
95–100
Length:
300
400
500
600
650
658
Type
Region
Length
MM17A257d1 Lepisiota nigra (Italy)
MM21B209a1 Lepisiota melas (Greece, Crete)
MM21E638c1 Lepisiota nigra (Italy)
MM18A117c1 Lepisiota frauenfeldi (Italy)
MT606342 Lepisiota nigra (Italy, Lipari) *
MT606341 Lepisiota nigra (Italy, Salina) *
MT606343 Lepisiota nigra (Italy, Panarea) *
MT606340 Lepisiota nigra (Italy) *
MM20C191a1 Lepisiota melas (Greece, Zakynthos)
RVcoll17C004e1 Lepisiota nigra (Greece, Crete)
RVcoll17C003d1 Lepisiota nigra (Greece, Crete)
MM21B228a1 Lepisiota melas (Greece, Rhodes)
MM19A947a1 Lepisiota velox (Malta)
MM19A763a1 Lepisiota frauenfeldi (Italy, Pantelleria)
MM20C015a1 Lepisiota nigra (Italy, Levanzo)
MM21D342a1 Lepisiota cf. melas (Malta, Gozo)
MM19A949a1 Lepisiota velox (Malta)
MM21D670c1 Lepisiota melas (Croatia)
MM21B198a1 Lepisiota nigra (Greece, Crete)
ASANK2524 Lepisiota capensis (South Africa) *
ASANK2523 Lepisiota capensis (South Africa) *
ASANK2522 Lepisiota capensis (South Africa) *
KG01331_1a1 Lepisiota frauenfeldi (Spain, Mallorca)
MM21D156a1 Lepisiota frauenfeldi (Spain)
MM20C042c1 Lepisiota frauenfeldi (Italy, Lampedusa)
MT606339 Lepisiota frauenfeldi (Italy) *
MT606336 Lepisiota frauenfeldi (Italy, Lipari) *
MT606337 Lepisiota frauenfeldi (Italy, Sicily) *
MM21D391b1 Lepisiota frauenfeldi (Greece)
MM21D434a1 Lepisiota frauenfeldi (Greece)
MM21C198a1 Lepisiota frauenfeldi (Bulgaria)
MM21D670b1 Lepisiota frauenfeldi (Croatia)
LBC_GR02270 Lepisiota syriaca (Greece, Attica)
MM20B120c1 Lepisiota frauenfeldi (Montenegro)
MM20C120a1 Lepisiota frauenfeldi (Greece, Lesbos)
MM21F449a1 Lepisiota frauenfeldi (Greece, Thassos)
KG03785E01a1 Lepisiota frauenfeldi (Greece)
MM20B563b1 Lepisiota frauenfeldi (Greece)
MM21D373c1 Lepisiota frauenfeldi (Bulgaria)
MM19A019e1 Lepisiota frauenfeldi (Greece)
MM21C147a1 Lepisiota frauenfeldi (Croatia)
MM21C583a1 Lepisiota frauenfeldi (Greece)
MM21B271a1 Lepisiota frauenfeldi (Greece, Samos)
MT606338 Lepisiota frauenfeldi (Italy, Sicily) *
MM21F016a1 Lepisiota melas (Greece, Serifos)
LBC_GR01199 Lepisiota cf. frauenfeldi (Greece, Samos)
MM21A097a1 Lepisiota melas (Spain)
MM21A109a1 Lepisiota melas (Spain)
MK769445 Acropyga panamensis (Panama) *
MN618336 Acropyga acutiventris (Thailand) *
0.04
substitution/site

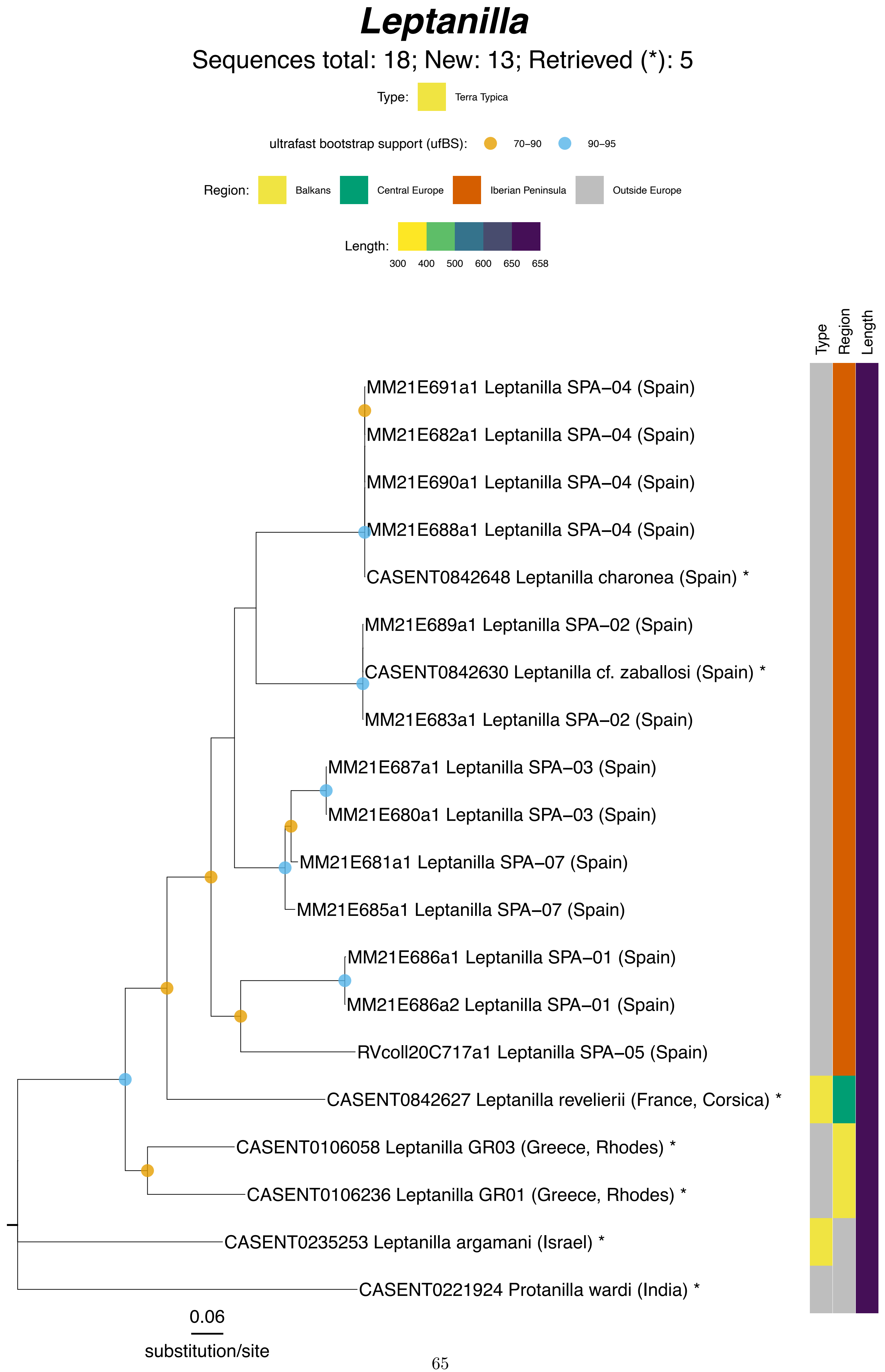
Leptanilla
Sequences total: 18; New: 13; Retrieved (*): 5
Type:
Terra Typica
ultrafast bootstrap support (ufBS):
70–90
90–95
Region:
Balkans
Central Europe
Iberian Peninsula
Outside Europe
Length:
300
400
500
600
650
658
Type
Region
Length
MM21E691a1 Leptanilla SPA–04 (Spain)
MM21E682a1 Leptanilla SPA–04 (Spain)
MM21E690a1 Leptanilla SPA–04 (Spain)
MM21E688a1 Leptanilla SPA–04 (Spain)
CASENT0842648 Leptanilla charonea (Spain) *
MM21E689a1 Leptanilla SPA–02 (Spain)
CASENT0842630 Leptanilla cf. zaballosi (Spain) *
MM21E683a1 Leptanilla SPA–02 (Spain)
MM21E687a1 Leptanilla SPA–03 (Spain)
MM21E680a1 Leptanilla SPA–03 (Spain)
MM21E681a1 Leptanilla SPA–07 (Spain)
MM21E685a1 Leptanilla SPA–07 (Spain)
MM21E686a1 Leptanilla SPA–01 (Spain)
MM21E686a2 Leptanilla SPA–01 (Spain)
RVcoll20C717a1 Leptanilla SPA–05 (Spain)
CASENT0842627 Leptanilla revelierii (France, Corsica) *
CASENT0106058 Leptanilla GR03 (Greece, Rhodes) *
CASENT0106236 Leptanilla GR01 (Greece, Rhodes) *
CASENT0235253 Leptanilla argamani (Israel) *
CASENT0221924 Protanilla wardi (India) *
0.06
substitution/site

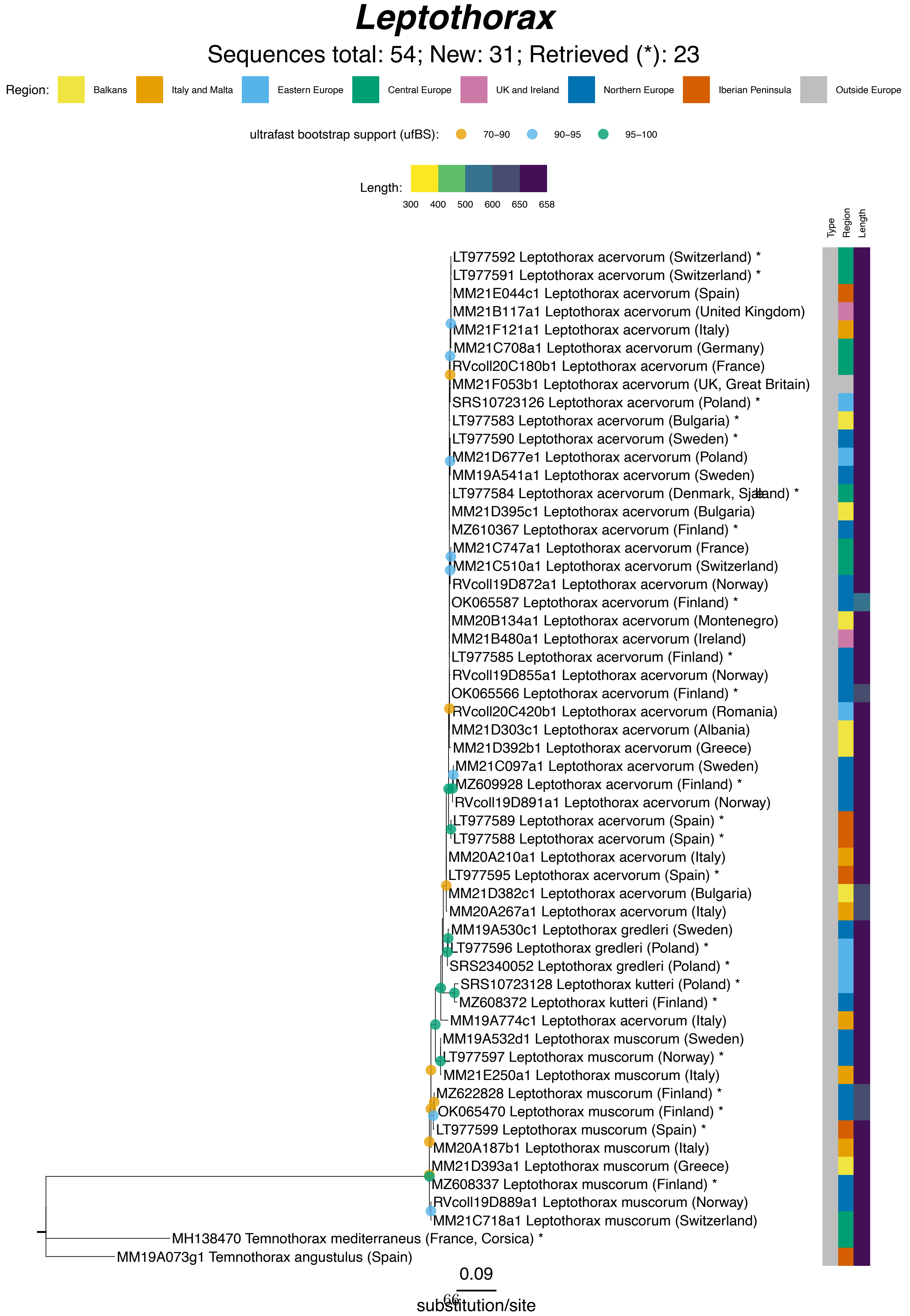
Leptothorax
Sequences total: 54; New: 31; Retrieved (*): 23
Region:
Balkans
Italy and Malta
Eastern Europe
Central Europe
UK and Ireland
Northern Europe
Iberian Peninsula
Outside Europe
ultrafast bootstrap support (ufBS):
70–90
90–95
95–100
Length:
300
400
500
600
650
658
Type
Region
Length
LT977592 Leptothorax acervorum (Switzerland) *
LT977591 Leptothorax acervorum (Switzerland) *
MM21E044c1 Leptothorax acervorum (Spain)
MM21B117a1 Leptothorax acervorum (United Kingdom)
MM21F121a1 Leptothorax acervorum (Italy)
MM21C708a1 Leptothorax acervorum (Germany)
RVcoll20C180b1 Leptothorax acervorum (France)
MM21F053b1 Leptothorax acervorum (UK, Great Britain)
SRS10723126 Leptothorax acervorum (Poland) *
LT977583 Leptothorax acervorum (Bulgaria) *
LT977590 Leptothorax acervorum (Sweden) *
MM21D677e1 Leptothorax acervorum (Poland)
MM19A541a1 Leptothorax acervorum (Sweden)
LT977584 Leptothorax acervorum (Denmark, Sjælland) *
MM21D395c1 Leptothorax acervorum (Bulgaria)
MZ610367 Leptothorax acervorum (Finland) *
MM21C747a1 Leptothorax acervorum (France)
MM21C510a1 Leptothorax acervorum (Switzerland)
RVcoll19D872a1 Leptothorax acervorum (Norway)
OK065587 Leptothorax acervorum (Finland) *
MM20B134a1 Leptothorax acervorum (Montenegro)
MM21B480a1 Leptothorax acervorum (Ireland)
LT977585 Leptothorax acervorum (Finland) *
RVcoll19D855a1 Leptothorax acervorum (Norway)
OK065566 Leptothorax acervorum (Finland) *
RVcoll20C420b1 Leptothorax acervorum (Romania)
MM21D303c1 Leptothorax acervorum (Albania)
MM21D392b1 Leptothorax acervorum (Greece)
MM21C097a1 Leptothorax acervorum (Sweden)
MZ609928 Leptothorax acervorum (Finland) *
RVcoll19D891a1 Leptothorax acervorum (Norway)
LT977589 Leptothorax acervorum (Spain) *
LT977588 Leptothorax acervorum (Spain) *
MM20A210a1 Leptothorax acervorum (Italy)
LT977595 Leptothorax acervorum (Spain) *
MM21D382c1 Leptothorax acervorum (Bulgaria)
MM20A267a1 Leptothorax acervorum (Italy)
MM19A530c1 Leptothorax gredleri (Sweden)
LT977596 Leptothorax gredleri (Poland) *
SRS2340052 Leptothorax gredleri (Poland) *
SRS10723128 Leptothorax kutteri (Poland) *
MZ608372 Leptothorax kutteri (Finland) *
MM19A774c1 Leptothorax acervorum (Italy)
MM19A532d1 Leptothorax muscorum (Sweden)
LT977597 Leptothorax muscorum (Norway) *
MM21E250a1 Leptothorax muscorum (Italy)
MZ622828 Leptothorax muscorum (Finland) *
OK065470 Leptothorax muscorum (Finland) *
LT977599 Leptothorax muscorum (Spain) *
MM20A187b1 Leptothorax muscorum (Italy)
MM21D393a1 Leptothorax muscorum (Greece)
MZ608337 Leptothorax muscorum (Finland) *
RVcoll19D889a1 Leptothorax muscorum (Norway)
MM21C718a1 Leptothorax muscorum (Switzerland)
MH138470 Temnothorax mediterraneus (France, Corsica) *
MM19A073g1 Temnothorax angustulus (Spain)
0.09
substitution/site

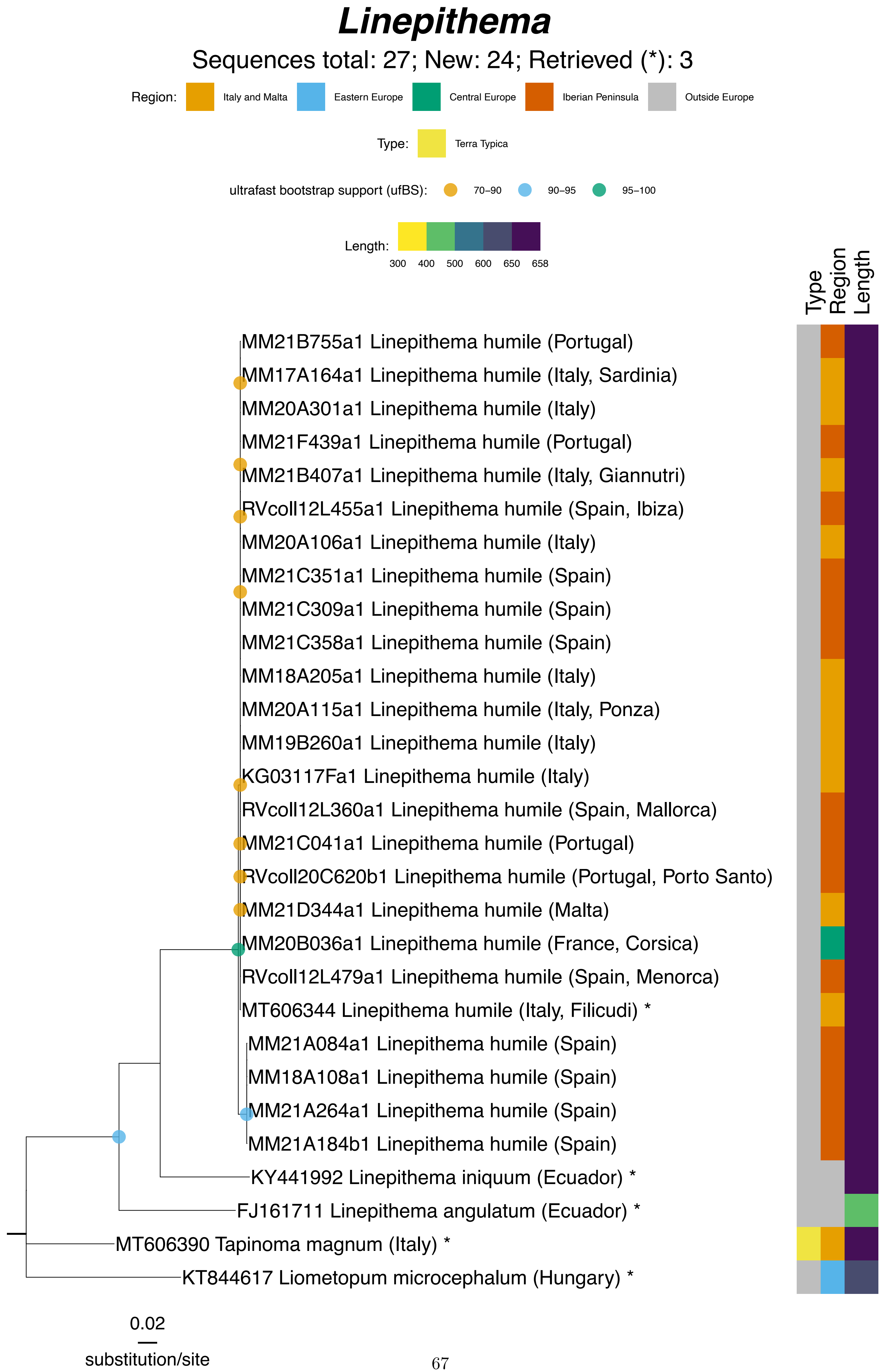
Linepithema
Sequences total: 27; New: 24; Retrieved (*): 3
Region:
Italy and Malta
Eastern Europe
Central Europe
Iberian Peninsula
Outside Europe
Type:
Terra Typica
ultrafast bootstrap support (ufBS):
70–90
90–95
95–100
Length:
300
400
500
600
650
658
Type
Region
Length
MM21B755a1 Linepithema humile (Portugal)
MM17A164a1 Linepithema humile (Italy, Sardinia)
MM20A301a1 Linepithema humile (Italy)
MM21F439a1 Linepithema humile (Portugal)
MM21B407a1 Linepithema humile (Italy, Giannutri)
RVcoll12L455a1 Linepithema humile (Spain, Ibiza)
MM20A106a1 Linepithema humile (Italy)
MM21C351a1 Linepithema humile (Spain)
MM21C309a1 Linepithema humile (Spain)
MM21C358a1 Linepithema humile (Spain)
MM18A205a1 Linepithema humile (Italy)
MM20A115a1 Linepithema humile (Italy, Ponza)
MM19B260a1 Linepithema humile (Italy)
KG03117Fa1 Linepithema humile (Italy)
RVcoll12L360a1 Linepithema humile (Spain, Mallorca)
MM21C041a1 Linepithema humile (Portugal)
RVcoll20C620b1 Linepithema humile (Portugal, Porto Santo)
MM21D344a1 Linepithema humile (Malta)
MM20B036a1 Linepithema humile (France, Corsica)
RVcoll12L479a1 Linepithema humile (Spain, Menorca)
MT606344 Linepithema humile (Italy, Filicudi) *
MM21A084a1 Linepithema humile (Spain)
MM18A108a1 Linepithema humile (Spain)
MM21A264a1 Linepithema humile (Spain)
MM21A184b1 Linepithema humile (Spain)
KY441992 Linepithema iniquum (Ecuador) *
FJ161711 Linepithema angulatum (Ecuador) *
MT606390 Tapinoma magnum (Italy) *
KT844617 Liometopum microcephalum (Hungary) *
0.02
substitution/site

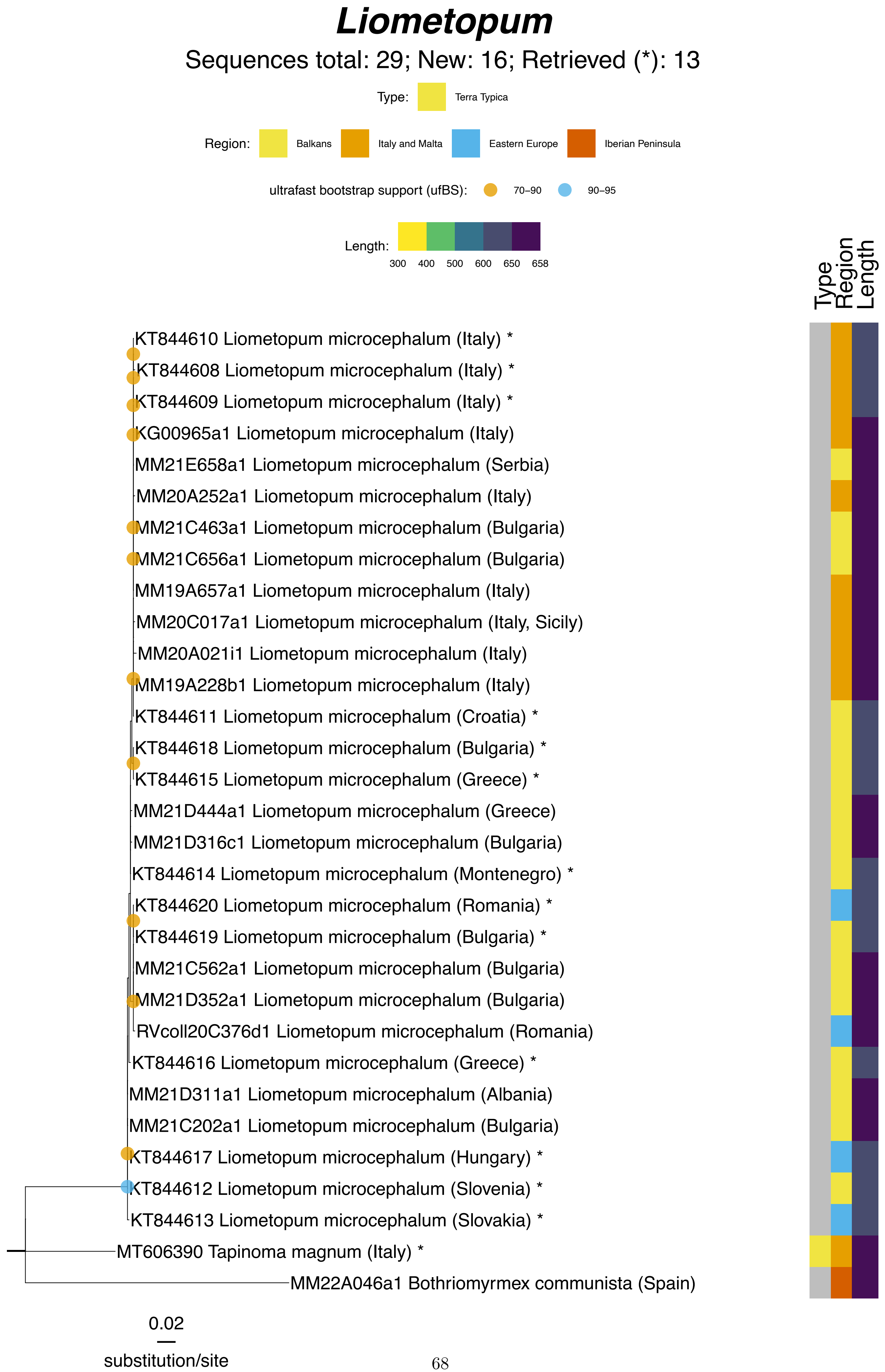

Liometopum
Sequences total: 29; New: 16; Retrieved (*): 13
Type:
Terra Typica
Region:
Balkans
Italy and Malta
Eastern Europe
Iberian Peninsula
ultrafast bootstrap support (ufBS):
70–90
90–95
Length:
300
400
500
600
650
658
Type
Region
Length
KT844610 Liometopum microcephalum (Italy) *
KT844608 Liometopum microcephalum (Italy) *
KT844609 Liometopum microcephalum (Italy) *
KG00965a1 Liometopum microcephalum (Italy)
MM21E658a1 Liometopum microcephalum (Serbia)
MM20A252a1 Liometopum microcephalum (Italy)
MM21C463a1 Liometopum microcephalum (Bulgaria)
MM21C656a1 Liometopum microcephalum (Bulgaria)
MM19A657a1 Liometopum microcephalum (Italy)
MM20C017a1 Liometopum microcephalum (Italy, Sicily)
MM20A021i1 Liometopum microcephalum (Italy)
MM19A228b1 Liometopum microcephalum (Italy)
KT844611 Liometopum microcephalum (Croatia) *
KT844618 Liometopum microcephalum (Bulgaria) *
KT844615 Liometopum microcephalum (Greece) *
MM21D444a1 Liometopum microcephalum (Greece)
MM21D316c1 Liometopum microcephalum (Bulgaria)
KT844614 Liometopum microcephalum (Montenegro) *
KT844620 Liometopum microcephalum (Romania) *
KT844619 Liometopum microcephalum (Bulgaria) *
MM21C562a1 Liometopum microcephalum (Bulgaria)
MM21D352a1 Liometopum microcephalum (Bulgaria)
RVcoll20C376d1 Liometopum microcephalum (Romania)
KT844616 Liometopum microcephalum (Greece) *
MM21D311a1 Liometopum microcephalum (Albania)
MM21C202a1 Liometopum microcephalum (Bulgaria)
KT844617 Liometopum microcephalum (Hungary) *
KT844612 Liometopum microcephalum (Slovenia) *
KT844613 Liometopum microcephalum (Slovakia) *
MT606390 Tapinoma magnum (Italy) *
MM22A046a1 Bothriomyrmex communista (Spain)
0.02
substitution/site

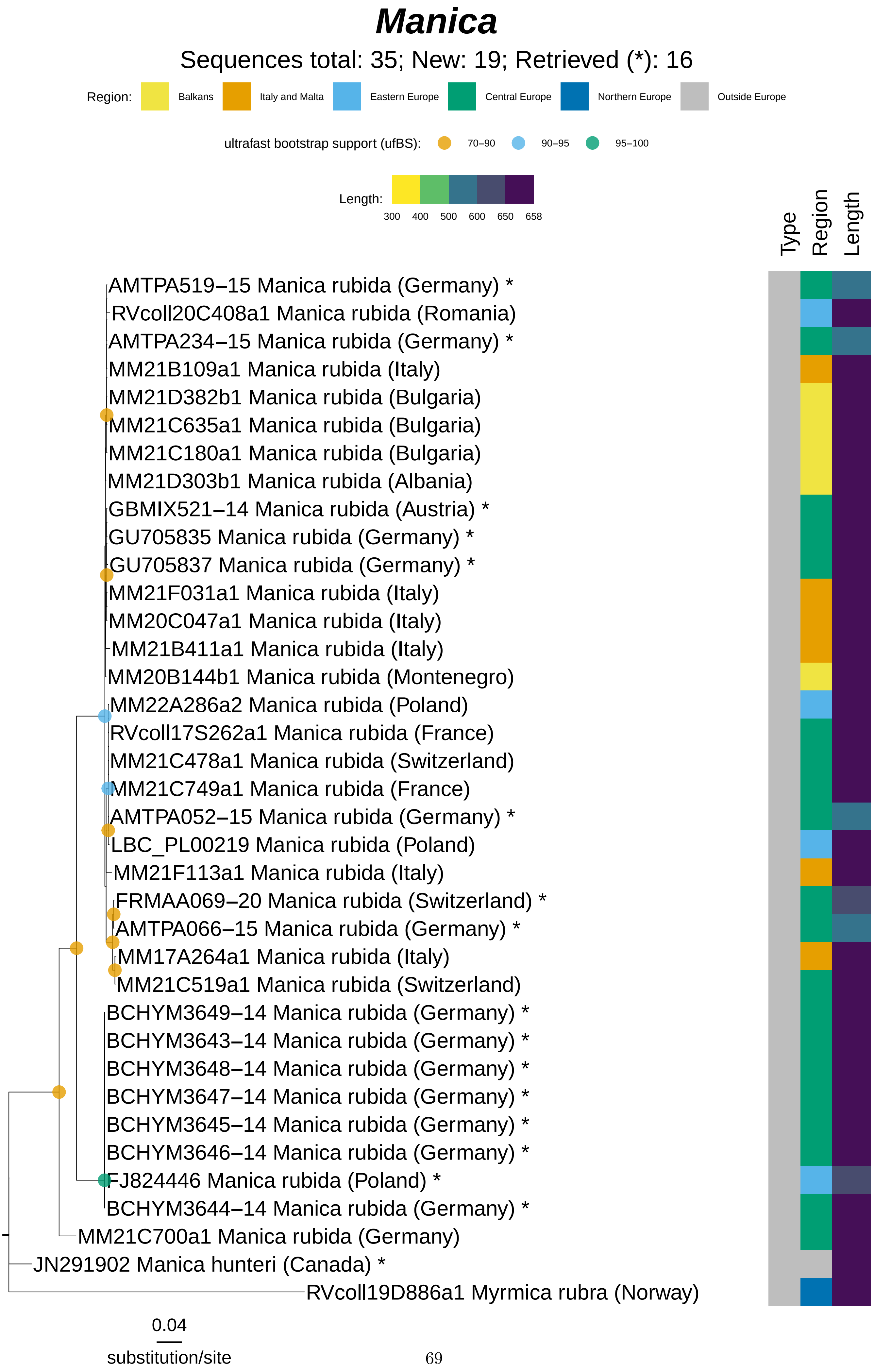
Manica
Sequences total: 35; New: 19; Retrieved (*): 16
Region:
Balkans
Italy and Malta
Eastern Europe
Central Europe
Northern Europe
Outside Europe
ultrafast bootstrap support (ufBS):
70–90
90–95
95–100
Length:
300
400
500
600
650
658
Type
Region
Length
AMTPA519–15 Manica rubida (Germany) *
RVcoll20C408a1 Manica rubida (Romania)
AMTPA234–15 Manica rubida (Germany) *
MM21B109a1 Manica rubida (Italy)
MM21D382b1 Manica rubida (Bulgaria)
MM21C635a1 Manica rubida (Bulgaria)
MM21C180a1 Manica rubida (Bulgaria)
MM21D303b1 Manica rubida (Albania)
GBMIX521–14 Manica rubida (Austria) *
GU705835 Manica rubida (Germany) *
GU705837 Manica rubida (Germany) *
MM21F031a1 Manica rubida (Italy)
MM20C047a1 Manica rubida (Italy)
MM21B411a1 Manica rubida (Italy)
MM20B144b1 Manica rubida (Montenegro)
MM22A286a2 Manica rubida (Poland)
RVcoll17S262a1 Manica rubida (France)
MM21C478a1 Manica rubida (Switzerland)
MM21C749a1 Manica rubida (France)
AMTPA052–15 Manica rubida (Germany) *
LBC_PL00219 Manica rubida (Poland)
MM21F113a1 Manica rubida (Italy)
FRMAA069–20 Manica rubida (Switzerland) *
AMTPA066–15 Manica rubida (Germany) *
MM17A264a1 Manica rubida (Italy)
MM21C519a1 Manica rubida (Switzerland)
BCHYM3649–14 Manica rubida (Germany) *
BCHYM3643–14 Manica rubida (Germany) *
BCHYM3648–14 Manica rubida (Germany) *
BCHYM3647–14 Manica rubida (Germany) *
BCHYM3645–14 Manica rubida (Germany) *
BCHYM3646–14 Manica rubida (Germany) *
FJ824446 Manica rubida (Poland) *
BCHYM3644–14 Manica rubida (Germany) *
MM21C700a1 Manica rubida (Germany)
JN291902 Manica hunteri (Canada) *
RVcoll19D886a1 Myrmica rubra (Norway)
0.04
substitution/site

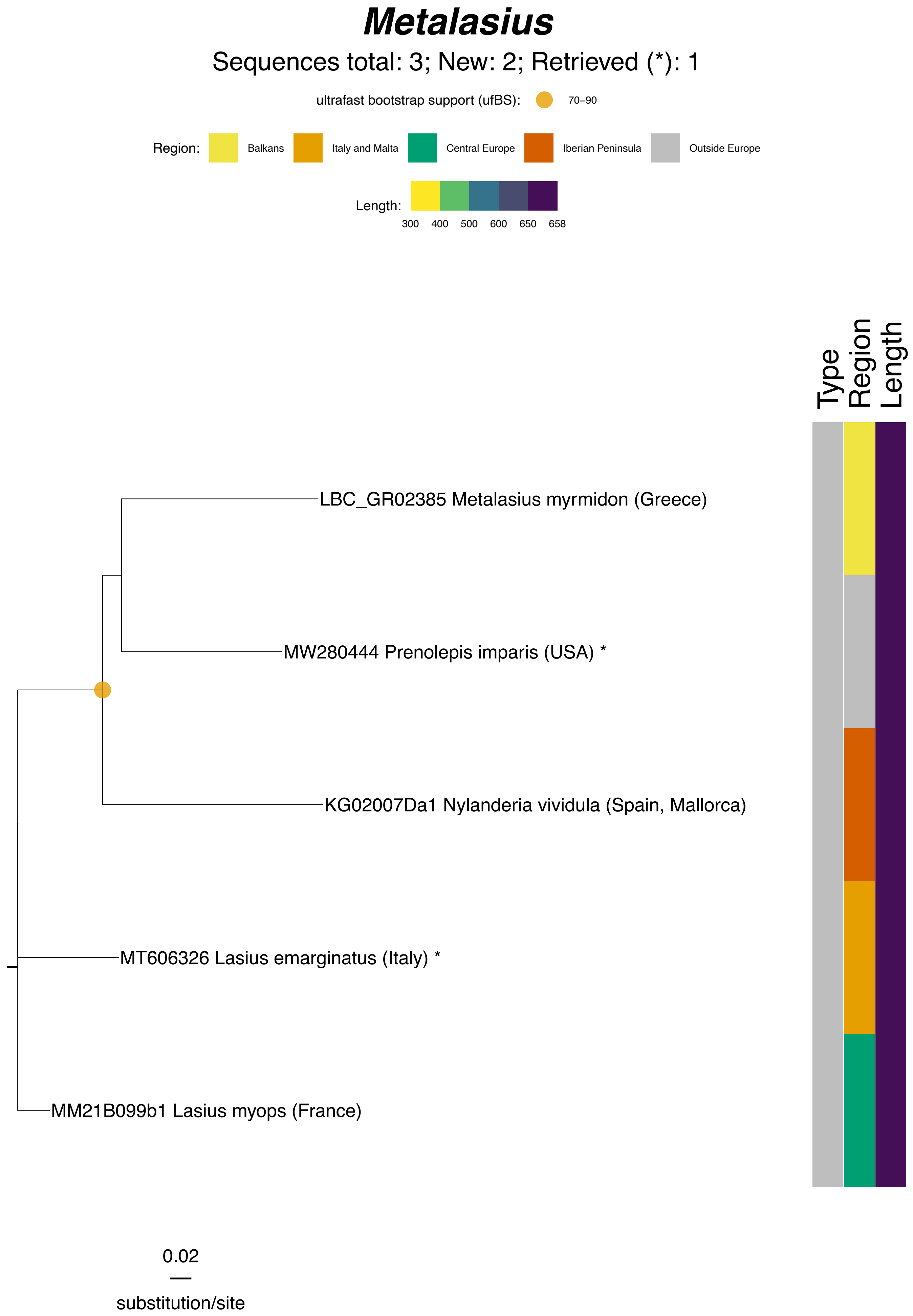
Metalasius
Sequences total: 3; New: 2; Retrieved (*): 1
ultrafast bootstrap support (ufBS):
70–90
Region:
Balkans
Italy and Malta
Central Europe
Iberian Peninsula
Outside Europe
Length:
300
400
500
600
650
658
Type
Region
Length
LBC_GR02385 Metalasius myrmidon (Greece)
MW280444 Prenolepis imparis (USA) *
KG02007Da1 Nylanderia vividula (Spain, Mallorca)
MT606326 Lasius emarginatus (Italy) *
MM21B099b1 Lasius myops (France)
0.02
substitution/site

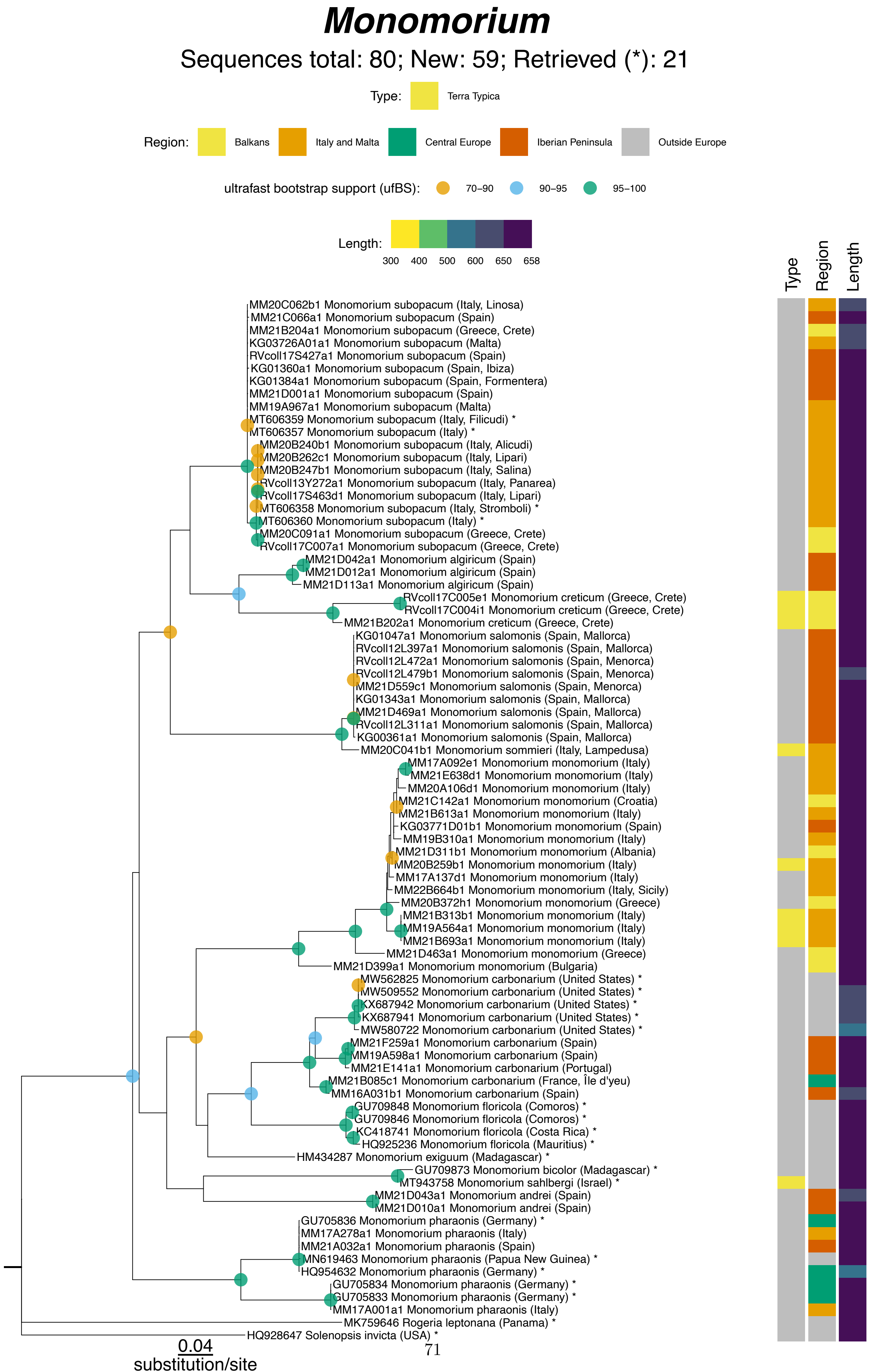
Monomorium
Sequences total: 80; New: 59; Retrieved (*): 21
Type:
Terra Typica
Region:
Balkans
Italy and Malta
Central Europe
Iberian Peninsula
Outside Europe
ultrafast bootstrap support (ufBS):
70–90
90–95
95–100
Length:
300
400
500
600
650
658
Type
Region
Length
MM20C062b1 Monomorium subopacum (Italy, Linosa)
MM21C066a1 Monomorium subopacum (Spain)
MM21B204a1 Monomorium subopacum (Greece, Crete)
KG03726A01a1 Monomorium subopacum (Malta)
RVcoll17S427a1 Monomorium subopacum (Spain)
KG01360a1 Monomorium subopacum (Spain, Ibiza)
KG01384a1 Monomorium subopacum (Spain, Formentera)
MM21D001a1 Monomorium subopacum (Spain)
MM19A967a1 Monomorium subopacum (Malta)
MT606359 Monomorium subopacum (Italy, Filicudi) *
MT606357 Monomorium subopacum (Italy) *
MM20B240b1 Monomorium subopacum (Italy, Alicudi)
MM20B262c1 Monomorium subopacum (Italy, Lipari)
MM20B247b1 Monomorium subopacum (Italy, Salina)
RVcoll13Y272a1 Monomorium subopacum (Italy, Panarea)
RVcoll17S463d1 Monomorium subopacum (Italy, Lipari)
MT606358 Monomorium subopacum (Italy, Stromboli) *
MT606360 Monomorium subopacum (Italy) *
MM20C091a1 Monomorium subopacum (Greece, Crete)
RVcoll17C007a1 Monomorium subopacum (Greece, Crete)
MM21D042a1 Monomorium algiricum (Spain)
MM21D012a1 Monomorium algiricum (Spain)
MM21D113a1 Monomorium algiricum (Spain)
RVcoll17C005e1 Monomorium creticum (Greece, Crete)
RVcoll17C004i1 Monomorium creticum (Greece, Crete)
MM21B202a1 Monomorium creticum (Greece, Crete)
KG01047a1 Monomorium salomonis (Spain, Mallorca)
RVcoll12L397a1 Monomorium salomonis (Spain, Mallorca)
RVcoll12L472a1 Monomorium salomonis (Spain, Menorca)
RVcoll12L479b1 Monomorium salomonis (Spain, Menorca)
MM21D559c1 Monomorium salomonis (Spain, Menorca)
KG01343a1 Monomorium salomonis (Spain, Mallorca)
MM21D469a1 Monomorium salomonis (Spain, Mallorca)
RVcoll12L311a1 Monomorium salomonis (Spain, Mallorca)
KG00361a1 Monomorium salomonis (Spain, Mallorca)
MM20C041b1 Monomorium sommieri (Italy, Lampedusa)
MM17A092e1 Monomorium monomorium (Italy)
MM21E638d1 Monomorium monomorium (Italy)
MM20A106d1 Monomorium monomorium (Italy)
MM21C142a1 Monomorium monomorium (Croatia)
MM21B613a1 Monomorium monomorium (Italy)
KG03771D01b1 Monomorium monomorium (Spain)
MM19B310a1 Monomorium monomorium (Italy)
MM21D311b1 Monomorium monomorium (Albania)
MM20B259b1 Monomorium monomorium (Italy)
MM17A137d1 Monomorium monomorium (Italy)
MM22B664b1 Monomorium monomorium (Italy, Sicily)
MM20B372h1 Monomorium monomorium (Greece)
MM21B313b1 Monomorium monomorium (Italy)
MM19A564a1 Monomorium monomorium (Italy)
MM21B693a1 Monomorium monomorium (Italy)
MM21D463a1 Monomorium monomorium (Greece)
MM21D399a1 Monomorium monomorium (Bulgaria)
MW562825 Monomorium carbonarium (United States) *
MW509552 Monomorium carbonarium (United States) *
KX687942 Monomorium carbonarium (United States) *
KX687941 Monomorium carbonarium (United States) *
MW580722 Monomorium carbonarium (United States) *
MM21F259a1 Monomorium carbonarium (Spain)
MM19A598a1 Monomorium carbonarium (Spain)
MM21E141a1 Monomorium carbonarium (Portugal)
MM21B085c1 Monomorium carbonarium (France, Île d'yeu)
MM16A031b1 Monomorium carbonarium (Spain)
GU709848 Monomorium floricola (Comoros) *
GU709846 Monomorium floricola (Comoros) *
KC418741 Monomorium floricola (Costa Rica) *
HQ925236 Monomorium floricola (Mauritius) *
HM434287 Monomorium exiguum (Madagascar) *
GU709873 Monomorium bicolor (Madagascar) *
MT943758 Monomorium sahlbergi (Israel) *
MM21D043a1 Monomorium andrei (Spain)
MM21D010a1 Monomorium andrei (Spain)
GU705836 Monomorium pharaonis (Germany) *
MM17A278a1 Monomorium pharaonis (Italy)
MM21A032a1 Monomorium pharaonis (Spain)
MN619463 Monomorium pharaonis (Papua New Guinea) *
HQ954632 Monomorium pharaonis (Germany) *
GU705834 Monomorium pharaonis (Germany) *
GU705833 Monomorium pharaonis (Germany) *
MM17A001a1 Monomorium pharaonis (Italy)
MK759646 Rogeria leptonana (Panama) *
HQ928647 Solenopsis invicta (USA) *
0.04
substitution/site

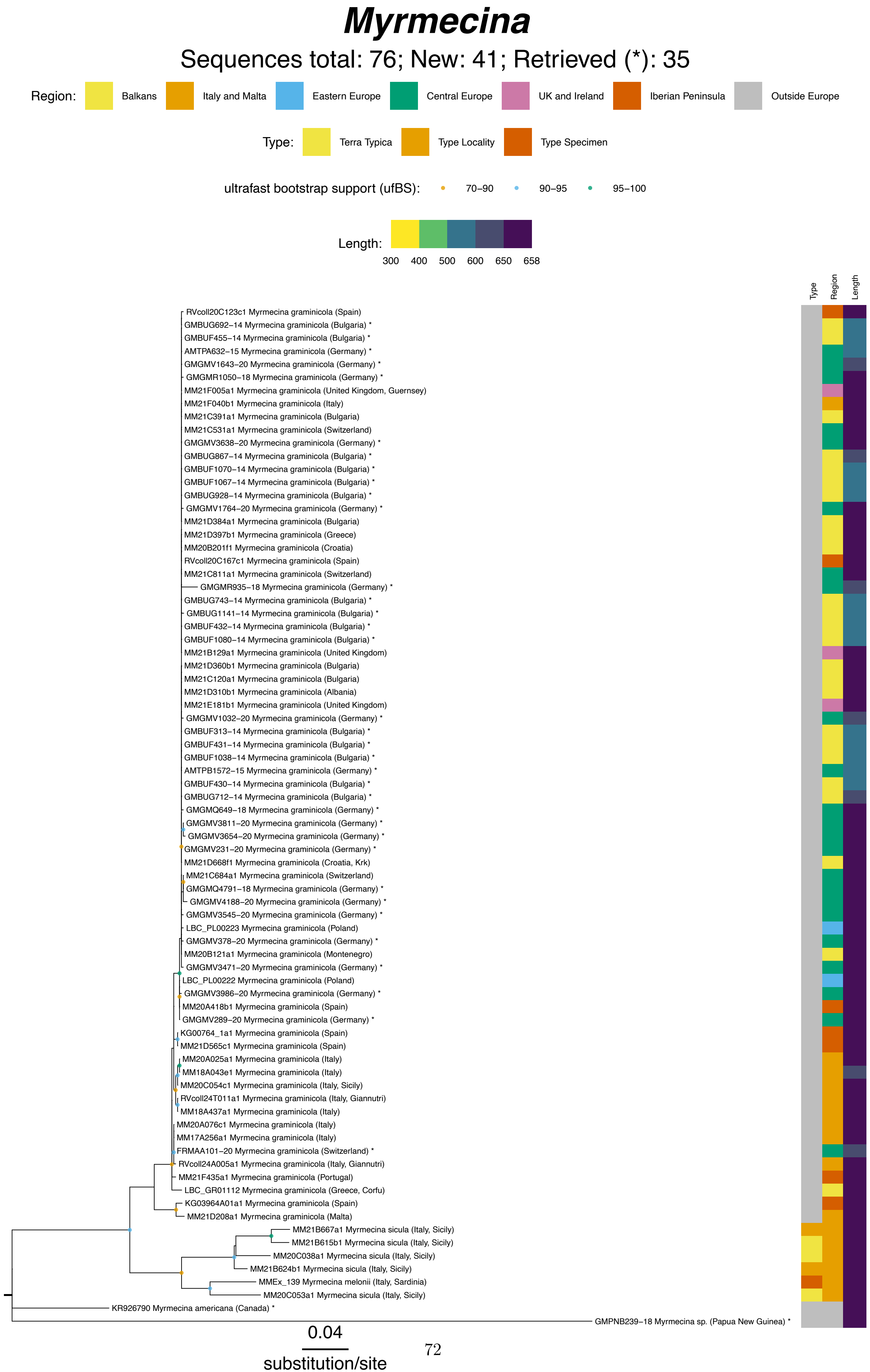
Myrmecina
Sequences total: 76; New: 41; Retrieved (*): 35
Region: Balkans, Italy and Malta, Eastern Europe, Central Europe, UK and Ireland, Iberian Peninsula, Outside Europe
Type: Terra Typica, Type Locality, Type Specimen
ultrafast bootstrap support (ufBS): 70–90, 90–95, 95–100
Length: 300 400 500 600 650 658
Type
Region
Length
RVcoll20C123c1 Myrmecina graminicola (Spain)
GMBUG692–14 Myrmecina graminicola (Bulgaria) *
GMBUF455–14 Myrmecina graminicola (Bulgaria) *
AMTPA632–15 Myrmecina graminicola (Germany) *
GMGMV1643–20 Myrmecina graminicola (Germany) *
GMGMR1050–18 Myrmecina graminicola (Germany) *
MM21F005a1 Myrmecina graminicola (United Kingdom, Guernsey)
MM21F040b1 Myrmecina graminicola (Italy)
MM21C391a1 Myrmecina graminicola (Bulgaria)
MM21C531a1 Myrmecina graminicola (Switzerland)
GMGMV3638–20 Myrmecina graminicola (Germany) *
GMBUG867–14 Myrmecina graminicola (Bulgaria) *
GMBUF1070–14 Myrmecina graminicola (Bulgaria) *
GMBUF1067–14 Myrmecina graminicola (Bulgaria) *
GMBUG928–14 Myrmecina graminicola (Bulgaria) *
GMGMV1764–20 Myrmecina graminicola (Germany) *
MM21D384a1 Myrmecina graminicola (Bulgaria)
MM21D397b1 Myrmecina graminicola (Greece)
MM20B201f1 Myrmecina graminicola (Croatia)
RVcoll20C167c1 Myrmecina graminicola (Spain)
MM21C811a1 Myrmecina graminicola (Switzerland)
GMGMR935–18 Myrmecina graminicola (Germany) *
GMBUG743–14 Myrmecina graminicola (Bulgaria) *
GMBUG1141–14 Myrmecina graminicola (Bulgaria) *
GMBUF432–14 Myrmecina graminicola (Bulgaria) *
GMBUF1080–14 Myrmecina graminicola (Bulgaria) *
MM21B129a1 Myrmecina graminicola (United Kingdom)
MM21D360b1 Myrmecina graminicola (Bulgaria)
MM21C120a1 Myrmecina graminicola (Bulgaria)
MM21D310b1 Myrmecina graminicola (Albania)
MM21E181b1 Myrmecina graminicola (United Kingdom)
GMGMV1032–20 Myrmecina graminicola (Germany) *
GMBUF313–14 Myrmecina graminicola (Bulgaria) *
GMBUF431–14 Myrmecina graminicola (Bulgaria) *
GMBUF1038–14 Myrmecina graminicola (Bulgaria) *
AMTPB1572–15 Myrmecina graminicola (Germany) *
GMBUF430–14 Myrmecina graminicola (Bulgaria) *
GMBUG712–14 Myrmecina graminicola (Bulgaria) *
GMGMQ649–18 Myrmecina graminicola (Germany) *
GMGMV3811–20 Myrmecina graminicola (Germany) *
GMGMV3654–20 Myrmecina graminicola (Germany) *
GMGMV231–20 Myrmecina graminicola (Germany) *
MM21D668f1 Myrmecina graminicola (Croatia, Krk)
MM21C684a1 Myrmecina graminicola (Switzerland)
GMGMQ4791–18 Myrmecina graminicola (Germany) *
GMGMV4188–20 Myrmecina graminicola (Germany) *
GMGMV3545–20 Myrmecina graminicola (Germany) *
LBC_PL00223 Myrmecina graminicola (Poland)
GMGMV378–20 Myrmecina graminicola (Germany) *
MM20B121a1 Myrmecina graminicola (Montenegro)
GMGMV3471–20 Myrmecina graminicola (Germany) *
LBC_PL00222 Myrmecina graminicola (Poland)
GMGMV3986–20 Myrmecina graminicola (Germany) *
MM20A418b1 Myrmecina graminicola (Spain)
GMGMV289–20 Myrmecina graminicola (Germany) *
KG00764_1a1 Myrmecina graminicola (Spain)
MM21D565c1 Myrmecina graminicola (Spain)
MM20A025a1 Myrmecina graminicola (Italy)
MM18A043e1 Myrmecina graminicola (Italy)
MM20C054c1 Myrmecina graminicola (Italy, Sicily)
RVcoll24T011a1 Myrmecina graminicola (Italy, Giannutri)
MM18A437a1 Myrmecina graminicola (Italy)
MM20A076c1 Myrmecina graminicola (Italy)
MM17A256a1 Myrmecina graminicola (Italy)
FRMAA101–20 Myrmecina graminicola (Switzerland) *
RVcoll24A005a1 Myrmecina graminicola (Italy, Giannutri)
MM21F435a1 Myrmecina graminicola (Portugal)
LBC_GR01112 Myrmecina graminicola (Greece, Corfu)
KG03964A01a1 Myrmecina graminicola (Spain)
MM21D208a1 Myrmecina graminicola (Malta)
MM21B667a1 Myrmecina sicula (Italy, Sicily)
MM21B615b1 Myrmecina sicula (Italy, Sicily)
MM20C038a1 Myrmecina sicula (Italy, Sicily)
MM21B624b1 Myrmecina sicula (Italy, Sicily)
MMEx_139 Myrmecina melonii (Italy, Sardinia)
MM20C053a1 Myrmecina sicula (Italy, Sicily)
KR926790 Myrmecina americana (Canada) *
GMPNB239–18 Myrmecina sp. (Papua New Guinea) *
0.04
substitution/site

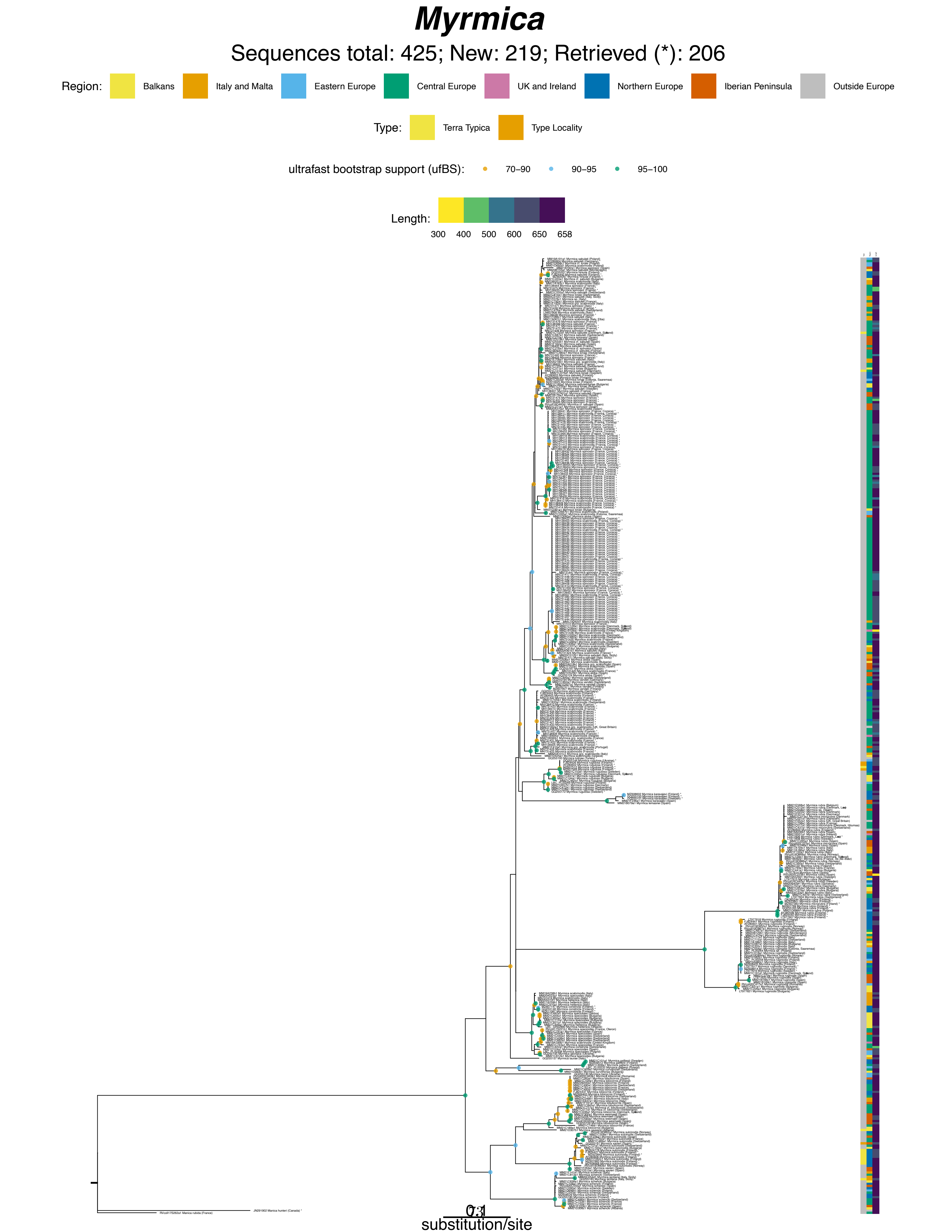
Myrmica
Sequences total: 425; New: 219; Retrieved (*): 206
Region:
Balkans
Italy and Malta
Eastern Europe
Central Europe
UK and Ireland
Northern Europe
Iberian Peninsula
Outside Europe
Type:
Terra Typica
Type Locality
ultrafast bootstrap support (ufBS):
70–90
90–95
95–100
Length:
300
400
500
600
650
658
0.1
substitution/site

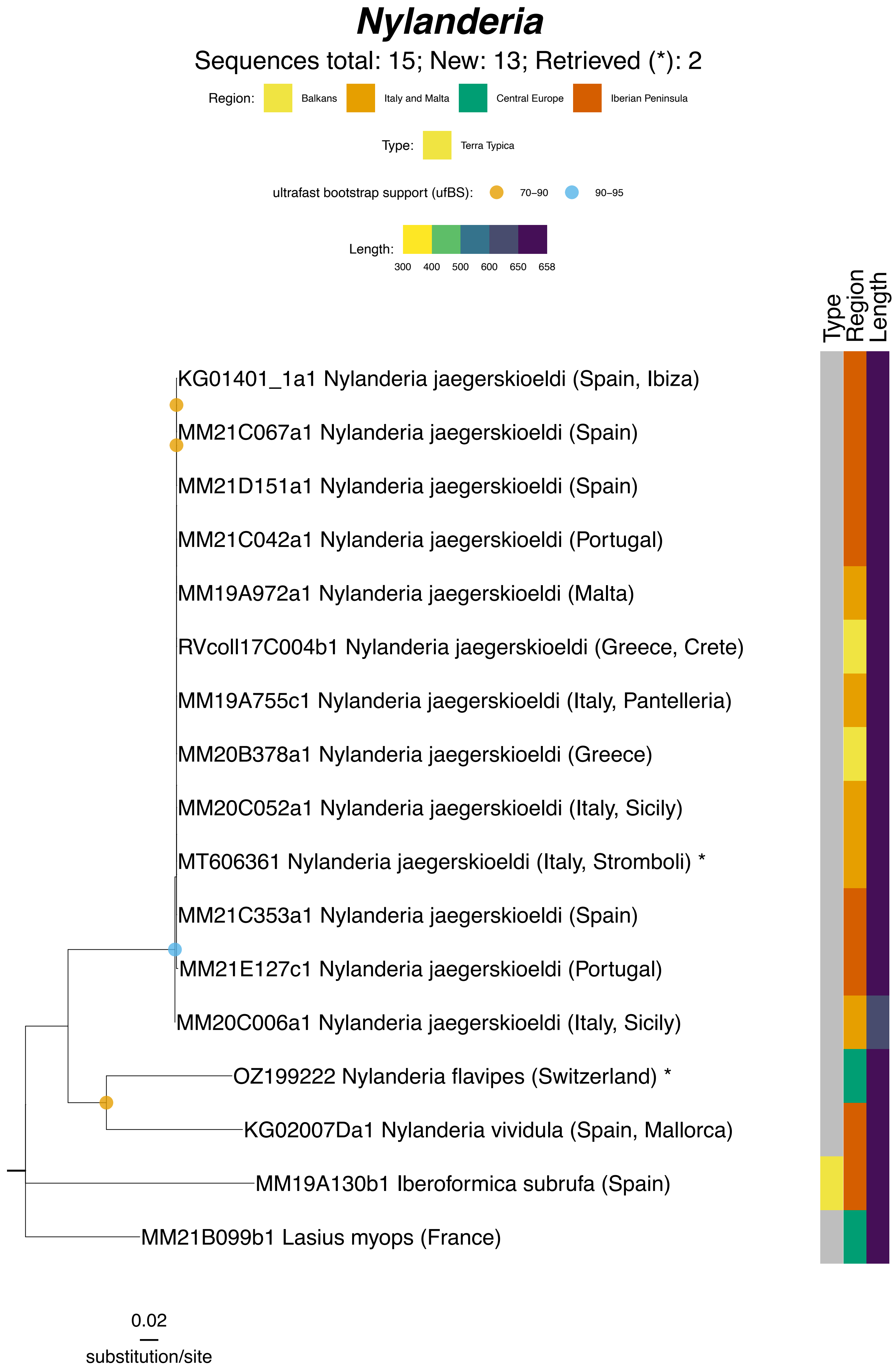

Nylanderia
Sequences total: 15; New: 13; Retrieved (*): 2
Region: Balkans Italy and Malta Central Europe Iberian Peninsula
Type: Terra Typica
ultrafast bootstrap support (ufBS): 70–90 90–95
Length: 300 400 500 600 650 658
Type
Region
Length
KG01401_1a1 Nylanderia jaegerskioeldi (Spain, Ibiza)
MM21C067a1 Nylanderia jaegerskioeldi (Spain)
MM21D151a1 Nylanderia jaegerskioeldi (Spain)
MM21C042a1 Nylanderia jaegerskioeldi (Portugal)
MM19A972a1 Nylanderia jaegerskioeldi (Malta)
RVcoll17C004b1 Nylanderia jaegerskioeldi (Greece, Crete)
MM19A755c1 Nylanderia jaegerskioeldi (Italy, Pantelleria)
MM20B378a1 Nylanderia jaegerskioeldi (Greece)
MM20C052a1 Nylanderia jaegerskioeldi (Italy, Sicily)
MT606361 Nylanderia jaegerskioeldi (Italy, Stromboli) *
MM21C353a1 Nylanderia jaegerskioeldi (Spain)
MM21E127c1 Nylanderia jaegerskioeldi (Portugal)
MM20C006a1 Nylanderia jaegerskioeldi (Italy, Sicily)
OZ199222 Nylanderia flavipes (Switzerland) *
KG02007Da1 Nylanderia vividula (Spain, Mallorca)
MM19A130b1 Iberoformica subrufa (Spain)
MM21B099b1 Lasius myops (France)
0.02
substitution/site

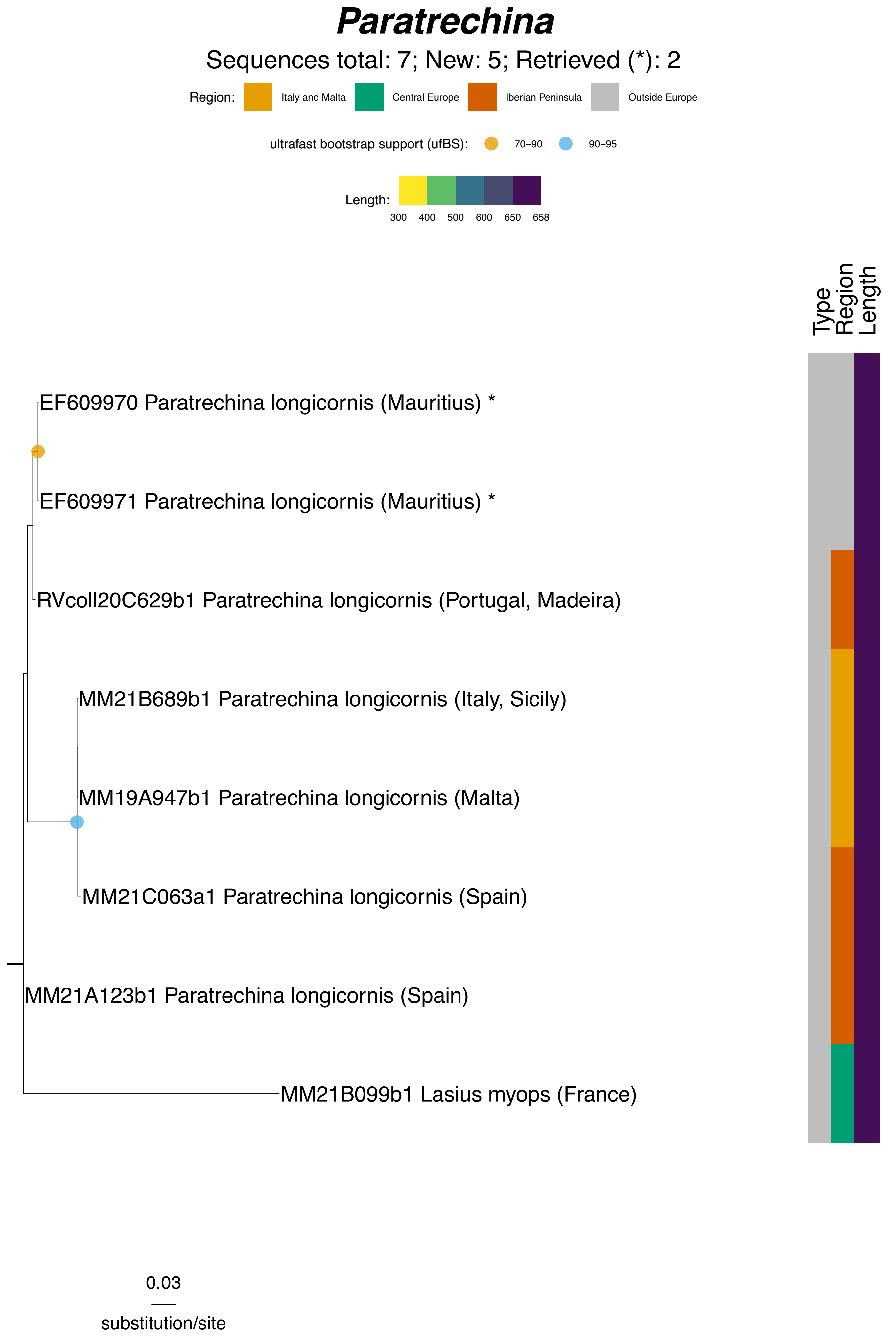

Paratrechina
Sequences total: 7; New: 5; Retrieved (*): 2
Region:
Italy and Malta
Central Europe
Iberian Peninsula
Outside Europe
ultrafast bootstrap support (ufBS):
70–90
90–95
Length:
300
400
500
600
650
658
Type
Region
Length
EF609970 Paratrechina longicornis (Mauritius) *
EF609971 Paratrechina longicornis (Mauritius) *
RVcoll20C629b1 Paratrechina longicornis (Portugal, Madeira)
MM21B689b1 Paratrechina longicornis (Italy, Sicily)
MM19A947b1 Paratrechina longicornis (Malta)
MM21C063a1 Paratrechina longicornis (Spain)
MM21A123b1 Paratrechina longicornis (Spain)
MM21B099b1 Lasius myops (France)
0.03
substitution/site

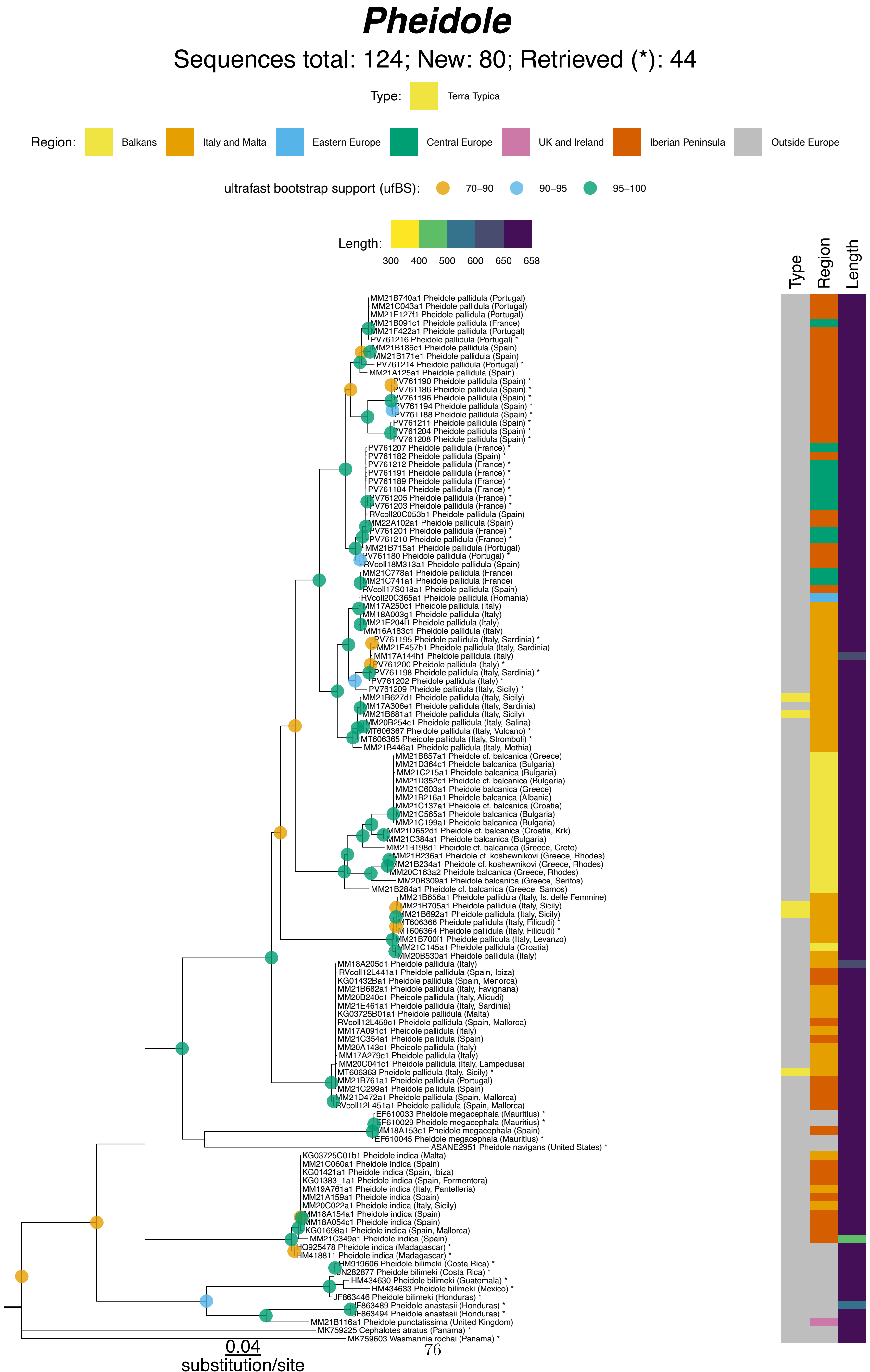

Pheidole
Sequences total: 124; New: 80; Retrieved (*): 44
Type:
Terra Typica
Region:
Balkans
Italy and Malta
Eastern Europe
Central Europe
UK and Ireland
Iberian Peninsula
Outside Europe
ultrafast bootstrap support (ufBS):
70–90
90–95
95–100
Length:
300
400
500
600
650
658
Type
Region
Length
MM21B740a1 Pheidole pallidula (Portugal)
MM21C043a1 Pheidole pallidula (Portugal)
MM21E127f1 Pheidole pallidula (Portugal)
MM21B091c1 Pheidole pallidula (France)
MM21F422a1 Pheidole pallidula (Portugal)
PV761216 Pheidole pallidula (Portugal) *
MM21B186c1 Pheidole pallidula (Spain)
MM21B171e1 Pheidole pallidula (Spain)
PV761214 Pheidole pallidula (Portugal) *
MM21A125a1 Pheidole pallidula (Spain)
PV761190 Pheidole pallidula (Spain) *
PV761186 Pheidole pallidula (Spain) *
PV761196 Pheidole pallidula (Spain) *
PV761194 Pheidole pallidula (Spain) *
PV761188 Pheidole pallidula (Spain) *
PV761211 Pheidole pallidula (Spain) *
PV761204 Pheidole pallidula (Spain) *
PV761208 Pheidole pallidula (Spain) *
PV761207 Pheidole pallidula (France) *
PV761182 Pheidole pallidula (Spain) *
PV761212 Pheidole pallidula (France) *
PV761191 Pheidole pallidula (France) *
PV761189 Pheidole pallidula (France) *
PV761184 Pheidole pallidula (France) *
PV761205 Pheidole pallidula (France) *
PV761203 Pheidole pallidula (France) *
RVcoll20C053b1 Pheidole pallidula (Spain)
MM22A102a1 Pheidole pallidula (Spain)
PV761201 Pheidole pallidula (France) *
PV761210 Pheidole pallidula (France) *
MM21B715a1 Pheidole pallidula (Portugal)
PV761180 Pheidole pallidula (Portugal) *
RVcoll18M313a1 Pheidole pallidula (Spain)
MM21C778a1 Pheidole pallidula (France)
MM21C741a1 Pheidole pallidula (France)
RVcoll17S018a1 Pheidole pallidula (Spain)
RVcoll20C365a1 Pheidole pallidula (Romania)
MM17A250c1 Pheidole pallidula (Italy)
MM18A003g1 Pheidole pallidula (Italy)
MM21E204l1 Pheidole pallidula (Italy)
MM16A183c1 Pheidole pallidula (Italy)
PV761195 Pheidole pallidula (Italy, Sardinia) *
MM21E457b1 Pheidole pallidula (Italy, Sardinia)
MM17A144h1 Pheidole pallidula (Italy)
PV761200 Pheidole pallidula (Italy) *
PV761198 Pheidole pallidula (Italy, Sardinia) *
PV761202 Pheidole pallidula (Italy) *
PV761209 Pheidole pallidula (Italy, Sicily) *
MM21B627d1 Pheidole pallidula (Italy, Sicily)
MM17A306e1 Pheidole pallidula (Italy, Sardinia)
MM21B681a1 Pheidole pallidula (Italy, Sicily)
MM20B254c1 Pheidole pallidula (Italy, Salina)
MT606367 Pheidole pallidula (Italy, Vulcano) *
MT606365 Pheidole pallidula (Italy, Stromboli) *
MM21B446a1 Pheidole pallidula (Italy, Mothia)
MM21B857a1 Pheidole cf. balcanica (Greece)
MM21D364c1 Pheidole balcanica (Bulgaria)
MM21C215a1 Pheidole balcanica (Bulgaria)
MM21D352c1 Pheidole cf. balcanica (Bulgaria)
MM21C603a1 Pheidole balcanica (Greece)
MM21B216a1 Pheidole balcanica (Albania)
MM21C137a1 Pheidole cf. balcanica (Croatia)
MM21C565a1 Pheidole balcanica (Bulgaria)
MM21C199a1 Pheidole balcanica (Bulgaria)
MM21D652d1 Pheidole cf. balcanica (Croatia, Krk)
MM21C384a1 Pheidole balcanica (Bulgaria)
MM21B198d1 Pheidole cf. balcanica (Greece, Crete)
MM21B236a1 Pheidole cf. koshewnikovi (Greece, Rhodes)
MM21B234a1 Pheidole cf. koshewnikovi (Greece, Rhodes)
MM20C163a2 Pheidole balcanica (Greece, Rhodes)
MM20B309a1 Pheidole balcanica (Greece, Serifos)
MM21B284a1 Pheidole cf. balcanica (Greece, Samos)
MM21B656a1 Pheidole pallidula (Italy, Is. delle Femmine)
MM21B705a1 Pheidole pallidula (Italy, Sicily)
MM21B692a1 Pheidole pallidula (Italy, Sicily)
MT606366 Pheidole pallidula (Italy, Filicudi) *
MT606364 Pheidole pallidula (Italy, Filicudi) *
MM21B700f1 Pheidole pallidula (Italy, Levanzo)
MM21C145a1 Pheidole pallidula (Croatia)
MM20B530a1 Pheidole pallidula (Italy)
MM18A205d1 Pheidole pallidula (Italy)
RVcoll12L441a1 Pheidole pallidula (Spain, Ibiza)
KG01432Ba1 Pheidole pallidula (Spain, Menorca)
MM21B682a1 Pheidole pallidula (Italy, Favignana)
MM20B240c1 Pheidole pallidula (Italy, Alicudi)
MM21E461a1 Pheidole pallidula (Italy, Sardinia)
KG03725B01a1 Pheidole pallidula (Malta)
RVcoll12L459c1 Pheidole pallidula (Spain, Mallorca)
MM17A091c1 Pheidole pallidula (Italy)
MM21C354a1 Pheidole pallidula (Spain)
MM20A143c1 Pheidole pallidula (Italy)
MM17A279c1 Pheidole pallidula (Italy)
MM20C041c1 Pheidole pallidula (Italy, Lampedusa)
MT606363 Pheidole pallidula (Italy, Sicily) *
MM21B761a1 Pheidole pallidula (Portugal)
MM21C299a1 Pheidole pallidula (Spain)
MM21D472a1 Pheidole pallidula (Spain, Mallorca)
RVcoll12L451a1 Pheidole pallidula (Spain, Mallorca)
EF610033 Pheidole megacephala (Mauritius) *
EF610029 Pheidole megacephala (Mauritius) *
MM18A153c1 Pheidole megacephala (Spain)
EF610045 Pheidole megacephala (Mauritius) *
ASANE2951 Pheidole navigans (United States) *
KG03725C01b1 Pheidole indica (Malta)
MM21C060a1 Pheidole indica (Spain)
KG01421a1 Pheidole indica (Spain, Ibiza)
KG01383_1a1 Pheidole indica (Spain, Formentera)
MM19A761a1 Pheidole indica (Italy, Pantelleria)
MM21A159a1 Pheidole indica (Spain)
MM20C022a1 Pheidole indica (Italy, Sicily)
MM18A154a1 Pheidole indica (Spain)
MM18A054c1 Pheidole indica (Spain)
KG01698a1 Pheidole indica (Spain, Mallorca)
MM21C349a1 Pheidole indica (Spain)
HQ925478 Pheidole indica (Madagascar) *
HM418811 Pheidole indica (Madagascar) *
HM919606 Pheidole bilimeki (Costa Rica) *
JN282877 Pheidole bilimeki (Costa Rica) *
HM434630 Pheidole bilimeki (Guatemala) *
HM434633 Pheidole bilimeki (Mexico) *
JF863446 Pheidole bilimeki (Honduras) *
JF863489 Pheidole anastasii (Honduras) *
JF863494 Pheidole anastasii (Honduras) *
MM21B116a1 Pheidole punctatissima (United Kingdom)
MK759225 Cephalotes atratus (Panama) *
MK759603 Wasmannia rochai (Panama) *
0.04
substitution/site

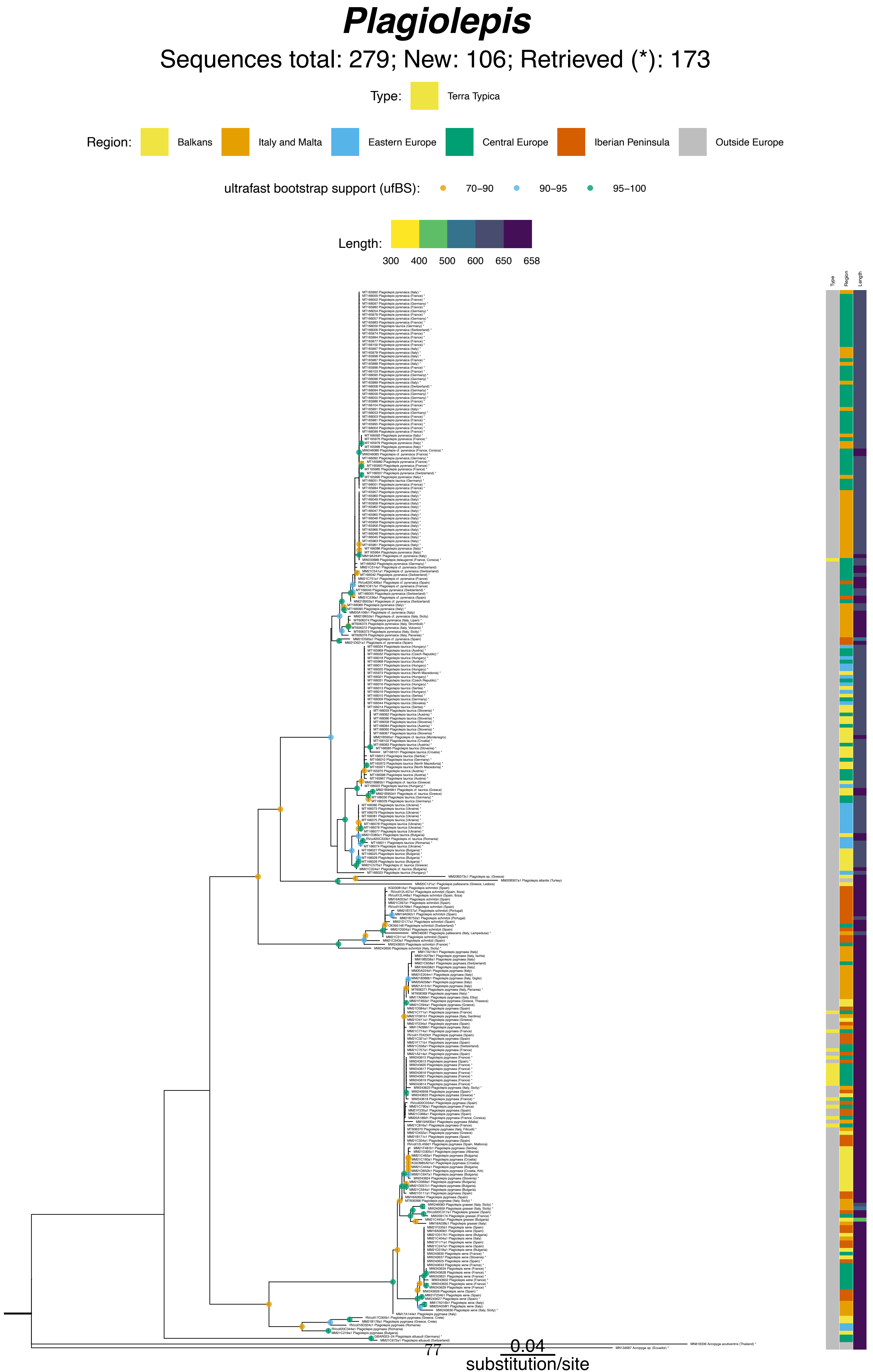
Plagiolepis
Sequences total: 279; New: 106; Retrieved (*): 173
Type:
Terra Typica
Region:
Balkans
Italy and Malta
Eastern Europe
Central Europe
Iberian Peninsula
Outside Europe
ultrafast bootstrap support (ufBS):
70–90
90–95
95–100
Length:
300
400
500
600
650
658
Type
Region
Length
0.04
substitution/site

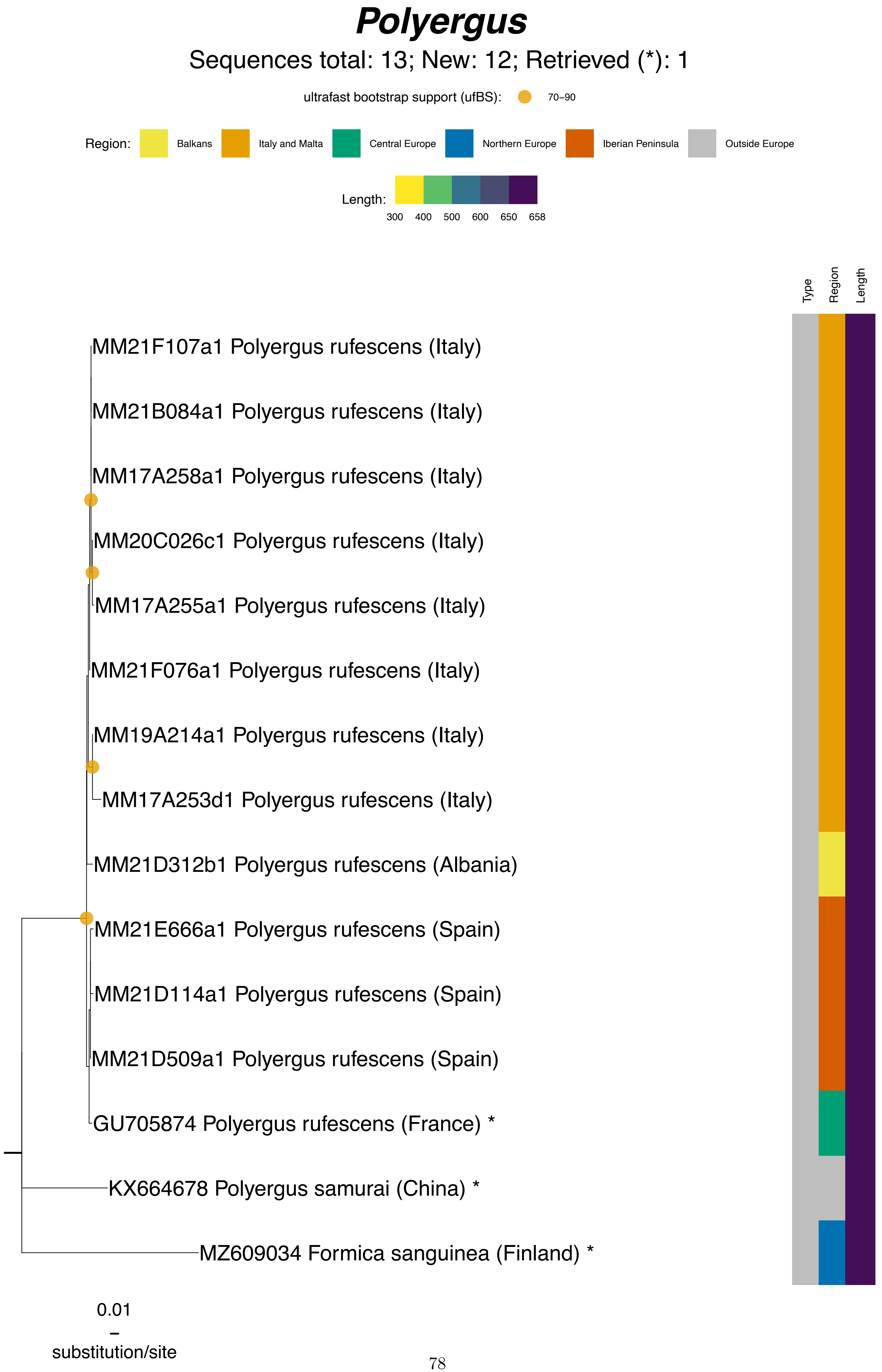
Polyergus
Sequences total: 13; New: 12; Retrieved (*): 1
ultrafast bootstrap support (ufBS):
70–90
Region:
Balkans
Italy and Malta
Central Europe
Northern Europe
Iberian Peninsula
Outside Europe
Length:
300
400
500
600
650
658
Type
Region
Length
MM21F107a1 Polyergus rufescens (Italy)
MM21B084a1 Polyergus rufescens (Italy)
MM17A258a1 Polyergus rufescens (Italy)
MM20C026c1 Polyergus rufescens (Italy)
MM17A255a1 Polyergus rufescens (Italy)
MM21F076a1 Polyergus rufescens (Italy)
MM19A214a1 Polyergus rufescens (Italy)
MM17A253d1 Polyergus rufescens (Italy)
MM21D312b1 Polyergus rufescens (Albania)
MM21E666a1 Polyergus rufescens (Spain)
MM21D114a1 Polyergus rufescens (Spain)
MM21D509a1 Polyergus rufescens (Spain)
GU705874 Polyergus rufescens (France) *
KX664678 Polyergus samurai (China) *
MZ609034 Formica sanguinea (Finland) *
0.01
substitution/site

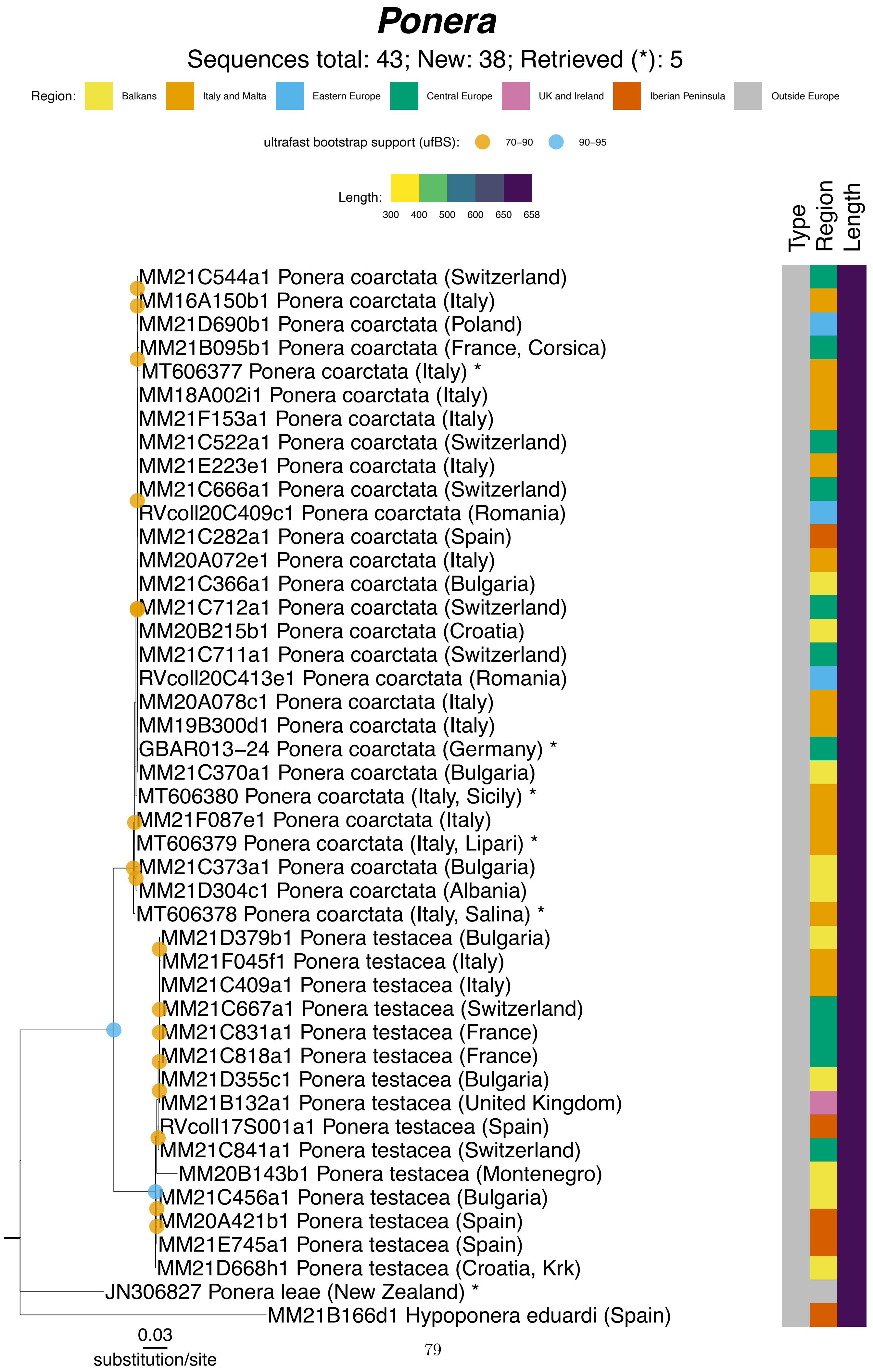
Ponera
Sequences total: 43; New: 38; Retrieved (*): 5
Region:
Balkans
Italy and Malta
Eastern Europe
Central Europe
UK and Ireland
Iberian Peninsula
Outside Europe
ultrafast bootstrap support (ufBS):
70–90
90–95
Length:
300
400
500
600
650
658
Type
Region
Length
MM21C544a1 Ponera coarctata (Switzerland)
MM16A150b1 Ponera coarctata (Italy)
MM21D690b1 Ponera coarctata (Poland)
MM21B095b1 Ponera coarctata (France, Corsica)
MT606377 Ponera coarctata (Italy) *
MM18A002i1 Ponera coarctata (Italy)
MM21F153a1 Ponera coarctata (Italy)
MM21C522a1 Ponera coarctata (Switzerland)
MM21E223e1 Ponera coarctata (Italy)
MM21C666a1 Ponera coarctata (Switzerland)
RVcoll20C409c1 Ponera coarctata (Romania)
MM21C282a1 Ponera coarctata (Spain)
MM20A072e1 Ponera coarctata (Italy)
MM21C366a1 Ponera coarctata (Bulgaria)
MM21C712a1 Ponera coarctata (Switzerland)
MM20B215b1 Ponera coarctata (Croatia)
MM21C711a1 Ponera coarctata (Switzerland)
RVcoll20C413e1 Ponera coarctata (Romania)
MM20A078c1 Ponera coarctata (Italy)
MM19B300d1 Ponera coarctata (Italy)
GBAR013–24 Ponera coarctata (Germany) *
MM21C370a1 Ponera coarctata (Bulgaria)
MT606380 Ponera coarctata (Italy, Sicily) *
MM21F087e1 Ponera coarctata (Italy)
MT606379 Ponera coarctata (Italy, Lipari) *
MM21C373a1 Ponera coarctata (Bulgaria)
MM21D304c1 Ponera coarctata (Albania)
MT606378 Ponera coarctata (Italy, Salina) *
MM21D379b1 Ponera testacea (Bulgaria)
MM21F045f1 Ponera testacea (Italy)
MM21C409a1 Ponera testacea (Italy)
MM21C667a1 Ponera testacea (Switzerland)
MM21C831a1 Ponera testacea (France)
MM21C818a1 Ponera testacea (France)
MM21D355c1 Ponera testacea (Bulgaria)
MM21B132a1 Ponera testacea (United Kingdom)
RVcoll17S001a1 Ponera testacea (Spain)
MM21C841a1 Ponera testacea (Switzerland)
MM20B143b1 Ponera testacea (Montenegro)
MM21C456a1 Ponera testacea (Bulgaria)
MM20A421b1 Ponera testacea (Spain)
MM21E745a1 Ponera testacea (Spain)
MM21D668h1 Ponera testacea (Croatia, Krk)
JN306827 Ponera leae (New Zealand) *
MM21B166d1 Hypoponera eduardi (Spain)
0.03
substitution/site

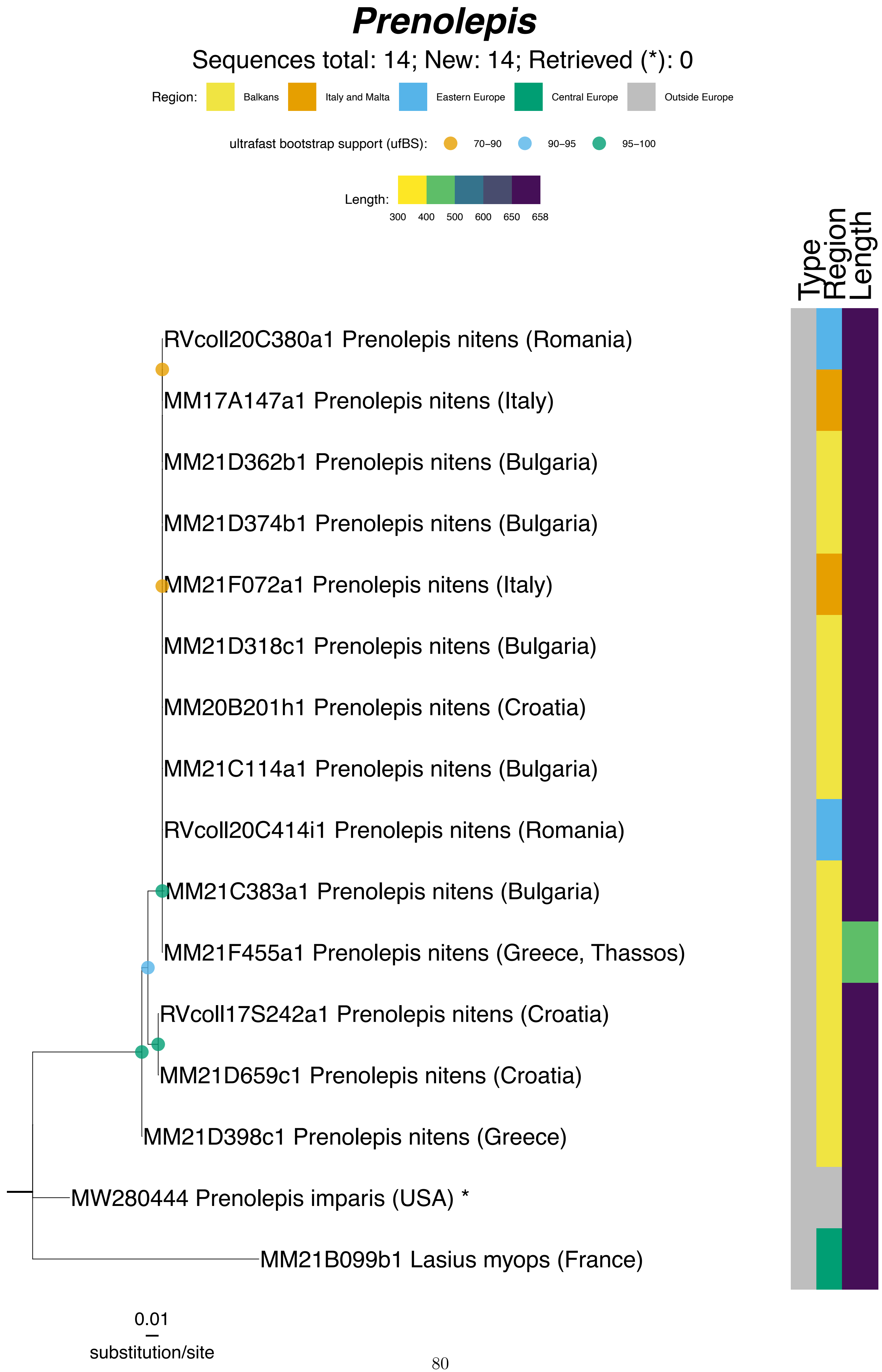
Prenolepis
Sequences total: 14; New: 14; Retrieved (*): 0
Region: Balkans Italy and Malta Eastern Europe Central Europe Outside Europe
ultrafast bootstrap support (ufBS): 70–90 90–95 95–100
Length: 300 400 500 600 650 658
Type Region Length
RVcoll20C380a1 Prenolepis nitens (Romania)
MM17A147a1 Prenolepis nitens (Italy)
MM21D362b1 Prenolepis nitens (Bulgaria)
MM21D374b1 Prenolepis nitens (Bulgaria)
MM21F072a1 Prenolepis nitens (Italy)
MM21D318c1 Prenolepis nitens (Bulgaria)
MM20B201h1 Prenolepis nitens (Croatia)
MM21C114a1 Prenolepis nitens (Bulgaria)
RVcoll20C414i1 Prenolepis nitens (Romania)
MM21C383a1 Prenolepis nitens (Bulgaria)
MM21F455a1 Prenolepis nitens (Greece, Thassos)
RVcoll17S242a1 Prenolepis nitens (Croatia)
MM21D659c1 Prenolepis nitens (Croatia)
MM21D398c1 Prenolepis nitens (Greece)
MW280444 Prenolepis imparis (USA) *
MM21B099b1 Lasius myops (France)
0.01
substitution/site

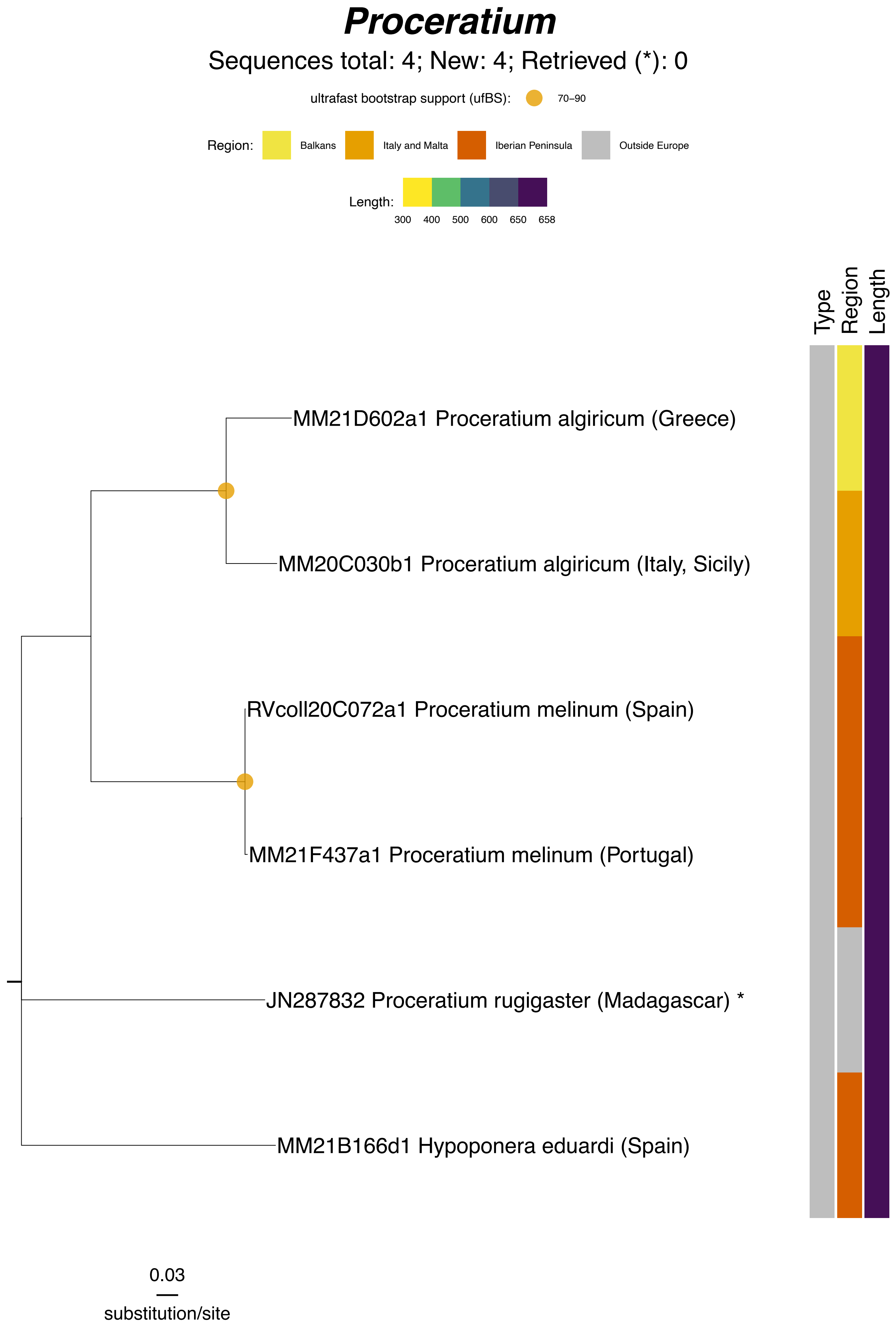
Proceratium
Sequences total: 4; New: 4; Retrieved (*): 0
ultrafast bootstrap support (ufBS):
70–90
Region:
Balkans
Italy and Malta
Iberian Peninsula
Outside Europe
Length:
300
400
500
600
650
658
Type
Region
Length
MM21D602a1 Proceratium algiricum (Greece)
MM20C030b1 Proceratium algiricum (Italy, Sicily)
RVcoll20C072a1 Proceratium melinum (Spain)
MM21F437a1 Proceratium melinum (Portugal)
JN287832 Proceratium rugigaster (Madagascar) *
MM21B166d1 Hypoponera eduardi (Spain)
0.03
substitution/site

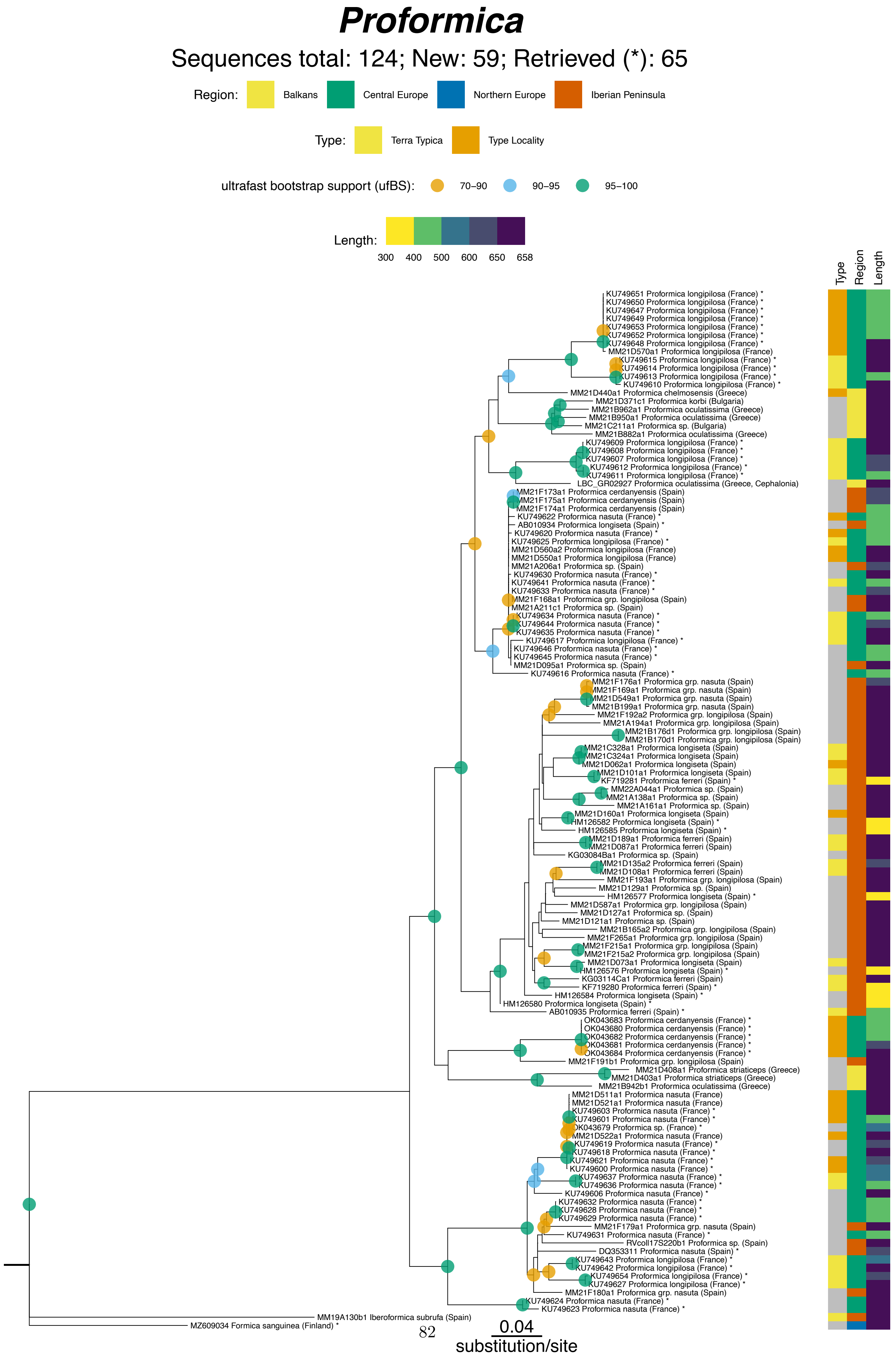
Proformica
Sequences total: 124; New: 59; Retrieved (*): 65
Region: Balkans Central Europe Northern Europe Iberian Peninsula
Type: Terra Typica Type Locality
ultrafast bootstrap support (ufBS): 70–90 90–95 95–100
Length: 300 400 500 600 650 658
Type Region Length
KU749651 Proformica longipilosa (France) *
KU749650 Proformica longipilosa (France) *
KU749647 Proformica longipilosa (France) *
KU749649 Proformica longipilosa (France) *
KU749653 Proformica longipilosa (France) *
KU749652 Proformica longipilosa (France) *
KU749648 Proformica longipilosa (France) *
MM21D570a1 Proformica longipilosa (France)
KU749615 Proformica longipilosa (France) *
KU749614 Proformica longipilosa (France) *
KU749613 Proformica longipilosa (France) *
KU749610 Proformica longipilosa (France) *
MM21D440a1 Proformica chelmosensis (Greece)
MM21D371c1 Proformica korbi (Bulgaria)
MM21B962a1 Proformica oculatissima (Greece)
MM21B950a1 Proformica oculatissima (Greece)
MM21C211a1 Proformica sp. (Bulgaria)
MM21B882a1 Proformica oculatissima (Greece)
KU749609 Proformica longipilosa (France) *
KU749608 Proformica longipilosa (France) *
KU749607 Proformica longipilosa (France) *
KU749612 Proformica longipilosa (France) *
KU749611 Proformica longipilosa (France) *
LBC_GR02927 Proformica oculatissima (Greece, Cephalonia)
MM21F173a1 Proformica cerdanyensis (Spain)
MM21F175a1 Proformica cerdanyensis (Spain)
MM21F174a1 Proformica cerdanyensis (Spain)
KU749622 Proformica nasuta (France) *
AB010934 Proformica longiseta (Spain) *
KU749620 Proformica nasuta (France) *
KU749625 Proformica longipilosa (France) *
MM21D560a2 Proformica longipilosa (France)
MM21D550a1 Proformica longipilosa (France)
MM21A206a1 Proformica sp. (Spain)
KU749630 Proformica nasuta (France) *
KU749641 Proformica nasuta (France) *
KU749633 Proformica nasuta (France) *
MM21F168a1 Proformica grp. longipilosa (Spain)
MM21A211c1 Proformica sp. (Spain)
KU749634 Proformica nasuta (France) *
KU749644 Proformica nasuta (France) *
KU749635 Proformica nasuta (France) *
KU749617 Proformica longipilosa (France) *
KU749646 Proformica nasuta (France) *
KU749645 Proformica nasuta (France) *
MM21D095a1 Proformica sp. (Spain)
KU749616 Proformica nasuta (France) *
MM21F176a1 Proformica grp. nasuta (Spain)
MM21F169a1 Proformica grp. nasuta (Spain)
MM21D549a1 Proformica grp. nasuta (Spain)
MM21B199a1 Proformica grp. nasuta (Spain)
MM21F192a2 Proformica grp. longipilosa (Spain)
MM21A194a1 Proformica grp. longipilosa (Spain)
MM21B176d1 Proformica grp. longipilosa (Spain)
MM21B170d1 Proformica grp. longipilosa (Spain)
MM21C328a1 Proformica longiseta (Spain)
MM21C324a1 Proformica longiseta (Spain)
MM21D062a1 Proformica longiseta (Spain)
MM21D101a1 Proformica longiseta (Spain)
KF719281 Proformica ferreri (Spain) *
MM22A044a1 Proformica sp. (Spain)
MM21A138a1 Proformica sp. (Spain)
MM21A161a1 Proformica sp. (Spain)
MM21D160a1 Proformica longiseta (Spain)
HM126582 Proformica longiseta (Spain) *
HM126585 Proformica longiseta (Spain) *
MM21D189a1 Proformica ferreri (Spain)
MM21D087a1 Proformica ferreri (Spain)
KG03084Ba1 Proformica sp. (Spain)
MM21D135a2 Proformica ferreri (Spain)
MM21D108a1 Proformica ferreri (Spain)
MM21F193a1 Proformica grp. longipilosa (Spain)
MM21D129a1 Proformica sp. (Spain)
HM126577 Proformica longiseta (Spain) *
MM21D587a1 Proformica grp. longipilosa (Spain)
MM21D127a1 Proformica sp. (Spain)
MM21D121a1 Proformica sp. (Spain)
MM21B165a2 Proformica grp. longipilosa (Spain)
MM21F265a1 Proformica grp. longipilosa (Spain)
MM21F215a1 Proformica grp. longipilosa (Spain)
MM21F215a2 Proformica grp. longipilosa (Spain)
MM21D073a1 Proformica longiseta (Spain)
HM126576 Proformica longiseta (Spain) *
KG03114Ca1 Proformica ferreri (Spain)
KF719280 Proformica ferreri (Spain) *
HM126584 Proformica longiseta (Spain) *
HM126580 Proformica longiseta (Spain) *
AB010935 Proformica ferreri (Spain) *
OK043683 Proformica cerdanyensis (France) *
OK043680 Proformica cerdanyensis (France) *
OK043682 Proformica cerdanyensis (France) *
OK043681 Proformica cerdanyensis (France) *
OK043684 Proformica cerdanyensis (France) *
MM21F191b1 Proformica grp. longipilosa (Spain)
MM21D408a1 Proformica striaticeps (Greece)
MM21D403a1 Proformica striaticeps (Greece)
MM21B942b1 Proformica oculatissima (Greece)
MM21D511a1 Proformica nasuta (France)
MM21D521a1 Proformica nasuta (France)
KU749603 Proformica nasuta (France) *
KU749601 Proformica nasuta (France) *
OK043679 Proformica sp. (France) *
MM21D522a1 Proformica nasuta (France)
KU749619 Proformica nasuta (France) *
KU749618 Proformica nasuta (France) *
KU749621 Proformica nasuta (France) *
KU749600 Proformica nasuta (France) *
KU749637 Proformica nasuta (France) *
KU749636 Proformica nasuta (France) *
KU749606 Proformica nasuta (France) *
KU749632 Proformica nasuta (France) *
KU749628 Proformica nasuta (France) *
KU749629 Proformica nasuta (France) *
MM21F179a1 Proformica grp. nasuta (Spain)
KU749631 Proformica nasuta (France) *
RVcoll17S220b1 Proformica sp. (Spain)
DQ353311 Proformica nasuta (Spain) *
KU749643 Proformica longipilosa (France) *
KU749642 Proformica longipilosa (France) *
KU749654 Proformica longipilosa (France) *
KU749627 Proformica longipilosa (France) *
MM21F180a1 Proformica grp. nasuta (Spain)
KU749624 Proformica nasuta (France) *
KU749623 Proformica nasuta (France) *
MM19A130b1 Iberoformica subrufa (Spain)
MZ609034 Formica sanguinea (Finland) *
0.04
substitution/site

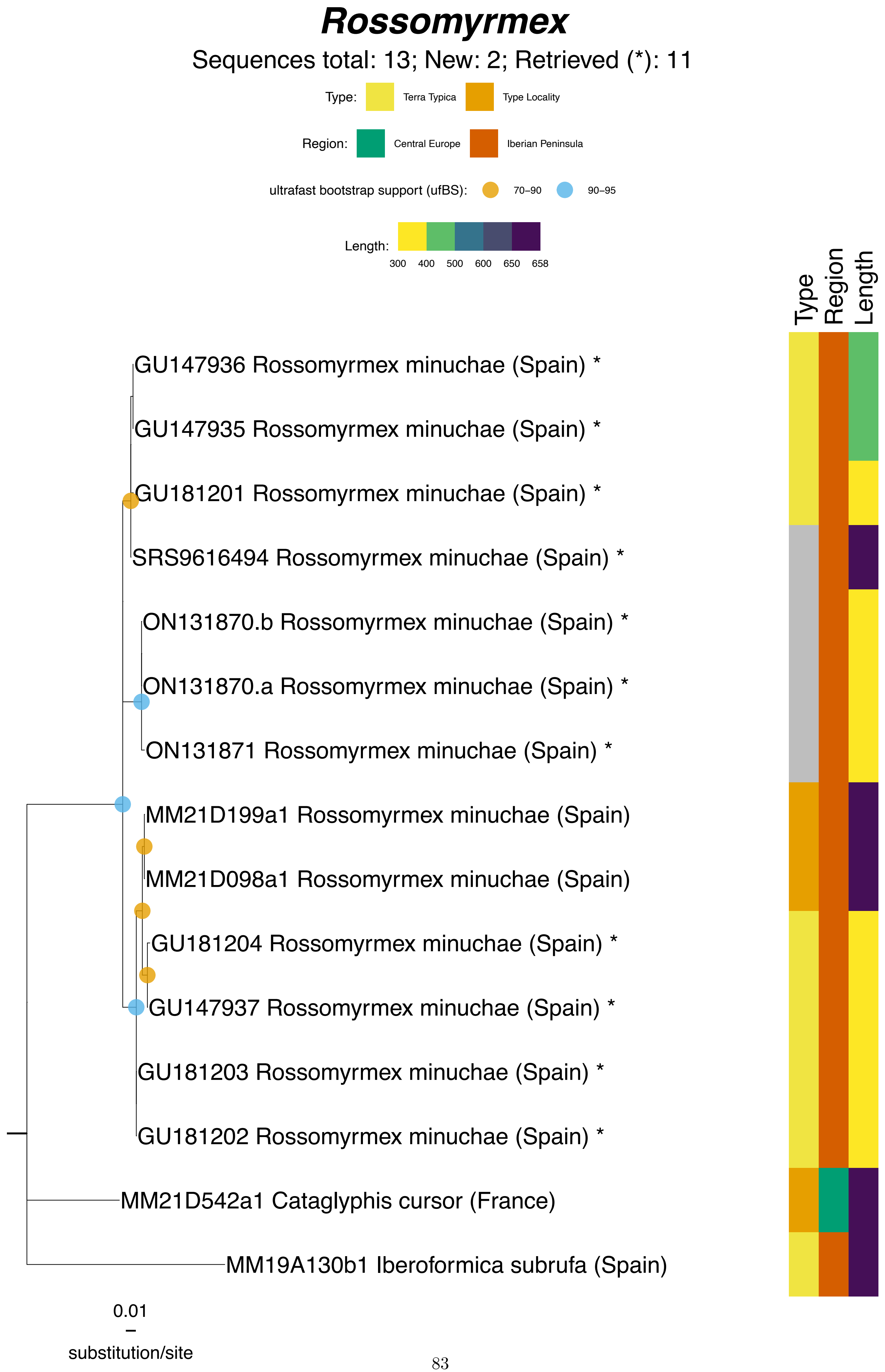
Rossomyrmex
Sequences total: 13; New: 2; Retrieved (*): 11
Type: Terra Typica Type Locality
Region: Central Europe Iberian Peninsula
ultrafast bootstrap support (ufBS): 70–90 90–95
Length: 300 400 500 600 650 658
Type
Region
Length
GU147936 Rossomyrmex minuchae (Spain) *
GU147935 Rossomyrmex minuchae (Spain) *
GU181201 Rossomyrmex minuchae (Spain) *
SRS9616494 Rossomyrmex minuchae (Spain) *
ON131870.b Rossomyrmex minuchae (Spain) *
ON131870.a Rossomyrmex minuchae (Spain) *
ON131871 Rossomyrmex minuchae (Spain) *
MM21D199a1 Rossomyrmex minuchae (Spain)
MM21D098a1 Rossomyrmex minuchae (Spain)
GU181204 Rossomyrmex minuchae (Spain) *
GU147937 Rossomyrmex minuchae (Spain) *
GU181203 Rossomyrmex minuchae (Spain) *
GU181202 Rossomyrmex minuchae (Spain) *
MM21D542a1 Cataglyphis cursor (France)
MM19A130b1 Iberoformica subrufa (Spain)
0.01
substitution/site

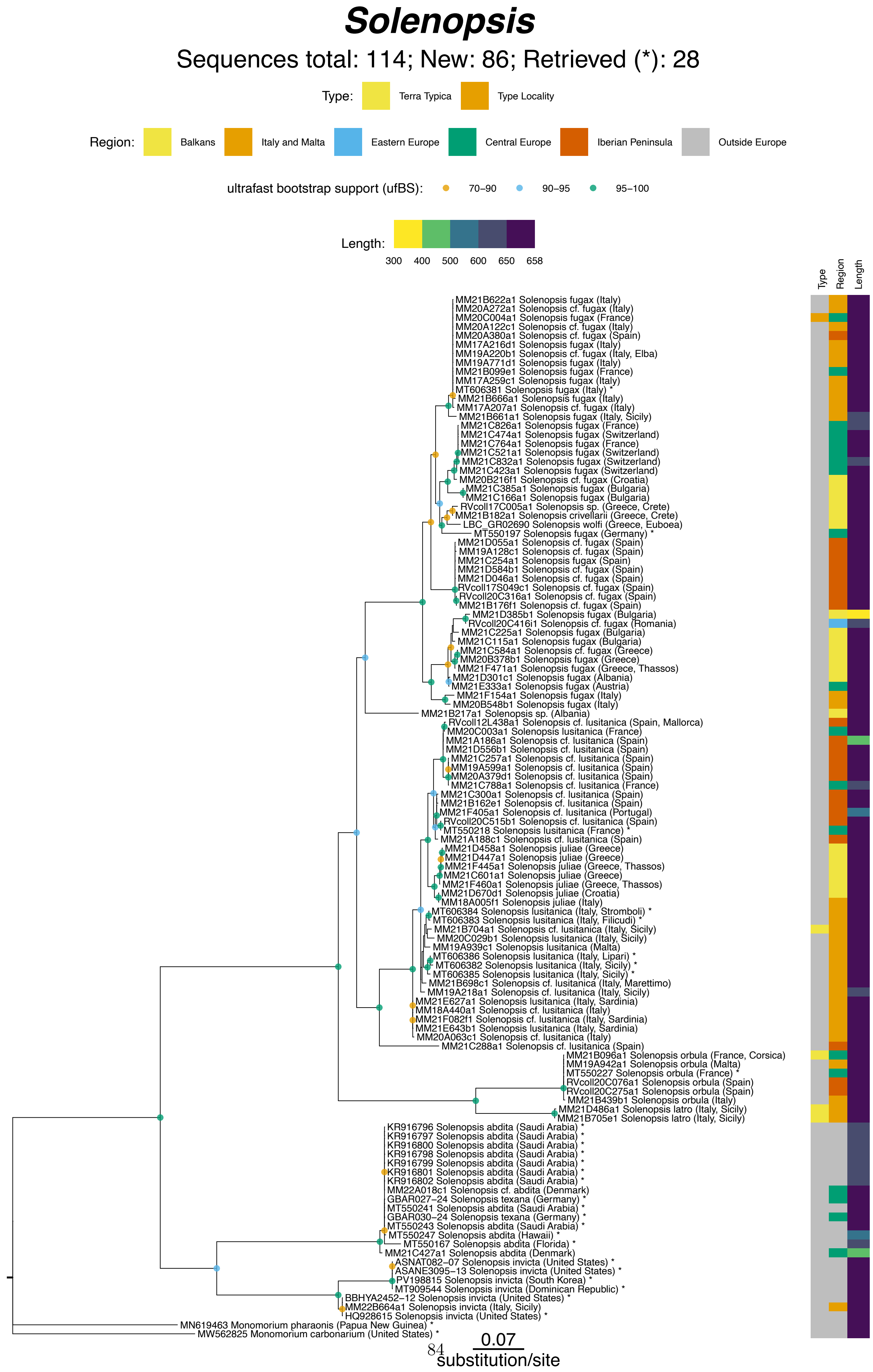
Solenopsis
Sequences total: 114; New: 86; Retrieved (*): 28
Type:
Terra Typica
Type Locality
Region:
Balkans
Italy and Malta
Eastern Europe
Central Europe
Iberian Peninsula
Outside Europe
ultrafast bootstrap support (ufBS):
70–90
90–95
95–100
Length:
300
400
500
600
650
658
Type
Region
Length
MM21B622a1 Solenopsis fugax (Italy)
MM20A272a1 Solenopsis cf. fugax (Italy)
MM20C004a1 Solenopsis fugax (France)
MM20A122c1 Solenopsis cf. fugax (Italy)
MM20A380a1 Solenopsis cf. fugax (Spain)
MM17A216d1 Solenopsis fugax (Italy)
MM19A220b1 Solenopsis cf. fugax (Italy, Elba)
MM19A771d1 Solenopsis fugax (Italy)
MM21B099e1 Solenopsis fugax (France)
MM17A259c1 Solenopsis fugax (Italy)
MT606381 Solenopsis fugax (Italy) *
MM21B666a1 Solenopsis fugax (Italy)
MM17A207a1 Solenopsis cf. fugax (Italy)
MM21B661a1 Solenopsis fugax (Italy, Sicily)
MM21C826a1 Solenopsis fugax (France)
MM21C474a1 Solenopsis fugax (Switzerland)
MM21C764a1 Solenopsis fugax (France)
MM21C521a1 Solenopsis fugax (Switzerland)
MM21C832a1 Solenopsis fugax (Switzerland)
MM21C423a1 Solenopsis fugax (Switzerland)
MM20B216f1 Solenopsis cf. fugax (Croatia)
MM21C385a1 Solenopsis fugax (Bulgaria)
MM21C166a1 Solenopsis fugax (Bulgaria)
RVcoll17C005a1 Solenopsis sp. (Greece, Crete)
MM21B182a1 Solenopsis crivellarii (Greece, Crete)
LBC_GR02690 Solenopsis wolfi (Greece, Euboea)
MT550197 Solenopsis fugax (Germany) *
MM21D055a1 Solenopsis cf. fugax (Spain)
MM19A128c1 Solenopsis cf. fugax (Spain)
MM21C254a1 Solenopsis fugax (Spain)
MM21D584b1 Solenopsis cf. fugax (Spain)
MM21D046a1 Solenopsis cf. fugax (Spain)
RVcoll17S049c1 Solenopsis cf. fugax (Spain)
RVcoll20C316a1 Solenopsis cf. fugax (Spain)
MM21B176f1 Solenopsis cf. fugax (Spain)
MM21D385b1 Solenopsis fugax (Bulgaria)
RVcoll20C416i1 Solenopsis cf. fugax (Romania)
MM21C225a1 Solenopsis fugax (Bulgaria)
MM21C115a1 Solenopsis fugax (Bulgaria)
MM21C584a1 Solenopsis cf. fugax (Greece)
MM20B378b1 Solenopsis fugax (Greece)
MM21F471a1 Solenopsis fugax (Greece, Thassos)
MM21D301c1 Solenopsis fugax (Albania)
MM21E333a1 Solenopsis fugax (Austria)
MM21F154a1 Solenopsis fugax (Italy)
MM20B548b1 Solenopsis fugax (Italy)
MM21B217a1 Solenopsis sp. (Albania)
RVcoll12L438a1 Solenopsis cf. lusitanica (Spain, Mallorca)
MM20C003a1 Solenopsis lusitanica (France)
MM21A186a1 Solenopsis cf. lusitanica (Spain)
MM21D556b1 Solenopsis cf. lusitanica (Spain)
MM21C257a1 Solenopsis cf. lusitanica (Spain)
MM19A599a1 Solenopsis cf. lusitanica (Spain)
MM20A379d1 Solenopsis cf. lusitanica (Spain)
MM21C788a1 Solenopsis cf. lusitanica (France)
MM21C300a1 Solenopsis cf. lusitanica (Spain)
MM21B162e1 Solenopsis cf. lusitanica (Spain)
MM21F405a1 Solenopsis cf. lusitanica (Portugal)
RVcoll20C515b1 Solenopsis cf. lusitanica (Spain)
MT550218 Solenopsis lusitanica (France) *
MM21A188c1 Solenopsis cf. lusitanica (Spain)
MM21D458a1 Solenopsis juliae (Greece)
MM21D447a1 Solenopsis juliae (Greece)
MM21F445a1 Solenopsis juliae (Greece, Thassos)
MM21C601a1 Solenopsis juliae (Greece)
MM21F460a1 Solenopsis juliae (Greece, Thassos)
MM21D670d1 Solenopsis juliae (Croatia)
MM18A005f1 Solenopsis juliae (Italy)
MT606384 Solenopsis lusitanica (Italy, Stromboli) *
MT606383 Solenopsis lusitanica (Italy, Filicudi) *
MM21B704a1 Solenopsis cf. lusitanica (Italy, Sicily)
MM20C029b1 Solenopsis lusitanica (Italy, Sicily)
MM19A939c1 Solenopsis lusitanica (Malta)
MT606386 Solenopsis lusitanica (Italy, Lipari) *
MT606382 Solenopsis lusitanica (Italy, Sicily) *
MT606385 Solenopsis lusitanica (Italy, Sicily) *
MM21B698c1 Solenopsis cf. lusitanica (Italy, Marettimo)
MM19A218a1 Solenopsis cf. lusitanica (Italy, Sicily)
MM21E627a1 Solenopsis lusitanica (Italy, Sardinia)
MM18A440a1 Solenopsis cf. lusitanica (Italy)
MM21F082f1 Solenopsis cf. lusitanica (Italy, Sardinia)
MM21E643b1 Solenopsis lusitanica (Italy, Sardinia)
MM20A063c1 Solenopsis cf. lusitanica (Italy)
MM21C288a1 Solenopsis cf. lusitanica (Spain)
MM21B096a1 Solenopsis orbula (France, Corsica)
MM19A942a1 Solenopsis orbula (Malta)
MT550227 Solenopsis orbula (France) *
RVcoll20C076a1 Solenopsis orbula (Spain)
RVcoll20C275a1 Solenopsis orbula (Spain)
MM21B439b1 Solenopsis orbula (Italy)
MM21D486a1 Solenopsis latro (Italy, Sicily)
MM21B705e1 Solenopsis latro (Italy, Sicily)
KR916796 Solenopsis abdita (Saudi Arabia) *
KR916797 Solenopsis abdita (Saudi Arabia) *
KR916800 Solenopsis abdita (Saudi Arabia) *
KR916798 Solenopsis abdita (Saudi Arabia) *
KR916799 Solenopsis abdita (Saudi Arabia) *
KR916801 Solenopsis abdita (Saudi Arabia) *
KR916802 Solenopsis abdita (Saudi Arabia) *
MM22A018c1 Solenopsis cf. abdita (Denmark)
GBAR027–24 Solenopsis texana (Germany) *
MT550241 Solenopsis abdita (Saudi Arabia) *
GBAR030–24 Solenopsis texana (Germany) *
MT550243 Solenopsis abdita (Saudi Arabia) *
MT550247 Solenopsis abdita (Hawaii) *
MT550167 Solenopsis abdita (Florida) *
MM21C427a1 Solenopsis abdita (Denmark)
ASNAT082–07 Solenopsis invicta (United States) *
ASANE3095–13 Solenopsis invicta (United States) *
PV198815 Solenopsis invicta (South Korea) *
MT909544 Solenopsis invicta (Dominican Republic) *
BBHYA2452–12 Solenopsis invicta (United States) *
MM22B664a1 Solenopsis invicta (Italy, Sicily)
HQ928615 Solenopsis invicta (United States) *
MN619463 Monomorium pharaonis (Papua New Guinea) *
MW562825 Monomorium carbonarium (United States) *
0.07
substitution/site

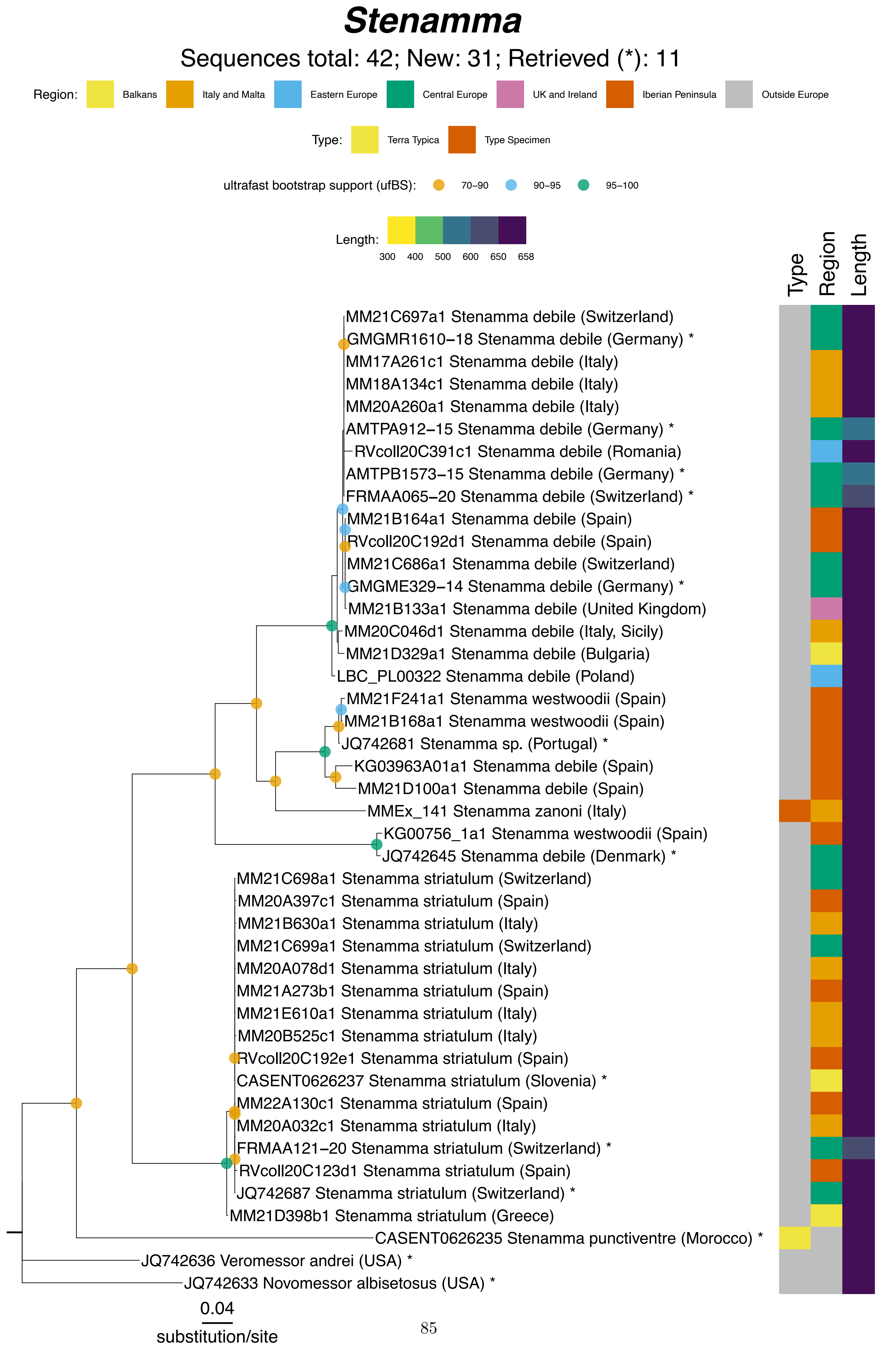
Stenamma
Sequences total: 42; New: 31; Retrieved (*): 11
Region:
Balkans
Italy and Malta
Eastern Europe
Central Europe
UK and Ireland
Iberian Peninsula
Outside Europe
Type:
Terra Typica
Type Specimen
ultrafast bootstrap support (ufBS):
70–90
90–95
95–100
Length:
300
400
500
600
650
658
Type
Region
Length
MM21C697a1 Stenamma debile (Switzerland)
GMGMR1610–18 Stenamma debile (Germany) *
MM17A261c1 Stenamma debile (Italy)
MM18A134c1 Stenamma debile (Italy)
MM20A260a1 Stenamma debile (Italy)
AMTPA912–15 Stenamma debile (Germany) *
RVcoll20C391c1 Stenamma debile (Romania)
AMTPB1573–15 Stenamma debile (Germany) *
FRMAA065–20 Stenamma debile (Switzerland) *
MM21B164a1 Stenamma debile (Spain)
RVcoll20C192d1 Stenamma debile (Spain)
MM21C686a1 Stenamma debile (Switzerland)
GMGME329–14 Stenamma debile (Germany) *
MM21B133a1 Stenamma debile (United Kingdom)
MM20C046d1 Stenamma debile (Italy, Sicily)
MM21D329a1 Stenamma debile (Bulgaria)
LBC_PL00322 Stenamma debile (Poland)
MM21F241a1 Stenamma westwoodii (Spain)
MM21B168a1 Stenamma westwoodii (Spain)
JQ742681 Stenamma sp. (Portugal) *
KG03963A01a1 Stenamma debile (Spain)
MM21D100a1 Stenamma debile (Spain)
MMEx_141 Stenamma zanoni (Italy)
KG00756_1a1 Stenamma westwoodii (Spain)
JQ742645 Stenamma debile (Denmark) *
MM21C698a1 Stenamma striatulum (Switzerland)
MM20A397c1 Stenamma striatulum (Spain)
MM21B630a1 Stenamma striatulum (Italy)
MM21C699a1 Stenamma striatulum (Switzerland)
MM20A078d1 Stenamma striatulum (Italy)
MM21A273b1 Stenamma striatulum (Spain)
MM21E610a1 Stenamma striatulum (Italy)
MM20B525c1 Stenamma striatulum (Italy)
RVcoll20C192e1 Stenamma striatulum (Spain)
CASENT0626237 Stenamma striatulum (Slovenia) *
MM22A130c1 Stenamma striatulum (Spain)
MM20A032c1 Stenamma striatulum (Italy)
FRMAA121–20 Stenamma striatulum (Switzerland) *
RVcoll20C123d1 Stenamma striatulum (Spain)
JQ742687 Stenamma striatulum (Switzerland) *
MM21D398b1 Stenamma striatulum (Greece)
CASENT0626235 Stenamma punctiventre (Morocco) *
JQ742636 Veromessor andrei (USA) *
JQ742633 Novomessor albisetosus (USA) *
0.04
substitution/site

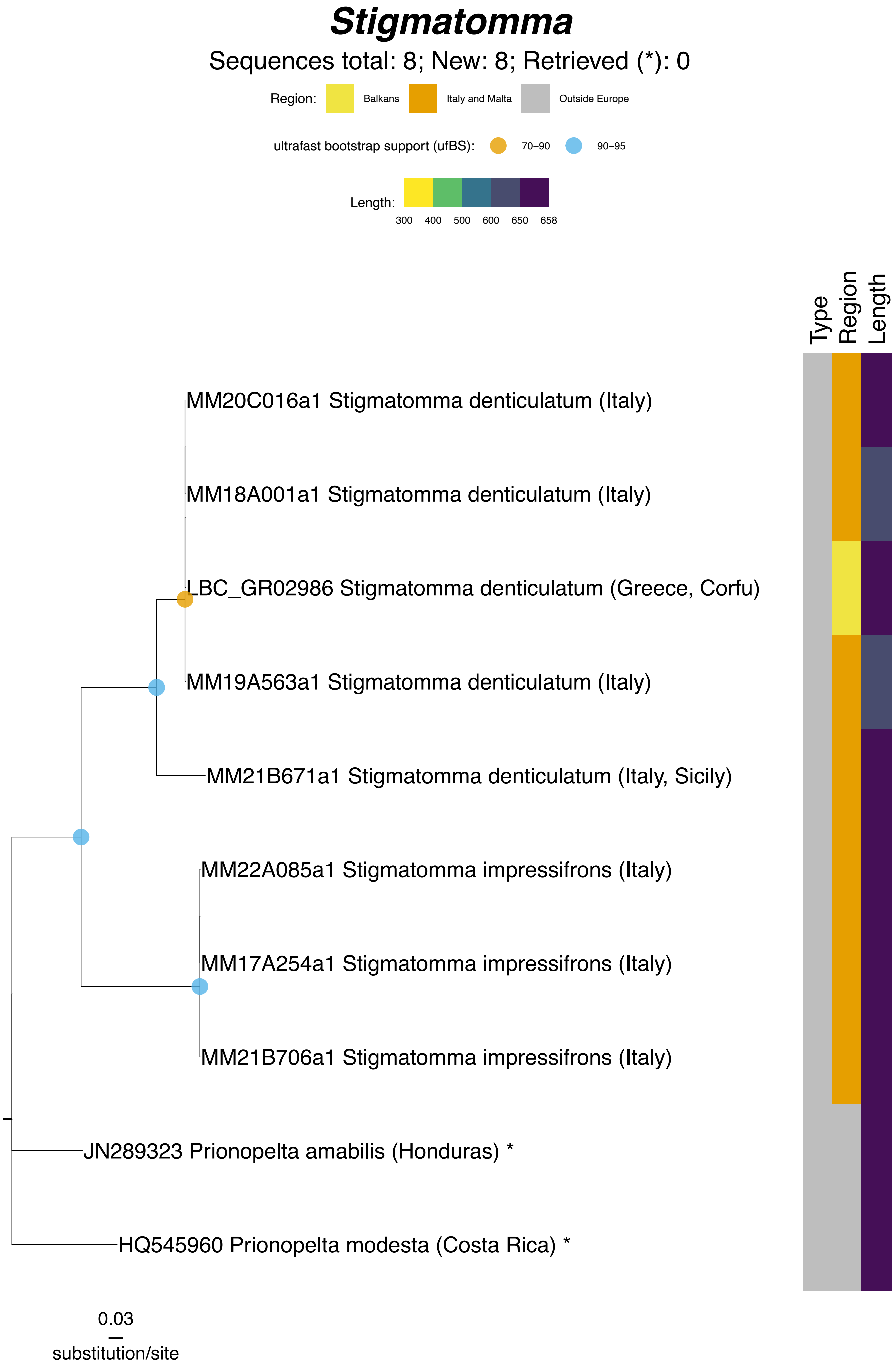
Stigmatomma
Sequences total: 8; New: 8; Retrieved (*): 0
Region:
Balkans
Italy and Malta
Outside Europe
ultrafast bootstrap support (ufBS):
70–90
90–95
Length:
300
400
500
600
650
658
Type
Region
Length
MM20C016a1 Stigmatomma denticulatum (Italy)
MM18A001a1 Stigmatomma denticulatum (Italy)
LBC_GR02986 Stigmatomma denticulatum (Greece, Corfu)
MM19A563a1 Stigmatomma denticulatum (Italy)
MM21B671a1 Stigmatomma denticulatum (Italy, Sicily)
MM22A085a1 Stigmatomma impressifrons (Italy)
MM17A254a1 Stigmatomma impressifrons (Italy)
MM21B706a1 Stigmatomma impressifrons (Italy)
JN289323 Prionopelta amabilis (Honduras) *
HQ545960 Prionopelta modesta (Costa Rica) *
0.03
substitution/site

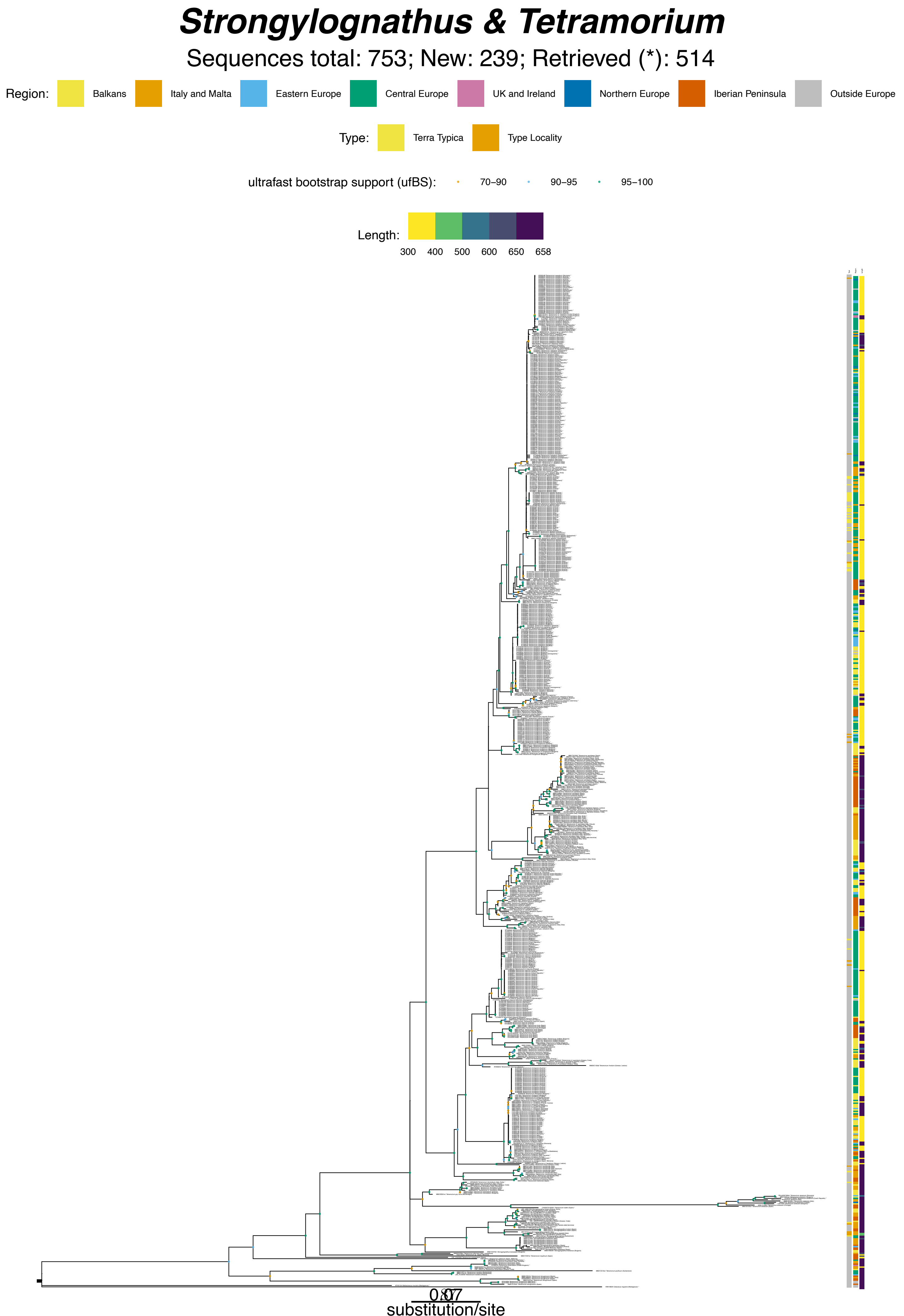

Strongylognathus & Tetramorium
Sequences total: 753; New: 239; Retrieved (*): 514
Region:
Balkans
Italy and Malta
Eastern Europe
Central Europe
UK and Ireland
Northern Europe
Iberian Peninsula
Outside Europe
Type:
Terra Typica
Type Locality
ultrafast bootstrap support (ufBS):
70–90
90–95
95–100
Length:
300
400
500
600
650
658
substitution/site

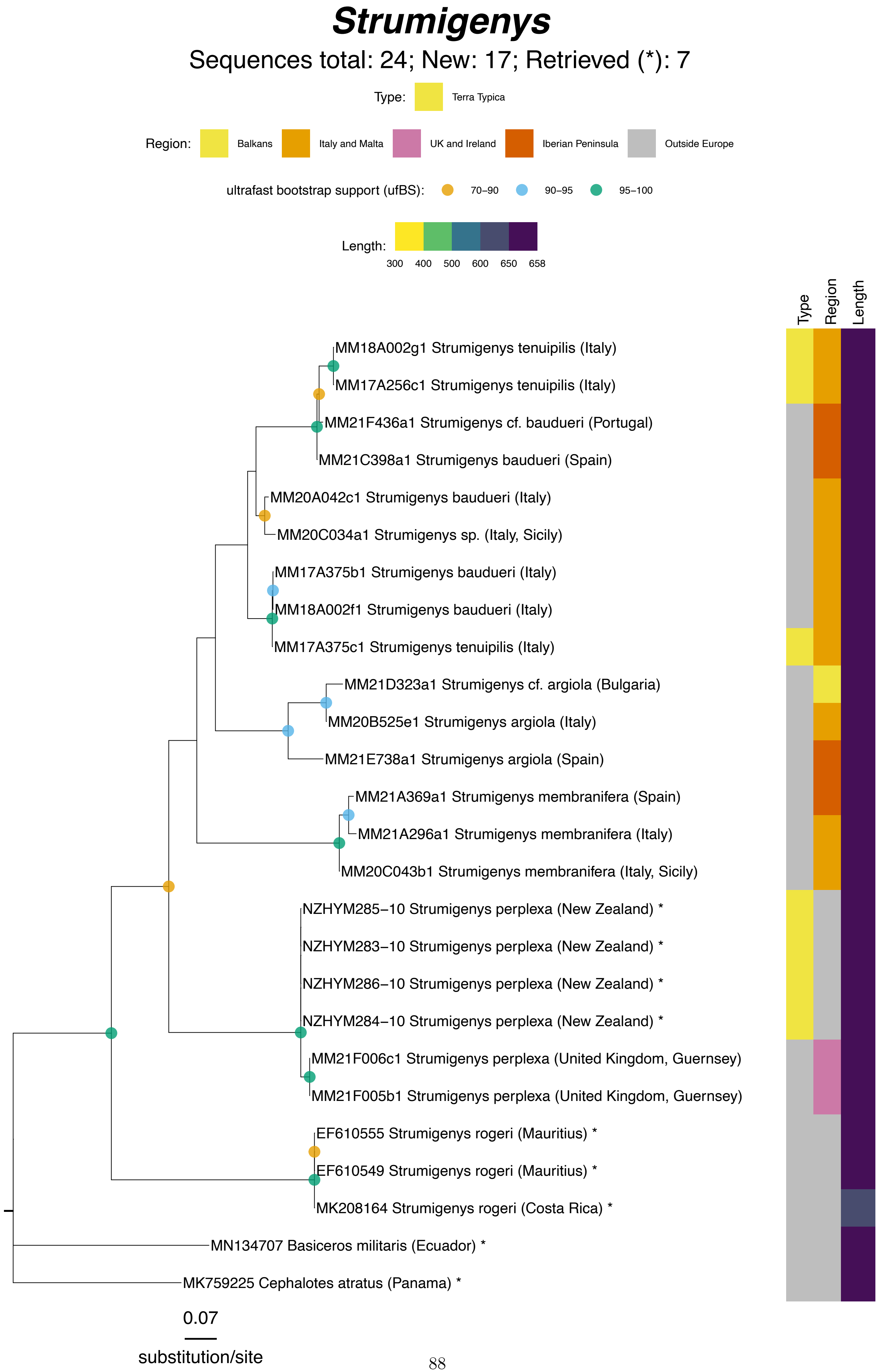

Strumigenys
Sequences total: 24; New: 17; Retrieved (*): 7
Type:
Terra Typica
Region:
Balkans
Italy and Malta
UK and Ireland
Iberian Peninsula
Outside Europe
ultrafast bootstrap support (ufBS):
70–90
90–95
95–100
Length:
300
400
500
600
650
658
Type
Region
Length
MM18A002g1 Strumigenys tenuipilis (Italy)
MM17A256c1 Strumigenys tenuipilis (Italy)
MM21F436a1 Strumigenys cf. baudueri (Portugal)
MM21C398a1 Strumigenys baudueri (Spain)
MM20A042c1 Strumigenys baudueri (Italy)
MM20C034a1 Strumigenys sp. (Italy, Sicily)
MM17A375b1 Strumigenys baudueri (Italy)
MM18A002f1 Strumigenys baudueri (Italy)
MM17A375c1 Strumigenys tenuipilis (Italy)
MM21D323a1 Strumigenys cf. argiola (Bulgaria)
MM20B525e1 Strumigenys argiola (Italy)
MM21E738a1 Strumigenys argiola (Spain)
MM21A369a1 Strumigenys membranifera (Spain)
MM21A296a1 Strumigenys membranifera (Italy)
MM20C043b1 Strumigenys membranifera (Italy, Sicily)
NZHYM285–10 Strumigenys perplexa (New Zealand) *
NZHYM283–10 Strumigenys perplexa (New Zealand) *
NZHYM286–10 Strumigenys perplexa (New Zealand) *
NZHYM284–10 Strumigenys perplexa (New Zealand) *
MM21F006c1 Strumigenys perplexa (United Kingdom, Guernsey)
MM21F005b1 Strumigenys perplexa (United Kingdom, Guernsey)
EF610555 Strumigenys rogeri (Mauritius) *
EF610549 Strumigenys rogeri (Mauritius) *
MK208164 Strumigenys rogeri (Costa Rica) *
MN134707 Basiceros militaris (Ecuador) *
MK759225 Cephalotes atratus (Panama) *
0.07
substitution/site

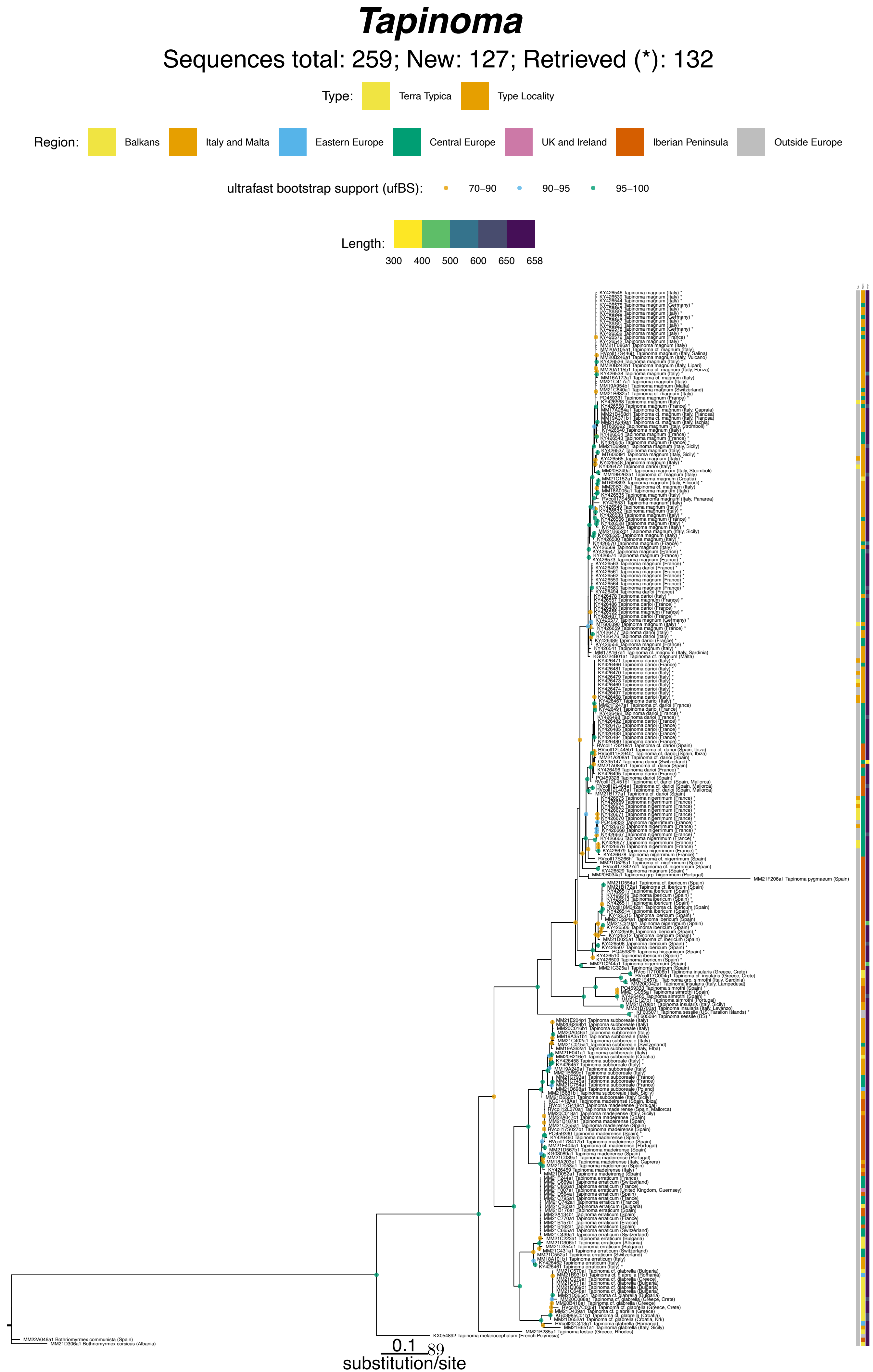
Tapinoma
Sequences total: 259; New: 127; Retrieved (*): 132
Type: Terra Typica, Type Locality
Region: Balkans, Italy and Malta, Eastern Europe, Central Europe, UK and Ireland, Iberian Peninsula, Outside Europe
ultrafast bootstrap support (ufBS): 70−90, 90−95, 95−100
Length: 300, 400, 500, 600, 650, 658
0.1
substitution/site

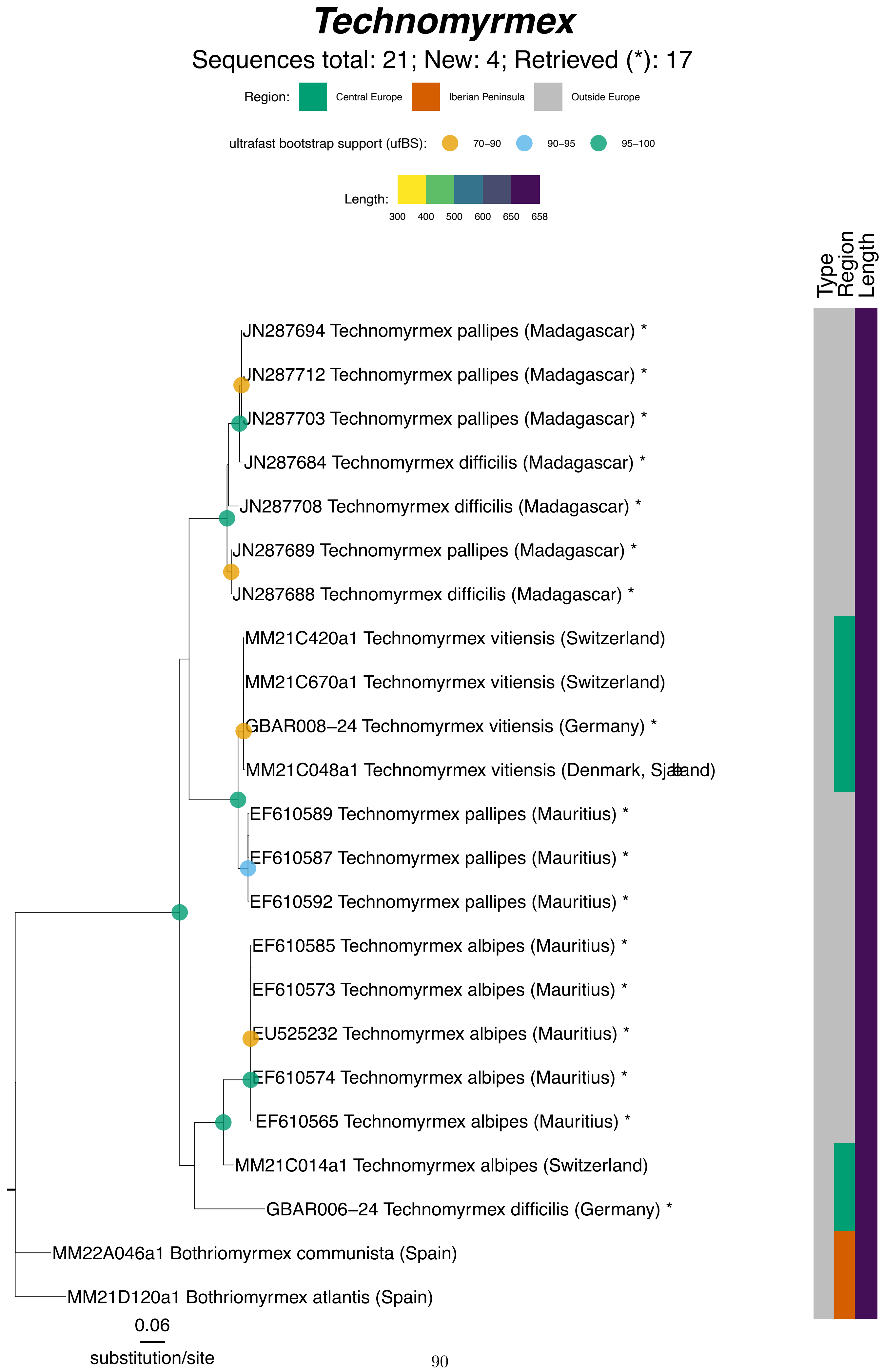

Technomyrmex
Sequences total: 21; New: 4; Retrieved (*): 17
Region: Central Europe Iberian Peninsula Outside Europe
ultrafast bootstrap support (ufBS): 70–90 90–95 95–100
Length: 300 400 500 600 650 658
Type
Region
Length
JN287694 Technomyrmex pallipes (Madagascar) *
JN287712 Technomyrmex pallipes (Madagascar) *
JN287703 Technomyrmex pallipes (Madagascar) *
JN287684 Technomyrmex difficilis (Madagascar) *
JN287708 Technomyrmex difficilis (Madagascar) *
JN287689 Technomyrmex pallipes (Madagascar) *
JN287688 Technomyrmex difficilis (Madagascar) *
MM21C420a1 Technomyrmex vitiensis (Switzerland)
MM21C670a1 Technomyrmex vitiensis (Switzerland)
GBAR008–24 Technomyrmex vitiensis (Germany) *
MM21C048a1 Technomyrmex vitiensis (Denmark, Sjælland)
EF610589 Technomyrmex pallipes (Mauritius) *
EF610587 Technomyrmex pallipes (Mauritius) *
EF610592 Technomyrmex pallipes (Mauritius) *
EF610585 Technomyrmex albipes (Mauritius) *
EF610573 Technomyrmex albipes (Mauritius) *
EU525232 Technomyrmex albipes (Mauritius) *
EF610574 Technomyrmex albipes (Mauritius) *
EF610565 Technomyrmex albipes (Mauritius) *
MM21C014a1 Technomyrmex albipes (Switzerland)
GBAR006–24 Technomyrmex difficilis (Germany) *
MM22A046a1 Bothriomyrmex communista (Spain)
MM21D120a1 Bothriomyrmex atlantis (Spain)
0.06
substitution/site

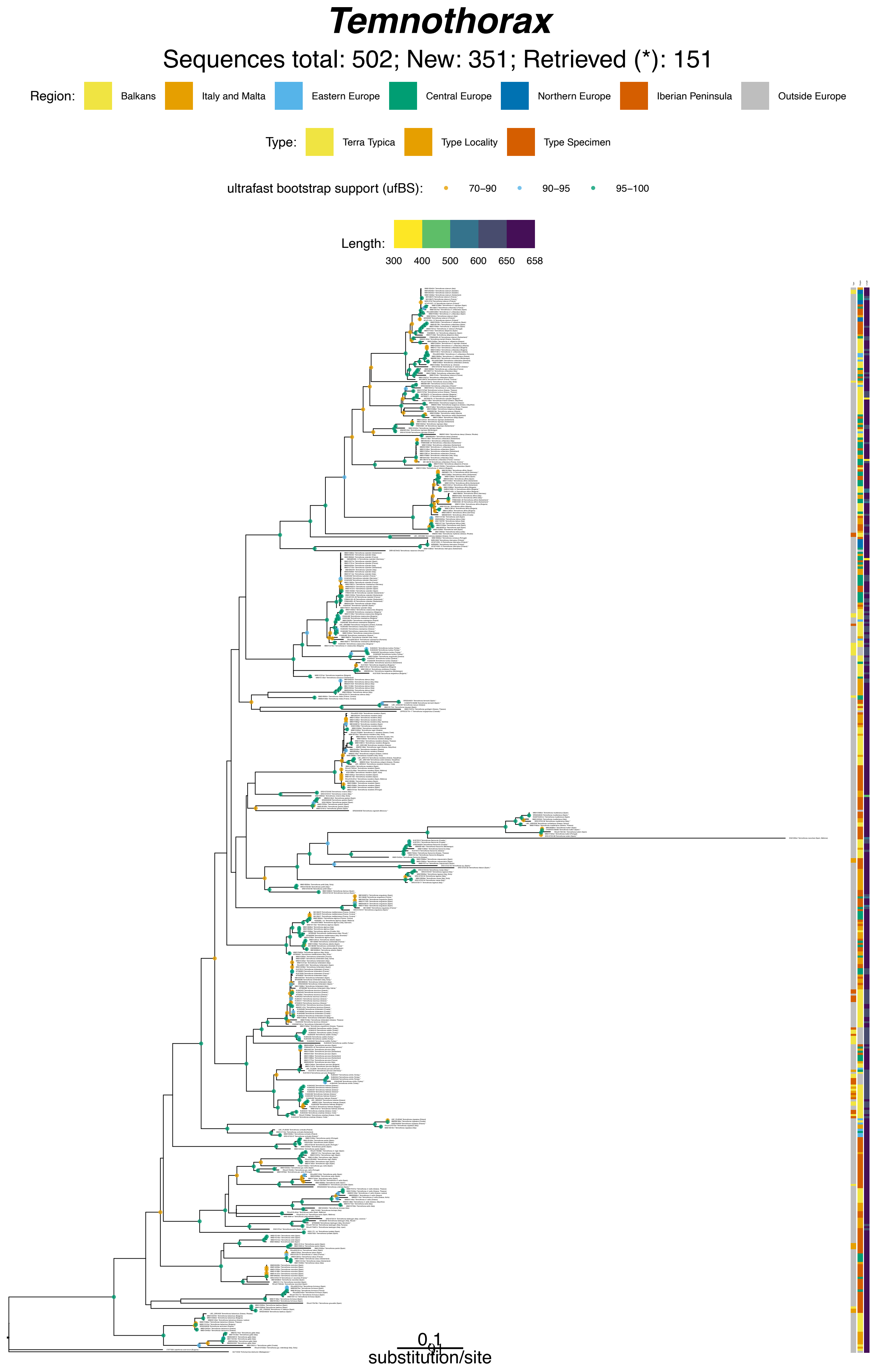

Temnothorax
Sequences total: 502; New: 351; Retrieved (*): 151
Region:
Balkans
Italy and Malta
Eastern Europe
Central Europe
Northern Europe
Iberian Peninsula
Outside Europe
Type:
Terra Typica
Type Locality
Type Specimen
ultrafast bootstrap support (ufBS):
70–90
90–95
95–100
Length:
300
400
500
600
650
658
0.1
substitution/site

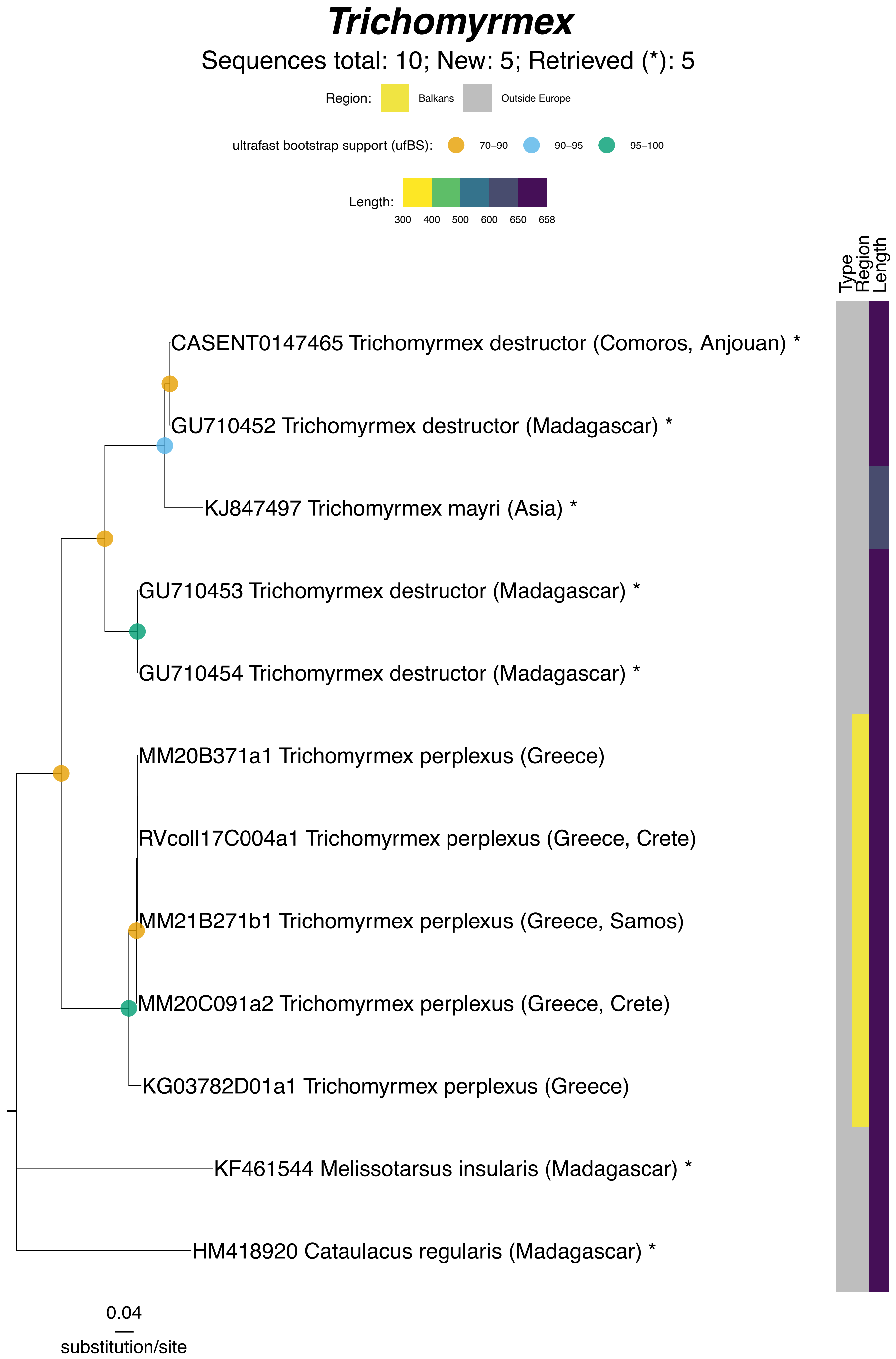

Trichomyrmex
Sequences total: 10; New: 5; Retrieved (*): 5
Region:
Balkans
Outside Europe
ultrafast bootstrap support (ufBS):
70–90
90–95
95–100
Length:
300
400
500
600
650
658
Type
Region
Length
CASENT0147465 Trichomyrmex destructor (Comoros, Anjouan) *
GU710452 Trichomyrmex destructor (Madagascar) *
KJ847497 Trichomyrmex mayri (Asia) *
GU710453 Trichomyrmex destructor (Madagascar) *
GU710454 Trichomyrmex destructor (Madagascar) *
MM20B371a1 Trichomyrmex perplexus (Greece)
RVcoll17C004a1 Trichomyrmex perplexus (Greece, Crete)
MM21B271b1 Trichomyrmex perplexus (Greece, Samos)
MM20C091a2 Trichomyrmex perplexus (Greece, Crete)
KG03782D01a1 Trichomyrmex perplexus (Greece)
KF461544 Melissotarsus insularis (Madagascar) *
HM418920 Cataulacus regularis (Madagascar) *
0.04
substitution/site

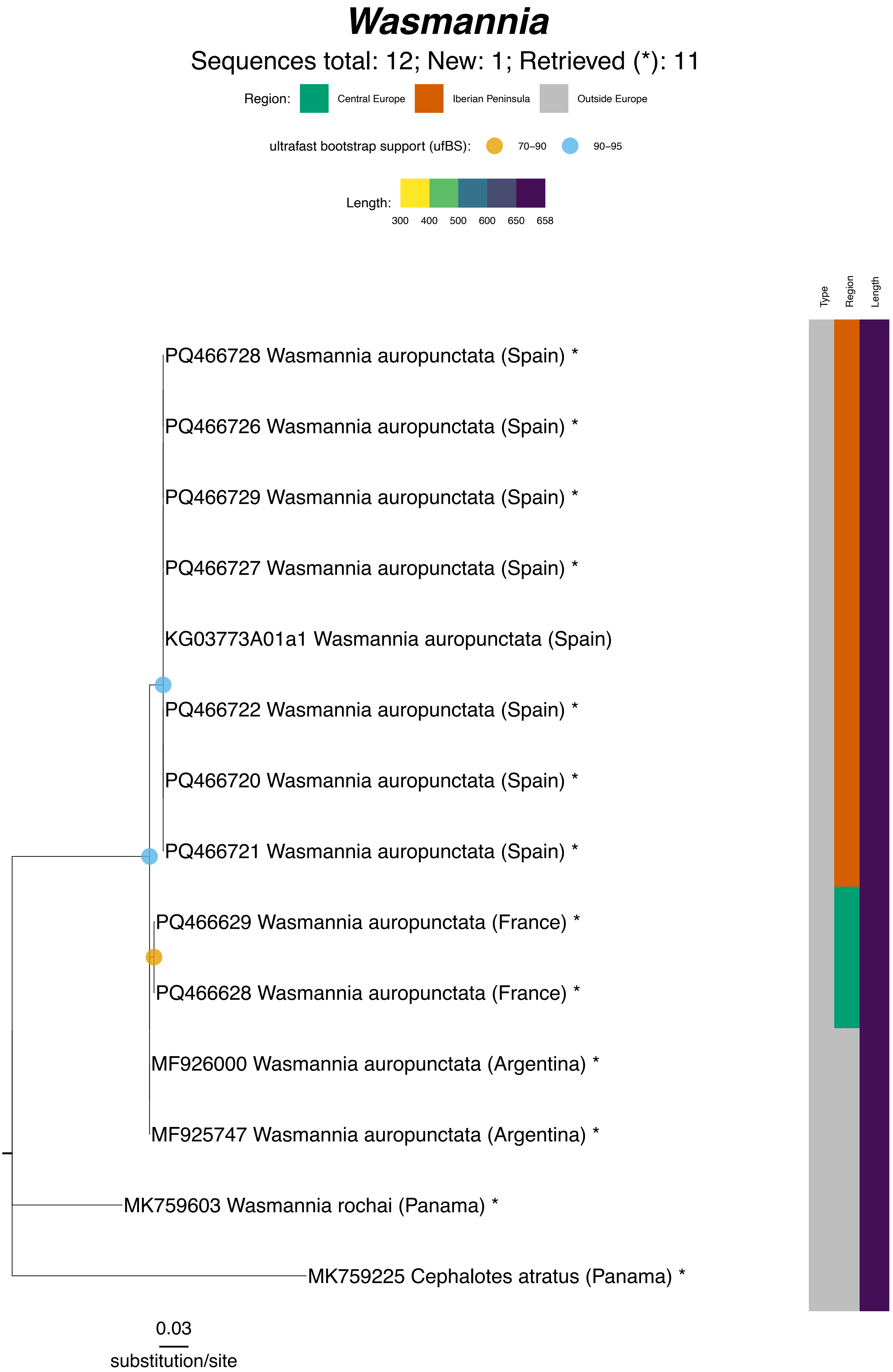
Wasmannia
Sequences total: 12; New: 1; Retrieved (*): 11
Region:
Central Europe
Iberian Peninsula
Outside Europe
ultrafast bootstrap support (ufBS):
70–90
90–95
Length:
300
400
500
600
650
658
Type
Region
Length
PQ466728 Wasmannia auropunctata (Spain) *
PQ466726 Wasmannia auropunctata (Spain) *
PQ466729 Wasmannia auropunctata (Spain) *
PQ466727 Wasmannia auropunctata (Spain) *
KG03773A01a1 Wasmannia auropunctata (Spain)
PQ466722 Wasmannia auropunctata (Spain) *
PQ466720 Wasmannia auropunctata (Spain) *
PQ466721 Wasmannia auropunctata (Spain) *
PQ466629 Wasmannia auropunctata (France) *
PQ466628 Wasmannia auropunctata (France) *
MF926000 Wasmannia auropunctata (Argentina) *
MF925747 Wasmannia auropunctata (Argentina) *
MK759603 Wasmannia rochai (Panama) *
MK759225 Cephalotes atratus (Panama) *
0.03
substitution/site

The mitochondrial genetic diversity maps

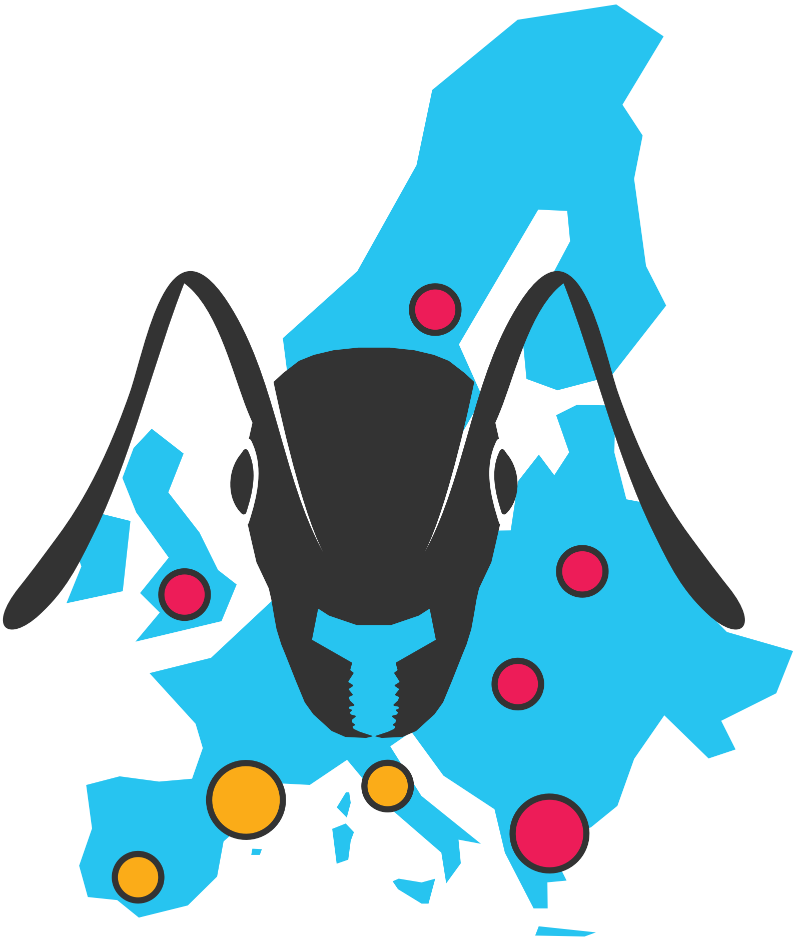

Acropyga

Acropyga paleartica

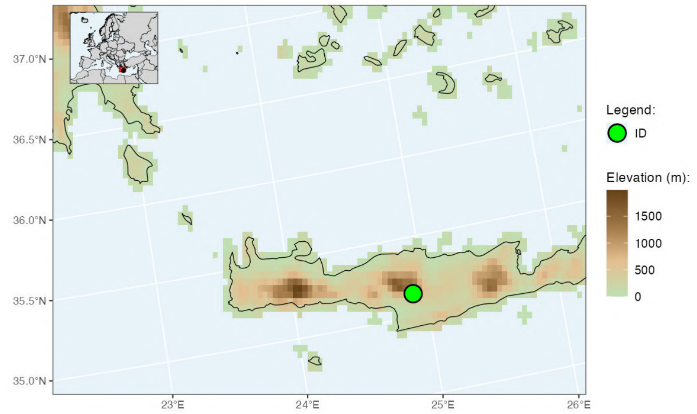

Figure 5: Map of *Acropyga paleartica* Menozzi, 1936. Due to the presence of a single sequence, the genetic diversity map and the PCoA projection were not done. Specimen identification (ID or cf.) and source (newly sequenced or retrieved) are represented by colours, while specimen attribute (terra typica, type locality, type specimen or faunistic novelty) is represented by the shape. Sequences: ID = 1, cf. = 0; maximum p-distance: strict = NA, less strict = NA.

Haplotype network analysis of *Acropyga paleartica* was not possible.

Aenictus

Aenictus rhodiensis

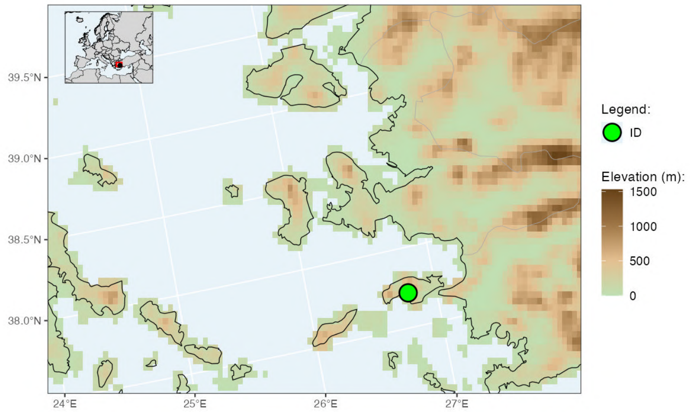

Figure 6: Map of *Aenictus rhodiensis* Menozzi, 1936. Due to the presence of a single sequence, the genetic diversity map and the PCoA projection were not done. Specimen identification (ID or cf.) and source (newly sequenced or retrieved) are represented by colours, while specimen attribute (terra typica, type locality, type specimen or faunistic novelty) is represented by the shape. Sequences: ID = 1, cf. = 0; maximum p-distance: strict = NA, less strict = NA.

Haplotype network analysis of *Aenictus rhodiensis* was not possible.

Anochetus

Anochetus ghilianii

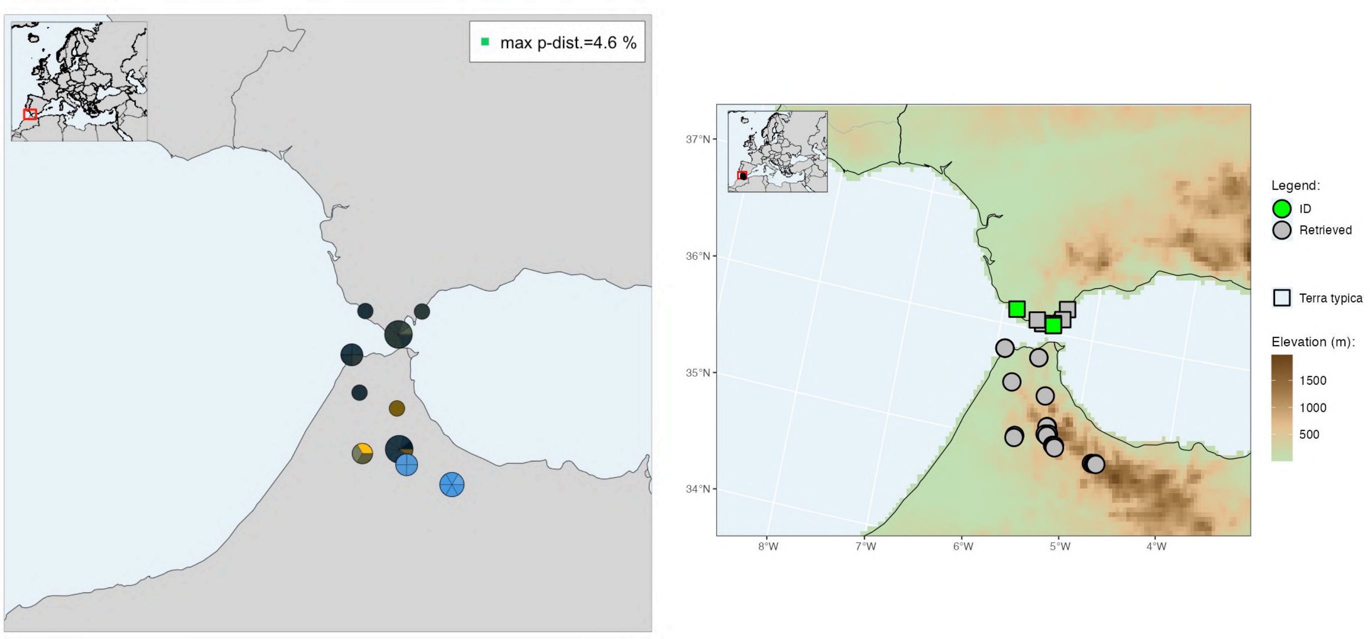

Figure 7: Genetic diversity map of *Anochetus ghilianii* (Spinola, 1851). Nearby localities of sequenced specimens are merged in pies (left). Colours match the bidimensional colour space of the PCoA projection (Fig. 7 left) of p-dist between sequences (dots). Specimen identification (ID or cf.) and source (newly sequenced or retrieved) are represented by colours, while specimen attribute (terra typica, type locality, type specimen or faunistic novelty) is represented by the shape (right). Sequences: ID = 41, cf. = 0; maximum p-distance: strict = 4.6 %, less strict = 4.6 %.

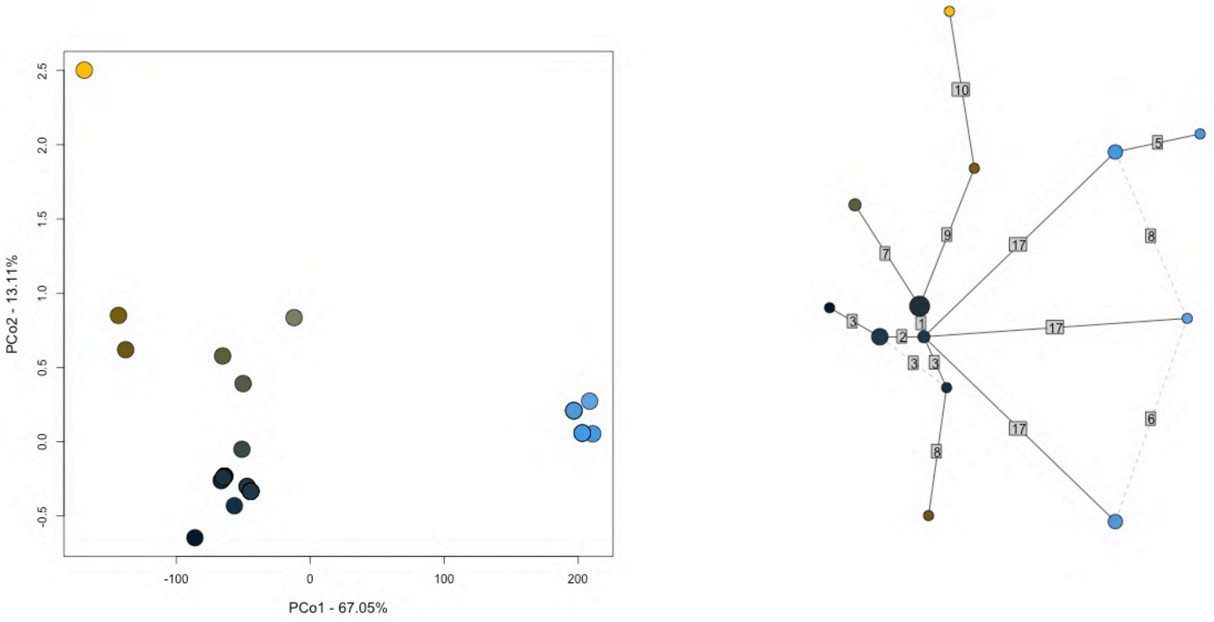

Figure 8: PCoA based on pairwise p-distances between *Anochetus ghilianii* sequences (left). Colours match a bidimensional colour space. Haplotype network of *Anochetus ghilianii* (right). Sequences > 599 bp: ID = 41, cf. = 0.

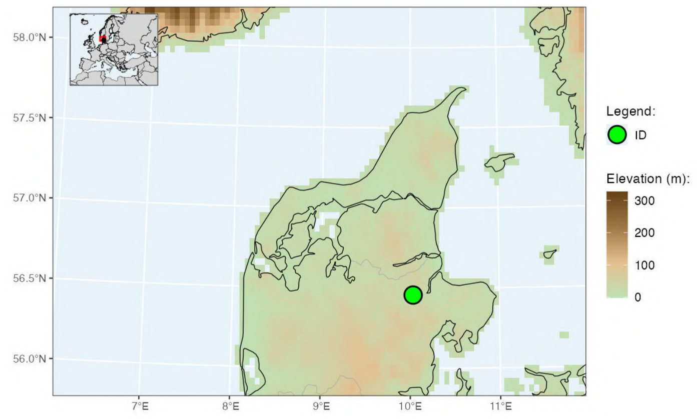

Figure 9: Map of *Anochetus mayri* Emery, 1884. Due to the presence of a single sequence, the genetic diversity map and the PCoA projection were not done. Specimen identification (ID or cf.) and source (newly sequenced or retrieved) are represented by colours, while specimen attribute (terra typica, type locality, type specimen or faunistic novelty) is represented by the shape. Sequences: ID = 2, cf. = 0; maximum p-distance: strict = NA, less strict = NA.

Haplotype network analysis of *Anochetus mayri* was not possible.

Aphaenogaster

Aphaenogaster aktaci

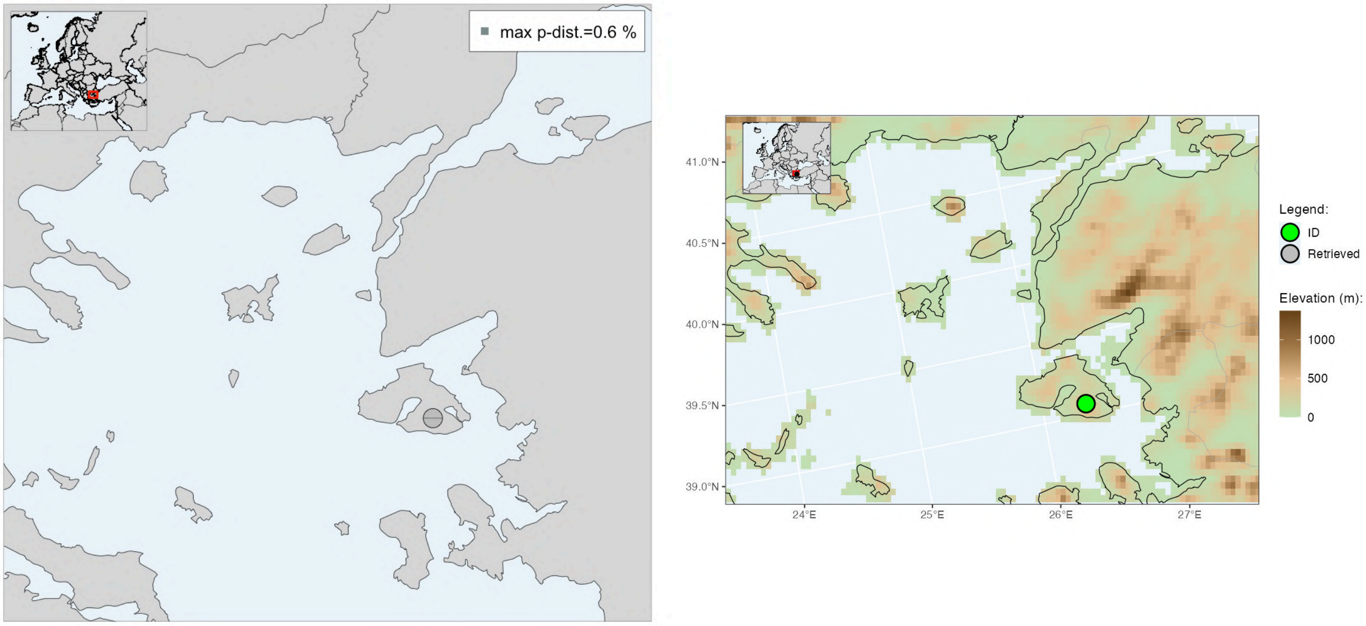

Figure 10: Genetic diversity map of *Aphaenogaster aktaci* Kiran & Tezcan, 2008. PCoA projection was not done and therefore sequenced specimens in the genetic diversity map are coloured in gray (left). Specimen identification (ID or cf.) and source (newly sequenced or retrieved) are represented by colours, while specimen attribute (terra typica, type locality, type specimen or faunistic novelty) is represented by the shape (right). Sequences: ID = 2, cf. = 0; maximum p-distance: strict = NA, less strict = 0.6 %.

Haplotype network analysis of *Aphaenogaster aktaci* was not possible.

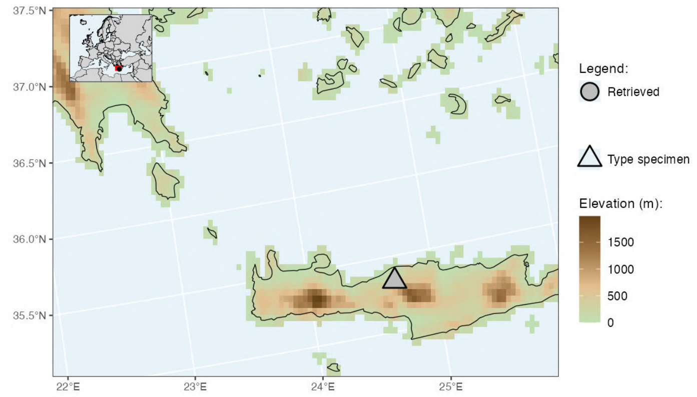

Figure 11: Map of *Aphaenogaster asterioni* Borowiec, Menchetti, Salata, Vila & Zięcina 2024. Due to the presence of a single sequence, the genetic diversity map and the PCoA projection were not done. Specimen identification (ID or cf.) and source (newly sequenced or retrieved) are represented by colours, while specimen attribute (terra typica, type locality, type specimen or faunistic novelty) is represented by the shape. Sequences: ID = 1, cf. = 0; maximum p-distance: strict = NA, less strict = NA.

Haplotype network analysis of *Aphaenogaster asterioni* was not possible.

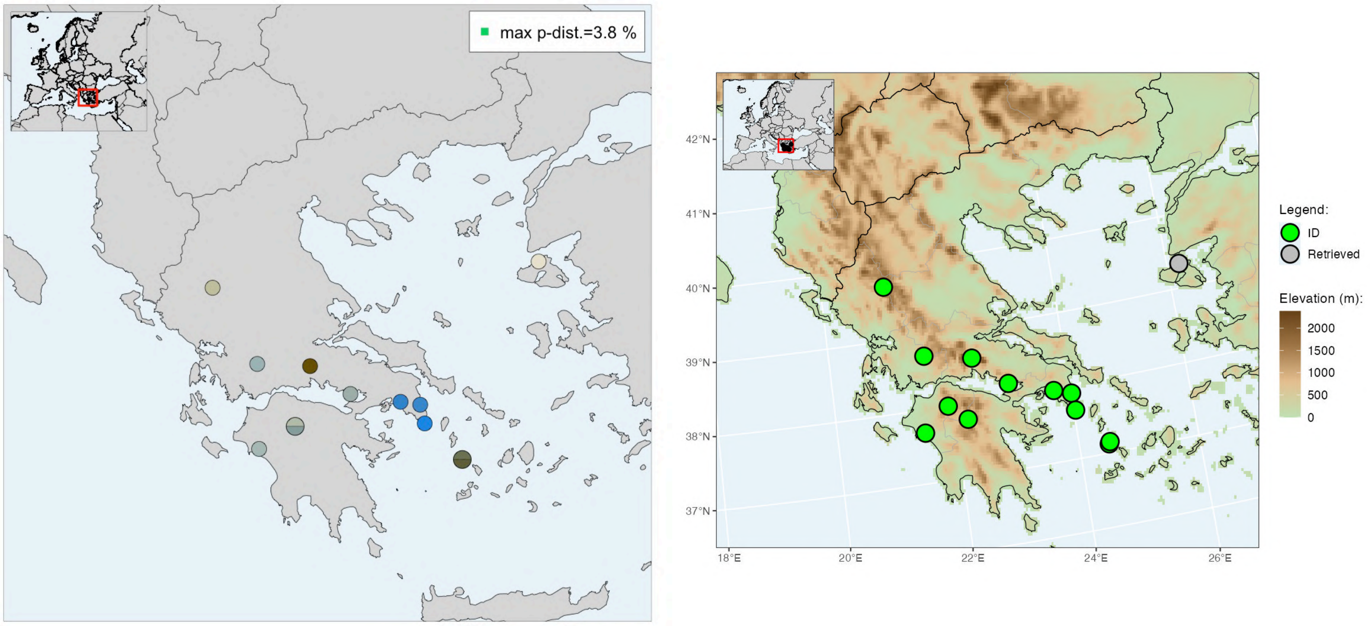

Figure 12: Genetic diversity map of *Aphaenogaster balcanica* (Emery, 1898). Nearby localities of sequenced specimens are merged in pies (left). Colours match the bidimensional colour space of the PCoA projection (Fig. 12 left) of p-dist between sequences (dots). Specimen identification (ID or cf.) and source (newly sequenced or retrieved) are represented by colours, while specimen attribute (terra typica, type locality, type specimen or faunistic novelty) is represented by the shape (right). Sequences: ID = 13, cf. = 0; maximum p-distance: strict = 3.8 %, less strict = 3.8 %.

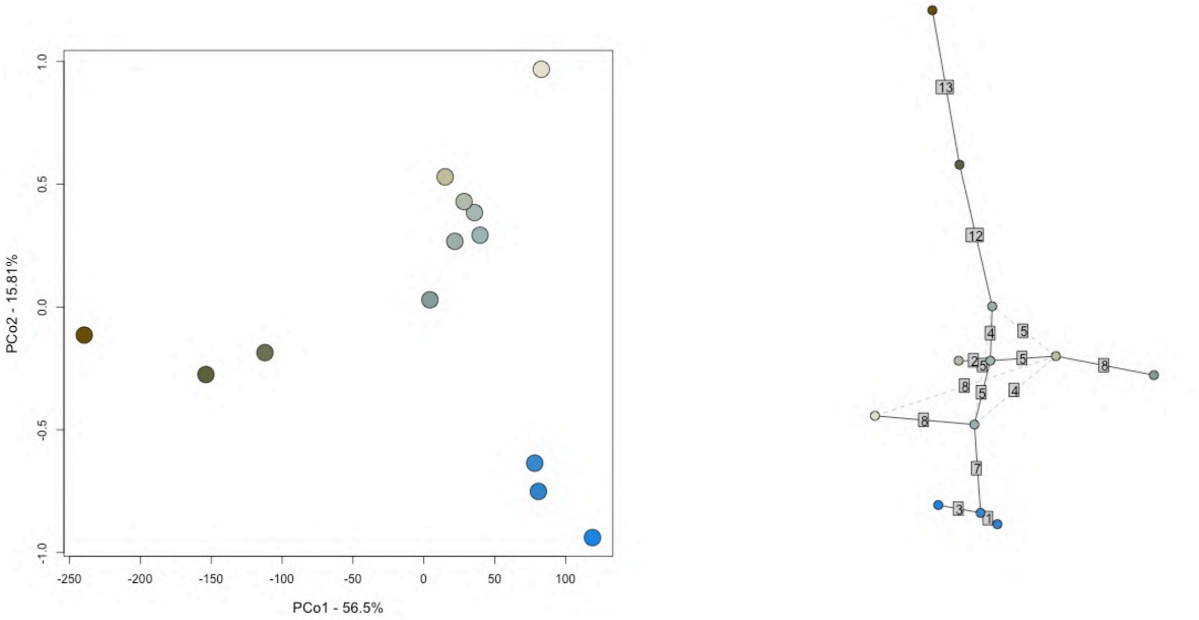

Figure 13: PCoA based on pairwise p-distances between *Aphaenogaster balcanica* sequences (left). Colours match a bidimensional colour space. Haplotype network of *Aphaenogaster balcanica* (right). Sequences > 599 bp: ID = 12, cf. = 0.

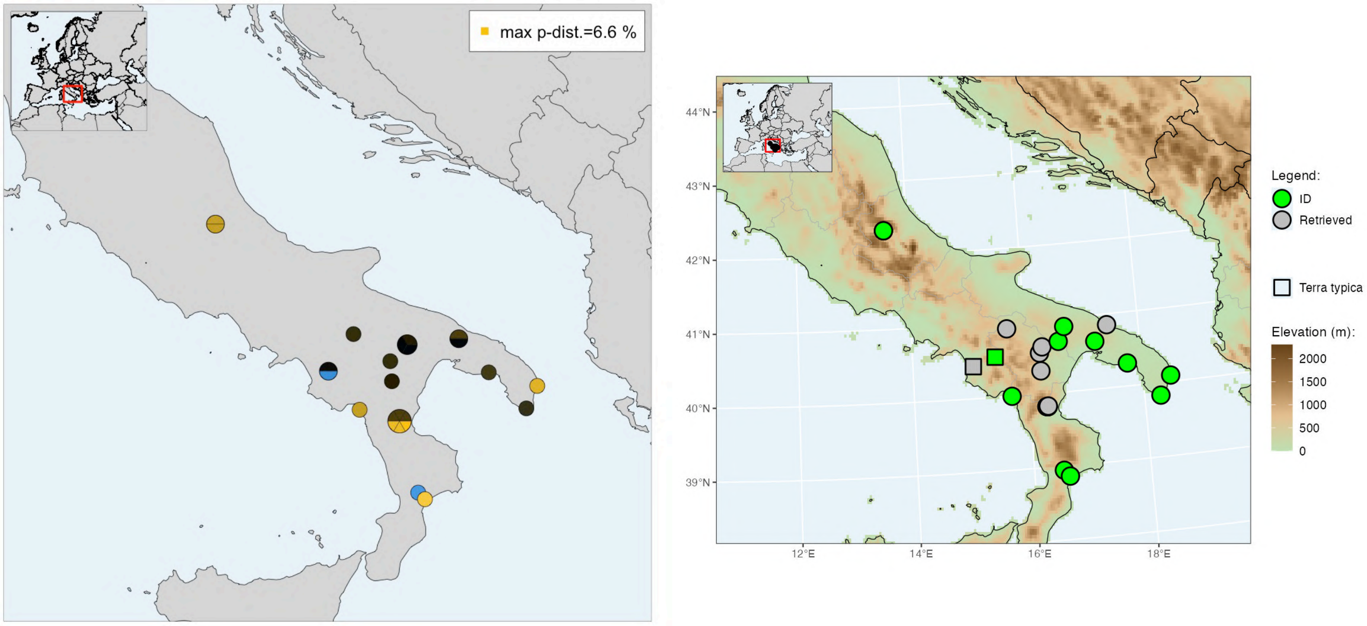

Figure 14: Genetic diversity map of *Aphaenogaster campana* Emery, 1878. Nearby localities of sequenced specimens are merged in pies (left). Colours match the bidimensional colour space of the PCoA projection (Fig. 14 left) of p-dist between sequences (dots). Specimen identification (ID or cf.) and source (newly sequenced or retrieved) are represented by colours, while specimen attribute (terra typica, type locality, type specimen or faunistic novelty) is represented by the shape (right). Sequences: ID = 24, cf. = 0; maximum p-distance: strict = 6.3 %, less strict = 6.6 %.

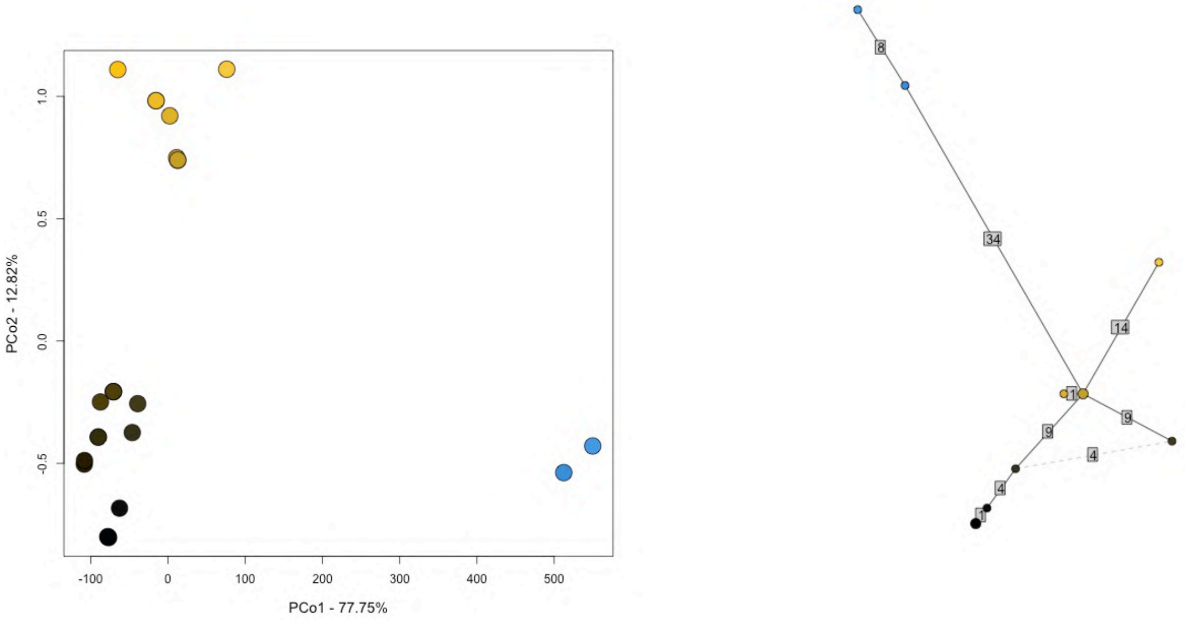

Figure 15: PCoA based on pairwise p-distances between *Aphaenogaster campana* sequences (left). Colours match a bidimensional colour space. Haplotype network of *Aphaenogaster campana* (right). Sequences > 599 bp: ID = 13, cf. = 0.

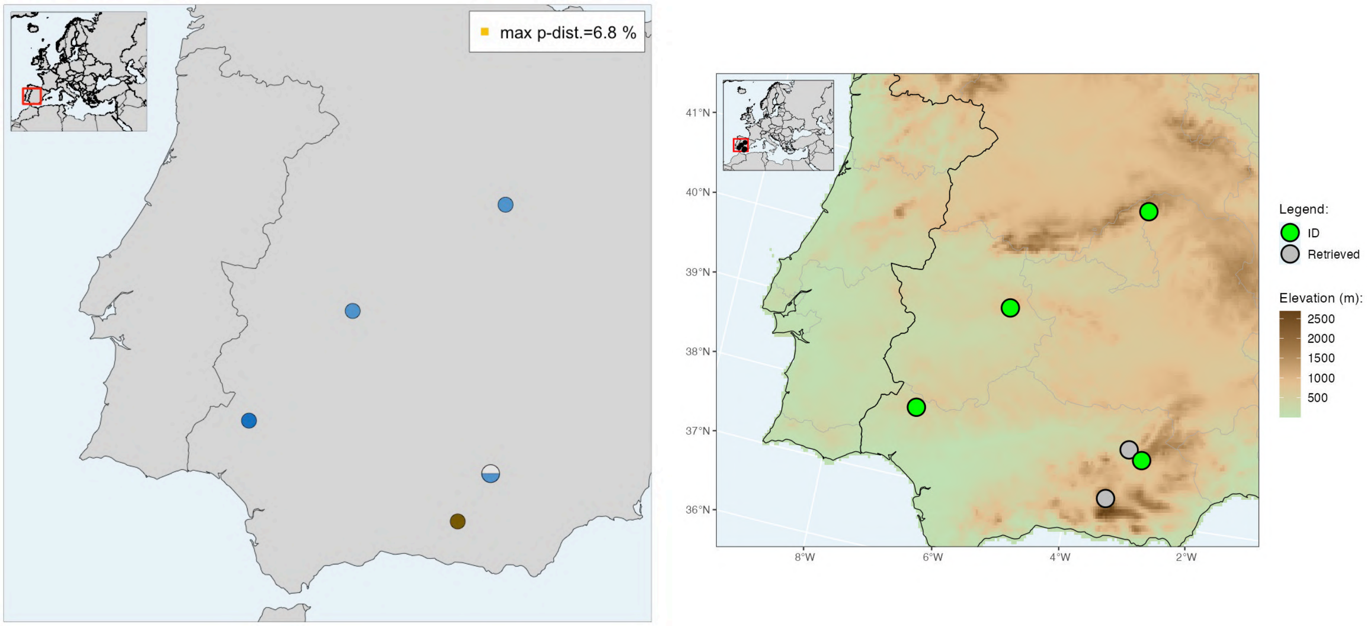

Figure 16: Genetic diversity map of *Aphaenogaster cardenai* Espadaler, 1981. Nearby localities of sequenced specimens are merged in pies (left). Colours match the bidimensional colour space of the PCoA projection (Fig. 16 left) of p-dist between sequences (dots). Specimen identification (ID or cf.) and source (newly sequenced or retrieved) are represented by colours, while specimen attribute (terra typica, type locality, type specimen or faunistic novelty) is represented by the shape (right). Sequences: ID = 6, cf. = 0; maximum p-distance: strict = 6.8 %, less strict = 6.8 %.

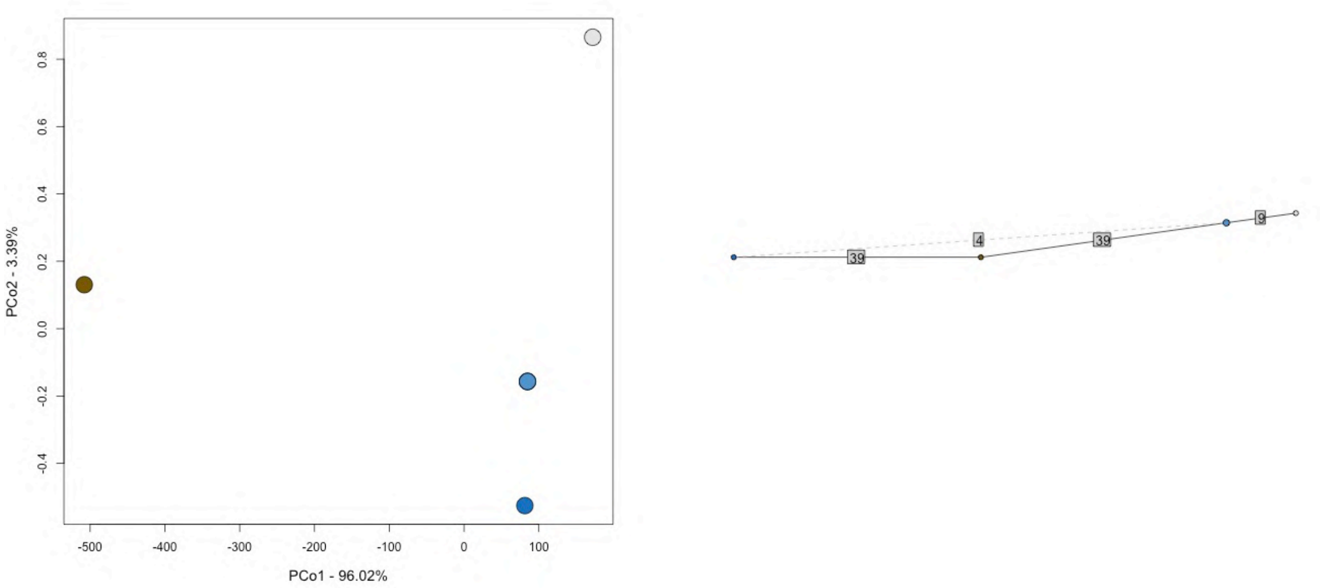

Figure 17: PCoA based on pairwise p-distances between *Aphaenogaster cardenai* sequences (left). Colours match a bidimensional colour space. Haplotype network of *Aphaenogaster cardenai* (right). Sequences > 599 bp: ID = 6, cf. = 0.

Aphaenogaster cecconii

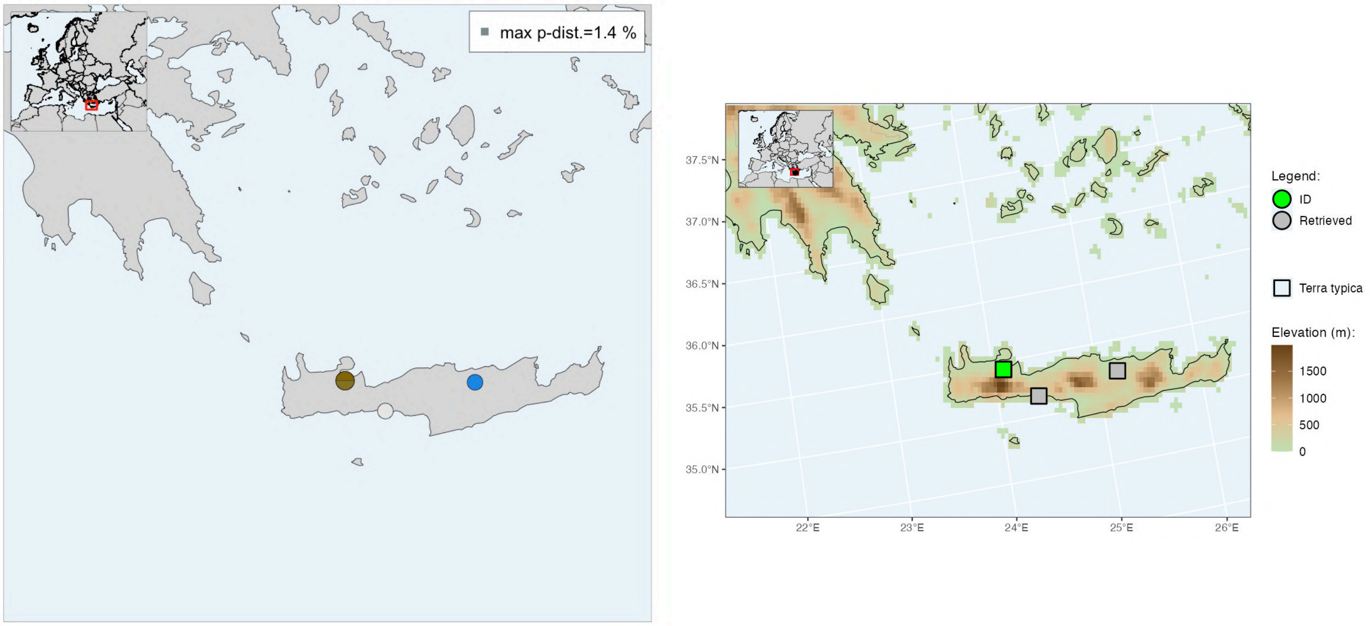

Figure 18: Genetic diversity map of *Aphaenogaster cecconii* Emery, 1894. Nearby localities of sequenced specimens are merged in pies (left). Colours match the bidimensional colour space of the PCoA projection (Fig. 18 left) of p-dist between sequences (dots). Specimen identification (ID or cf.) and source (newly sequenced or retrieved) are represented by colours, while specimen attribute (terra typica, type locality, type specimen or faunistic novelty) is represented by the shape (right). Sequences: ID = 4, cf. = 0; maximum p-distance: strict = 1.4 %, less strict = 1.4 %.

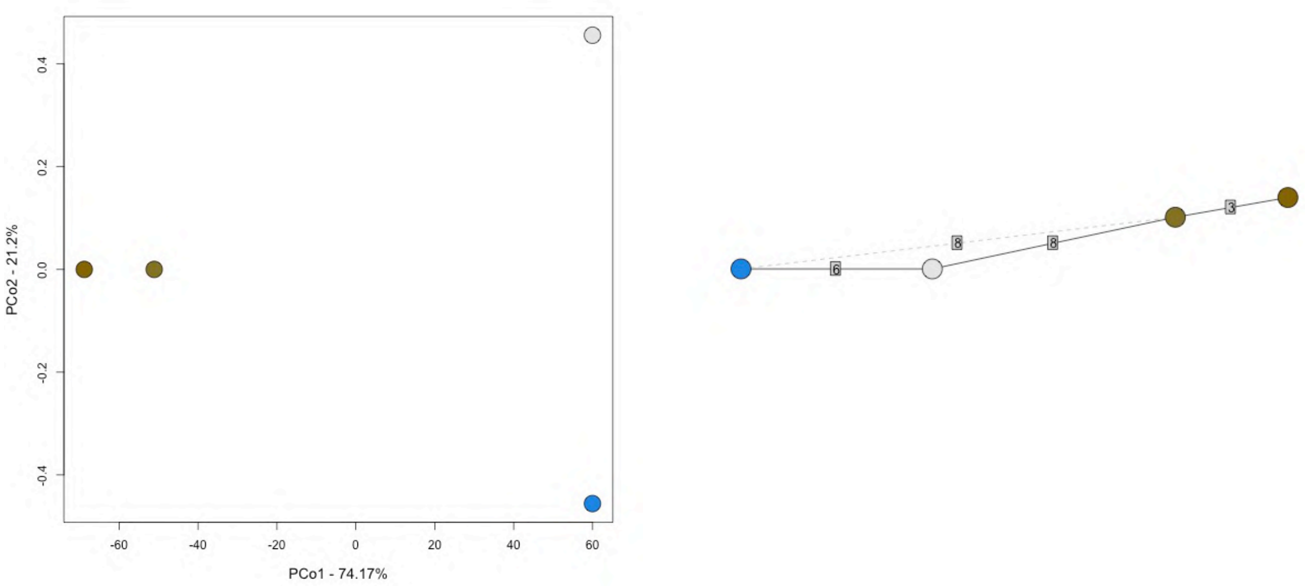

Figure 19: PCoA based on pairwise p-distances between *Aphaenogaster cecconii* sequences (left). Colours match a bidimensional colour space. Haplotype network of *Aphaenogaster cecconii* (right). Sequences > 599 bp: ID = 4, cf. = 0.

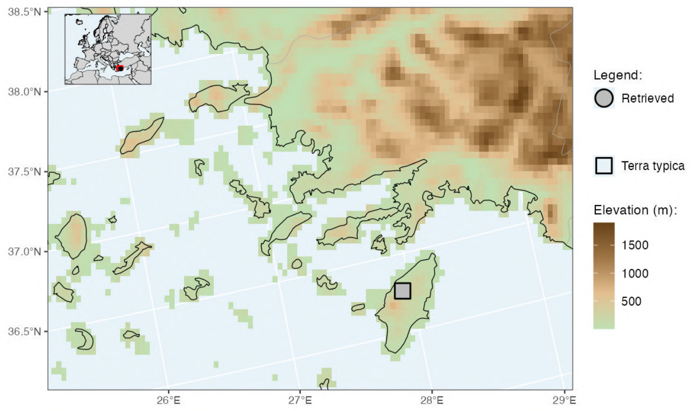

Figure 20: Map of *Aphaenogaster charesi* Salata & Borowiec, 2016. Due to the presence of a single sequence, the genetic diversity map and the PCoA projection were not done. Specimen identification (ID or cf.) and source (newly sequenced or retrieved) are represented by colours, while specimen attribute (terra typica, type locality, type specimen or faunistic novelty) is represented by the shape. Sequences: ID = 1, cf. = 0; maximum p-distance: strict = NA, less strict = NA.

Haplotype network analysis of *Aphaenogaster charesi* was not possible.

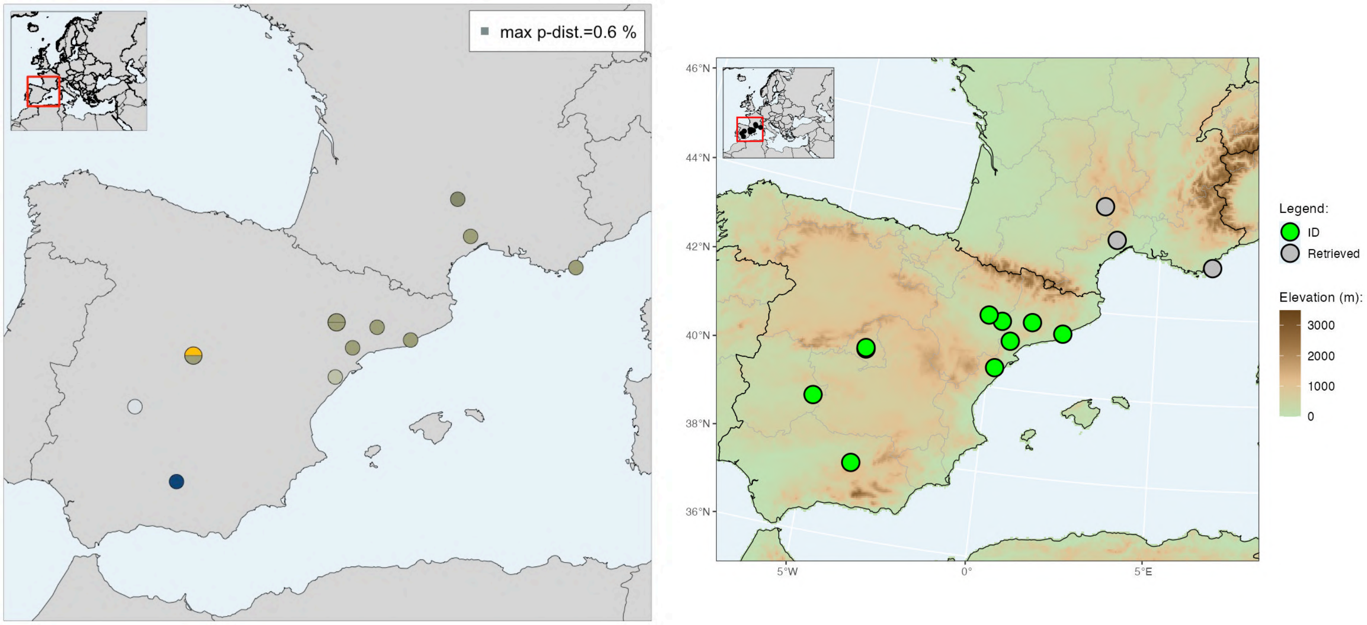

Figure 21: Genetic diversity map of *Aphaenogaster dulcineae* Emery, 1924. Nearby localities of sequenced specimens are merged in pies (left). Colours match the bidimensional colour space of the PCoA projection (Fig. 21 left) of p-dist between sequences (dots). Specimen identification (ID or cf.) and source (newly sequenced or retrieved) are represented by colours, while specimen attribute (terra typica, type locality, type specimen or faunistic novelty) is represented by the shape (right). Sequences: ID = 13, cf. = 0; maximum p-distance: strict = 0.6 %, less strict = 0.6 %.

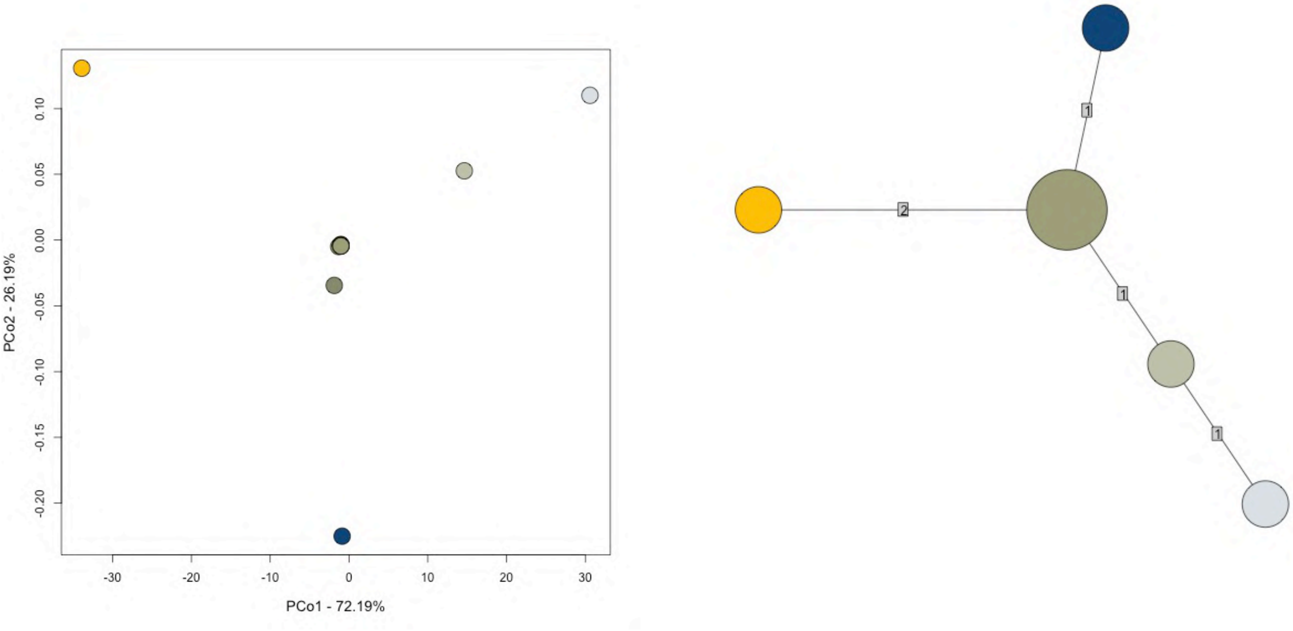

Figure 22: PCoA based on pairwise p-distances between *Aphaenogaster dulcineae* sequences (left). Colours match a bidimensional colour space. Haplotype network of *Aphaenogaster dulcineae* (right). Sequences > 599 bp: ID = 13, cf. = 0.

Aphaenogaster epirotes

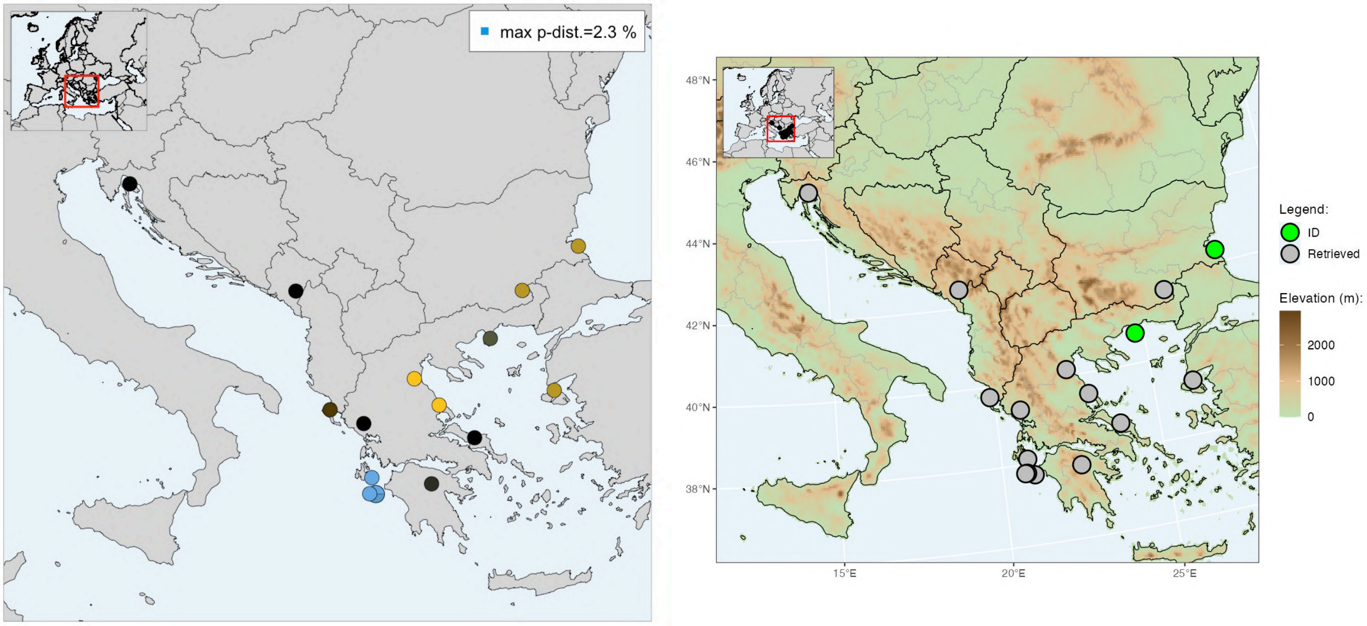

Figure 23: Genetic diversity map of *Aphaenogaster epirotes* (Emery, 1895). Nearby localities of sequenced specimens are merged in pies (left). Colours match the bidimensional colour space of the PCoA projection (Fig. 23 left) of p-dist between sequences (dots). Specimen identification (ID or cf.) and source (newly sequenced or retrieved) are represented by colours, while specimen attribute (terra typica, type locality, type specimen or faunistic novelty) is represented by the shape (right). Sequences: ID = 16, cf. = 0; maximum p-distance: strict = 2.3 %, less strict = 2.3 %.

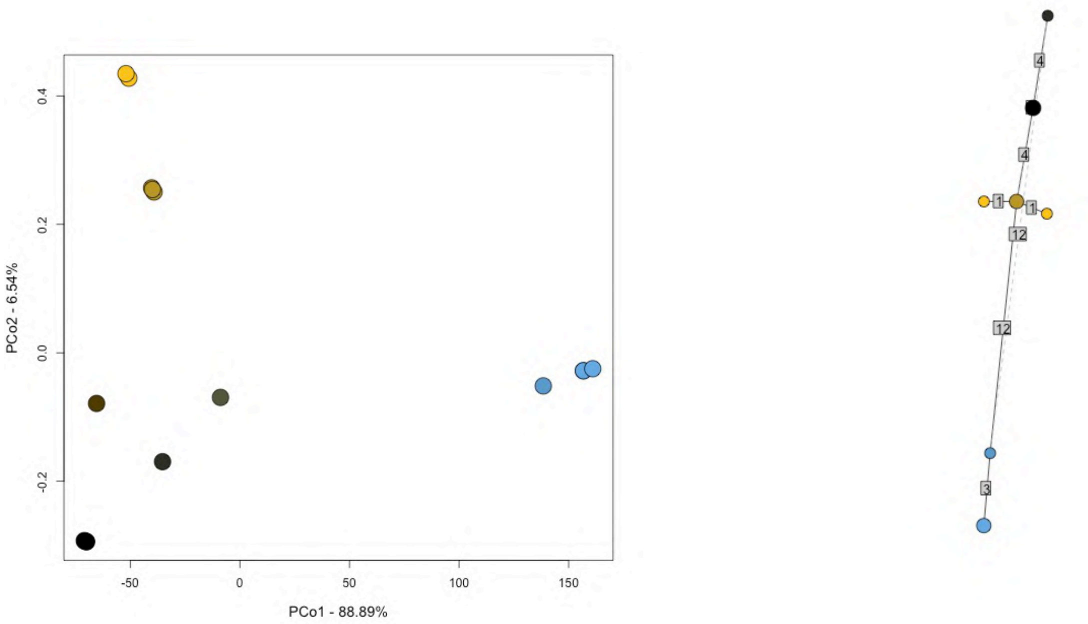

Figure 24: PCoA based on pairwise p-distances between *Aphaenogaster epirotes* sequences (left). Colours match a bidimensional colour space. Haplotype network of *Aphaenogaster epirotes* (right). Sequences > 599 bp: ID = 14, cf. = 0.

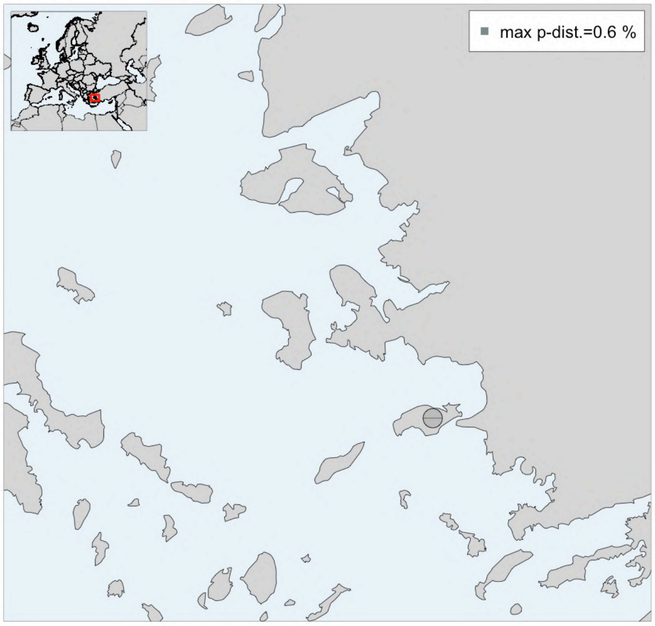

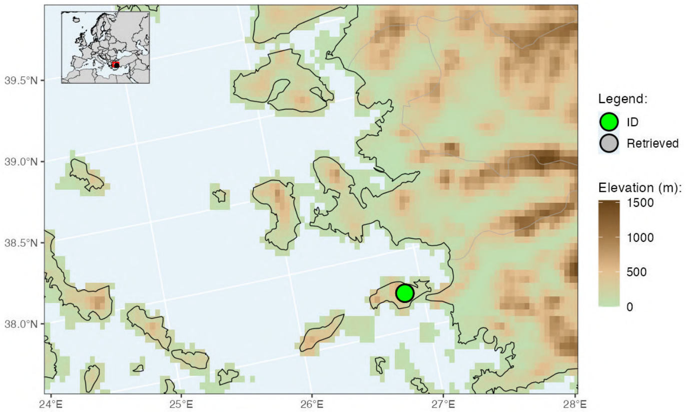

Figure 25: Genetic diversity map of *Aphaenogaster festae* Emery, 1915. PCoA projection was not done and therefore sequenced specimens in the genetic diversity map are coloured in gray (left). Specimen identification (ID or cf.) and source (newly sequenced or retrieved) are represented by colours, while specimen attribute (terra typica, type locality, type specimen or faunistic novelty) is represented by the shape (right). Sequences: ID = 2, cf. = 0; maximum p-distance: strict = NA, less strict = 0.6 %.

Haplotype network analysis of *Aphaenogaster festae* was not possible.

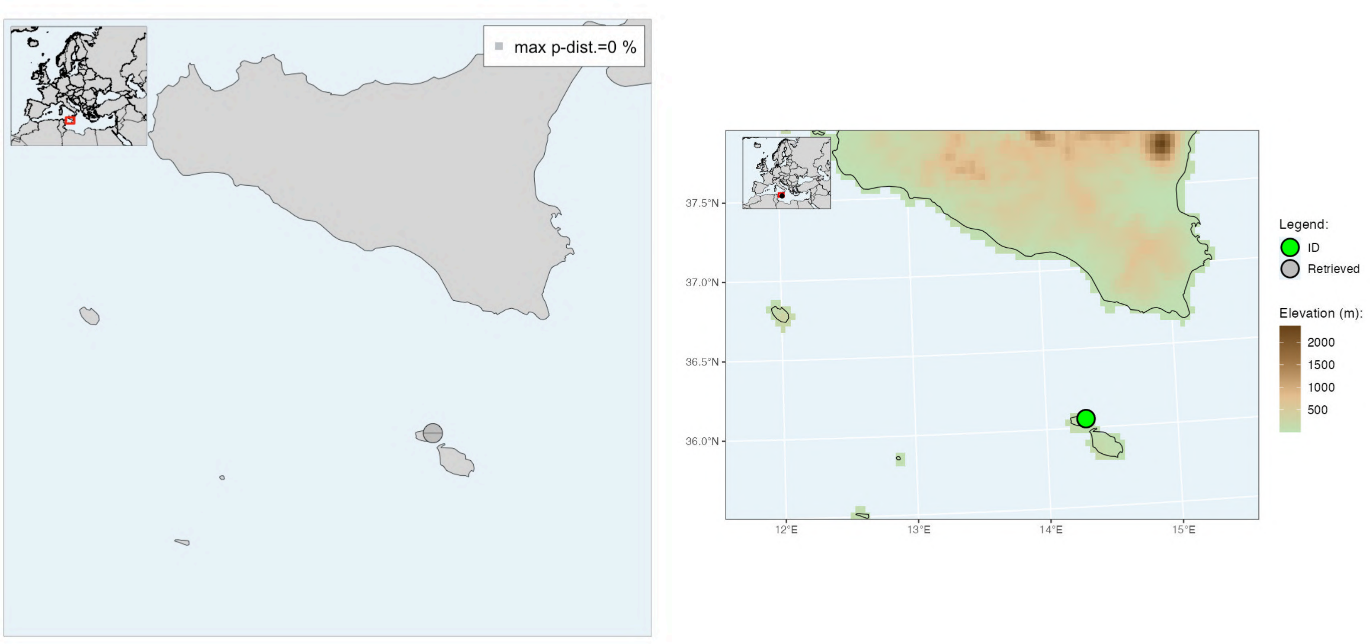

Figure 26: Genetic diversity map of *Aphaenogaster fiorii* Emery, 1915. PCoA projection was not done and therefore sequenced specimens in the genetic diversity map are coloured in gray (left). Specimen identification (ID or cf.) and source (newly sequenced or retrieved) are represented by colours, while specimen attribute (terra typica, type locality, type specimen or faunistic novelty) is represented by the shape (right). Sequences: ID = 2, cf. = 0; maximum p-distance: strict = NA, less strict = 0 %.

Haplotype network analysis of *Aphaenogaster fiorii* was not possible.

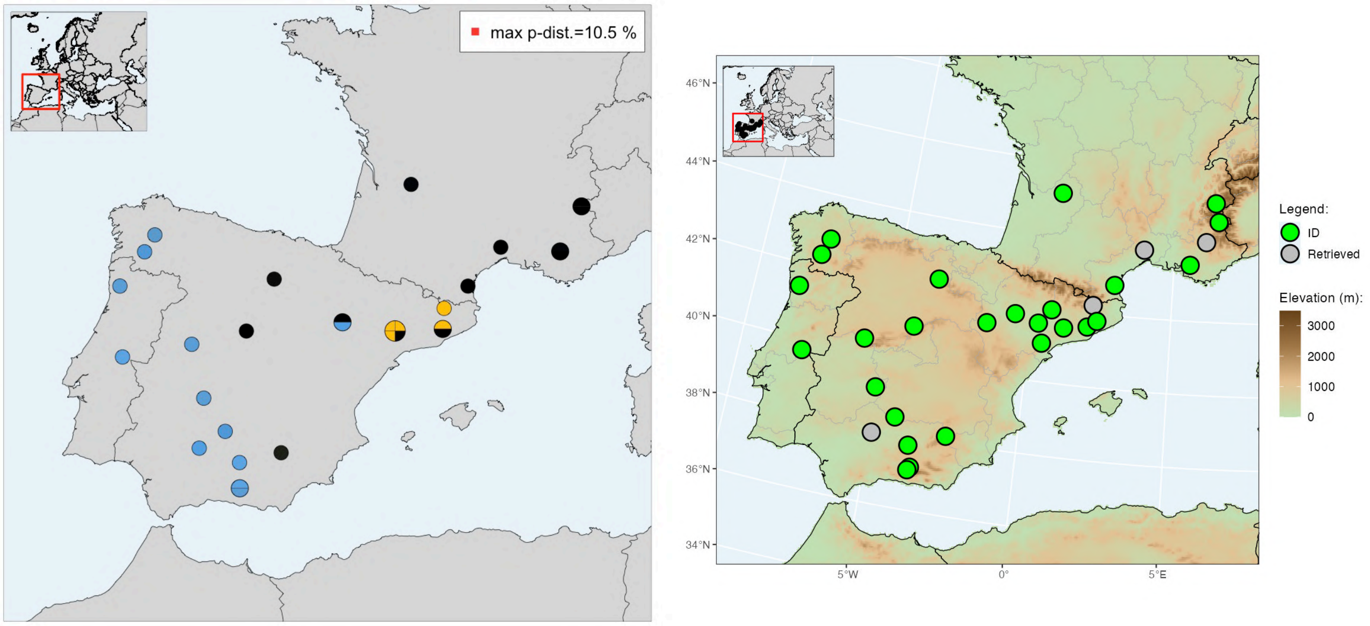

Figure 27: Genetic diversity map of *Aphaenogaster gibbosa* (Latreille, 1798). Nearby localities of sequenced specimens are merged in pies (left). Colours match the bidimensional colour space of the PCoA projection (Fig. 27 left) of p-dist between sequences (dots). Specimen identification (ID or cf.) and source (newly sequenced or retrieved) are represented by colours, while specimen attribute (terra typica, type locality, type specimen or faunistic novelty) is represented by the shape (right). Sequences: ID = 30, cf. = 0; maximum p-distance: strict = 10.5 %, less strict = 10.5 %.

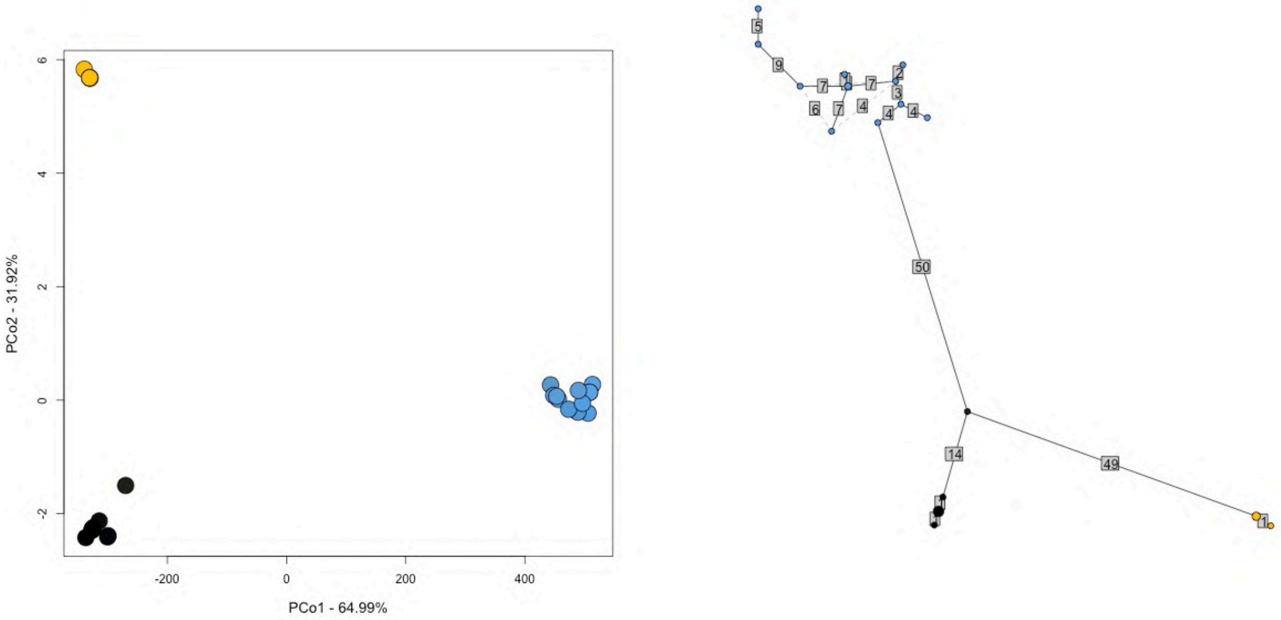

Figure 28: PCoA based on pairwise p-distances between *Aphaenogaster gibbosa* sequences (left). Colours match a bidimensional colour space. Haplotype network of *Aphaenogaster gibbosa* (right). Sequences > 599 bp: ID = 30, cf. = 0.

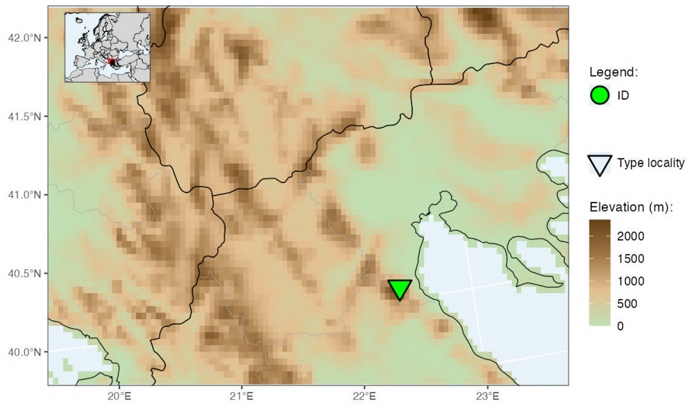

Figure 29: Map of *Aphaenogaster graeca* Schulz, 1994. Due to the presence of a single sequence, the genetic diversity map and the PCoA projection were not done. Specimen identification (ID or cf.) and source (newly sequenced or retrieved) are represented by colours, while specimen attribute (terra typica, type locality, type specimen or faunistic novelty) is represented by the shape. Sequences: ID = 1, cf. = 0; maximum p-distance: strict = NA, less strict = NA.

Haplotype network analysis of *Aphaenogaster graeca* was not possible.

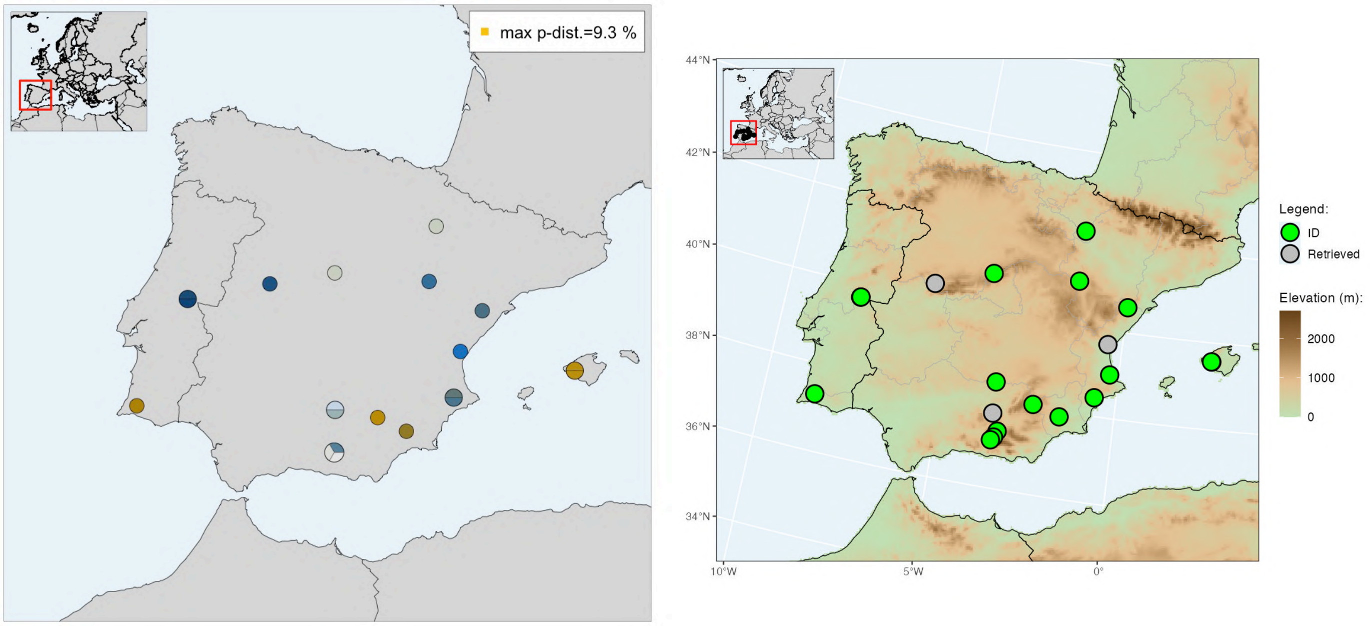

Figure 30: Genetic diversity map of *Aphaenogaster iberica* Emery, 1908. Nearby localities of sequenced specimens are merged in pies (left). Colours match the bidimensional colour space of the PCoA projection (Fig. 30 left) of p-dist between sequences (dots). Specimen identification (ID or cf.) and source (newly sequenced or retrieved) are represented by colours, while specimen attribute (terra typica, type locality, type specimen or faunistic novelty) is represented by the shape (right). Sequences: ID = 20, cf. = 0; maximum p-distance: strict = 9.3 %, less strict = 9.3 %.

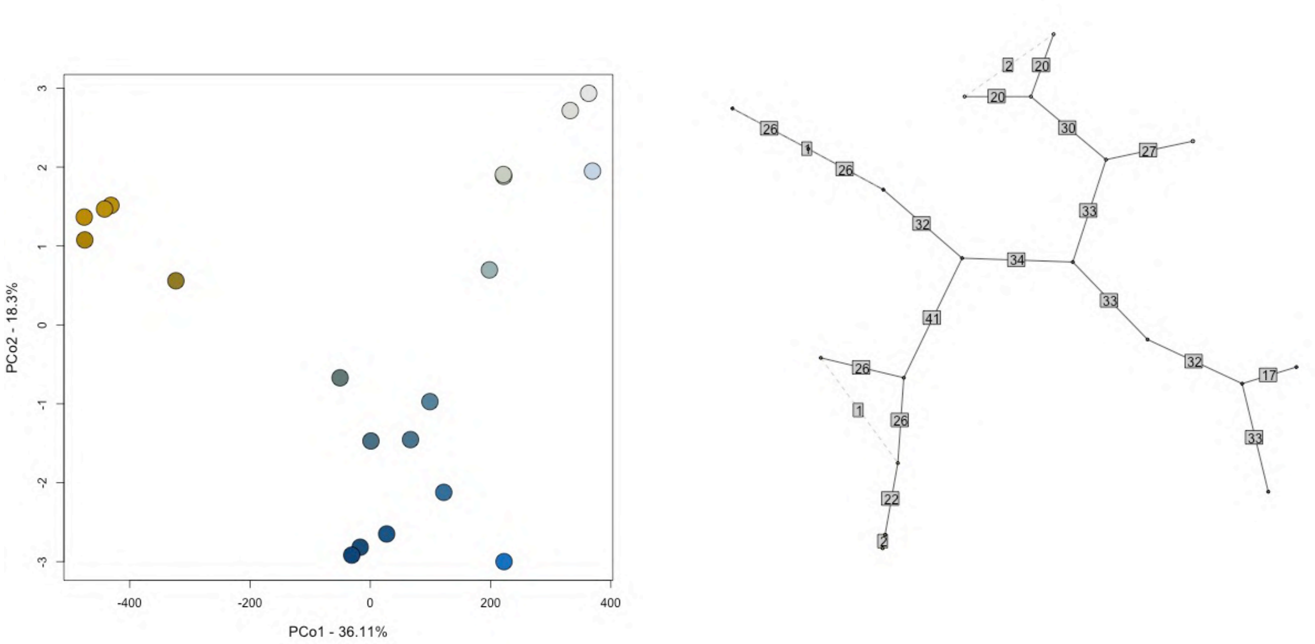

Figure 31: PCoA based on pairwise p-distances between *Aphaenogaster iberica* sequences (left). Colours match a bidimensional colour space. Haplotype network of *Aphaenogaster iberica* (right). Sequences > 599 bp: ID = 20, cf. = 0.

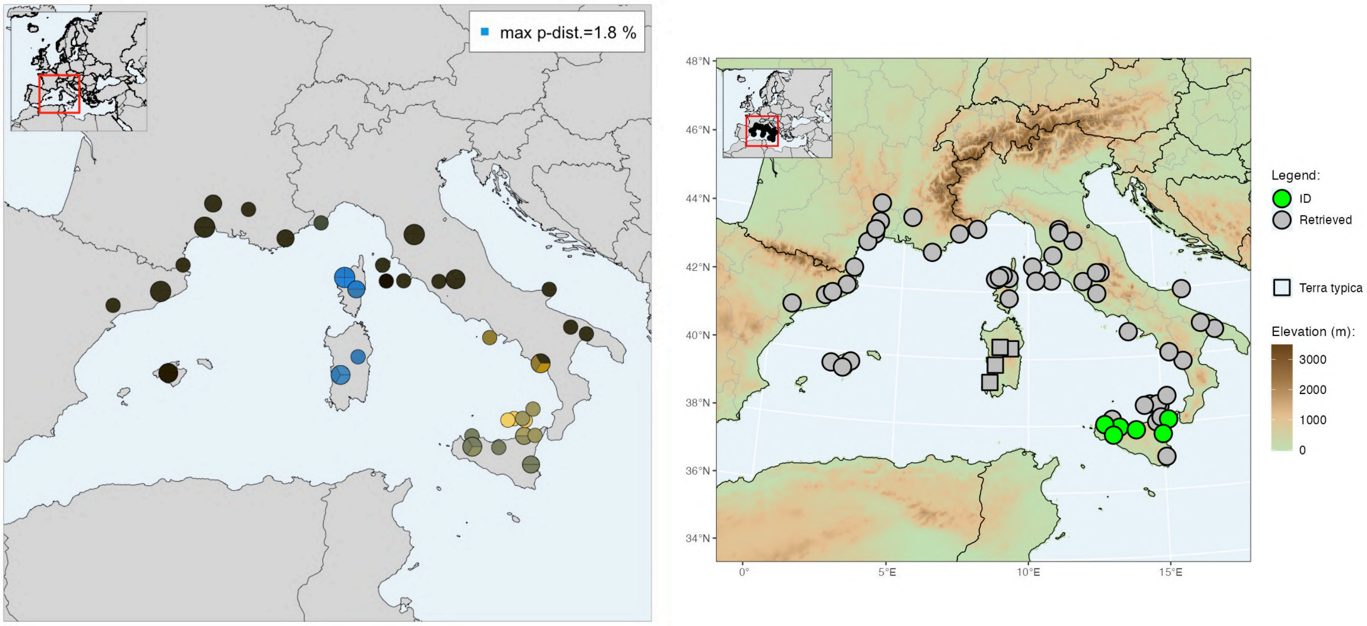

Figure 32: Genetic diversity map of *Aphaenogaster ichnusa* Santschi, 1925. Nearby localities of sequenced specimens are merged in pies (left). Colours match the bidimensional colour space of the PCoA projection (Fig. 32 left) of p-dist between sequences (dots). Specimen identification (ID or cf.) and source (newly sequenced or retrieved) are represented by colours, while specimen attribute (terra typica, type locality, type specimen or faunistic novelty) is represented by the shape (right). Sequences: ID = 63, cf. = 0; maximum p-distance: strict = 1.8 %, less strict = 1.8 %.

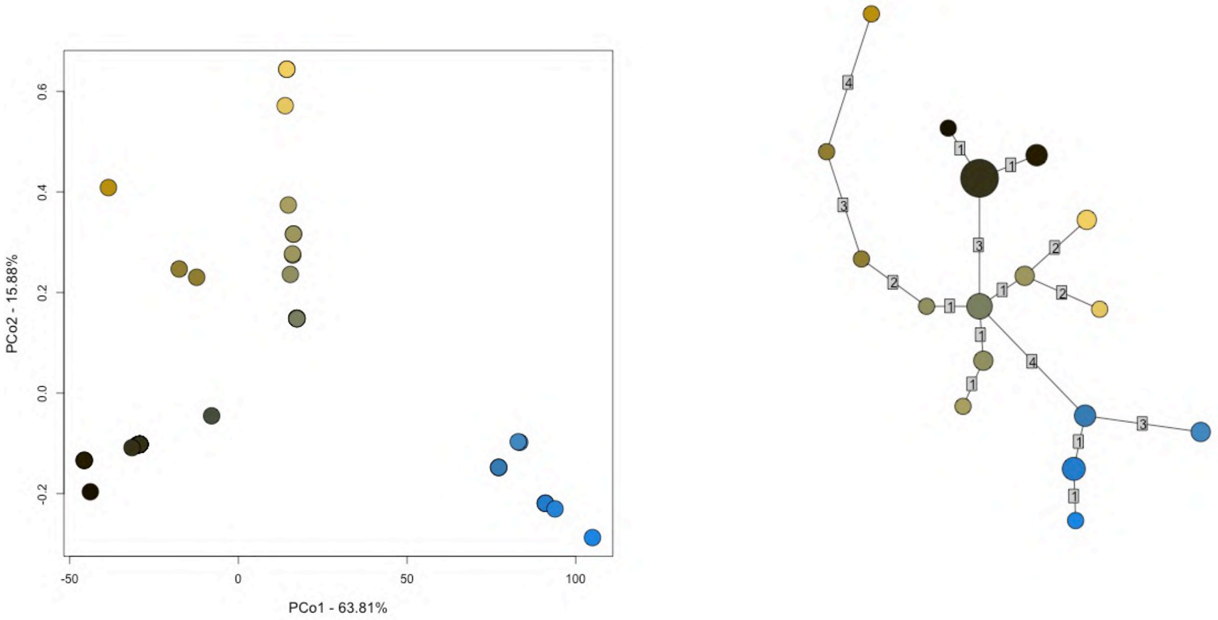

Figure 33: PCoA based on pairwise p-distances between *Aphaenogaster ichnusa* sequences (left). Colours match a bidimensional colour space. Haplotype network of *Aphaenogaster ichnusa* (right). Sequences > 599 bp: ID = 62, cf. = 0.

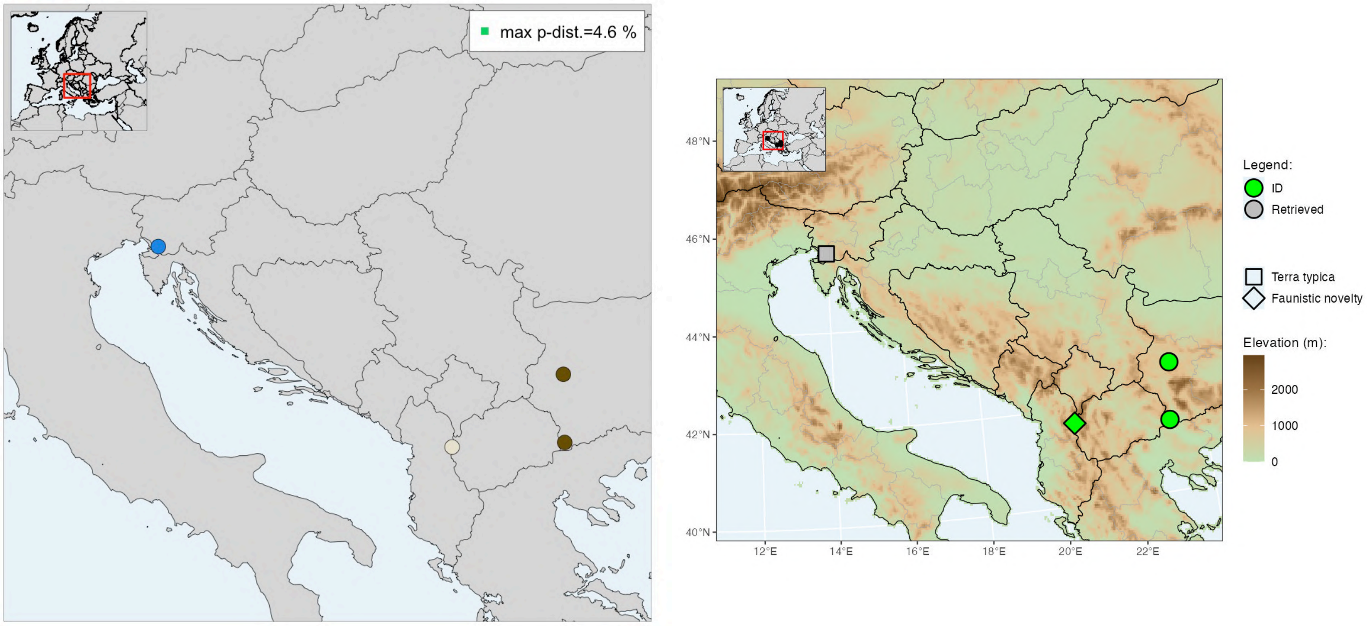

Figure 34: Genetic diversity map of *Aphaenogaster illyrica* Bračko, Lapeva-Gjonova, Salata, Borowiec & Polak, 2019. Nearby localities of sequenced specimens are merged in pies (left). Colours match the bidimensional colour space of the PCoA projection (Fig. 34 left) of p-dist between sequences (dots). Specimen identification (ID or cf.) and source (newly sequenced or retrieved) are represented by colours, while specimen attribute (terra typica, type locality, type specimen or faunistic novelty) is represented by the shape (right). Sequences: ID = 4, cf. = 0; maximum p-distance: strict = 4.6 %, less strict = 4.6 %.

The species is reported for the first time in Albania.

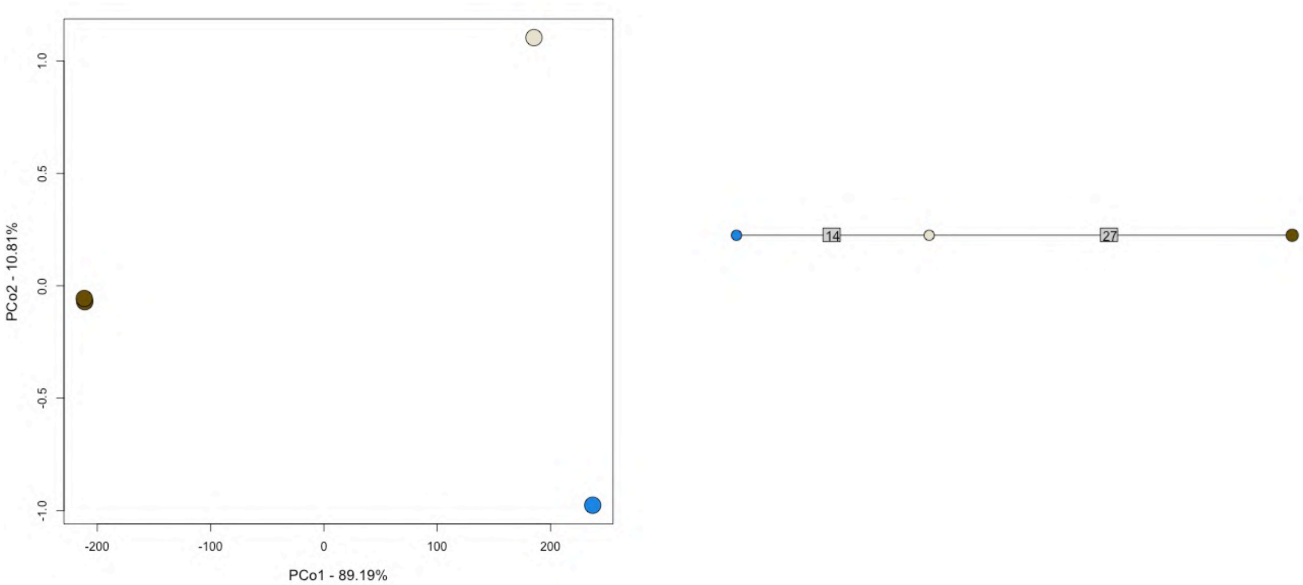

Figure 35: PCoA based on pairwise p-distances between *Aphaenogaster illyrica* sequences (left). Colours match a bidimensional colour space. Haplotype network of *Aphaenogaster illyrica* (right). Sequences > 599 bp: ID = 4, cf. = 0.

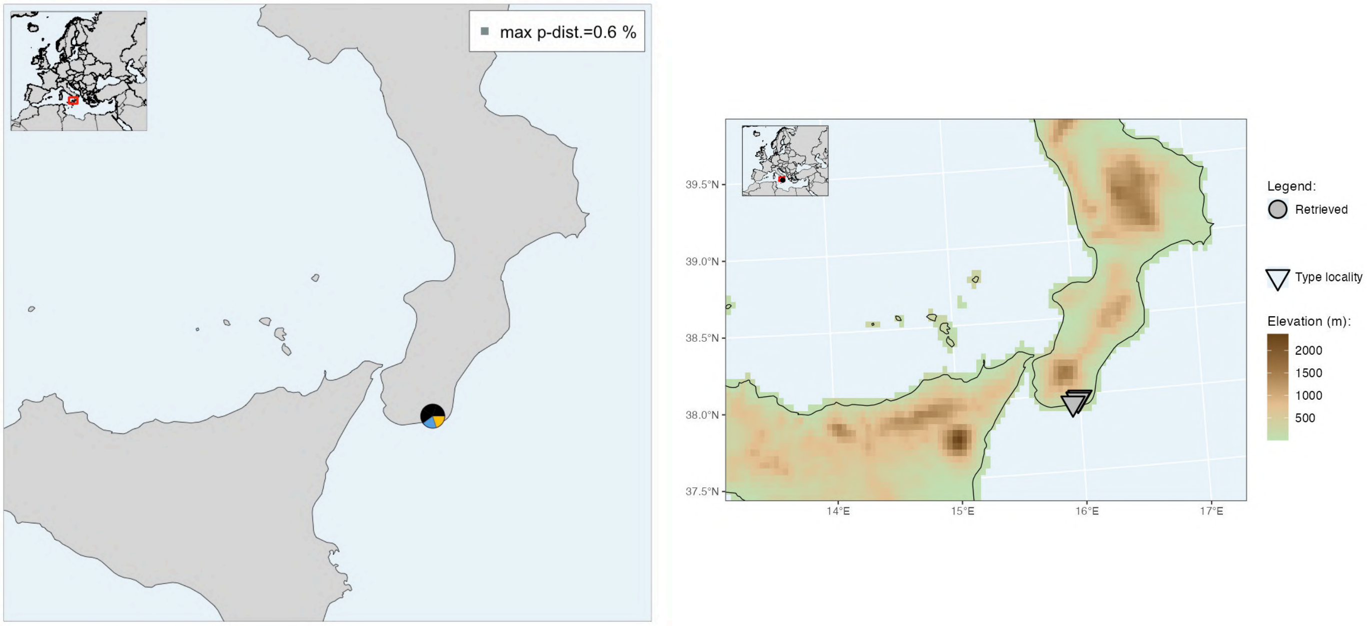

Figure 36: Genetic diversity map of *Aphaenogaster inermita* Bolton, 1995. Nearby localities of sequenced specimens are merged in pies (left). Colours match the bidimensional colour space of the PCoA projection (Fig. 36 left) of p-dist between sequences (dots). Specimen identification (ID or cf.) and source (newly sequenced or retrieved) are represented by colours, while specimen attribute (terra typica, type locality, type specimen or faunistic novelty) is represented by the shape (right). Sequences: ID = 5, cf. = 0; maximum p-distance: strict = NA, less strict = 0.6 %.

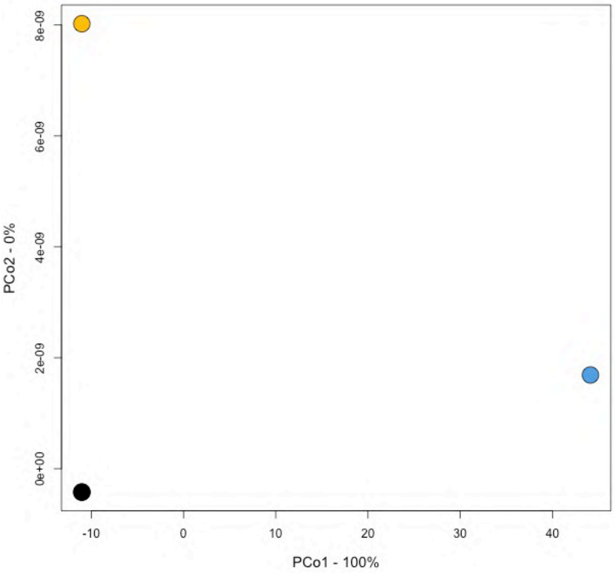

Figure 37: PCoA based on pairwise p-distances between *Aphaenogaster inermita* sequences (left). Colours match a bidimensional colour space. Haplotype network analysis of *Aphaenogaster inermita* was not possible.

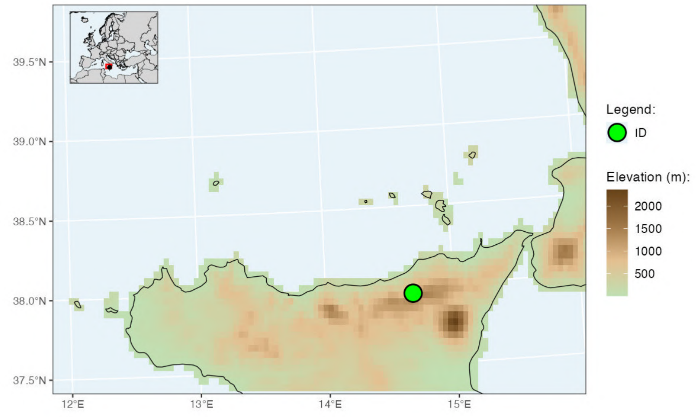

Figure 38: Map of *Aphaenogaster italica* Bondroit, 1918. Due to the presence of a single sequence, the genetic diversity map and the PCoA projection were not done. Specimen identification (ID or cf.) and source (newly sequenced or retrieved) are represented by colours, while specimen attribute (terra typica, type locality, type specimen or faunistic novelty) is represented by the shape. Sequences: ID = 1, cf. = 0; maximum p-distance: strict = NA, less strict = NA.

Haplotype network analysis of *Aphaenogaster italica* was not possible.

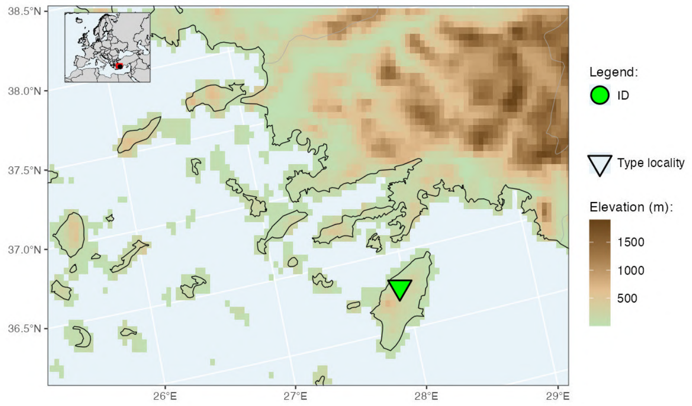

Figure 39: Map of *Aphaenogaster jolantae* Borowiec & Salata, 2014. Due to the presence of a single sequence, the genetic diversity map and the PCoA projection were not done. Specimen identification (ID or cf.) and source (newly sequenced or retrieved) are represented by colours, while specimen attribute (terra typica, type locality, type specimen or faunistic novelty) is represented by the shape. Sequences: ID = 1, cf. = 0; maximum p-distance: strict = NA, less strict = NA.

Haplotype network analysis of *Aphaenogaster jolantae* was not possible.

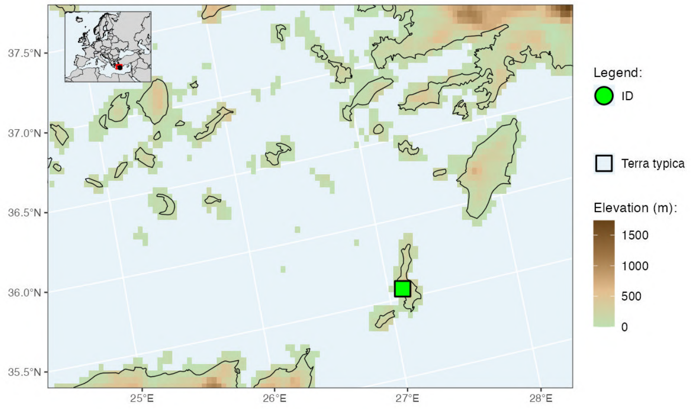

Figure 40: Map of *Aphaenogaster karpathica* Boer, 2013. Due to the presence of a single sequence, the genetic diversity map and the PCoA projection were not done. Specimen identification (ID or cf.) and source (newly sequenced or retrieved) are represented by colours, while specimen attribute (terra typica, type locality, type specimen or faunistic novelty) is represented by the shape. Sequences: ID = 1, cf. = 0; maximum p-distance: strict = NA, less strict = NA.

Haplotype network analysis of *Aphaenogaster karpathica* was not possible.

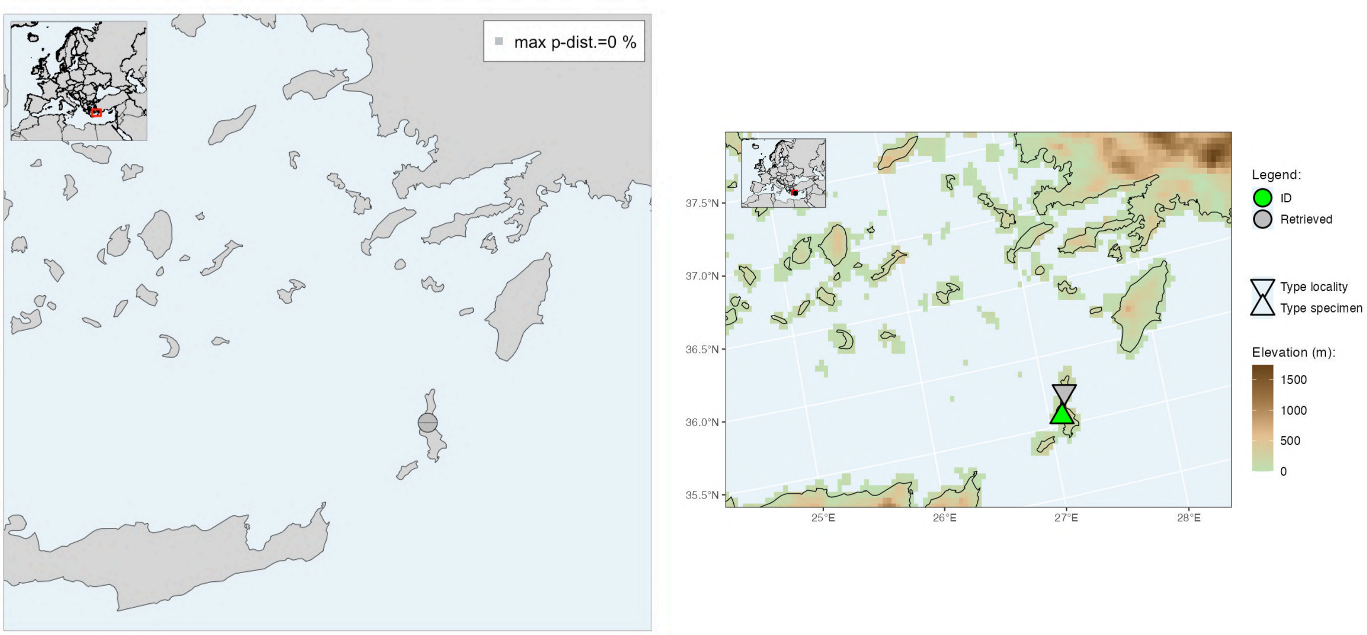

Figure 41: Genetic diversity map of *Aphaenogaster olympica* Borowiec & Salata, 2014. PCoA projection was not done and therefore sequenced specimens in the genetic diversity map are coloured in gray (left). Specimen identification (ID or cf.) and source (newly sequenced or retrieved) are represented by colours, while specimen attribute (terra typica, type locality, type specimen or faunistic novelty) is represented by the shape (right). Sequences: ID = 2, cf. = 0; maximum p-distance: strict = NA, less strict = 0 %.

Haplotype network analysis of *Aphaenogaster olympica* was not possible.

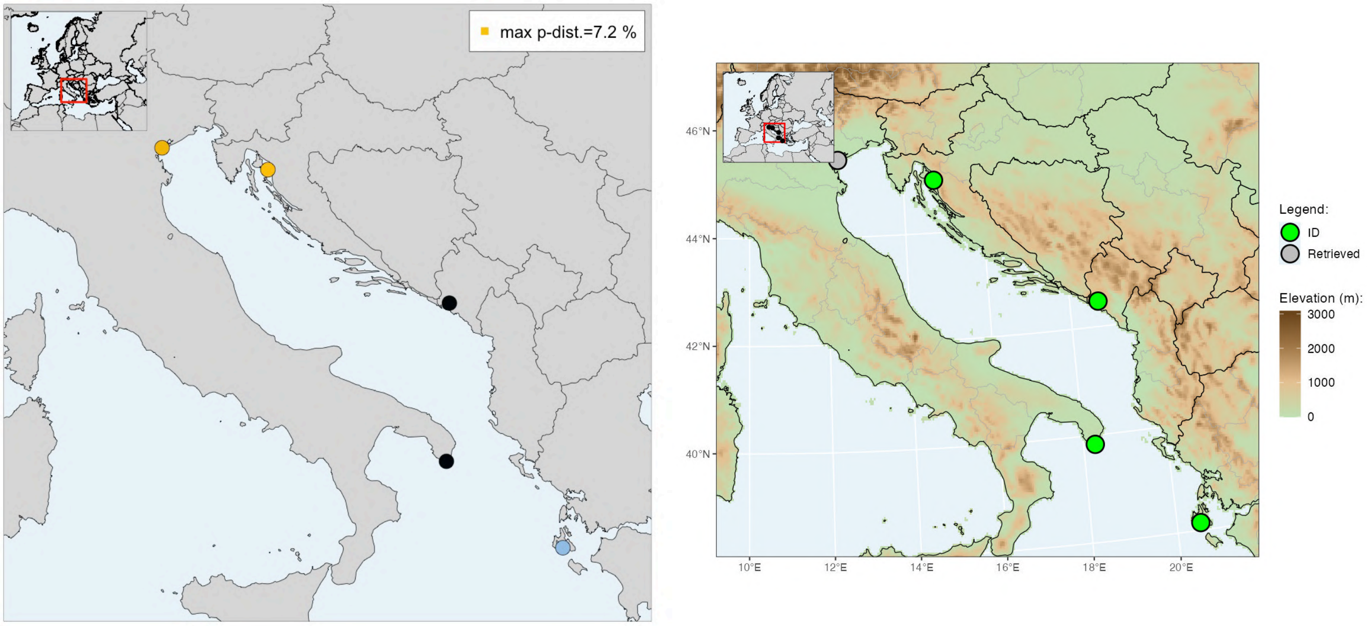

Figure 42: Genetic diversity map of *Aphaenogaster ovaticeps* (Emery, 1898). Nearby localities of sequenced specimens are merged in pies (left). Colours match the bidimensional colour space of the PCoA projection (Fig. 42 left) of p-dist between sequences (dots). Specimen identification (ID or cf.) and source (newly sequenced or retrieved) are represented by colours, while specimen attribute (terra typica, type locality, type specimen or faunistic novelty) is represented by the shape (right). Sequences: ID = 5, cf. = 0; maximum p-distance: strict = 7.2 %, less strict = 7.2 %.

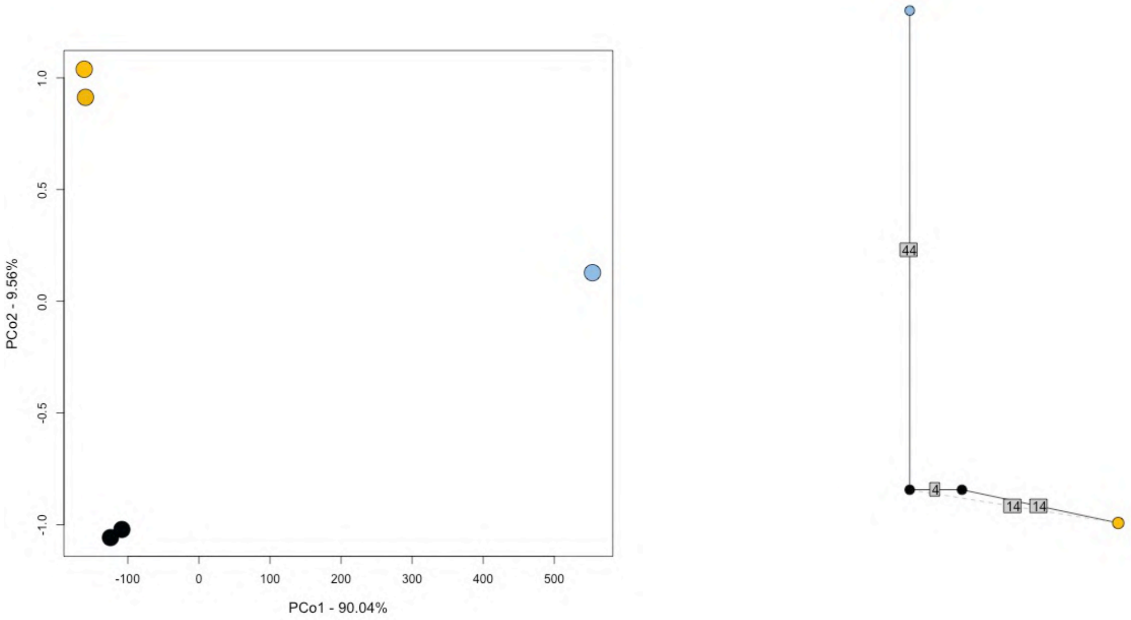

Figure 43: PCoA based on pairwise p-distances between *Aphaenogaster ovaticeps* sequences (left). Colours match a bidimensional colour space. Haplotype network of *Aphaenogaster ovaticeps* (right). Sequences > 599 bp: ID = 5, cf. = 0.

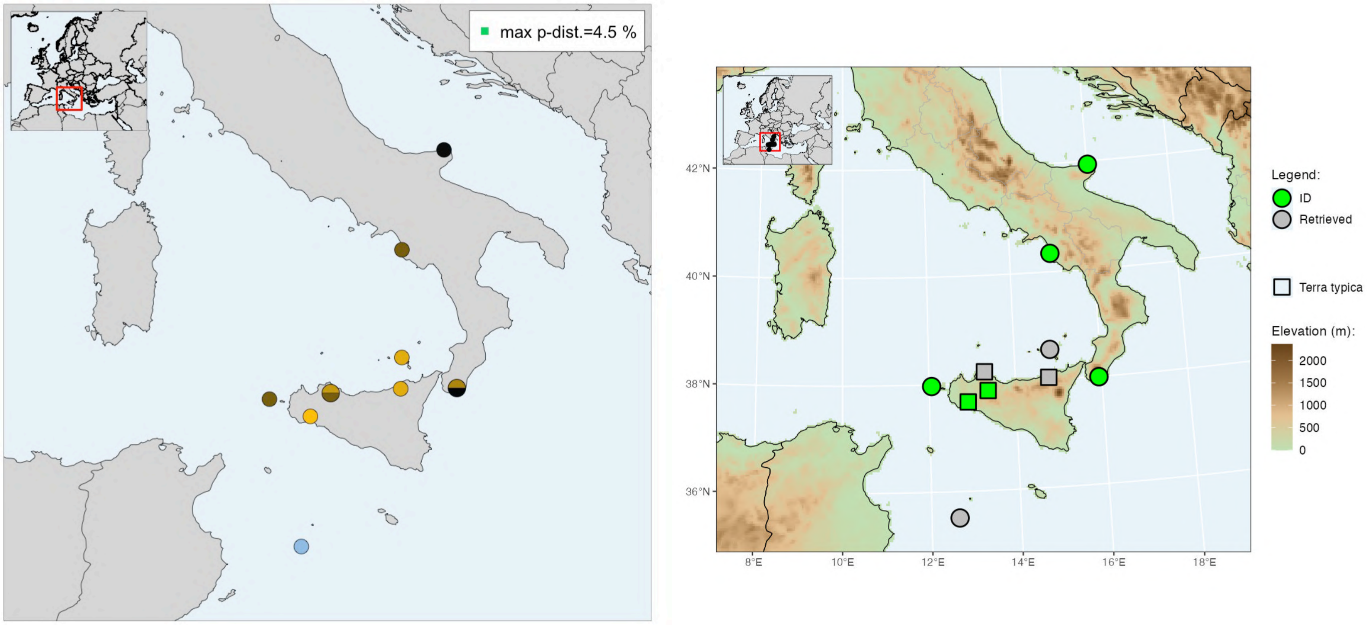

Figure 44: Genetic diversity map of *Aphaenogaster pallida* (Nylander, 1849). Nearby localities of sequenced specimens are merged in pies (left). Colours match the bidimensional colour space of the PCoA projection (Fig. 44 left) of p-dist between sequences (dots). Specimen identification (ID or cf.) and source (newly sequenced or retrieved) are represented by colours, while specimen attribute (terra typica, type locality, type specimen or faunistic novelty) is represented by the shape (right). Sequences: ID = 11, cf. = 0; maximum p-distance: strict = 4.5 %, less strict = 4.5 %.

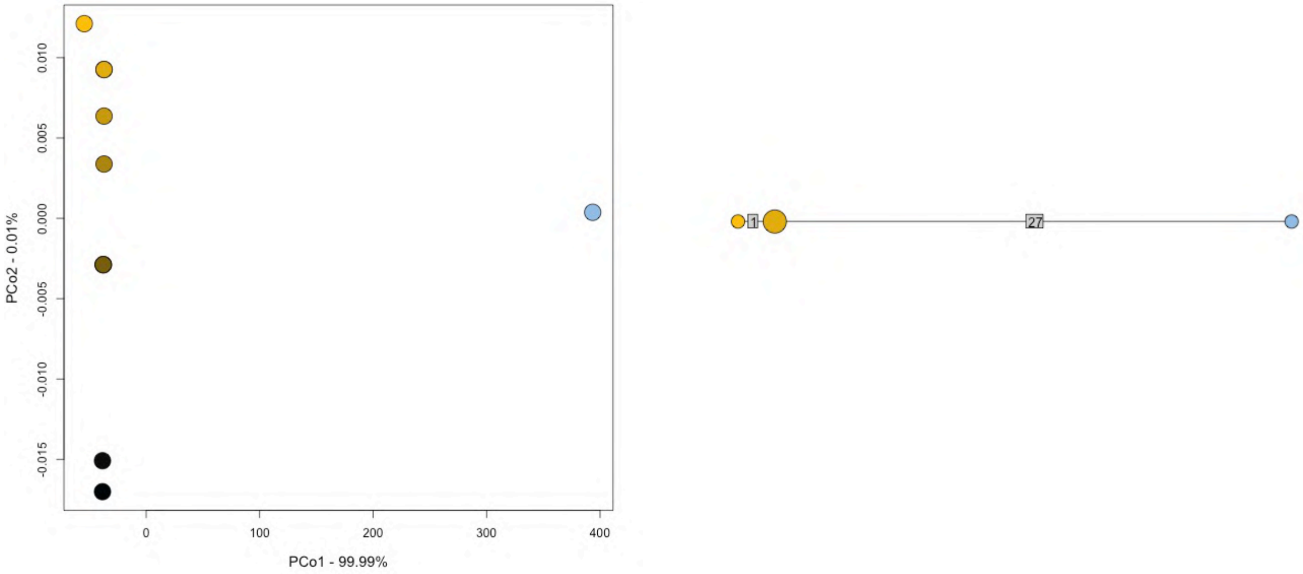

Figure 45: PCoA based on pairwise p-distances between *Aphaenogaster pallida* sequences (left). Colours match a bidimensional colour space. Haplotype network of *Aphaenogaster pallida* (right). Sequences > 599 bp: ID = 11, cf. = 0.

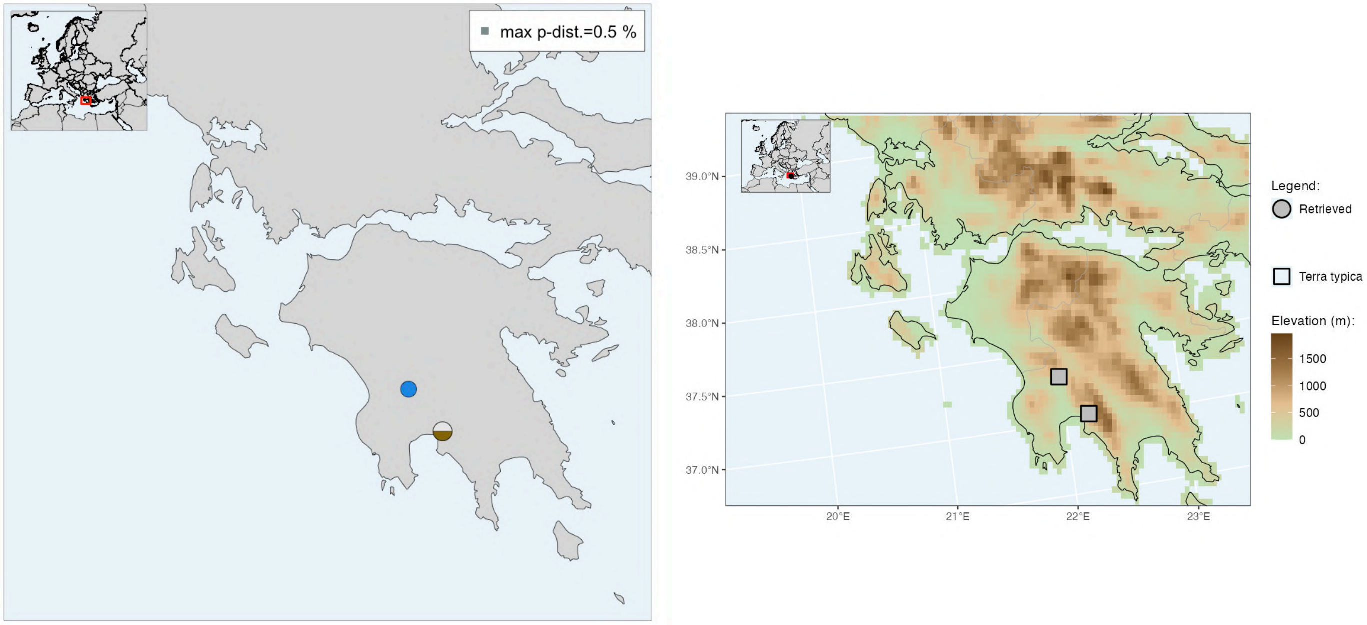

Figure 46: Genetic diversity map of *Aphaenogaster peloponnesiaca* Salata, Karaman, Kiran, Borowiec 2021. Nearby localities of sequenced specimens are merged in pies (left). Colours match the bidimensional colour space of the PCoA projection (Fig. 46 left) of p-dist between sequences (dots). Specimen identification (ID or cf.) and source (newly sequenced or retrieved) are represented by colours, while specimen attribute (terra typica, type locality, type specimen or faunistic novelty) is represented by the shape (right). Sequences: ID = 3, cf. = 0; maximum p-distance: strict = 0.5 %, less strict = 0.5 %.

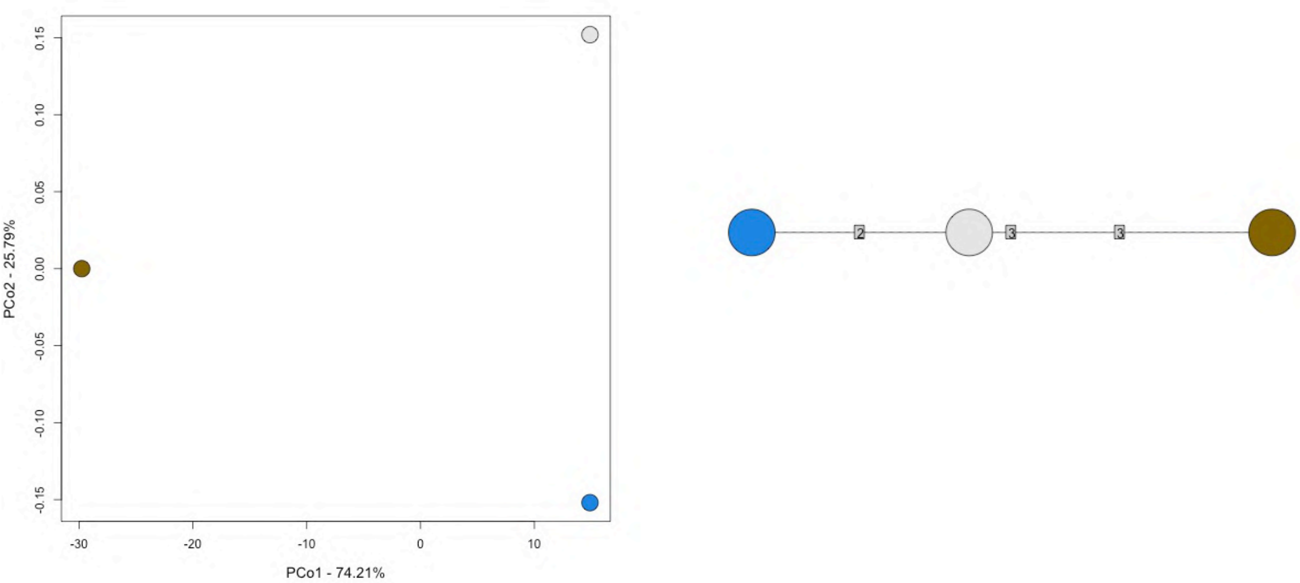

Figure 47: PCoA based on pairwise p-distances between *Aphaenogaster peloponnesiaca* sequences (left). Colours match a bidimensional colour space. Haplotype network of *Aphaenogaster peloponnesiaca* (right). Sequences > 599 bp: ID = 3, cf. = 0.

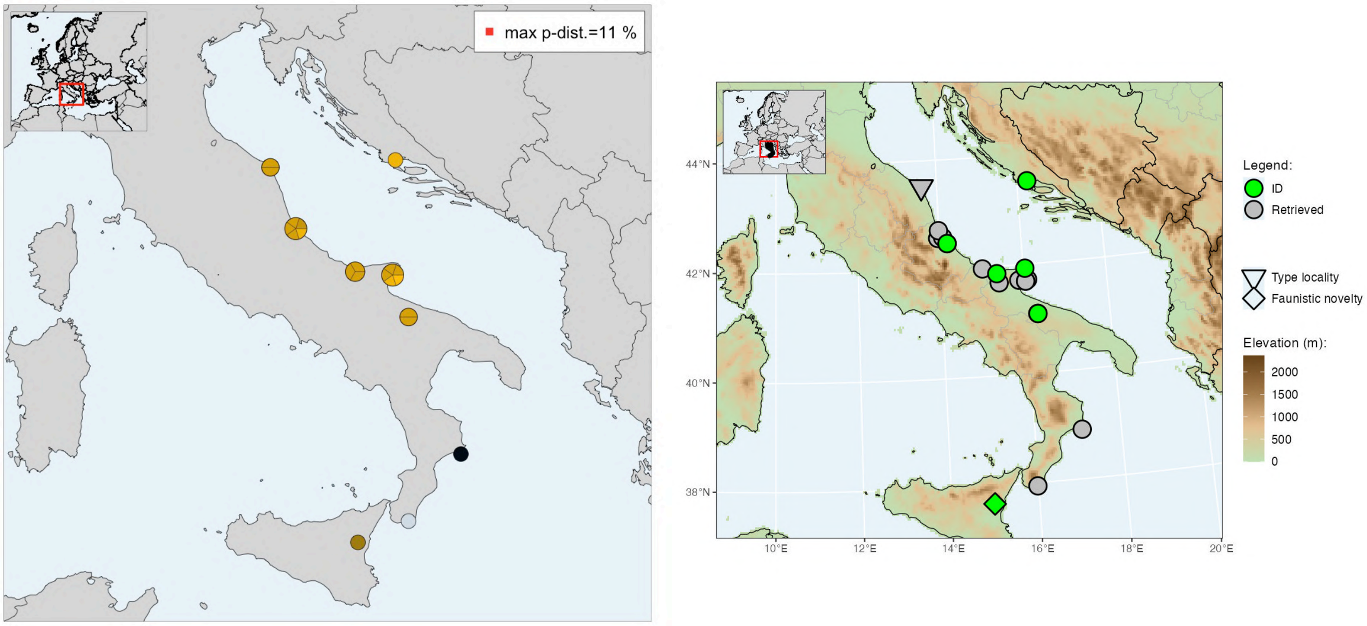

Figure 48: Genetic diversity map of *Aphaenogaster picena* Baroni Urbani, 1971. Nearby localities of sequenced specimens are merged in pies (left). Colours match the bidimensional colour space of the PCoA projection (Fig. 48 left) of p-dist between sequences (dots). Specimen identification (ID or cf.) and source (newly sequenced or retrieved) are represented by colours, while specimen attribute (terra typica, type locality, type specimen or faunistic novelty) is represented by the shape (right). Sequences: ID = 21, cf. = 0; maximum p-distance: strict = 1.1 %, less strict = 11 %.

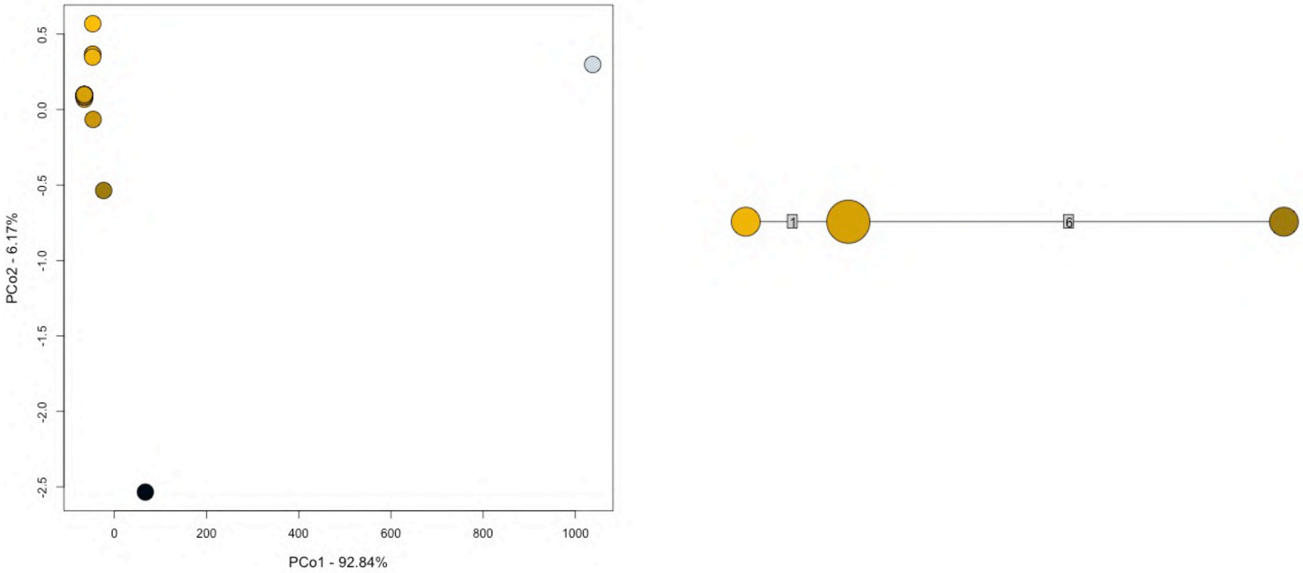

Figure 49: PCoA based on pairwise p-distances between *Aphaenogaster picena* sequences (left). Colours match a bidimensional colour space. Haplotype network of *Aphaenogaster picena* (right). Sequences > 599 bp: ID = 7, cf. = 0.

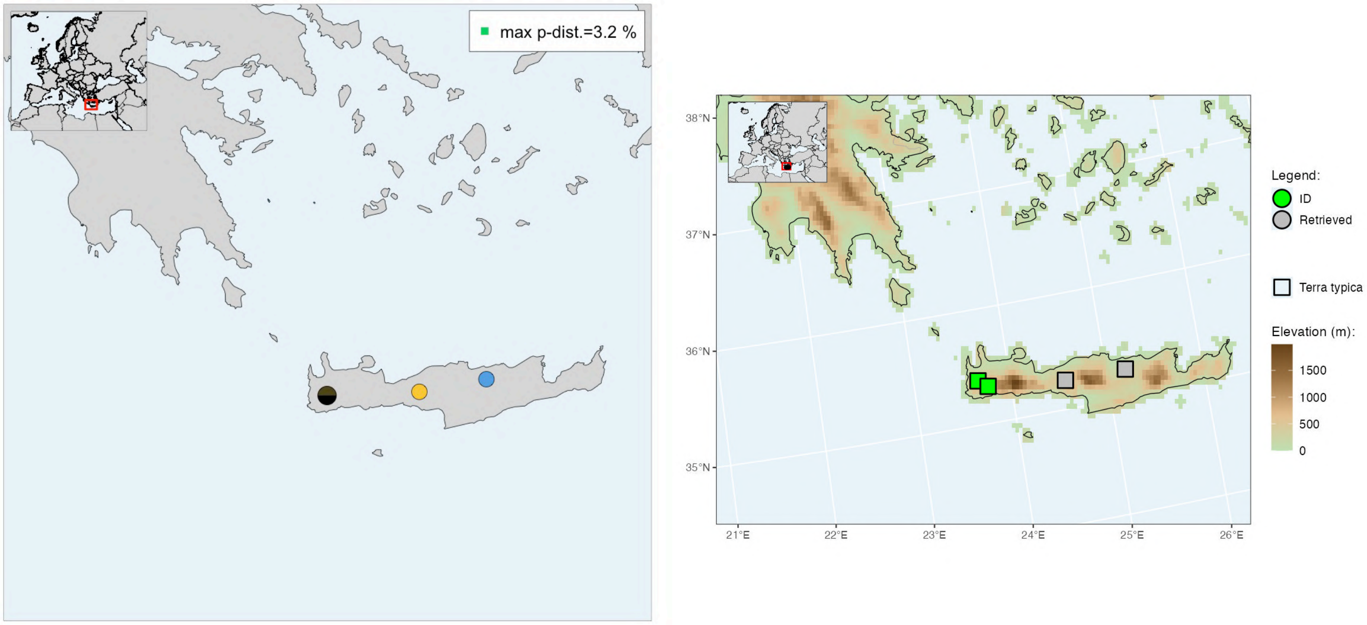

Figure 50: Genetic diversity map of *Aphaenogaster rugosoferruginea* Forel, 1889. Nearby localities of sequenced specimens are merged in pies (left). Colours match the bidimensional colour space of the PCoA projection (Fig. 50 left) of p-dist between sequences (dots). Specimen identification (ID or cf.) and source (newly sequenced or retrieved) are represented by colours, while specimen attribute (terra typica, type locality, type specimen or faunistic novelty) is represented by the shape (right). Sequences: ID = 4, cf. = 0; maximum p-distance: strict = 3.2 %, less strict = 3.2 %.

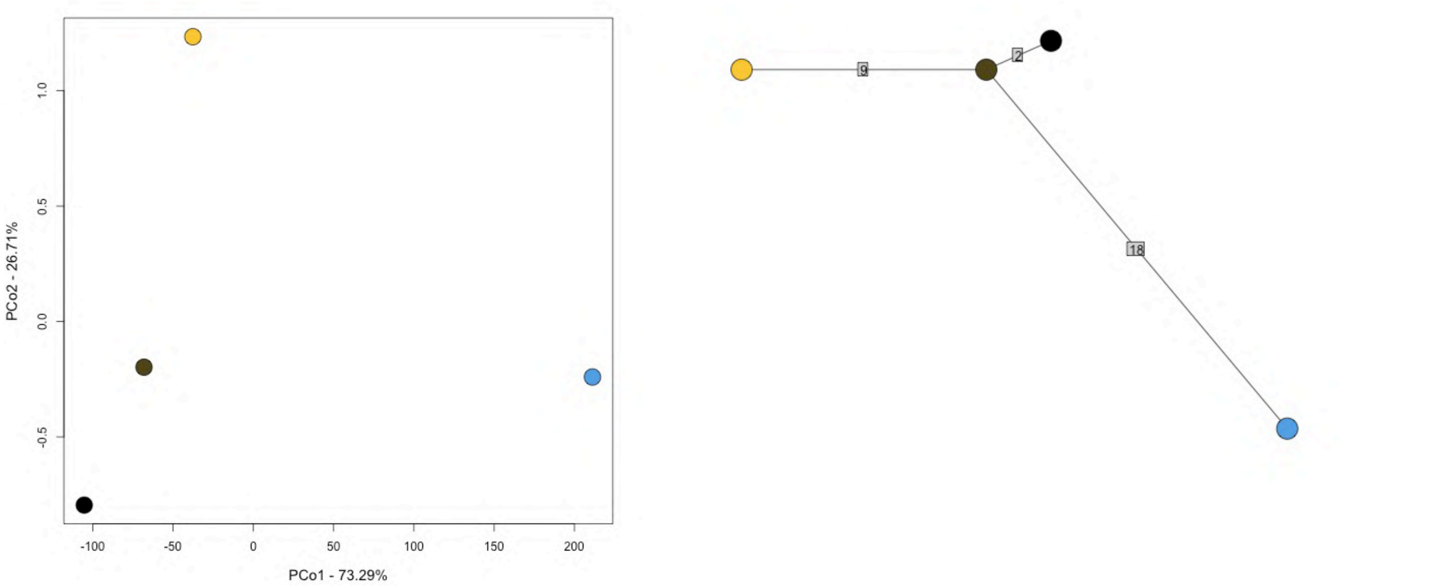

Figure 51: PCoA based on pairwise p-distances between *Aphaenogaster rugosoferruginea* sequences (left). Colours match a bidimensional colour space. Haplotype network of *Aphaenogaster rugosoferruginea* (right). Sequences > 599 bp: ID = 4, cf. = 0.

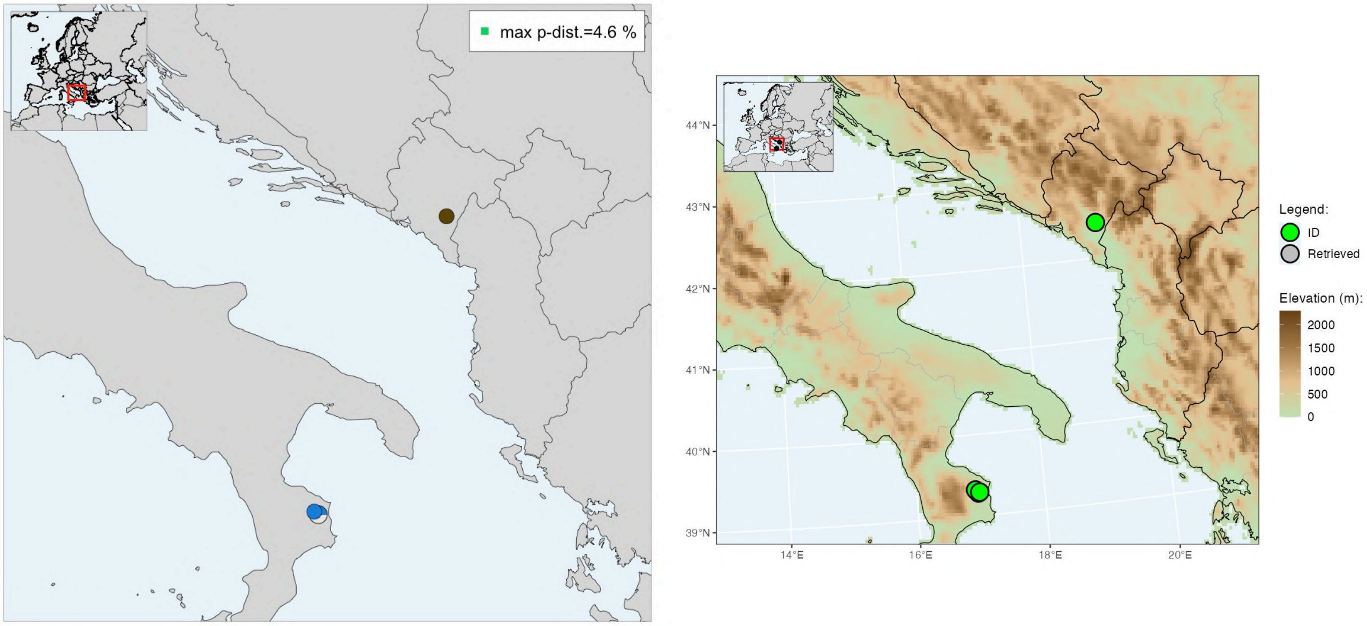

Figure 52: Genetic diversity map of *Aphaenogaster sangiorgii* (Emery, 1901). Nearby localities of sequenced specimens are merged in pies (left). Colours match the bidimensional colour space of the PCoA projection (Fig. 52 left) of p-dist between sequences (dots). Specimen identification (ID or cf.) and source (newly sequenced or retrieved) are represented by colours, while specimen attribute (terra typica, type locality, type specimen or faunistic novelty) is represented by the shape (right). Sequences: ID = 4, cf. = 0; maximum p-distance: strict = 4.6 %, less strict = 4.6 %.

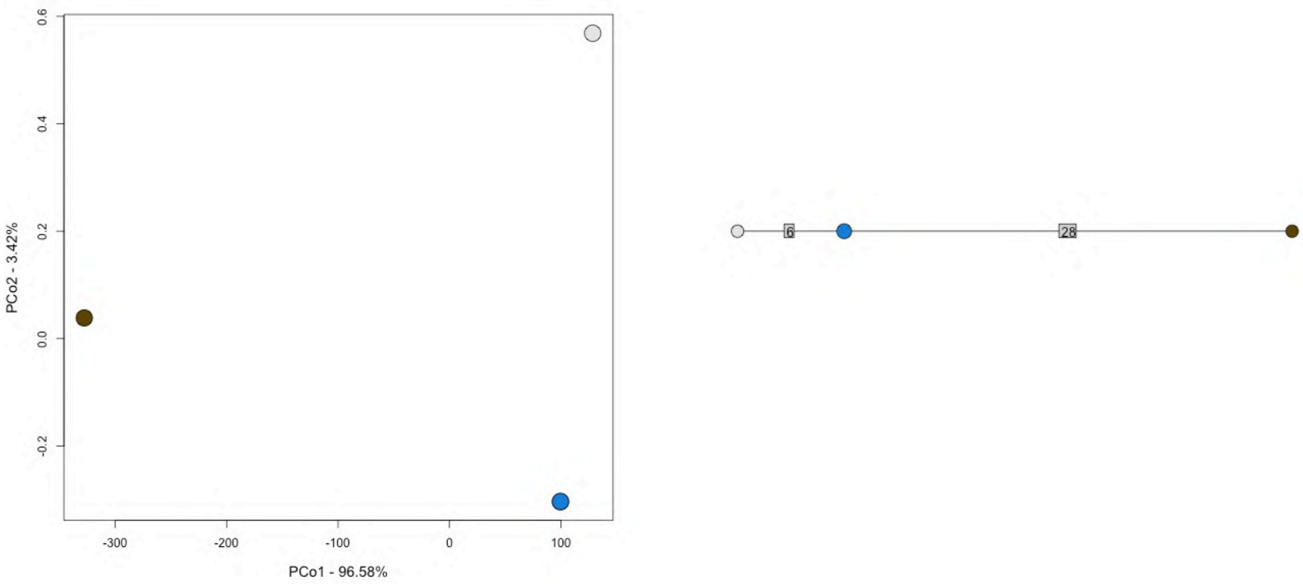

Figure 53: PCoA based on pairwise p-distances between *Aphaenogaster sangiorgii* sequences (left). Colours match a bidimensional colour space. Haplotype network of *Aphaenogaster sangiorgii* (right). Sequences > 599 bp: ID = 4, cf. = 0.

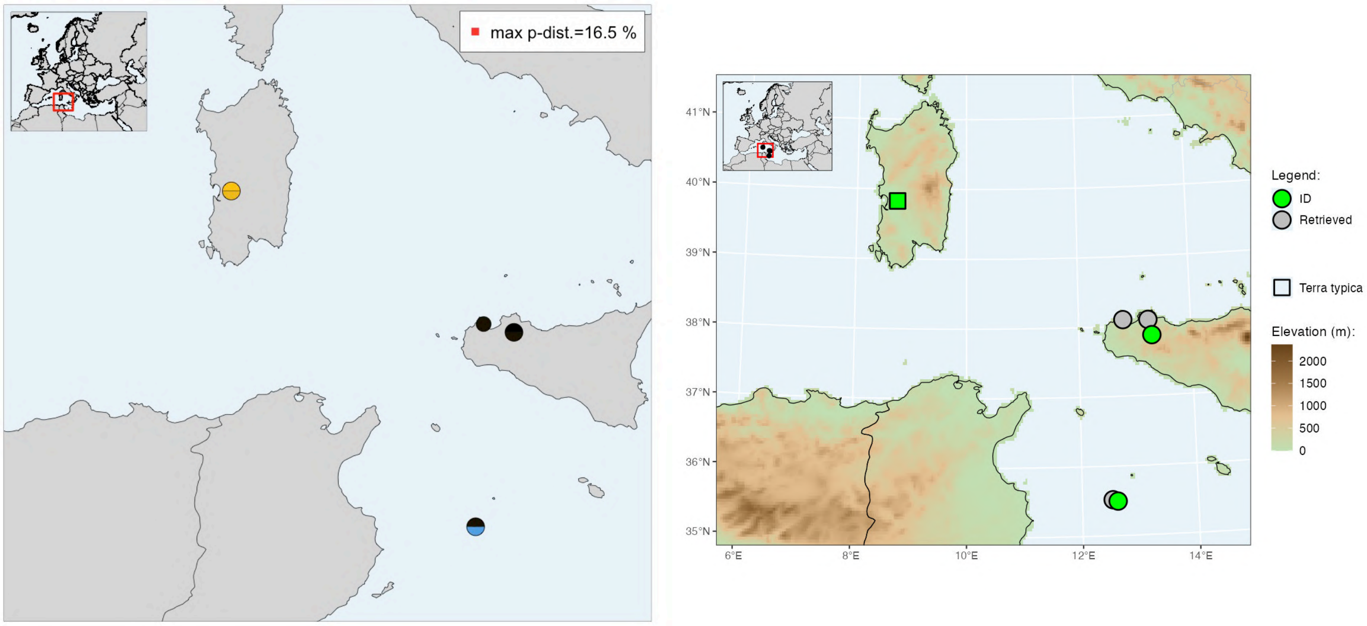

Figure 54: Genetic diversity map of *Aphaenogaster sardoa* Mayr, 1853. Nearby localities of sequenced specimens are merged in pies (left). Colours match the bidimensional colour space of the PCoA projection (Fig. 54 left) of p-dist between sequences (dots). Specimen identification (ID or cf.) and source (newly sequenced or retrieved) are represented by colours, while specimen attribute (terra typica, type locality, type specimen or faunistic novelty) is represented by the shape (right). Sequences: ID = 7, cf. = 0; maximum p-distance: strict = 16.5 %, less strict = 16.5 %.

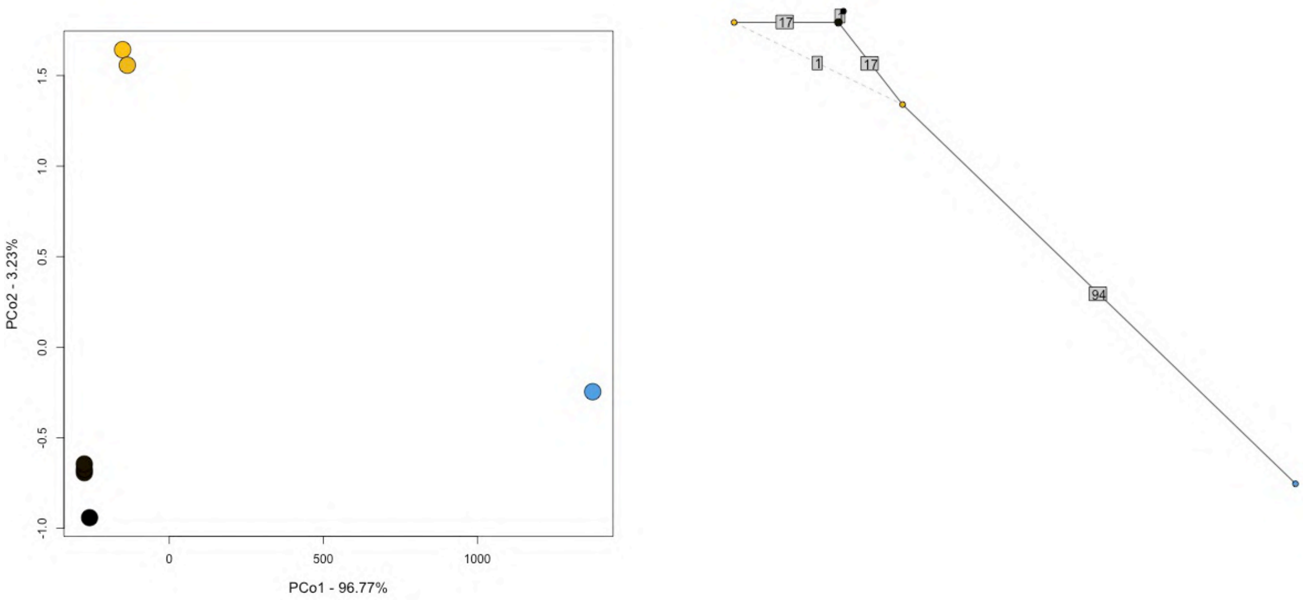

Figure 55: PCoA based on pairwise p-distances between *Aphaenogaster sardoa* sequences (left). Colours match a bidimensional colour space. Haplotype network of *Aphaenogaster sardoa* (right). Sequences > 599 bp: ID = 7, cf. = 0.

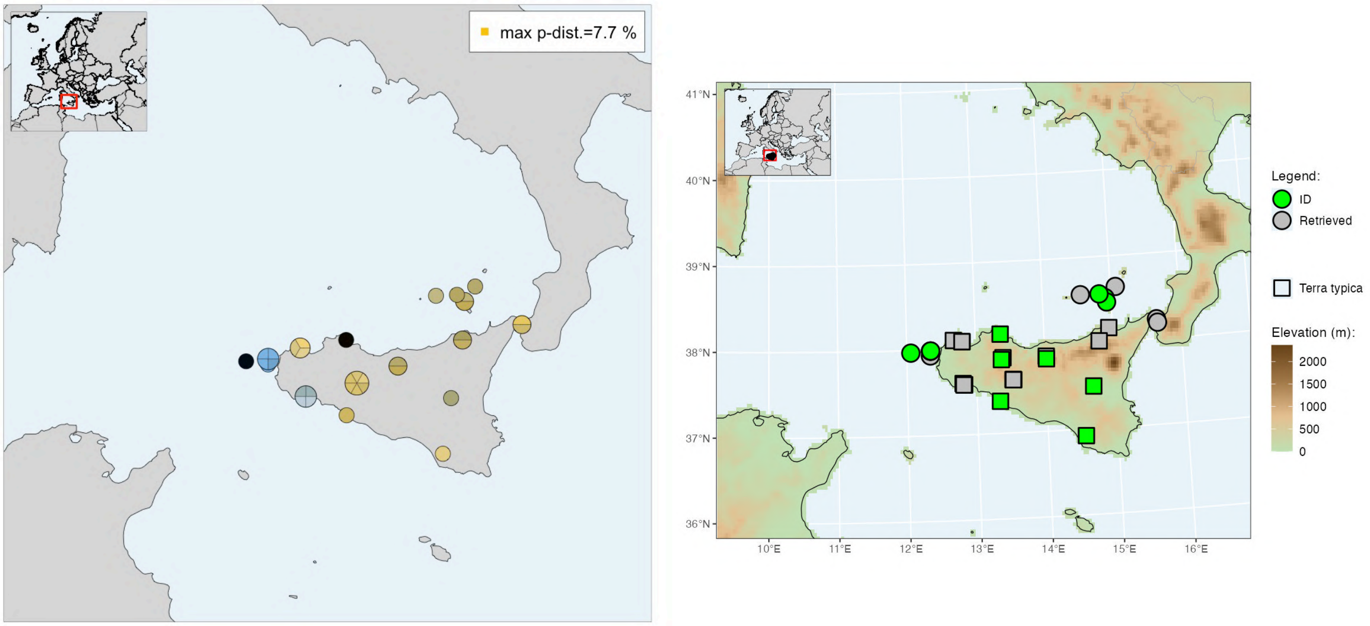

Figure 56: Genetic diversity map of *Aphaenogaster semipolita* (Nylander, 1856). Nearby localities of sequenced specimens are merged in pies (left). Colours match the bidimensional colour space of the PCoA projection (Fig. 56 left) of p-dist between sequences (dots). Specimen identification (ID or cf.) and source (newly sequenced or retrieved) are represented by colours, while specimen attribute (terra typica, type locality, type specimen or faunistic novelty) is represented by the shape (right). Sequences: ID = 34, cf. = 0; maximum p-distance: strict = 7.2 %, less strict = 7.7 %.

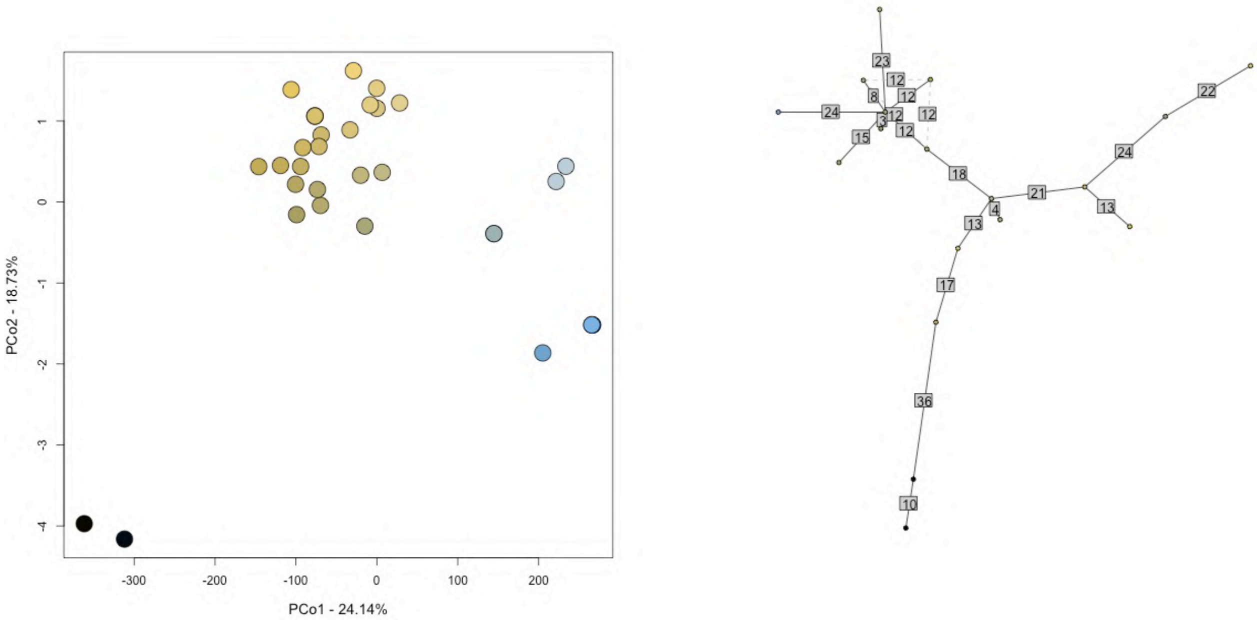

Figure 57: PCoA based on pairwise p-distances between *Aphaenogaster semipolita* sequences (left). Colours match a bidimensional colour space. Haplotype network of *Aphaenogaster semipolita* (right). Sequences > 599 bp: ID = 18, cf. = 0.

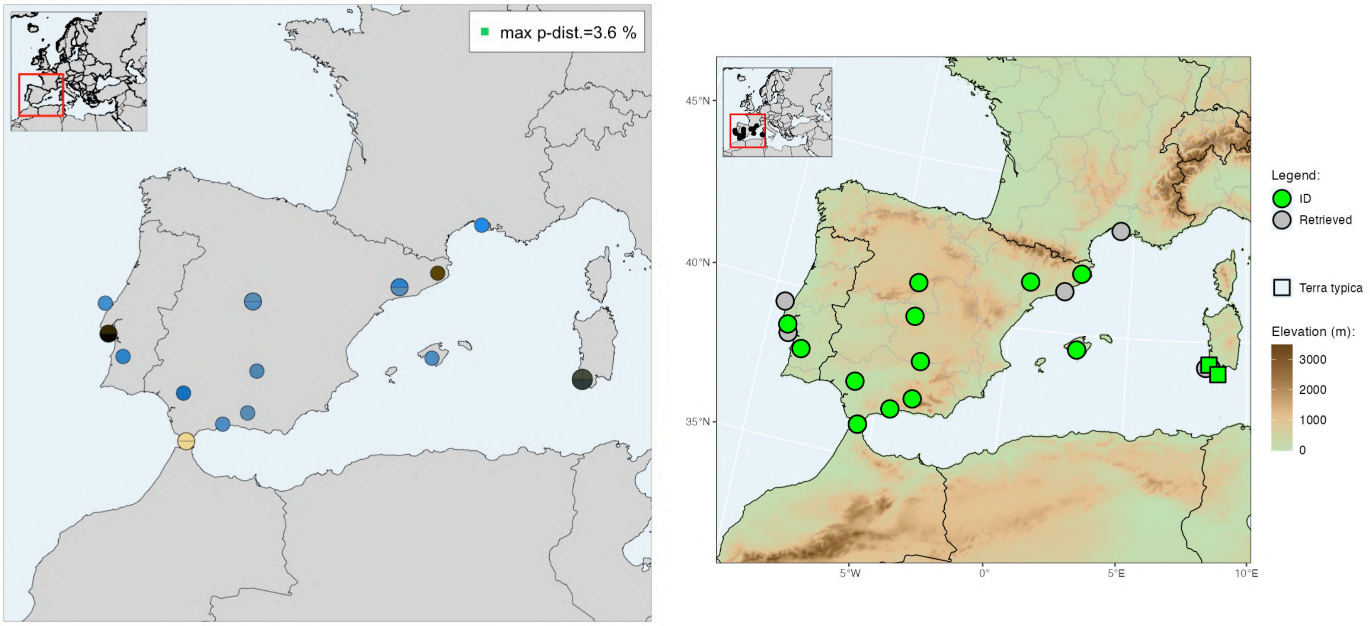

Figure 58: Genetic diversity map of *Aphaenogaster senilis* Mayr, 1853. Nearby localities of sequenced specimens are merged in pies (left). Colours match the bidimensional colour space of the PCoA projection (Fig. 58 left) of p-dist between sequences (dots). Specimen identification (ID or cf.) and source (newly sequenced or retrieved) are represented by colours, while specimen attribute (terra typica, type locality, type specimen or faunistic novelty) is represented by the shape (right). Sequences: ID = 21, cf. = 0; maximum p-distance: strict = 3.6 %, less strict = 3.6 %.

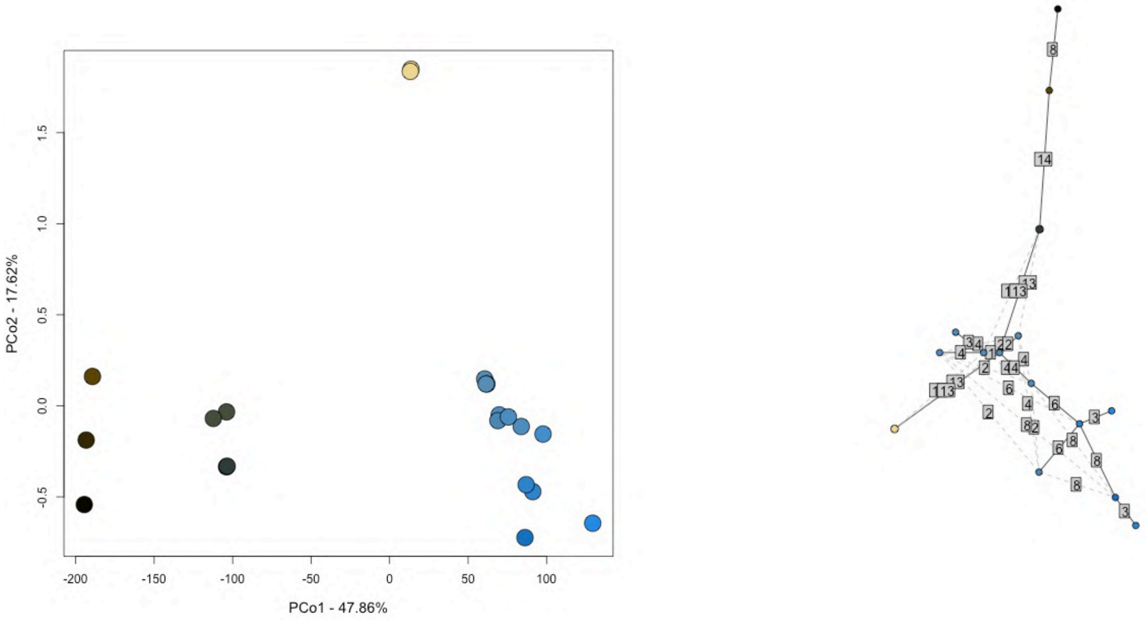

Figure 59: PCoA based on pairwise p-distances between *Aphaenogaster senilis* sequences (left). Colours match a bidimensional colour space. Haplotype network of *Aphaenogaster senilis* (right). Sequences > 599 bp: ID = 17, cf. = 0.

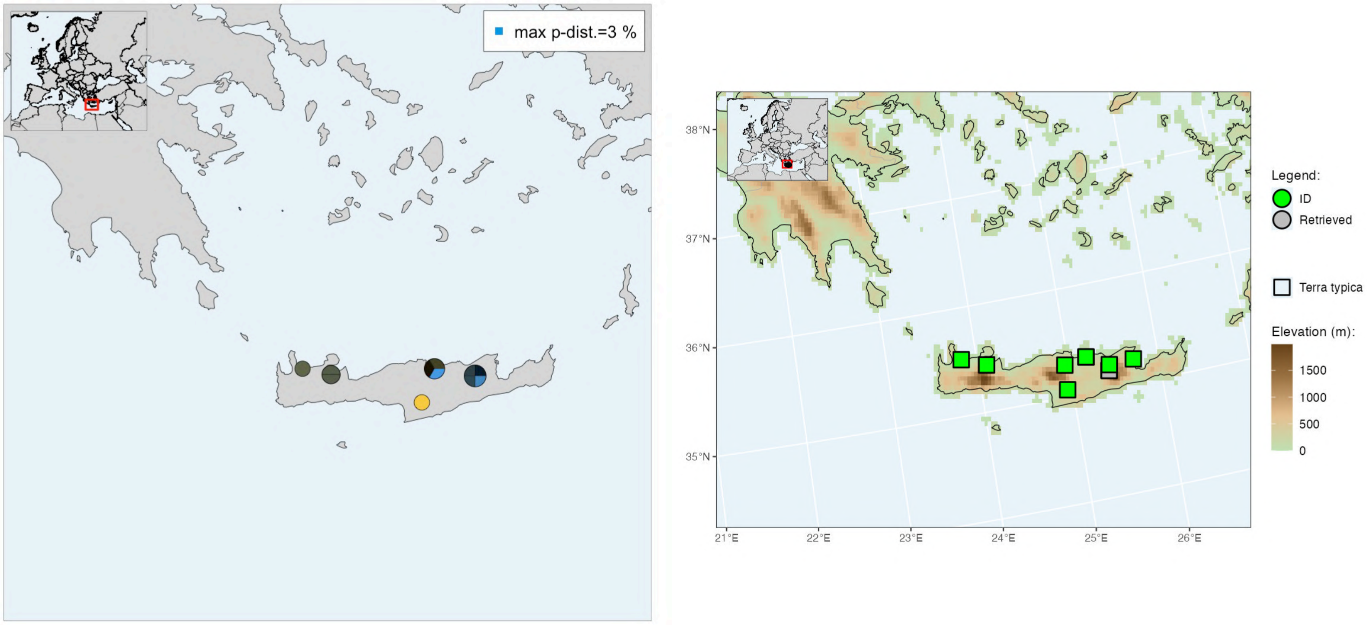

Figure 60: Genetic diversity map of *Aphaenogaster simonellii* Emery, 1894. Nearby localities of sequenced specimens are merged in pies (left). Colours match the bidimensional colour space of the PCoA projection (Fig. 60 left) of p-dist between sequences (dots). Specimen identification (ID or cf.) and source (newly sequenced or retrieved) are represented by colours, while specimen attribute (terra typica, type locality, type specimen or faunistic novelty) is represented by the shape (right). Sequences: ID = 11, cf. = 0; maximum p-distance: strict = 3 %, less strict = 3 %.

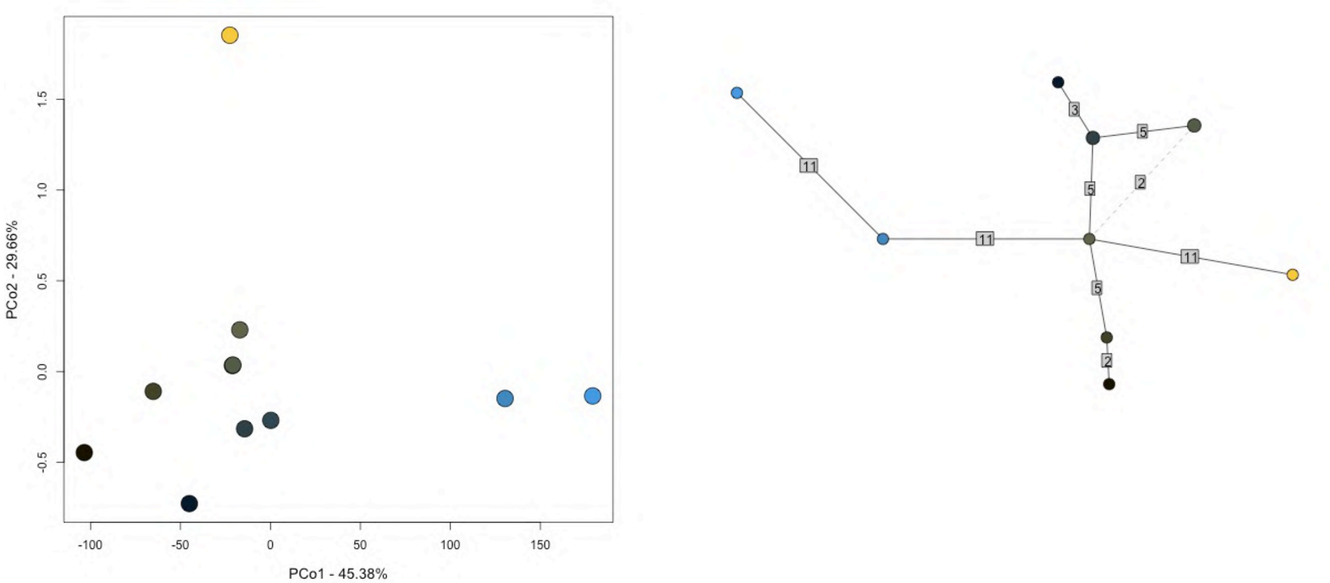

Figure 61: PCoA based on pairwise p-distances between *Aphaenogaster simonellii* sequences (left). Colours match a bidimensional colour space. Haplotype network of *Aphaenogaster simonellii* (right). Sequences > 599 bp: ID = 11, cf. = 0.

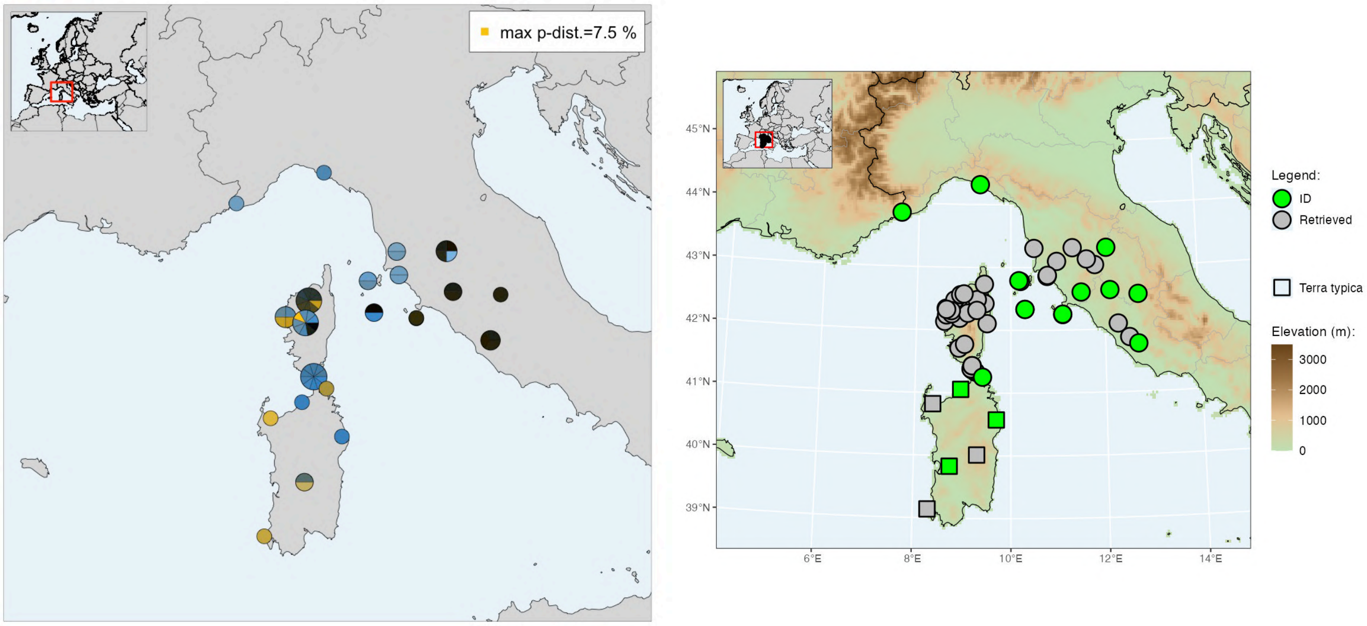

Figure 62: Genetic diversity map of *Aphaenogaster spinosa* Emery, 1878. Nearby localities of sequenced specimens are merged in pies (left). Colours match the bidimensional colour space of the PCoA projection (Fig. 62 left) of p-dist between sequences (dots). Specimen identification (ID or cf.) and source (newly sequenced or retrieved) are represented by colours, while specimen attribute (terra typica, type locality, type specimen or faunistic novelty) is represented by the shape (right). Sequences: ID = 61, cf. = 0; maximum p-distance: strict = 7.5 %, less strict = 7.5 %.

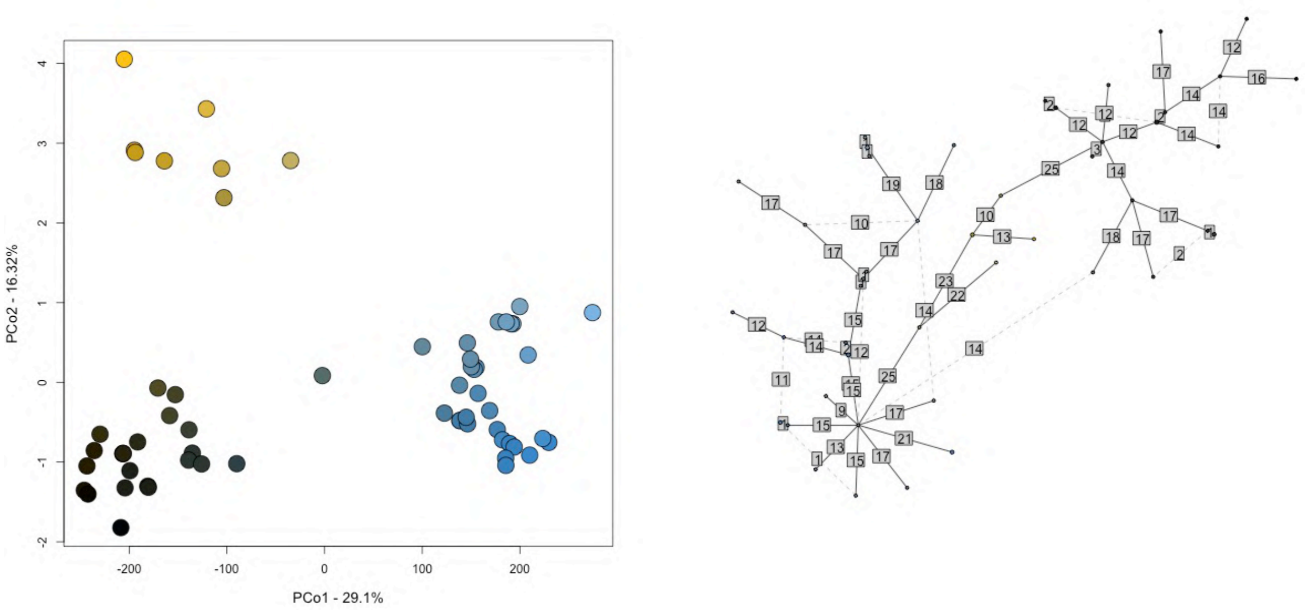

Figure 63: PCoA based on pairwise p-distances between *Aphaenogaster spinosa* sequences (left). Colours match a bidimensional colour space. Haplotype network of *Aphaenogaster spinosa* (right). Sequences > 599 bp: ID = 56, cf. = 0.

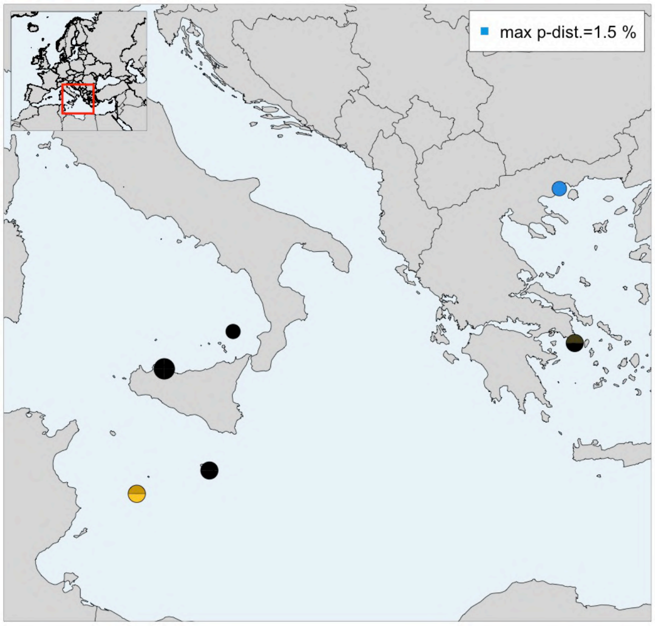

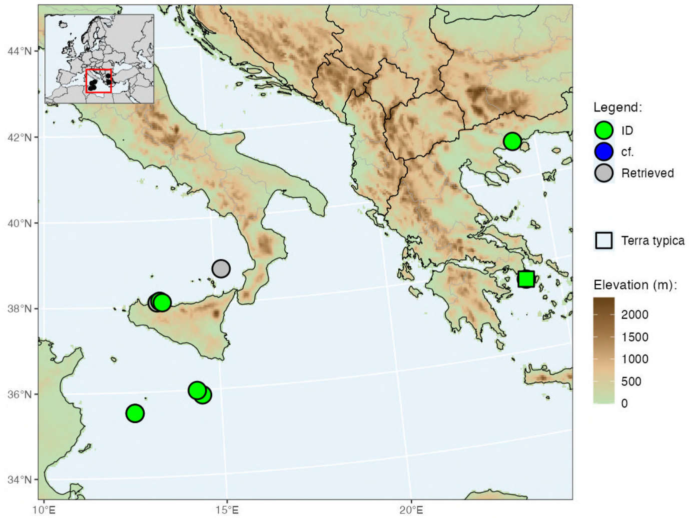

Figure 64: Genetic diversity map of *Aphaenogaster splendida* (Roger, 1859). Nearby localities of sequenced specimens are merged in pies (left). Colours match the bidimensional colour space of the PCoA projection (Fig. 64 left) of p-dist between sequences (dots). Specimen identification (ID or cf.) and source (newly sequenced or retrieved) are represented by colours, while specimen attribute (terra typica, type locality, type specimen or faunistic novelty) is represented by the shape (right). Sequences: ID = 11, cf. = 1; maximum p-distance: strict = 1.5 %, less strict = 1.5 %.

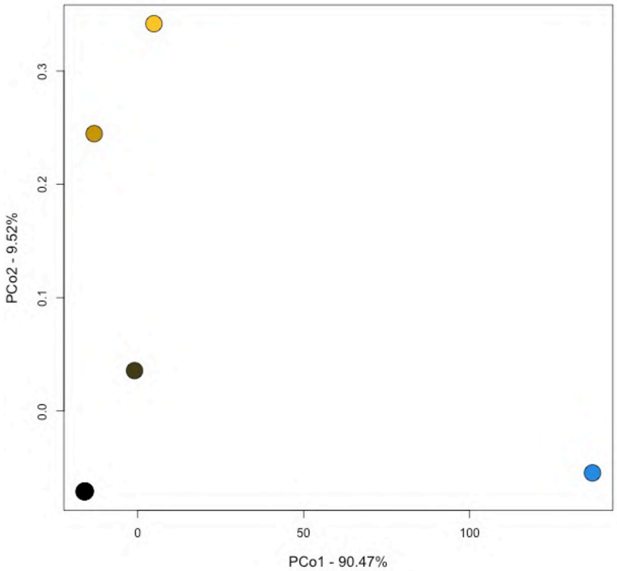

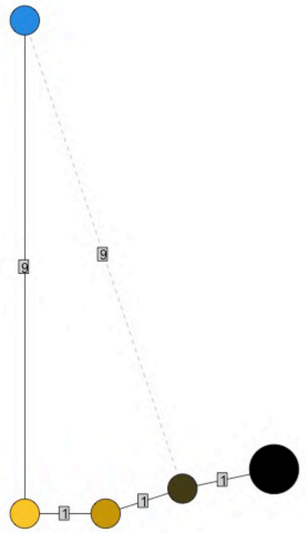

Figure 65: PCoA based on pairwise p-distances between *Aphaenogaster splendida* sequences (left). Colours match a bidimensional colour space. Haplotype network of *Aphaenogaster splendida* (right). Sequences > 599 bp: ID = 11, cf. = 1.

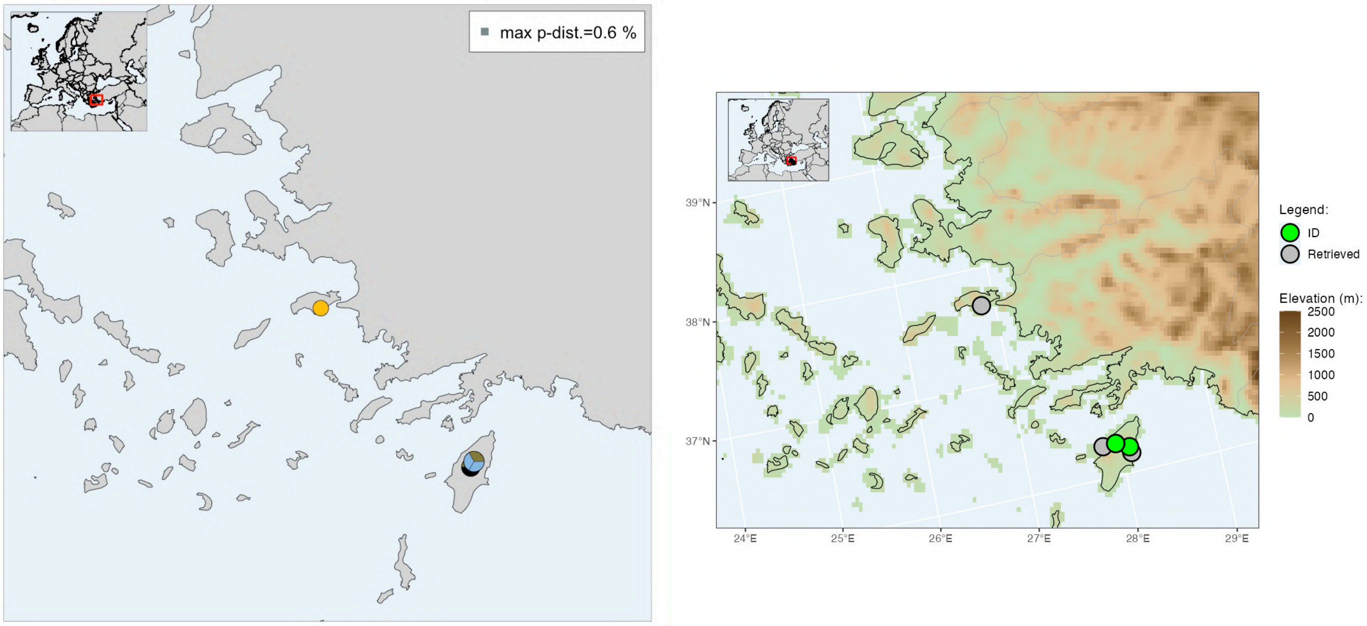

Figure 66: Genetic diversity map of *Aphaenogaster sporadis* Santschi, 1933. Nearby localities of sequenced specimens are merged in pies (left). Colours match the bidimensional colour space of the PCoA projection (Fig. 66 left) of p-dist between sequences (dots). Specimen identification (ID or cf.) and source (newly sequenced or retrieved) are represented by colours, while specimen attribute (terra typica, type locality, type specimen or faunistic novelty) is represented by the shape (right). Sequences: ID = 6, cf. = 0; maximum p-distance: strict = 0.6 %, less strict = 0.6 %.

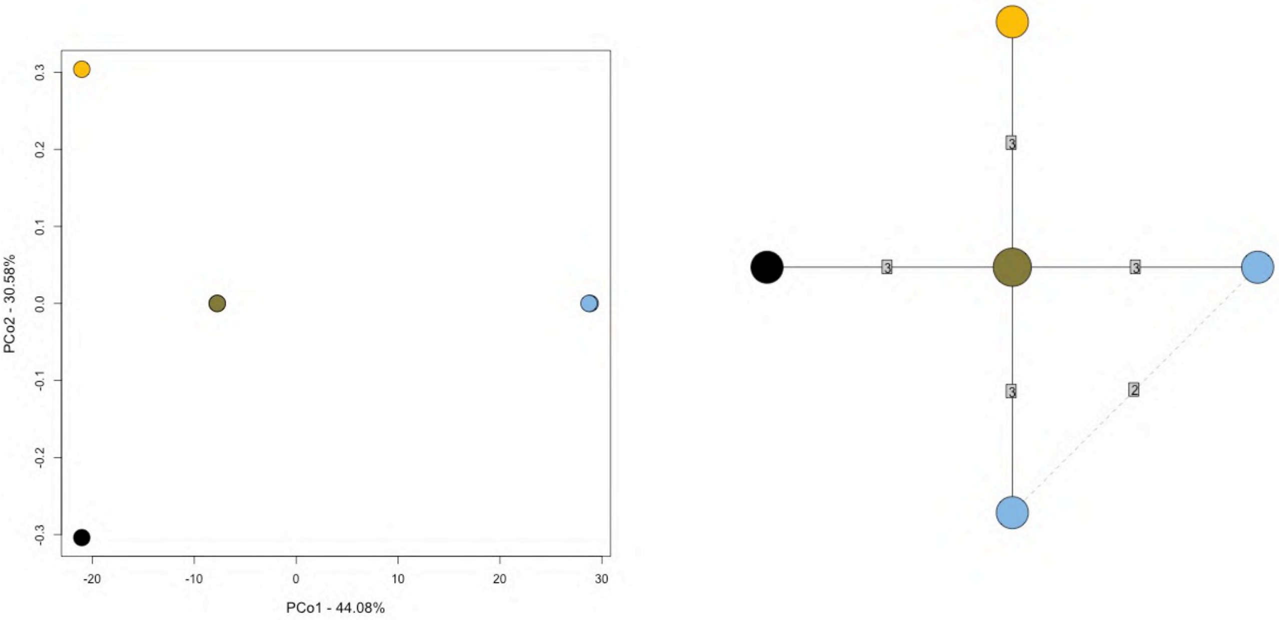

Figure 67: PCoA based on pairwise p-distances between *Aphaenogaster sporadis* sequences (left). Colours match a bidimensional colour space. Haplotype network of *Aphaenogaster sporadis* (right). Sequences > 599 bp: ID = 6, cf. = 0.

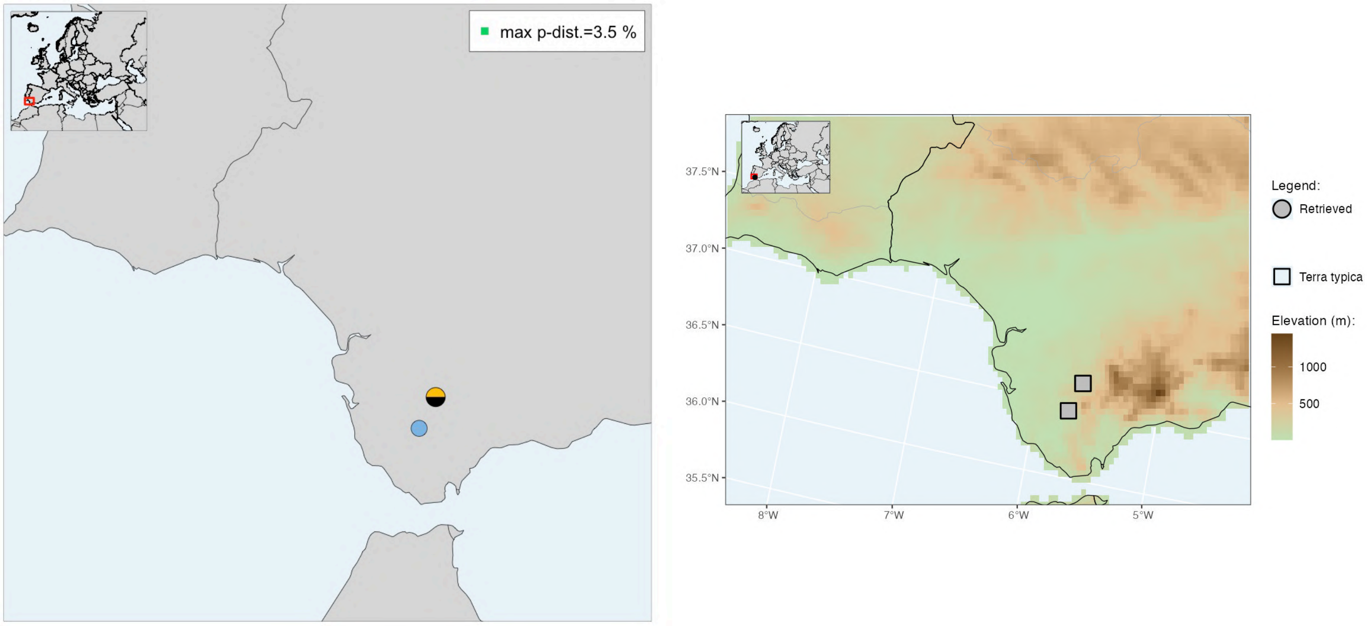

Figure 68: Genetic diversity map of *Aphaenogaster striativentris* Forel, 1895. Nearby localities of sequenced specimens are merged in pies (left). Colours match the bidimensional colour space of the PCoA projection (Fig. 68 left) of p-dist between sequences (dots). Specimen identification (ID or cf.) and source (newly sequenced or retrieved) are represented by colours, while specimen attribute (terra typica, type locality, type specimen or faunistic novelty) is represented by the shape (right). Sequences: ID = 3, cf. = 0; maximum p-distance: strict = 3.5 %, less strict = 3.5 %.

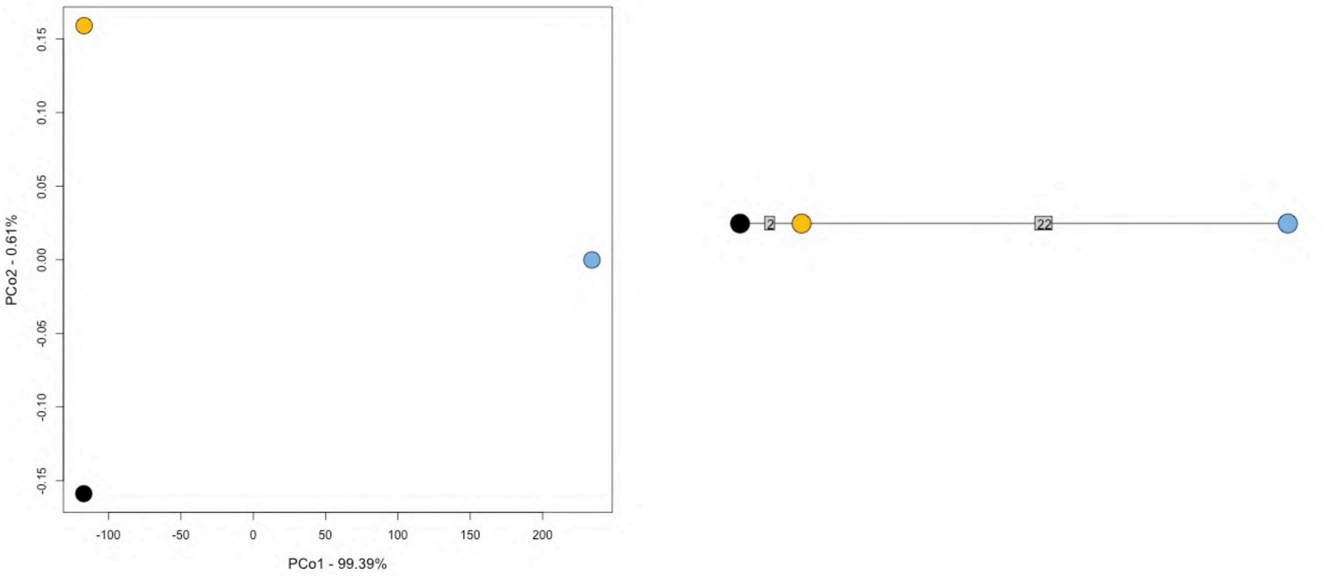

Figure 69: PCoA based on pairwise p-distances between *Aphaenogaster striativentris* sequences (left). Colours match a bidimensional colour space. Haplotype network of *Aphaenogaster striativentris* (right). Sequences > 599 bp: ID = 3, cf. = 0.

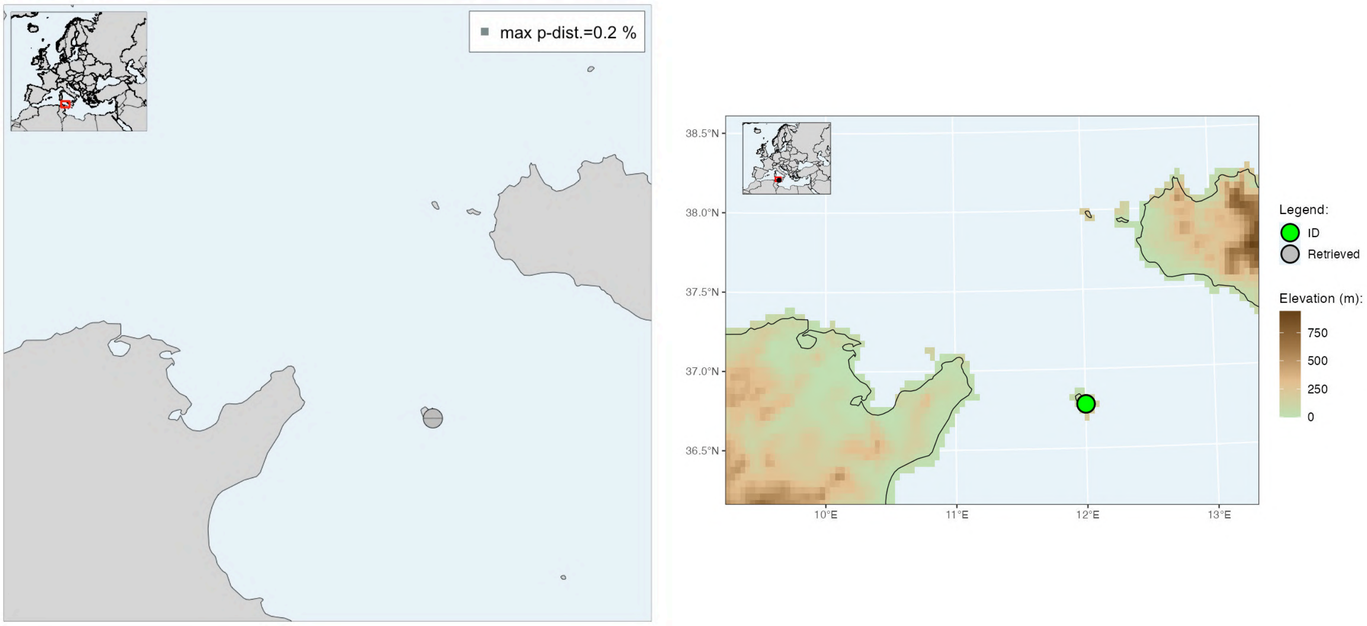

Figure 70: Genetic diversity map of *Aphaenogaster strioloides* Forel, 1890. PCoA projection was not done and therefore sequenced specimens in the genetic diversity map are coloured in gray (left). Specimen identification (ID or cf.) and source (newly sequenced or retrieved) are represented by colours, while specimen attribute (terra typica, type locality, type specimen or faunistic novelty) is represented by the shape (right). Sequences: ID = 2, cf. = 0; maximum p-distance: strict = NA, less strict = 0.2 %.

Haplotype network analysis of *Aphaenogaster strioloides* was not possible.

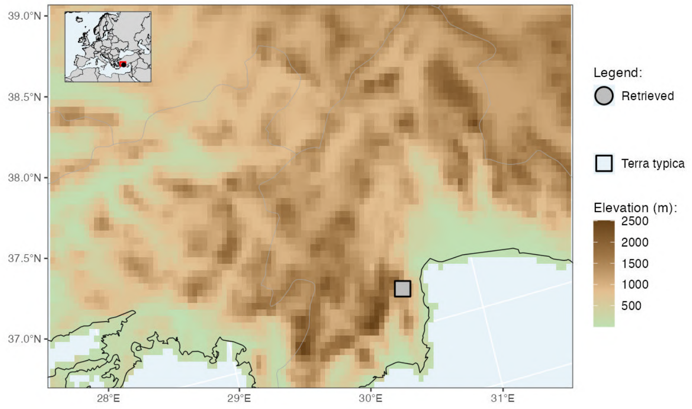

Figure 71: Map of *Aphaenogaster subcostata* Viehmeyer, 1922. Due to the presence of a single sequence, the genetic diversity map and the PCoA projection were not done. Specimen identification (ID or cf.) and source (newly sequenced or retrieved) are represented by colours, while specimen attribute (terra typica, type locality, type specimen or faunistic novelty) is represented by the shape. Sequences: ID = 1, cf. = 0; maximum p-distance: strict = NA, less strict = NA.

Haplotype network analysis of *Aphaenogaster subcostata* was not possible.

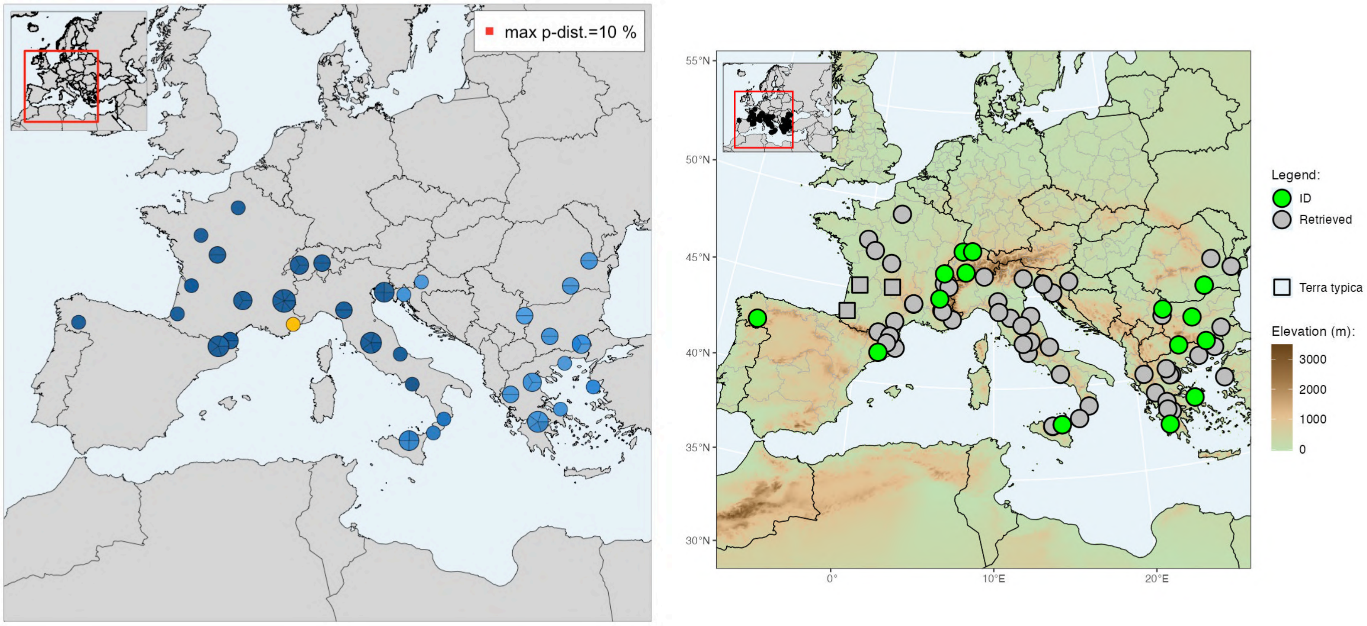

Figure 72: Genetic diversity map of *Aphaenogaster subterranea* (Latreille, 1798). Nearby localities of sequenced specimens are merged in pies (left). Colours match the bidimensional colour space of the PCoA projection (Fig. 72 left) of p-dist between sequences (dots). Specimen identification (ID or cf.) and source (newly sequenced or retrieved) are represented by colours, while specimen attribute (terra typica, type locality, type specimen or faunistic novelty) is represented by the shape (right). Sequences: ID = 75, cf. = 0; maximum p-distance: strict = 10 %, less strict = 10 %.

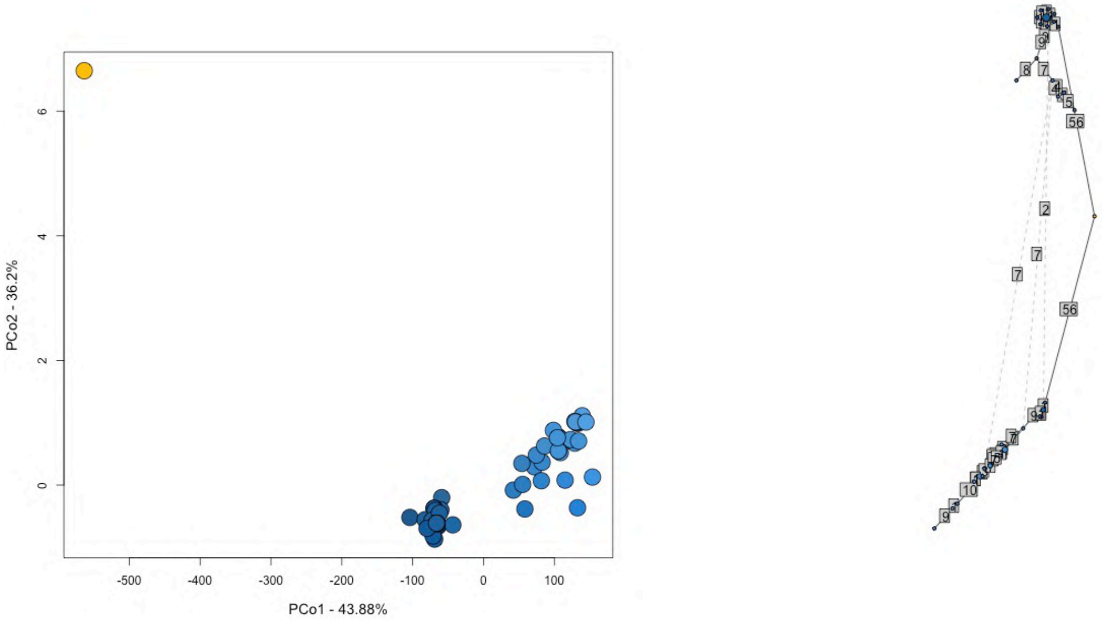

Figure 73: PCoA based on pairwise p-distances between *Aphaenogaster subterranea* sequences (left). Colours match a bidimensional colour space. Haplotype network of *Aphaenogaster subterranea* (right). Sequences > 599 bp: ID = 74, cf. = 0.

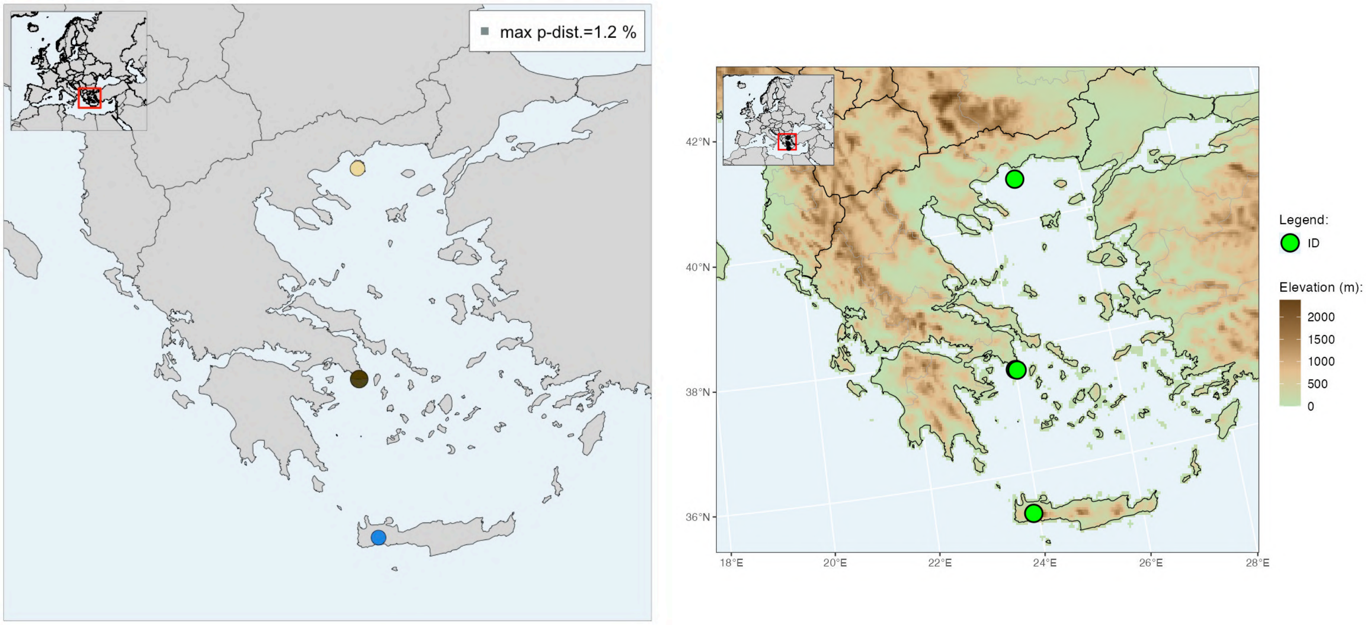

Figure 74: Genetic diversity map of *Aphaenogaster subterraneoides* Emery, 1881. Nearby localities of sequenced specimens are merged in pies (left). Colours match the bidimensional colour space of the PCoA projection (Fig. 74 left) of p-dist between sequences (dots). Specimen identification (ID or cf.) and source (newly sequenced or retrieved) are represented by colours, while specimen attribute (terra typica, type locality, type specimen or faunistic novelty) is represented by the shape (right). Sequences: ID = 4, cf. = 0; maximum p-distance: strict = 1.2 %, less strict = 1.2 %.

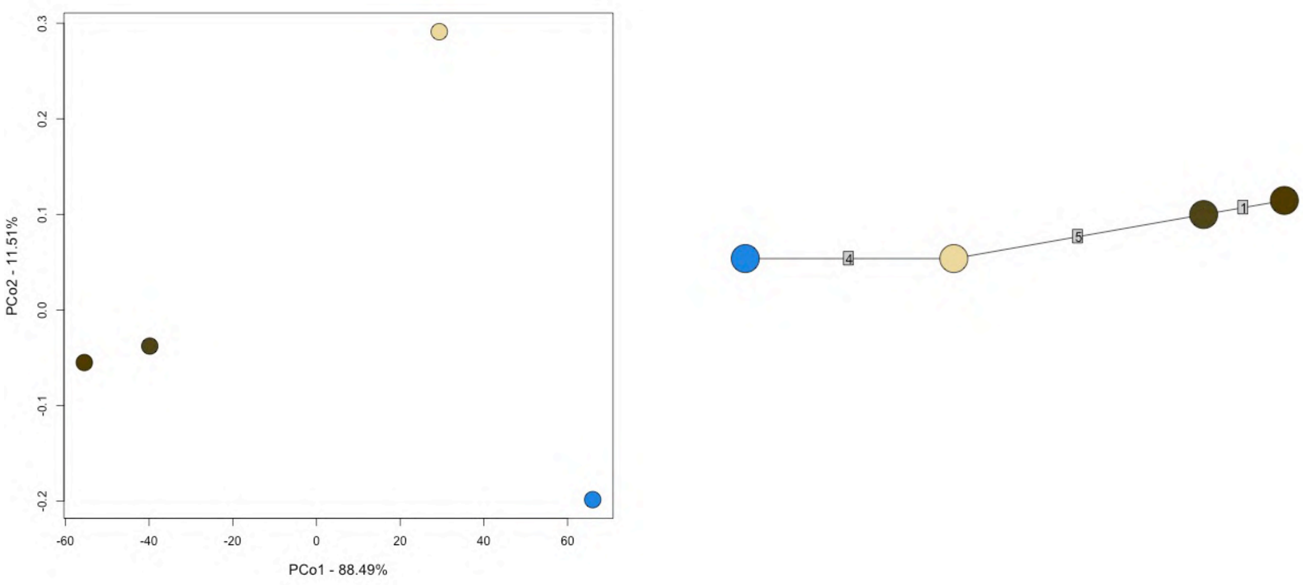

Figure 75: PCoA based on pairwise p-distances between *Aphaenogaster subterraneoides* sequences (left). Colours match a bidimensional colour space. Haplotype network of *Aphaenogaster subterraneoides* (right). Sequences > 599 bp: ID = 4, cf. = 0.

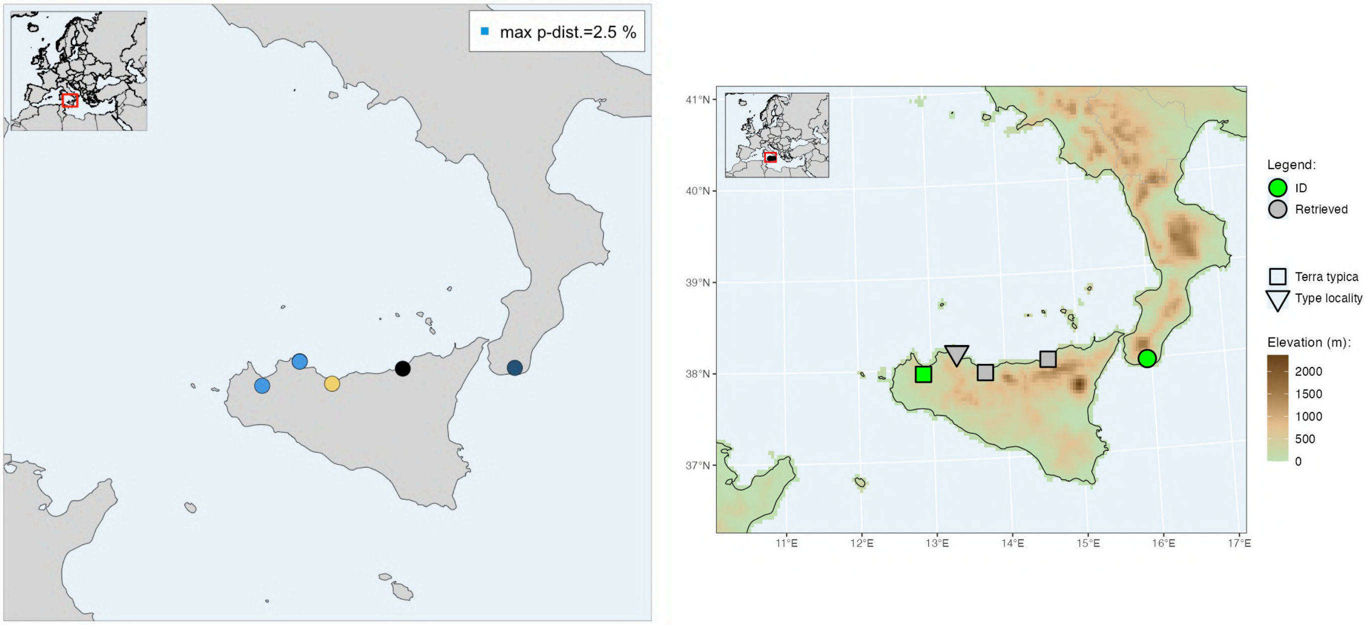

Figure 76: Genetic diversity map of *Aphaenogaster subterraneosplendida* Emery, 1908. Nearby localities of sequenced specimens are merged in pies (left). Colours match the bidimensional colour space of the PCoA projection (Fig. 76 left) of p-dist between sequences (dots). Specimen identification (ID or cf.) and source (newly sequenced or retrieved) are represented by colours, while specimen attribute (terra typica, type locality, type specimen or faunistic novelty) is represented by the shape (right). Sequences: ID = 6, cf. = 0; maximum p-distance: strict = 2.5 %, less strict = 2.5 %.

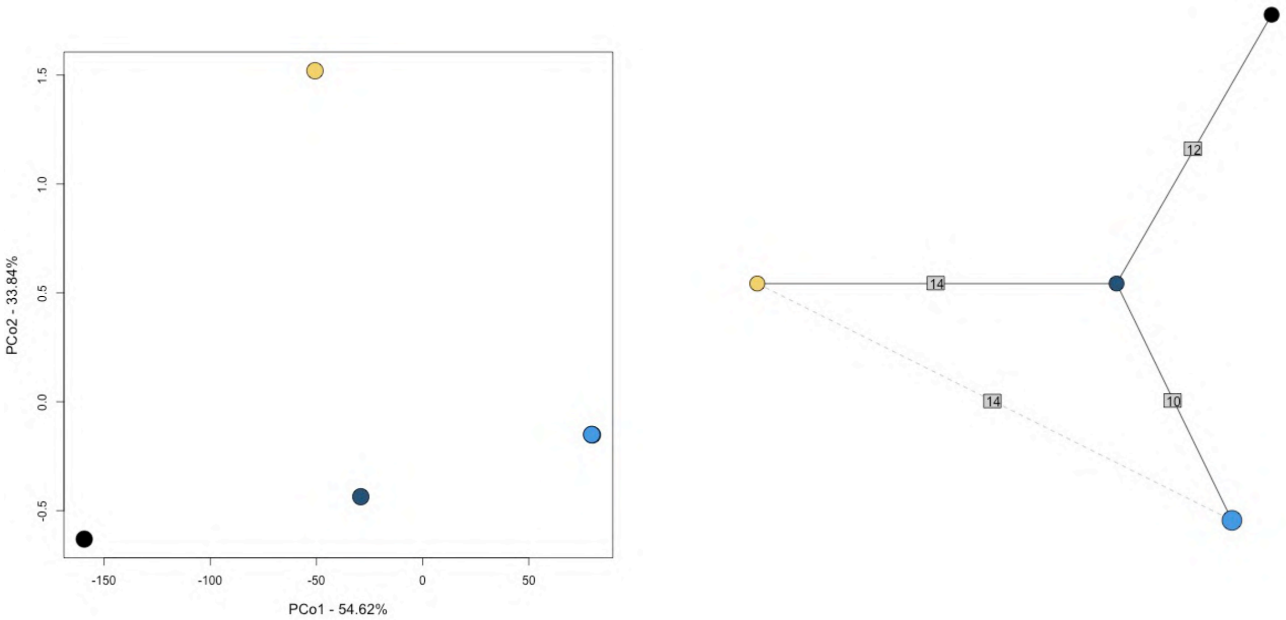

Figure 77: PCoA based on pairwise p-distances between *Aphaenogaster subterraneosplendida* sequences (left). Colours match a bidimensional colour space. Haplotype network of *Aphaenogaster subterraneosplendida* (right). Sequences > 599 bp: ID = 6, cf. = 0.

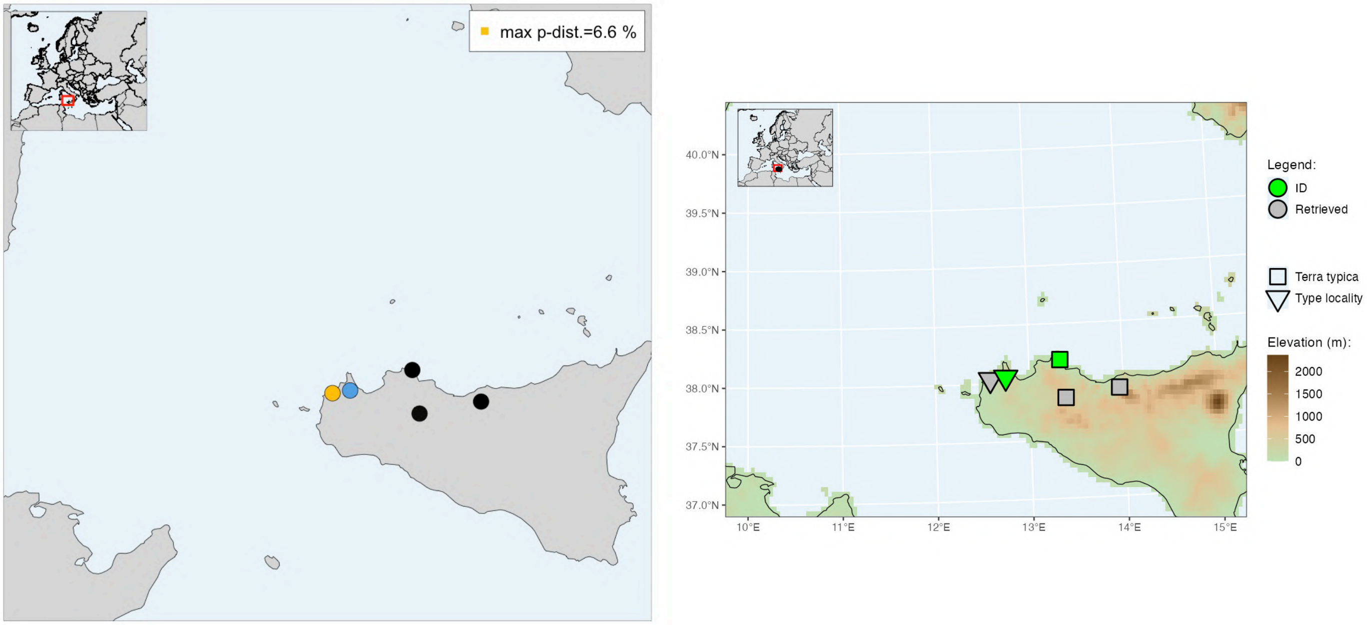

Figure 78: Genetic diversity map of *Aphaenogaster trinacriae* Alicata & Schifani, 2019. Nearby localities of sequenced specimens are merged in pies (left). Colours match the bidimensional colour space of the PCoA projection (Fig. 78 left) of p-dist between sequences (dots). Specimen identification (ID or cf.) and source (newly sequenced or retrieved) are represented by colours, while specimen attribute (terra typica, type locality, type specimen or faunistic novelty) is represented by the shape (right). Sequences: ID = 5, cf. = 0; maximum p-distance: strict = 6.6 %, less strict = 6.6 %.

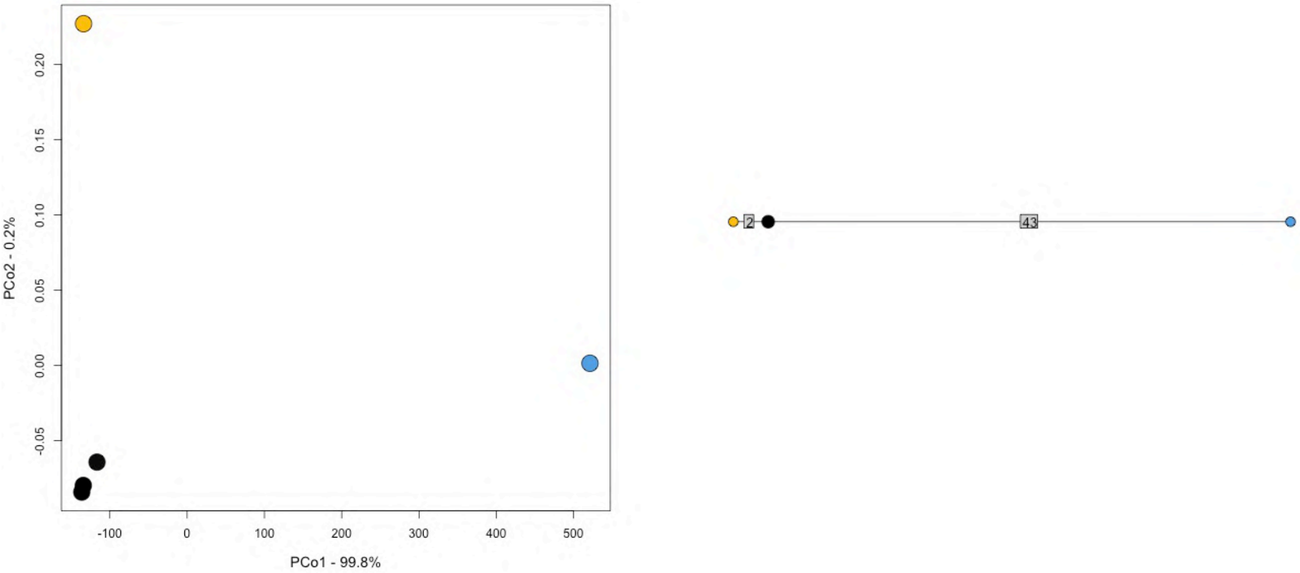

Figure 79: PCoA based on pairwise p-distances between *Aphaenogaster trinacriae* sequences (left). Colours match a bidimensional colour space. Haplotype network of *Aphaenogaster trinacriae* (right). Sequences > 599 bp: ID = 5, cf. = 0.

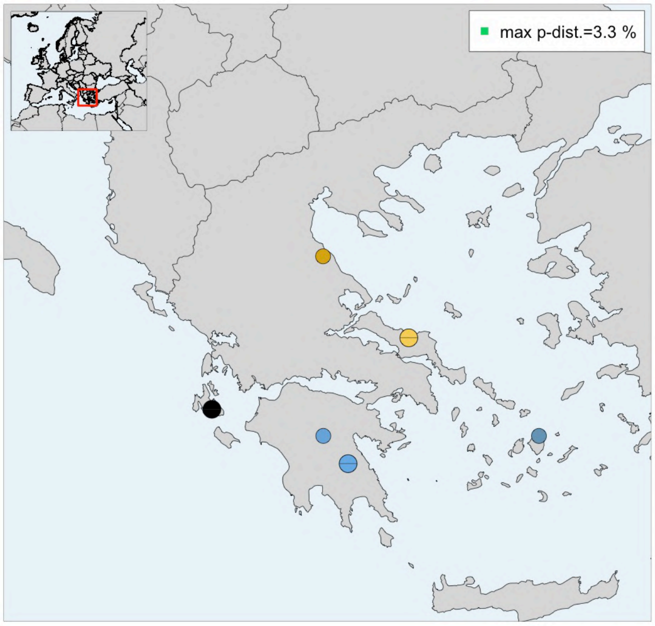

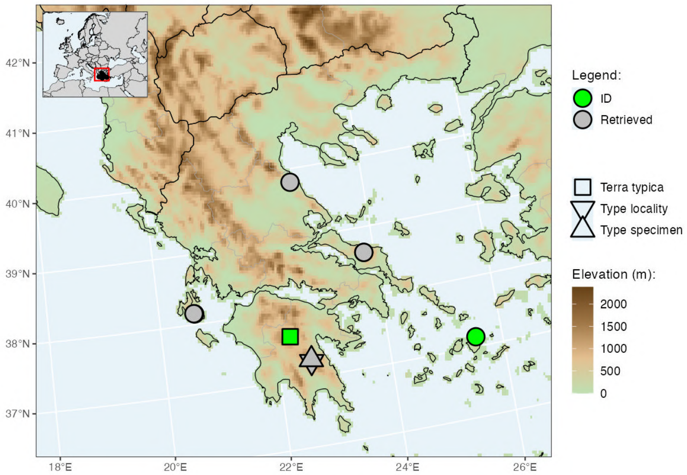

Figure 80: Genetic diversity map of *Aphaenogaster tristis* Borowiec, Menchetti, Salata, Vila & Zięcina 2024. Nearby localities of sequenced specimens are merged in pies (left). Colours match the bidimensional colour space of the PCoA projection (Fig. 80 left) of p-dist between sequences (dots). Specimen identification (ID or cf.) and source (newly sequenced or retrieved) are represented by colours, while specimen attribute (terra typica, type locality, type specimen or faunistic novelty) is represented by the shape (right). Sequences: ID = 9, cf. = 0; maximum p-distance: strict = 3.3 %, less strict = 3.3 %.

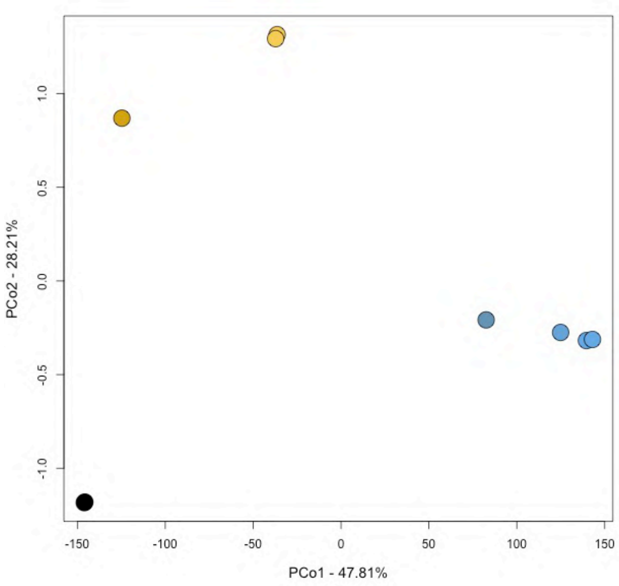

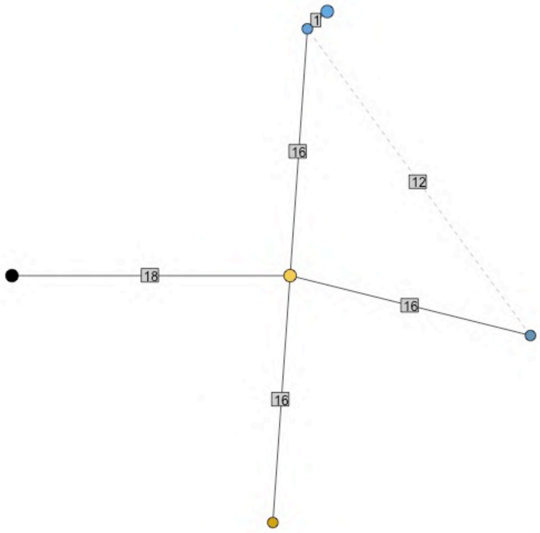

Figure 81: PCoA based on pairwise p-distances between *Aphaenogaster tristis* sequences (left). Colours match a bidimensional colour space. Haplotype network of *Aphaenogaster tristis* (right). Sequences > 599 bp: ID = 9, cf. = 0.

Aphaenogaster ulibeli

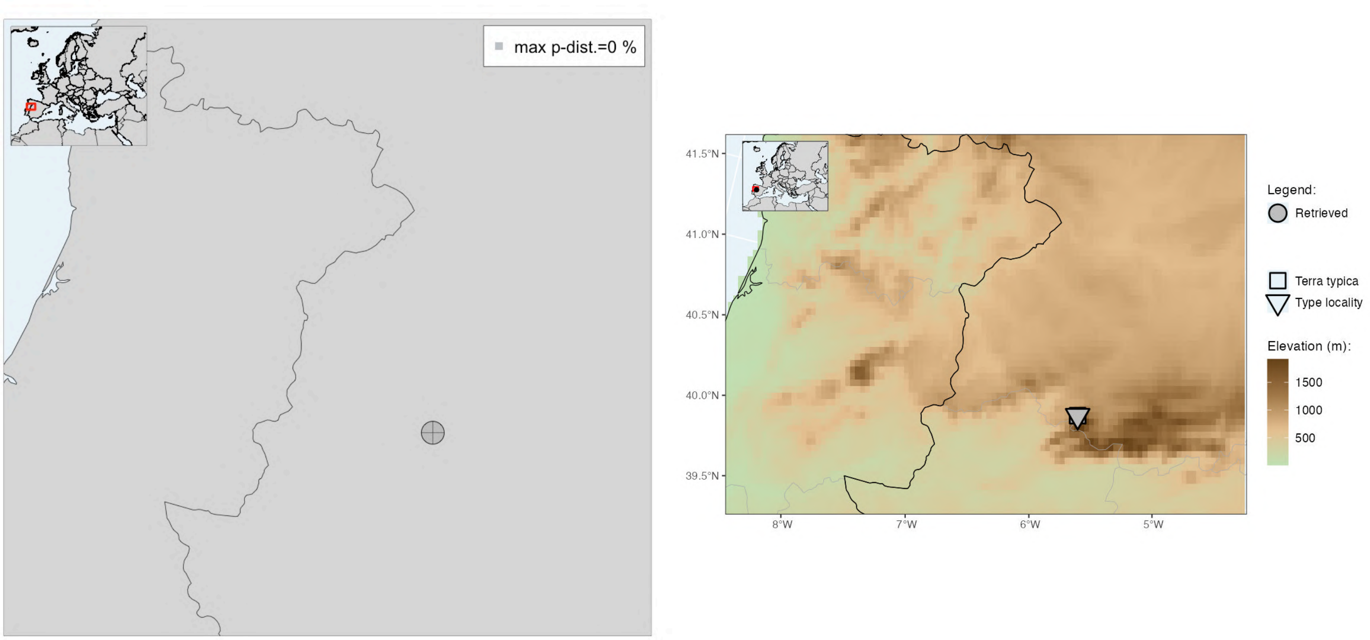

Figure 82: Genetic diversity map of *Aphaenogaster ulibeli* Gomez & Espadaler, 2018. PCoA projection was not done and therefore sequenced specimens in the genetic diversity map are coloured in gray (left). Specimen identification (ID or cf.) and source (newly sequenced or retrieved) are represented by colours, while specimen attribute (terra typica, type locality, type specimen or faunistic novelty) is represented by the shape (right). Sequences: ID = 4, cf. = 0; maximum p-distance: strict = 0 %, less strict = 0 %.

Haplotype network analysis of *Aphaenogaster ulibeli* was not possible.

Bothriomyrmex

Bothriomyrmex atlantis

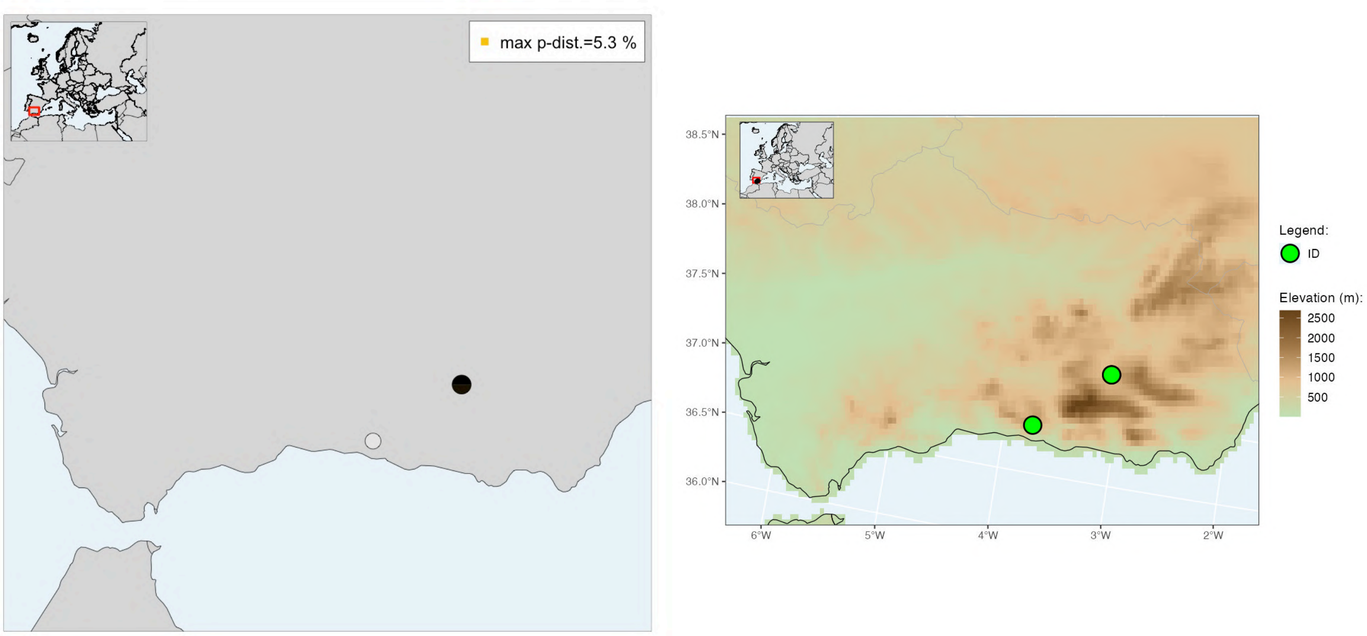

Figure 83: Genetic diversity map of *Bothriomyrmex atlantis* Santschi, 1922. Nearby localities of sequenced specimens are merged in pies (left). Colours match the bidimensional colour space of the PCoA projection (Fig. 83 left) of p-dist between sequences (dots). Specimen identification (ID or cf.) and source (newly sequenced or retrieved) are represented by colours, while specimen attribute (terra typica, type locality, type specimen or faunistic novelty) is represented by the shape (right). Sequences: ID = 3, cf. = 0; maximum p-distance: strict = 5.3 %, less strict = 5.3 %.

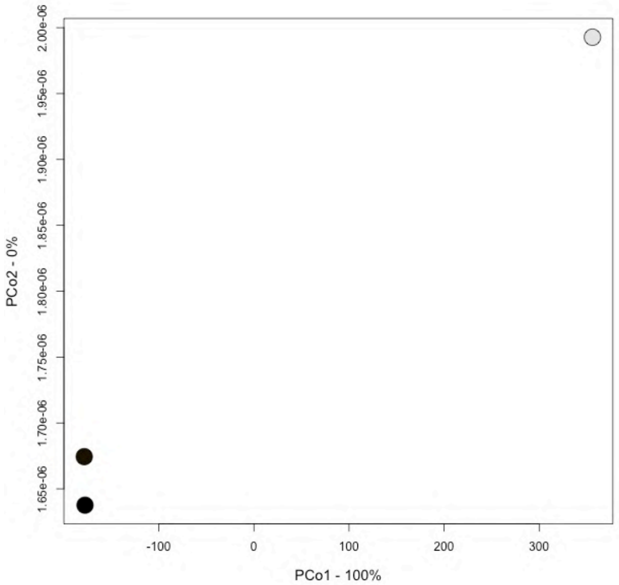

Figure 84: PCoA based on pairwise p-distances between *Bothriomyrmex atlantis* sequences (left). Colours match a bidimensional colour space. Haplotype network analysis of *Bothriomyrmex atlantis* was not possible.

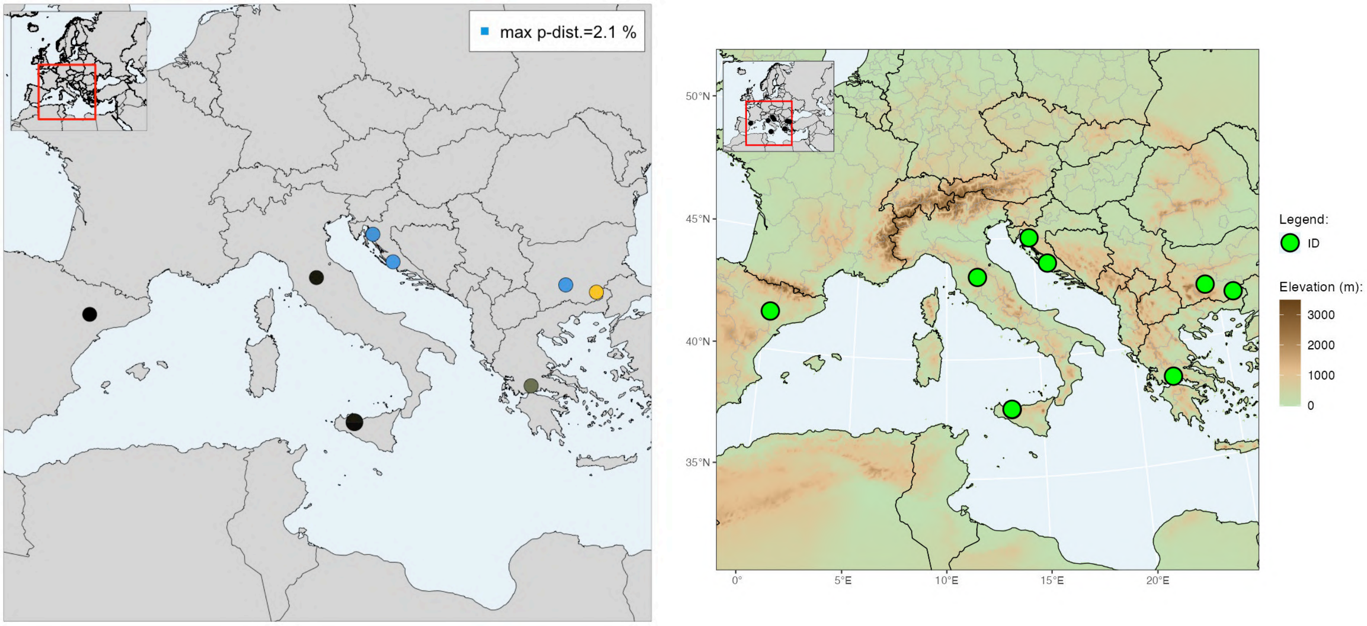

Figure 85: Genetic diversity map of *Bothriomyrmex communista* Santschi, 1919. Nearby localities of sequenced specimens are merged in pies (left). Colours match the bidimensional colour space of the PCoA projection (Fig. 85 left) of p-dist between sequences (dots). Specimen identification (ID or cf.) and source (newly sequenced or retrieved) are represented by colours, while specimen attribute (terra typica, type locality, type specimen or faunistic novelty) is represented by the shape (right). Sequences: ID = 9, cf. = 0; maximum p-distance: strict = 2.1 %, less strict = 2.1 %.

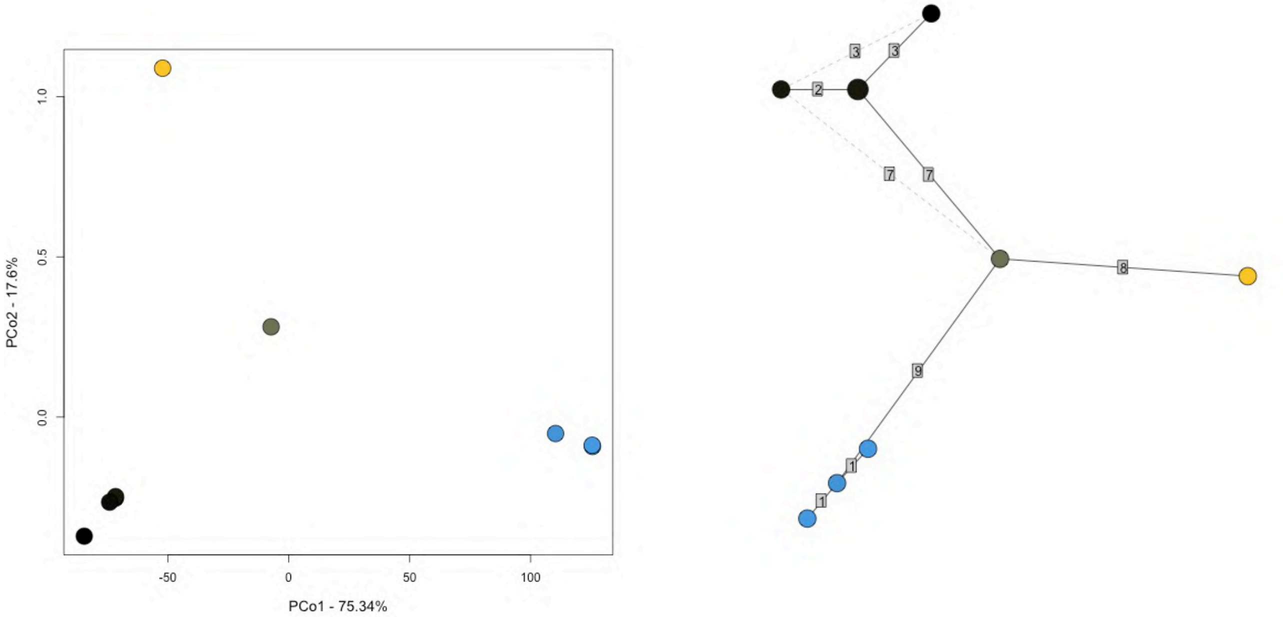

Figure 86: PCoA based on pairwise p-distances between *Bothriomyrmex communista* sequences (left). Colours match a bidimensional colour space. Haplotype network of *Bothriomyrmex communista* (right). Sequences > 599 bp: ID = 9, cf. = 0.

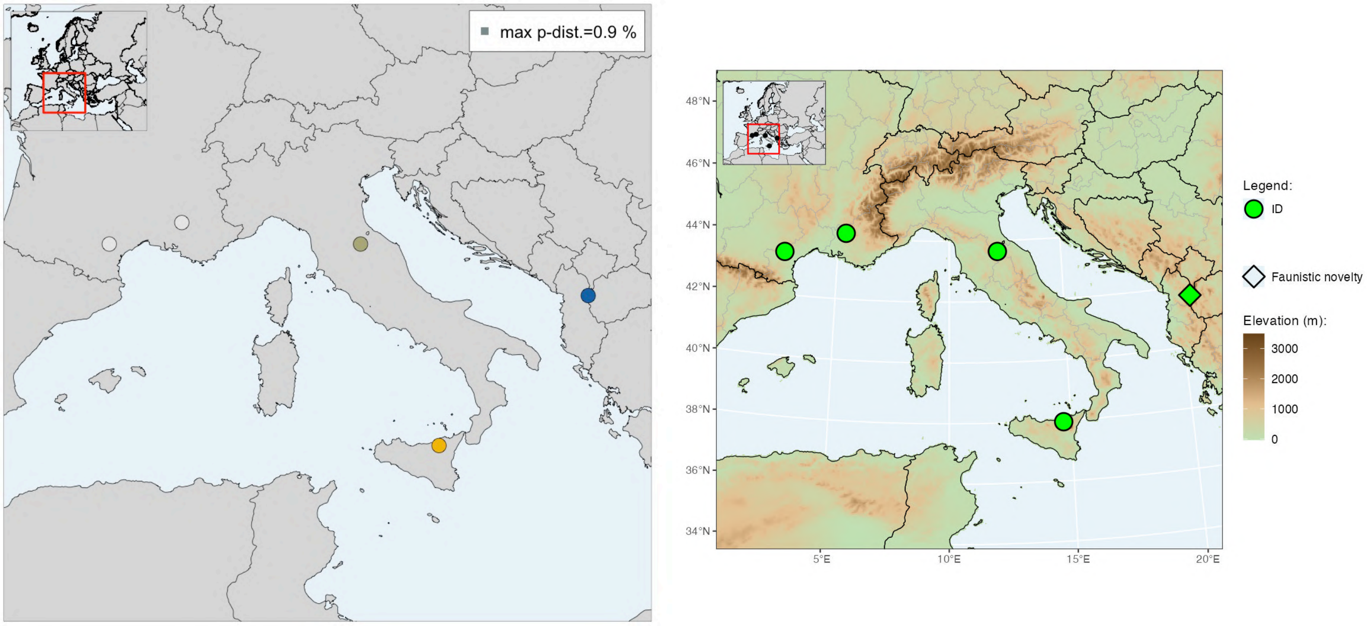

Figure 87: Genetic diversity map of *Bothriomyrmex corsicus* Santschi, 1923. Nearby localities of sequenced specimens are merged in pies (left). Colours match the bidimensional colour space of the PCoA projection (Fig. 87 left) of p-dist between sequences (dots). Specimen identification (ID or cf.) and source (newly sequenced or retrieved) are represented by colours, while specimen attribute (terra typica, type locality, type specimen or faunistic novelty) is represented by the shape (right). Sequences: ID = 6, cf. = 0; maximum p-distance: strict = 0.9 %, less strict = 0.9 %.

The species is reported for the first time in Albania.

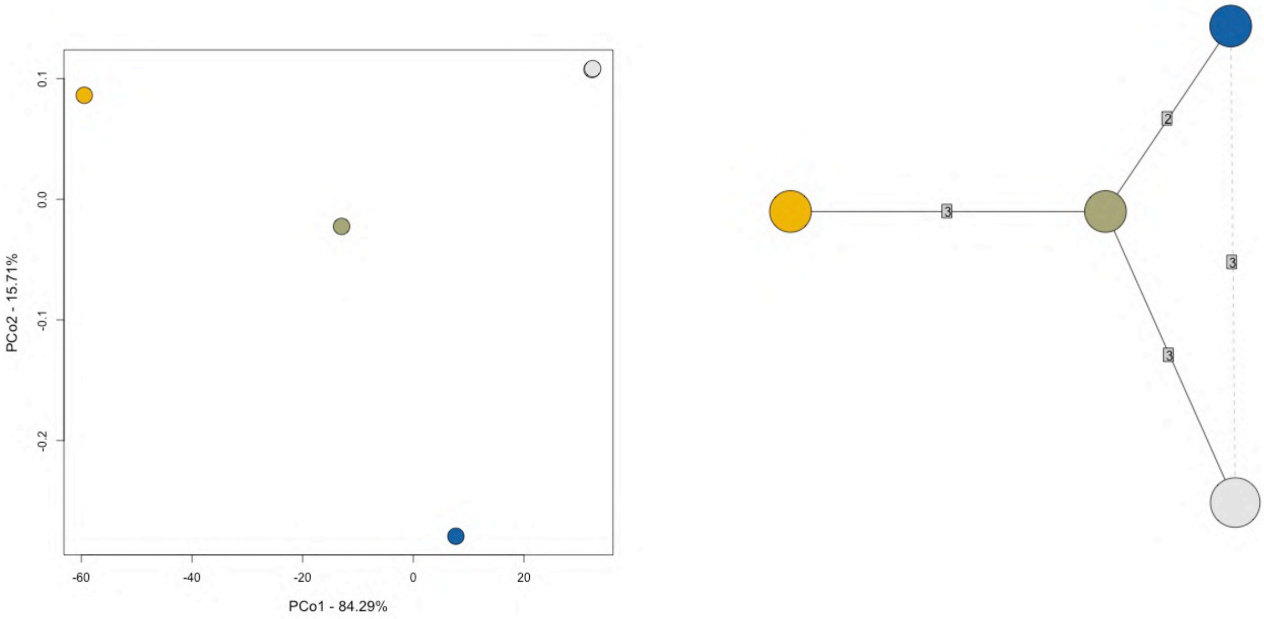

Figure 88: PCoA based on pairwise p-distances between *Bothriomyrmex corsicus* sequences (left). Colours match a bidimensional colour space. Haplotype network of *Bothriomyrmex corsicus* (right). Sequences > 599 bp: ID = 6, cf. = 0.

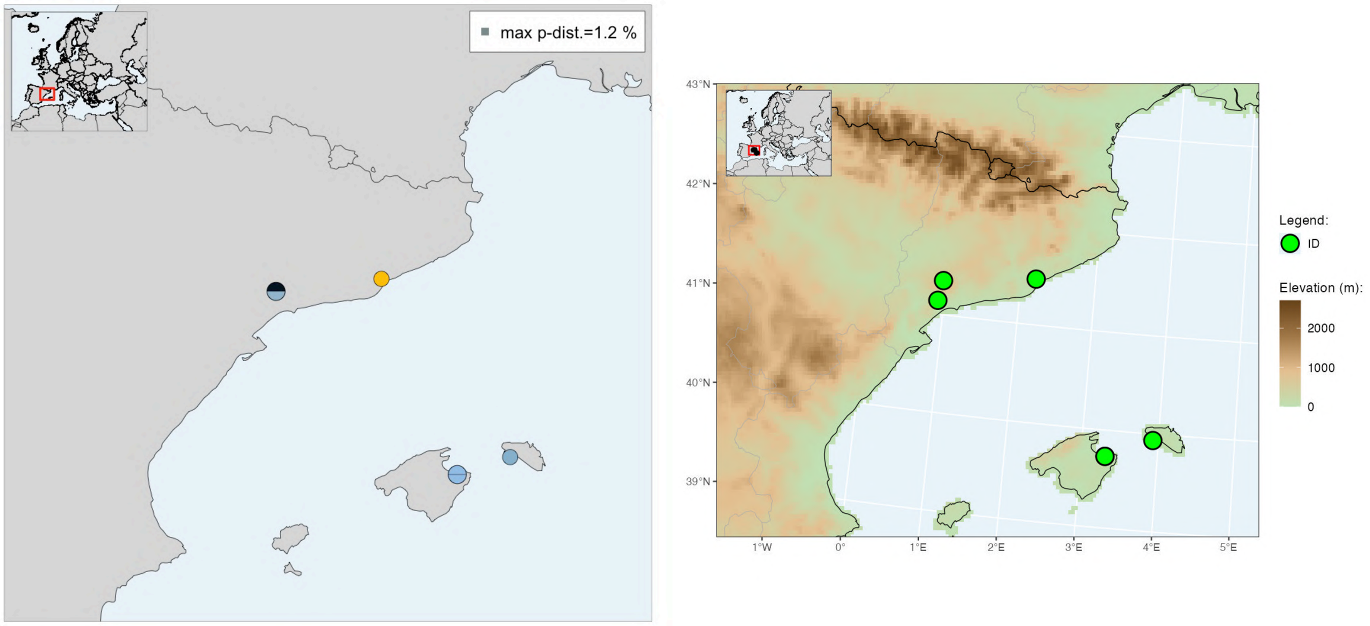

Figure 89: Genetic diversity map of *Bothriomyrmex meridionalis* (Roger, 1863). Nearby localities of sequenced specimens are merged in pies (left). Colours match the bidimensional colour space of the PCoA projection (Fig. 89 left) of p-dist between sequences (dots). Specimen identification (ID or cf.) and source (newly sequenced or retrieved) are represented by colours, while specimen attribute (terra typica, type locality, type specimen or faunistic novelty) is represented by the shape (right). Sequences: ID = 6, cf. = 0; maximum p-distance: strict = 1.2 %, less strict = 1.2 %.

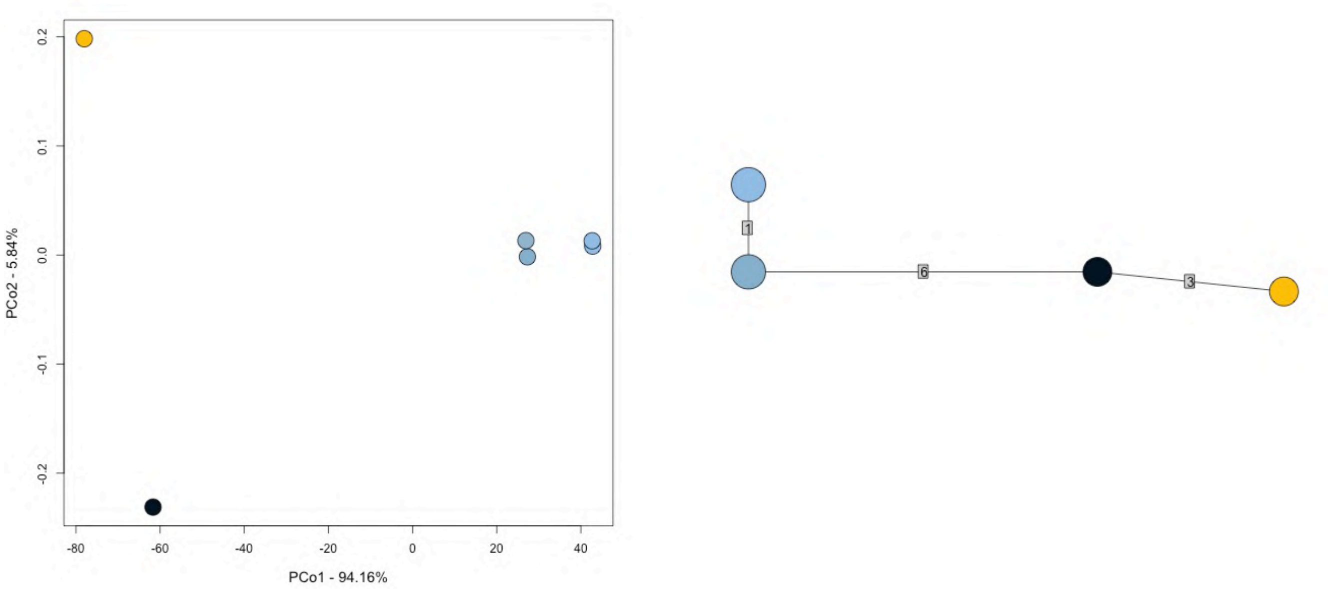

Figure 90: PCoA based on pairwise p-distances between *Bothriomyrmex meridionalis* sequences (left). Colours match a bidimensional colour space. Haplotype network of *Bothriomyrmex meridionalis* (right). Sequences > 599 bp: ID = 6, cf. = 0.

Brachymyrmex

Brachymyrmex patagonicus

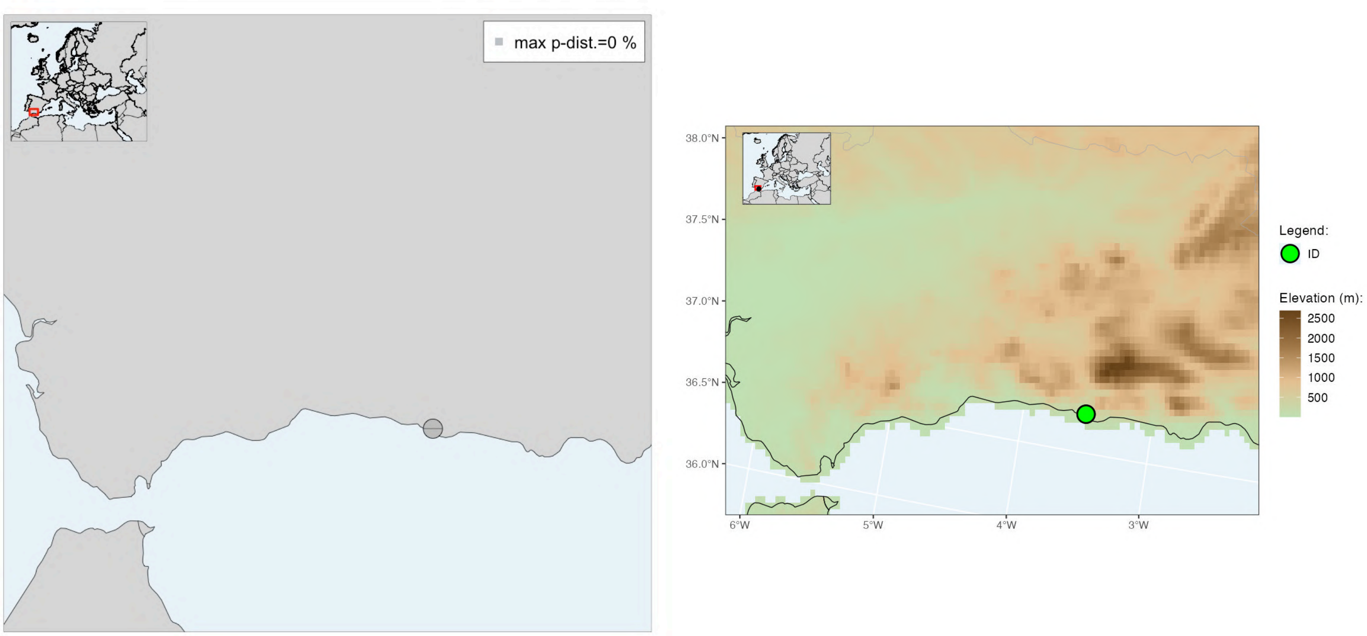

Figure 91: Genetic diversity map of *Brachymyrmex patagonicus* Mayr, 1868. PCoA projection was not done and therefore sequenced specimens in the genetic diversity map are coloured in gray (left). Specimen identification (ID or cf.) and source (newly sequenced or retrieved) are represented by colours, while specimen attribute (terra typica, type locality, type specimen or faunistic novelty) is represented by the shape (right). Sequences: ID = 3, cf. = 0; maximum p-distance: strict = NA, less strict = 0 %.

Haplotype network analysis of *Brachymyrmex patagonicus* was not possible.

Brachyponera

Brachyponera chinensis

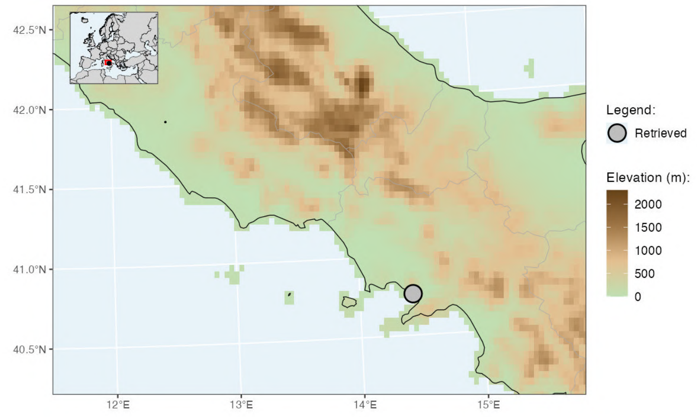

Figure 92: Map of *Brachyponera chinensis* (Emery, 1895). Due to the presence of a single sequence, the genetic diversity map and the PCoA projection were not done. Specimen identification (ID or cf.) and source (newly sequenced or retrieved) are represented by colours, while specimen attribute (terra typica, type locality, type specimen or faunistic novelty) is represented by the shape. Sequences: ID = 5, cf. = 0; maximum p-distance: strict = NA, less strict = NA.

Haplotype network analysis of *Brachyponera chinensis* was not possible.

Camponotus

Camponotus aegaeus

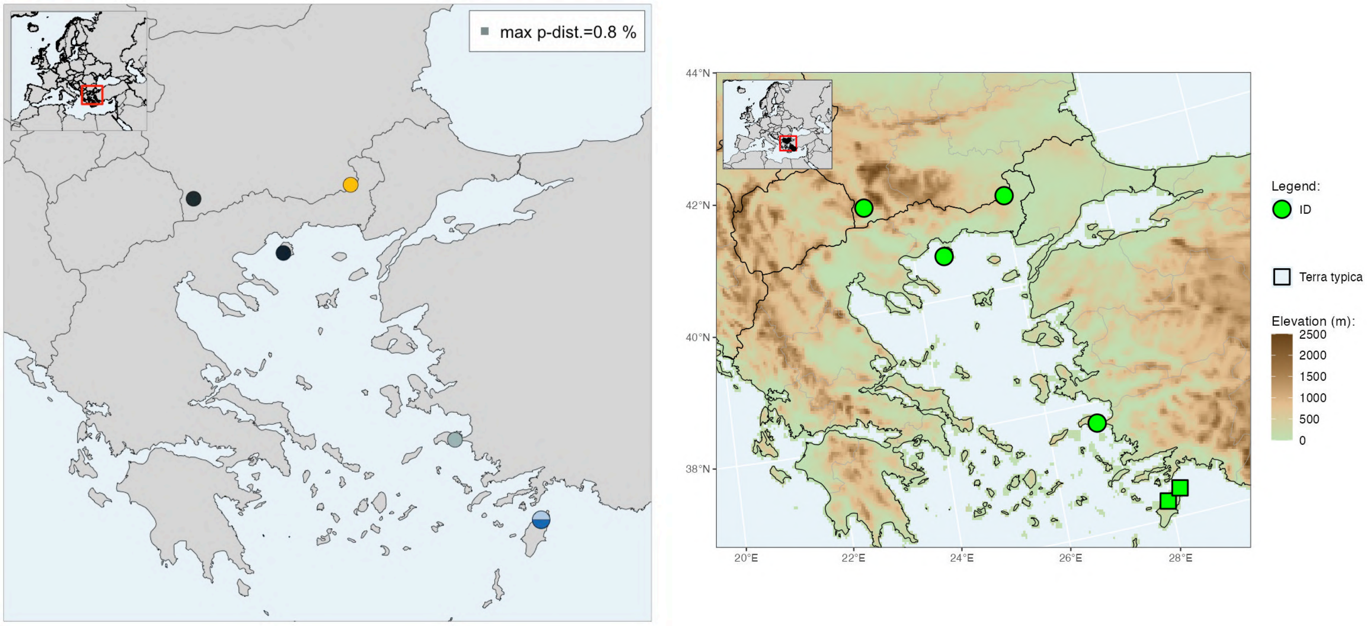

Figure 93: Genetic diversity map of *Camponotus aegaeus* Emery, 1915. Nearby localities of sequenced specimens are merged in pies (left). Colours match the bidimensional colour space of the PCoA projection (Fig. 93 left) of p-dist between sequences (dots). Specimen identification (ID or cf.) and source (newly sequenced or retrieved) are represented by colours, while specimen attribute (terra typica, type locality, type specimen or faunistic novelty) is represented by the shape (right). Sequences: ID = 6, cf. = 0; maximum p-distance: strict = 0.8 %, less strict = 0.8 %.

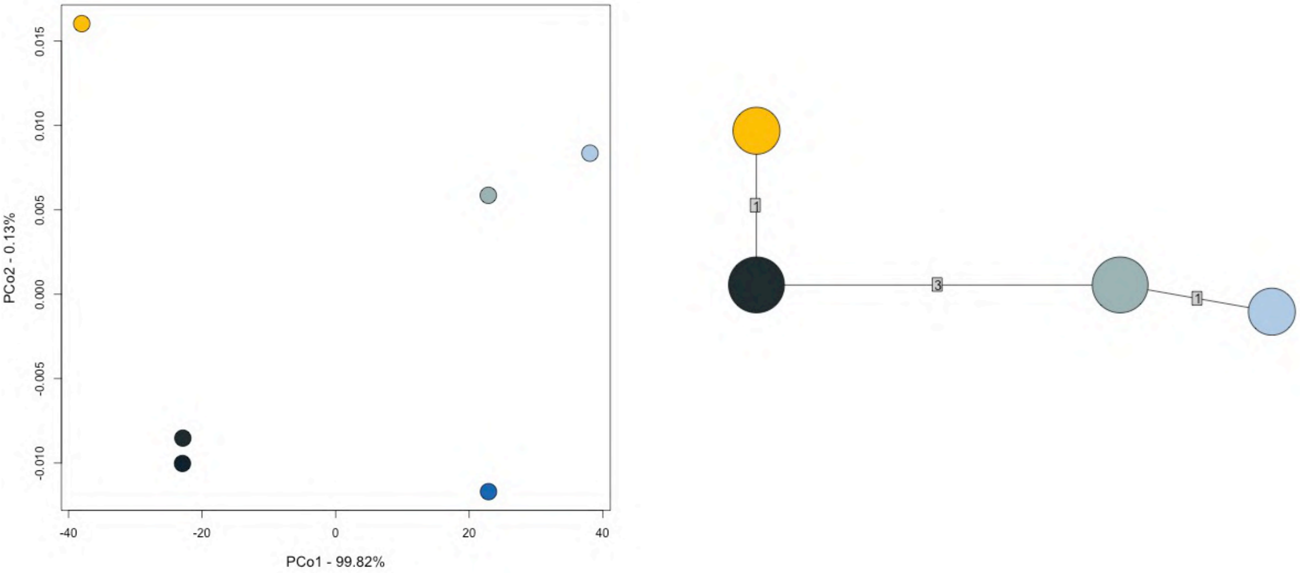

Figure 94: PCoA based on pairwise p-distances between *Camponotus aegaeus* sequences (left). Colours match a bidimensional colour space. Haplotype network of *Camponotus aegaeus* (right). Sequences > 599 bp: ID = 6, cf. = 0.

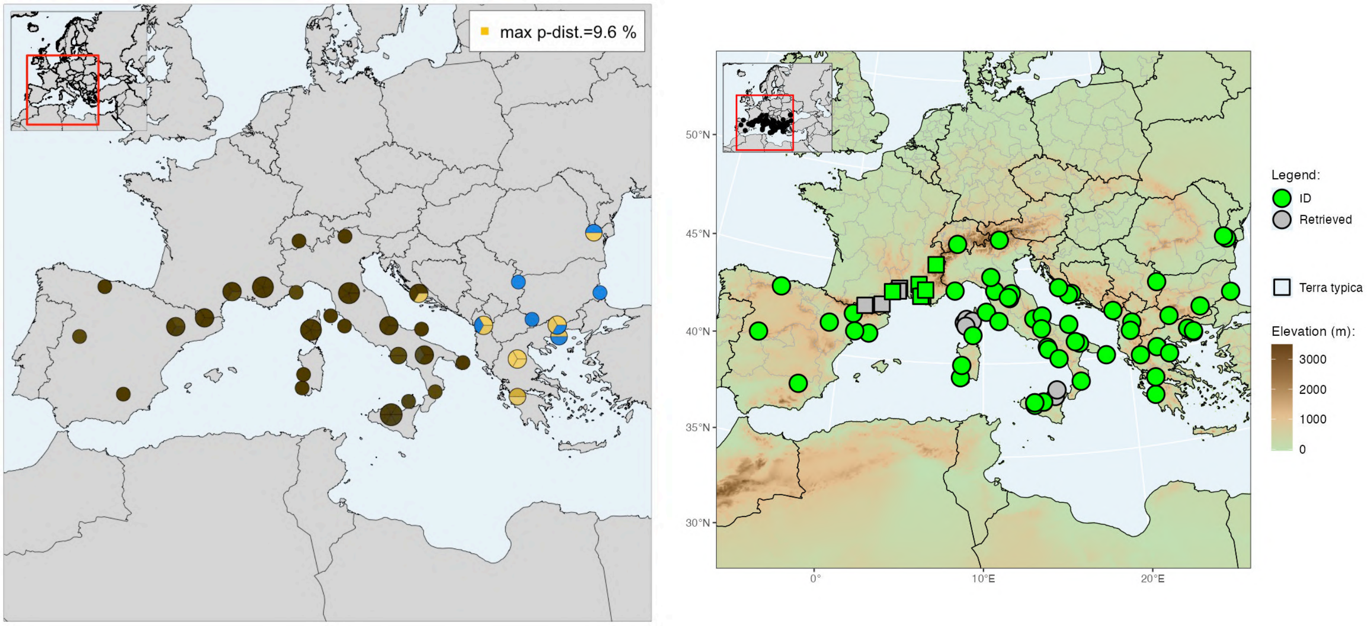

Figure 95: Genetic diversity map of *Camponotus aethiops* (Latreille, 1798). Nearby localities of sequenced specimens are merged in pies (left). Colours match the bidimensional colour space of the PCoA projection (Fig. 95 left) of p-dist between sequences (dots). Specimen identification (ID or cf.) and source (newly sequenced or retrieved) are represented by colours, while specimen attribute (terra typica, type locality, type specimen or faunistic novelty) is represented by the shape (right). Sequences: ID = 73, cf. = 0; maximum p-distance: strict = 9.6 %, less strict = 9.6 %.

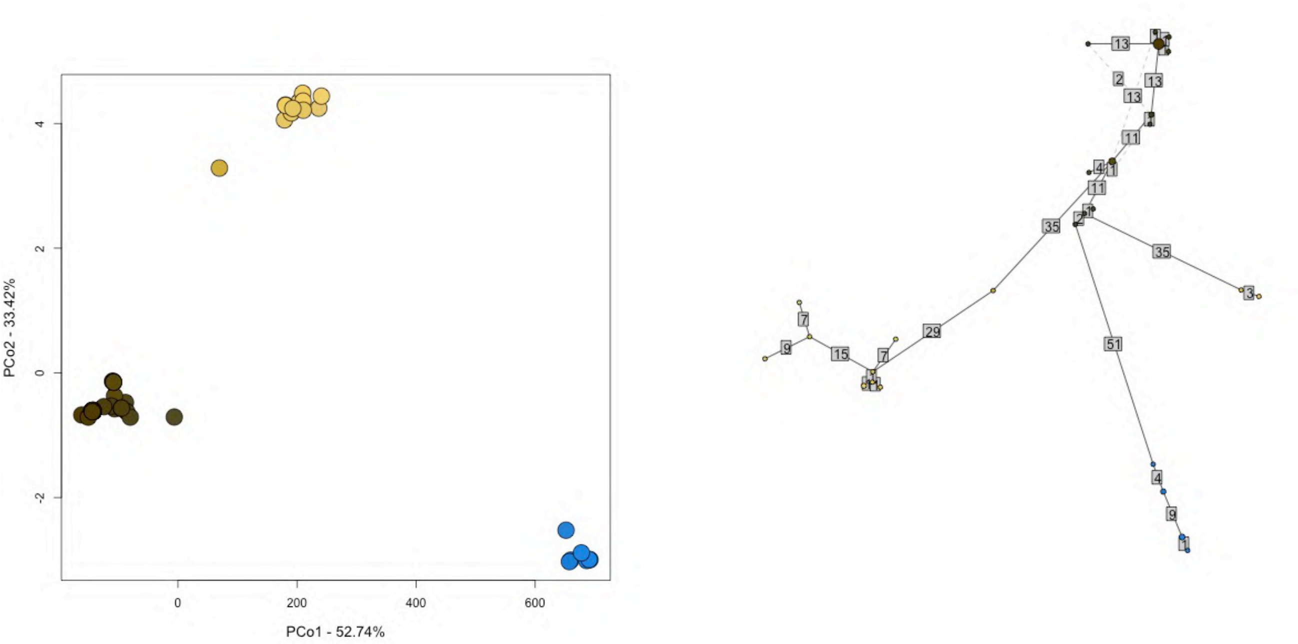

Figure 96: PCoA based on pairwise p-distances between *Camponotus aethiops* sequences (left). Colours match a bidimensional colour space. Haplotype network of *Camponotus aethiops* (right). Sequences > 599 bp: ID = 72, cf. = 0.

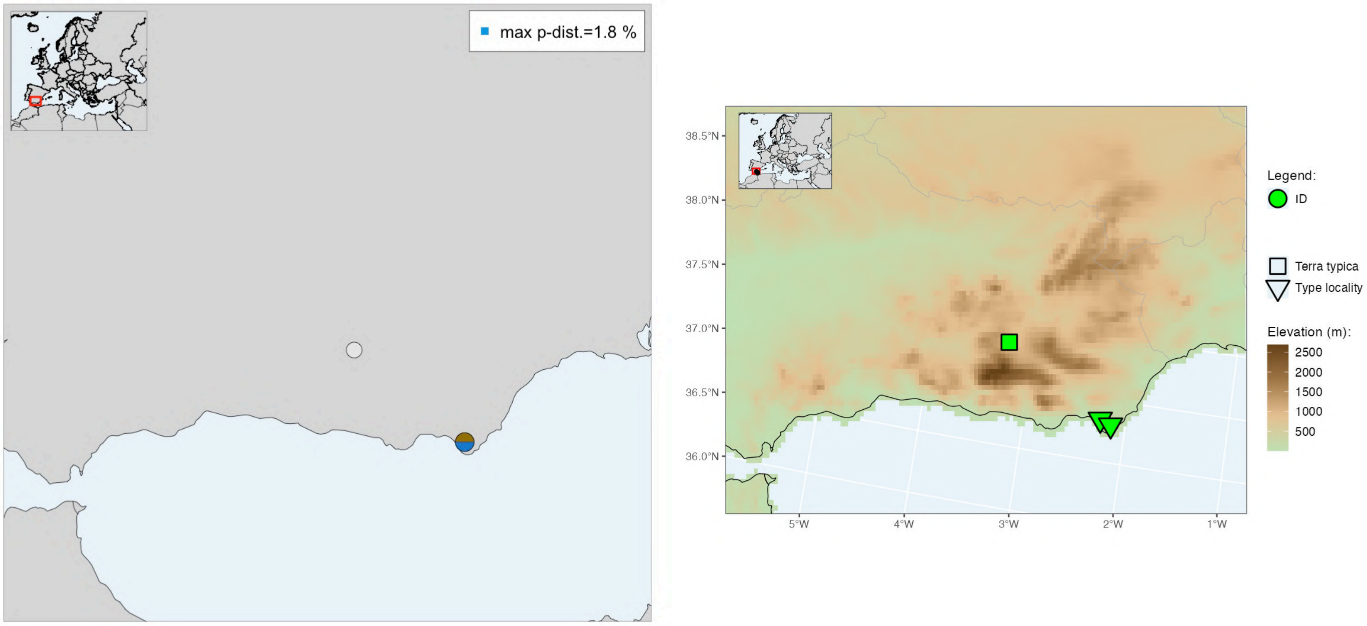

Figure 97: Genetic diversity map of *Camponotus amaurus* Espadaler, 1997. Nearby localities of sequenced specimens are merged in pies (left). Colours match the bidimensional colour space of the PCoA projection (Fig. 97 left) of p-dist between sequences (dots). Specimen identification (ID or cf.) and source (newly sequenced or retrieved) are represented by colours, while specimen attribute (terra typica, type locality, type specimen or faunistic novelty) is represented by the shape (right). Sequences: ID = 3, cf. = 0; maximum p-distance: strict = 1.8 %, less strict = 1.8 %.

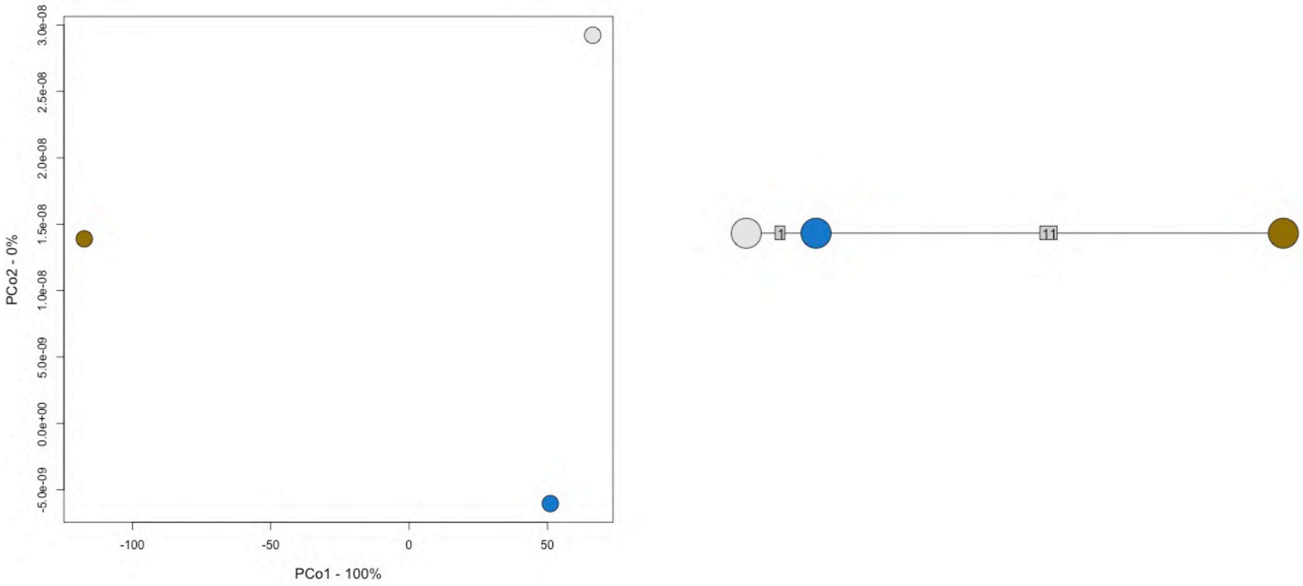

Figure 98: PCoA based on pairwise p-distances between *Camponotus amaurus* sequences (left). Colours match a bidimensional colour space. Haplotype network of *Camponotus amaurus* (right). Sequences > 599 bp: ID = 3, cf. = 0.

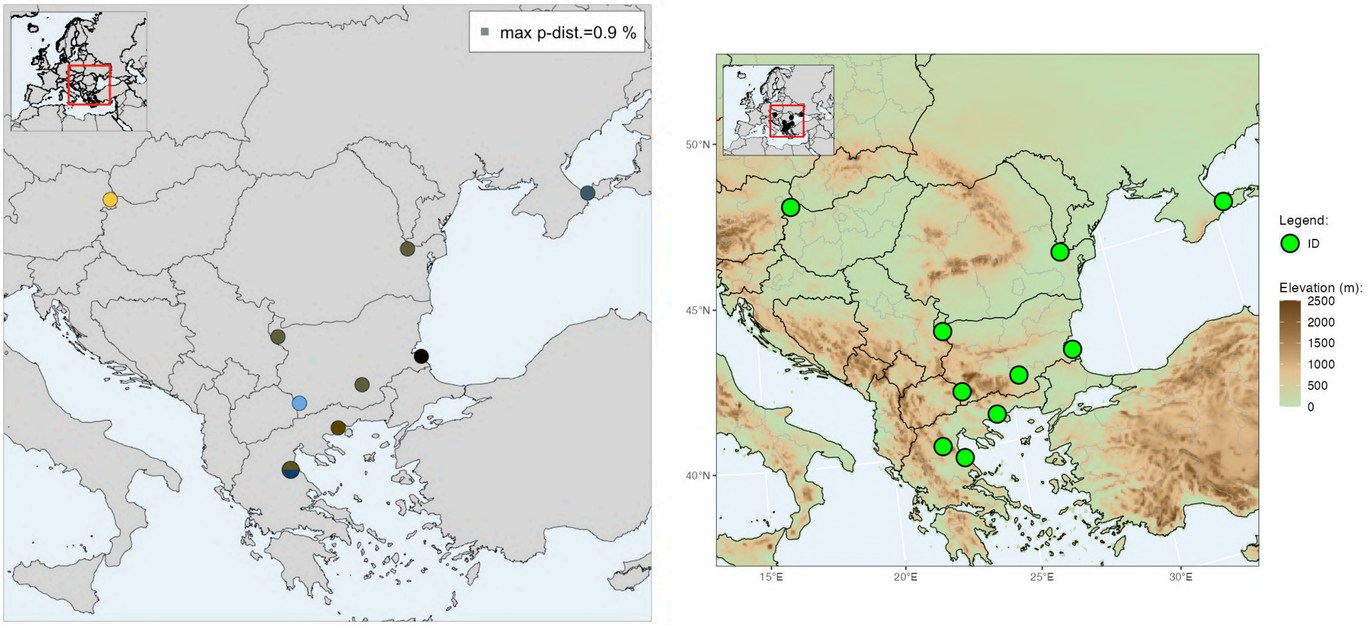

Figure 99: Genetic diversity map of *Camponotus atricolor* (Nylander, 1849). Nearby localities of sequenced specimens are merged in pies (left). Colours match the bidimensional colour space of the PCoA projection (Fig. 99 left) of p-dist between sequences (dots). Specimen identification (ID or cf.) and source (newly sequenced or retrieved) are represented by colours, while specimen attribute (terra typica, type locality, type specimen or faunistic novelty) is represented by the shape (right). Sequences: ID = 10, cf. = 0; maximum p-distance: strict = 0.9 %, less strict = 0.9 %.

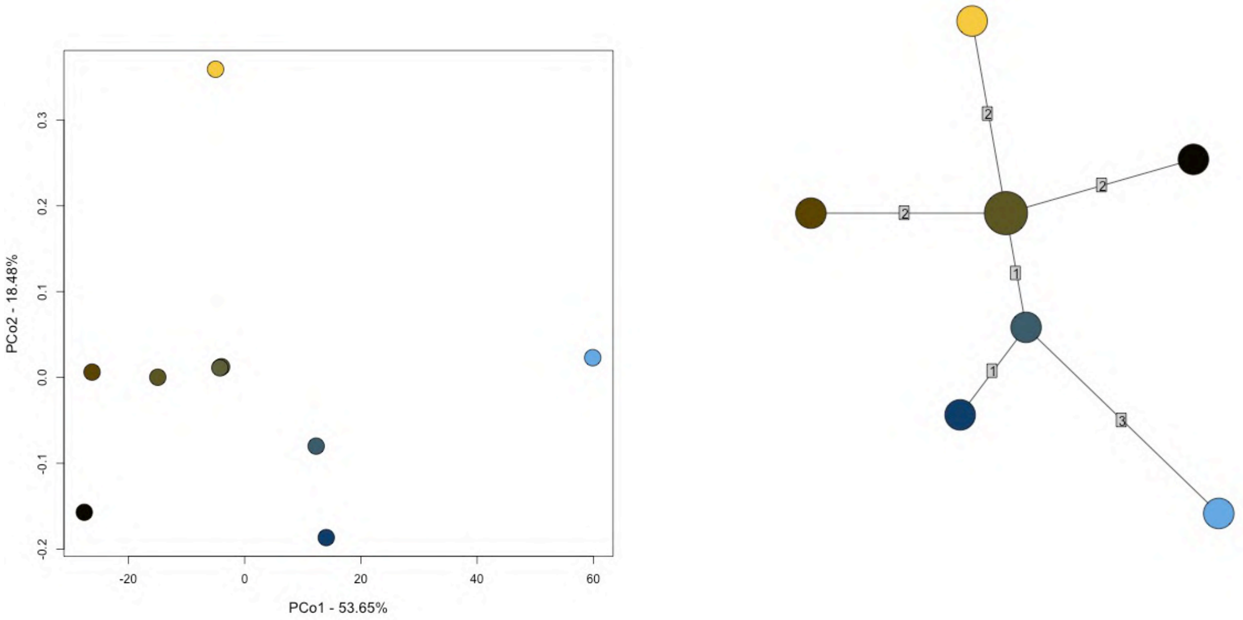

Figure 100: PCoA based on pairwise p-distances between *Camponotus atricolor* sequences (left). Colours match a bidimensional colour space. Haplotype network of *Camponotus atricolor* (right). Sequences > 599 bp: ID = 10, cf. = 0.

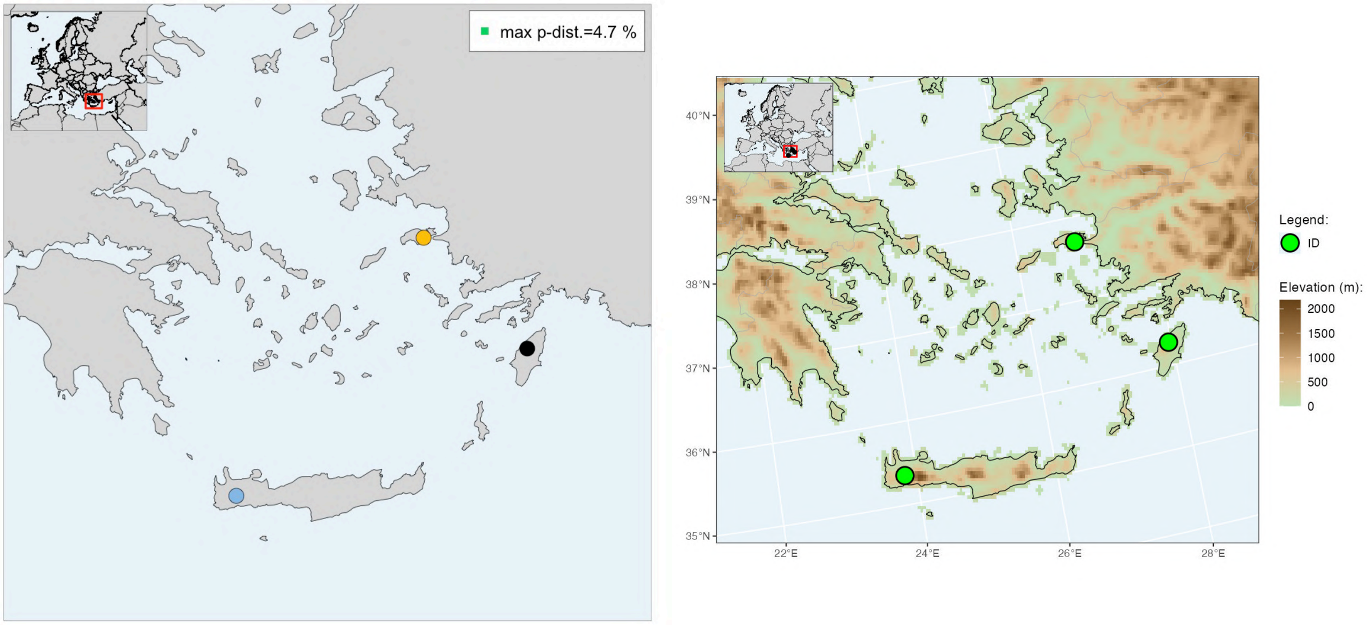

Figure 101: Genetic diversity map of *Camponotus baldaccii* Emery, 1908. Nearby localities of sequenced specimens are merged in pies (left). Colours match the bidimensional colour space of the PCoA projection (Fig. 101 left) of p-dist between sequences (dots). Specimen identification (ID or cf.) and source (newly sequenced or retrieved) are represented by colours, while specimen attribute (terra typica, type locality, type specimen or faunistic novelty) is represented by the shape (right). Sequences: ID = 3, cf. = 0; maximum p-distance: strict = 4.7 %, less strict = 4.7 %.

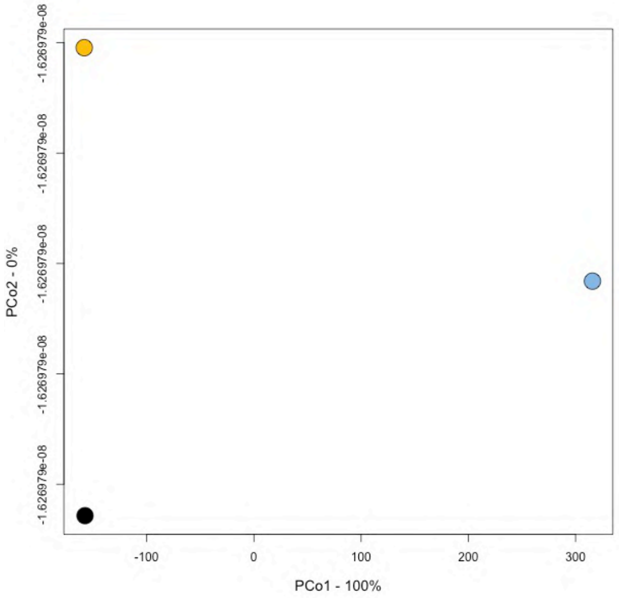

Figure 102: PCoA based on pairwise p-distances between *Camponotus baldaccii* sequences (left). Colours match a bidimensional colour space. Haplotype network analysis of *Camponotus baldaccii* was not possible.

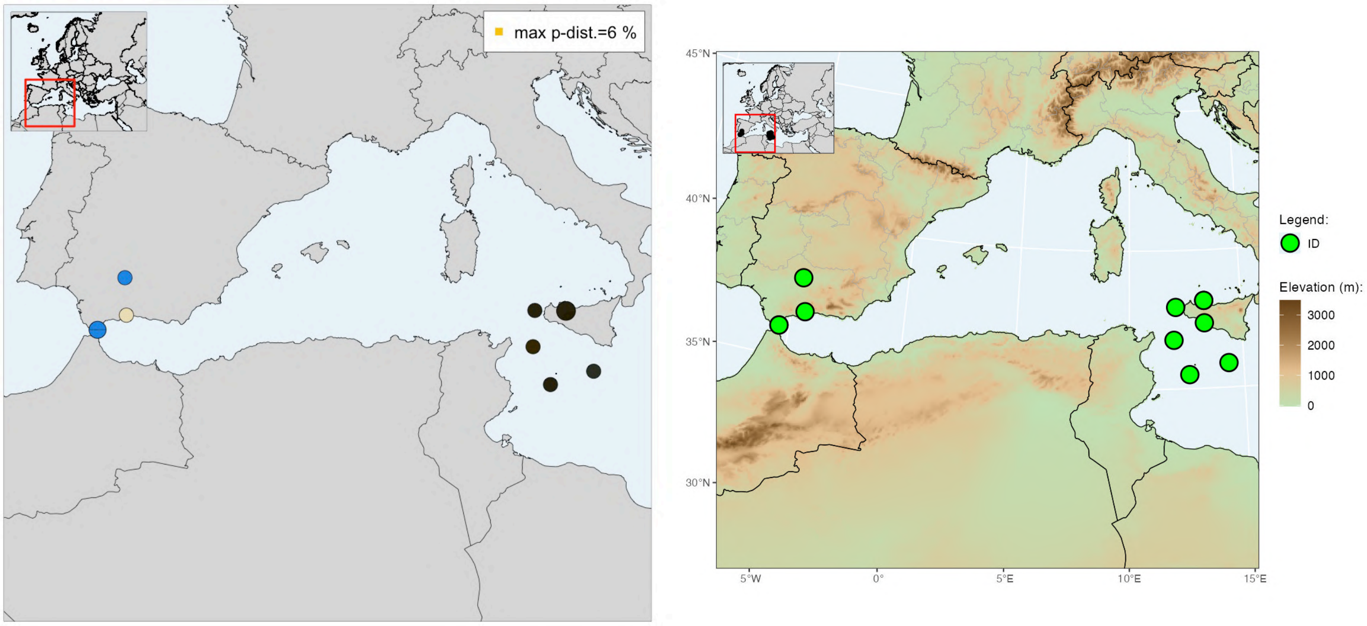

Figure 103: Genetic diversity map of *Camponotus barbaricus* Emery, 1905. Nearby localities of sequenced specimens are merged in pies (left). Colours match the bidimensional colour space of the PCoA projection (Fig. 103 left) of p-dist between sequences (dots). Specimen identification (ID or cf.) and source (newly sequenced or retrieved) are represented by colours, while specimen attribute (terra typica, type locality, type specimen or faunistic novelty) is represented by the shape (right). Sequences: ID = 11, cf. = 0; maximum p-distance: strict = 6 %, less strict = 6 %.

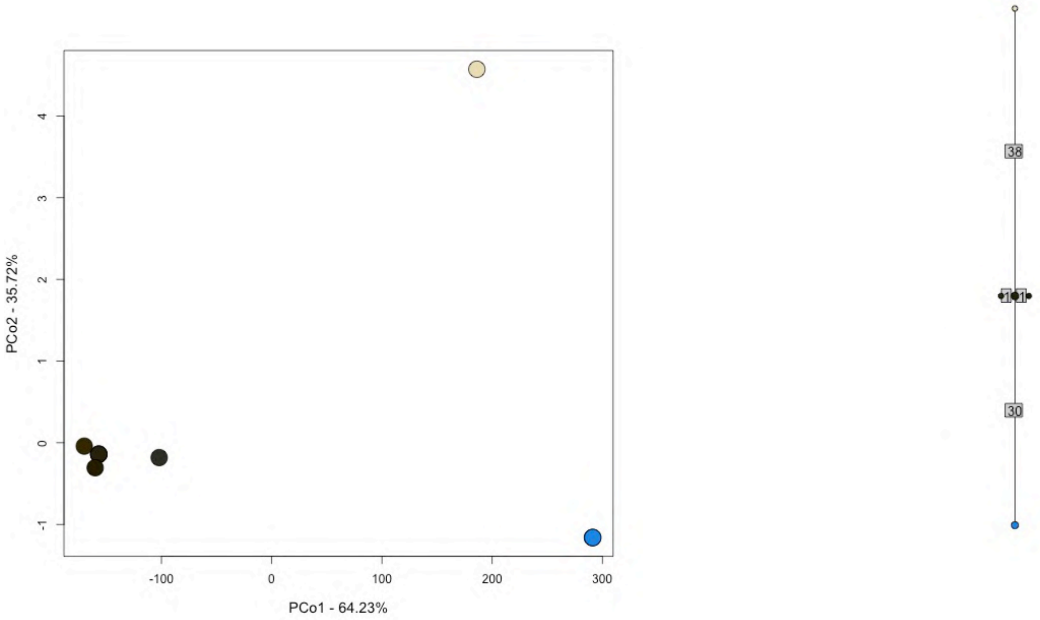

Figure 104: PCoA based on pairwise p-distances between *Camponotus barbaricus* sequences (left). Colours match a bidimensional colour space. Haplotype network of *Camponotus barbaricus* (right). Sequences > 599 bp: ID = 10, cf. = 0.

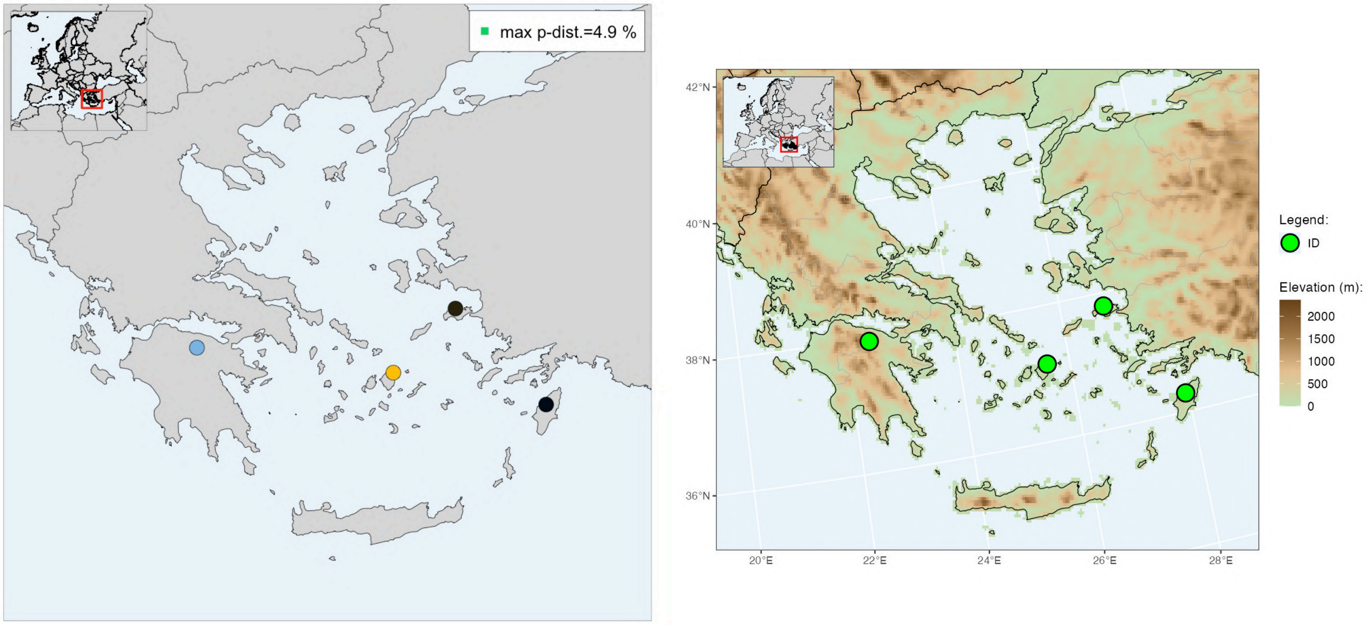

Figure 105: Genetic diversity map of *Camponotus boghossiani* Forel, 1911. Nearby localities of sequenced specimens are merged in pies (left). Colours match the bidimensional colour space of the PCoA projection (Fig. 105 left) of p-dist between sequences (dots). Specimen identification (ID or cf.) and source (newly sequenced or retrieved) are represented by colours, while specimen attribute (terra typica, type locality, type specimen or faunistic novelty) is represented by the shape (right). Sequences: ID = 4, cf. = 0; maximum p-distance: strict = 4.6 %, less strict = 4.9 %.

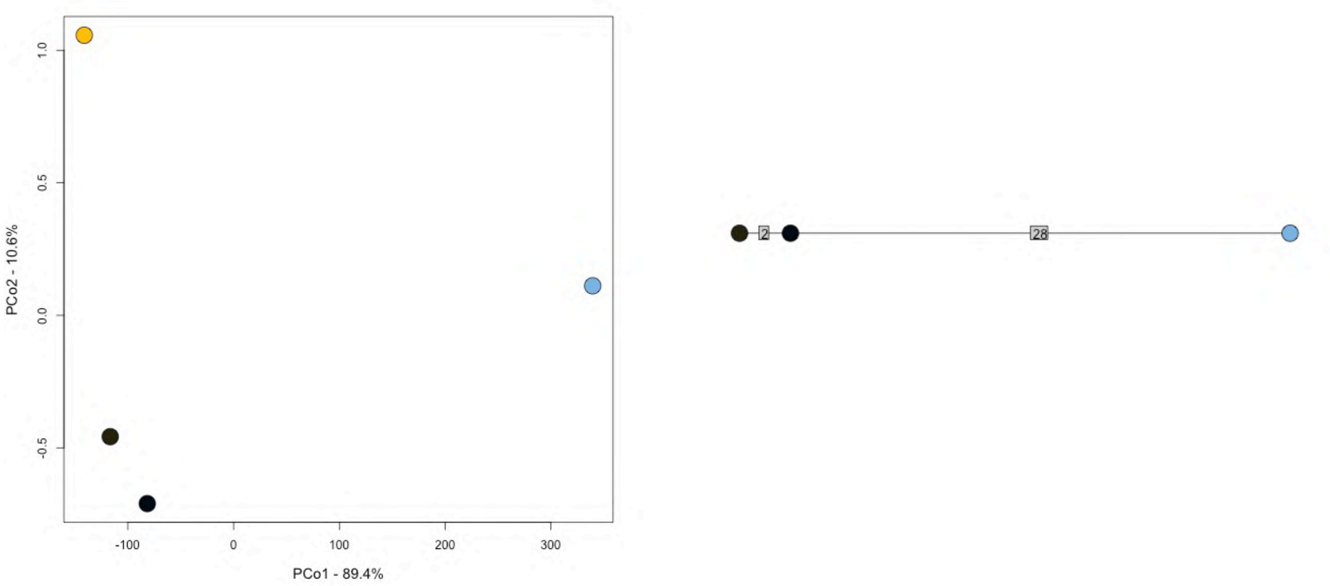

Figure 106: PCoA based on pairwise p-distances between *Camponotus boghossiani* sequences (left). Colours match a bidimensional colour space. Haplotype network of *Camponotus boghossiani* (right). Sequences > 599 bp: ID = 3, cf. = 0.

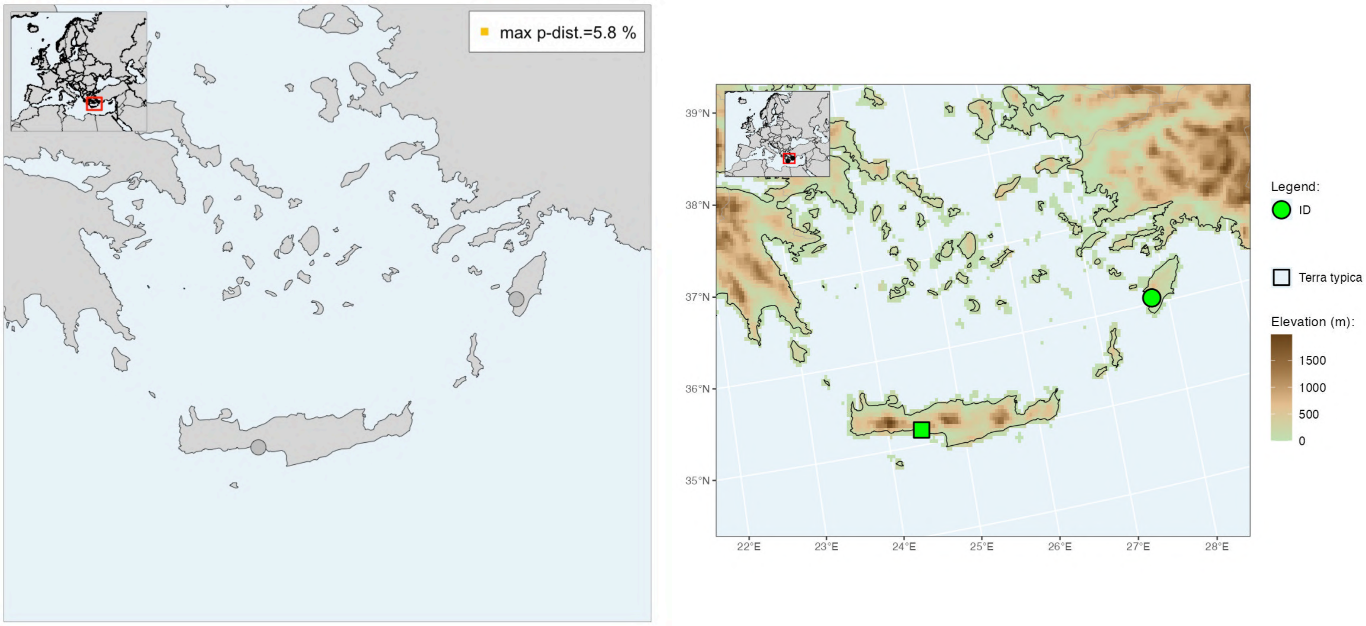

Figure 107: Genetic diversity map of *Camponotus candiotes* Emery, 1894. PCoA projection was not done and therefore sequenced specimens in the genetic diversity map are coloured in gray (left). Specimen identification (ID or cf.) and source (newly sequenced or retrieved) are represented by colours, while specimen attribute (terra typica, type locality, type specimen or faunistic novelty) is represented by the shape (right). Sequences: ID = 2, cf. = 0; maximum p-distance: strict = NA, less strict = 5.8 %.

Haplotype network analysis of *Camponotus candiotes* was not possible.

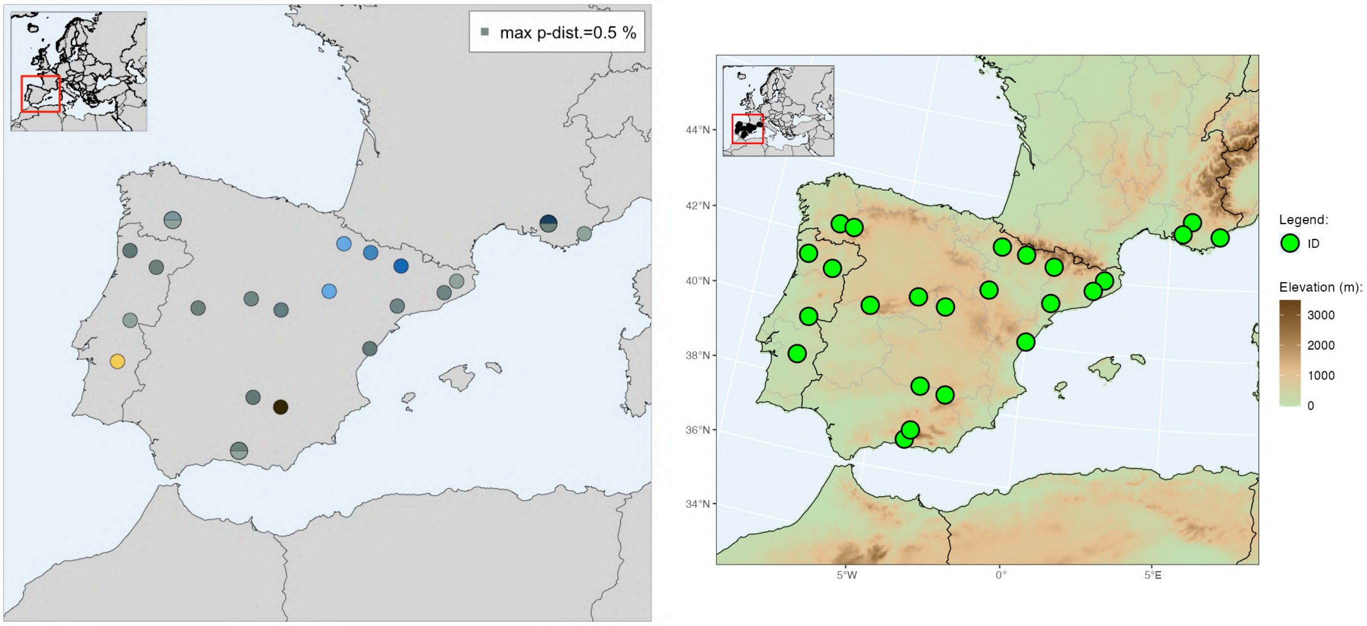

Figure 108: Genetic diversity map of *Camponotus cruentatus* (Latreille, 1802). Nearby localities of sequenced specimens are merged in pies (left). Colours match the bidimensional colour space of the PCoA projection (Fig. 108 left) of p-dist between sequences (dots). Specimen identification (ID or cf.) and source (newly sequenced or retrieved) are represented by colours, while specimen attribute (terra typica, type locality, type specimen or faunistic novelty) is represented by the shape (right). Sequences: ID = 24, cf. = 0; maximum p-distance: strict = 0.5 %, less strict = 0.5 %.

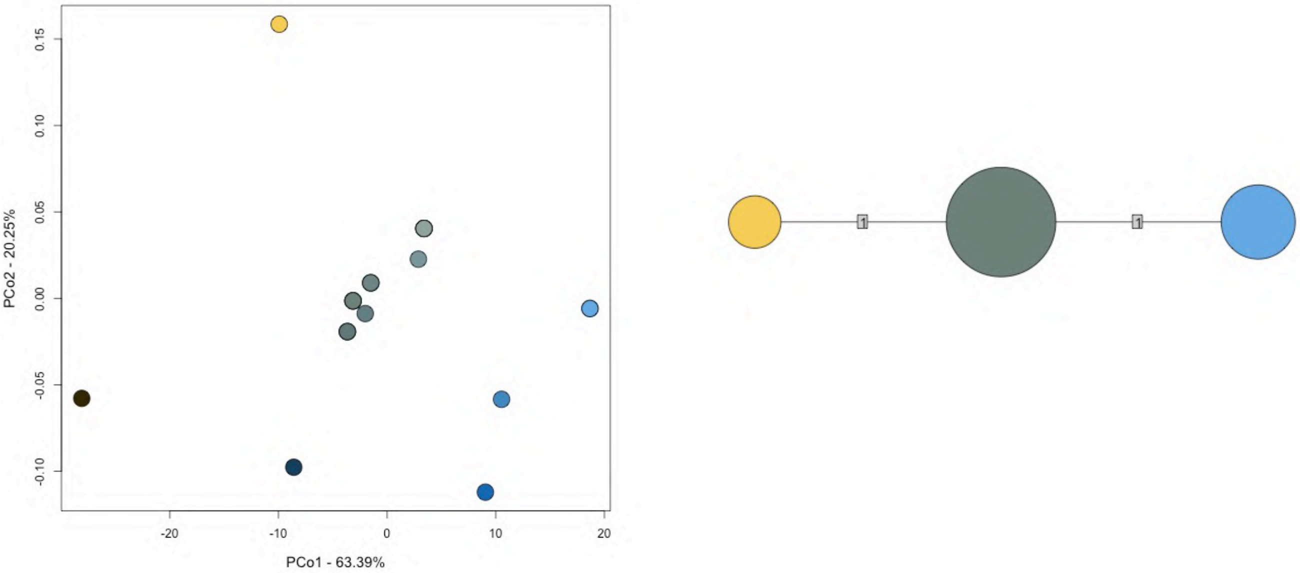

Figure 109: PCoA based on pairwise p-distances between *Camponotus cruentatus* sequences (left). Colours match a bidimensional colour space. Haplotype network of *Camponotus cruentatus* (right). Sequences > 599 bp: ID = 24, cf. = 0.

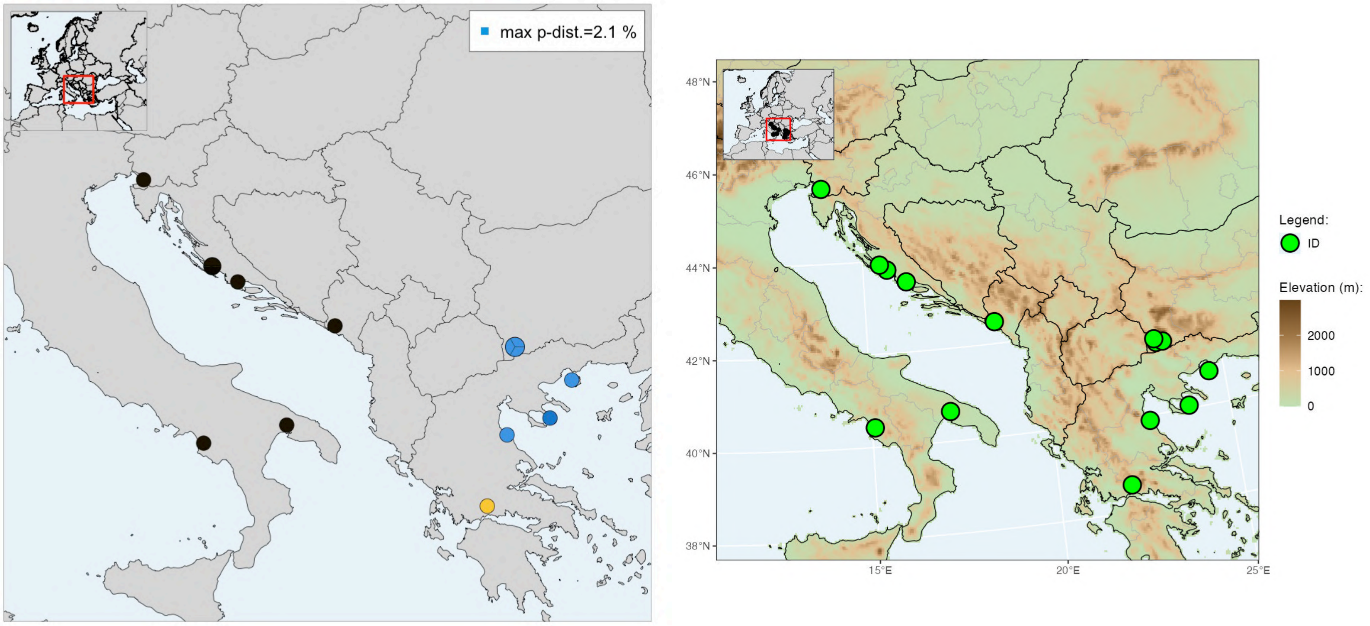

Figure 110: Genetic diversity map of *Camponotus dalmaticus* (Nylander, 1849). Nearby localities of sequenced specimens are merged in pies (left). Colours match the bidimensional colour space of the PCoA projection (Fig. 110 left) of p-dist between sequences (dots). Specimen identification (ID or cf.) and source (newly sequenced or retrieved) are represented by colours, while specimen attribute (terra typica, type locality, type specimen or faunistic novelty) is represented by the shape (right). Sequences: ID = 14, cf. = 0; maximum p-distance: strict = 2.1 %, less strict = 2.1 %.

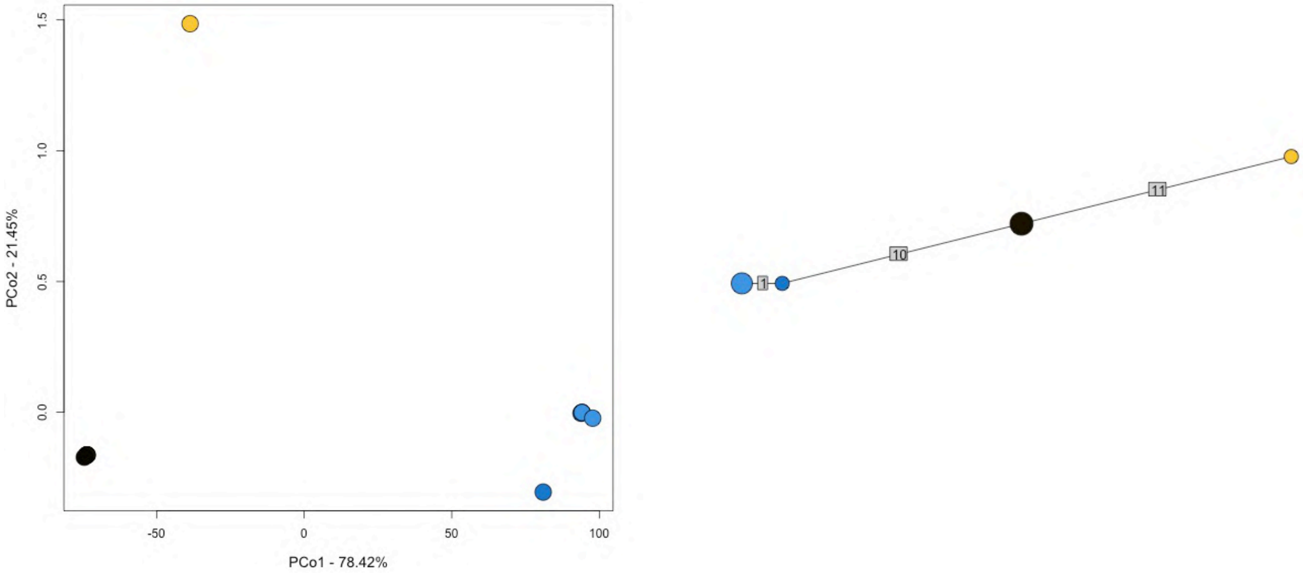

Figure 111: PCoA based on pairwise p-distances between *Camponotus dalmaticus* sequences (left). Colours match a bidimensional colour space. Haplotype network of *Camponotus dalmaticus* (right). Sequences > 599 bp: ID = 14, cf. = 0.

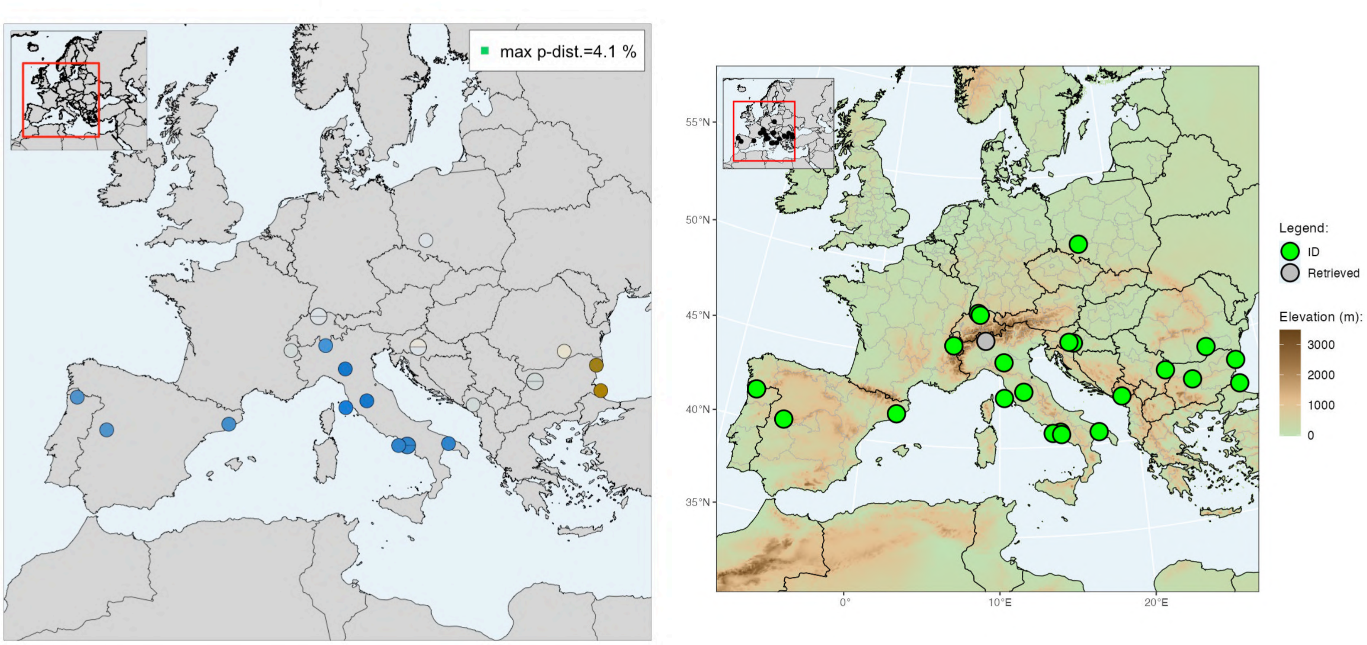

Figure 112: Genetic diversity map of *Camponotus fallax* (Nylander, 1856). Nearby localities of sequenced specimens are merged in pies (left). Colours match the bidimensional colour space of the PCoA projection (Fig. 112 left) of p-dist between sequences (dots). Specimen identification (ID or cf.) and source (newly sequenced or retrieved) are represented by colours, while specimen attribute (terra typica, type locality, type specimen or faunistic novelty) is represented by the shape (right). Sequences: ID = 23, cf. = 0; maximum p-distance: strict = 4.1 %, less strict = 4.1 %.

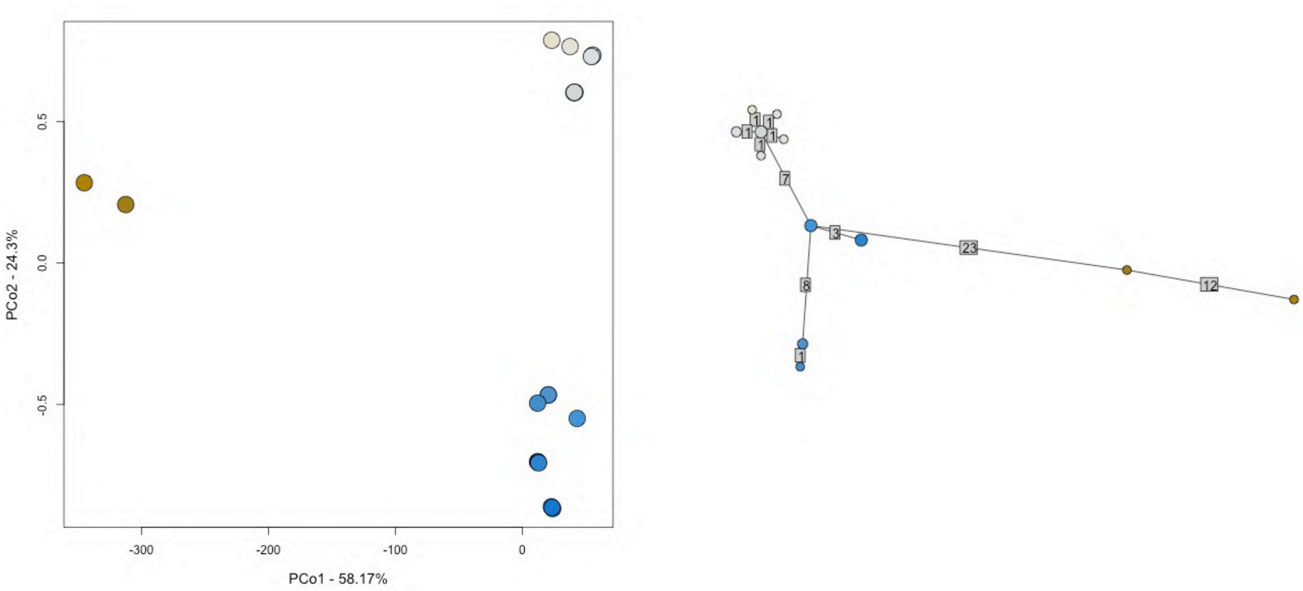

Figure 113: PCoA based on pairwise p-distances between *Camponotus fallax* sequences (left). Colours match a bidimensional colour space. Haplotype network of *Camponotus fallax* (right). Sequences > 599 bp: ID = 23, cf. = 0.

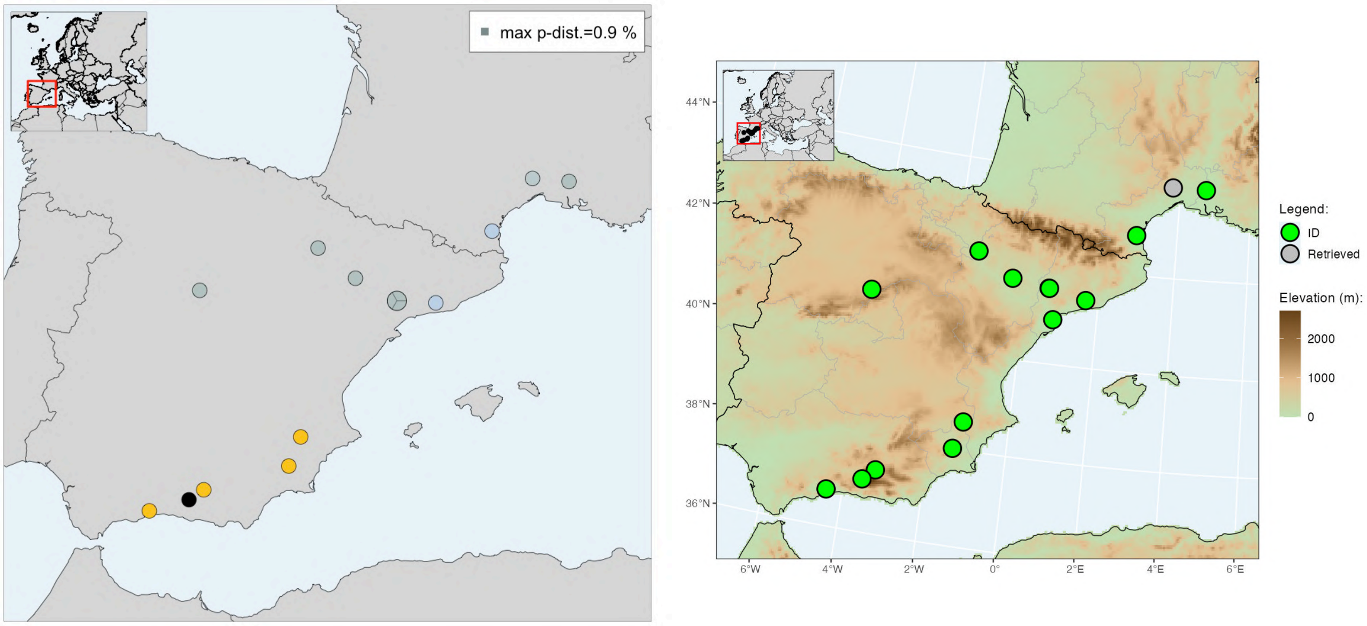

Figure 114: Genetic diversity map of *Camponotus foreli* Emery, 1881. Nearby localities of sequenced specimens are merged in pies (left). Colours match the bidimensional colour space of the PCoA projection (Fig. 114 left) of p-dist between sequences (dots). Specimen identification (ID or cf.) and source (newly sequenced or retrieved) are represented by colours, while specimen attribute (terra typica, type locality, type specimen or faunistic novelty) is represented by the shape (right). Sequences: ID = 15, cf. = 0; maximum p-distance: strict = 0.9 %, less strict = 0.9 %.

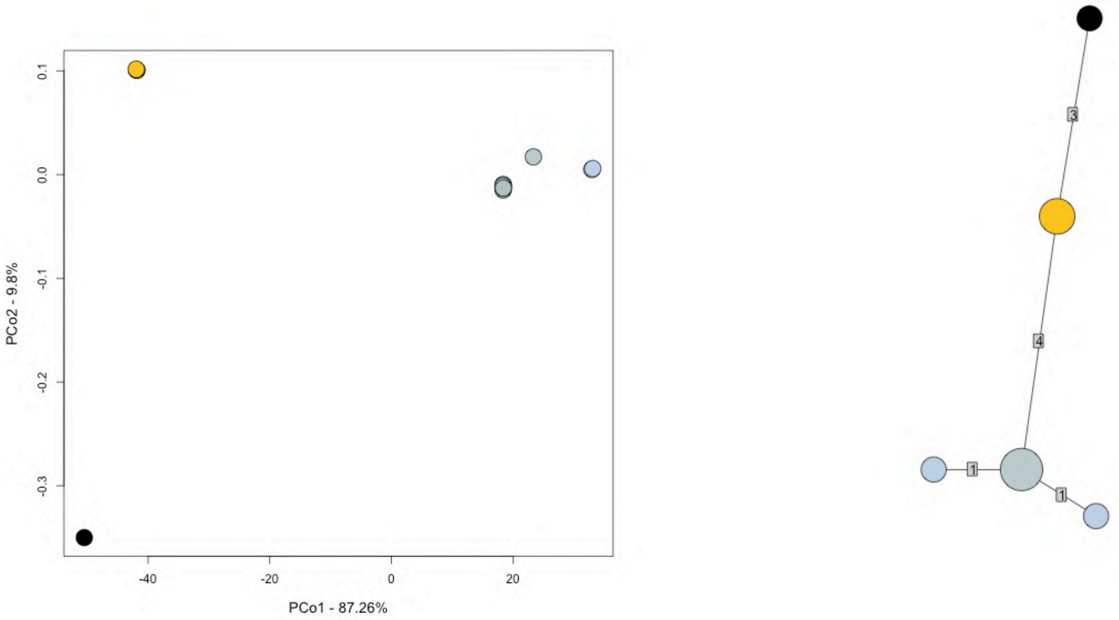

Figure 115: PCoA based on pairwise p-distances between *Camponotus foreli* sequences (left). Colours match a bidimensional colour space. Haplotype network of *Camponotus foreli* (right). Sequences > 599 bp: ID = 15, cf. = 0.

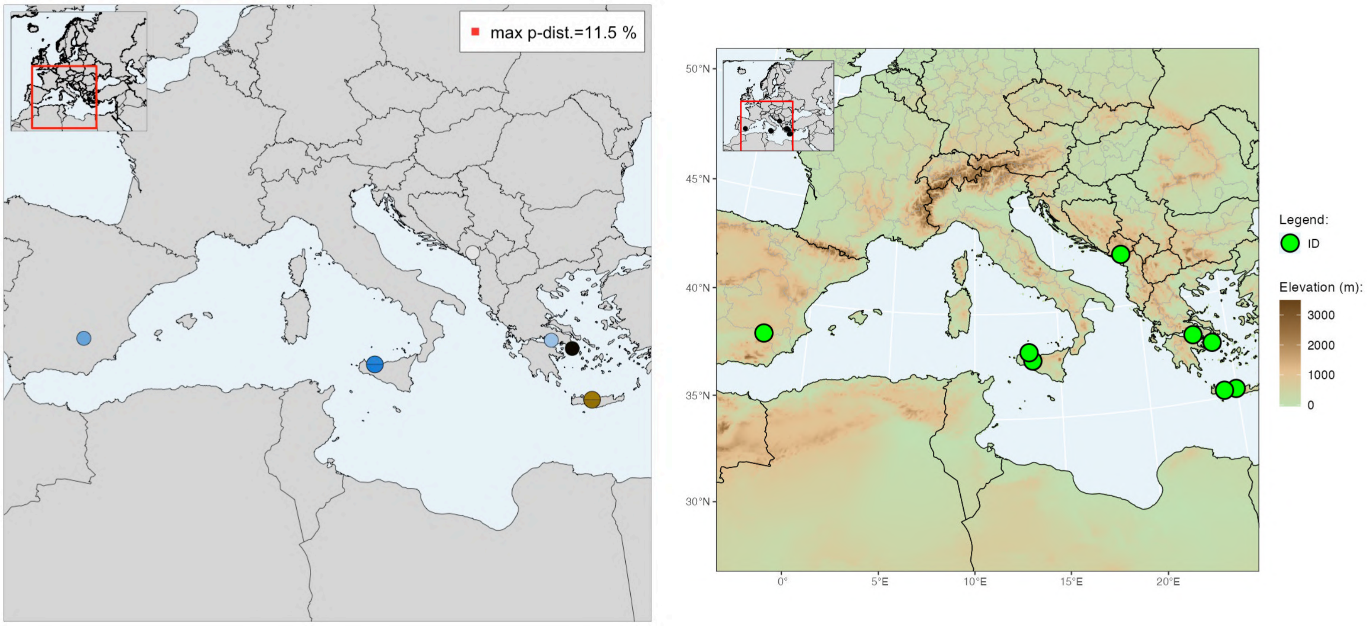

Figure 116: Genetic diversity map of *Camponotus gestroi* Emery, 1878. Nearby localities of sequenced specimens are merged in pies (left). Colours match the bidimensional colour space of the PCoA projection (Fig. 116 left) of p-dist between sequences (dots). Specimen identification (ID or cf.) and source (newly sequenced or retrieved) are represented by colours, while specimen attribute (terra typica, type locality, type specimen or faunistic novelty) is represented by the shape (right). Sequences: ID = 8, cf. = 0; maximum p-distance: strict = 11.5 %, less strict = 11.5 %.

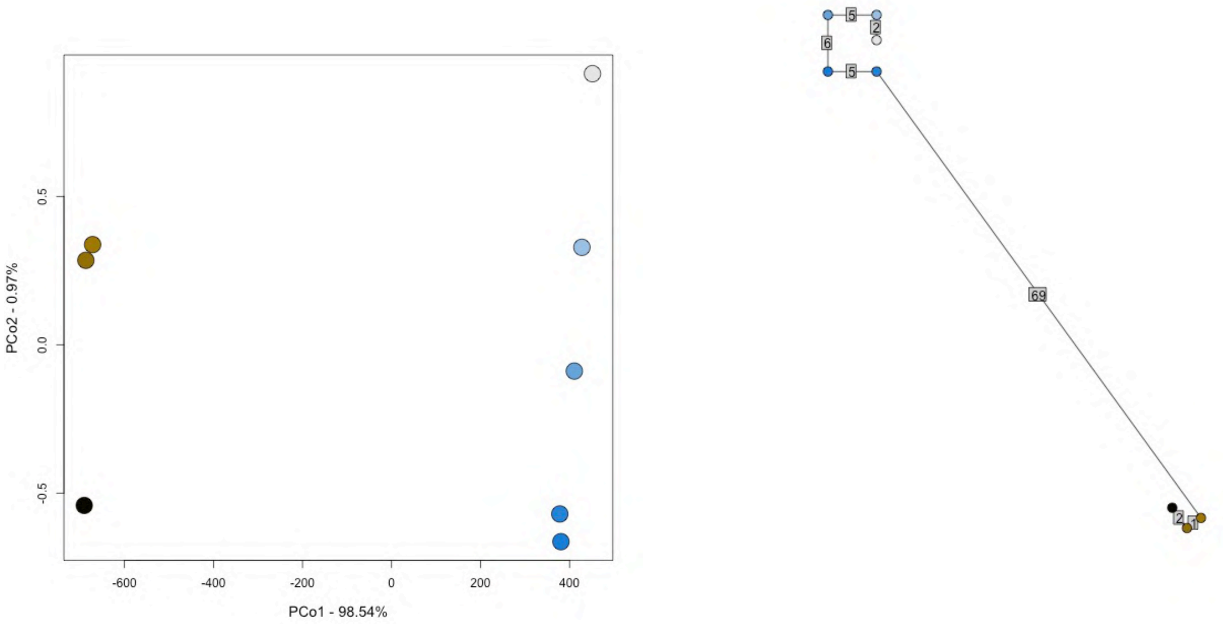

Figure 117: PCoA based on pairwise p-distances between *Camponotus gestroi* sequences (left). Colours match a bidimensional colour space. Haplotype network of *Camponotus gestroi* (right). Sequences > 599 bp: ID = 8, cf. = 0.

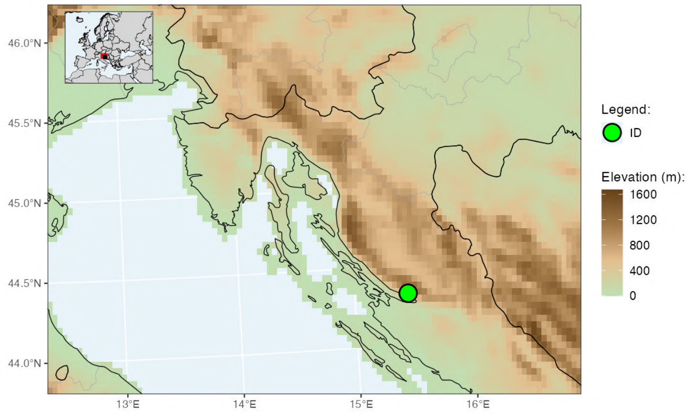

Figure 118: Map of *Camponotus heidrunvogtae* Seifert, 2019. Due to the presence of a single sequence, the genetic diversity map and the PCoA projection were not done. Specimen identification (ID or cf.) and source (newly sequenced or retrieved) are represented by colours, while specimen attribute (terra typica, type locality, type specimen or faunistic novelty) is represented by the shape. Sequences: ID = 1, cf. = 0; maximum p-distance: strict = NA, less strict = NA.

Haplotype network analysis of *Camponotus heidrunvogtae* was not possible.

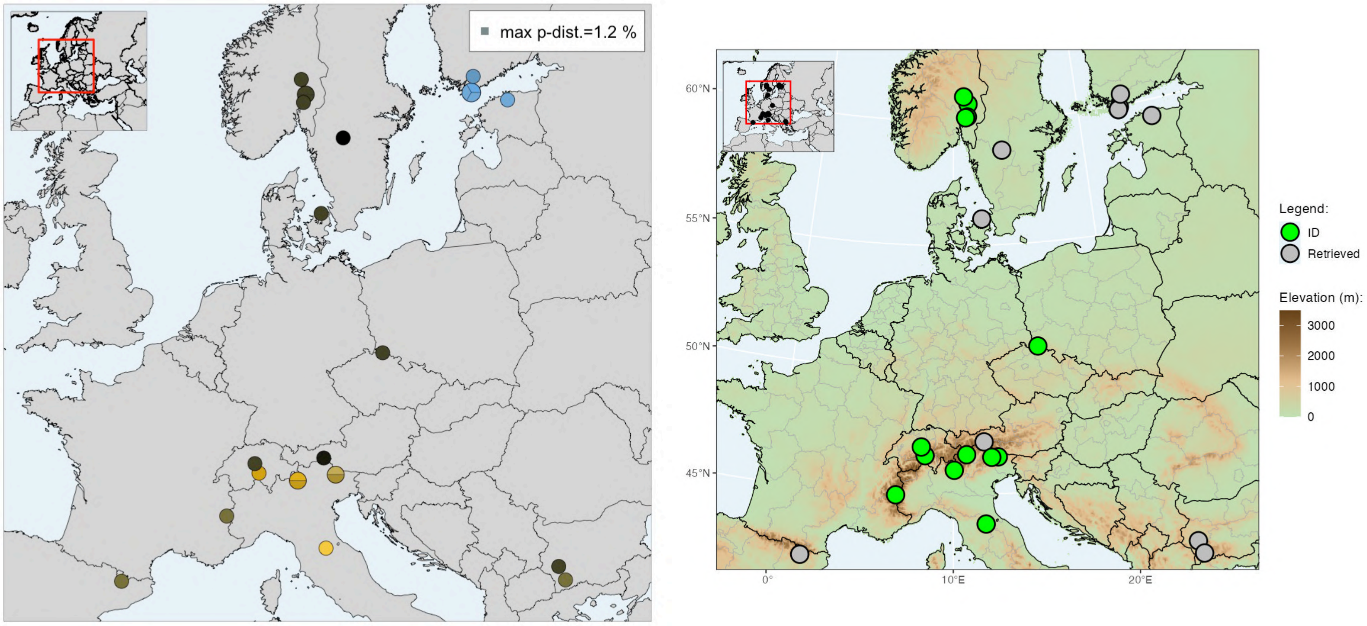

Figure 119: Genetic diversity map of *Camponotus herculeanus* (Linnaeus, 1758). Nearby localities of sequenced specimens are merged in pies (left). Colours match the bidimensional colour space of the PCoA projection (Fig. 119 left) of p-dist between sequences (dots). Specimen identification (ID or cf.) and source (newly sequenced or retrieved) are represented by colours, while specimen attribute (terra typica, type locality, type specimen or faunistic novelty) is represented by the shape (right). Sequences: ID = 24, cf. = 0; maximum p-distance: strict = 1.2 %, less strict = 1.2 %.

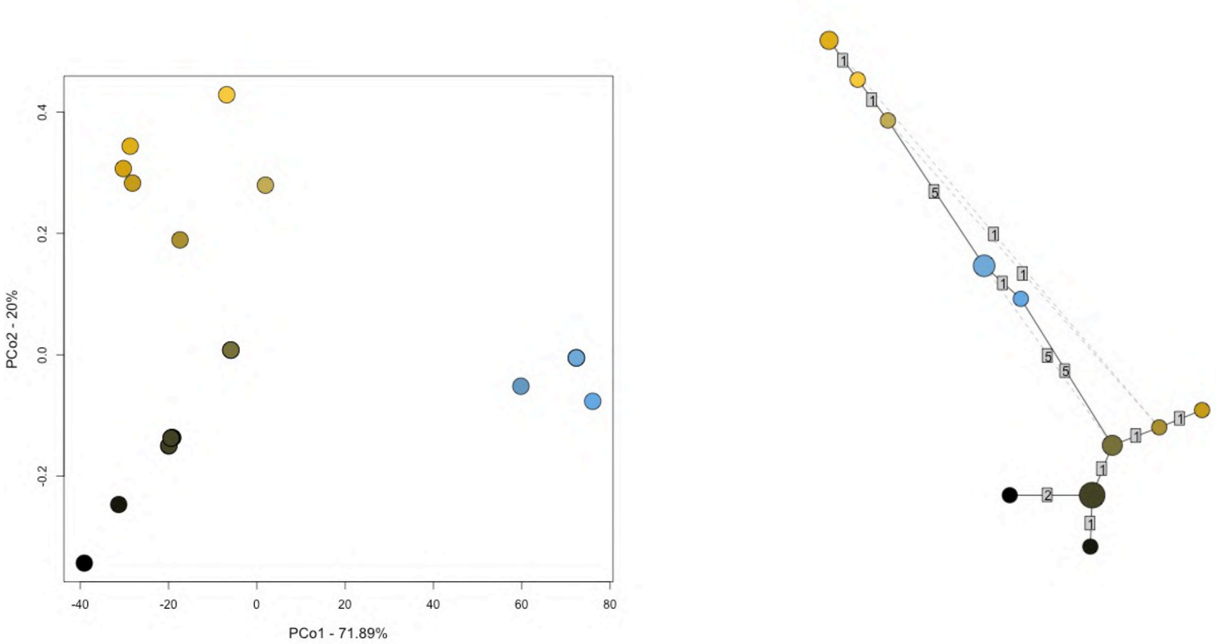

Figure 120: PCoA based on pairwise p-distances between *Camponotus herculeanus* sequences (left). Colours match a bidimensional colour space. Haplotype network of *Camponotus herculeanus* (right). Sequences > 599 bp: ID = 24, cf. = 0.

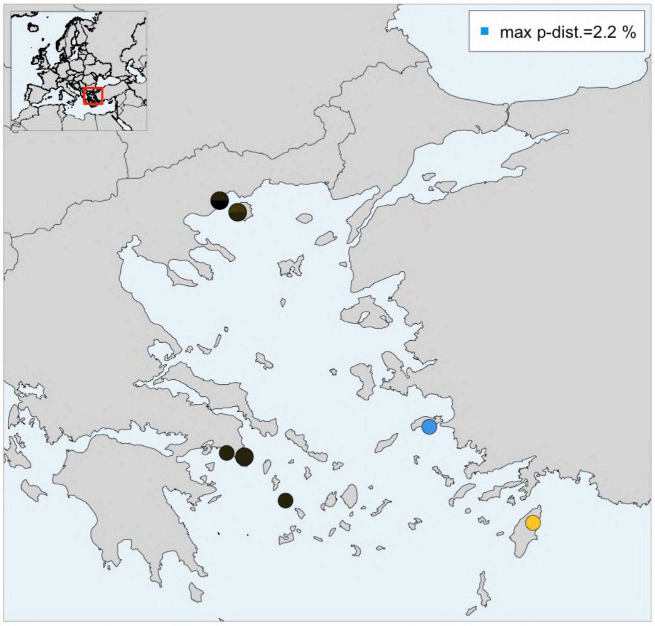

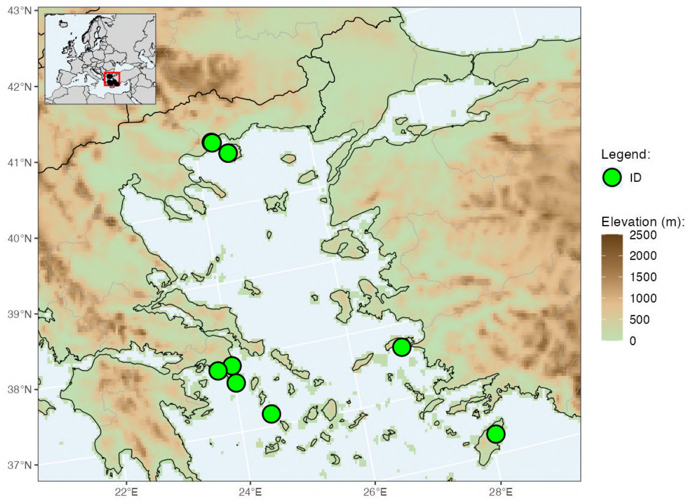

Figure 121: Genetic diversity map of *Camponotus ionius* Emery, 1920. Nearby localities of sequenced specimens are merged in pies (left). Colours match the bidimensional colour space of the PCoA projection (Fig. 121 left) of p-dist between sequences (dots). Specimen identification (ID or cf.) and source (newly sequenced or retrieved) are represented by colours, while specimen attribute (terra typica, type locality, type specimen or faunistic novelty) is represented by the shape (right). Sequences: ID = 10, cf. = 0; maximum p-distance: strict = 2.2 %, less strict = 2.2 %.

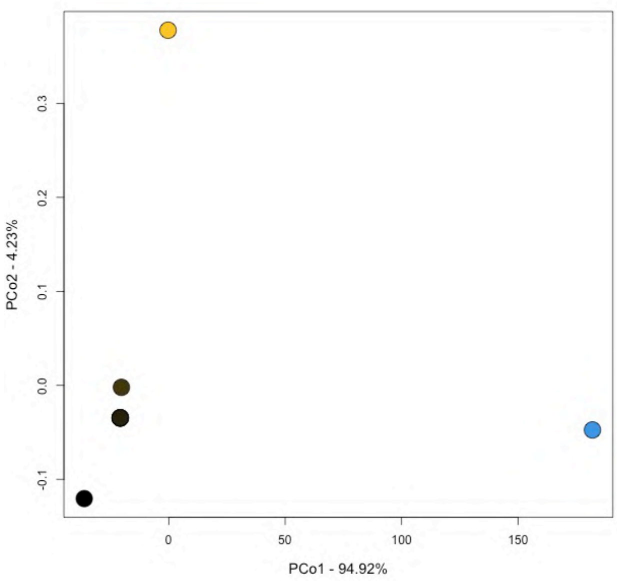

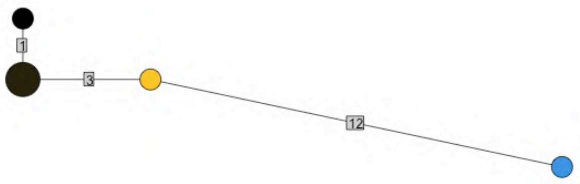

Figure 122: PCoA based on pairwise p-distances between *Camponotus ionius* sequences (left). Colours match a bidimensional colour space. Haplotype network of *Camponotus ionius* (right). Sequences > 599 bp: ID = 10, cf. = 0.

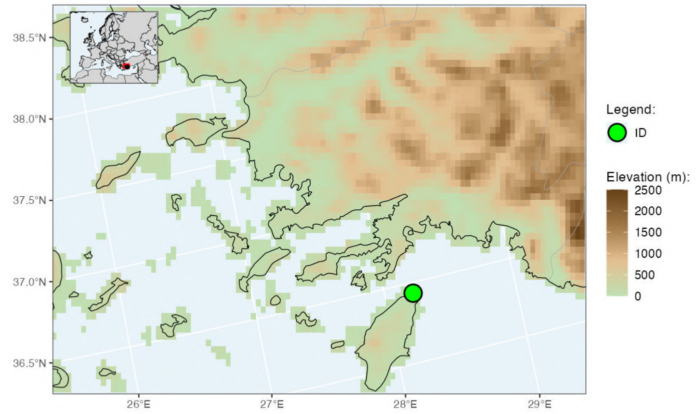

Figure 123: Map of *Camponotus jaliensis* Dalla Torre, 1893. Due to the presence of a single sequence, the genetic diversity map and the PCoA projection were not done. Specimen identification (ID or cf.) and source (newly sequenced or retrieved) are represented by colours, while specimen attribute (terra typica, type locality, type specimen or faunistic novelty) is represented by the shape. Sequences: ID = 1, cf. = 0; maximum p-distance: strict = NA, less strict = NA.

Haplotype network analysis of *Camponotus jaliensis* was not possible.

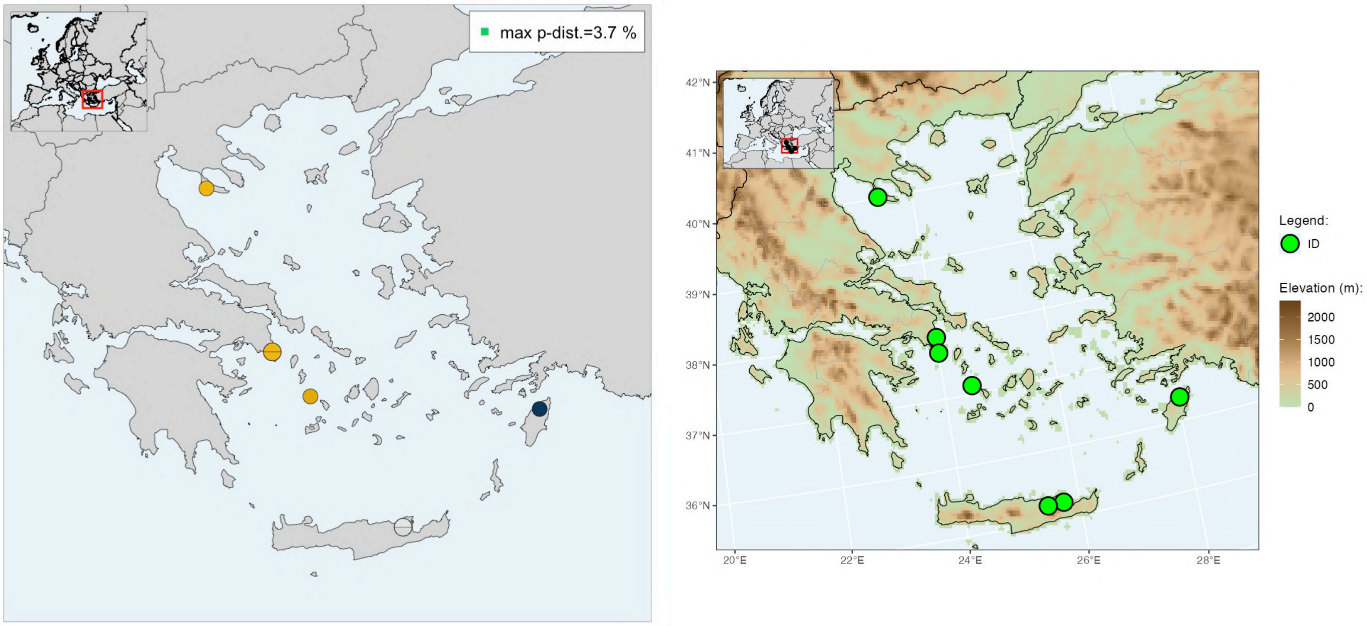

Figure 124: Genetic diversity map of *Camponotus kiesenwetteri* (Roger, 1859). Nearby localities of sequenced specimens are merged in pies (left). Colours match the bidimensional colour space of the PCoA projection (Fig. 124 left) of p-dist between sequences (dots). Specimen identification (ID or cf.) and source (newly sequenced or retrieved) are represented by colours, while specimen attribute (terra typica, type locality, type specimen or faunistic novelty) is represented by the shape (right). Sequences: ID = 7, cf. = 0; maximum p-distance: strict = 3.7 %, less strict = 3.7 %.

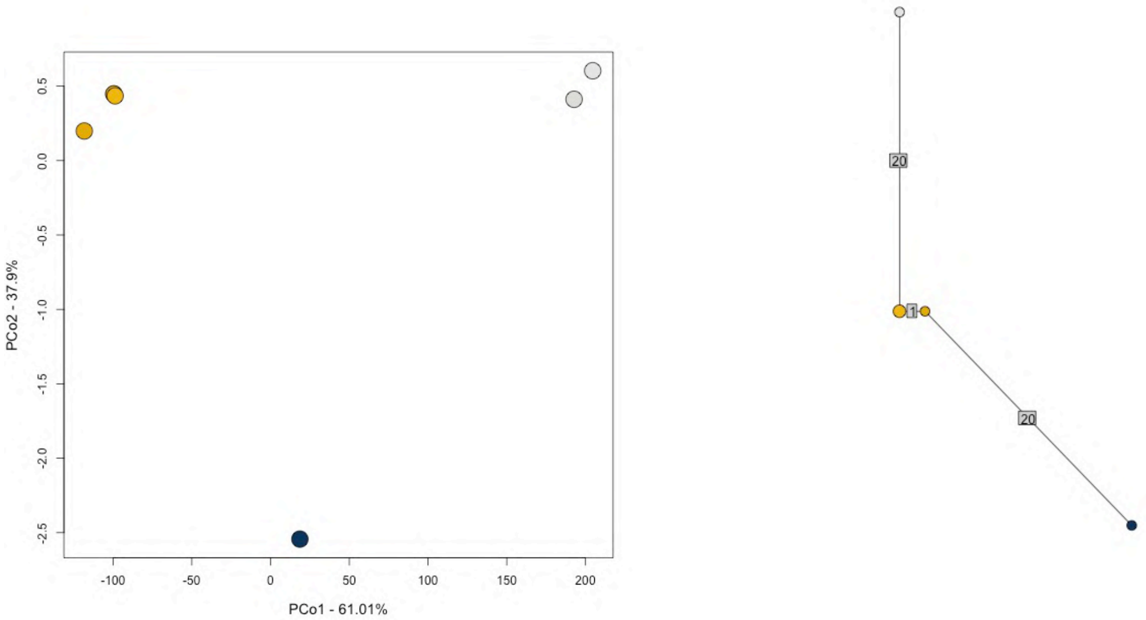

Figure 125: PCoA based on pairwise p-distances between *Camponotus kiesenwetteri* sequences (left). Colours match a bidimensional colour space. Haplotype network of *Camponotus kiesenwetteri* (right). Sequences > 599 bp: ID = 6, cf. = 0.

Camponotus laconicus

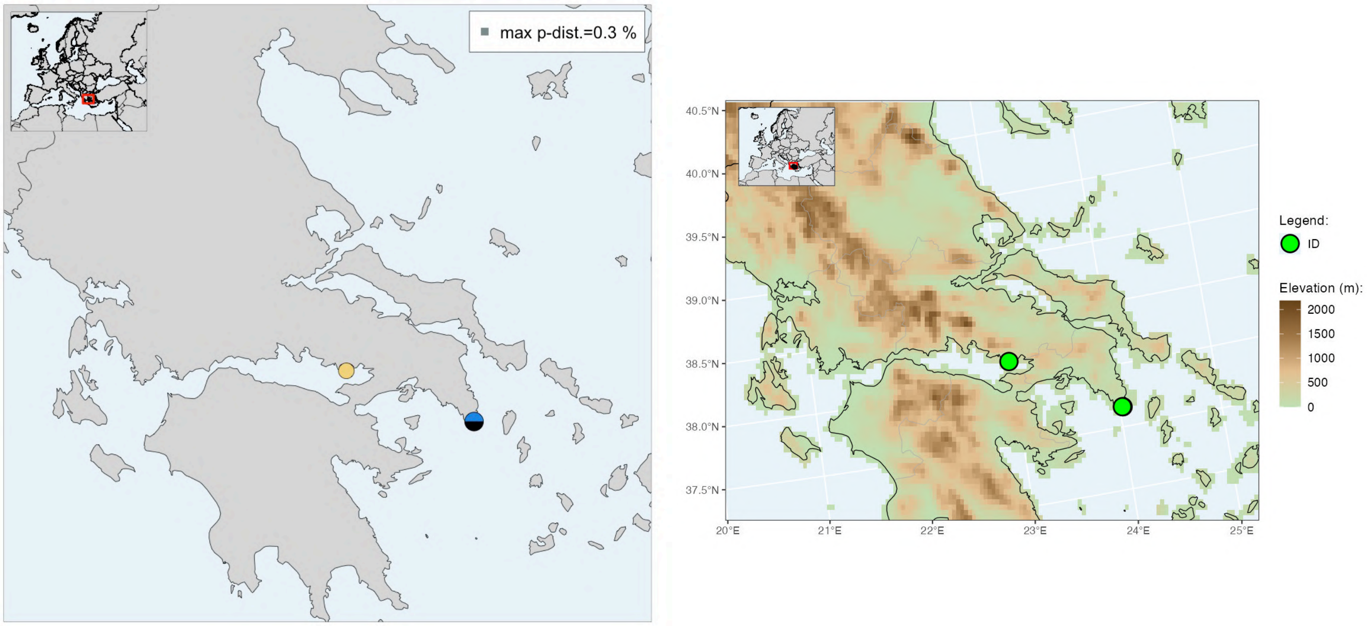

Figure 126: Genetic diversity map of *Camponotus laconicus* Emery, 1920. Nearby localities of sequenced specimens are merged in pies (left). Colours match the bidimensional colour space of the PCoA projection (Fig. 126 left) of p-dist between sequences (dots). Specimen identification (ID or cf.) and source (newly sequenced or retrieved) are represented by colours, while specimen attribute (terra typica, type locality, type specimen or faunistic novelty) is represented by the shape (right). Sequences: ID = 3, cf. = 0; maximum p-distance: strict = 0.3 %, less strict = 0.3 %.

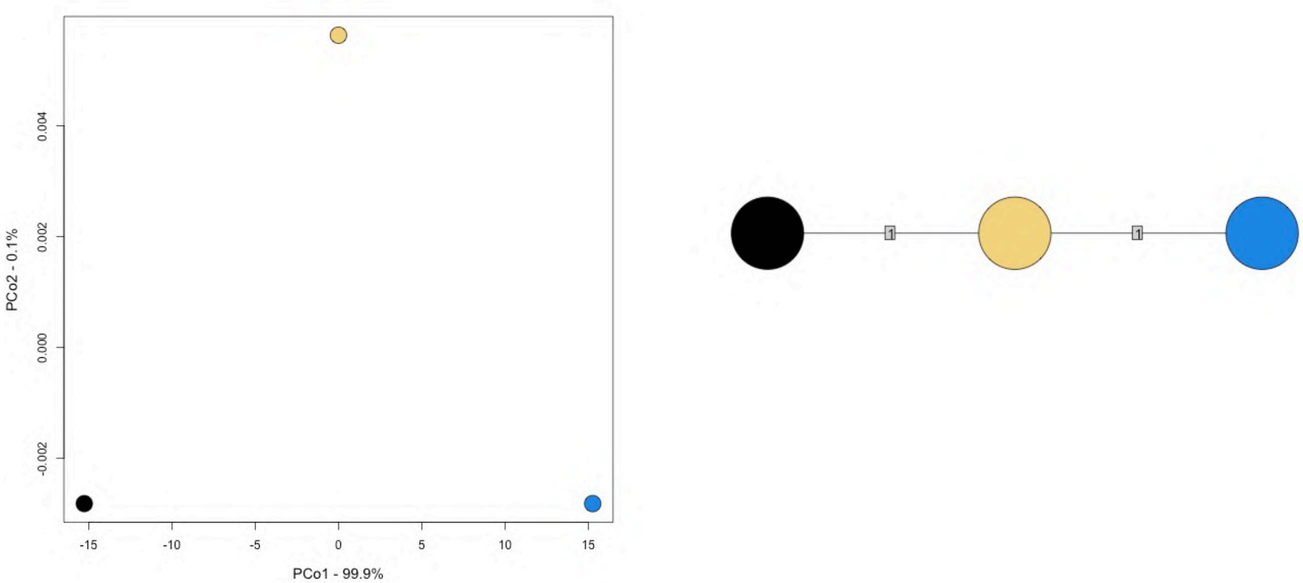

Figure 127: PCoA based on pairwise p-distances between *Camponotus laconicus* sequences (left). Colours match a bidimensional colour space. Haplotype network of *Camponotus laconicus* (right). Sequences > 599 bp: ID = 3, cf. = 0.

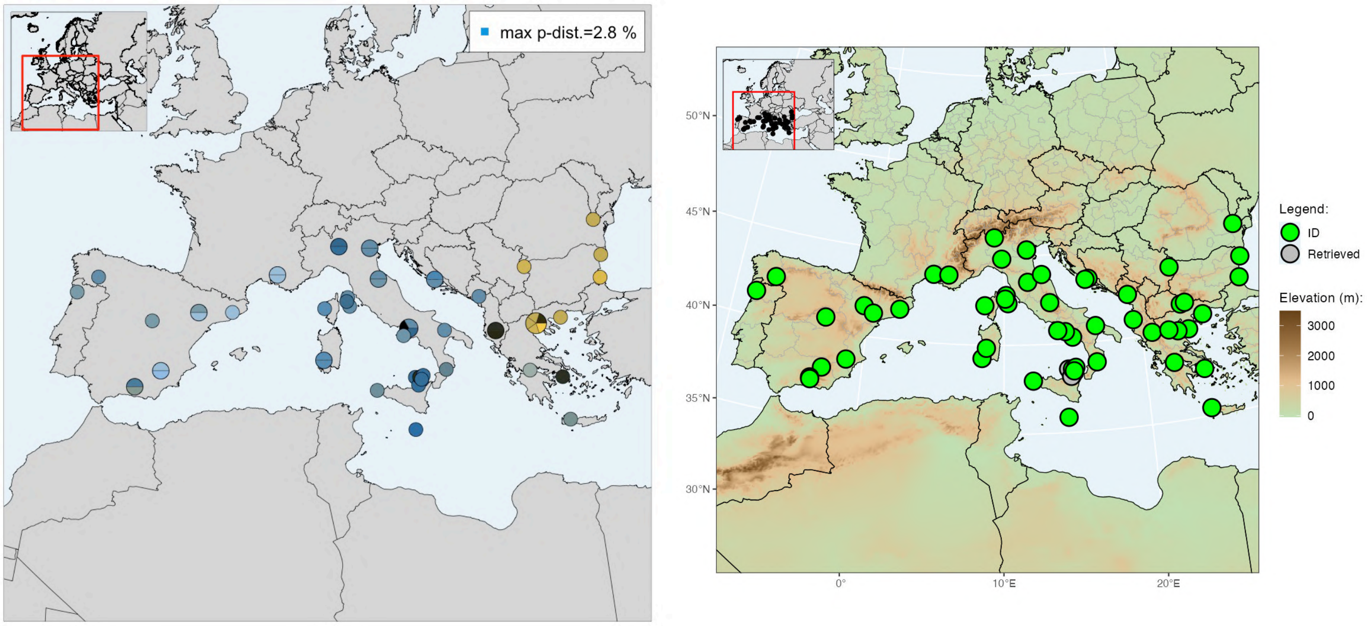

Figure 128: Genetic diversity map of *Camponotus lateralis* (Olivier, 1792). Nearby localities of sequenced specimens are merged in pies (left). Colours match the bidimensional colour space of the PCoA projection (Fig. 128 left) of p-dist between sequences (dots). Specimen identification (ID or cf.) and source (newly sequenced or retrieved) are represented by colours, while specimen attribute (terra typica, type locality, type specimen or faunistic novelty) is represented by the shape (right). Sequences: ID = 56, cf. = 0; maximum p-distance: strict = 2.6 %, less strict = 2.8 %.

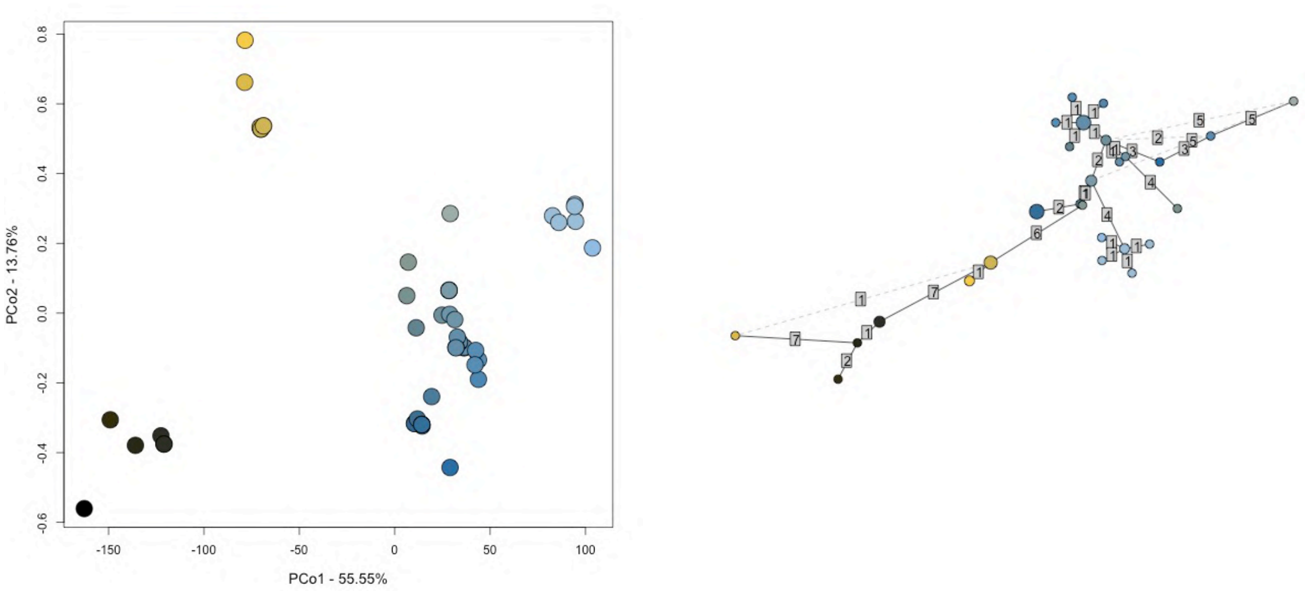

Figure 129: PCoA based on pairwise p-distances between *Camponotus lateralis* sequences (left). Colours match a bidimensional colour space. Haplotype network of *Camponotus lateralis* (right). Sequences > 599 bp: ID = 55, cf. = 0.

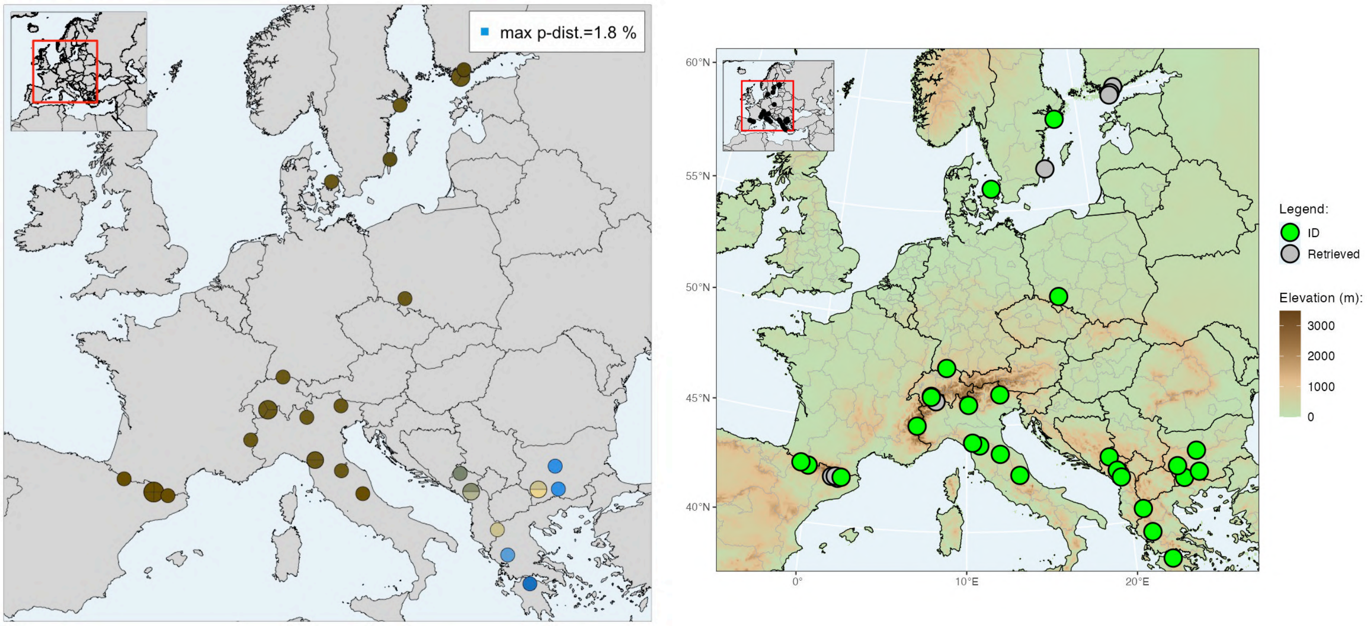

Figure 130: Genetic diversity map of *Camponotus ligniperda* (Latreille, 1802). Nearby localities of sequenced specimens are merged in pies (left). Colours match the bidimensional colour space of the PCoA projection (Fig. 130 left) of p-dist between sequences (dots). Specimen identification (ID or cf.) and source (newly sequenced or retrieved) are represented by colours, while specimen attribute (terra typica, type locality, type specimen or faunistic novelty) is represented by the shape (right). Sequences: ID = 35, cf. = 0; maximum p-distance: strict = 1.8 %, less strict = 1.8 %.

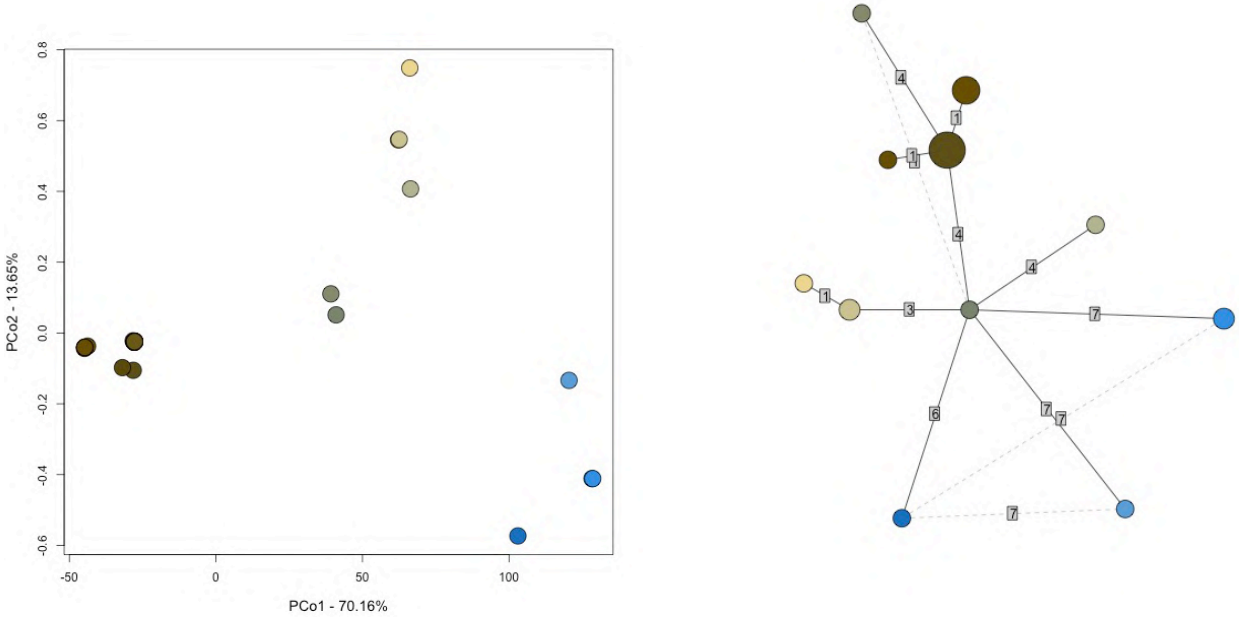

Figure 131: PCoA based on pairwise p-distances between *Camponotus ligniperda* sequences (left). Colours match a bidimensional colour space. Haplotype network of *Camponotus ligniperda* (right). Sequences > 599 bp: ID = 35, cf. = 0.

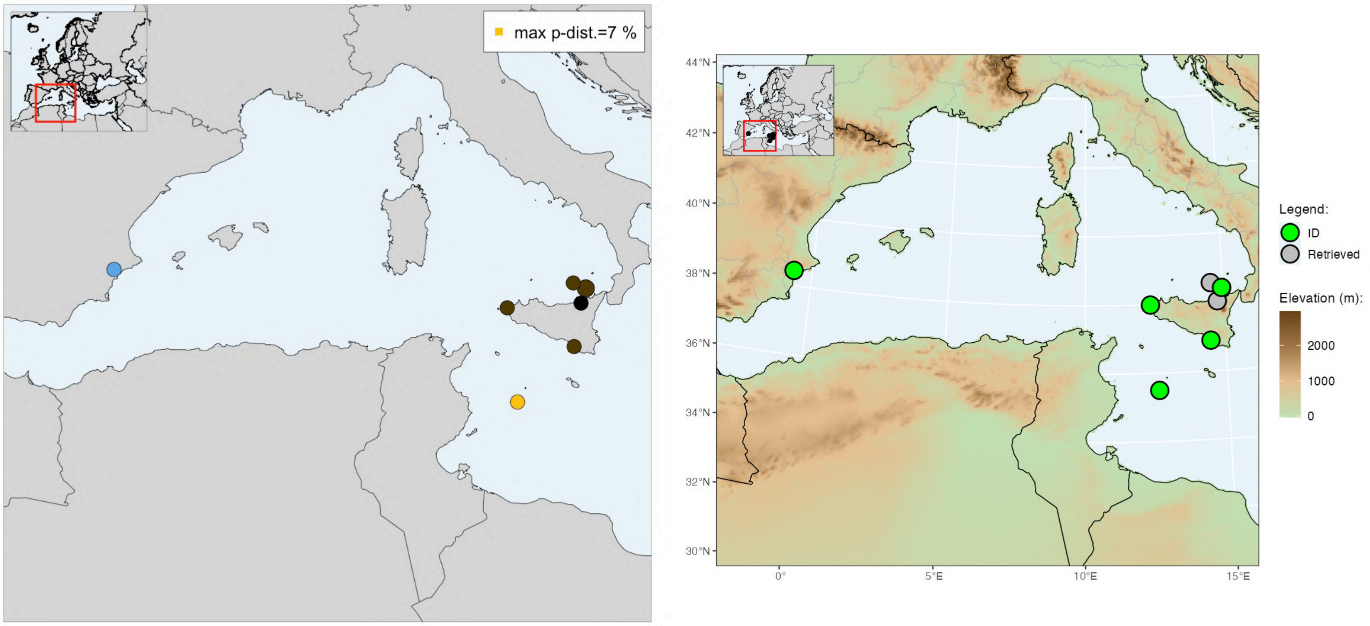

Figure 132: Genetic diversity map of *Camponotus micans* (Nylander, 1856). Nearby localities of sequenced specimens are merged in pies (left). Colours match the bidimensional colour space of the PCoA projection (Fig. 132 left) of p-dist between sequences (dots). Specimen identification (ID or cf.) and source (newly sequenced or retrieved) are represented by colours, while specimen attribute (terra typica, type locality, type specimen or faunistic novelty) is represented by the shape (right). Sequences: ID = 8, cf. = 0; maximum p-distance: strict = 7 %, less strict = 7 %.

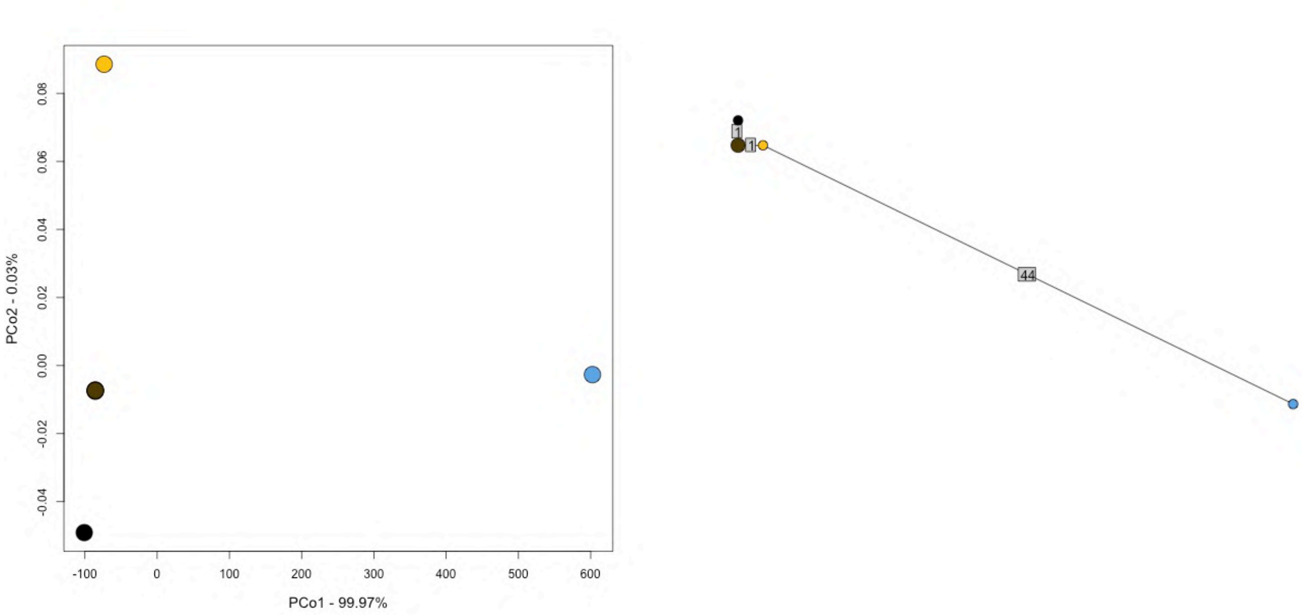

Figure 133: PCoA based on pairwise p-distances between *Camponotus micans* sequences (left). Colours match a bidimensional colour space. Haplotype network of *Camponotus micans* (right). Sequences > 599 bp: ID = 8, cf. = 0.

Camponotus nitidescens

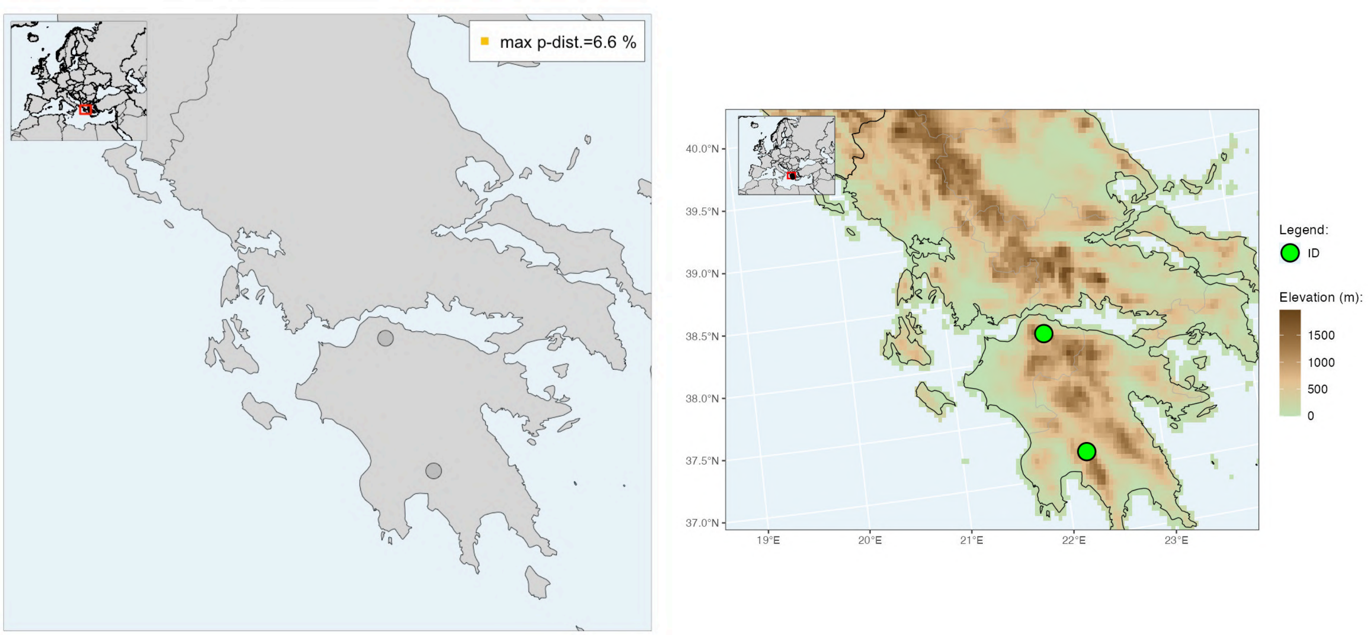

Figure 134: Genetic diversity map of *Camponotus nitidescens* Forel, 1889. PCoA projection was not done and therefore sequenced specimens in the genetic diversity map are coloured in gray (left). Specimen identification (ID or cf.) and source (newly sequenced or retrieved) are represented by colours, while specimen attribute (terra typica, type locality, type specimen or faunistic novelty) is represented by the shape (right). Sequences: ID = 2, cf. = 0; maximum p-distance: strict = NA, less strict = 6.6 %.

Haplotype network analysis of *Camponotus nitidescens* was not possible.

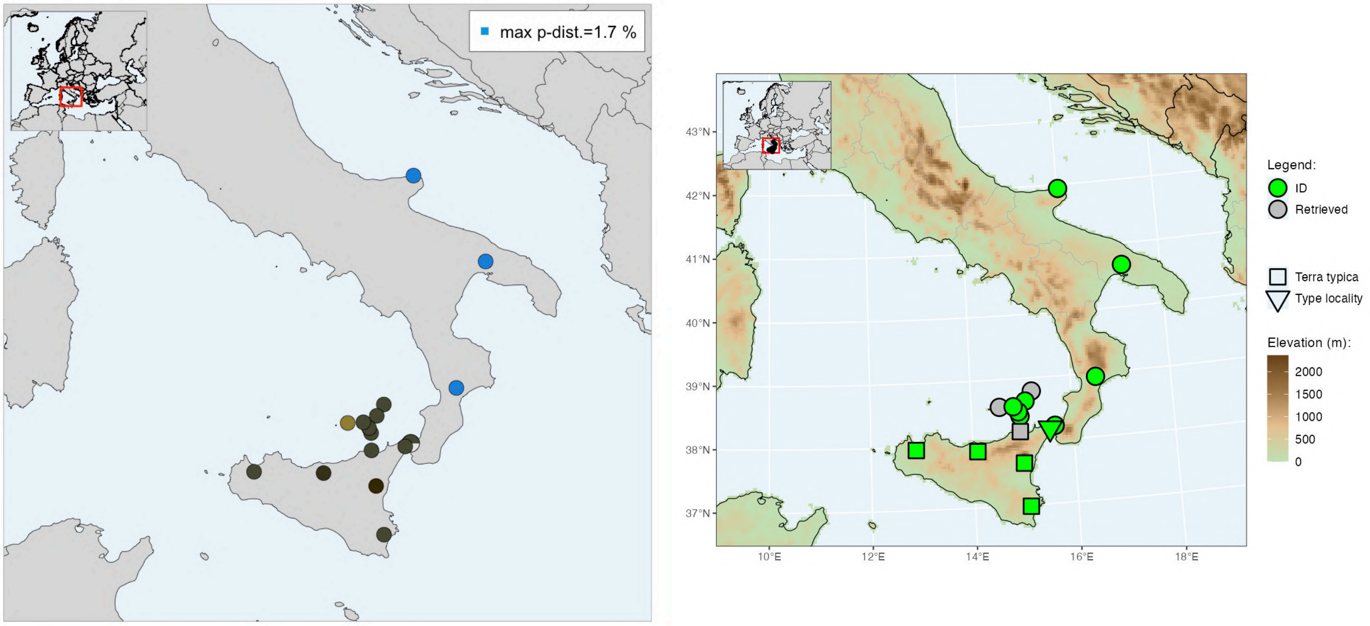

Figure 135: Genetic diversity map of *Camponotus nylanderi* Emery, 1921. Nearby localities of sequenced specimens are merged in pies (left). Colours match the bidimensional colour space of the PCoA projection (Fig. 135 left) of p-dist between sequences (dots). Specimen identification (ID or cf.) and source (newly sequenced or retrieved) are represented by colours, while specimen attribute (terra typica, type locality, type specimen or faunistic novelty) is represented by the shape (right). Sequences: ID = 17, cf. = 0; maximum p-distance: strict = 1.7 %, less strict = 1.7 %.

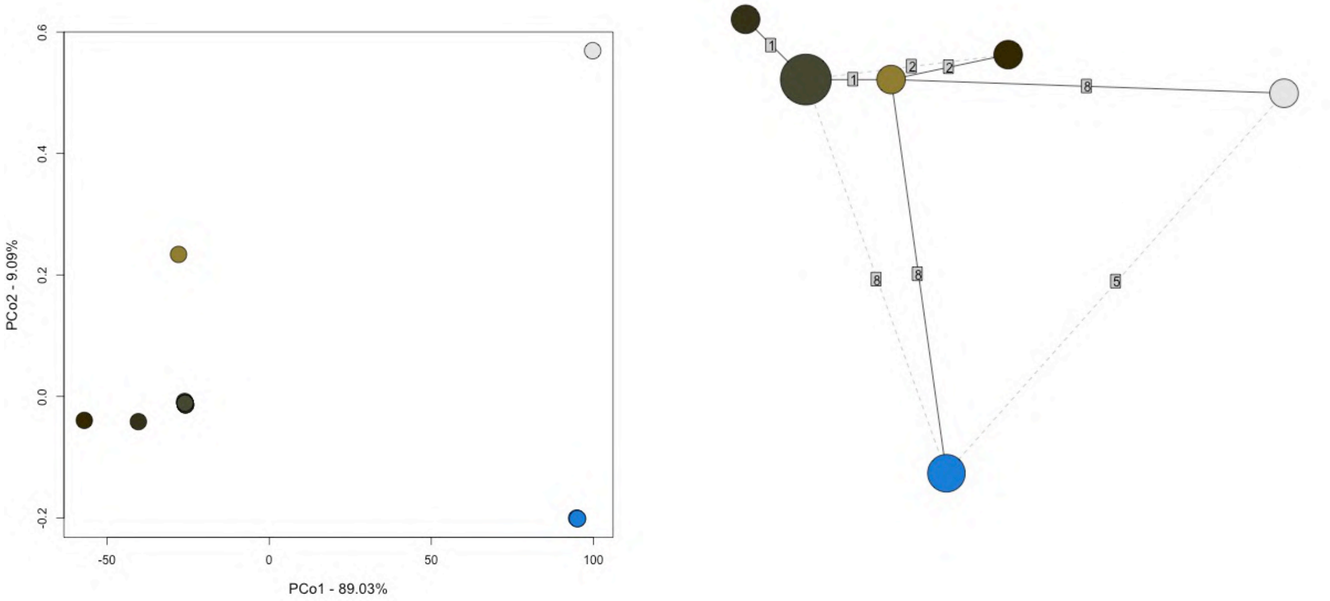

Figure 136: PCoA based on pairwise p-distances between *Camponotus nylanderi* sequences (left). Colours match a bidimensional colour space. Haplotype network of *Camponotus nylanderi* (right). Sequences > 599 bp: ID = 17, cf. = 0.

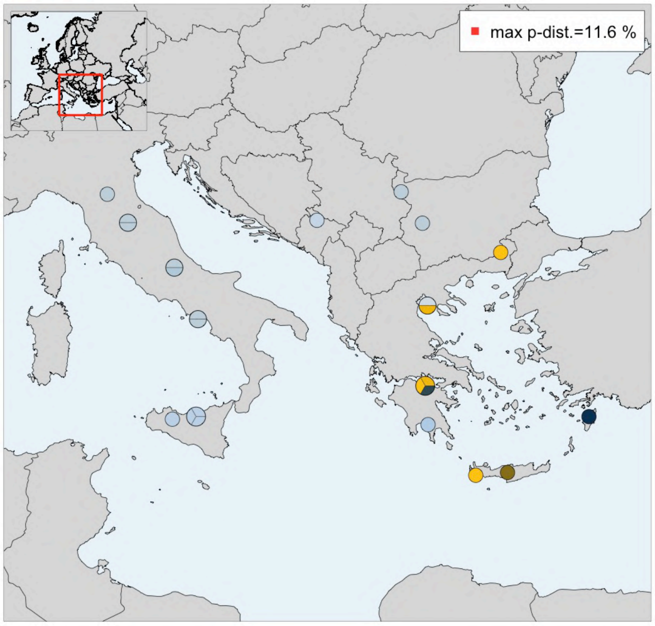

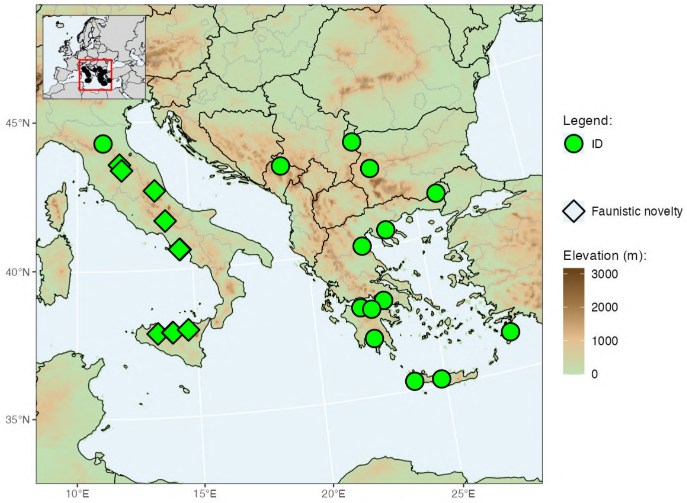

Figure 137: Genetic diversity map of *Camponotus oertzeni* Forel, 1889. Nearby localities of sequenced specimens are merged in pies (left). Colours match the bidimensional colour space of the PCoA projection (Fig. 137 left) of p-dist between sequences (dots). Specimen identification (ID or cf.) and source (newly sequenced or retrieved) are represented by colours, while specimen attribute (terra typica, type locality, type specimen or faunistic novelty) is represented by the shape (right). Sequences: ID = 24, cf. = 0; maximum p-distance: strict = 11.6 %, less strict = 11.6 %.

The species is reported for the first time in Italy.

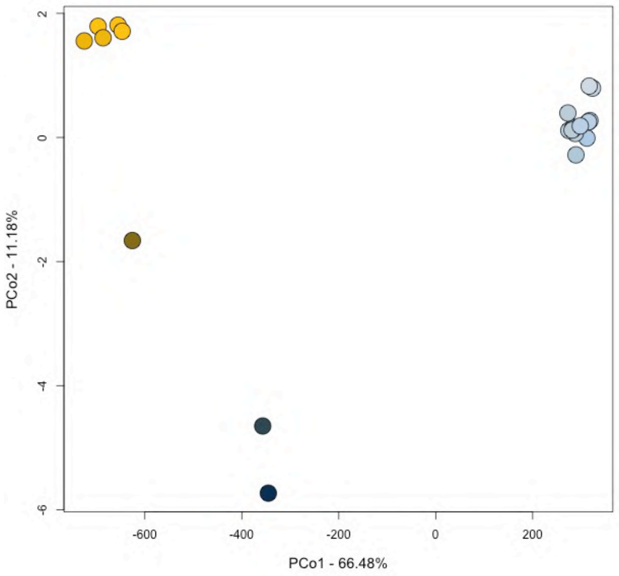

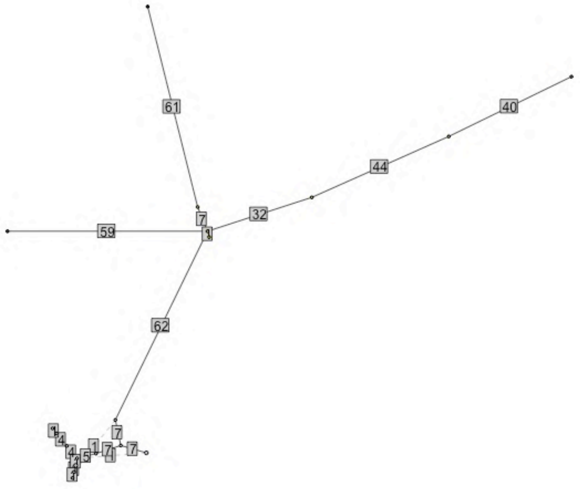

Figure 138: PCoA based on pairwise p-distances between *Camponotus oertzeni* sequences (left). Colours match a bidimensional colour space. Haplotype network of *Camponotus oertzeni* (right). Sequences > 599 bp: ID = 24, cf. = 0.

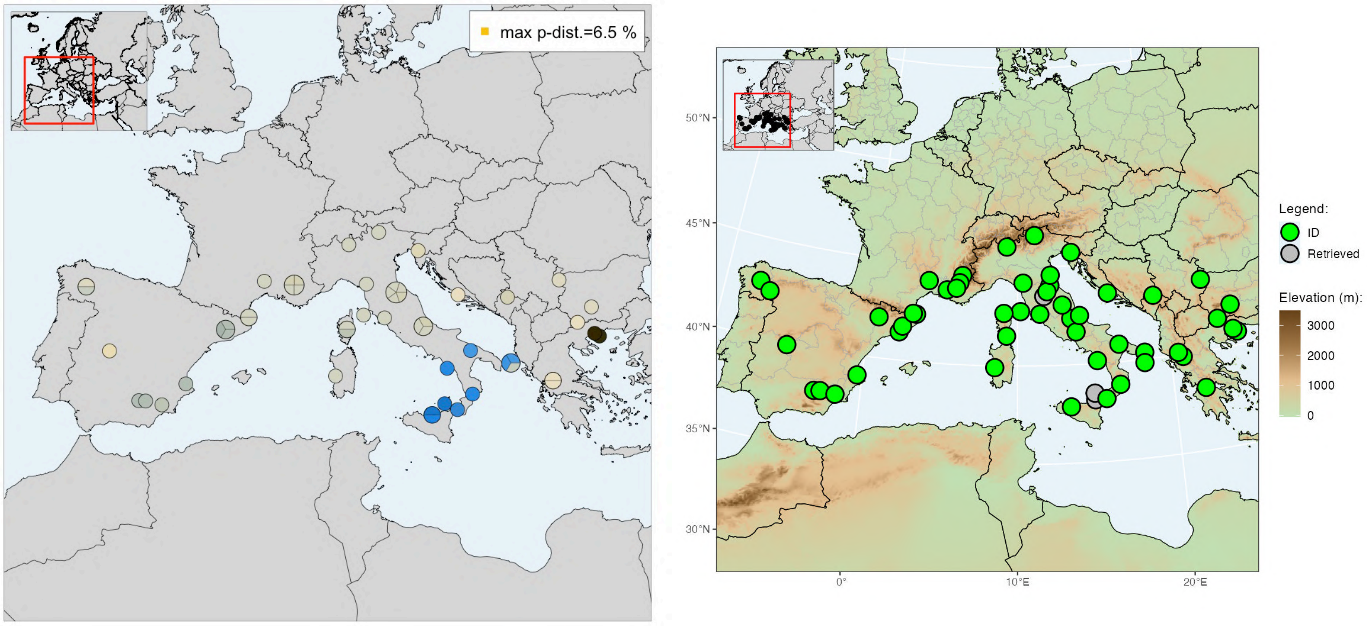

Figure 139: Genetic diversity map of *Camponotus piceus* (Leach, 1825). Nearby localities of sequenced specimens are merged in pies (left). Colours match the bidimensional colour space of the PCoA projection (Fig. 139 left) of p-dist between sequences (dots). Specimen identification (ID or cf.) and source (newly sequenced or retrieved) are represented by colours, while specimen attribute (terra typica, type locality, type specimen or faunistic novelty) is represented by the shape (right). Sequences: ID = 53, cf. = 0; maximum p-distance: strict = 6.5 %, less strict = 6.5 %.

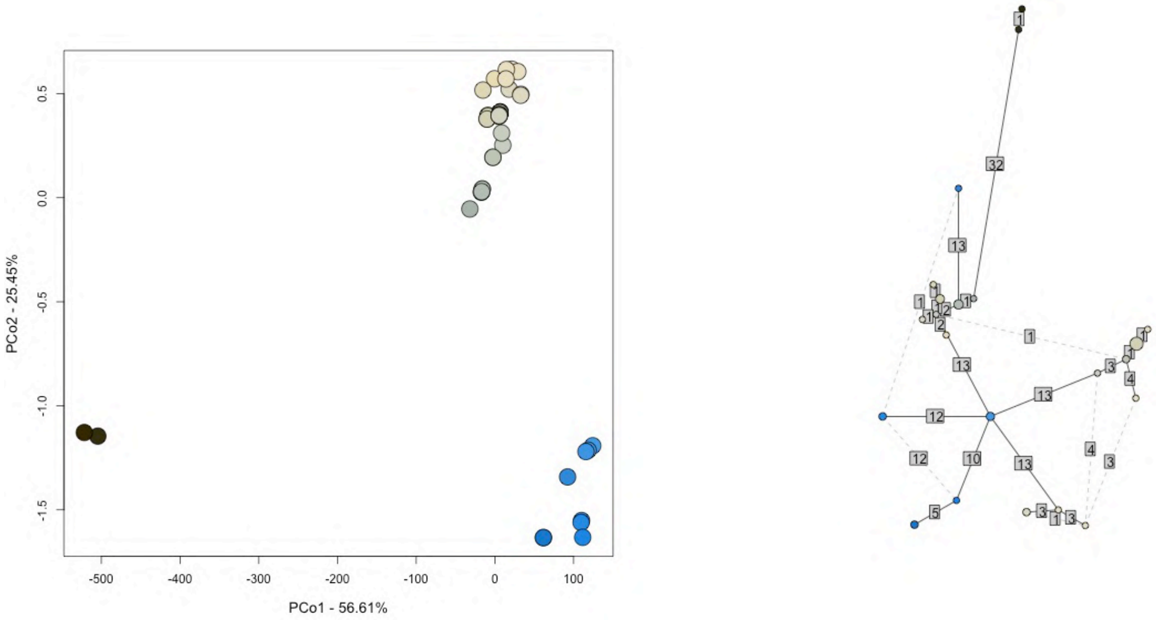

Figure 140: PCoA based on pairwise p-distances between *Camponotus piceus* sequences (left). Colours match a bidimensional colour space. Haplotype network of *Camponotus piceus* (right). Sequences > 599 bp: ID = 53, cf. = 0.

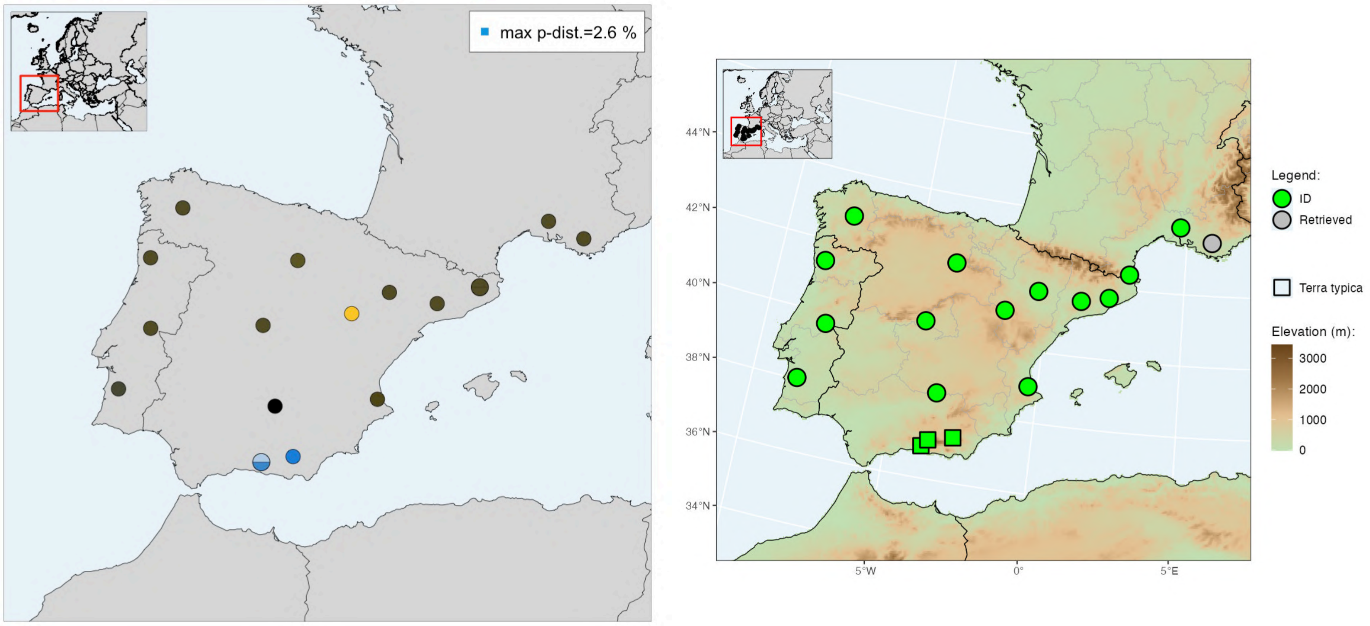

Figure 141: Genetic diversity map of *Camponotus pilicornis* (Roger, 1859). Nearby localities of sequenced specimens are merged in pies (left). Colours match the bidimensional colour space of the PCoA projection (Fig. 141 left) of p-dist between sequences (dots). Specimen identification (ID or cf.) and source (newly sequenced or retrieved) are represented by colours, while specimen attribute (terra typica, type locality, type specimen or faunistic novelty) is represented by the shape (right). Sequences: ID = 18, cf. = 0; maximum p-distance: strict = 2.6 %, less strict = 2.6 %.

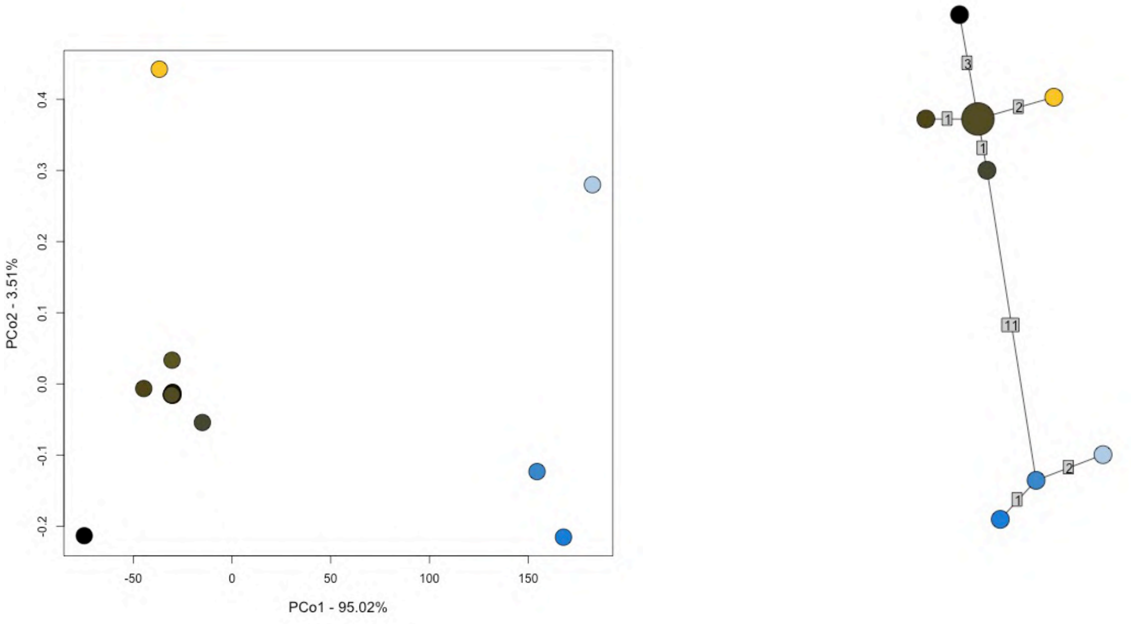

Figure 142: PCoA based on pairwise p-distances between *Camponotus pilicornis* sequences (left). Colours match a bidimensional colour space. Haplotype network of *Camponotus pilicornis* (right). Sequences > 599 bp: ID = 18, cf. = 0.

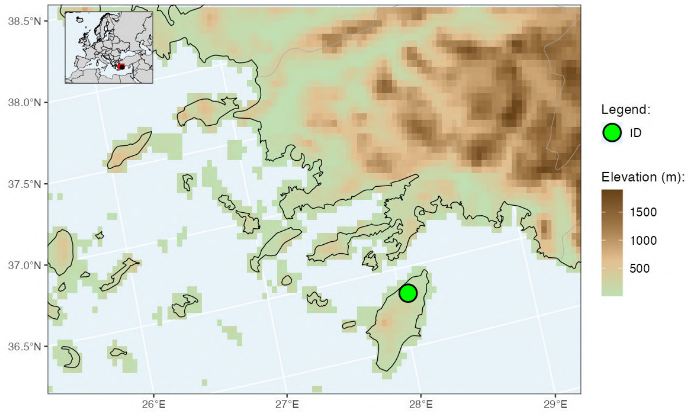

Figure 143: Map of *Camponotus rebeccae* Forel, 1913. Due to the presence of a single sequence, the genetic diversity map and the PCoA projection were not done. Specimen identification (ID or cf.) and source (newly sequenced or retrieved) are represented by colours, while specimen attribute (terra typica, type locality, type specimen or faunistic novelty) is represented by the shape. Sequences: ID = 1, cf. = 0; maximum p-distance: strict = NA, less strict = NA.

Haplotype network analysis of *Camponotus rebeccae* was not possible.

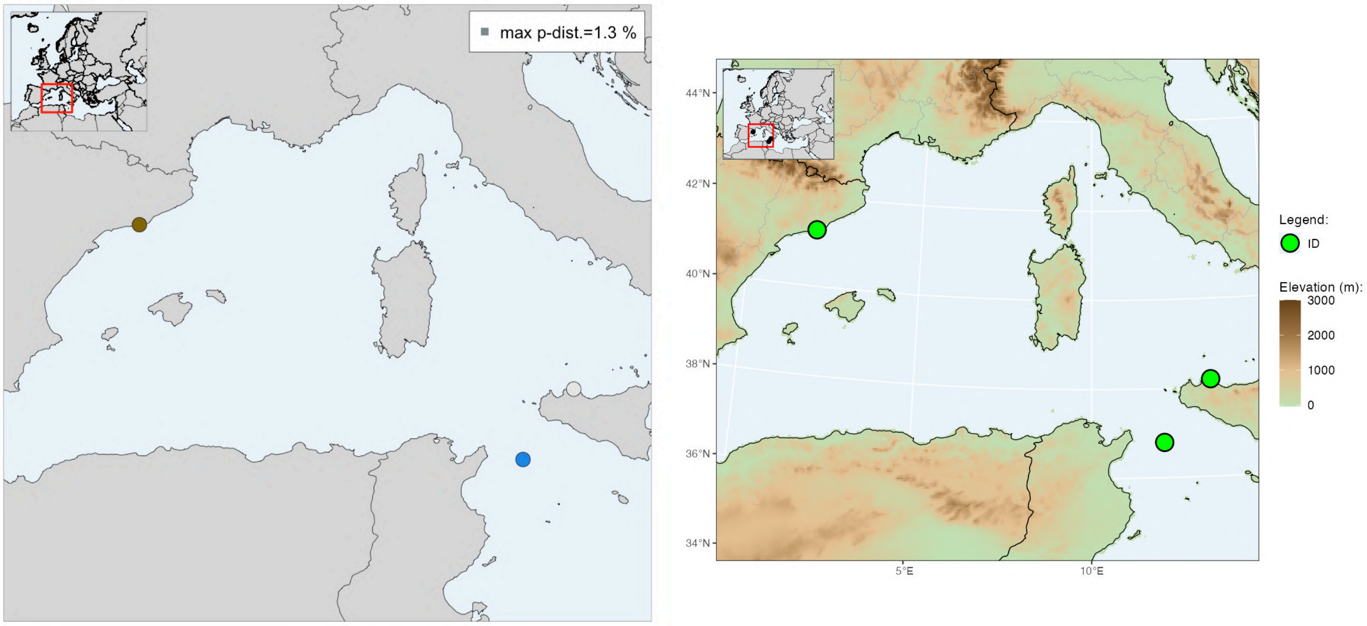

Figure 144: Genetic diversity map of *Camponotus ruber* Emery, 1925. Nearby localities of sequenced specimens are merged in pies (left). Colours match the bidimensional colour space of the PCoA projection (Fig. 144 left) of p-dist between sequences (dots). Specimen identification (ID or cf.) and source (newly sequenced or retrieved) are represented by colours, while specimen attribute (terra typica, type locality, type specimen or faunistic novelty) is represented by the shape (right). Sequences: ID = 5, cf. = 0; maximum p-distance: strict = NA, less strict = 1.3 %.

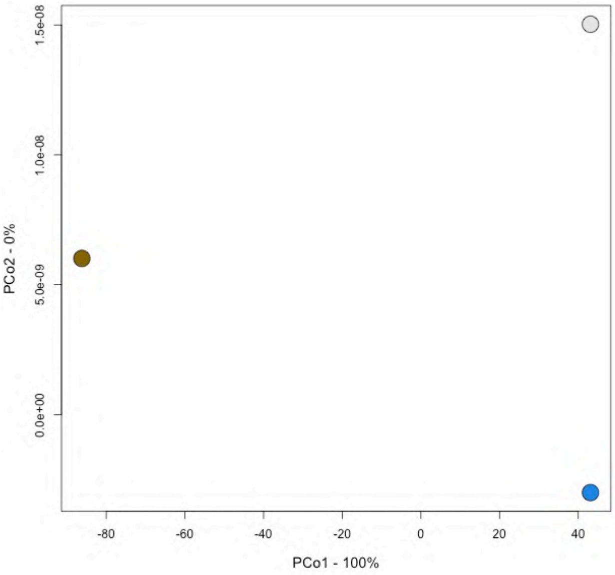

Figure 145: PCoA based on pairwise p-distances between *Camponotus ruber* sequences (left). Colours match a bidimensional colour space. Haplotype network analysis of *Camponotus ruber* was not possible.

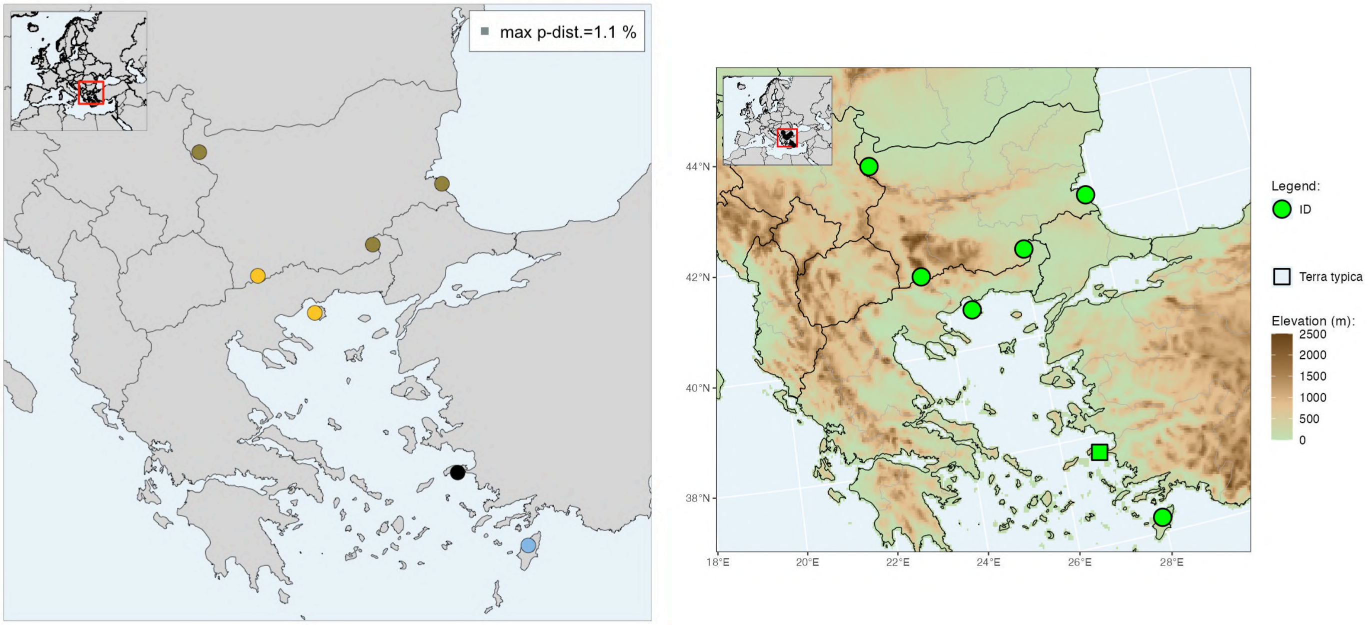

Figure 146: Genetic diversity map of *Camponotus samius* Forel, 1889. Nearby localities of sequenced specimens are merged in pies (left). Colours match the bidimensional colour space of the PCoA projection (Fig. 146 left) of p-dist between sequences (dots). Specimen identification (ID or cf.) and source (newly sequenced or retrieved) are represented by colours, while specimen attribute (terra typica, type locality, type specimen or faunistic novelty) is represented by the shape (right). Sequences: ID = 7, cf. = 0; maximum p-distance: strict = 1.1 %, less strict = 1.1 %.

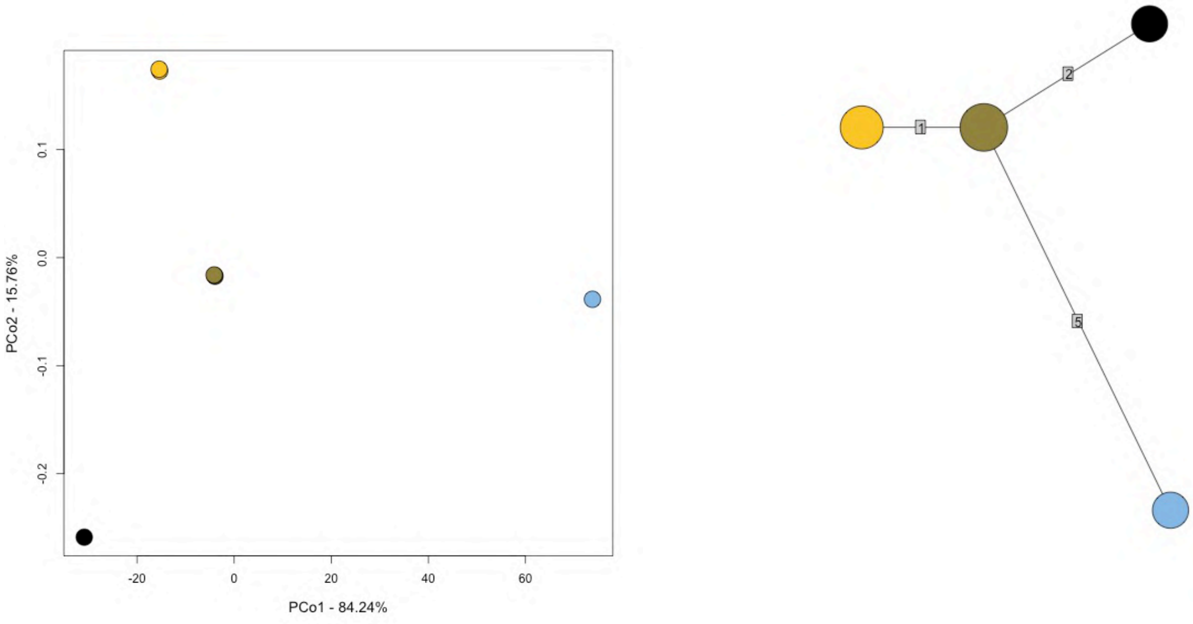

Figure 147: PCoA based on pairwise p-distances between *Camponotus samius* sequences (left). Colours match a bidimensional colour space. Haplotype network of *Camponotus samius* (right). Sequences > 599 bp: ID = 7, cf. = 0.

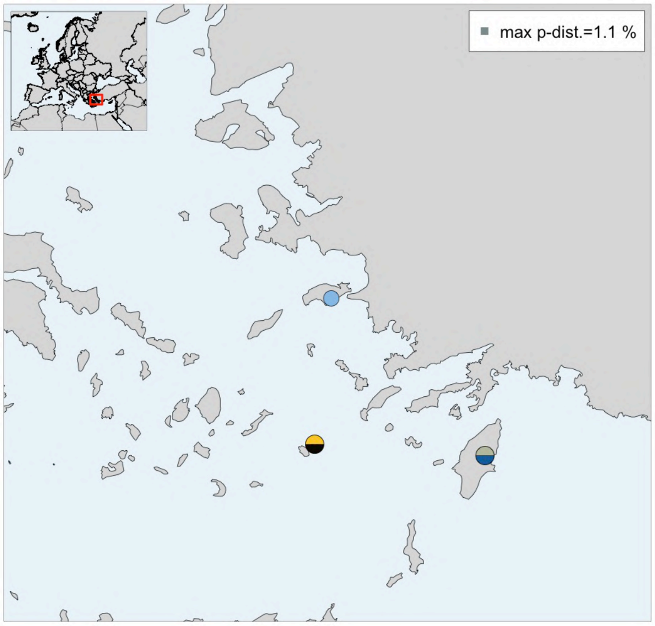

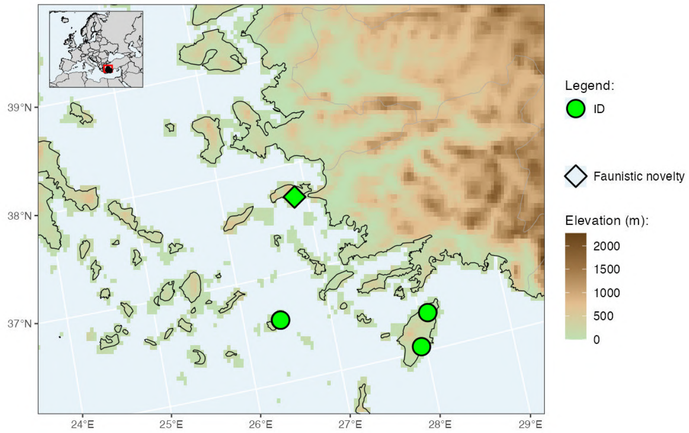

Figure 148: Genetic diversity map of *Camponotus sanctus* Forel, 1904. Nearby localities of sequenced specimens are merged in pies (left). Colours match the bidimensional colour space of the PCoA projection (Fig. 148 left) of p-dist between sequences (dots). Specimen identification (ID or cf.) and source (newly sequenced or retrieved) are represented by colours, while specimen attribute (terra typica, type locality, type specimen or faunistic novelty) is represented by the shape (right). Sequences: ID = 5, cf. = 0; maximum p-distance: strict = 1.1 %, less strict = 1.1 %.

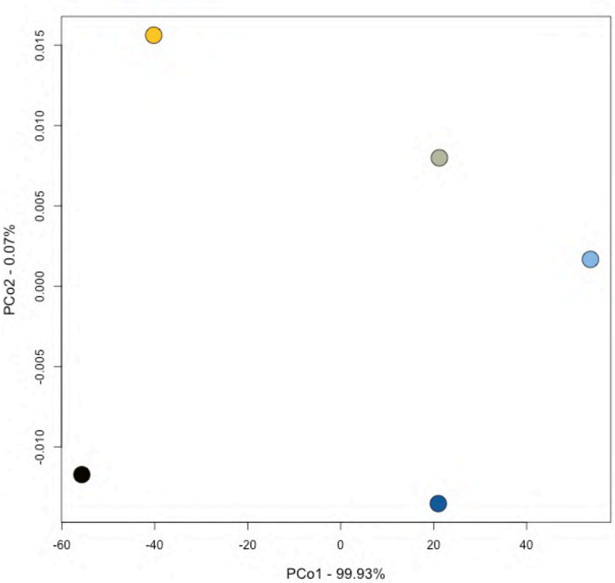

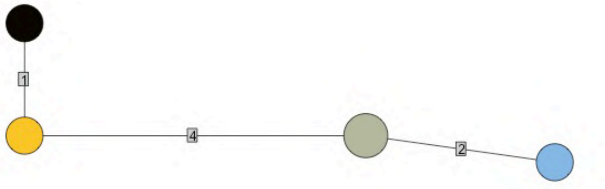

Figure 149: PCoA based on pairwise p-distances between *Camponotus sanctus* sequences (left). Colours match a bidimensional colour space. Haplotype network of *Camponotus sanctus* (right). Sequences > 599 bp: ID = 5, cf. = 0.

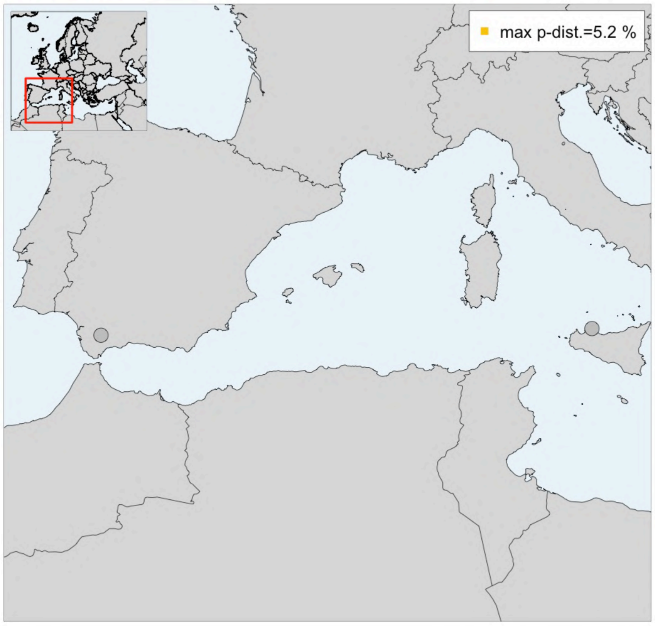

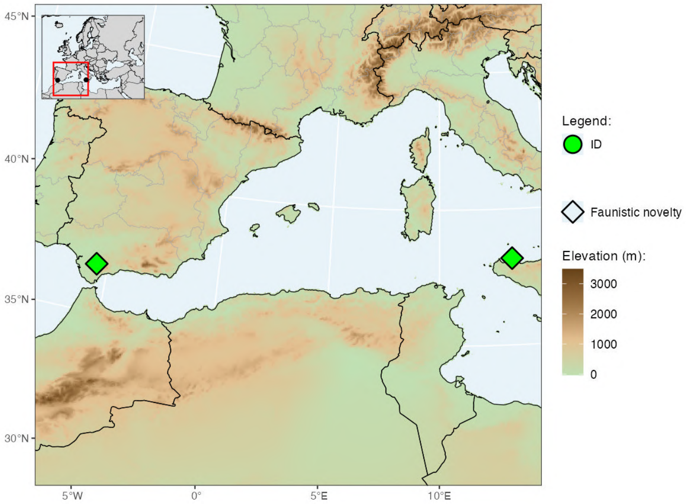

Figure 150: Genetic diversity map of *Camponotus spissinodis* Forel, 1909. PCoA projection was not done and therefore sequenced specimens in the genetic diversity map are coloured in gray (left). Specimen identification (ID or cf.) and source (newly sequenced or retrieved) are represented by colours, while specimen attribute (terra typica, type locality, type specimen or faunistic novelty) is represented by the shape (right). Sequences: ID = 2, cf. = 0; maximum p-distance: strict = NA, less strict = 5.2 %.

The species is reported for the first time in Europe in Italy and Spain.

Haplotype network analysis of *Camponotus spissinodis* was not possible.

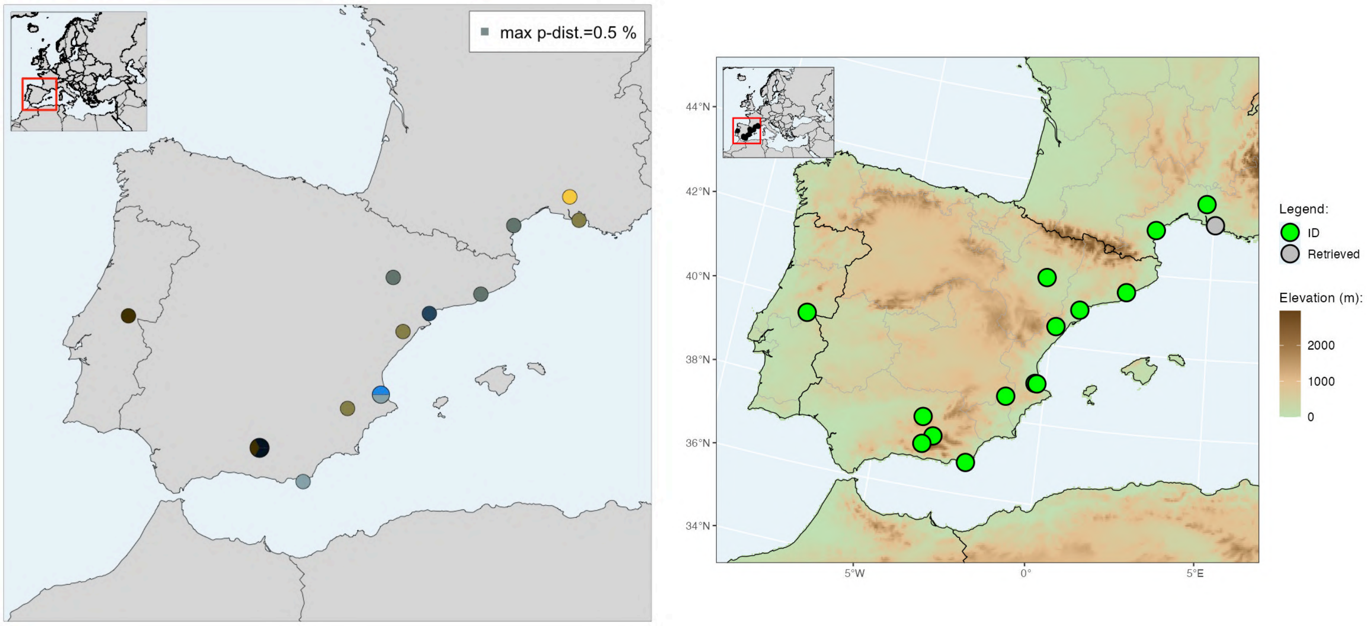

Figure 151: Genetic diversity map of *Camponotus sylvaticus* (Olivier, 1792). Nearby localities of sequenced specimens are merged in pies (left). Colours match the bidimensional colour space of the PCoA projection (Fig. 151 left) of p-dist between sequences (dots). Specimen identification (ID or cf.) and source (newly sequenced or retrieved) are represented by colours, while specimen attribute (terra typica, type locality, type specimen or faunistic novelty) is represented by the shape (right). Sequences: ID = 15, cf. = 0; maximum p-distance: strict = 0.5 %, less strict = 0.5 %.

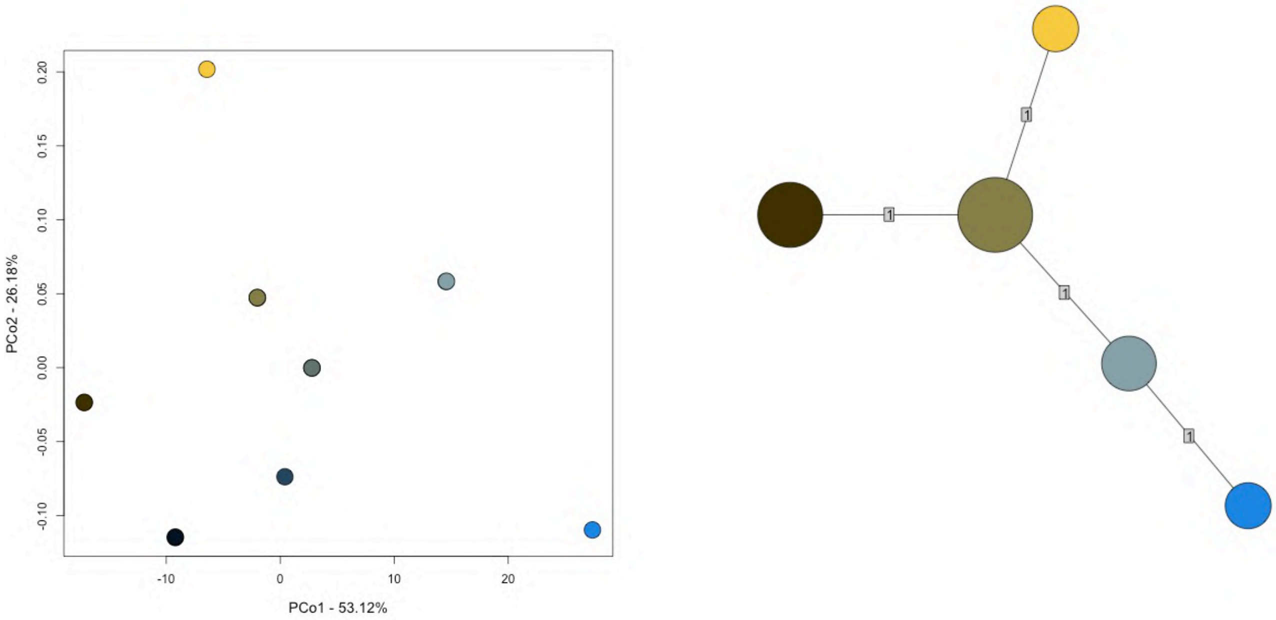

Figure 152: PCoA based on pairwise p-distances between *Camponotus sylvaticus* sequences (left). Colours match a bidimensional colour space. Haplotype network of *Camponotus sylvaticus* (right). Sequences > 599 bp: ID = 15, cf. = 0.

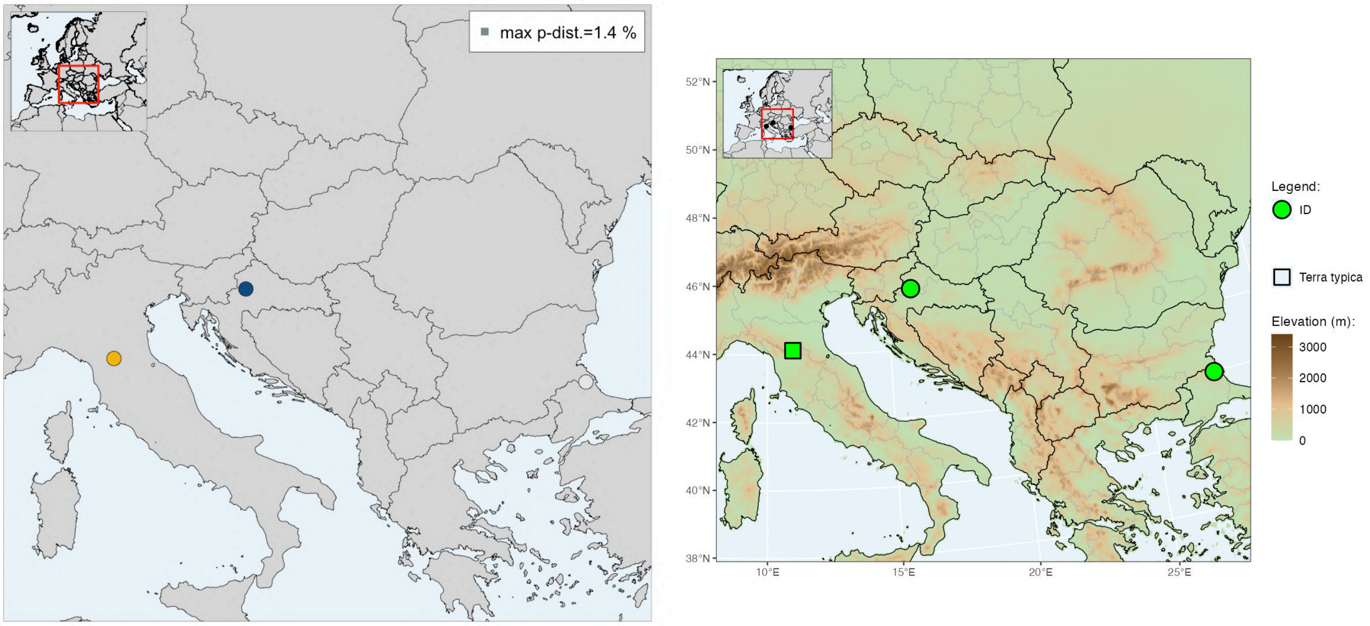

Figure 153: Genetic diversity map of *Camponotus tergestinus* Müller, 1921. Nearby localities of sequenced specimens are merged in pies (left). Colours match the bidimensional colour space of the PCoA projection (Fig. 153 left) of p-dist between sequences (dots). Specimen identification (ID or cf.) and source (newly sequenced or retrieved) are represented by colours, while specimen attribute (terra typica, type locality, type specimen or faunistic novelty) is represented by the shape (right). Sequences: ID = 3, cf. = 0; maximum p-distance: strict = 1.4 %, less strict = 1.4 %.

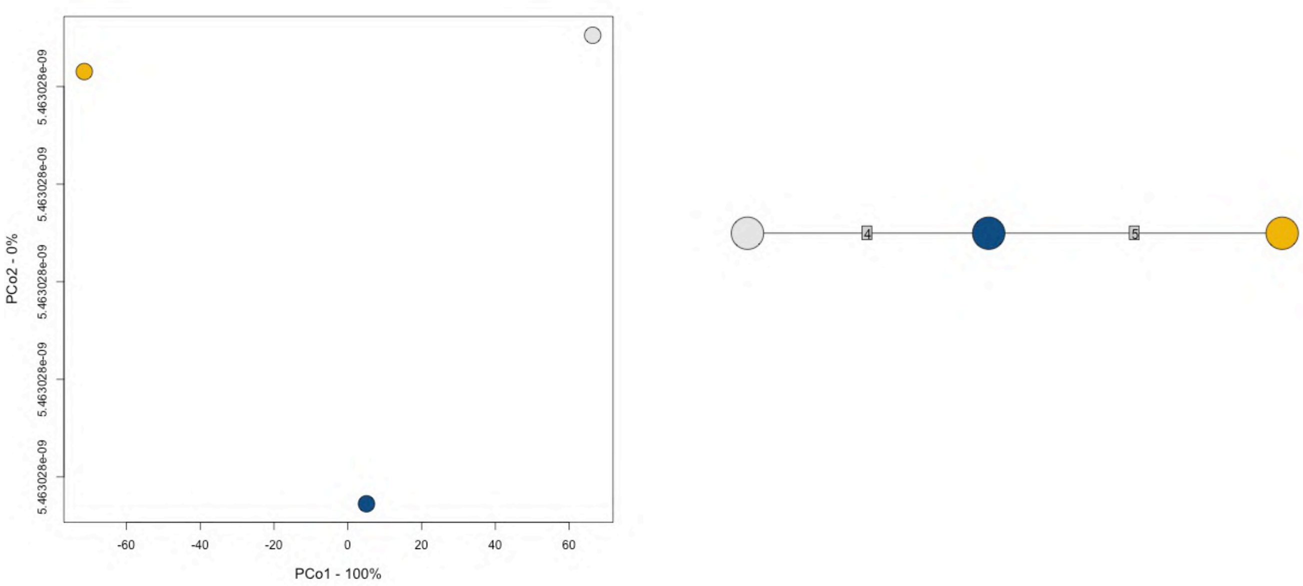

Figure 154: PCoA based on pairwise p-distances between *Camponotus tergestinus* sequences (left). Colours match a bidimensional colour space. Haplotype network of *Camponotus tergestinus* (right). Sequences > 599 bp: ID = 3, cf. = 0.

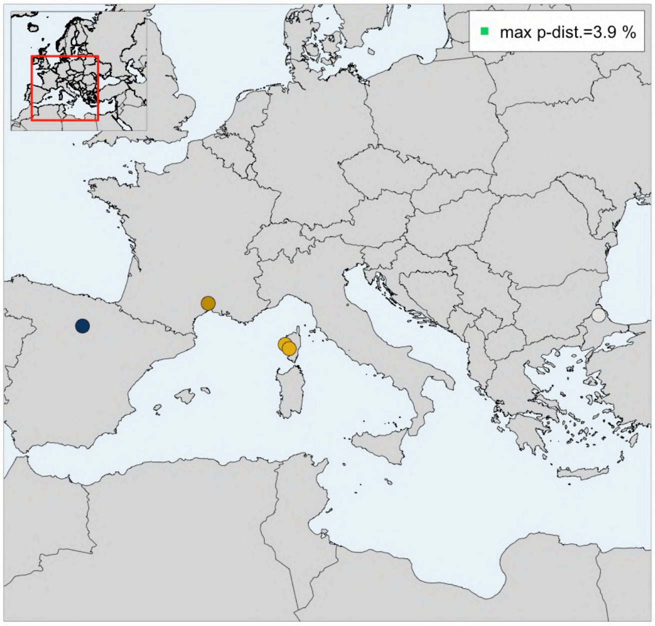

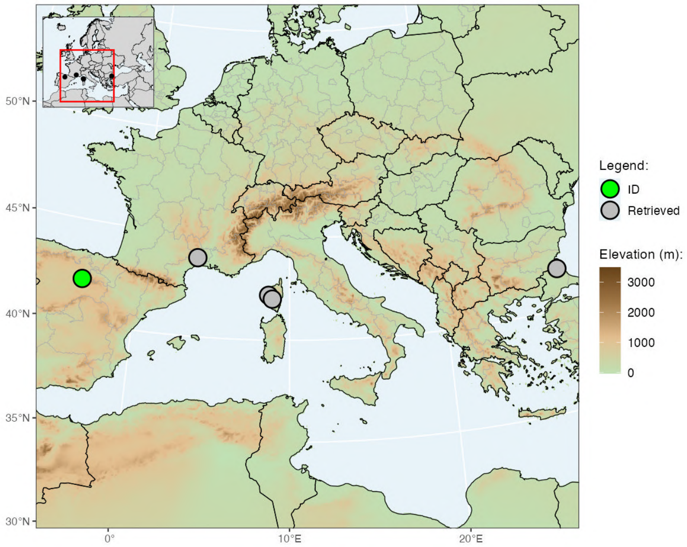

Figure 155: Genetic diversity map of *Camponotus universitatis* Forel, 1890. Nearby localities of sequenced specimens are merged in pies (left). Colours match the bidimensional colour space of the PCoA projection (Fig. 155 left) of p-dist between sequences (dots). Specimen identification (ID or cf.) and source (newly sequenced or retrieved) are represented by colours, while specimen attribute (terra typica, type locality, type specimen or faunistic novelty) is represented by the shape (right). Sequences: ID = 5, cf. = 0; maximum p-distance: strict = 3.8 %, less strict = 3.9 %.

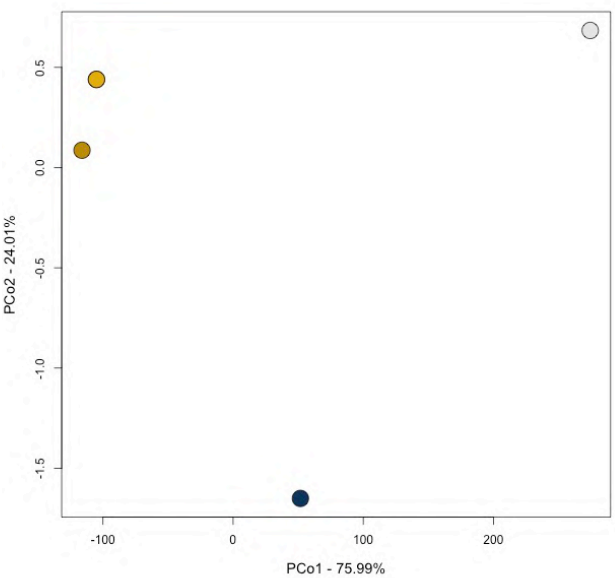

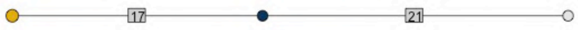

Figure 156: PCoA based on pairwise p-distances between *Camponotus universitatis* sequences (left). Colours match a bidimensional colour space. Haplotype network of *Camponotus universitatis* (right). Sequences > 599 bp: ID = 4, cf. = 0.

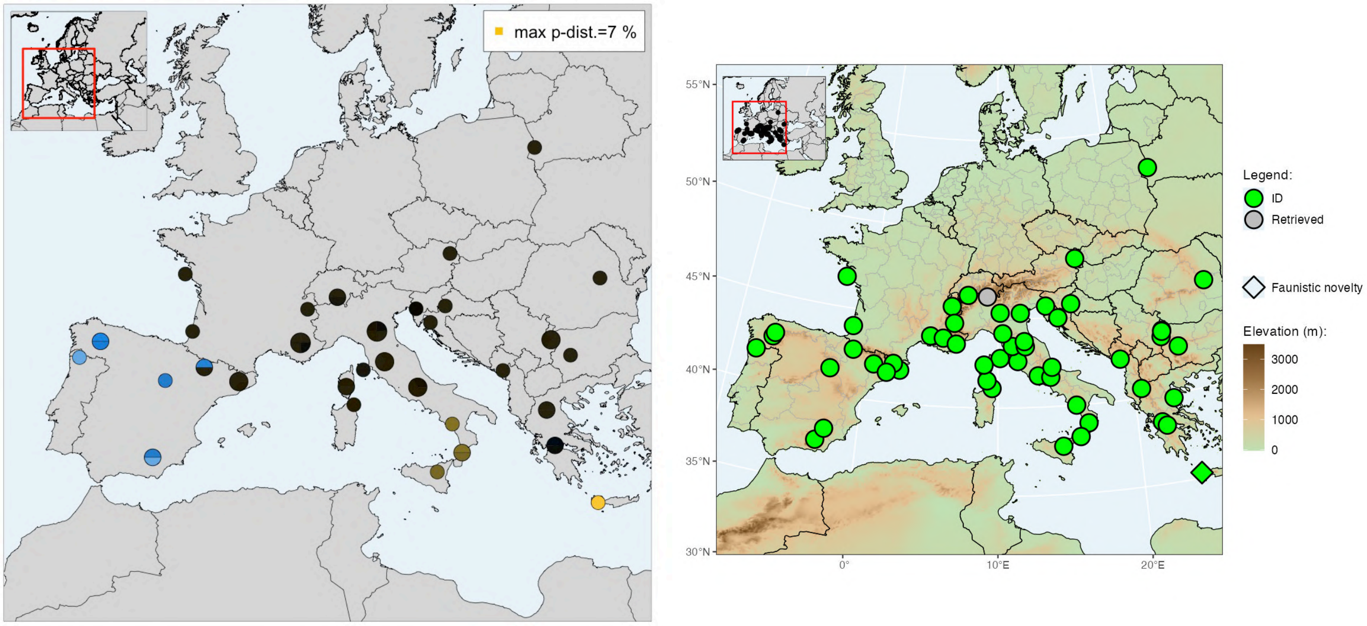

Figure 157: Genetic diversity map of *Camponotus vagus* (Scopoli, 1763). Nearby localities of sequenced specimens are merged in pies (left). Colours match the bidimensional colour space of the PCoA projection (Fig. 157 left) of p-dist between sequences (dots). Specimen identification (ID or cf.) and source (newly sequenced or retrieved) are represented by colours, while specimen attribute (terra typica, type locality, type specimen or faunistic novelty) is represented by the shape (right). Sequences: ID = 54, cf. = 0; maximum p-distance: strict = 7 %, less strict = 7 %.

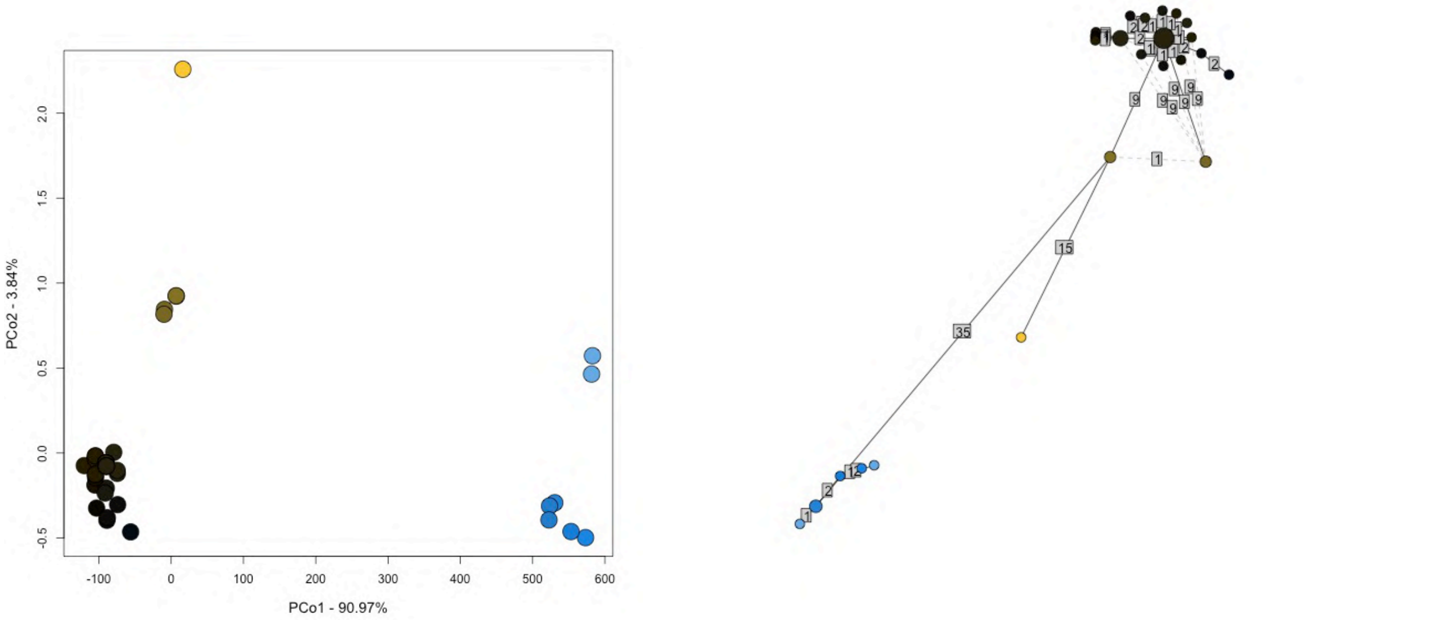

Figure 158: PCoA based on pairwise p-distances between *Camponotus vagus* sequences (left). Colours match a bidimensional colour space. Haplotype network of *Camponotus vagus* (right). Sequences > 599 bp: ID = 54, cf. = 0.

Cardiocondyla

Cardiocondyla batesii

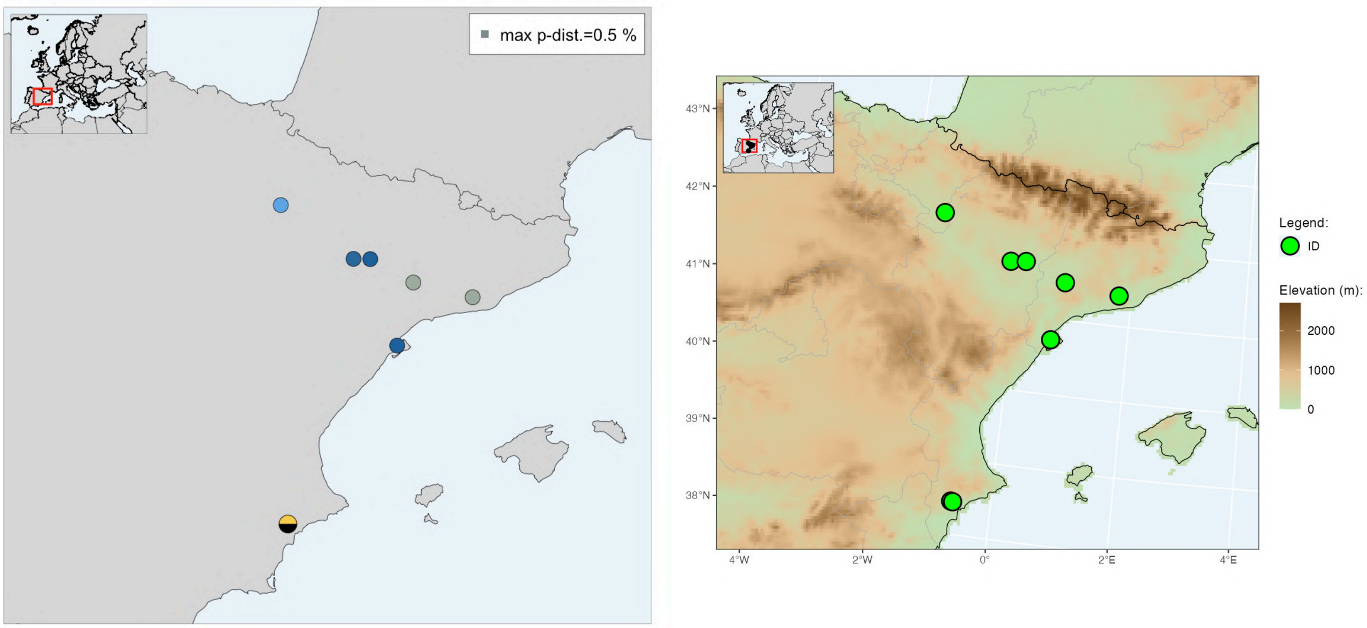

Figure 159: Genetic diversity map of *Cardiocondyla batesii* Forel, 1894. Nearby localities of sequenced specimens are merged in pies (left). Colours match the bidimensional colour space of the PCoA projection (Fig. 159 left) of p-dist between sequences (dots). Specimen identification (ID or cf.) and source (newly sequenced or retrieved) are represented by colours, while specimen attribute (terra typica, type locality, type specimen or faunistic novelty) is represented by the shape (right). Sequences: ID = 8, cf. = 0; maximum p-distance: strict = 0.5 %, less strict = 0.5 %.

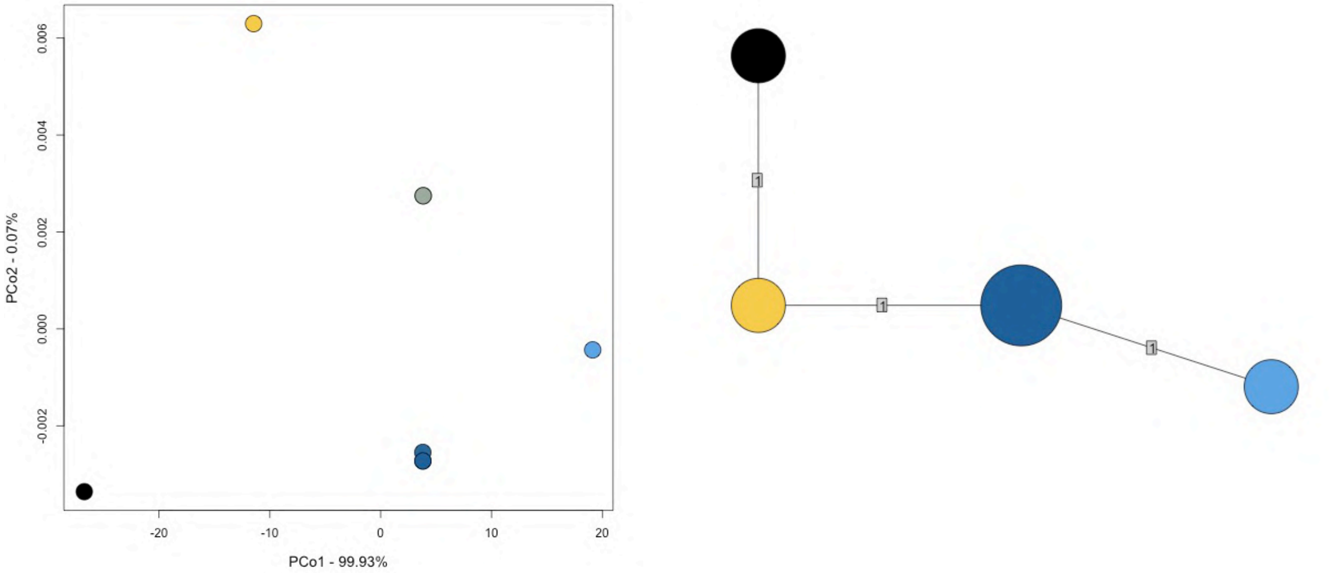

Figure 160: PCoA based on pairwise p-distances between *Cardiocondyla batesii* sequences (left). Colours match a bidimensional colour space. Haplotype network of *Cardiocondyla batesii* (right). Sequences > 599 bp: ID = 8, cf. = 0.

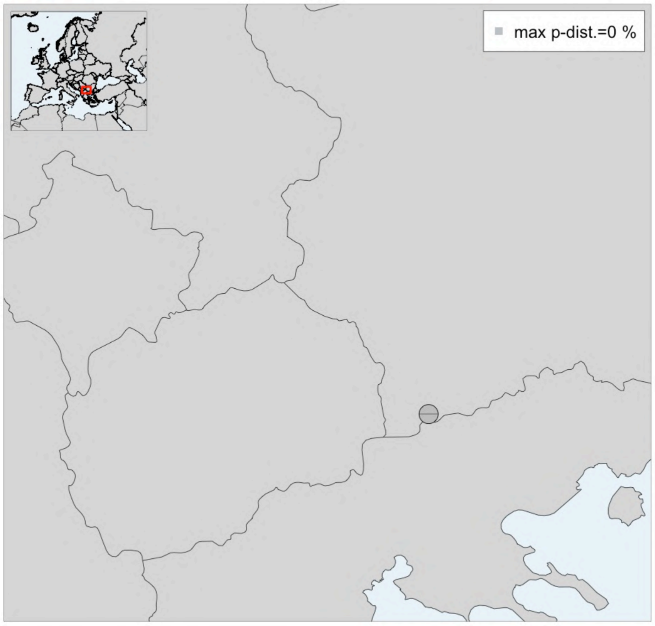

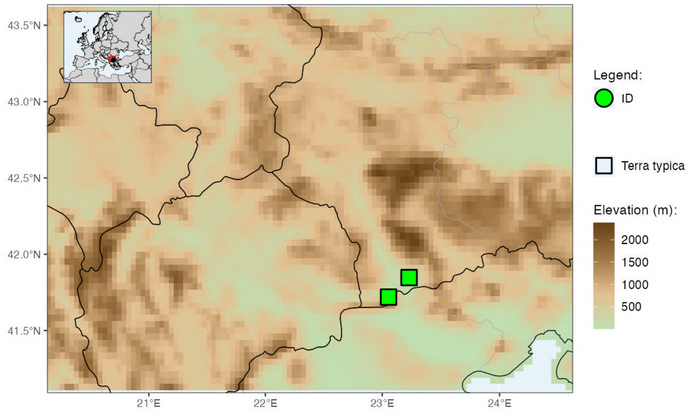

Figure 161: Genetic diversity map of *Cardiocondyla bulgarica* Forel, 1892. PCoA projection was not done and therefore sequenced specimens in the genetic diversity map are coloured in gray (left). Specimen identification (ID or cf.) and source (newly sequenced or retrieved) are represented by colours, while specimen attribute (terra typica, type locality, type specimen or faunistic novelty) is represented by the shape (right). Sequences: ID = 2, cf. = 0; maximum p-distance: strict = NA, less strict = 0 %.

Haplotype network analysis of *Cardiocondyla bulgarica* was not possible.

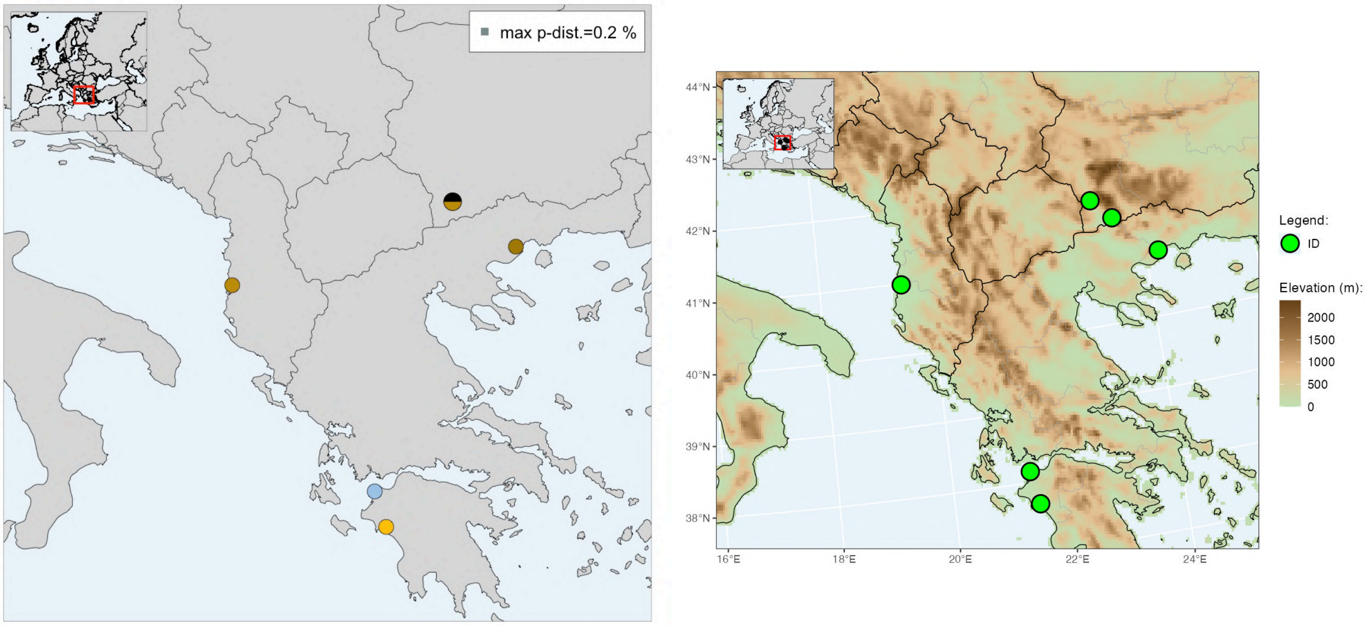

Figure 162: Genetic diversity map of *Cardiocondyla dalmatica* Soudek, 1925. Nearby localities of sequenced specimens are merged in pies (left). Colours match the bidimensional colour space of the PCoA projection (Fig. 162 left) of p-dist between sequences (dots). Specimen identification (ID or cf.) and source (newly sequenced or retrieved) are represented by colours, while specimen attribute (terra typica, type locality, type specimen or faunistic novelty) is represented by the shape (right). Sequences: ID = 6, cf. = 0; maximum p-distance: strict = 0.2 %, less strict = 0.2 %.

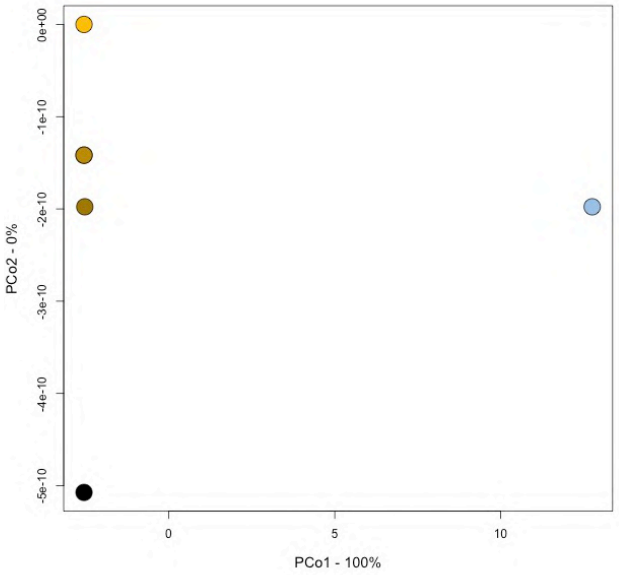

Figure 163: PCoA based on pairwise p-distances between *Cardiocondyla dalmatica* sequences (left). Colours match a bidimensional colour space. Haplotype network analysis of *Cardiocondyla dalmatica* was not possible.

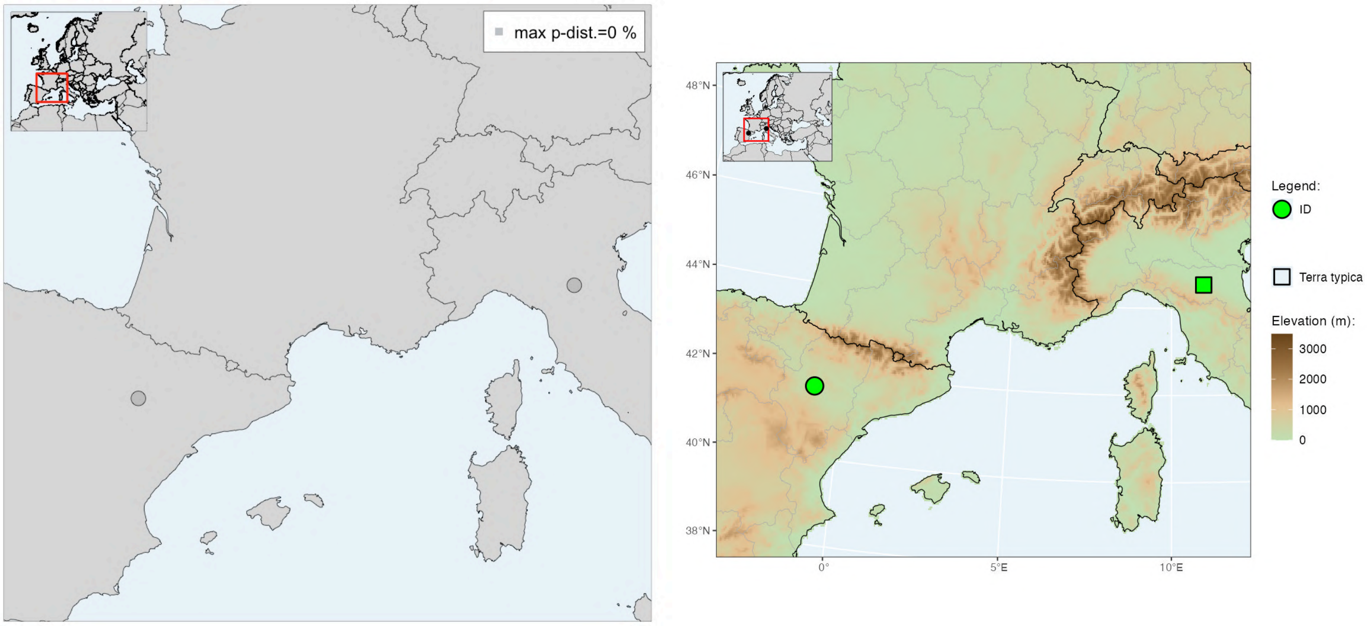

Figure 164: Genetic diversity map of *Cardiocondyla elegans* Emery, 1869. PCoA projection was not done and therefore sequenced specimens in the genetic diversity map are coloured in gray (left). Specimen identification (ID or cf.) and source (newly sequenced or retrieved) are represented by colours, while specimen attribute (terra typica, type locality, type specimen or faunistic novelty) is represented by the shape (right). Sequences: ID = 2, cf. = 0; maximum p-distance: strict = NA, less strict = 0 %.

Haplotype network analysis of *Cardiocondyla elegans* was not possible.

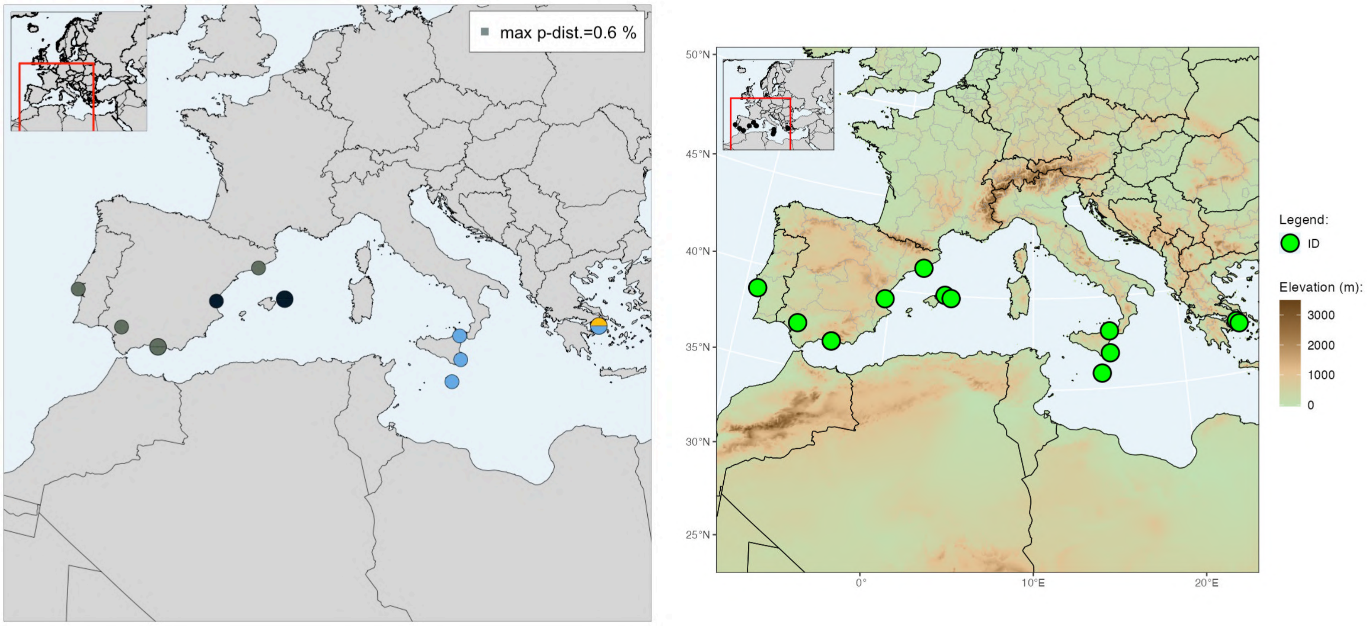

Figure 165: Genetic diversity map of *Cardiocondyla mauritanica* Forel, 1890. Nearby localities of sequenced specimens are merged in pies (left). Colours match the bidimensional colour space of the PCoA projection (Fig. 165 left) of p-dist between sequences (dots). Specimen identification (ID or cf.) and source (newly sequenced or retrieved) are represented by colours, while specimen attribute (terra typica, type locality, type specimen or faunistic novelty) is represented by the shape (right). Sequences: ID = 16, cf. = 0; maximum p-distance: strict = 0.6 %, less strict = 0.6 %.

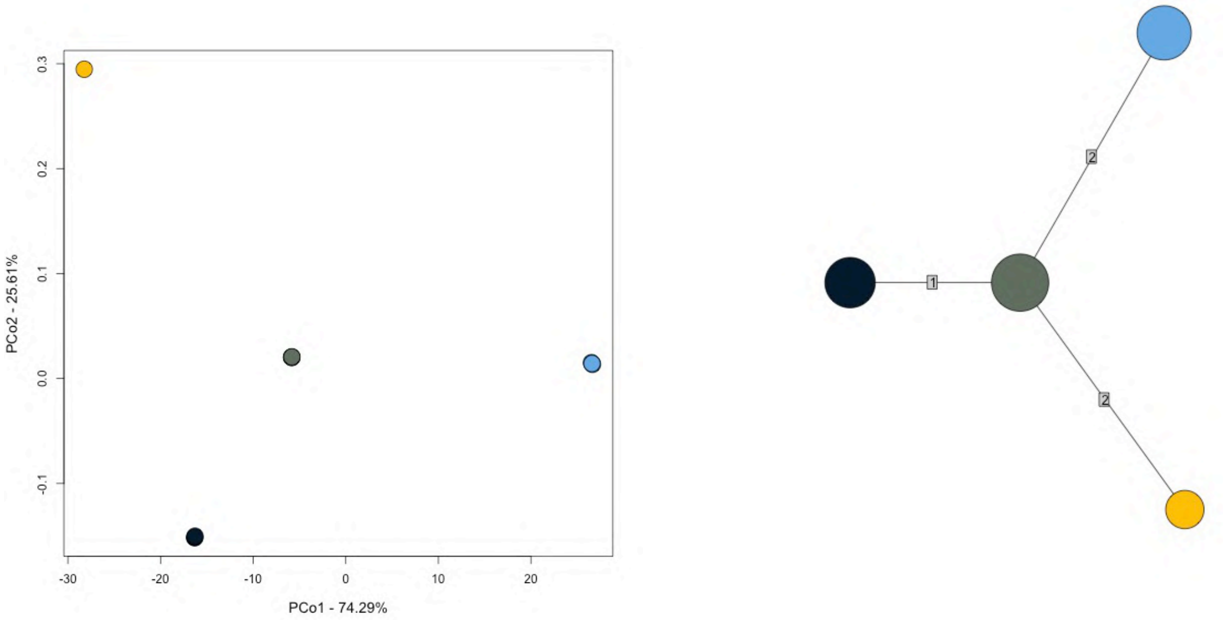

Figure 166: PCoA based on pairwise p-distances between *Cardiocondyla mauritanica* sequences (left). Colours match a bidimensional colour space. Haplotype network of *Cardiocondyla mauritanica* (right). Sequences > 599 bp: ID = 16, cf. = 0.

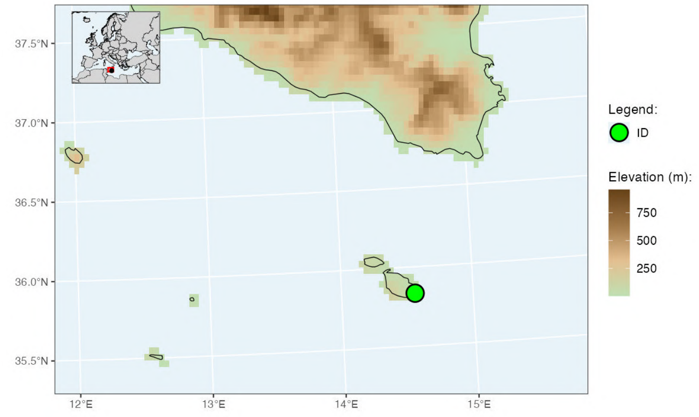

Figure 167: Map of *Cardiocondyla nigra* Forel, 1905. Due to the presence of a single sequence, the genetic diversity map and the PCoA projection were not done. Specimen identification (ID or cf.) and source (newly sequenced or retrieved) are represented by colours, while specimen attribute (terra typica, type locality, type specimen or faunistic novelty) is represented by the shape. Sequences: ID = 1, cf. = 0; maximum p-distance: strict = NA, less strict = NA.

Haplotype network analysis of *Cardiocondyla nigra* was not possible.

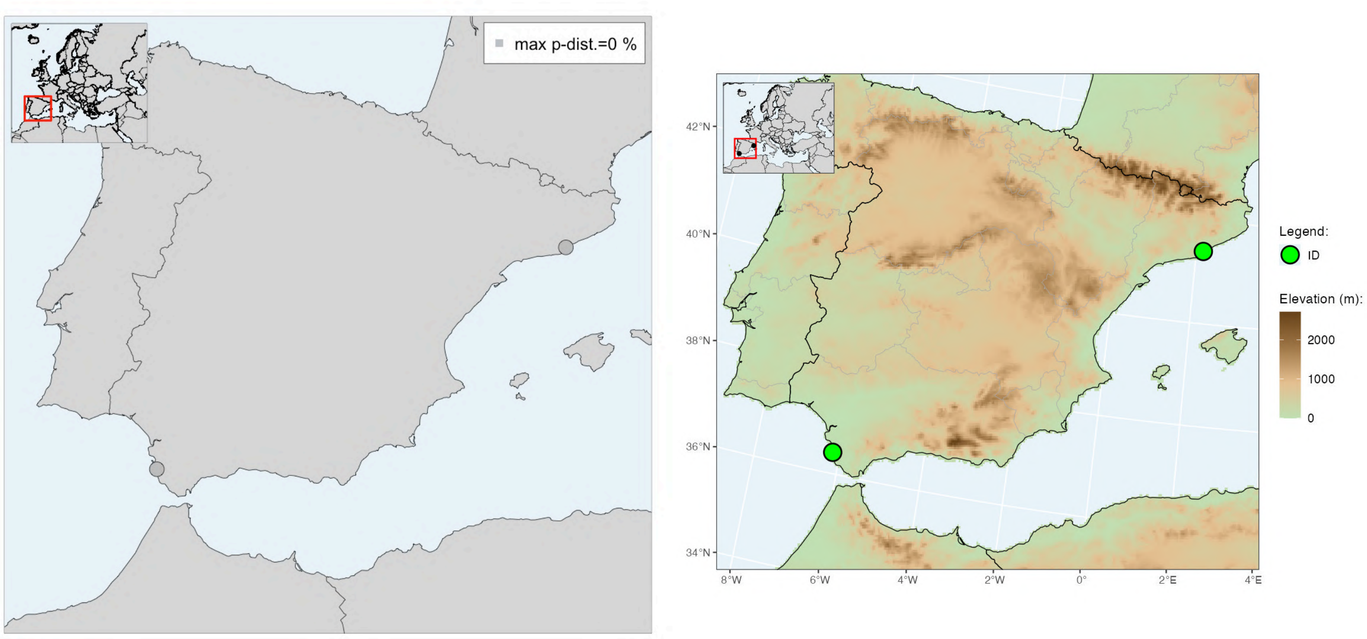

Figure 168: Genetic diversity map of *Cardiocondyla obscurior* Wheeler, W.M., 1929. PCoA projection was not done and therefore sequenced specimens in the genetic diversity map are coloured in gray (left). Specimen identification (ID or cf.) and source (newly sequenced or retrieved) are represented by colours, while specimen attribute (terra typica, type locality, type specimen or faunistic novelty) is represented by the shape (right). Sequences: ID = 2, cf. = 0; maximum p-distance: strict = NA, less strict = 0 %.

Haplotype network analysis of *Cardiocondyla obscurior* was not possible.

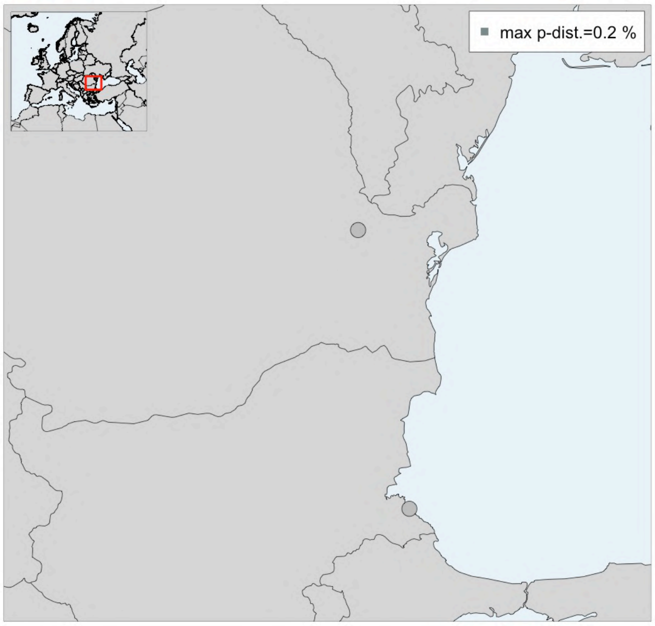

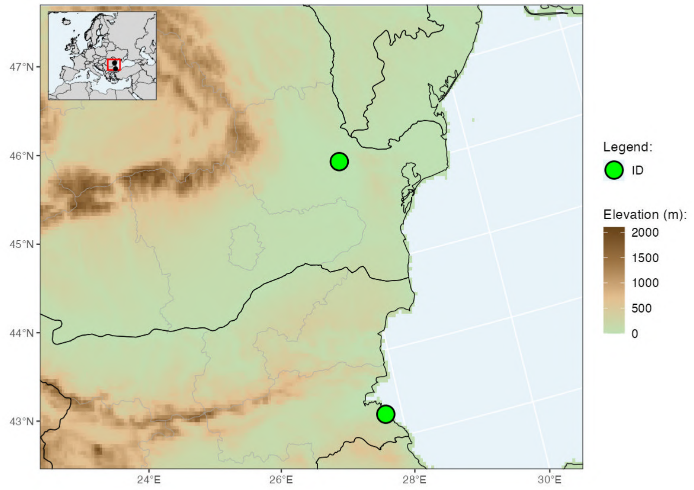

Figure 169: Genetic diversity map of *Cardiocondyla stambuloffii* Forel, 1913. PCoA projection was not done and therefore sequenced specimens in the genetic diversity map are coloured in gray (left). Specimen identification (ID or cf.) and source (newly sequenced or retrieved) are represented by colours, while specimen attribute (terra typica, type locality, type specimen or faunistic novelty) is represented by the shape (right). Sequences: ID = 2, cf. = 0; maximum p-distance: strict = NA, less strict = 0.2 %.

Haplotype network analysis of *Cardiocondyla stambuloffii* was not possible.

Carebara

Carebara oertzeni

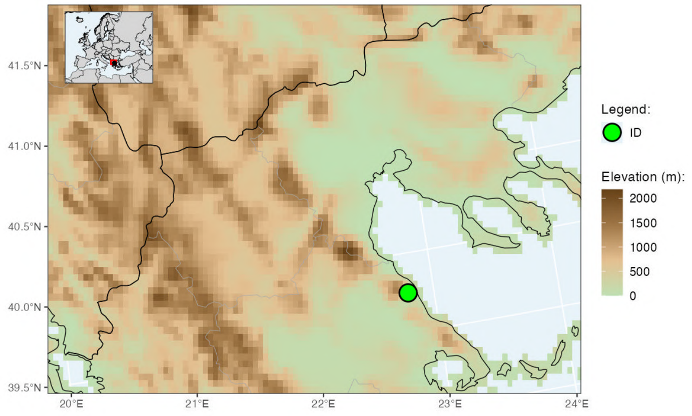

Figure 170: Map of *Carebara oertzeni* (Forel, 1886). Due to the presence of a single sequence, the genetic diversity map and the PCoA projection were not done. Specimen identification (ID or cf.) and source (newly sequenced or retrieved) are represented by colours, while specimen attribute (terra typica, type locality, type specimen or faunistic novelty) is represented by the shape. Sequences: ID = 1, cf. = 0; maximum p-distance: strict = NA, less strict = NA.

Haplotype network analysis of *Carebara oertzeni* was not possible.

Cataglyphis

Cataglyphis cretica

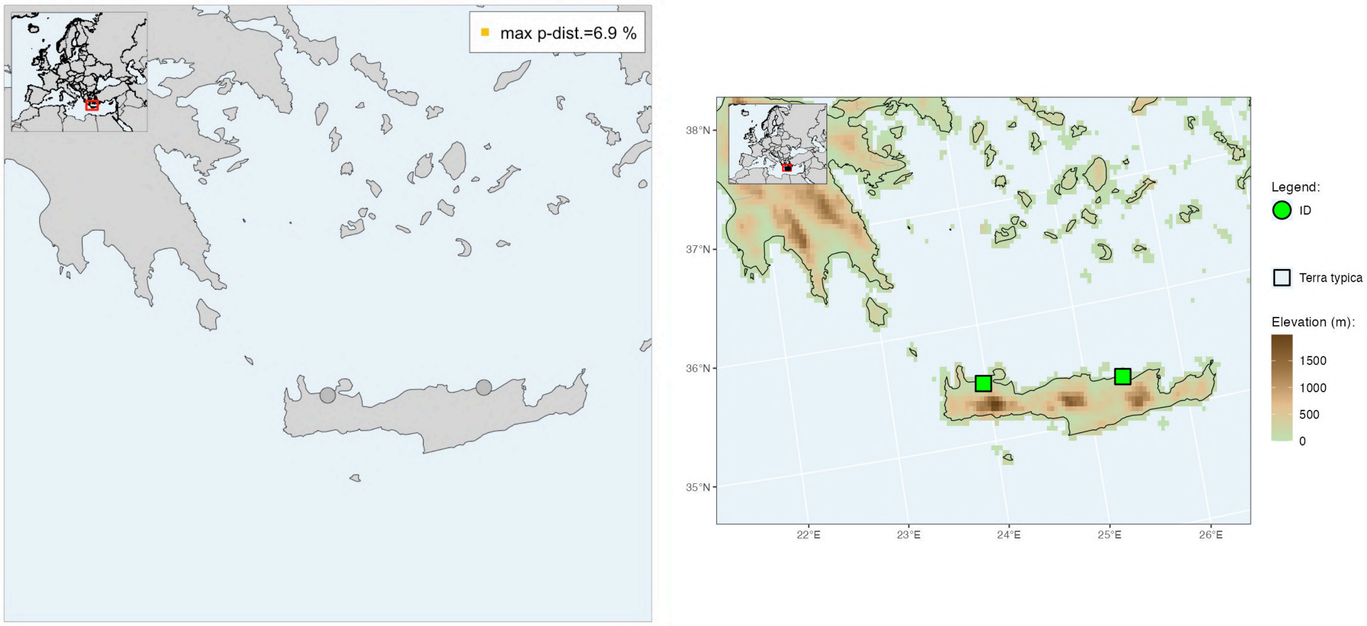

Figure 171: Genetic diversity map of *Cataglyphis cretica* (Forel, 1910). PCoA projection was not done and therefore sequenced specimens in the genetic diversity map are coloured in gray (left). Specimen identification (ID or cf.) and source (newly sequenced or retrieved) are represented by colours, while specimen attribute (terra typica, type locality, type specimen or faunistic novelty) is represented by the shape (right). Sequences: ID = 2, cf. = 0; maximum p-distance: strict = NA, less strict = 6.9 %.

Haplotype network analysis of *Cataglyphis cretica* was not possible.

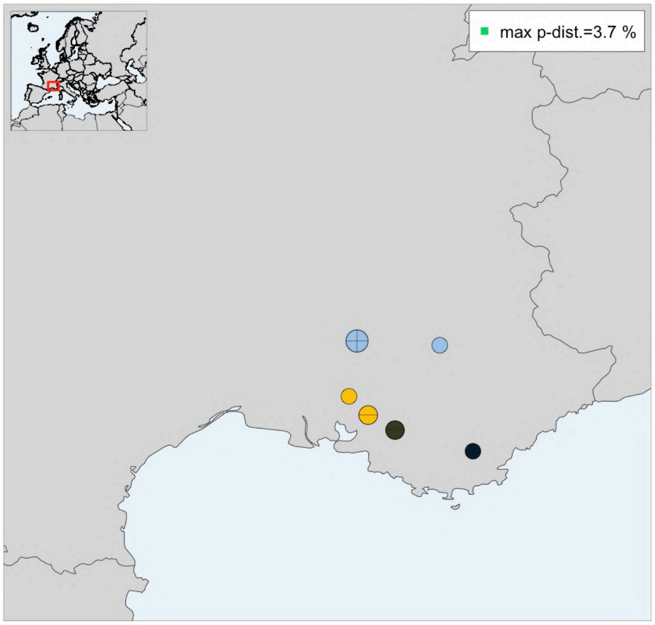

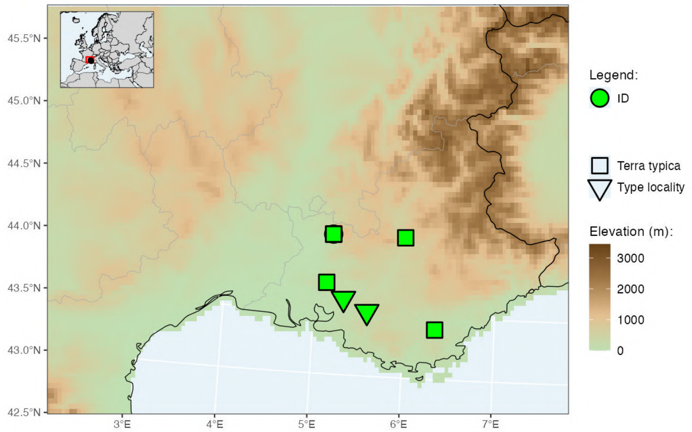

Figure 172: Genetic diversity map of *Cataglyphis cursor* (Fonscolombe, 1846). Nearby localities of sequenced specimens are merged in pies (left). Colours match the bidimensional colour space of the PCoA projection (Fig. 172 left) of p-dist between sequences (dots). Specimen identification (ID or cf.) and source (newly sequenced or retrieved) are represented by colours, while specimen attribute (terra typica, type locality, type specimen or faunistic novelty) is represented by the shape (right). Sequences: ID = 11, cf. = 0; maximum p-distance: strict = 3.7 %, less strict = 3.7 %.

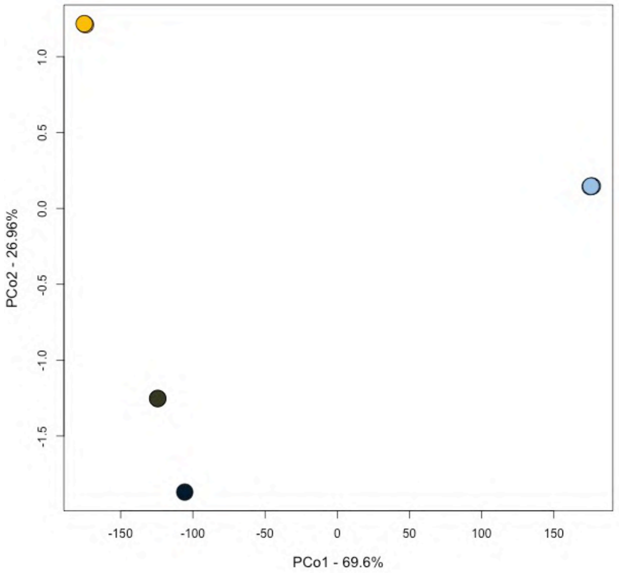

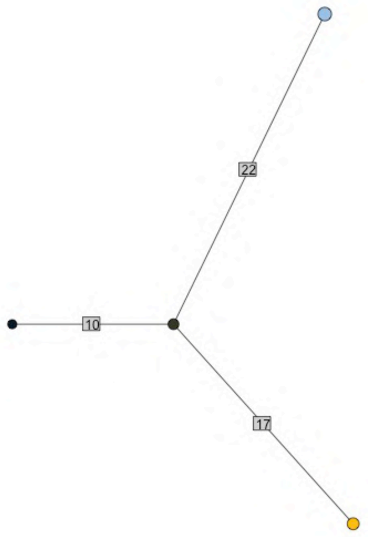

Figure 173: PCoA based on pairwise p-distances between *Cataglyphis cursor* sequences (left). Colours match a bidimensional colour space. Haplotype network of *Cataglyphis cursor* (right). Sequences > 599 bp: ID = 11, cf. = 0.

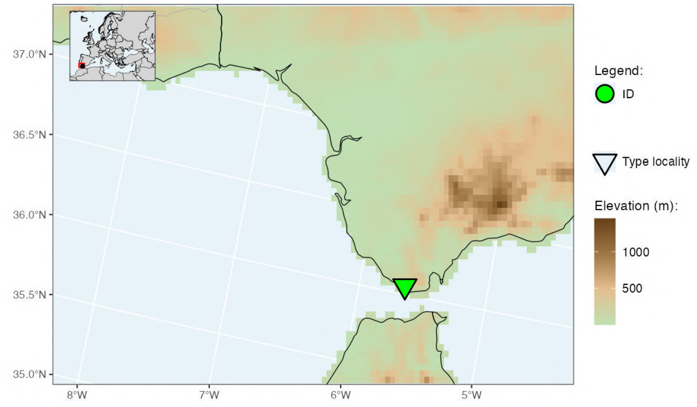

Figure 174: Map of *Cataglyphis douwesi* De Haro & Collingwood, 2000. Due to the presence of a single sequence, the genetic diversity map and the PCoA projection were not done. Specimen identification (ID or cf.) and source (newly sequenced or retrieved) are represented by colours, while specimen attribute (terra typica, type locality, type specimen or faunistic novelty) is represented by the shape. Sequences: ID = 1, cf. = 0; maximum p-distance: strict = NA, less strict = NA.

Haplotype network analysis of *Cataglyphis douwesi* was not possible.

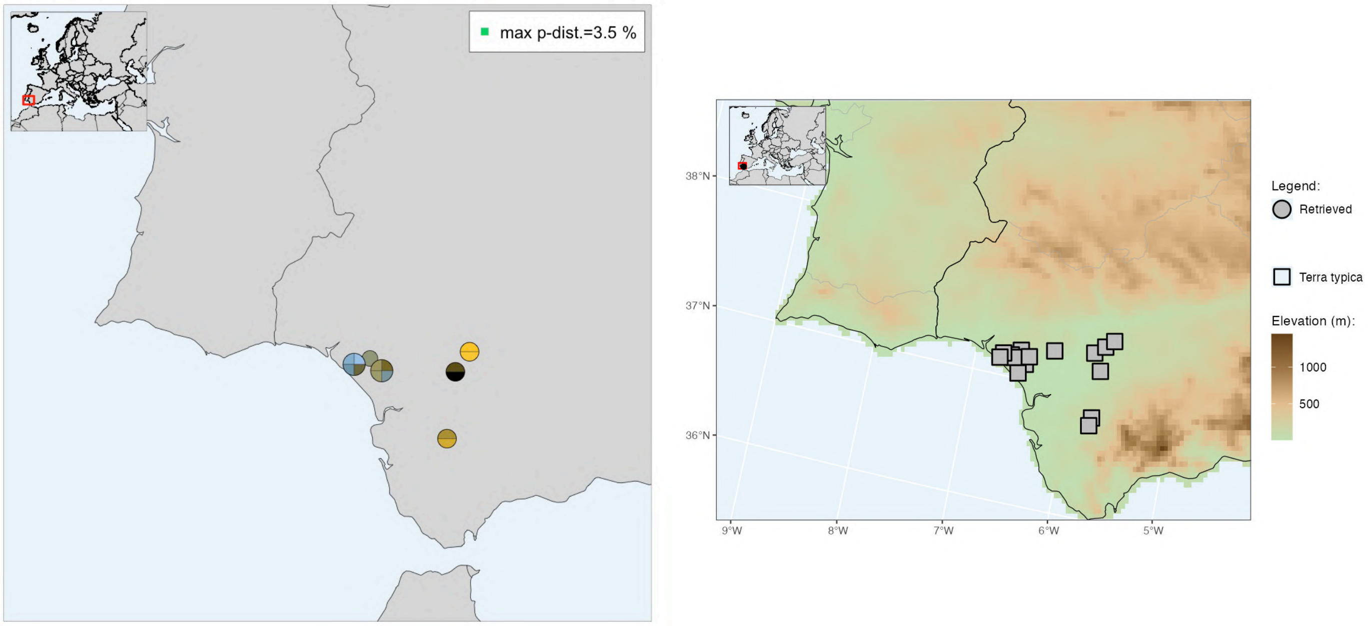

Figure 175: Genetic diversity map of *Cataglyphis floricola* Tinaut, 1993. Nearby localities of sequenced specimens are merged in pies (left). Colours match the bidimensional colour space of the PCoA projection (Fig. 175 left) of p-dist between sequences (dots). Specimen identification (ID or cf.) and source (newly sequenced or retrieved) are represented by colours, while specimen attribute (terra typica, type locality, type specimen or faunistic novelty) is represented by the shape (right). Sequences: ID = 15, cf. = 0; maximum p-distance: strict = 3.5 %, less strict = 3.5 %.

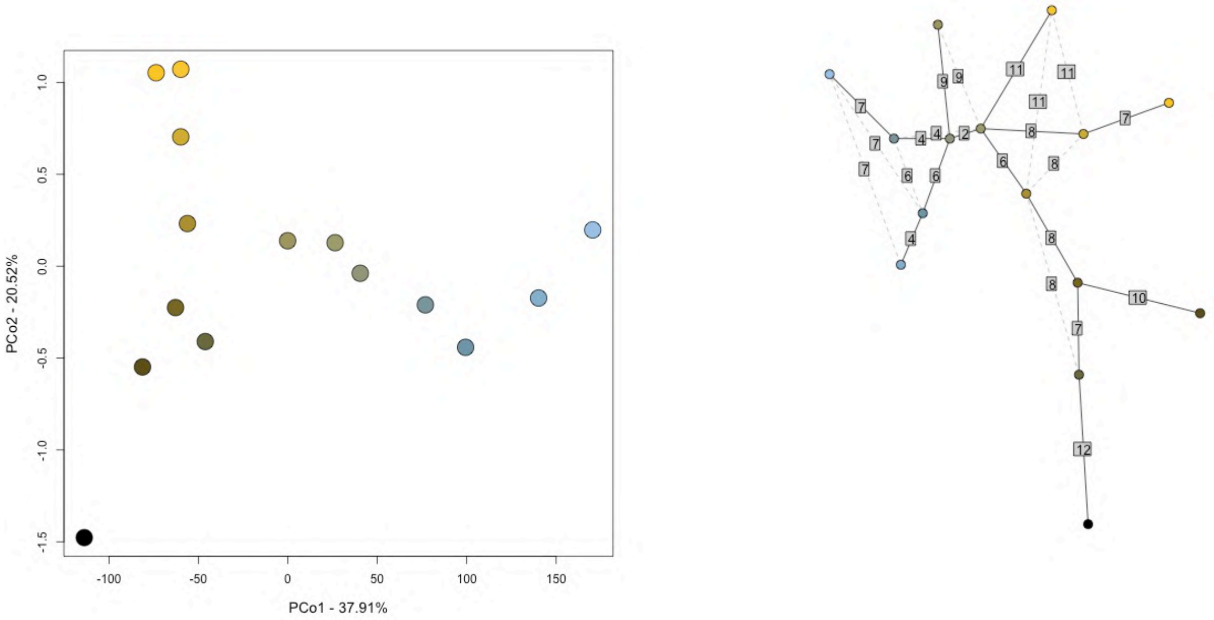

Figure 176: PCoA based on pairwise p-distances between *Cataglyphis floricola* sequences (left). Colours match a bidimensional colour space. Haplotype network of *Cataglyphis floricola* (right). Sequences > 599 bp: ID = 15, cf. = 0.

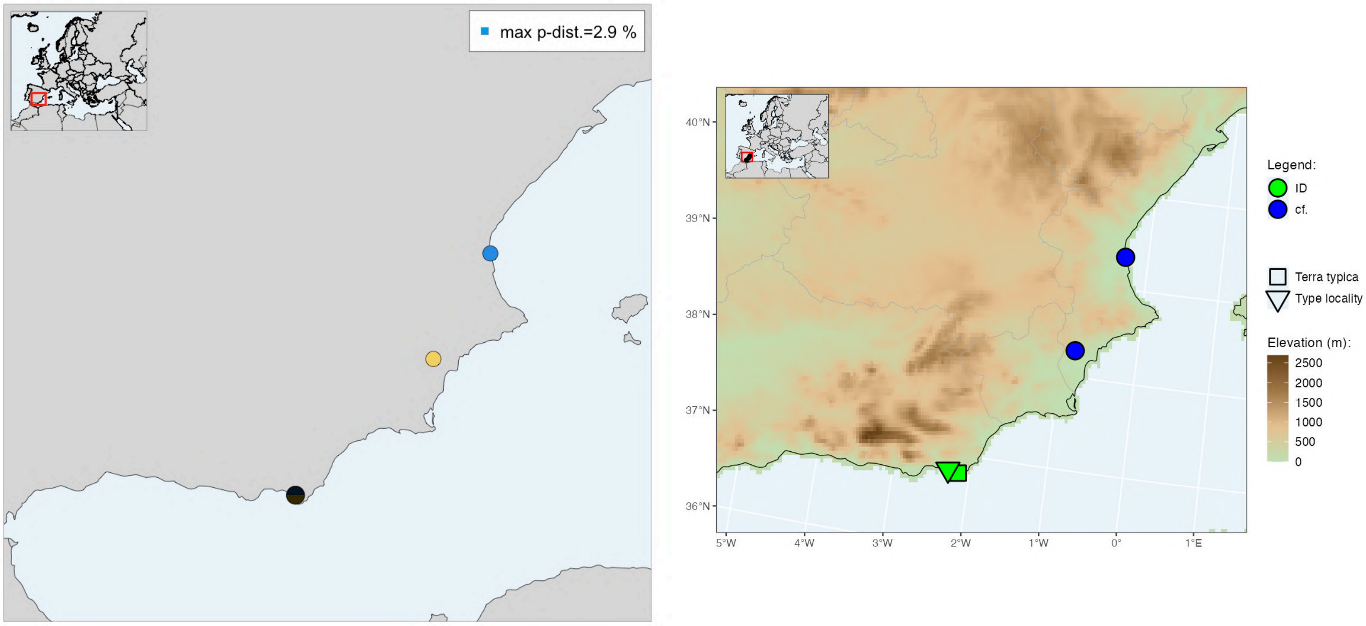

Figure 177: Genetic diversity map of *Cataglyphis gadeai* De Haro & Collingwood, 2003. Nearby localities of sequenced specimens are merged in pies (left). Colours match the bidimensional colour space of the PCoA projection (Fig. 177 left) of p-dist between sequences (dots). Specimen identification (ID or cf.) and source (newly sequenced or retrieved) are represented by colours, while specimen attribute (terra typica, type locality, type specimen or faunistic novelty) is represented by the shape (right). Sequences: ID = 2, cf. = 2; maximum p-distance: strict = NA, less strict = 2.9 %.

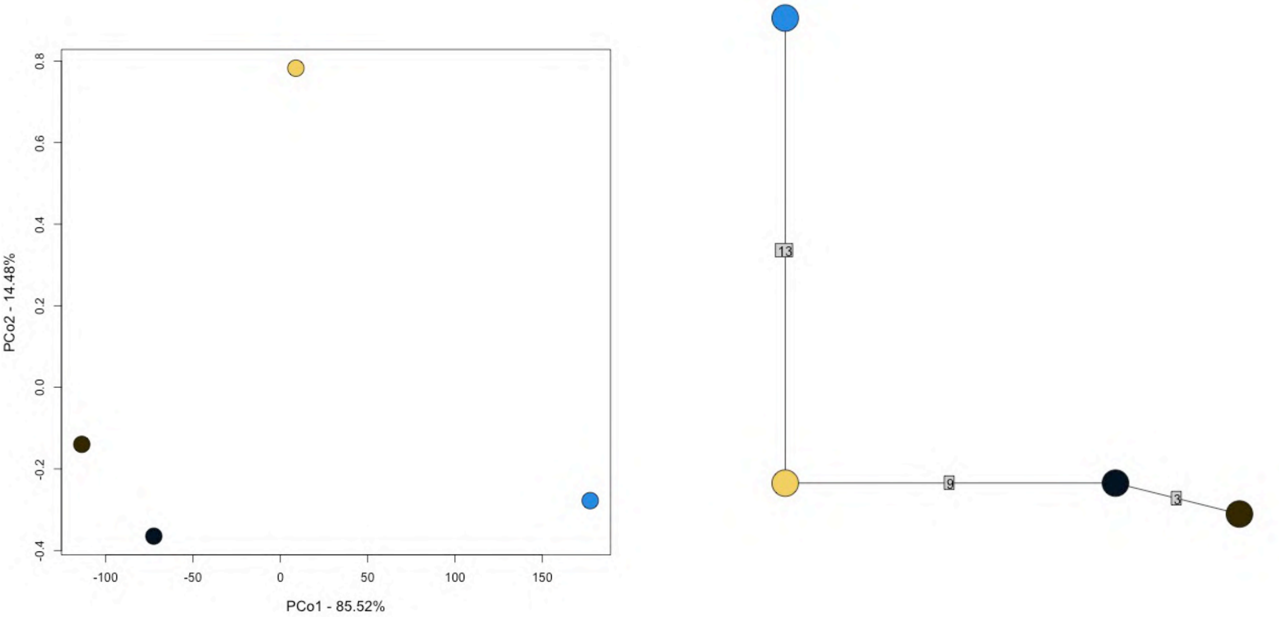

Figure 178: PCoA based on pairwise p-distances between *Cataglyphis gadeai* sequences (left). Colours match a bidimensional colour space. Haplotype network of *Cataglyphis gadeai* (right). Sequences > 599 bp: ID = 2, cf. = 2.

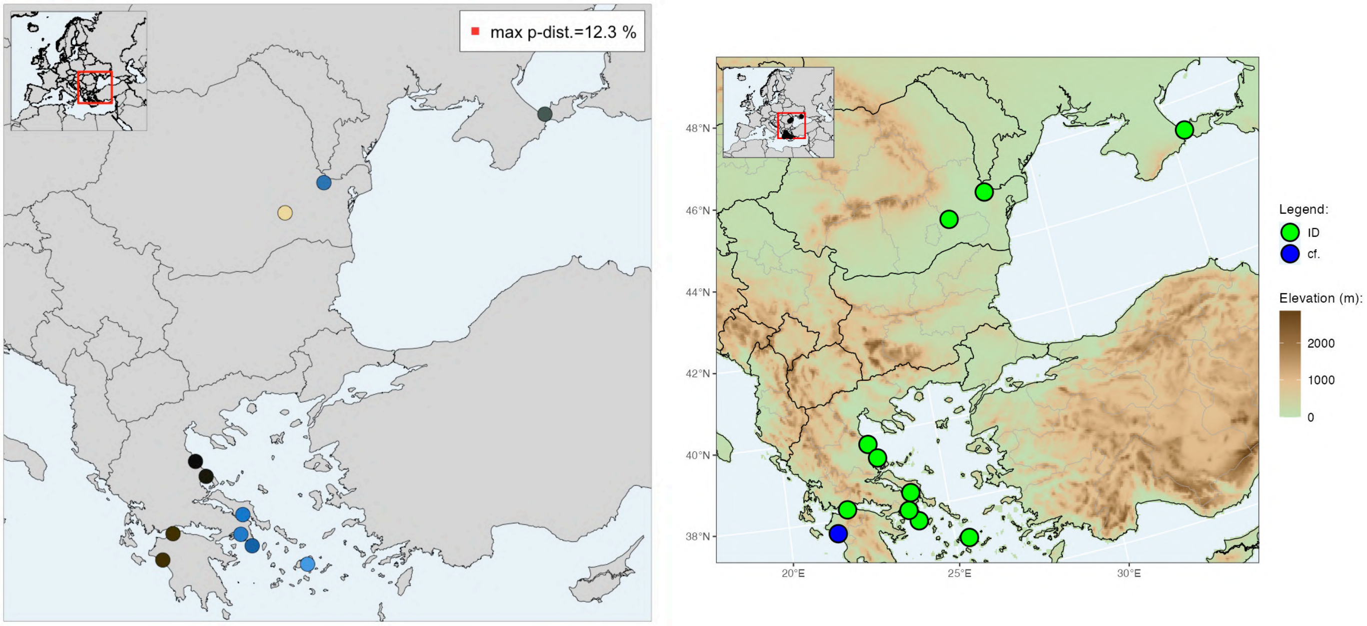

Figure 179: Genetic diversity map of *Cataglyphis hellenica* ForeL, 1886. Nearby localities of sequenced specimens are merged in pies (left). Colours match the bidimensional colour space of the PCoA projection (Fig. 179 left) of p-dist between sequences (dots). Specimen identification (ID or cf.) and source (newly sequenced or retrieved) are represented by colours, while specimen attribute (terra typica, type locality, type specimen or faunistic novelty) is represented by the shape (right). Sequences: ID = 10, cf. = 1; maximum p-distance: strict = 12.3 %, less strict = 12.3 %.

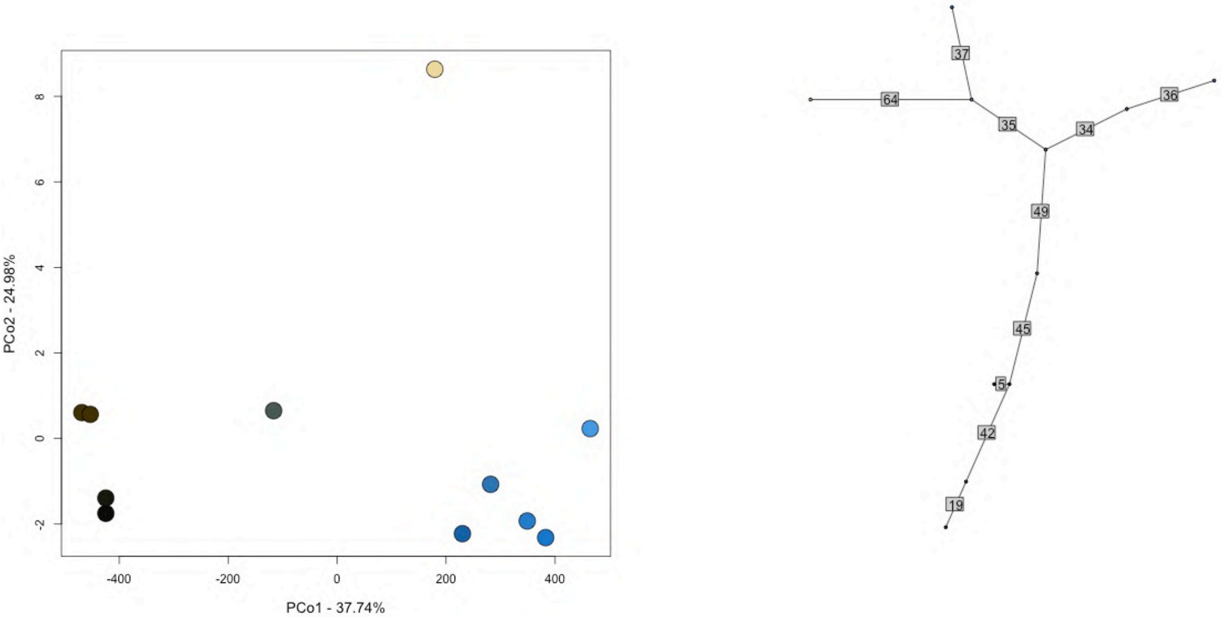

Figure 180: PCoA based on pairwise p-distances between *Cataglyphis hellenica* sequences (left). Colours match a bidimensional colour space. Haplotype network of *Cataglyphis hellenica* (right). Sequences > 599 bp: ID = 10, cf. = 1.

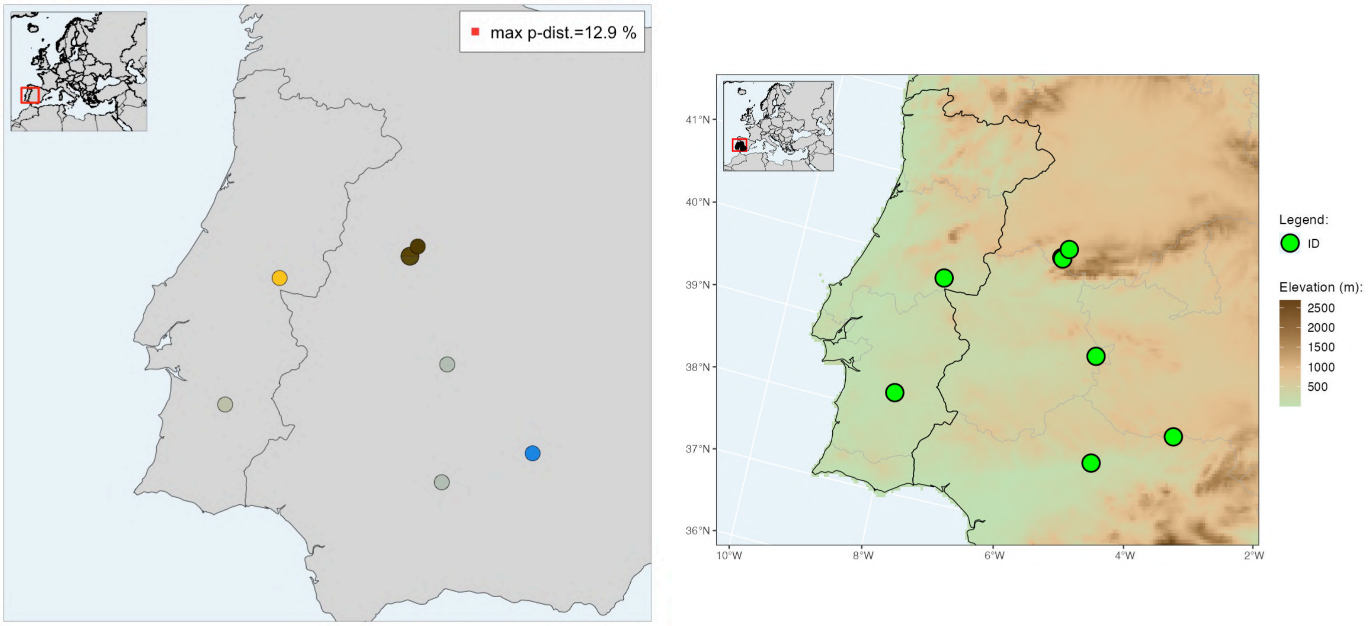

Figure 181: Genetic diversity map of *Cataglyphis hispanica* (Emery, 1906). Nearby localities of sequenced specimens are merged in pies (left). Colours match the bidimensional colour space of the PCoA projection (Fig. 181 left) of p-dist between sequences (dots). Specimen identification (ID or cf.) and source (newly sequenced or retrieved) are represented by colours, while specimen attribute (terra typica, type locality, type specimen or faunistic novelty) is represented by the shape (right). Sequences: ID = 8, cf. = 0; maximum p-distance: strict = 10.5 %, less strict = 12.9 %.

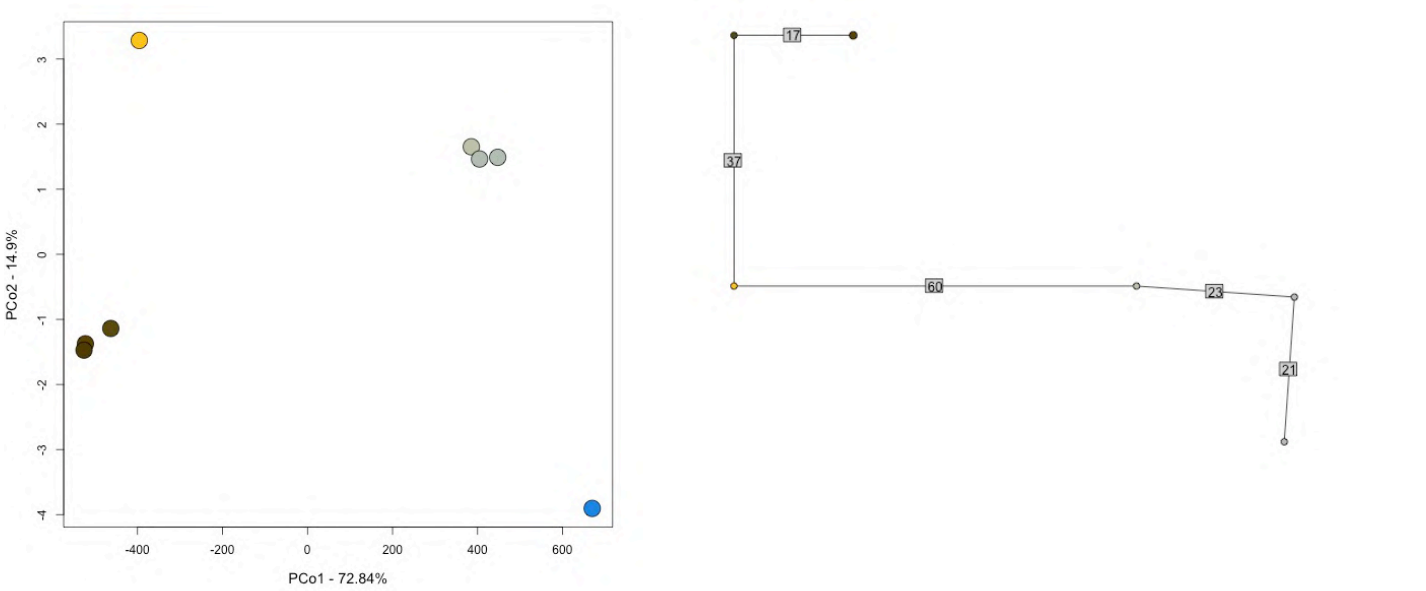

Figure 182: PCoA based on pairwise p-distances between *Cataglyphis hispanica* sequences (left). Colours match a bidimensional colour space. Haplotype network of *Cataglyphis hispanica* (right). Sequences > 599 bp: ID = 7, cf. = 0.

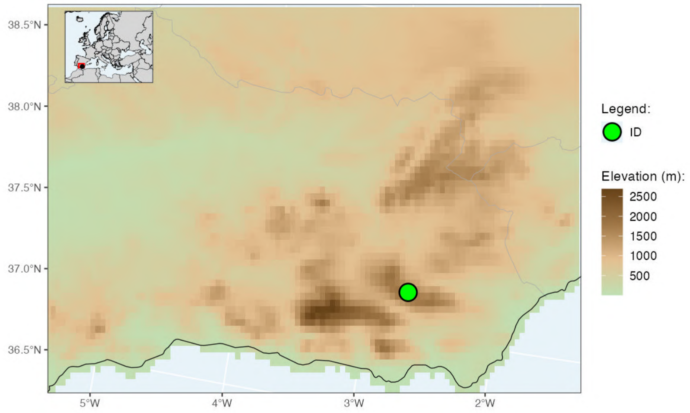

Figure 183: Map of *Cataglyphis humeya* Tinaut, 1991. Due to the presence of a single sequence, the genetic diversity map and the PCoA projection were not done. Specimen identification (ID or cf.) and source (newly sequenced or retrieved) are represented by colours, while specimen attribute (terra typica, type locality, type specimen or faunistic novelty) is represented by the shape. Sequences: ID = 1, cf. = 0; maximum p-distance: strict = NA, less strict = NA.

Haplotype network analysis of *Cataglyphis humeya* was not possible.

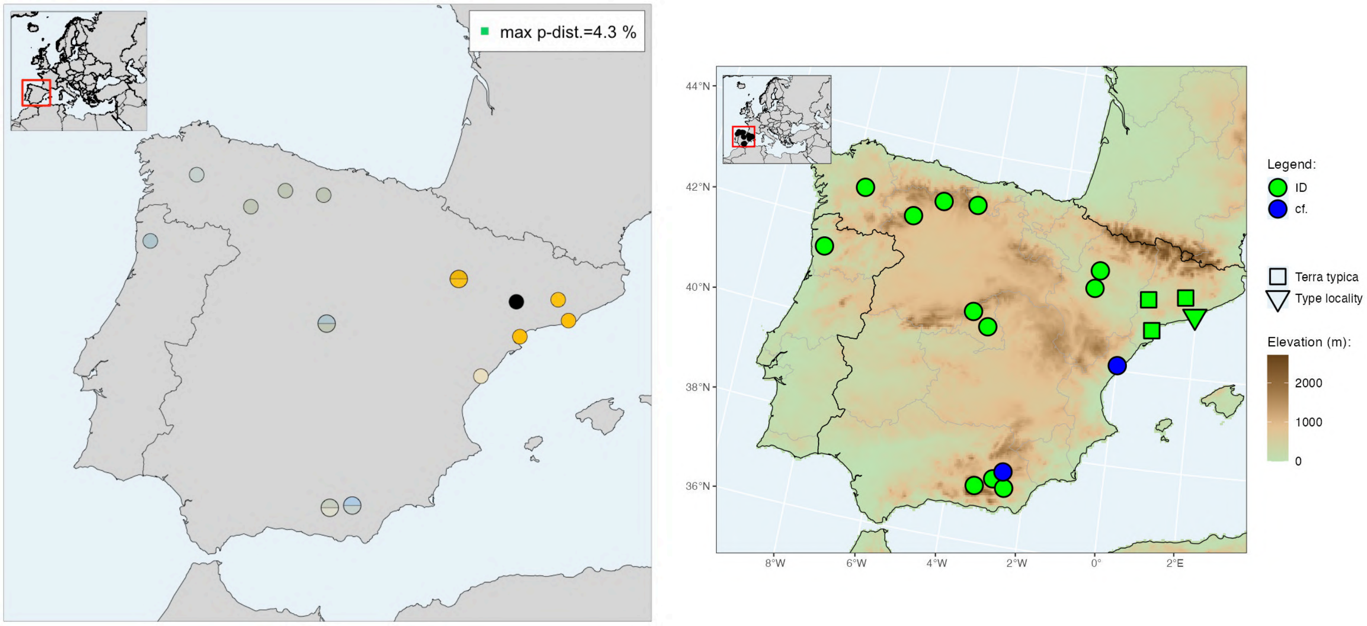

Figure 184: Genetic diversity map of *Cataglyphis iberica* (Emery, 1906). Nearby localities of sequenced specimens are merged in pies (left). Colours match the bidimensional colour space of the PCoA projection (Fig. 184 left) of p-dist between sequences (dots). Specimen identification (ID or cf.) and source (newly sequenced or retrieved) are represented by colours, while specimen attribute (terra typica, type locality, type specimen or faunistic novelty) is represented by the shape (right). Sequences: ID = 17, cf. = 2; maximum p-distance: strict = 4.3 %, less strict = 4.3 %.

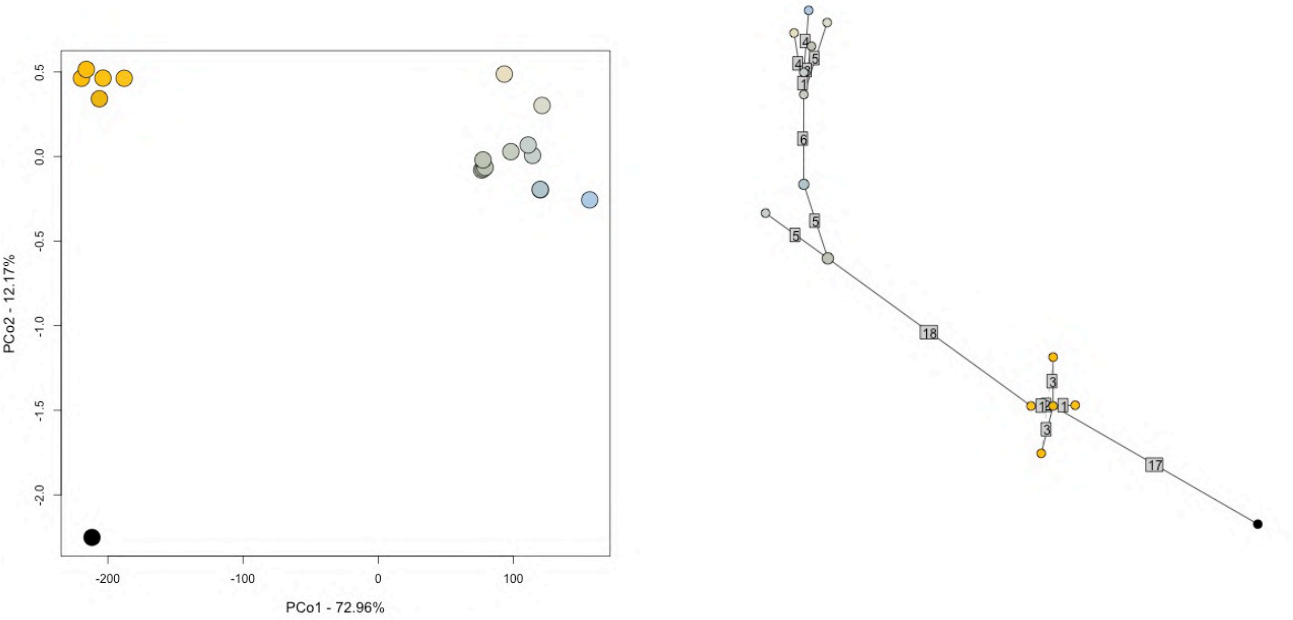

Figure 185: PCoA based on pairwise p-distances between *Cataglyphis iberica* sequences (left). Colours match a bidimensional colour space. Haplotype network of *Cataglyphis iberica* (right). Sequences > 599 bp: ID = 17, cf. = 2.

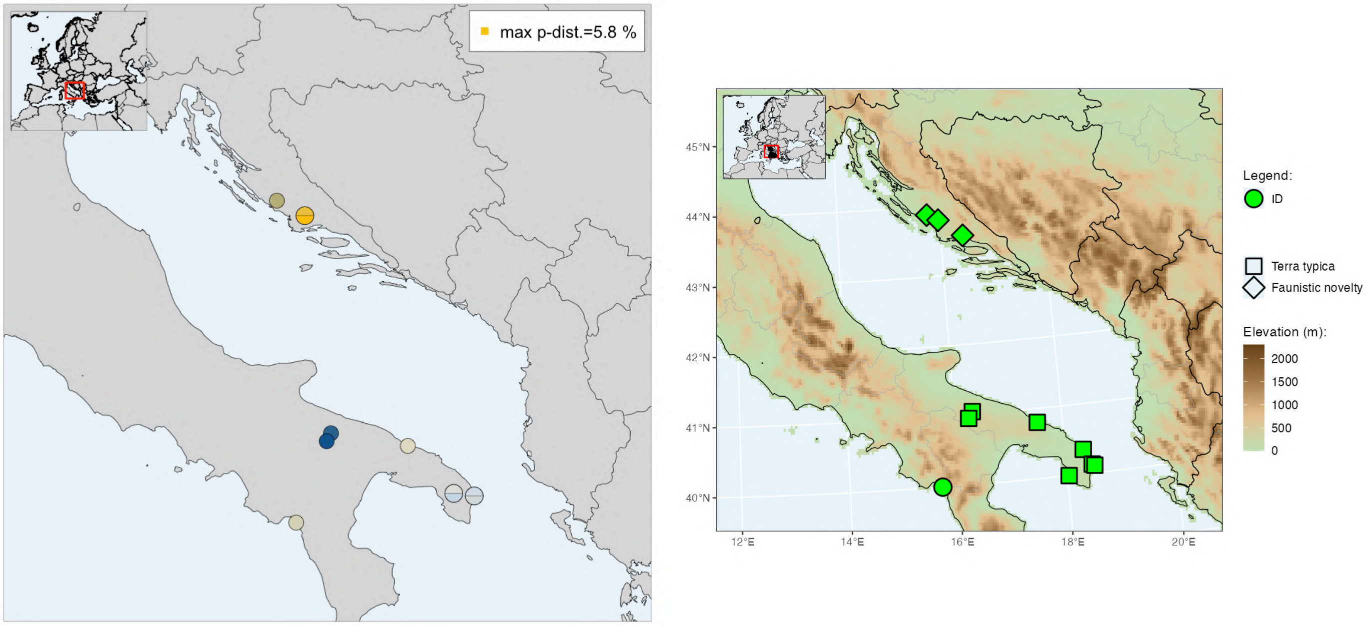

Figure 186: Genetic diversity map of *Cataglyphis italica* (Emery, 1906). Nearby localities of sequenced specimens are merged in pies (left). Colours match the bidimensional colour space of the PCoA projection (Fig. 186 left) of p-dist between sequences (dots). Specimen identification (ID or cf.) and source (newly sequenced or retrieved) are represented by colours, while specimen attribute (terra typica, type locality, type specimen or faunistic novelty) is represented by the shape (right). Sequences: ID = 11, cf. = 0; maximum p-distance: strict = 5.8 %, less strict = 5.8 %.

The species is reported for the first time in Croatia.

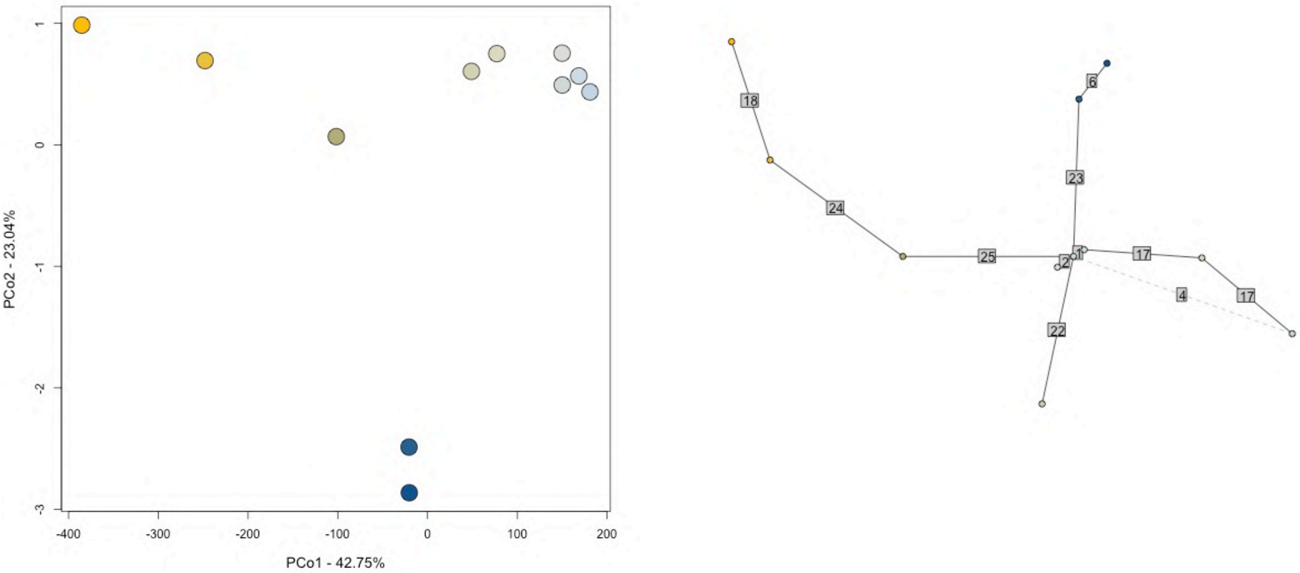

Figure 187: PCoA based on pairwise p-distances between *Cataglyphis italica* sequences (left). Colours match a bidimensional colour space. Haplotype network of *Cataglyphis italica* (right). Sequences > 599 bp: ID = 11, cf. = 0.

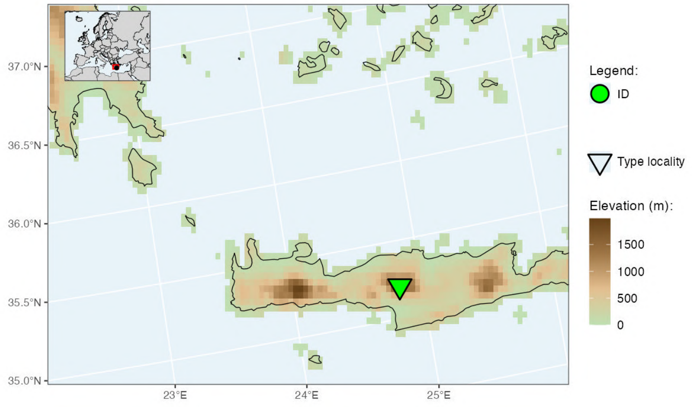

Figure 188: Map of *Cataglyphis minos* Borowiec & Salata, 2022. Due to the presence of a single sequence, the genetic diversity map and the PCoA projection were not done. Specimen identification (ID or cf.) and source (newly sequenced or retrieved) are represented by colours, while specimen attribute (terra typica, type locality, type specimen or faunistic novelty) is represented by the shape. Sequences: ID = 1, cf. = 0; maximum p-distance: strict = NA, less strict = NA.

Haplotype network analysis of *Cataglyphis minos* was not possible.

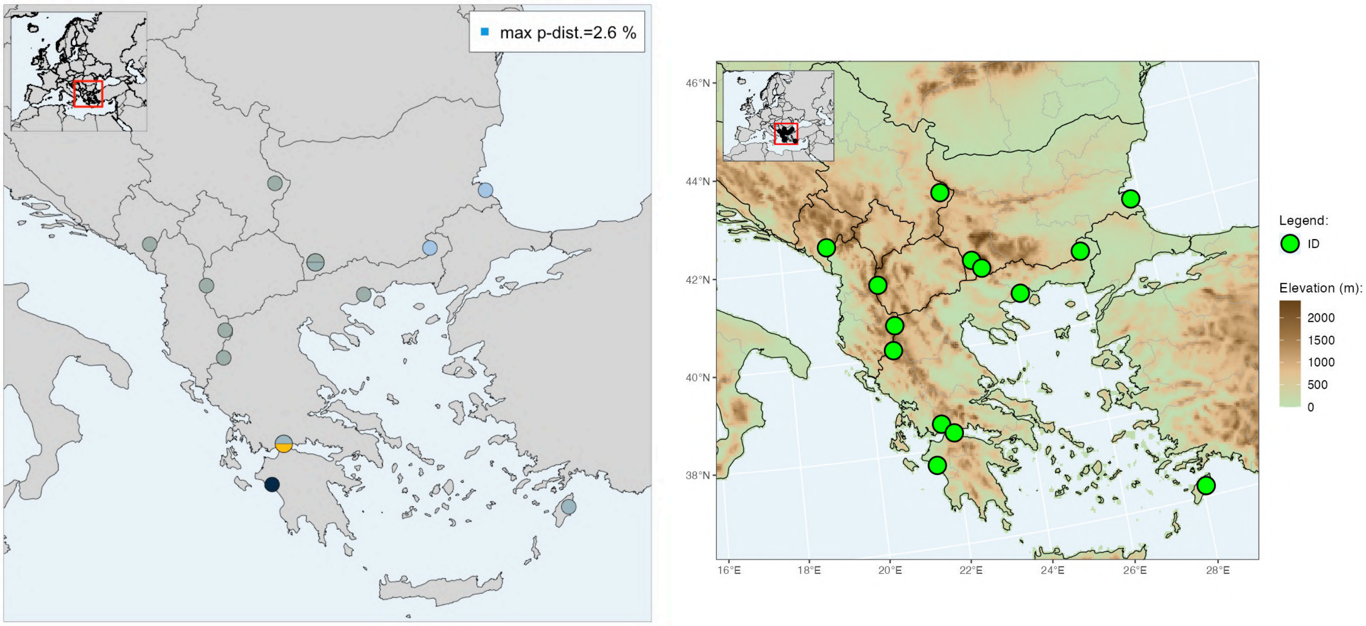

Figure 189: Genetic diversity map of *Cataglyphis nodus* (Brullé, 1833). Nearby localities of sequenced specimens are merged in pies (left). Colours match the bidimensional colour space of the PCoA projection (Fig. 189 left) of p-dist between sequences (dots). Specimen identification (ID or cf.) and source (newly sequenced or retrieved) are represented by colours, while specimen attribute (terra typica, type locality, type specimen or faunistic novelty) is represented by the shape (right). Sequences: ID = 14, cf. = 0; maximum p-distance: strict = 2.6 %, less strict = 2.6 %.

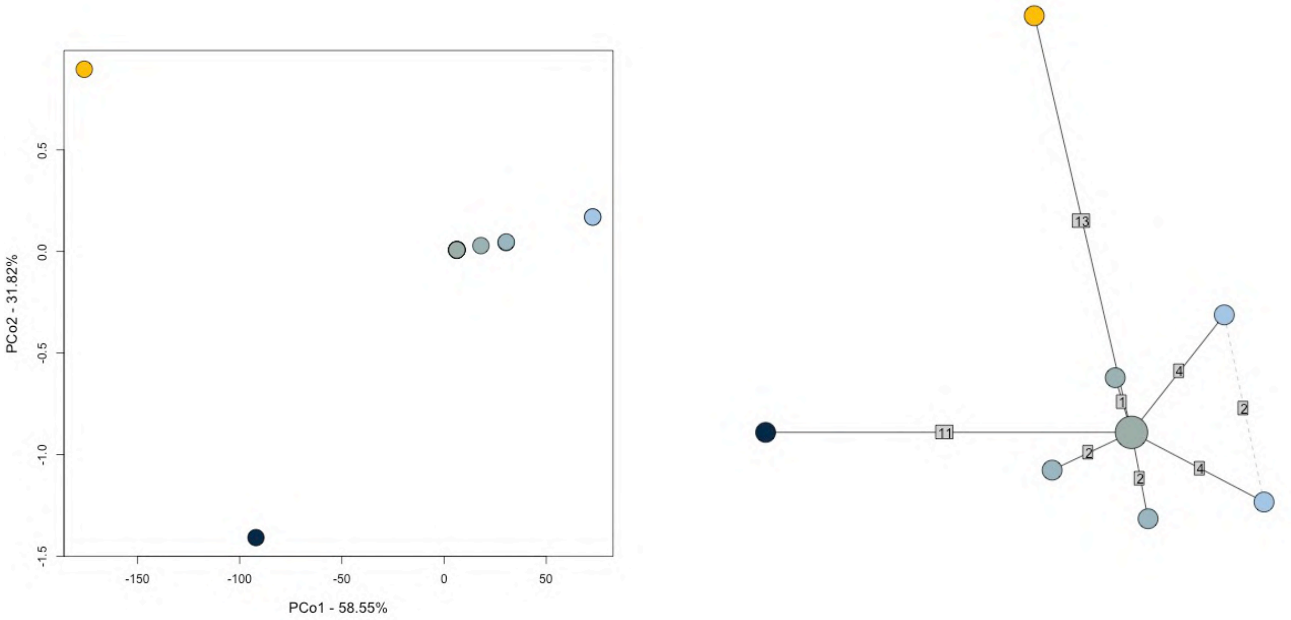

Figure 190: PCoA based on pairwise p-distances between *Cataglyphis nodus* sequences (left). Colours match a bidimensional colour space. Haplotype network of *Cataglyphis nodus* (right). Sequences > 599 bp: ID = 14, cf. = 0.

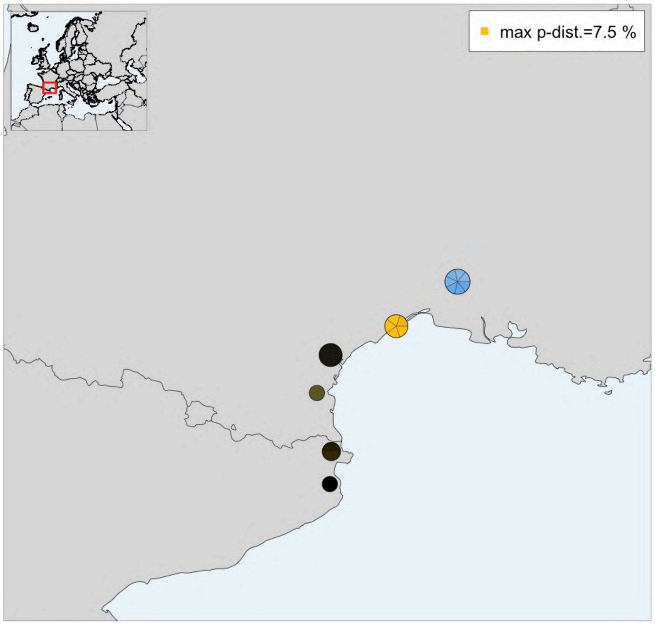

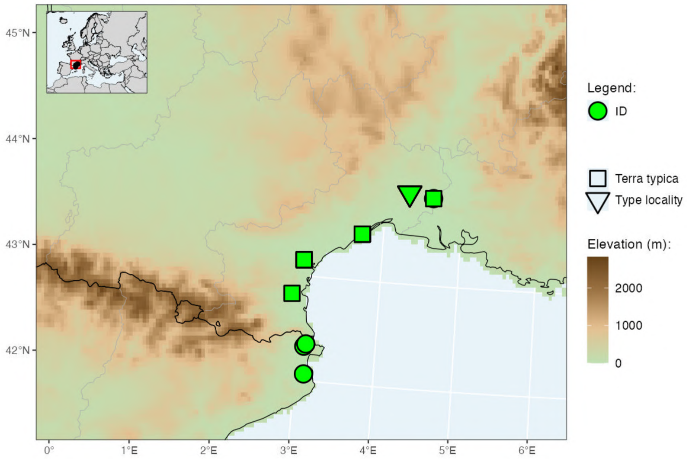

Figure 191: Genetic diversity map of *Cataglyphis piliscapa* (Forel, 1901). Nearby localities of sequenced specimens are merged in pies (left). Colours match the bidimensional colour space of the PCoA projection (Fig. 191 left) of p-dist between sequences (dots). Specimen identification (ID or cf.) and source (newly sequenced or retrieved) are represented by colours, while specimen attribute (terra typica, type locality, type specimen or faunistic novelty) is represented by the shape (right). Sequences: ID = 21, cf. = 0; maximum p-distance: strict = 7.5 %, less strict = 7.5 %.

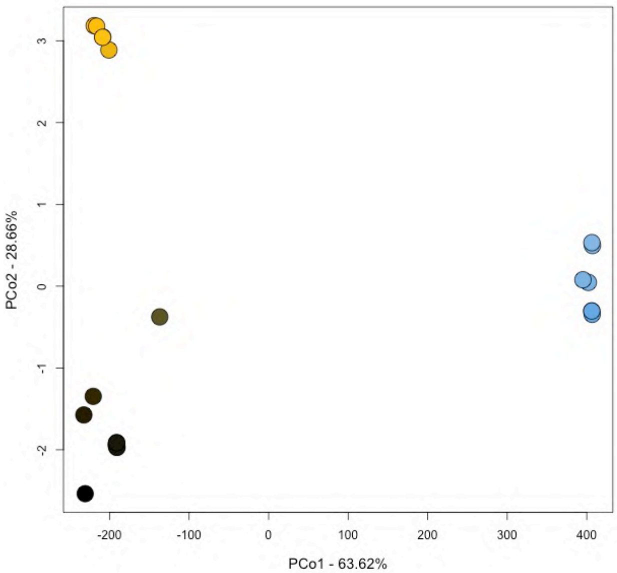

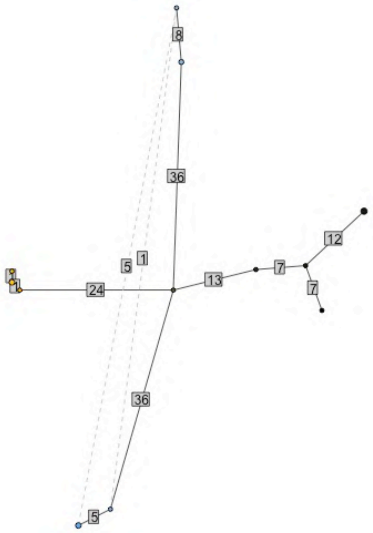

Figure 192: PCoA based on pairwise p-distances between *Cataglyphis piliscapa* sequences (left). Colours match a bidimensional colour space. Haplotype network of *Cataglyphis piliscapa* (right). Sequences > 599 bp: ID = 21, cf. = 0.

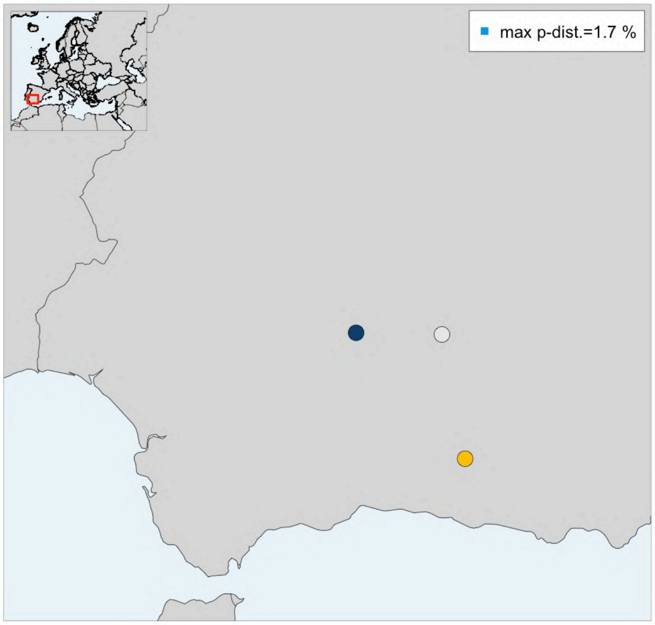

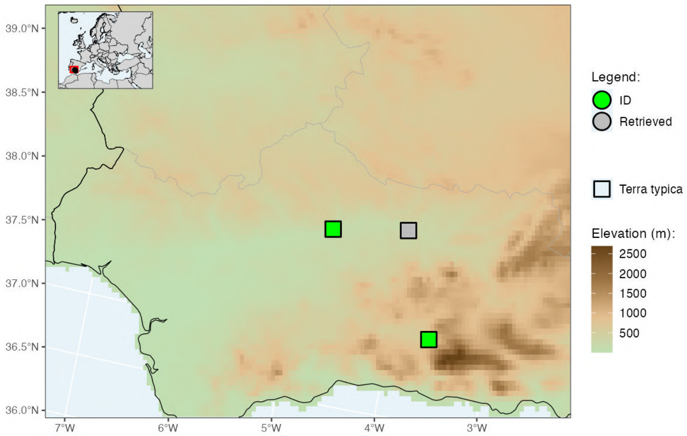

Figure 193: Genetic diversity map of *Cataglyphis rosenhaueri* Santschi, 1925. Nearby localities of sequenced specimens are merged in pies (left). Colours match the bidimensional colour space of the PCoA projection (Fig. 193 left) of p-dist between sequences (dots). Specimen identification (ID or cf.) and source (newly sequenced or retrieved) are represented by colours, while specimen attribute (terra typica, type locality, type specimen or faunistic novelty) is represented by the shape (right). Sequences: ID = 3, cf. = 0; maximum p-distance: strict = NA, less strict = 1.7 %.

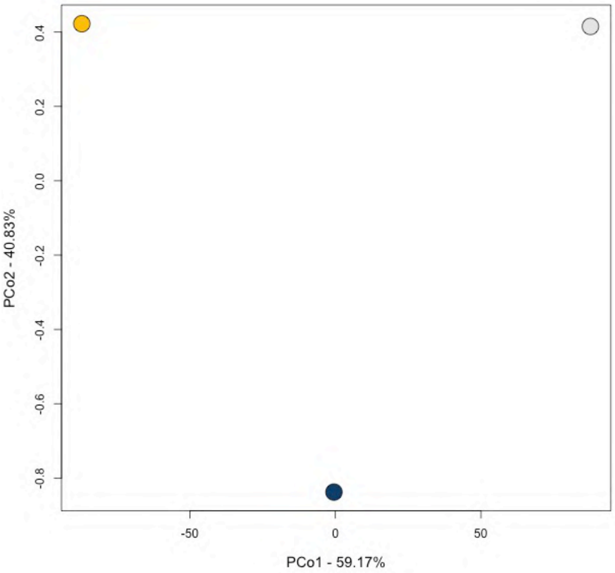

Figure 194: PCoA based on pairwise p-distances between *Cataglyphis rosenhaueri* sequences (left). Colours match a bidimensional colour space. Haplotype network analysis of *Cataglyphis rosenhaueri* was not possible.

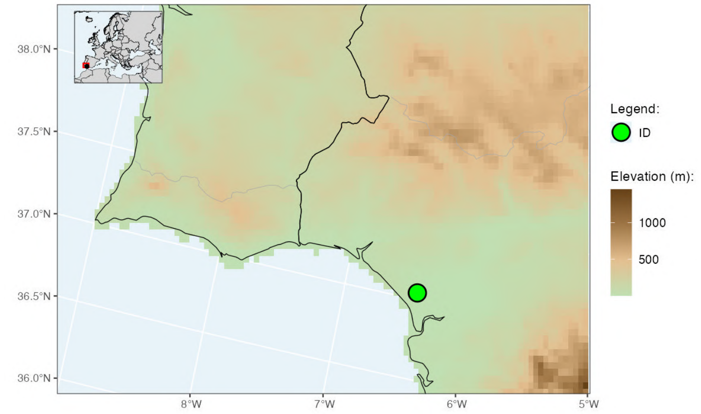

Figure 195: Map of *Cataglyphis tartessica* Amor & Ortega, 2014. Due to the presence of a single sequence, the genetic diversity map and the PCoA projection were not done. Specimen identification (ID or cf.) and source (newly sequenced or retrieved) are represented by colours, while specimen attribute (terra typica, type locality, type specimen or faunistic novelty) is represented by the shape. Sequences: ID = 1, cf. = 0; maximum p-distance: strict = NA, less strict = NA.

Haplotype network analysis of *Cataglyphis tartessica* was not possible.

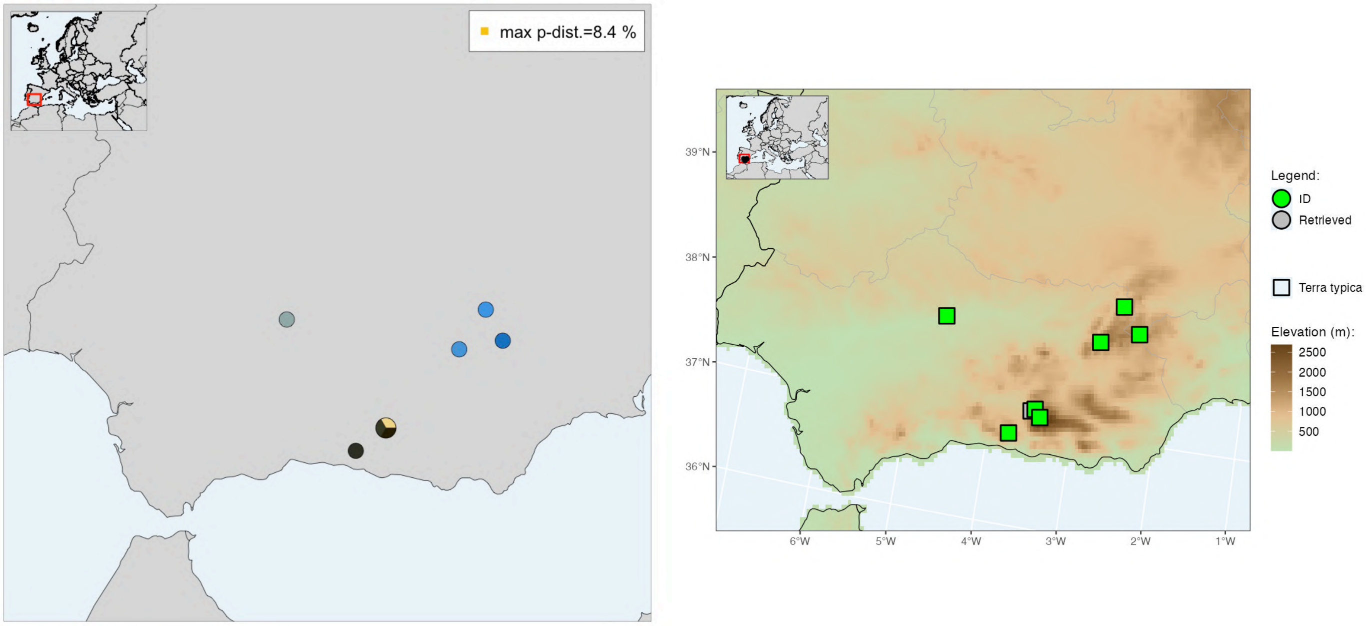

Figure 196: Genetic diversity map of *Cataglyphis velox* Santschi, 1929. Nearby localities of sequenced specimens are merged in pies (left). Colours match the bidimensional colour space of the PCoA projection (Fig. 196 left) of p-dist between sequences (dots). Specimen identification (ID or cf.) and source (newly sequenced or retrieved) are represented by colours, while specimen attribute (terra typica, type locality, type specimen or faunistic novelty) is represented by the shape (right). Sequences: ID = 8, cf. = 0; maximum p-distance: strict = 8.4 %, less strict = 8.4 %.

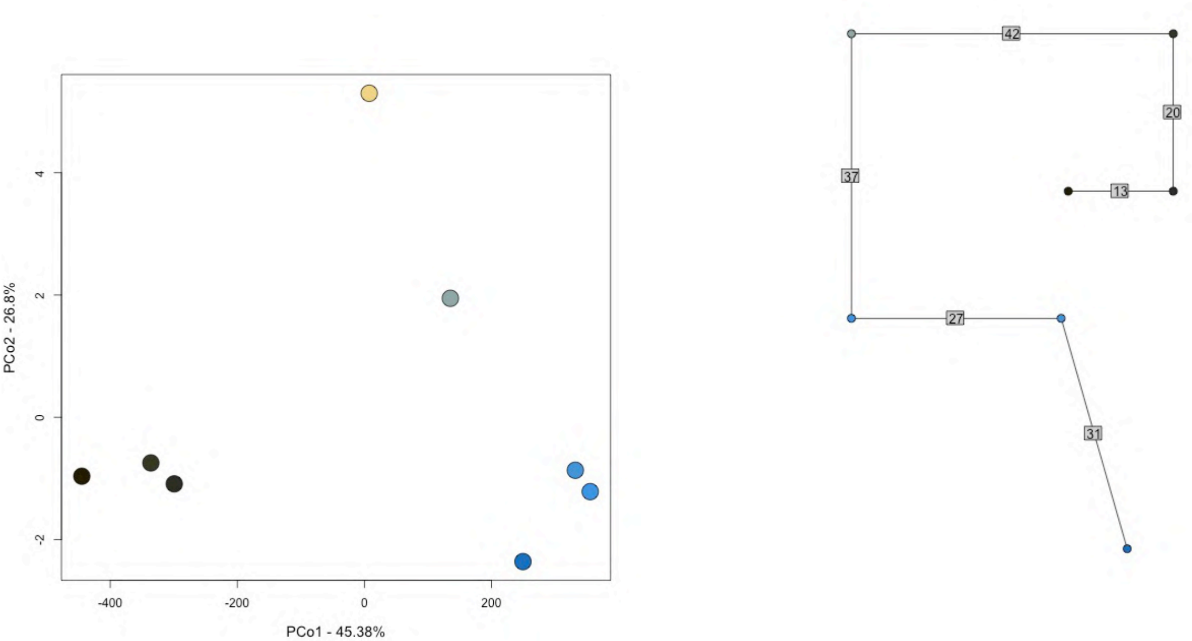

Figure 197: PCoA based on pairwise p-distances between *Cataglyphis velox* sequences (left). Colours match a bidimensional colour space. Haplotype network of *Cataglyphis velox* (right). Sequences > 599 bp: ID = 7, cf. = 0.

Cataglyphis viatica

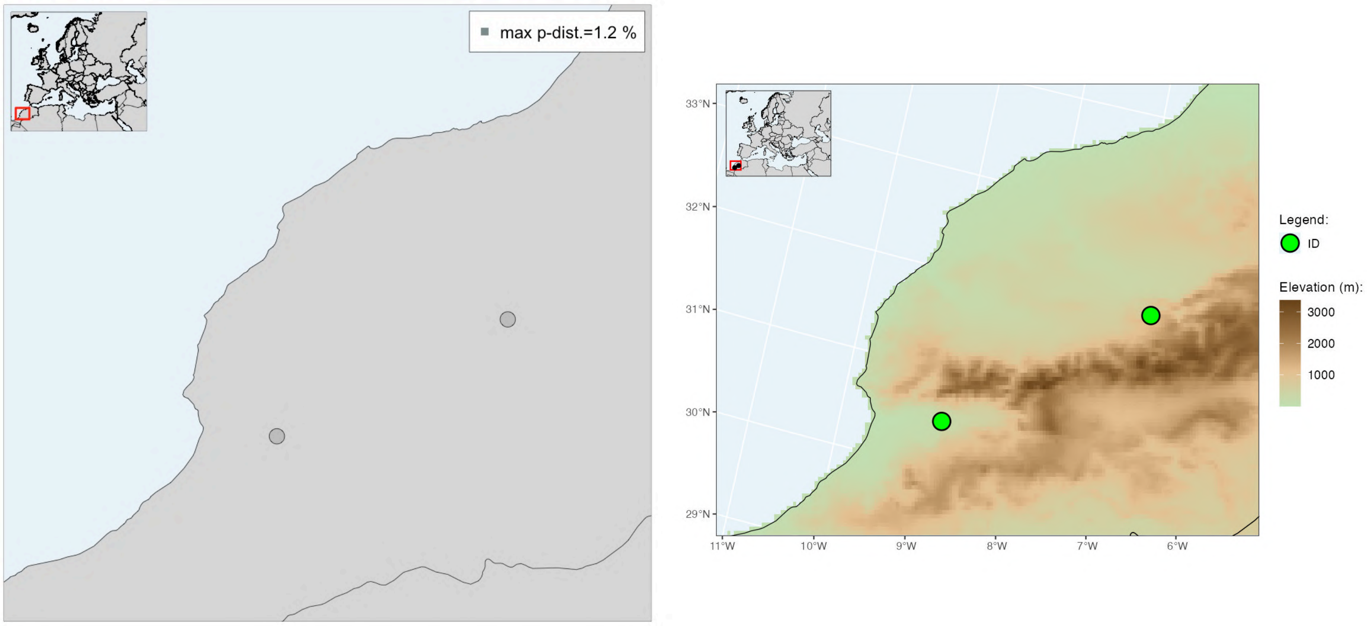

Figure 198: Genetic diversity map of *Cataglyphis viatica* (Fabricius, 1787). PCoA projection was not done and therefore sequenced specimens in the genetic diversity map are coloured in gray (left). Specimen identification (ID or cf.) and source (newly sequenced or retrieved) are represented by colours, while specimen attribute (terra typica, type locality, type specimen or faunistic novelty) is represented by the shape (right). Sequences: ID = 2, cf. = 0; maximum p-distance: strict = NA, less strict = 1.2 %.

Haplotype network analysis of *Cataglyphis viatica* was not possible.

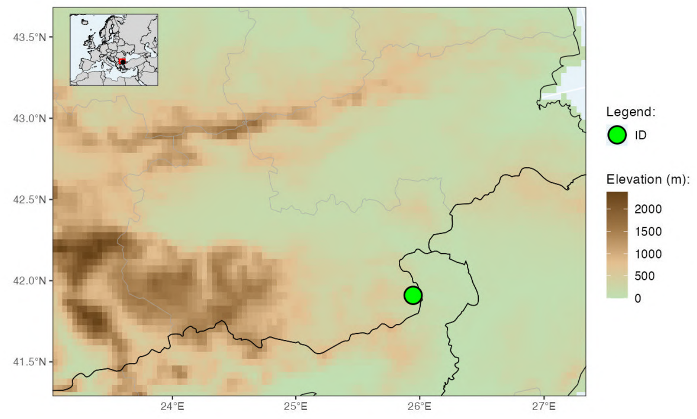

Figure 199: Map of *Cataglyphis viaticoides* (André, 1881). Due to the presence of a single sequence, the genetic diversity map and the PCoA projection were not done. Specimen identification (ID or cf.) and source (newly sequenced or retrieved) are represented by colours, while specimen attribute (terra typica, type locality, type specimen or faunistic novelty) is represented by the shape. Sequences: ID = 1, cf. = 0; maximum p-distance: strict = NA, less strict = NA.

Haplotype network analysis of *Cataglyphis viaticoides* was not possible.

Colobopsis

Colobopsis imitans

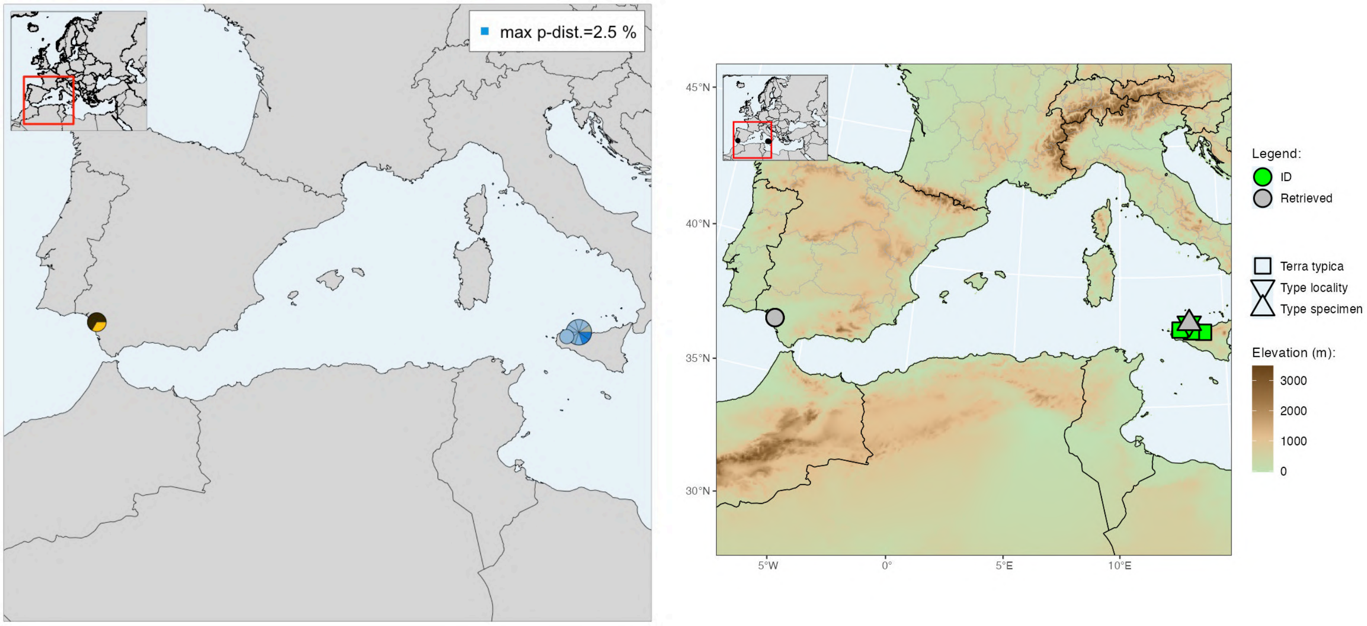

Figure 200: Genetic diversity map of *Colobopsis imitans* Schifani, Giannetti, Csősz, Castellucci, Luchetti, Castracani, Spotti, Mori & Grasso, 2021. Nearby localities of sequenced specimens are merged in pies (left). Colours match the bidimensional colour space of the PCoA projection (Fig. 200 left) of p-dist between sequences (dots). Specimen identification (ID or cf.) and source (newly sequenced or retrieved) are represented by colours, while specimen attribute (terra typica, type locality, type specimen or faunistic novelty) is represented by the shape (right). Sequences: ID = 14, cf. = 0; maximum p-distance: strict = 2.5 %, less strict = 2.5 %.

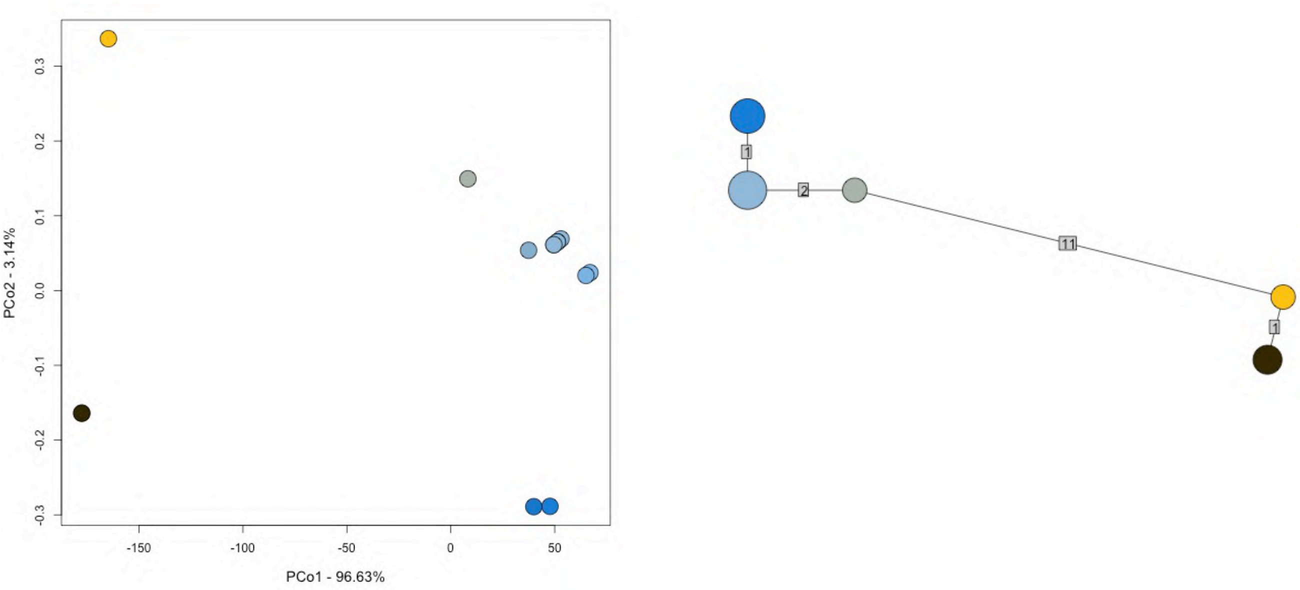

Figure 201: PCoA based on pairwise p-distances between *Colobopsis imitans* sequences (left). Colours match a bidimensional colour space. Haplotype network of *Colobopsis imitans* (right). Sequences > 599 bp: ID = 14, cf. = 0.

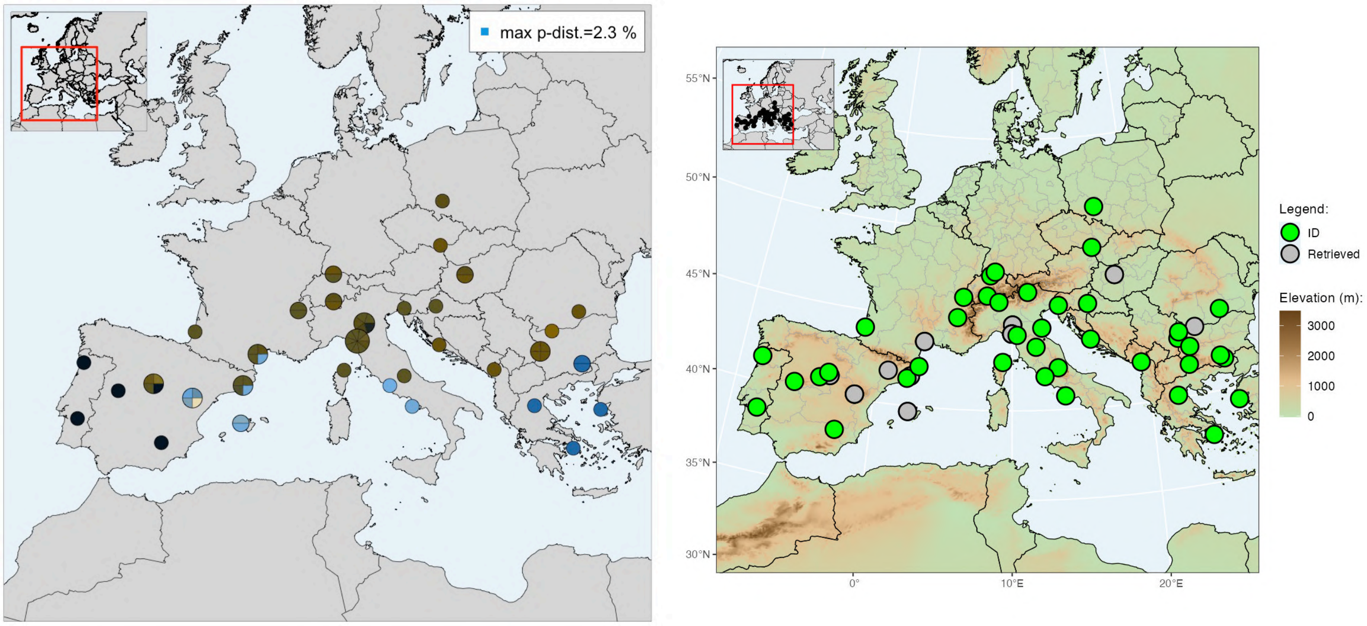

Figure 202: Genetic diversity map of *Colobopsis truncata* (Spinola, 1808). Nearby localities of sequenced specimens are merged in pies (left). Colours match the bidimensional colour space of the PCoA projection (Fig. 202 left) of p-dist between sequences (dots). Specimen identification (ID or cf.) and source (newly sequenced or retrieved) are represented by colours, while specimen attribute (terra typica, type locality, type specimen or faunistic novelty) is represented by the shape (right). Sequences: ID = 66, cf. = 0; maximum p-distance: strict = 2.3 %, less strict = 2.3 %.

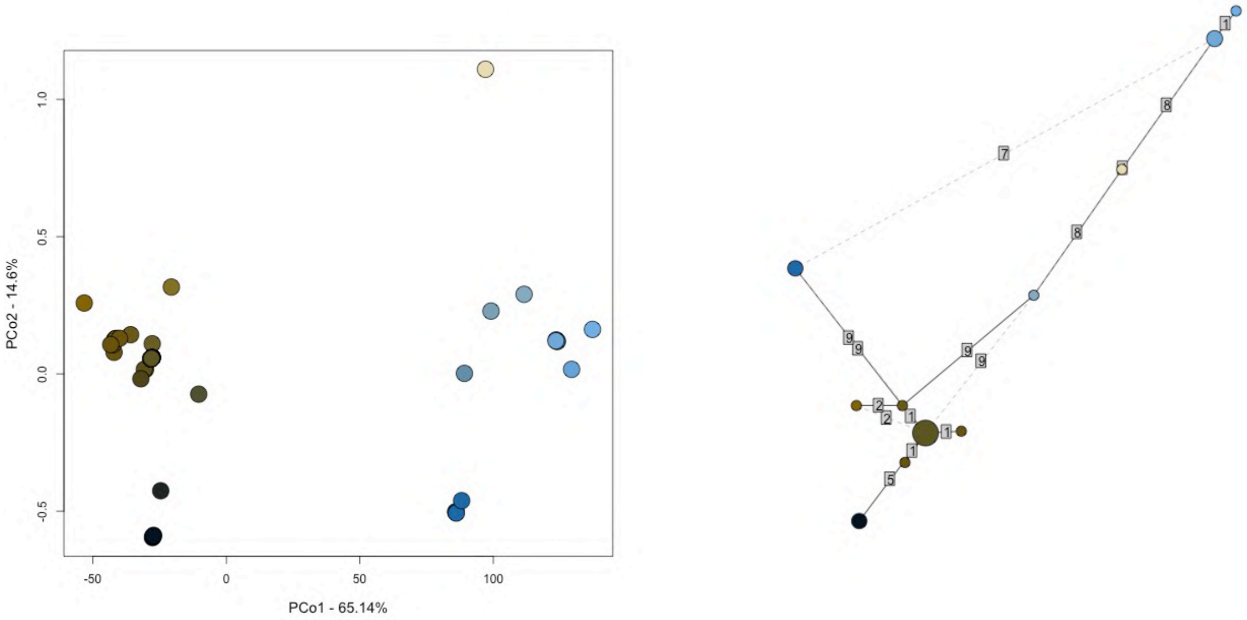

Figure 203: PCoA based on pairwise p-distances between *Colobopsis truncata* sequences (left). Colours match a bidimensional colour space. Haplotype network of *Colobopsis truncata* (right). Sequences > 599 bp: ID = 63, cf. = 0.

Crematogaster

Crematogaster auberti

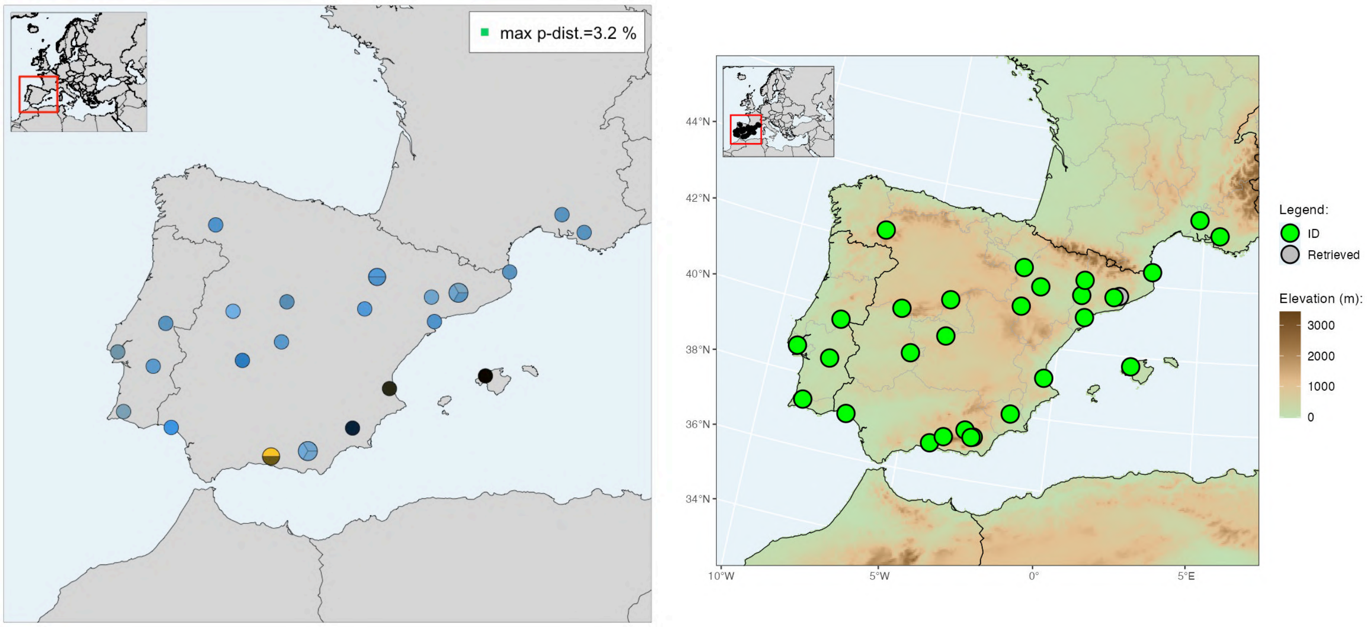

Figure 204: Genetic diversity map of *Crematogaster auberti* Emery, 1869. Nearby localities of sequenced specimens are merged in pies (left). Colours match the bidimensional colour space of the PCoA projection (Fig. 204 left) of p-dist between sequences (dots). Specimen identification (ID or cf.) and source (newly sequenced or retrieved) are represented by colours, while specimen attribute (terra typica, type locality, type specimen or faunistic novelty) is represented by the shape (right). Sequences: ID = 29, cf. = 0; maximum p-distance: strict = 3.2 %, less strict = 3.2 %.

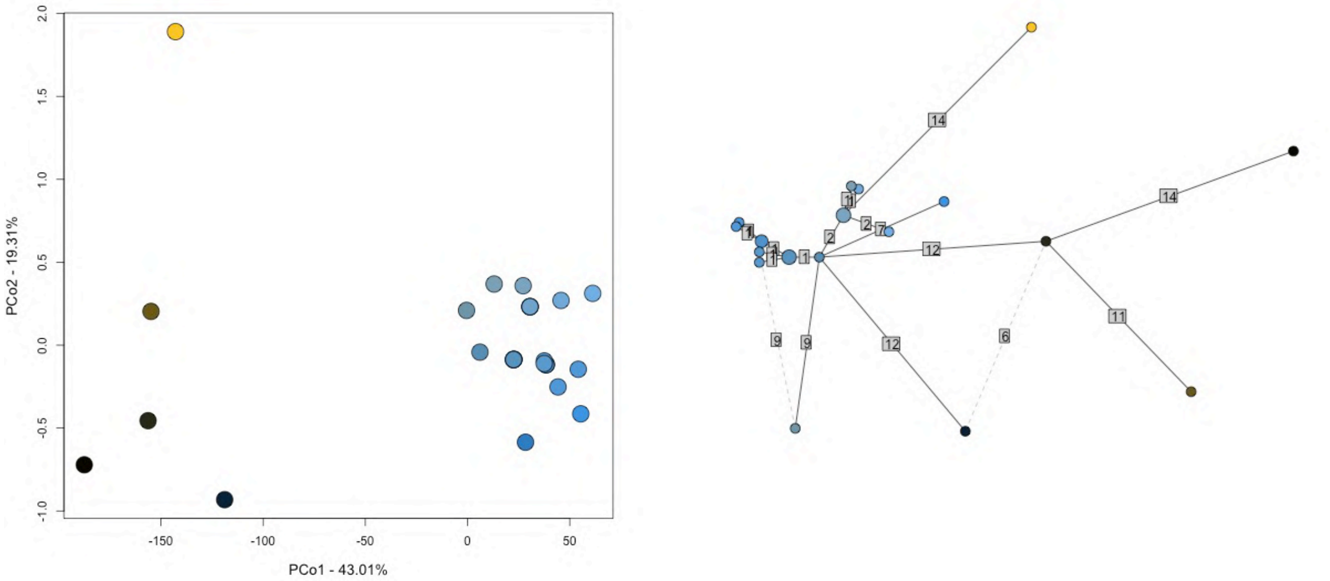

Figure 205: PCoA based on pairwise p-distances between *Crematogaster auberti* sequences (left). Colours match a bidimensional colour space. Haplotype network of *Crematogaster auberti* (right). Sequences > 599 bp: ID = 28, cf. = 0.

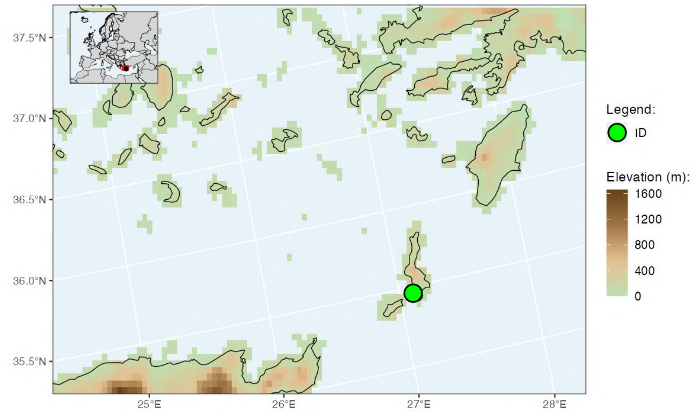

Figure 206: Map of *Crematogaster erectepilosa* Salata & Borowiec, 2015. Due to the presence of a single sequence, the genetic diversity map and the PCoA projection were not done. Specimen identification (ID or cf.) and source (newly sequenced or retrieved) are represented by colours, while specimen attribute (terra typica, type locality, type specimen or faunistic novelty) is represented by the shape. Sequences: ID = 1, cf. = 0; maximum p-distance: strict = NA, less strict = NA.

Haplotype network analysis of *Crematogaster erectepilosa* was not possible.

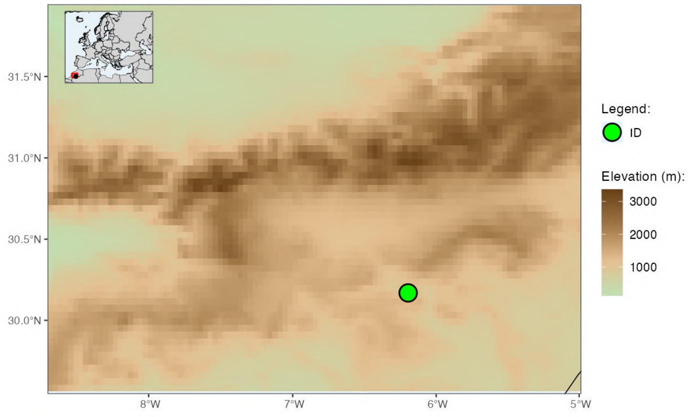

Figure 207: Map of *Crematogaster inermis* Mayr, 1862. Due to the presence of a single sequence, the genetic diversity map and the PCoA projection were not done. Specimen identification (ID or cf.) and source (newly sequenced or retrieved) are represented by colours, while specimen attribute (terra typica, type locality, type specimen or faunistic novelty) is represented by the shape. Sequences: ID = 1, cf. = 0; maximum p-distance: strict = NA, less strict = NA.

Haplotype network analysis of *Crematogaster inermis* was not possible.

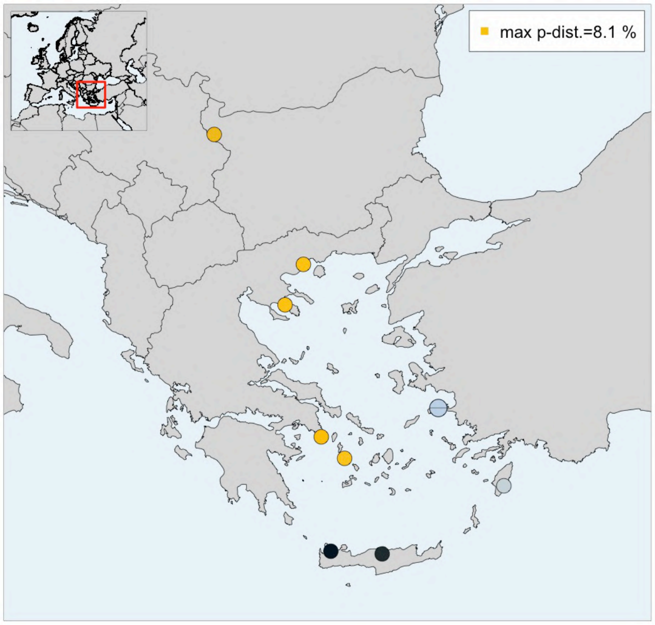

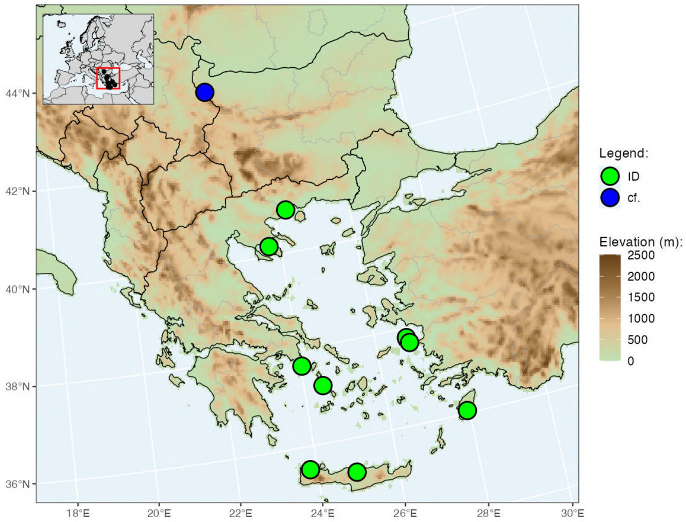

Figure 208: Genetic diversity map of *Crematogaster ionia* Forel, 1911. Nearby localities of sequenced specimens are merged in pies (left). Colours match the bidimensional colour space of the PCoA projection (Fig. 208 left) of p-dist between sequences (dots). Specimen identification (ID or cf.) and source (newly sequenced or retrieved) are represented by colours, while specimen attribute (terra typica, type locality, type specimen or faunistic novelty) is represented by the shape (right). Sequences: ID = 9, cf. = 1; maximum p-distance: strict = 8.1 %, less strict = 8.1 %.

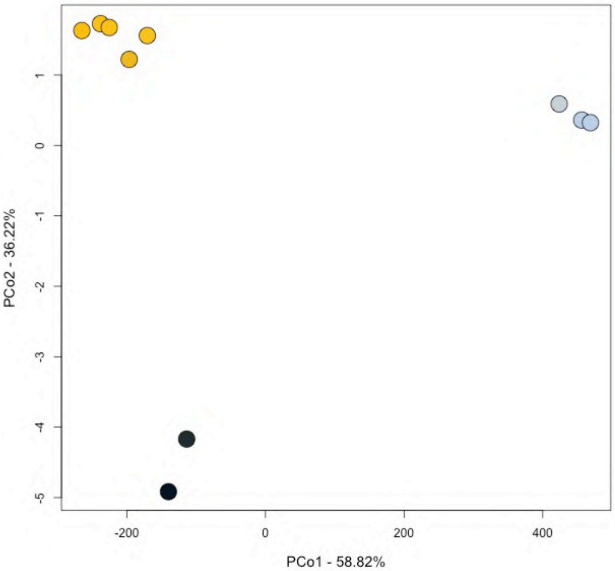

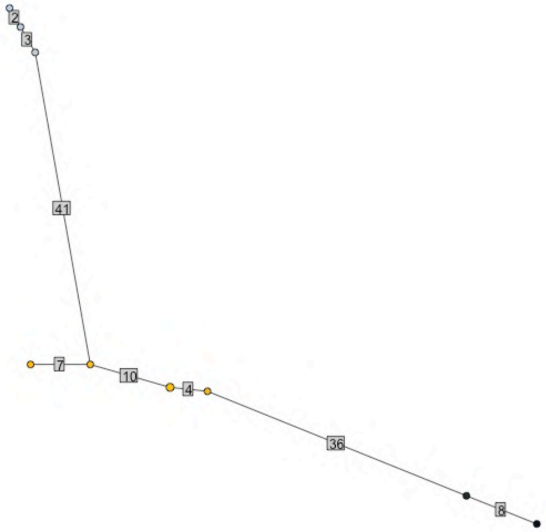

Figure 209: PCoA based on pairwise p-distances between *Crematogaster ionia* sequences (left). Colours match a bidimensional colour space. Haplotype network of *Crematogaster ionia* (right). Sequences > 599 bp: ID = 9, cf. = 1.

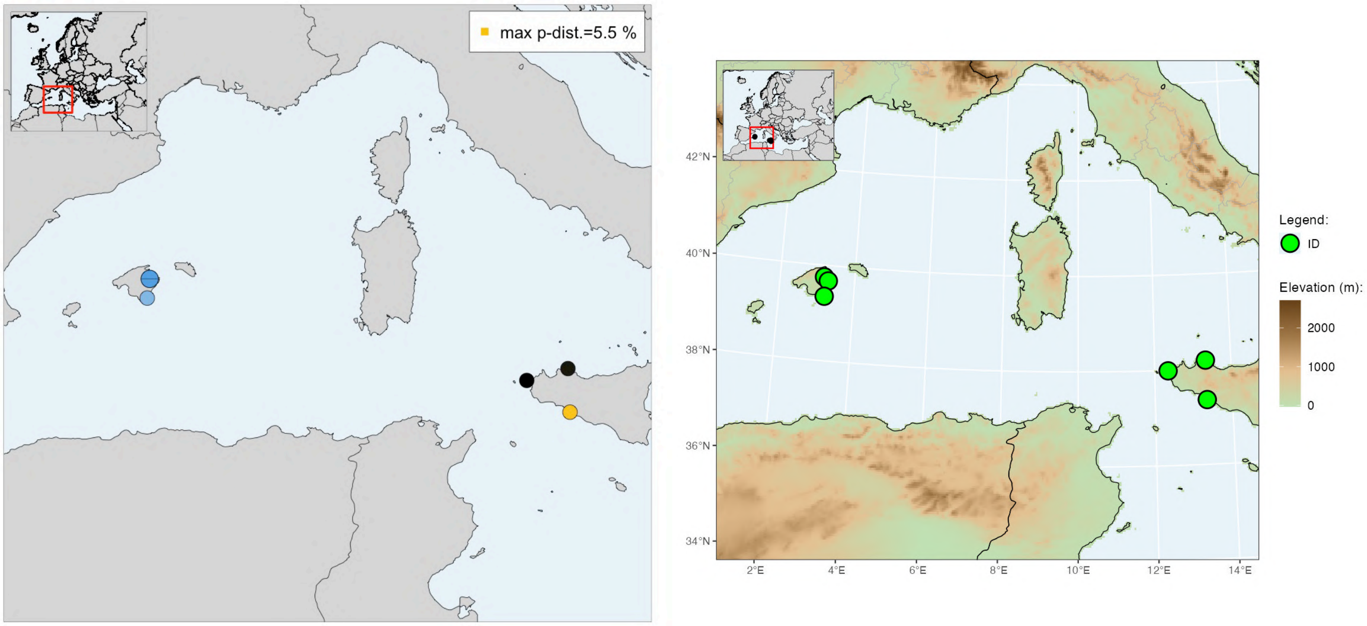

Figure 210: Genetic diversity map of *Crematogaster laestrygon* Emery, 1869. Nearby localities of sequenced specimens are merged in pies (left). Colours match the bidimensional colour space of the PCoA projection (Fig. 210 left) of p-dist between sequences (dots). Specimen identification (ID or cf.) and source (newly sequenced or retrieved) are represented by colours, while specimen attribute (terra typica, type locality, type specimen or faunistic novelty) is represented by the shape (right). Sequences: ID = 6, cf. = 0; maximum p-distance: strict = 5.5 %, less strict = 5.5 %.

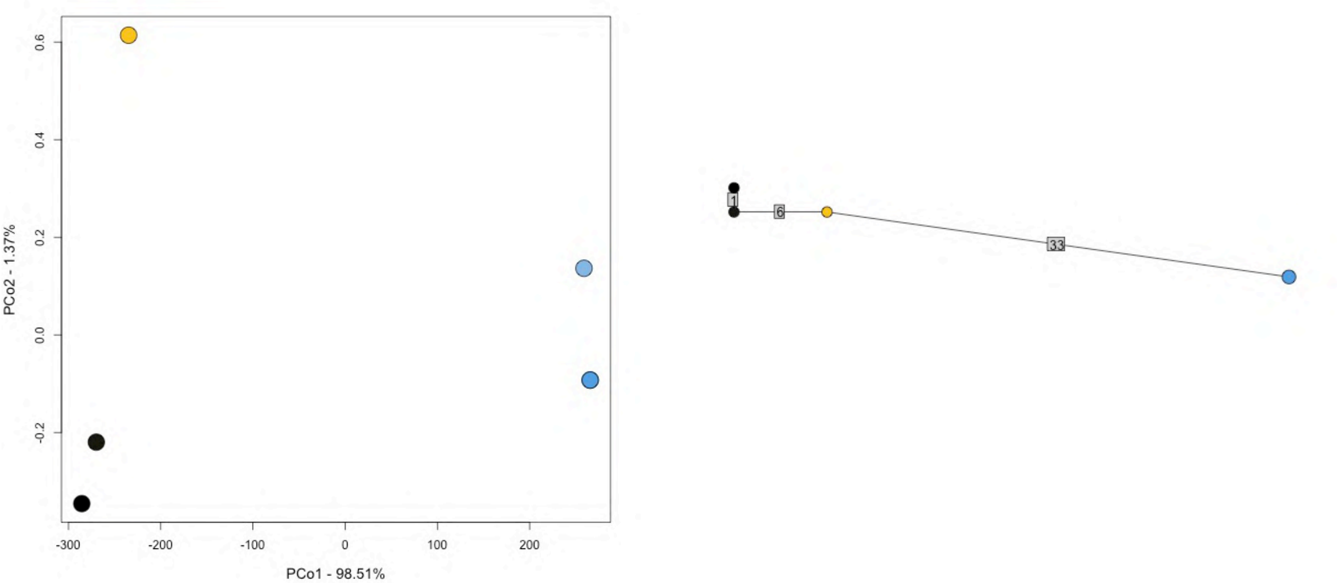

Figure 211: PCoA based on pairwise p-distances between *Crematogaster laestrygon* sequences (left). Colours match a bidimensional colour space. Haplotype network of *Crematogaster laestrygon* (right). Sequences > 599 bp: ID = 6, cf. = 0.

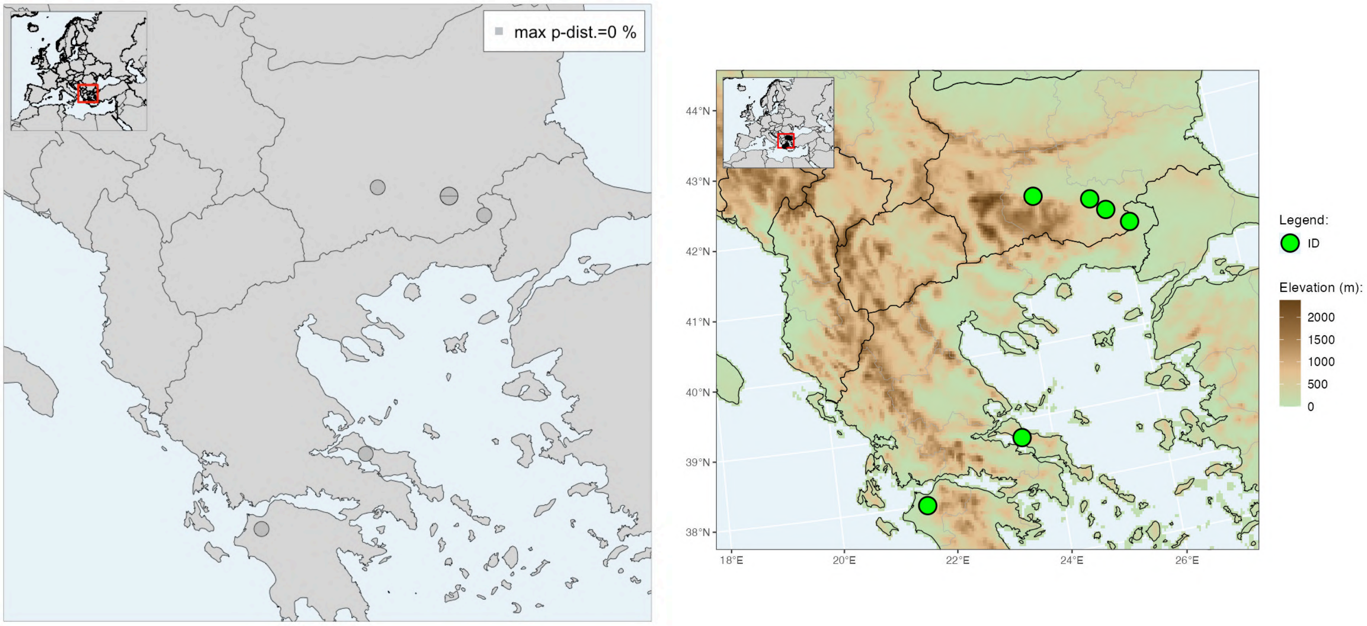

Figure 212: Genetic diversity map of *Crematogaster lorteti* Forel, 1910. PCoA projection was not done and therefore sequenced specimens in the genetic diversity map are coloured in gray (left). Specimen identification (ID or cf.) and source (newly sequenced or retrieved) are represented by colours, while specimen attribute (terra typica, type locality, type specimen or faunistic novelty) is represented by the shape (right). Sequences: ID = 6, cf. = 0; maximum p-distance: strict = 0 %, less strict = 0 %.

Haplotype network analysis of *Crematogaster lorteti* was not possible.

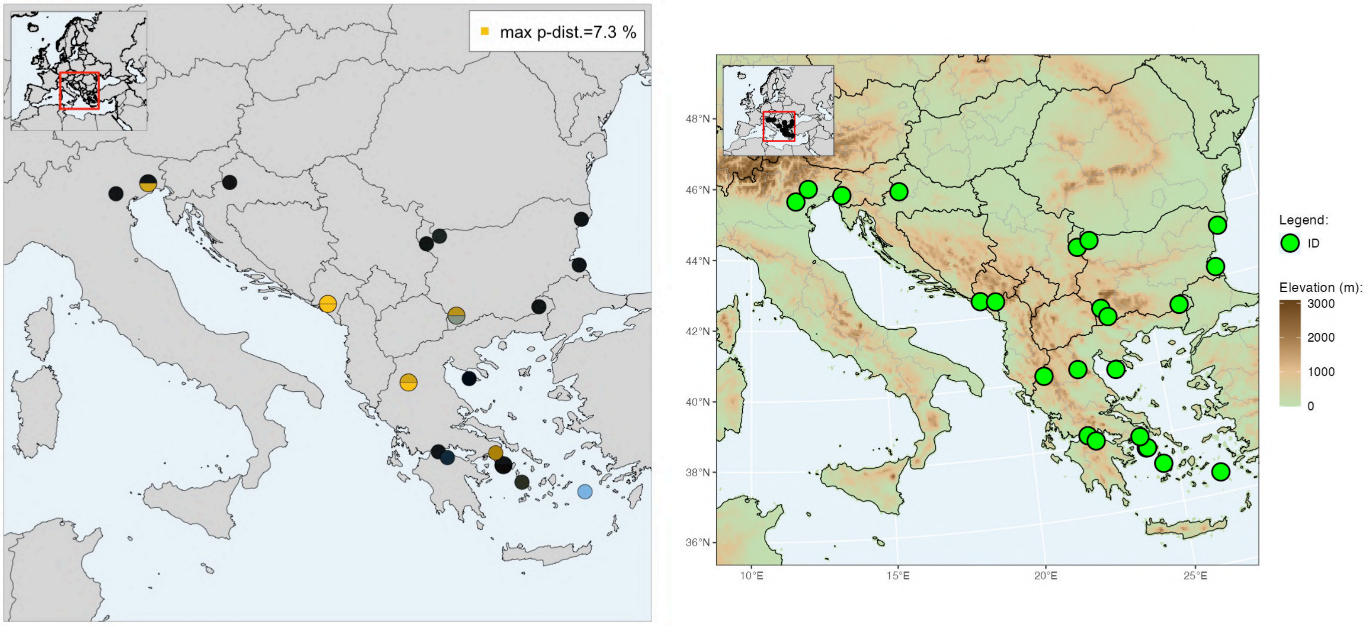

Figure 213: Genetic diversity map of *Crematogaster schmidti* (Mayr, 1853). Nearby localities of sequenced specimens are merged in pies (left). Colours match the bidimensional colour space of the PCoA projection (Fig. 213 left) of p-dist between sequences (dots). Specimen identification (ID or cf.) and source (newly sequenced or retrieved) are represented by colours, while specimen attribute (terra typica, type locality, type specimen or faunistic novelty) is represented by the shape (right). Sequences: ID = 23, cf. = 0; maximum p-distance: strict = 7.3 %, less strict = 7.3 %.

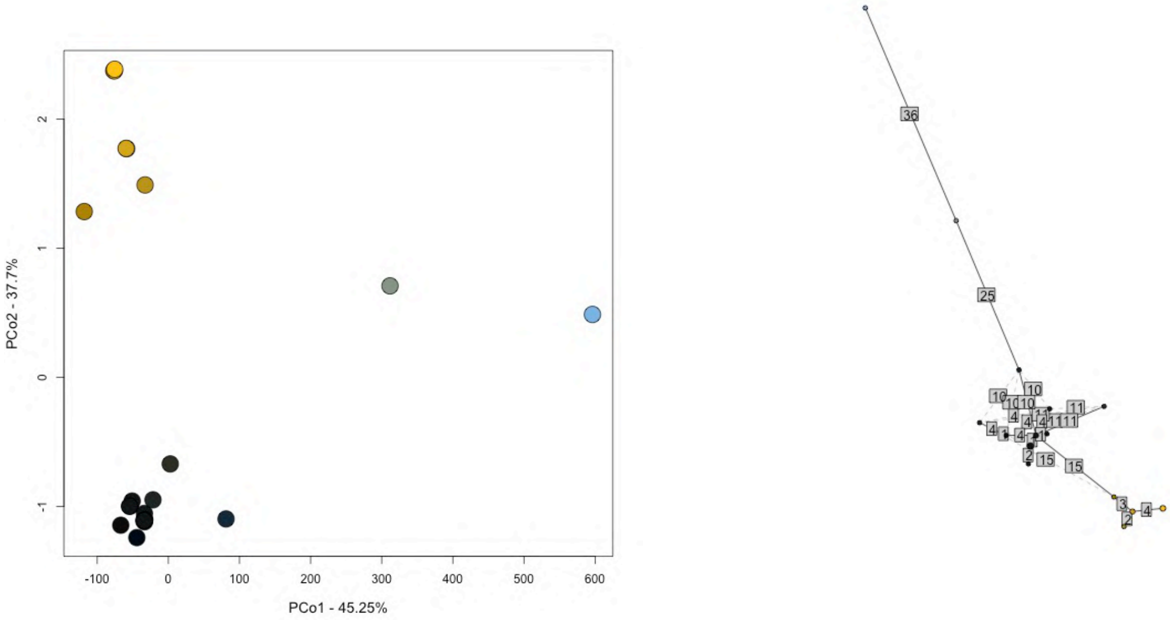

Figure 214: PCoA based on pairwise p-distances between *Crematogaster schmidti* sequences (left). Colours match a bidimensional colour space. Haplotype network of *Crematogaster schmidti* (right). Sequences > 599 bp: ID = 23, cf. = 0.

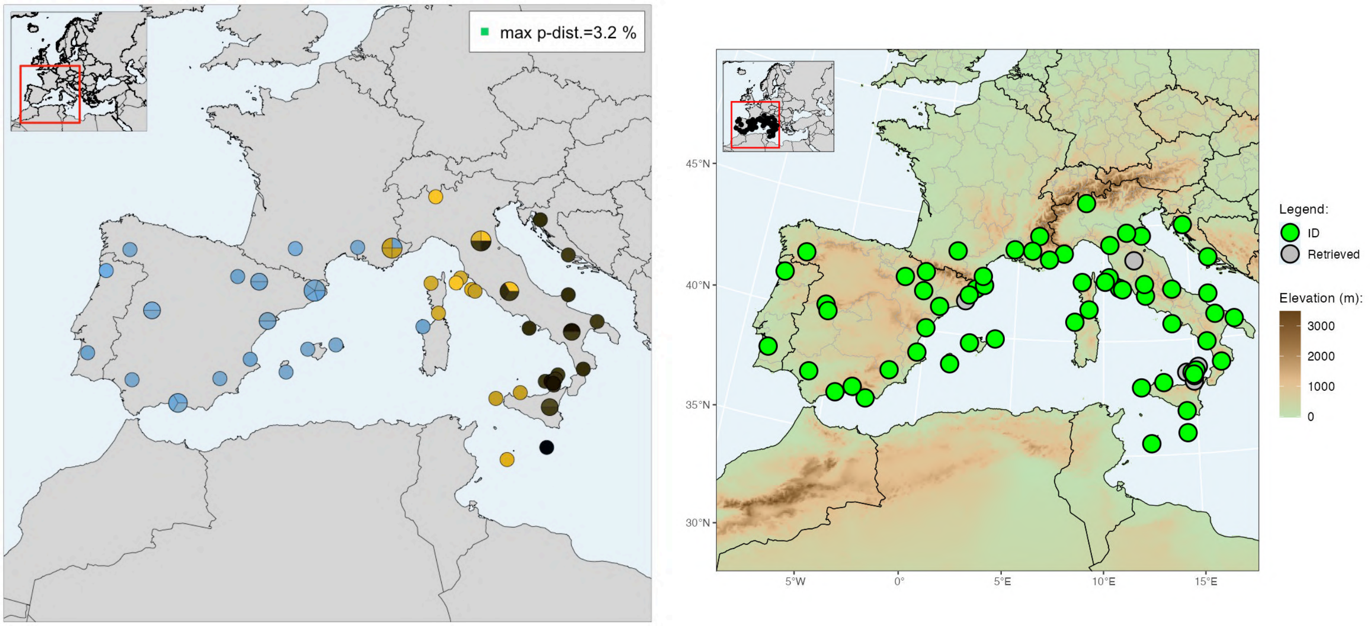

Figure 215: Genetic diversity map of *Crematogaster scutellaris* (Olivier, 1792). Nearby localities of sequenced specimens are merged in pies (left). Colours match the bidimensional colour space of the PCoA projection (Fig. 215 left) of p-dist between sequences (dots). Specimen identification (ID or cf.) and source (newly sequenced or retrieved) are represented by colours, while specimen attribute (terra typica, type locality, type specimen or faunistic novelty) is represented by the shape (right). Sequences: ID = 65, cf. = 0; maximum p-distance: strict = 3.2 %, less strict = 3.2 %.

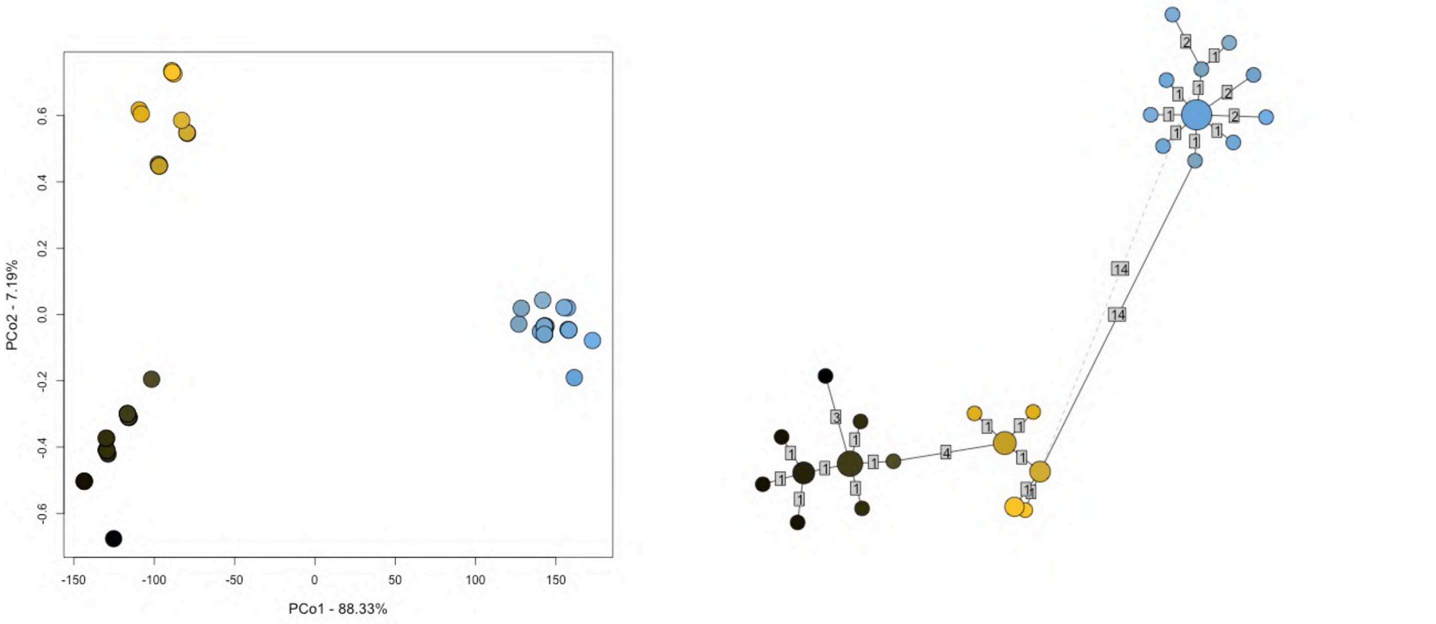

Figure 216: PCoA based on pairwise p-distances between *Crematogaster scutellaris* sequences (left). Colours match a bidimensional colour space. Haplotype network of *Crematogaster scutellaris* (right). Sequences > 599 bp: ID = 65, cf. = 0.

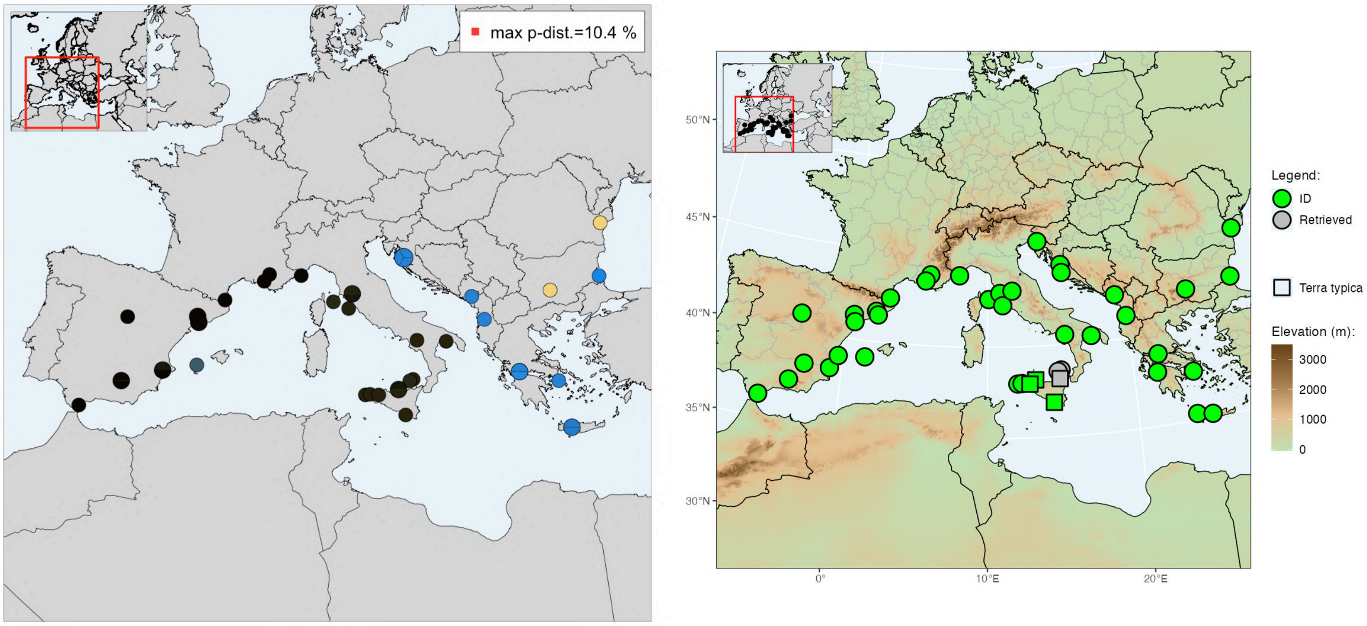

Figure 217: Genetic diversity map of *Crematogaster sordidula* (Nylander, 1849). Nearby localities of sequenced specimens are merged in pies (left). Colours match the bidimensional colour space of the PCoA projection (Fig. 217 left) of p-dist between sequences (dots). Specimen identification (ID or cf.) and source (newly sequenced or retrieved) are represented by colours, while specimen attribute (terra typica, type locality, type specimen or faunistic novelty) is represented by the shape (right). Sequences: ID = 42, cf. = 0; maximum p-distance: strict = 10.4 %, less strict = 10.4 %.

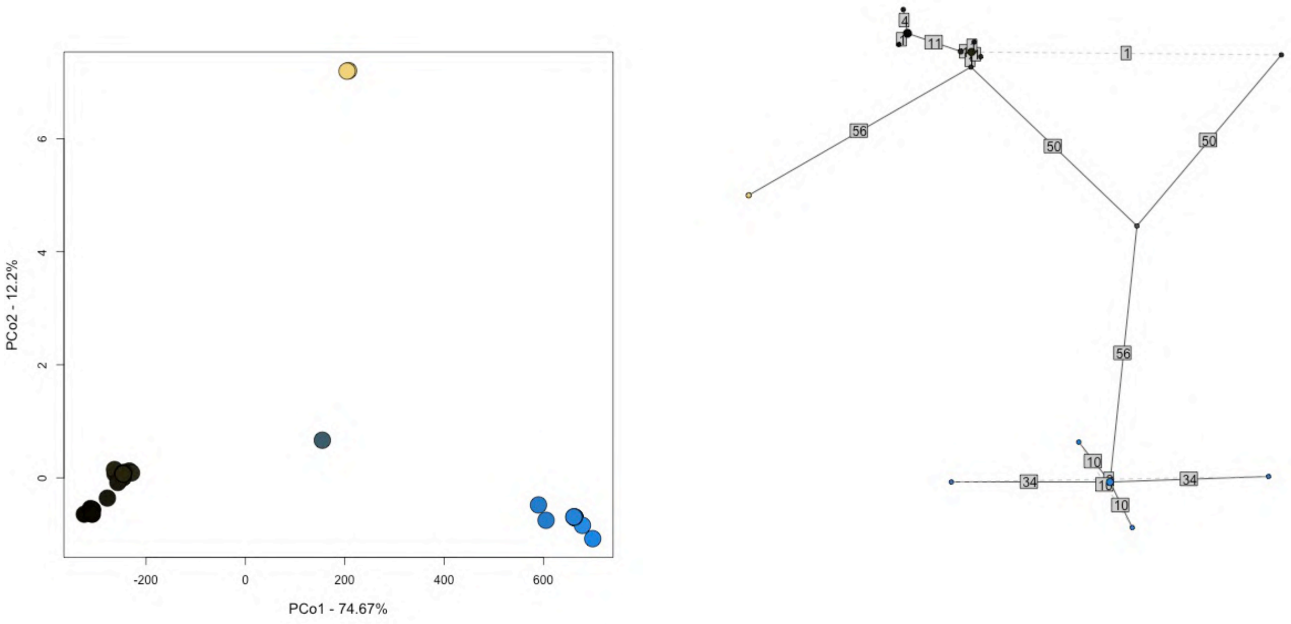

Figure 218: PCoA based on pairwise p-distances between *Crematogaster sordidula* sequences (left). Colours match a bidimensional colour space. Haplotype network of *Crematogaster sordidula* (right). Sequences > 599 bp: ID = 42, cf. = 0.

Cryptopone

Cryptopone ochracea

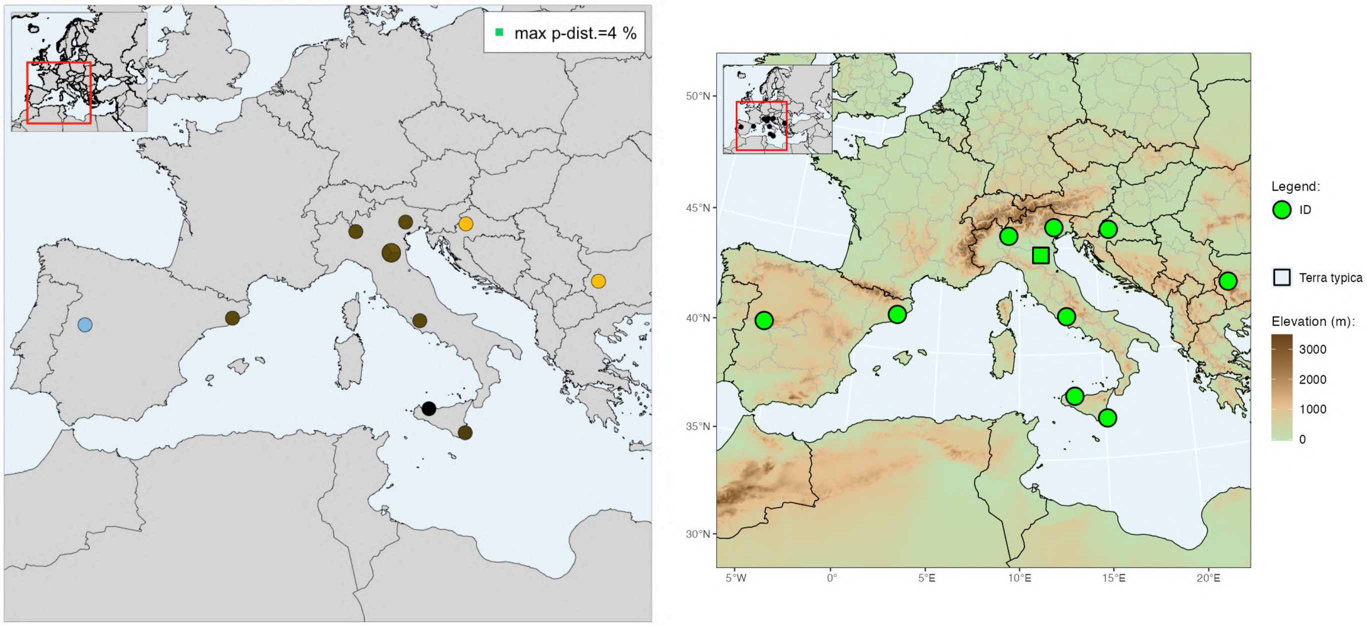

Figure 219: Genetic diversity map of *Cryptopone ochracea* (Mayr, 1855). Nearby localities of sequenced specimens are merged in pies (left). Colours match the bidimensional colour space of the PCoA projection (Fig. 219 left) of p-dist between sequences (dots). Specimen identification (ID or cf.) and source (newly sequenced or retrieved) are represented by colours, while specimen attribute (terra typica, type locality, type specimen or faunistic novelty) is represented by the shape (right). Sequences: ID = 12, cf. = 0; maximum p-distance: strict = 4 %, less strict = 4 %.

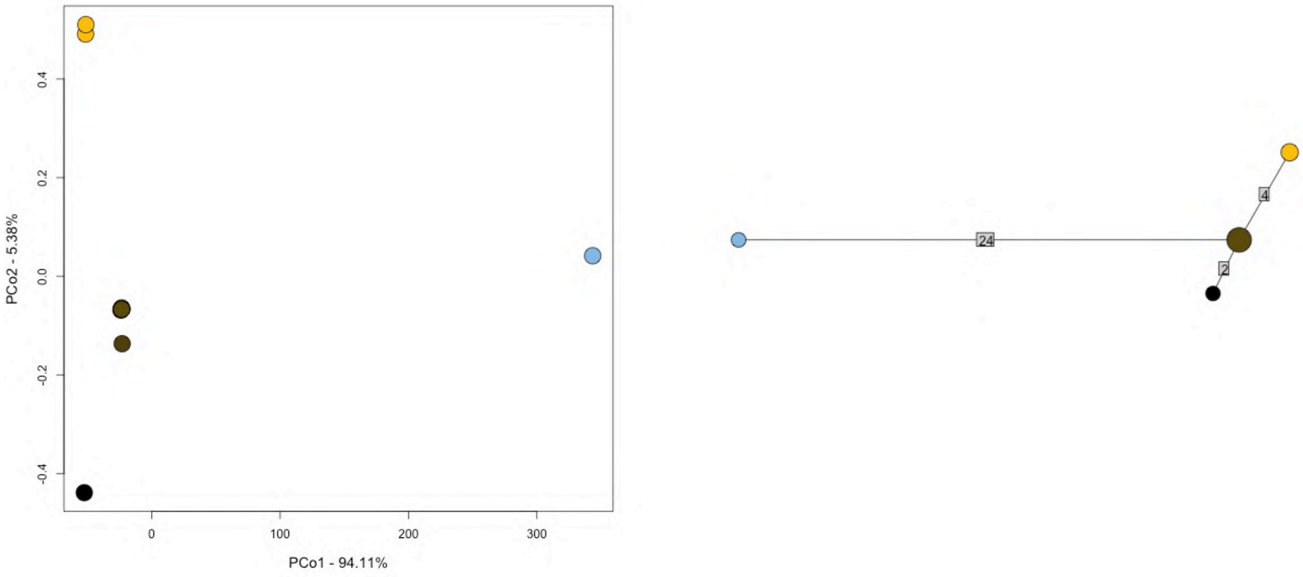

Figure 220: PCoA based on pairwise p-distances between *Cryptopone ochracea* sequences (left). Colours match a bidimensional colour space. Haplotype network of *Cryptopone ochracea* (right). Sequences > 599 bp: ID = 12, cf. = 0.

Dolichoderus

Dolichoderus quadripunctatus

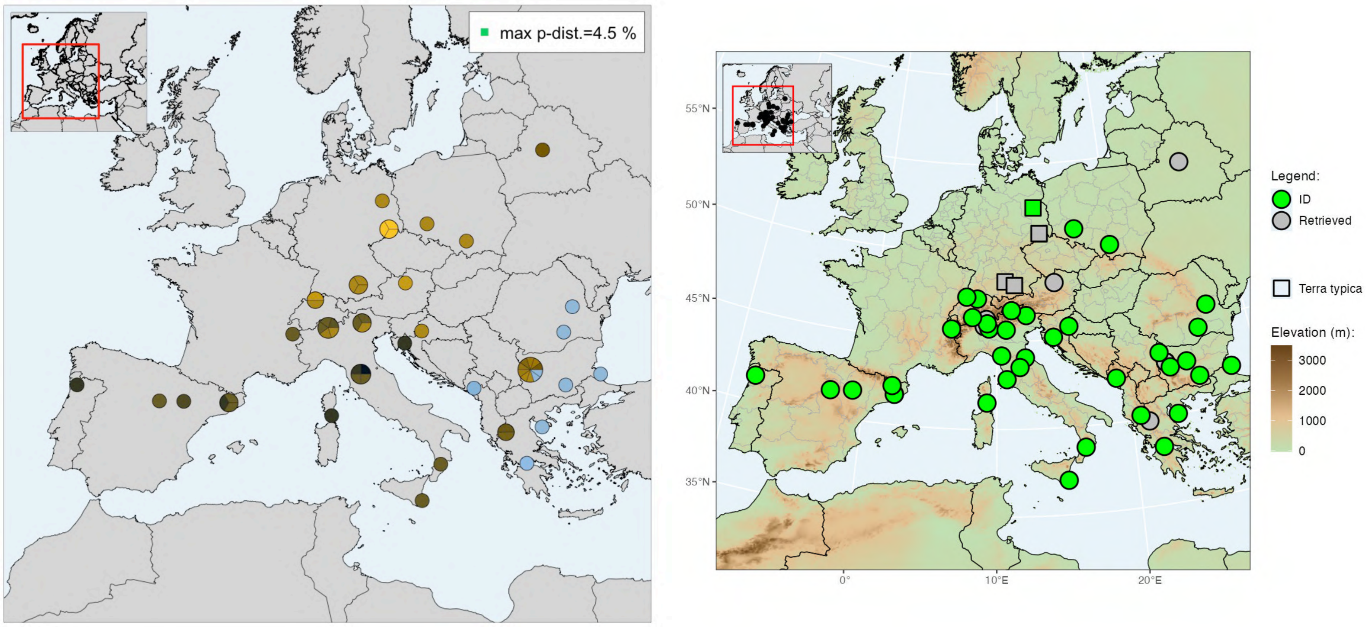

Figure 221: Genetic diversity map of *Dolichoderus quadripunctatus* (Linnaeus, 1771). Nearby localities of sequenced specimens are merged in pies (left). Colours match the bidimensional colour space of the PCoA projection (Fig. 221 left) of p-dist between sequences (dots). Specimen identification (ID or cf.) and source (newly sequenced or retrieved) are represented by colours, while specimen attribute (terra typica, type locality, type specimen or faunistic novelty) is represented by the shape (right). Sequences: ID = 56, cf. = 0; maximum p-distance: strict = 4.1 %, less strict = 4.5 %.

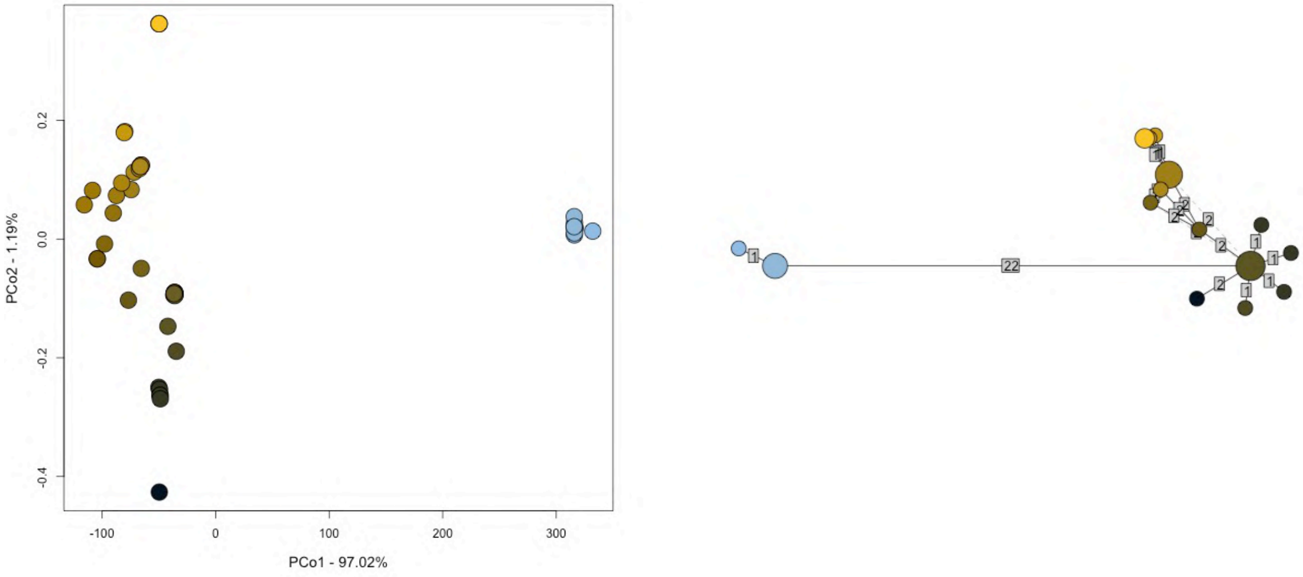

Figure 222: PCoA based on pairwise p-distances between *Dolichoderus quadripunctatus* sequences (left). Colours match a bidimensional colour space. Haplotype network of *Dolichoderus quadripunctatus* (right). Sequences > 599 bp: ID = 48, cf. = 0.

Formica

Formica aquilonia

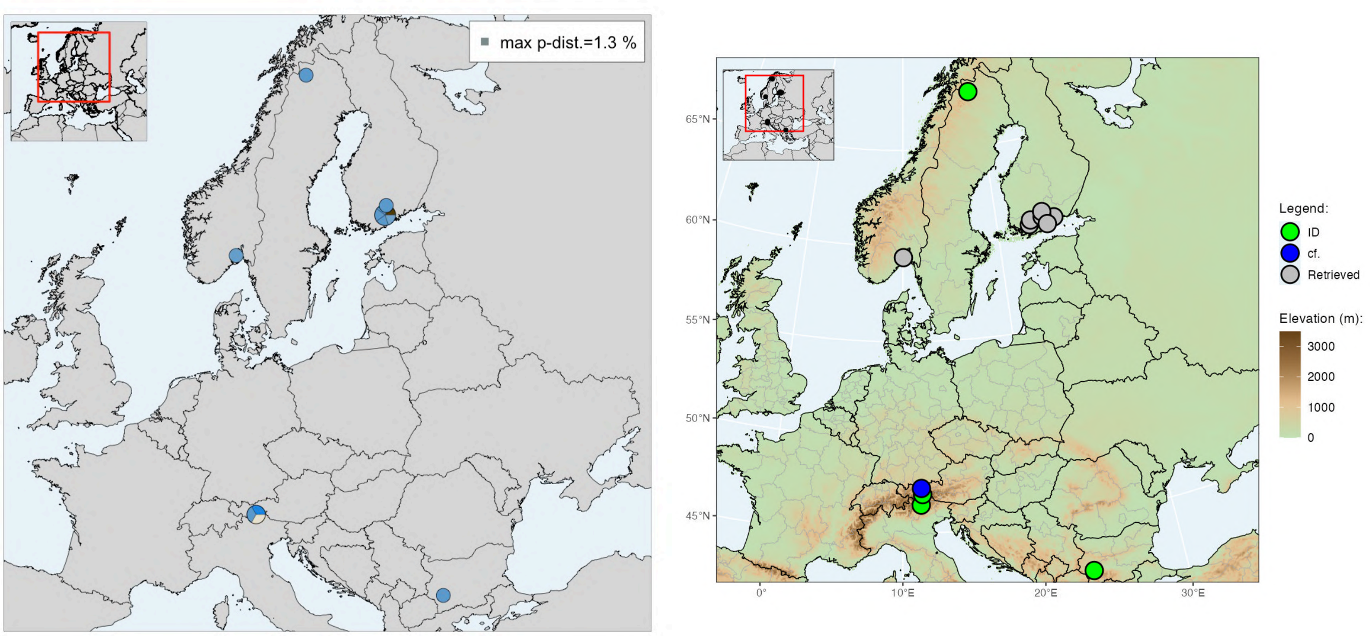

Figure 223: Genetic diversity map of *Formica aquilonia* Yarrow, 1955. Nearby localities of sequenced specimens are merged in pies (left). Colours match the bidimensional colour space of the PCoA projection (Fig. 223 left) of p-dist between sequences (dots). Specimen identification (ID or cf.) and source (newly sequenced or retrieved) are represented by colours, while specimen attribute (terra typica, type locality, type specimen or faunistic novelty) is represented by the shape (right). Sequences: ID = 11, cf. = 1; maximum p-distance: strict = 1.3 %, less strict = 1.3 %.

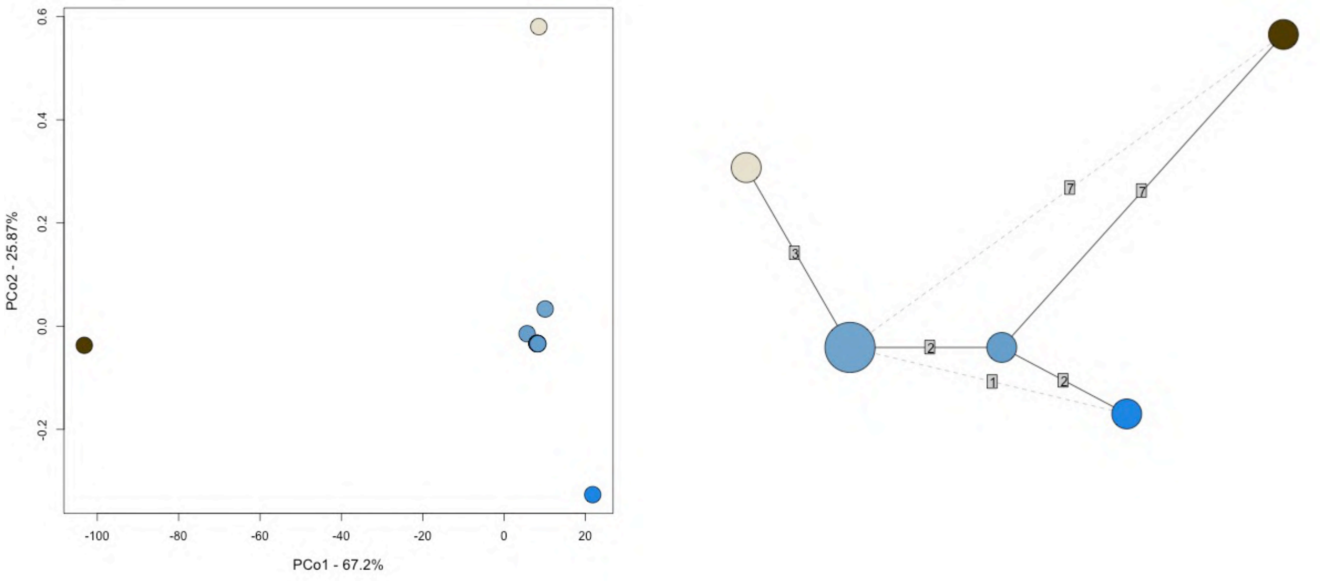

Figure 224: PCoA based on pairwise p-distances between *Formica aquilonia* sequences (left). Colours match a bidimensional colour space. Haplotype network of *Formica aquilonia* (right). Sequences > 599 bp: ID = 11, cf. = 1.

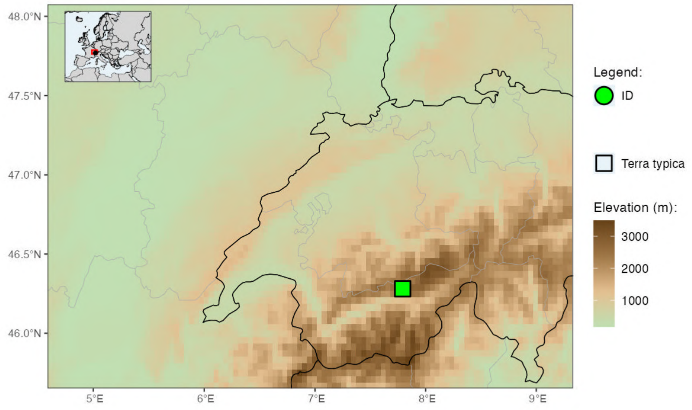

Figure 225: Map of *Formica bruni* Kutter, 1967. Due to the presence of a single sequence, the genetic diversity map and the PCoA projection were not done. Specimen identification (ID or cf.) and source (newly sequenced or retrieved) are represented by colours, while specimen attribute (terra typica, type locality, type specimen or faunistic novelty) is represented by the shape. Sequences: ID = 1, cf. = 0; maximum p-distance: strict = NA, less strict = NA.

Haplotype network analysis of *Formica bruni* was not possible.

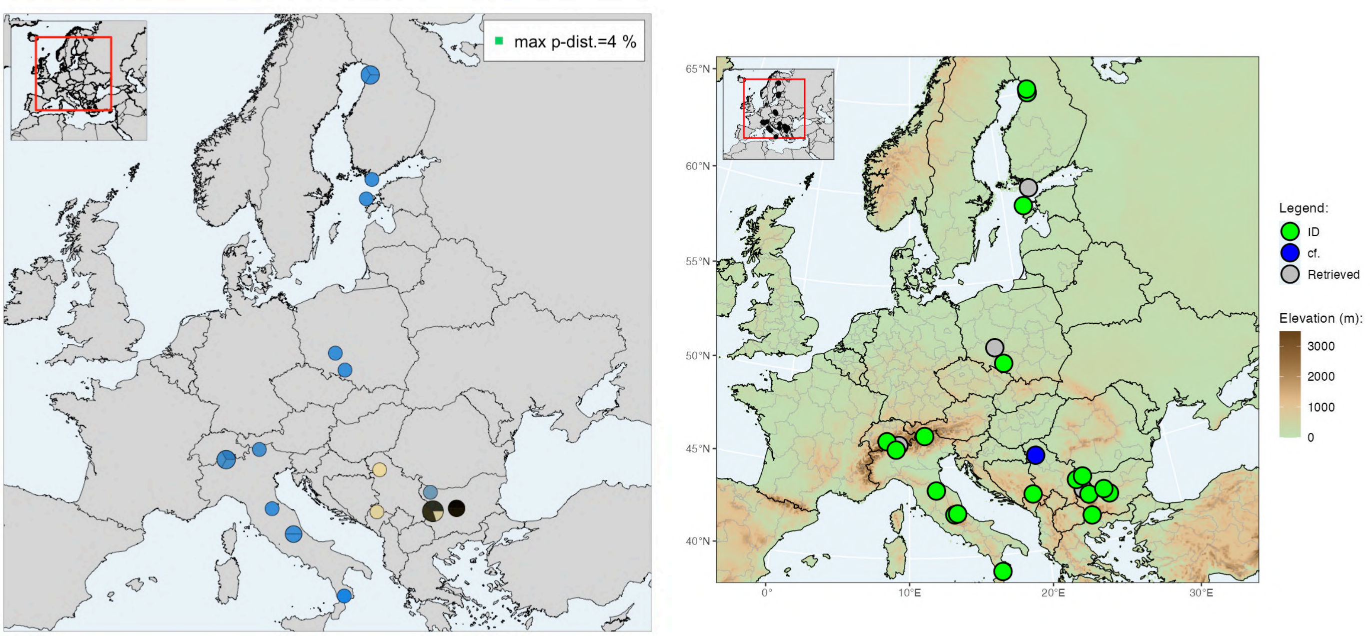

Figure 226: Genetic diversity map of *Formica cinerea* Mayr, 1853. Nearby localities of sequenced specimens are merged in pies (left). Colours match the bidimensional colour space of the PCoA projection (Fig. 226 left) of p-dist between sequences (dots). Specimen identification (ID or cf.) and source (newly sequenced or retrieved) are represented by colours, while specimen attribute (terra typica, type locality, type specimen or faunistic novelty) is represented by the shape (right). Sequences: ID = 25, cf. = 1; maximum p-distance: strict = 4 %, less strict = 4 %.

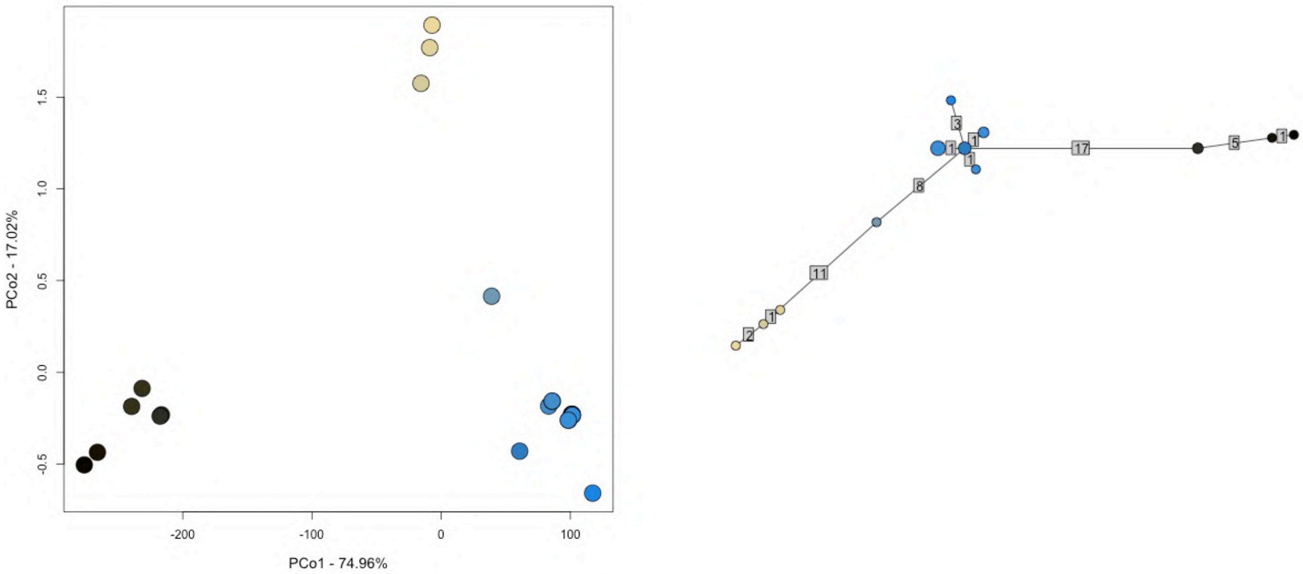

Figure 227: PCoA based on pairwise p-distances between *Formica cinerea* sequences (left). Colours match a bidimensional colour space. Haplotype network of *Formica cinerea* (right). Sequences > 599 bp: ID = 23, cf. = 1.

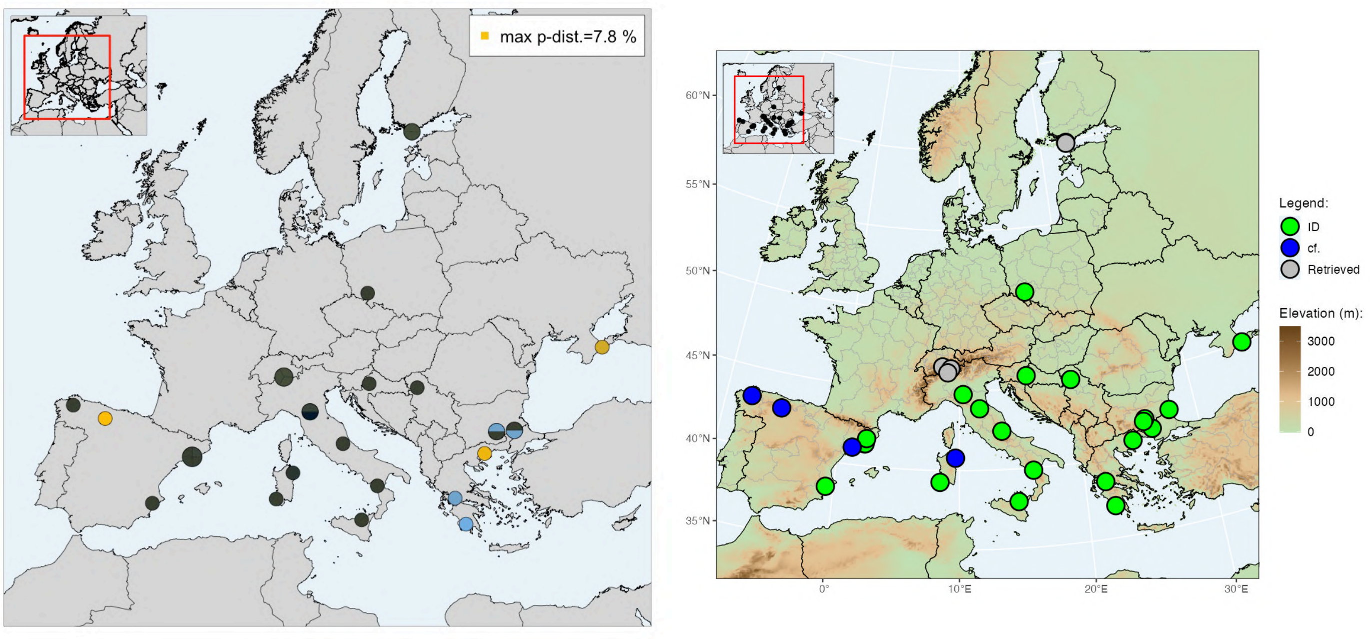

Figure 228: Genetic diversity map of *Formica clara* Forel, 1886. Nearby localities of sequenced specimens are merged in pies (left). Colours match the bidimensional colour space of the PCoA projection (Fig. 228 left) of p-dist between sequences (dots). Specimen identification (ID or cf.) and source (newly sequenced or retrieved) are represented by colours, while specimen attribute (terra typica, type locality, type specimen or faunistic novelty) is represented by the shape (right). Sequences: ID = 26, cf. = 4; maximum p-distance: strict = 7.2 %, less strict = 7.8 %.

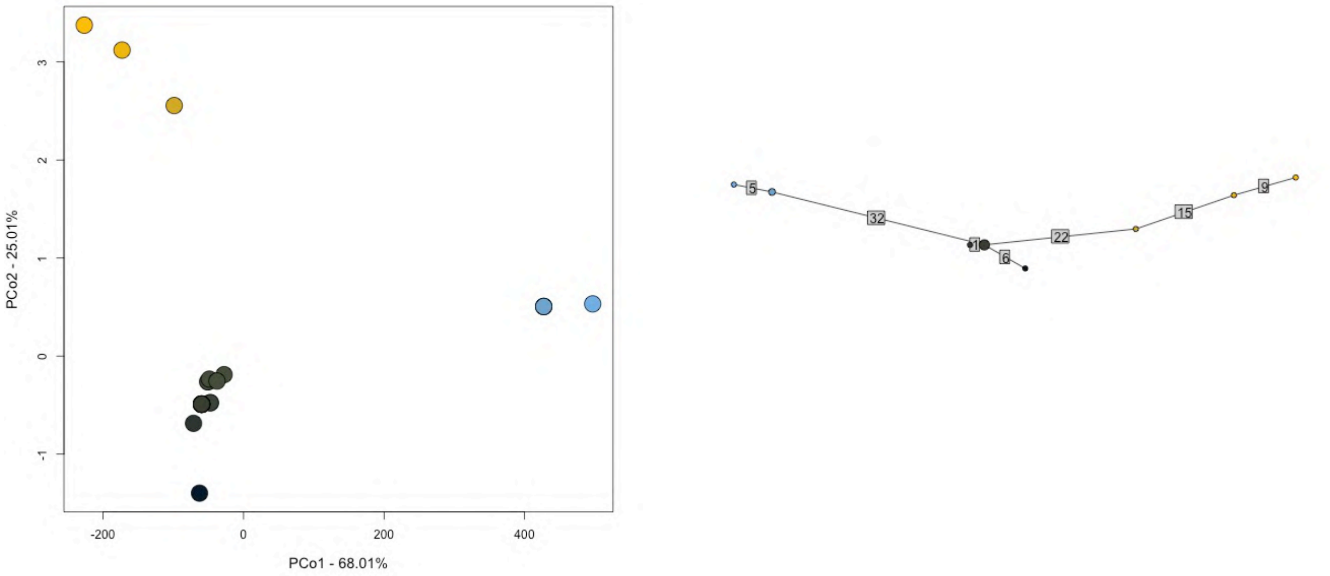

Figure 229: PCoA based on pairwise p-distances between *Formica clara* sequences (left). Colours match a bidimensional colour space. Haplotype network of *Formica clara* (right). Sequences > 599 bp: ID = 21, cf. = 4.

Formica corsica

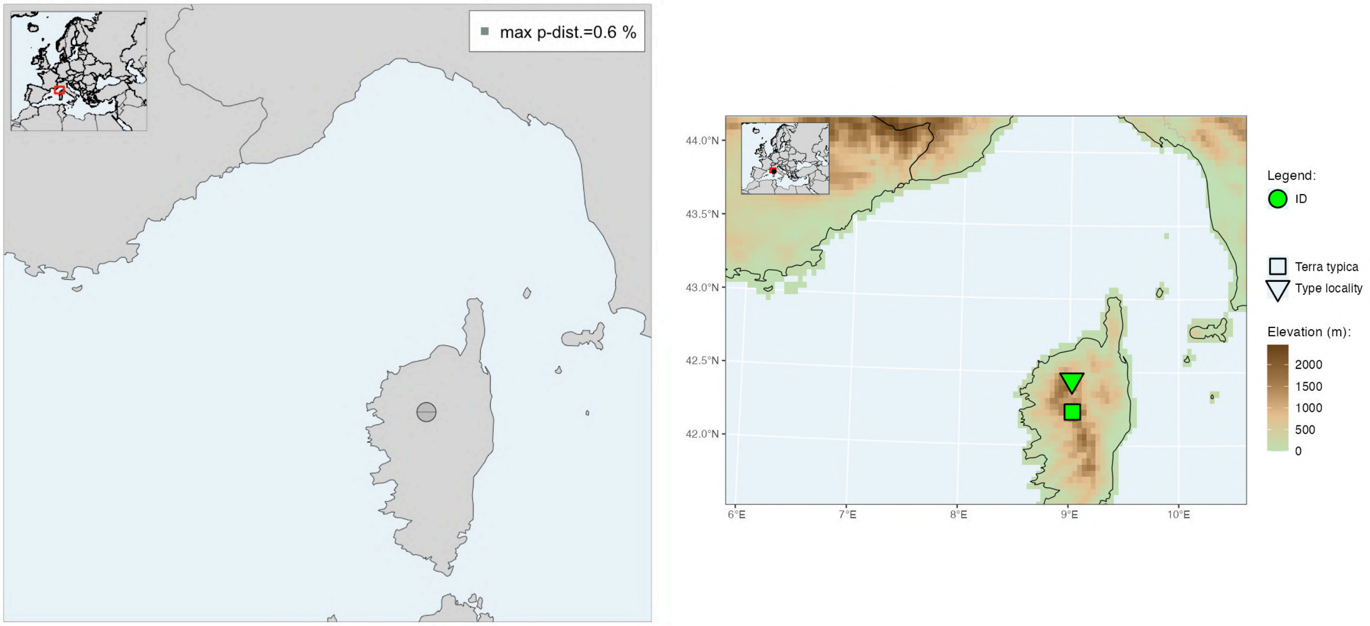

Figure 230: Genetic diversity map of *Formica corsica* Seifert, 2002. PCoA projection was not done and therefore sequenced specimens in the genetic diversity map are coloured in gray (left). Specimen identification (ID or cf.) and source (newly sequenced or retrieved) are represented by colours, while specimen attribute (terra typica, type locality, type specimen or faunistic novelty) is represented by the shape (right). Sequences: ID = 2, cf. = 0; maximum p-distance: strict = NA, less strict = 0.6 %.

Haplotype network analysis of *Formica corsica* was not possible.

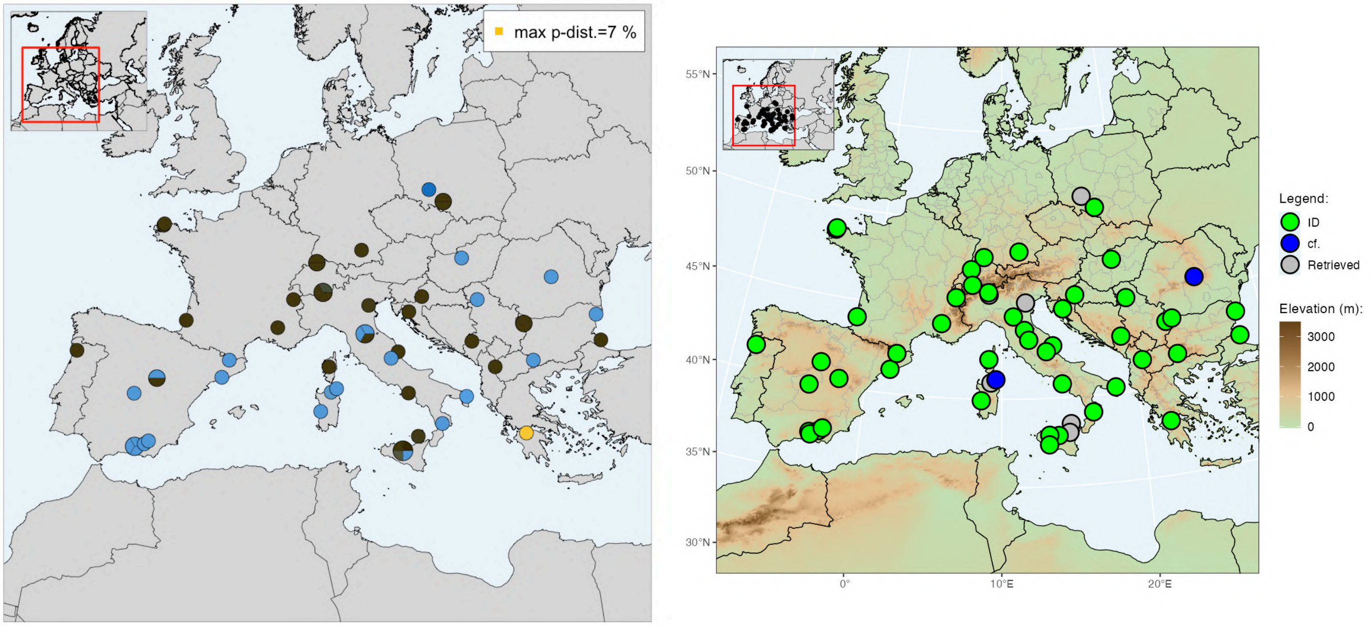

Figure 231: Genetic diversity map of *Formica cunicularia* Latreille, 1798. Nearby localities of sequenced specimens are merged in pies (left). Colours match the bidimensional colour space of the PCoA projection (Fig. 231 left) of p-dist between sequences (dots). Specimen identification (ID or cf.) and source (newly sequenced or retrieved) are represented by colours, while specimen attribute (terra typica, type locality, type specimen or faunistic novelty) is represented by the shape (right). Sequences: ID = 54, cf. = 2; maximum p-distance: strict = 7 %, less strict = 7 %.

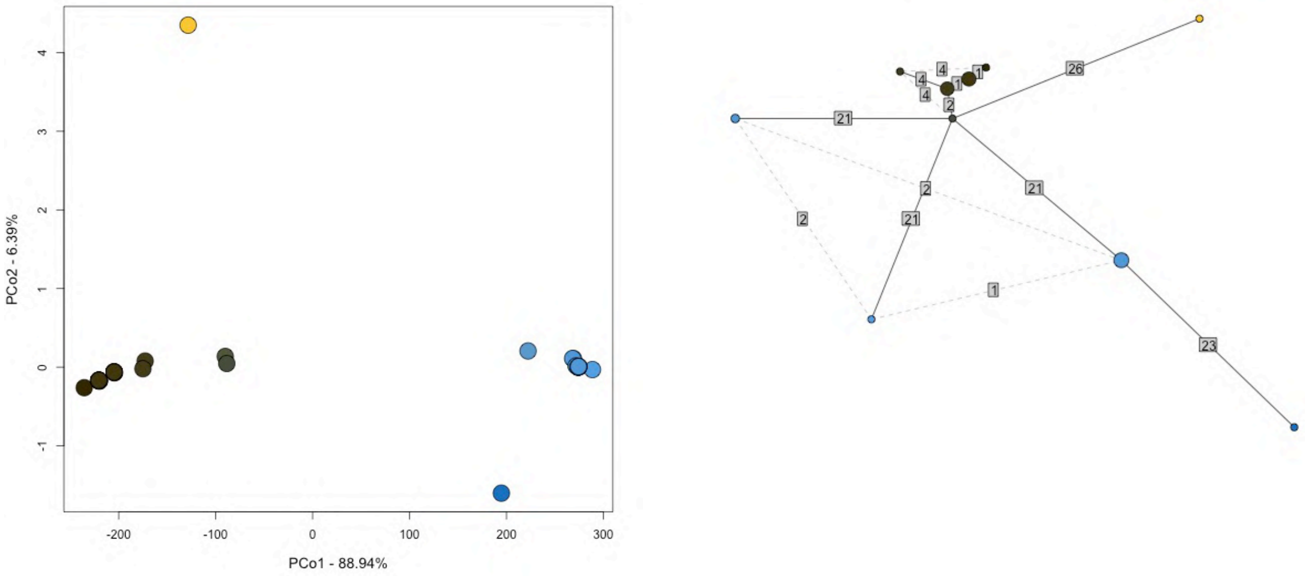

Figure 232: PCoA based on pairwise p-distances between *Formica cunicularia* sequences (left). Colours match a bidimensional colour space. Haplotype network of *Formica cunicularia* (right). Sequences > 599 bp: ID = 53, cf. = 2.

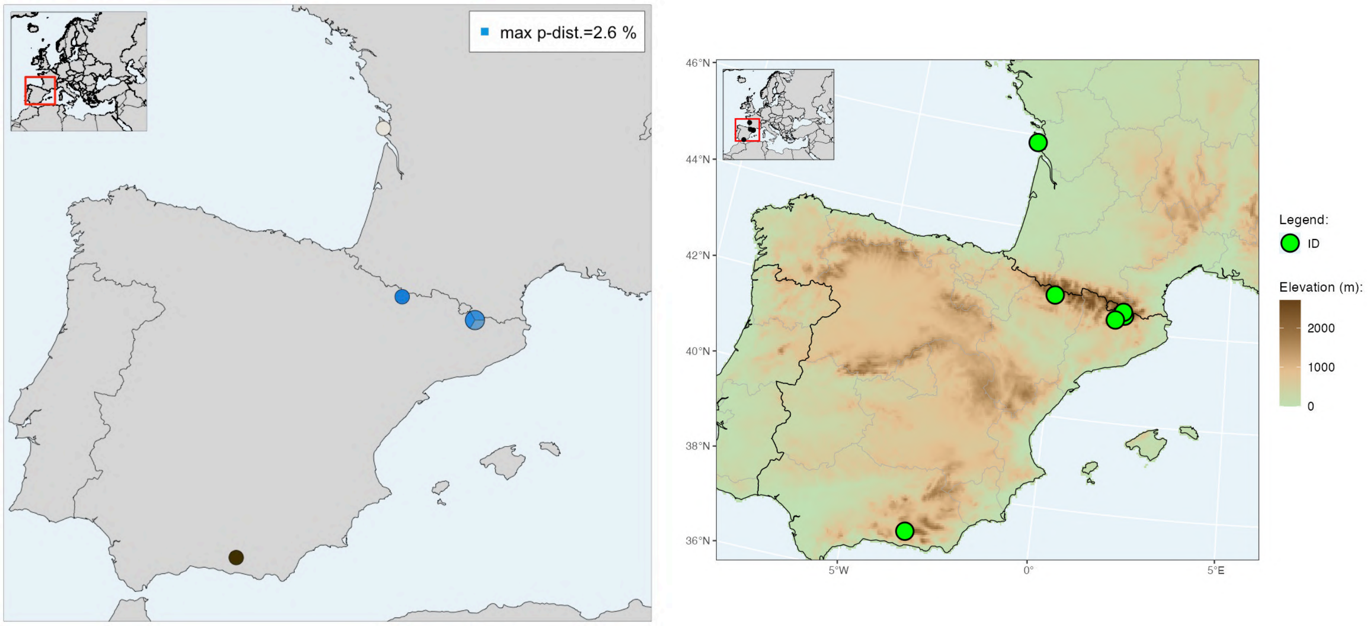

Figure 233: Genetic diversity map of *Formica decipiens* Bondroit, 1918. Nearby localities of sequenced specimens are merged in pies (left). Colours match the bidimensional colour space of the PCoA projection (Fig. 233 left) of p-dist between sequences (dots). Specimen identification (ID or cf.) and source (newly sequenced or retrieved) are represented by colours, while specimen attribute (terra typica, type locality, type specimen or faunistic novelty) is represented by the shape (right). Sequences: ID = 8, cf. = 0; maximum p-distance: strict = 2.6 %, less strict = 2.6 %.

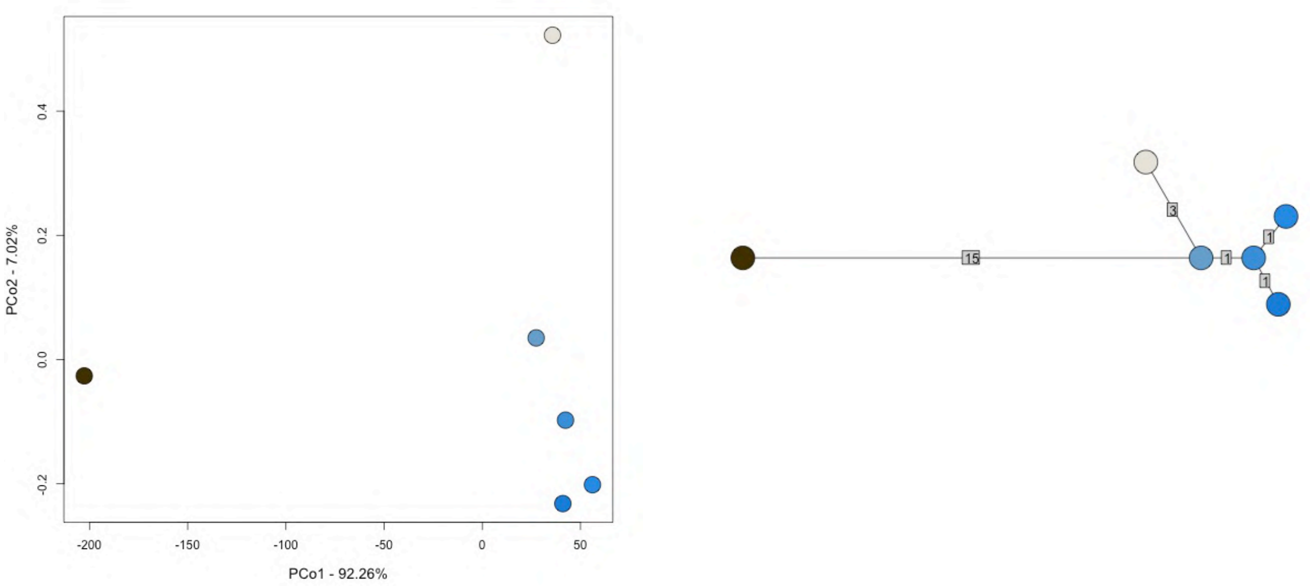

Figure 234: PCoA based on pairwise p-distances between *Formica decipiens* sequences (left). Colours match a bidimensional colour space. Haplotype network of *Formica decipiens* (right). Sequences > 599 bp: ID = 8, cf. = 0.

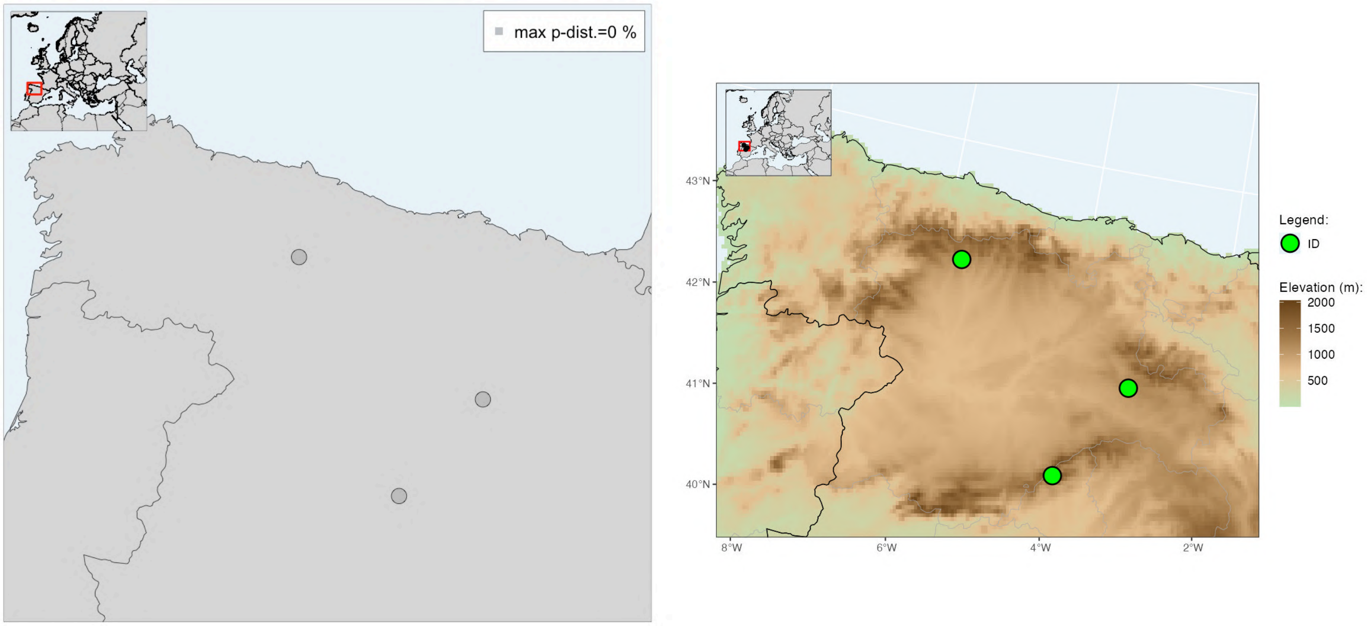

Figure 235: Genetic diversity map of *Formica dusmeti* Emery, 1909. PCoA projection was not done and therefore sequenced specimens in the genetic diversity map are coloured in gray (left). Specimen identification (ID or cf.) and source (newly sequenced or retrieved) are represented by colours, while specimen attribute (terra typica, type locality, type specimen or faunistic novelty) is represented by the shape (right). Sequences: ID = 3, cf. = 0; maximum p-distance: strict = 0 %, less strict = 0 %.

Haplotype network analysis of *Formica dusmeti* was not possible.

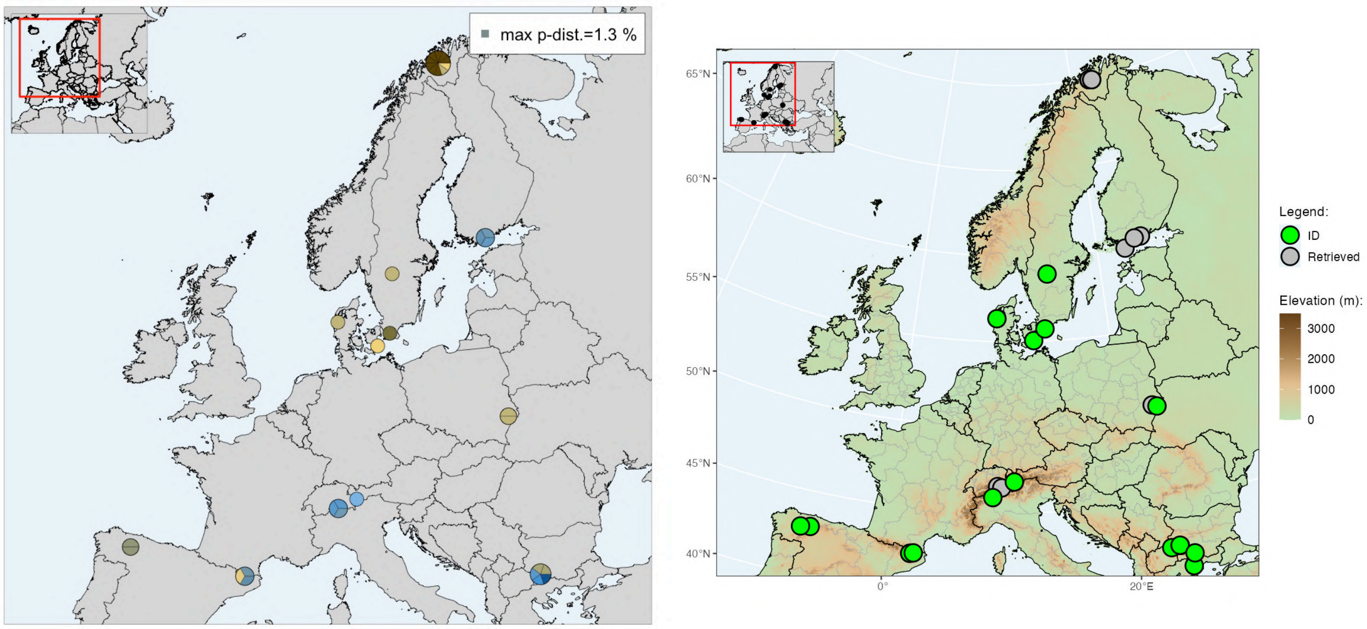

Figure 236: Genetic diversity map of *Formica exsecta* Nylander, 1846. Nearby localities of sequenced specimens are merged in pies (left). Colours match the bidimensional colour space of the PCoA projection (Fig. 236 left) of p-dist between sequences (dots). Specimen identification (ID or cf.) and source (newly sequenced or retrieved) are represented by colours, while specimen attribute (terra typica, type locality, type specimen or faunistic novelty) is represented by the shape (right). Sequences: ID = 33, cf. = 0; maximum p-distance: strict = 1.2 %, less strict = 1.3 %.

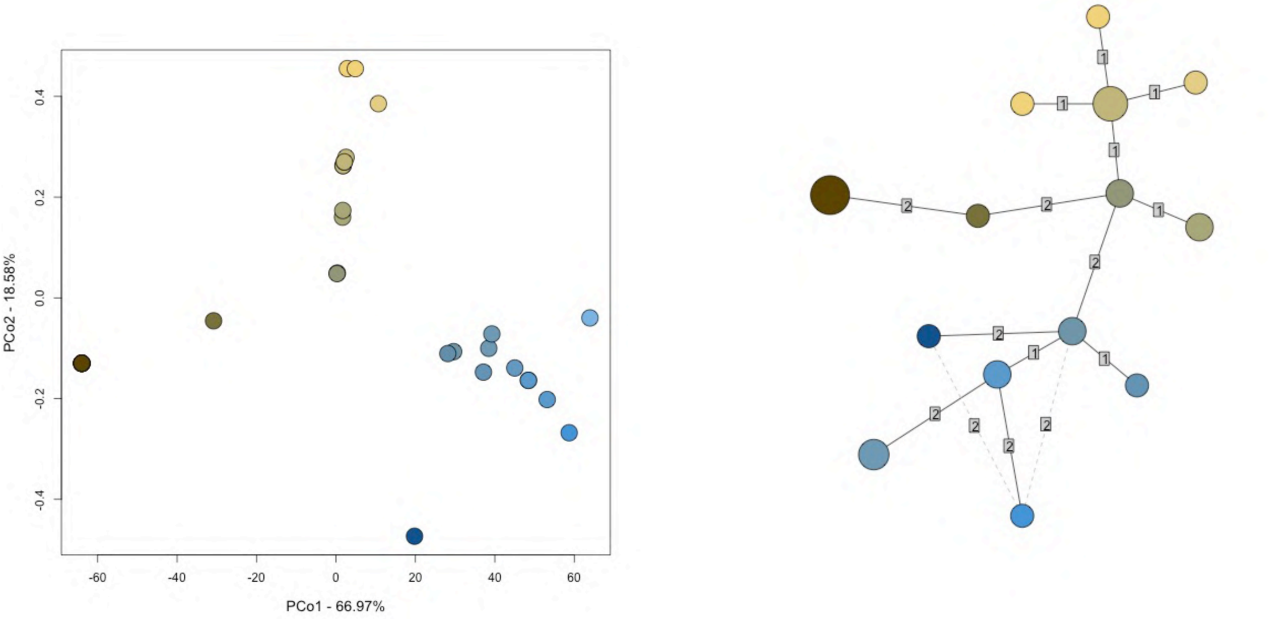

Figure 237: PCoA based on pairwise p-distances between *Formica exsecta* sequences (left). Colours match a bidimensional colour space. Haplotype network of *Formica exsecta* (right). Sequences > 599 bp: ID = 31, cf. = 0.

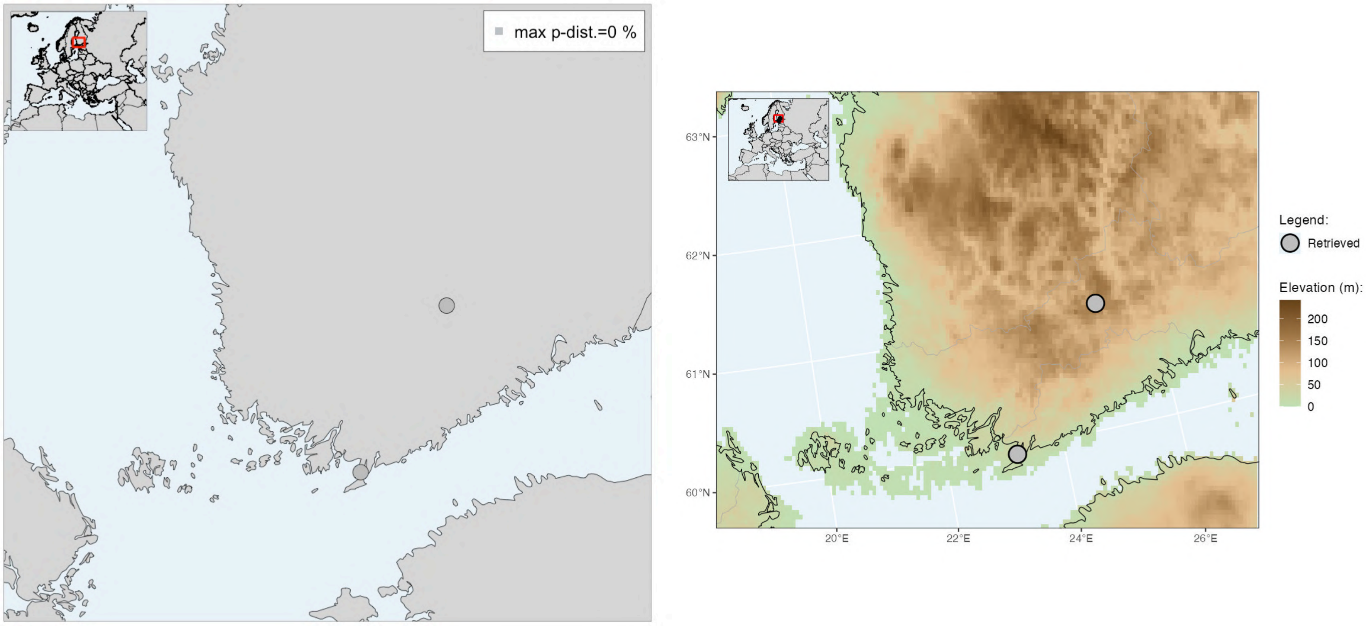

Figure 238: Genetic diversity map of *Formica fennica* Seifert, 2000. PCoA projection was not done and therefore sequenced specimens in the genetic diversity map are coloured in gray (left). Specimen identification (ID or cf.) and source (newly sequenced or retrieved) are represented by colours, while specimen attribute (terra typica, type locality, type specimen or faunistic novelty) is represented by the shape (right). Sequences: ID = 2, cf. = 0; maximum p-distance: strict = NA, less strict = 0 %.

Haplotype network analysis of *Formica fennica* was not possible.

Formica foreli

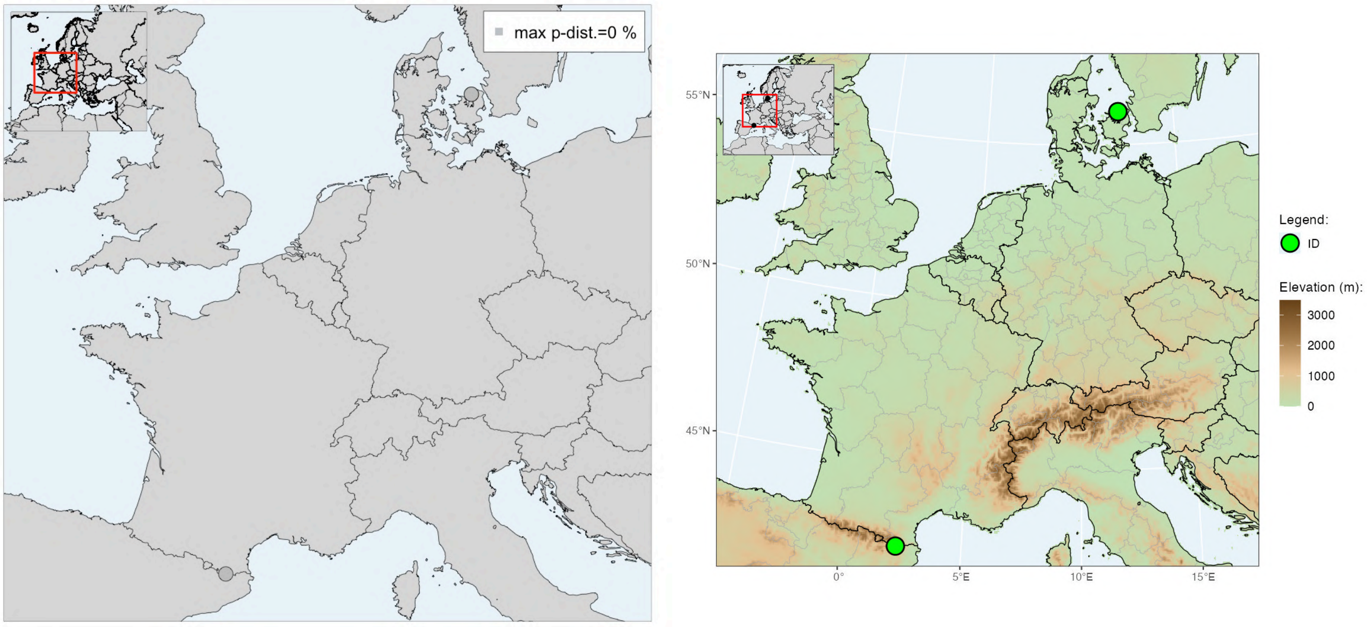

Figure 239: Genetic diversity map of *Formica foreli* Bondroit, 1918. PCoA projection was not done and therefore sequenced specimens in the genetic diversity map are coloured in gray (left). Specimen identification (ID or cf.) and source (newly sequenced or retrieved) are represented by colours, while specimen attribute (terra typica, type locality, type specimen or faunistic novelty) is represented by the shape (right). Sequences: ID = 3, cf. = 0; maximum p-distance: strict = 0 %, less strict = 0 %.

Haplotype network analysis of *Formica foreli* was not possible.

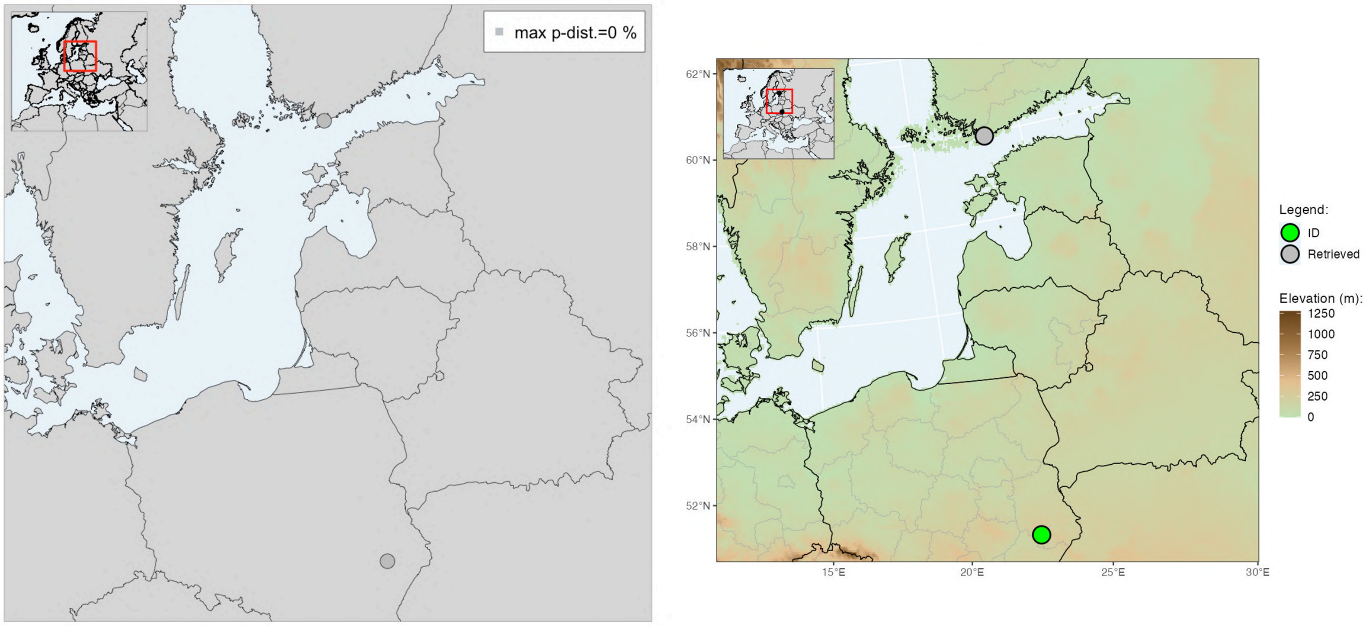

Figure 240: Genetic diversity map of *Formica forsslundi* Lohmander, 1949. PCoA projection was not done and therefore sequenced specimens in the genetic diversity map are coloured in gray (left). Specimen identification (ID or cf.) and source (newly sequenced or retrieved) are represented by colours, while specimen attribute (terra typica, type locality, type specimen or faunistic novelty) is represented by the shape (right). Sequences: ID = 2, cf. = 0; maximum p-distance: strict = NA, less strict = 0 %.

Haplotype network analysis of *Formica forsslundi* was not possible.

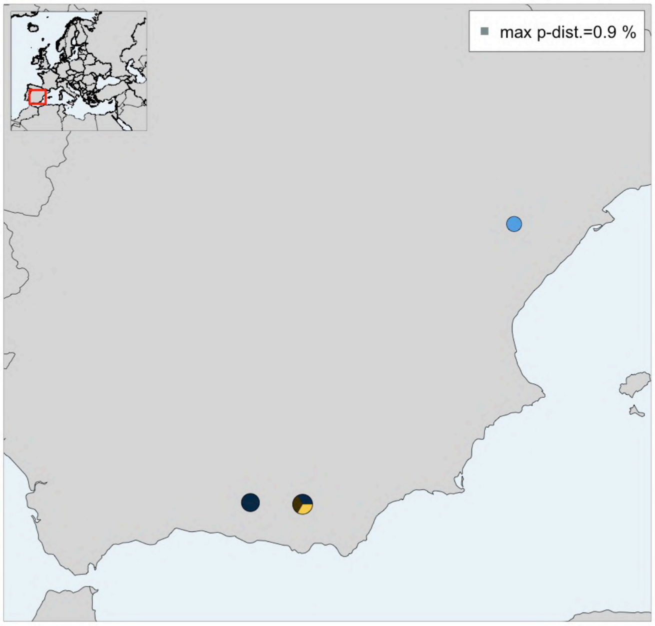

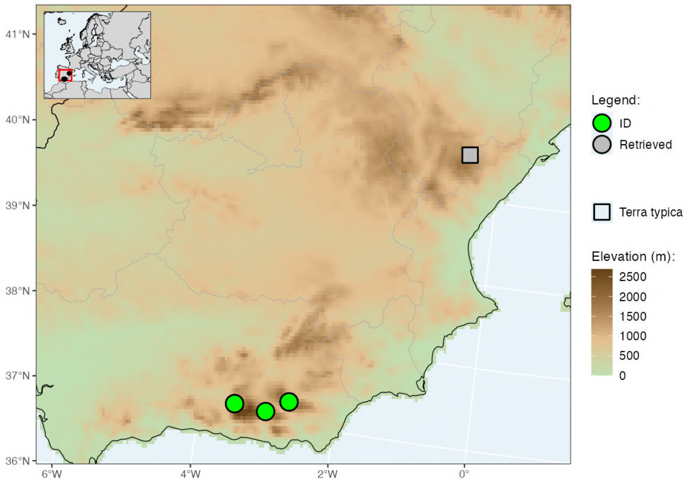

Figure 241: Genetic diversity map of *Formica frontalis* Santschi, 1919. Nearby localities of sequenced specimens are merged in pies (left). Colours match the bidimensional colour space of the PCoA projection (Fig. 241 left) of p-dist between sequences (dots). Specimen identification (ID or cf.) and source (newly sequenced or retrieved) are represented by colours, while specimen attribute (terra typica, type locality, type specimen or faunistic novelty) is represented by the shape (right). Sequences: ID = 6, cf. = 0; maximum p-distance: strict = 0.2 %, less strict = 0.9 %.

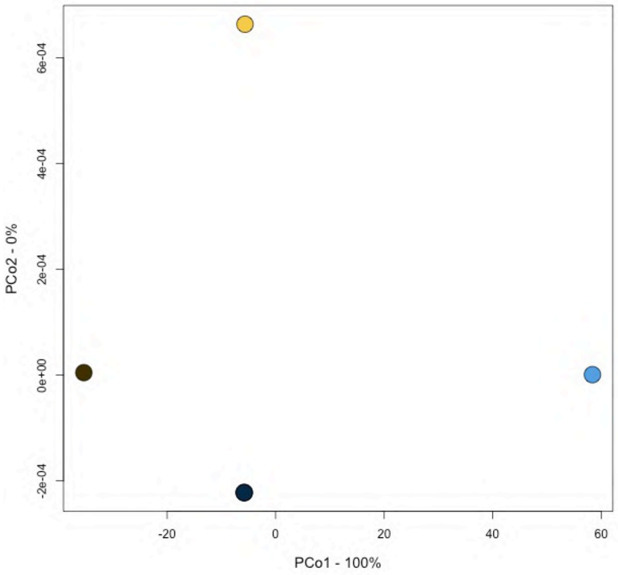

Figure 242: PCoA based on pairwise p-distances between *Formica frontalis* sequences (left). Colours match a bidimensional colour space. Haplotype network analysis of *Formica frontalis* was not possible.

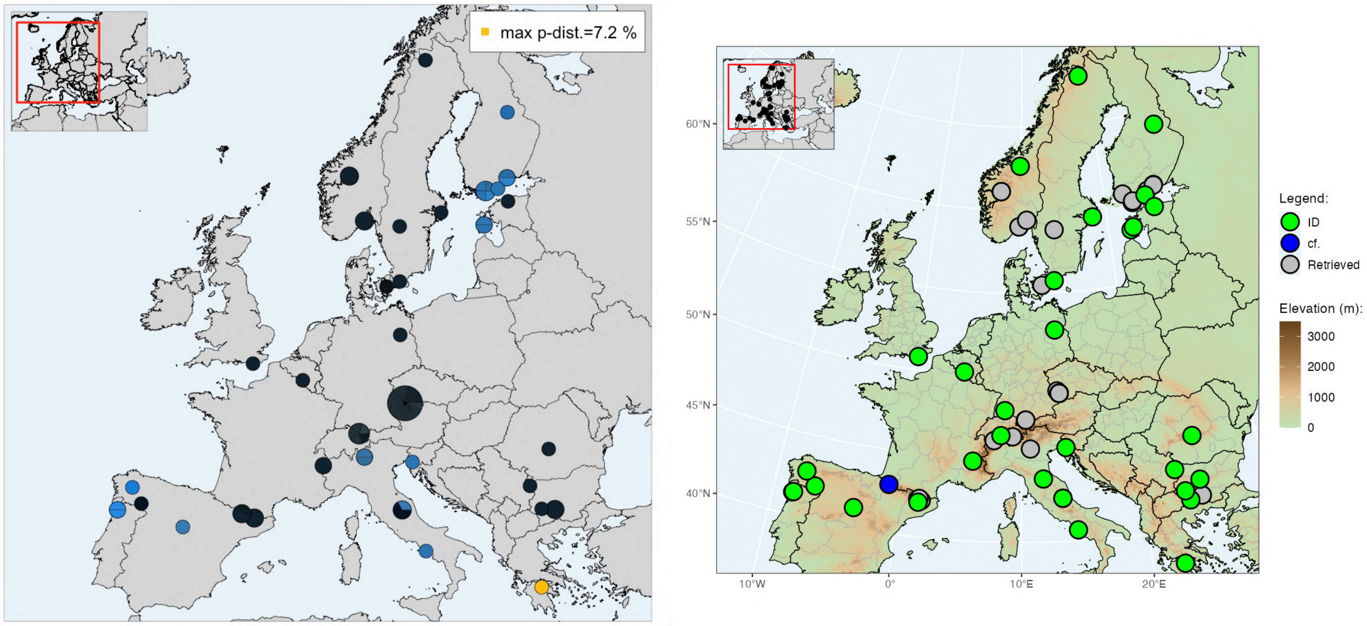

Figure 243: Genetic diversity map of *Formica fusca* Linnaeus, 1758. Nearby localities of sequenced specimens are merged in pies (left). Colours match the bidimensional colour space of the PCoA projection (Fig. 243 left) of p-dist between sequences (dots). Specimen identification (ID or cf.) and source (newly sequenced or retrieved) are represented by colours, while specimen attribute (terra typica, type locality, type specimen or faunistic novelty) is represented by the shape (right). Sequences: ID = 96, cf. = 1; maximum p-distance: strict = 6.9 %, less strict = 7.2 %.

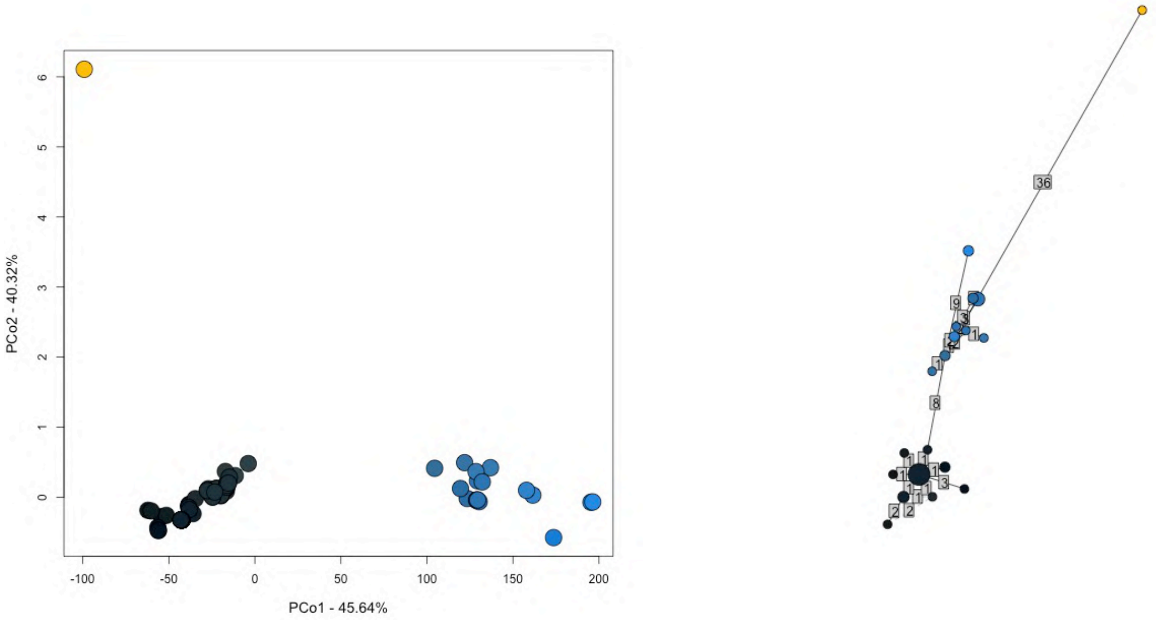

Figure 244: PCoA based on pairwise p-distances between *Formica fusca* sequences (left). Colours match a bidimensional colour space. Haplotype network of *Formica fusca* (right). Sequences > 599 bp: ID = 69, cf. = 1.

Formica fuscocinerea

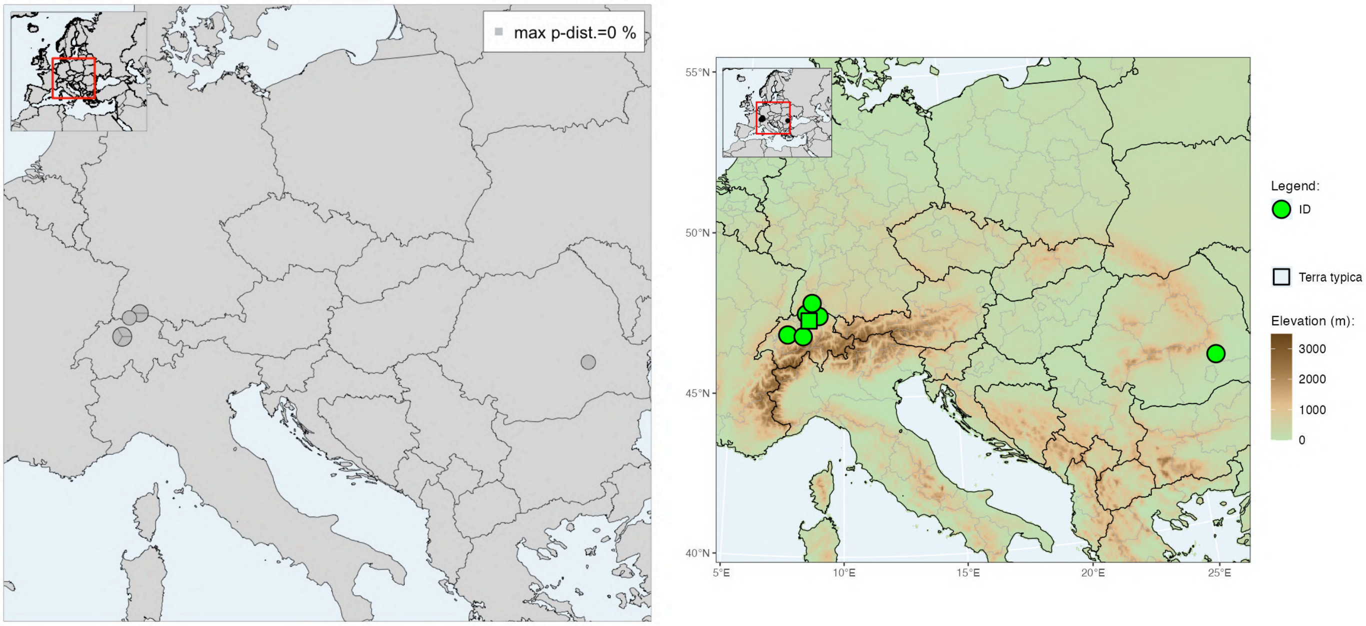

Figure 245: Genetic diversity map of *Formica fuscocinerea* Forel, 1874. PCoA projection was not done and therefore sequenced specimens in the genetic diversity map are coloured in gray (left). Specimen identification (ID or cf.) and source (newly sequenced or retrieved) are represented by colours, while specimen attribute (terra typica, type locality, type specimen or faunistic novelty) is represented by the shape (right). Sequences: ID = 7, cf. = 0; maximum p-distance: strict = 0 %, less strict = 0 %.

Haplotype network analysis of *Formica fuscocinerea* was not possible.

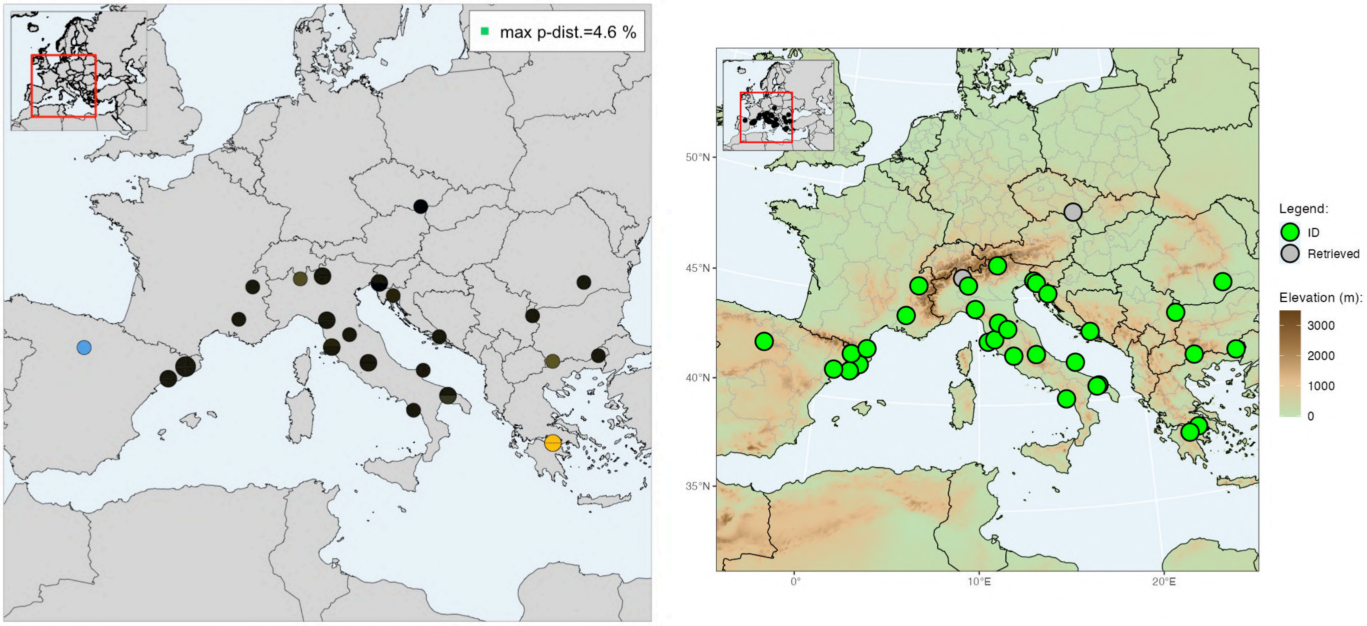

Figure 246: Genetic diversity map of *Formica gagates* Latreille, 1798. Nearby localities of sequenced specimens are merged in pies (left). Colours match the bidimensional colour space of the PCoA projection (Fig. 246 left) of p-dist between sequences (dots). Specimen identification (ID or cf.) and source (newly sequenced or retrieved) are represented by colours, while specimen attribute (terra typica, type locality, type specimen or faunistic novelty) is represented by the shape (right). Sequences: ID = 35, cf. = 0; maximum p-distance: strict = 4.6 %, less strict = 4.6 %.

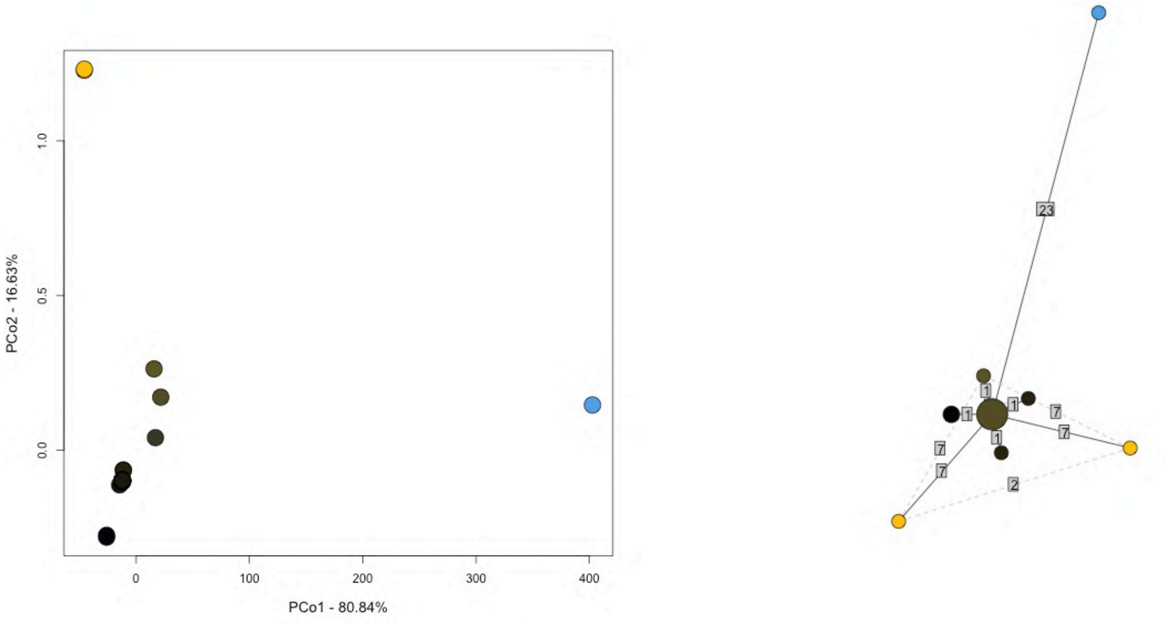

Figure 247: PCoA based on pairwise p-distances between *Formica gagates* sequences (left). Colours match a bidimensional colour space. Haplotype network of *Formica gagates* (right). Sequences > 599 bp: ID = 34, cf. = 0.

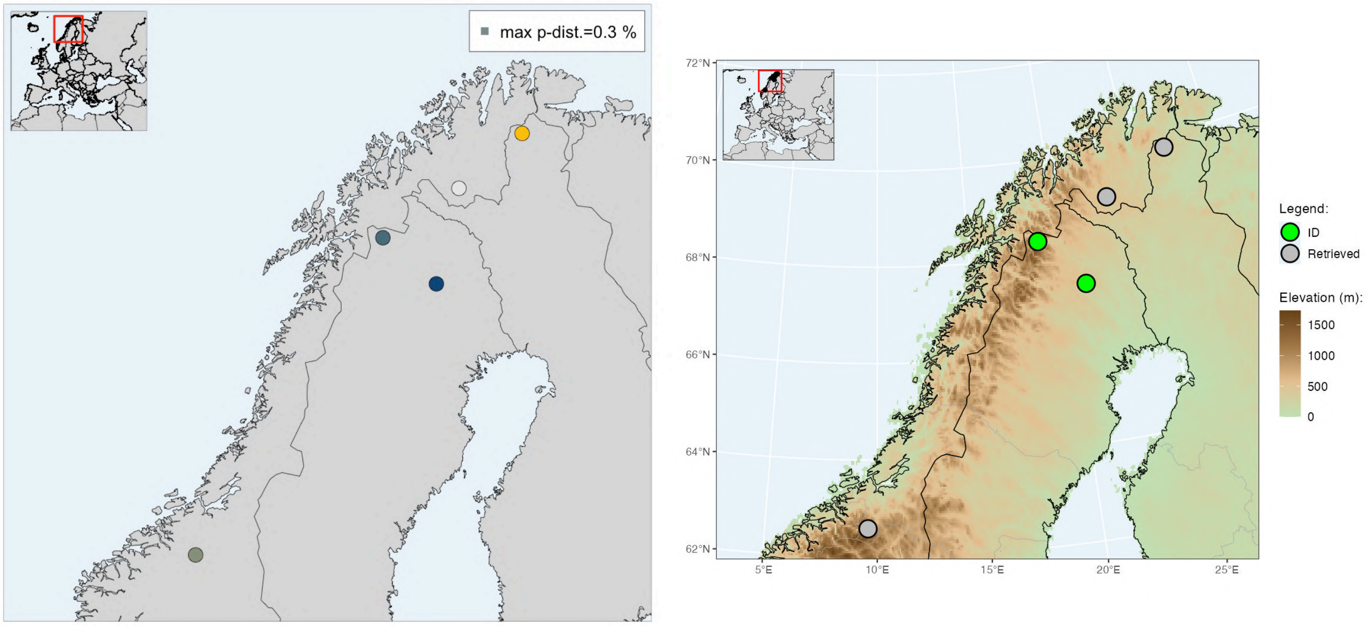

Figure 248: Genetic diversity map of *Formica gagatoides* Ruzsky, 1904. Nearby localities of sequenced specimens are merged in pies (left). Colours match the bidimensional colour space of the PCoA projection (Fig. 248 left) of p-dist between sequences (dots). Specimen identification (ID or cf.) and source (newly sequenced or retrieved) are represented by colours, while specimen attribute (terra typica, type locality, type specimen or faunistic novelty) is represented by the shape (right). Sequences: ID = 5, cf. = 0; maximum p-distance: strict = 0.3 %, less strict = 0.3 %.

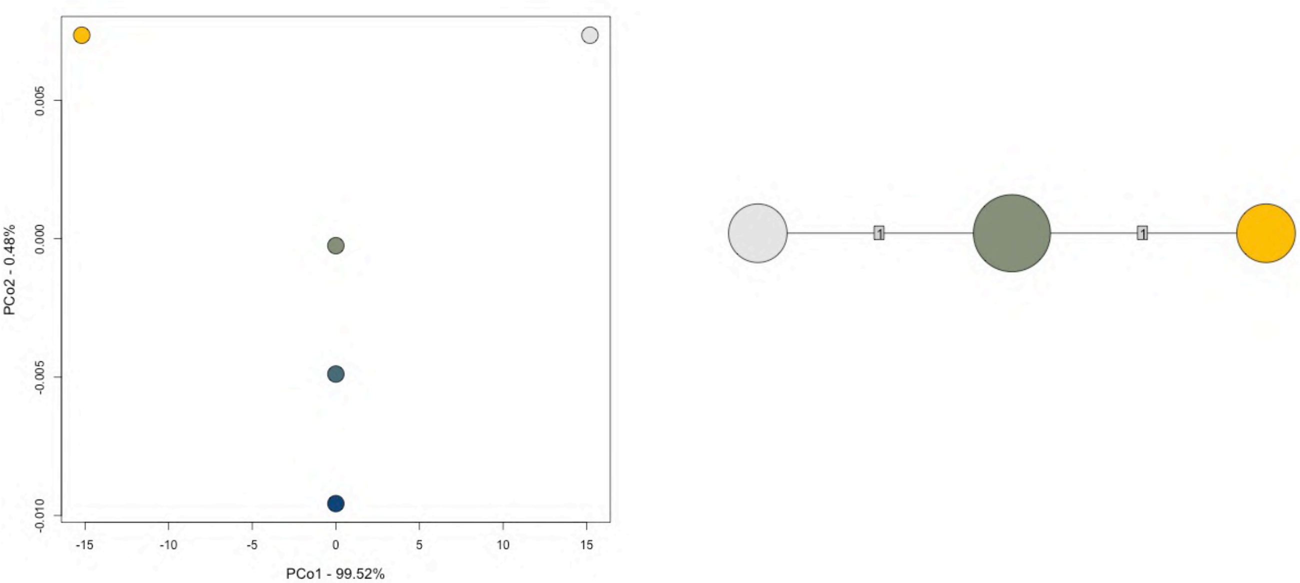

Figure 249: PCoA based on pairwise p-distances between *Formica gagatoides* sequences (left). Colours match a bidimensional colour space. Haplotype network of *Formica gagatoides* (right). Sequences > 599 bp: ID = 5, cf. = 0.

Formica gerardi

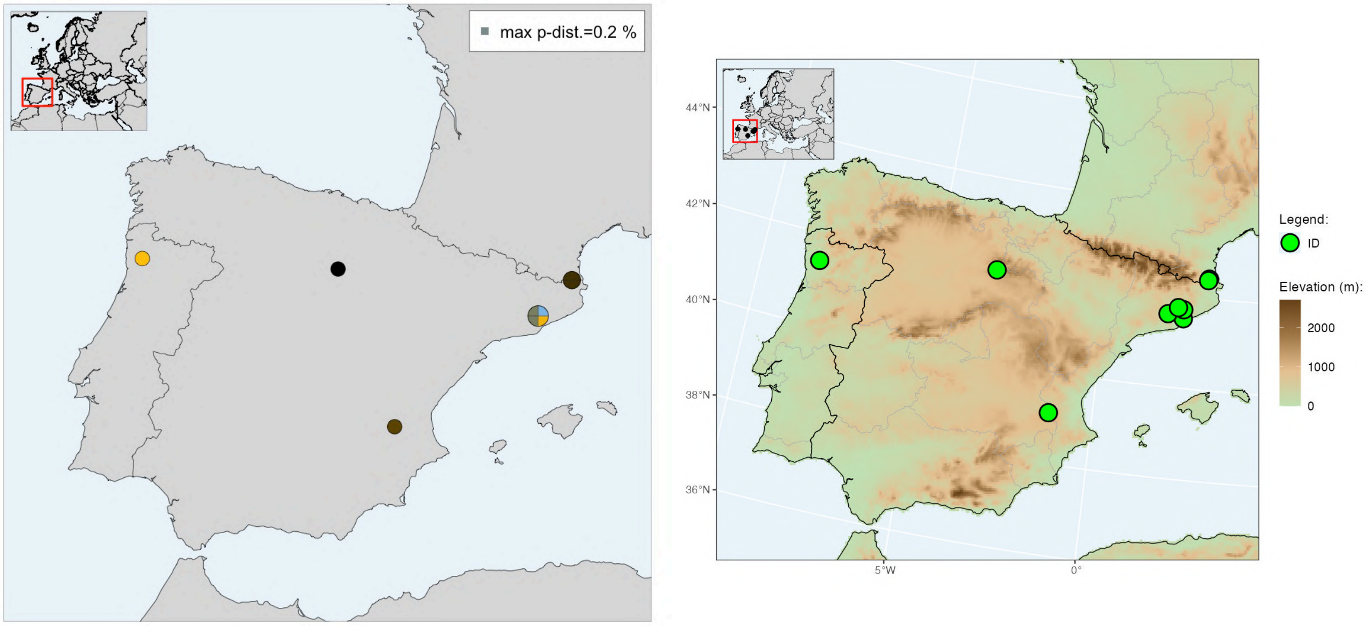

Figure 250: Genetic diversity map of *Formica gerardi* Bondroit, 1917. Nearby localities of sequenced specimens are merged in pies (left). Colours match the bidimensional colour space of the PCoA projection (Fig. 250 left) of p-dist between sequences (dots). Specimen identification (ID or cf.) and source (newly sequenced or retrieved) are represented by colours, while specimen attribute (terra typica, type locality, type specimen or faunistic novelty) is represented by the shape (right). Sequences: ID = 9, cf. = 0; maximum p-distance: strict = 0.2 %, less strict = 0.2 %.

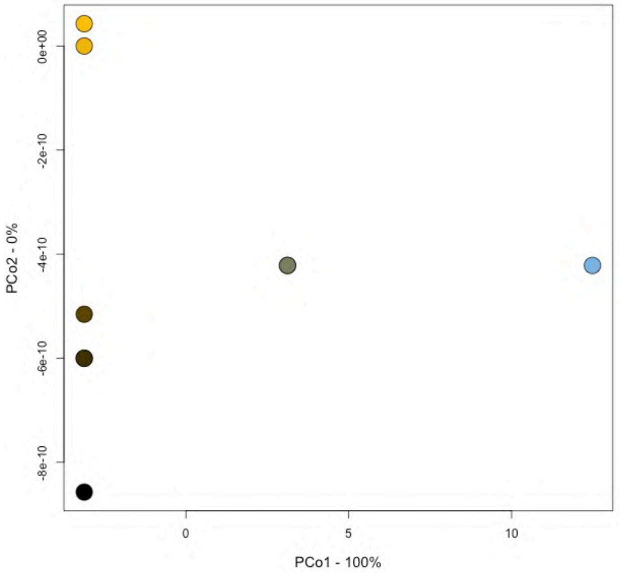

Figure 251: PCoA based on pairwise p-distances between *Formica gerardi* sequences (left). Colours match a bidimensional colour space. Haplotype network analysis of *Formica gerardi* was not possible.

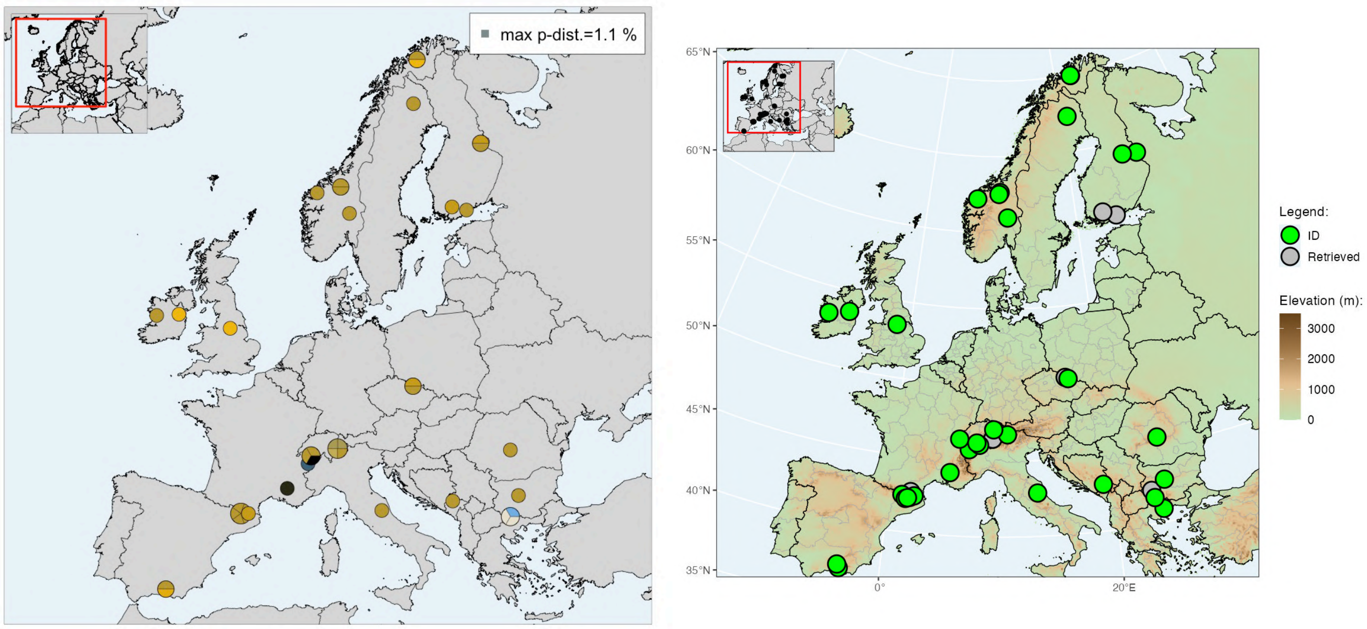

Figure 252: Genetic diversity map of *Formica lemani* Bondroit, 1917. Nearby localities of sequenced specimens are merged in pies (left). Colours match the bidimensional colour space of the PCoA projection (Fig. 252 left) of p-dist between sequences (dots). Specimen identification (ID or cf.) and source (newly sequenced or retrieved) are represented by colours, while specimen attribute (terra typica, type locality, type specimen or faunistic novelty) is represented by the shape (right). Sequences: ID = 40, cf. = 0; maximum p-distance: strict = 1.1 %, less strict = 1.1 %.

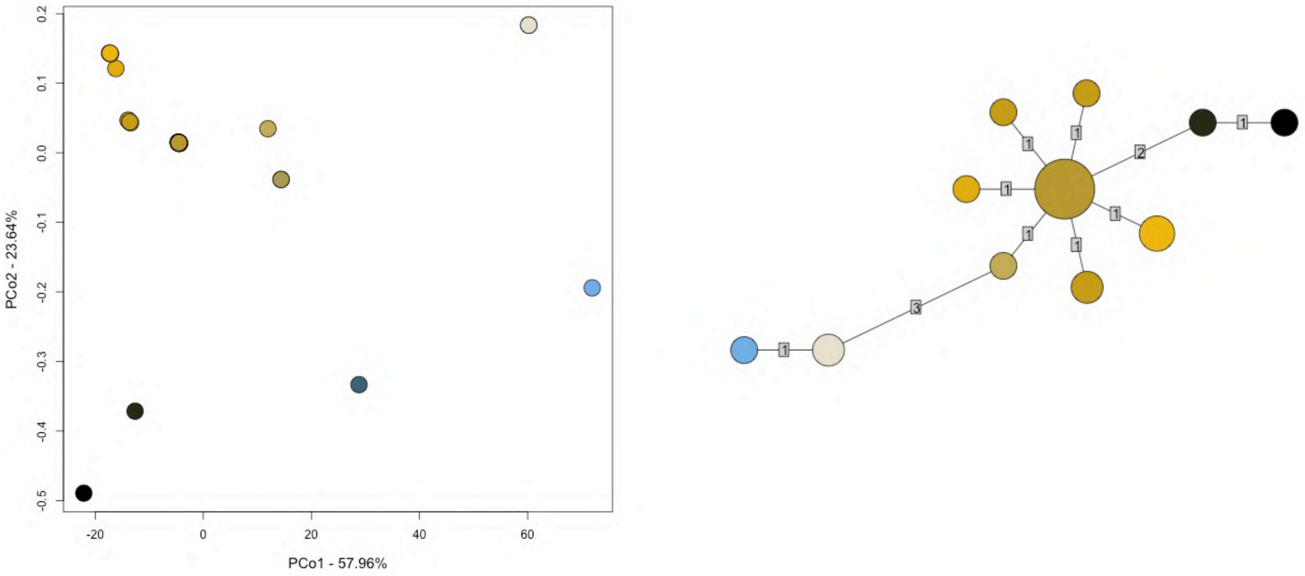

Figure 253: PCoA based on pairwise p-distances between *Formica lemani* sequences (left). Colours match a bidimensional colour space. Haplotype network of *Formica lemani* (right). Sequences > 599 bp: ID = 39, cf. = 0.

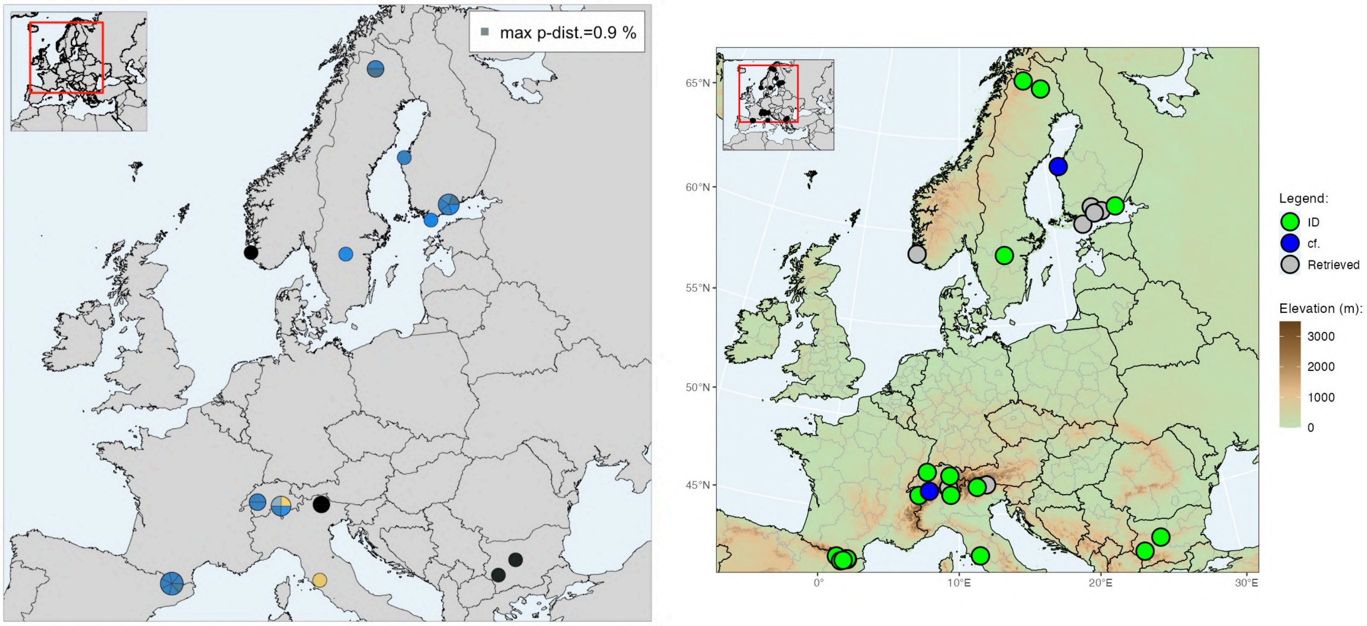

Figure 254: Genetic diversity map of *Formica lugubris* Zetterstedt, 1838. Nearby localities of sequenced specimens are merged in pies (left). Colours match the bidimensional colour space of the PCoA projection (Fig. 254 left) of p-dist between sequences (dots). Specimen identification (ID or cf.) and source (newly sequenced or retrieved) are represented by colours, while specimen attribute (terra typica, type locality, type specimen or faunistic novelty) is represented by the shape (right). Sequences: ID = 28, cf. = 2; maximum p-distance: strict = 0.9 %, less strict = 0.9 %.

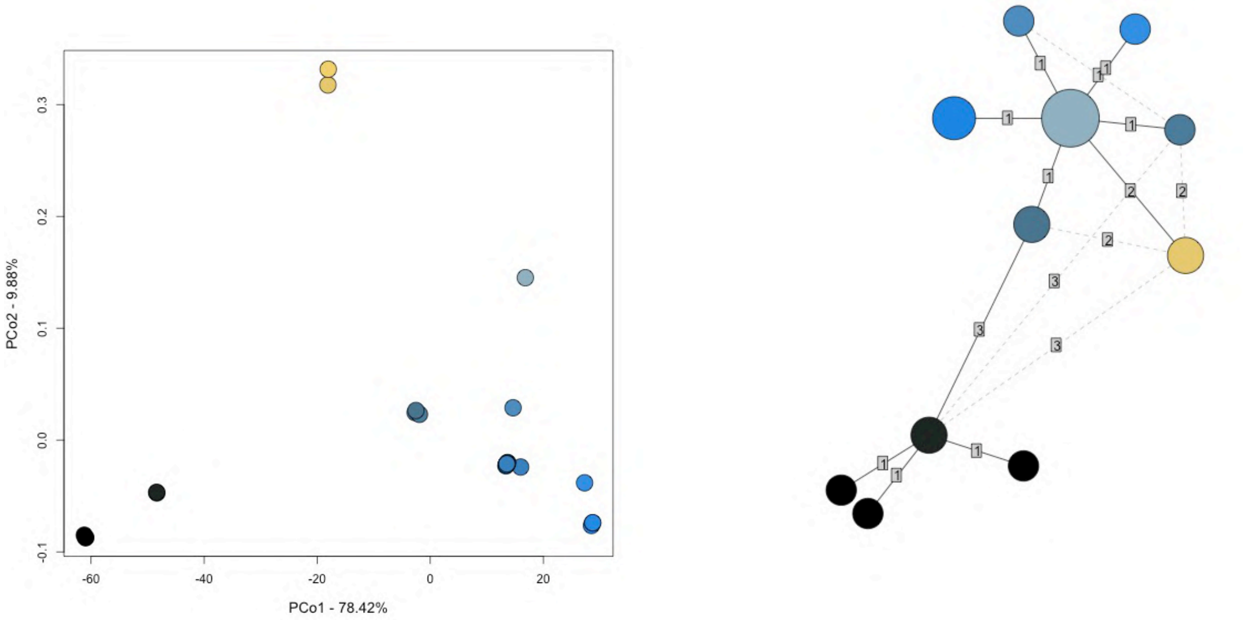

Figure 255: PCoA based on pairwise p-distances between *Formica lugubris* sequences (left). Colours match a bidimensional colour space. Haplotype network of *Formica lugubris* (right). Sequences > 599 bp: ID = 28, cf. = 2.

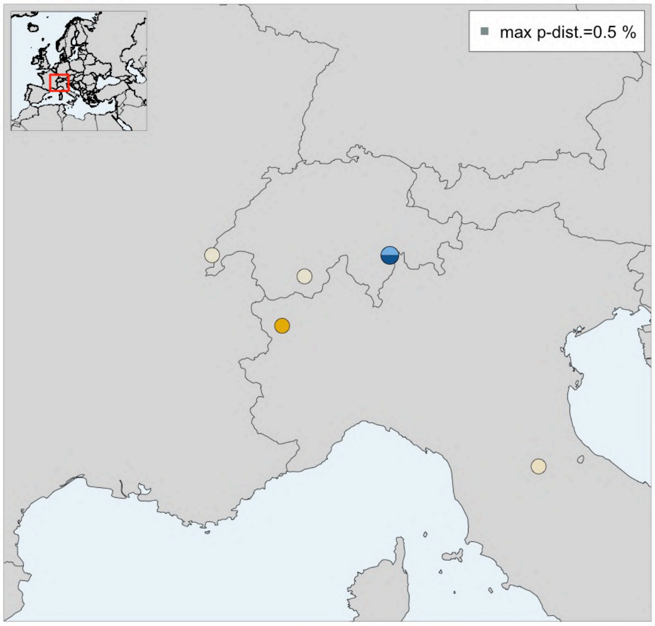

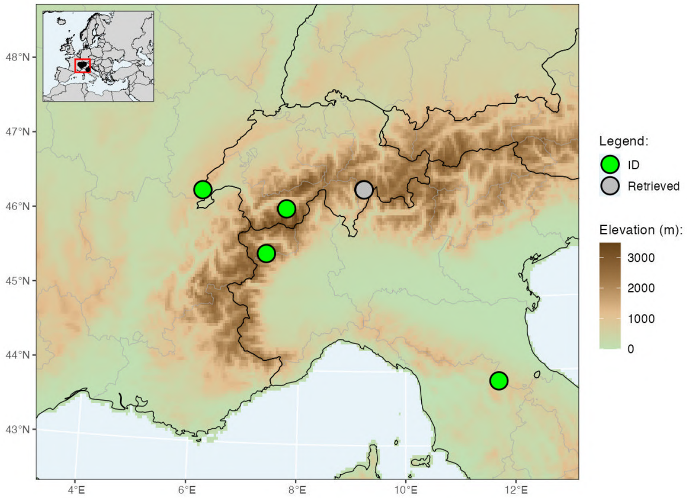

Figure 256: Genetic diversity map of *Formica paralugubris* Seifert, 1996. Nearby localities of sequenced specimens are merged in pies (left). Colours match the bidimensional colour space of the PCoA projection (Fig. 256 left) of p-dist between sequences (dots). Specimen identification (ID or cf.) and source (newly sequenced or retrieved) are represented by colours, while specimen attribute (terra typica, type locality, type specimen or faunistic novelty) is represented by the shape (right). Sequences: ID = 6, cf. = 0; maximum p-distance: strict = 0.5 %, less strict = 0.5 %.

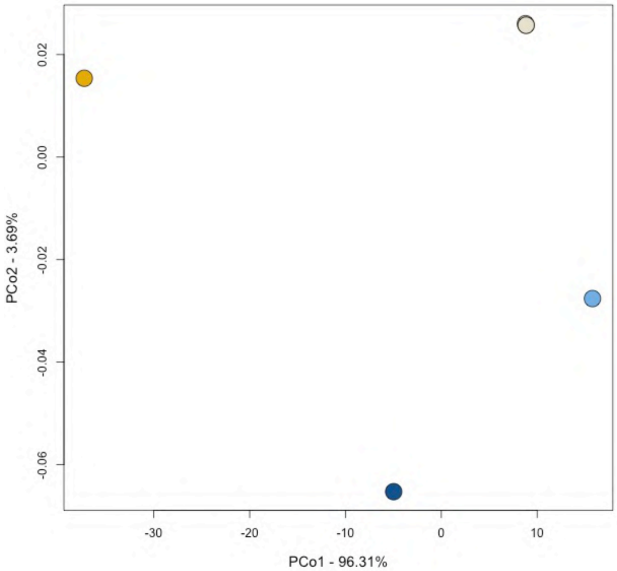

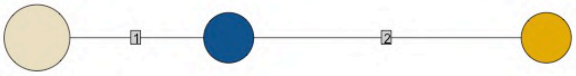

Figure 257: PCoA based on pairwise p-distances between *Formica paralugubris* sequences (left). Colours match a bidimensional colour space. Haplotype network of *Formica paralugubris* (right). Sequences > 599 bp: ID = 5, cf. = 0.

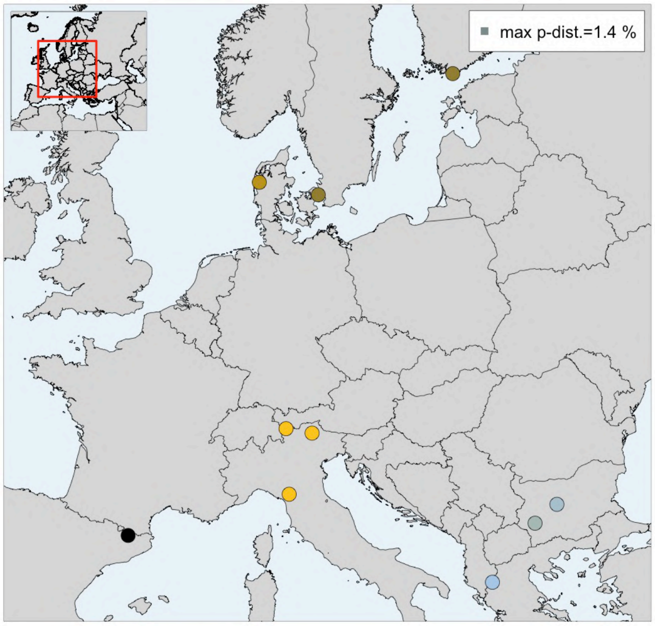

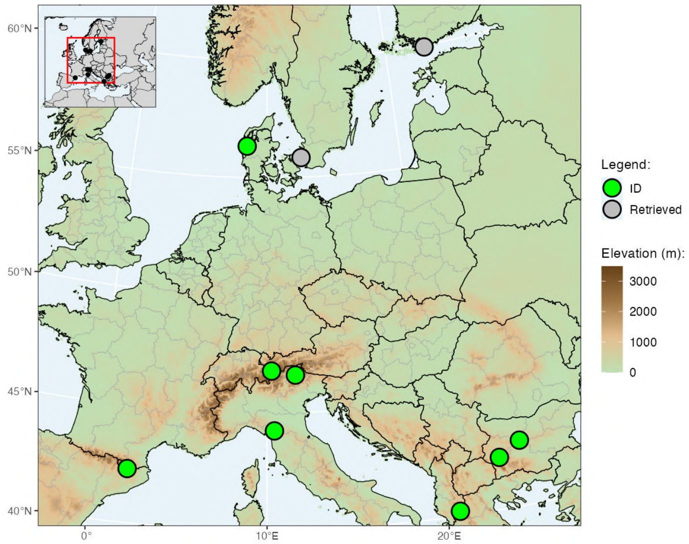

Figure 258: Genetic diversity map of *Formica picea* Nylander, 1846. Nearby localities of sequenced specimens are merged in pies (left). Colours match the bidimensional colour space of the PCoA projection (Fig. 258 left) of p-dist between sequences (dots). Specimen identification (ID or cf.) and source (newly sequenced or retrieved) are represented by colours, while specimen attribute (terra typica, type locality, type specimen or faunistic novelty) is represented by the shape (right). Sequences: ID = 10, cf. = 0; maximum p-distance: strict = 1.4 %, less strict = 1.4 %.

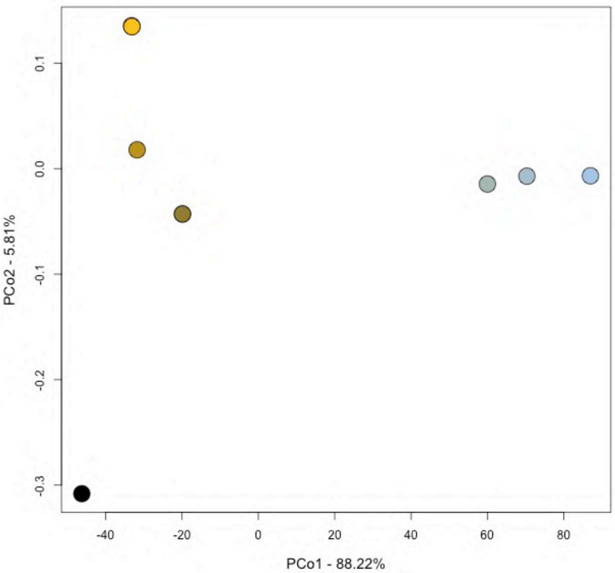

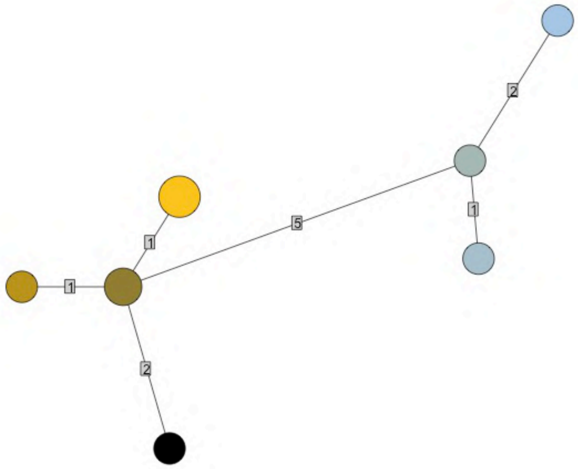

Figure 259: PCoA based on pairwise p-distances between *Formica picea* sequences (left). Colours match a bidimensional colour space. Haplotype network of *Formica picea* (right). Sequences > 599 bp: ID = 10, cf. = 0.

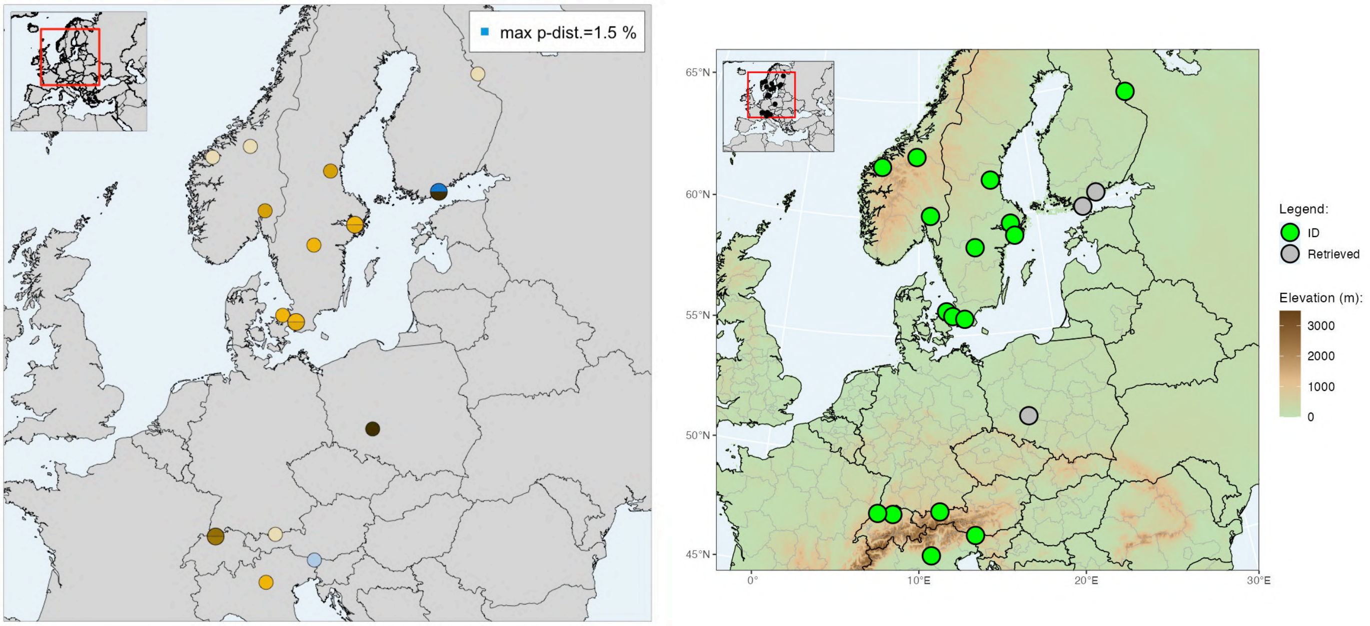

Figure 260: Genetic diversity map of *Formica polyctena* Foerster, 1850. Nearby localities of sequenced specimens are merged in pies (left). Colours match the bidimensional colour space of the PCoA projection (Fig. 260 left) of p-dist between sequences (dots). Specimen identification (ID or cf.) and source (newly sequenced or retrieved) are represented by colours, while specimen attribute (terra typica, type locality, type specimen or faunistic novelty) is represented by the shape (right). Sequences: ID = 19, cf. = 0; maximum p-distance: strict = 1.5 %, less strict = 1.5 %.

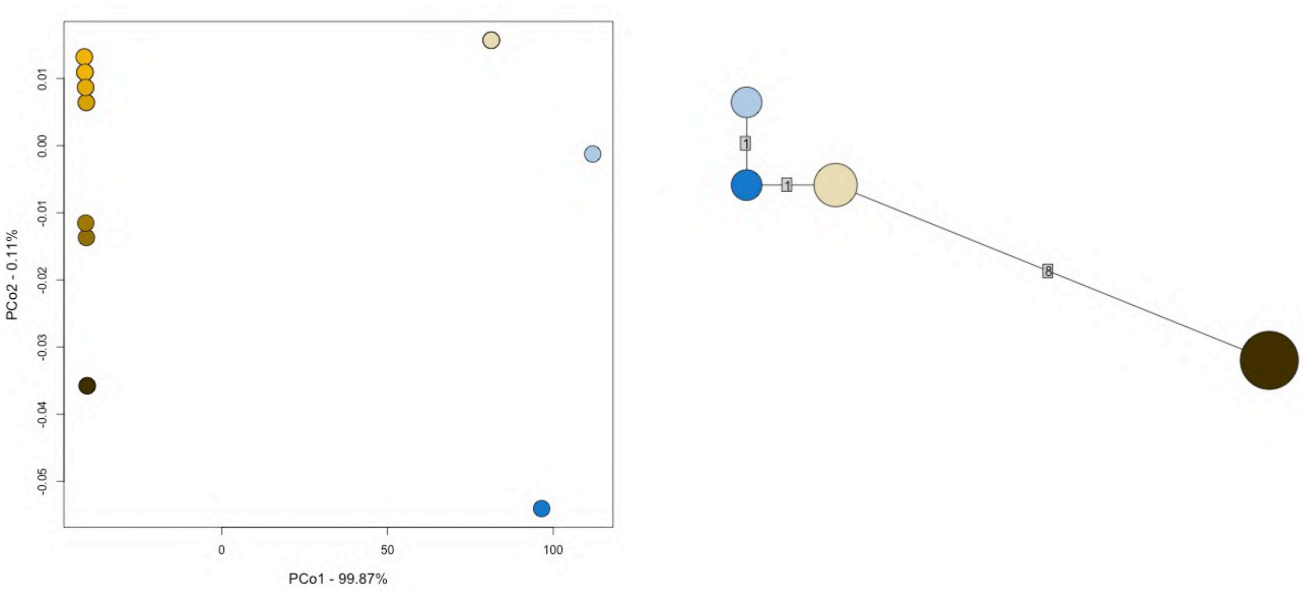

Figure 261: PCoA based on pairwise p-distances between *Formica polyctena* sequences (left). Colours match a bidimensional colour space. Haplotype network of *Formica polyctena* (right). Sequences > 599 bp: ID = 19, cf. = 0.

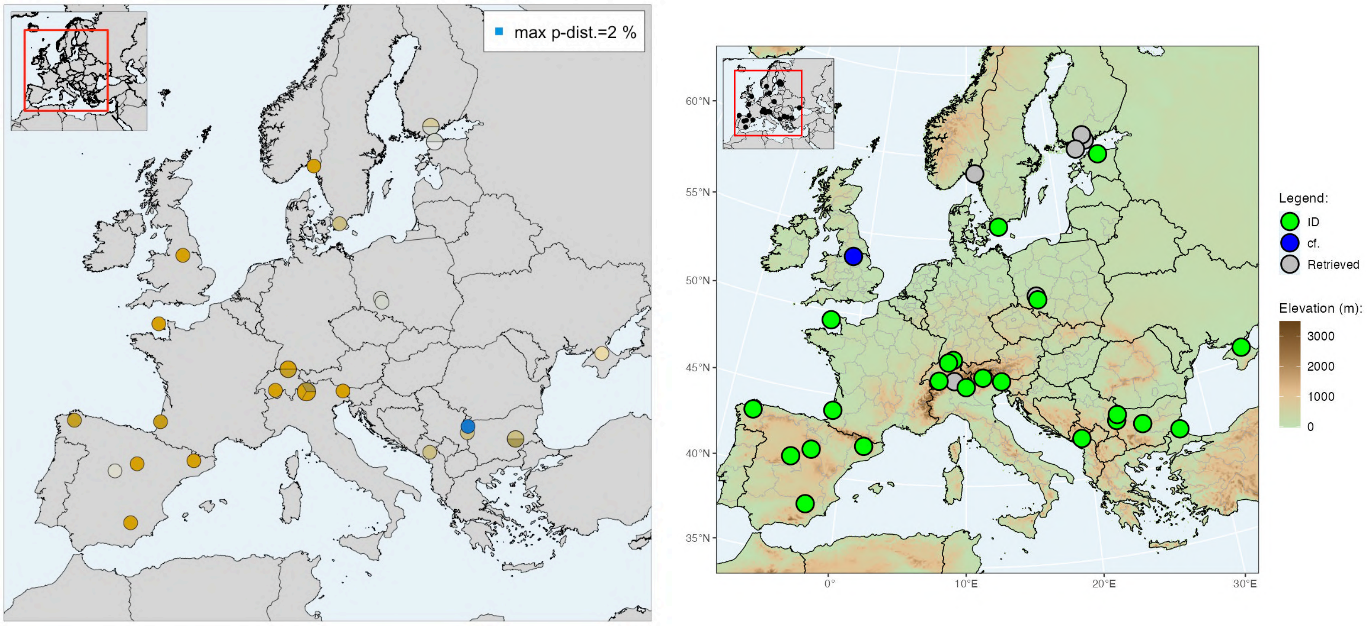

Figure 262: Genetic diversity map of *Formica pratensis* Retzius, 1783. Nearby localities of sequenced specimens are merged in pies (left). Colours match the bidimensional colour space of the PCoA projection (Fig. 262 left) of p-dist between sequences (dots). Specimen identification (ID or cf.) and source (newly sequenced or retrieved) are represented by colours, while specimen attribute (terra typica, type locality, type specimen or faunistic novelty) is represented by the shape (right). Sequences: ID = 28, cf. = 1; maximum p-distance: strict = 2 %, less strict = 2 %.

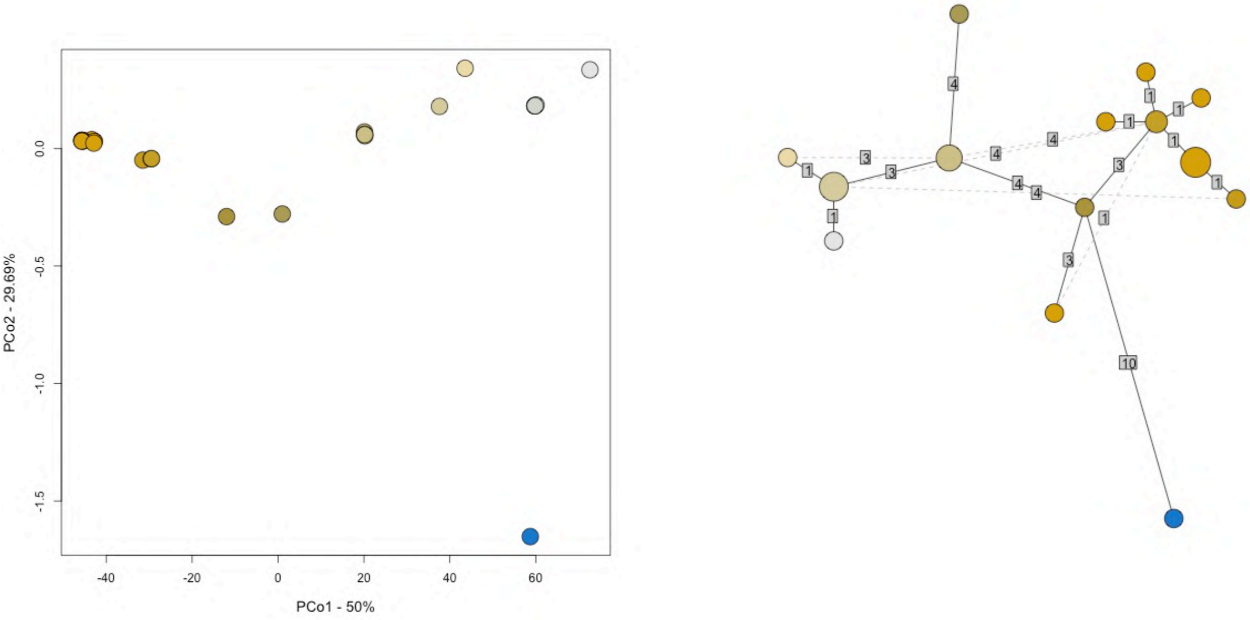

Figure 263: PCoA based on pairwise p-distances between *Formica pratensis* sequences (left). Colours match a bidimensional colour space. Haplotype network of *Formica pratensis* (right). Sequences > 599 bp: ID = 28, cf. = 1.

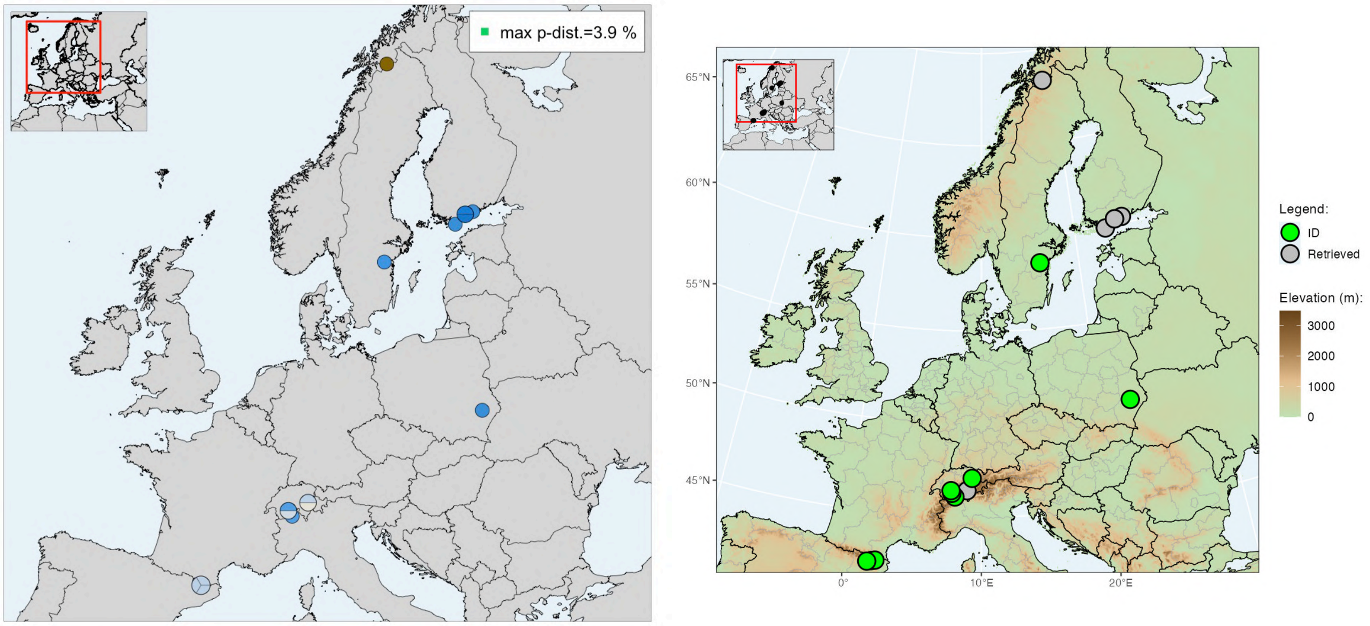

Figure 264: Genetic diversity map of *Formica pressilabris* Nylander, 1846. Nearby localities of sequenced specimens are merged in pies (left). Colours match the bidimensional colour space of the PCoA projection (Fig. 264 left) of p-dist between sequences (dots). Specimen identification (ID or cf.) and source (newly sequenced or retrieved) are represented by colours, while specimen attribute (terra typica, type locality, type specimen or faunistic novelty) is represented by the shape (right). Sequences: ID = 15, cf. = 0; maximum p-distance: strict = 3.9 %, less strict = 3.9 %.

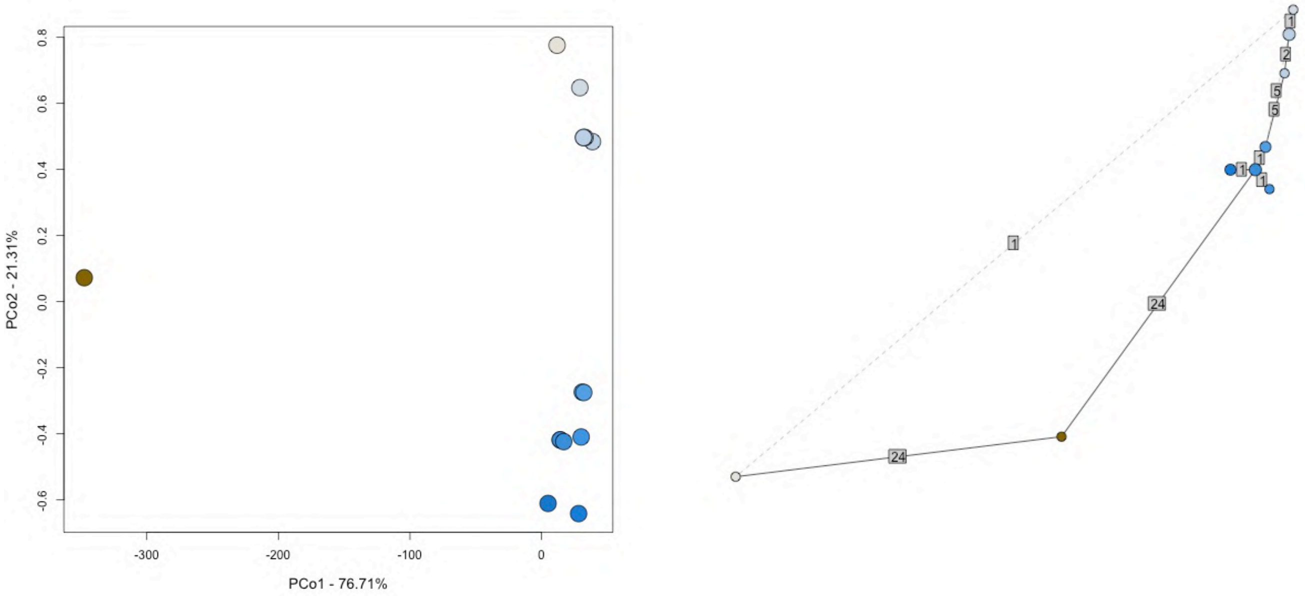

Figure 265: PCoA based on pairwise p-distances between *Formica pressilabris* sequences (left). Colours match a bidimensional colour space. Haplotype network of *Formica pressilabris* (right). Sequences > 599 bp: ID = 15, cf. = 0.

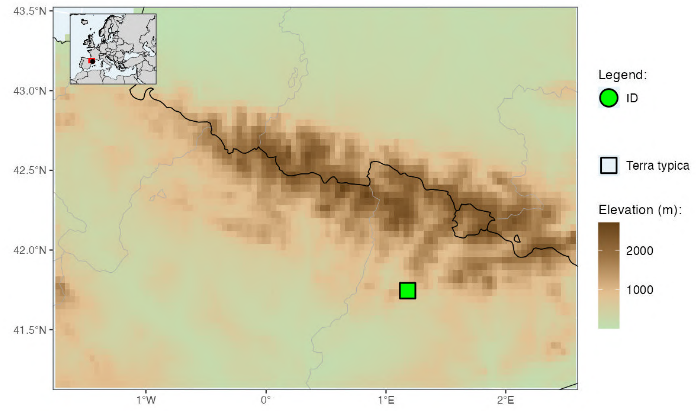

Figure 266: Map of *Formica pyrenaea* Bondroit, 1918. Due to the presence of a single sequence, the genetic diversity map and the PCoA projection were not done. Specimen identification (ID or cf.) and source (newly sequenced or retrieved) are represented by colours, while specimen attribute (terra typica, type locality, type specimen or faunistic novelty) is represented by the shape. Sequences: ID = 1, cf. = 0; maximum p-distance: strict = NA, less strict = NA.

Haplotype network analysis of *Formica pyrenaea* was not possible.

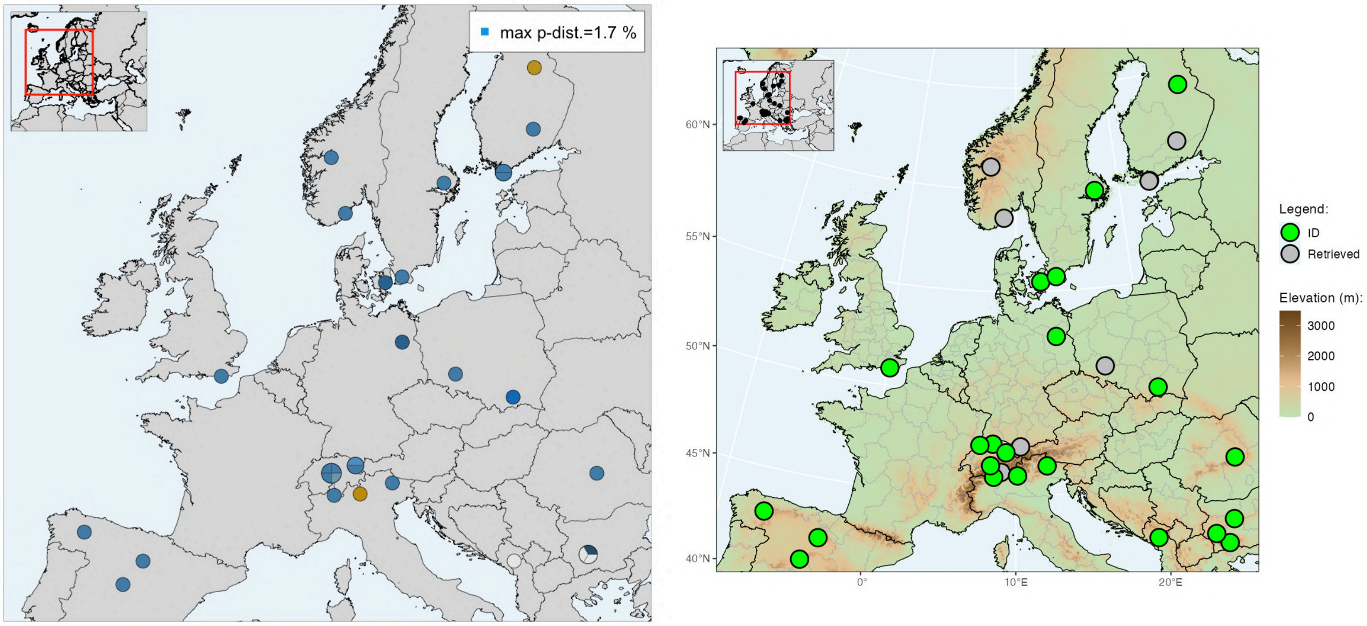

Figure 267: Genetic diversity map of *Formica rufa* Linnaeus, 1761. Nearby localities of sequenced specimens are merged in pies (left). Colours match the bidimensional colour space of the PCoA projection (Fig. 267 left) of p-dist between sequences (dots). Specimen identification (ID or cf.) and source (newly sequenced or retrieved) are represented by colours, while specimen attribute (terra typica, type locality, type specimen or faunistic novelty) is represented by the shape (right). Sequences: ID = 30, cf. = 0; maximum p-distance: strict = 1.7 %, less strict = 1.7 %.

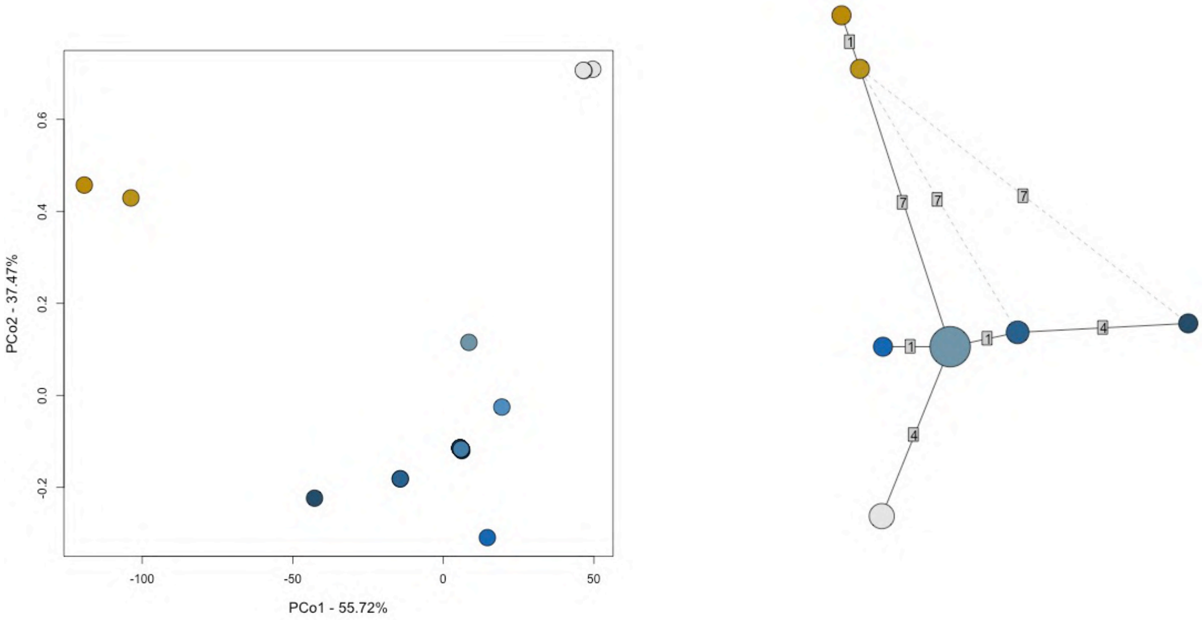

Figure 268: PCoA based on pairwise p-distances between *Formica rufa* sequences (left). Colours match a bidimensional colour space. Haplotype network of *Formica rufa* (right). Sequences > 599 bp: ID = 29, cf. = 0.

Formica rufibarbis

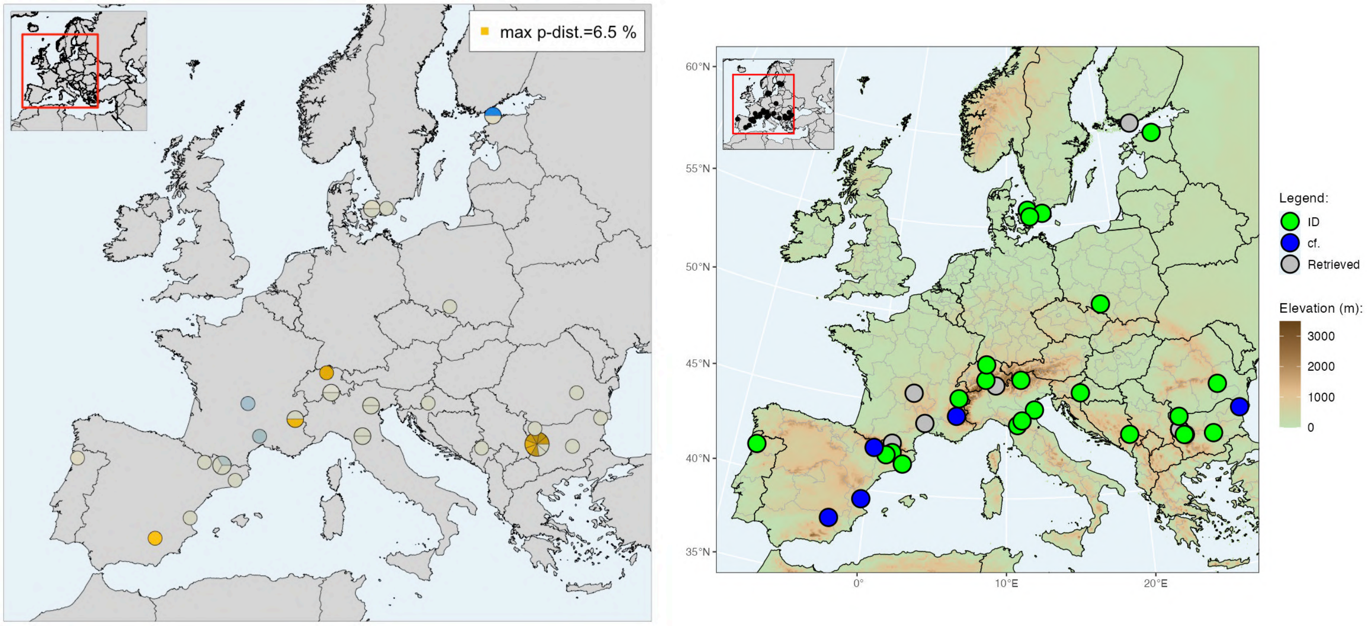

Figure 269: Genetic diversity map of *Formica rufibarbis* Fabricius, 1793. Nearby localities of sequenced specimens are merged in pies (left). Colours match the bidimensional colour space of the PCoA projection (Fig. 269 left) of p-dist between sequences (dots). Specimen identification (ID or cf.) and source (newly sequenced or retrieved) are represented by colours, while specimen attribute (terra typica, type locality, type specimen or faunistic novelty) is represented by the shape (right). Sequences: ID = 35, cf. = 5; maximum p-distance: strict = 4.6 %, less strict = 6.5 %.

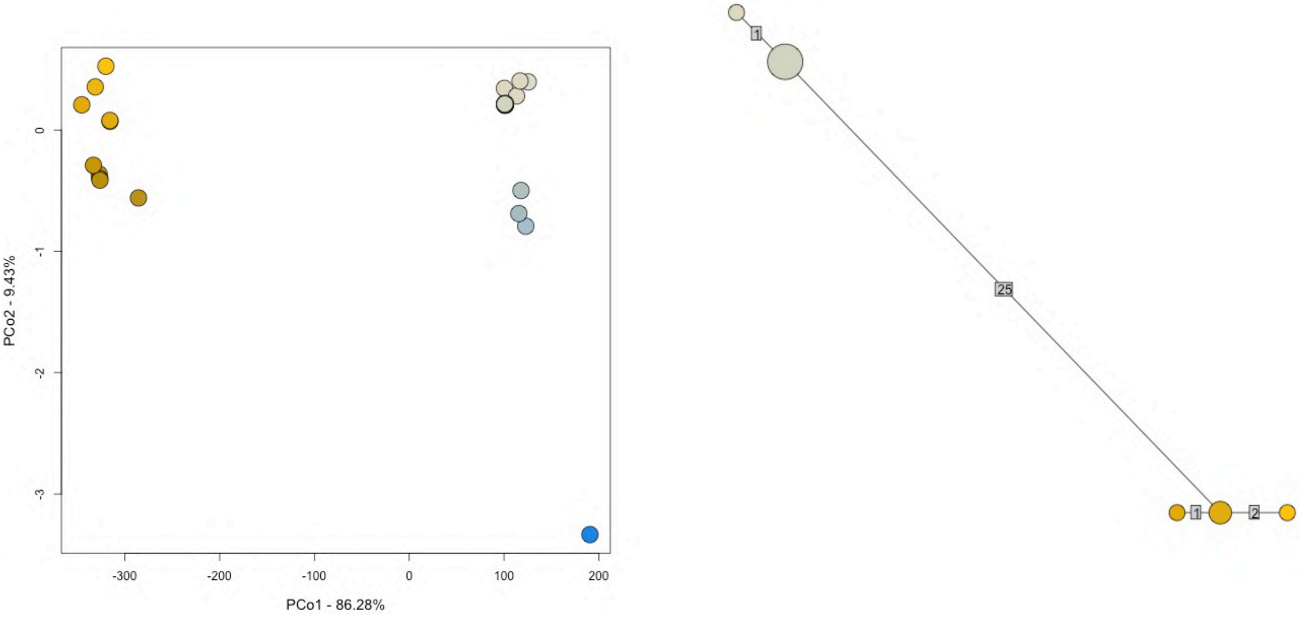

Figure 270: PCoA based on pairwise p-distances between *Formica rufibarbis* sequences (left). Colours match a bidimensional colour space. Haplotype network of *Formica rufibarbis* (right). Sequences > 599 bp: ID = 26, cf. = 5.

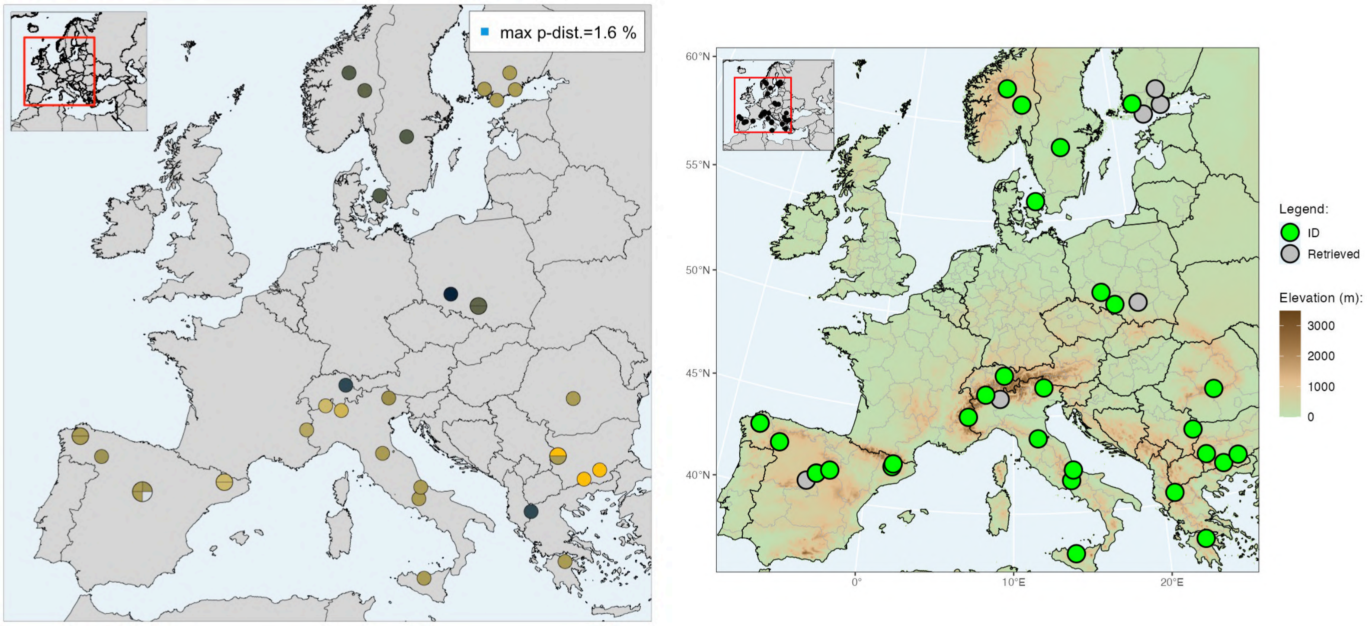

Figure 271: Genetic diversity map of *Formica sanguinea* Latreille, 1798. Nearby localities of sequenced specimens are merged in pies (left). Colours match the bidimensional colour space of the PCoA projection (Fig. 271 left) of p-dist between sequences (dots). Specimen identification (ID or cf.) and source (newly sequenced or retrieved) are represented by colours, while specimen attribute (terra typica, type locality, type specimen or faunistic novelty) is represented by the shape (right). Sequences: ID = 36, cf. = 0; maximum p-distance: strict = 0.8 %, less strict = 1.6 %.

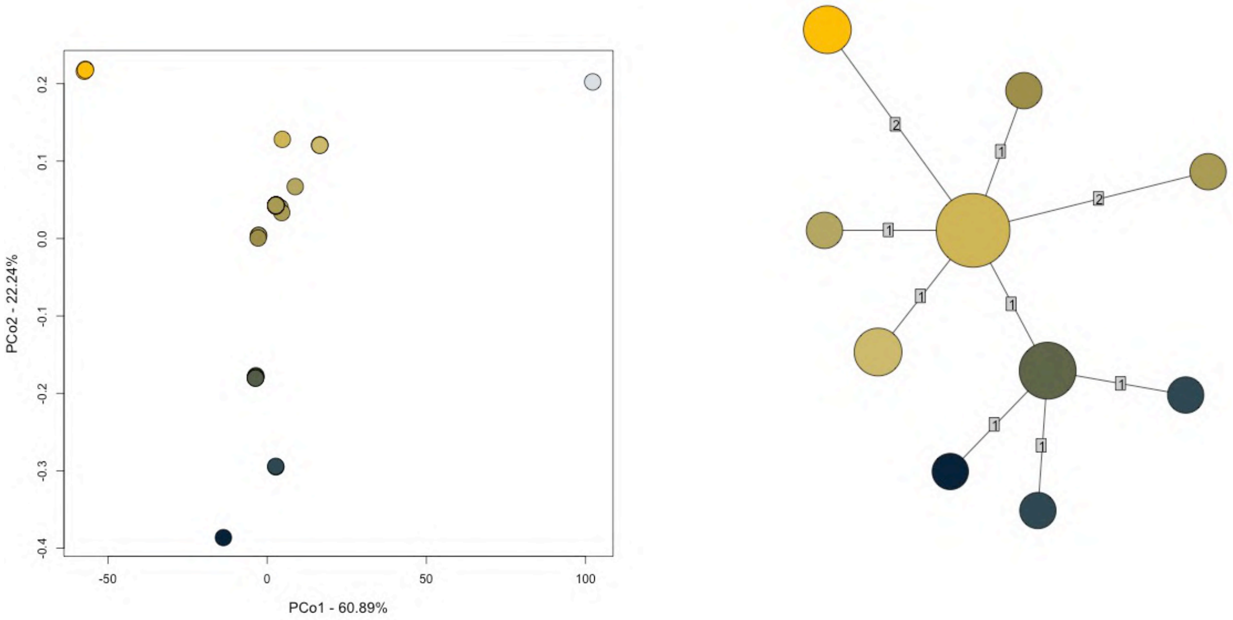

Figure 272: PCoA based on pairwise p-distances between *Formica sanguinea* sequences (left). Colours match a bidimensional colour space. Haplotype network of *Formica sanguinea* (right). Sequences > 599 bp: ID = 35, cf. = 0.

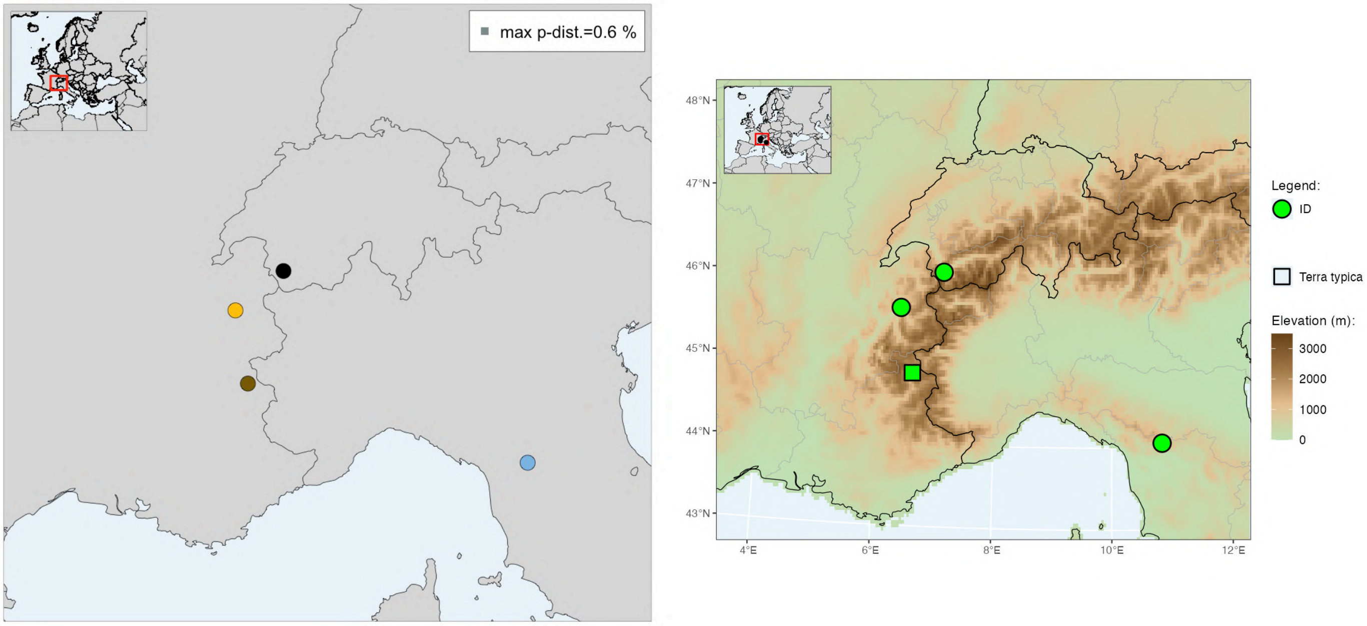

Figure 273: Genetic diversity map of *Formica selysi* Bondroit, 1918. Nearby localities of sequenced specimens are merged in pies (left). Colours match the bidimensional colour space of the PCoA projection (Fig. 273 left) of p-dist between sequences (dots). Specimen identification (ID or cf.) and source (newly sequenced or retrieved) are represented by colours, while specimen attribute (terra typica, type locality, type specimen or faunistic novelty) is represented by the shape (right). Sequences: ID = 5, cf. = 0; maximum p-distance: strict = 0.6 %, less strict = 0.6 %.

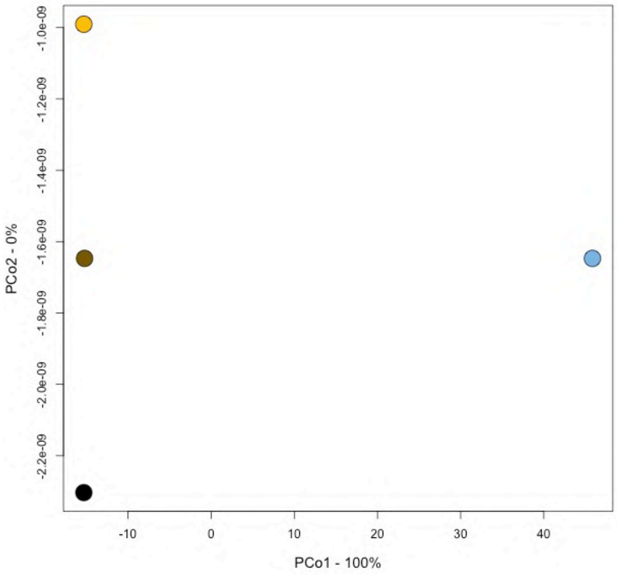

Figure 274: PCoA based on pairwise p-distances between *Formica selysi* sequences (left). Colours match a bidimensional colour space. Haplotype network analysis of *Formica selysi* was not possible.

Formica suecica

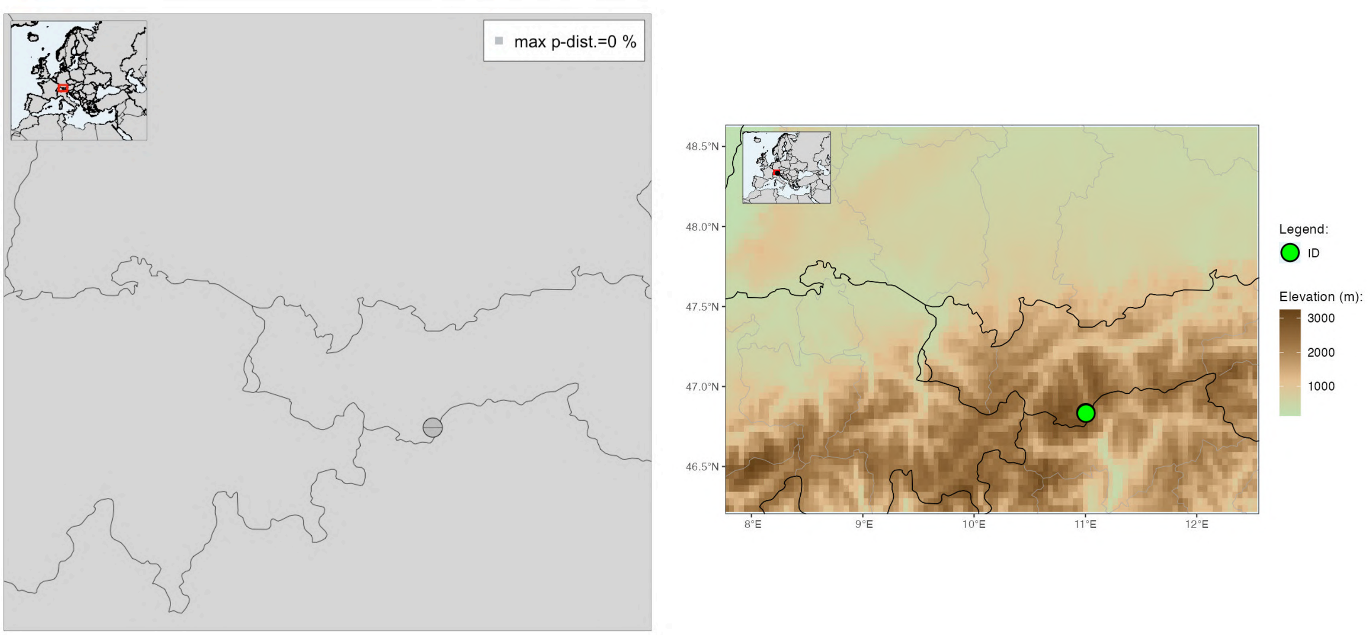

Figure 275: Genetic diversity map of *Formica suecica* Adlerz, 1902. PCoA projection was not done and therefore sequenced specimens in the genetic diversity map are coloured in gray (left). Specimen identification (ID or cf.) and source (newly sequenced or retrieved) are represented by colours, while specimen attribute (terra typica, type locality, type specimen or faunistic novelty) is represented by the shape (right). Sequences: ID = 2, cf. = 0; maximum p-distance: strict = NA, less strict = 0 %.

Haplotype network analysis of *Formica suecica* was not possible.

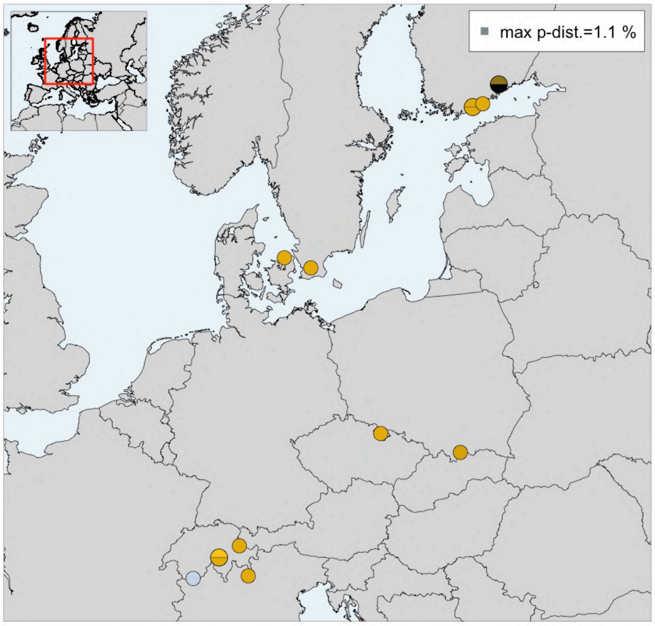

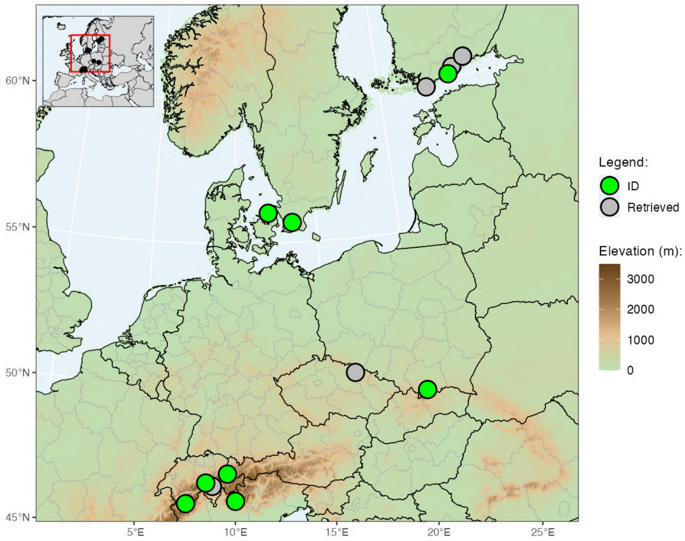

Figure 276: Genetic diversity map of *Formica truncorum* Fabricius, 1804. Nearby localities of sequenced specimens are merged in pies (left). Colours match the bidimensional colour space of the PCoA projection (Fig. 276 left) of p-dist between sequences (dots). Specimen identification (ID or cf.) and source (newly sequenced or retrieved) are represented by colours, while specimen attribute (terra typica, type locality, type specimen or faunistic novelty) is represented by the shape (right). Sequences: ID = 14, cf. = 0; maximum p-distance: strict = 1.1 %, less strict = 1.1 %.

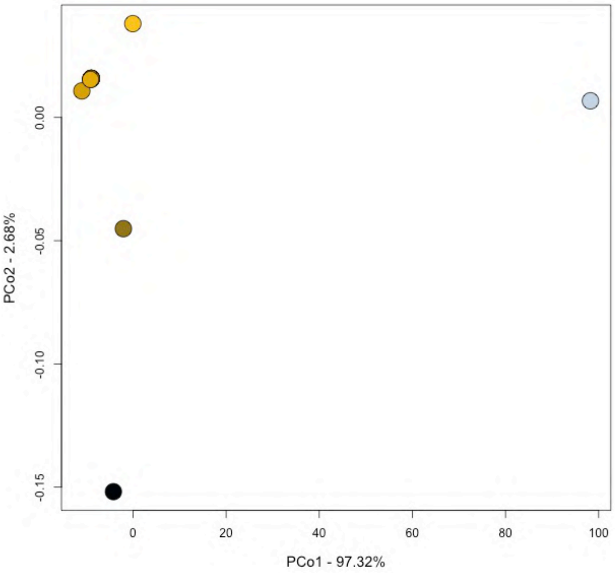

Figure 277: PCoA based on pairwise p-distances between *Formica truncorum* sequences (left). Colours match a bidimensional colour space. Haplotype network analysis of *Formica truncorum* was not possible.

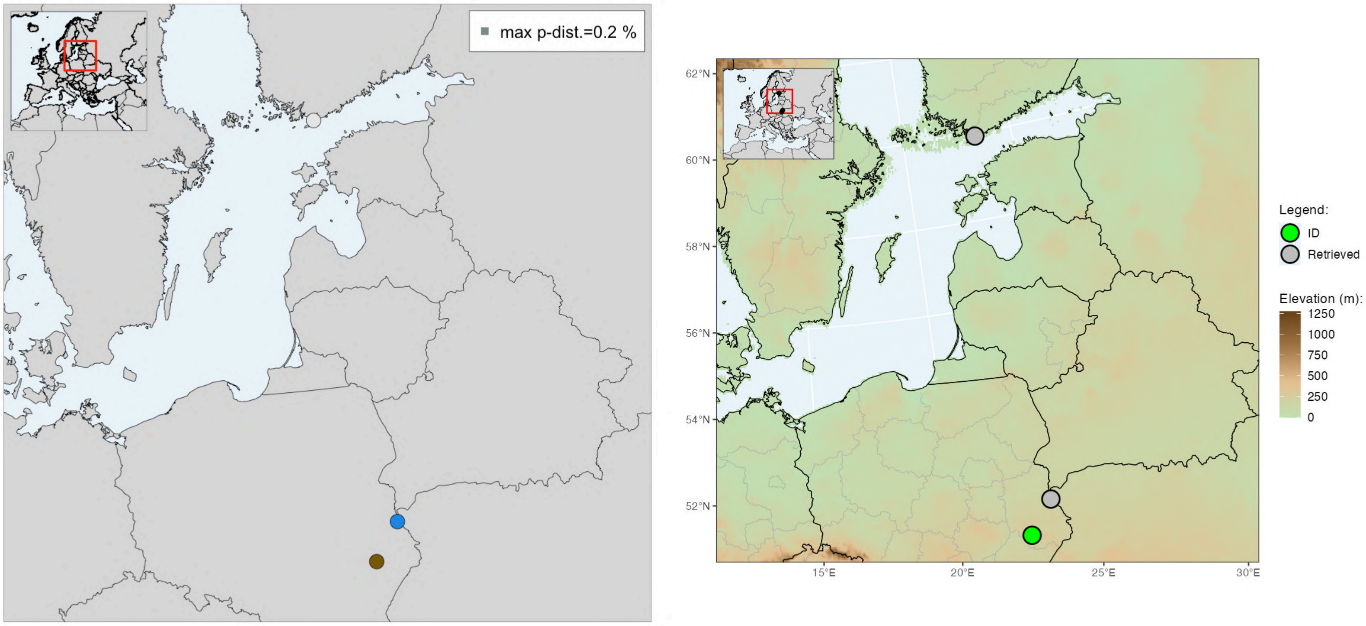

Figure 278: Genetic diversity map of *Formica uralensis* Ruzsky, 1895. Nearby localities of sequenced specimens are merged in pies (left). Colours match the bidimensional colour space of the PCoA projection (Fig. 278 left) of p-dist between sequences (dots). Specimen identification (ID or cf.) and source (newly sequenced or retrieved) are represented by colours, while specimen attribute (terra typica, type locality, type specimen or faunistic novelty) is represented by the shape (right). Sequences: ID = 3, cf. = 0; maximum p-distance: strict = 0.2 %, less strict = 0.2 %.

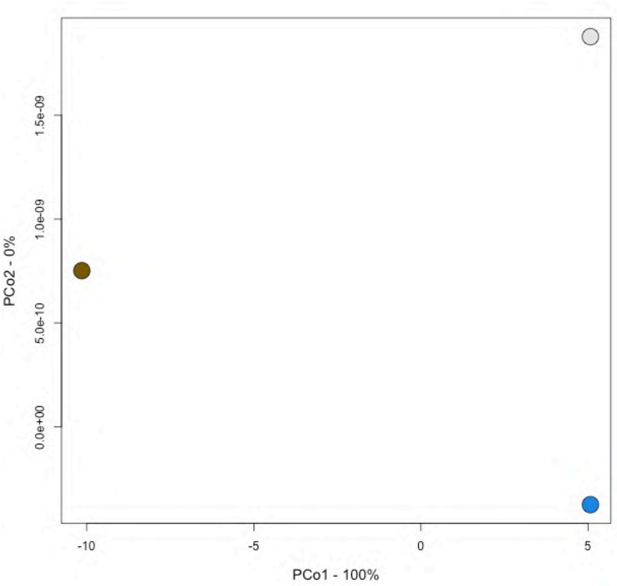

Figure 279: PCoA based on pairwise p-distances between *Formica uralensis* sequences (left). Colours match a bidimensional colour space. Haplotype network analysis of *Formica uralensis* was not possible.

Formicoxenus

Formicoxenus nitidulus

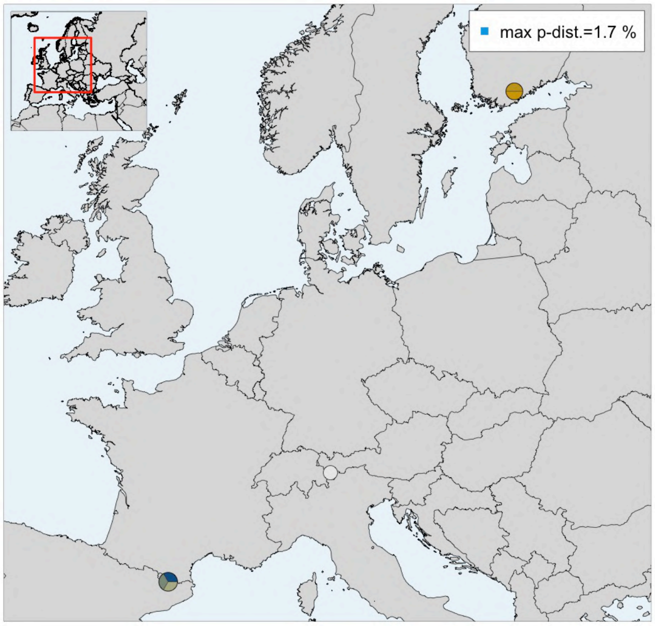

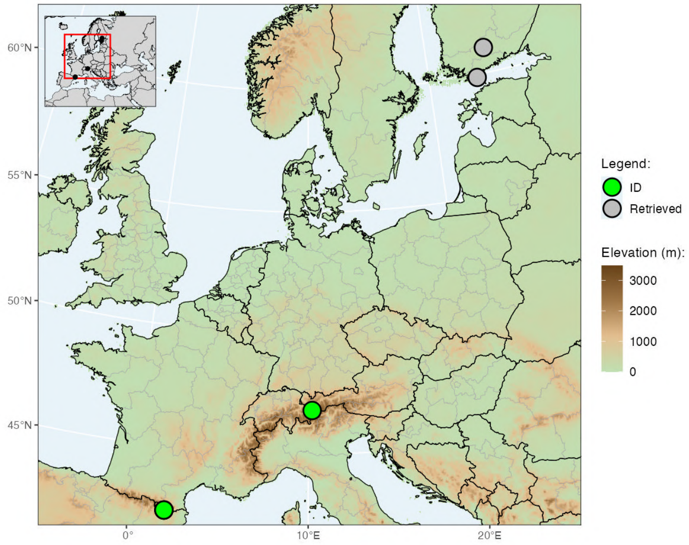

Figure 280: Genetic diversity map of *Formicoxenus nitidulus* (Nylander, 1846). Nearby localities of sequenced specimens are merged in pies (left). Colours match the bidimensional colour space of the PCoA projection (Fig. 280 left) of p-dist between sequences (dots). Specimen identification (ID or cf.) and source (newly sequenced or retrieved) are represented by colours, while specimen attribute (terra typica, type locality, type specimen or faunistic novelty) is represented by the shape (right). Sequences: ID = 5, cf. = 0; maximum p-distance: strict = 1.7 %, less strict = 1.7 %.

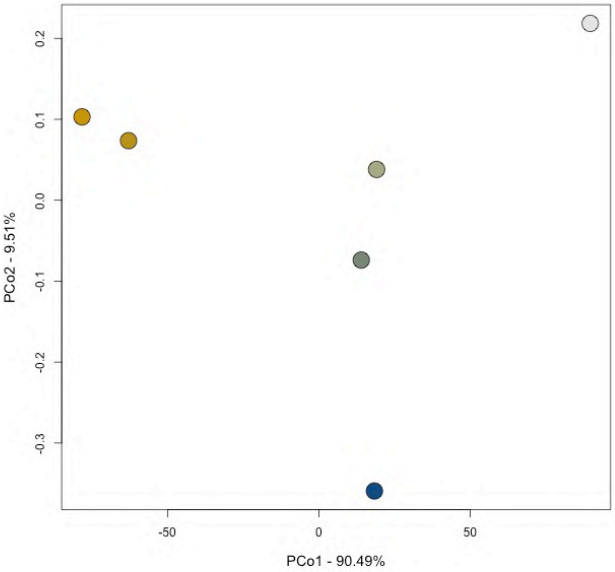

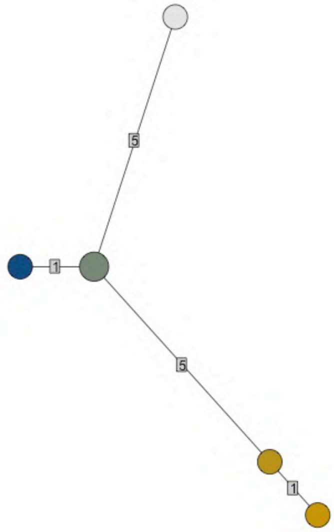

Figure 281: PCoA based on pairwise p-distances between *Formicoxenus nitidulus* sequences (left). Colours match a bidimensional colour space. Haplotype network of *Formicoxenus nitidulus* (right). Sequences > 599 bp: ID = 5, cf. = 0.

Goniomma

Goniomma baeticum

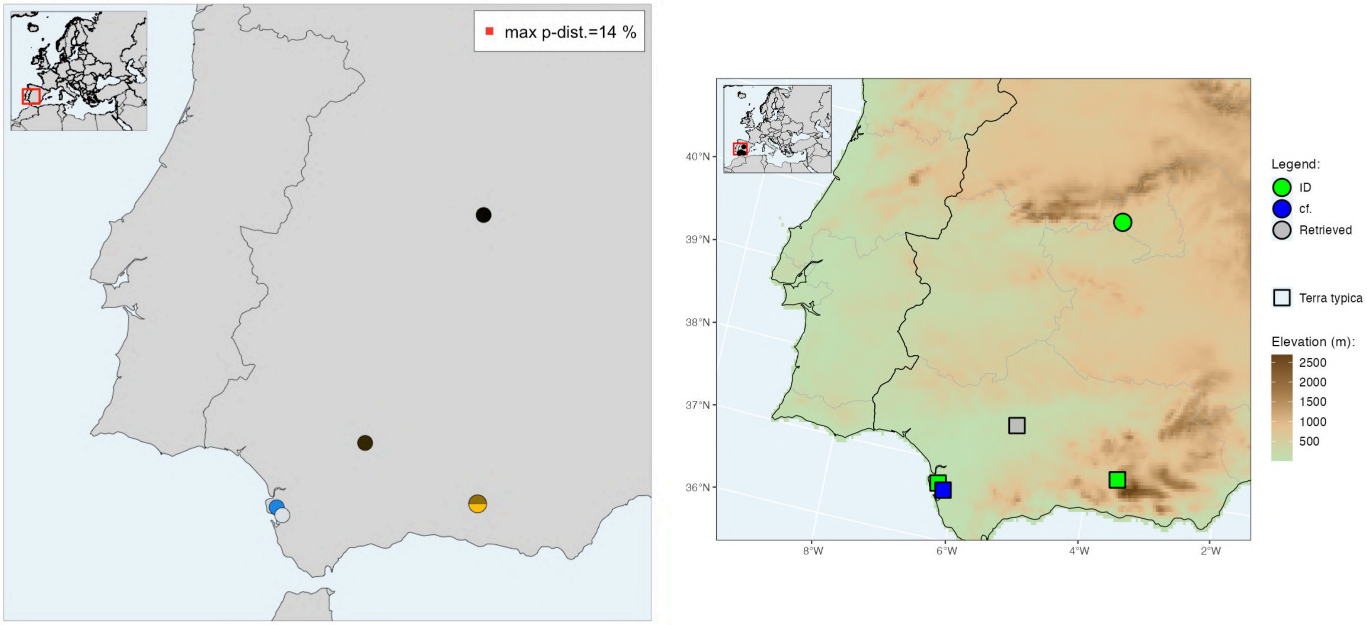

Figure 282: Genetic diversity map of *Goniomma baeticum* Reyes, Espadaler & Rodriguez, 1987. Nearby localities of sequenced specimens are merged in pies (left). Colours match the bidimensional colour space of the PCoA projection (Fig. 282 left) of p-dist between sequences (dots). Specimen identification (ID or cf.) and source (newly sequenced or retrieved) are represented by colours, while specimen attribute (terra typica, type locality, type specimen or faunistic novelty) is represented by the shape (right). Sequences: ID = 5, cf. = 1; maximum p-distance: strict = 14 %, less strict = 14 %.

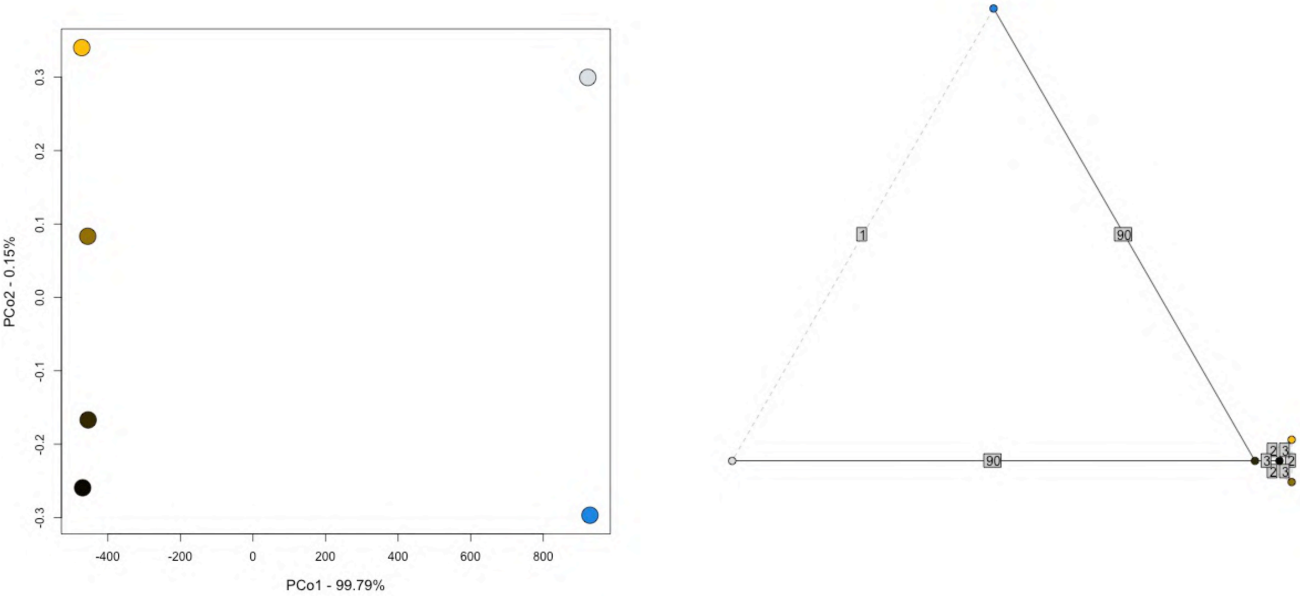

Figure 283: PCoA based on pairwise p-distances between *Goniomma baeticum* sequences (left). Colours match a bidimensional colour space. Haplotype network of *Goniomma baeticum* (right). Sequences > 599 bp: ID = 5, cf. = 1.

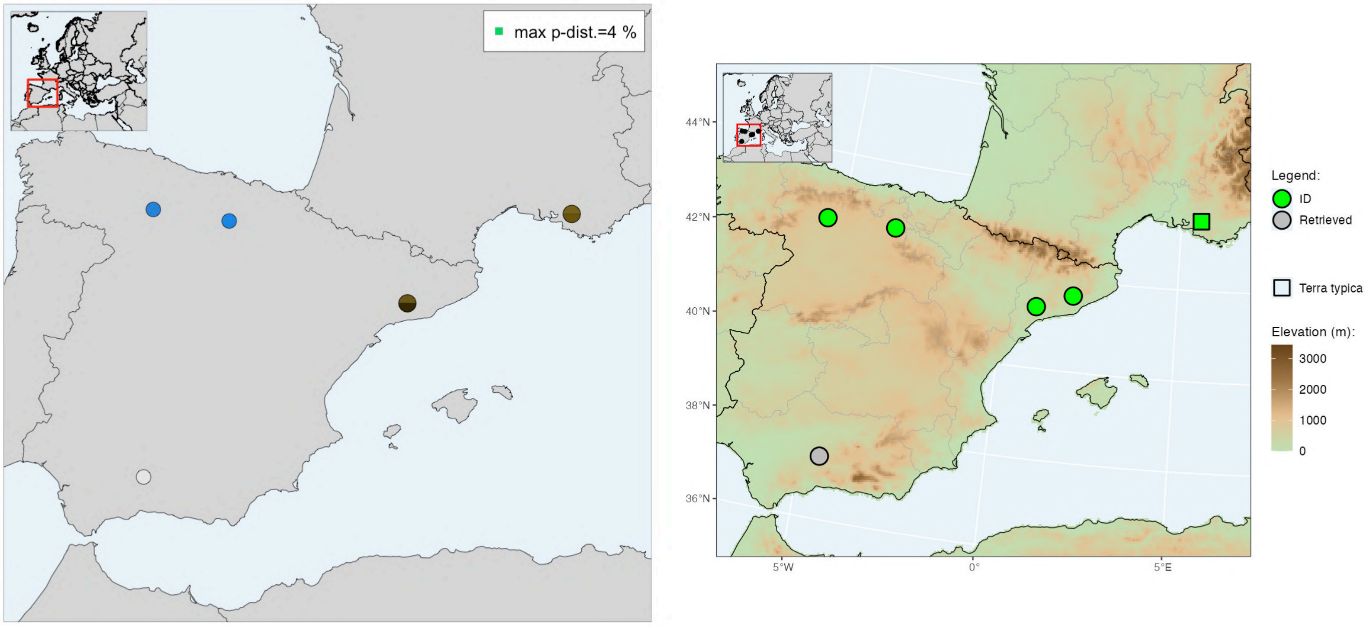

Figure 284: Genetic diversity map of *Goniomma blanci* (André, 1881). Nearby localities of sequenced specimens are merged in pies (left). Colours match the bidimensional colour space of the PCoA projection (Fig. 284 left) of p-dist between sequences (dots). Specimen identification (ID or cf.) and source (newly sequenced or retrieved) are represented by colours, while specimen attribute (terra typica, type locality, type specimen or faunistic novelty) is represented by the shape (right). Sequences: ID = 7, cf. = 0; maximum p-distance: strict = 4 %, less strict = 4 %.

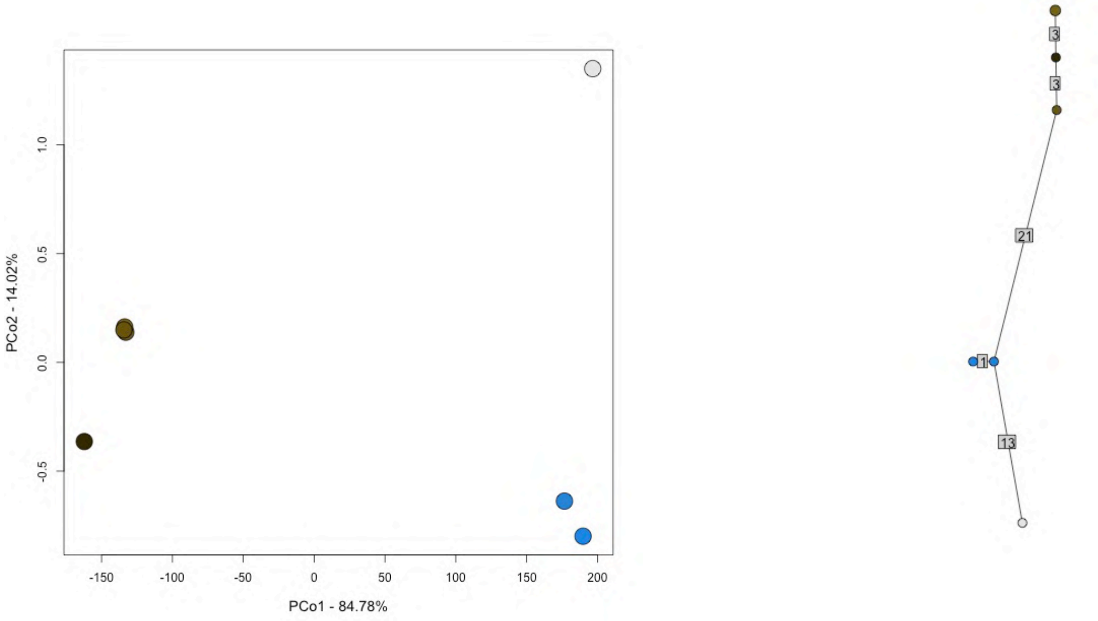

Figure 285: PCoA based on pairwise p-distances between *Goniomma blanci* sequences (left). Colours match a bidimensional colour space. Haplotype network of *Goniomma blanci* (right). Sequences > 599 bp: ID = 7, cf. = 0.

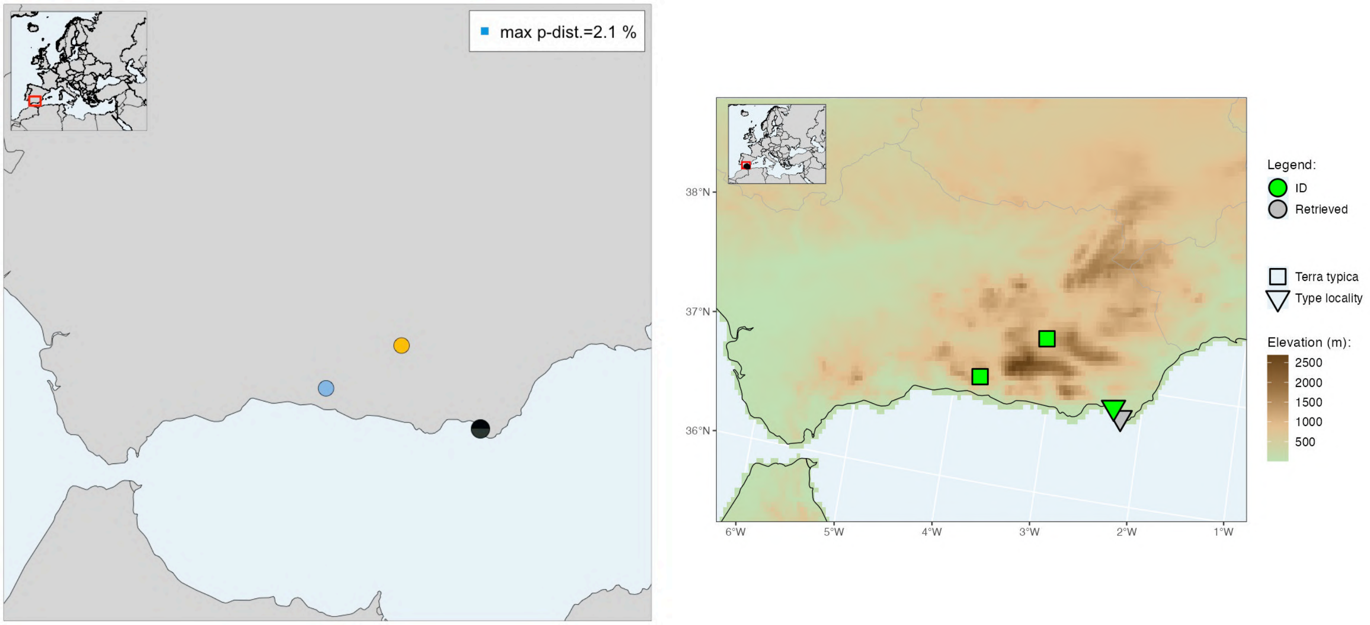

Figure 286: Genetic diversity map of *Goniomma collingwoodi* Espadaler, 1997. Nearby localities of sequenced specimens are merged in pies (left). Colours match the bidimensional colour space of the PCoA projection (Fig. 286 left) of p-dist between sequences (dots). Specimen identification (ID or cf.) and source (newly sequenced or retrieved) are represented by colours, while specimen attribute (terra typica, type locality, type specimen or faunistic novelty) is represented by the shape (right). Sequences: ID = 4, cf. = 0; maximum p-distance: strict = 2.1 %, less strict = 2.1 %.

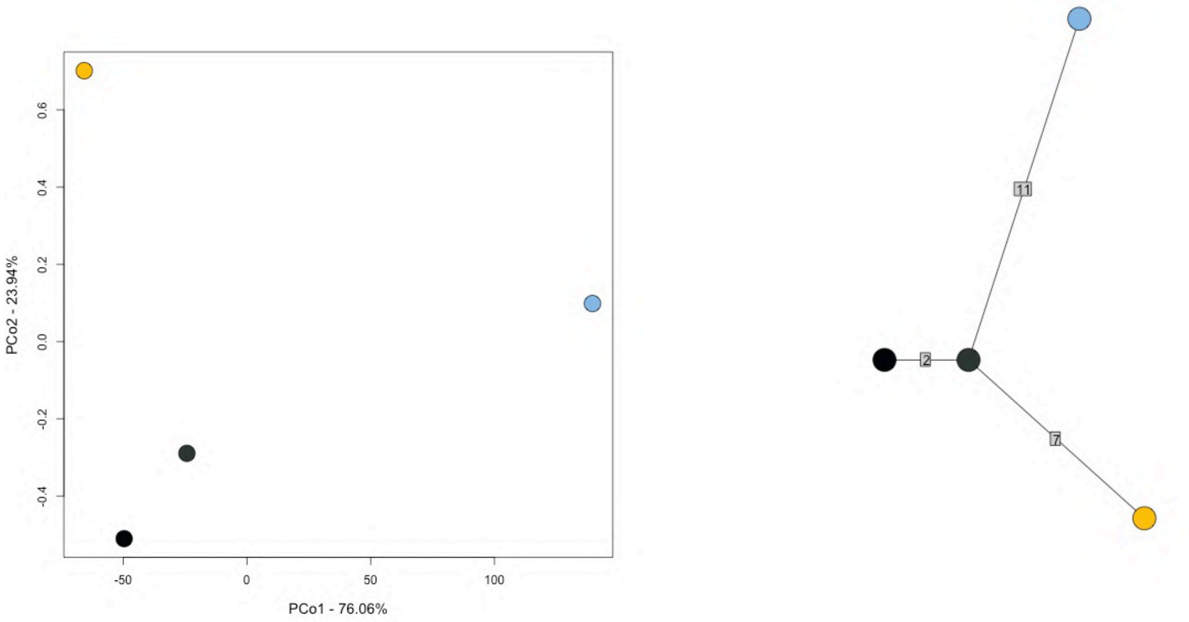

Figure 287: PCoA based on pairwise p-distances between *Goniomma collingwoodi* sequences (left). Colours match a bidimensional colour space. Haplotype network of *Goniomma collingwoodi* (right). Sequences > 599 bp: ID = 4, cf. = 0.

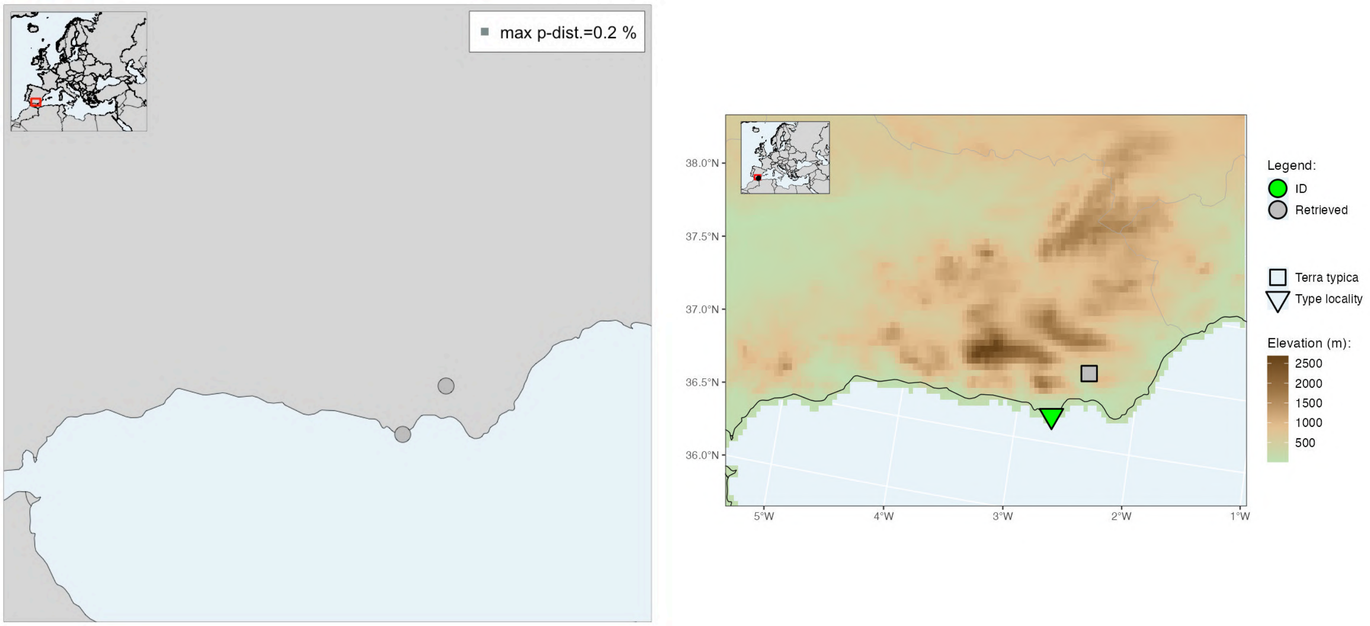

Figure 288: Genetic diversity map of *Goniomma compressisquama* Tinaut, 1995. PCoA projection was not done and therefore sequenced specimens in the genetic diversity map are coloured in gray (left). Specimen identification (ID or cf.) and source (newly sequenced or retrieved) are represented by colours, while specimen attribute (terra typica, type locality, type specimen or faunistic novelty) is represented by the shape (right). Sequences: ID = 2, cf. = 0; maximum p-distance: strict = NA, less strict = 0.2 %.

Haplotype network analysis of *Goniomma compressisquama* was not possible.

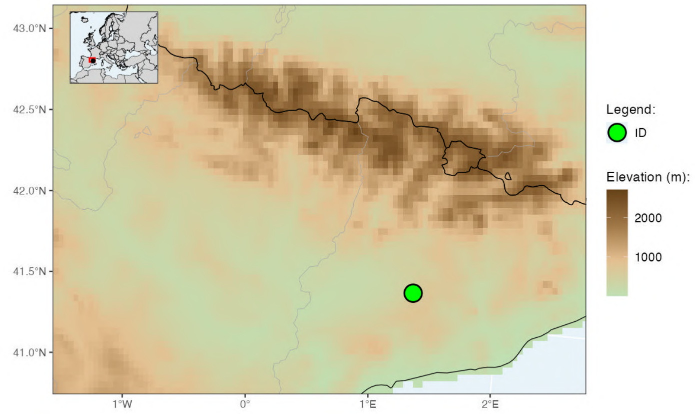

Figure 289: Map of *Goniomma decipiens* Espadaler, 1997. Due to the presence of a single sequence, the genetic diversity map and the PCoA projection were not done. Specimen identification (ID or cf.) and source (newly sequenced or retrieved) are represented by colours, while specimen attribute (terra typica, type locality, type specimen or faunistic novelty) is represented by the shape. Sequences: ID = 1, cf. = 0; maximum p-distance: strict = NA, less strict = NA.

Haplotype network analysis of *Goniomma decipiens* was not possible.

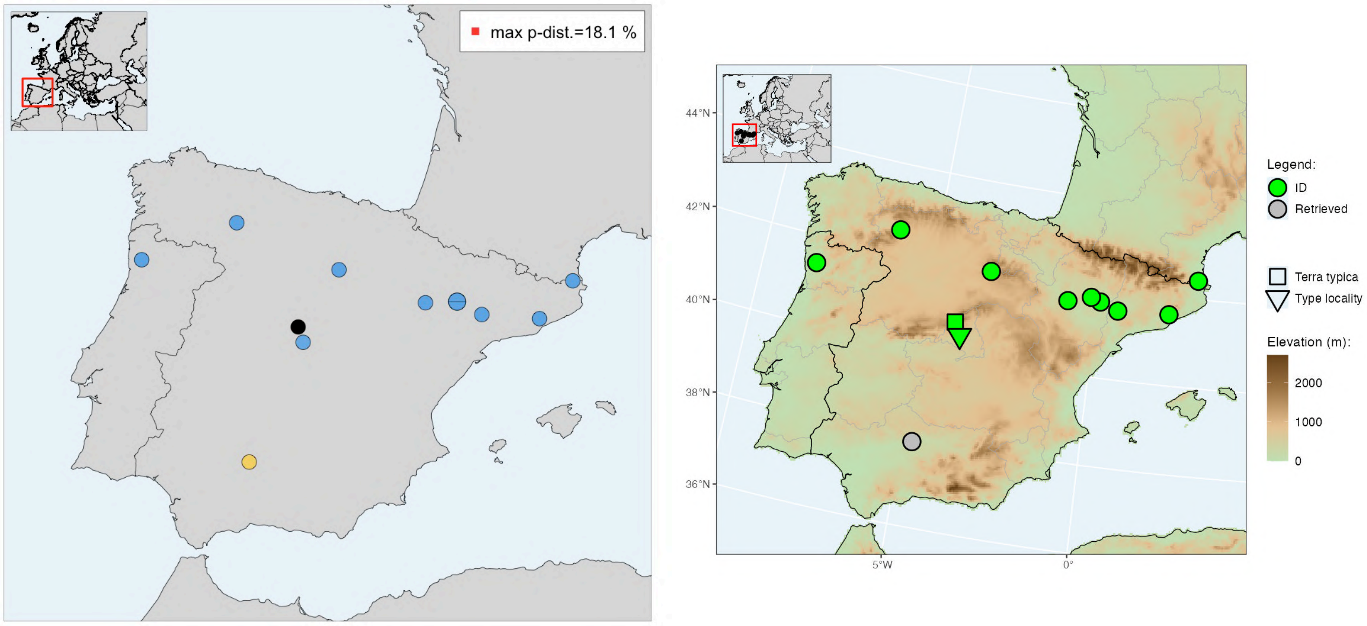

Figure 290: Genetic diversity map of *Goniomma hispanicum* (André, 1883). Nearby localities of sequenced specimens are merged in pies (left). Colours match the bidimensional colour space of the PCoA projection (Fig. 290 left) of p-dist between sequences (dots). Specimen identification (ID or cf.) and source (newly sequenced or retrieved) are represented by colours, while specimen attribute (terra typica, type locality, type specimen or faunistic novelty) is represented by the shape (right). Sequences: ID = 12, cf. = 0; maximum p-distance: strict = 14.7 %, less strict = 18.1 %.

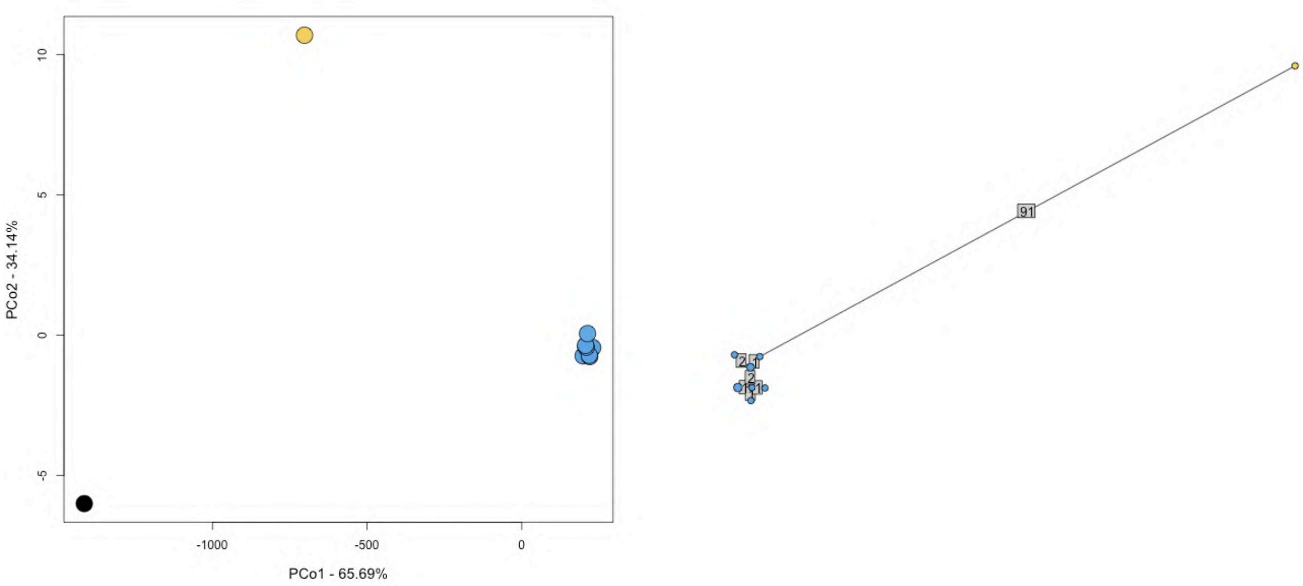

Figure 291: PCoA based on pairwise p-distances between *Goniomma hispanicum* sequences (left). Colours match a bidimensional colour space. Haplotype network of *Goniomma hispanicum* (right). Sequences > 599 bp: ID = 11, cf. = 0.

Harpagoxenus

Harpagoxenus sublaevis

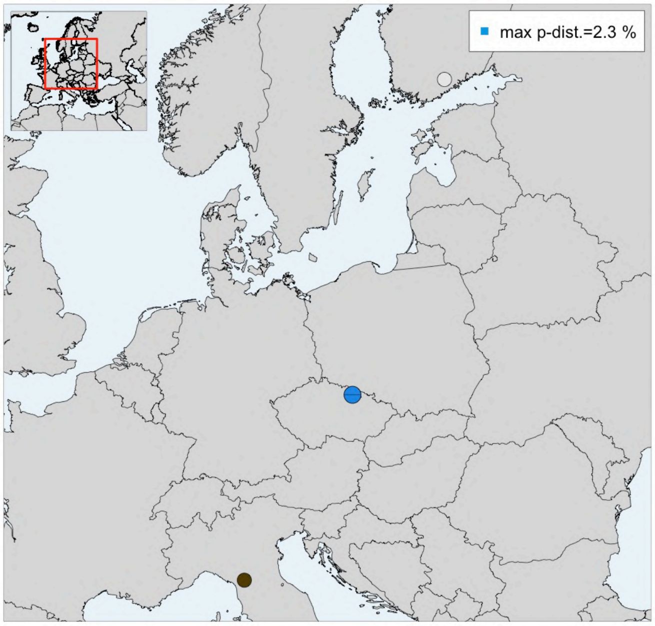

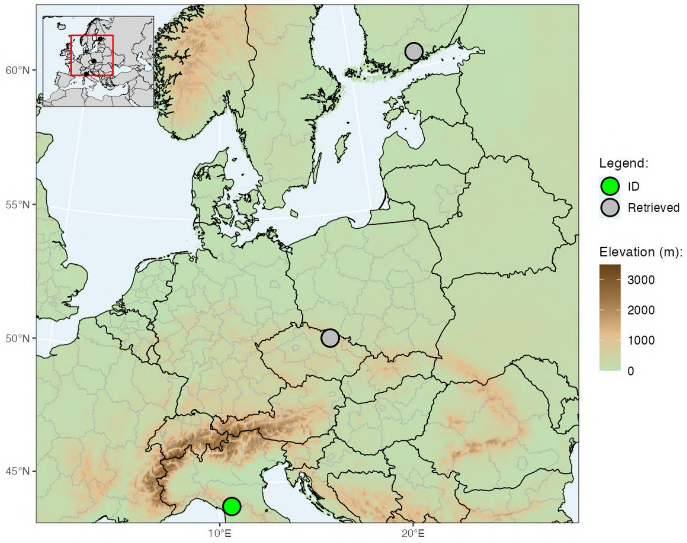

Figure 292: Genetic diversity map of *Harpagoxenus sublaevis* (Nylander, 1849). Nearby localities of sequenced specimens are merged in pies (left). Colours match the bidimensional colour space of the PCoA projection (Fig. 292 left) of p-dist between sequences (dots). Specimen identification (ID or cf.) and source (newly sequenced or retrieved) are represented by colours, while specimen attribute (terra typica, type locality, type specimen or faunistic novelty) is represented by the shape (right). Sequences: ID = 5, cf. = 0; maximum p-distance: strict = 2.3 %, less strict = 2.3 %.

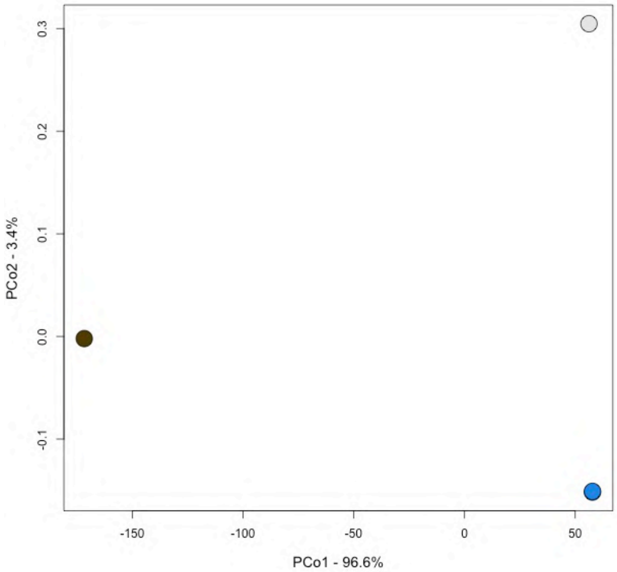

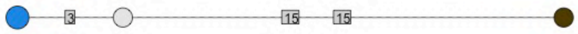

Figure 293: PCoA based on pairwise p-distances between *Harpagoxenus sublaevis* sequences (left). Colours match a bidimensional colour space. Haplotype network of *Harpagoxenus sublaevis* (right). Sequences > 599 bp: ID = 5, cf. = 0.

Hypoponera

Hypoponera abeillei

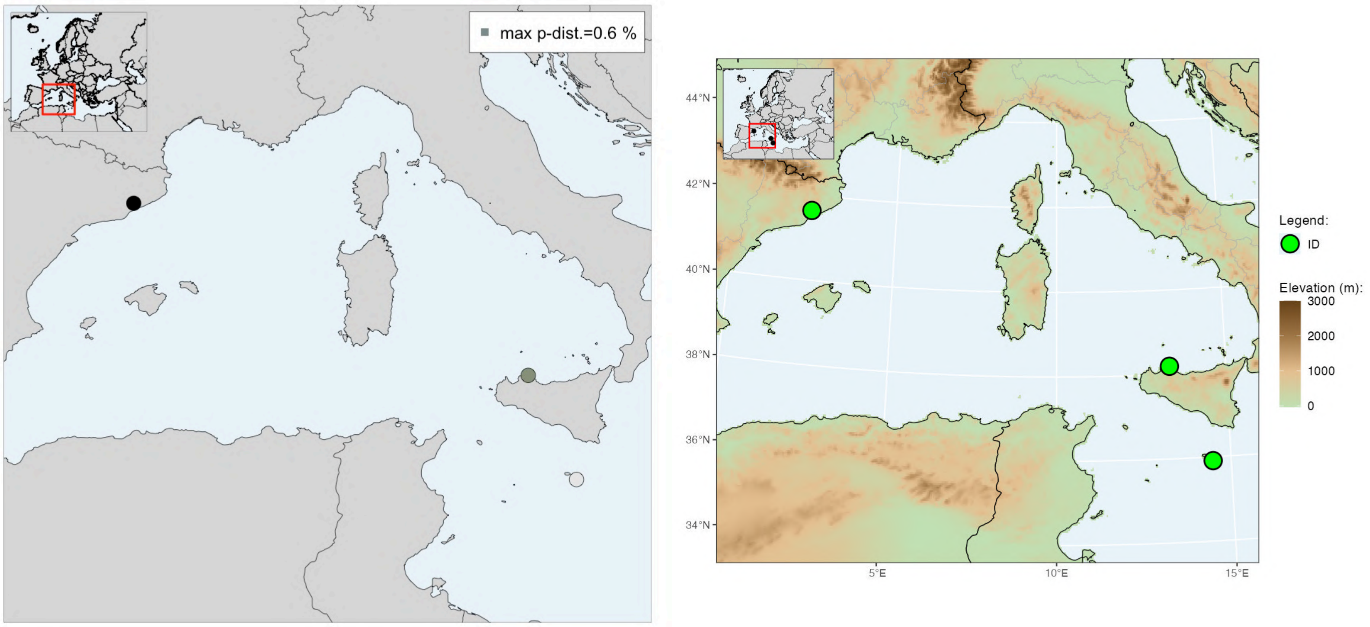

Figure 294: Genetic diversity map of *Hypoponera abeillei* (André, 1881). Nearby localities of sequenced specimens are merged in pies (left). Colours match the bidimensional colour space of the PCoA projection (Fig. 294 left) of p-dist between sequences (dots). Specimen identification (ID or cf.) and source (newly sequenced or retrieved) are represented by colours, while specimen attribute (terra typica, type locality, type specimen or faunistic novelty) is represented by the shape (right). Sequences: ID = 4, cf. = 0; maximum p-distance: strict = 0.6 %, less strict = 0.6 %.

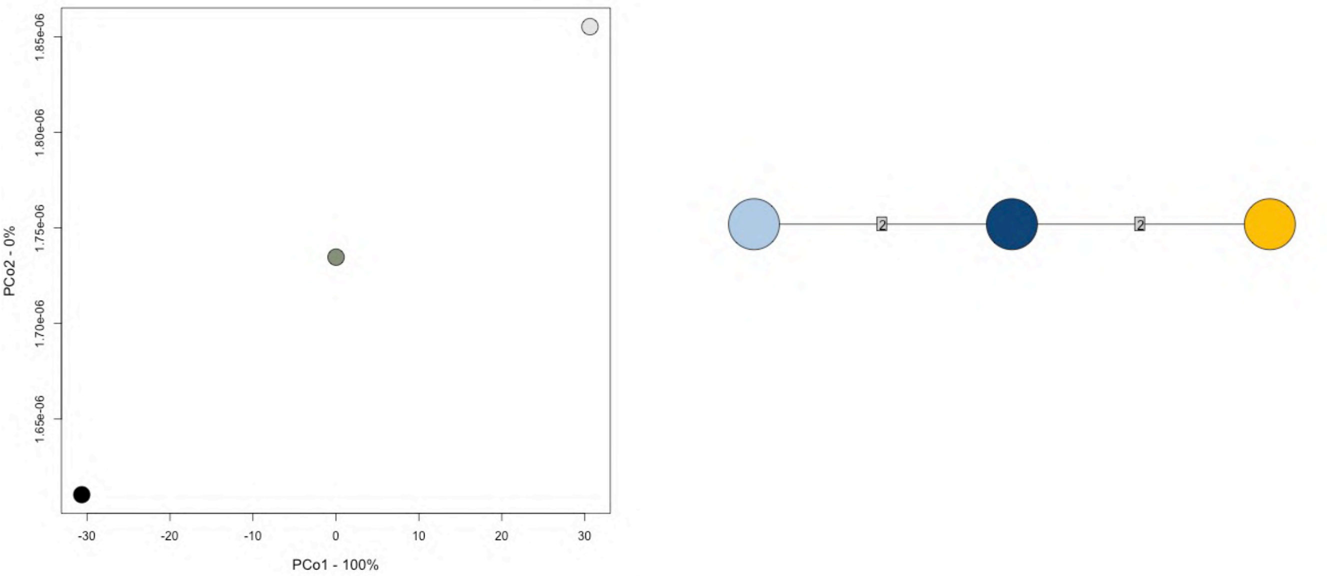

Figure 295: PCoA based on pairwise p-distances between *Hypoponera abeillei* sequences (left). Colours match a bidimensional colour space. Haplotype network of *Hypoponera abeillei* (right). Sequences > 599 bp: ID = 4, cf. = 0.

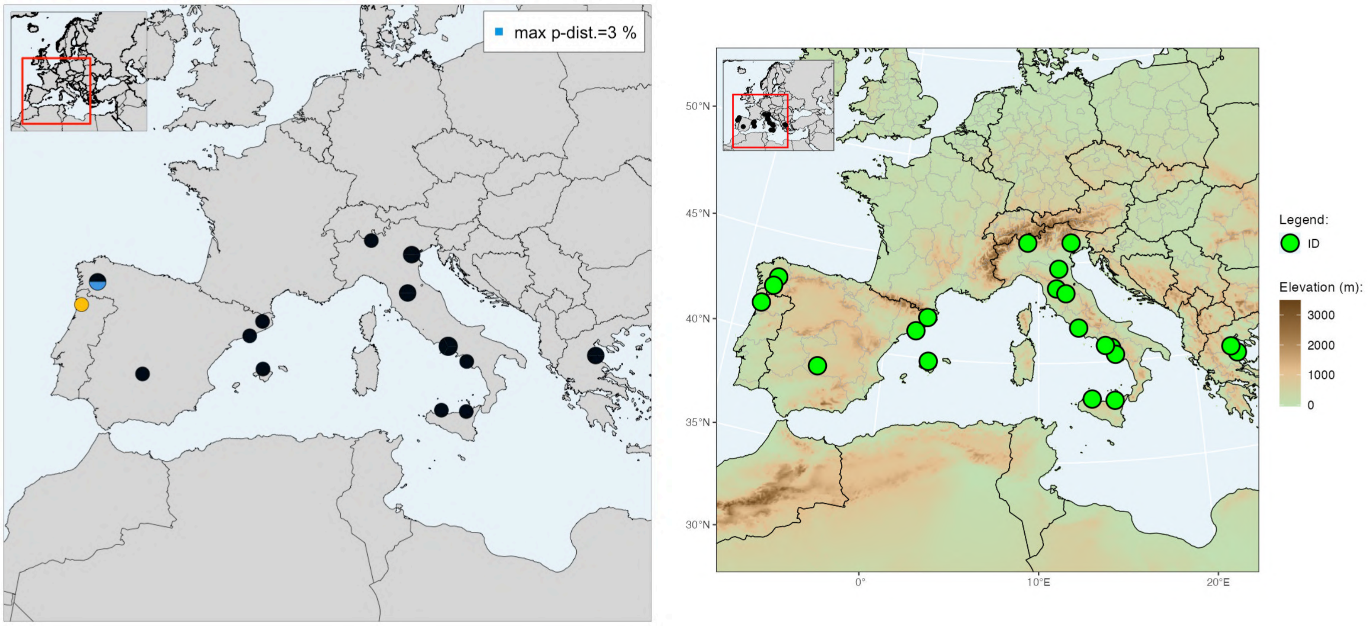

Figure 296: Genetic diversity map of *Hypoponera eduardi* (Forel, 1894). Nearby localities of sequenced specimens are merged in pies (left). Colours match the bidimensional colour space of the PCoA projection (Fig. 296 left) of p-dist between sequences (dots). Specimen identification (ID or cf.) and source (newly sequenced or retrieved) are represented by colours, while specimen attribute (terra typica, type locality, type specimen or faunistic novelty) is represented by the shape (right). Sequences: ID = 20, cf. = 0; maximum p-distance: strict = 3 %, less strict = 3 %.

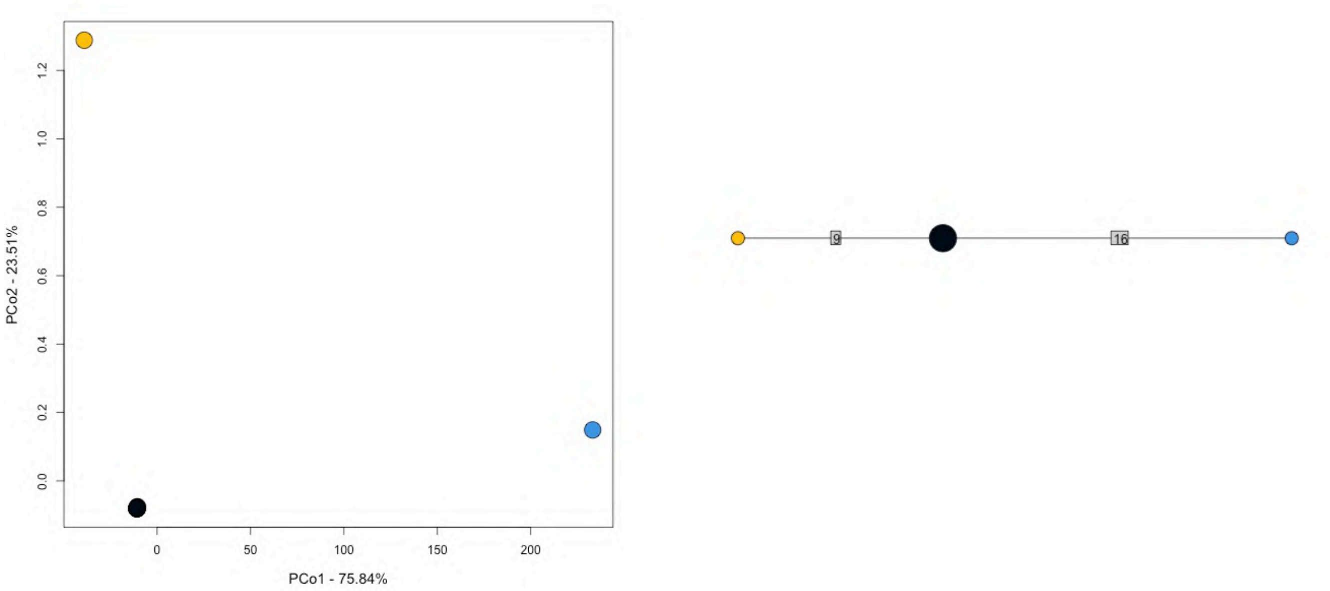

Figure 297: PCoA based on pairwise p-distances between *Hypoponera eduardi* sequences (left). Colours match a bidimensional colour space. Haplotype network of *Hypoponera eduardi* (right). Sequences > 599 bp: ID = 20, cf. = 0.

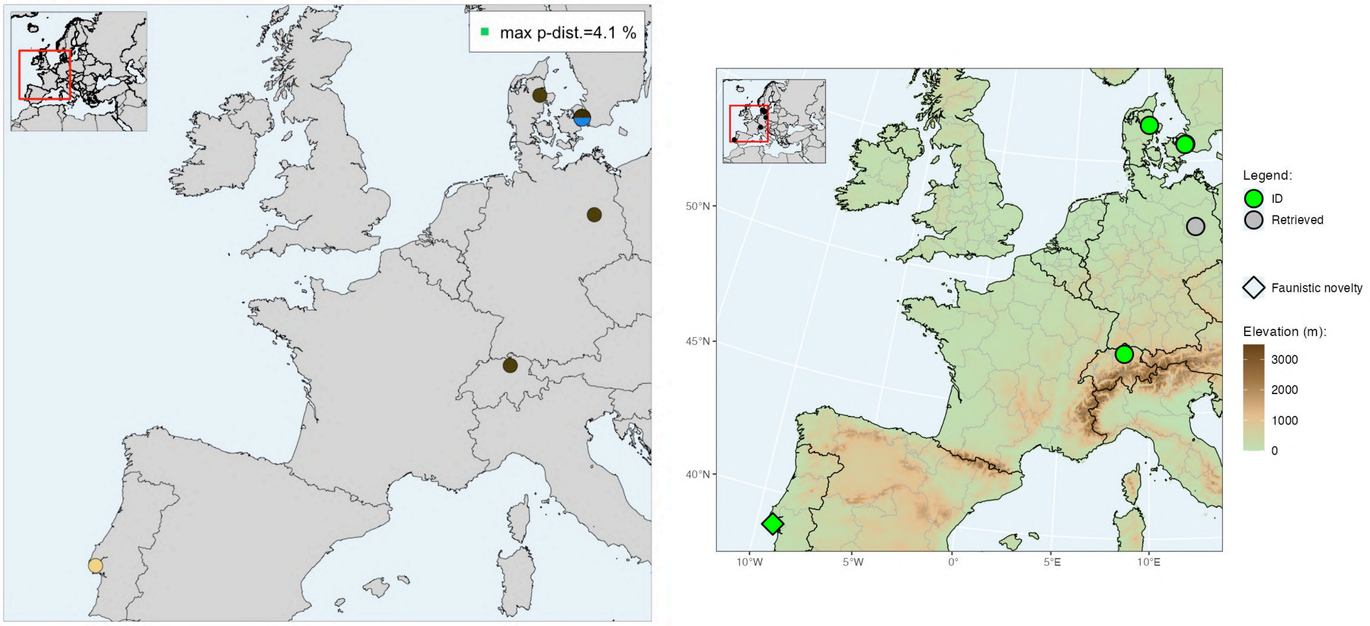

Figure 298: Genetic diversity map of *Hypoponera ergatandria* (Forel, 1893). Nearby localities of sequenced specimens are merged in pies (left). Colours match the bidimensional colour space of the PCoA projection (Fig. 298 left) of p-dist between sequences (dots). Specimen identification (ID or cf.) and source (newly sequenced or retrieved) are represented by colours, while specimen attribute (terra typica, type locality, type specimen or faunistic novelty) is represented by the shape (right). Sequences: ID = 6, cf. = 0; maximum p-distance: strict = 4.1 %, less strict = 4.1 %.

The species is reported for the first time in Portugal.

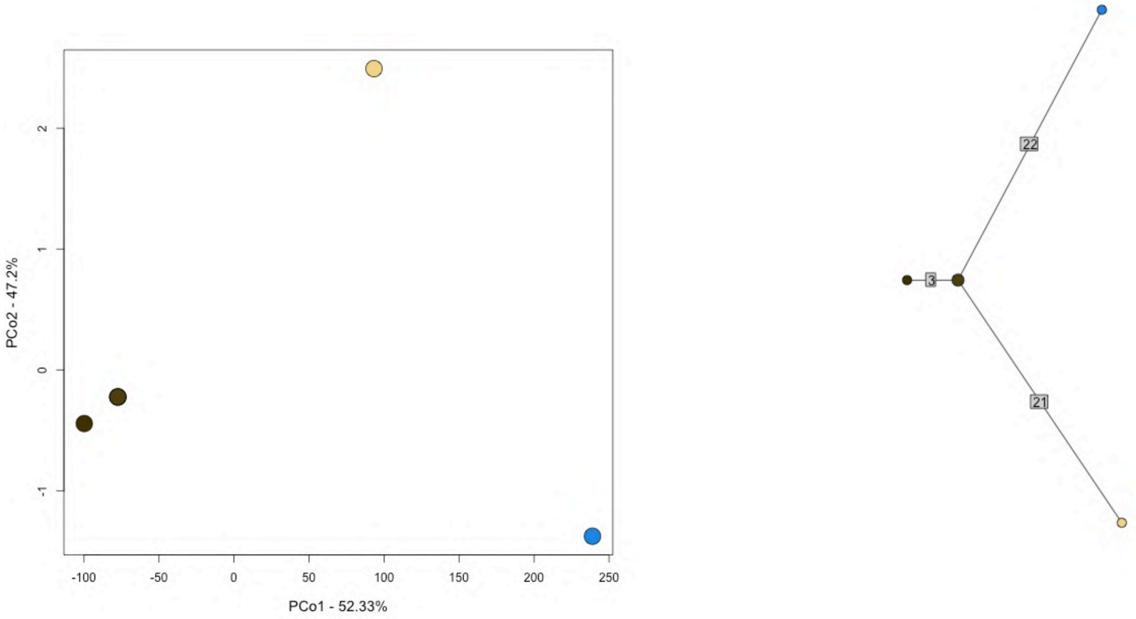

Figure 299: PCoA based on pairwise p-distances between *Hypoponera ergatandria* sequences (left). Colours match a bidimensional colour space. Haplotype network of *Hypoponera ergatandria* (right). Sequences > 599 bp: ID = 6, cf. = 0.

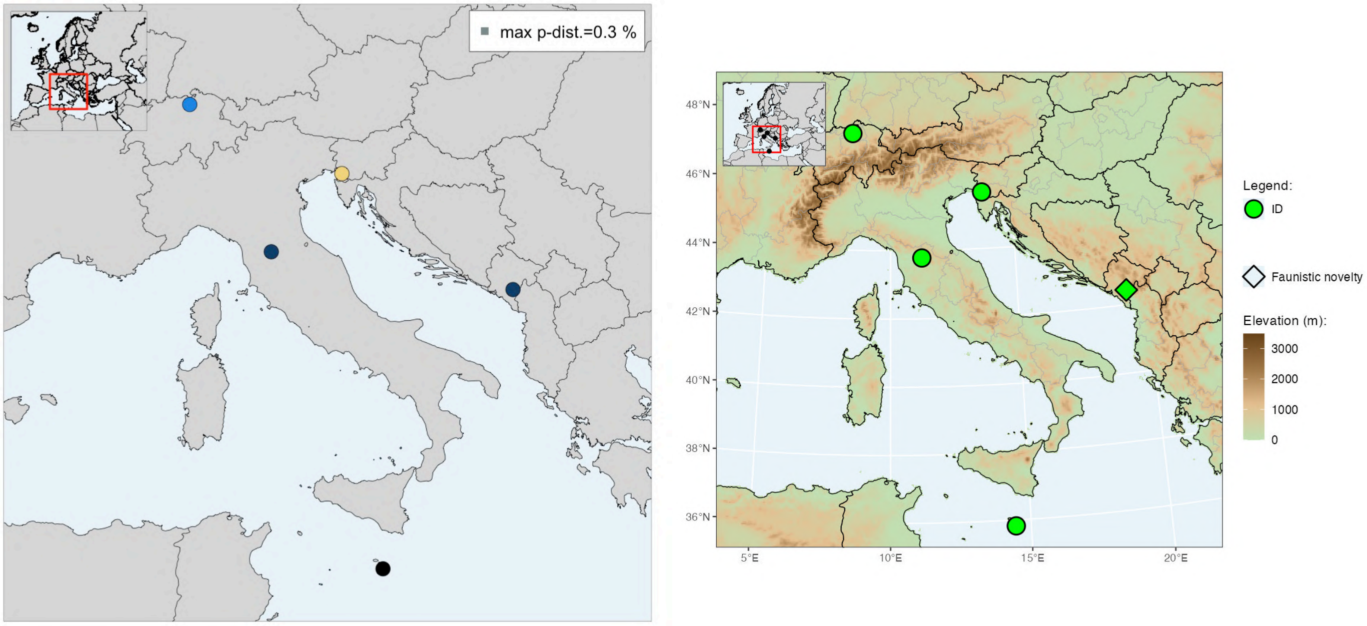

Figure 300: Genetic diversity map of *Hypoponera punctatissima* (Roger, 1859). Nearby localities of sequenced specimens are merged in pies (left). Colours match the bidimensional colour space of the PCoA projection (Fig. 300 left) of p-dist between sequences (dots). Specimen identification (ID or cf.) and source (newly sequenced or retrieved) are represented by colours, while specimen attribute (terra typica, type locality, type specimen or faunistic novelty) is represented by the shape (right). Sequences: ID = 5, cf. = 0; maximum p-distance: strict = 0.3 %, less strict = 0.3 %.

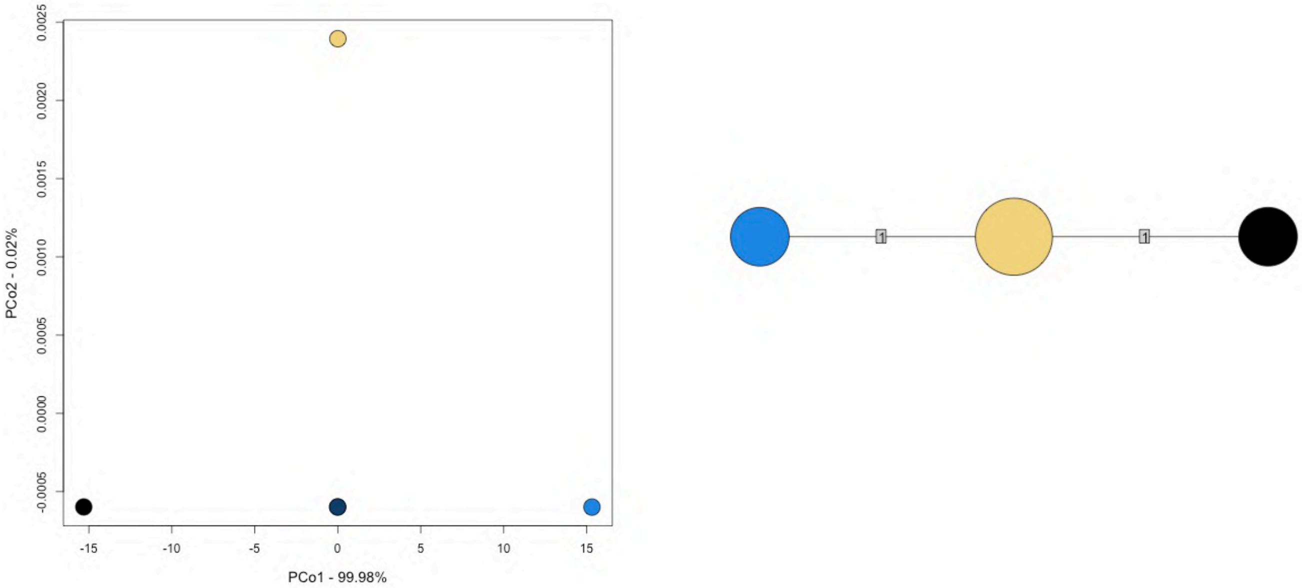

Figure 301: PCoA based on pairwise p-distances between *Hypoponera punctatissima* sequences (left). Colours match a bidimensional colour space. Haplotype network of *Hypoponera punctatissima* (right). Sequences > 599 bp: ID = 5, cf. = 0.

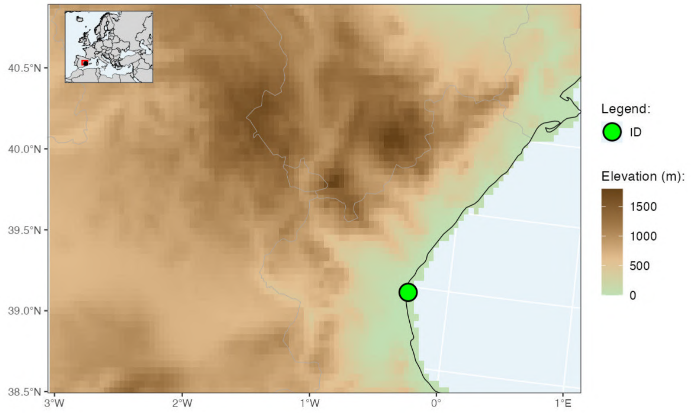

Figure 302: Map of *Hypoponera ragusai* (Emery, 1894). Due to the presence of a single sequence, the genetic diversity map and the PCoA projection were not done. Specimen identification (ID or cf.) and source (newly sequenced or retrieved) are represented by colours, while specimen attribute (terra typica, type locality, type specimen or faunistic novelty) is represented by the shape. Sequences: ID = 1, cf. = 0; maximum p-distance: strict = NA, less strict = NA.

Haplotype network analysis of *Hypoponera ragusai* was not possible.

Iberoformica

Iberoformica subrufa

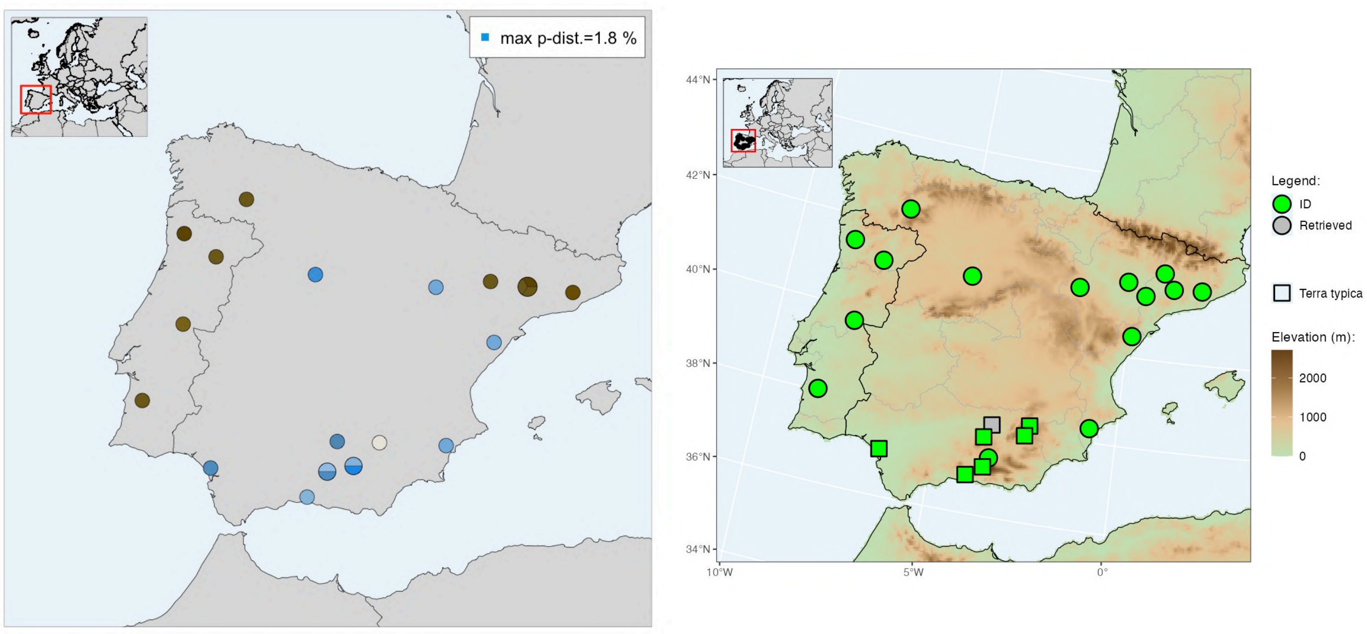

Figure 303: Genetic diversity map of *Iberoformica subrufa* (Roger, 1859). Nearby localities of sequenced specimens are merged in pies (left). Colours match the bidimensional colour space of the PCoA projection (Fig. 303 left) of p-dist between sequences (dots). Specimen identification (ID or cf.) and source (newly sequenced or retrieved) are represented by colours, while specimen attribute (terra typica, type locality, type specimen or faunistic novelty) is represented by the shape (right). Sequences: ID = 23, cf. = 0; maximum p-distance: strict = 1.8 %, less strict = 1.8 %.

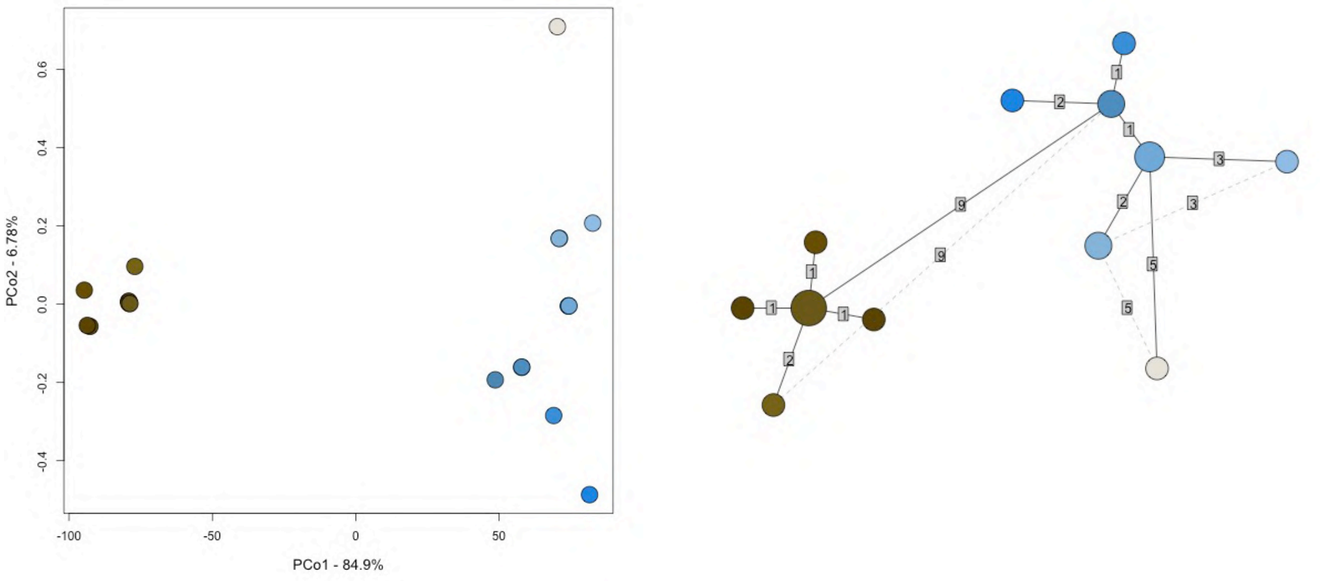

Figure 304: PCoA based on pairwise p-distances between *Iberoformica subrufa* sequences (left). Colours match a bidimensional colour space. Haplotype network of *Iberoformica subrufa* (right). Sequences > 599 bp: ID = 22, cf. = 0.

Lasius

Lasius alienus

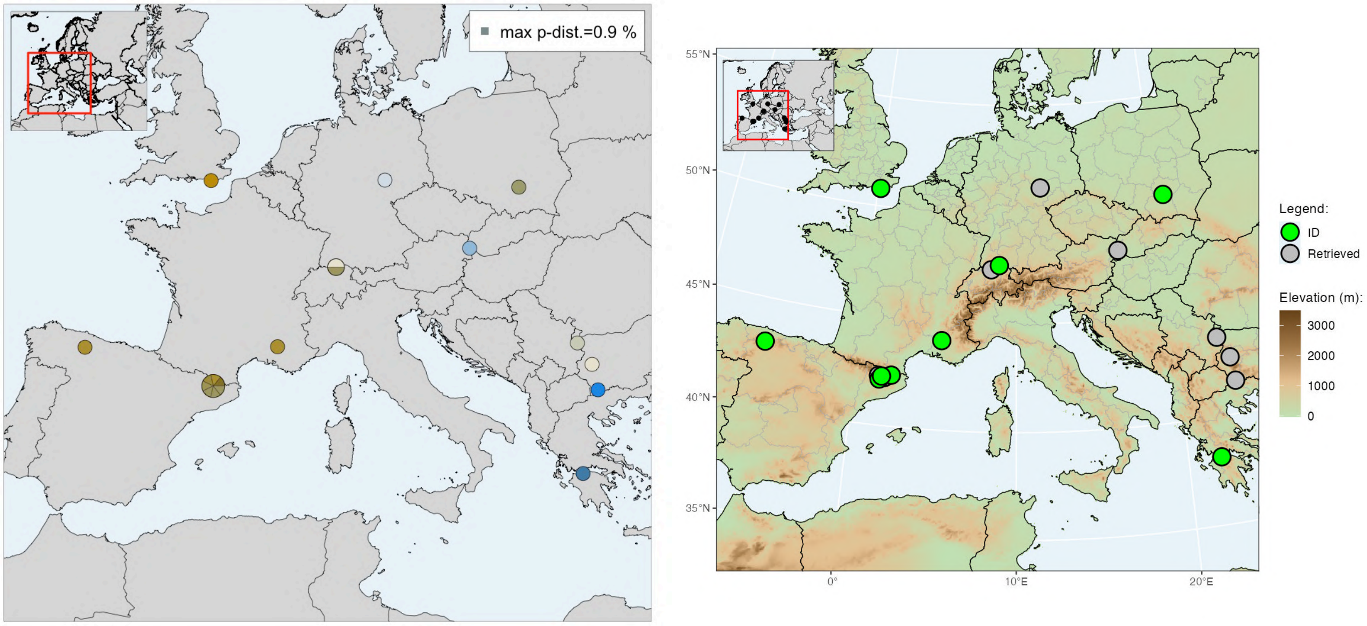

Figure 305: Genetic diversity map of *Lasius alienus* (Foerster, 1850). Nearby localities of sequenced specimens are merged in pies (left). Colours match the bidimensional colour space of the PCoA projection (Fig. 305 left) of p-dist between sequences (dots). Specimen identification (ID or cf.) and source (newly sequenced or retrieved) are represented by colours, while specimen attribute (terra typica, type locality, type specimen or faunistic novelty) is represented by the shape (right). Sequences: ID = 19, cf. = 0; maximum p-distance: strict = 0.9 %, less strict = 0.9 %.

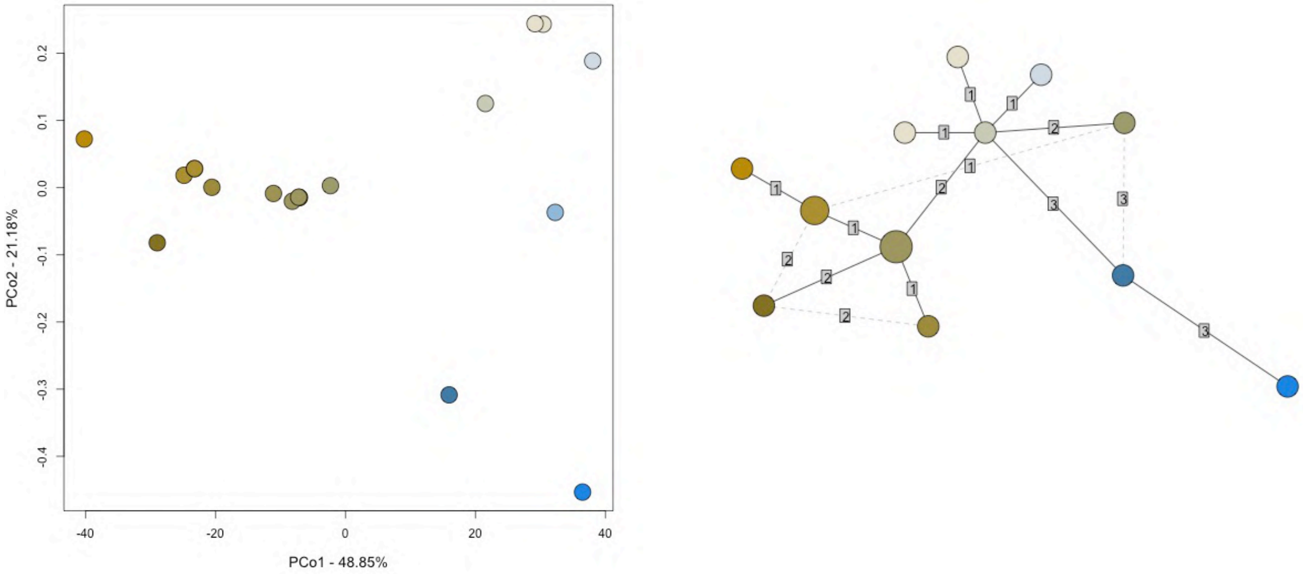

Figure 306: PCoA based on pairwise p-distances between *Lasius alienus* sequences (left). Colours match a bidimensional colour space. Haplotype network of *Lasius alienus* (right). Sequences > 599 bp: ID = 18, cf. = 0.

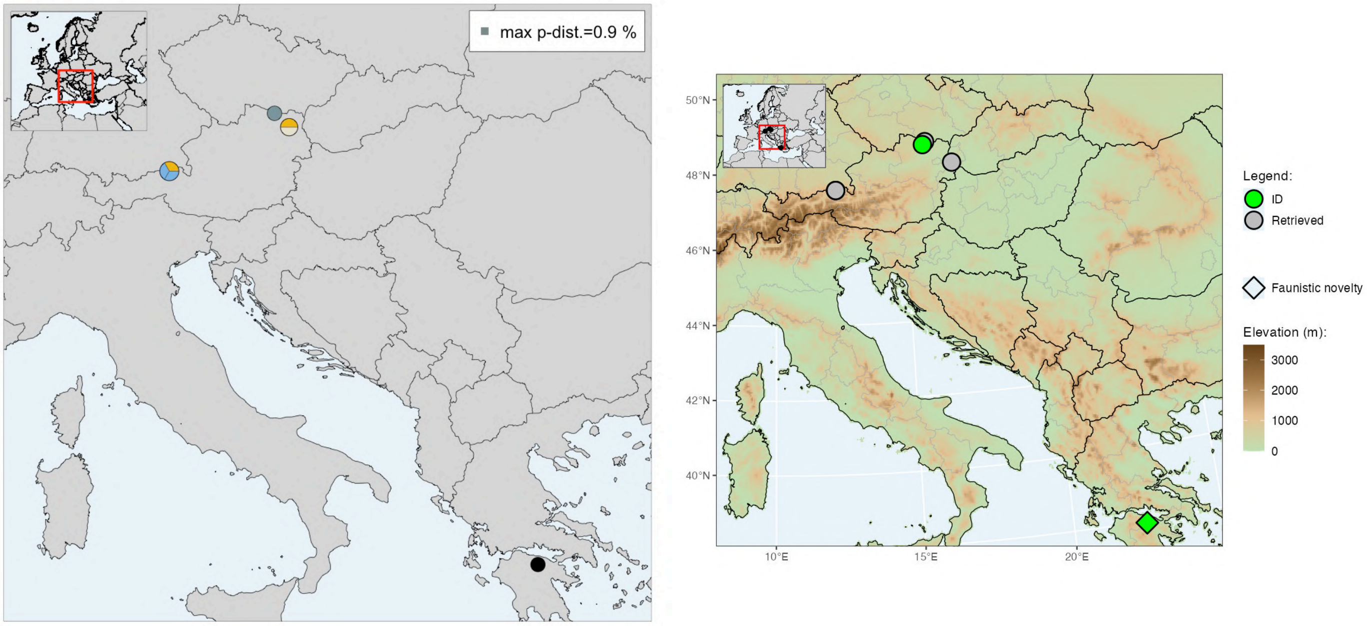

Figure 307: Genetic diversity map of *Lasius austriacus* Schlick-Steiner, 2003. Nearby localities of sequenced specimens are merged in pies (left). Colours match the bidimensional colour space of the PCoA projection (Fig. 307 left) of p-dist between sequences (dots). Specimen identification (ID or cf.) and source (newly sequenced or retrieved) are represented by colours, while specimen attribute (terra typica, type locality, type specimen or faunistic novelty) is represented by the shape (right). Sequences: ID = 7, cf. = 0; maximum p-distance: strict = NA, less strict = 0.9 %.

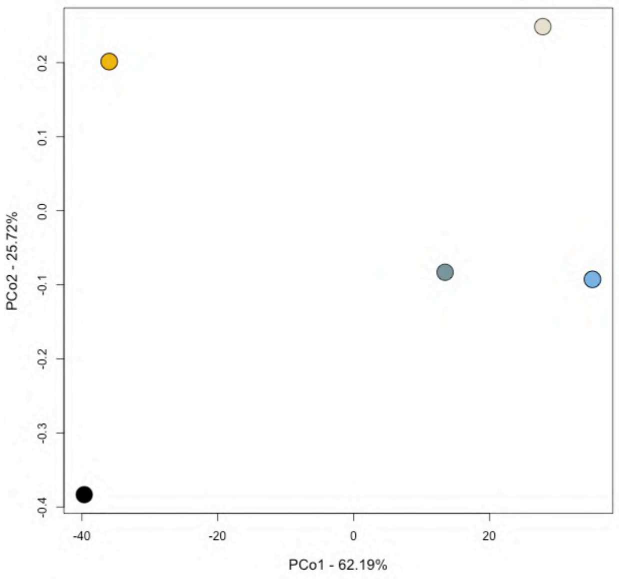

Figure 308: PCoA based on pairwise p-distances between *Lasius austriacus* sequences (left). Colours match a bidimensional colour space. Haplotype network analysis of *Lasius austriacus* was not possible.

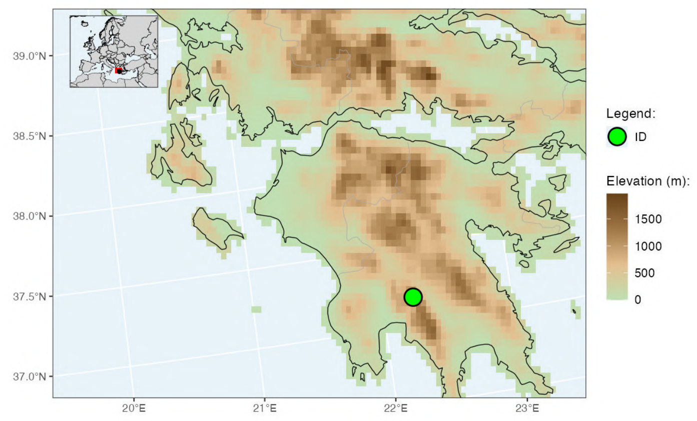

Figure 309: Map of *Lasius balcanicus* Seifert, 1988. Due to the presence of a single sequence, the genetic diversity map and the PCoA projection were not done. Specimen identification (ID or cf.) and source (newly sequenced or retrieved) are represented by colours, while specimen attribute (terra typica, type locality, type specimen or faunistic novelty) is represented by the shape. Sequences: ID = 1, cf. = 0; maximum p-distance: strict = NA, less strict = NA.

Haplotype network analysis of *Lasius balcanicus* was not possible.

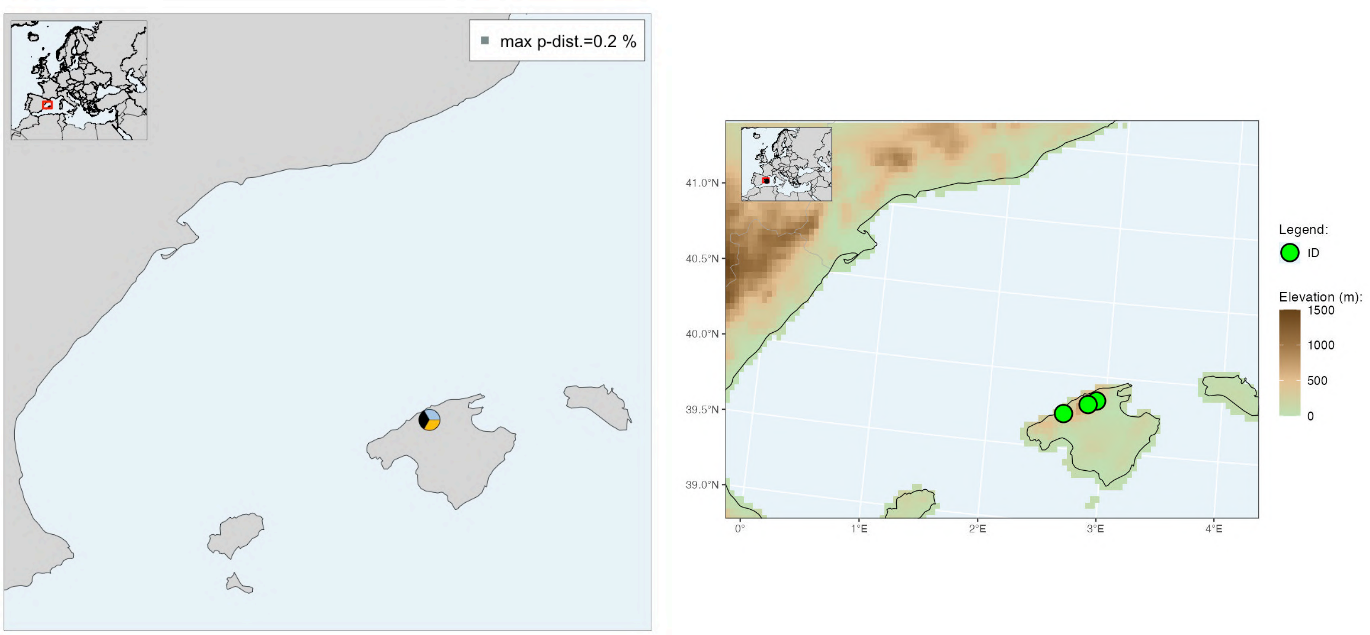

Figure 310: Genetic diversity map of *Lasius balearicus* Talavera, Espadaler & Vila, 2014. Nearby localities of sequenced specimens are merged in pies (left). Colours match the bidimensional colour space of the PCoA projection (Fig. 310 left) of p-dist between sequences (dots). Specimen identification (ID or cf.) and source (newly sequenced or retrieved) are represented by colours, while specimen attribute (terra typica, type locality, type specimen or faunistic novelty) is represented by the shape (right). Sequences: ID = 3, cf. = 0; maximum p-distance: strict = 0.2 %, less strict = 0.2 %.

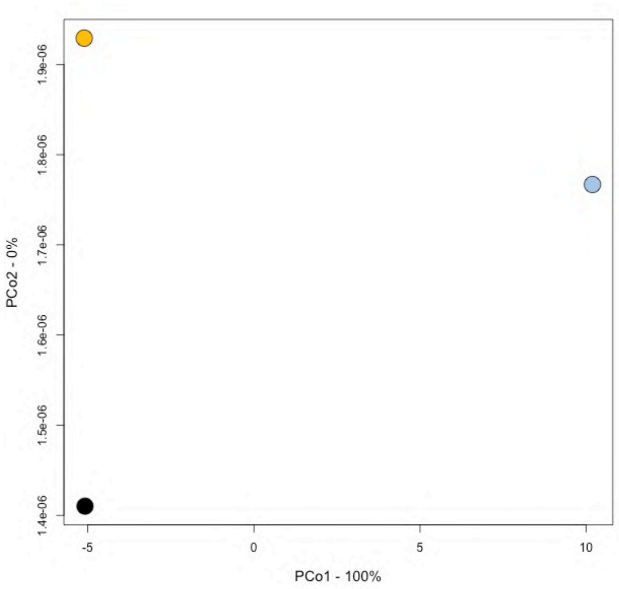

Figure 311: PCoA based on pairwise p-distances between *Lasius balearicus* sequences (left). Colours match a bidimensional colour space. Haplotype network analysis of *Lasius balearicus* was not possible.

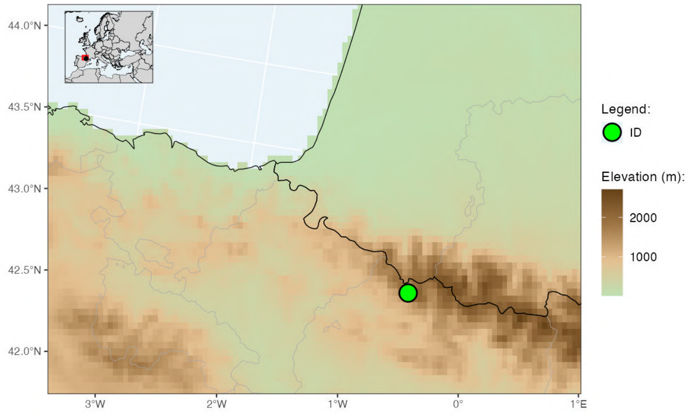

Figure 312: Map of *Lasius bicornis* (Foerster, 1850). Due to the presence of a single sequence, the genetic diversity map and the PCoA projection were not done. Specimen identification (ID or cf.) and source (newly sequenced or retrieved) are represented by colours, while specimen attribute (terra typica, type locality, type specimen or faunistic novelty) is represented by the shape. Sequences: ID = 1, cf. = 0; maximum p-distance: strict = NA, less strict = NA.

Haplotype network analysis of *Lasius bicornis* was not possible.

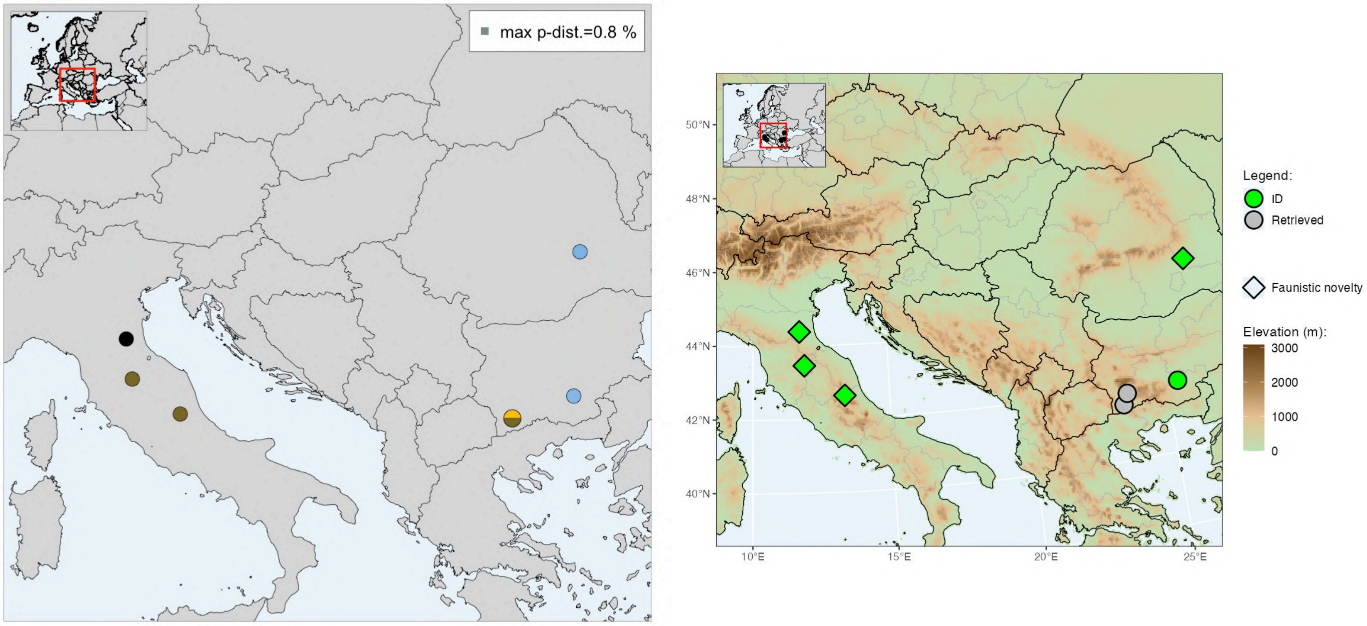

Figure 313: Genetic diversity map of *Lasius bombycinus* Seifert & Galkowski, 2016. Nearby localities of sequenced specimens are merged in pies (left). Colours match the bidimensional colour space of the PCoA projection (Fig. 313 left) of p-dist between sequences (dots). Specimen identification (ID or cf.) and source (newly sequenced or retrieved) are represented by colours, while specimen attribute (terra typica, type locality, type specimen or faunistic novelty) is represented by the shape (right). Sequences: ID = 7, cf. = 0; maximum p-distance: strict = 0.8 %, less strict = 0.8 %.

The species is reported for the first time in Italy and Romania.

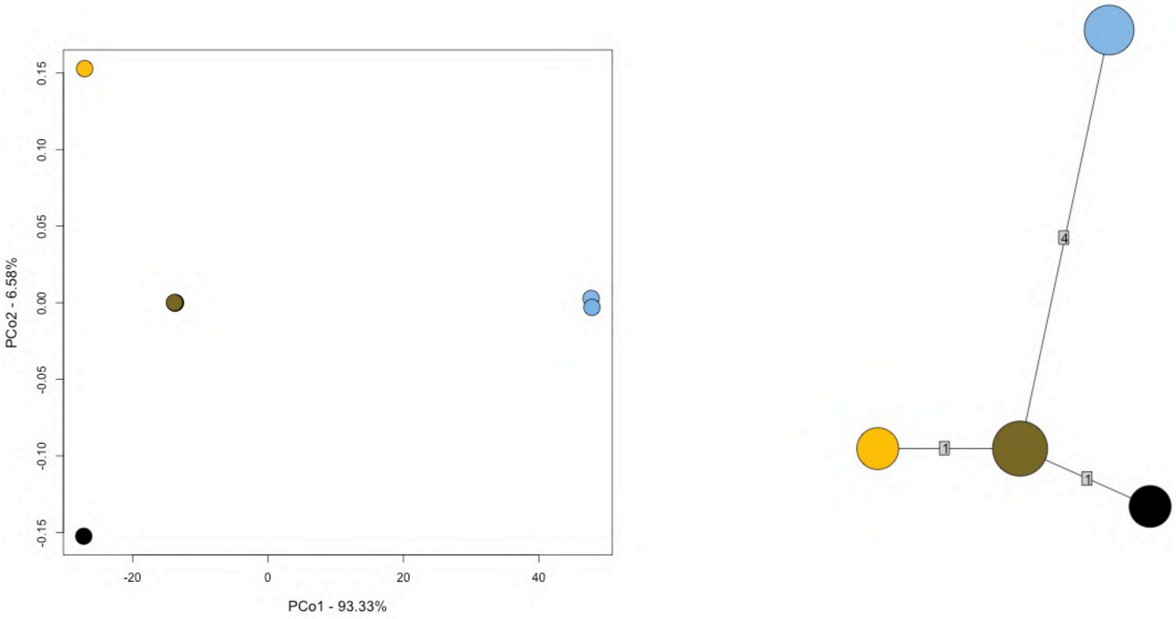

Figure 314: PCoA based on pairwise p-distances between *Lasius bombycinus* sequences (left). Colours match a bidimensional colour space. Haplotype network of *Lasius bombycinus* (right). Sequences > 599 bp: ID = 7, cf. = 0.

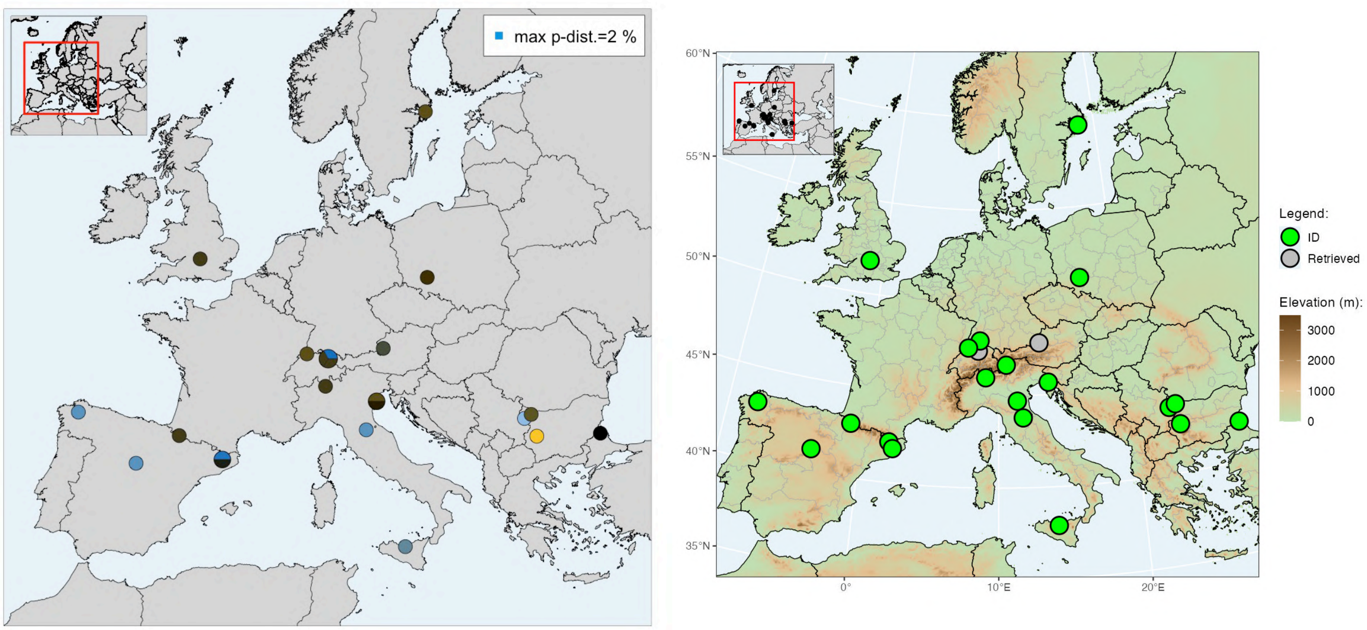

Figure 315: Genetic diversity map of *Lasius brunneus* (Latreille, 1798). Nearby localities of sequenced specimens are merged in pies (left). Colours match the bidimensional colour space of the PCoA projection (Fig. 315 left) of p-dist between sequences (dots). Specimen identification (ID or cf.) and source (newly sequenced or retrieved) are represented by colours, while specimen attribute (terra typica, type locality, type specimen or faunistic novelty) is represented by the shape (right). Sequences: ID = 22, cf. = 0; maximum p-distance: strict = 2 %, less strict = 2 %.

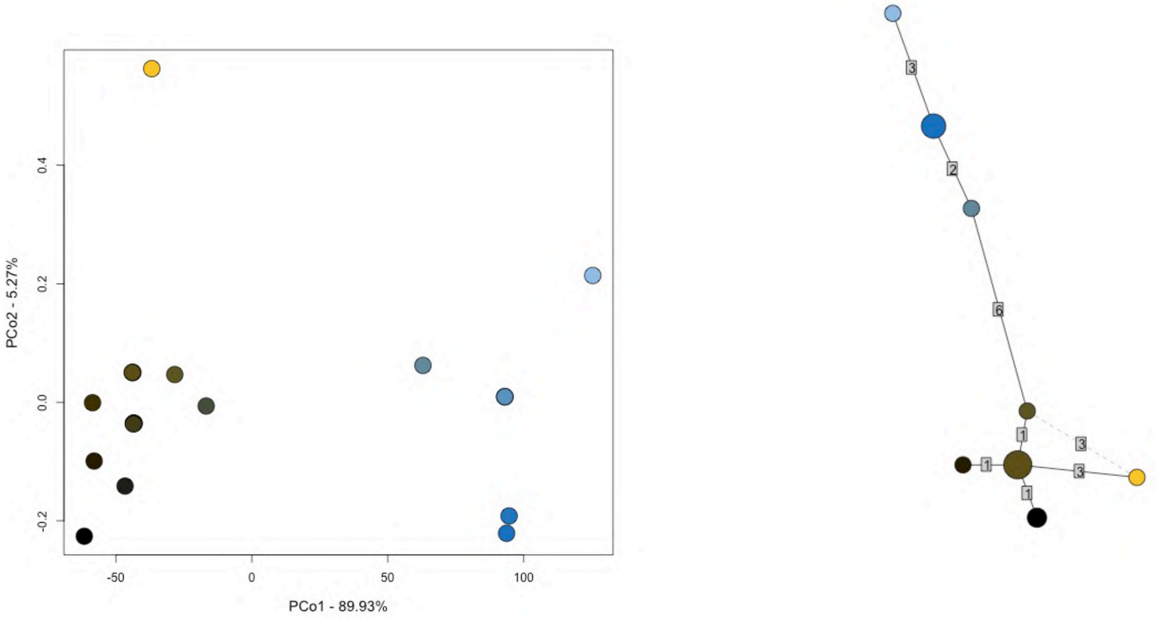

Figure 316: PCoA based on pairwise p-distances between *Lasius brunneus* sequences (left). Colours match a bidimensional colour space. Haplotype network of *Lasius brunneus* (right). Sequences > 599 bp: ID = 21, cf. = 0.

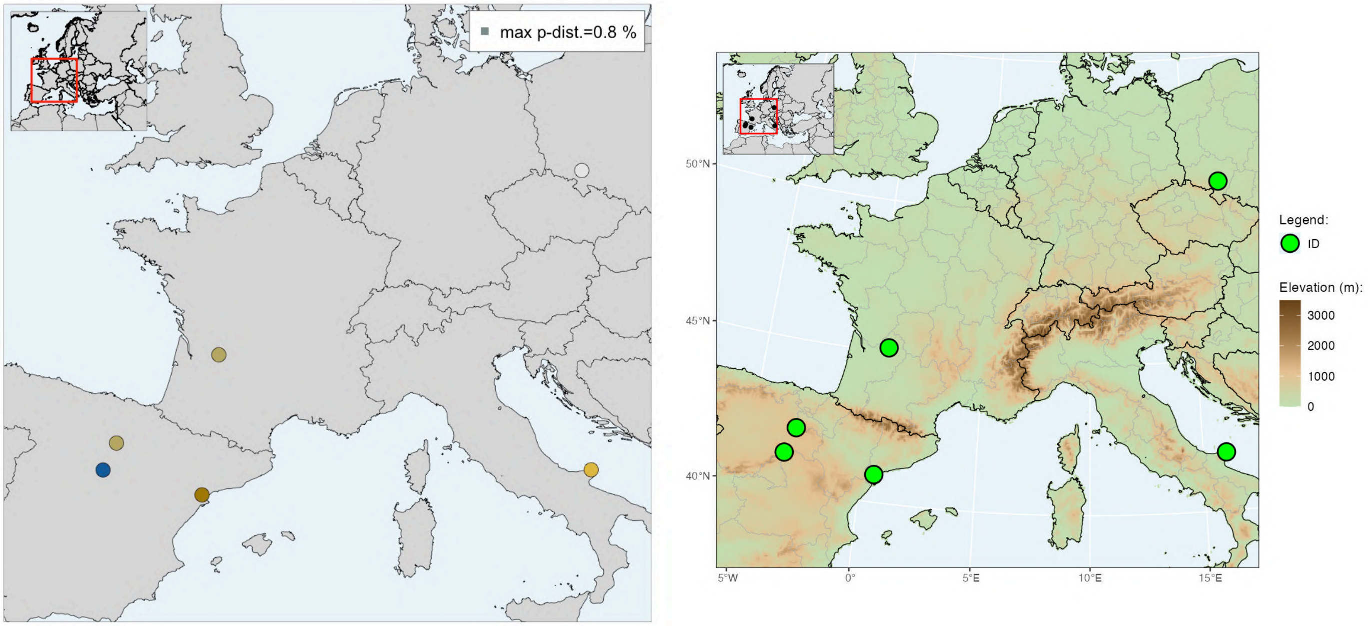

Figure 317: Genetic diversity map of *Lasius carniolicus* Mayr, 1861. Nearby localities of sequenced specimens are merged in pies (left). Colours match the bidimensional colour space of the PCoA projection (Fig. 317 left) of p-dist between sequences (dots). Specimen identification (ID or cf.) and source (newly sequenced or retrieved) are represented by colours, while specimen attribute (terra typica, type locality, type specimen or faunistic novelty) is represented by the shape (right). Sequences: ID = 6, cf. = 0; maximum p-distance: strict = 0.6 %, less strict = 0.8 %.

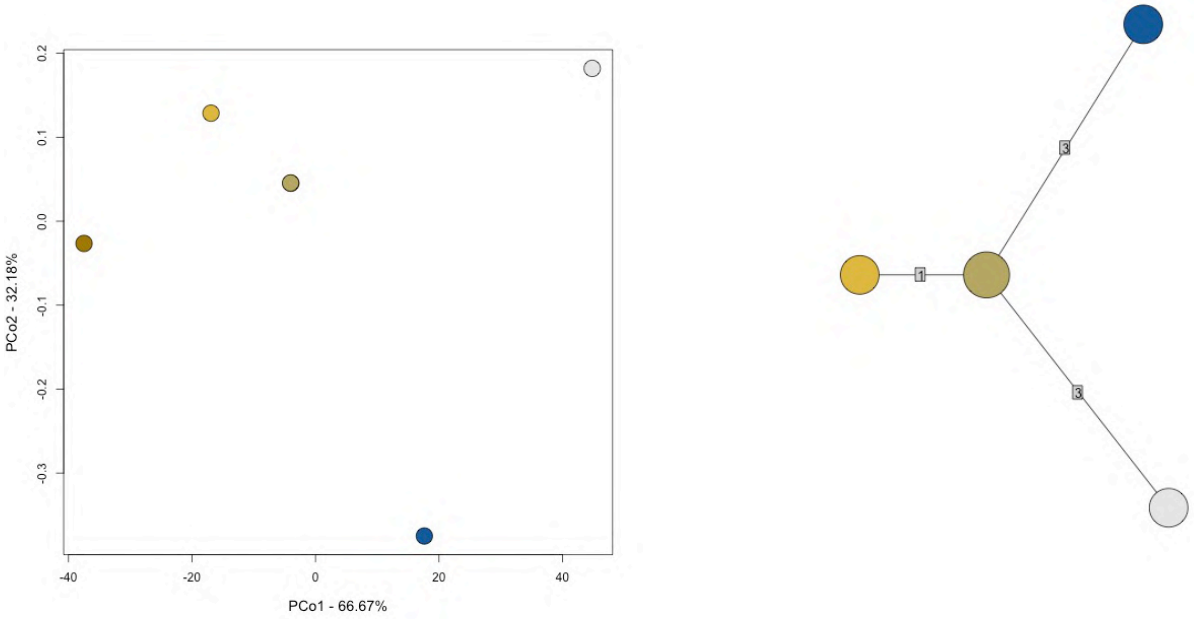

Figure 318: PCoA based on pairwise p-distances between *Lasius carniolicus* sequences (left). Colours match a bidimensional colour space. Haplotype network of *Lasius carniolicus* (right). Sequences > 599 bp: ID = 5, cf. = 0.

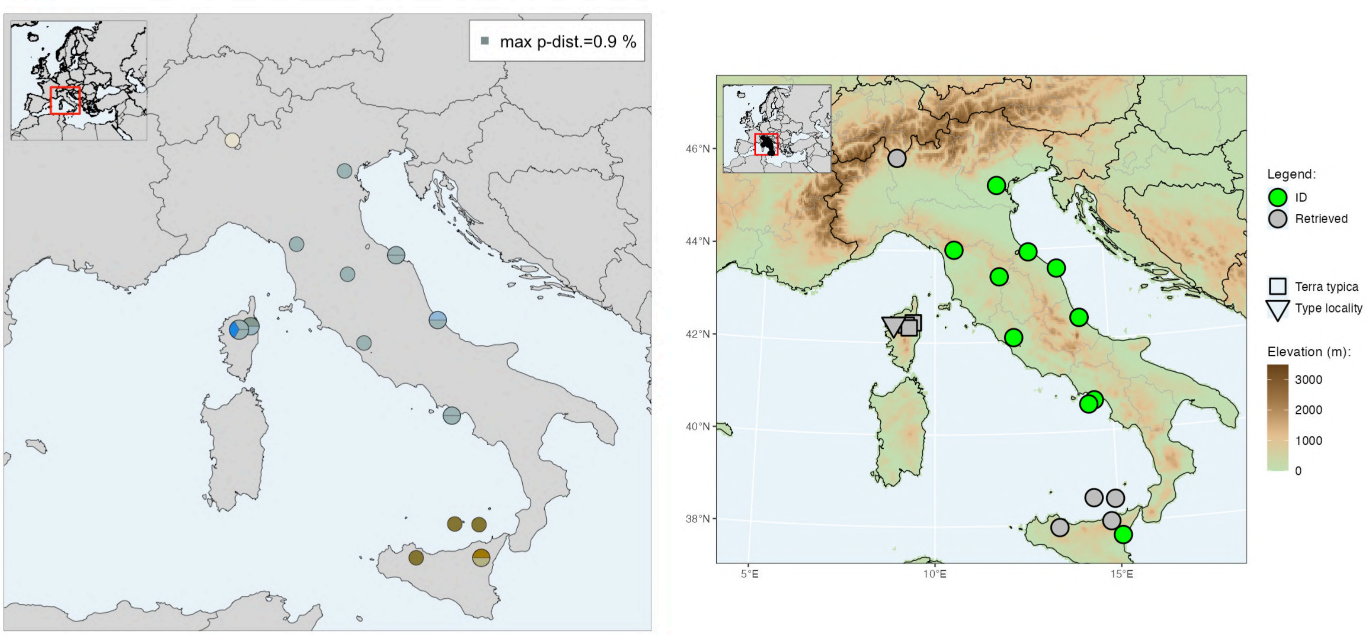

Figure 319: Genetic diversity map of *Lasius casevitzi* Seifert & Galkowski, 2016. Nearby localities of sequenced specimens are merged in pies (left). Colours match the bidimensional colour space of the PCoA projection (Fig. 319 left) of p-dist between sequences (dots). Specimen identification (ID or cf.) and source (newly sequenced or retrieved) are represented by colours, while specimen attribute (terra typica, type locality, type specimen or faunistic novelty) is represented by the shape (right). Sequences: ID = 21, cf. = 0; maximum p-distance: strict = 0.8 %, less strict = 0.9 %.

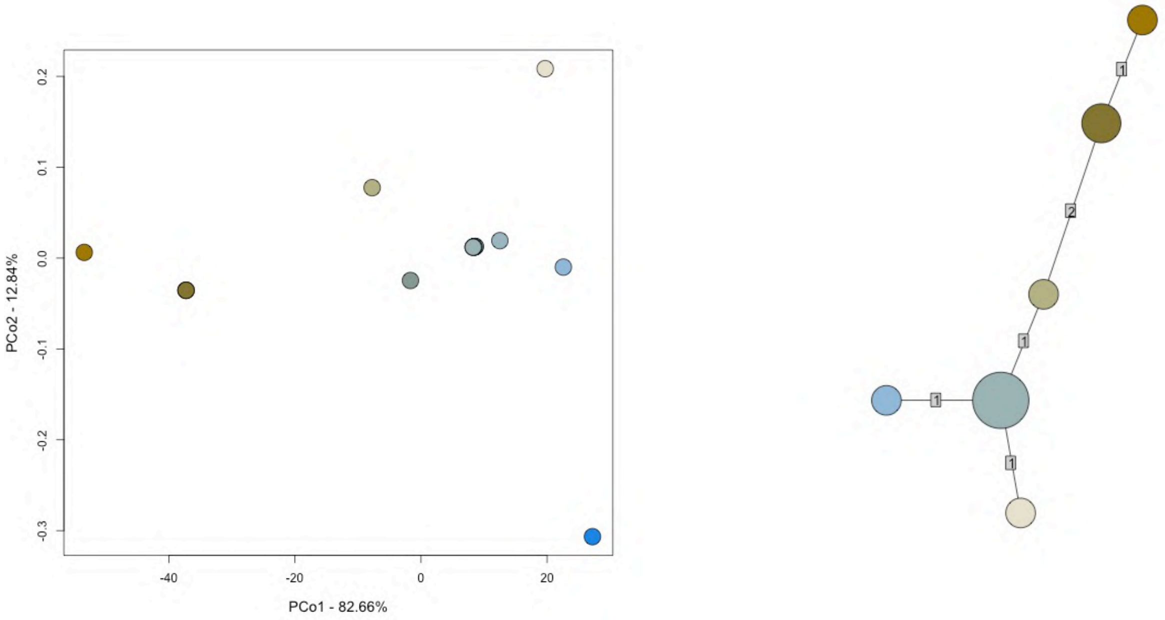

Figure 320: PCoA based on pairwise p-distances between *Lasius casevitzi* sequences (left). Colours match a bidimensional colour space. Haplotype network of *Lasius casevitzi* (right). Sequences > 599 bp: ID = 20, cf. = 0.

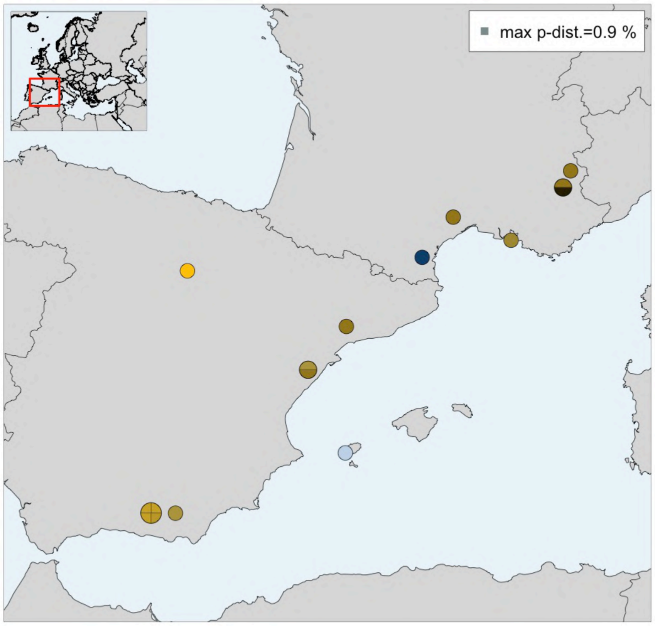

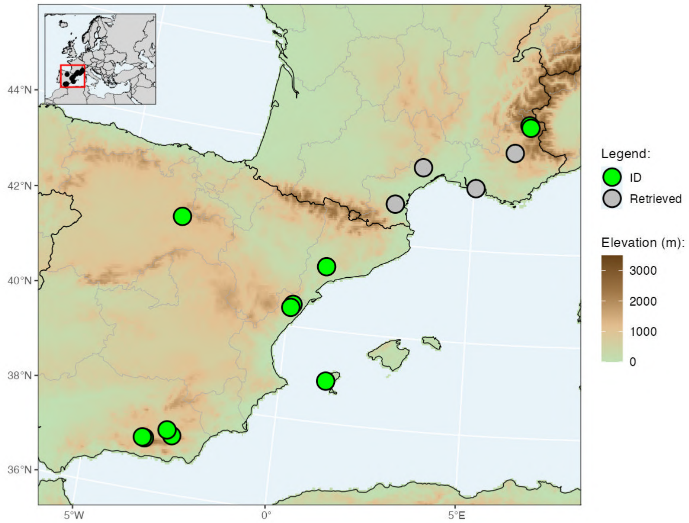

Figure 321: Genetic diversity map of *Lasius cinereus* Seifert, 1992. Nearby localities of sequenced specimens are merged in pies (left). Colours match the bidimensional colour space of the PCoA projection (Fig. 321 left) of p-dist between sequences (dots). Specimen identification (ID or cf.) and source (newly sequenced or retrieved) are represented by colours, while specimen attribute (terra typica, type locality, type specimen or faunistic novelty) is represented by the shape (right). Sequences: ID = 16, cf. = 0; maximum p-distance: strict = 0.9 %, less strict = 0.9 %.

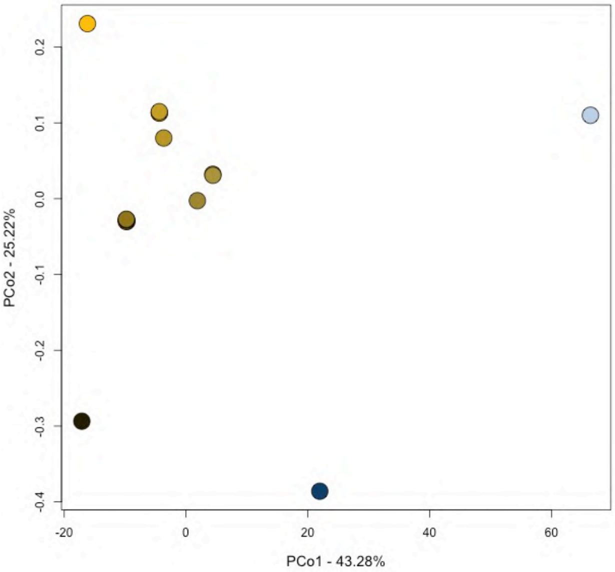

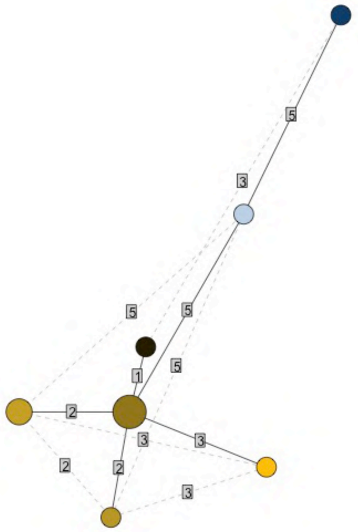

Figure 322: PCoA based on pairwise p-distances between *Lasius cinereus* sequences (left). Colours match a bidimensional colour space. Haplotype network of *Lasius cinereus* (right). Sequences > 599 bp: ID = 16, cf. = 0.

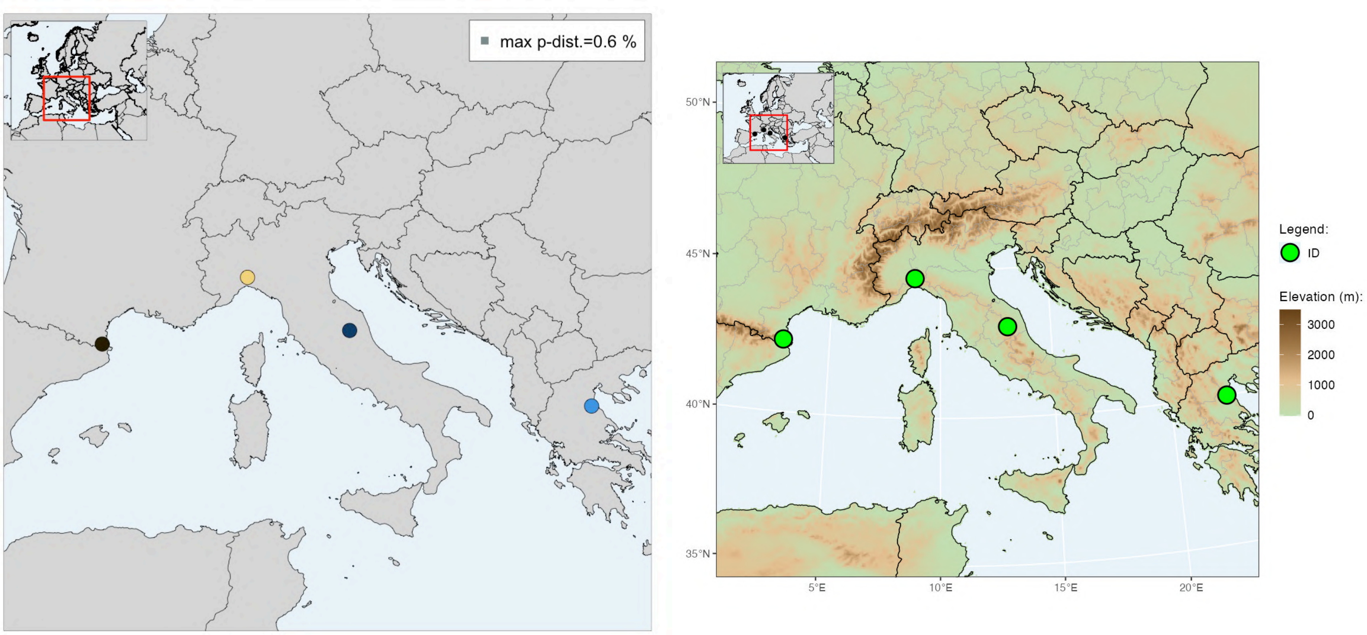

Figure 323: Genetic diversity map of *Lasius citrinus* Emery, 1922. Nearby localities of sequenced specimens are merged in pies (left). Colours match the bidimensional colour space of the PCoA projection (Fig. 323 left) of p-dist between sequences (dots). Specimen identification (ID or cf.) and source (newly sequenced or retrieved) are represented by colours, while specimen attribute (terra typica, type locality, type specimen or faunistic novelty) is represented by the shape (right). Sequences: ID = 4, cf. = 0; maximum p-distance: strict = 0.6 %, less strict = 0.6 %.

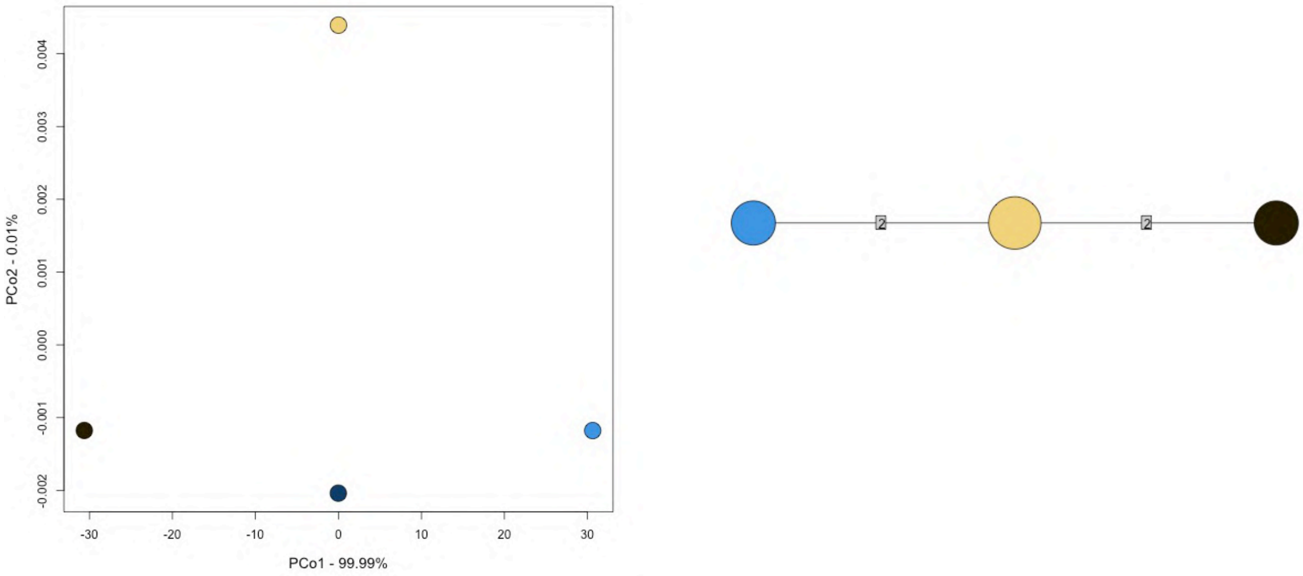

Figure 324: PCoA based on pairwise p-distances between *Lasius citrinus* sequences (left). Colours match a bidimensional colour space. Haplotype network of *Lasius citrinus* (right). Sequences > 599 bp: ID = 4, cf. = 0.

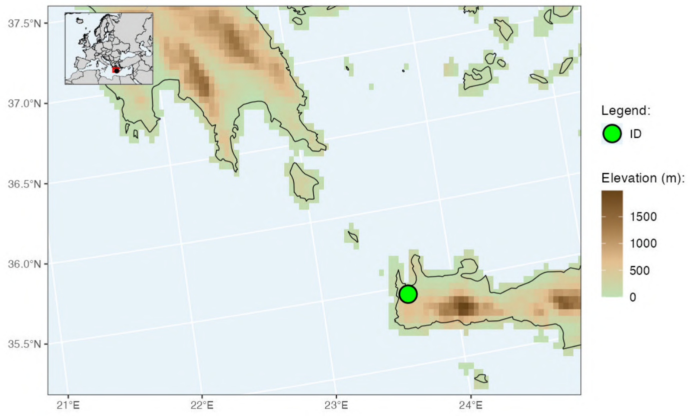

Figure 325: Map of *Lasius creticus* Seifert, 2020. Due to the presence of a single sequence, the genetic diversity map and the PCoA projection were not done. Specimen identification (ID or cf.) and source (newly sequenced or retrieved) are represented by colours, while specimen attribute (terra typica, type locality, type specimen or faunistic novelty) is represented by the shape. Sequences: ID = 1, cf. = 0; maximum p-distance: strict = NA, less strict = NA.

Haplotype network analysis of *Lasius creticus* was not possible.

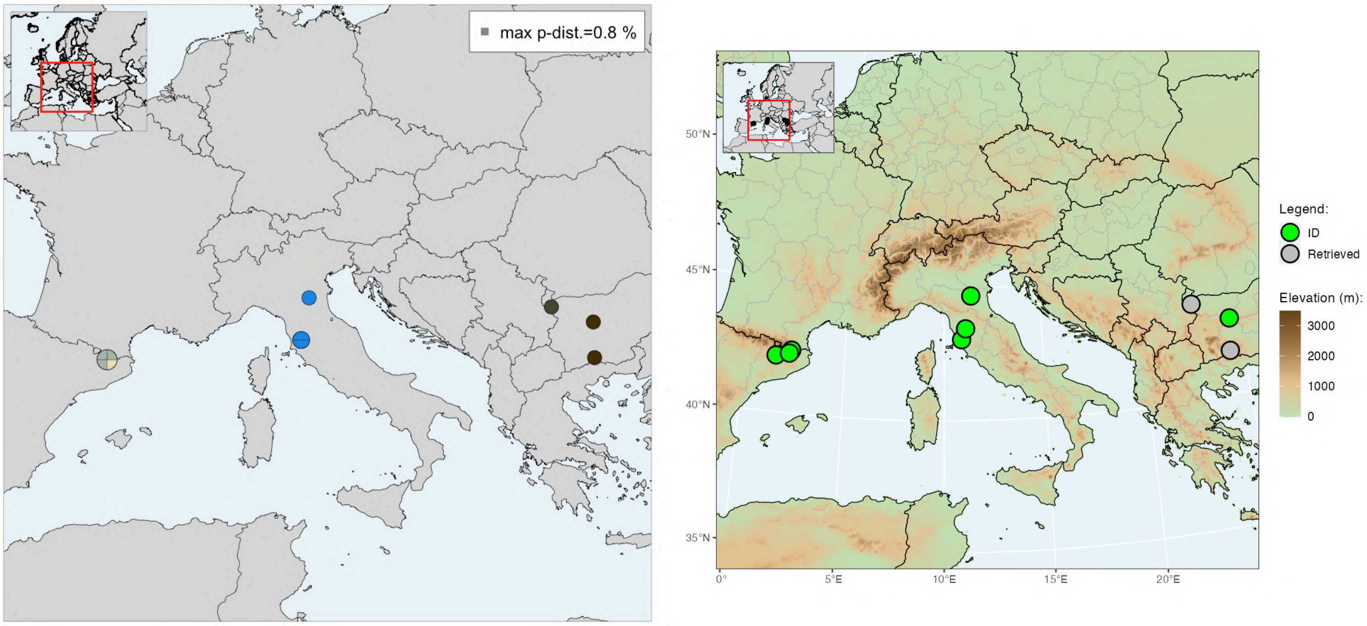

Figure 326: Genetic diversity map of *Lasius distinguendus* (Emery, 1916). Nearby localities of sequenced specimens are merged in pies (left). Colours match the bidimensional colour space of the PCoA projection (Fig. 326 left) of p-dist between sequences (dots). Specimen identification (ID or cf.) and source (newly sequenced or retrieved) are represented by colours, while specimen attribute (terra typica, type locality, type specimen or faunistic novelty) is represented by the shape (right). Sequences: ID = 10, cf. = 0; maximum p-distance: strict = 0.8 %, less strict = 0.8 %.

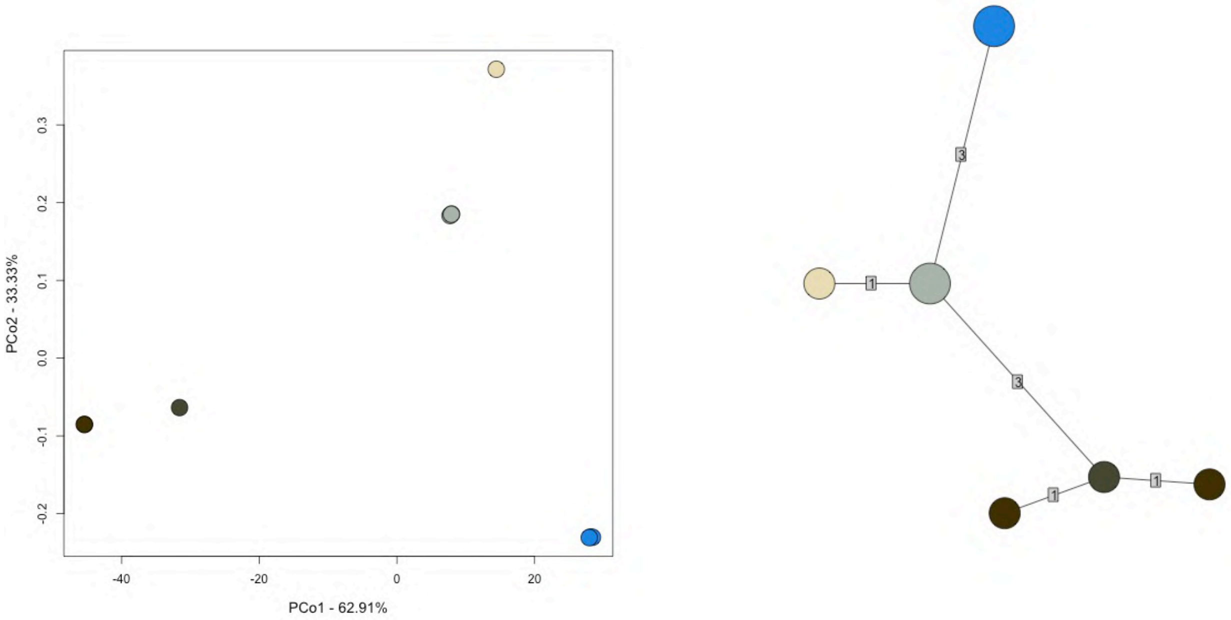

Figure 327: PCoA based on pairwise p-distances between *Lasius distinguendus* sequences (left). Colours match a bidimensional colour space. Haplotype network of *Lasius distinguendus* (right). Sequences > 599 bp: ID = 10, cf. = 0.

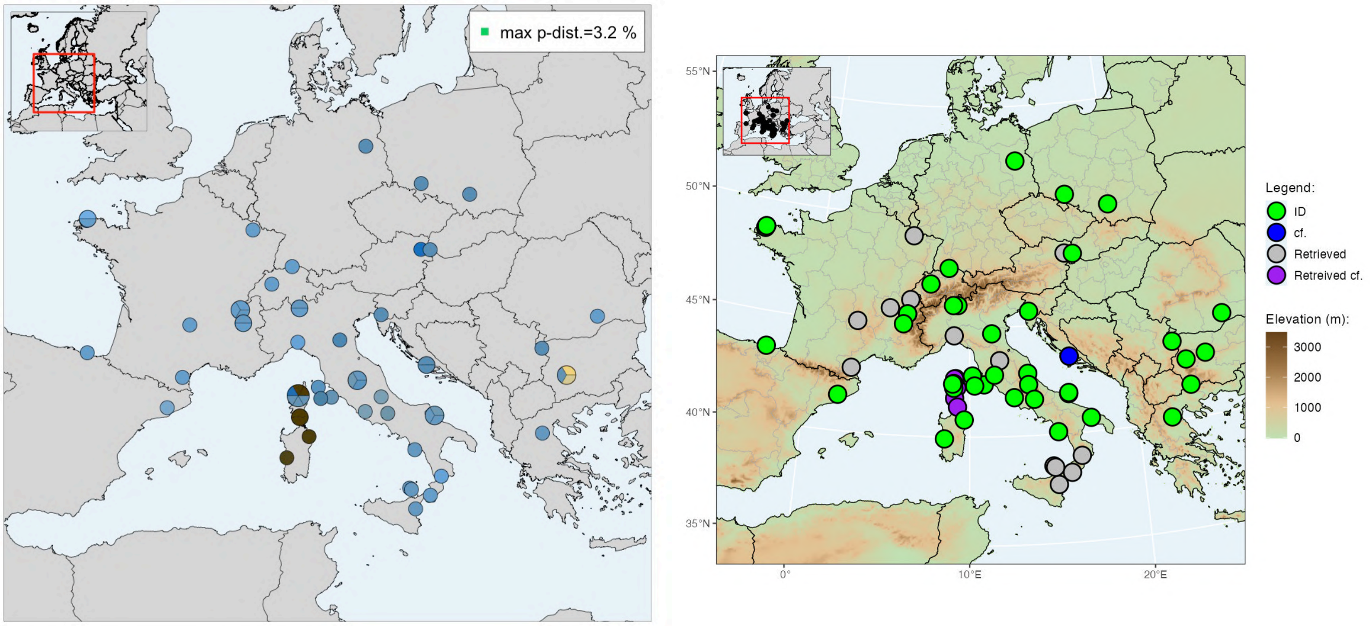

Figure 328: Genetic diversity map of *Lasius emarginatus* (Olivier, 1792). Nearby localities of sequenced specimens are merged in pies (left). Colours match the bidimensional colour space of the PCoA projection (Fig. 328 left) of p-dist between sequences (dots). Specimen identification (ID or cf.) and source (newly sequenced or retrieved) are represented by colours, while specimen attribute (terra typica, type locality, type specimen or faunistic novelty) is represented by the shape (right). Sequences: ID = 55, cf. = 6; maximum p-distance: strict = 2.9 %, less strict = 3.2 %.

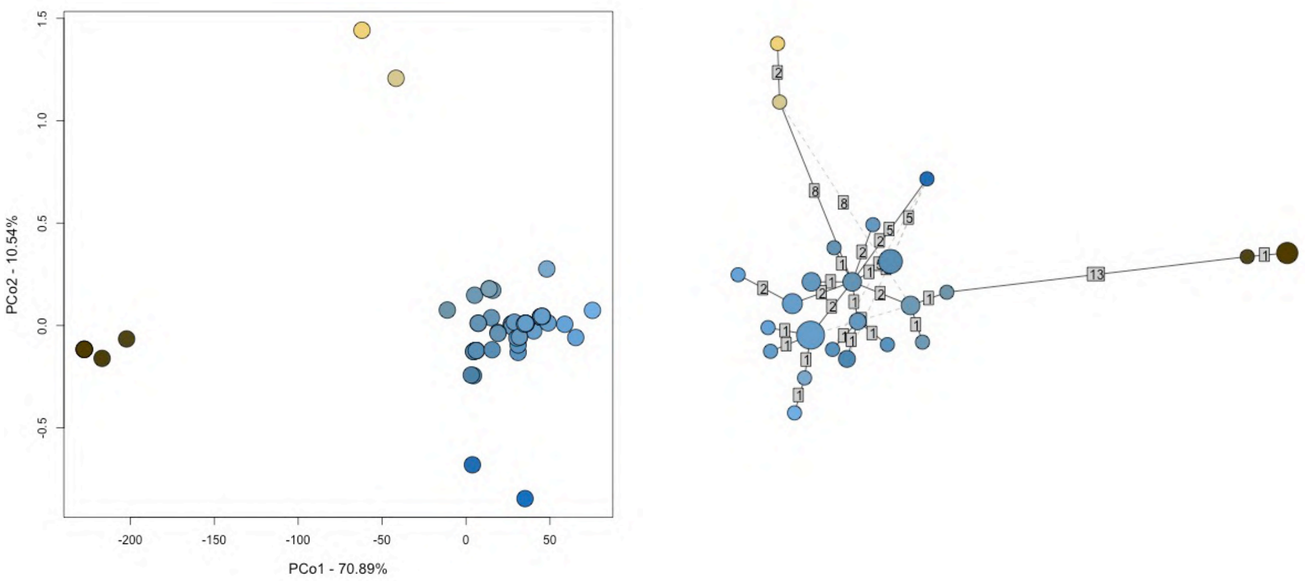

Figure 329: PCoA based on pairwise p-distances between *Lasius emarginatus* sequences (left). Colours match a bidimensional colour space. Haplotype network of *Lasius emarginatus* (right). Sequences > 599 bp: ID = 54, cf. = 6.

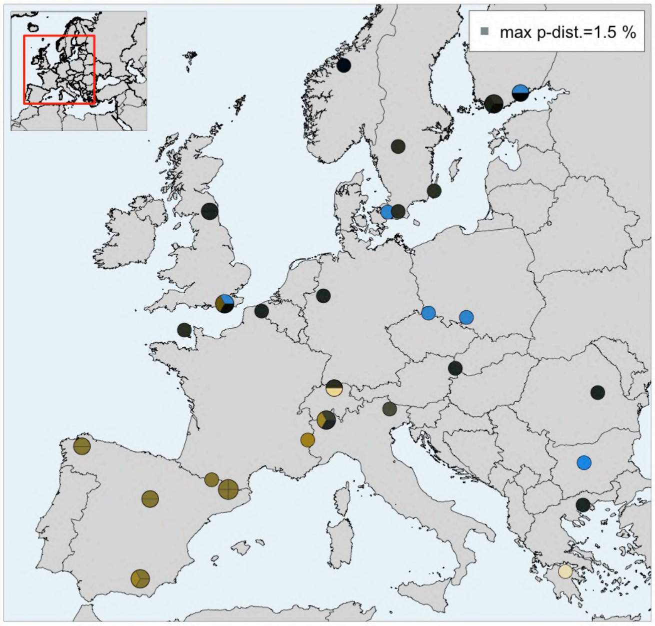

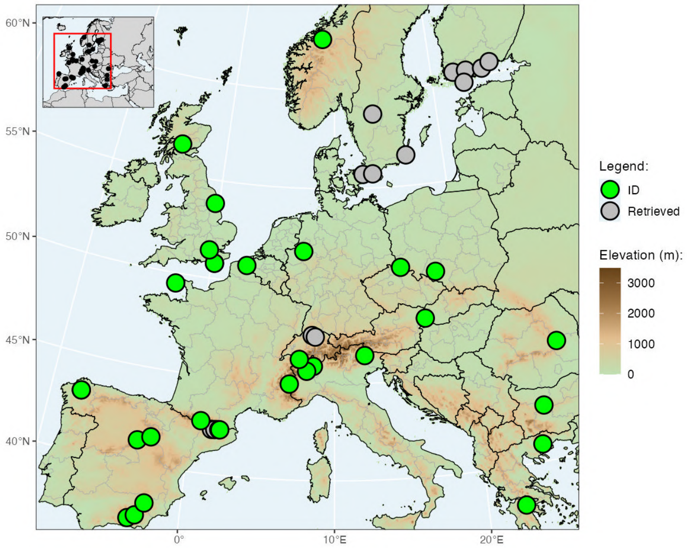

Figure 330: Genetic diversity map of *Lasius flavus* (Fabricius, 1782). Nearby localities of sequenced specimens are merged in pies (left). Colours match the bidimensional colour space of the PCoA projection (Fig. 330 left) of p-dist between sequences (dots). Specimen identification (ID or cf.) and source (newly sequenced or retrieved) are represented by colours, while specimen attribute (terra typica, type locality, type specimen or faunistic novelty) is represented by the shape (right). Sequences: ID = 44, cf. = 0; maximum p-distance: strict = 1.5 %, less strict = 1.5 %.

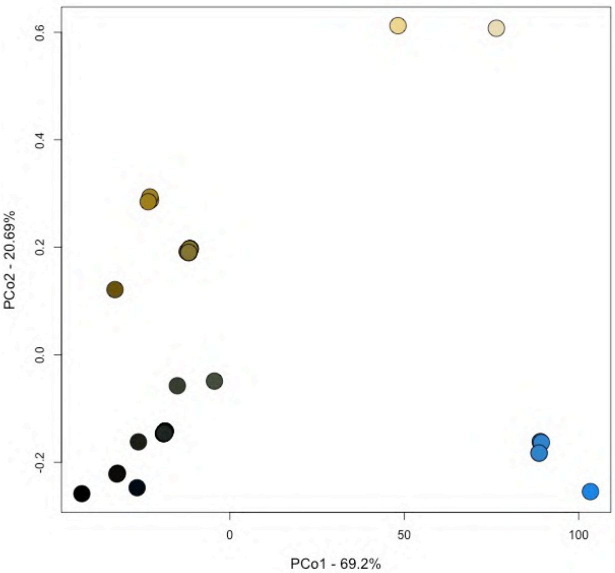

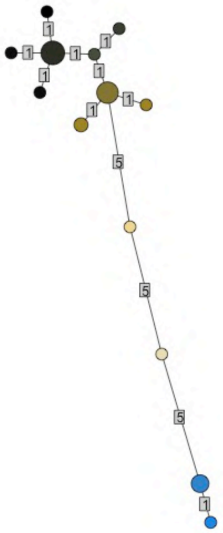

Figure 331: PCoA based on pairwise p-distances between *Lasius flavus* sequences (left). Colours match a bidimensional colour space. Haplotype network of *Lasius flavus* (right). Sequences > 599 bp: ID = 43, cf. = 0.

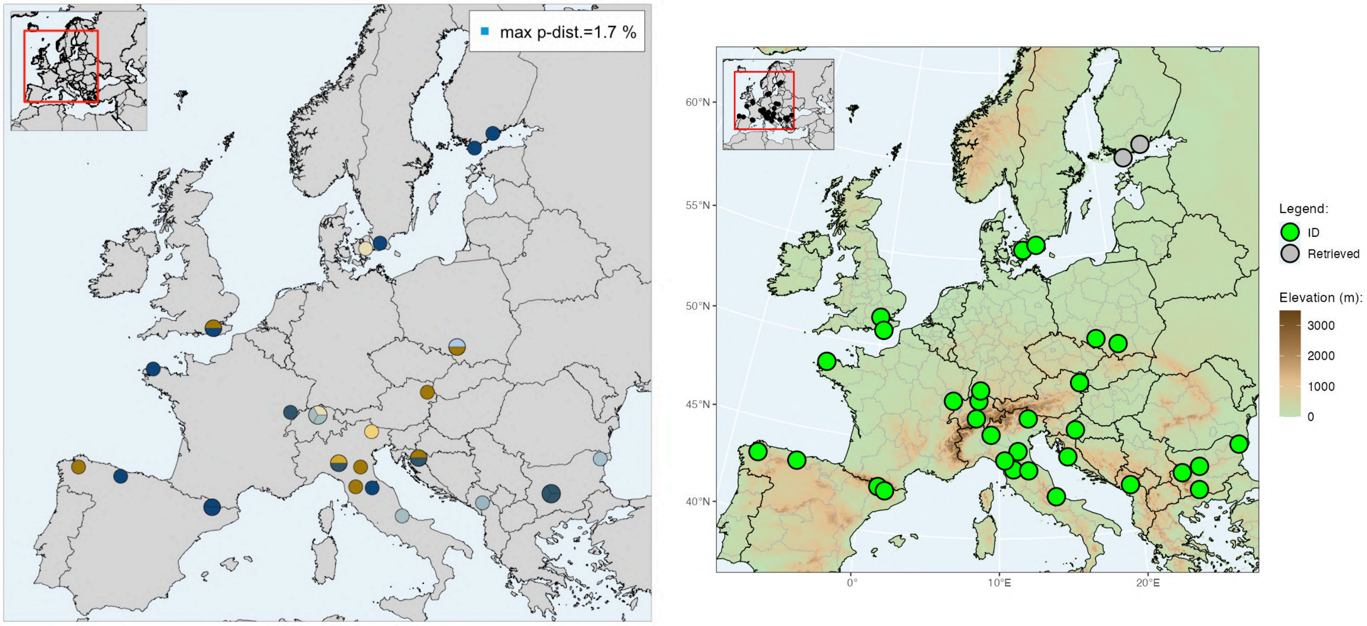

Figure 332: Genetic diversity map of *Lasius fuliginosus* (Latreille, 1798). Nearby localities of sequenced specimens are merged in pies (left). Colours match the bidimensional colour space of the PCoA projection (Fig. 332 left) of p-dist between sequences (dots). Specimen identification (ID or cf.) and source (newly sequenced or retrieved) are represented by colours, while specimen attribute (terra typica, type locality, type specimen or faunistic novelty) is represented by the shape (right). Sequences: ID = 32, cf. = 0; maximum p-distance: strict = 1.7 %, less strict = 1.7 %.

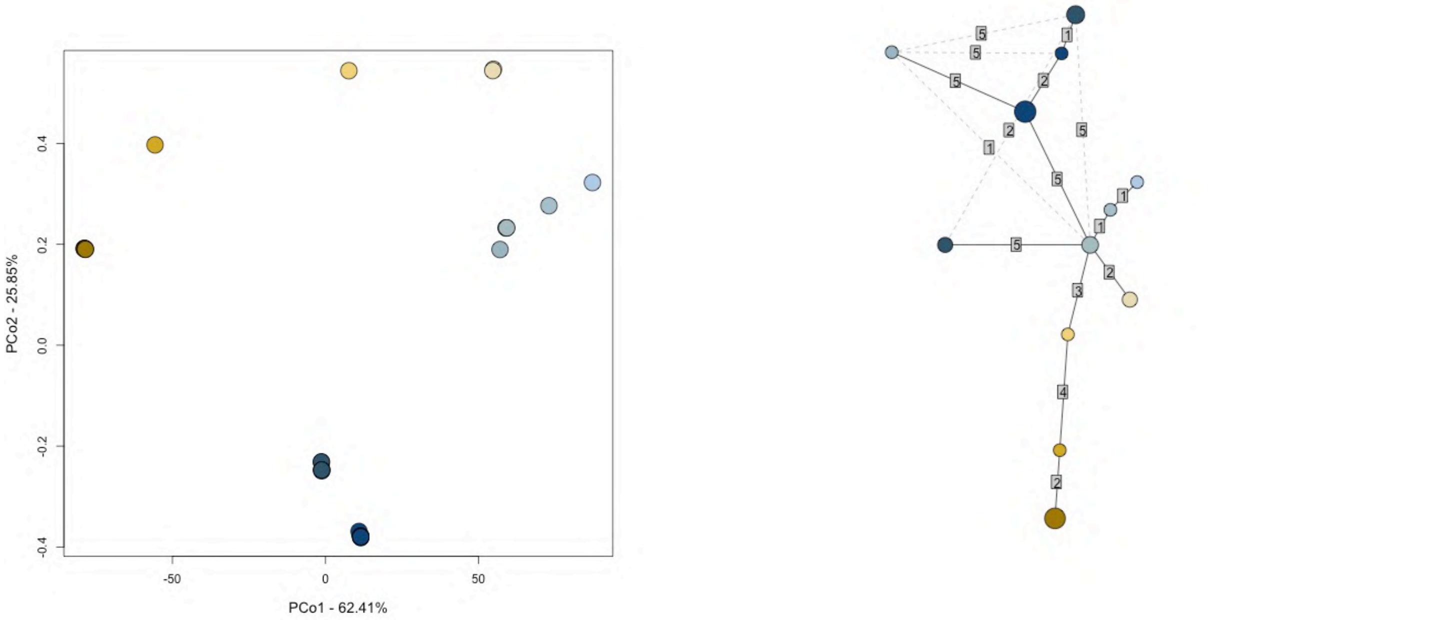

Figure 333: PCoA based on pairwise p-distances between *Lasius fuliginosus* sequences (left). Colours match a bidimensional colour space. Haplotype network of *Lasius fuliginosus* (right). Sequences > 599 bp: ID = 32, cf. = 0.

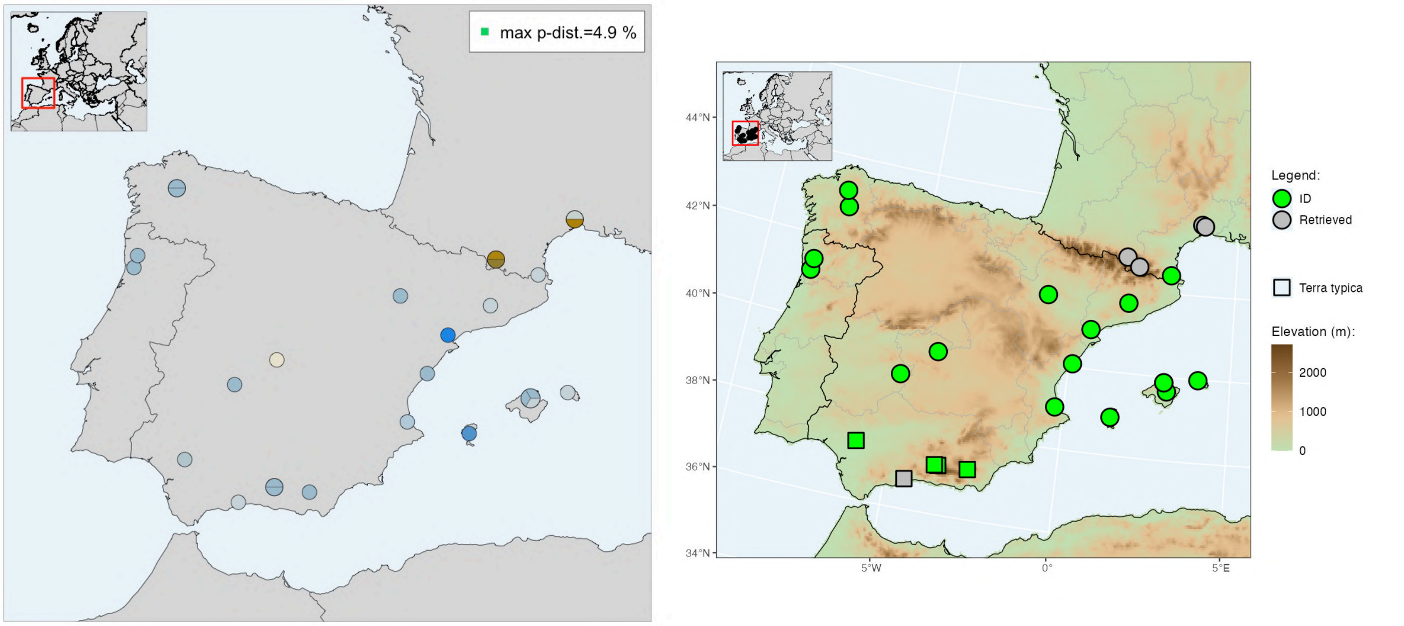

Figure 334: Genetic diversity map of *Lasius grandis* Forel, 1909. Nearby localities of sequenced specimens are merged in pies (left). Colours match the bidimensional colour space of the PCoA projection (Fig. 334 left) of p-dist between sequences (dots). Specimen identification (ID or cf.) and source (newly sequenced or retrieved) are represented by colours, while specimen attribute (terra typica, type locality, type specimen or faunistic novelty) is represented by the shape (right). Sequences: ID = 26, cf. = 0; maximum p-distance: strict = 4.9 %, less strict = 4.9 %.

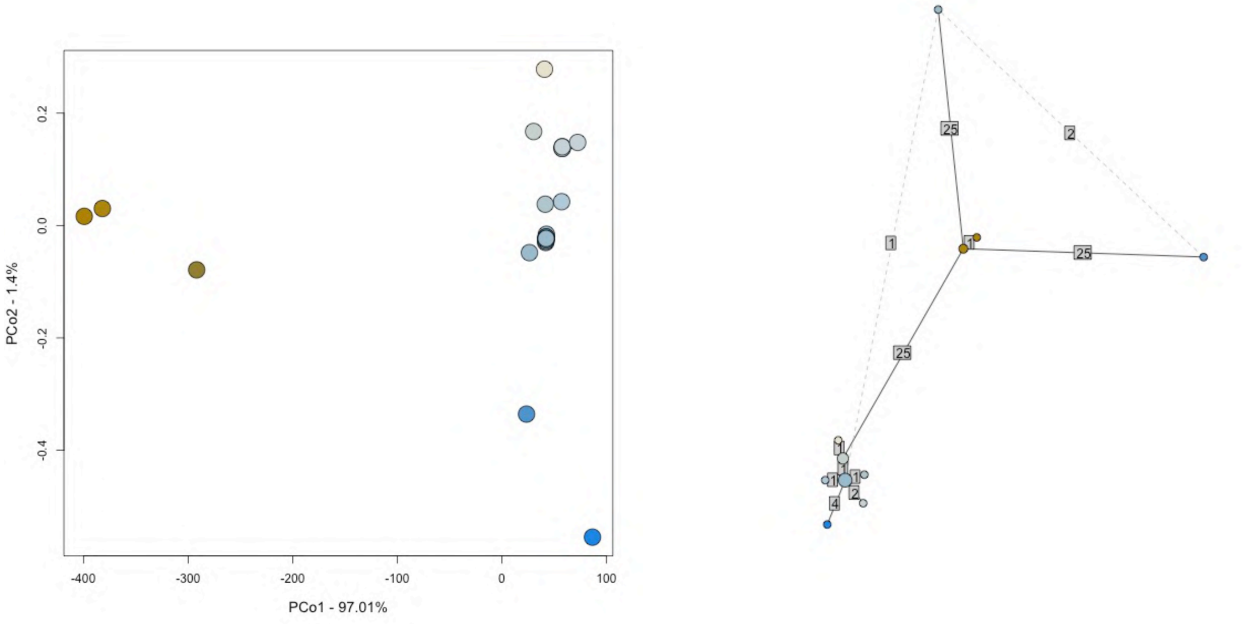

Figure 335: PCoA based on pairwise p-distances between *Lasius grandis* sequences (left). Colours match a bidimensional colour space. Haplotype network of *Lasius grandis* (right). Sequences > 599 bp: ID = 26, cf. = 0.

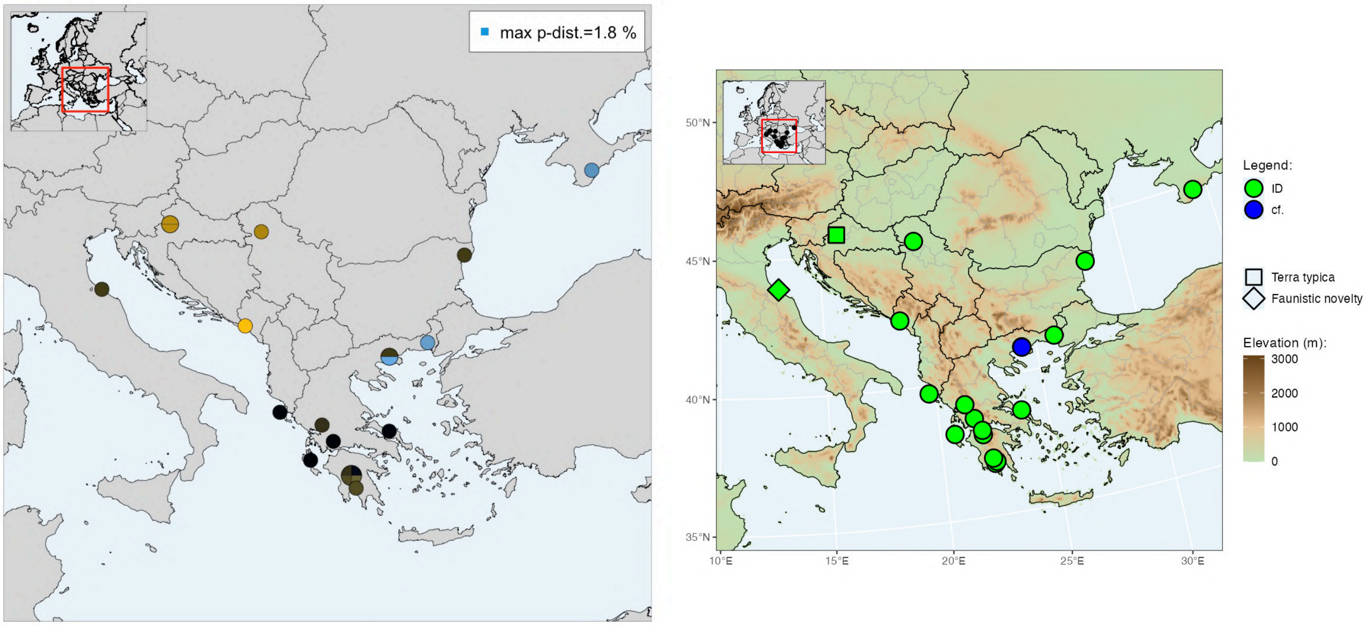

Figure 336: Genetic diversity map of *Lasius illyricus* Zimmermann, 1935. Nearby localities of sequenced specimens are merged in pies (left). Colours match the bidimensional colour space of the PCoA projection (Fig. 336 left) of p-dist between sequences (dots). Specimen identification (ID or cf.) and source (newly sequenced or retrieved) are represented by colours, while specimen attribute (terra typica, type locality, type specimen or faunistic novelty) is represented by the shape (right). Sequences: ID = 19, cf. = 1; maximum p-distance: strict = 1.7 %, less strict = 1.8 %.

The species is reported for the first time in Italy.

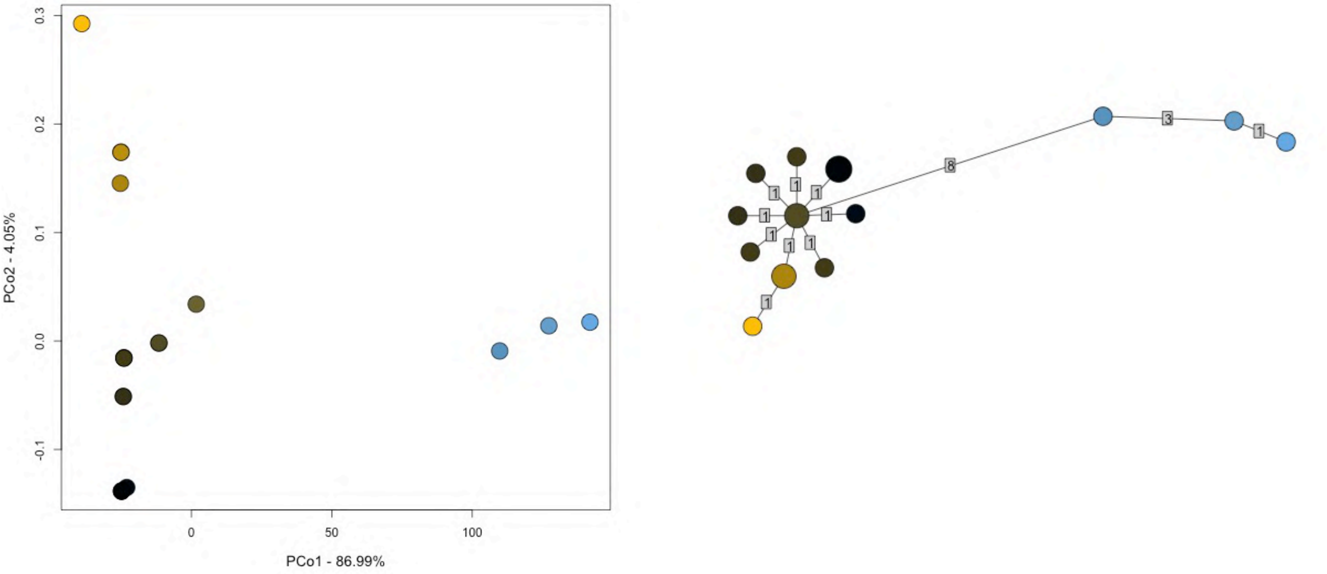

Figure 337: PCoA based on pairwise p-distances between *Lasius illyricus* sequences (left). Colours match a bidimensional colour space. Haplotype network of *Lasius illyricus* (right). Sequences > 599 bp: ID = 19, cf. = 1.

Lasius jensi

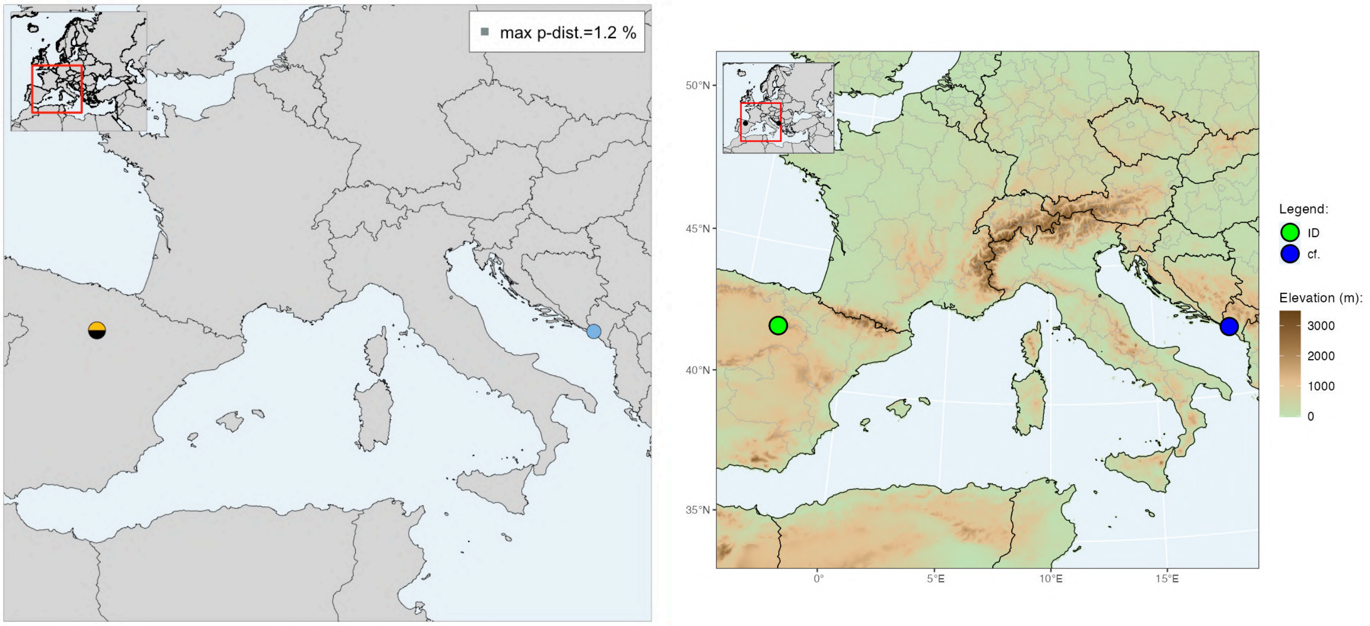

Figure 338: Genetic diversity map of *Lasius jensi* Seifert, 1982. Nearby localities of sequenced specimens are merged in pies (left). Colours match the bidimensional colour space of the PCoA projection (Fig. 338 left) of p-dist between sequences (dots). Specimen identification (ID or cf.) and source (newly sequenced or retrieved) are represented by colours, while specimen attribute (terra typica, type locality, type specimen or faunistic novelty) is represented by the shape (right). Sequences: ID = 2, cf. = 1; maximum p-distance: strict = NA, less strict = 1.2 %.

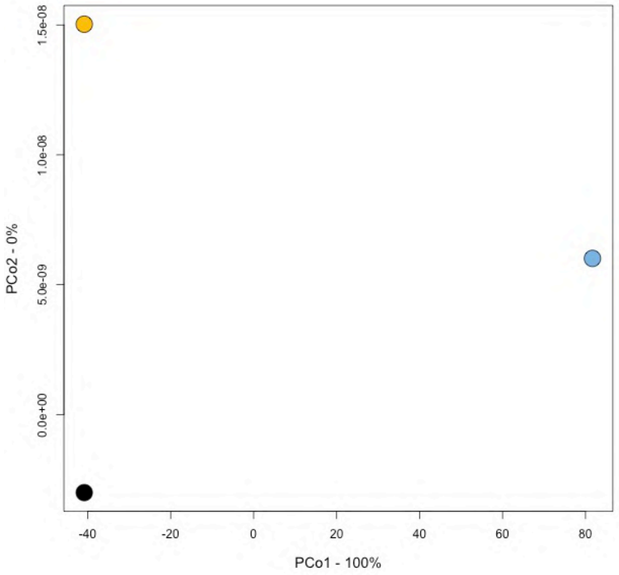

Figure 339: PCoA based on pairwise p-distances between *Lasius jensi* sequences (left). Colours match a bidimensional colour space. Haplotype network analysis of *Lasius jensi* was not possible.

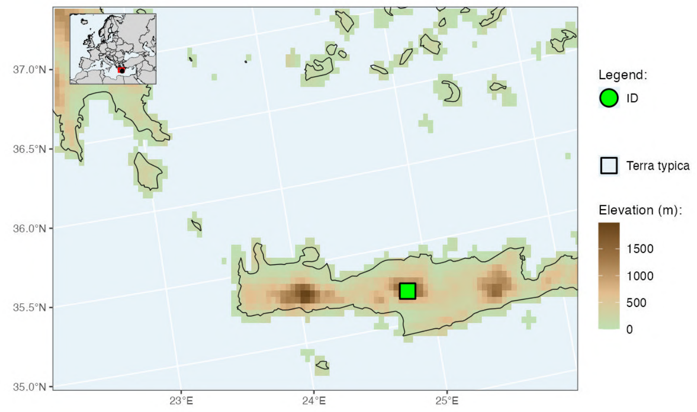

Figure 340: Map of *Lasius kritikos* Seifert, 2020. Due to the presence of a single sequence, the genetic diversity map and the PCoA projection were not done. Specimen identification (ID or cf.) and source (newly sequenced or retrieved) are represented by colours, while specimen attribute (terra typica, type locality, type specimen or faunistic novelty) is represented by the shape. Sequences: ID = 2, cf. = 0; maximum p-distance: strict = NA, less strict = NA.

Haplotype network analysis of *Lasius kritikos* was not possible.

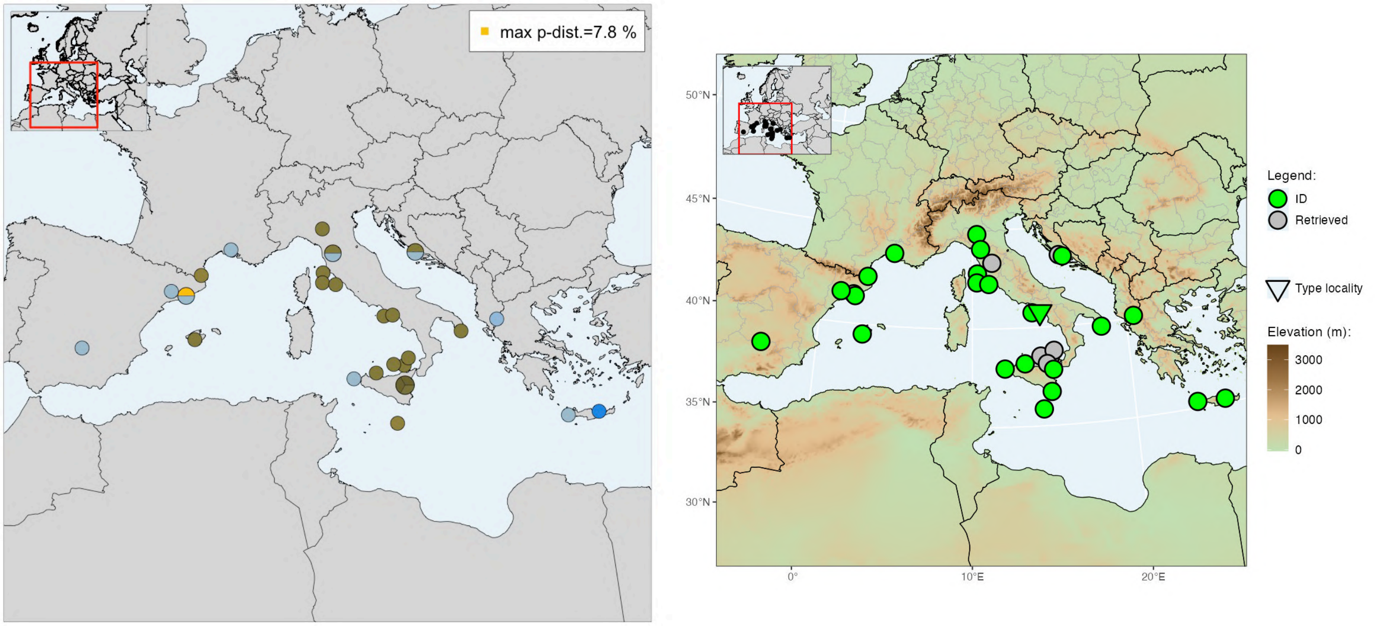

Figure 341: Genetic diversity map of *Lasius lasioides* (Emery, 1869). Nearby localities of sequenced specimens are merged in pies (left). Colours match the bidimensional colour space of the PCoA projection (Fig. 341 left) of p-dist between sequences (dots). Specimen identification (ID or cf.) and source (newly sequenced or retrieved) are represented by colours, while specimen attribute (terra typica, type locality, type specimen or faunistic novelty) is represented by the shape (right). Sequences: ID = 30, cf. = 0; maximum p-distance: strict = 5.3 %, less strict = 7.8 %.

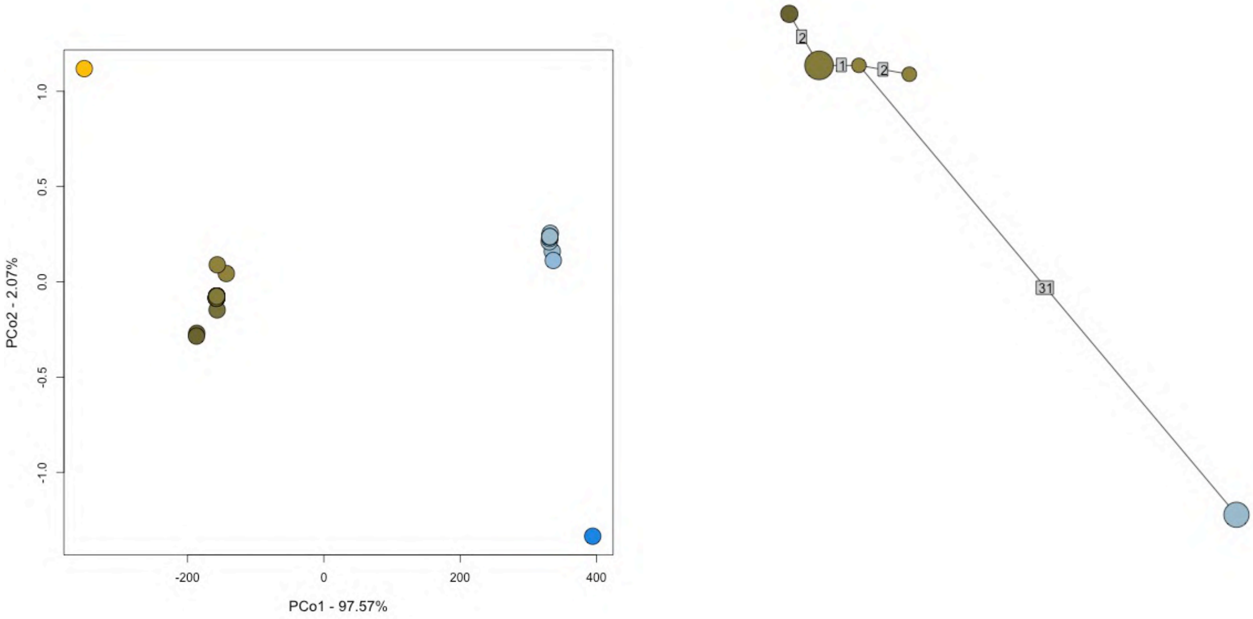

Figure 342: PCoA based on pairwise p-distances between *Lasius lasioides* sequences (left). Colours match a bidimensional colour space. Haplotype network of *Lasius lasioides* (right). Sequences > 599 bp: ID = 28, cf. = 0.

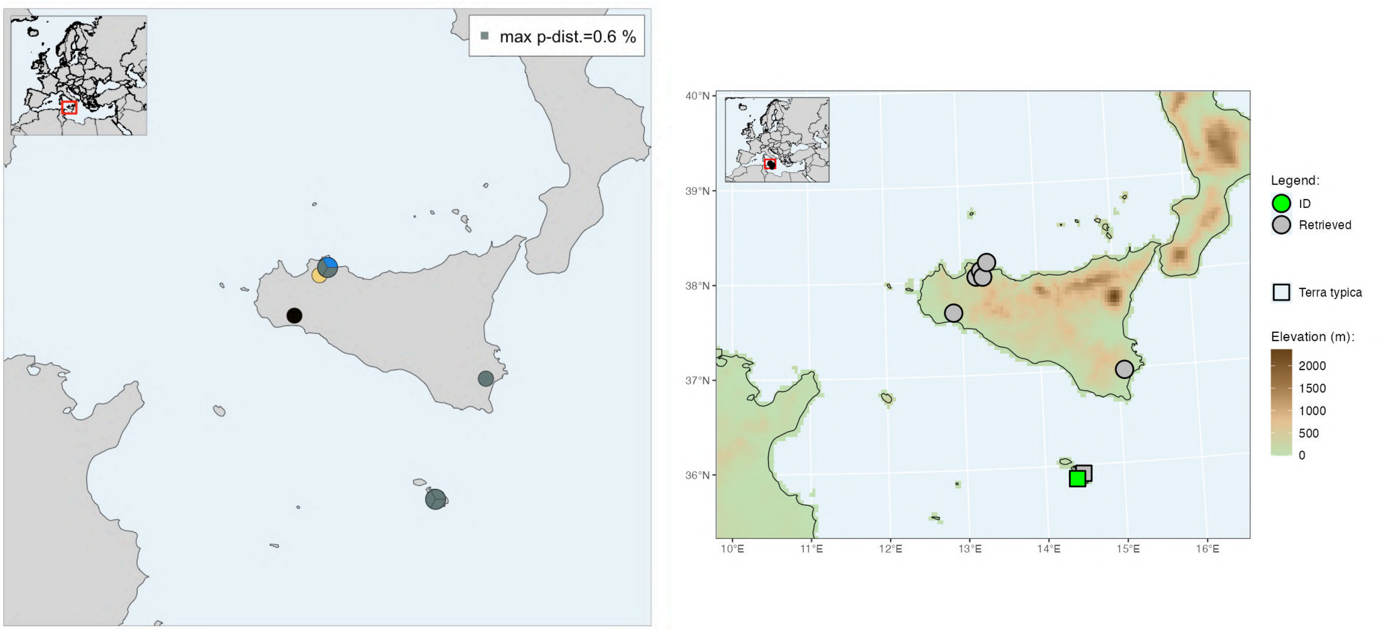

Figure 343: Genetic diversity map of *Lasius maltaeus* Seifert, 2020. Nearby localities of sequenced specimens are merged in pies (left). Colours match the bidimensional colour space of the PCoA projection (Fig. 343 left) of p-dist between sequences (dots). Specimen identification (ID or cf.) and source (newly sequenced or retrieved) are represented by colours, while specimen attribute (terra typica, type locality, type specimen or faunistic novelty) is represented by the shape (right). Sequences: ID = 9, cf. = 0; maximum p-distance: strict = 0.6 %, less strict = 0.6 %.

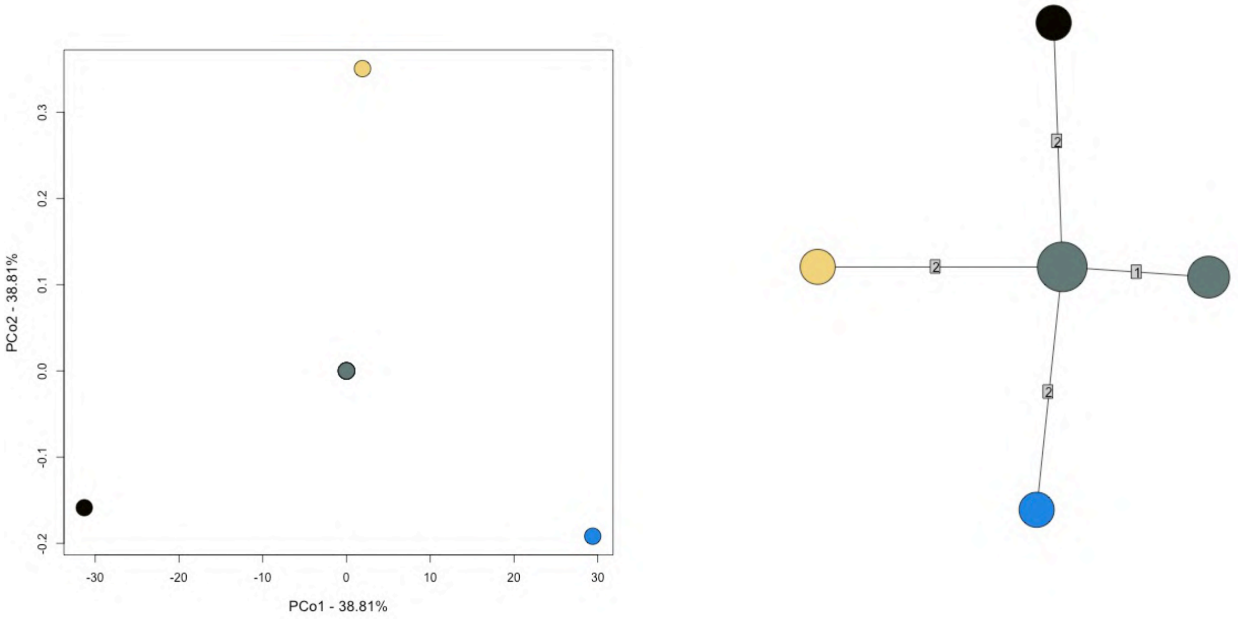

Figure 344: PCoA based on pairwise p-distances between *Lasius maltaeus* sequences (left). Colours match a bidimensional colour space. Haplotype network of *Lasius maltaeus* (right). Sequences > 599 bp: ID = 9, cf. = 0.

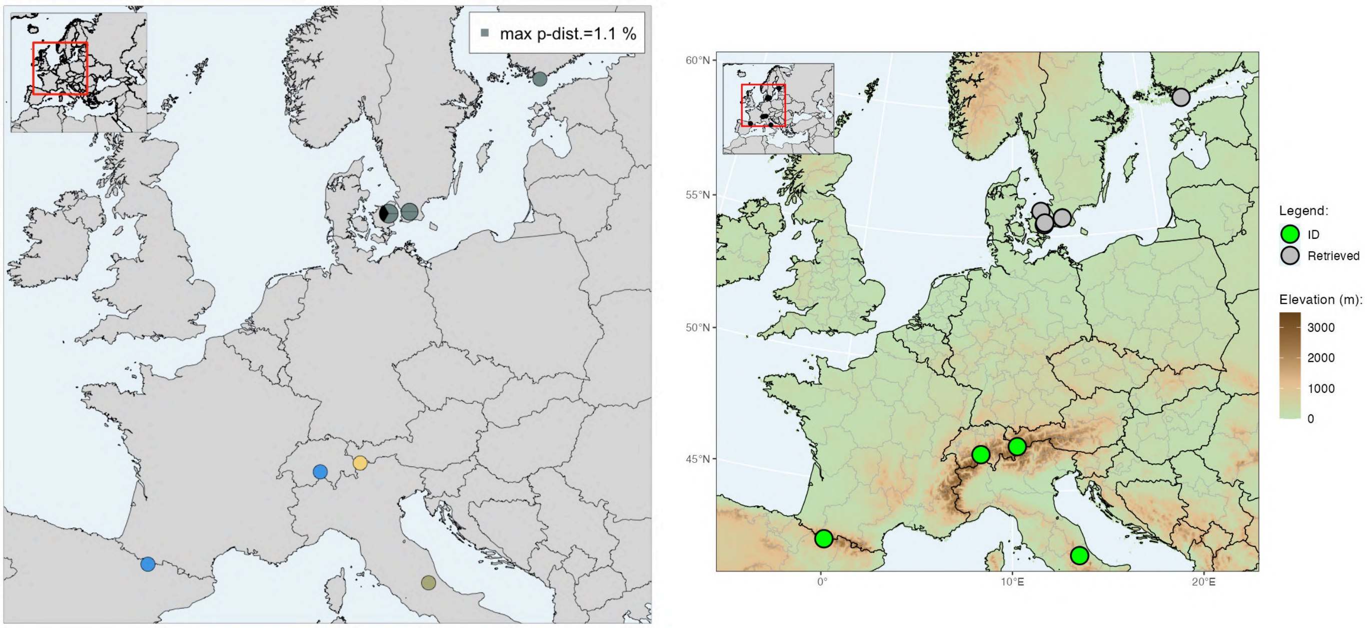

Figure 345: Genetic diversity map of *Lasius meridionalis* (Bondroit, 1920). Nearby localities of sequenced specimens are merged in pies (left). Colours match the bidimensional colour space of the PCoA projection (Fig. 345 left) of p-dist between sequences (dots). Specimen identification (ID or cf.) and source (newly sequenced or retrieved) are represented by colours, while specimen attribute (terra typica, type locality, type specimen or faunistic novelty) is represented by the shape (right). Sequences: ID = 11, cf. = 0; maximum p-distance: strict = 1.1 %, less strict = 1.1 %.

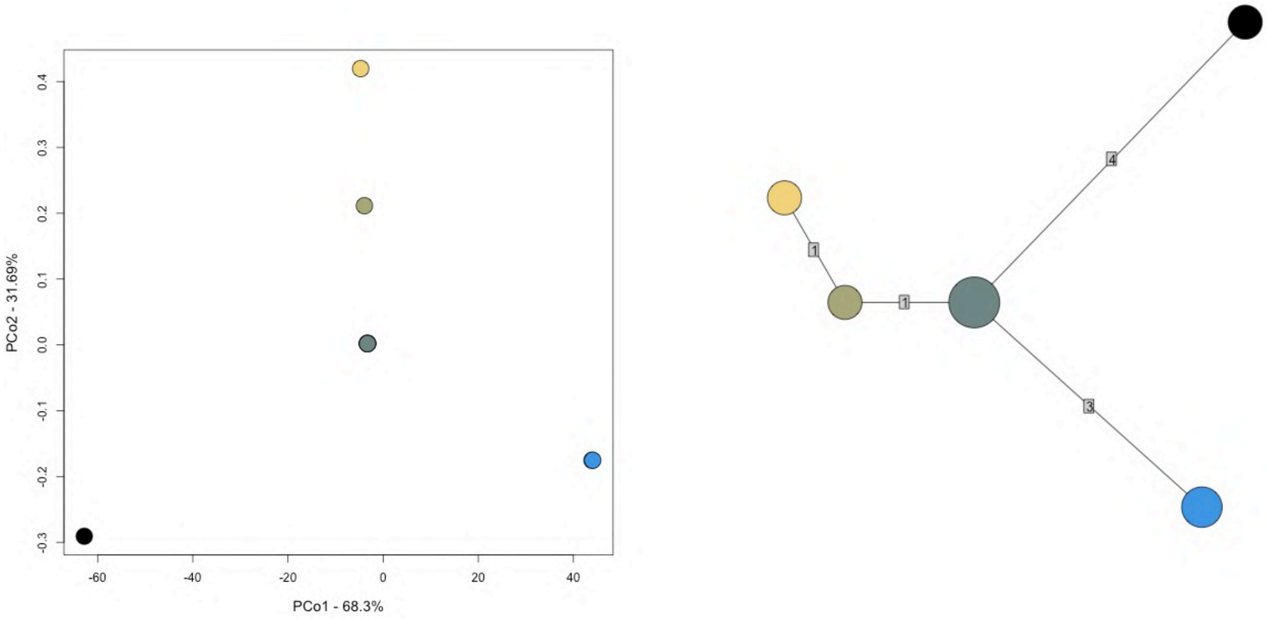

Figure 346: PCoA based on pairwise p-distances between *Lasius meridionalis* sequences (left). Colours match a bidimensional colour space. Haplotype network of *Lasius meridionalis* (right). Sequences > 599 bp: ID = 11, cf. = 0.

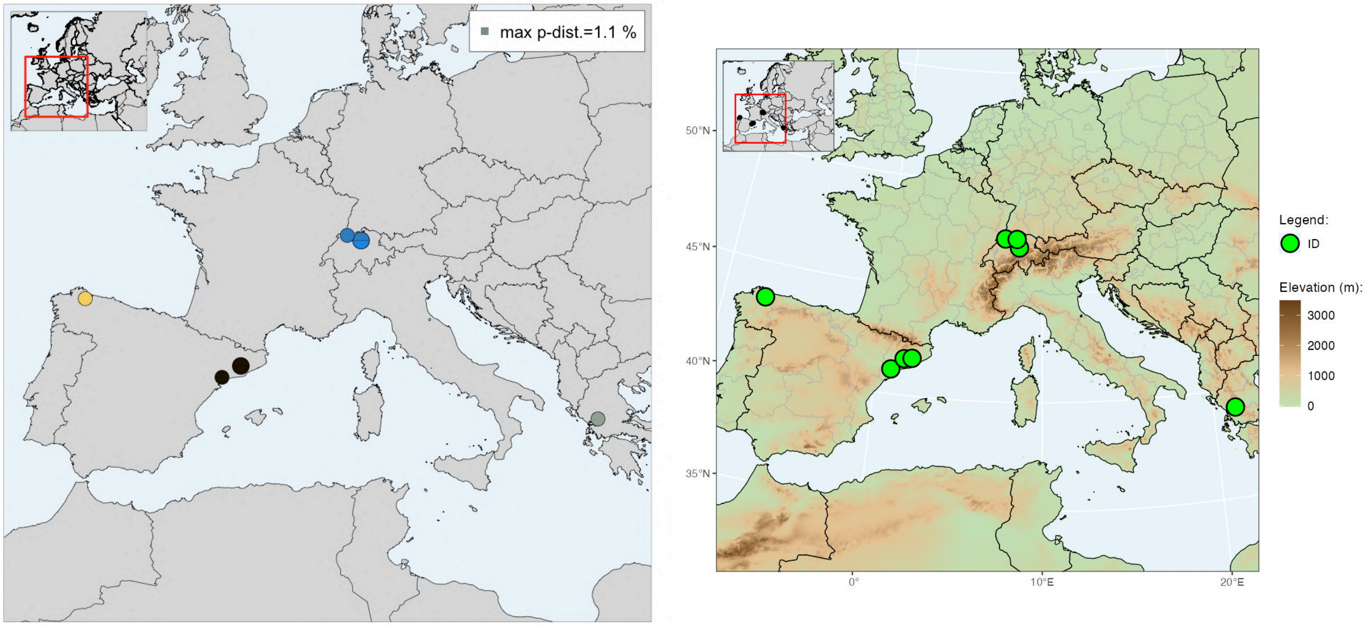

Figure 347: Genetic diversity map of *Lasius mixtus* (Nylander, 1846). Nearby localities of sequenced specimens are merged in pies (left). Colours match the bidimensional colour space of the PCoA projection (Fig. 347 left) of p-dist between sequences (dots). Specimen identification (ID or cf.) and source (newly sequenced or retrieved) are represented by colours, while specimen attribute (terra typica, type locality, type specimen or faunistic novelty) is represented by the shape (right). Sequences: ID = 8, cf. = 0; maximum p-distance: strict = 1.1 %, less strict = 1.1 %.

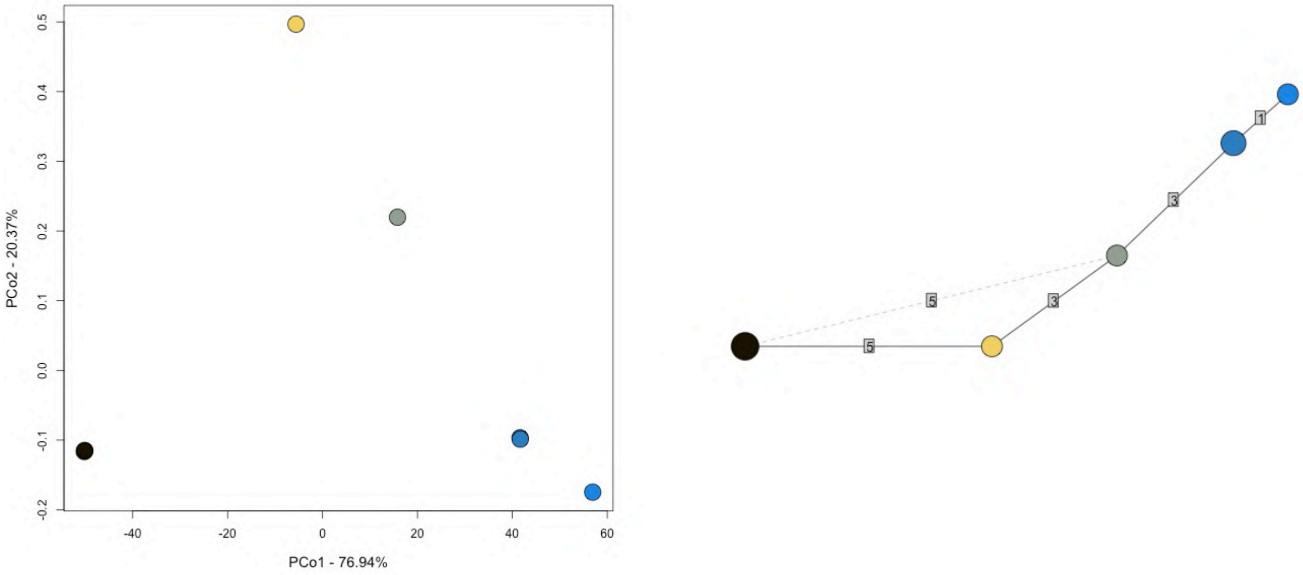

Figure 348: PCoA based on pairwise p-distances between *Lasius mixtus* sequences (left). Colours match a bidimensional colour space. Haplotype network of *Lasius mixtus* (right). Sequences > 599 bp: ID = 8, cf. = 0.

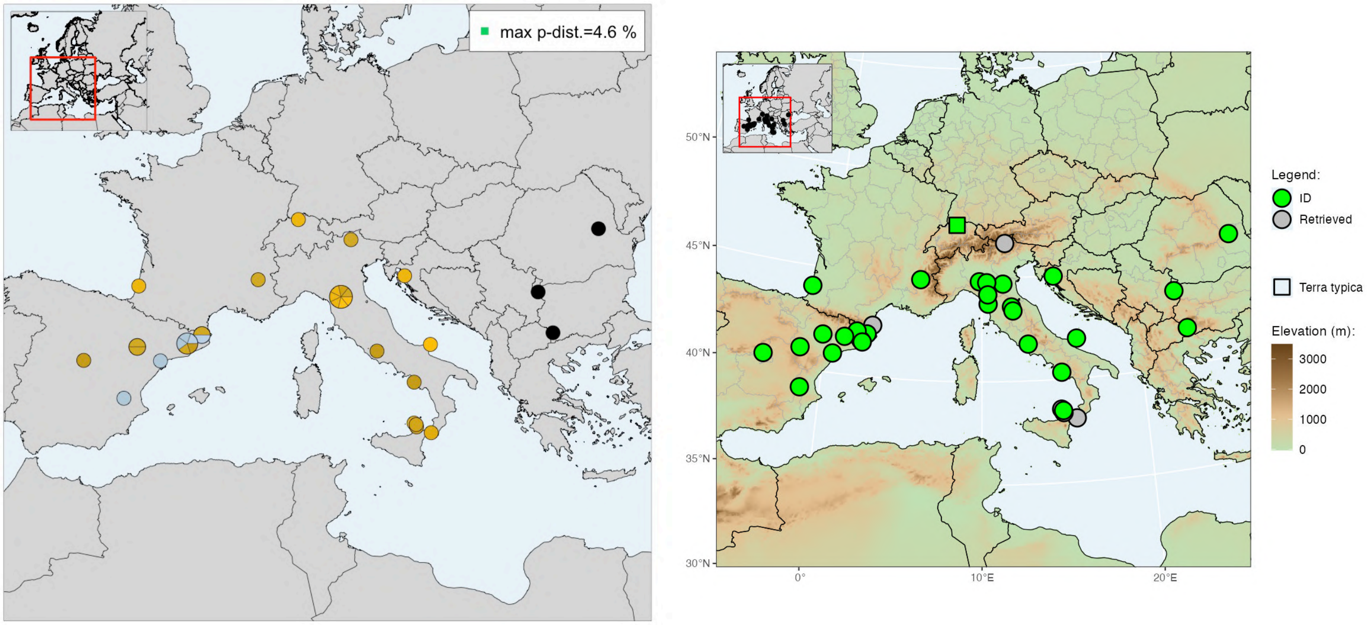

Figure 349: Genetic diversity map of *Lasius myops* Forel, 1894. Nearby localities of sequenced specimens are merged in pies (left). Colours match the bidimensional colour space of the PCoA projection (Fig. 349 left) of p-dist between sequences (dots). Specimen identification (ID or cf.) and source (newly sequenced or retrieved) are represented by colours, while specimen attribute (terra typica, type locality, type specimen or faunistic novelty) is represented by the shape (right). Sequences: ID = 42, cf. = 0; maximum p-distance: strict = 4.6 %, less strict = 4.6 %.

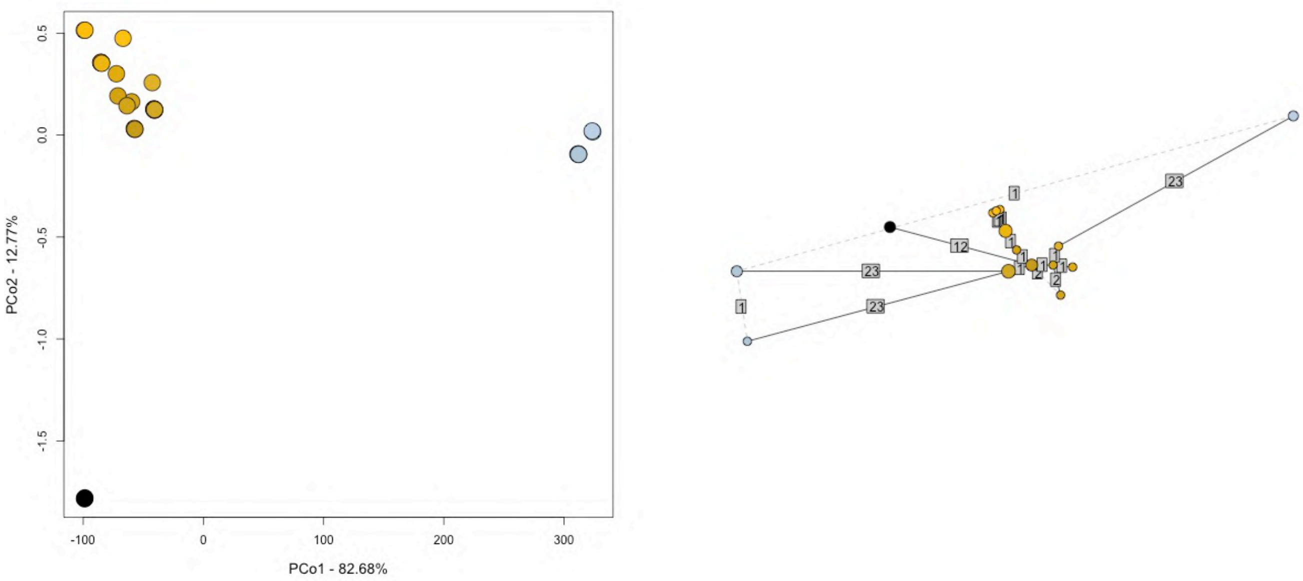

Figure 350: PCoA based on pairwise p-distances between *Lasius myops* sequences (left). Colours match a bidimensional colour space. Haplotype network of *Lasius myops* (right). Sequences > 599 bp: ID = 42, cf. = 0.

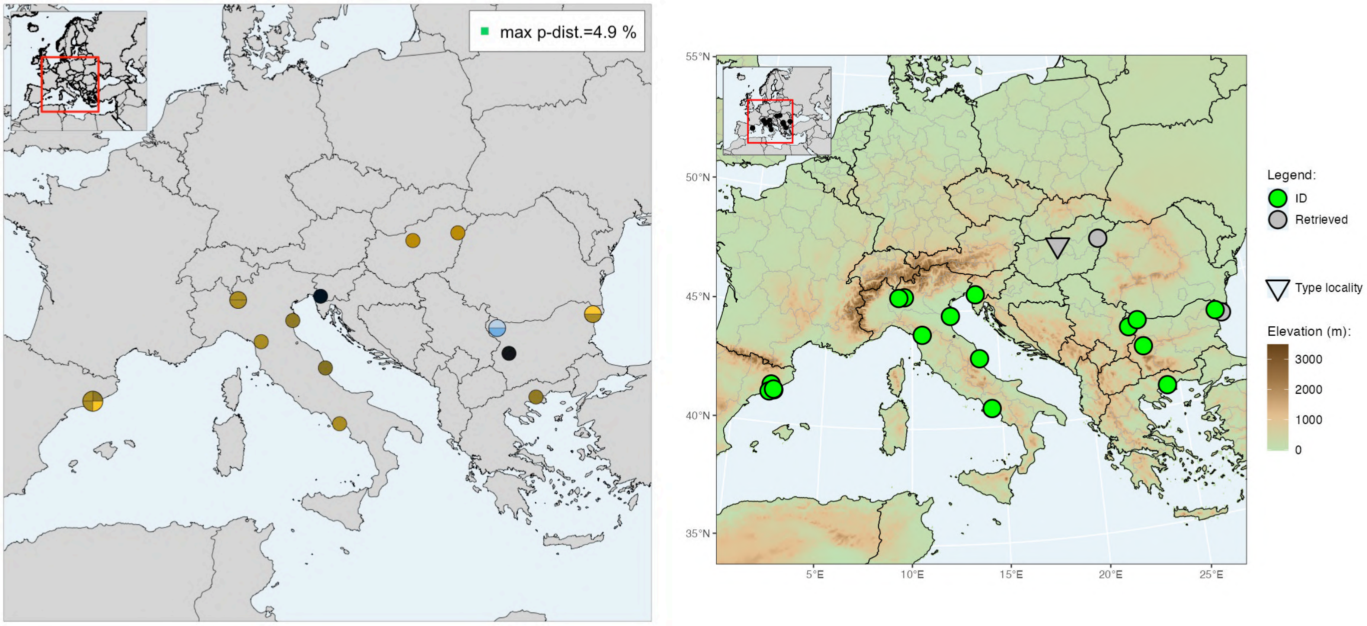

Figure 351: Genetic diversity map of *Lasius neglectus* Van Loon, Boomsma & Andrasfalvy, 1990. Nearby localities of sequenced specimens are merged in pies (left). Colours match the bidimensional colour space of the PCoA projection (Fig. 351 left) of p-dist between sequences (dots). Specimen identification (ID or cf.) and source (newly sequenced or retrieved) are represented by colours, while specimen attribute (terra typica, type locality, type specimen or faunistic novelty) is represented by the shape (right). Sequences: ID = 19, cf. = 0; maximum p-distance: strict = 4.3 %, less strict = 4.9 %.

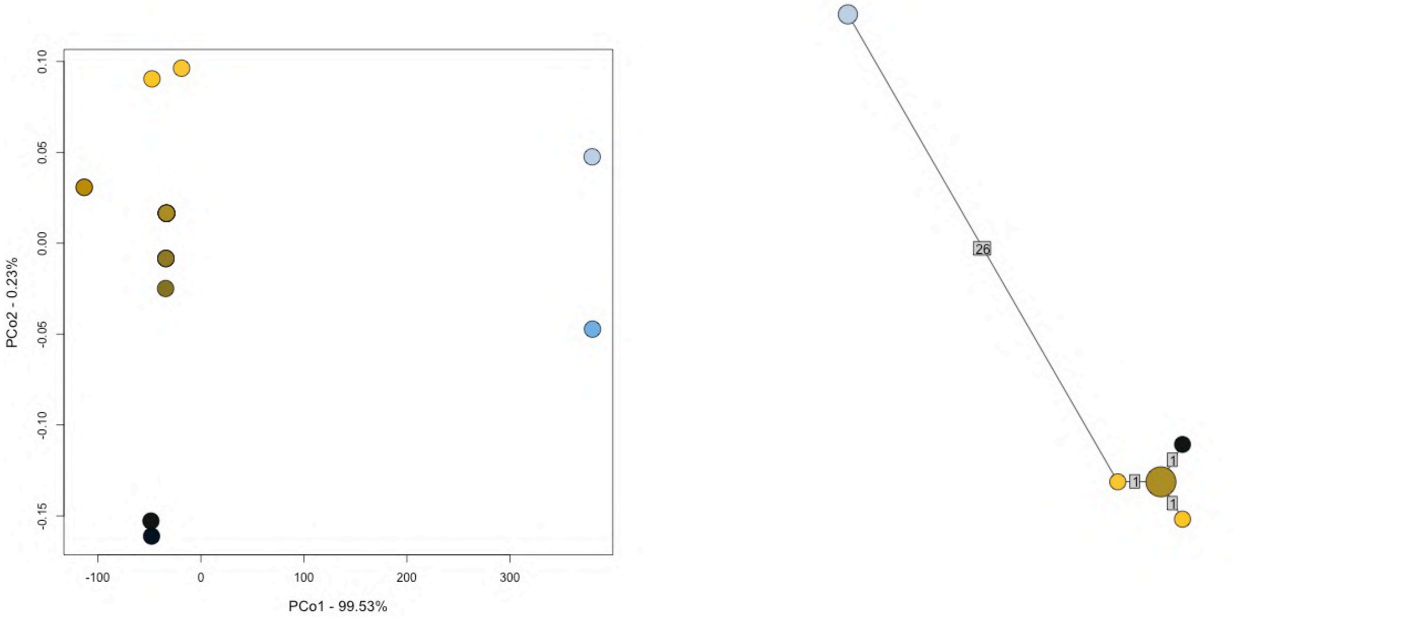

Figure 352: PCoA based on pairwise p-distances between *Lasius neglectus* sequences (left). Colours match a bidimensional colour space. Haplotype network of *Lasius neglectus* (right). Sequences > 599 bp: ID = 17, cf. = 0.

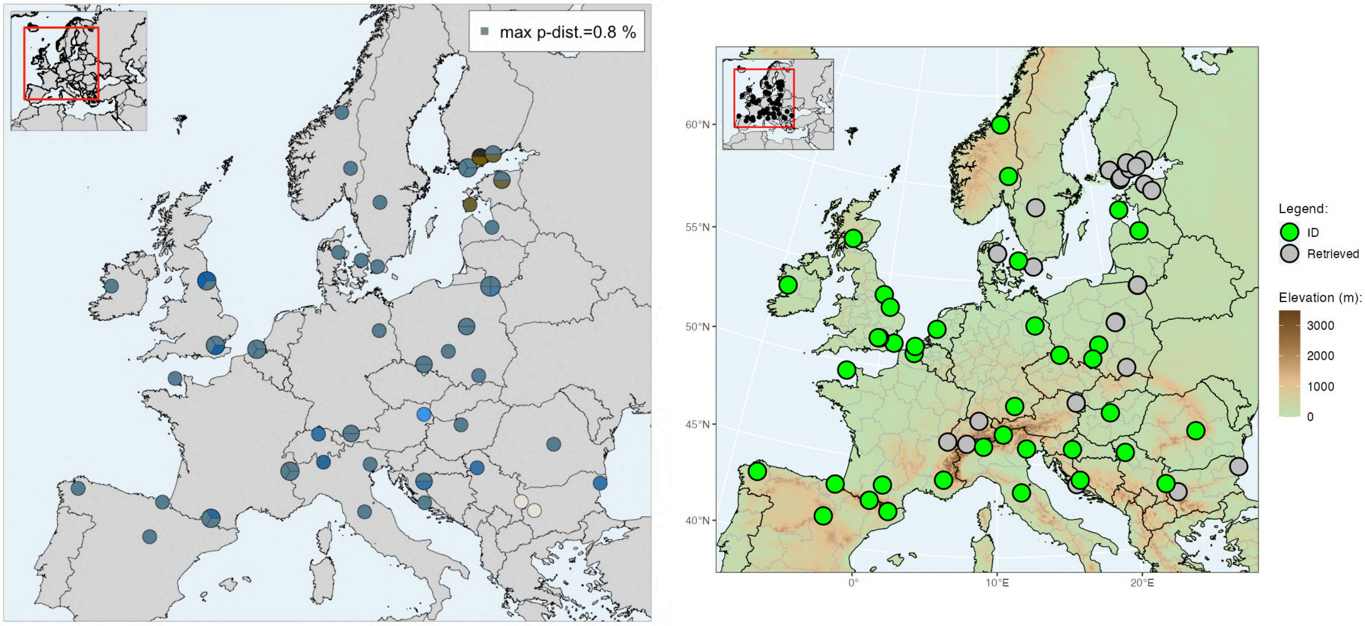

Figure 353: Genetic diversity map of *Lasius niger* (Linnaeus, 1758). Nearby localities of sequenced specimens are merged in pies (left). Colours match the bidimensional colour space of the PCoA projection (Fig. 353 left) of p-dist between sequences (dots). Specimen identification (ID or cf.) and source (newly sequenced or retrieved) are represented by colours, while specimen attribute (terra typica, type locality, type specimen or faunistic novelty) is represented by the shape (right). Sequences: ID = 64, cf. = 0; maximum p-distance: strict = 0.8 %, less strict = 0.8 %.

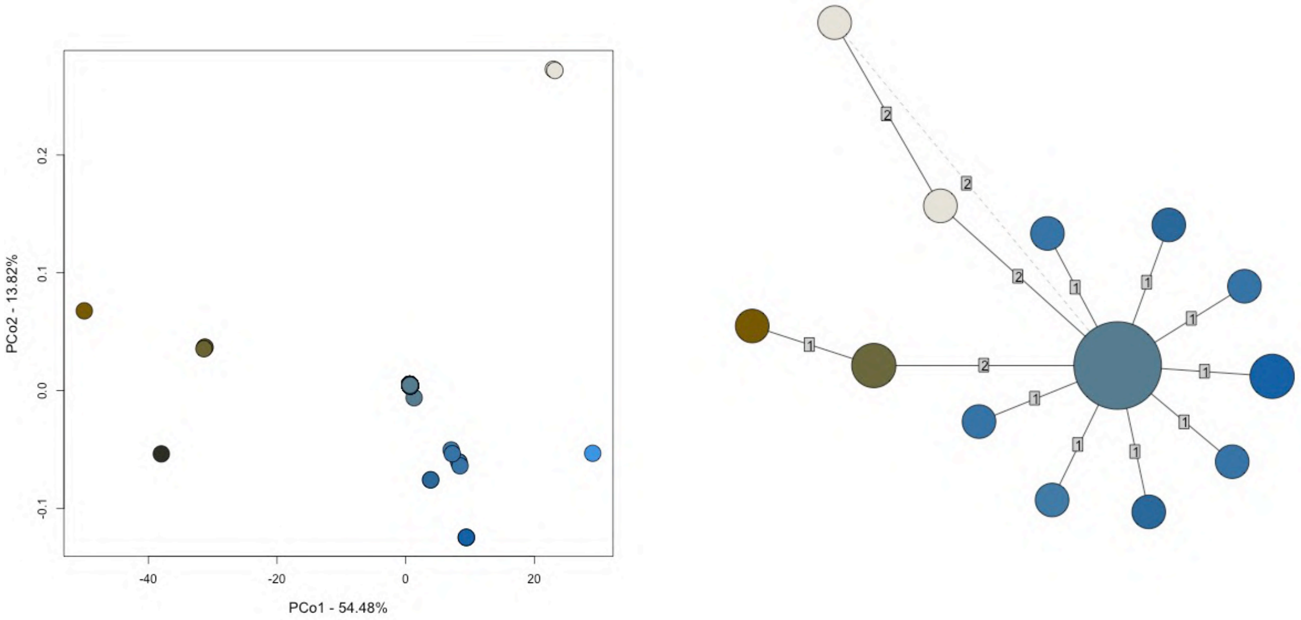

Figure 354: PCoA based on pairwise p-distances between *Lasius niger* sequences (left). Colours match a bidimensional colour space. Haplotype network of *Lasius niger* (right). Sequences > 599 bp: ID = 62, cf. = 0.

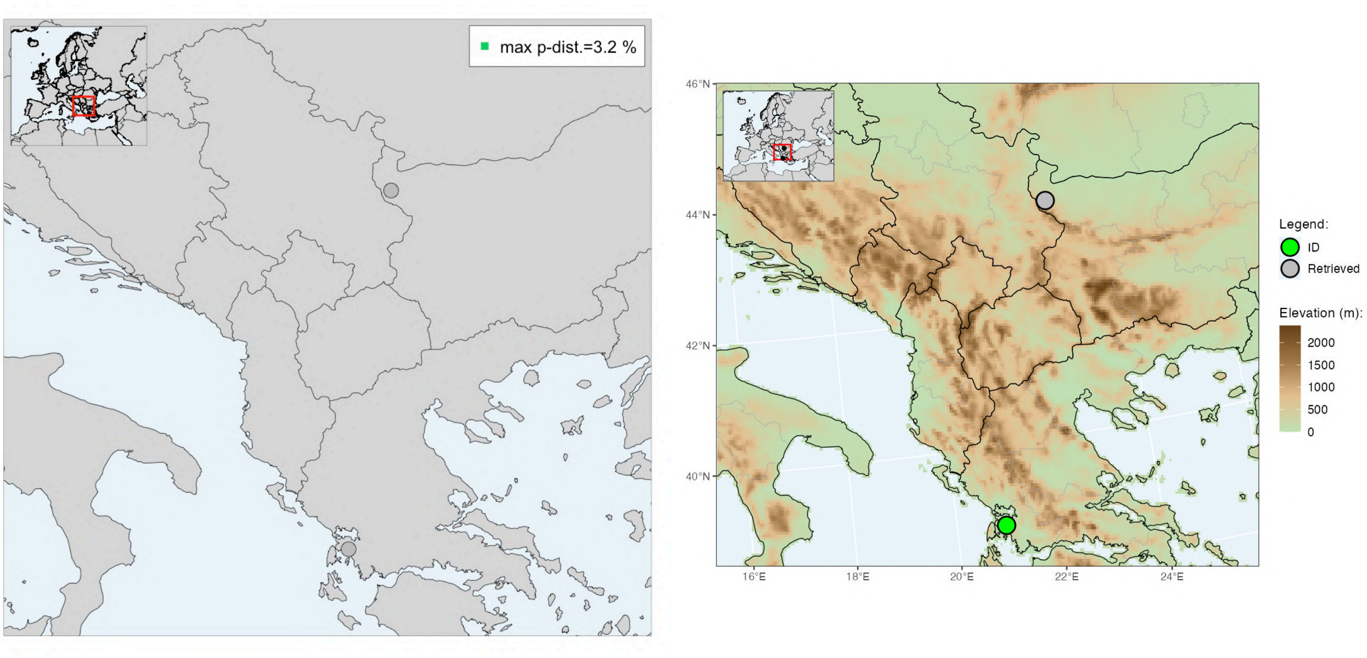

Figure 355: Genetic diversity map of *Lasius nitidigaster* Seifert, 1996. PCoA projection was not done and therefore sequenced specimens in the genetic diversity map are coloured in gray (left). Specimen identification (ID or cf.) and source (newly sequenced or retrieved) are represented by colours, while specimen attribute (terra typica, type locality, type specimen or faunistic novelty) is represented by the shape (right). Sequences: ID = 2, cf. = 0; maximum p-distance: strict = NA, less strict = 3.2 %.

Haplotype network analysis of *Lasius nitidigaster* was not possible.

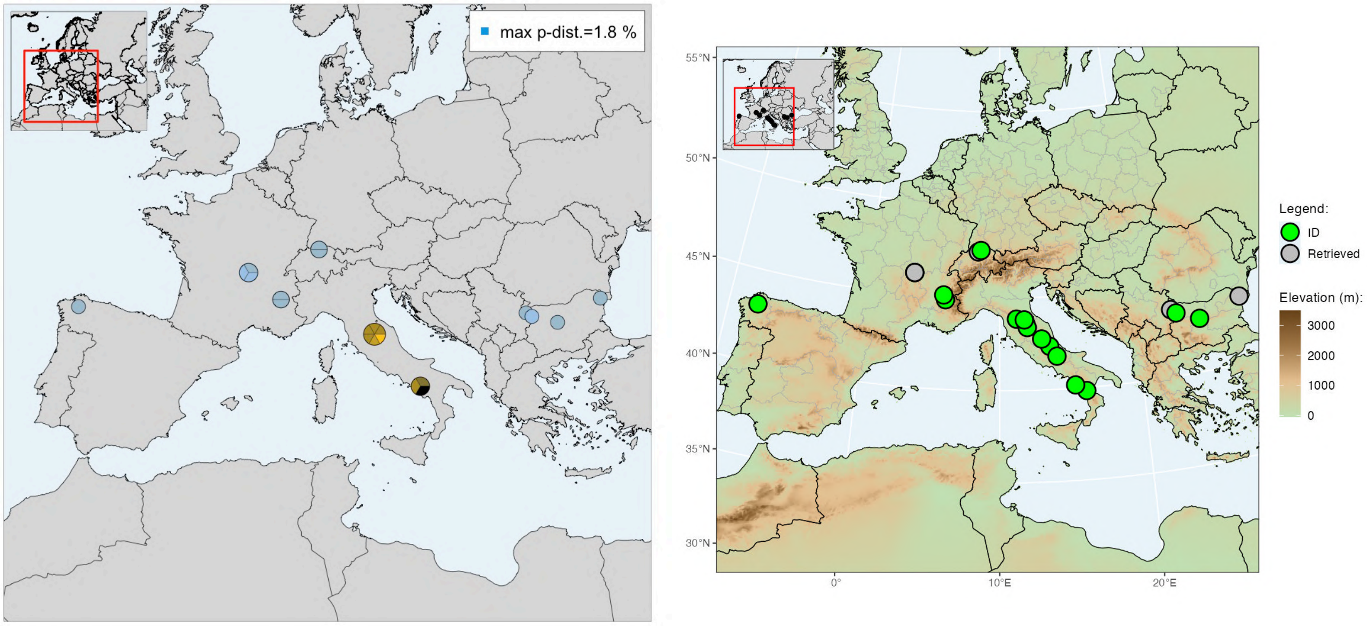

Figure 356: Genetic diversity map of *Lasius paralienus* Seifert, 1992. Nearby localities of sequenced specimens are merged in pies (left). Colours match the bidimensional colour space of the PCoA projection (Fig. 356 left) of p-dist between sequences (dots). Specimen identification (ID or cf.) and source (newly sequenced or retrieved) are represented by colours, while specimen attribute (terra typica, type locality, type specimen or faunistic novelty) is represented by the shape (right). Sequences: ID = 21, cf. = 0; maximum p-distance: strict = 1.8 %, less strict = 1.8 %.

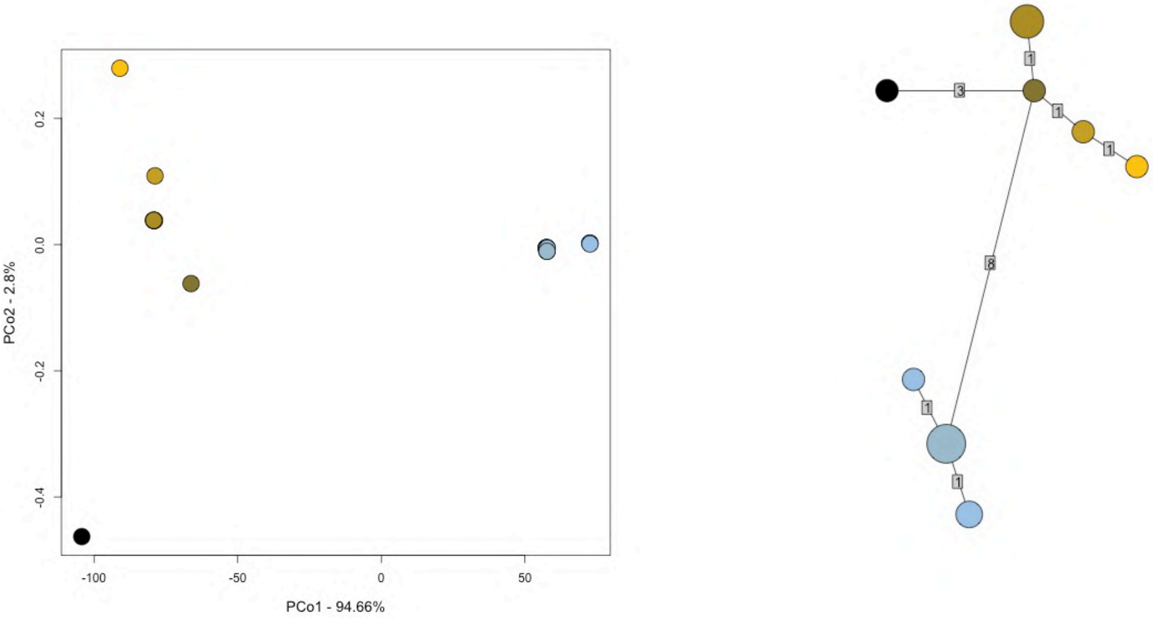

Figure 357: PCoA based on pairwise p-distances between *Lasius paralienus* sequences (left). Colours match a bidimensional colour space. Haplotype network of *Lasius paralienus* (right). Sequences > 599 bp: ID = 21, cf. = 0.

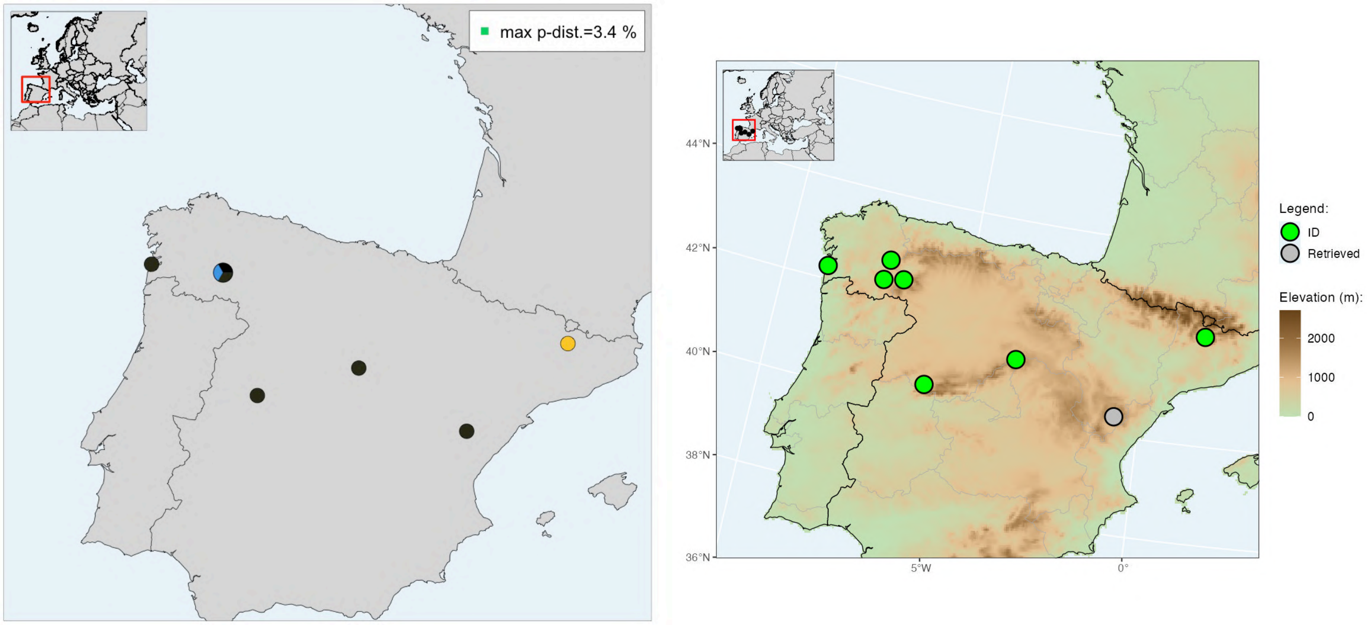

Figure 358: Genetic diversity map of *Lasius piliferus* Seifert, 1992. Nearby localities of sequenced specimens are merged in pies (left). Colours match the bidimensional colour space of the PCoA projection (Fig. 358 left) of p-dist between sequences (dots). Specimen identification (ID or cf.) and source (newly sequenced or retrieved) are represented by colours, while specimen attribute (terra typica, type locality, type specimen or faunistic novelty) is represented by the shape (right). Sequences: ID = 8, cf. = 0; maximum p-distance: strict = 3.4 %, less strict = 3.4 %.

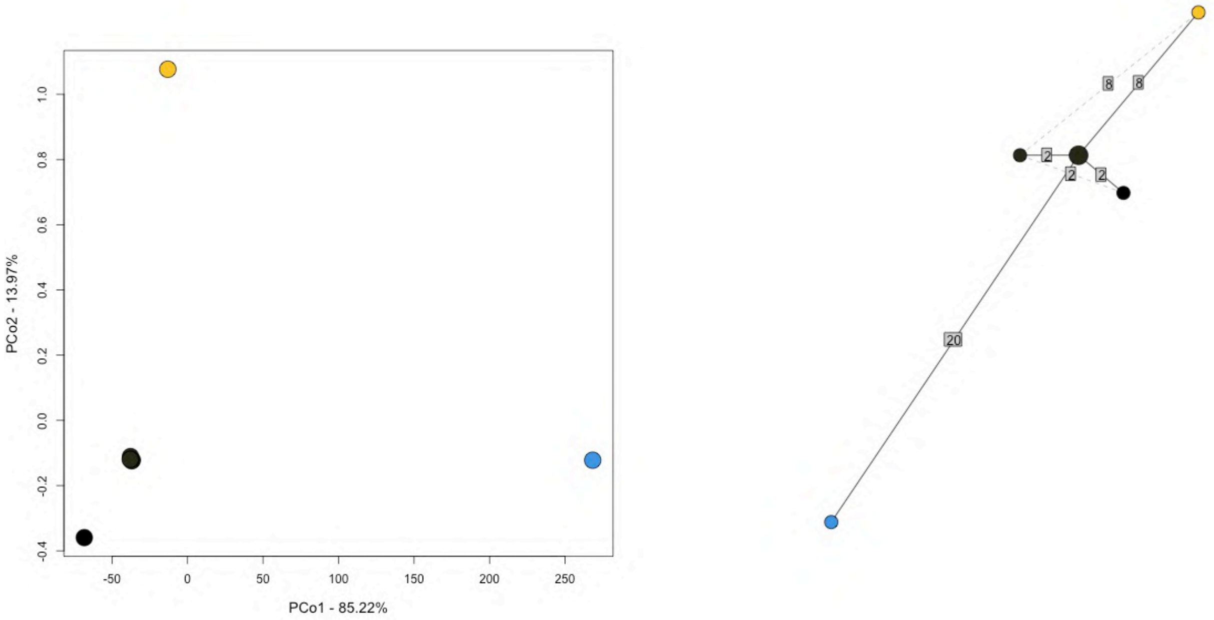

Figure 359: PCoA based on pairwise p-distances between *Lasius piliferus* sequences (left). Colours match a bidimensional colour space. Haplotype network of *Lasius piliferus* (right). Sequences > 599 bp: ID = 8, cf. = 0.

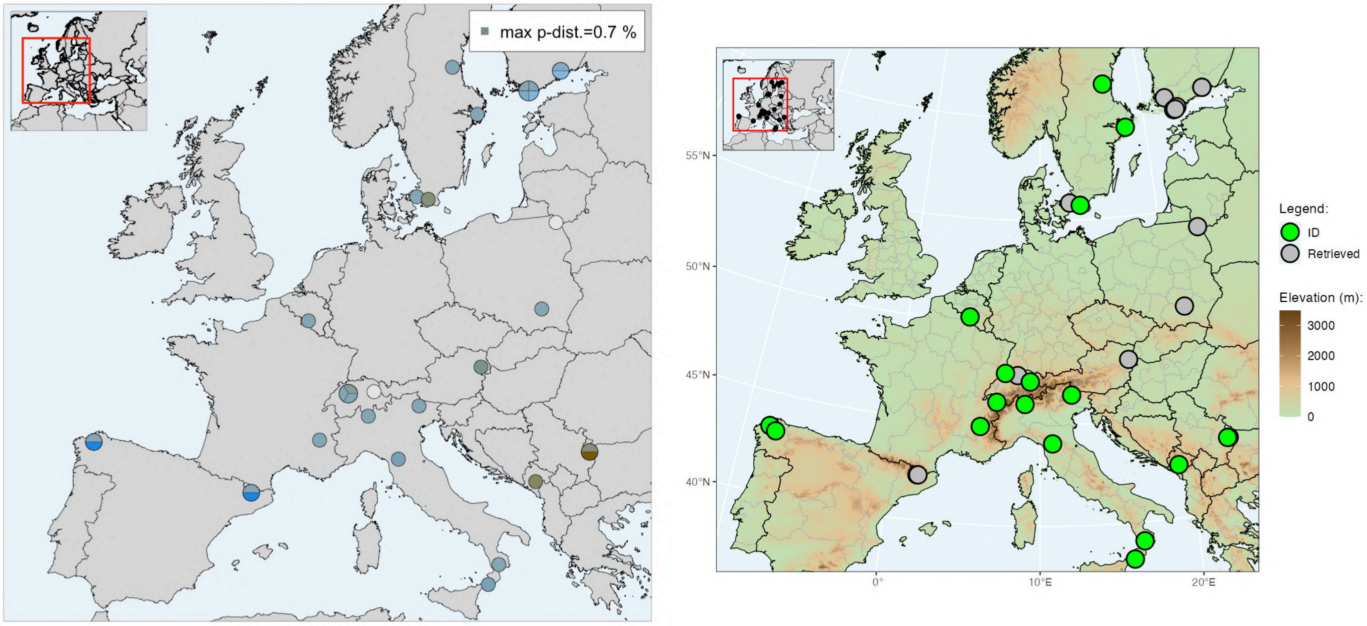

Figure 360: Genetic diversity map of *Lasius platythorax* Seifert, 1991. Nearby localities of sequenced specimens are merged in pies (left). Colours match the bidimensional colour space of the PCoA projection (Fig. 360 left) of p-dist between sequences (dots). Specimen identification (ID or cf.) and source (newly sequenced or retrieved) are represented by colours, while specimen attribute (terra typica, type locality, type specimen or faunistic novelty) is represented by the shape (right). Sequences: ID = 31, cf. = 0; maximum p-distance: strict = 0.7 %, less strict = 0.7 %.

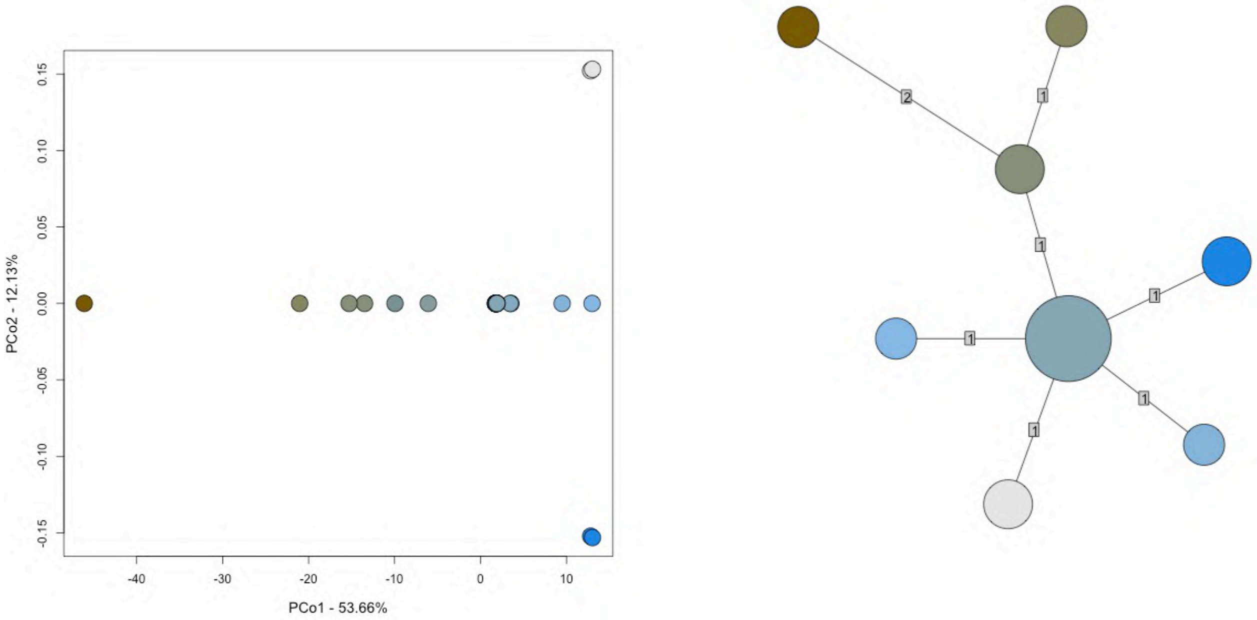

Figure 361: PCoA based on pairwise p-distances between *Lasius platythorax* sequences (left). Colours match a bidimensional colour space. Haplotype network of *Lasius platythorax* (right). Sequences > 599 bp: ID = 29, cf. = 0.

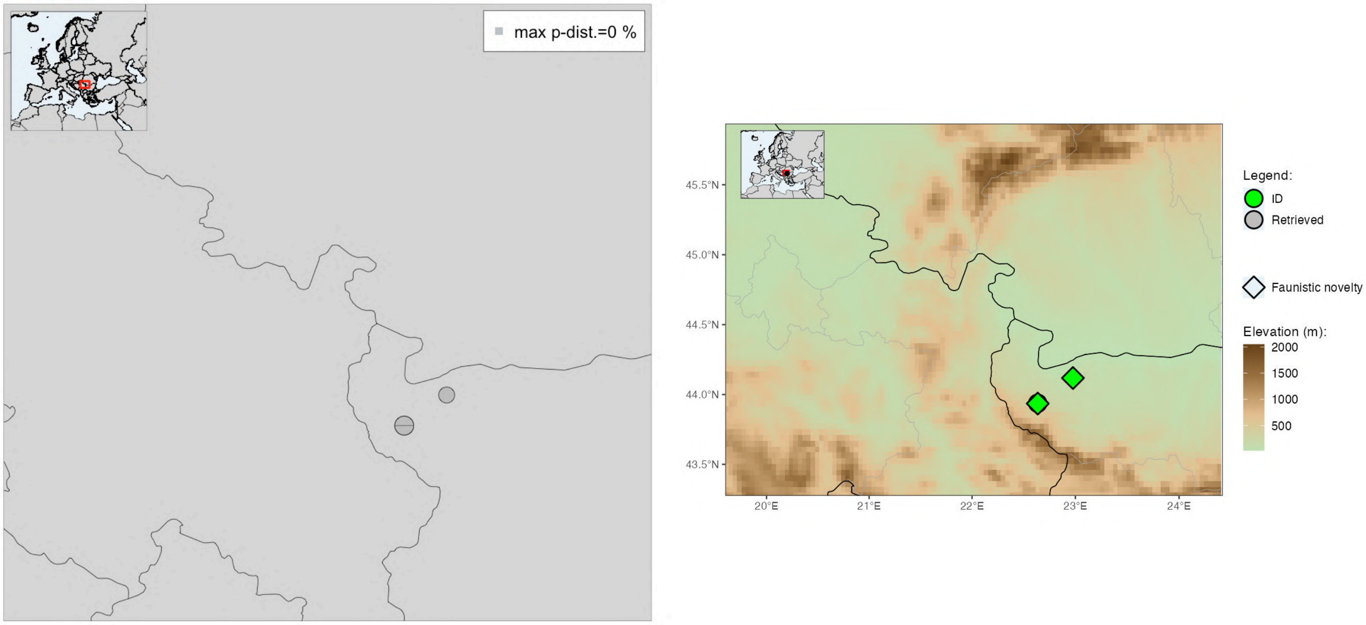

Figure 362: Genetic diversity map of *Lasius precursor* Seifert, 2020. PCoA projection was not done and therefore sequenced specimens in the genetic diversity map are coloured in gray (left). Specimen identification (ID or cf.) and source (newly sequenced or retrieved) are represented by colours, while specimen attribute (terra typica, type locality, type specimen or faunistic novelty) is represented by the shape (right). Sequences: ID = 3, cf. = 0; maximum p-distance: strict = 0 %, less strict = 0 %.

The species is reported for the first time in Bulgaria.

Haplotype network analysis of *Lasius precursor* was not possible.

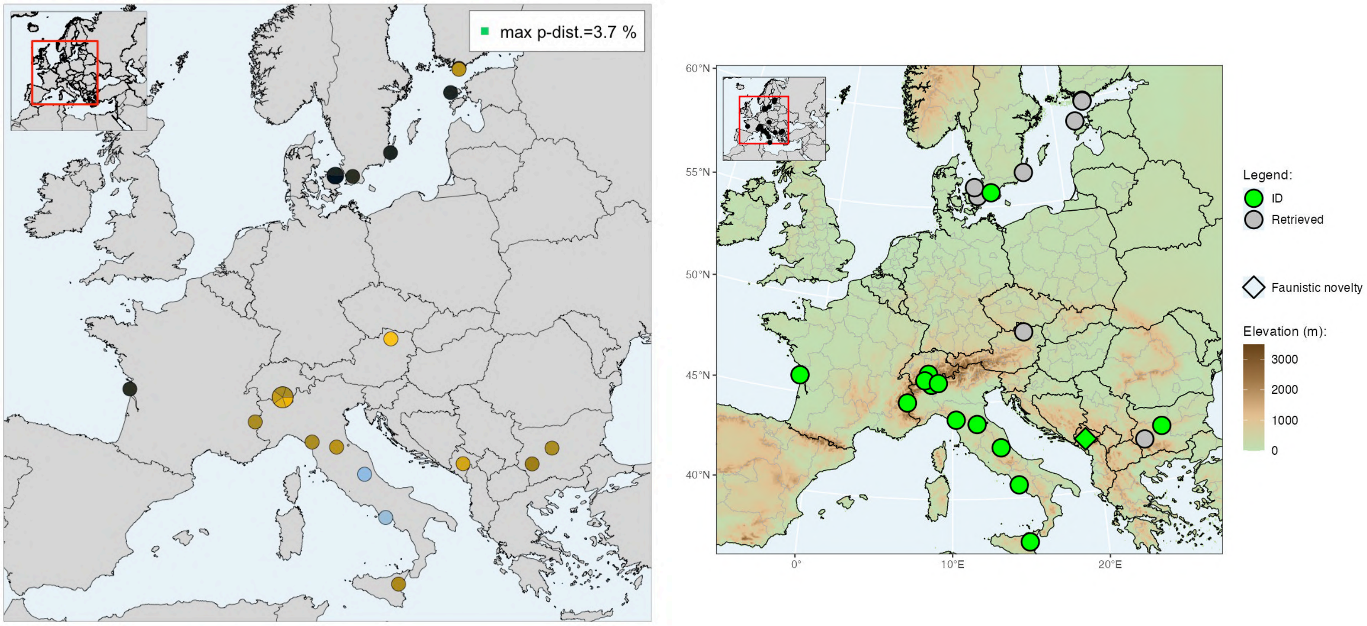

Figure 363: Genetic diversity map of *Lasius psammophilus* Seifert, 1992. Nearby localities of sequenced specimens are merged in pies (left). Colours match the bidimensional colour space of the PCoA projection (Fig. 363 left) of p-dist between sequences (dots). Specimen identification (ID or cf.) and source (newly sequenced or retrieved) are represented by colours, while specimen attribute (terra typica, type locality, type specimen or faunistic novelty) is represented by the shape (right). Sequences: ID = 23, cf. = 0; maximum p-distance: strict = 3.7 %, less strict = 3.7 %.

The species is reported for the first time in Montenegro.

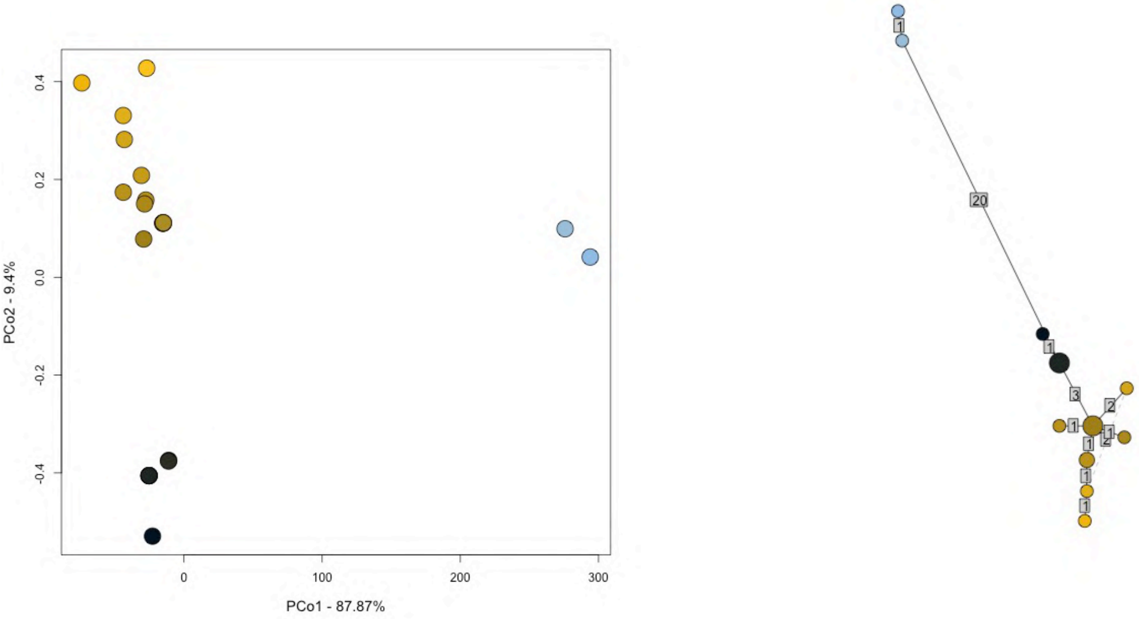

Figure 364: PCoA based on pairwise p-distances between *Lasius psammophilus* sequences (left). Colours match a bidimensional colour space. Haplotype network of *Lasius psammophilus* (right). Sequences > 599 bp: ID = 22, cf. = 0.

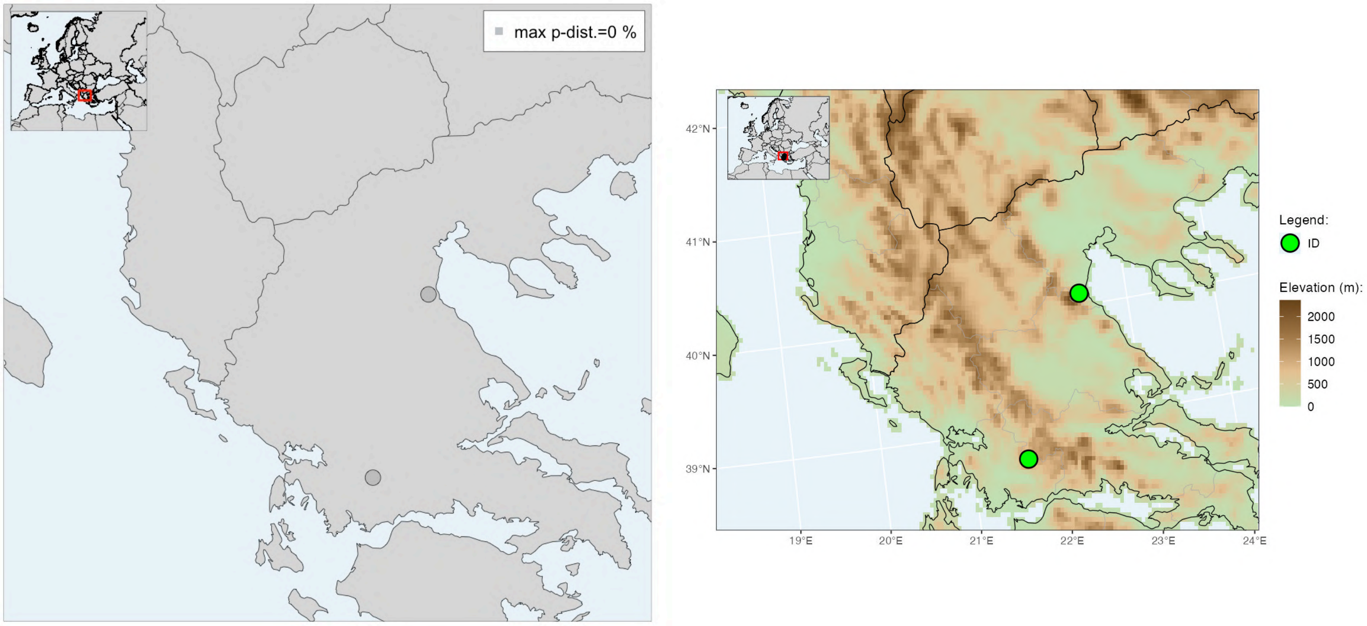

Figure 365: Genetic diversity map of *Lasius reginae* Faber, 1967. PCoA projection was not done and therefore sequenced specimens in the genetic diversity map are coloured in gray (left). Specimen identification (ID or cf.) and source (newly sequenced or retrieved) are represented by colours, while specimen attribute (terra typica, type locality, type specimen or faunistic novelty) is represented by the shape (right). Sequences: ID = 2, cf. = 0; maximum p-distance: strict = NA, less strict = 0 %.

Haplotype network analysis of *Lasius reginae* was not possible.

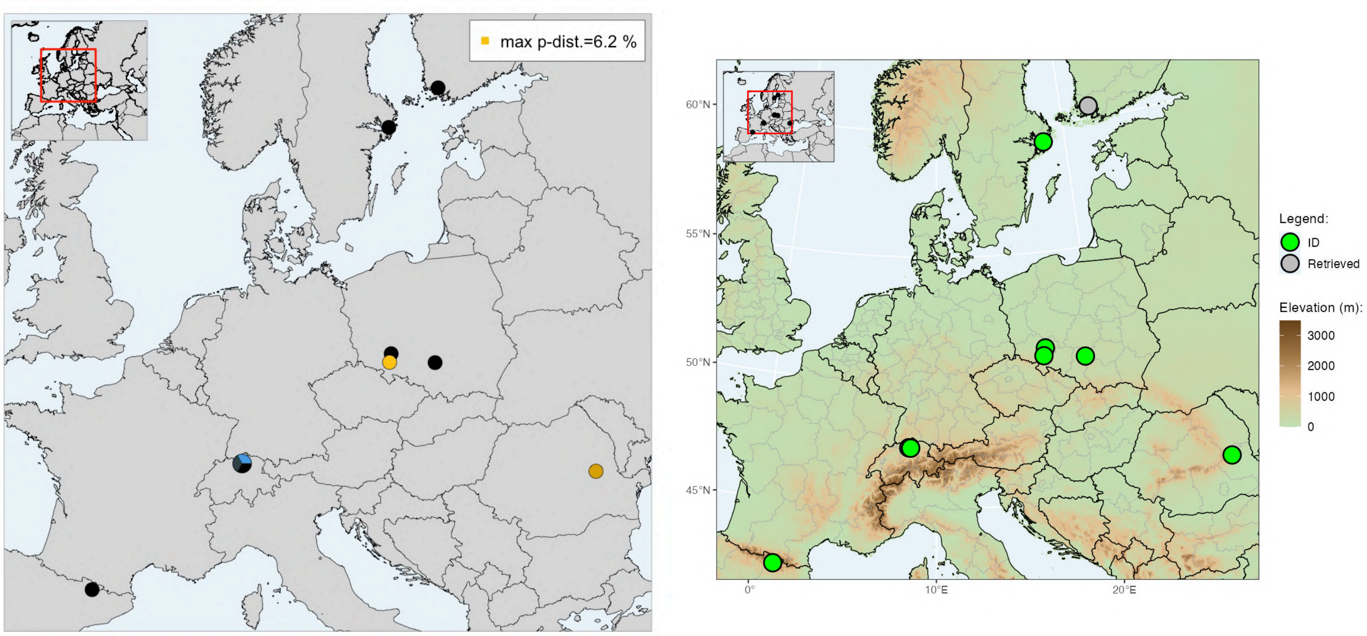

Figure 366: Genetic diversity map of *Lasius sabularum* (Bondroit, 1918). Nearby localities of sequenced specimens are merged in pies (left). Colours match the bidimensional colour space of the PCoA projection (Fig. 366 left) of p-dist between sequences (dots). Specimen identification (ID or cf.) and source (newly sequenced or retrieved) are represented by colours, while specimen attribute (terra typica, type locality, type specimen or faunistic novelty) is represented by the shape (right). Sequences: ID = 10, cf. = 0; maximum p-distance: strict = 6.2 %, less strict = 6.2 %.

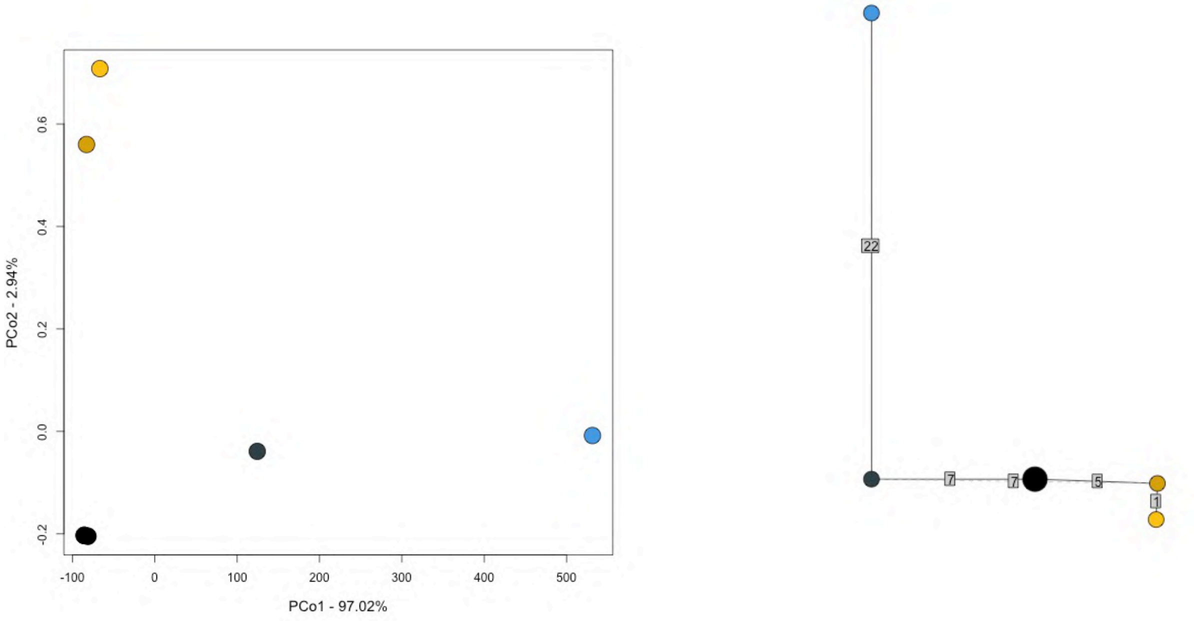

Figure 367: PCoA based on pairwise p-distances between *Lasius sabularum* sequences (left). Colours match a bidimensional colour space. Haplotype network of *Lasius sabularum* (right). Sequences > 599 bp: ID = 10, cf. = 0.

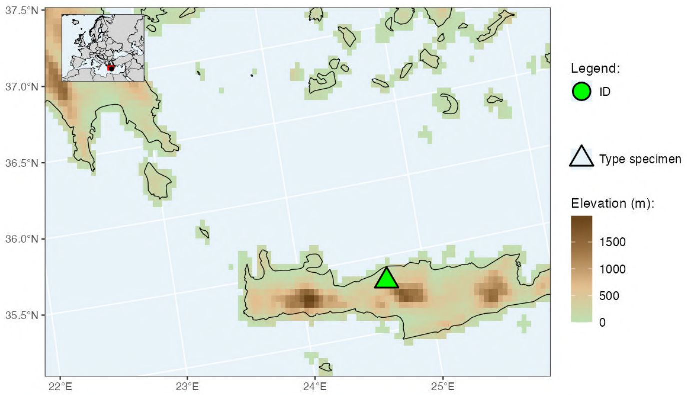

Figure 368: Map of *Lasius tapinomoides* Salata & Borowiec, 2018. Due to the presence of a single sequence, the genetic diversity map and the PCoA projection were not done. Specimen identification (ID or cf.) and source (newly sequenced or retrieved) are represented by colours, while specimen attribute (terra typica, type locality, type specimen or faunistic novelty) is represented by the shape. Sequences: ID = 1, cf. = 0; maximum p-distance: strict = NA, less strict = NA.

Haplotype network analysis of *Lasius tapinomoides* was not possible.

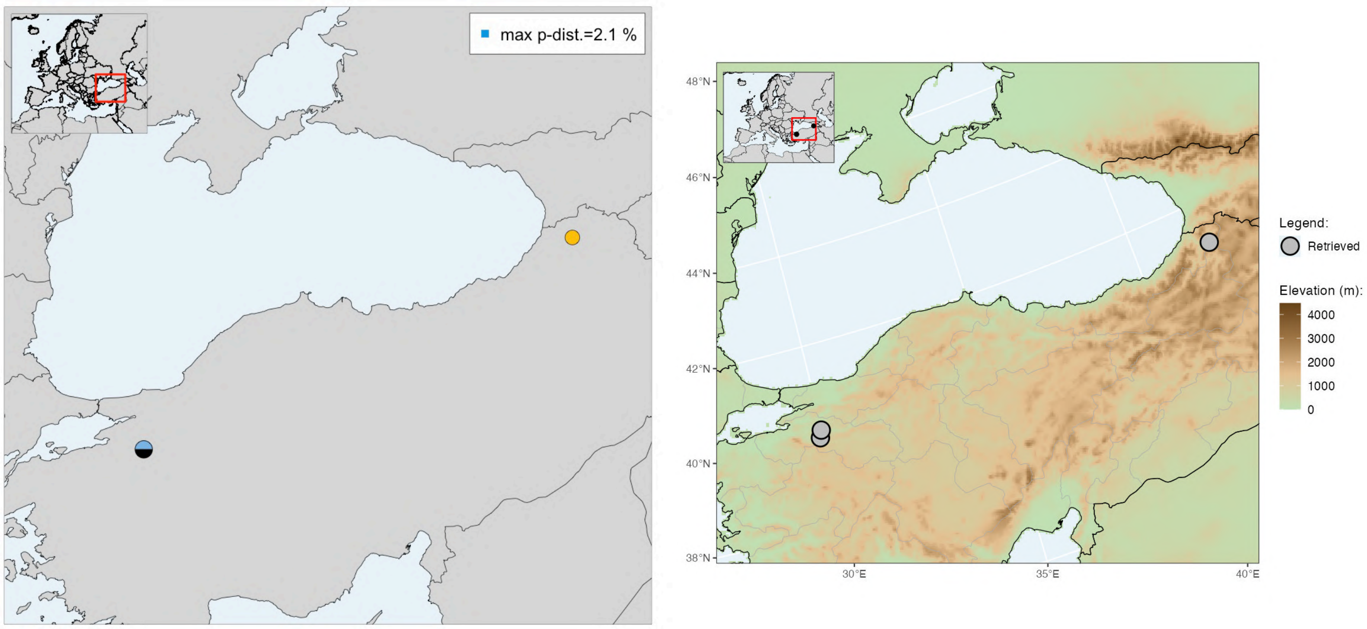

Figure 369: Genetic diversity map of *Lasius turcicus* Santschi, 1921. Nearby localities of sequenced specimens are merged in pies (left). Colours match the bidimensional colour space of the PCoA projection (Fig. 369 left) of p-dist between sequences (dots). Specimen identification (ID or cf.) and source (newly sequenced or retrieved) are represented by colours, while specimen attribute (terra typica, type locality, type specimen or faunistic novelty) is represented by the shape (right). Sequences: ID = 3, cf. = 0; maximum p-distance: strict = NA, less strict = 2.1 %.

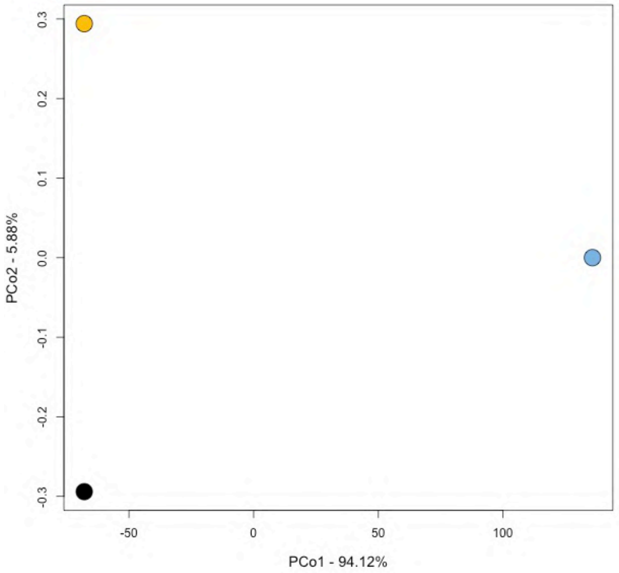

Figure 370: PCoA based on pairwise p-distances between *Lasius turcicus* sequences (left). Colours match a bidimensional colour space. Haplotype network analysis of *Lasius turcicus* was not possible.

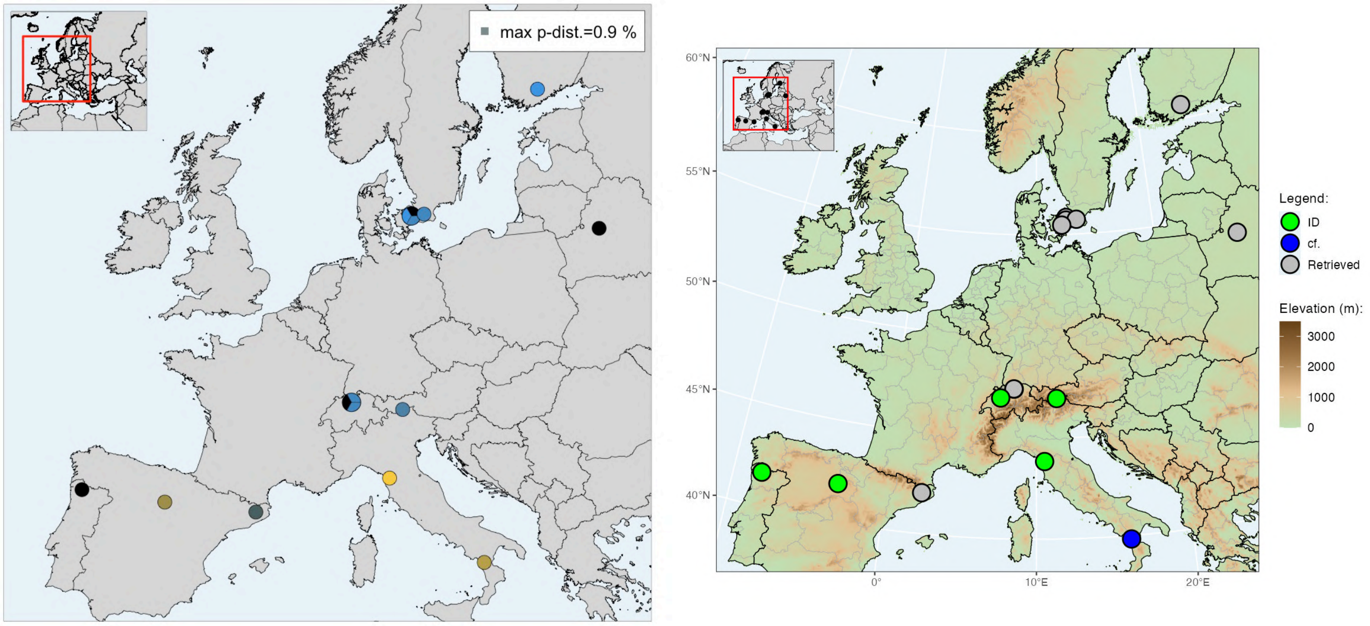

Figure 371: Genetic diversity map of *Lasius umbratus* (Nylander, 1846). Nearby localities of sequenced specimens are merged in pies (left). Colours match the bidimensional colour space of the PCoA projection (Fig. 371 left) of p-dist between sequences (dots). Specimen identification (ID or cf.) and source (newly sequenced or retrieved) are represented by colours, while specimen attribute (terra typica, type locality, type specimen or faunistic novelty) is represented by the shape (right). Sequences: ID = 14, cf. = 1; maximum p-distance: strict = 0.9 %, less strict = 0.9 %.

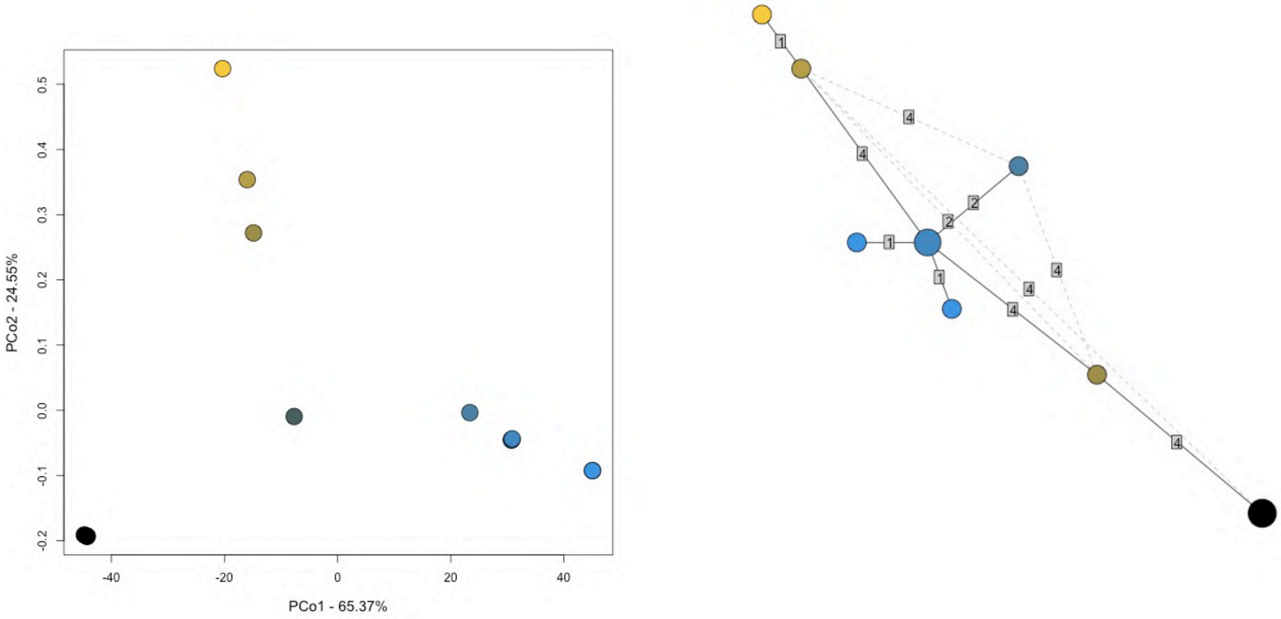

Figure 372: PCoA based on pairwise p-distances between *Lasius umbratus* sequences (left). Colours match a bidimensional colour space. Haplotype network of *Lasius umbratus* (right). Sequences > 599 bp: ID = 14, cf. = 1.

Lepisiota

Lepisiota frauenfeldi

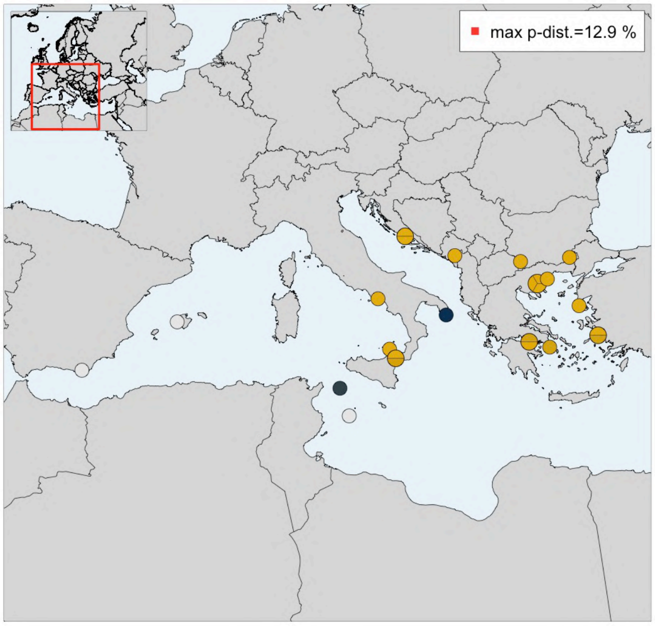

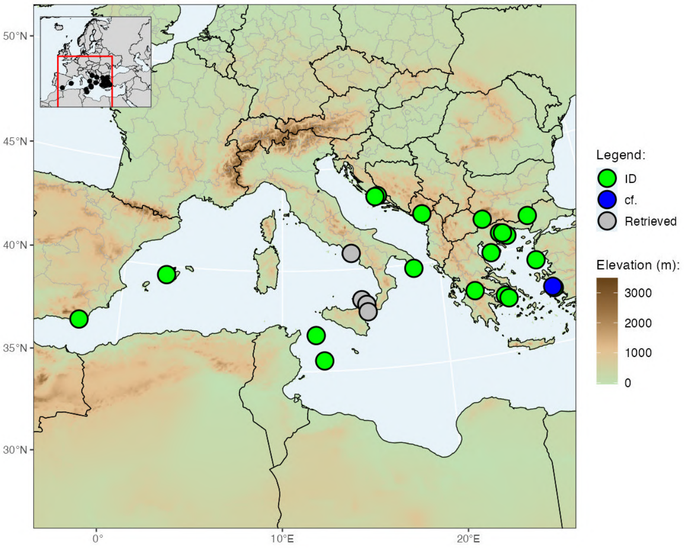

Figure 373: Genetic diversity map of *Lepisiota frauenfeldi* (Mayr, 1855). Nearby localities of sequenced specimens are merged in pies (left). Colours match the bidimensional colour space of the PCoA projection (Fig. 373 left) of p-dist between sequences (dots). Specimen identification (ID or cf.) and source (newly sequenced or retrieved) are represented by colours, while specimen attribute (terra typica, type locality, type specimen or faunistic novelty) is represented by the shape (right). Sequences: ID = 23, cf. = 1; maximum p-distance: strict = 12.9 %, less strict = 12.9 %.

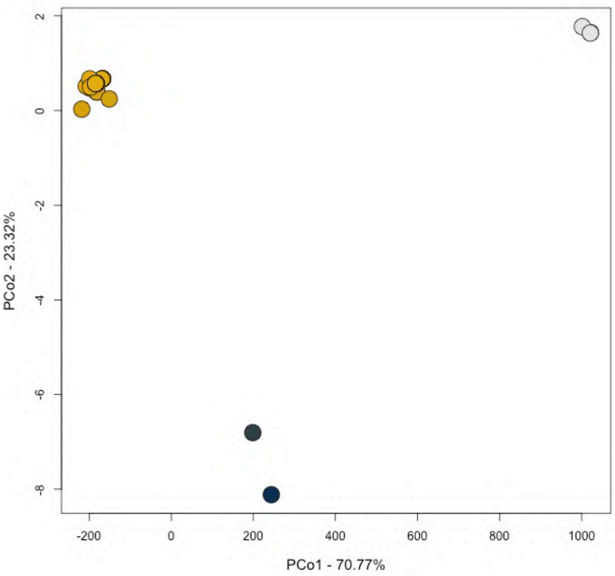

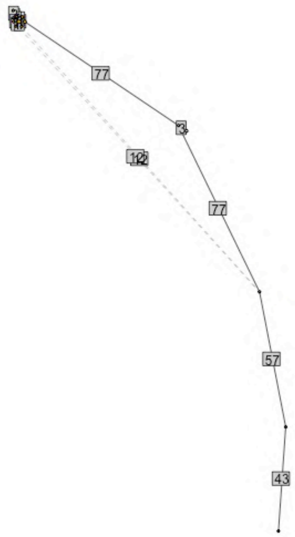

Figure 374: PCoA based on pairwise p-distances between *Lepisiota frauenfeldi* sequences (left). Colours match a bidimensional colour space. Haplotype network of *Lepisiota frauenfeldi* (right). Sequences > 599 bp: ID = 22, cf. = 1.

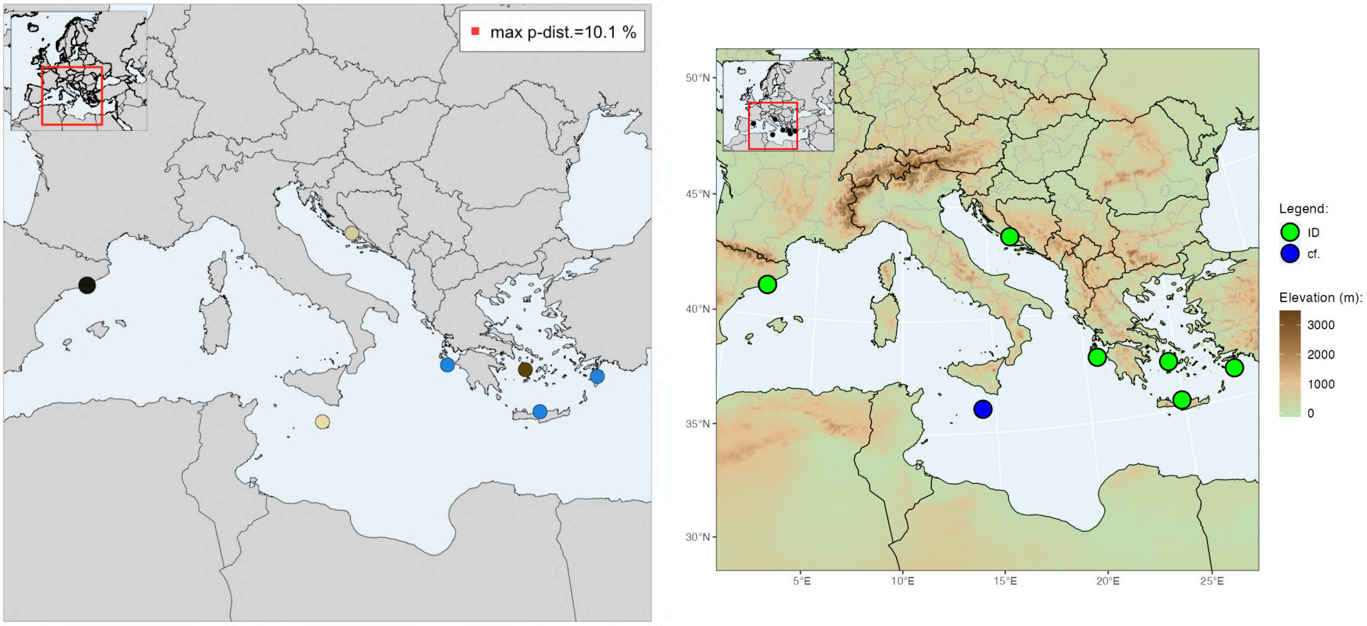

Figure 375: Genetic diversity map of *Lepisiota melas* (Emery, 1915). Nearby localities of sequenced specimens are merged in pies (left). Colours match the bidimensional colour space of the PCoA projection (Fig. 375 left) of p-dist between sequences (dots). Specimen identification (ID or cf.) and source (newly sequenced or retrieved) are represented by colours, while specimen attribute (terra typica, type locality, type specimen or faunistic novelty) is represented by the shape (right). Sequences: ID = 7, cf. = 1; maximum p-distance: strict = 10.1 %, less strict = 10.1 %.

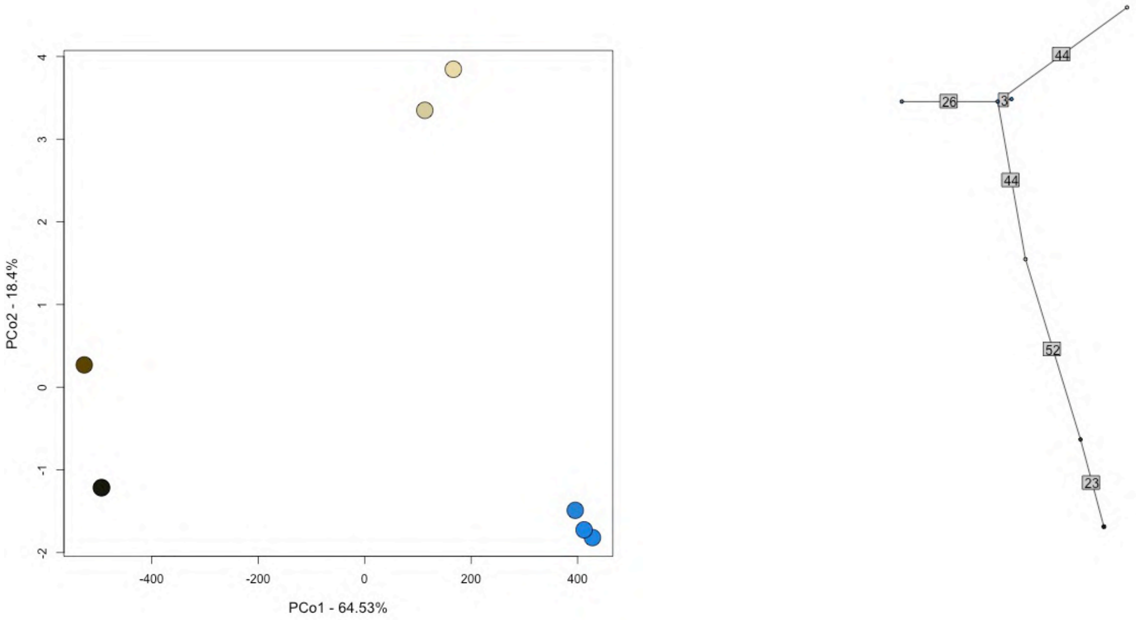

Figure 376: PCoA based on pairwise p-distances between *Lepisiota melas* sequences (left). Colours match a bidimensional colour space. Haplotype network of *Lepisiota melas* (right). Sequences > 599 bp: ID = 7, cf. = 1.

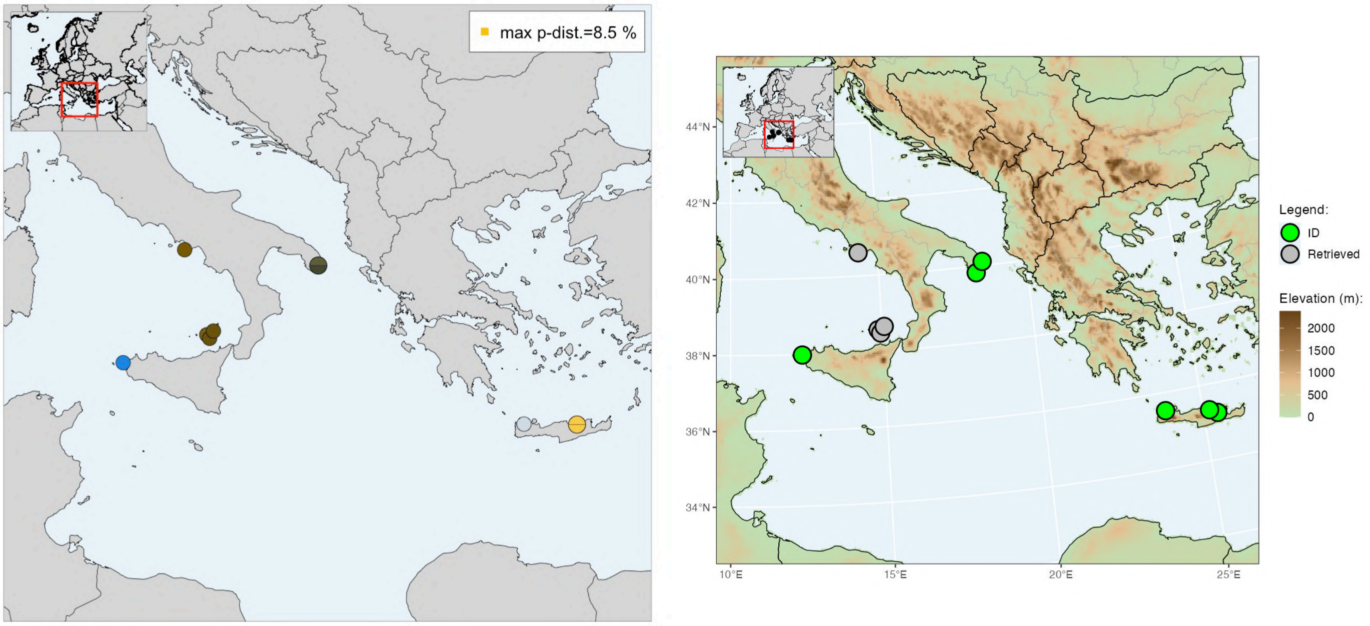

Figure 377: Genetic diversity map of *Lepisiota nigra* (Dalla Torre, 1893). Nearby localities of sequenced specimens are merged in pies (left). Colours match the bidimensional colour space of the PCoA projection (Fig. 377 left) of p-dist between sequences (dots). Specimen identification (ID or cf.) and source (newly sequenced or retrieved) are represented by colours, while specimen attribute (terra typica, type locality, type specimen or faunistic novelty) is represented by the shape (right). Sequences: ID = 10, cf. = 0; maximum p-distance: strict = 7.2 %, less strict = 8.5 %.

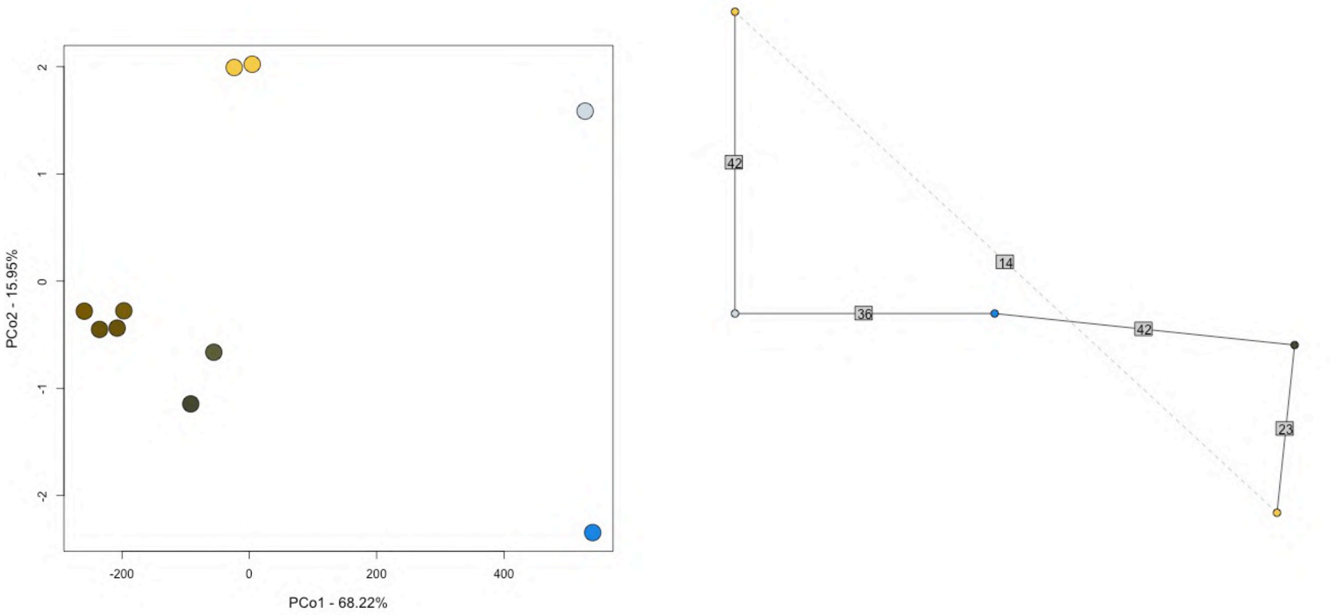

Figure 378: PCoA based on pairwise p-distances between *Lepisiota nigra* sequences (left). Colours match a bidimensional colour space. Haplotype network of *Lepisiota nigra* (right). Sequences > 599 bp: ID = 5, cf. = 0.

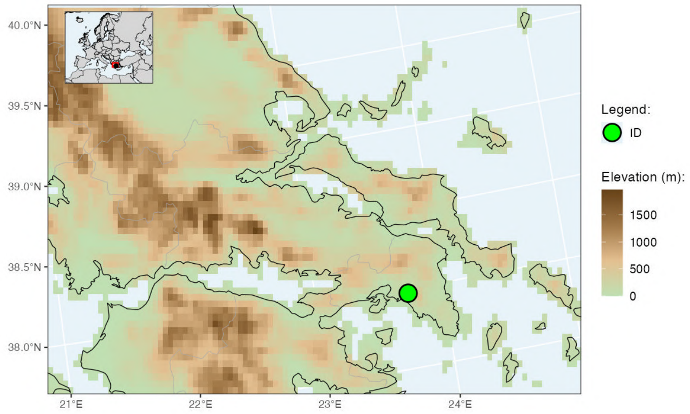

Figure 379: Map of *Lepisiota syriaca* (André, 1881). Due to the presence of a single sequence, the genetic diversity map and the PCoA projection were not done. Specimen identification (ID or cf.) and source (newly sequenced or retrieved) are represented by colours, while specimen attribute (terra typica, type locality, type specimen or faunistic novelty) is represented by the shape. Sequences: ID = 1, cf. = 0; maximum p-distance: strict = NA, less strict = NA.

Haplotype network analysis of *Lepisiota syriaca* was not possible.

Lepisiota velox

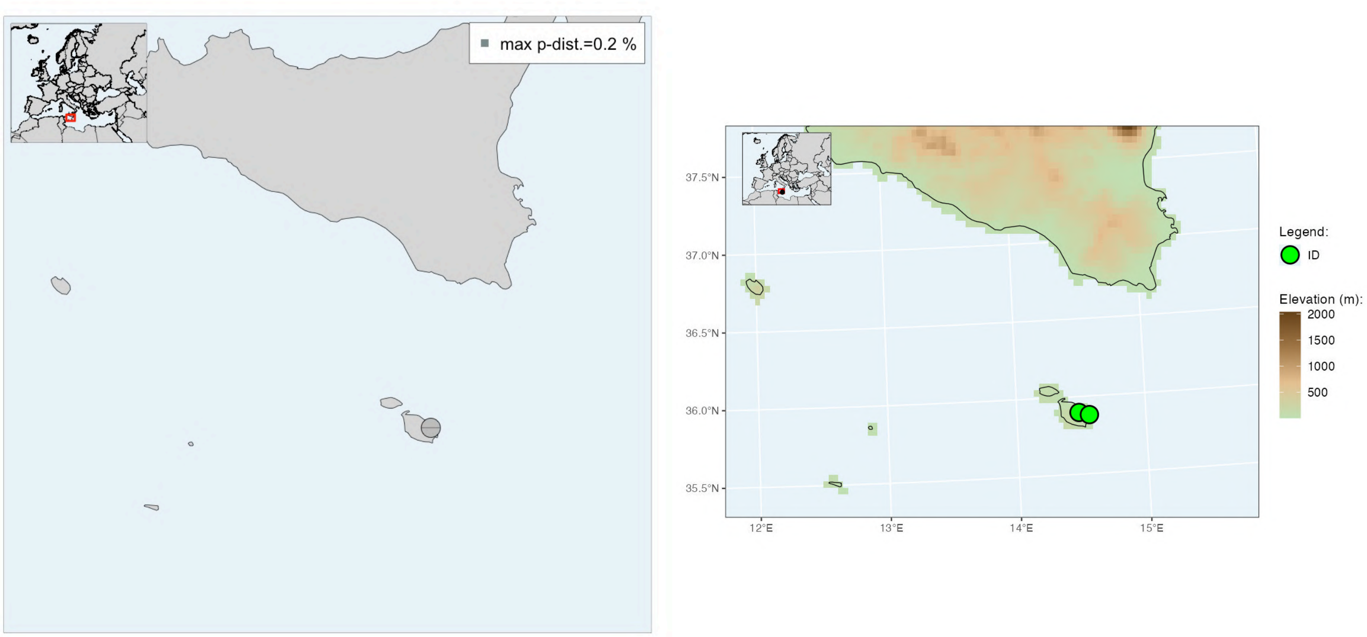

Figure 380: Genetic diversity map of *Lepisiota velox* (Baroni Urbani, 1968). PCoA projection was not done and therefore sequenced specimens in the genetic diversity map are coloured in gray (left). Specimen identification (ID or cf.) and source (newly sequenced or retrieved) are represented by colours, while specimen attribute (terra typica, type locality, type specimen or faunistic novelty) is represented by the shape (right). Sequences: ID = 2, cf. = 0; maximum p-distance: strict = NA, less strict = 0.2 %.

Haplotype network analysis of *Lepisiota velox* was not possible.

Leptanilla

Leptanilla charonea

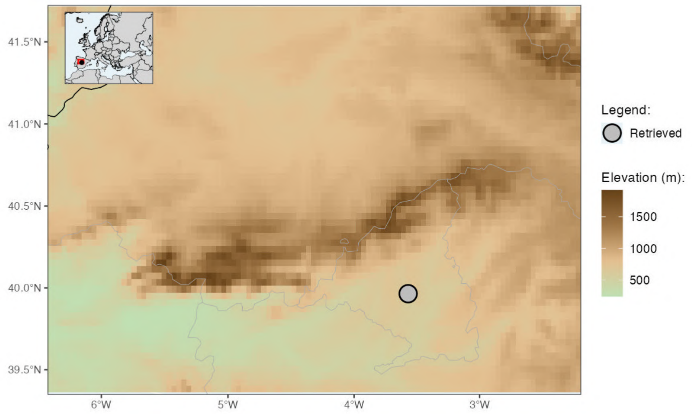

Figure 381: Map of *Leptanilla charonea* Barandica, López, Martínez & Ortuno, 1994. Due to the presence of a single sequence, the genetic diversity map and the PCoA projection were not done. Specimen identification (ID or cf.) and source (newly sequenced or retrieved) are represented by colours, while specimen attribute (terra typica, type locality, type specimen or faunistic novelty) is represented by the shape. Sequences: ID = 1, cf. = 0; maximum p-distance: strict = NA, less strict = NA.

Haplotype network analysis of *Leptanilla charonea* was not possible.

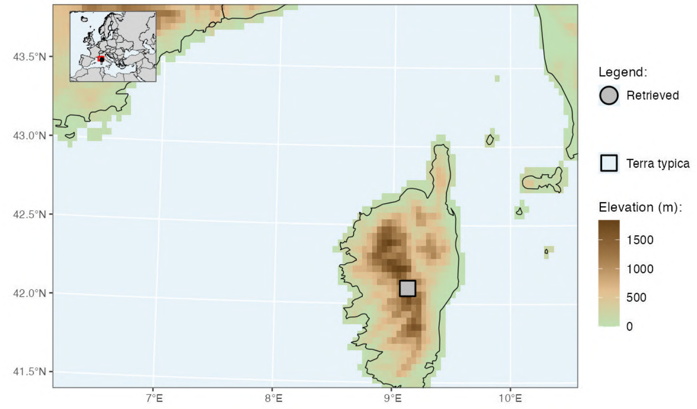

Figure 382: Map of *Leptanilla revelierii* Emery, 1870. Due to the presence of a single sequence, the genetic diversity map and the PCoA projection were not done. Specimen identification (ID or cf.) and source (newly sequenced or retrieved) are represented by colours, while specimen attribute (terra typica, type locality, type specimen or faunistic novelty) is represented by the shape. Sequences: ID = 1, cf. = 0; maximum p-distance: strict = NA, less strict = NA.

Haplotype network analysis of *Leptanilla revelierii* was not possible.

Leptanilla cf. zaballosi

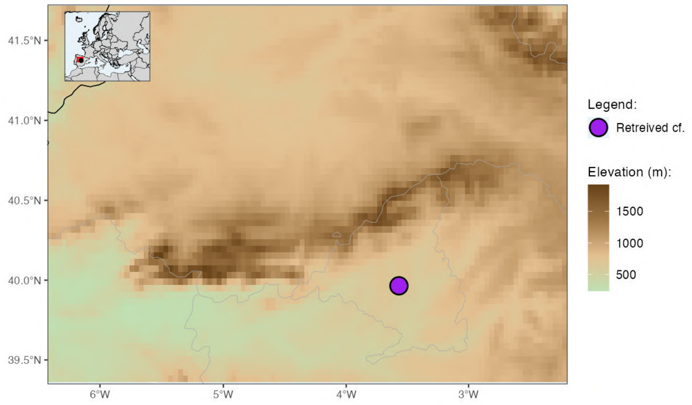

Figure 383: Map of *Leptanilla cf. zaballosi* Barandica, López, Martínez & Ortuno, 1994. Due to the presence of a single sequence, the genetic diversity map and the PCoA projection were not done. Specimen identification (ID or cf.) and source (newly sequenced or retrieved) are represented by colours, while specimen attribute (terra typica, type locality, type specimen or faunistic novelty) is represented by the shape. Sequences: ID = 0, cf. = 1; maximum p-distance: strict = NA, less strict = NA.

Haplotype network analysis of *Leptanilla cf. zaballosi* was not possible.

Leptothorax

Leptothorax acervorum

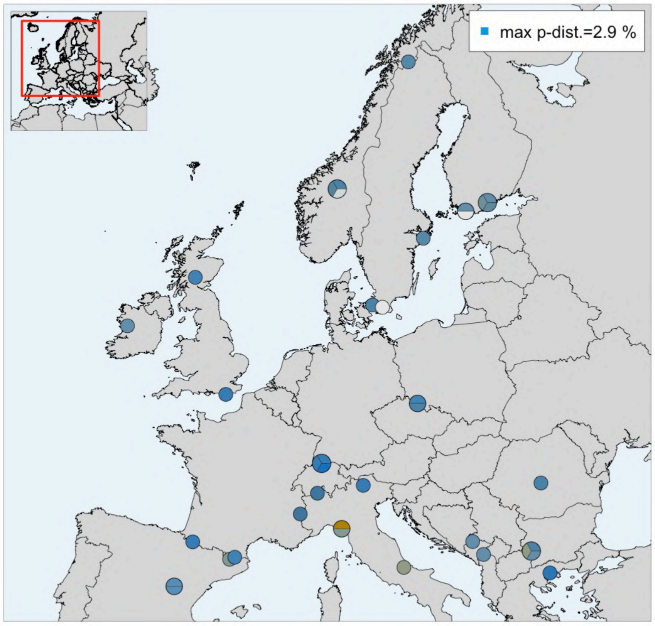

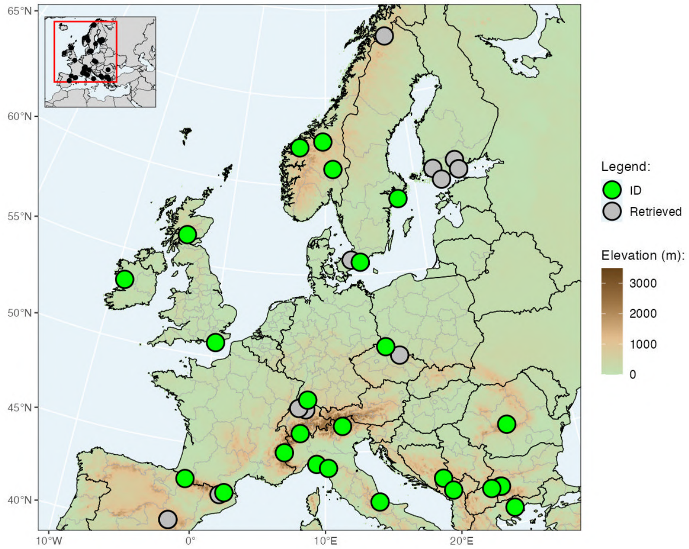

Figure 384: Genetic diversity map of *Leptothorax acervorum* (Fabricius, 1793). Nearby localities of sequenced specimens are merged in pies (left). Colours match the bidimensional colour space of the PCoA projection (Fig. 384 left) of p-dist between sequences (dots). Specimen identification (ID or cf.) and source (newly sequenced or retrieved) are represented by colours, while specimen attribute (terra typica, type locality, type specimen or faunistic novelty) is represented by the shape (right). Sequences: ID = 39, cf. = 0; maximum p-distance: strict = 2.9 %, less strict = 2.9 %.

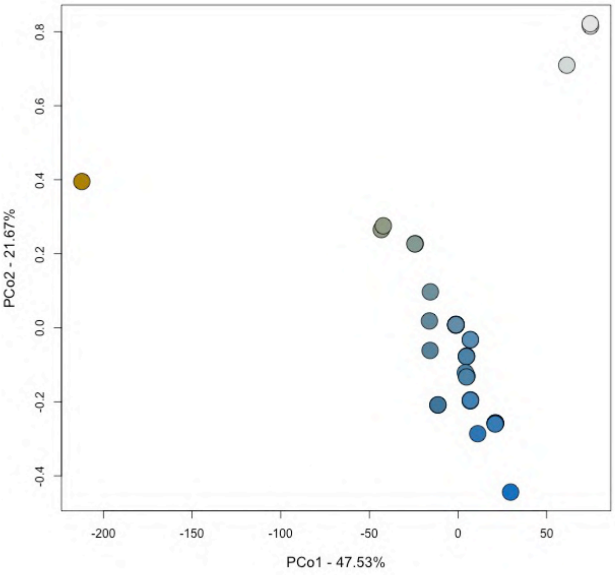

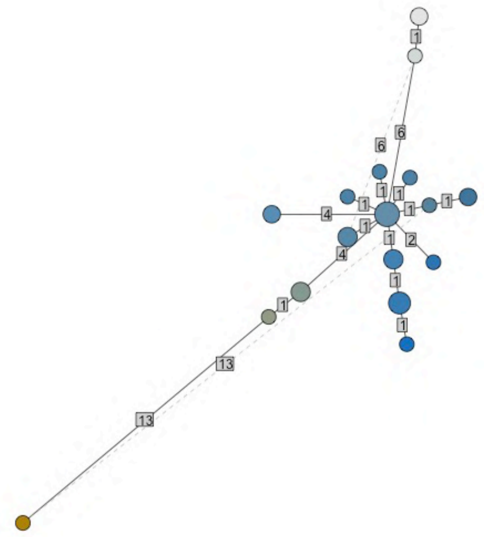

Figure 385: PCoA based on pairwise p-distances between *Leptothorax acervorum* sequences (left). Colours match a bidimensional colour space. Haplotype network of *Leptothorax acervorum* (right). Sequences > 599 bp: ID = 38, cf. = 0.

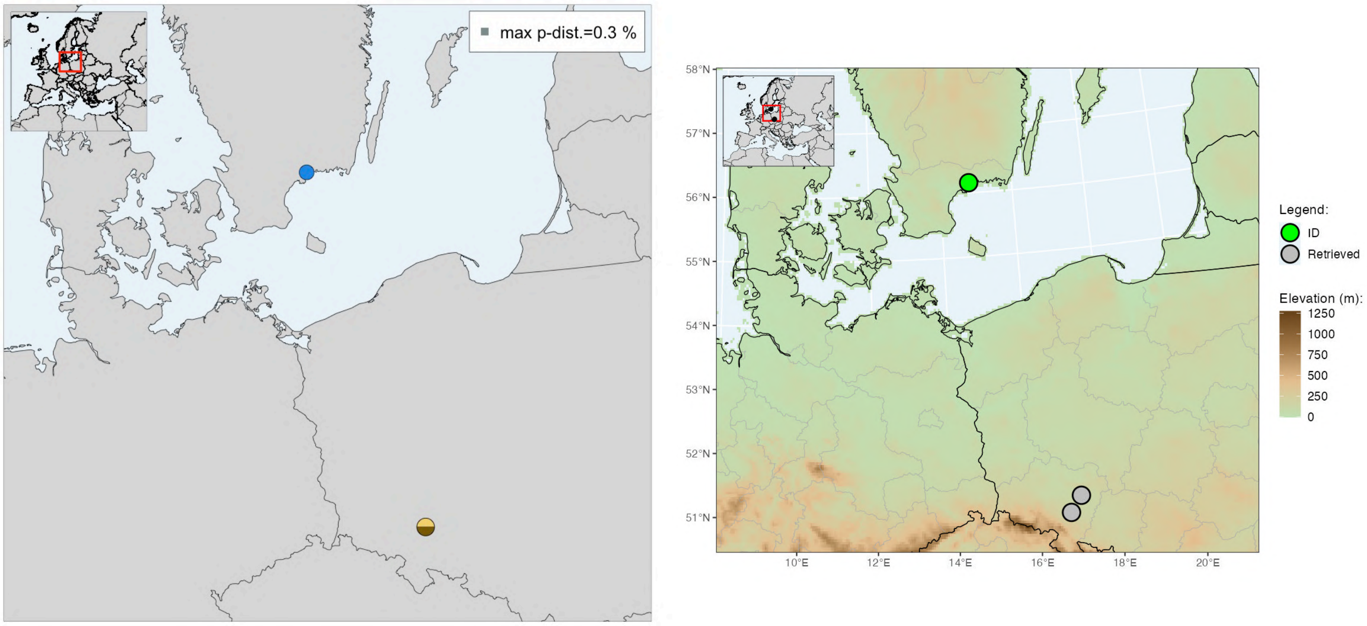

Figure 386: Genetic diversity map of *Leptothorax gredleri* Mayr, 1855. Nearby localities of sequenced specimens are merged in pies (left). Colours match the bidimensional colour space of the PCoA projection (Fig. 386 left) of p-dist between sequences (dots). Specimen identification (ID or cf.) and source (newly sequenced or retrieved) are represented by colours, while specimen attribute (terra typica, type locality, type specimen or faunistic novelty) is represented by the shape (right). Sequences: ID = 3, cf. = 0; maximum p-distance: strict = 0.3 %, less strict = 0.3 %.

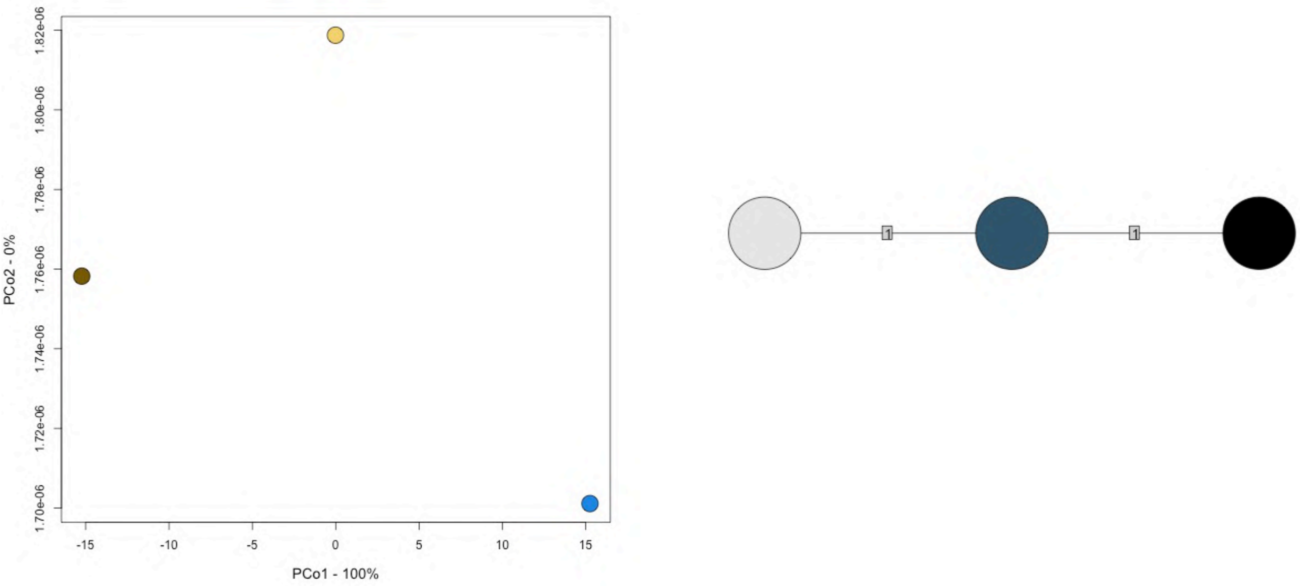

Figure 387: PCoA based on pairwise p-distances between *Leptothorax gredleri* sequences (left). Colours match a bidimensional colour space. Haplotype network of *Leptothorax gredleri* (right). Sequences > 599 bp: ID = 3, cf. = 0.

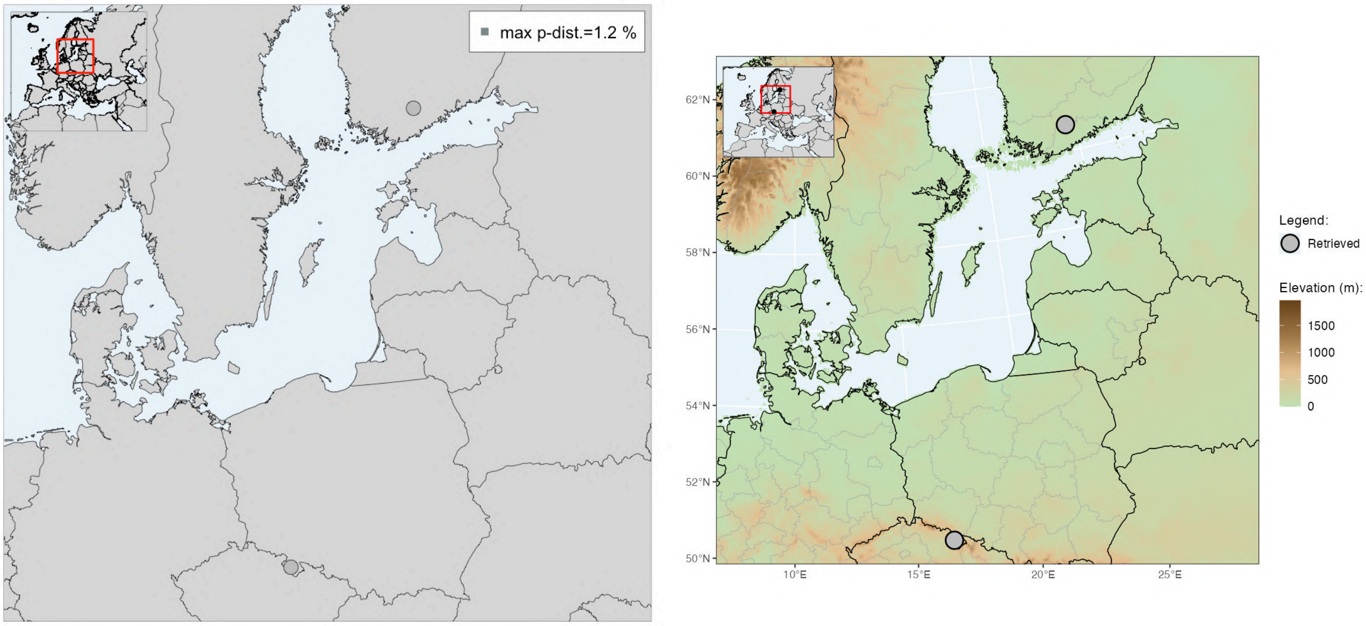

Figure 388: Genetic diversity map of *Leptothorax kutteri* Buschinger, 1966. PCoA projection was not done and therefore sequenced specimens in the genetic diversity map are coloured in gray (left). Specimen identification (ID or cf.) and source (newly sequenced or retrieved) are represented by colours, while specimen attribute (terra typica, type locality, type specimen or faunistic novelty) is represented by the shape (right). Sequences: ID = 2, cf. = 0; maximum p-distance: strict = NA, less strict = 1.2 %.

Haplotype network analysis of *Leptothorax kutteri* was not possible.

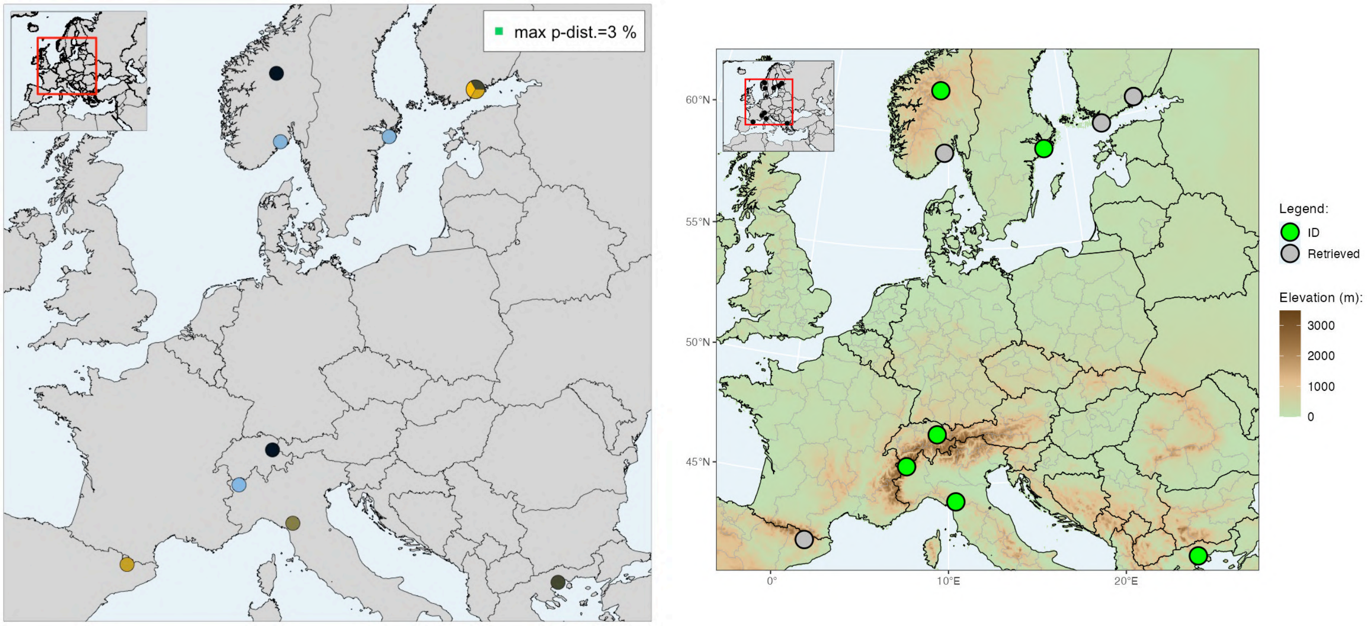

Figure 389: Genetic diversity map of *Leptothorax muscorum* (Nylander, 1846). Nearby localities of sequenced specimens are merged in pies (left). Colours match the bidimensional colour space of the PCoA projection (Fig. 389 left) of p-dist between sequences (dots). Specimen identification (ID or cf.) and source (newly sequenced or retrieved) are represented by colours, while specimen attribute (terra typica, type locality, type specimen or faunistic novelty) is represented by the shape (right). Sequences: ID = 11, cf. = 0; maximum p-distance: strict = 3 %, less strict = 3 %.

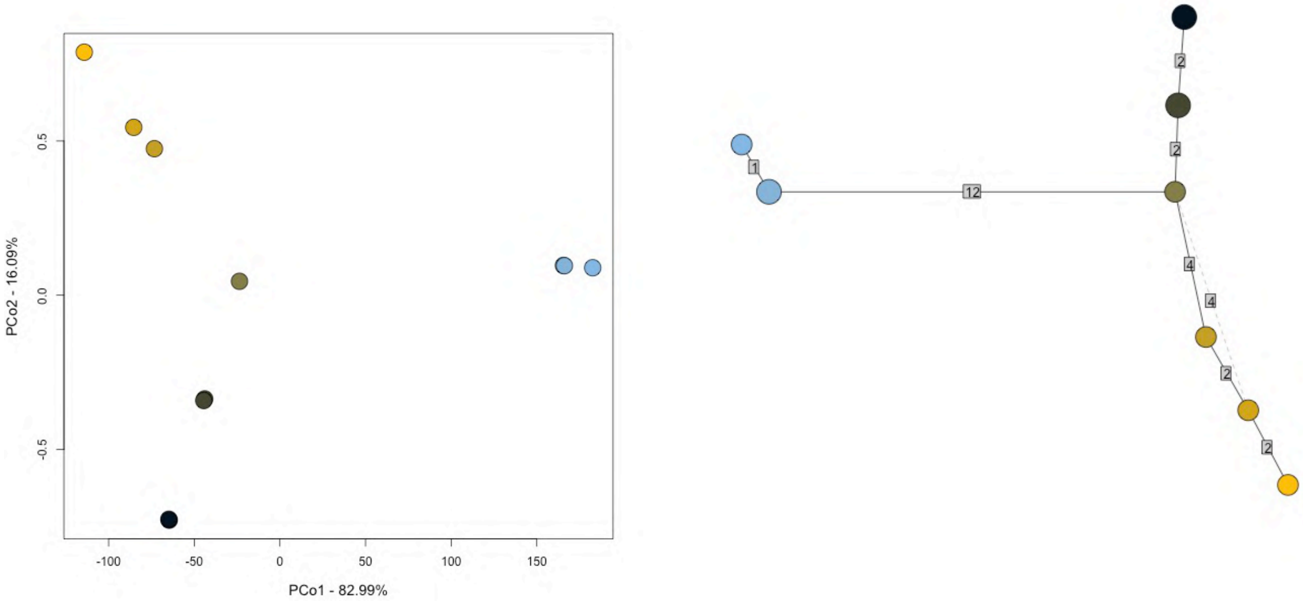

Figure 390: PCoA based on pairwise p-distances between *Leptothorax muscorum* sequences (left). Colours match a bidimensional colour space. Haplotype network of *Leptothorax muscorum* (right). Sequences > 599 bp: ID = 11, cf. = 0.

Linepithema

Linepithema humile

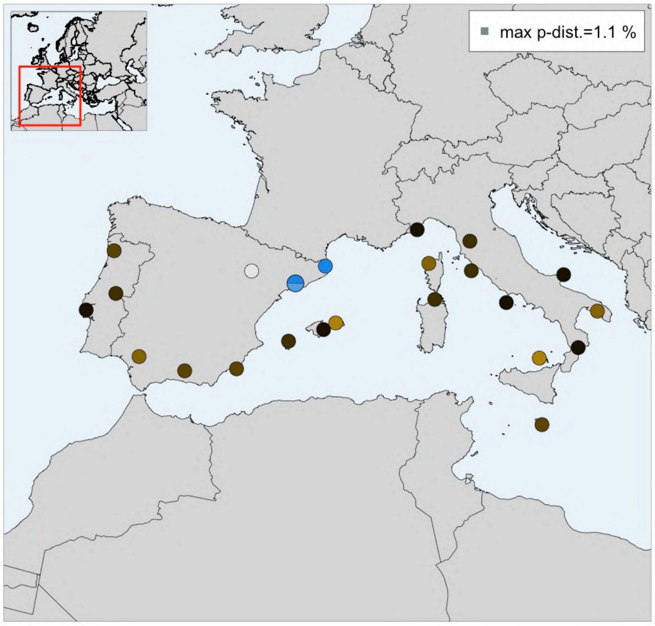

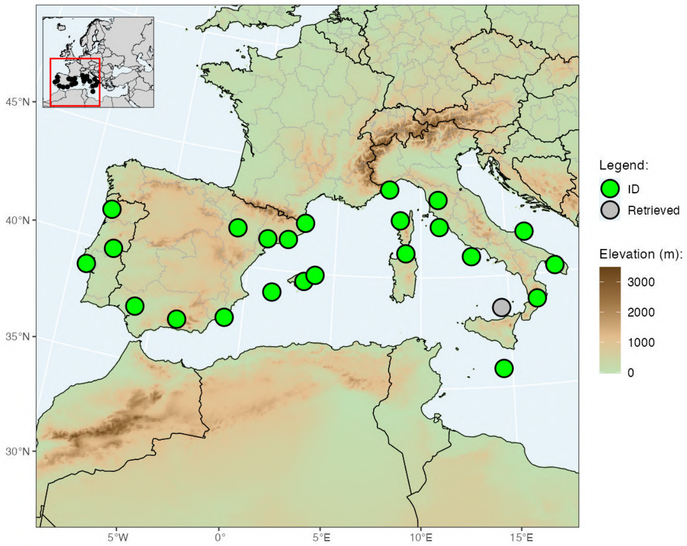

Figure 391: Genetic diversity map of *Linepithema humile* (Mayr, 1868). Nearby localities of sequenced specimens are merged in pies (left). Colours match the bidimensional colour space of the PCoA projection (Fig. 391 left) of p-dist between sequences (dots). Specimen identification (ID or cf.) and source (newly sequenced or retrieved) are represented by colours, while specimen attribute (terra typica, type locality, type specimen or faunistic novelty) is represented by the shape (right). Sequences: ID = 24, cf. = 0; maximum p-distance: strict = 1.1 %, less strict = 1.1 %.

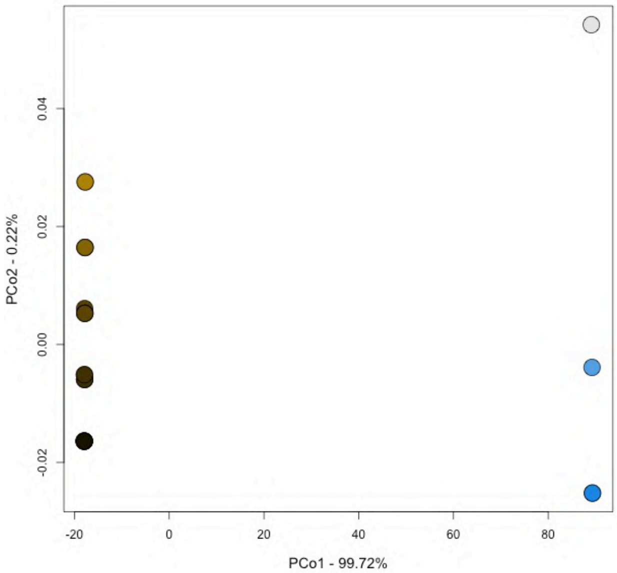

Figure 392: PCoA based on pairwise p-distances between *Linepithema humile* sequences (left). Colours match a bidimensional colour space. Haplotype network analysis of *Linepithema humile* was not possible.

Liometopum

Liometopum microcephalum

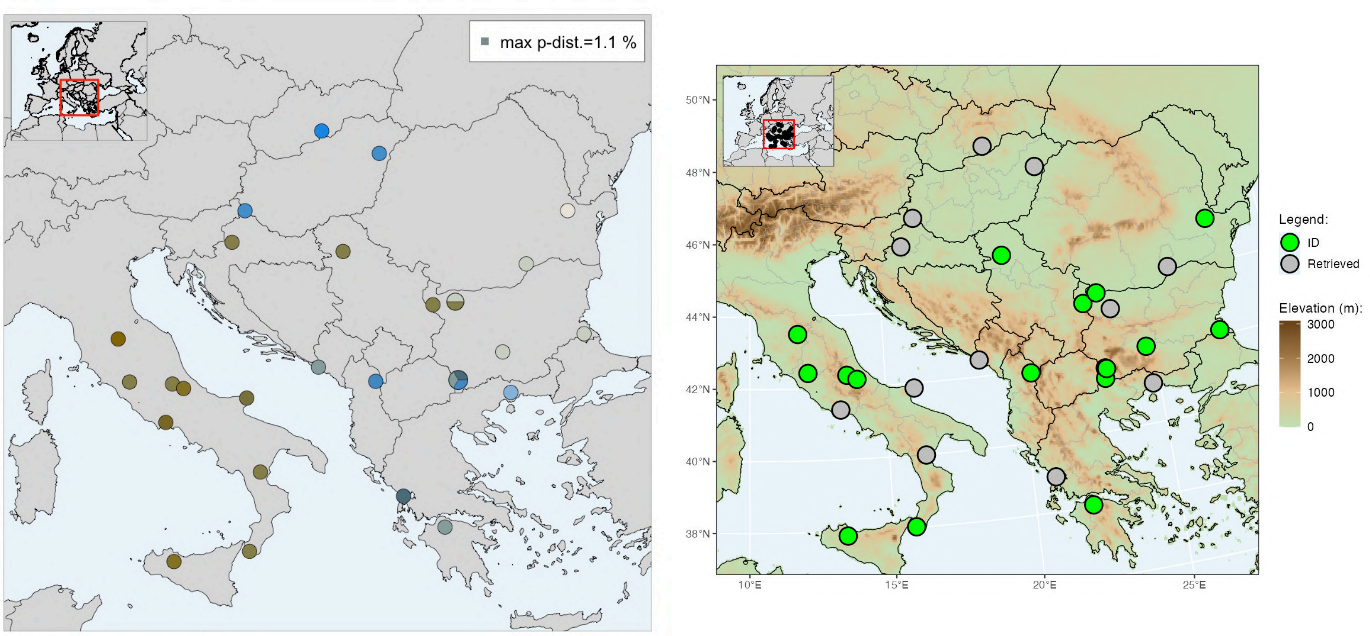

Figure 393: Genetic diversity map of *Liometopum microcephalum* (Panzer, 1798). Nearby localities of sequenced specimens are merged in pies (left). Colours match the bidimensional colour space of the PCoA projection (Fig. 393 left) of p-dist between sequences (dots). Specimen identification (ID or cf.) and source (newly sequenced or retrieved) are represented by colours, while specimen attribute (terra typica, type locality, type specimen or faunistic novelty) is represented by the shape (right). Sequences: ID = 29, cf. = 0; maximum p-distance: strict = 1.1 %, less strict = 1.1 %.

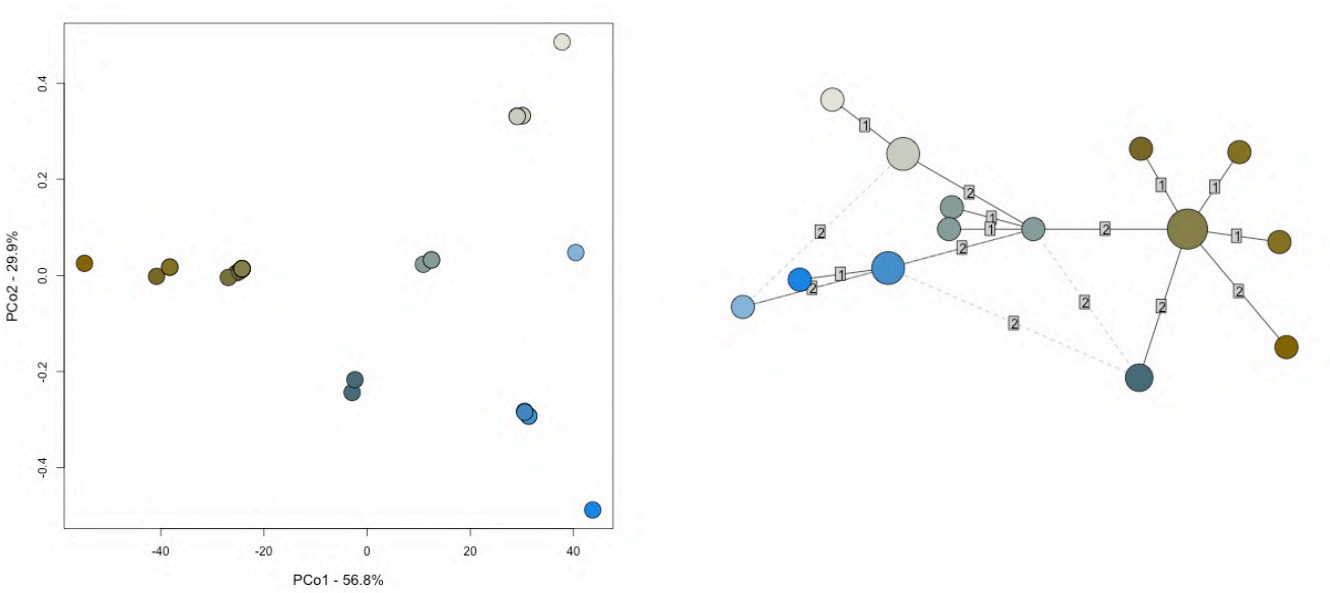

Figure 394: PCoA based on pairwise p-distances between *Liometopum microcephalum* sequences (left). Colours match a bidimensional colour space. Haplotype network of *Liometopum microcephalum* (right). Sequences > 599 bp: ID = 29, cf. = 0.

Manica

Manica rubida

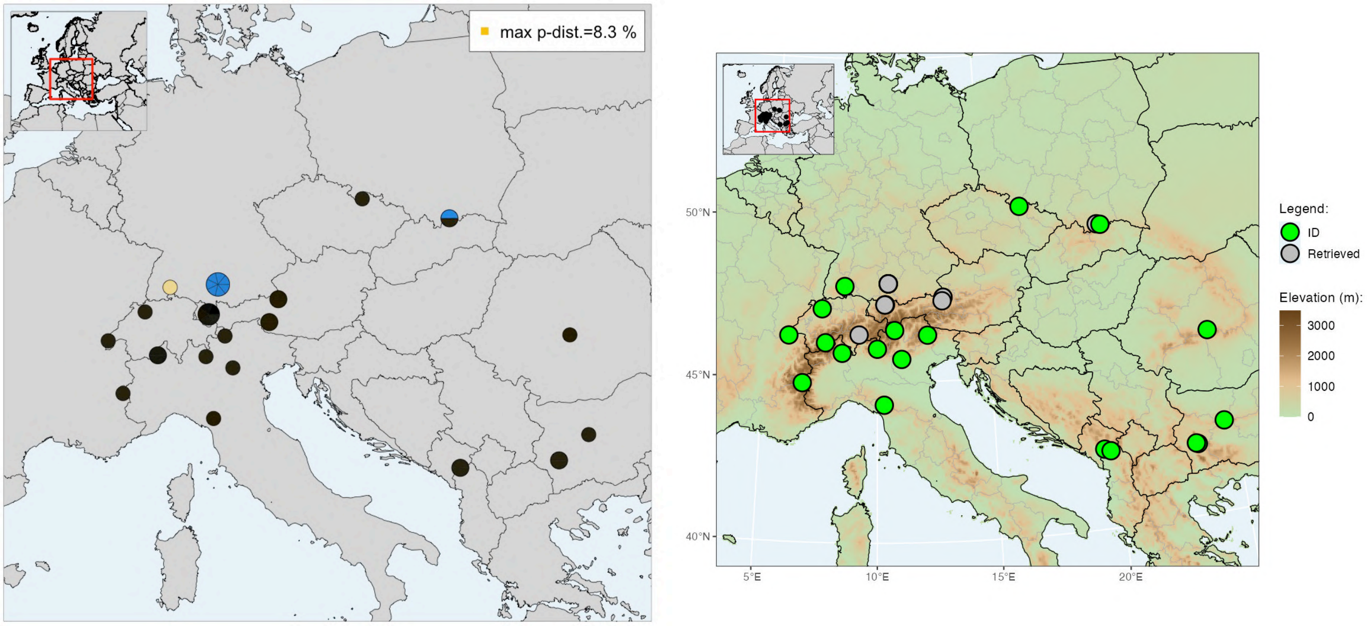

Figure 395: Genetic diversity map of *Manica rubida* (Latreille, 1802). Nearby localities of sequenced specimens are merged in pies (left). Colours match the bidimensional colour space of the PCoA projection (Fig. 395 left) of p-dist between sequences (dots). Specimen identification (ID or cf.) and source (newly sequenced or retrieved) are represented by colours, while specimen attribute (terra typica, type locality, type specimen or faunistic novelty) is represented by the shape (right). Sequences: ID = 37, cf. = 0; maximum p-distance: strict = 7.8 %, less strict = 8.3 %.

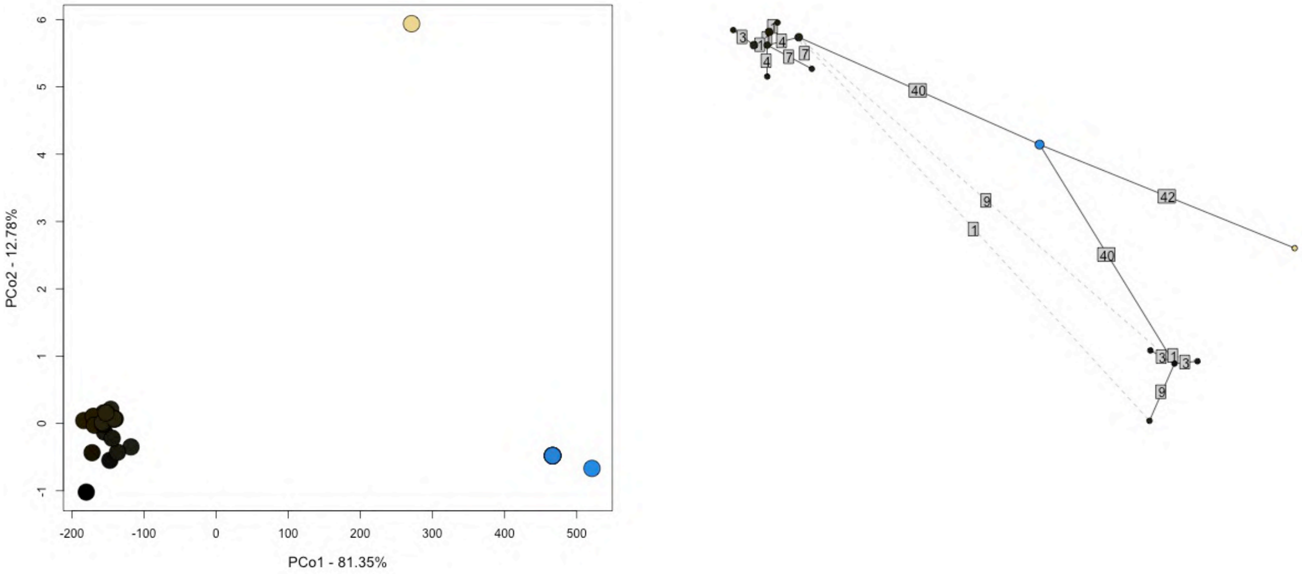

Figure 396: PCoA based on pairwise p-distances between *Manica rubida* sequences (left). Colours match a bidimensional colour space. Haplotype network of *Manica rubida* (right). Sequences > 599 bp: ID = 33, cf. = 0.

Messor

Messor atanassovii

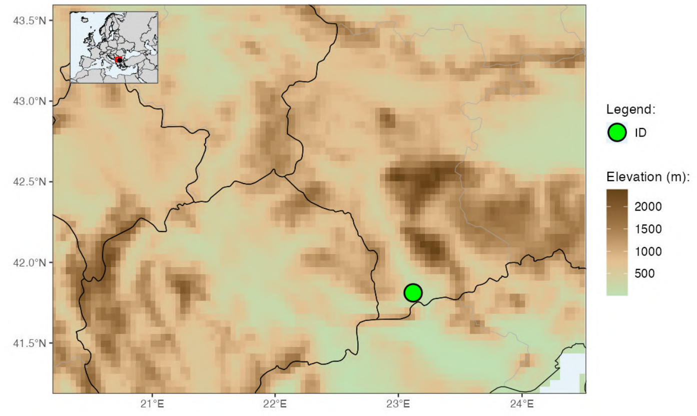

Figure 397: Map of *Messor atanassovii* Atanassov, 1982. Due to the presence of a single sequence, the genetic diversity map and the PCoA projection were not done. Specimen identification (ID or cf.) and source (newly sequenced or retrieved) are represented by colours, while specimen attribute (terra typica, type locality, type specimen or faunistic novelty) is represented by the shape. Sequences: ID = 1, cf. = 0; maximum p-distance: strict = NA, less strict = NA.

Haplotype network analysis of *Messor atanassovii* was not possible.

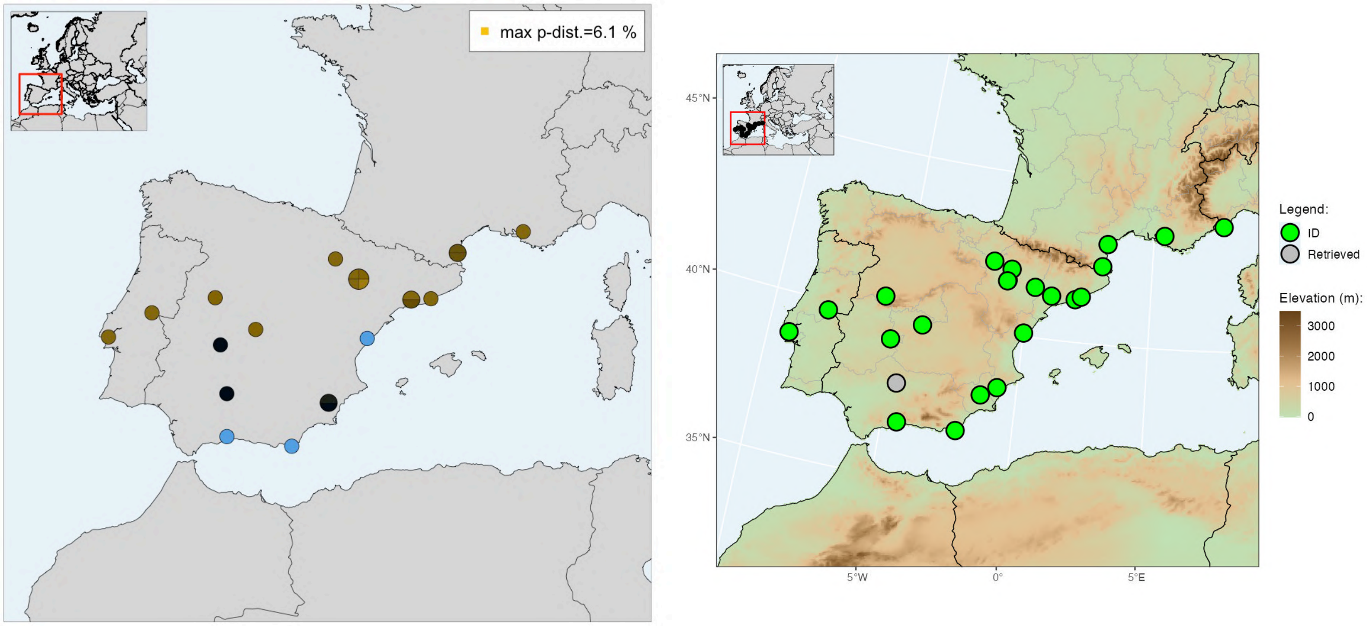

Figure 398: Genetic diversity map of *Messor barbarus* (Linnaeus, 1767). Nearby localities of sequenced specimens are merged in pies (left). Colours match the bidimensional colour space of the PCoA projection (Fig. 398 left) of p-dist between sequences (dots). Specimen identification (ID or cf.) and source (newly sequenced or retrieved) are represented by colours, while specimen attribute (terra typica, type locality, type specimen or faunistic novelty) is represented by the shape (right). Sequences: ID = 23, cf. = 0; maximum p-distance: strict = 6.1 %, less strict = 6.1 %.

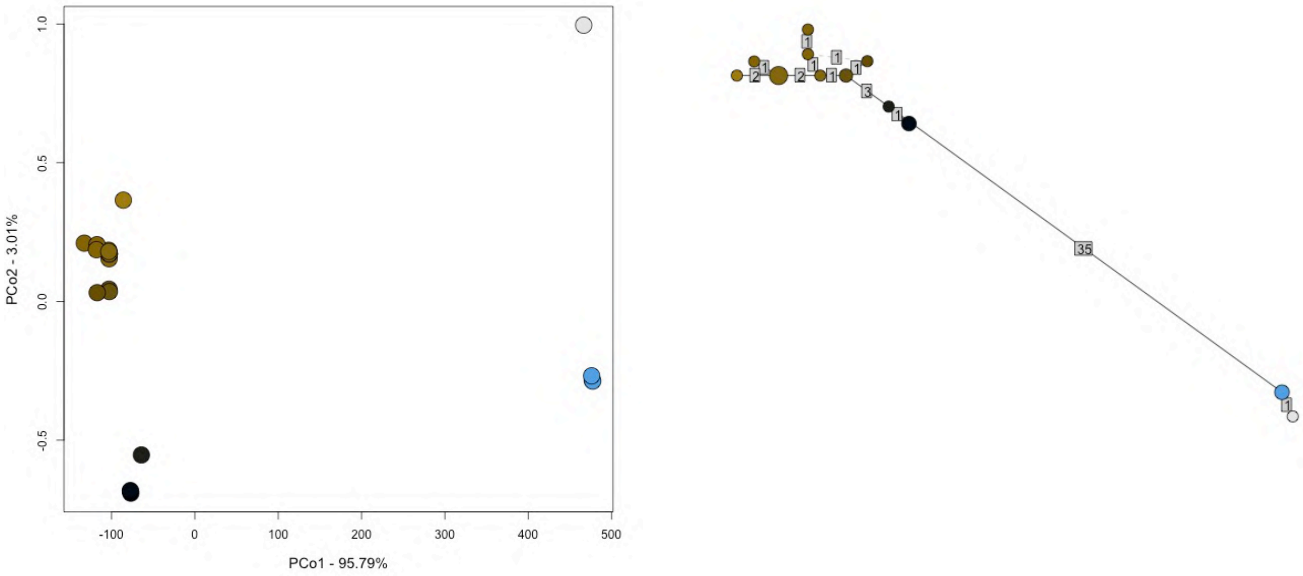

Figure 399: PCoA based on pairwise p-distances between *Messor barbarus* sequences (left). Colours match a bidimensional colour space. Haplotype network of *Messor barbarus* (right). Sequences > 599 bp: ID = 23, cf. = 0.

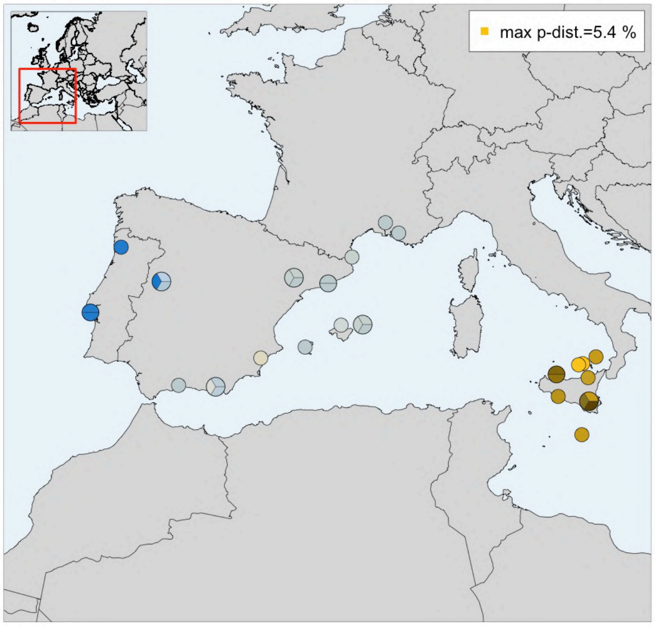

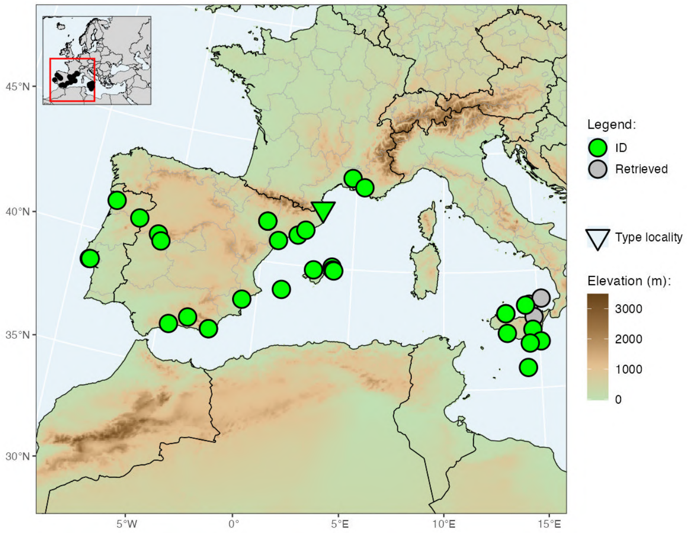

Figure 400: Genetic diversity map of *Messor bouvieri* Bondroit, 1918. Nearby localities of sequenced specimens are merged in pies (left). Colours match the bidimensional colour space of the PCoA projection (Fig. 400 left) of p-dist between sequences (dots). Specimen identification (ID or cf.) and source (newly sequenced or retrieved) are represented by colours, while specimen attribute (terra typica, type locality, type specimen or faunistic novelty) is represented by the shape (right). Sequences: ID = 35, cf. = 0; maximum p-distance: strict = 5.4 %, less strict = 5.4 %.

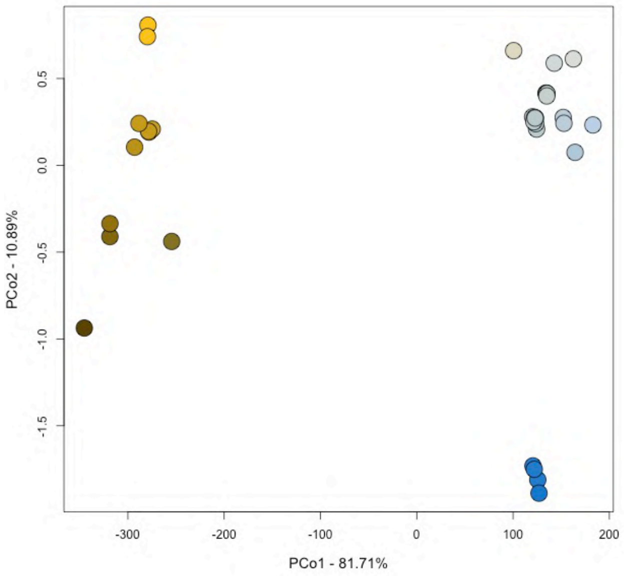

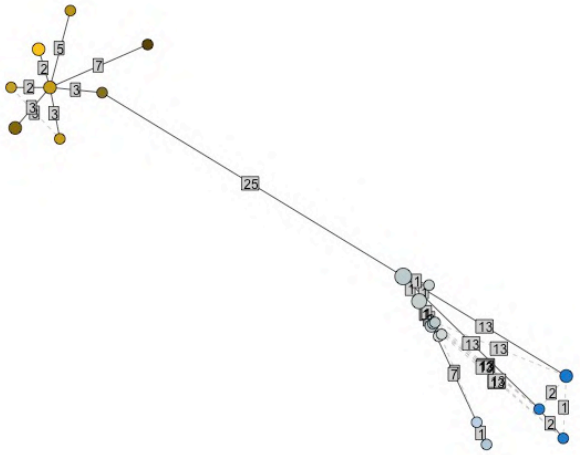

Figure 401: PCoA based on pairwise p-distances between *Messor bouvieri* sequences (left). Colours match a bidimensional colour space. Haplotype network of *Messor bouvieri* (right). Sequences > 599 bp: ID = 34, cf. = 0.

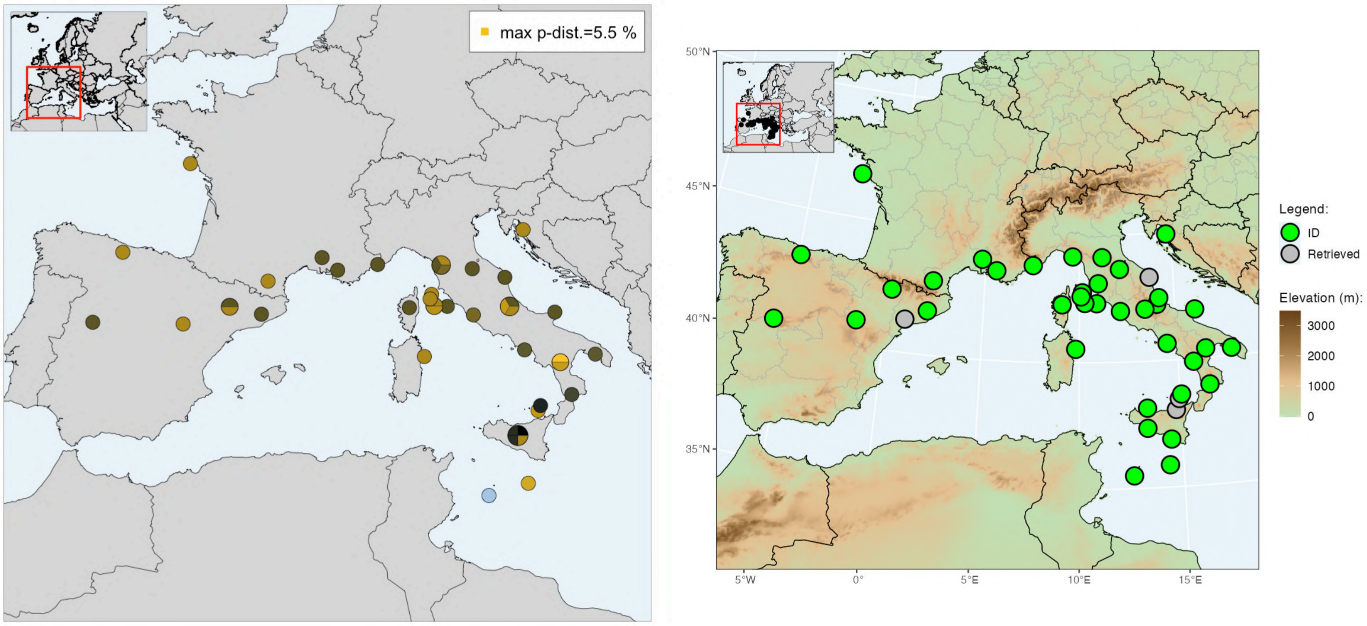

Figure 402: Genetic diversity map of *Messor capitatus* (Latreille, 1798). Nearby localities of sequenced specimens are merged in pies (left). Colours match the bidimensional colour space of the PCoA projection (Fig. 402 left) of p-dist between sequences (dots). Specimen identification (ID or cf.) and source (newly sequenced or retrieved) are represented by colours, while specimen attribute (terra typica, type locality, type specimen or faunistic novelty) is represented by the shape (right). Sequences: ID = 42, cf. = 0; maximum p-distance: strict = 5.5 %, less strict = 5.5 %.

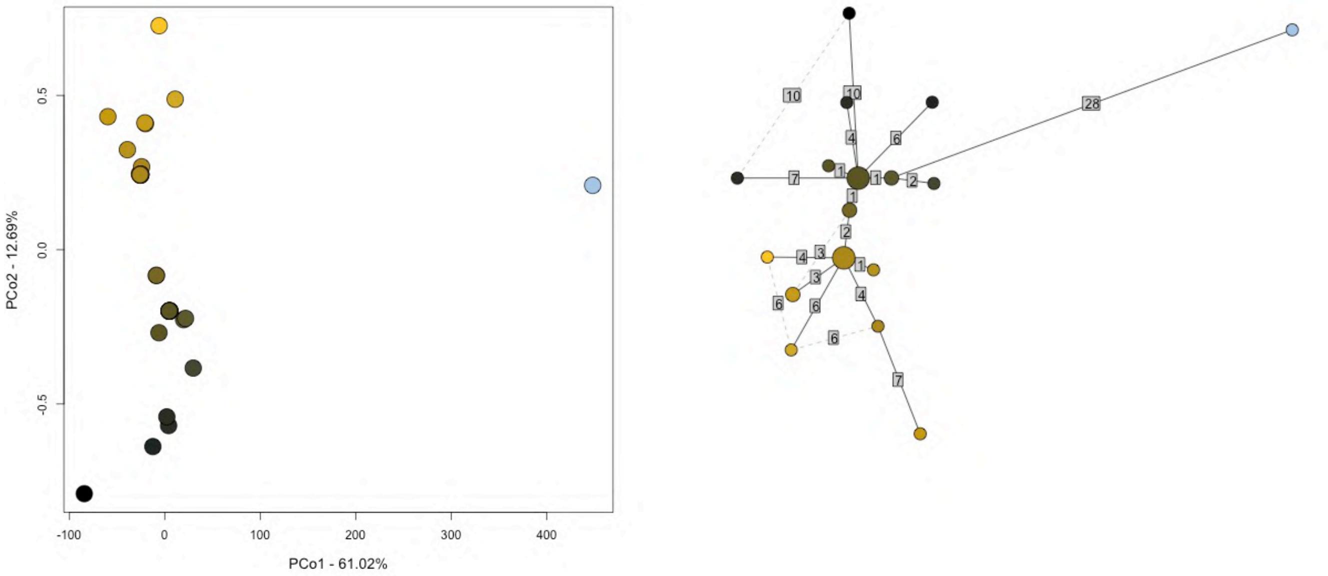

Figure 403: PCoA based on pairwise p-distances between *Messor capitatus* sequences (left). Colours match a bidimensional colour space. Haplotype network of *Messor capitatus* (right). Sequences > 599 bp: ID = 42, cf. = 0.

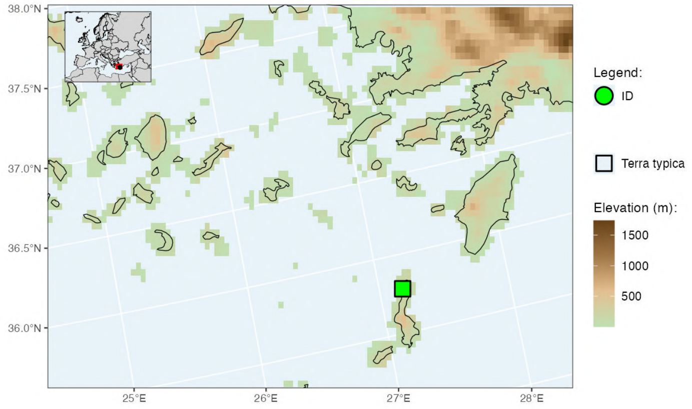

Figure 404: Map of *Messor carpathous* Menozzi, 1936. Due to the presence of a single sequence, the genetic diversity map and the PCoA projection were not done. Specimen identification (ID or cf.) and source (newly sequenced or retrieved) are represented by colours, while specimen attribute (terra typica, type locality, type specimen or faunistic novelty) is represented by the shape. Sequences: ID = 1, cf. = 0; maximum p-distance: strict = NA, less strict = NA.

Haplotype network analysis of *Messor carpathous* was not possible.

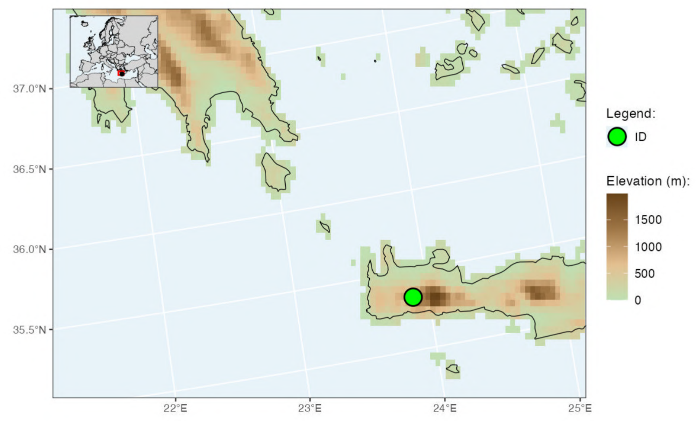

Figure 405: Map of *Messor creticus* Salata & Borowiec, 2019. Due to the presence of a single sequence, the genetic diversity map and the PCoA projection were not done. Specimen identification (ID or cf.) and source (newly sequenced or retrieved) are represented by colours, while specimen attribute (terra typica, type locality, type specimen or faunistic novelty) is represented by the shape. Sequences: ID = 1, cf. = 0; maximum p-distance: strict = NA, less strict = NA.

Haplotype network analysis of *Messor creticus* was not possible.

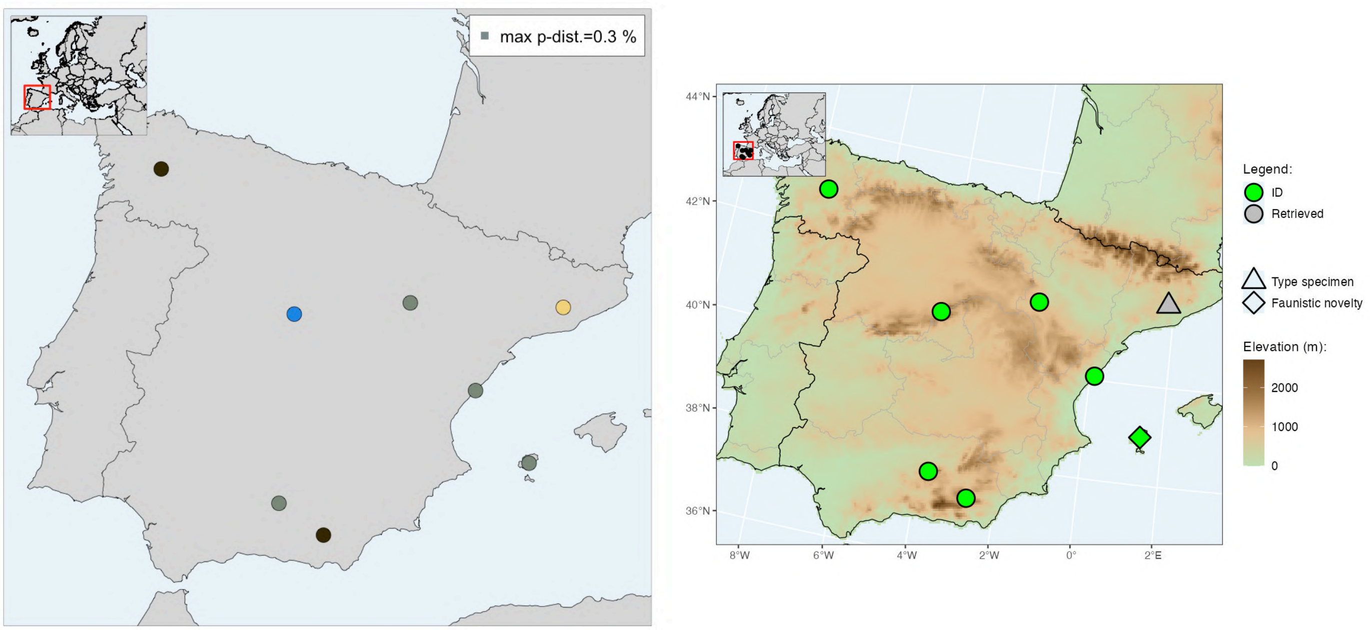

Figure 406: Genetic diversity map of *Messor erwini* Orou, Csősz, Arnan, Pol, Arthofe, Schlick-Steiner & Steiner 2023. Nearby localities of sequenced specimens are merged in pies (left). Colours match the bidimensional colour space of the PCoA projection (Fig. 406 left) of p-dist between sequences (dots). Specimen identification (ID or cf.) and source (newly sequenced or retrieved) are represented by colours, while specimen attribute (terra typica, type locality, type specimen or faunistic novelty) is represented by the shape (right). Sequences: ID = 8, cf. = 0; maximum p-distance: strict = 0.3 %, less strict = 0.3 %.

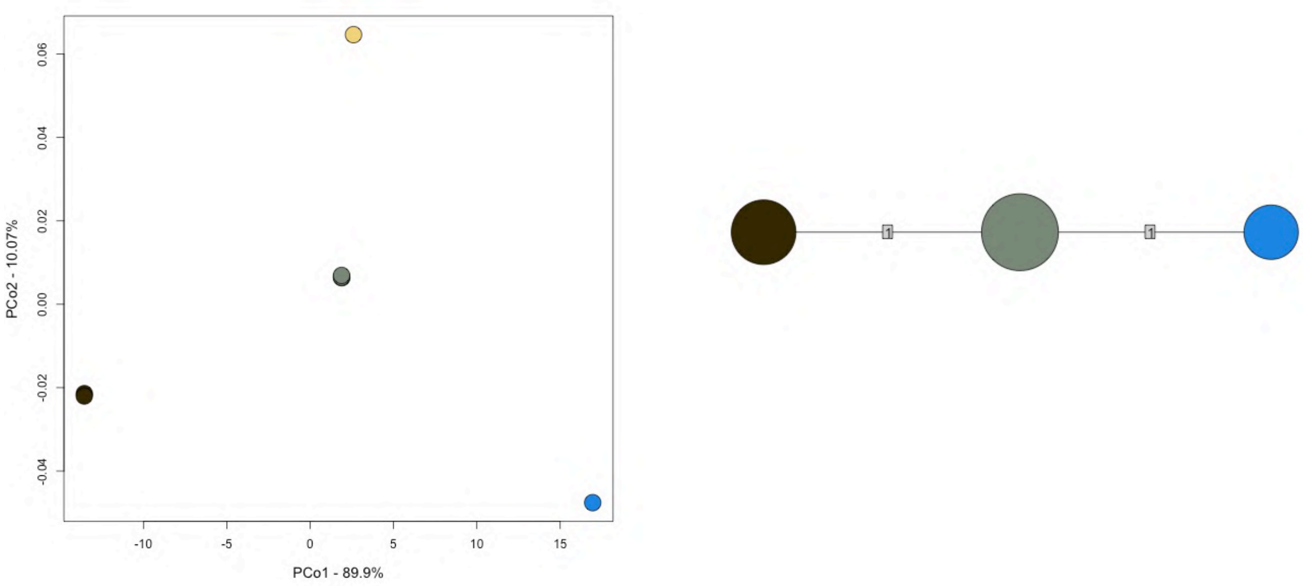

Figure 407: PCoA based on pairwise p-distances between *Messor erwini* sequences (left). Colours match a bidimensional colour space. Haplotype network of *Messor erwini* (right). Sequences > 599 bp: ID = 7, cf. = 0.

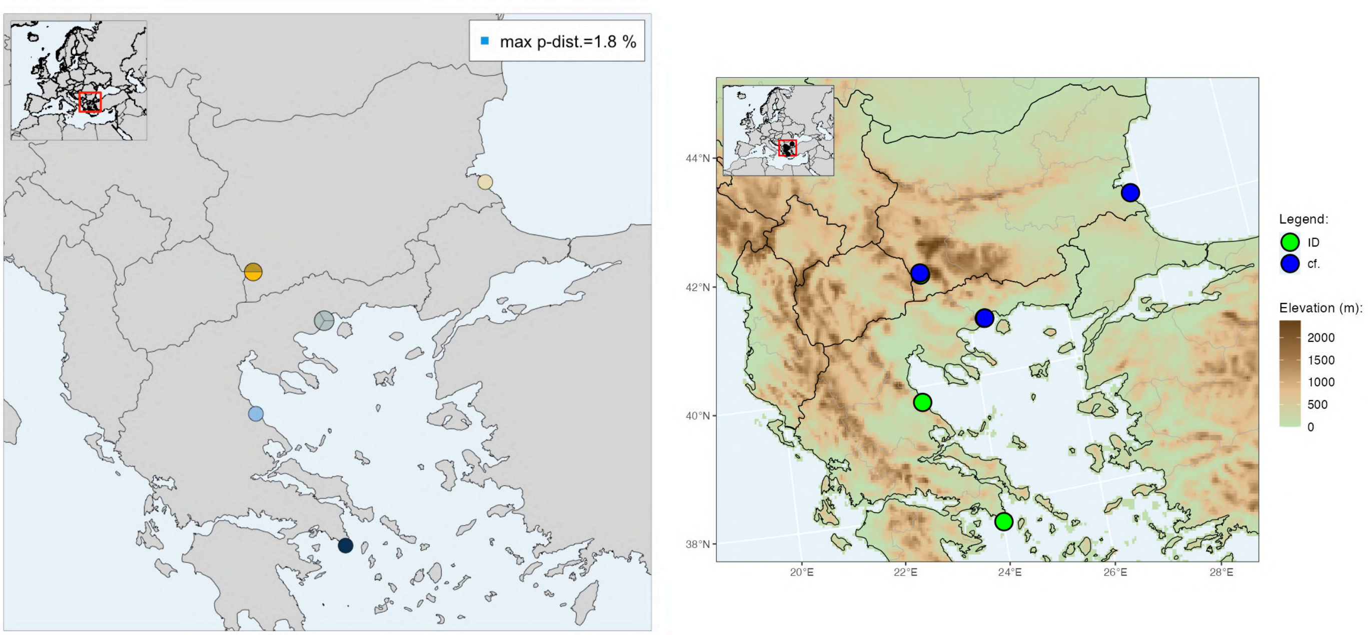

Figure 408: Genetic diversity map of *Messor hellenius* Agosti & Collingwood, 1987. Nearby localities of sequenced specimens are merged in pies (left). Colours match the bidimensional colour space of the PCoA projection (Fig. 408 left) of p-dist between sequences (dots). Specimen identification (ID or cf.) and source (newly sequenced or retrieved) are represented by colours, while specimen attribute (terra typica, type locality, type specimen or faunistic novelty) is represented by the shape (right). Sequences: ID = 5, cf. = 3; maximum p-distance: strict = 1.5 %, less strict = 1.8 %.

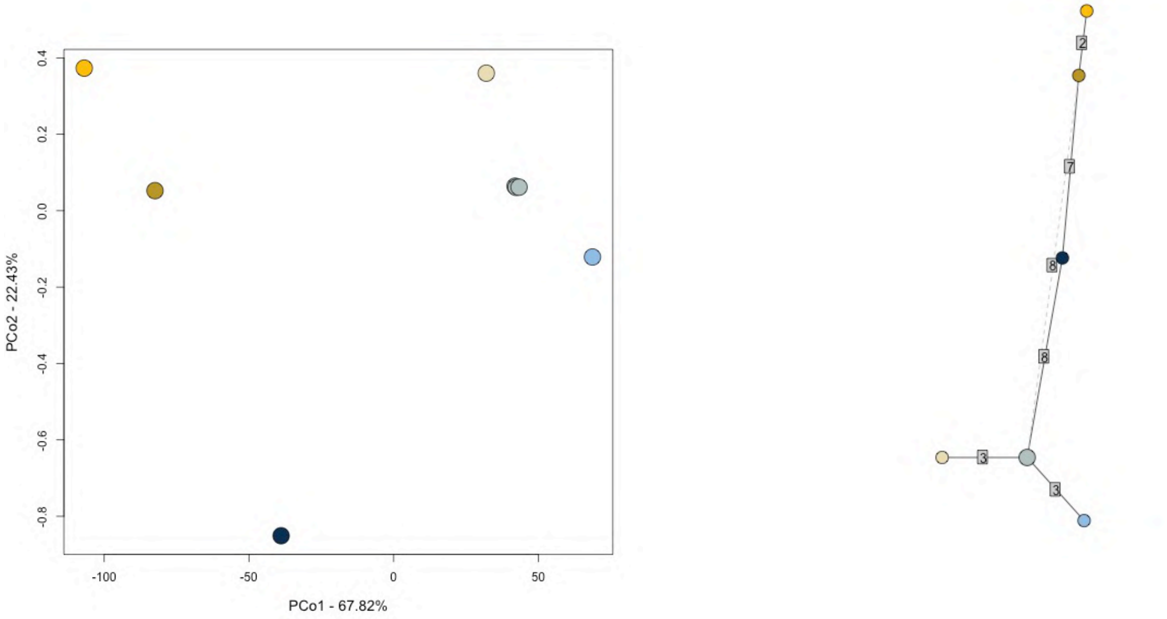

Figure 409: PCoA based on pairwise p-distances between *Messor hellenius* sequences (left). Colours match a bidimensional colour space. Haplotype network of *Messor hellenius* (right). Sequences > 599 bp: ID = 5, cf. = 3.

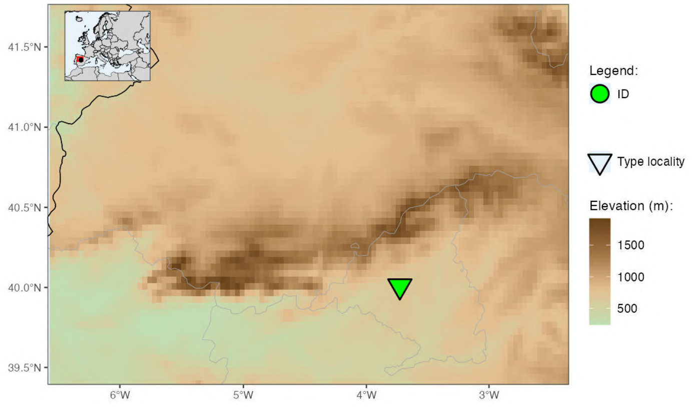

Figure 410: Map of *Messor hispanicus* Santschi, 1919. Due to the presence of a single sequence, the genetic diversity map and the PCoA projection were not done. Specimen identification (ID or cf.) and source (newly sequenced or retrieved) are represented by colours, while specimen attribute (terra typica, type locality, type specimen or faunistic novelty) is represented by the shape. Sequences: ID = 1, cf. = 0; maximum p-distance: strict = NA, less strict = NA.

Haplotype network analysis of *Messor hispanicus* was not possible.

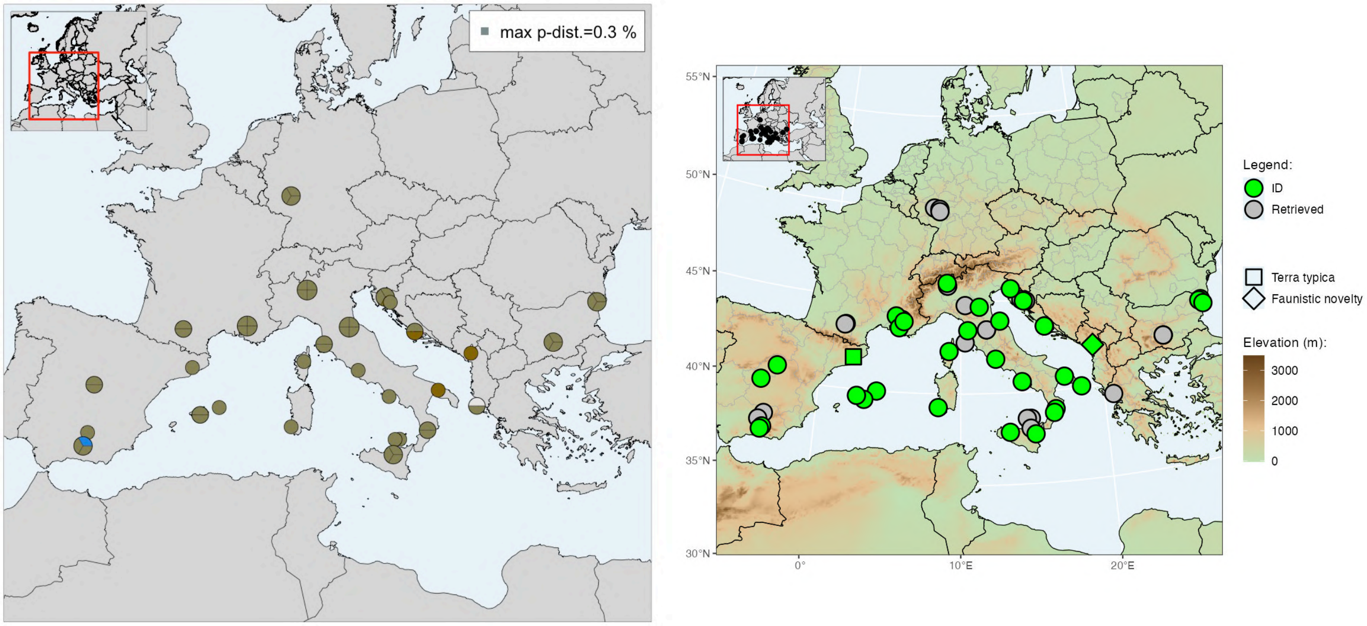

Figure 411: Genetic diversity map of *Messor ibericus* Santschi, 1931. Nearby localities of sequenced specimens are merged in pies (left). Colours match the bidimensional colour space of the PCoA projection (Fig. 411 left) of p-dist between sequences (dots). Specimen identification (ID or cf.) and source (newly sequenced or retrieved) are represented by colours, while specimen attribute (terra typica, type locality, type specimen or faunistic novelty) is represented by the shape (right). Sequences: ID = 56, cf. = 0; maximum p-distance: strict = 0.3 %, less strict = 0.3 %.

The species is reported for the first time in Albania.

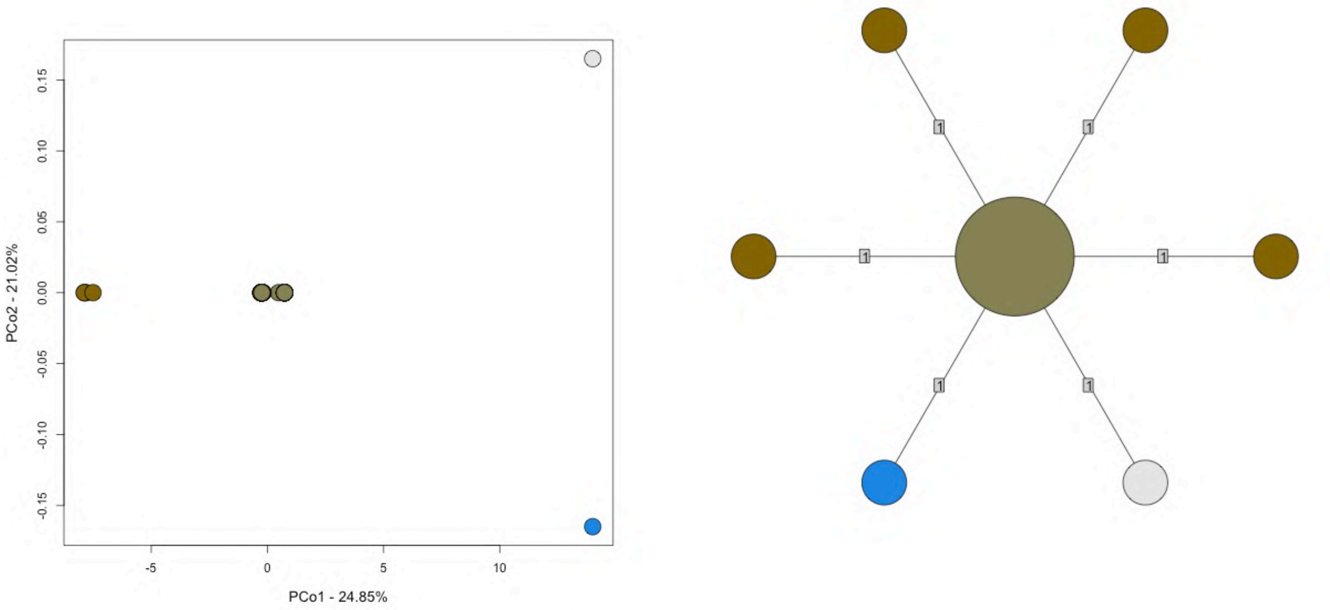

Figure 412: PCoA based on pairwise p-distances between *Messor ibericus* sequences (left). Colours match a bidimensional colour space. Haplotype network of *Messor ibericus* (right). Sequences > 599 bp: ID = 56, cf. = 0.

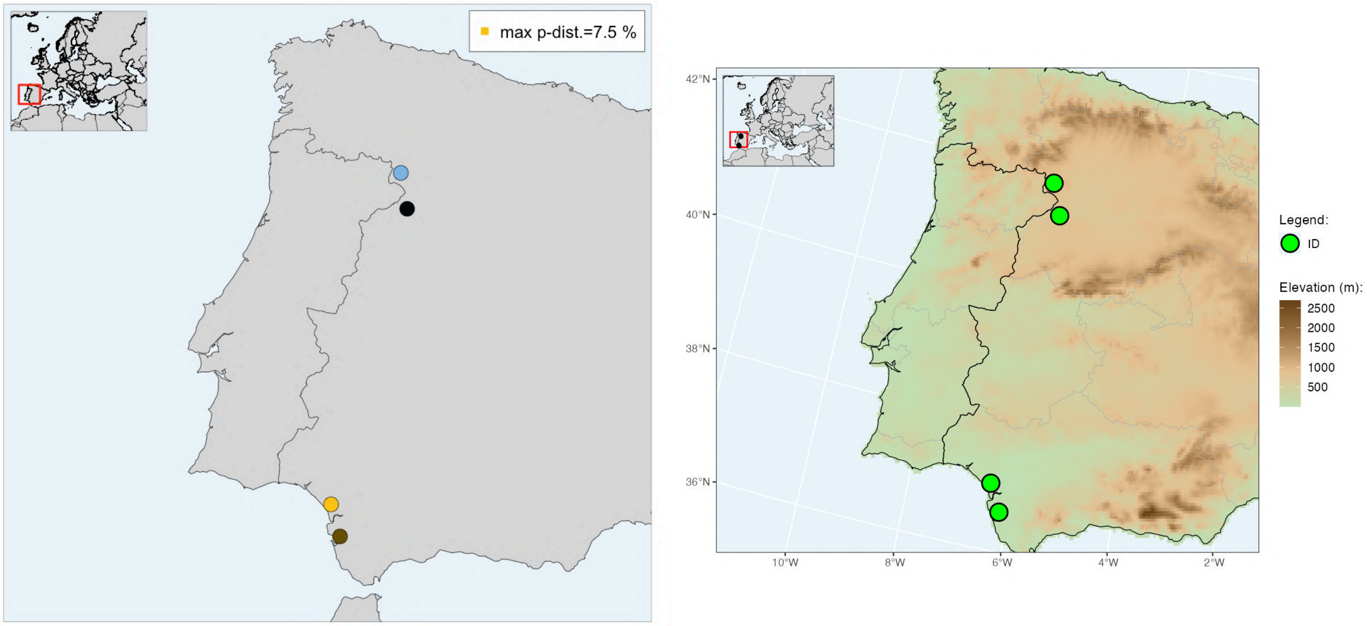

Figure 413: Genetic diversity map of *Messor lusitanicus* Tinaut, 1985. Nearby localities of sequenced specimens are merged in pies (left). Colours match the bidimensional colour space of the PCoA projection (Fig. 413 left) of p-dist between sequences (dots). Specimen identification (ID or cf.) and source (newly sequenced or retrieved) are represented by colours, while specimen attribute (terra typica, type locality, type specimen or faunistic novelty) is represented by the shape (right). Sequences: ID = 4, cf. = 0; maximum p-distance: strict = 7.5 %, less strict = 7.5 %.

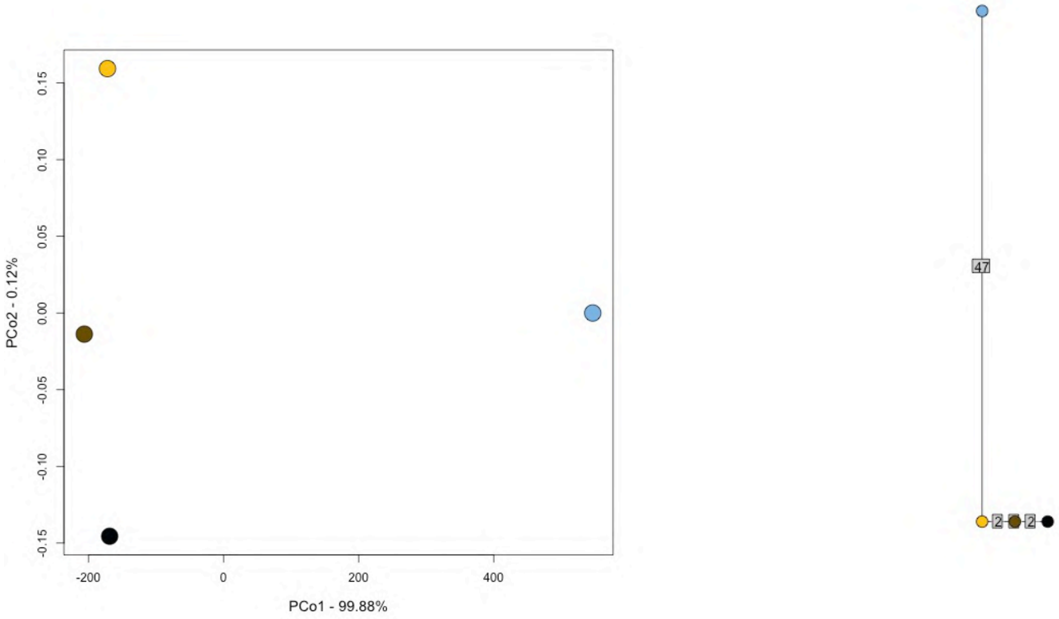

Figure 414: PCoA based on pairwise p-distances between *Messor lusitanicus* sequences (left). Colours match a bidimensional colour space. Haplotype network of *Messor lusitanicus* (right). Sequences > 599 bp: ID = 4, cf. = 0.

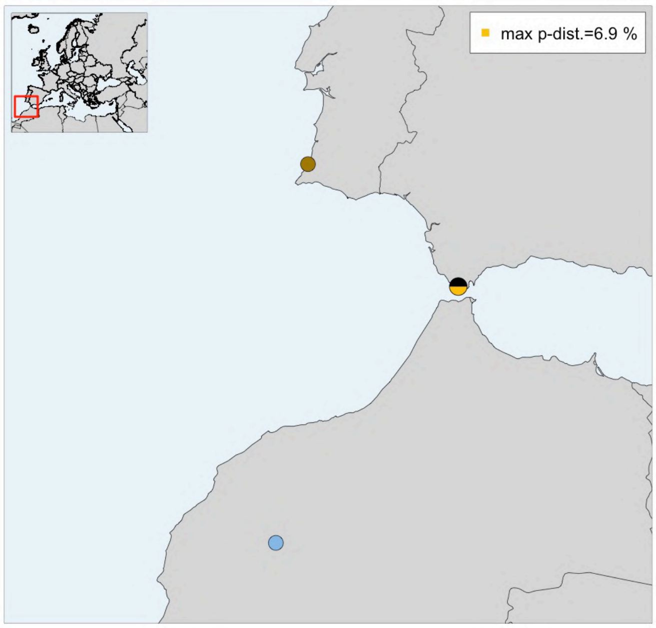

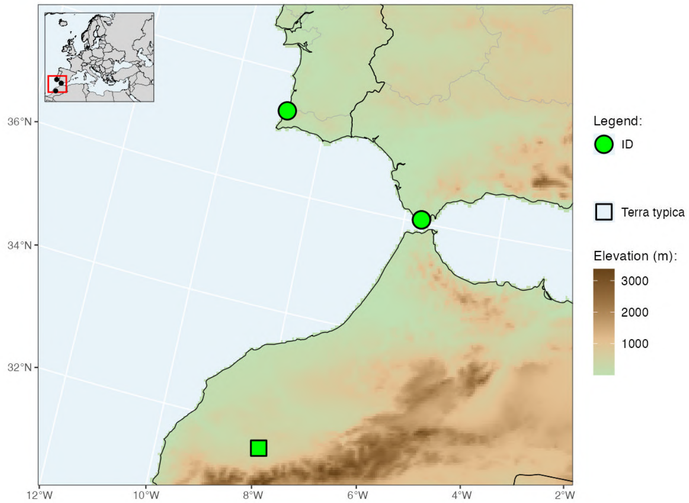

Figure 415: Genetic diversity map of *Messor marocanus* Santschi, 1927. Nearby localities of sequenced specimens are merged in pies (left). Colours match the bidimensional colour space of the PCoA projection (Fig. 415 left) of p-dist between sequences (dots). Specimen identification (ID or cf.) and source (newly sequenced or retrieved) are represented by colours, while specimen attribute (terra typica, type locality, type specimen or faunistic novelty) is represented by the shape (right). Sequences: ID = 4, cf. = 0; maximum p-distance: strict = 6.9 %, less strict = 6.9 %.

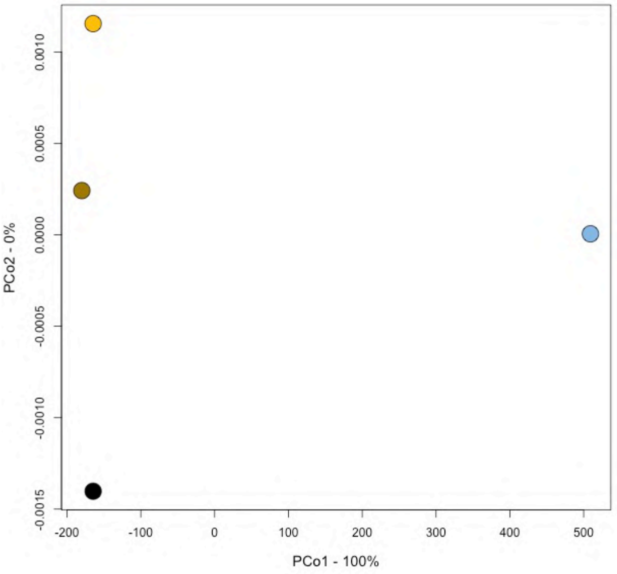

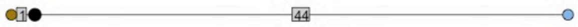

Figure 416: PCoA based on pairwise p-distances between *Messor marocanus* sequences (left). Colours match a bidimensional colour space. Haplotype network of *Messor marocanus* (right). Sequences > 599 bp: ID = 4, cf. = 0.

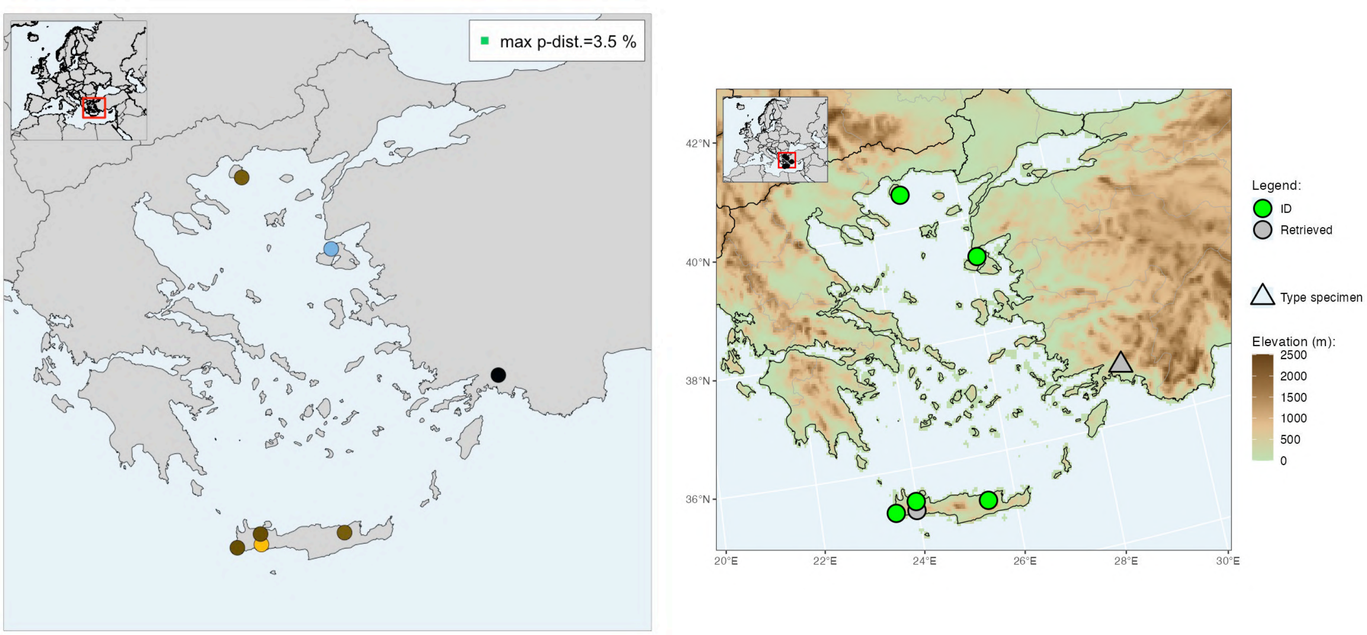

Figure 417: Genetic diversity map of *Messor mcarthuri* Steiner, Csősz, Markó, Gamisch, Rinnhofer, Folterbauer, Hammerle, Stauffer, Arthofer & Schlick-Steiner, 2018. Nearby localities of sequenced specimens are merged in pies (left). Colours match the bidimensional colour space of the PCoA projection (Fig. 417 left) of p-dist between sequences (dots). Specimen identification (ID or cf.) and source (newly sequenced or retrieved) are represented by colours, while specimen attribute (terra typica, type locality, type specimen or faunistic novelty) is represented by the shape (right). Sequences: ID = 7, cf. = 0; maximum p-distance: strict = 3.5 %, less strict = 3.5 %.

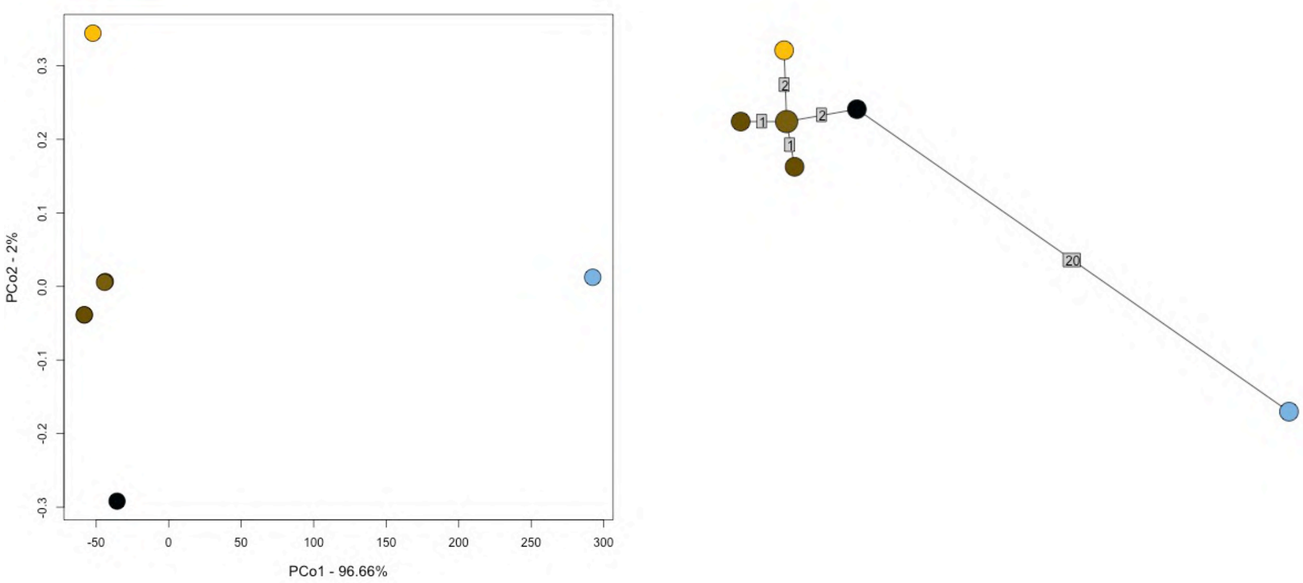

Figure 418: PCoA based on pairwise p-distances between *Messor mcarthuri* sequences (left). Colours match a bidimensional colour space. Haplotype network of *Messor mcarthuri* (right). Sequences > 599 bp: ID = 7, cf. = 0.

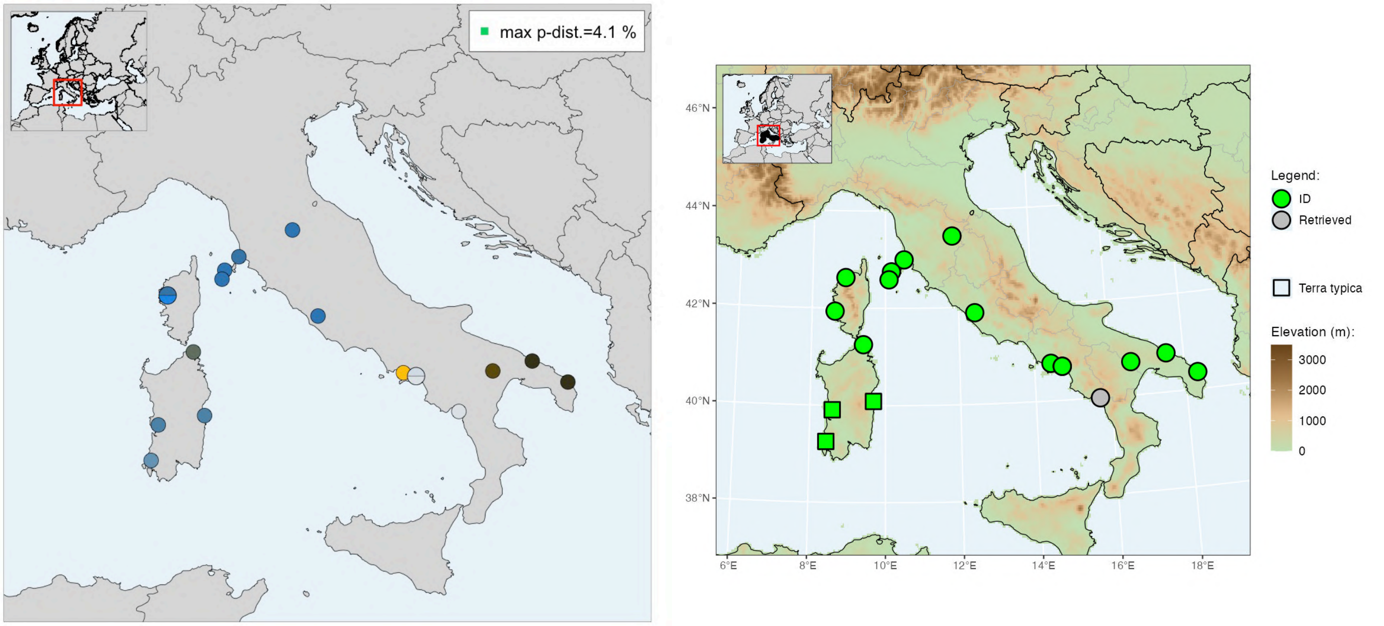

Figure 419: Genetic diversity map of *Messor minor* (André, 1883). Nearby localities of sequenced specimens are merged in pies (left). Colours match the bidimensional colour space of the PCoA projection (Fig. 419 left) of p-dist between sequences (dots). Specimen identification (ID or cf.) and source (newly sequenced or retrieved) are represented by colours, while specimen attribute (terra typica, type locality, type specimen or faunistic novelty) is represented by the shape (right). Sequences: ID = 18, cf. = 0; maximum p-distance: strict = 3.3 %, less strict = 4.1 %.

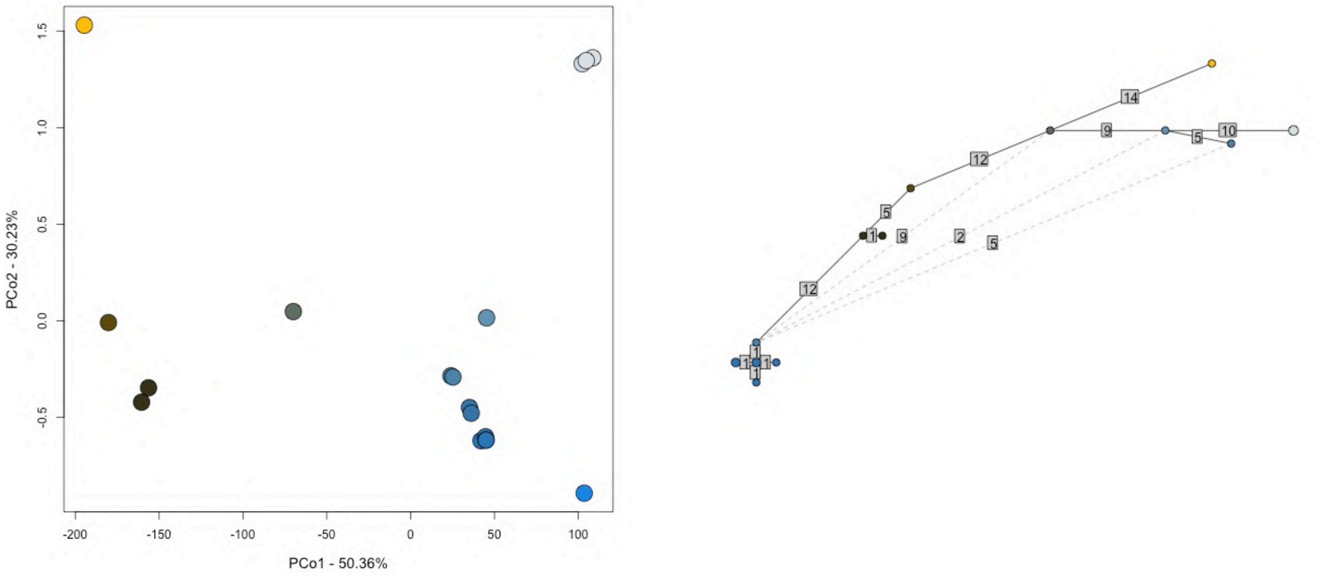

Figure 420: PCoA based on pairwise p-distances between *Messor minor* sequences (left). Colours match a bidimensional colour space. Haplotype network of *Messor minor* (right). Sequences > 599 bp: ID = 17, cf. = 0.

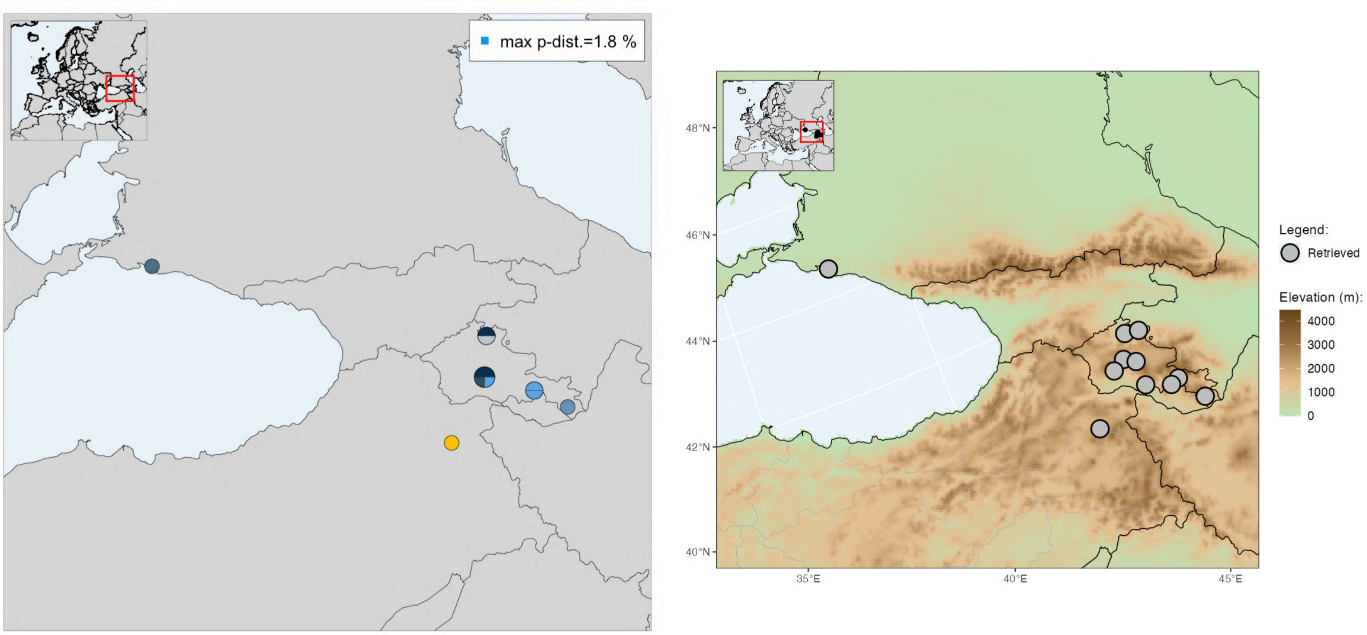

Figure 421: Genetic diversity map of *Messor muticus* (Nylander, 1849). Nearby localities of sequenced specimens are merged in pies (left). Colours match the bidimensional colour space of the PCoA projection (Fig. 421 left) of p-dist between sequences (dots). Specimen identification (ID or cf.) and source (newly sequenced or retrieved) are represented by colours, while specimen attribute (terra typica, type locality, type specimen or faunistic novelty) is represented by the shape (right). Sequences: ID = 11, cf. = 0; maximum p-distance: strict = 1.8 %, less strict = 1.8 %.

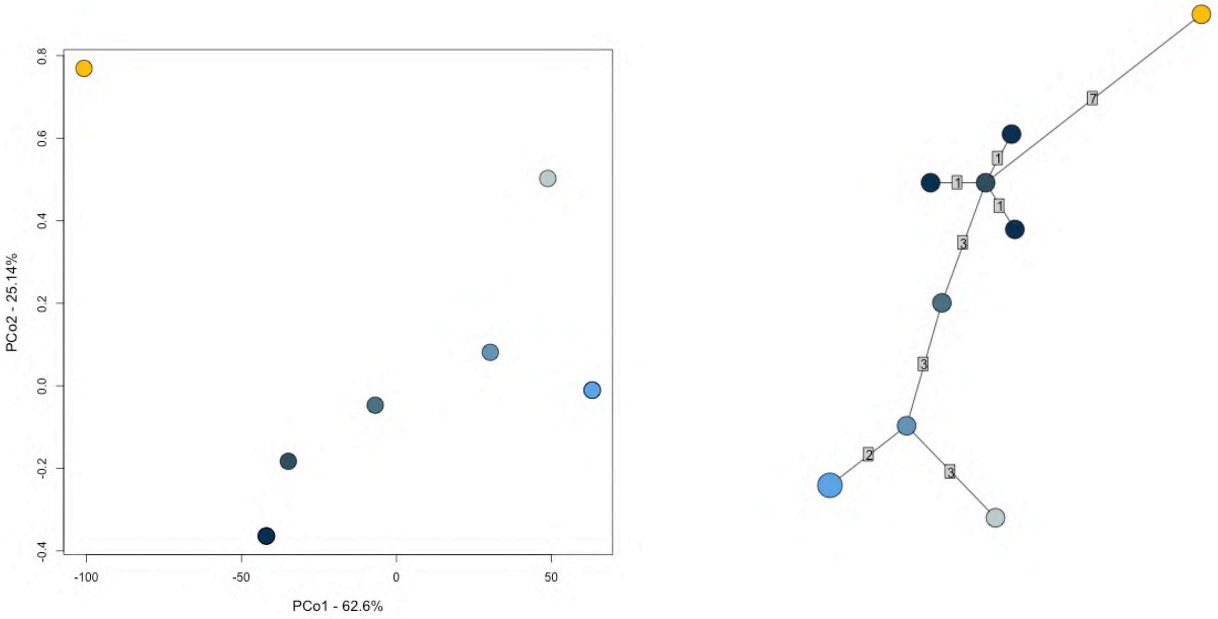

Figure 422: PCoA based on pairwise p-distances between *Messor muticus* sequences (left). Colours match a bidimensional colour space. Haplotype network of *Messor muticus* (right). Sequences > 599 bp: ID = 11, cf. = 0.

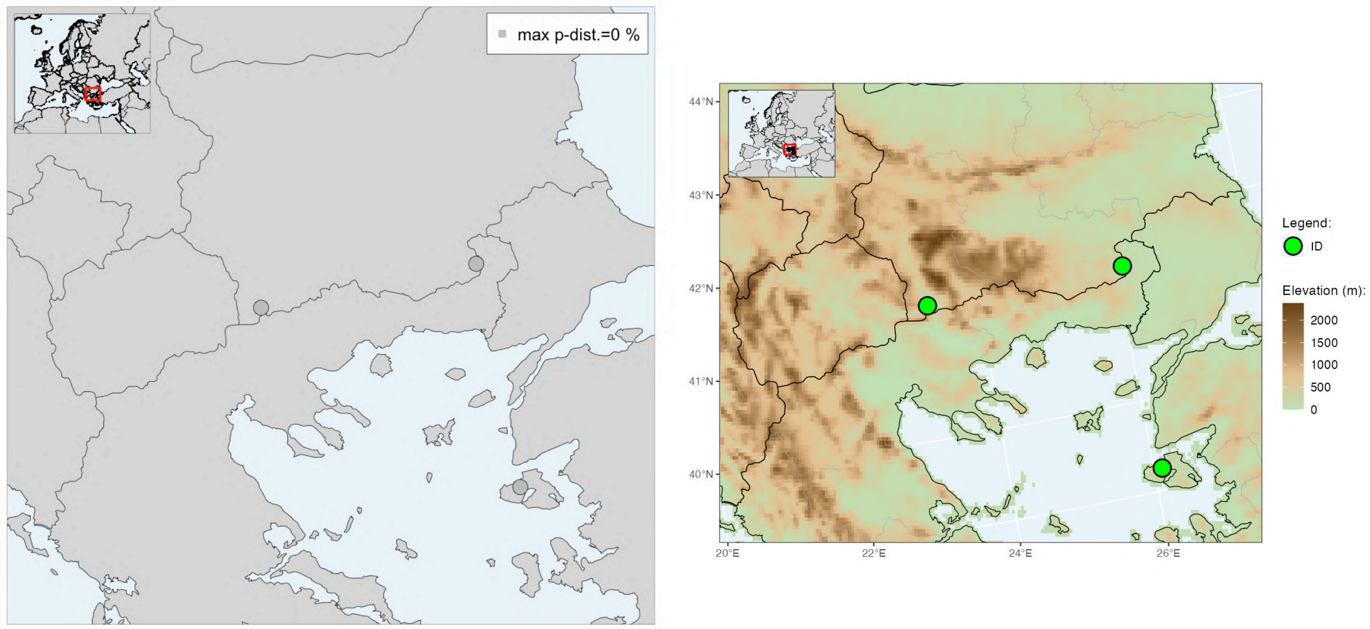

Figure 423: Genetic diversity map of *Messor oertzeni* Forel, 1910. PCoA projection was not done and therefore sequenced specimens in the genetic diversity map are coloured in gray (left). Specimen identification (ID or cf.) and source (newly sequenced or retrieved) are represented by colours, while specimen attribute (terra typica, type locality, type specimen or faunistic novelty) is represented by the shape (right). Sequences: ID = 3, cf. = 0; maximum p-distance: strict = 0 %, less strict = 0 %.

Haplotype network analysis of *Messor oertzeni* was not possible.

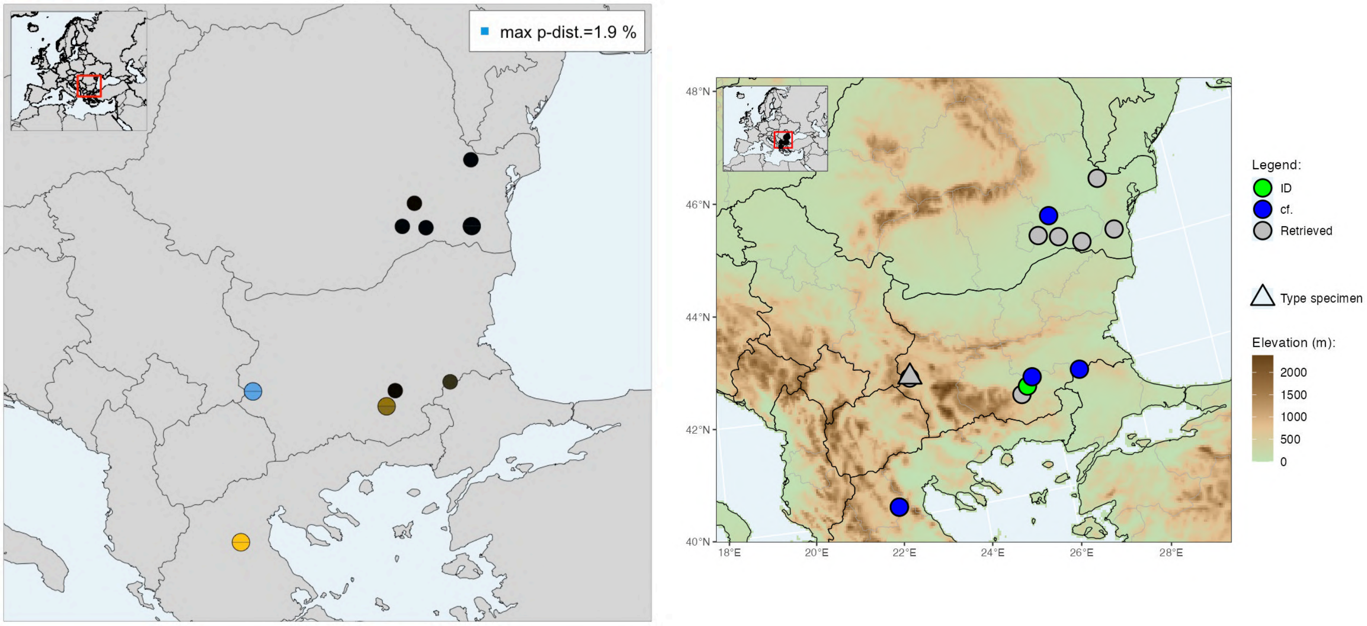

Figure 424: Genetic diversity map of *Messor ponticus* Steiner, Csősz, Markó, Gamisch, Rinnhofer, Folterbauer, Hammerle, Stauffer, Arthofer & Schlick-Steiner, 2018. Nearby localities of sequenced specimens are merged in pies (left). Colours match the bidimensional colour space of the PCoA projection (Fig. 424 left) of p-dist between sequences (dots). Specimen identification (ID or cf.) and source (newly sequenced or retrieved) are represented by colours, while specimen attribute (terra typica, type locality, type specimen or faunistic novelty) is represented by the shape (right). Sequences: ID = 9, cf. = 5; maximum p-distance: strict = 1.9 %, less strict = 1.9 %.

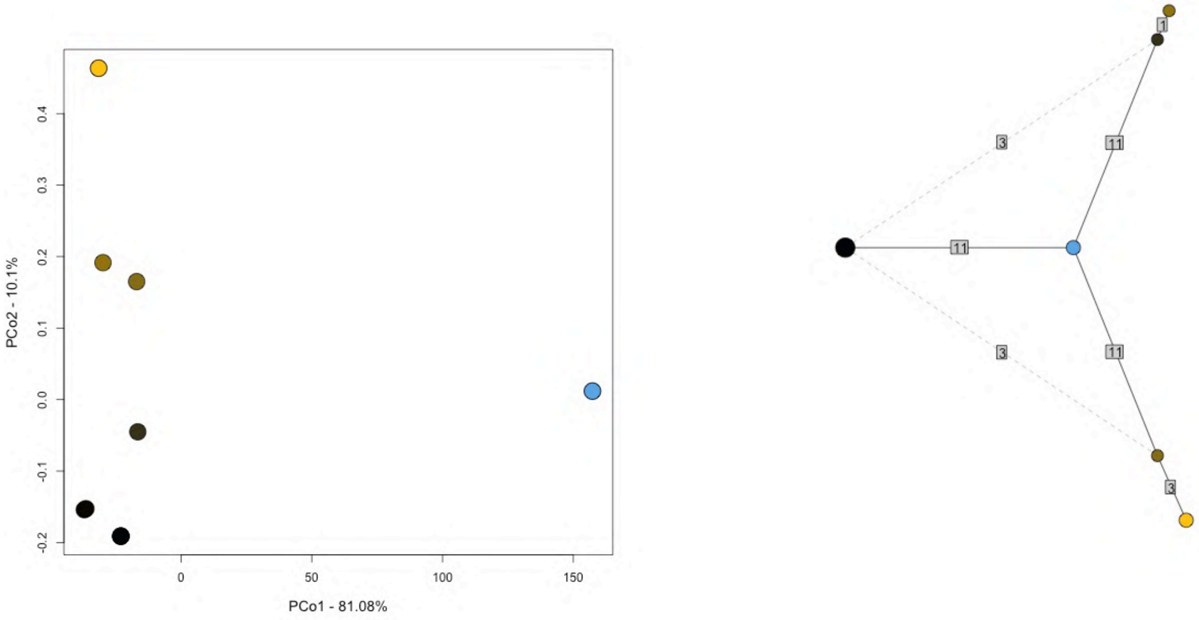

Figure 425: PCoA based on pairwise p-distances between *Messor ponticus* sequences (left). Colours match a bidimensional colour space. Haplotype network of *Messor ponticus* (right). Sequences > 599 bp: ID = 9, cf. = 5.

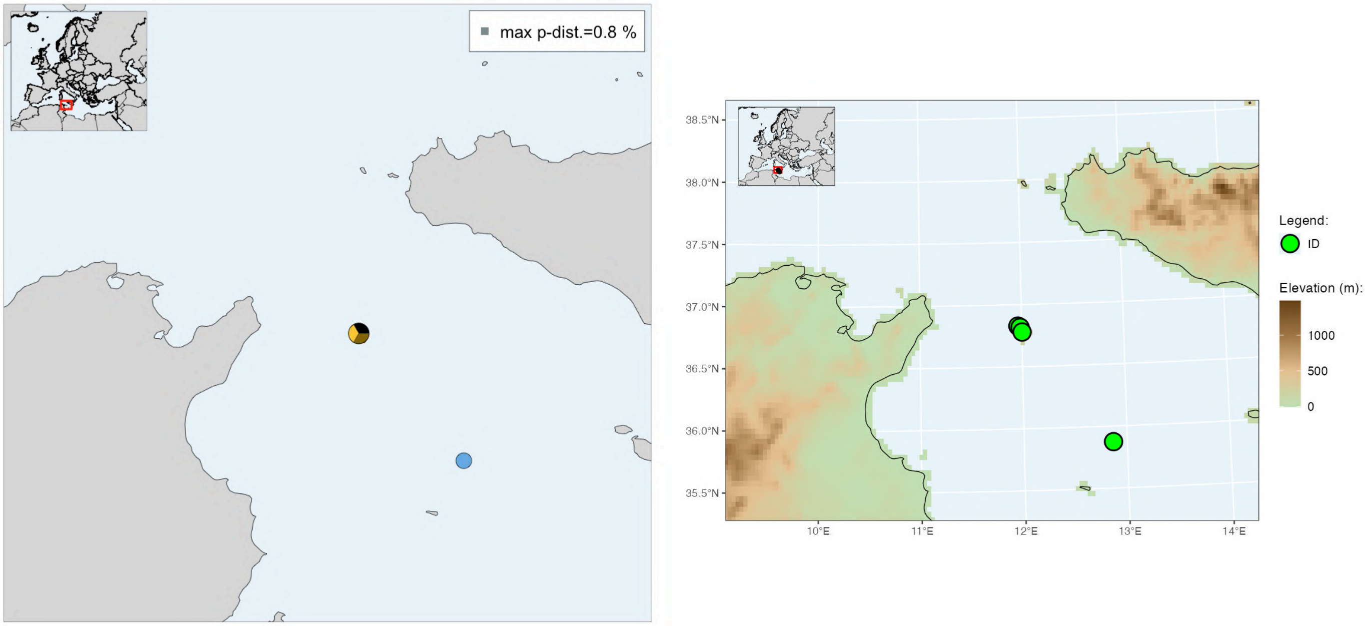

Figure 426: Genetic diversity map of *Messor sanctus* Emery, 1921. Nearby localities of sequenced specimens are merged in pies (left). Colours match the bidimensional colour space of the PCoA projection (Fig. 426 left) of p-dist between sequences (dots). Specimen identification (ID or cf.) and source (newly sequenced or retrieved) are represented by colours, while specimen attribute (terra typica, type locality, type specimen or faunistic novelty) is represented by the shape (right). Sequences: ID = 4, cf. = 0; maximum p-distance: strict = 0.8 %, less strict = 0.8 %.

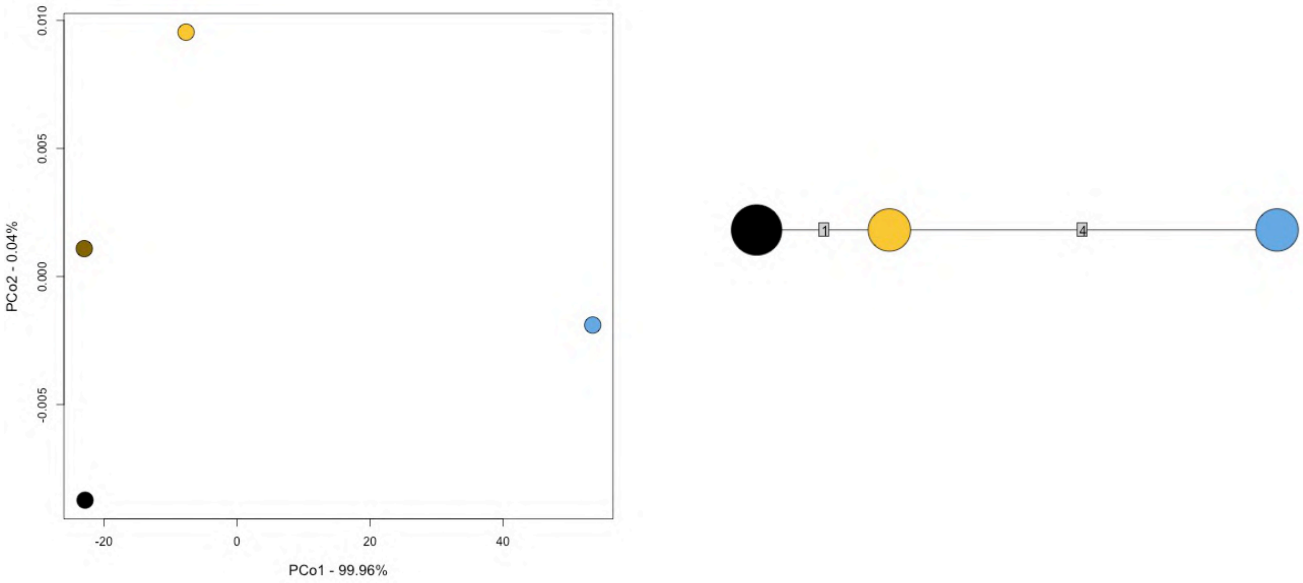

Figure 427: PCoA based on pairwise p-distances between *Messor sanctus* sequences (left). Colours match a bidimensional colour space. Haplotype network of *Messor sanctus* (right). Sequences > 599 bp: ID = 4, cf. = 0.

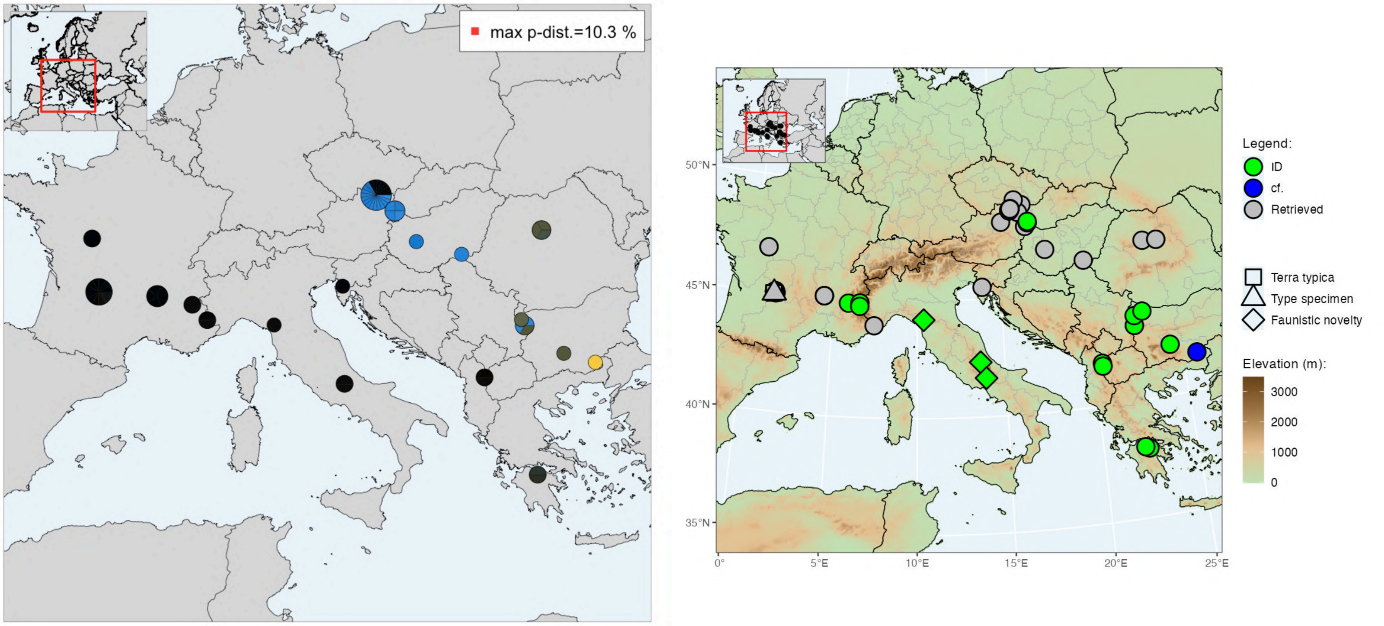

Figure 428: Genetic diversity map of *Messor structor* (Latreille, 1798). Nearby localities of sequenced specimens are merged in pies (left). Colours match the bidimensional colour space of the PCoA projection (Fig. 428 left) of p-dist between sequences (dots). Specimen identification (ID or cf.) and source (newly sequenced or retrieved) are represented by colours, while specimen attribute (terra typica, type locality, type specimen or faunistic novelty) is represented by the shape (right). Sequences: ID = 66, cf. = 1; maximum p-distance: strict = 5.5 %, less strict = 10.3 %.

The species is reported for the first time in Italy.

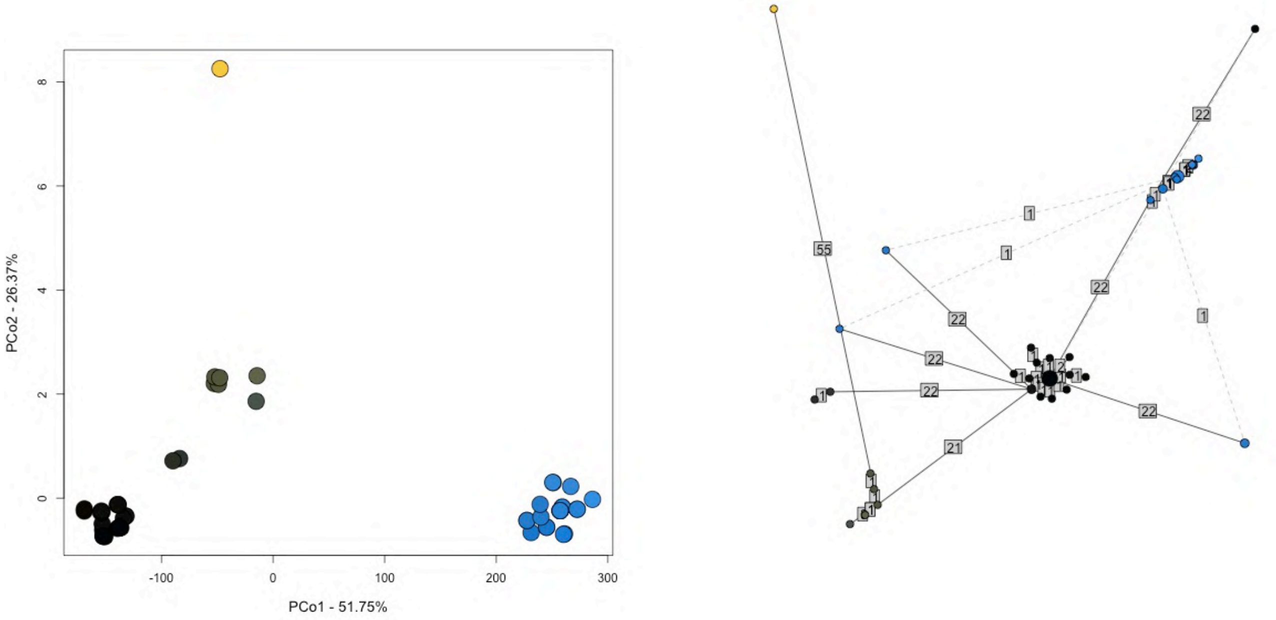

Figure 429: PCoA based on pairwise p-distances between *Messor structor* sequences (left). Colours match a bidimensional colour space. Haplotype network of *Messor structor* (right). Sequences > 599 bp: ID = 66, cf. = 1.

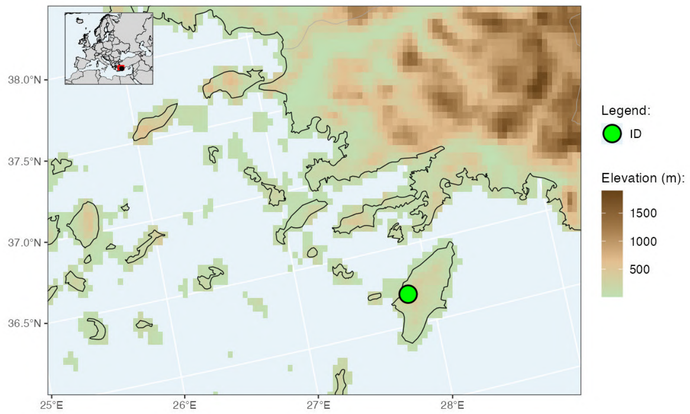

Figure 430: Map of *Messor varrialei* Emery, 1921. Due to the presence of a single sequence, the genetic diversity map and the PCoA projection were not done. Specimen identification (ID or cf.) and source (newly sequenced or retrieved) are represented by colours, while specimen attribute (terra typica, type locality, type specimen or faunistic novelty) is represented by the shape. Sequences: ID = 1, cf. = 0; maximum p-distance: strict = NA, less strict = NA.

Haplotype network analysis of *Messor varrialei* was not possible.

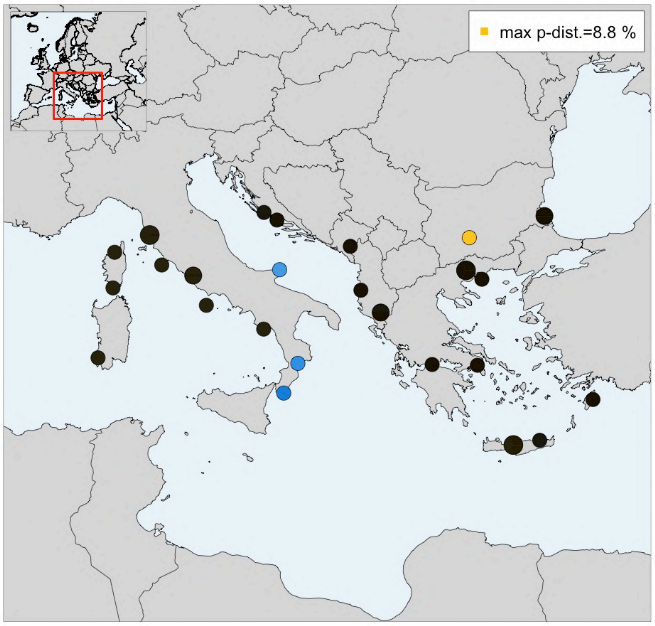

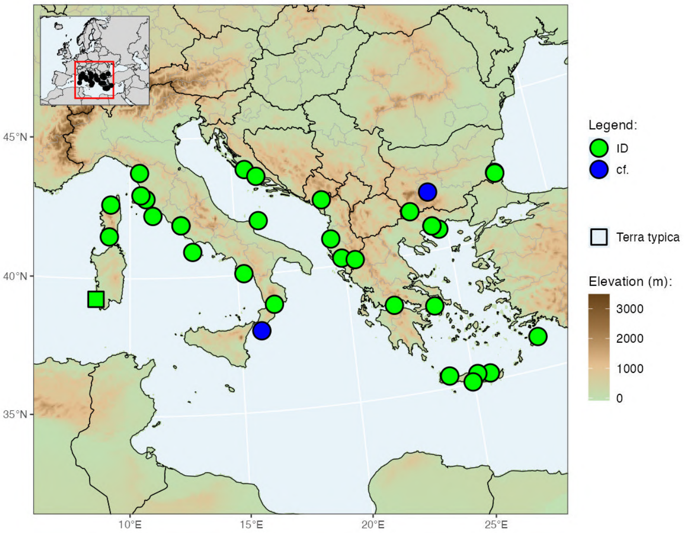

Figure 431: Genetic diversity map of *Messor wasmanni* Krausse, 1910. Nearby localities of sequenced specimens are merged in pies (left). Colours match the bidimensional colour space of the PCoA projection (Fig. 431 left) of p-dist between sequences (dots). Specimen identification (ID or cf.) and source (newly sequenced or retrieved) are represented by colours, while specimen attribute (terra typica, type locality, type specimen or faunistic novelty) is represented by the shape (right). Sequences: ID = 32, cf. = 2; maximum p-distance: strict = 8.1 %, less strict = 8.8 %.

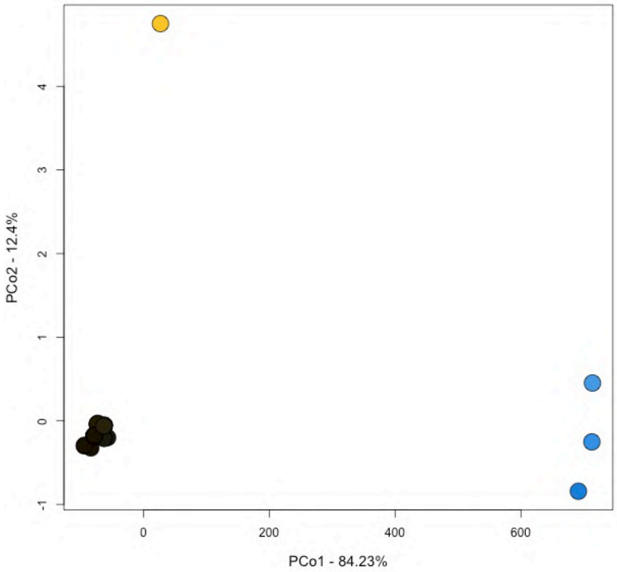

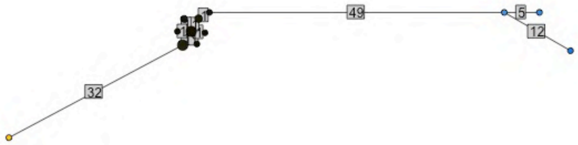

Figure 432: PCoA based on pairwise p-distances between *Messor wasmanni* sequences (left). Colours match a bidimensional colour space. Haplotype network of *Messor wasmanni* (right). Sequences > 599 bp: ID = 32, cf. = 2.

Metalasius

Metalasius myrmidon

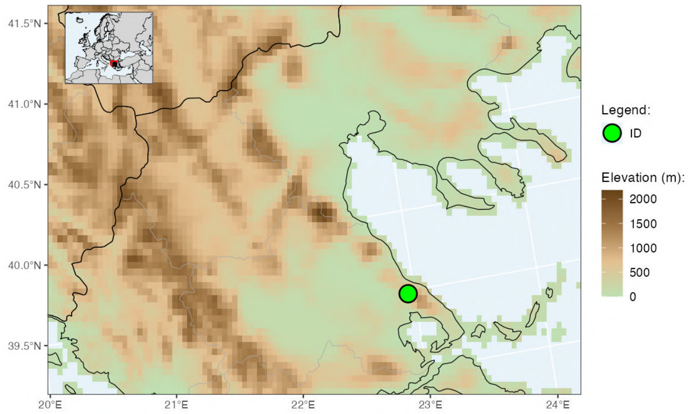

Figure 433: Map of *Metalasius myrmidon* (Mei, 1998). Due to the presence of a single sequence, the genetic diversity map and the PCoA projection were not done. Specimen identification (ID or cf.) and source (newly sequenced or retrieved) are represented by colours, while specimen attribute (terra typica, type locality, type specimen or faunistic novelty) is represented by the shape. Sequences: ID = 1, cf. = 0; maximum p-distance: strict = NA, less strict = NA.

Haplotype network analysis of *Metalasius myrmidon* was not possible.

Monomorium

Monomorium algiricum

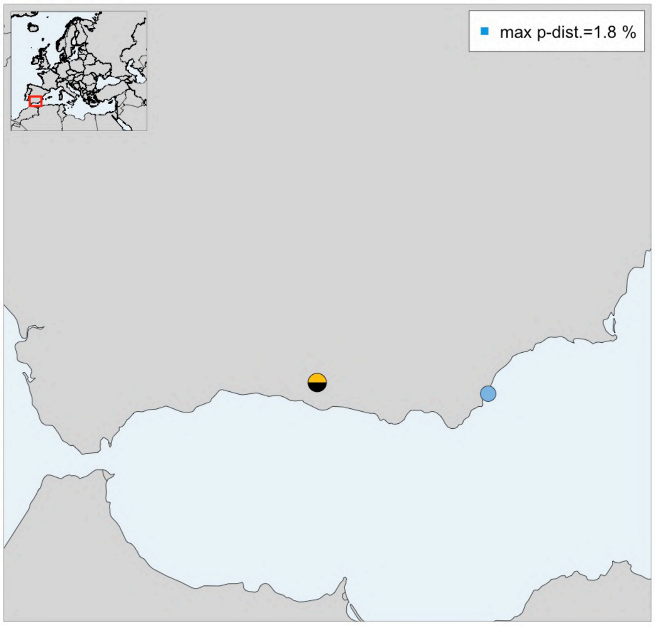

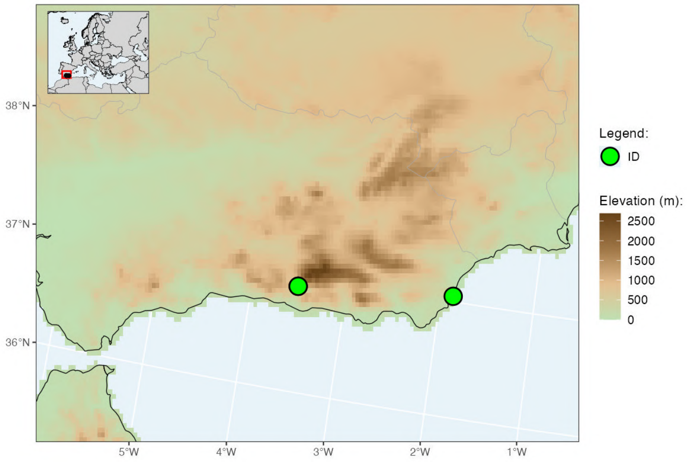

Figure 434: Genetic diversity map of *Monomorium algiricum* (Bernard, 1955). Nearby localities of sequenced specimens are merged in pies (left). Colours match the bidimensional colour space of the PCoA projection (Fig. 434 left) of p-dist between sequences (dots). Specimen identification (ID or cf.) and source (newly sequenced or retrieved) are represented by colours, while specimen attribute (terra typica, type locality, type specimen or faunistic novelty) is represented by the shape (right). Sequences: ID = 3, cf. = 0; maximum p-distance: strict = 1.8 %, less strict = 1.8 %.

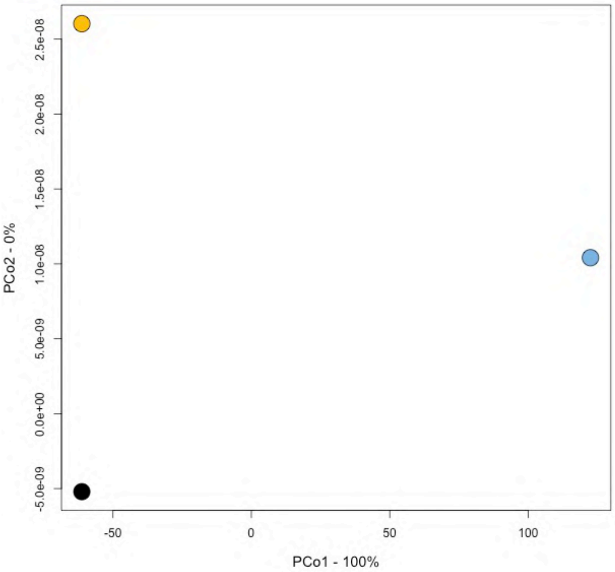

Figure 435: PCoA based on pairwise p-distances between *Monomorium algiricum* sequences (left). Colours match a bidimensional colour space. Haplotype network analysis of *Monomorium algiricum* was not possible.

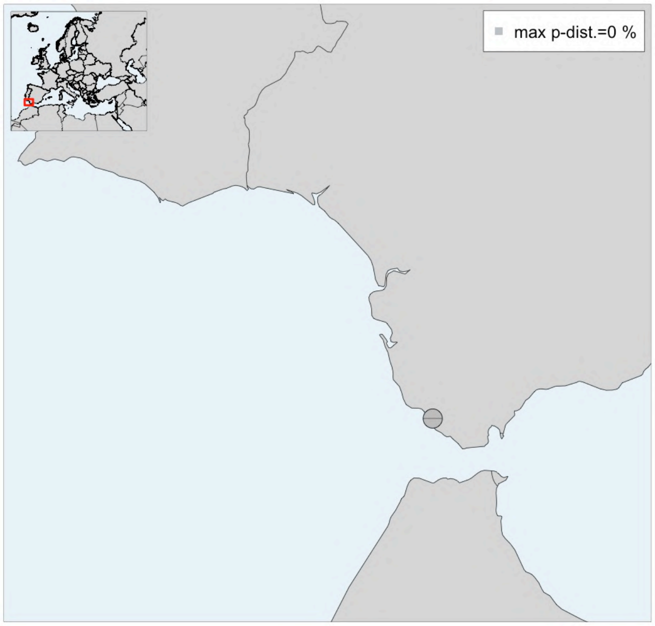

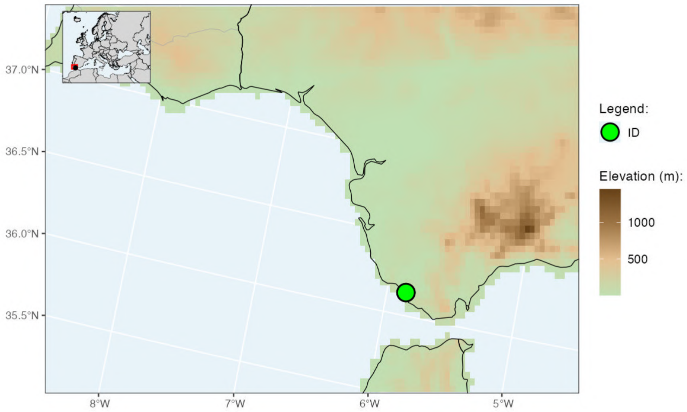

Figure 436: Genetic diversity map of *Monomorium andrei* Saunders, E., 1890. PCoA projection was not done and therefore sequenced specimens in the genetic diversity map are coloured in gray (left). Specimen identification (ID or cf.) and source (newly sequenced or retrieved) are represented by colours, while specimen attribute (terra typica, type locality, type specimen or faunistic novelty) is represented by the shape (right). Sequences: ID = 2, cf. = 0; maximum p-distance: strict = NA, less strict = 0 %.

Haplotype network analysis of *Monomorium andrei* was not possible.

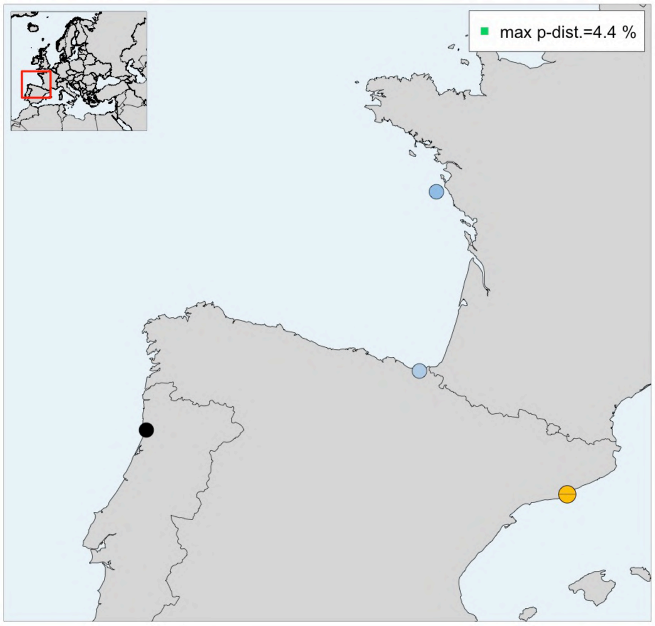

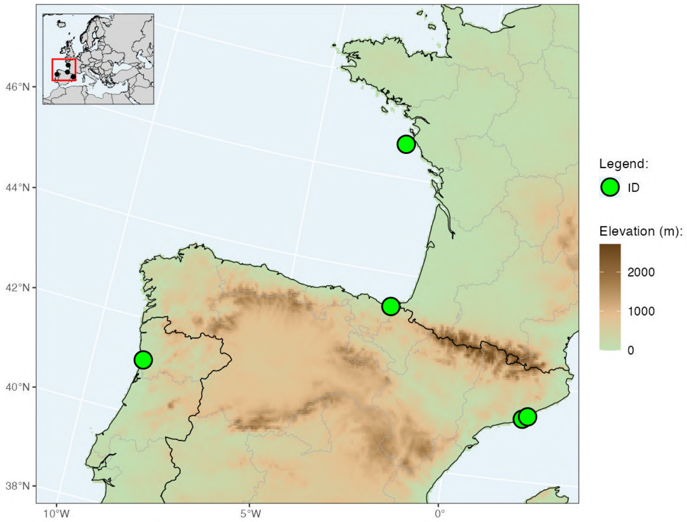

Figure 437: Genetic diversity map of *Monomorium carbonarium* (Smith, F., 1858). Nearby localities of sequenced specimens are merged in pies (left). Colours match the bidimensional colour space of the PCoA projection (Fig. 437 left) of p-dist between sequences (dots). Specimen identification (ID or cf.) and source (newly sequenced or retrieved) are represented by colours, while specimen attribute (terra typica, type locality, type specimen or faunistic novelty) is represented by the shape (right). Sequences: ID = 7, cf. = 0; maximum p-distance: strict = 4.4 %, less strict = 4.4 %.

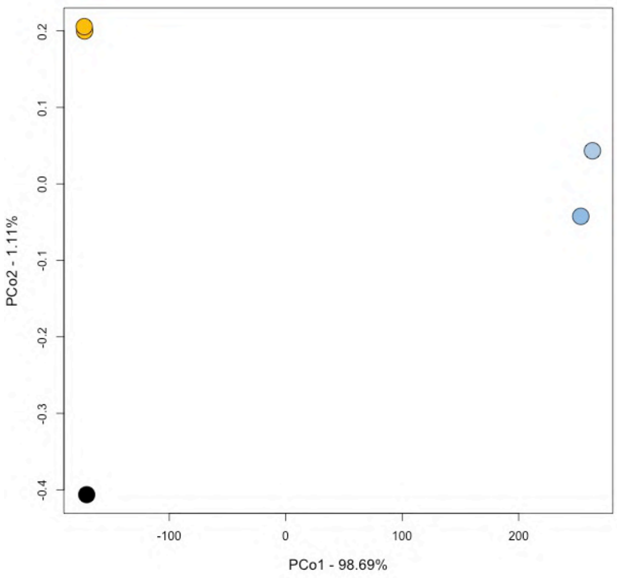

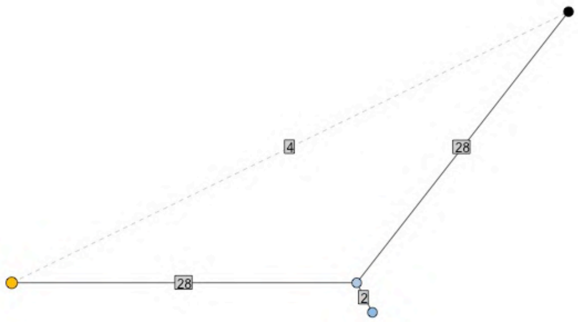

Figure 438: PCoA based on pairwise p-distances between *Monomorium carbonarium* sequences (left). Colours match a bidimensional colour space. Haplotype network of *Monomorium carbonarium* (right). Sequences > 599 bp: ID = 6, cf. = 0.

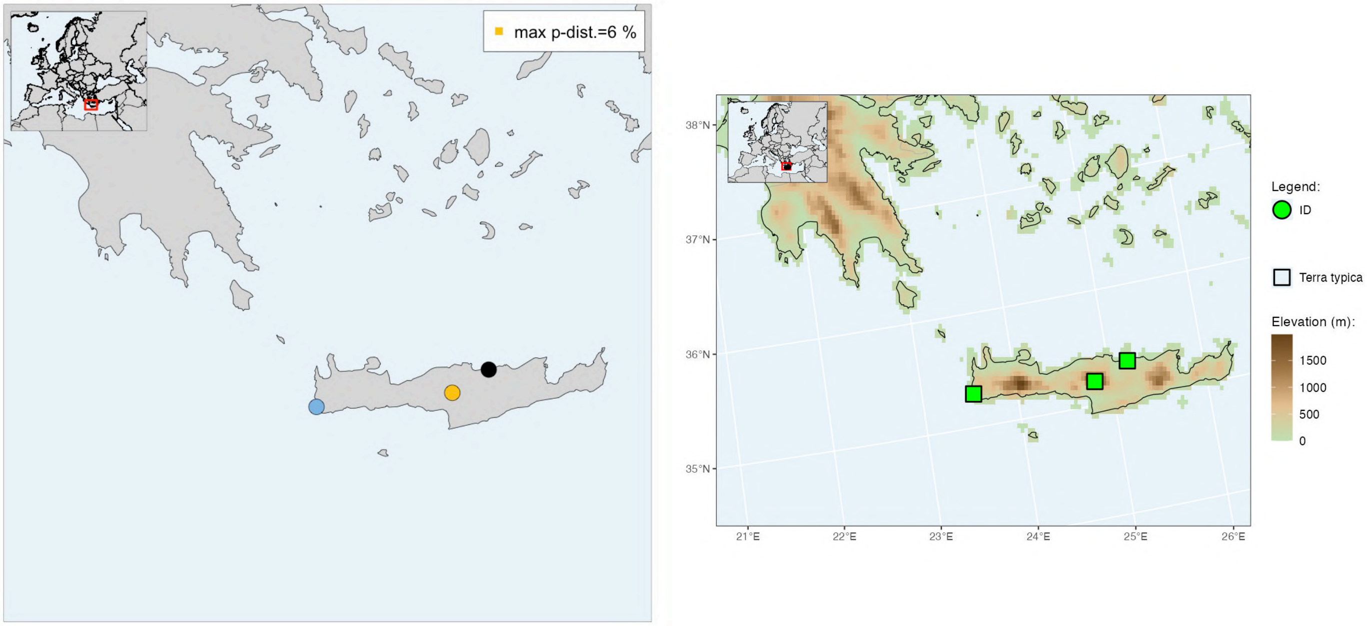

Figure 439: Genetic diversity map of *Monomorium creticum* Emery, 1895. Nearby localities of sequenced specimens are merged in pies (left). Colours match the bidimensional colour space of the PCoA projection (Fig. 439 left) of p-dist between sequences (dots). Specimen identification (ID or cf.) and source (newly sequenced or retrieved) are represented by colours, while specimen attribute (terra typica, type locality, type specimen or faunistic novelty) is represented by the shape (right). Sequences: ID = 3, cf. = 0; maximum p-distance: strict = 6 %, less strict = 6 %.

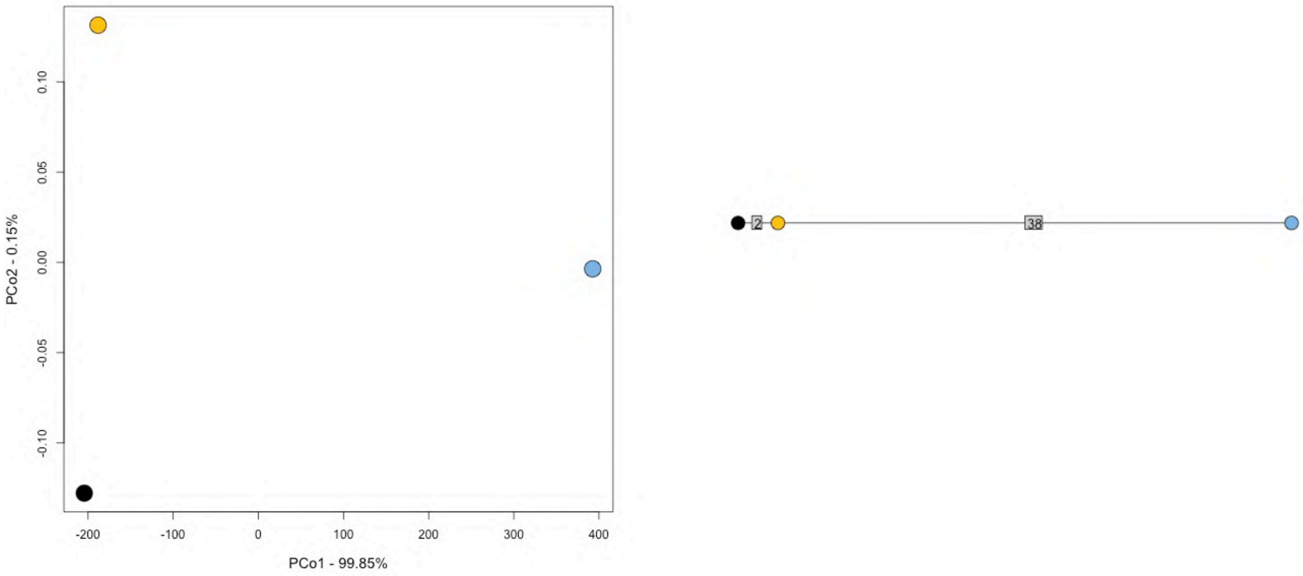

Figure 440: PCoA based on pairwise p-distances between *Monomorium creticum* sequences (left). Colours match a bidimensional colour space. Haplotype network of *Monomorium creticum* (right). Sequences > 599 bp: ID = 3, cf. = 0.

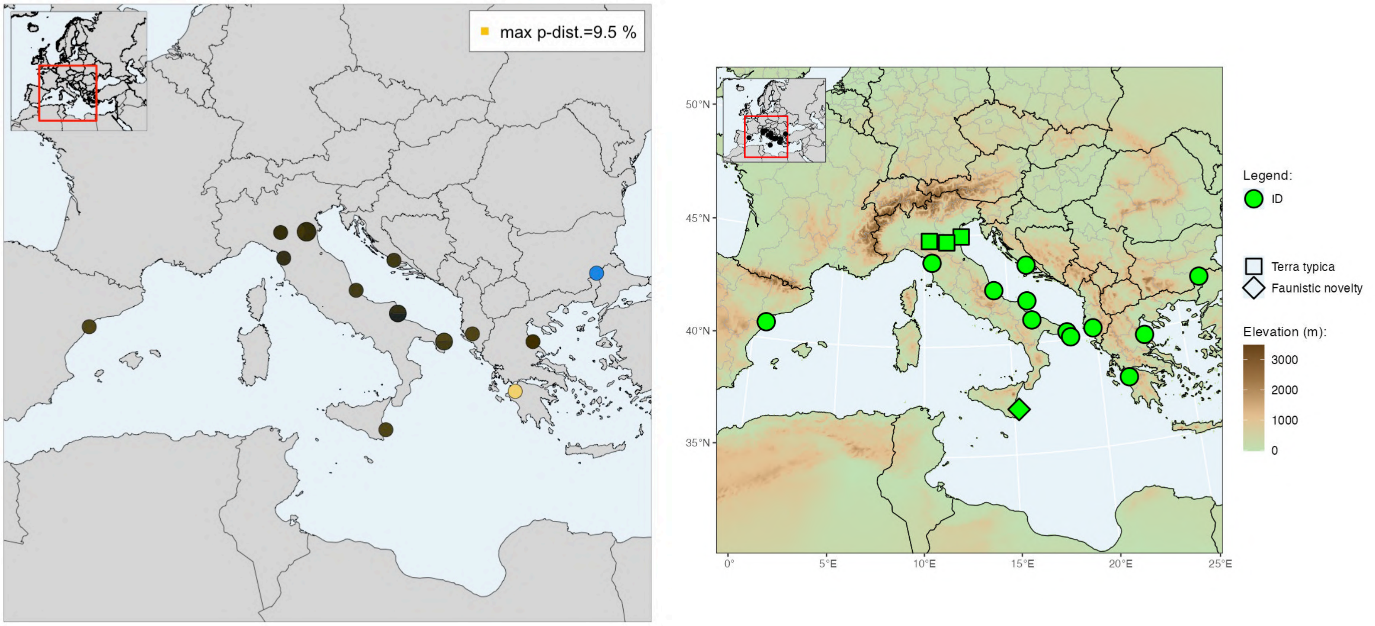

Figure 441: Genetic diversity map of *Monomorium monomorium* Bolton, 1987. Nearby localities of sequenced specimens are merged in pies (left). Colours match the bidimensional colour space of the PCoA projection (Fig. 441 left) of p-dist between sequences (dots). Specimen identification (ID or cf.) and source (newly sequenced or retrieved) are represented by colours, while specimen attribute (terra typica, type locality, type specimen or faunistic novelty) is represented by the shape (right). Sequences: ID = 17, cf. = 0; maximum p-distance: strict = 9.5 %, less strict = 9.5 %.

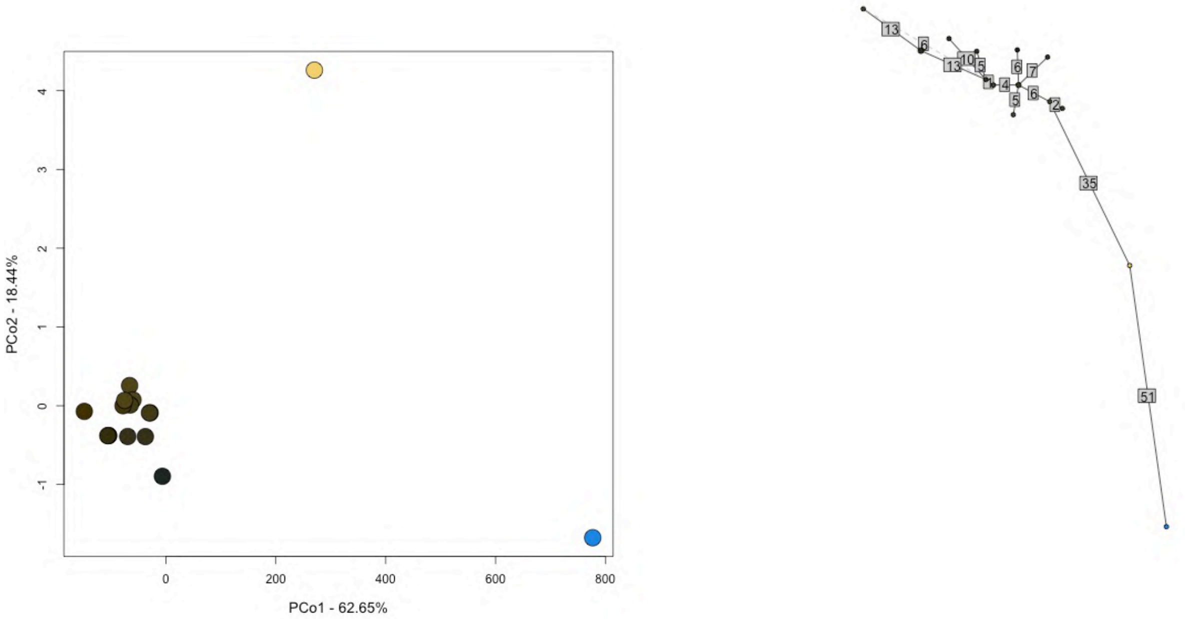

Figure 442: PCoA based on pairwise p-distances between *Monomorium monomorium* sequences (left). Colours match a bidimensional colour space. Haplotype network of *Monomorium monomorium* (right). Sequences > 599 bp: ID = 17, cf. = 0.

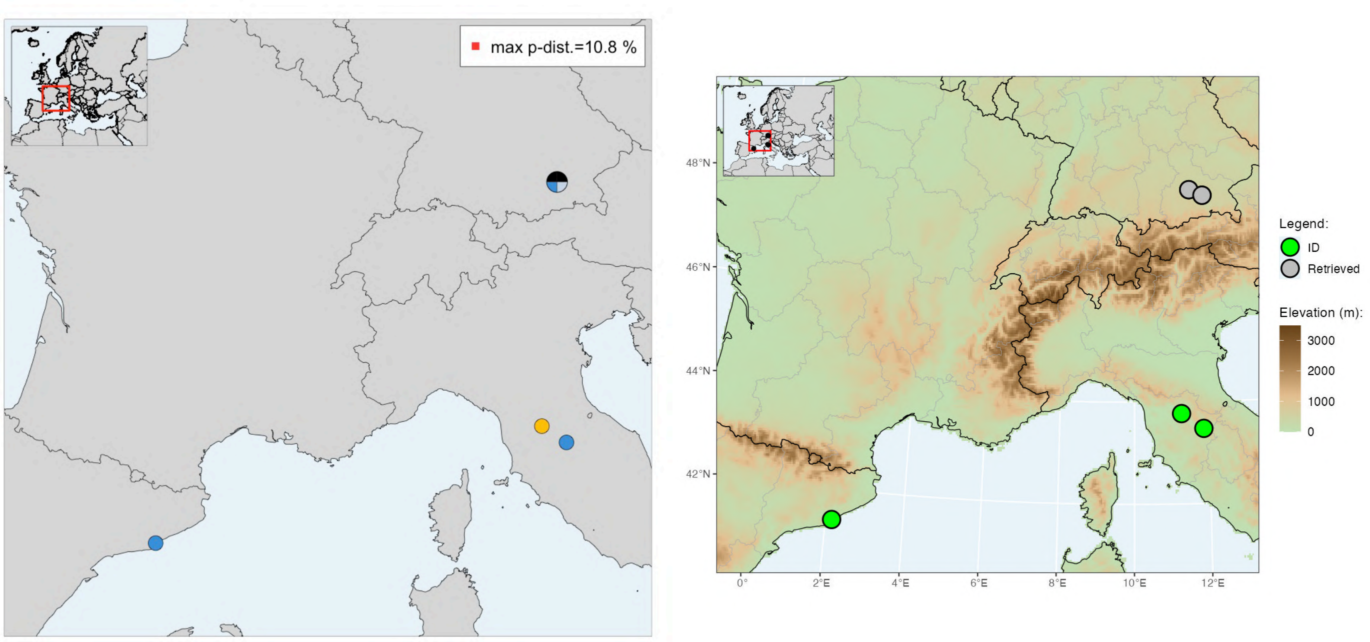

Figure 443: Genetic diversity map of *Monomorium pharaonis* (Linnaeus, 1758). Nearby localities of sequenced specimens are merged in pies (left). Colours match the bidimensional colour space of the PCoA projection (Fig. 443 left) of p-dist between sequences (dots). Specimen identification (ID or cf.) and source (newly sequenced or retrieved) are represented by colours, while specimen attribute (terra typica, type locality, type specimen or faunistic novelty) is represented by the shape (right). Sequences: ID = 8, cf. = 0; maximum p-distance: strict = 10.2 %, less strict = 10.8 %.

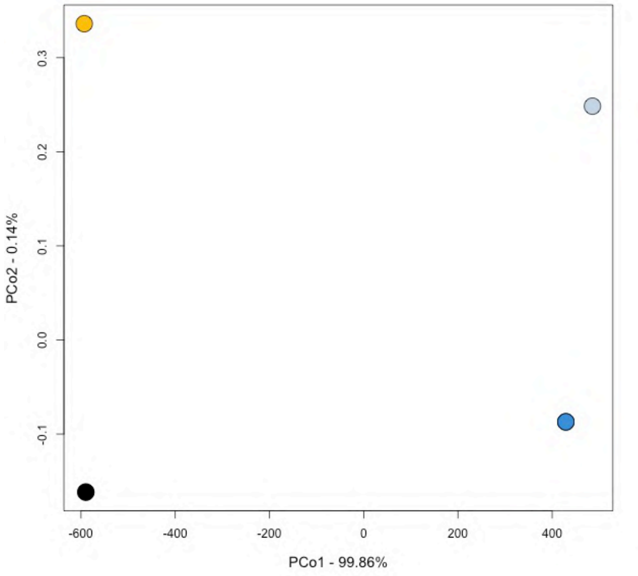

Figure 444: PCoA based on pairwise p-distances between *Monomorium pharaonis* sequences (left). Colours match a bidimensional colour space. Haplotype network analysis of *Monomorium pharaonis* was not possible.

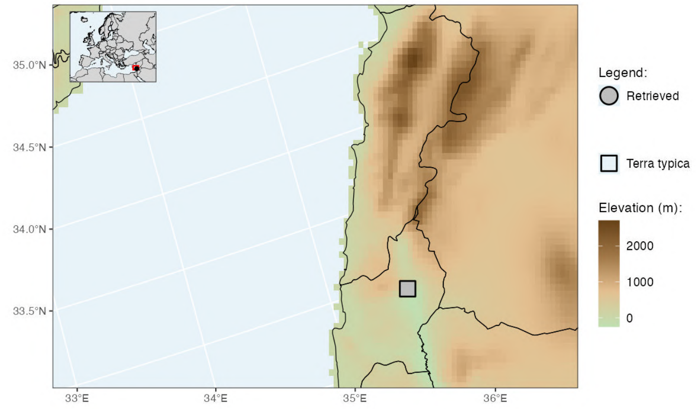

Figure 445: Map of *Monomorium sahlbergi* Emery, 1898. Due to the presence of a single sequence, the genetic diversity map and the PCoA projection were not done. Specimen identification (ID or cf.) and source (newly sequenced or retrieved) are represented by colours, while specimen attribute (terra typica, type locality, type specimen or faunistic novelty) is represented by the shape. Sequences: ID = 1, cf. = 0; maximum p-distance: strict = NA, less strict = NA.

Haplotype network analysis of *Monomorium sahlbergi* was not possible.

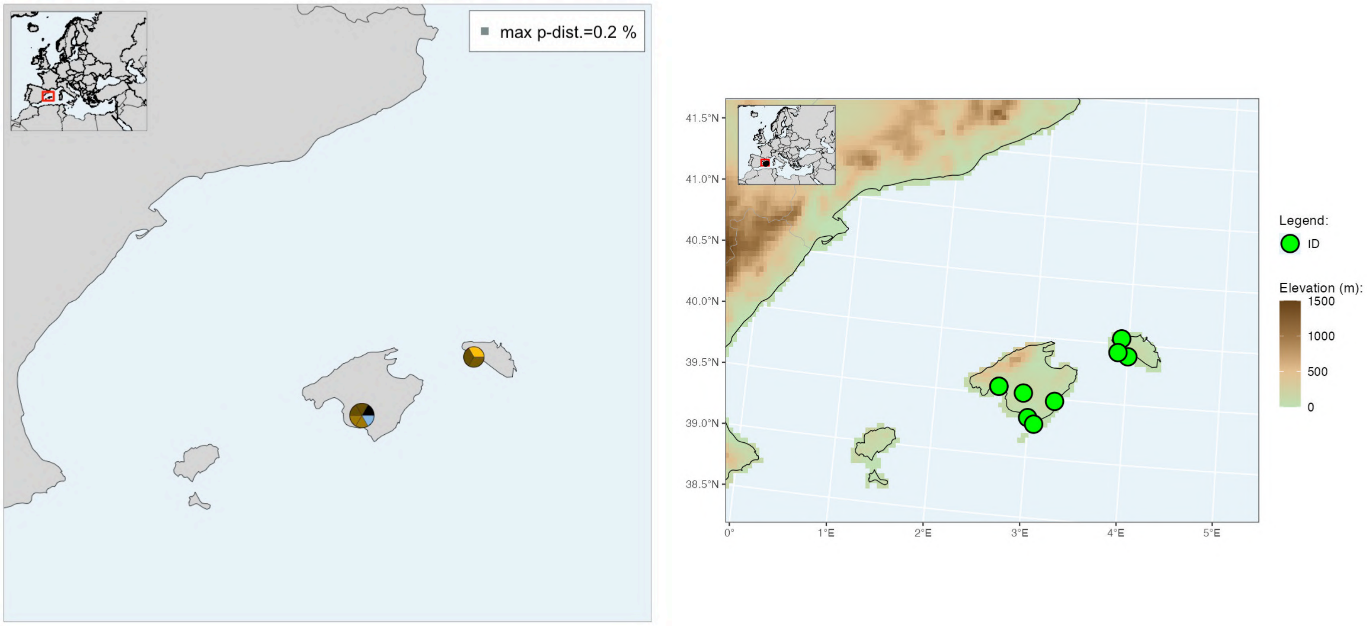

Figure 446: Genetic diversity map of *Monomorium salomonis* (Linnaeus, 1758). Nearby localities of sequenced specimens are merged in pies (left). Colours match the bidimensional colour space of the PCoA projection (Fig. 446 left) of p-dist between sequences (dots). Specimen identification (ID or cf.) and source (newly sequenced or retrieved) are represented by colours, while specimen attribute (terra typica, type locality, type specimen or faunistic novelty) is represented by the shape (right). Sequences: ID = 9, cf. = 0; maximum p-distance: strict = 0.2 %, less strict = 0.2 %.

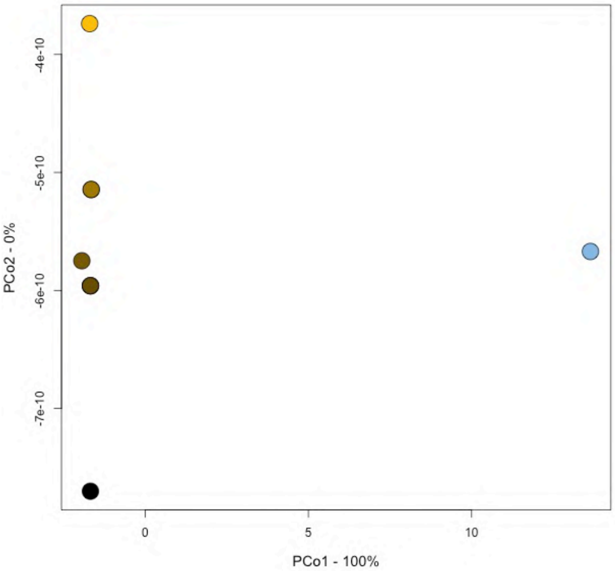

Figure 447: PCoA based on pairwise p-distances between *Monomorium salomonis* sequences (left). Colours match a bidimensional colour space. Haplotype network analysis of *Monomorium salomonis* was not possible.

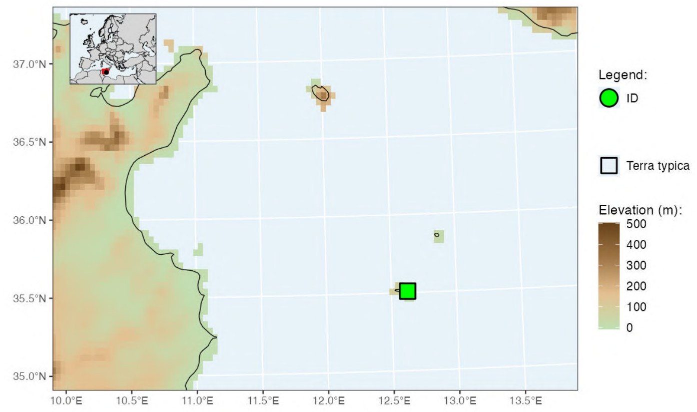

Figure 448: Map of *Monomorium sommieri* Emery, 1908. Due to the presence of a single sequence, the genetic diversity map and the PCoA projection were not done. Specimen identification (ID or cf.) and source (newly sequenced or retrieved) are represented by colours, while specimen attribute (terra typica, type locality, type specimen or faunistic novelty) is represented by the shape. Sequences: ID = 1, cf. = 0; maximum p-distance: strict = NA, less strict = NA.

Haplotype network analysis of *Monomorium sommieri* was not possible.

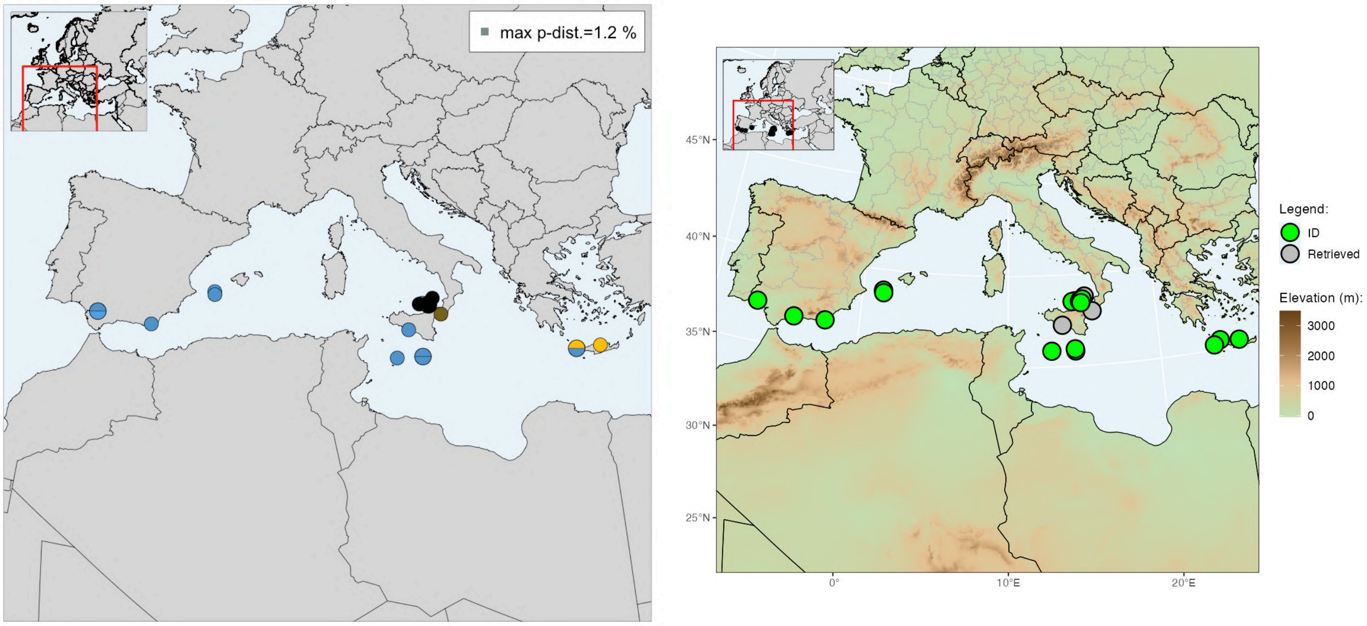

Figure 449: Genetic diversity map of *Monomorium subopacum* (Smith, F., 1858). Nearby localities of sequenced specimens are merged in pies (left). Colours match the bidimensional colour space of the PCoA projection (Fig. 449 left) of p-dist between sequences (dots). Specimen identification (ID or cf.) and source (newly sequenced or retrieved) are represented by colours, while specimen attribute (terra typica, type locality, type specimen or faunistic novelty) is represented by the shape (right). Sequences: ID = 20, cf. = 0; maximum p-distance: strict = 1.2 %, less strict = 1.2 %.

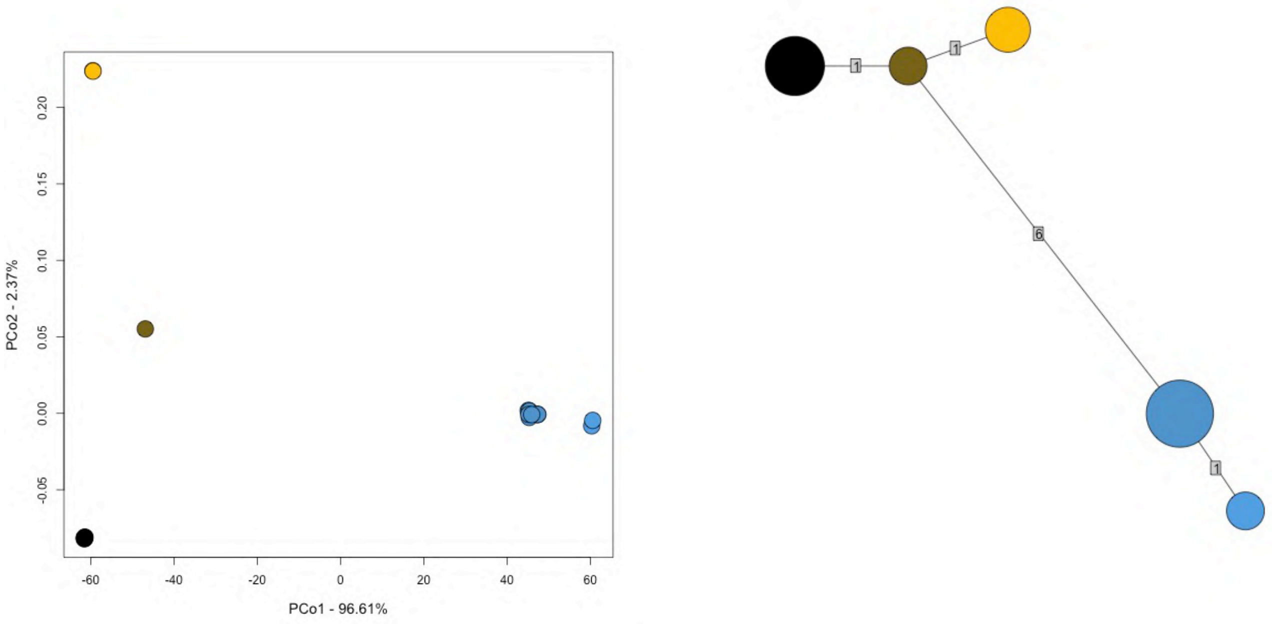

Figure 450: PCoA based on pairwise p-distances between *Monomorium subopacum* sequences (left). Colours match a bidimensional colour space. Haplotype network of *Monomorium subopacum* (right). Sequences > 599 bp: ID = 20, cf. = 0.

Myrmecina

Myrmecina graminicola

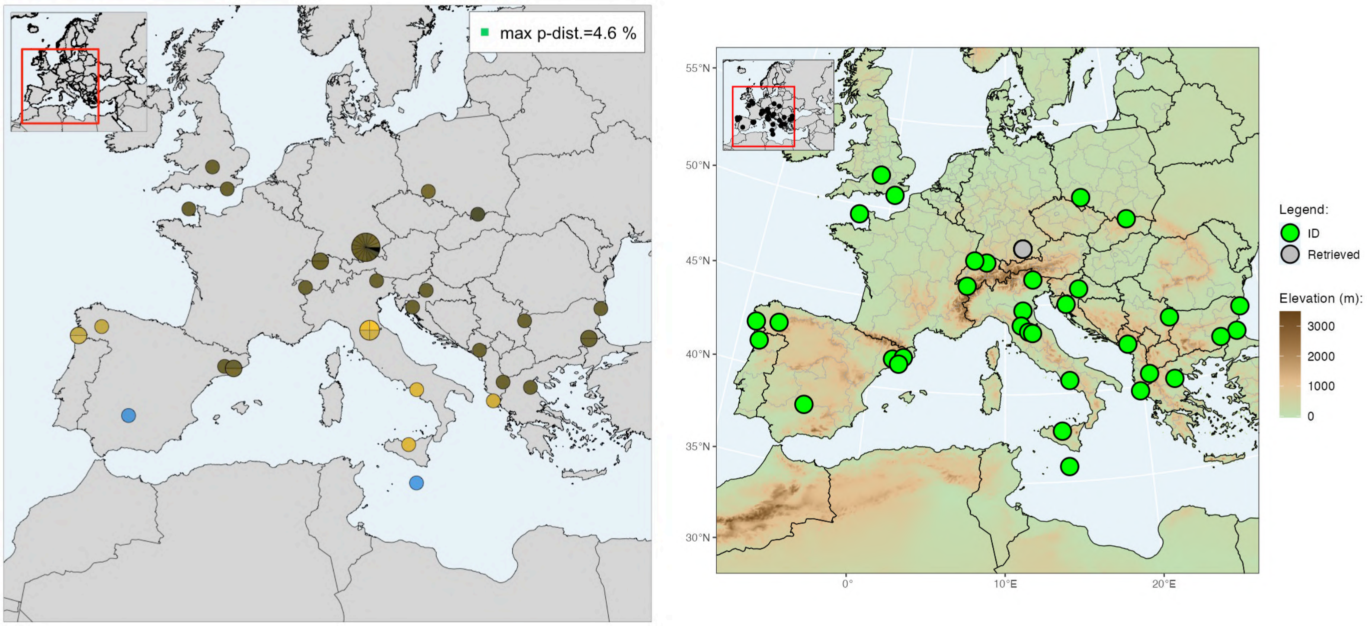

Figure 451: Genetic diversity map of *Myrmecina graminicola* (Latreille, 1802). Nearby localities of sequenced specimens are merged in pies (left). Colours match the bidimensional colour space of the PCoA projection (Fig. 451 left) of p-dist between sequences (dots). Specimen identification (ID or cf.) and source (newly sequenced or retrieved) are represented by colours, while specimen attribute (terra typica, type locality, type specimen or faunistic novelty) is represented by the shape (right). Sequences: ID = 50, cf. = 0; maximum p-distance: strict = 4.6 %, less strict = 4.6 %.

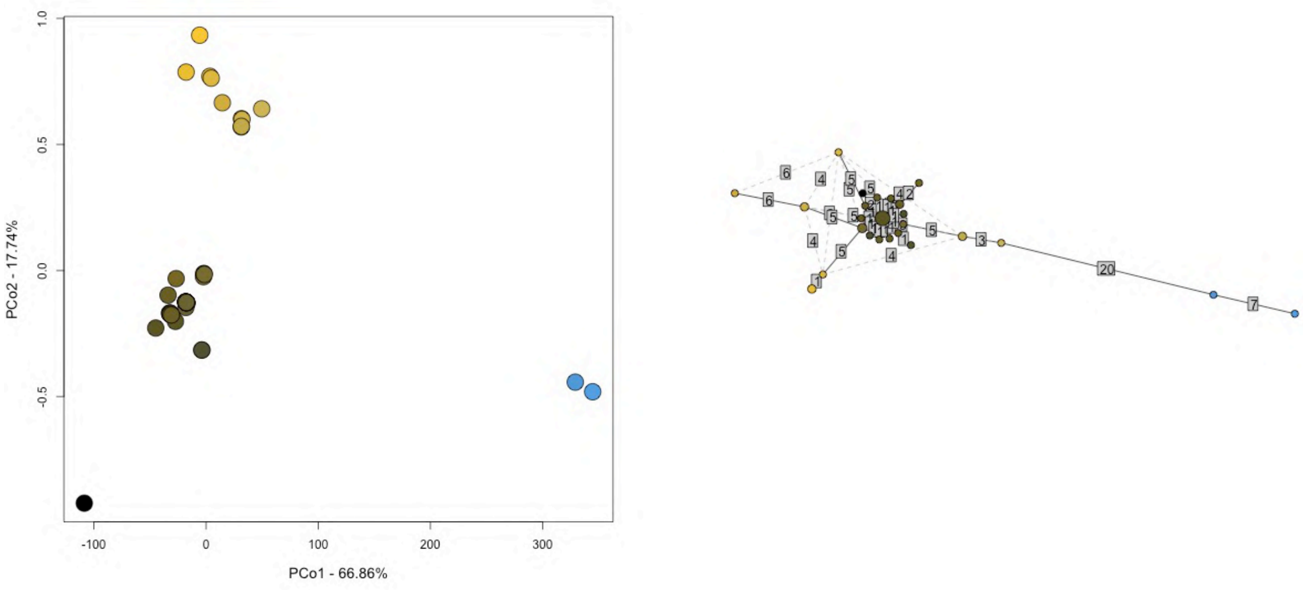

Figure 452: PCoA based on pairwise p-distances between *Myrmecina graminicola* sequences (left). Colours match a bidimensional colour space. Haplotype network of *Myrmecina graminicola* (right). Sequences > 599 bp: ID = 50, cf. = 0.

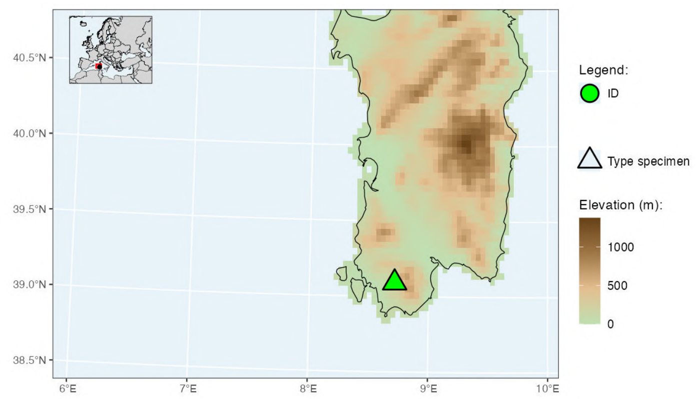

Figure 453: Map of *Myrmecina melonii* Rigato, 1999. Due to the presence of a single sequence, the genetic diversity map and the PCoA projection were not done. Specimen identification (ID or cf.) and source (newly sequenced or retrieved) are represented by colours, while specimen attribute (terra typica, type locality, type specimen or faunistic novelty) is represented by the shape. Sequences: ID = 1, cf. = 0; maximum p-distance: strict = NA, less strict = NA.

Haplotype network analysis of *Myrmecina melonii* was not possible.

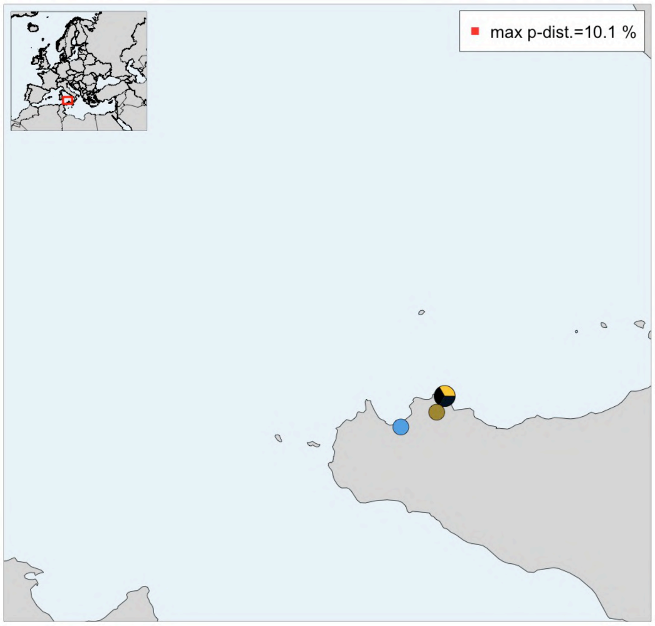

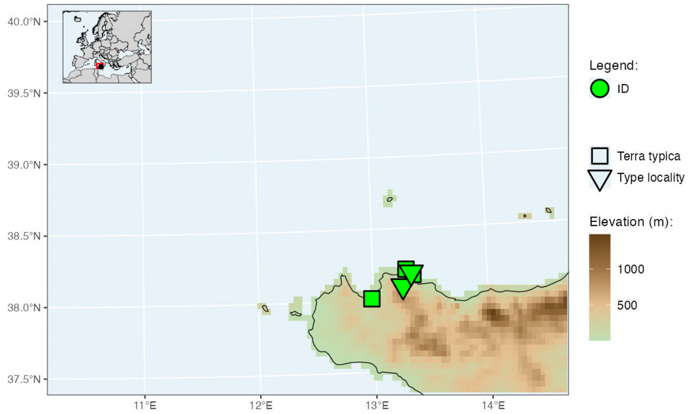

Figure 454: Genetic diversity map of *Myrmecina sicula* André, 1882. Nearby localities of sequenced specimens are merged in pies (left). Colours match the bidimensional colour space of the PCoA projection (Fig. 454 left) of p-dist between sequences (dots). Specimen identification (ID or cf.) and source (newly sequenced or retrieved) are represented by colours, while specimen attribute (terra typica, type locality, type specimen or faunistic novelty) is represented by the shape (right). Sequences: ID = 5, cf. = 0; maximum p-distance: strict = 10.1 %, less strict = 10.1 %.

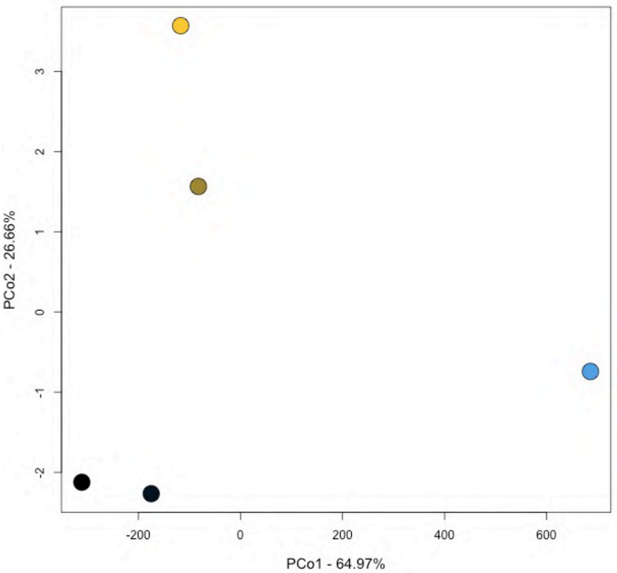

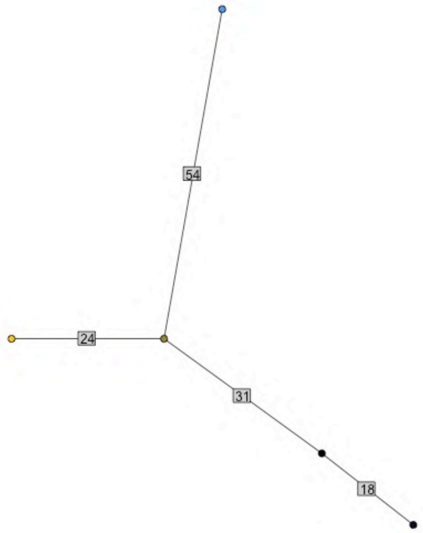

Figure 455: PCoA based on pairwise p-distances between *Myrmecina sicula* sequences (left). Colours match a bidimensional colour space. Haplotype network of *Myrmecina sicula* (right). Sequences > 599 bp: ID = 5, cf. = 0.

Myrmica

Myrmica aloba

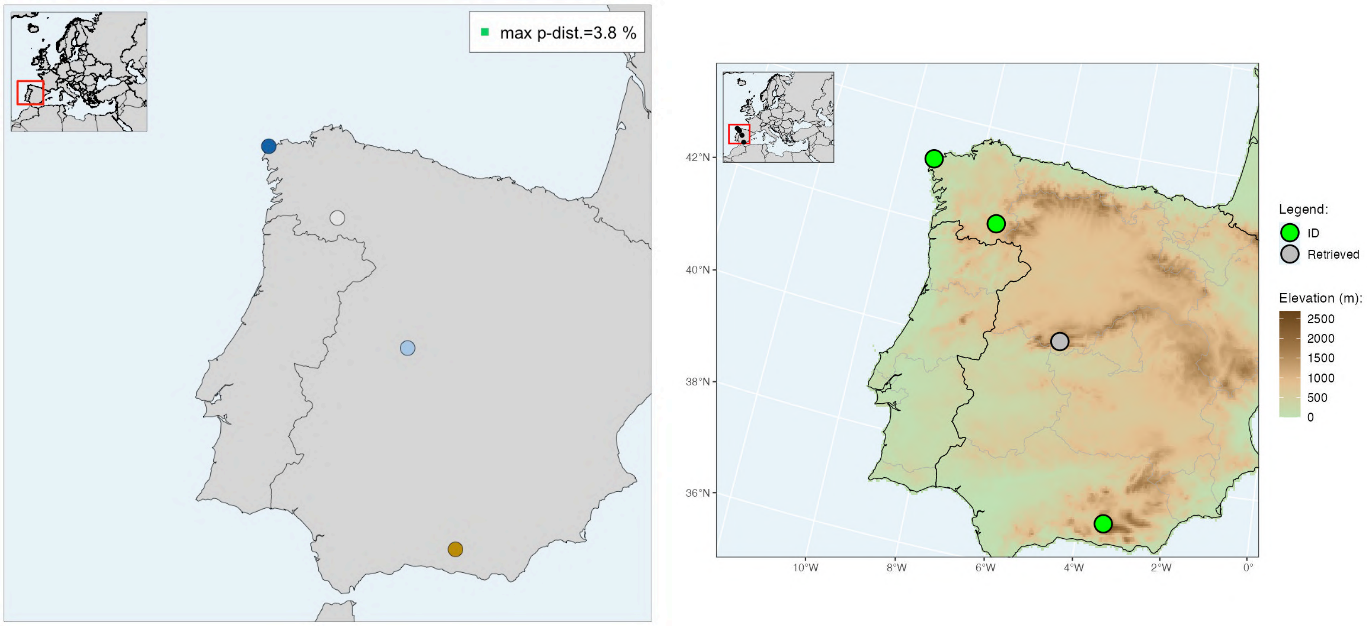

Figure 456: Genetic diversity map of *Myrmica aloba* Forel, 1909. Nearby localities of sequenced specimens are merged in pies (left). Colours match the bidimensional colour space of the PCoA projection (Fig. 456 left) of p-dist between sequences (dots). Specimen identification (ID or cf.) and source (newly sequenced or retrieved) are represented by colours, while specimen attribute (terra typica, type locality, type specimen or faunistic novelty) is represented by the shape (right). Sequences: ID = 5, cf. = 0; maximum p-distance: strict = 3.8 %, less strict = 3.8 %.

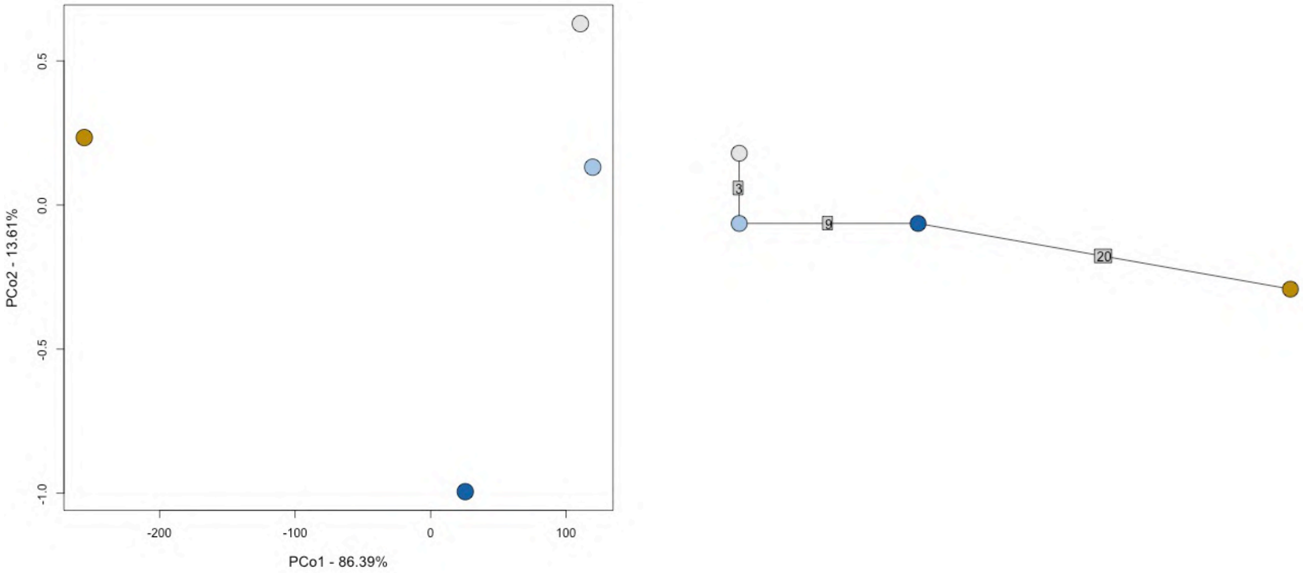

Figure 457: PCoA based on pairwise p-distances between *Myrmica aloba* sequences (left). Colours match a bidimensional colour space. Haplotype network of *Myrmica aloba* (right). Sequences > 599 bp: ID = 5, cf. = 0.

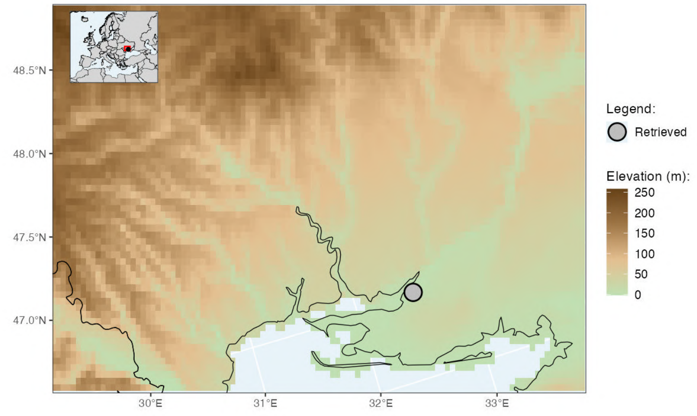

Figure 458: Map of *Myrmica bergi* Ruzsky, 1902. Due to the presence of a single sequence, the genetic diversity map and the PCoA projection were not done. Specimen identification (ID or cf.) and source (newly sequenced or retrieved) are represented by colours, while specimen attribute (terra typica, type locality, type specimen or faunistic novelty) is represented by the shape. Sequences: ID = 1, cf. = 0; maximum p-distance: strict = NA, less strict = NA.

Haplotype network analysis of *Myrmica bergi* was not possible.

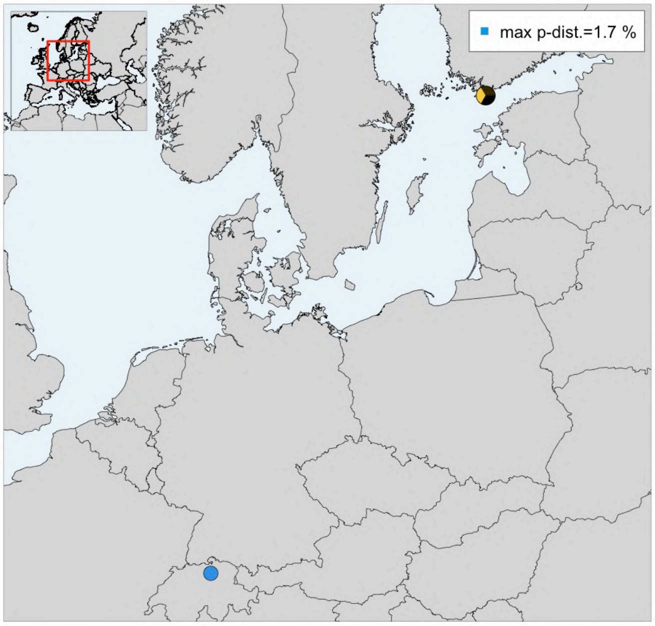

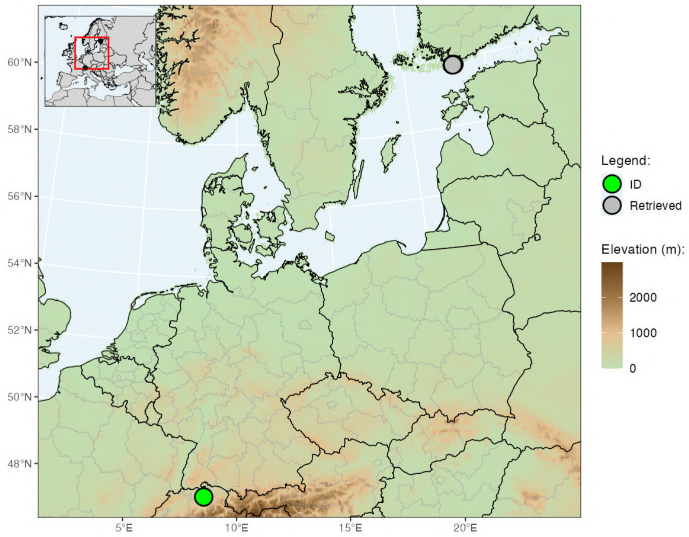

Figure 459: Genetic diversity map of *Myrmica constricta* Karavaiev, 1934. Nearby localities of sequenced specimens are merged in pies (left). Colours match the bidimensional colour space of the PCoA projection (Fig. 459 left) of p-dist between sequences (dots). Specimen identification (ID or cf.) and source (newly sequenced or retrieved) are represented by colours, while specimen attribute (terra typica, type locality, type specimen or faunistic novelty) is represented by the shape (right). Sequences: ID = 4, cf. = 0; maximum p-distance: strict = 0 %, less strict = 1.7 %.

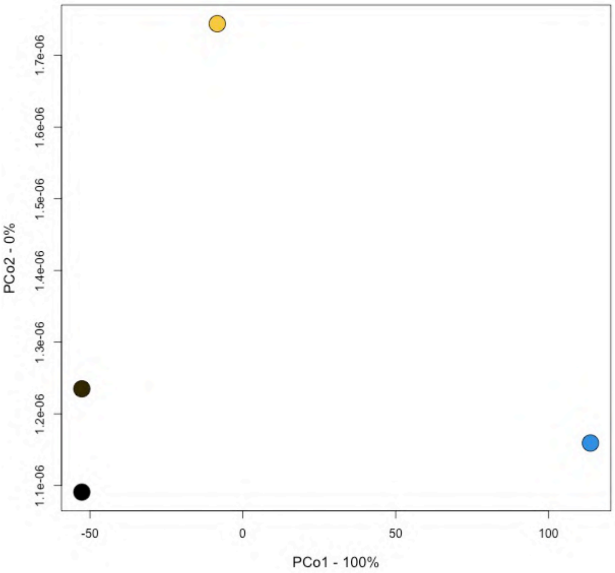

Figure 460: PCoA based on pairwise p-distances between *Myrmica constricta* sequences (left). Colours match a bidimensional colour space. Haplotype network analysis of *Myrmica constricta* was not possible.

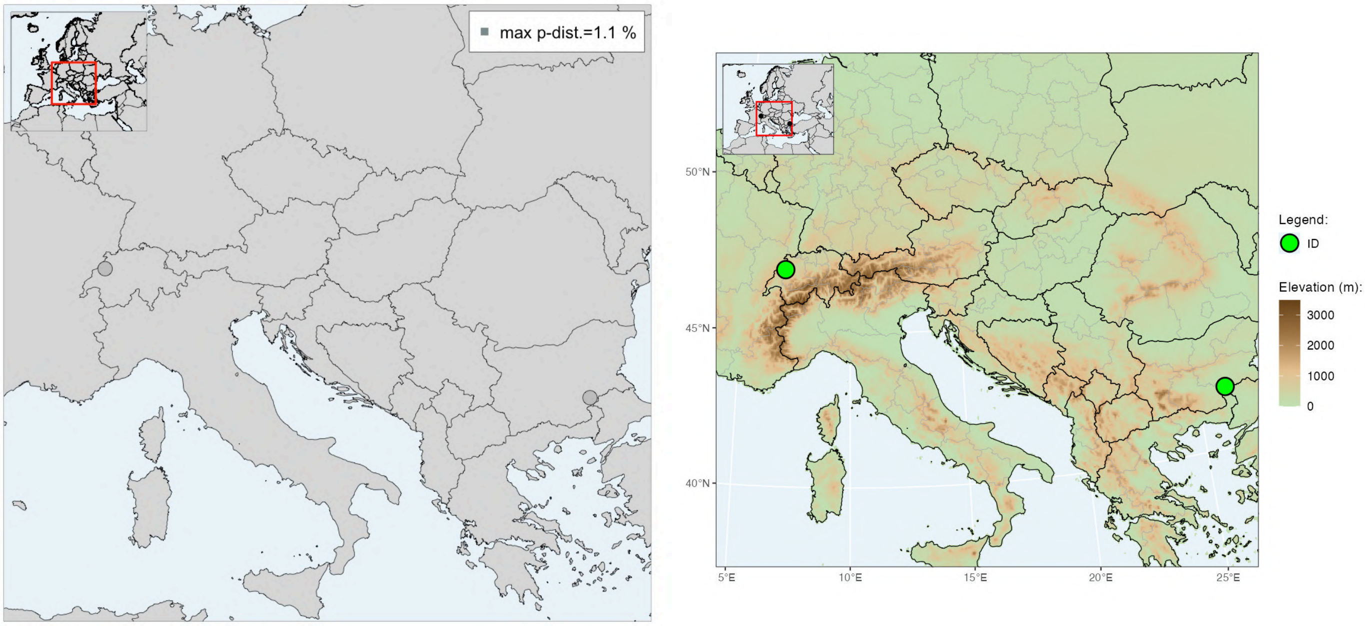

Figure 461: Genetic diversity map of *Myrmica curvithorax* Bondroit, 1920. PCoA projection was not done and therefore sequenced specimens in the genetic diversity map are coloured in gray (left). Specimen identification (ID or cf.) and source (newly sequenced or retrieved) are represented by colours, while specimen attribute (terra typica, type locality, type specimen or faunistic novelty) is represented by the shape (right). Sequences: ID = 2, cf. = 0; maximum p-distance: strict = NA, less strict = 1.1 %.

Haplotype network analysis of *Myrmica curvithorax* was not possible.

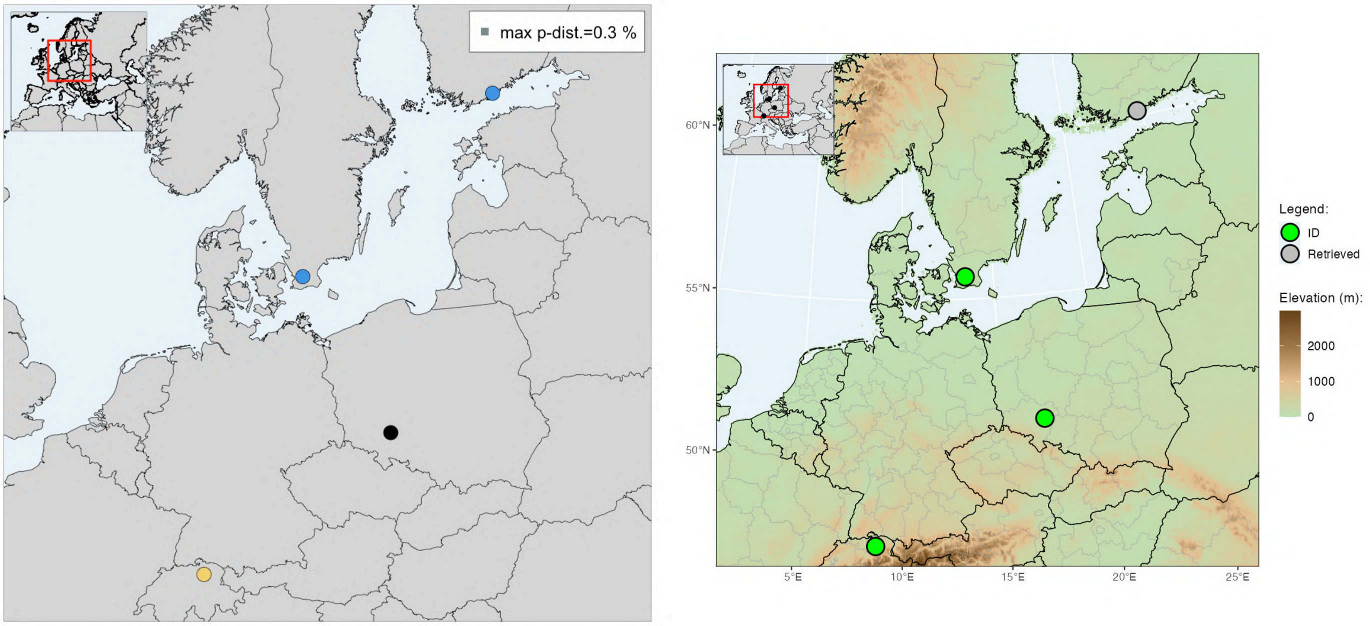

Figure 462: Genetic diversity map of *Myrmica gallienii* Bondroit, 1920. Nearby localities of sequenced specimens are merged in pies (left). Colours match the bidimensional colour space of the PCoA projection (Fig. 462 left) of p-dist between sequences (dots). Specimen identification (ID or cf.) and source (newly sequenced or retrieved) are represented by colours, while specimen attribute (terra typica, type locality, type specimen or faunistic novelty) is represented by the shape (right). Sequences: ID = 4, cf. = 0; maximum p-distance: strict = 0.3 %, less strict = 0.3 %.

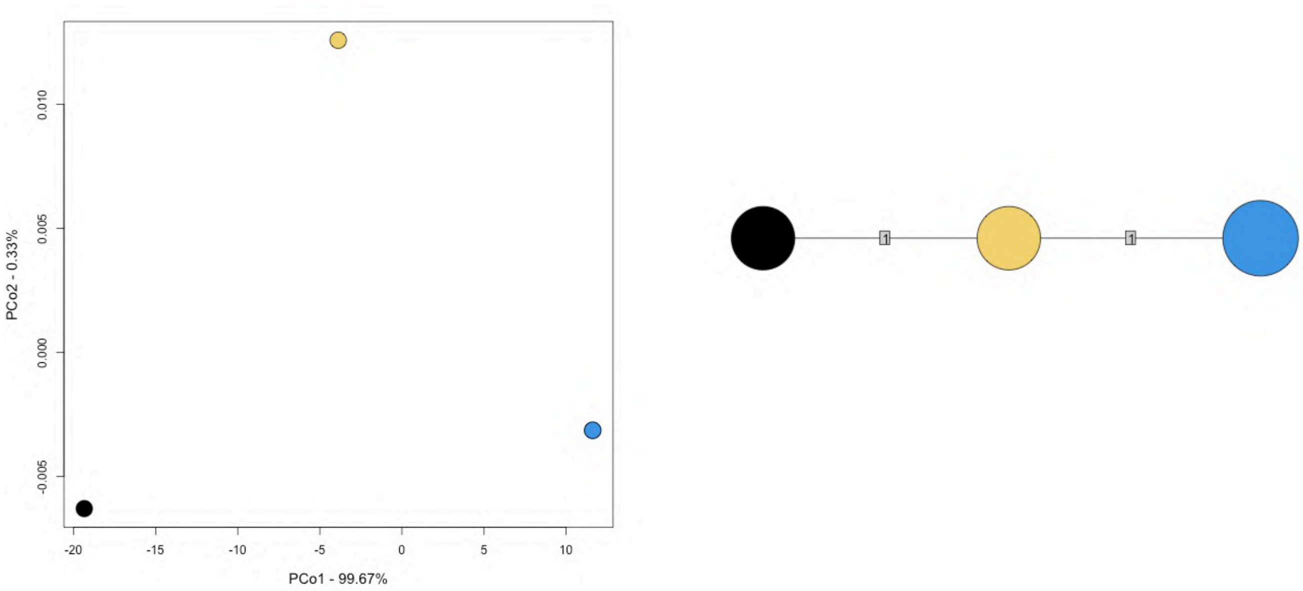

Figure 463: PCoA based on pairwise p-distances between *Myrmica gallienii* sequences (left). Colours match a bidimensional colour space. Haplotype network of *Myrmica gallienii* (right). Sequences > 599 bp: ID = 4, cf. = 0.

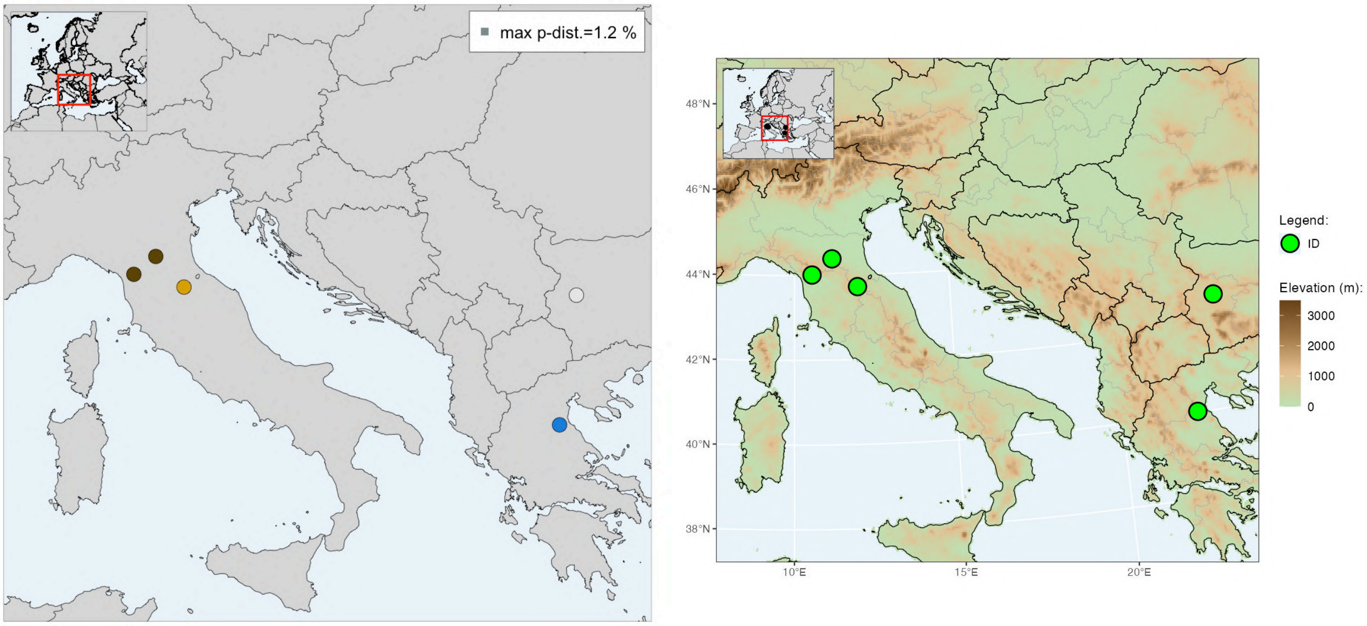

Figure 464: Genetic diversity map of *Myrmica hellenica* Finzi, 1926. Nearby localities of sequenced specimens are merged in pies (left). Colours match the bidimensional colour space of the PCoA projection (Fig. 464 left) of p-dist between sequences (dots). Specimen identification (ID or cf.) and source (newly sequenced or retrieved) are represented by colours, while specimen attribute (terra typica, type locality, type specimen or faunistic novelty) is represented by the shape (right). Sequences: ID = 5, cf. = 0; maximum p-distance: strict = 1.3 %, less strict = 1.3 %.

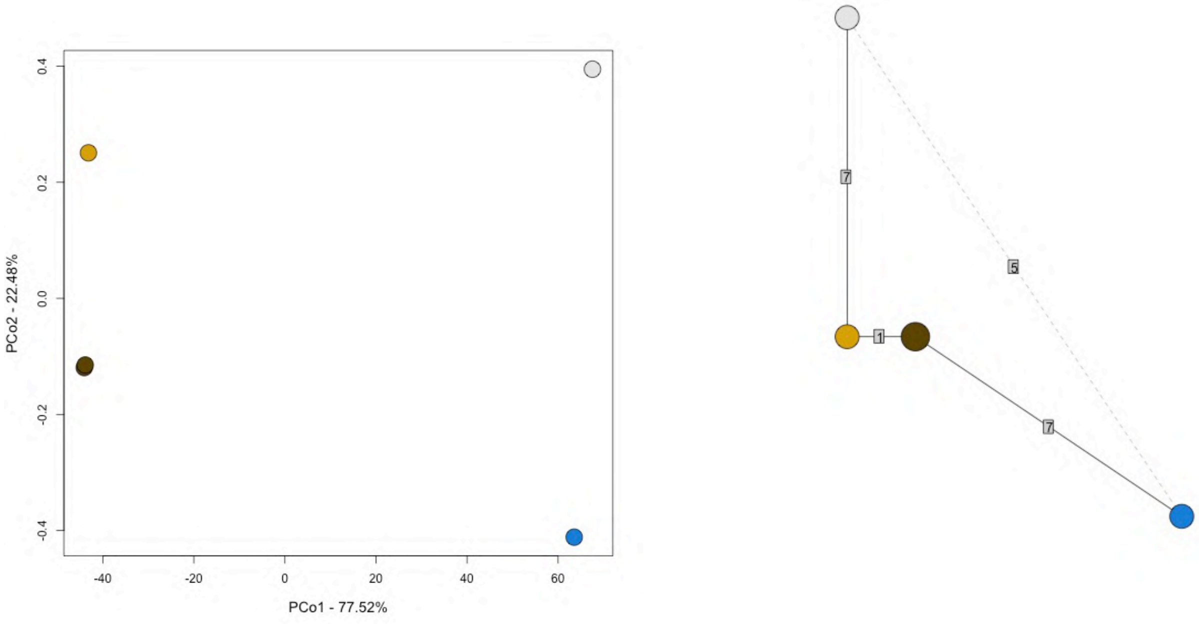

Figure 465: PCoA based on pairwise p-distances between *Myrmica hellenica* sequences (left). Colours match a bidimensional colour space. Haplotype network of *Myrmica hellenica* (right). Sequences > 599 bp: ID = 5, cf. = 0.

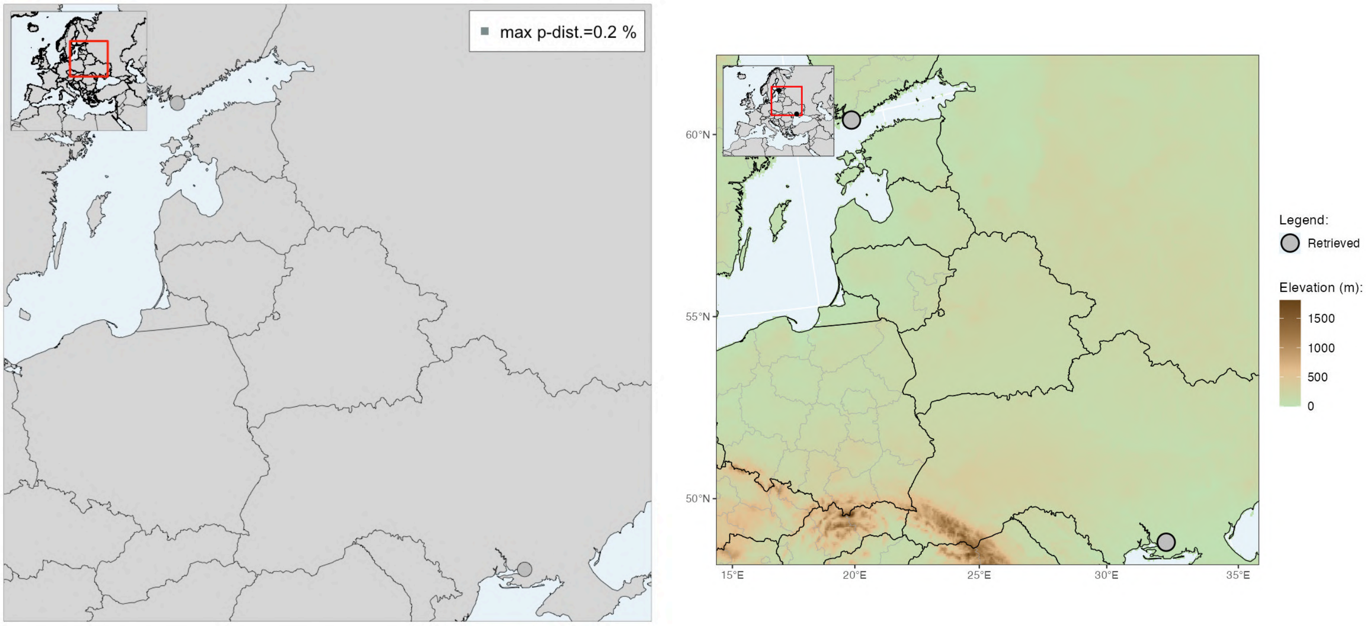

Figure 466: Genetic diversity map of *Myrmica hirsuta* Elmes, 1978. PCoA projection was not done and therefore sequenced specimens in the genetic diversity map are coloured in gray (left). Specimen identification (ID or cf.) and source (newly sequenced or retrieved) are represented by colours, while specimen attribute (terra typica, type locality, type specimen or faunistic novelty) is represented by the shape (right). Sequences: ID = 2, cf. = 0; maximum p-distance: strict = NA, less strict = 0.2 %.

Haplotype network analysis of *Myrmica hirsuta* was not possible.

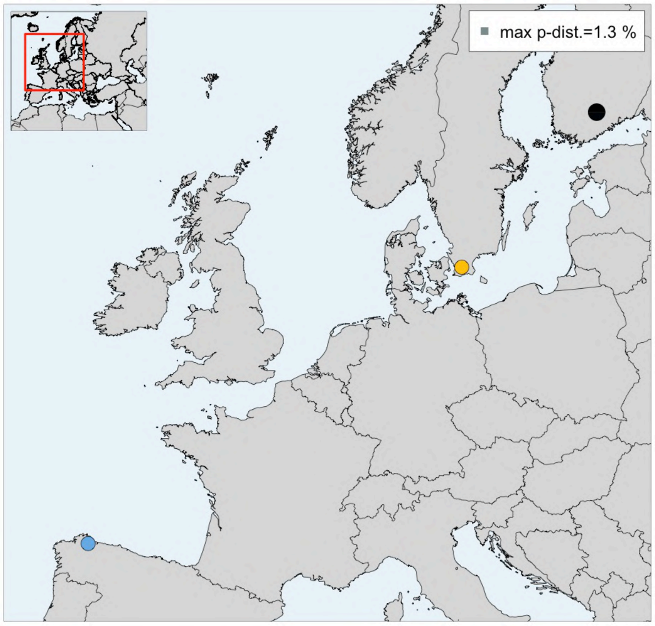

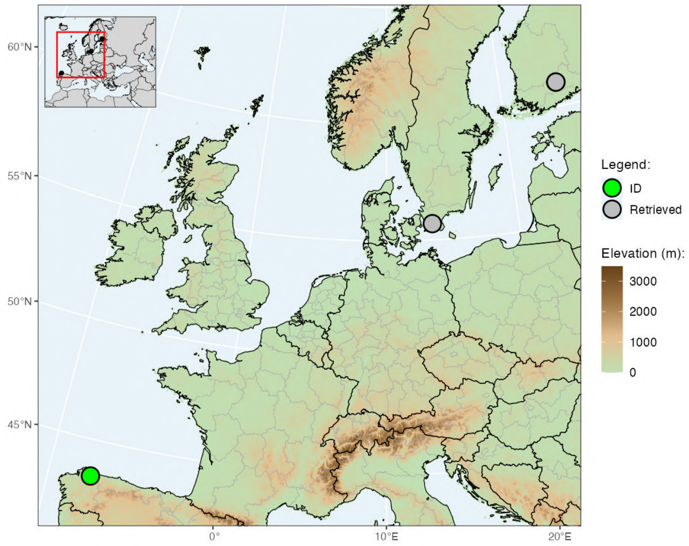

Figure 467: Genetic diversity map of *Myrmica karavajevi* (Arnol'di, 1930). Nearby localities of sequenced specimens are merged in pies (left). Colours match the bidimensional colour space of the PCoA projection (Fig. 467 left) of p-dist between sequences (dots). Specimen identification (ID or cf.) and source (newly sequenced or retrieved) are represented by colours, while specimen attribute (terra typica, type locality, type specimen or faunistic novelty) is represented by the shape (right). Sequences: ID = 4, cf. = 0; maximum p-distance: strict = 1.3 %, less strict = 1.3 %.

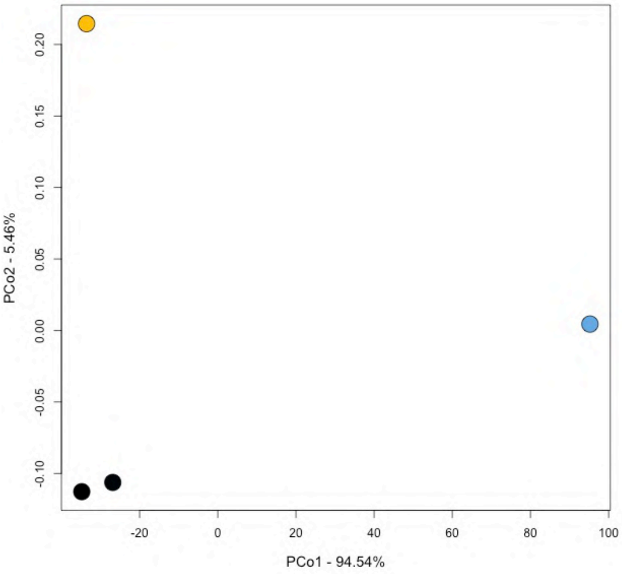

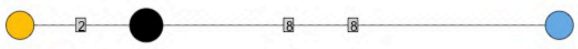

Figure 468: PCoA based on pairwise p-distances between *Myrmica karavajevi* sequences (left). Colours match a bidimensional colour space. Haplotype network of *Myrmica karavajevi* (right). Sequences > 599 bp: ID = 4, cf. = 0.

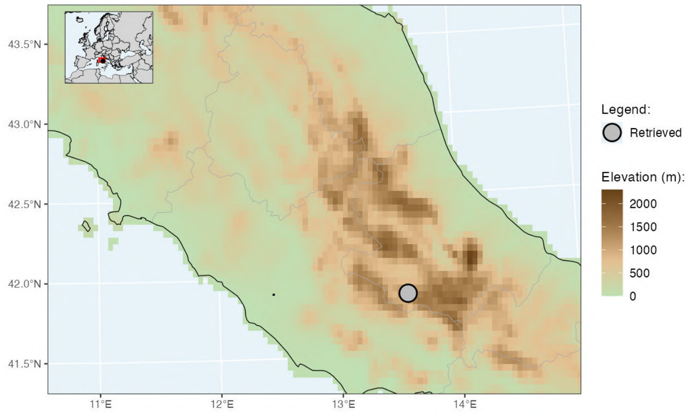

Figure 469: Map of *Myrmica laurae* (Emery, 1907). Due to the presence of a single sequence, the genetic diversity map and the PCoA projection were not done. Specimen identification (ID or cf.) and source (newly sequenced or retrieved) are represented by colours, while specimen attribute (terra typica, type locality, type specimen or faunistic novelty) is represented by the shape. Sequences: ID = 1, cf. = 0; maximum p-distance: strict = NA, less strict = NA.

Haplotype network analysis of *Myrmica laurae* was not possible.

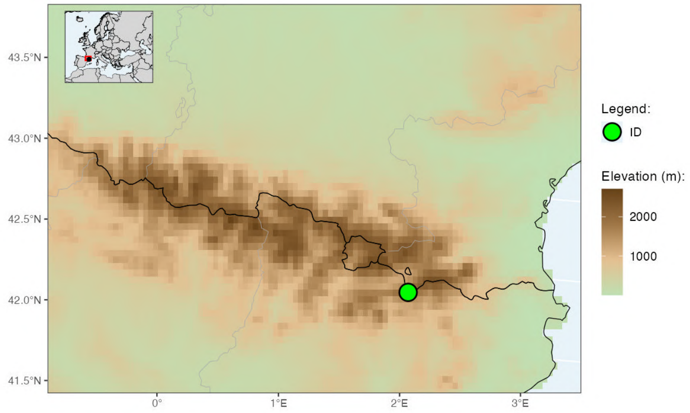

Figure 470: Map of *Myrmica lemasnei* Bernard, 1967. Due to the presence of a single sequence, the genetic diversity map and the PCoA projection were not done. Specimen identification (ID or cf.) and source (newly sequenced or retrieved) are represented by colours, while specimen attribute (terra typica, type locality, type specimen or faunistic novelty) is represented by the shape. Sequences: ID = 1, cf. = 0; maximum p-distance: strict = NA, less strict = NA.

Haplotype network analysis of *Myrmica lemasnei* was not possible.

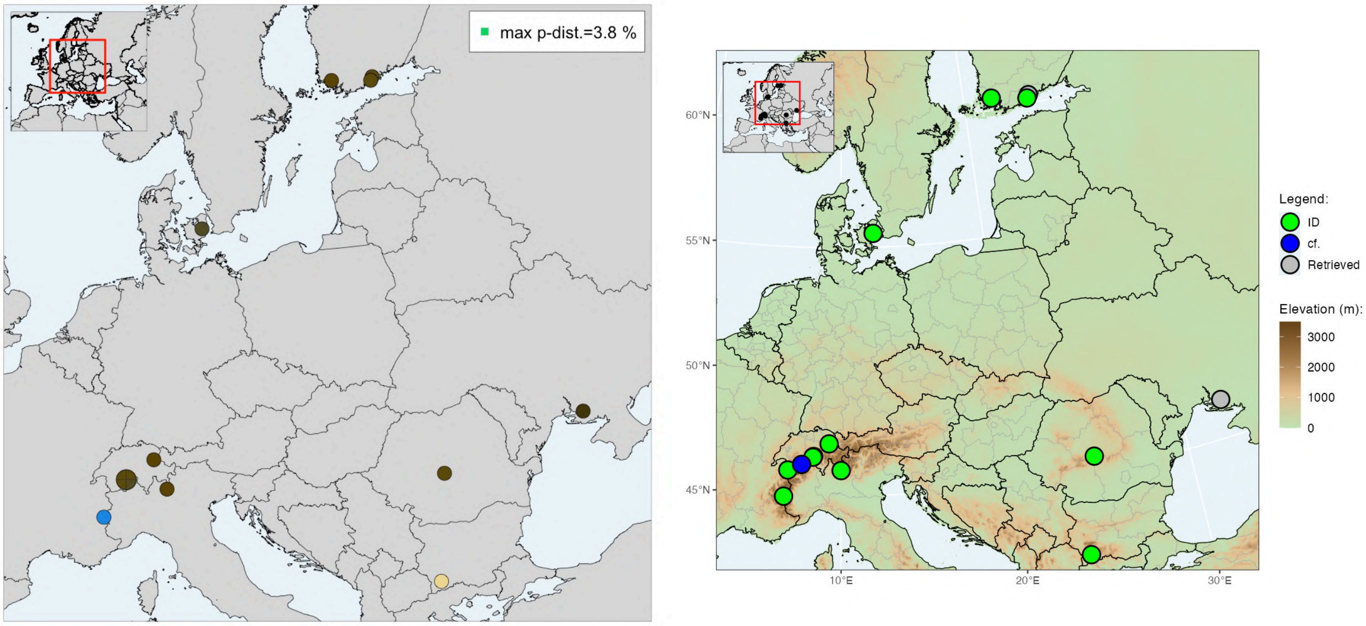

Figure 471: Genetic diversity map of *Myrmica lobicornis* Nylander, 1846. Nearby localities of sequenced specimens are merged in pies (left). Colours match the bidimensional colour space of the PCoA projection (Fig. 471 left) of p-dist between sequences (dots). Specimen identification (ID or cf.) and source (newly sequenced or retrieved) are represented by colours, while specimen attribute (terra typica, type locality, type specimen or faunistic novelty) is represented by the shape (right). Sequences: ID = 13, cf. = 1; maximum p-distance: strict = 3.8 %, less strict = 3.8 %.

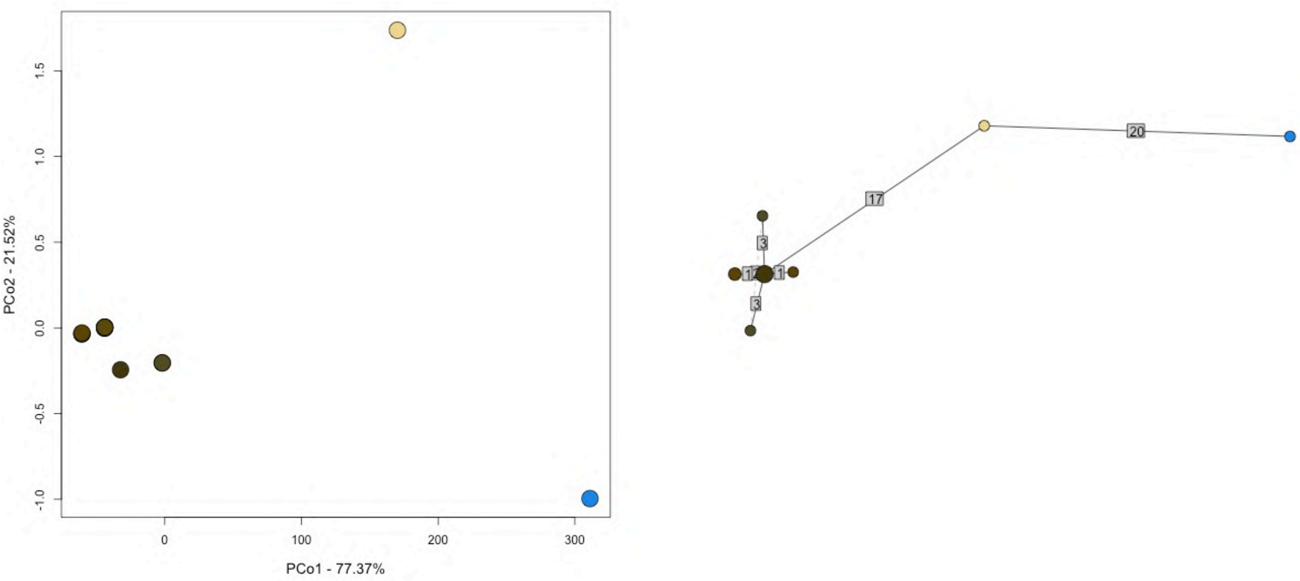

Figure 472: PCoA based on pairwise p-distances between *Myrmica lobicornis* sequences (left). Colours match a bidimensional colour space. Haplotype network of *Myrmica lobicornis* (right). Sequences > 599 bp: ID = 13, cf. = 1.

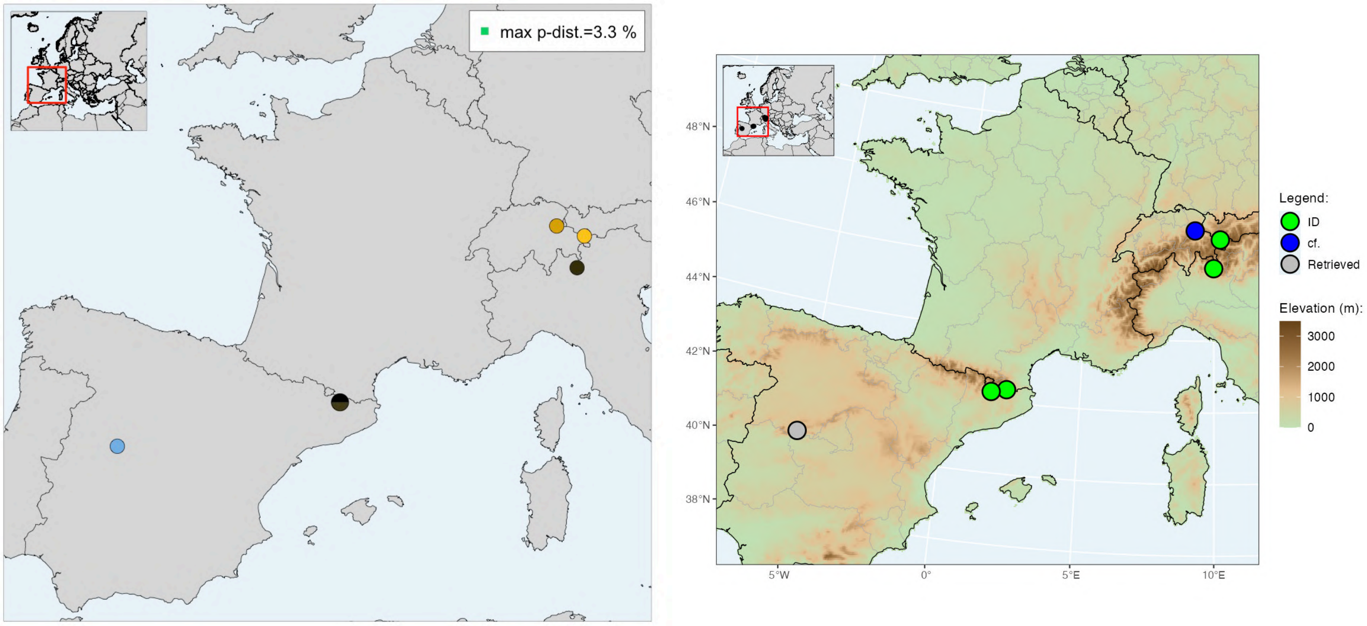

Figure 473: Genetic diversity map of *Myrmica lobulicornis* Nylander, 1857. Nearby localities of sequenced specimens are merged in pies (left). Colours match the bidimensional colour space of the PCoA projection (Fig. 473 left) of p-dist between sequences (dots). Specimen identification (ID or cf.) and source (newly sequenced or retrieved) are represented by colours, while specimen attribute (terra typica, type locality, type specimen or faunistic novelty) is represented by the shape (right). Sequences: ID = 5, cf. = 1; maximum p-distance: strict = 3.3 %, less strict = 3.3 %.

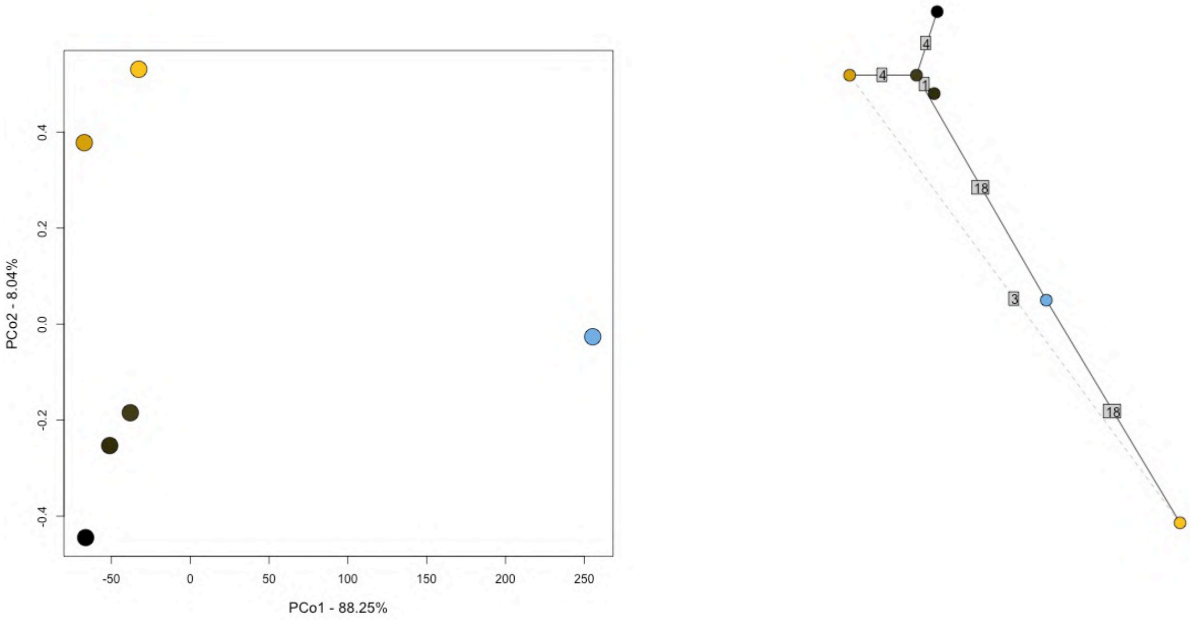

Figure 474: PCoA based on pairwise p-distances between *Myrmica lobulicornis* sequences (left). Colours match a bidimensional colour space. Haplotype network of *Myrmica lobulicornis* (right). Sequences > 599 bp: ID = 5, cf. = 1.

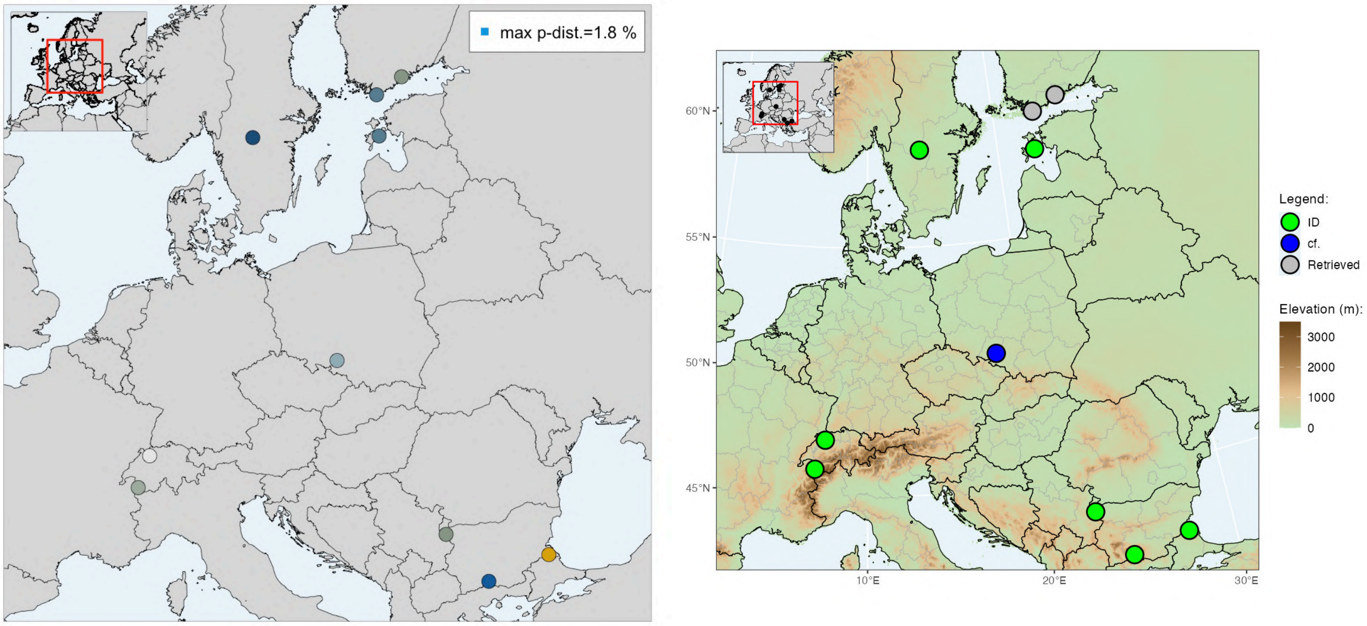

Figure 475: Genetic diversity map of *Myrmica lonae* Finzi, 1926. Nearby localities of sequenced specimens are merged in pies (left). Colours match the bidimensional colour space of the PCoA projection (Fig. 475 left) of p-dist between sequences (dots). Specimen identification (ID or cf.) and source (newly sequenced or retrieved) are represented by colours, while specimen attribute (terra typica, type locality, type specimen or faunistic novelty) is represented by the shape (right). Sequences: ID = 9, cf. = 1; maximum p-distance: strict = 1.8 %, less strict = 1.8 %.

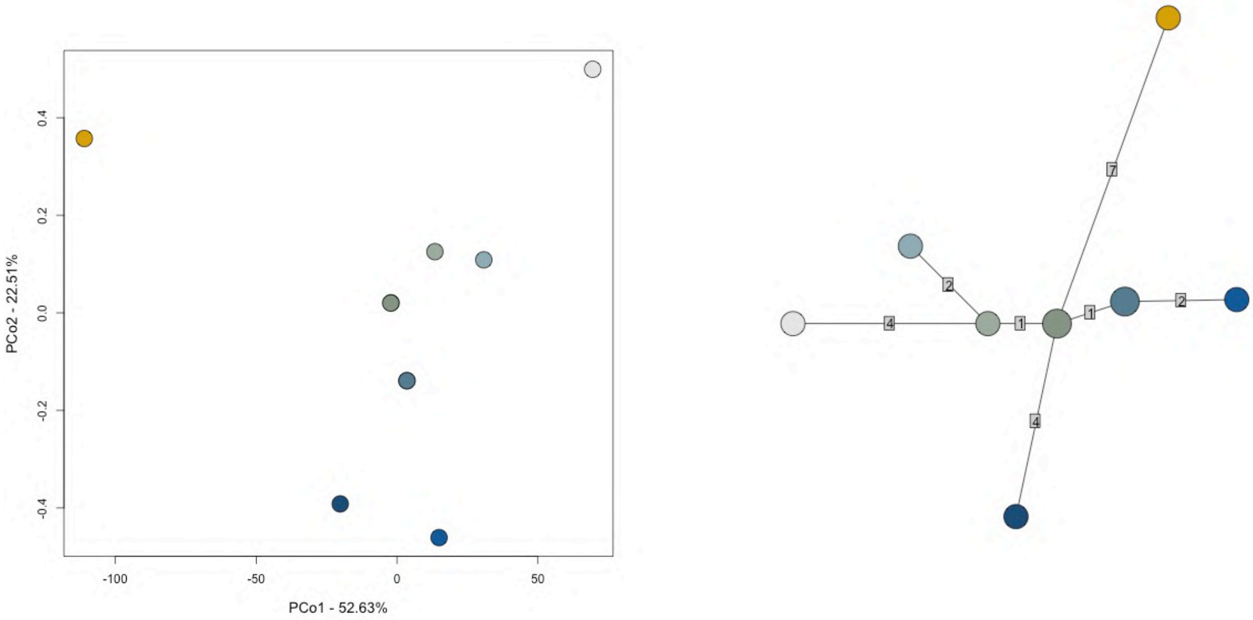

Figure 476: PCoA based on pairwise p-distances between *Myrmica lonae* sequences (left). Colours match a bidimensional colour space. Haplotype network of *Myrmica lonae* (right). Sequences > 599 bp: ID = 9, cf. = 1.

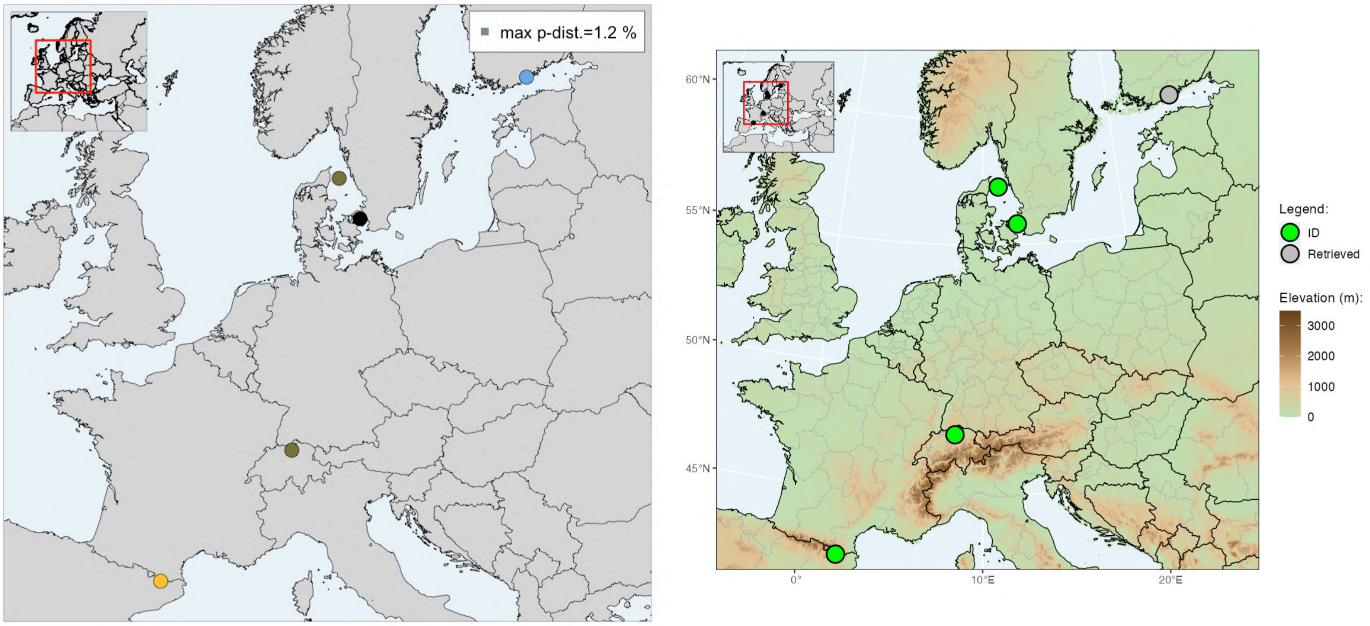

Figure 477: Genetic diversity map of *Myrmica microrubra* Seifert, 1993. Nearby localities of sequenced specimens are merged in pies (left). Colours match the bidimensional colour space of the PCoA projection (Fig. 477 left) of p-dist between sequences (dots). Specimen identification (ID or cf.) and source (newly sequenced or retrieved) are represented by colours, while specimen attribute (terra typica, type locality, type specimen or faunistic novelty) is represented by the shape (right). Sequences: ID = 5, cf. = 0; maximum p-distance: strict = 1.2 %, less strict = 1.2 %.

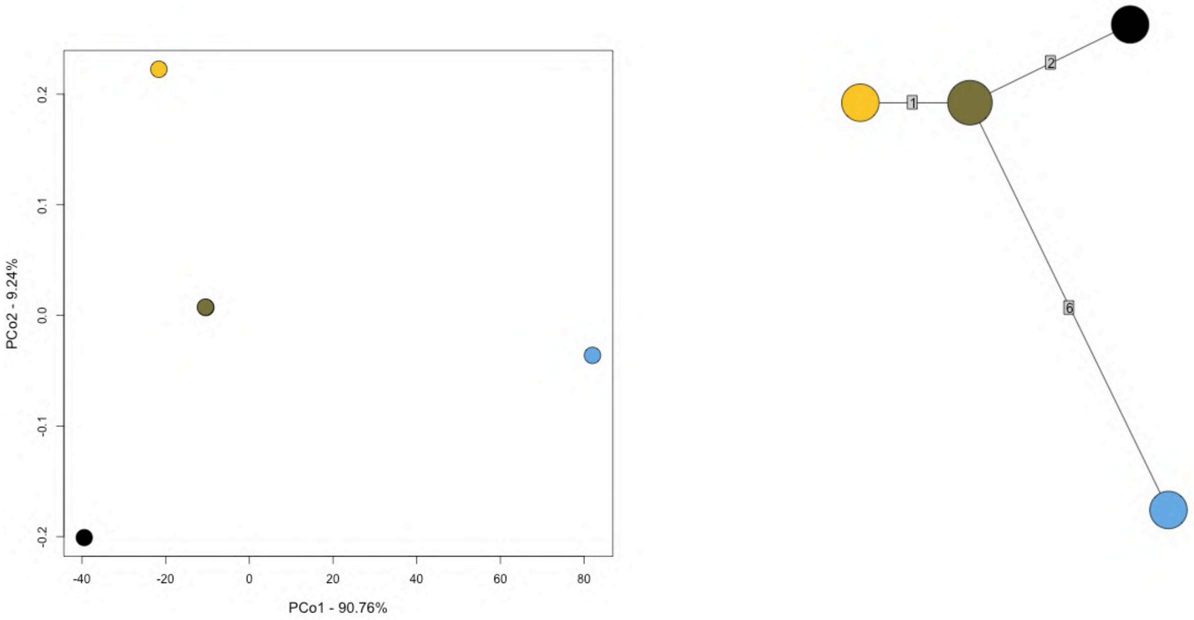

Figure 478: PCoA based on pairwise p-distances between *Myrmica microrubra* sequences (left). Colours match a bidimensional colour space. Haplotype network of *Myrmica microrubra* (right). Sequences > 599 bp: ID = 5, cf. = 0.

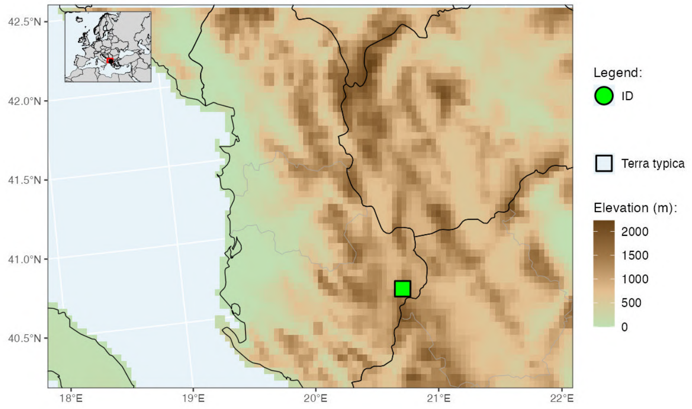

Figure 479: Map of *Myrmica ravasinii* Finzi, 1923. Due to the presence of a single sequence, the genetic diversity map and the PCoA projection were not done. Specimen identification (ID or cf.) and source (newly sequenced or retrieved) are represented by colours, while specimen attribute (terra typica, type locality, type specimen or faunistic novelty) is represented by the shape. Sequences: ID = 1, cf. = 0; maximum p-distance: strict = NA, less strict = NA.

Haplotype network analysis of *Myrmica ravasinii* was not possible.

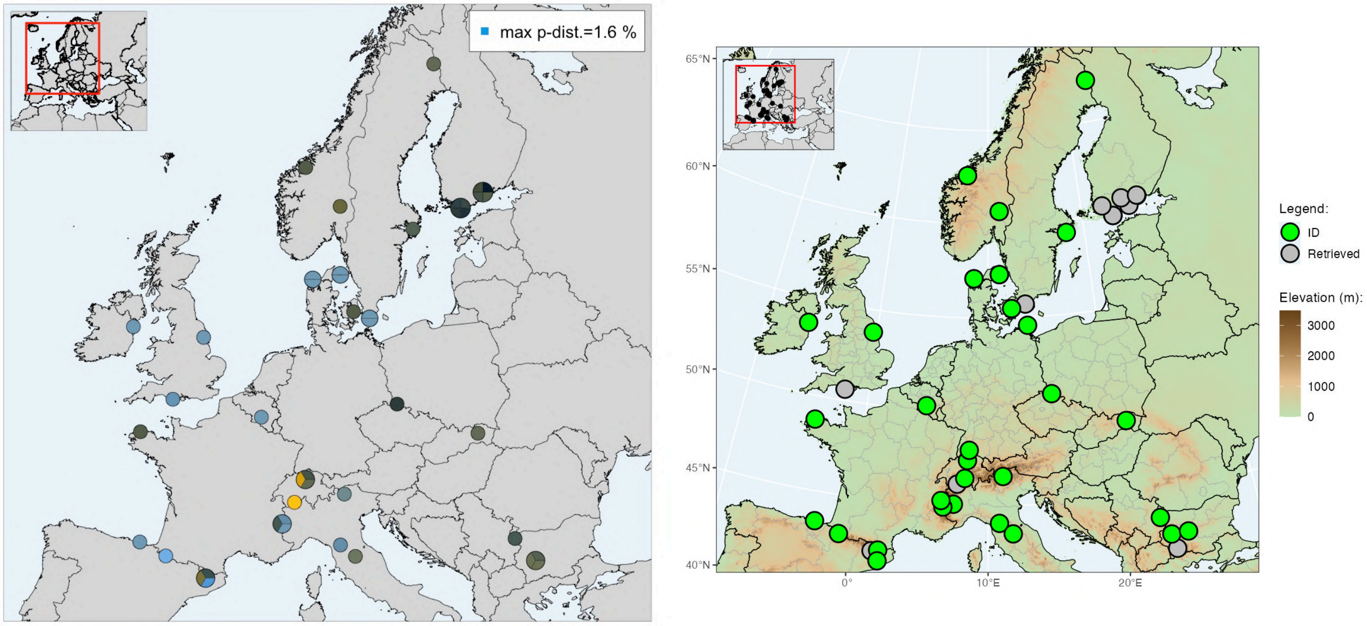

Figure 480: Genetic diversity map of *Myrmica rubra* (Linnaeus, 1758). Nearby localities of sequenced specimens are merged in pies (left). Colours match the bidimensional colour space of the PCoA projection (Fig. 480 left) of p-dist between sequences (dots). Specimen identification (ID or cf.) and source (newly sequenced or retrieved) are represented by colours, while specimen attribute (terra typica, type locality, type specimen or faunistic novelty) is represented by the shape (right). Sequences: ID = 45, cf. = 0; maximum p-distance: strict = 1.6 %, less strict = 1.6 %.

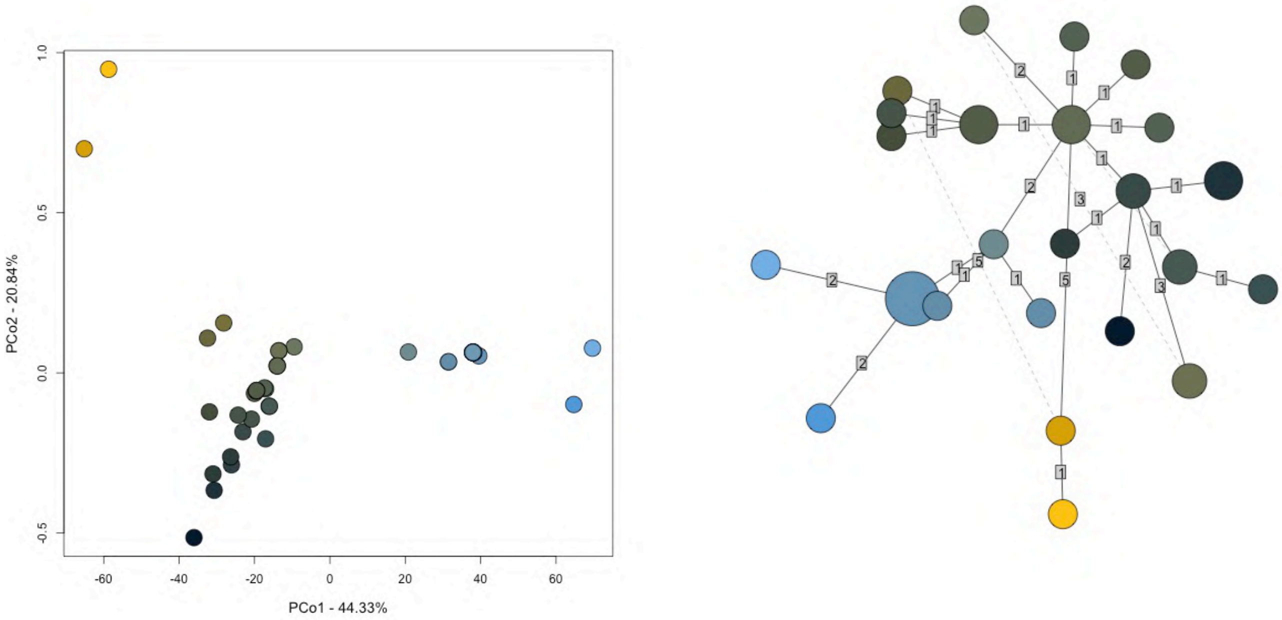

Figure 481: PCoA based on pairwise p-distances between *Myrmica rubra* sequences (left). Colours match a bidimensional colour space. Haplotype network of *Myrmica rubra* (right). Sequences > 599 bp: ID = 44, cf. = 0.

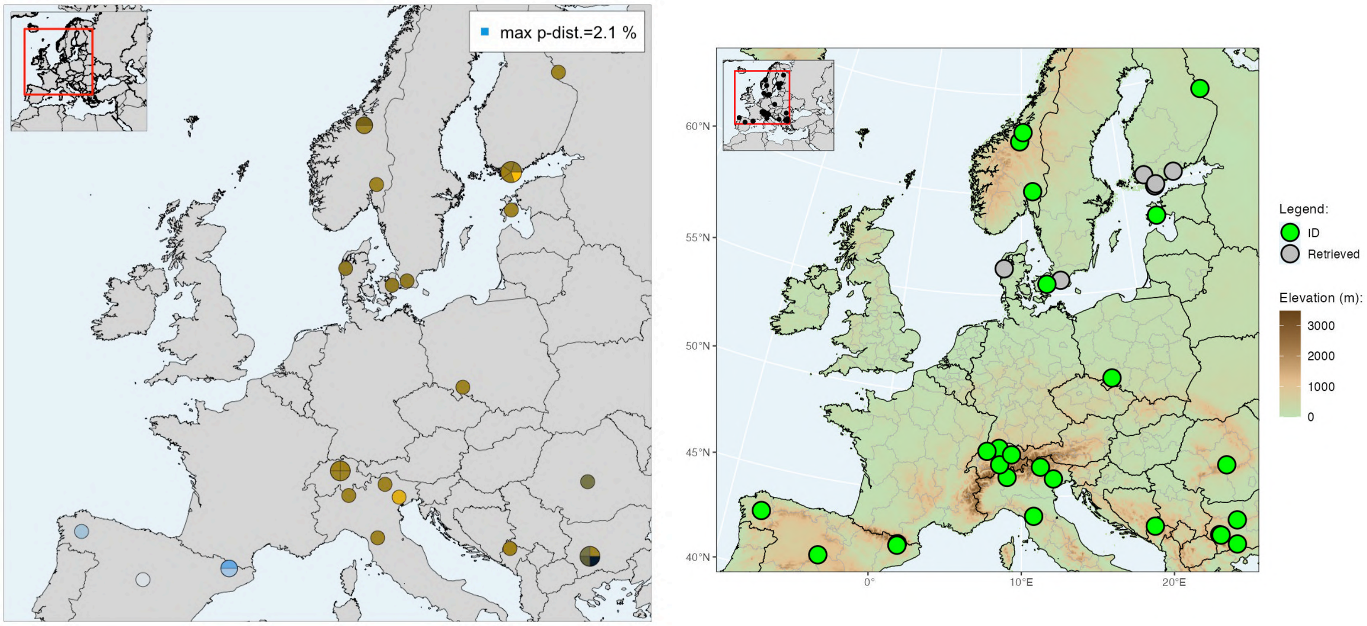

Figure 482: Genetic diversity map of *Myrmica ruginodis* Nylander, 1846. Nearby localities of sequenced specimens are merged in pies (left). Colours match the bidimensional colour space of the PCoA projection (Fig. 482 left) of p-dist between sequences (dots). Specimen identification (ID or cf.) and source (newly sequenced or retrieved) are represented by colours, while specimen attribute (terra typica, type locality, type specimen or faunistic novelty) is represented by the shape (right). Sequences: ID = 32, cf. = 0; maximum p-distance: strict = 2.1 %, less strict = 2.1 %.

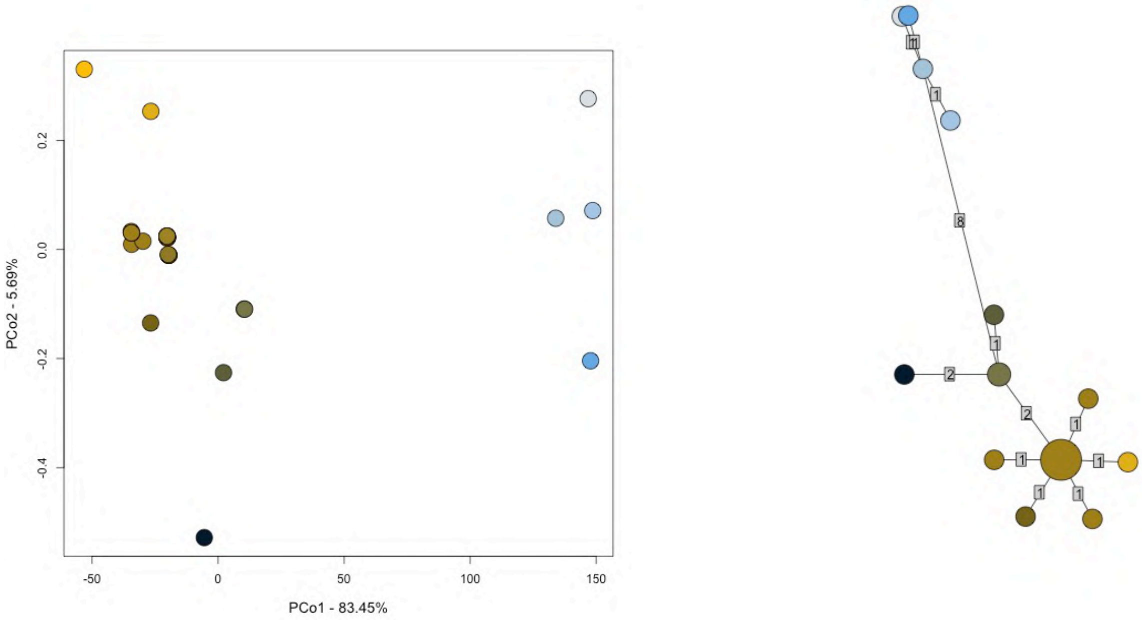

Figure 483: PCoA based on pairwise p-distances between *Myrmica ruginodis* sequences (left). Colours match a bidimensional colour space. Haplotype network of *Myrmica ruginodis* (right). Sequences > 599 bp: ID = 32, cf. = 0.

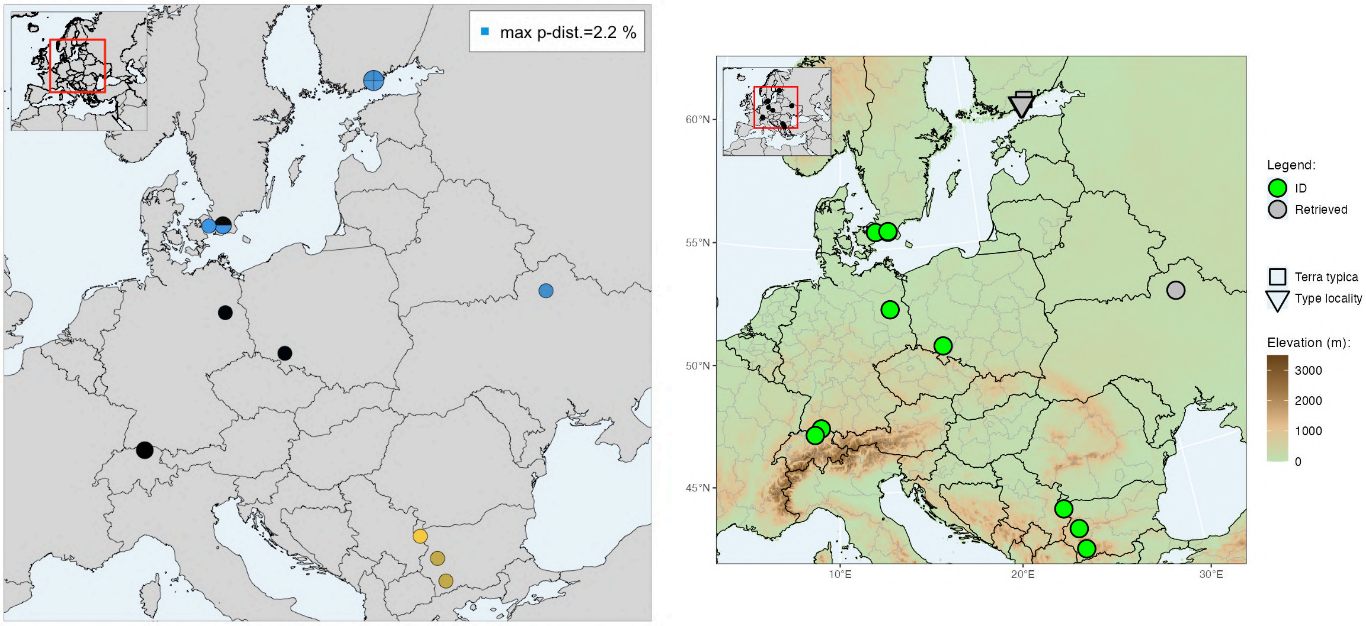

Figure 484: Genetic diversity map of *Myrmica rugulosa* Nylander, 1849. Nearby localities of sequenced specimens are merged in pies (left). Colours match the bidimensional colour space of the PCoA projection (Fig. 484 left) of p-dist between sequences (dots). Specimen identification (ID or cf.) and source (newly sequenced or retrieved) are represented by colours, while specimen attribute (terra typica, type locality, type specimen or faunistic novelty) is represented by the shape (right). Sequences: ID = 15, cf. = 0; maximum p-distance: strict = 2.2 %, less strict = 2.2 %.

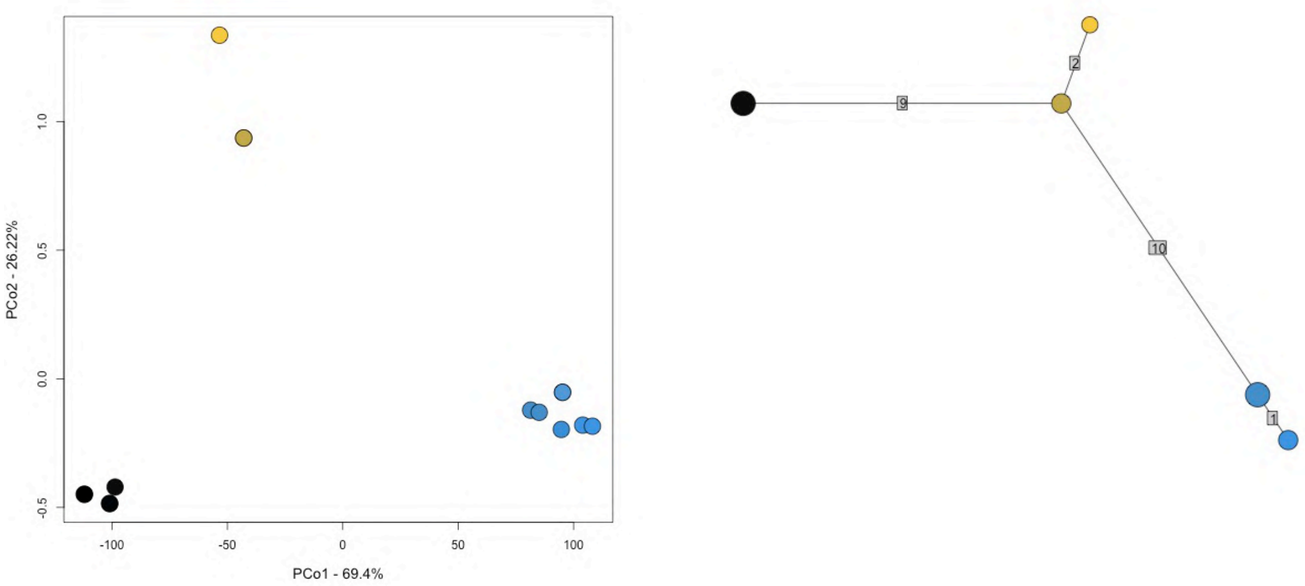

Figure 485: PCoA based on pairwise p-distances between *Myrmica rugulosa* sequences (left). Colours match a bidimensional colour space. Haplotype network of *Myrmica rugulosa* (right). Sequences > 599 bp: ID = 15, cf. = 0.

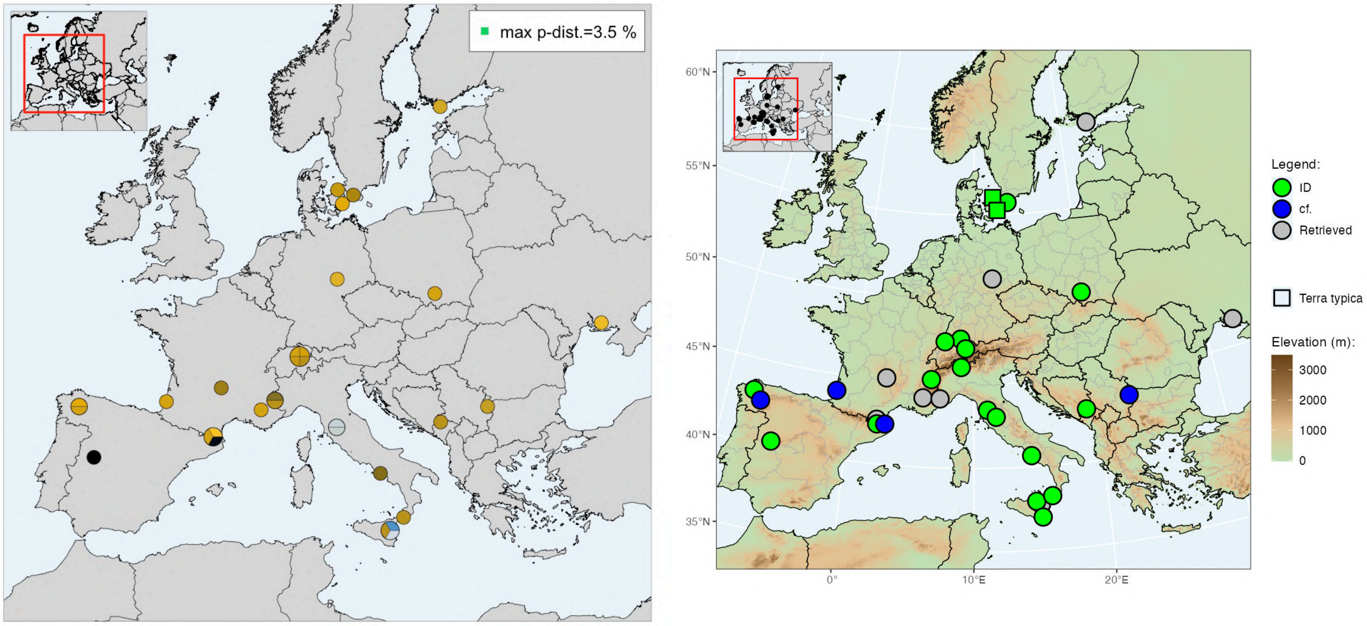

Figure 486: Genetic diversity map of *Myrmica sabuleti* Meinert, 1861. Nearby localities of sequenced specimens are merged in pies (left). Colours match the bidimensional colour space of the PCoA projection (Fig. 486 left) of p-dist between sequences (dots). Specimen identification (ID or cf.) and source (newly sequenced or retrieved) are represented by colours, while specimen attribute (terra typica, type locality, type specimen or faunistic novelty) is represented by the shape (right). Sequences: ID = 27, cf. = 4; maximum p-distance: strict = 3.5 %, less strict = 3.5 %.

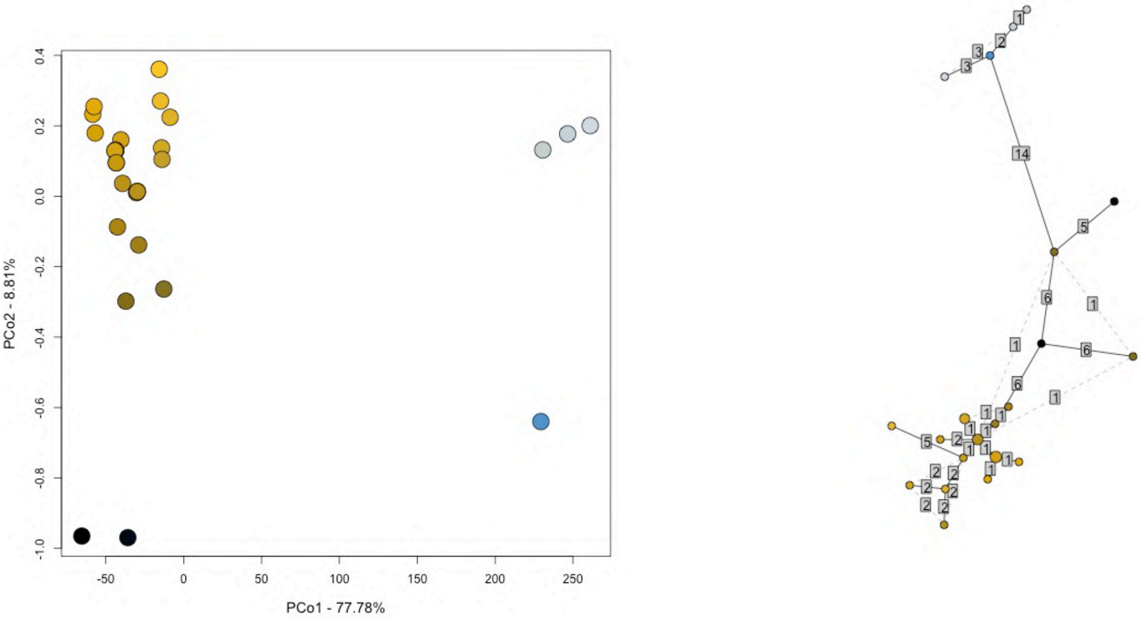

Figure 487: PCoA based on pairwise p-distances between *Myrmica sabuleti* sequences (left). Colours match a bidimensional colour space. Haplotype network of *Myrmica sabuleti* (right). Sequences > 599 bp: ID = 26, cf. = 4.

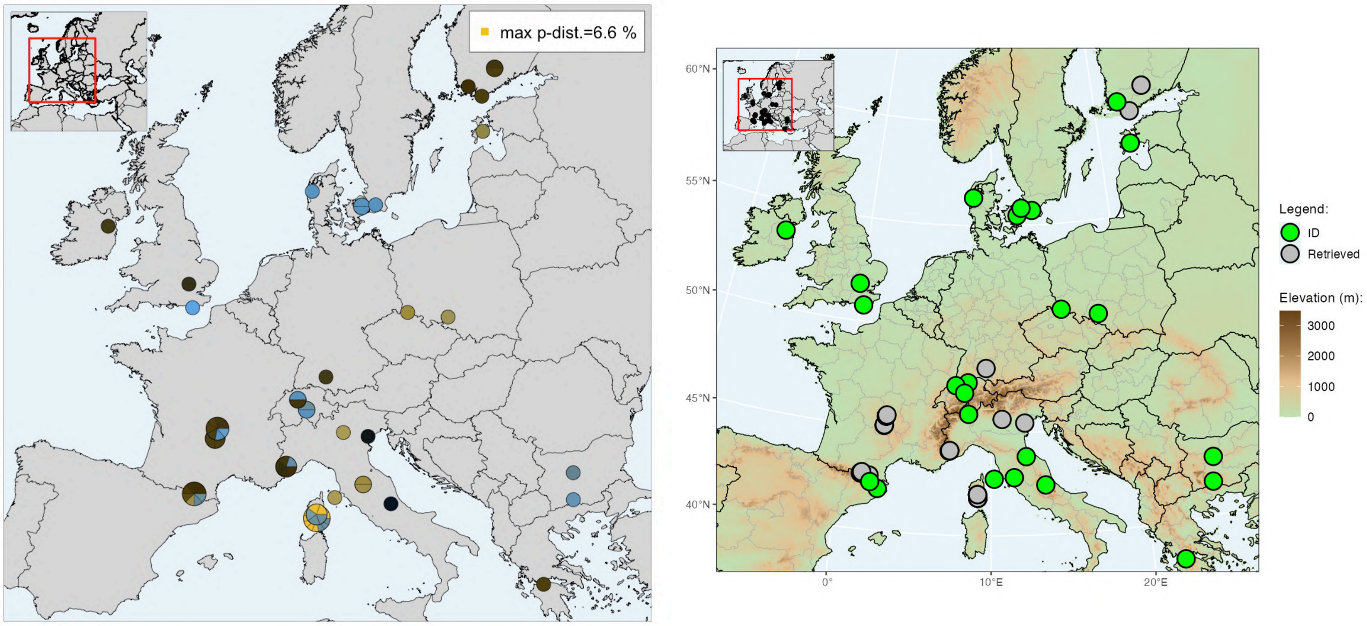

Figure 488: Genetic diversity map of *Myrmica scabrinodis* Nylander, 1846. Nearby localities of sequenced specimens are merged in pies (left). Colours match the bidimensional colour space of the PCoA projection (Fig. 488 left) of p-dist between sequences (dots). Specimen identification (ID or cf.) and source (newly sequenced or retrieved) are represented by colours, while specimen attribute (terra typica, type locality, type specimen or faunistic novelty) is represented by the shape (right). Sequences: ID = 70, cf. = 0; maximum p-distance: strict = 5.6 %, less strict = 6.6 %.

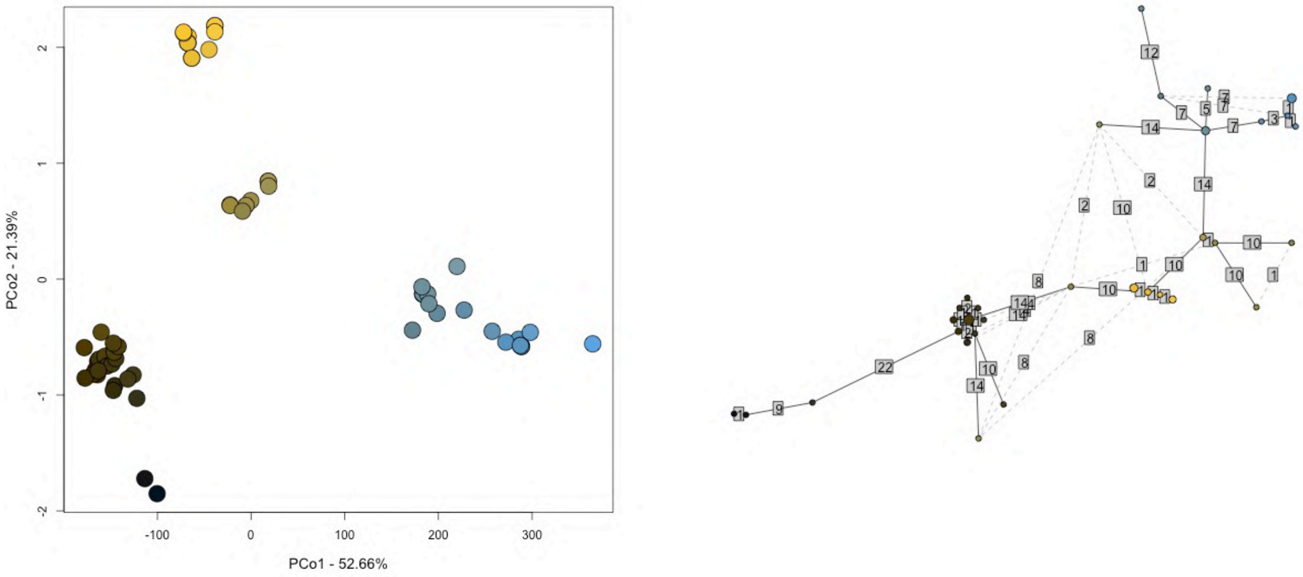

Figure 489: PCoA based on pairwise p-distances between *Myrmica scabrinodis* sequences (left). Colours match a bidimensional colour space. Haplotype network of *Myrmica scabrinodis* (right). Sequences > 599 bp: ID = 64, cf. = 0.

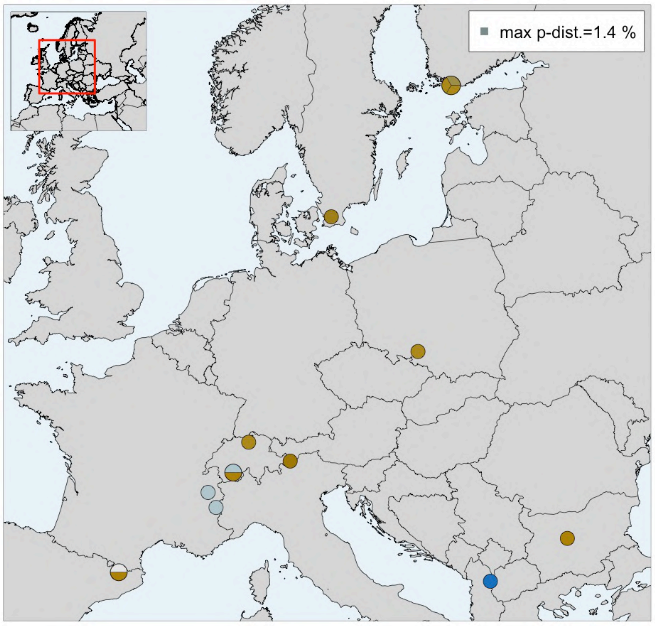

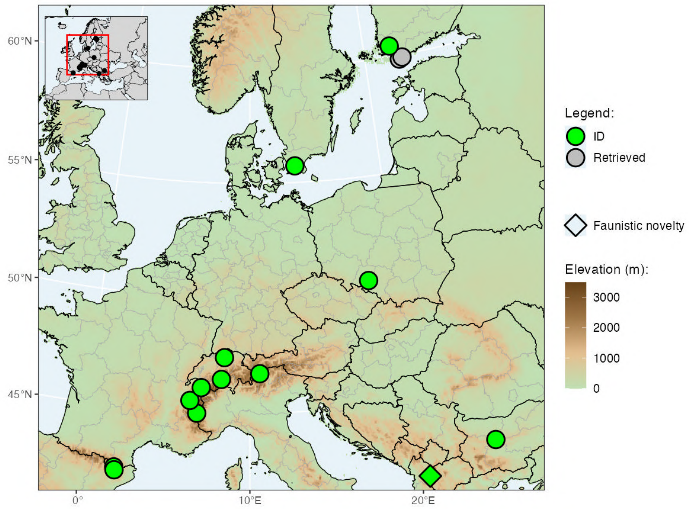

Figure 490: Genetic diversity map of *Myrmica schencki* Viereck, 1903. Nearby localities of sequenced specimens are merged in pies (left). Colours match the bidimensional colour space of the PCoA projection (Fig. 490 left) of p-dist between sequences (dots). Specimen identification (ID or cf.) and source (newly sequenced or retrieved) are represented by colours, while specimen attribute (terra typica, type locality, type specimen or faunistic novelty) is represented by the shape (right). Sequences: ID = 15, cf. = 0; maximum p-distance: strict = 1.4 %, less strict = 1.4 %.

The species is reported for the first time in Albania.

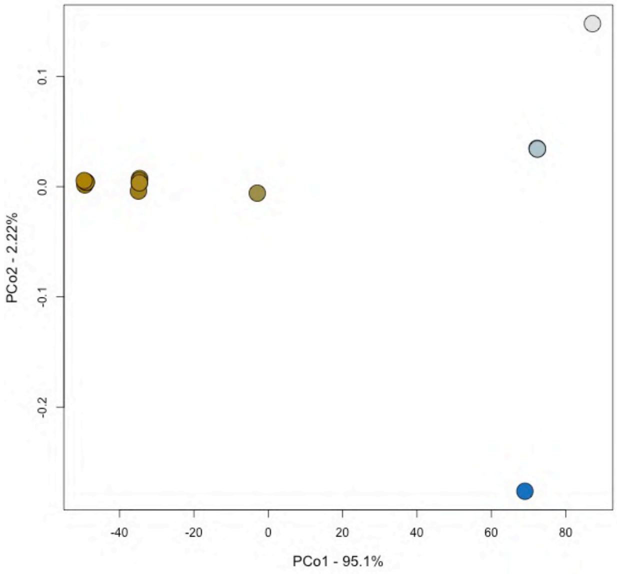

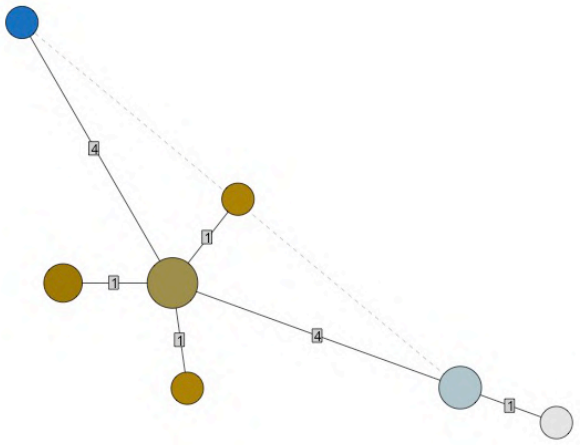

Figure 491: PCoA based on pairwise p-distances between *Myrmica schencki* sequences (left). Colours match a bidimensional colour space. Haplotype network of *Myrmica schencki* (right). Sequences > 599 bp: ID = 15, cf. = 0.

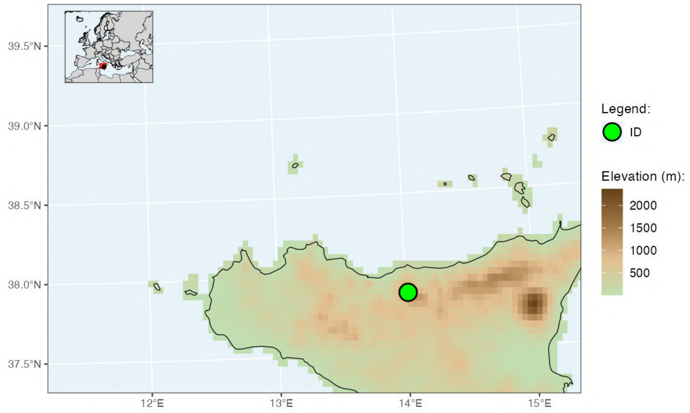

Figure 492: Map of *Myrmica siciliana* Radchenko, Elmes & Alicata, 2006. Due to the presence of a single sequence, the genetic diversity map and the PCoA projection were not done. Specimen identification (ID or cf.) and source (newly sequenced or retrieved) are represented by colours, while specimen attribute (terra typica, type locality, type specimen or faunistic novelty) is represented by the shape. Sequences: ID = 2, cf. = 0; maximum p-distance: strict = NA, less strict = NA.

Haplotype network analysis of *Myrmica siciliana* was not possible.

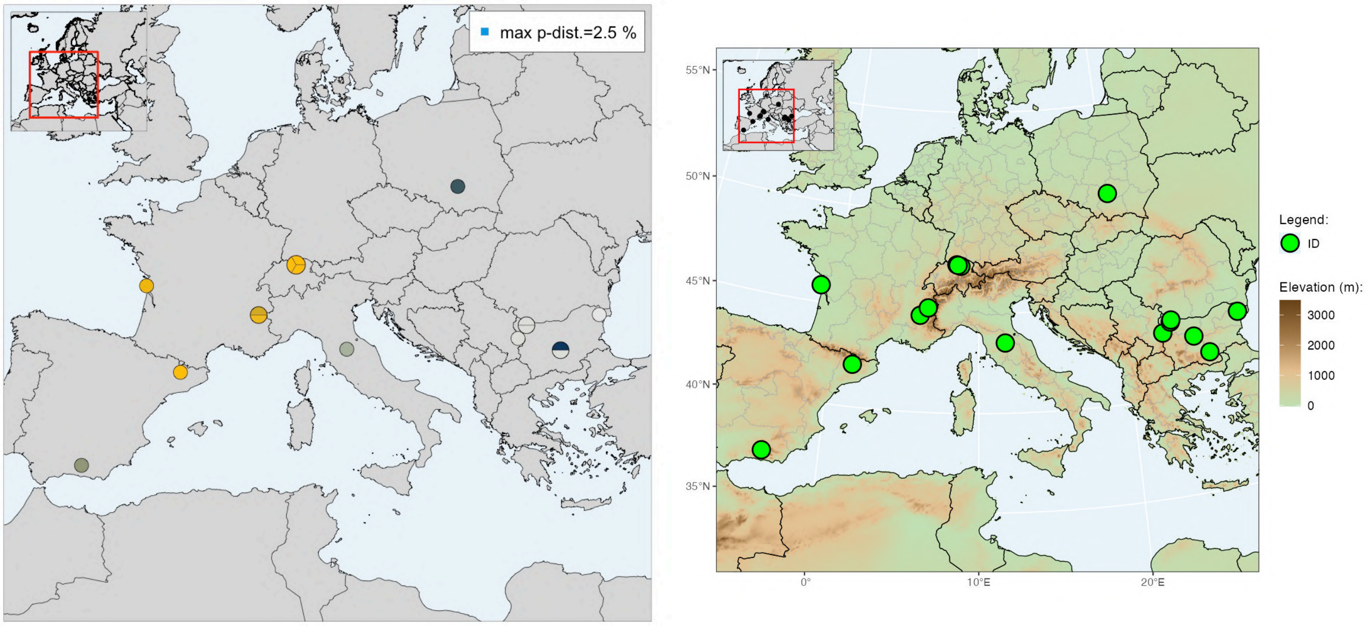

Figure 493: Genetic diversity map of *Myrmica specioides* Bondroit, 1918. Nearby localities of sequenced specimens are merged in pies (left). Colours match the bidimensional colour space of the PCoA projection (Fig. 493 left) of p-dist between sequences (dots). Specimen identification (ID or cf.) and source (newly sequenced or retrieved) are represented by colours, while specimen attribute (terra typica, type locality, type specimen or faunistic novelty) is represented by the shape (right). Sequences: ID = 16, cf. = 0; maximum p-distance: strict = 2.5 %, less strict = 2.5 %.

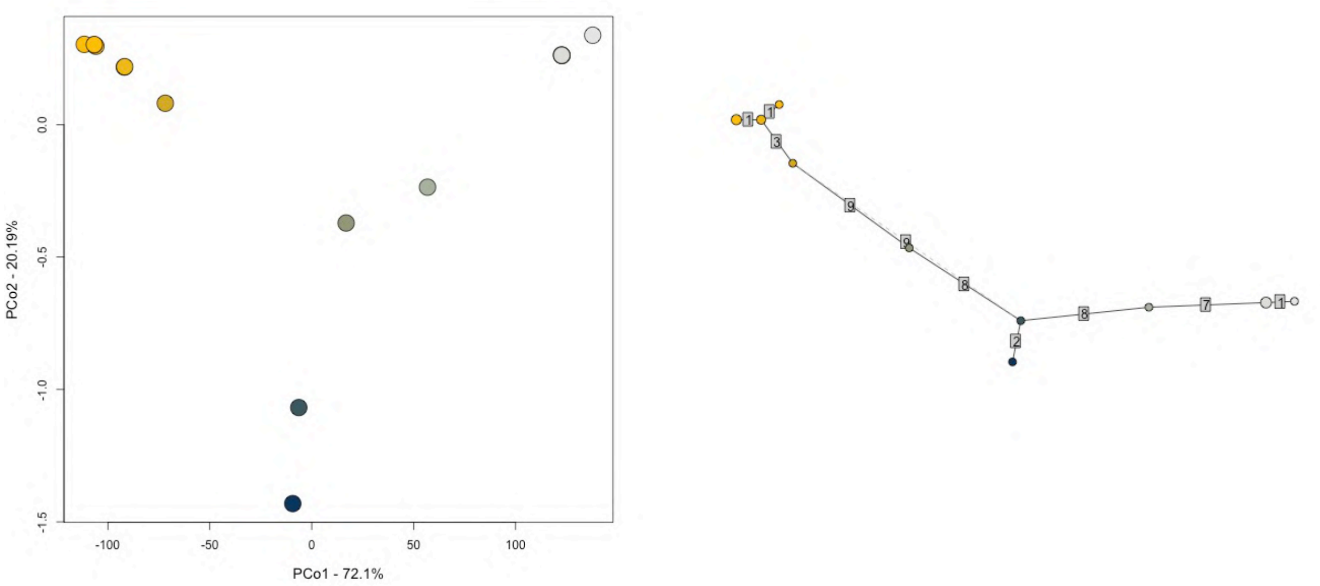

Figure 494: PCoA based on pairwise p-distances between *Myrmica specioides* sequences (left). Colours match a bidimensional colour space. Haplotype network of *Myrmica specioides* (right). Sequences > 599 bp: ID = 16, cf. = 0.

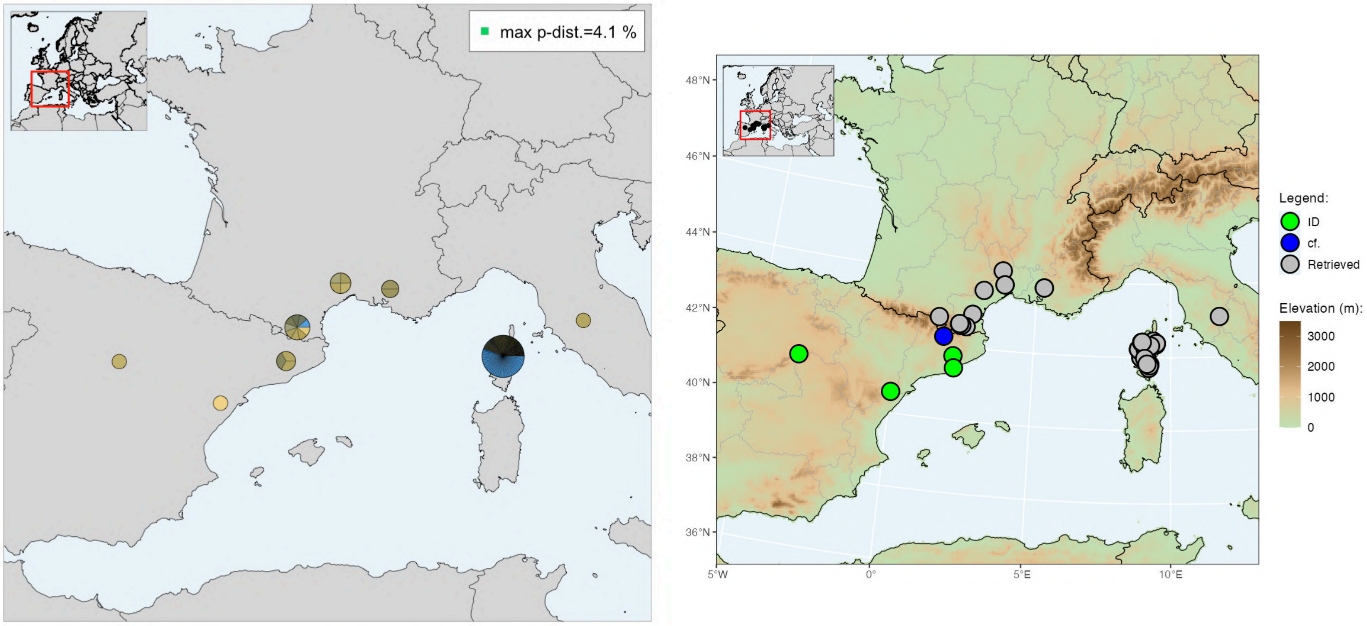

Figure 495: Genetic diversity map of *Myrmica spinosior* Santschi, 1931. Nearby localities of sequenced specimens are merged in pies (left). Colours match the bidimensional colour space of the PCoA projection (Fig. 495 left) of p-dist between sequences (dots). Specimen identification (ID or cf.) and source (newly sequenced or retrieved) are represented by colours, while specimen attribute (terra typica, type locality, type specimen or faunistic novelty) is represented by the shape (right). Sequences: ID = 92, cf. = 1; maximum p-distance: strict = 4.1 %, less strict = 4.1 %.

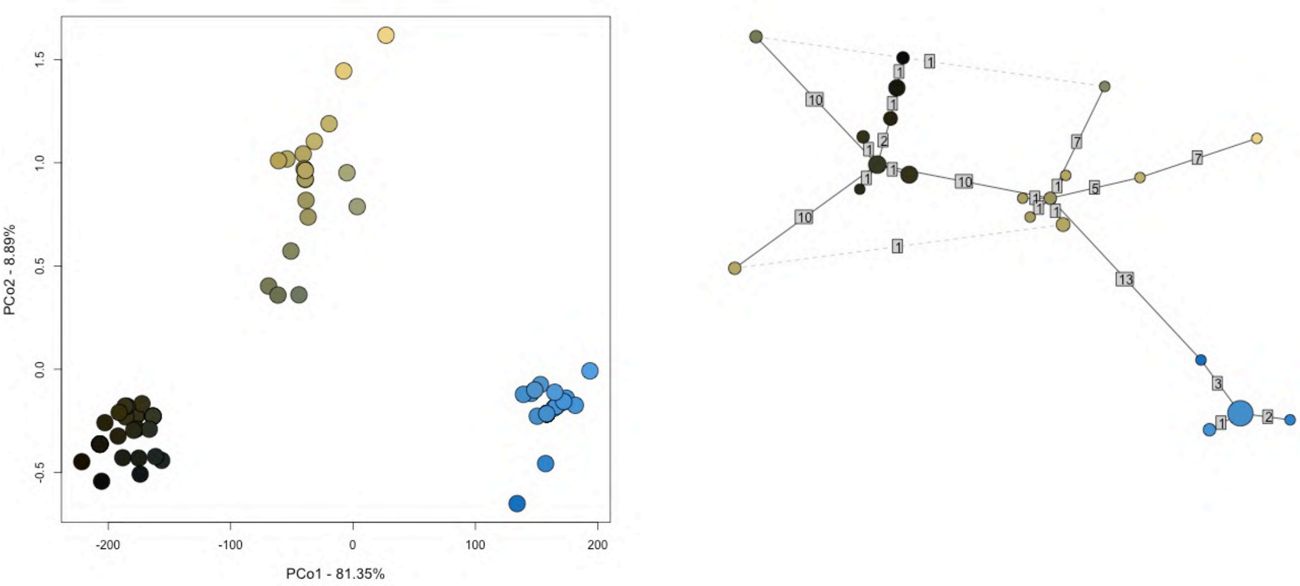

Figure 496: PCoA based on pairwise p-distances between *Myrmica spinosior* sequences (left). Colours match a bidimensional colour space. Haplotype network of *Myrmica spinosior* (right). Sequences > 599 bp: ID = 81, cf. = 1.

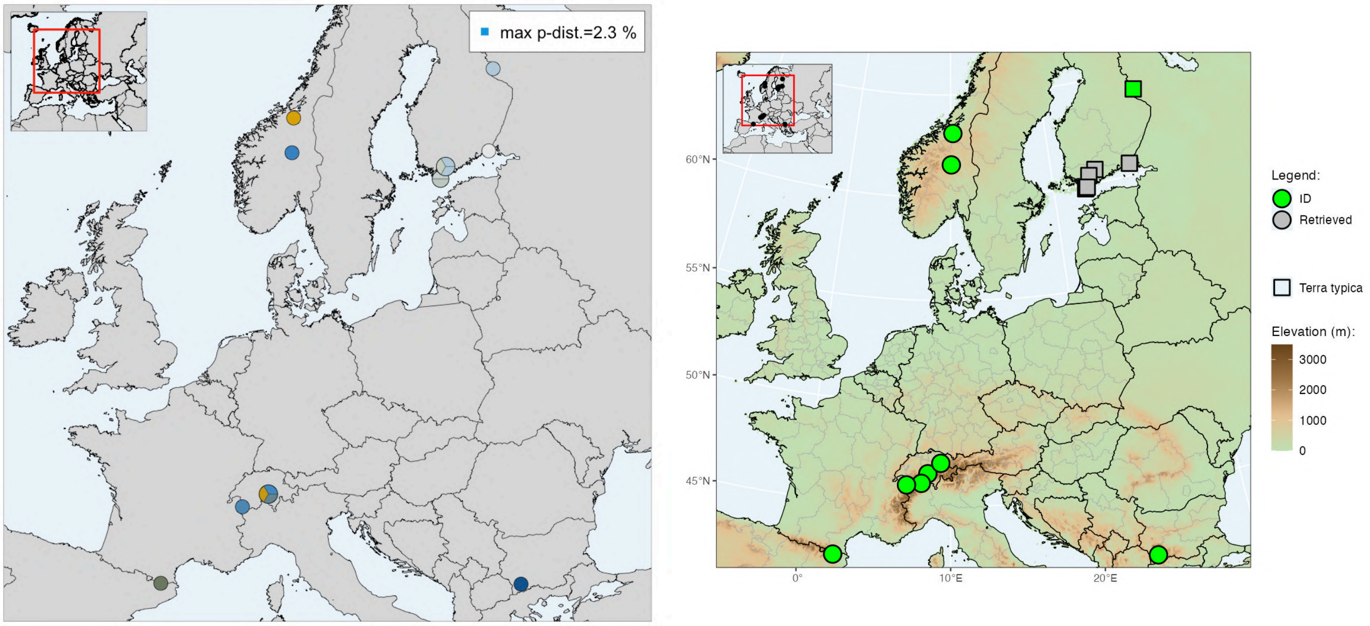

Figure 497: Genetic diversity map of *Myrmica sulcinodis* Nylander, 1846. Nearby localities of sequenced specimens are merged in pies (left). Colours match the bidimensional colour space of the PCoA projection (Fig. 497 left) of p-dist between sequences (dots). Specimen identification (ID or cf.) and source (newly sequenced or retrieved) are represented by colours, while specimen attribute (terra typica, type locality, type specimen or faunistic novelty) is represented by the shape (right). Sequences: ID = 15, cf. = 0; maximum p-distance: strict = 2.3 %, less strict = 2.3 %.

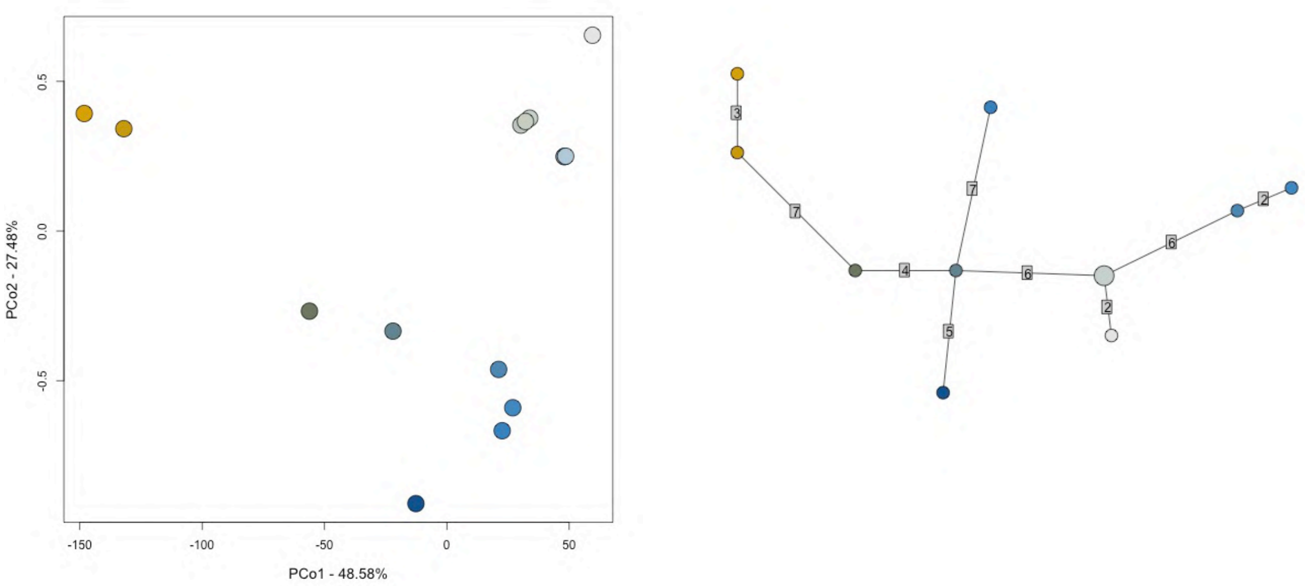

Figure 498: PCoA based on pairwise p-distances between *Myrmica sulcinodis* sequences (left). Colours match a bidimensional colour space. Haplotype network of *Myrmica sulcinodis* (right). Sequences > 599 bp: ID = 15, cf. = 0.

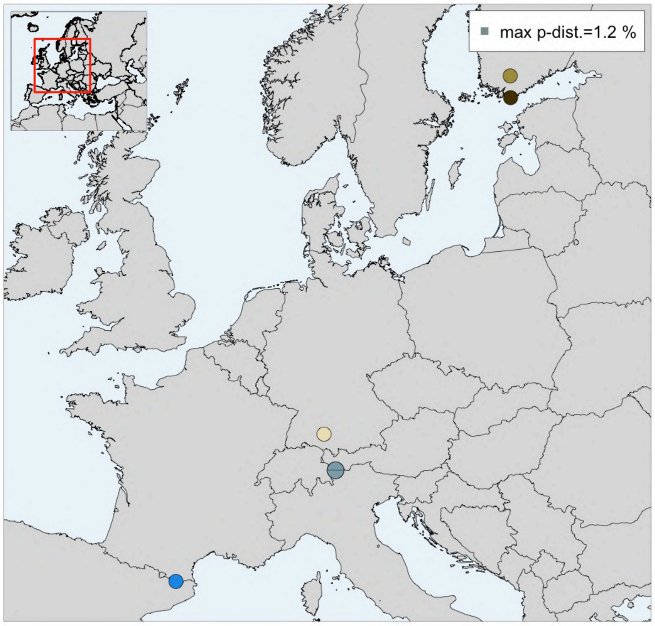

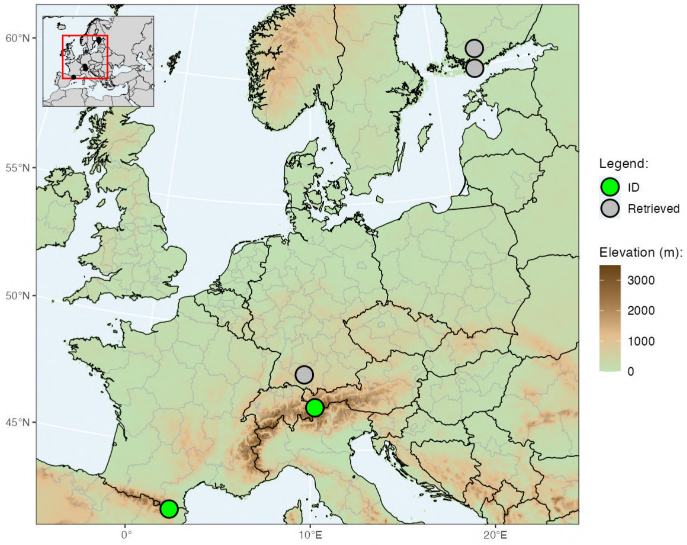

Figure 499: Genetic diversity map of *Myrmica vandeli* Bondroit, 1920. Nearby localities of sequenced specimens are merged in pies (left). Colours match the bidimensional colour space of the PCoA projection (Fig. 499 left) of p-dist between sequences (dots). Specimen identification (ID or cf.) and source (newly sequenced or retrieved) are represented by colours, while specimen attribute (terra typica, type locality, type specimen or faunistic novelty) is represented by the shape (right). Sequences: ID = 6, cf. = 0; maximum p-distance: strict = 1.2 %, less strict = 1.2 %.

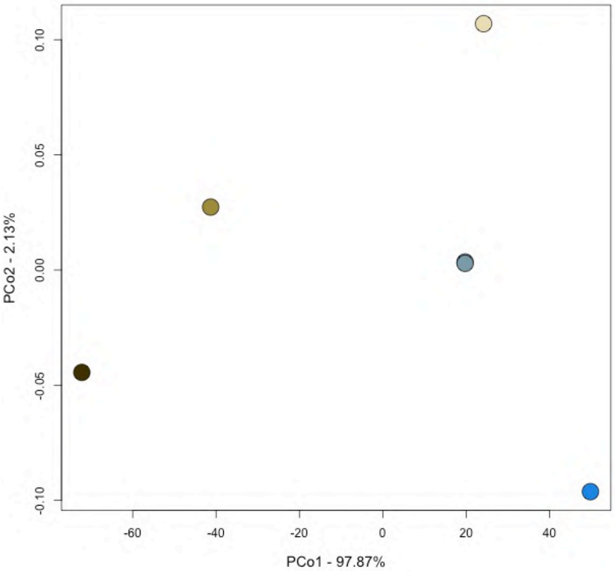

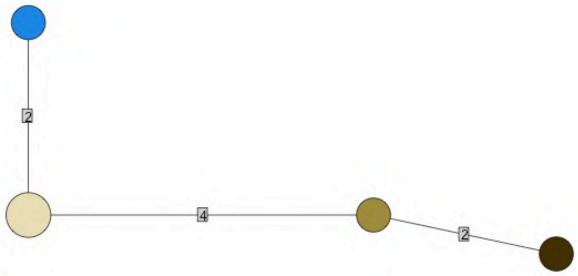

Figure 500: PCoA based on pairwise p-distances between *Myrmica vandeli* sequences (left). Colours match a bidimensional colour space. Haplotype network of *Myrmica vandeli* (right). Sequences > 599 bp: ID = 6, cf. = 0.

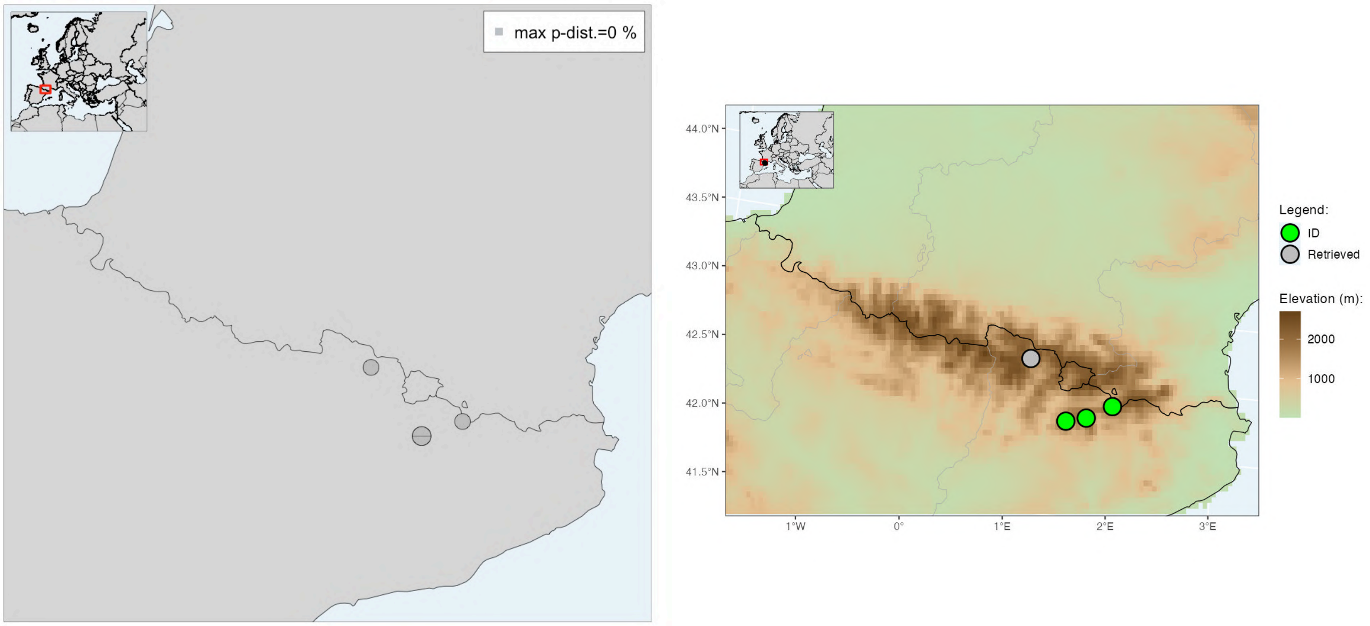

Figure 501: Genetic diversity map of *Myrmica wesmaeli* Bondroit, 1918. PCoA projection was not done and therefore sequenced specimens in the genetic diversity map are coloured in gray (left). Specimen identification (ID or cf.) and source (newly sequenced or retrieved) are represented by colours, while specimen attribute (terra typica, type locality, type specimen or faunistic novelty) is represented by the shape (right). Sequences: ID = 4, cf. = 0; maximum p-distance: strict = 0 %, less strict = 0 %.

Haplotype network analysis of *Myrmica wesmaeli* was not possible.

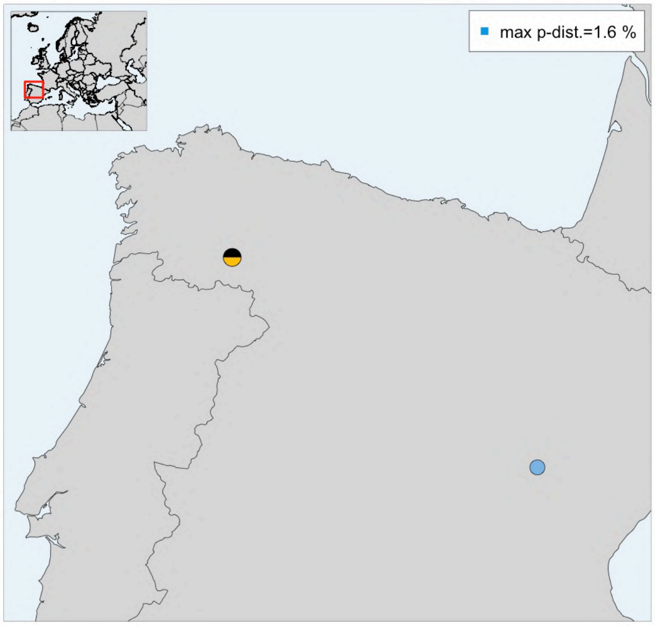

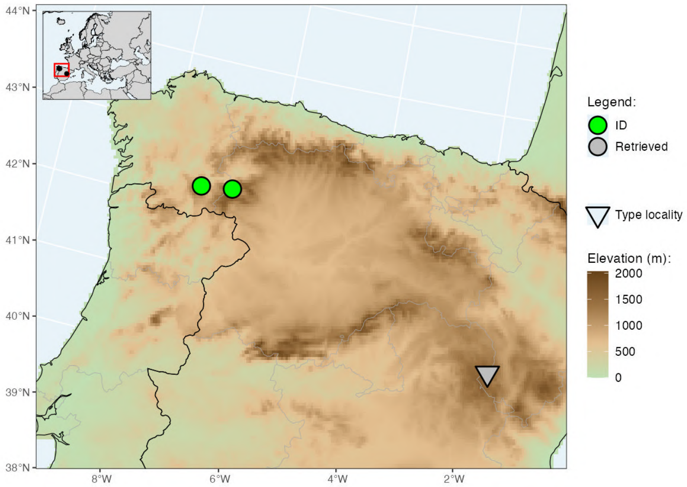

Figure 502: Genetic diversity map of *Myrmica xavieri* Radchenko, Elmes & Savolainen, 2008. Nearby localities of sequenced specimens are merged in pies (left). Colours match the bidimensional colour space of the PCoA projection (Fig. 502 left) of p-dist between sequences (dots). Specimen identification (ID or cf.) and source (newly sequenced or retrieved) are represented by colours, while specimen attribute (terra typica, type locality, type specimen or faunistic novelty) is represented by the shape (right). Sequences: ID = 3, cf. = 0; maximum p-distance: strict = 1.6 %, less strict = 1.6 %.

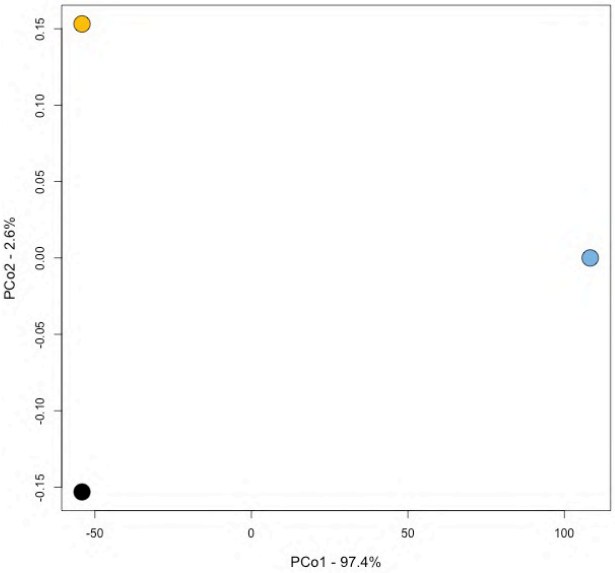

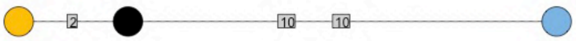

Figure 503: PCoA based on pairwise p-distances between *Myrmica xavieri* sequences (left). Colours match a bidimensional colour space. Haplotype network of *Myrmica xavieri* (right). Sequences > 599 bp: ID = 3, cf. = 0.

Nylanderia

Nylanderia flavipes

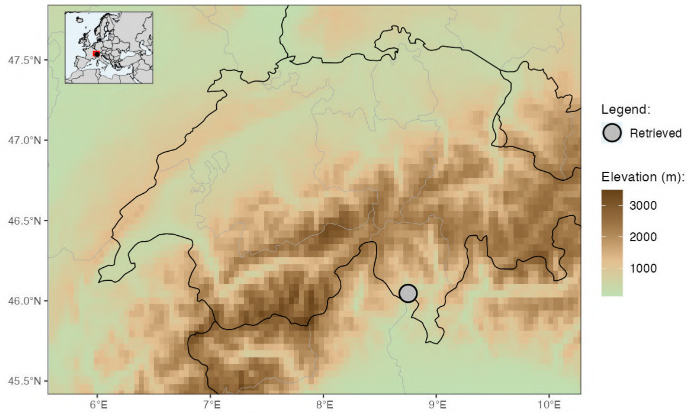

Figure 504: Map of *Nylanderia flavipes* (Smith, 1874). Due to the presence of a single sequence, the genetic diversity map and the PCoA projection were not done. Specimen identification (ID or cf.) and source (newly sequenced or retrieved) are represented by colours, while specimen attribute (terra typica, type locality, type specimen or faunistic novelty) is represented by the shape. Sequences: ID = 1, cf. = 0; maximum p-distance: strict = NA, less strict = NA.

Haplotype network analysis of *Nylanderia flavipes* was not possible.

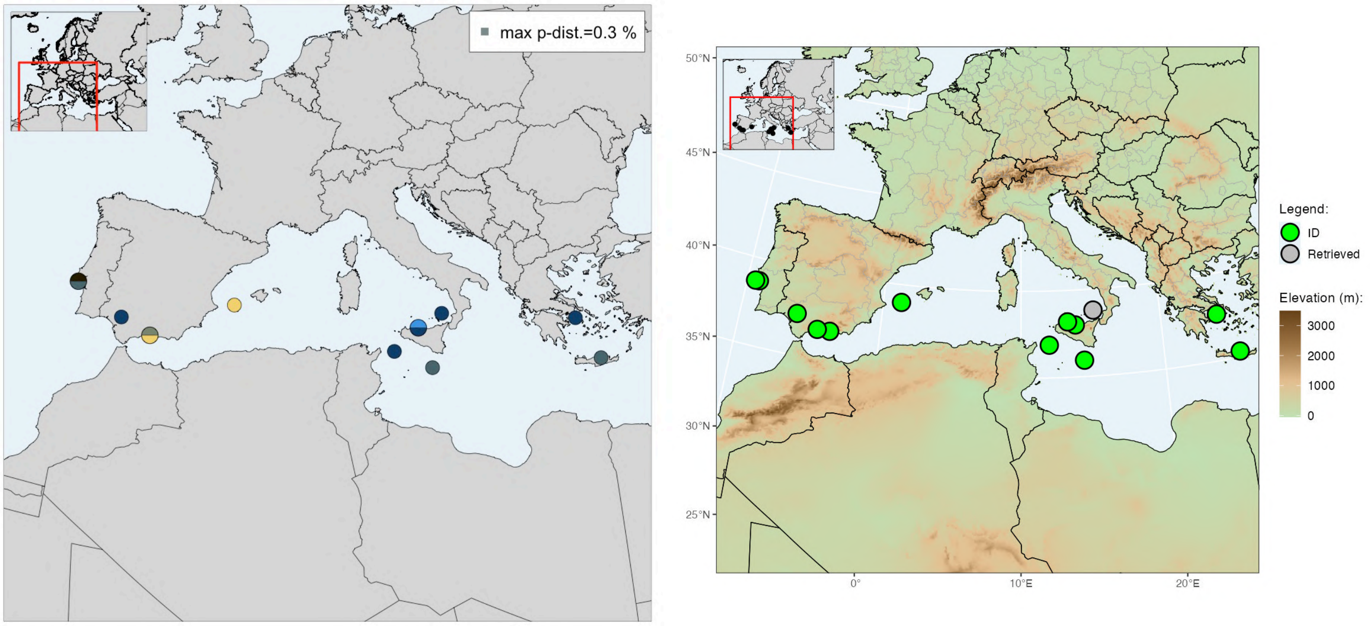

Figure 505: Genetic diversity map of *Nylanderia jaegerskioeldi* (Mayr, 1904). Nearby localities of sequenced specimens are merged in pies (left). Colours match the bidimensional colour space of the PCoA projection (Fig. 505 left) of p-dist between sequences (dots). Specimen identification (ID or cf.) and source (newly sequenced or retrieved) are represented by colours, while specimen attribute (terra typica, type locality, type specimen or faunistic novelty) is represented by the shape (right). Sequences: ID = 14, cf. = 0; maximum p-distance: strict = 0.3 %, less strict = 0.3 %.

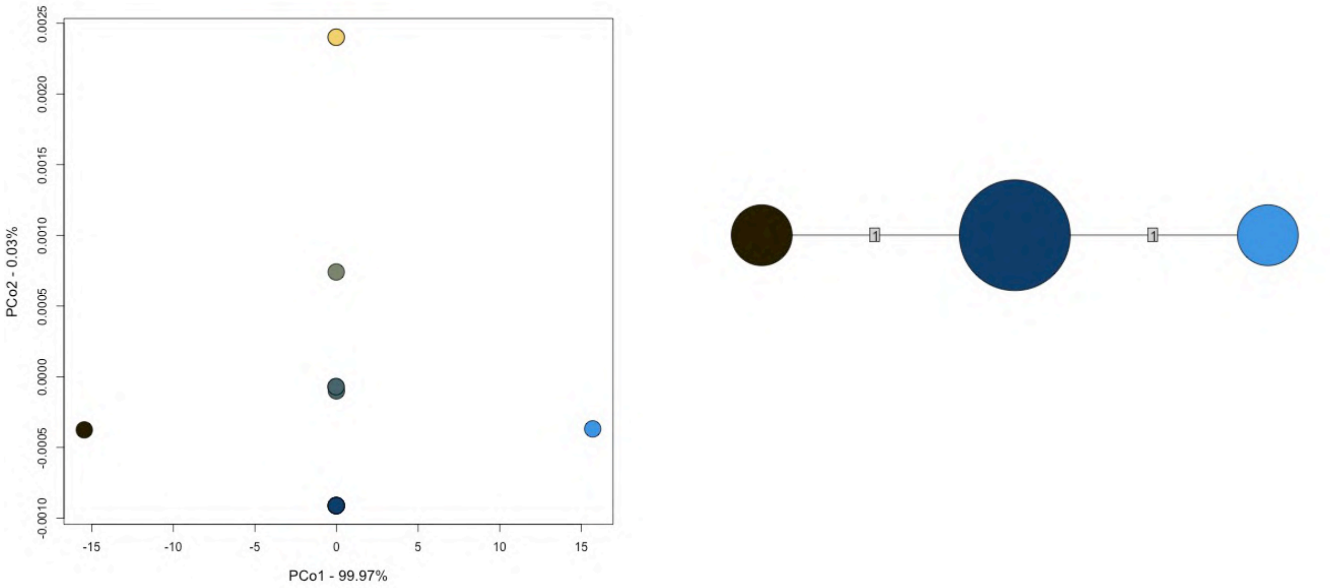

Figure 506: PCoA based on pairwise p-distances between *Nylanderia jaegerskioeldi* sequences (left). Colours match a bidimensional colour space. Haplotype network of *Nylanderia jaegerskioeldi* (right). Sequences > 599 bp: ID = 14, cf. = 0.

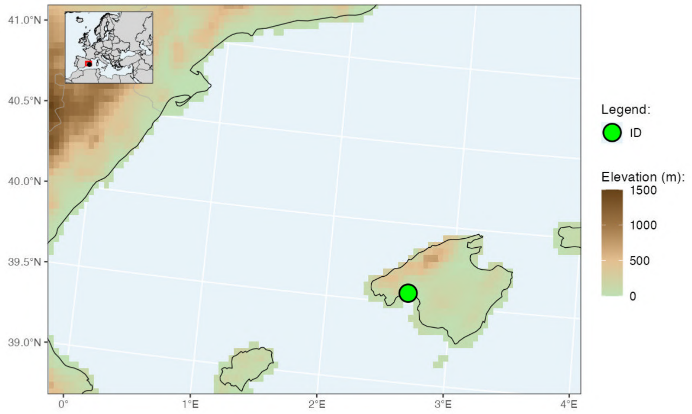

Figure 507: Map of *Nylanderia vividula* (Nylander, 1846). Due to the presence of a single sequence, the genetic diversity map and the PCoA projection were not done. Specimen identification (ID or cf.) and source (newly sequenced or retrieved) are represented by colours, while specimen attribute (terra typica, type locality, type specimen or faunistic novelty) is represented by the shape. Sequences: ID = 1, cf. = 0; maximum p-distance: strict = NA, less strict = NA.

Haplotype network analysis of *Nylanderia vividula* was not possible.

Oxyopomyrmex

Oxyopomyrmex krueperi

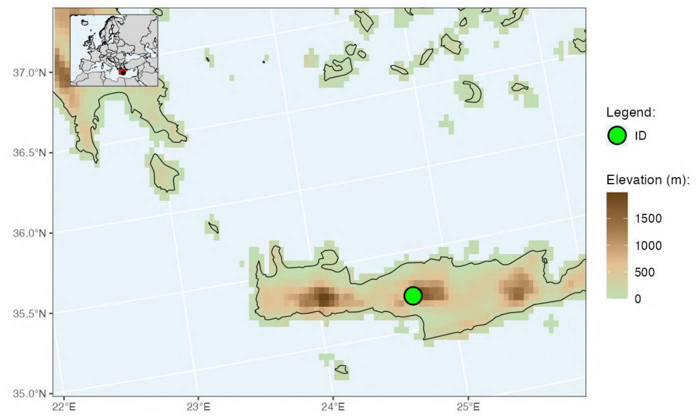

Figure 508: Map of *Oxyopomyrmex krueperi* Forel, 1911. Due to the presence of a single sequence, the genetic diversity map and the PCoA projection were not done. Specimen identification (ID or cf.) and source (newly sequenced or retrieved) are represented by colours, while specimen attribute (terra typica, type locality, type specimen or faunistic novelty) is represented by the shape. Sequences: ID = 1, cf. = 0; maximum p-distance: strict = NA, less strict = NA.

Haplotype network analysis of *Oxyopomyrmex krueperi* was not possible.

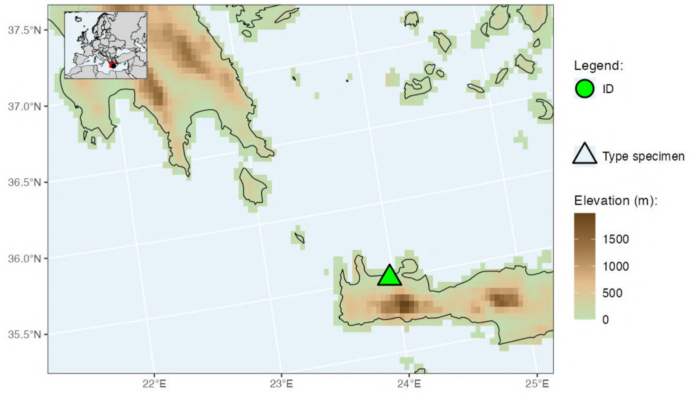

Figure 509: Map of *Oxyopomyrmex laevibus* Salata & Borowiec, 2015. Due to the presence of a single sequence, the genetic diversity map and the PCoA projection were not done. Specimen identification (ID or cf.) and source (newly sequenced or retrieved) are represented by colours, while specimen attribute (terra typica, type locality, type specimen or faunistic novelty) is represented by the shape. Sequences: ID = 1, cf. = 0; maximum p-distance: strict = NA, less strict = NA.

Haplotype network analysis of *Oxyopomyrmex laevibus* was not possible.

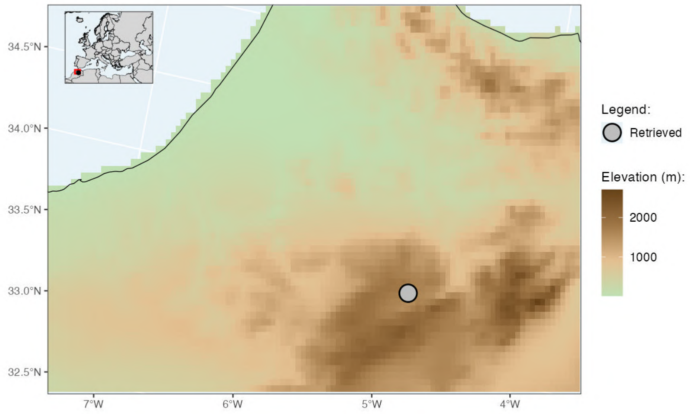

Figure 510: Map of *Oxyopomyrmex magnus* Salata & Borowiec, 2015. Due to the presence of a single sequence, the genetic diversity map and the PCoA projection were not done. Specimen identification (ID or cf.) and source (newly sequenced or retrieved) are represented by colours, while specimen attribute (terra typica, type locality, type specimen or faunistic novelty) is represented by the shape. Sequences: ID = 1, cf. = 0; maximum p-distance: strict = NA, less strict = NA.

Haplotype network analysis of *Oxyopomyrmex magnus* was not possible.

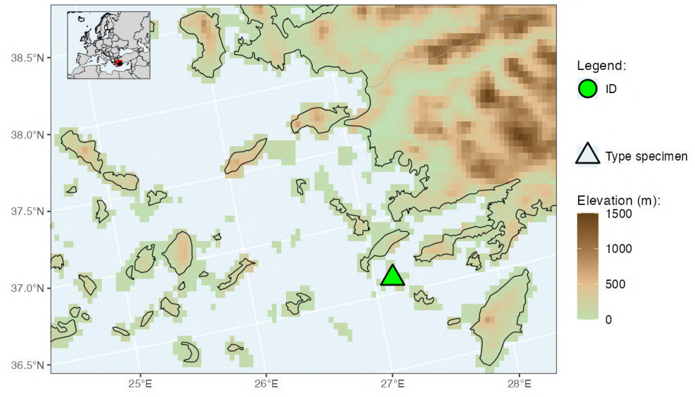

Figure 511: Map of *Oxyopomyrmex polybotesi* Salata & Borowiec, 2015. Due to the presence of a single sequence, the genetic diversity map and the PCoA projection were not done. Specimen identification (ID or cf.) and source (newly sequenced or retrieved) are represented by colours, while specimen attribute (terra typica, type locality, type specimen or faunistic novelty) is represented by the shape. Sequences: ID = 1, cf. = 0; maximum p-distance: strict = NA, less strict = NA.

Haplotype network analysis of *Oxyopomyrmex polybotesi* was not possible.

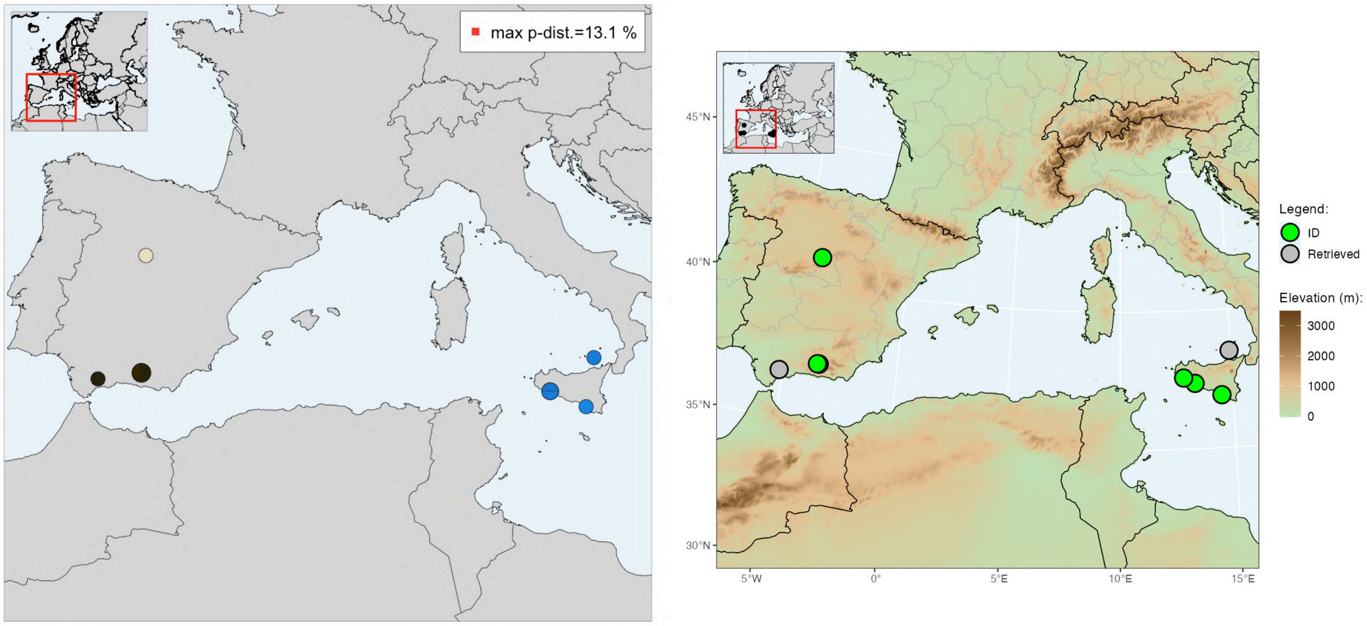

Figure 512: Genetic diversity map of *Oxyopomyrmex saulcyi* Emery, 1889. Nearby localities of sequenced specimens are merged in pies (left). Colours match the bidimensional colour space of the PCoA projection (Fig. 512 left) of p-dist between sequences (dots). Specimen identification (ID or cf.) and source (newly sequenced or retrieved) are represented by colours, while specimen attribute (terra typica, type locality, type specimen or faunistic novelty) is represented by the shape (right). Sequences: ID = 9, cf. = 0; maximum p-distance: strict = 13.1 %, less strict = 13.1 %.

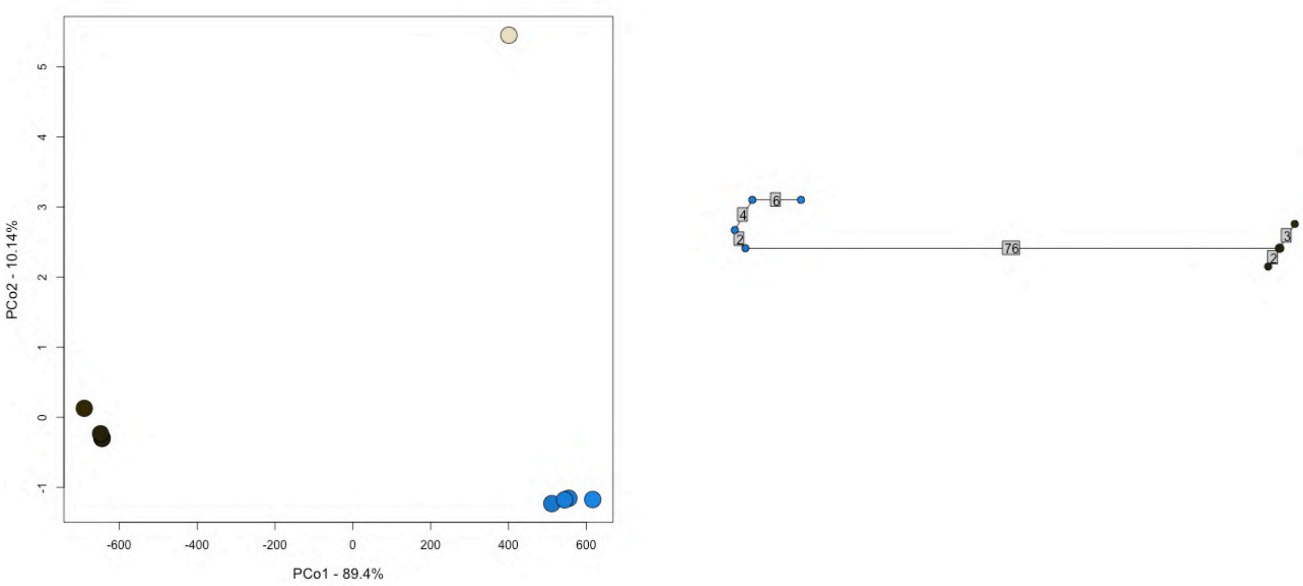

Figure 513: PCoA based on pairwise p-distances between *Oxyopomyrmex saulcyi* sequences (left). Colours match a bidimensional colour space. Haplotype network of *Oxyopomyrmex saulcyi* (right). Sequences > 599 bp: ID = 8, cf. = 0.

Paratrechina

Paratrechina longicornis

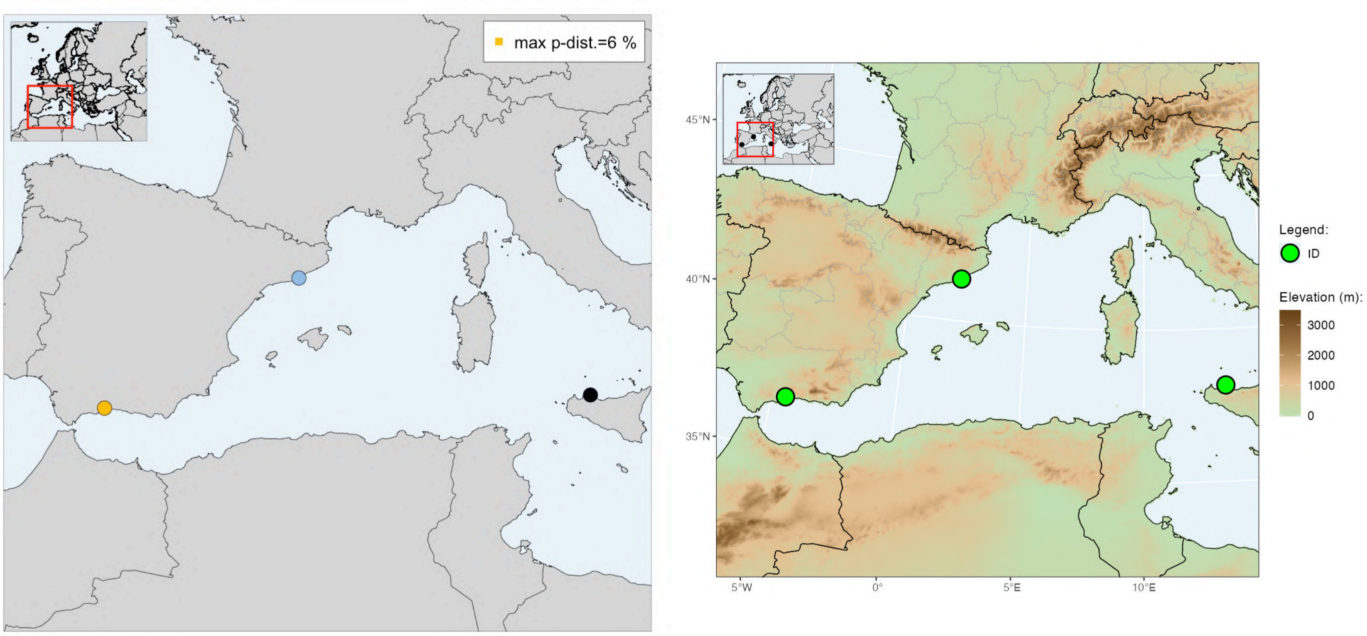

Figure 514: Genetic diversity map of *Paratrechina longicornis* (Latreille, 1802). Nearby localities of sequenced specimens are merged in pies (left). Colours match the bidimensional colour space of the PCoA projection (Fig. 514 left) of p-dist between sequences (dots). Specimen identification (ID or cf.) and source (newly sequenced or retrieved) are represented by colours, while specimen attribute (terra typica, type locality, type specimen or faunistic novelty) is represented by the shape (right). Sequences: ID = 6, cf. = 0; maximum p-distance: strict = 6 %, less strict = 6 %.

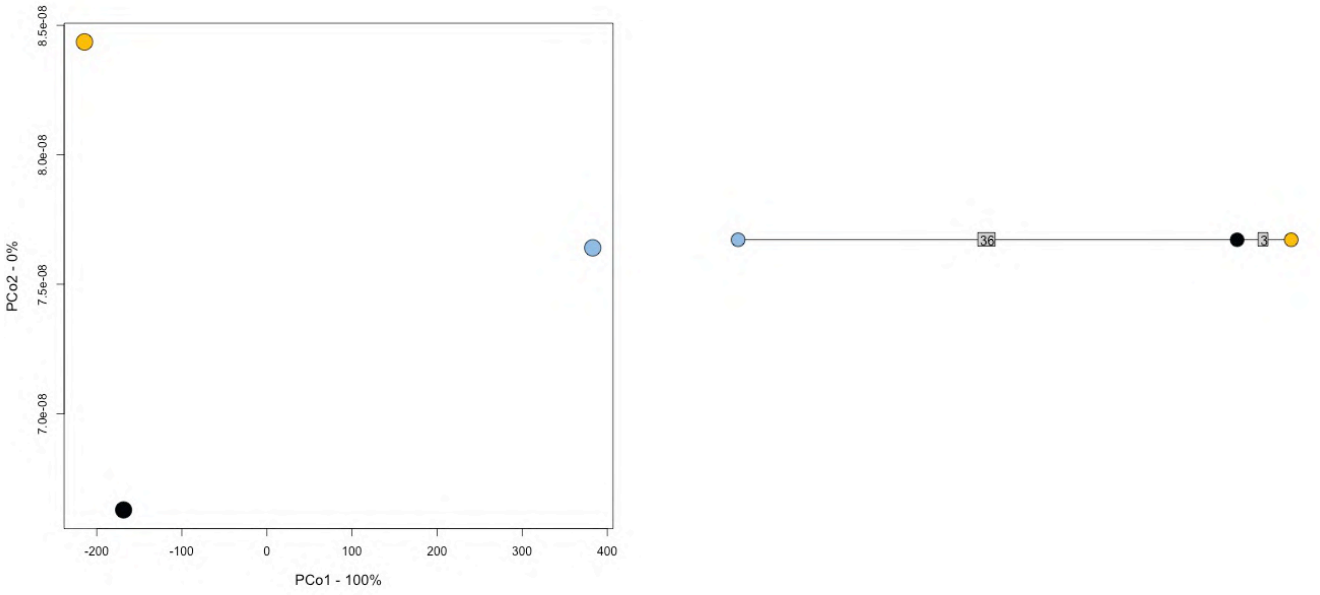

Figure 515: PCoA based on pairwise p-distances between *Paratrechina longicornis* sequences (left). Colours match a bidimensional colour space. Haplotype network of *Paratrechina longicornis* (right). Sequences > 599 bp: ID = 6, cf. = 0.

Pheidole

Pheidole balcanica

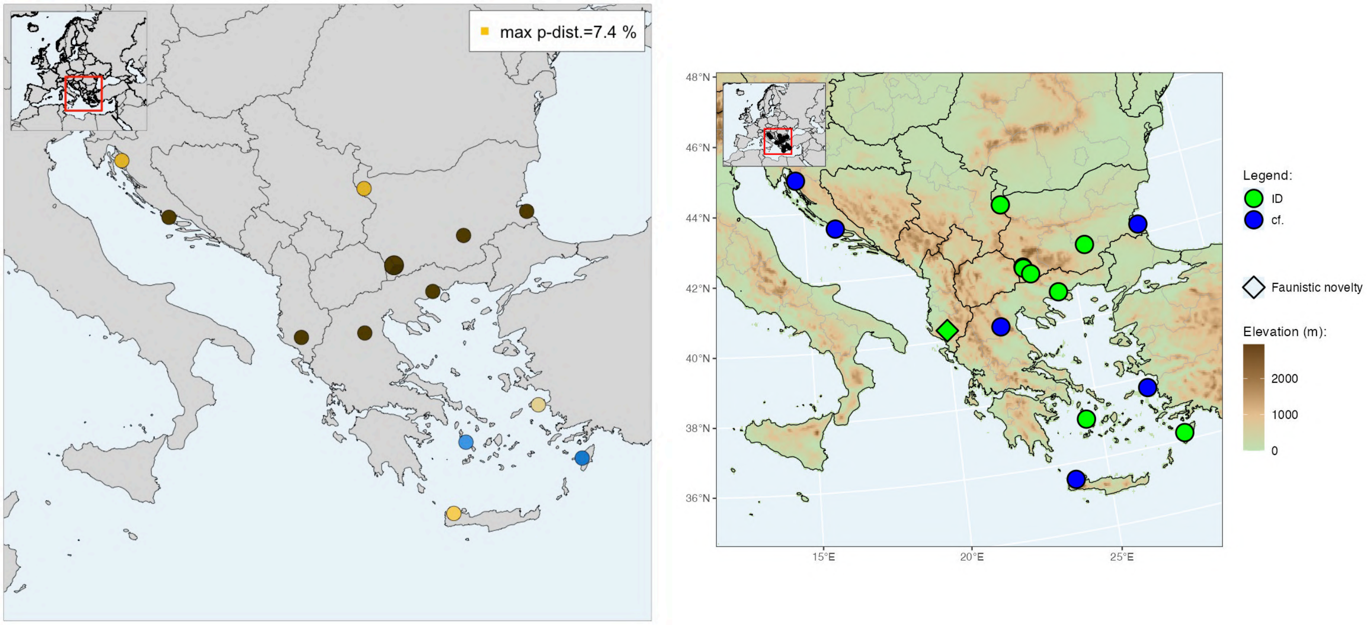

Figure 516: Genetic diversity map of *Pheidole balcanica* Seifert, 2016. Nearby localities of sequenced specimens are merged in pies (left). Colours match the bidimensional colour space of the PCoA projection (Fig. 516 left) of p-dist between sequences (dots). Specimen identification (ID or cf.) and source (newly sequenced or retrieved) are represented by colours, while specimen attribute (terra typica, type locality, type specimen or faunistic novelty) is represented by the shape (right). Sequences: ID = 9, cf. = 6; maximum p-distance: strict = 7.4 %, less strict = 7.4 %.

The species is reported for the first time in Albania.

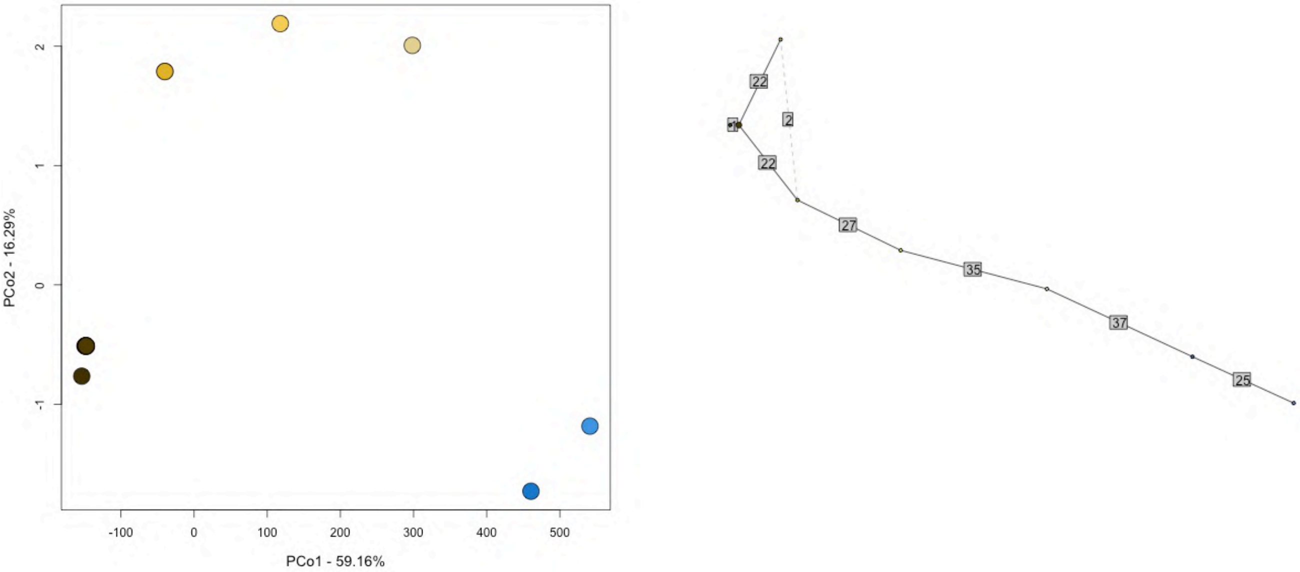

Figure 517: PCoA based on pairwise p-distances between *Pheidole balcanica* sequences (left). Colours match a bidimensional colour space. Haplotype network of *Pheidole balcanica* (right). Sequences > 599 bp: ID = 9, cf. = 6.

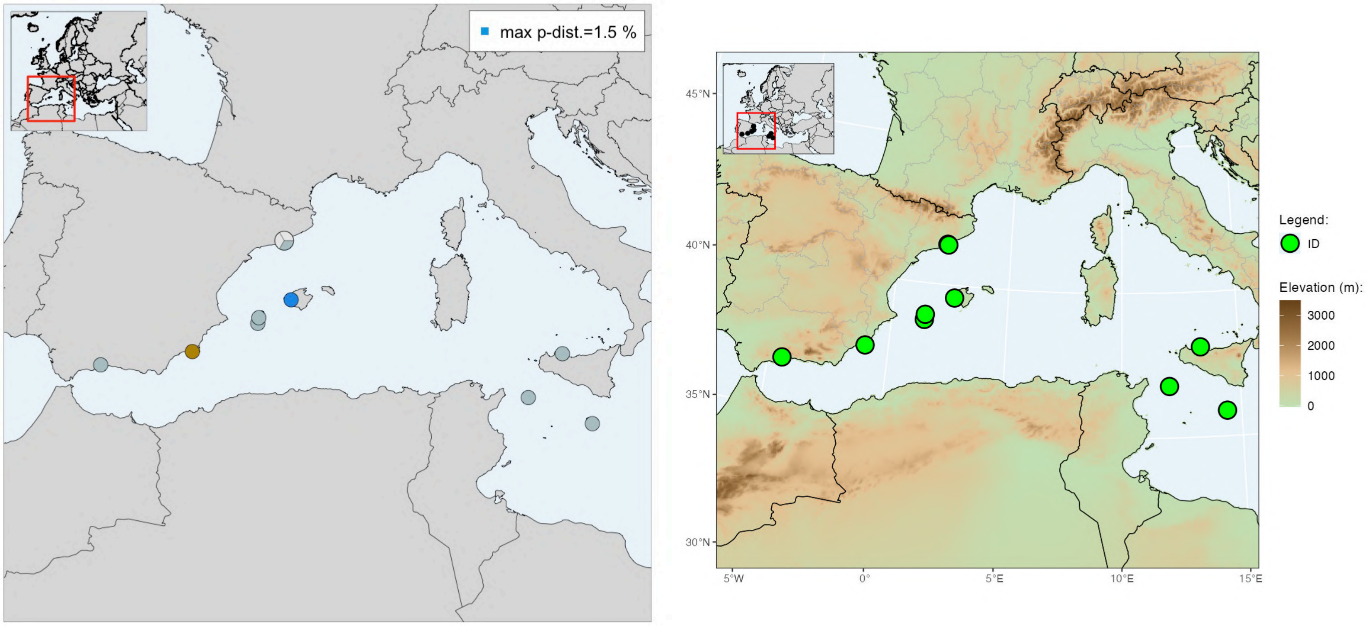

Figure 518: Genetic diversity map of *Pheidole indica* Mayr, 1879. Nearby localities of sequenced specimens are merged in pies (left). Colours match the bidimensional colour space of the PCoA projection (Fig. 518 left) of p-dist between sequences (dots). Specimen identification (ID or cf.) and source (newly sequenced or retrieved) are represented by colours, while specimen attribute (terra typica, type locality, type specimen or faunistic novelty) is represented by the shape (right). Sequences: ID = 13, cf. = 0; maximum p-distance: strict = 0.5 %, less strict = 1.5 %.

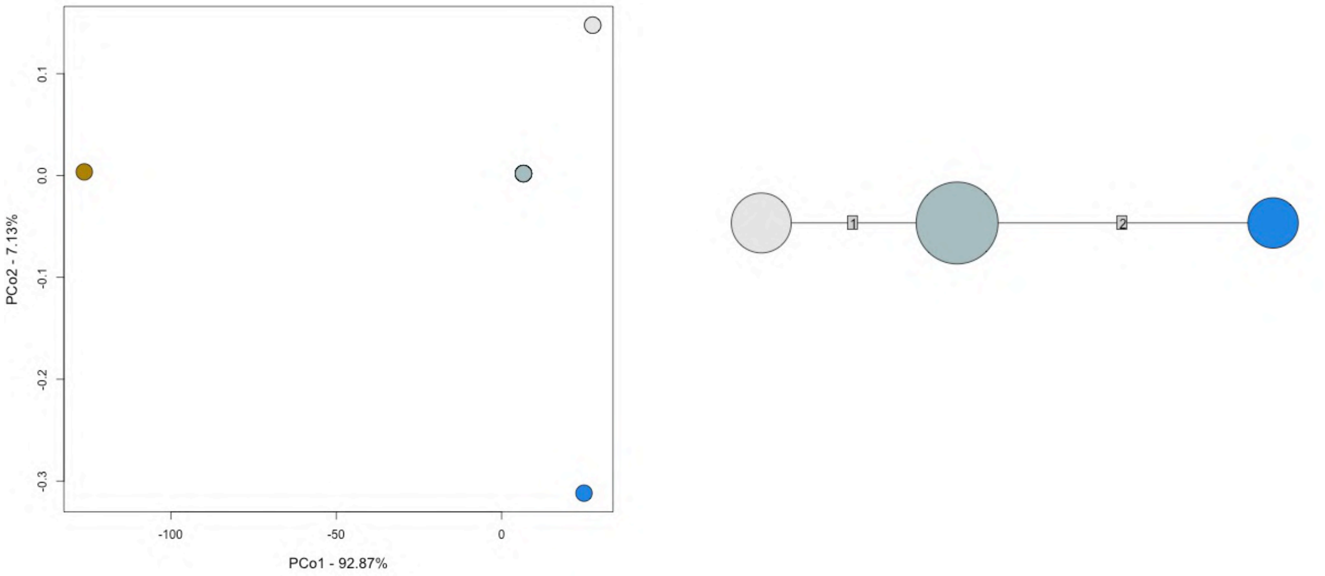

Figure 519: PCoA based on pairwise p-distances between *Pheidole indica* sequences (left). Colours match a bidimensional colour space. Haplotype network of *Pheidole indica* (right). Sequences > 599 bp: ID = 12, cf. = 0.

Pheidole cf. koshewnikovi

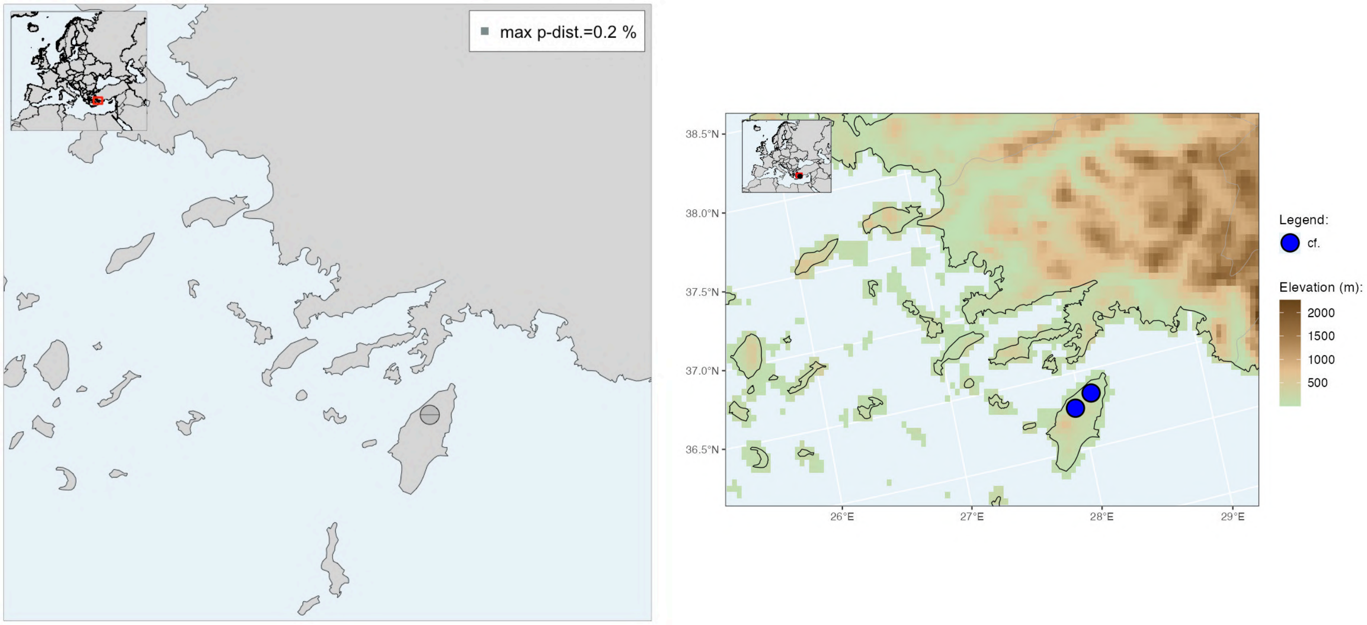

Figure 520: Genetic diversity map of *Pheidole cf. koshewnikovi* Ruzsky, 1905. PCoA projection was not done and therefore sequenced specimens in the genetic diversity map are coloured in gray (left). Specimen identification (ID or cf.) and source (newly sequenced or retrieved) are represented by colours, while specimen attribute (terra typica, type locality, type specimen or faunistic novelty) is represented by the shape (right). Sequences: ID = 0, cf. = 2; maximum p-distance: strict = NA, less strict = 0.2 %.

Haplotype network analysis of *Pheidole cf. koshewnikovi* was not possible.

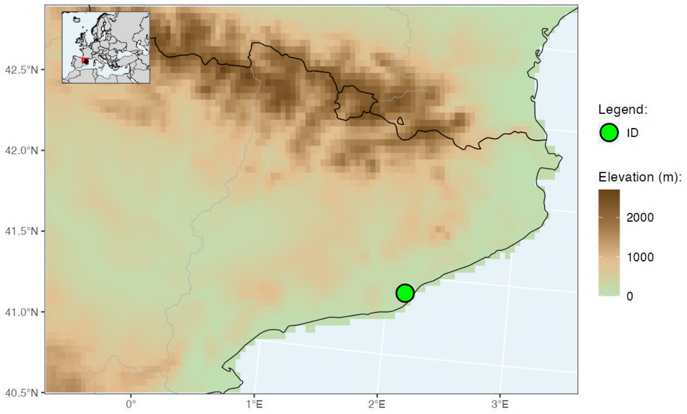

Figure 521: Map of *Pheidole megacephala* (Fabricius, 1793). Due to the presence of a single sequence, the genetic diversity map and the PCoA projection were not done. Specimen identification (ID or cf.) and source (newly sequenced or retrieved) are represented by colours, while specimen attribute (terra typica, type locality, type specimen or faunistic novelty) is represented by the shape. Sequences: ID = 4, cf. = 0; maximum p-distance: strict = NA, less strict = NA.

Haplotype network analysis of *Pheidole megacephala* was not possible.

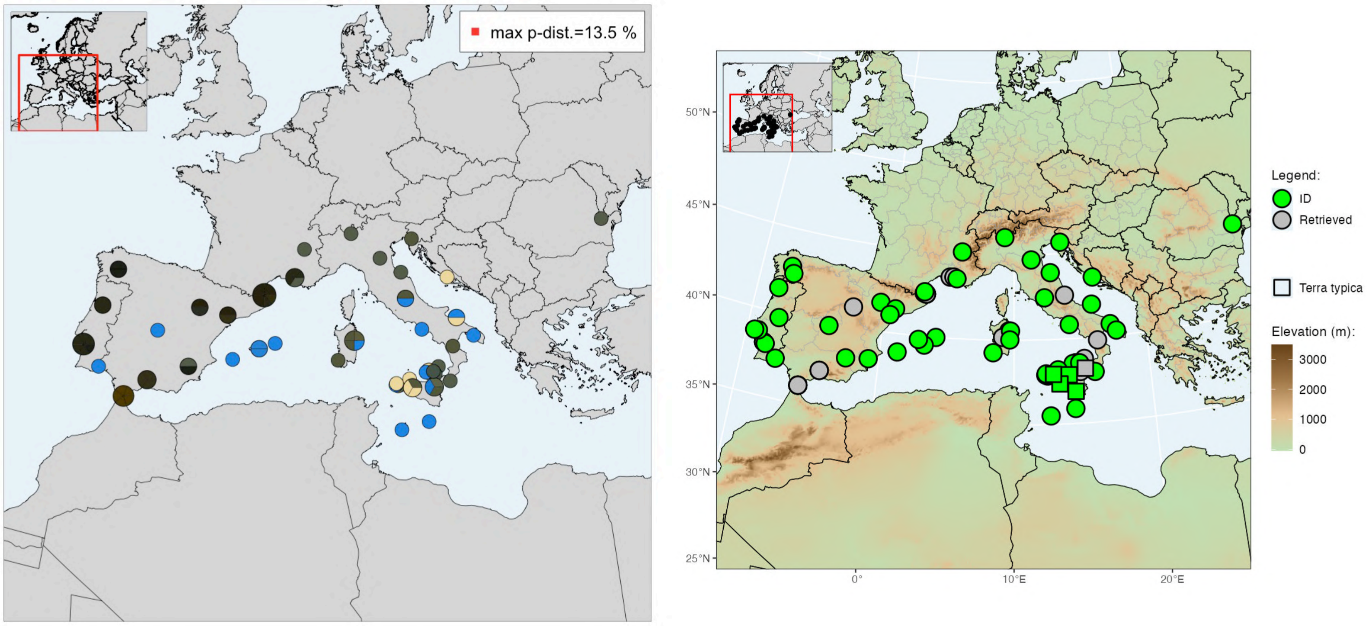

Figure 522: Genetic diversity map of *Pheidole pallidula* (Nylander, 1849). Nearby localities of sequenced specimens are merged in pies (left). Colours match the bidimensional colour space of the PCoA projection (Fig. 522 left) of p-dist between sequences (dots). Specimen identification (ID or cf.) and source (newly sequenced or retrieved) are represented by colours, while specimen attribute (terra typica, type locality, type specimen or faunistic novelty) is represented by the shape (right). Sequences: ID = NA, cf. = 0; maximum p-distance: strict = 13.5 %, less strict = 13.5 %.

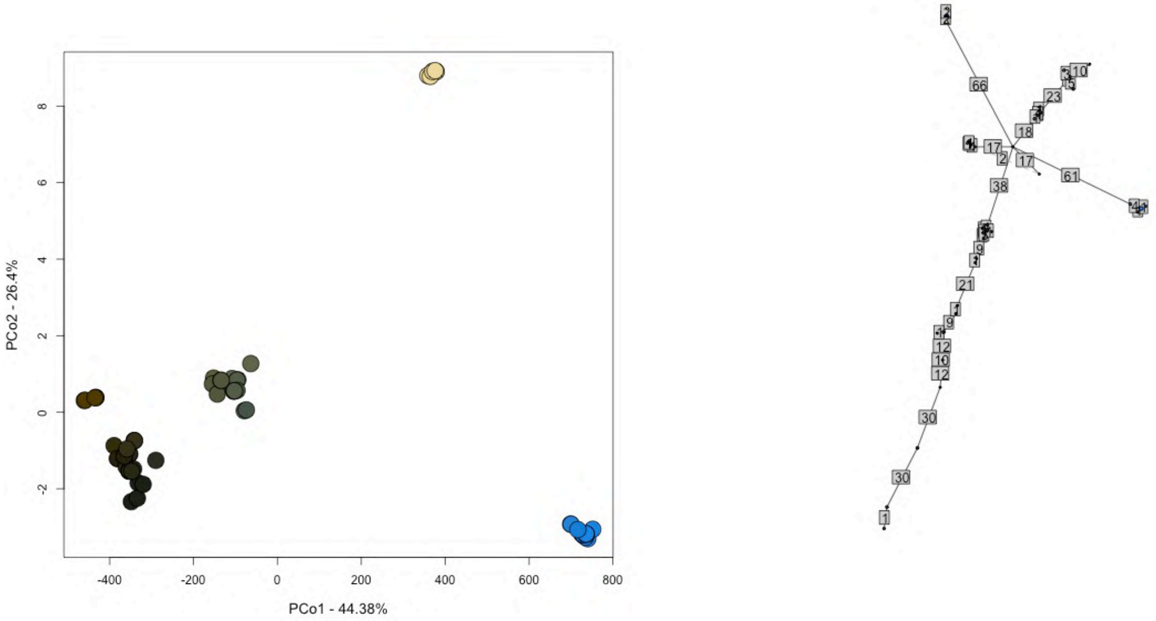

Figure 523: PCoA based on pairwise p-distances between *Pheidole pallidula* sequences (left). Colours match a bidimensional colour space. Haplotype network of *Pheidole pallidula* (right). Sequences > 599 bp: ID = NA, cf. = 0.

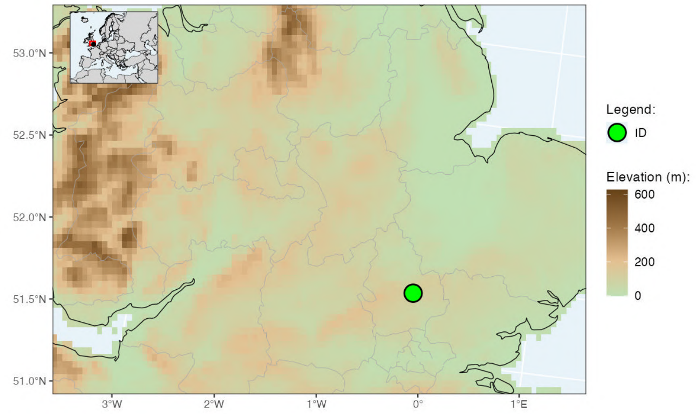

Figure 524: Map of *Pheidole punctatissima* Mayr, 1870. Due to the presence of a single sequence, the genetic diversity map and the PCoA projection were not done. Specimen identification (ID or cf.) and source (newly sequenced or retrieved) are represented by colours, while specimen attribute (terra typica, type locality, type specimen or faunistic novelty) is represented by the shape. Sequences: ID = 1, cf. = 0; maximum p-distance: strict = NA, less strict = NA.

Haplotype network analysis of *Pheidole punctatissima* was not possible.

Plagiolepis

Plagiolepis alluaudi

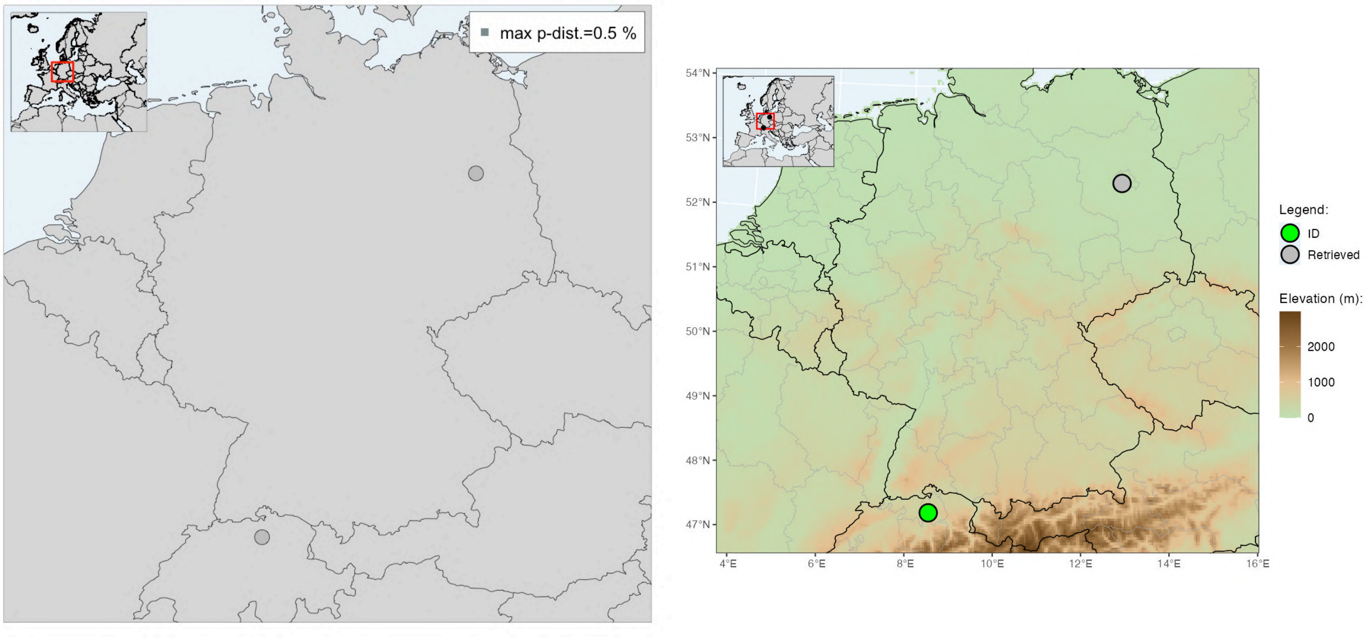

Figure 525: Genetic diversity map of *Plagiolepis alluaudi* Emery, 1894. PCoA projection was not done and therefore sequenced specimens in the genetic diversity map are coloured in gray (left). Specimen identification (ID or cf.) and source (newly sequenced or retrieved) are represented by colours, while specimen attribute (terra typica, type locality, type specimen or faunistic novelty) is represented by the shape (right). Sequences: ID = 2, cf. = 0; maximum p-distance: strict = NA, less strict = 0.5 %.

Haplotype network analysis of *Plagiolepis alluaudi* was not possible.

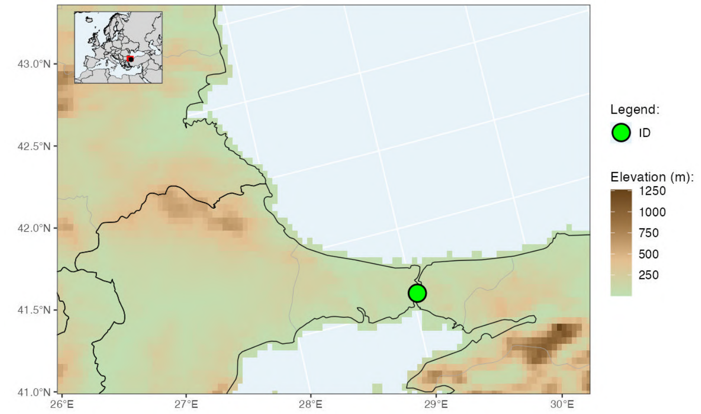

Figure 526: Map of *Plagiolepis atlantis* Santschi, 1920. Due to the presence of a single sequence, the genetic diversity map and the PCoA projection were not done. Specimen identification (ID or cf.) and source (newly sequenced or retrieved) are represented by colours, while specimen attribute (terra typica, type locality, type specimen or faunistic novelty) is represented by the shape. Sequences: ID = 1, cf. = 0; maximum p-distance: strict = NA, less strict = NA.

Haplotype network analysis of *Plagiolepis atlantis* was not possible.

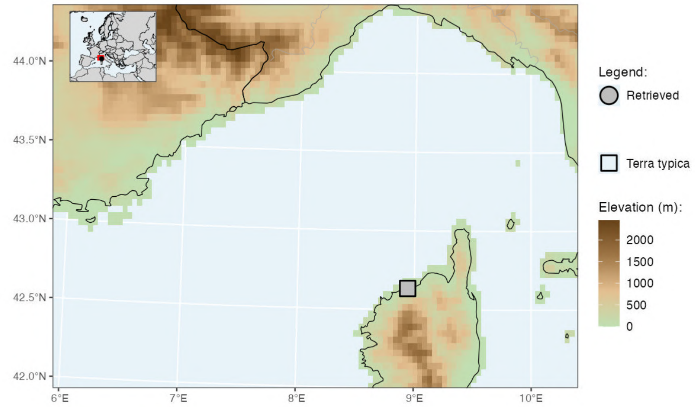

Figure 527: Map of *Plagiolepis delaugerrei* Casevitz-Weulersse, 2014. Due to the presence of a single sequence, the genetic diversity map and the PCoA projection were not done. Specimen identification (ID or cf.) and source (newly sequenced or retrieved) are represented by colours, while specimen attribute (terra typica, type locality, type specimen or faunistic novelty) is represented by the shape. Sequences: ID = 1, cf. = 0; maximum p-distance: strict = NA, less strict = NA.

Haplotype network analysis of *Plagiolepis delaugerrei* was not possible.

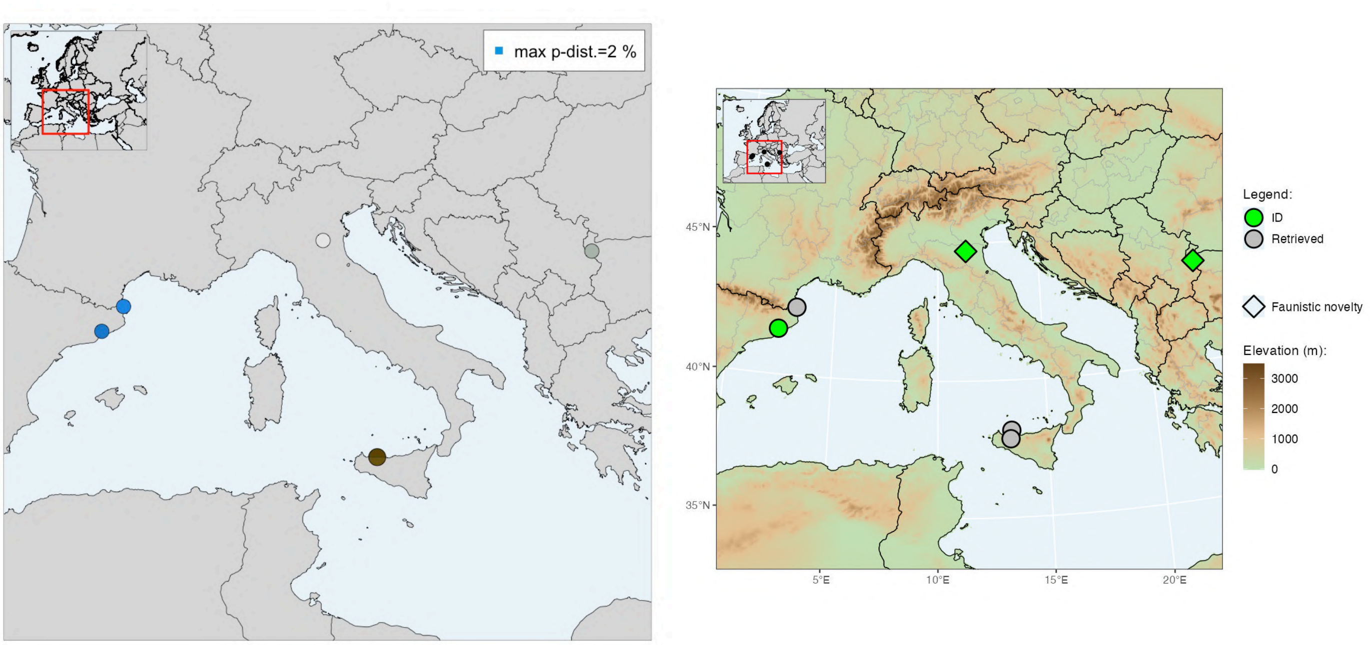

Figure 528: Genetic diversity map of *Plagiolepis grassei* Le Masne, 1956. Nearby localities of sequenced specimens are merged in pies (left). Colours match the bidimensional colour space of the PCoA projection (Fig. 528 left) of p-dist between sequences (dots). Specimen identification (ID or cf.) and source (newly sequenced or retrieved) are represented by colours, while specimen attribute (terra typica, type locality, type specimen or faunistic novelty) is represented by the shape (right). Sequences: ID = 6, cf. = 0; maximum p-distance: strict = 2 %, less strict = 2 %.

The species is reported for the first time in Albania.

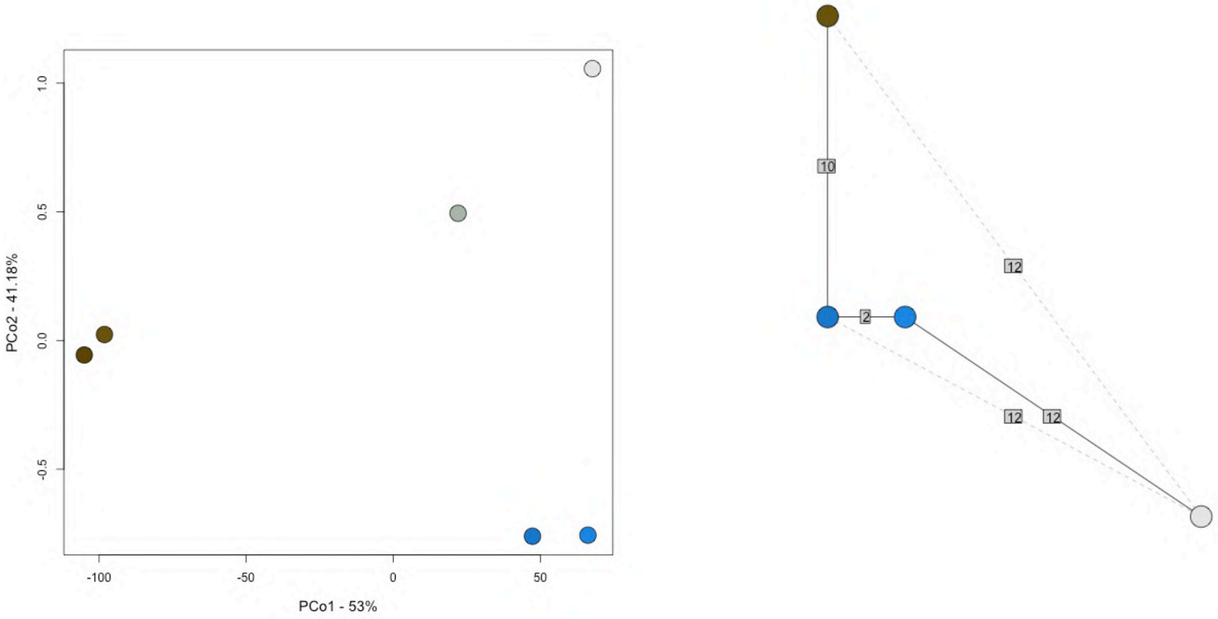

Figure 529: PCoA based on pairwise p-distances between *Plagiolepis grassei* sequences (left). Colours match a bidimensional colour space. Haplotype network of *Plagiolepis grassei* (right). Sequences > 599 bp: ID = 4, cf. = 0.

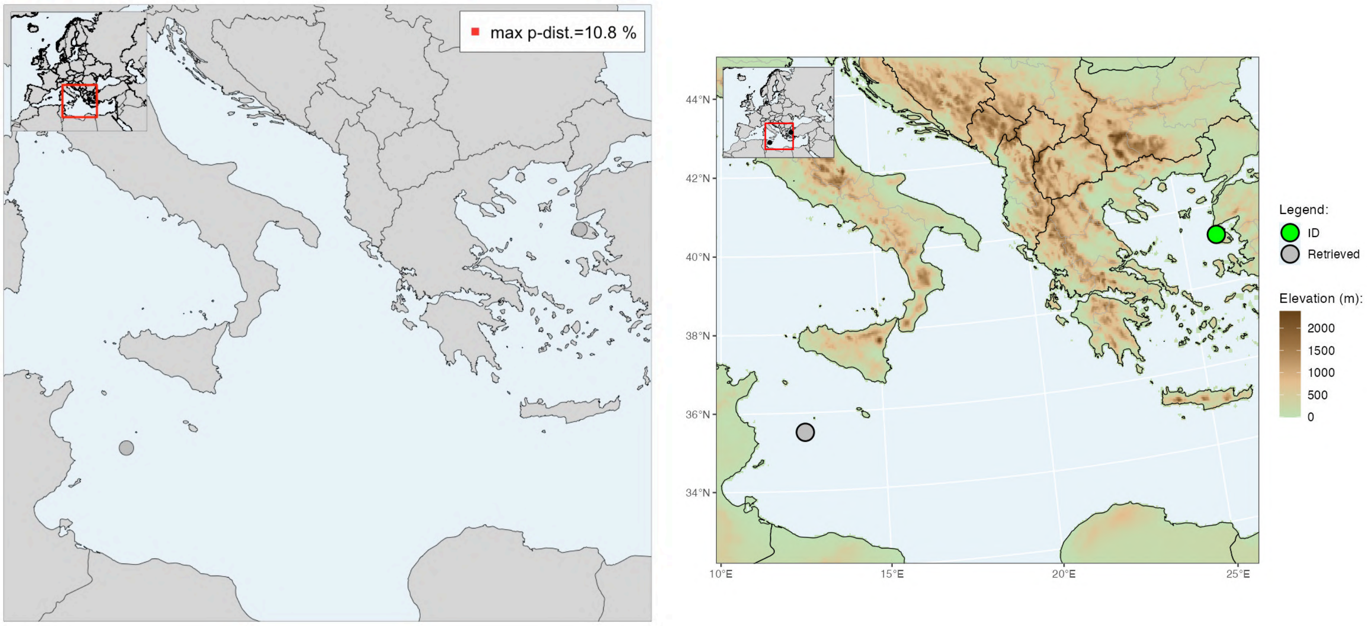

Figure 530: Genetic diversity map of *Plagiolepis pallescens* Forel, 1889. PCoA projection was not done and therefore sequenced specimens in the genetic diversity map are coloured in gray (left). Specimen identification (ID or cf.) and source (newly sequenced or retrieved) are represented by colours, while specimen attribute (terra typica, type locality, type specimen or faunistic novelty) is represented by the shape (right). Sequences: ID = 2, cf. = 0; maximum p-distance: strict = NA, less strict = 10.8 %.

Haplotype network analysis of *Plagiolepis pallescens* was not possible.

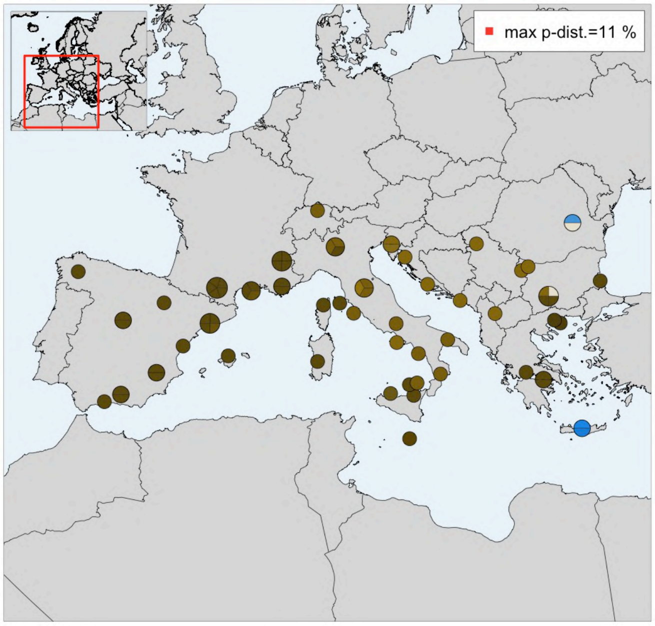

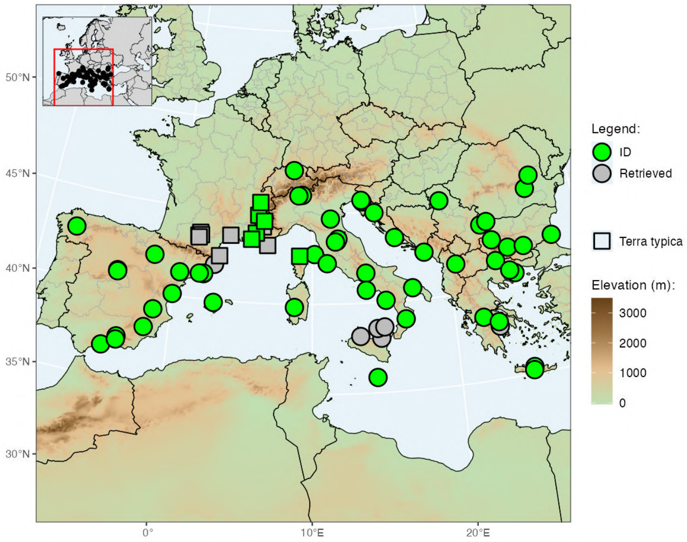

Figure 531: Genetic diversity map of *Plagiolepis pygmaea* (Latreille, 1798). Nearby localities of sequenced specimens are merged in pies (left). Colours match the bidimensional colour space of the PCoA projection (Fig. 531 left) of p-dist between sequences (dots). Specimen identification (ID or cf.) and source (newly sequenced or retrieved) are represented by colours, while specimen attribute (terra typica, type locality, type specimen or faunistic novelty) is represented by the shape (right). Sequences: ID = 73, cf. = 0; maximum p-distance: strict = 11 %, less strict = 11 %.

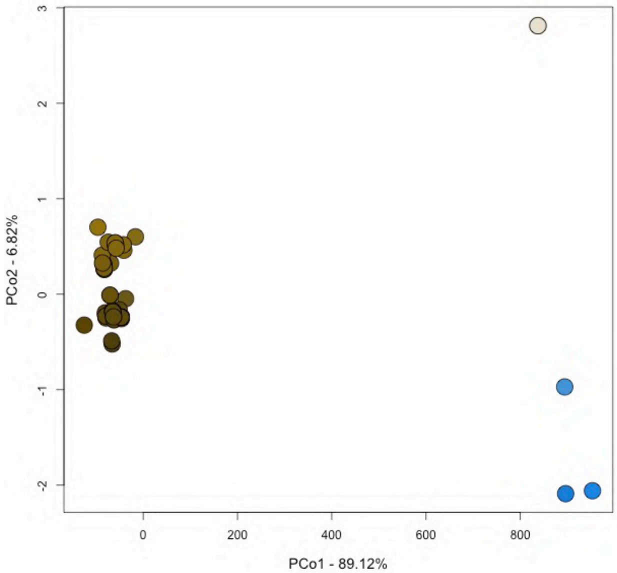

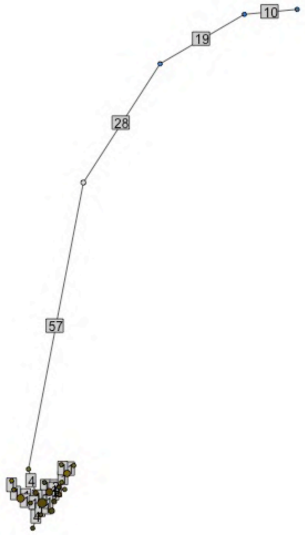

Figure 532: PCoA based on pairwise p-distances between *Plagiolepis pygmaea* sequences (left). Colours match a bidimensional colour space. Haplotype network of *Plagiolepis pygmaea* (right). Sequences > 599 bp: ID = 73, cf. = 0.

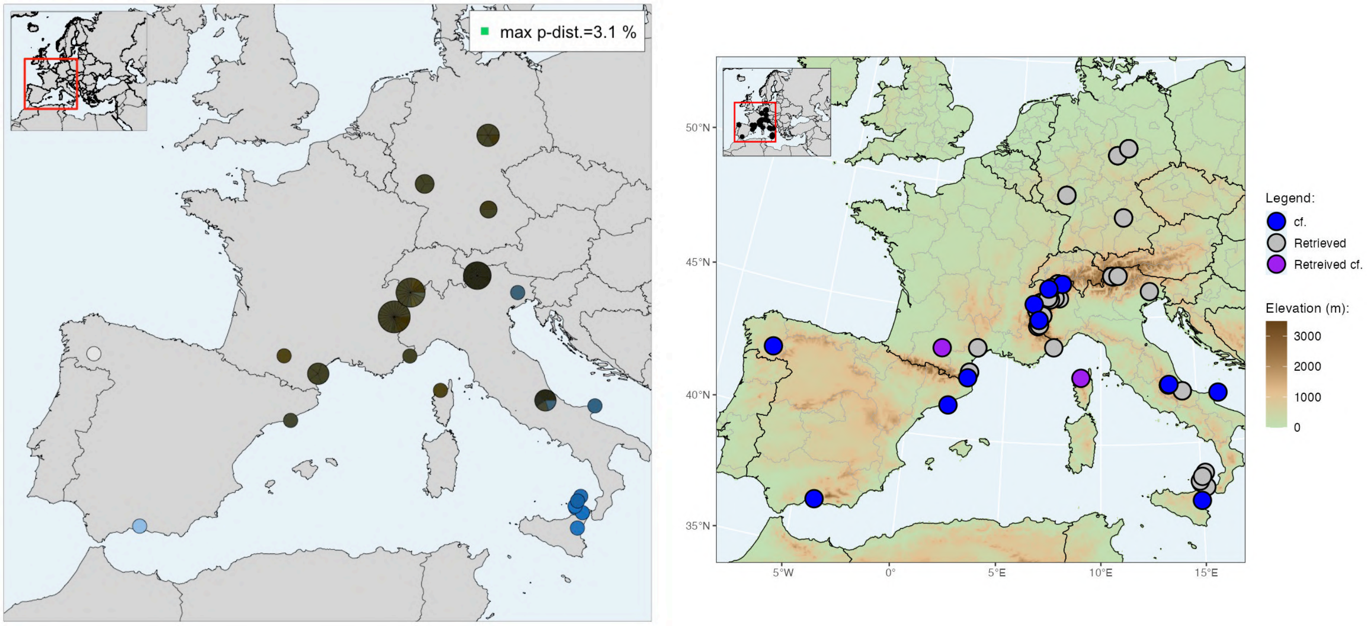

Figure 533: Genetic diversity map of *Plagiolepis pyrenaica* Emery, 1921. Nearby localities of sequenced specimens are merged in pies (left). Colours match the bidimensional colour space of the PCoA projection (Fig. 533 left) of p-dist between sequences (dots). Specimen identification (ID or cf.) and source (newly sequenced or retrieved) are represented by colours, while specimen attribute (terra typica, type locality, type specimen or faunistic novelty) is represented by the shape (right). Sequences: ID = 77, cf. = 14; maximum p-distance: strict = 2.3 %, less strict = 3.1 %.

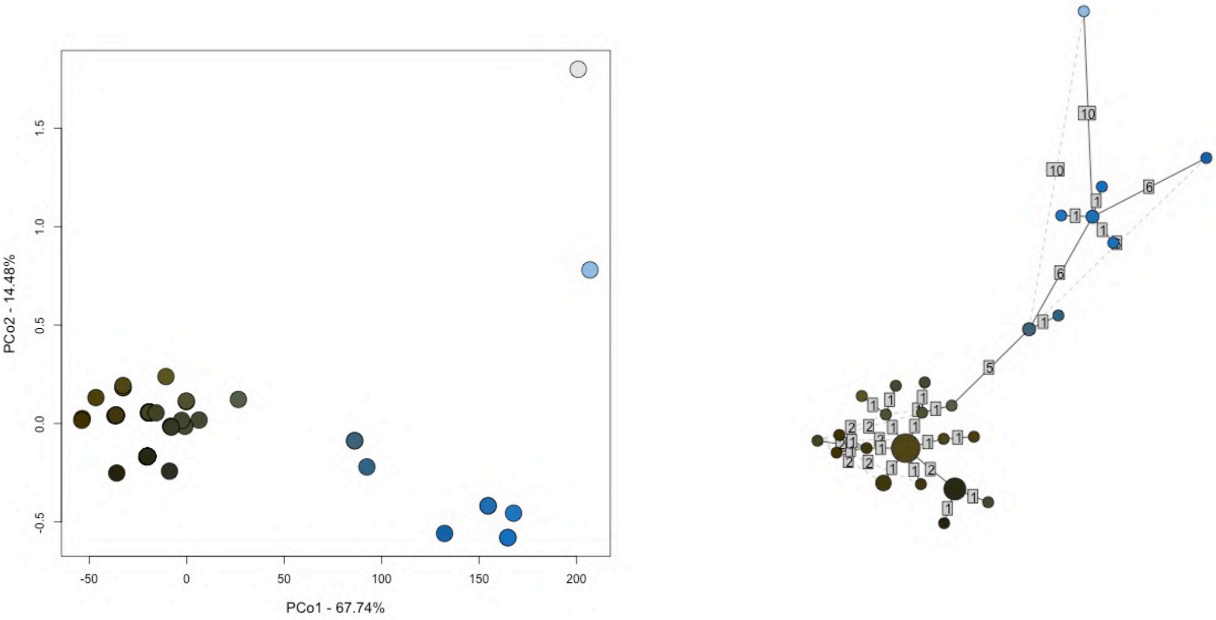

Figure 534: PCoA based on pairwise p-distances between *Plagiolepis pyrenaica* sequences (left). Colours match a bidimensional colour space. Haplotype network of *Plagiolepis pyrenaica* (right). Sequences > 599 bp: ID = 77, cf. = 13.

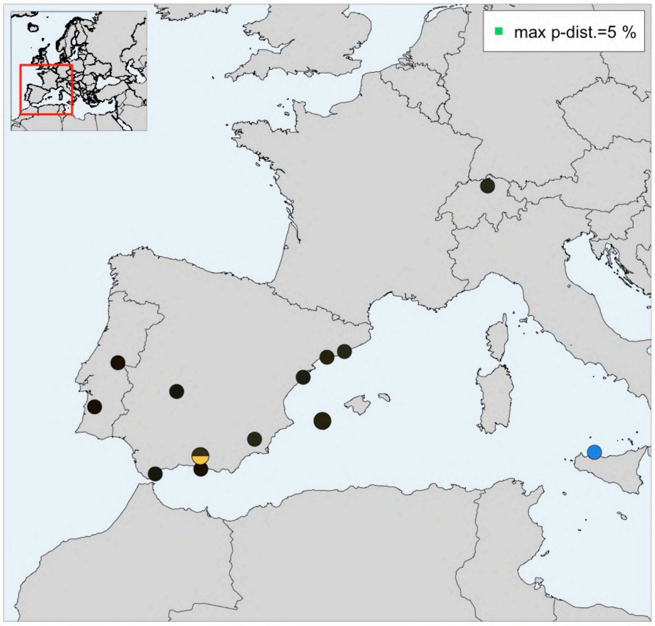

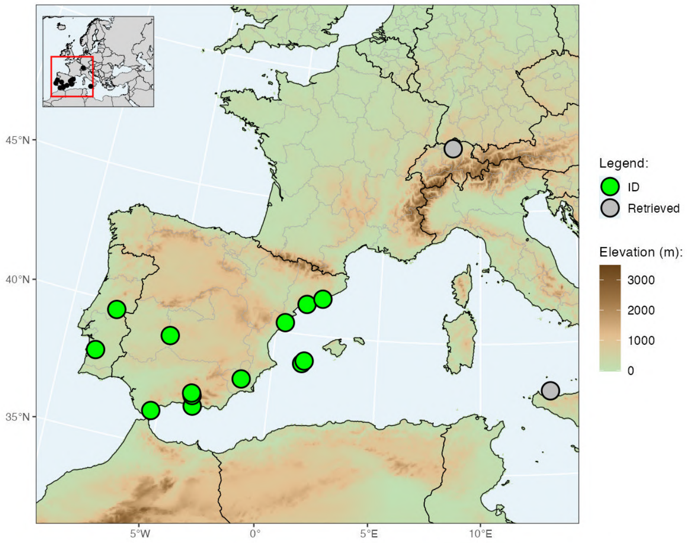

Figure 535: Genetic diversity map of *Plagiolepis schmitzii* Forel, 1895. Nearby localities of sequenced specimens are merged in pies (left). Colours match the bidimensional colour space of the PCoA projection (Fig. 535 left) of p-dist between sequences (dots). Specimen identification (ID or cf.) and source (newly sequenced or retrieved) are represented by colours, while specimen attribute (terra typica, type locality, type specimen or faunistic novelty) is represented by the shape (right). Sequences: ID = 15, cf. = 0; maximum p-distance: strict = 5 %, less strict = 5 %.

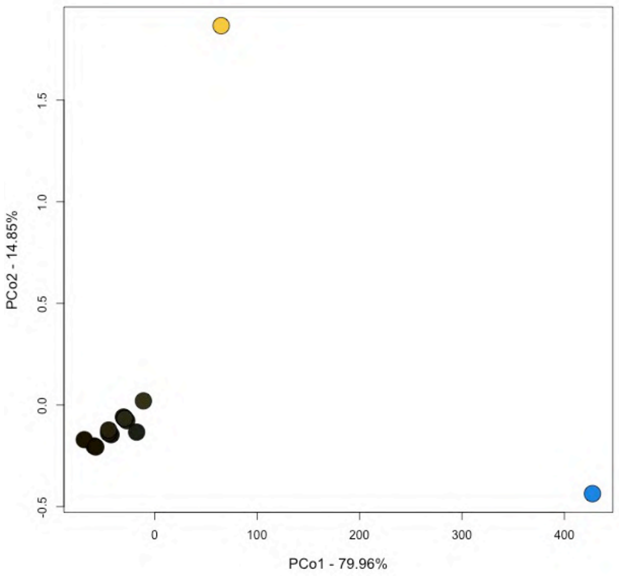

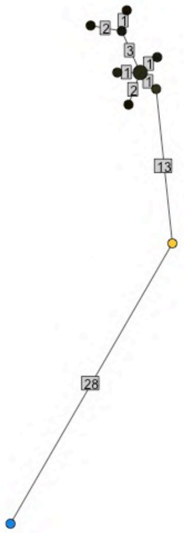

Figure 536: PCoA based on pairwise p-distances between *Plagiolepis schmitzii* sequences (left). Colours match a bidimensional colour space. Haplotype network of *Plagiolepis schmitzii* (right). Sequences > 599 bp: ID = 15, cf. = 0.

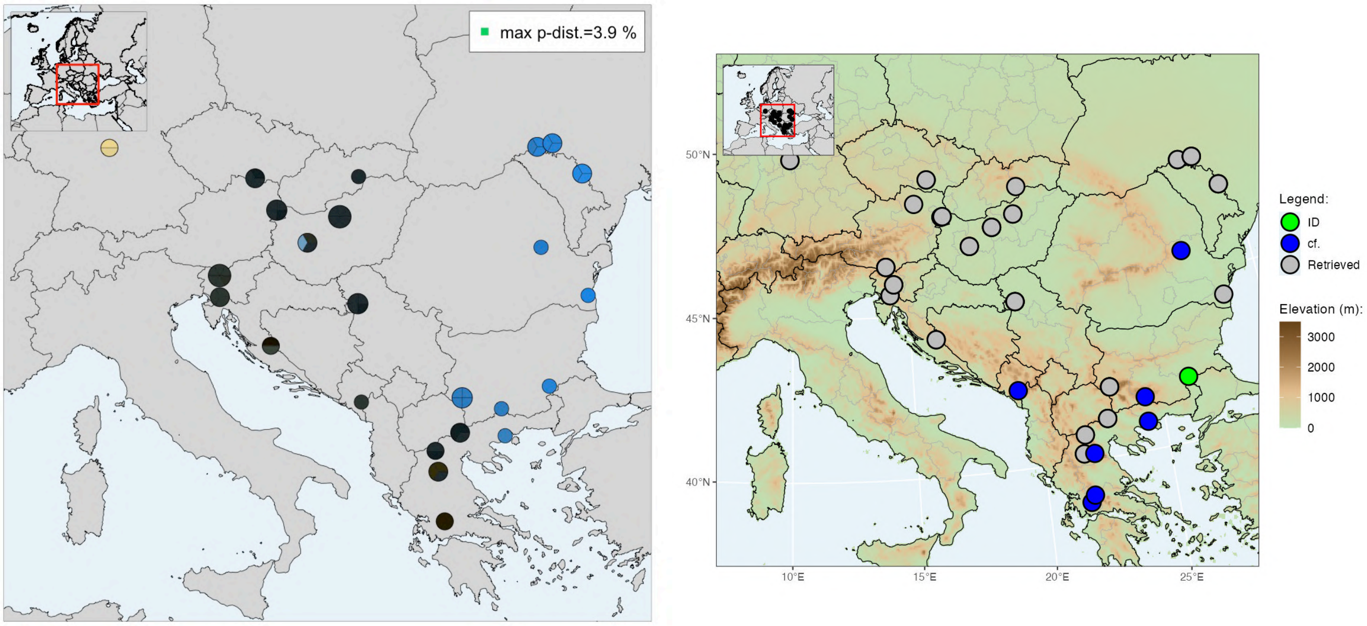

Figure 537: Genetic diversity map of *Plagiolepis taurica* Santschi, 1920. Nearby localities of sequenced specimens are merged in pies (left). Colours match the bidimensional colour space of the PCoA projection (Fig. 537 left) of p-dist between sequences (dots). Specimen identification (ID or cf.) and source (newly sequenced or retrieved) are represented by colours, while specimen attribute (terra typica, type locality, type specimen or faunistic novelty) is represented by the shape (right). Sequences: ID = 56, cf. = 7; maximum p-distance: strict = 3.9 %, less strict = 3.9 %.

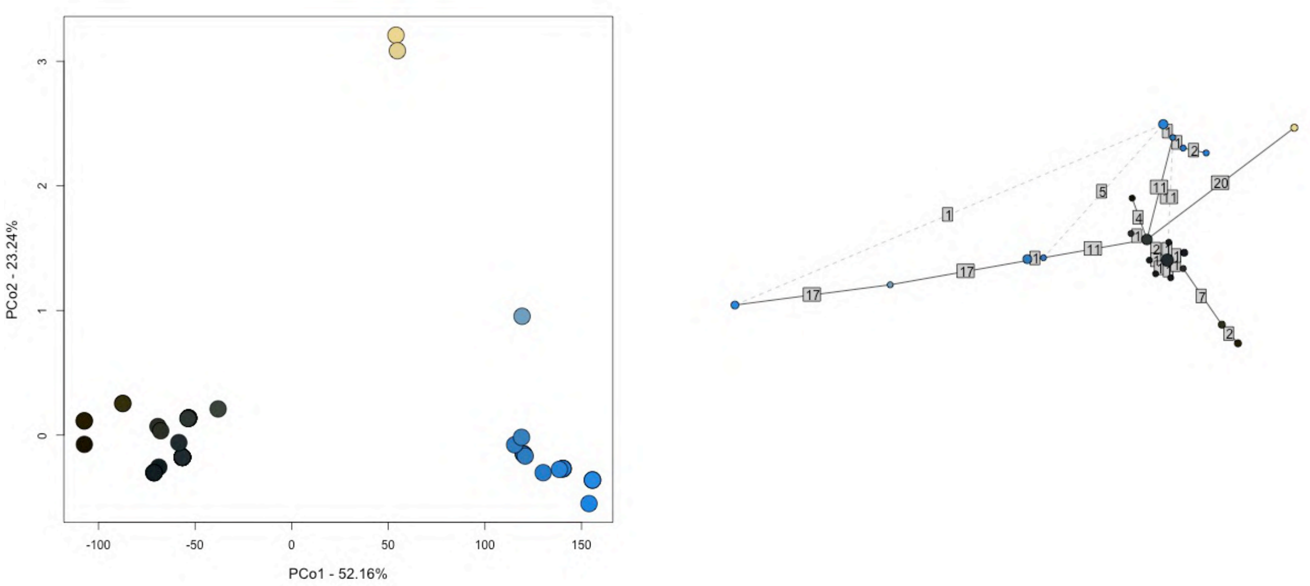

Figure 538: PCoA based on pairwise p-distances between *Plagiolepis taurica* sequences (left). Colours match a bidimensional colour space. Haplotype network of *Plagiolepis taurica* (right). Sequences > 599 bp: ID = 56, cf. = 7.

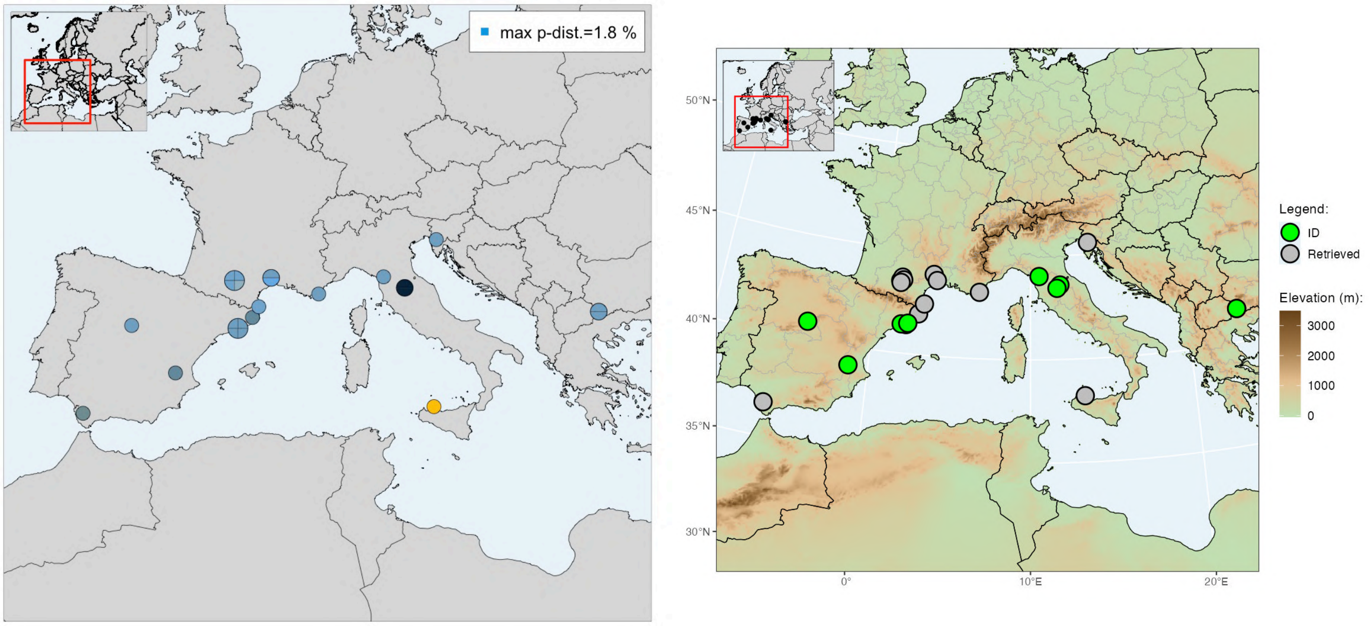

Figure 539: Genetic diversity map of *Plagiolepis xene* Stärcke, 1936. Nearby localities of sequenced specimens are merged in pies (left). Colours match the bidimensional colour space of the PCoA projection (Fig. 539 left) of p-dist between sequences (dots). Specimen identification (ID or cf.) and source (newly sequenced or retrieved) are represented by colours, while specimen attribute (terra typica, type locality, type specimen or faunistic novelty) is represented by the shape (right). Sequences: ID = 23, cf. = 0; maximum p-distance: strict = 1.8 %, less strict = 1.8 %.

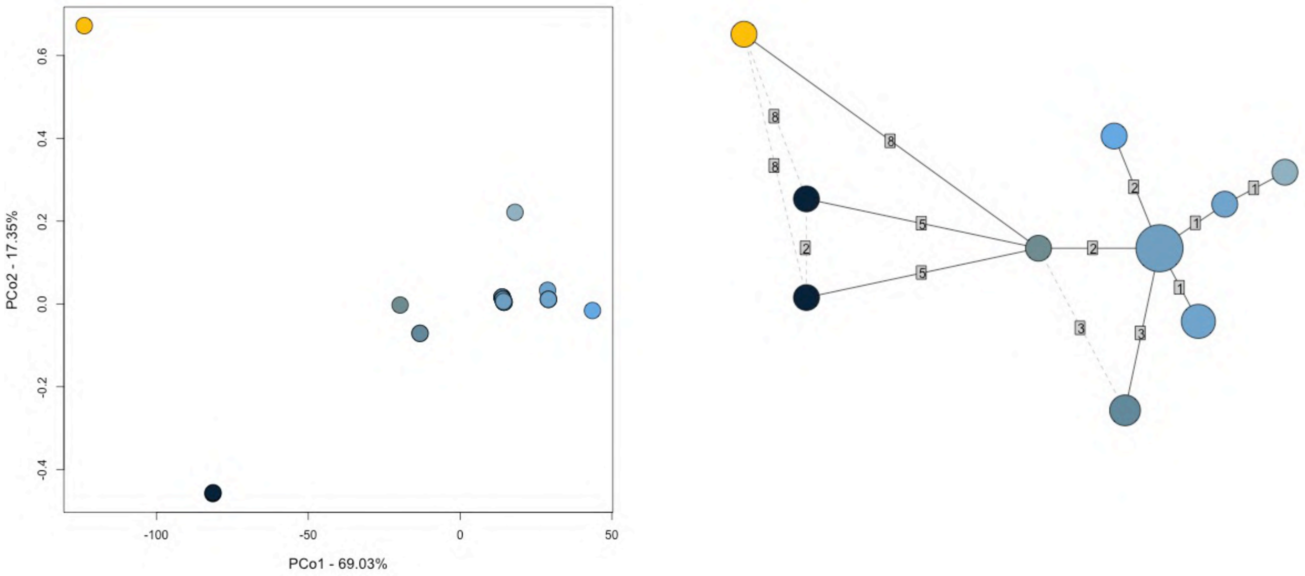

Figure 540: PCoA based on pairwise p-distances between *Plagiolepis xene* sequences (left). Colours match a bidimensional colour space. Haplotype network of *Plagiolepis xene* (right). Sequences > 599 bp: ID = 23, cf. = 0.

Polyergus

Polyergus rufescens

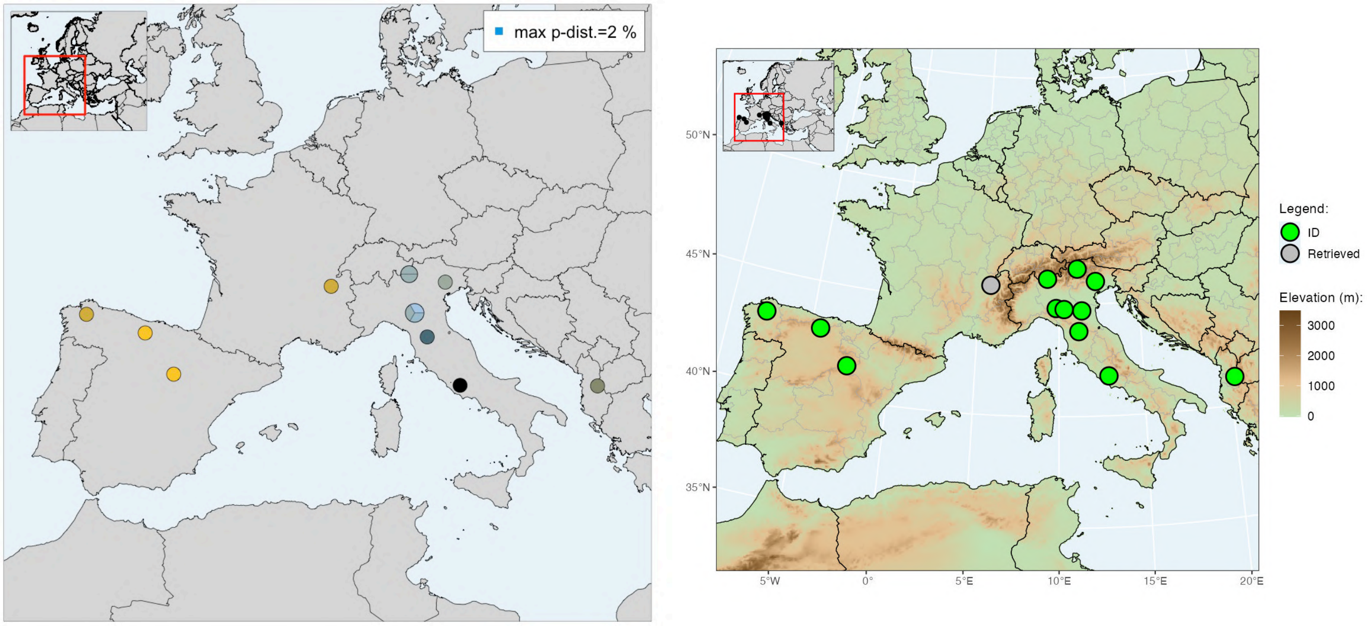

Figure 541: Genetic diversity map of *Polyergus rufescens* (Latreille, 1798). Nearby localities of sequenced specimens are merged in pies (left). Colours match the bidimensional colour space of the PCoA projection (Fig. 541 left) of p-dist between sequences (dots). Specimen identification (ID or cf.) and source (newly sequenced or retrieved) are represented by colours, while specimen attribute (terra typica, type locality, type specimen or faunistic novelty) is represented by the shape (right). Sequences: ID = 13, cf. = 0; maximum p-distance: strict = 2 %, less strict = 2 %.

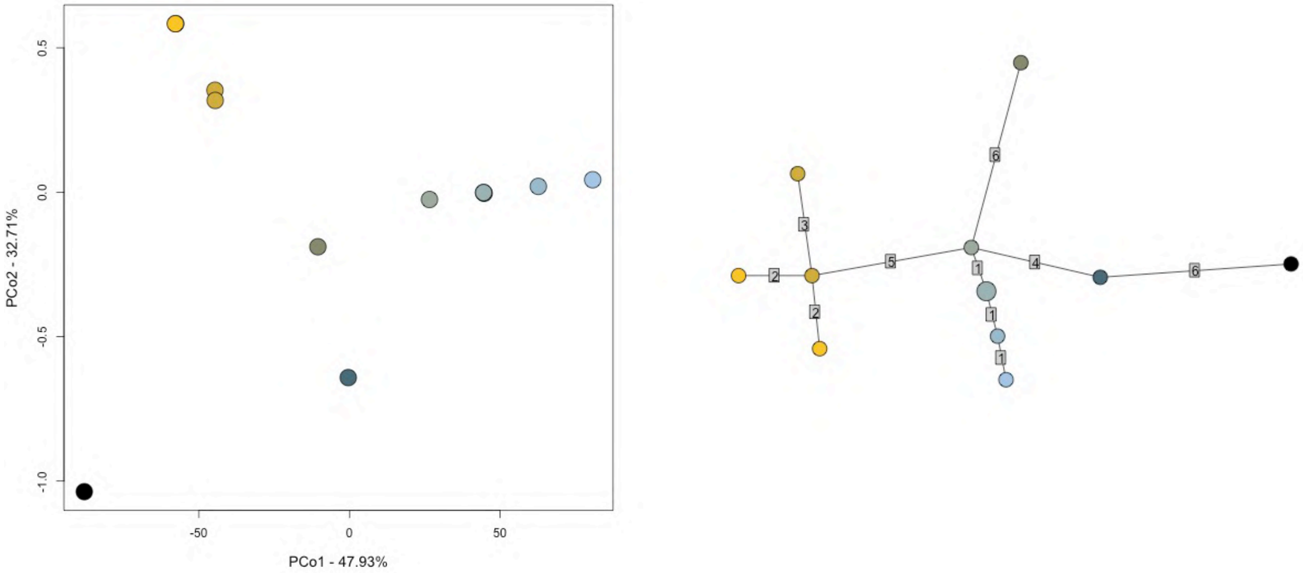

Figure 542: PCoA based on pairwise p-distances between *Polyergus rufescens* sequences (left). Colours match a bidimensional colour space. Haplotype network of *Polyergus rufescens* (right). Sequences > 599 bp: ID = 13, cf. = 0.

Ponera

Ponera coarctata

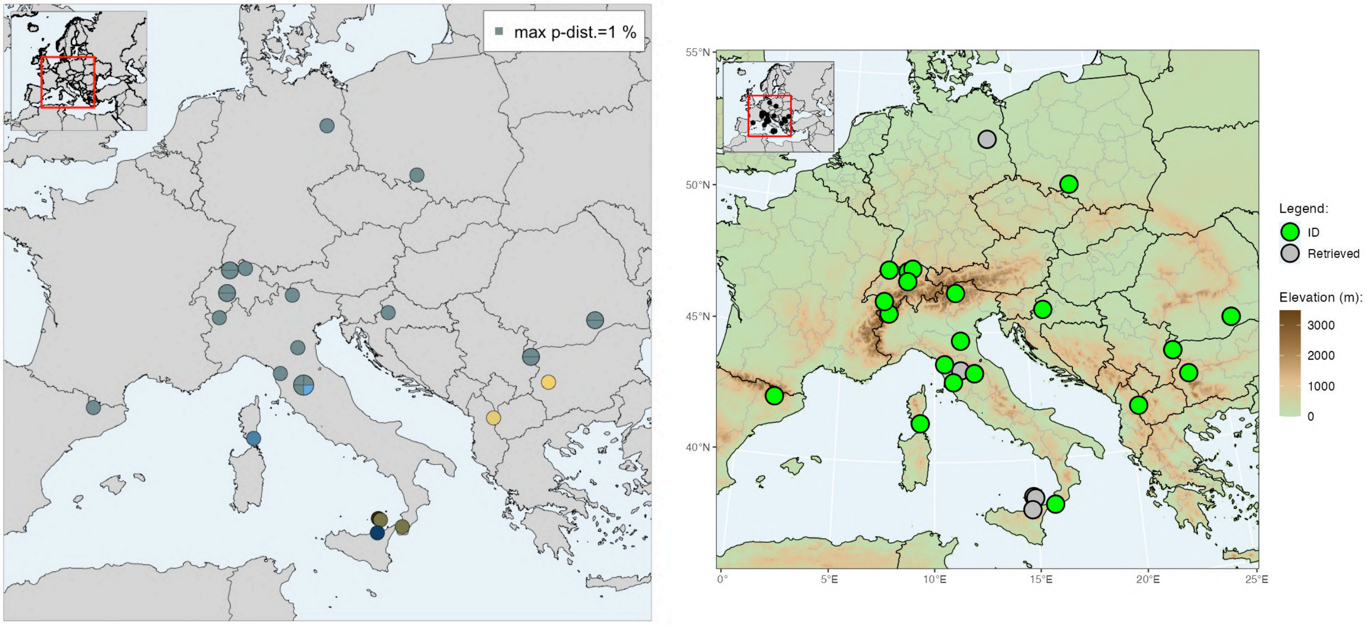

Figure 543: Genetic diversity map of *Ponera coarctata* (Latreille, 1802). Nearby localities of sequenced specimens are merged in pies (left). Colours match the bidimensional colour space of the PCoA projection (Fig. 543 left) of p-dist between sequences (dots). Specimen identification (ID or cf.) and source (newly sequenced or retrieved) are represented by colours, while specimen attribute (terra typica, type locality, type specimen or faunistic novelty) is represented by the shape (right). Sequences: ID = 28, cf. = 0; maximum p-distance: strict = 1 %, less strict = 1 %.

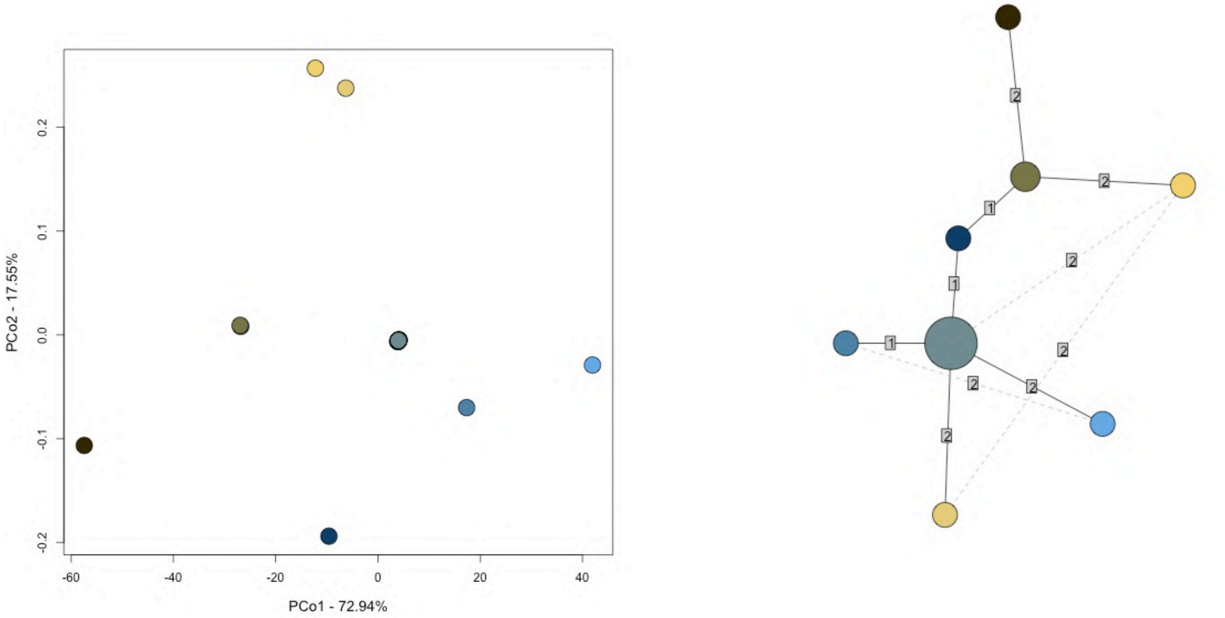

Figure 544: PCoA based on pairwise p-distances between *Ponera coarctata* sequences (left). Colours match a bidimensional colour space. Haplotype network of *Ponera coarctata* (right). Sequences > 599 bp: ID = 28, cf. = 0.

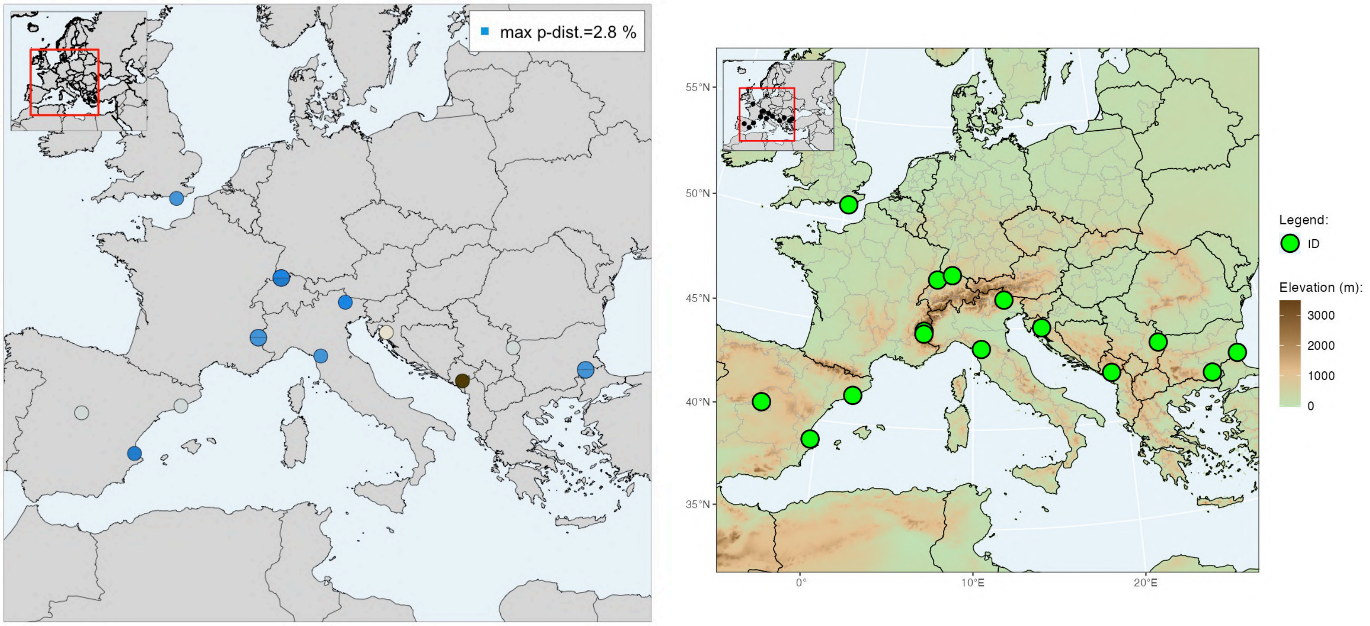

Figure 545: Genetic diversity map of *Ponera testacea* Emery, 1895. Nearby localities of sequenced specimens are merged in pies (left). Colours match the bidimensional colour space of the PCoA projection (Fig. 545 left) of p-dist between sequences (dots). Specimen identification (ID or cf.) and source (newly sequenced or retrieved) are represented by colours, while specimen attribute (terra typica, type locality, type specimen or faunistic novelty) is represented by the shape (right). Sequences: ID = 15, cf. = 0; maximum p-distance: strict = 2.8 %, less strict = 2.8 %.

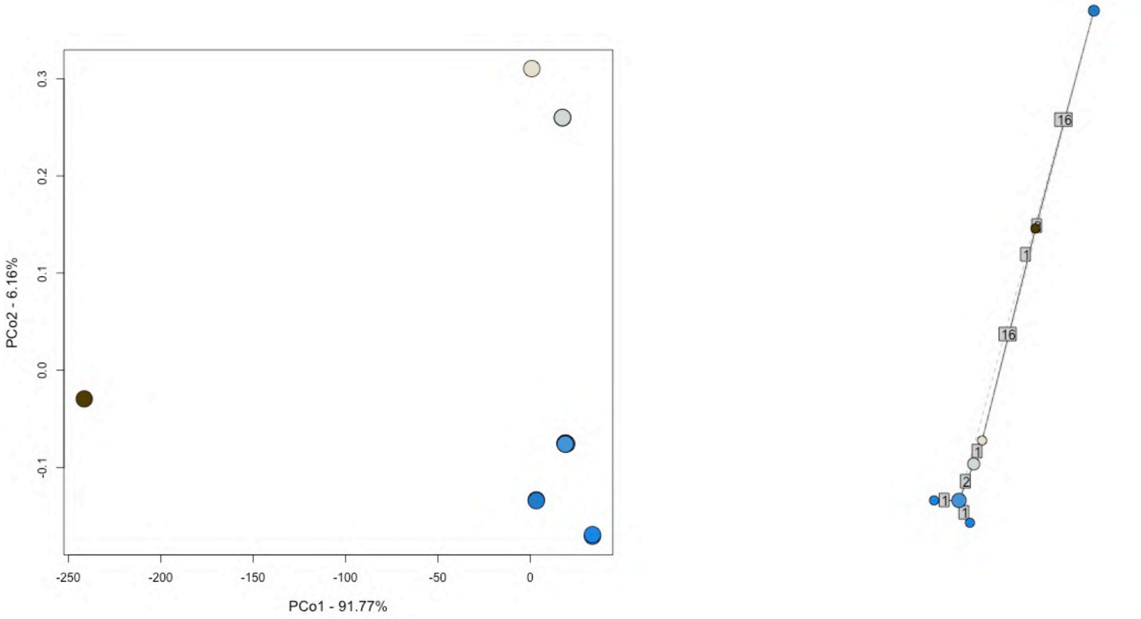

Figure 546: PCoA based on pairwise p-distances between *Ponera testacea* sequences (left). Colours match a bidimensional colour space. Haplotype network of *Ponera testacea* (right). Sequences > 599 bp: ID = 15, cf. = 0.

Prenolepis

Prenolepis nitens

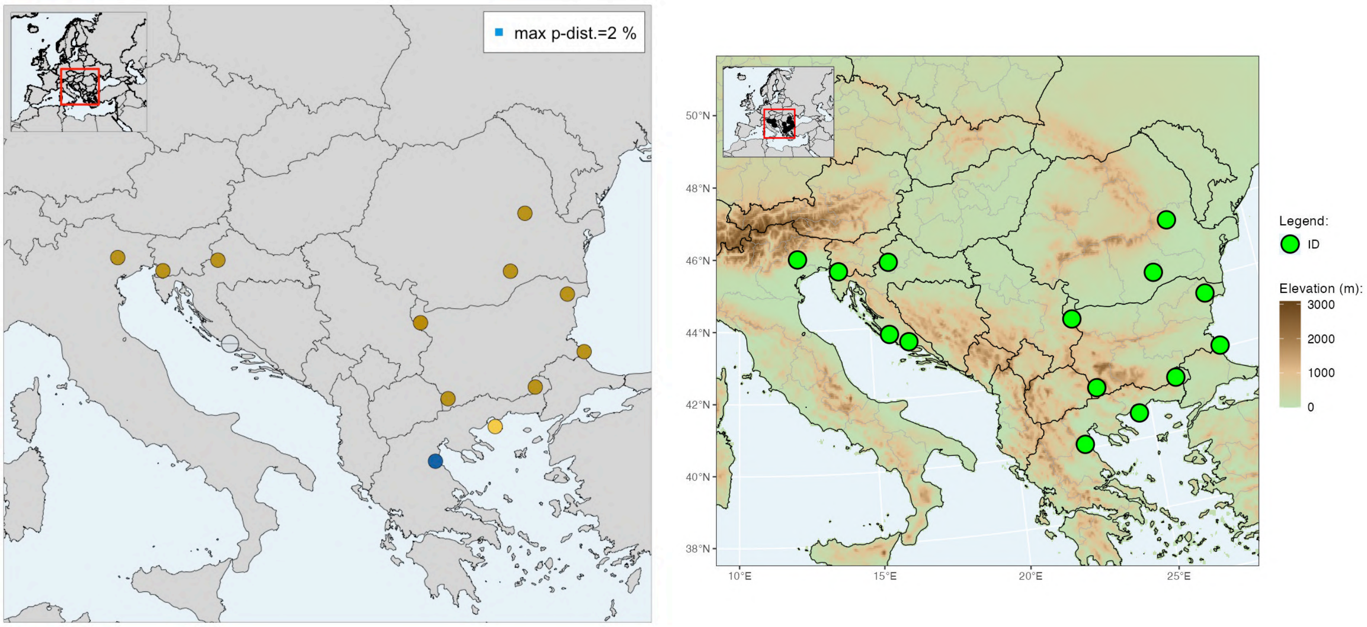

Figure 547: Genetic diversity map of *Prenolepis nitens* (Mayr, 1853). Nearby localities of sequenced specimens are merged in pies (left). Colours match the bidimensional colour space of the PCoA projection (Fig. 547 left) of p-dist between sequences (dots). Specimen identification (ID or cf.) and source (newly sequenced or retrieved) are represented by colours, while specimen attribute (terra typica, type locality, type specimen or faunistic novelty) is represented by the shape (right). Sequences: ID = 14, cf. = 0; maximum p-distance: strict = 2 %, less strict = 2 %.

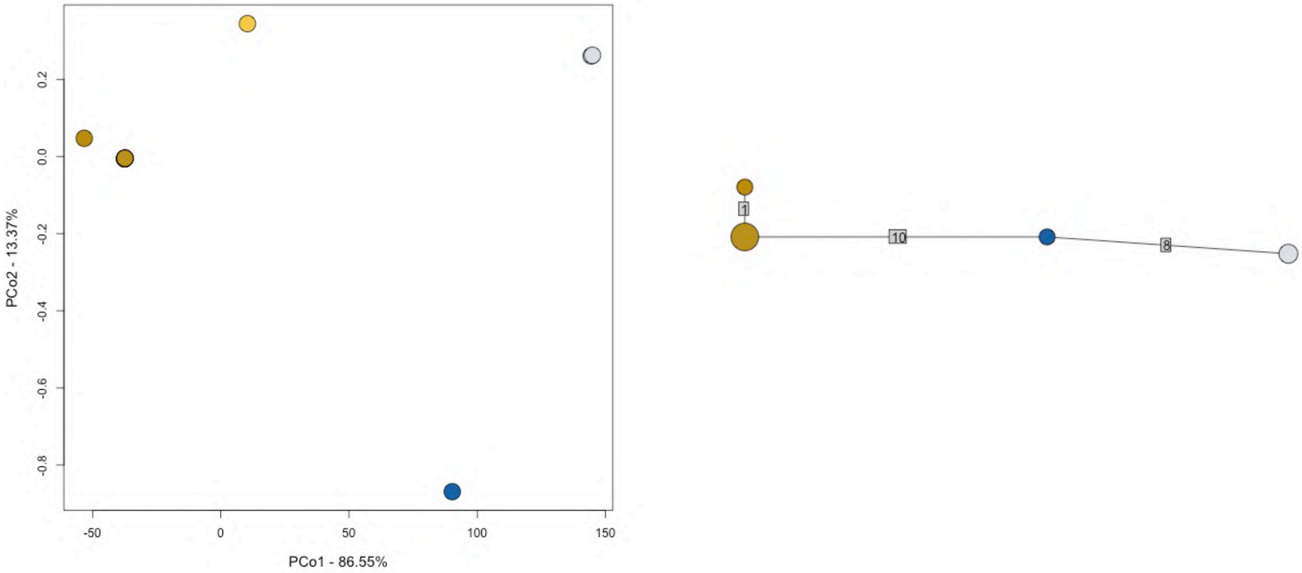

Figure 548: PCoA based on pairwise p-distances between *Prenolepis nitens* sequences (left). Colours match a bidimensional colour space. Haplotype network of *Prenolepis nitens* (right). Sequences > 599 bp: ID = 13, cf. = 0.

Proceratium

Proceratium algiricum

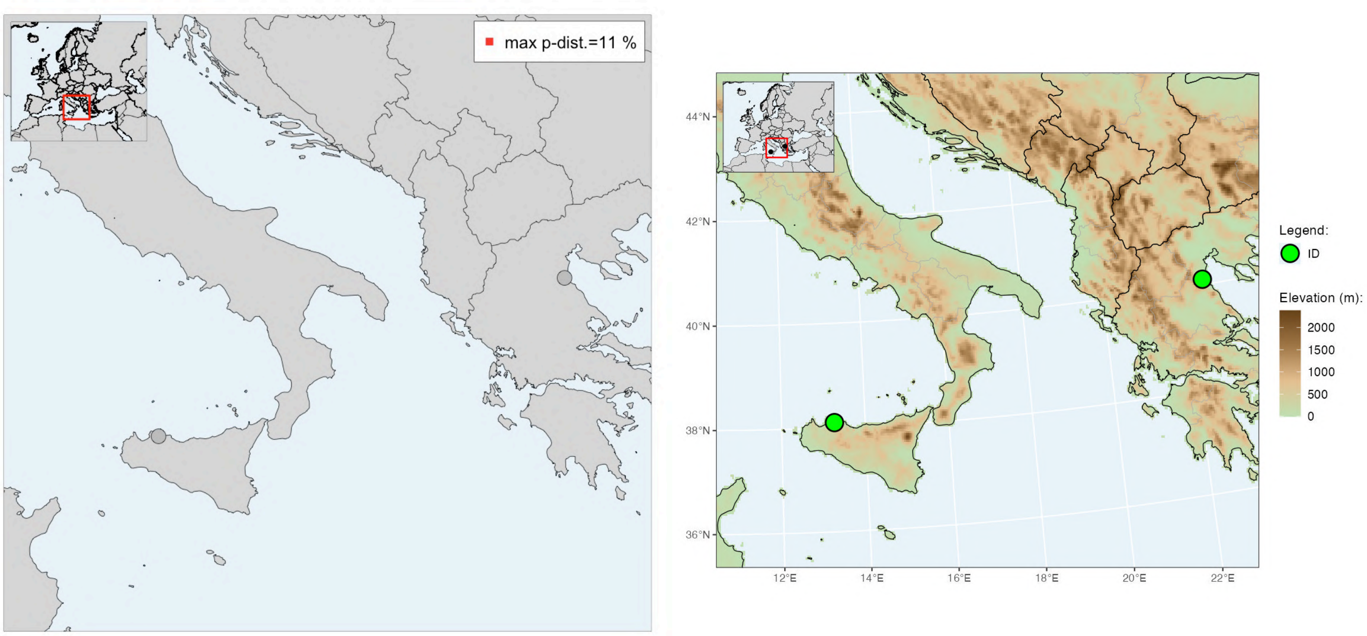

Figure 549: Genetic diversity map of *Proceratium algiricum* Forel, 1899. PCoA projection was not done and therefore sequenced specimens in the genetic diversity map are coloured in gray (left). Specimen identification (ID or cf.) and source (newly sequenced or retrieved) are represented by colours, while specimen attribute (terra typica, type locality, type specimen or faunistic novelty) is represented by the shape (right). Sequences: ID = 2, cf. = 0; maximum p-distance: strict = NA, less strict = 11 %.

Haplotype network analysis of *Proceratium algiricum* was not possible.

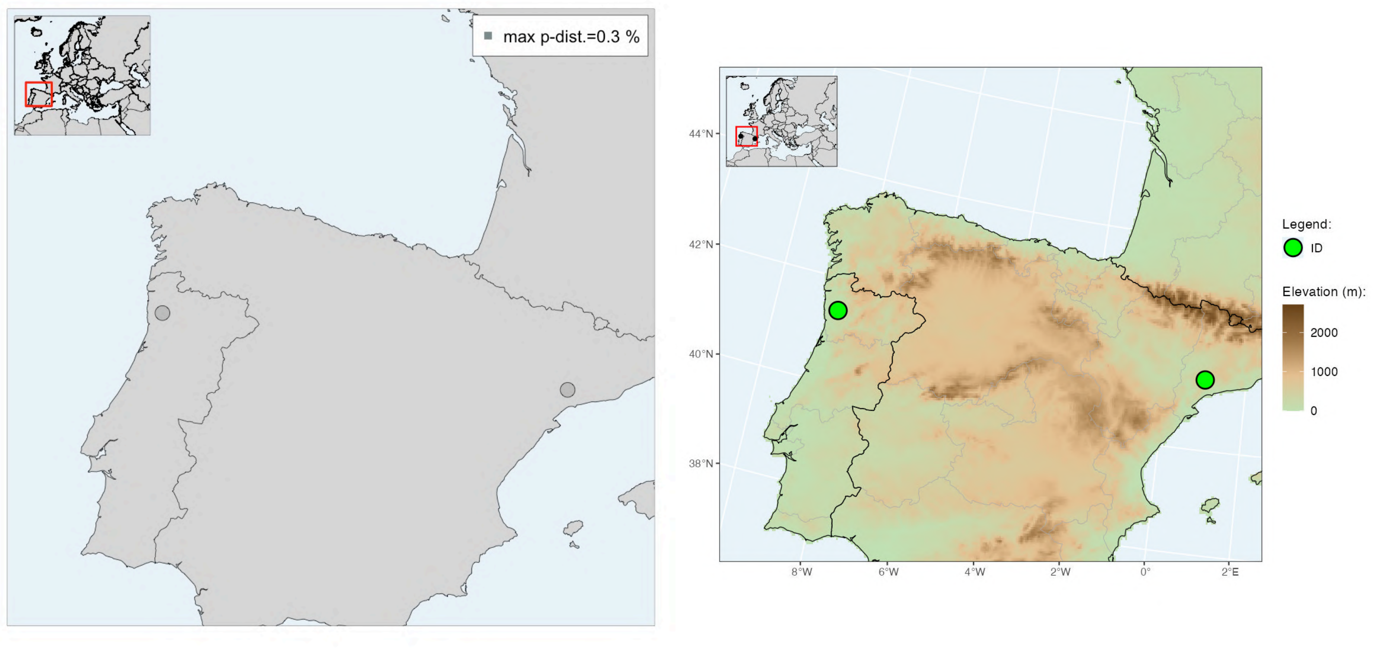

Figure 550: Genetic diversity map of *Proceratium melinum* (Roger, 1860). PCoA projection was not done and therefore sequenced specimens in the genetic diversity map are coloured in gray (left). Specimen identification (ID or cf.) and source (newly sequenced or retrieved) are represented by colours, while specimen attribute (terra typica, type locality, type specimen or faunistic novelty) is represented by the shape (right). Sequences: ID = 2, cf. = 0; maximum p-distance: strict = NA, less strict = 0.3 %.

Haplotype network analysis of *Proceratium melinum* was not possible.

Proformica

Proformica cerdanyensis

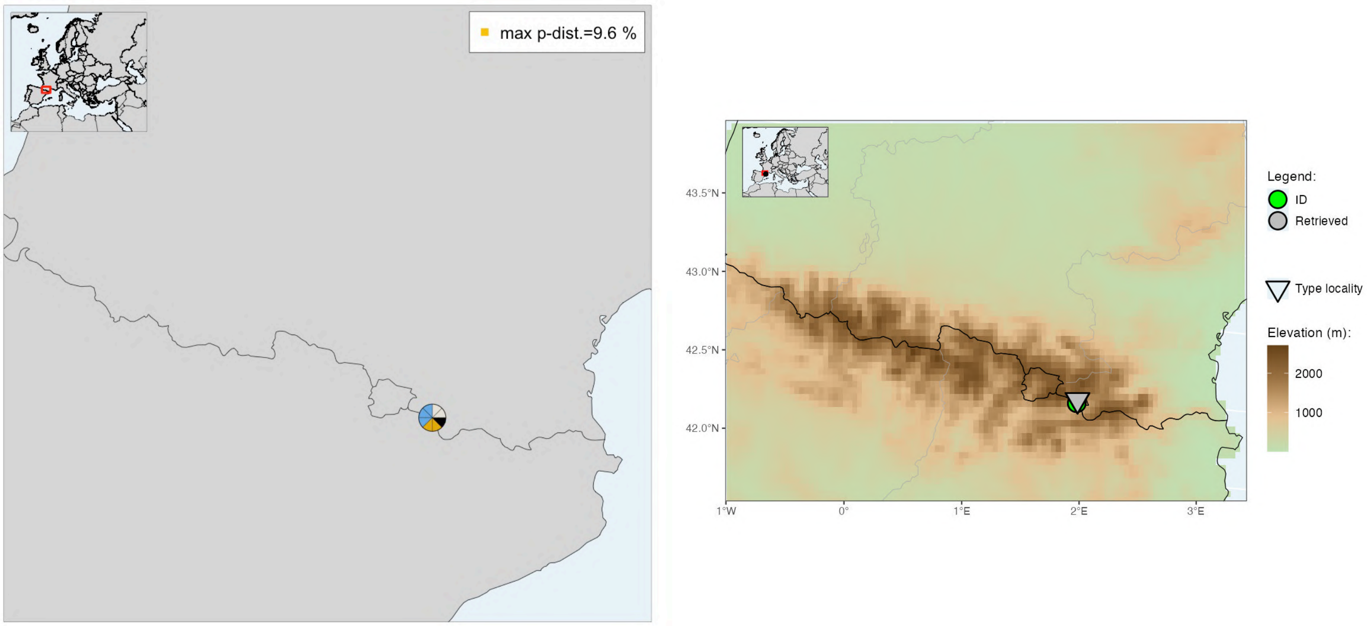

Figure 551: Genetic diversity map of *Proformica cerdanyensis* Galkowski, Lebas, Lenoir, Perdereau & Blatrix, 2022. Nearby localities of sequenced specimens are merged in pies (left). Colours match the bidimensional colour space of the PCoA projection (Fig. 551 left) of p-dist between sequences (dots). Specimen identification (ID or cf.) and source (newly sequenced or retrieved) are represented by colours, while specimen attribute (terra typica, type locality, type specimen or faunistic novelty) is represented by the shape (right). Sequences: ID = 8, cf. = 0; maximum p-distance: strict = 8.8 %, less strict = 9.6 %.

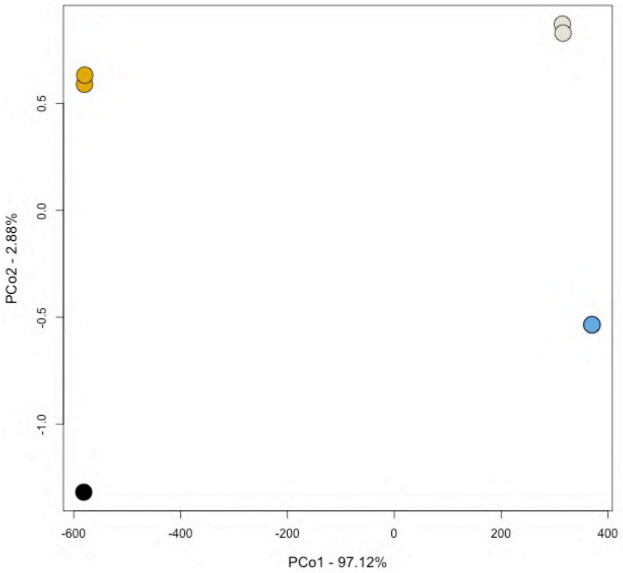

Figure 552: PCoA based on pairwise p-distances between *Proformica cerdanyensis* sequences (left). Colours match a bidimensional colour space. Haplotype network analysis of *Proformica cerdanyensis* was not possible.

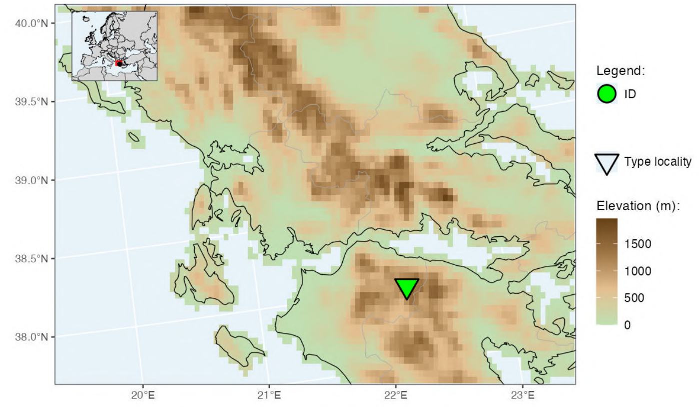

Figure 553: Map of *Proformica chelmosensis* Lebas & Galkowski, 2019. Due to the presence of a single sequence, the genetic diversity map and the PCoA projection were not done. Specimen identification (ID or cf.) and source (newly sequenced or retrieved) are represented by colours, while specimen attribute (terra typica, type locality, type specimen or faunistic novelty) is represented by the shape. Sequences: ID = 1, cf. = 0; maximum p-distance: strict = NA, less strict = NA.

Haplotype network analysis of *Proformica chelmosensis* was not possible.

Proformica ferreri

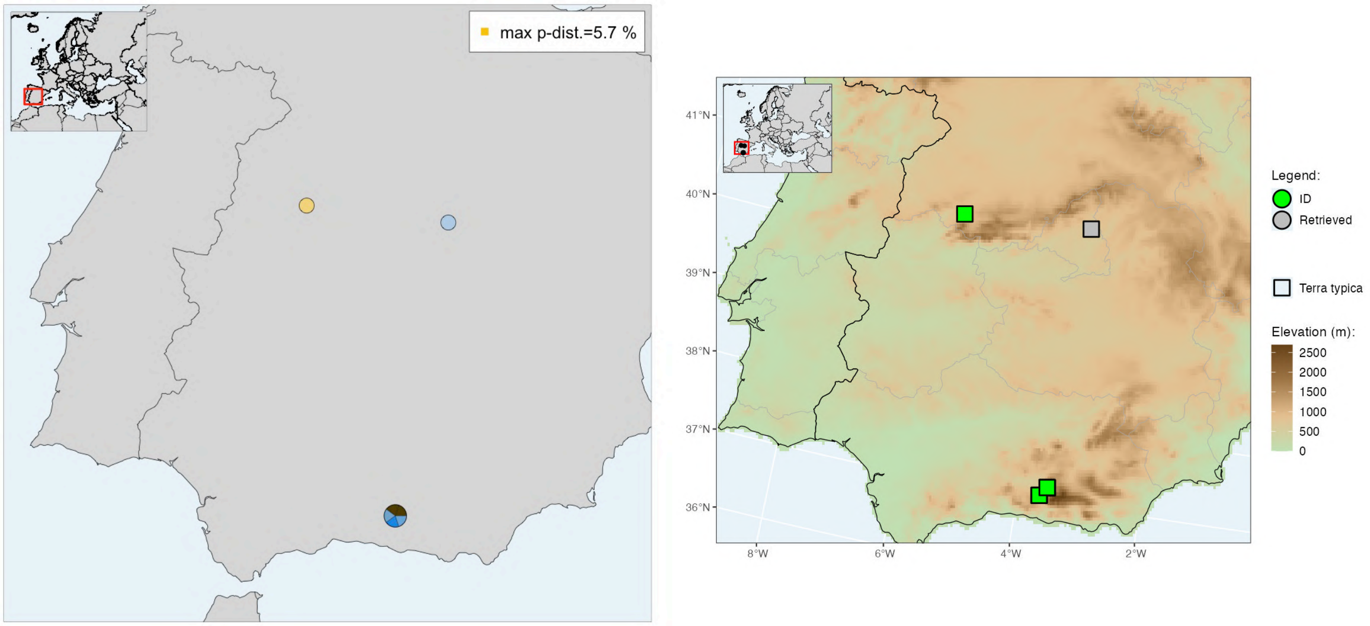

Figure 554: Genetic diversity map of *Proformica ferreri* Bondroit, 1918. Nearby localities of sequenced specimens are merged in pies (left). Colours match the bidimensional colour space of the PCoA projection (Fig. 554 left) of p-dist between sequences (dots). Specimen identification (ID or cf.) and source (newly sequenced or retrieved) are represented by colours, while specimen attribute (terra typica, type locality, type specimen or faunistic novelty) is represented by the shape (right). Sequences: ID = 8, cf. = 0; maximum p-distance: strict = 4.7 %, less strict = 5.7 %.

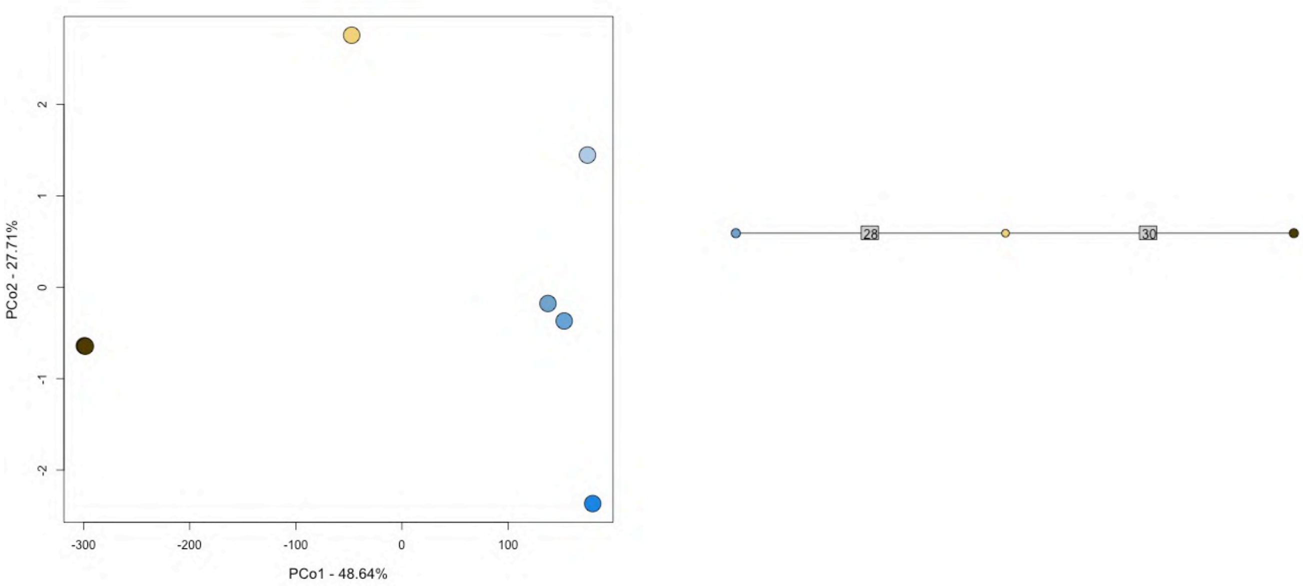

Figure 555: PCoA based on pairwise p-distances between *Proformica ferreri* sequences (left). Colours match a bidimensional colour space. Haplotype network of *Proformica ferreri* (right). Sequences > 599 bp: ID = 5, cf. = 0.

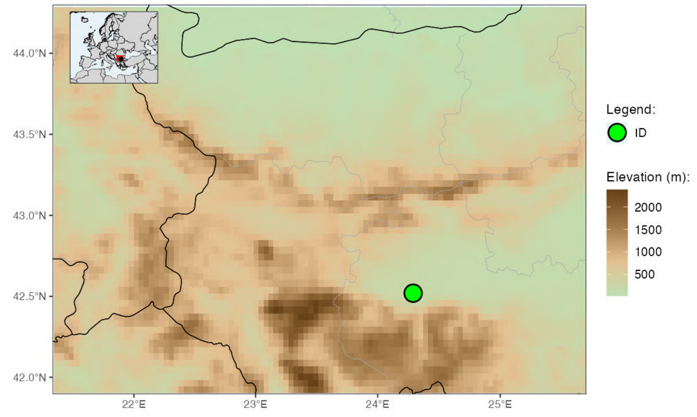

Figure 556: Map of *Proformica korbi* (Emery, 1909). Due to the presence of a single sequence, the genetic diversity map and the PCoA projection were not done. Specimen identification (ID or cf.) and source (newly sequenced or retrieved) are represented by colours, while specimen attribute (terra typica, type locality, type specimen or faunistic novelty) is represented by the shape. Sequences: ID = 1, cf. = 0; maximum p-distance: strict = NA, less strict = NA.

Haplotype network analysis of *Proformica korbi* was not possible.

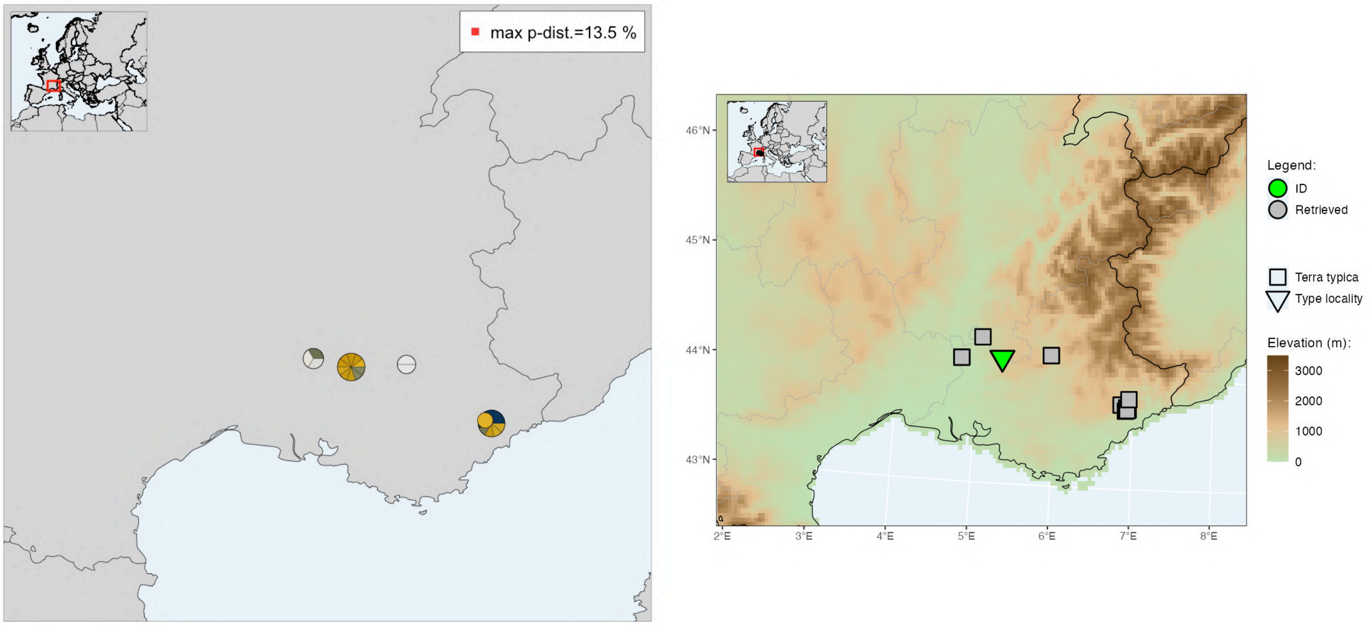

Figure 557: Genetic diversity map of *Proformica longipilosa* Galkowski, Lebas, Wegnez, Lenoir & Blatrix, 2017. Nearby localities of sequenced specimens are merged in pies (left). Colours match the bidimensional colour space of the PCoA projection (Fig. 557 left) of p-dist between sequences (dots). Specimen identification (ID or cf.) and source (newly sequenced or retrieved) are represented by colours, while specimen attribute (terra typica, type locality, type specimen or faunistic novelty) is represented by the shape (right). Sequences: ID = 25, cf. = 0; maximum p-distance: strict = 12.2 %, less strict = 13.5 %.

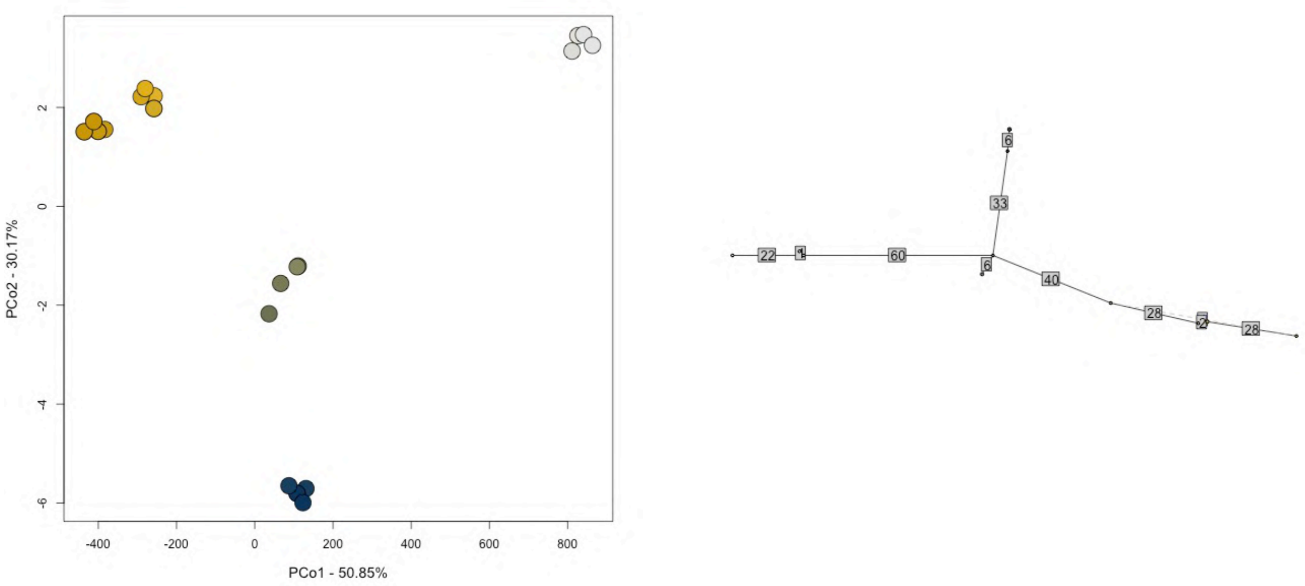

Figure 558: PCoA based on pairwise p-distances between *Proformica longipilosa* sequences (left). Colours match a bidimensional colour space. Haplotype network of *Proformica longipilosa* (right). Sequences > 599 bp: ID = 15, cf. = 0.

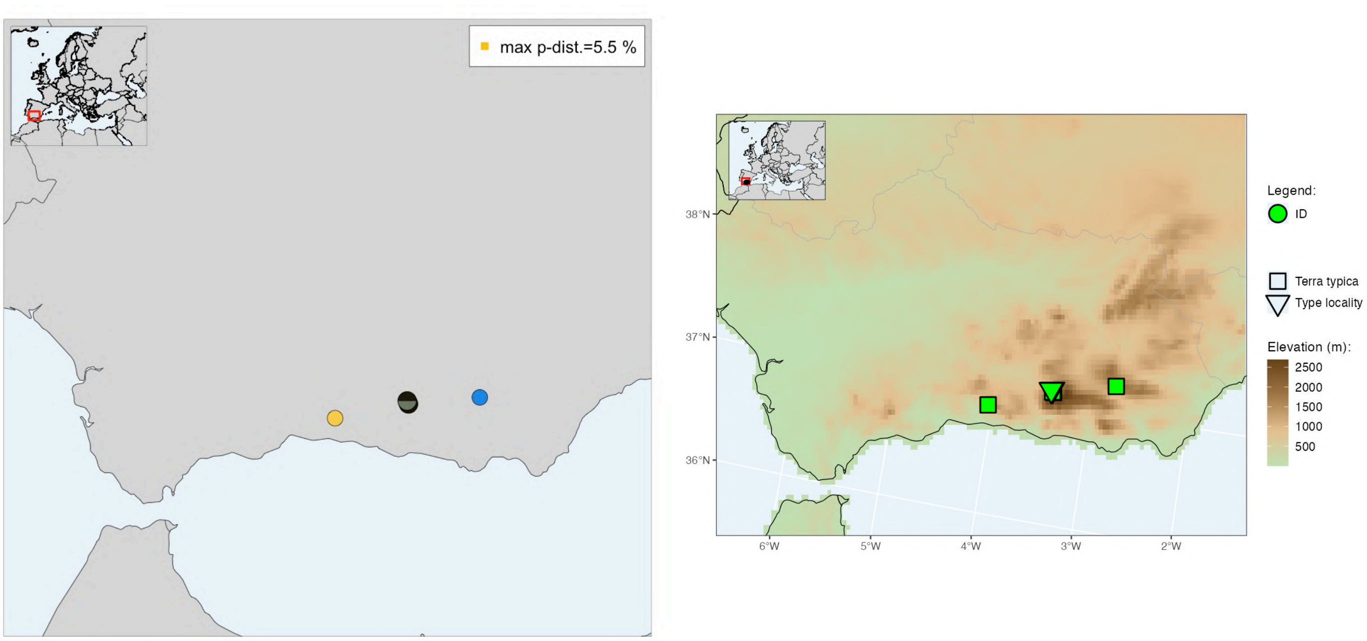

Figure 559: Genetic diversity map of *Proformica longiseta* Collingwood, 1978. Nearby localities of sequenced specimens are merged in pies (left). Colours match the bidimensional colour space of the PCoA projection (Fig. 559 left) of p-dist between sequences (dots). Specimen identification (ID or cf.) and source (newly sequenced or retrieved) are represented by colours, while specimen attribute (terra typica, type locality, type specimen or faunistic novelty) is represented by the shape (right). Sequences: ID = 13, cf. = 0; maximum p-distance: strict = 5.5 %, less strict = 5.5 %.

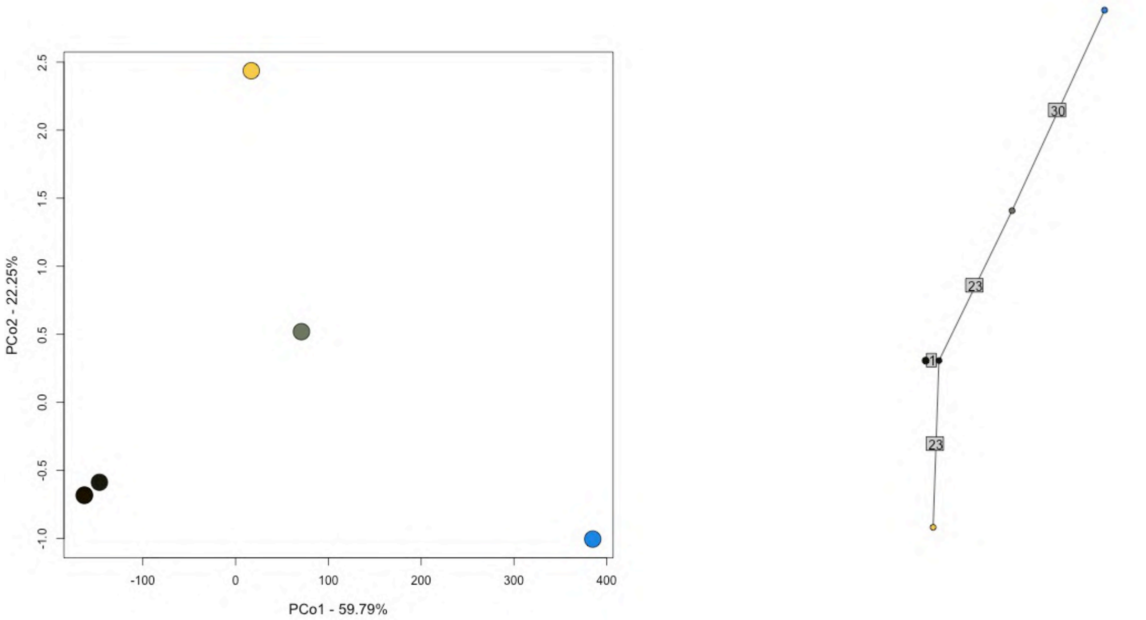

Figure 560: PCoA based on pairwise p-distances between *Proformica longiseta* sequences (left). Colours match a bidimensional colour space. Haplotype network of *Proformica longiseta* (right). Sequences > 599 bp: ID = 6, cf. = 0.

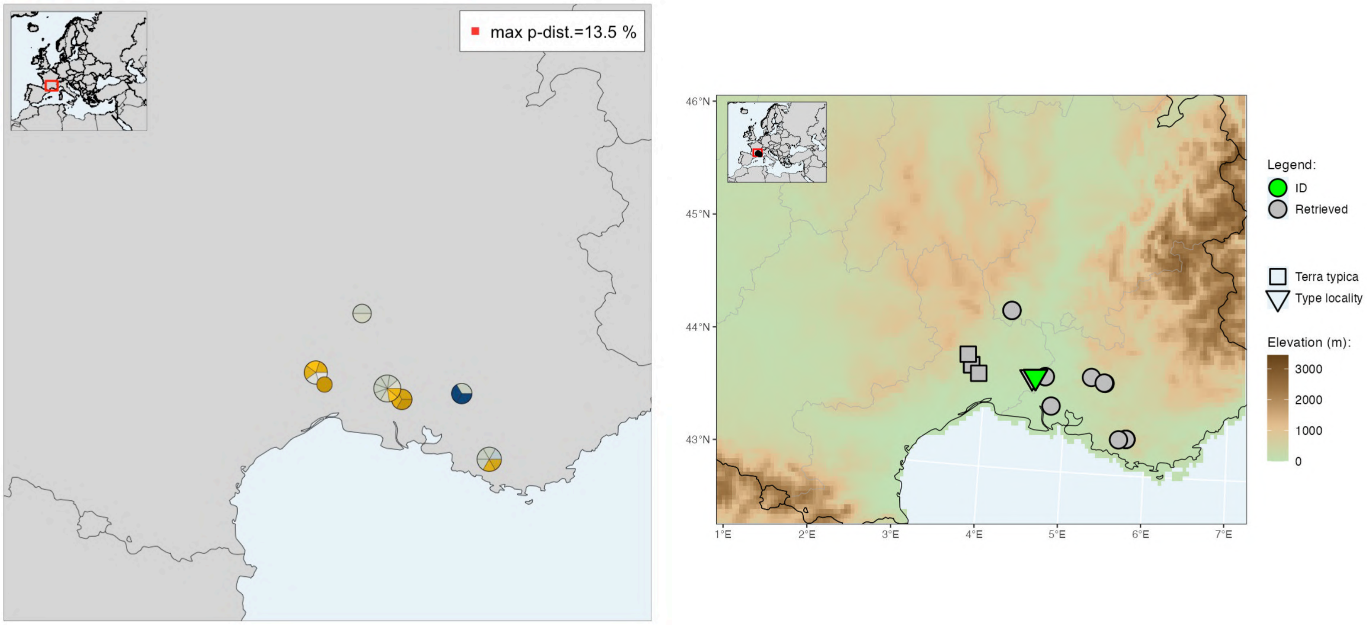

Figure 561: Genetic diversity map of *Proformica nasuta* (Nylander, 1856). Nearby localities of sequenced specimens are merged in pies (left). Colours match the bidimensional colour space of the PCoA projection (Fig. 561 left) of p-dist between sequences (dots). Specimen identification (ID or cf.) and source (newly sequenced or retrieved) are represented by colours, while specimen attribute (terra typica, type locality, type specimen or faunistic novelty) is represented by the shape (right). Sequences: ID = 30, cf. = 0; maximum p-distance: strict = 10.5 %, less strict = 13.5 %.

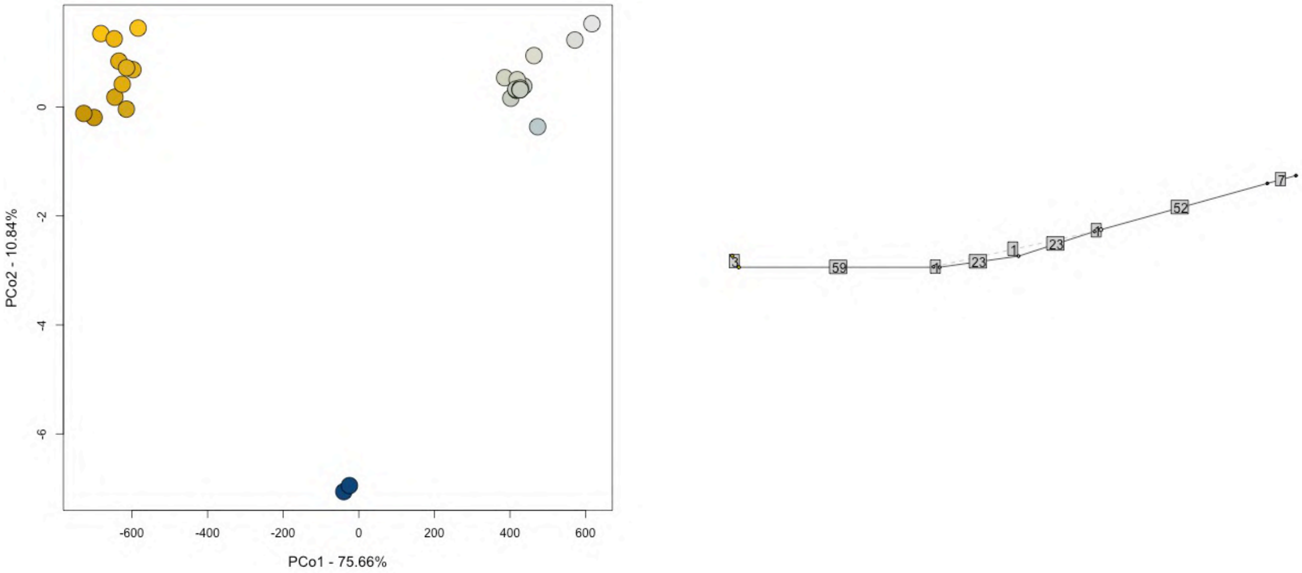

Figure 562: PCoA based on pairwise p-distances between *Proformica nasuta* sequences (left). Colours match a bidimensional colour space. Haplotype network of *Proformica nasuta* (right). Sequences > 599 bp: ID = 15, cf. = 0.

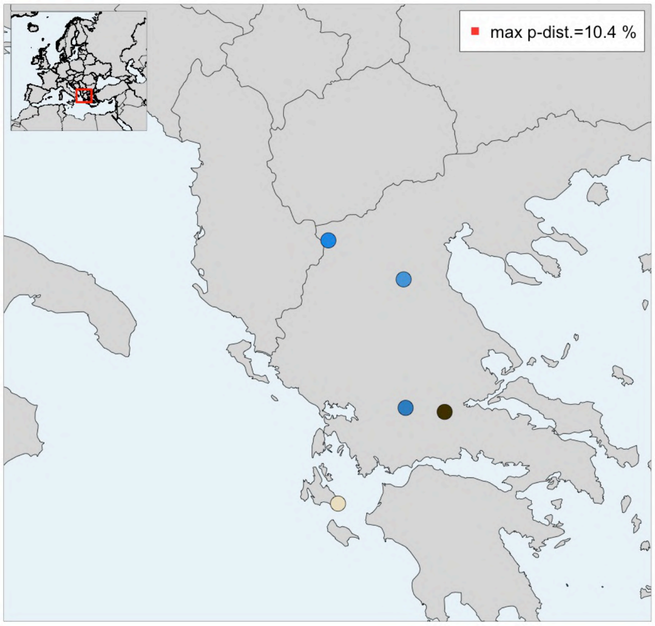

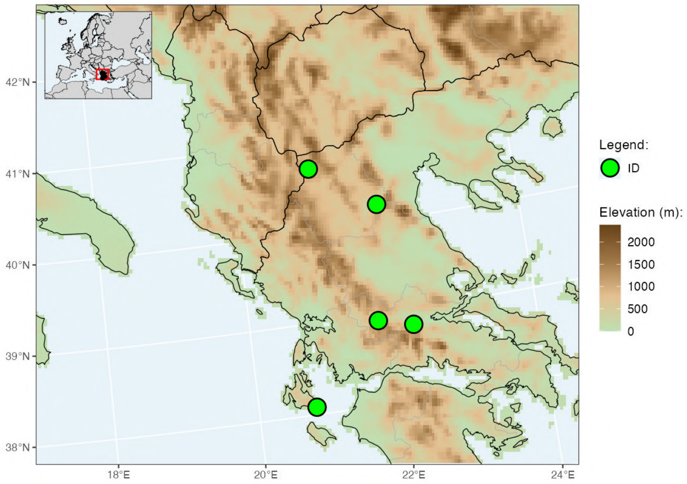

Figure 563: Genetic diversity map of *Proformica oculatissima* (Forel, 1886). Nearby localities of sequenced specimens are merged in pies (left). Colours match the bidimensional colour space of the PCoA projection (Fig. 563 left) of p-dist between sequences (dots). Specimen identification (ID or cf.) and source (newly sequenced or retrieved) are represented by colours, while specimen attribute (terra typica, type locality, type specimen or faunistic novelty) is represented by the shape (right). Sequences: ID = 5, cf. = 0; maximum p-distance: strict = 10.4 %, less strict = 10.4 %.

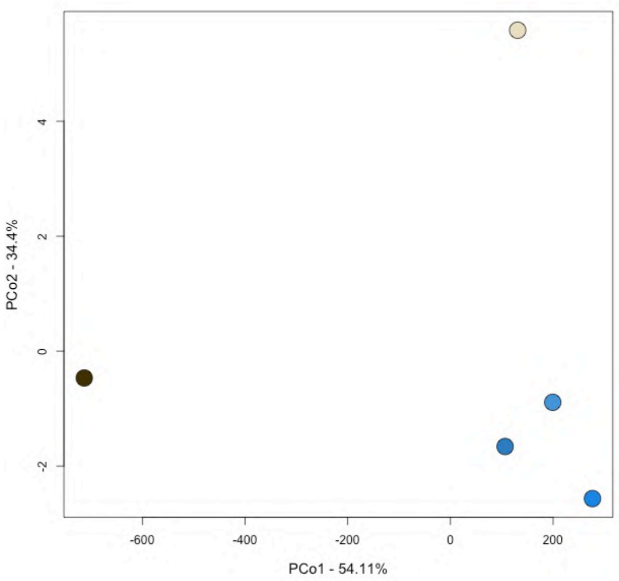

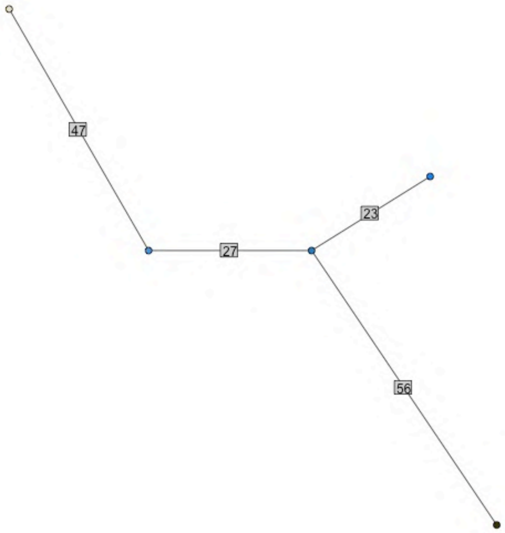

Figure 564: PCoA based on pairwise p-distances between *Proformica oculatissima* sequences (left). Colours match a bidimensional colour space. Haplotype network of *Proformica oculatissima* (right). Sequences > 599 bp: ID = 5, cf. = 0.

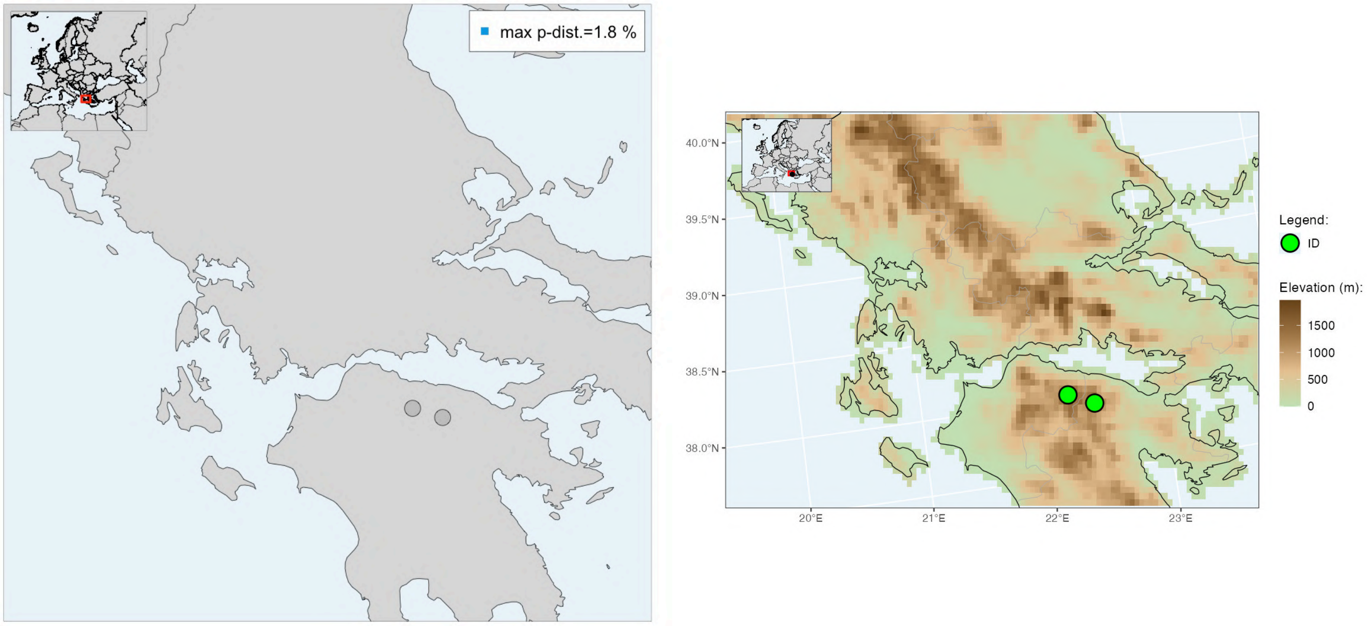

Figure 565: Genetic diversity map of *Proformica striaticeps* (Forel, 1911). PCoA projection was not done and therefore sequenced specimens in the genetic diversity map are coloured in gray (left). Specimen identification (ID or cf.) and source (newly sequenced or retrieved) are represented by colours, while specimen attribute (terra typica, type locality, type specimen or faunistic novelty) is represented by the shape (right). Sequences: ID = 2, cf. = 0; maximum p-distance: strict = NA, less strict = 1.8 %.

Haplotype network analysis of *Proformica striaticeps* was not possible.

Rossomyrmex

Rossomyrmex minuchae

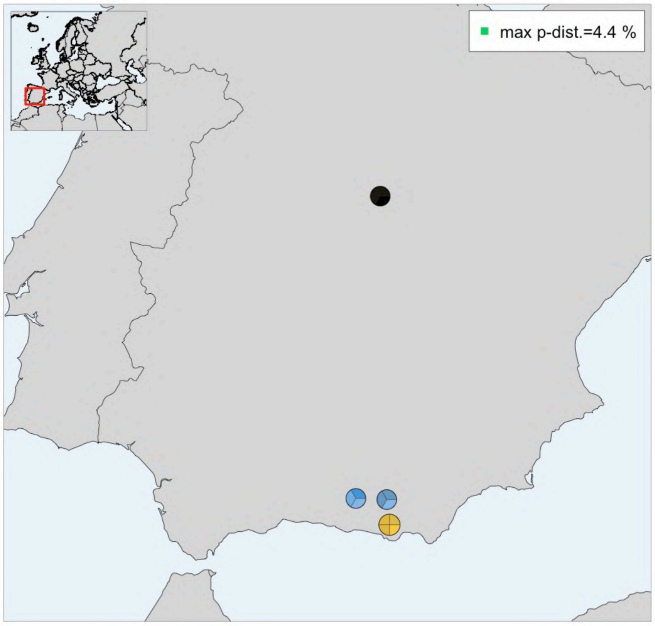

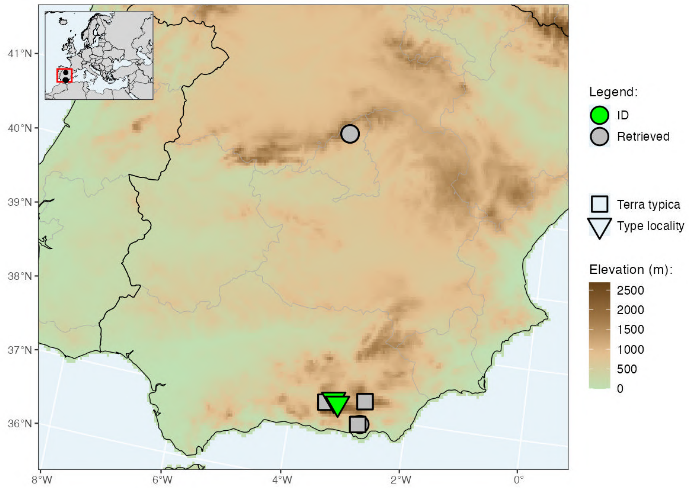

Figure 566: Genetic diversity map of *Rossomyrmex minuchae* Tinaut, 1981. Nearby localities of sequenced specimens are merged in pies (left). Colours match the bidimensional colour space of the PCoA projection (Fig. 566 left) of p-dist between sequences (dots). Specimen identification (ID or cf.) and source (newly sequenced or retrieved) are represented by colours, while specimen attribute (terra typica, type locality, type specimen or faunistic novelty) is represented by the shape (right). Sequences: ID = 13, cf. = 0; maximum p-distance: strict = 2.4 %, less strict = 4.4 %.

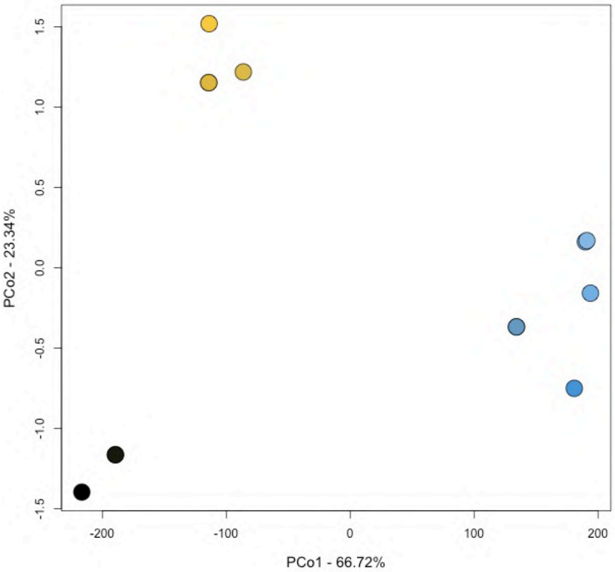

Figure 567: PCoA based on pairwise p-distances between *Rossomyrmex minuchae* sequences (left). Colours match a bidimensional colour space. Haplotype network analysis of *Rossomyrmex minuchae* was not possible.

Solenopsis

Solenopsis abdita

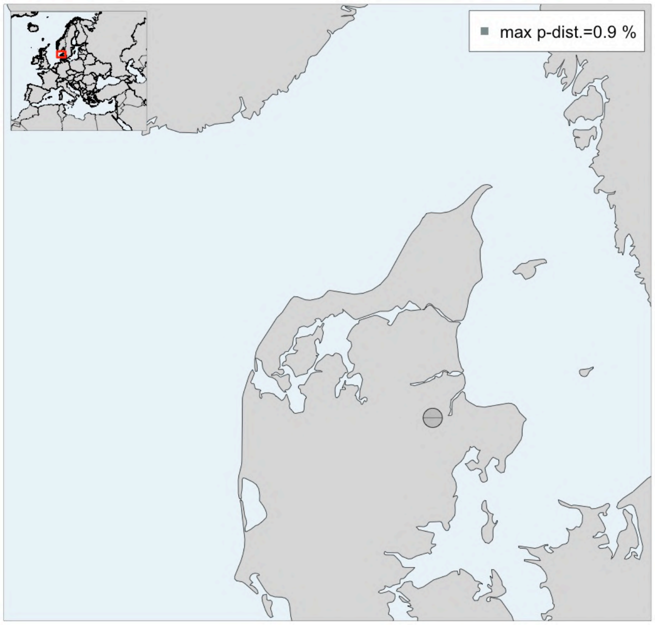

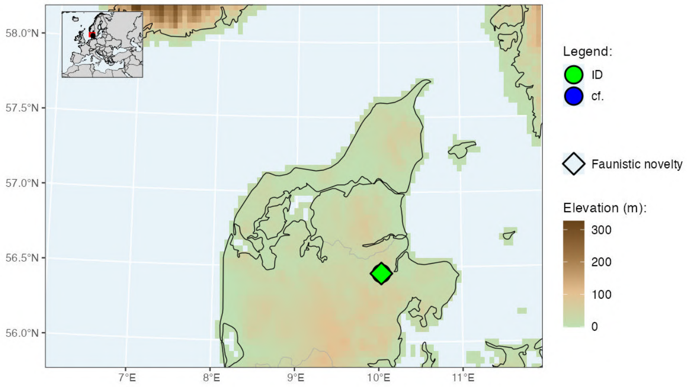

Figure 568: Genetic diversity map of *Solenopsis abdita* Thompson, 1989. PCoA projection was not done and therefore sequenced specimens in the genetic diversity map are coloured in gray (left). Specimen identification (ID or cf.) and source (newly sequenced or retrieved) are represented by colours, while specimen attribute (terra typica, type locality, type specimen or faunistic novelty) is represented by the shape (right). Sequences: ID = 12, cf. = 1; maximum p-distance: strict = NA, less strict = 0.9 %.

The species is reported for the first time in Denmark.

Haplotype network analysis of *Solenopsis abdita* was not possible.

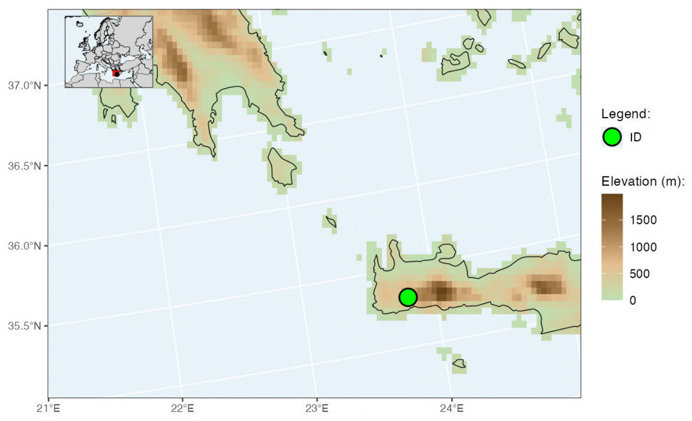

Figure 569: Map of *Solenopsis crivellarii* Menozzi, 1936. Due to the presence of a single sequence, the genetic diversity map and the PCoA projection were not done. Specimen identification (ID or cf.) and source (newly sequenced or retrieved) are represented by colours, while specimen attribute (terra typica, type locality, type specimen or faunistic novelty) is represented by the shape. Sequences: ID = 1, cf. = 0; maximum p-distance: strict = NA, less strict = NA.

Haplotype network analysis of *Solenopsis crivellarii* was not possible.

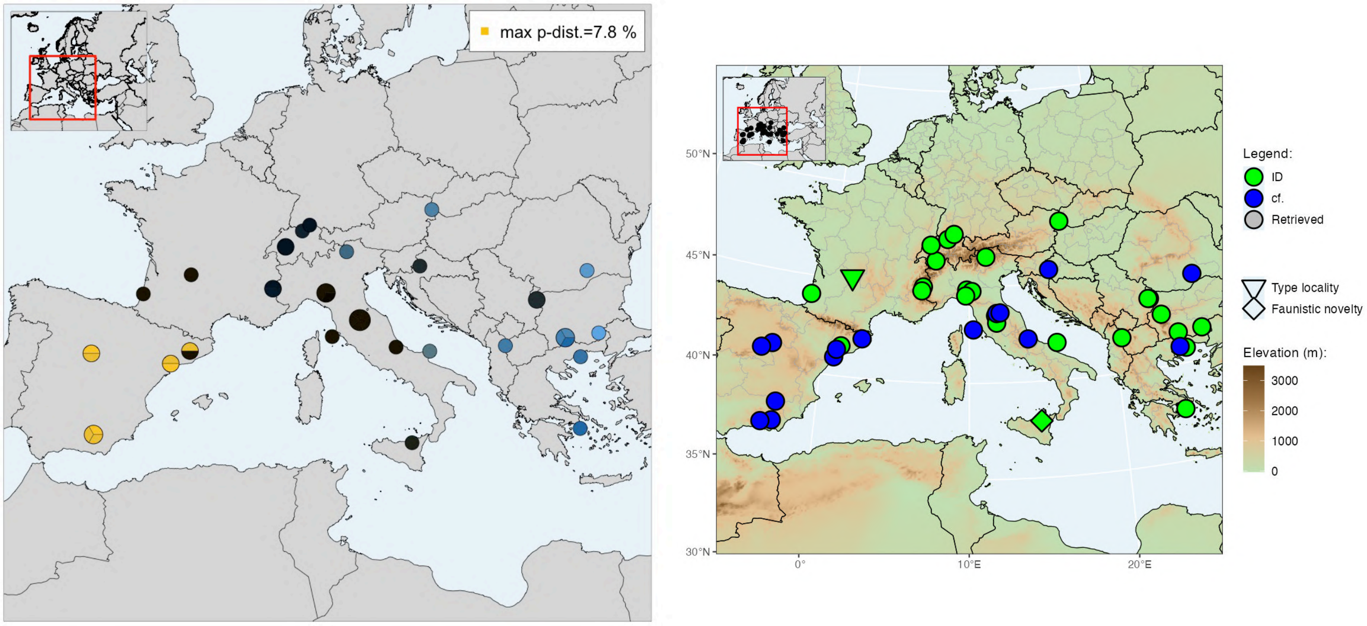

Figure 570: Genetic diversity map of *Solenopsis fugax* (Latreille, 1798). Nearby localities of sequenced specimens are merged in pies (left). Colours match the bidimensional colour space of the PCoA projection (Fig. 570 left) of p-dist between sequences (dots). Specimen identification (ID or cf.) and source (newly sequenced or retrieved) are represented by colours, while specimen attribute (terra typica, type locality, type specimen or faunistic novelty) is represented by the shape (right). Sequences: ID = 28, cf. = 15; maximum p-distance: strict = 6.3 %, less strict = 7.8 %.

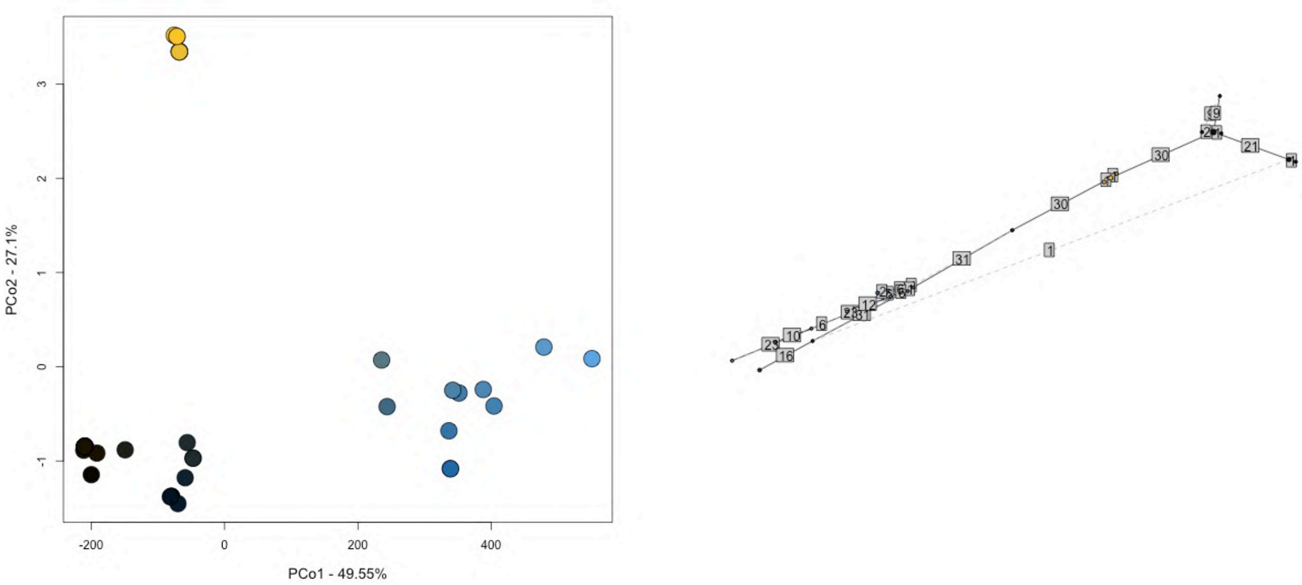

Figure 571: PCoA based on pairwise p-distances between *Solenopsis fugax* sequences (left). Colours match a bidimensional colour space. Haplotype network of *Solenopsis fugax* (right). Sequences > 599 bp: ID = 27, cf. = 15.

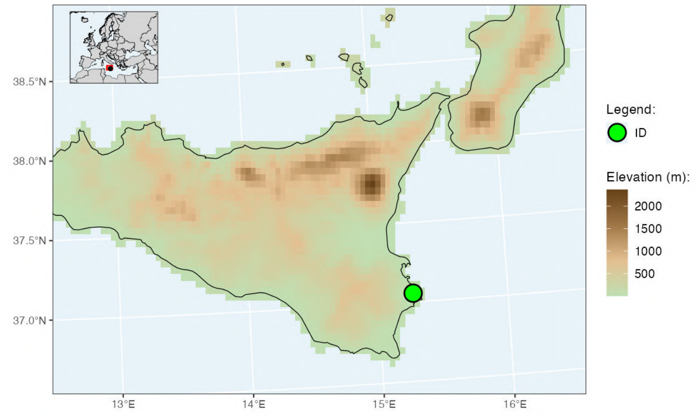

Figure 572: Map of *Solenopsis invicta* Buren, 1972. Due to the presence of a single sequence, the genetic diversity map and the PCoA projection were not done. Specimen identification (ID or cf.) and source (newly sequenced or retrieved) are represented by colours, while specimen attribute (terra typica, type locality, type specimen or faunistic novelty) is represented by the shape. Sequences: ID = 7, cf. = 0; maximum p-distance: strict = NA, less strict = NA.

Haplotype network analysis of *Solenopsis invicta* was not possible.

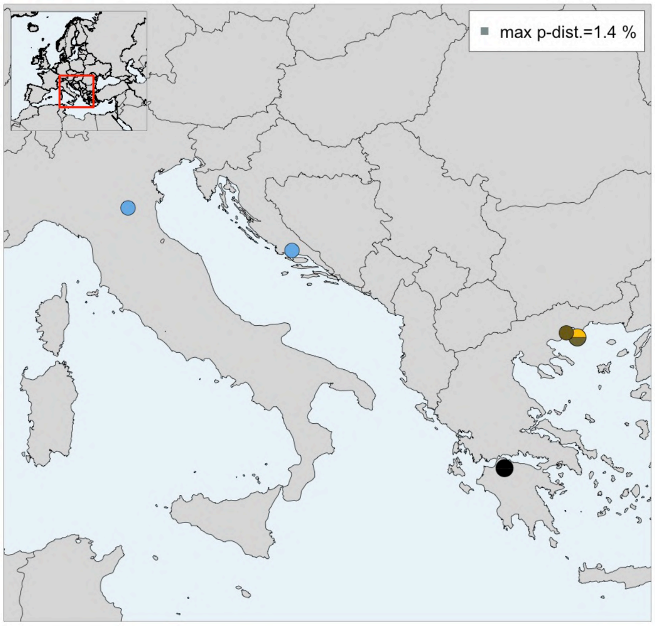

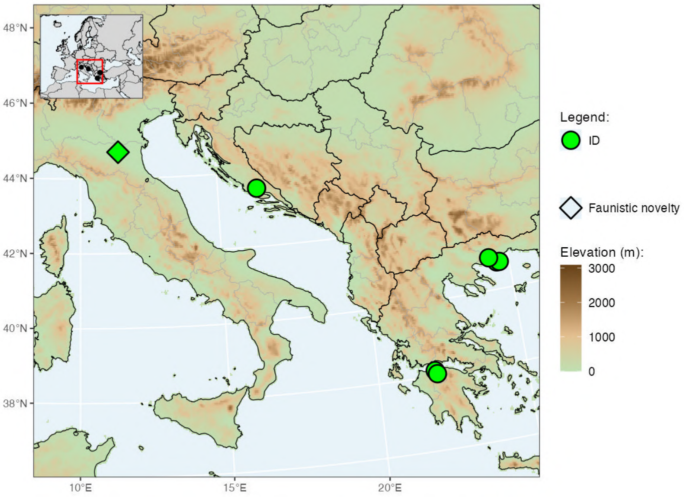

Figure 573: Genetic diversity map of *Solenopsis juliae* (Arakelian, 1991). Nearby localities of sequenced specimens are merged in pies (left). Colours match the bidimensional colour space of the PCoA projection (Fig. 573 left) of p-dist between sequences (dots). Specimen identification (ID or cf.) and source (newly sequenced or retrieved) are represented by colours, while specimen attribute (terra typica, type locality, type specimen or faunistic novelty) is represented by the shape (right). Sequences: ID = 7, cf. = 0; maximum p-distance: strict = 1.4 %, less strict = 1.4 %.

The species is reported for the first time in Italy.

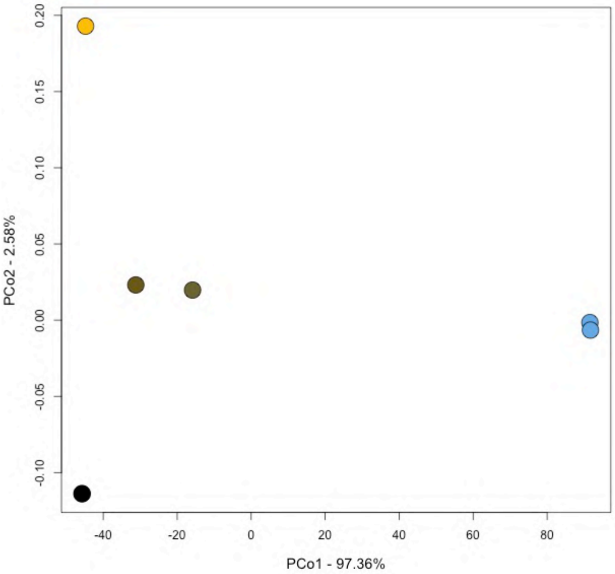

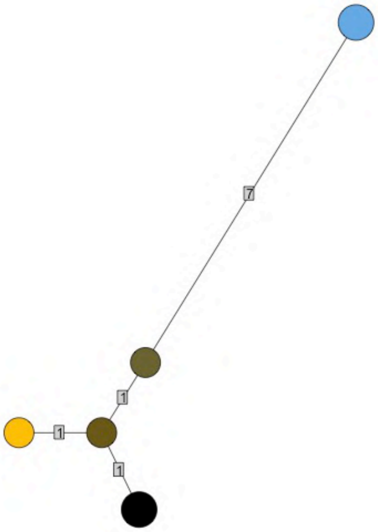

Figure 574: PCoA based on pairwise p-distances between *Solenopsis juliae* sequences (left). Colours match a bidimensional colour space. Haplotype network of *Solenopsis juliae* (right). Sequences > 599 bp: ID = 7, cf. = 0.

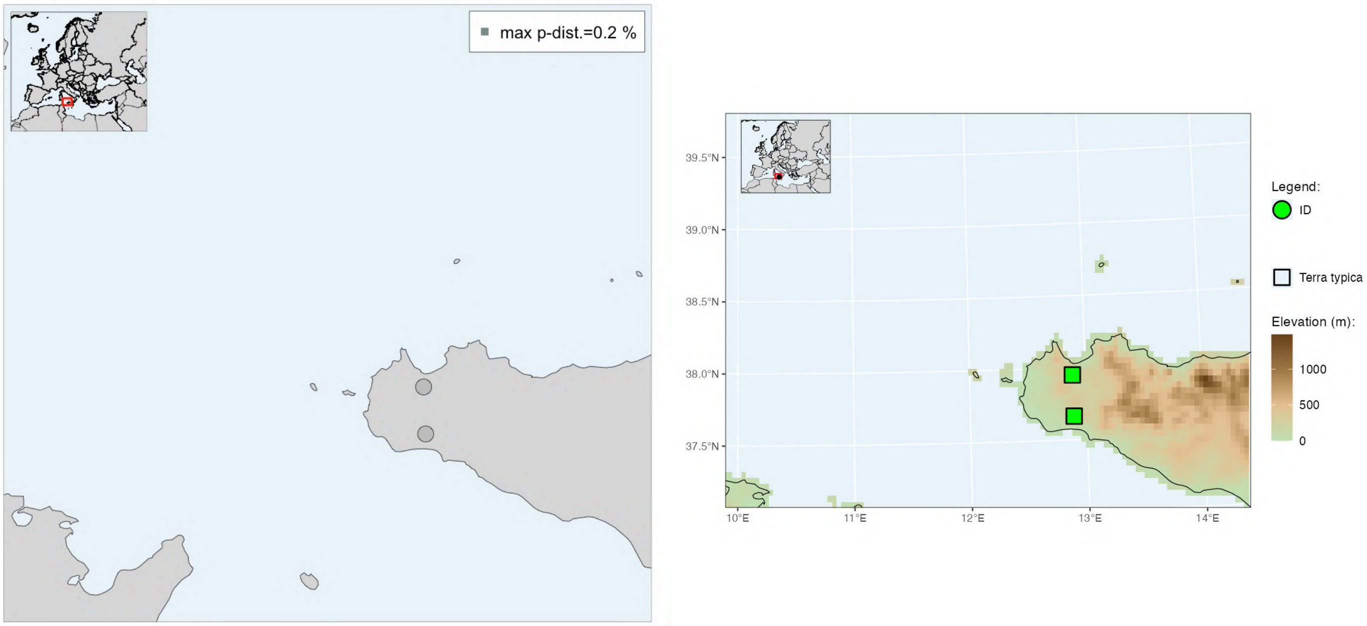

Figure 575: Genetic diversity map of *Solenopsis latro* Forel, 1894. PCoA projection was not done and therefore sequenced specimens in the genetic diversity map are coloured in gray (left). Specimen identification (ID or cf.) and source (newly sequenced or retrieved) are represented by colours, while specimen attribute (terra typica, type locality, type specimen or faunistic novelty) is represented by the shape (right). Sequences: ID = 2, cf. = 0; maximum p-distance: strict = NA, less strict = 0.2 %.

Haplotype network analysis of *Solenopsis latro* was not possible.

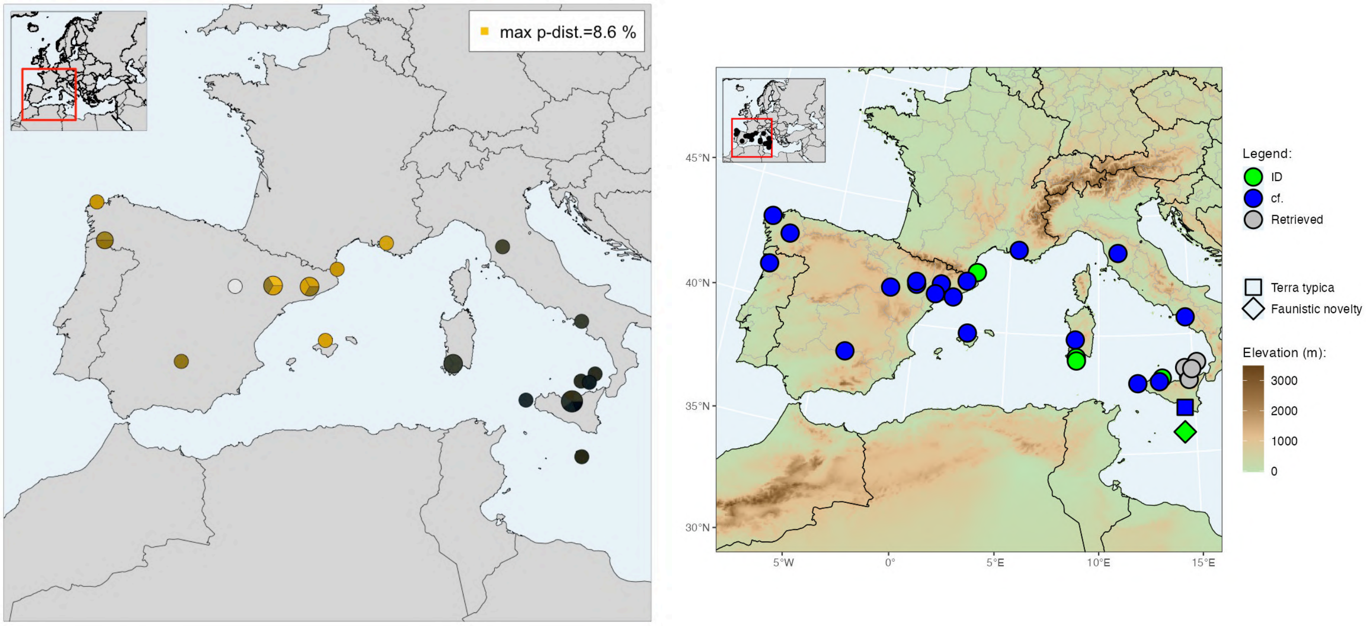

Figure 576: Genetic diversity map of *Solenopsis lusitanica* Emery, 1915. Nearby localities of sequenced specimens are merged in pies (left). Colours match the bidimensional colour space of the PCoA projection (Fig. 576 left) of p-dist between sequences (dots). Specimen identification (ID or cf.) and source (newly sequenced or retrieved) are represented by colours, while specimen attribute (terra typica, type locality, type specimen or faunistic novelty) is represented by the shape (right). Sequences: ID = 11, cf. = 19; maximum p-distance: strict = 3.2 %, less strict = 8.6 %.

The species is reported for the first time in Malta.

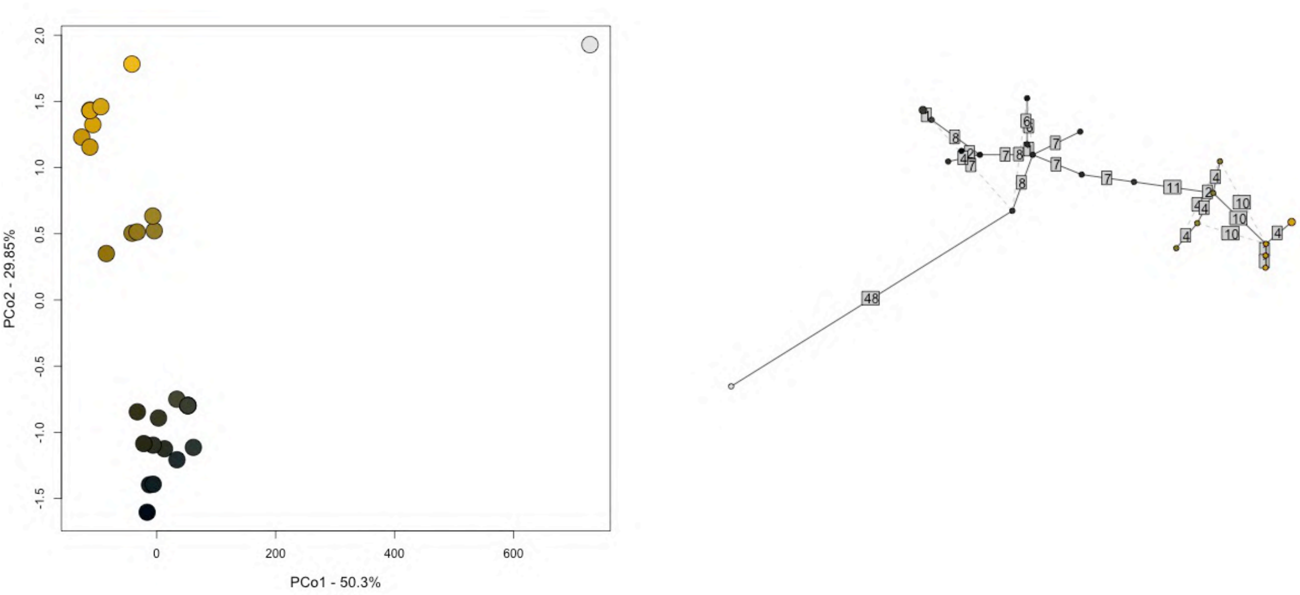

Figure 577: PCoA based on pairwise p-distances between *Solenopsis lusitanica* sequences (left). Colours match a bidimensional colour space. Haplotype network of *Solenopsis lusitanica* (right). Sequences > 599 bp: ID = 11, cf. = 17.

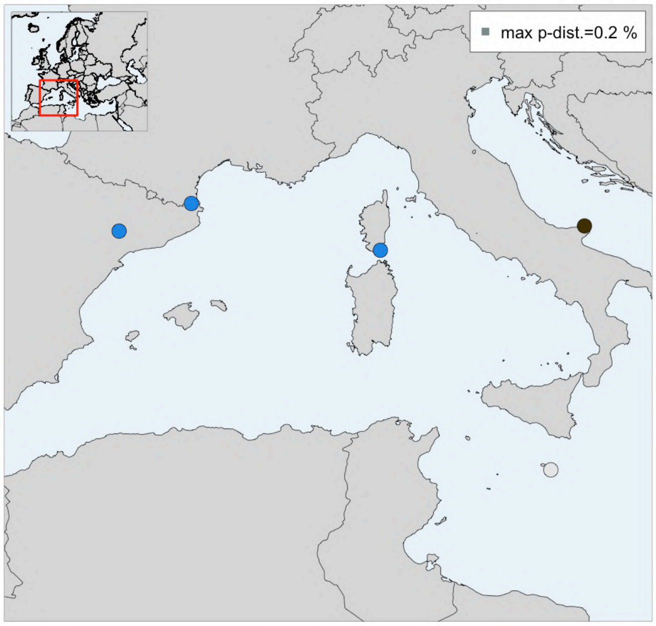

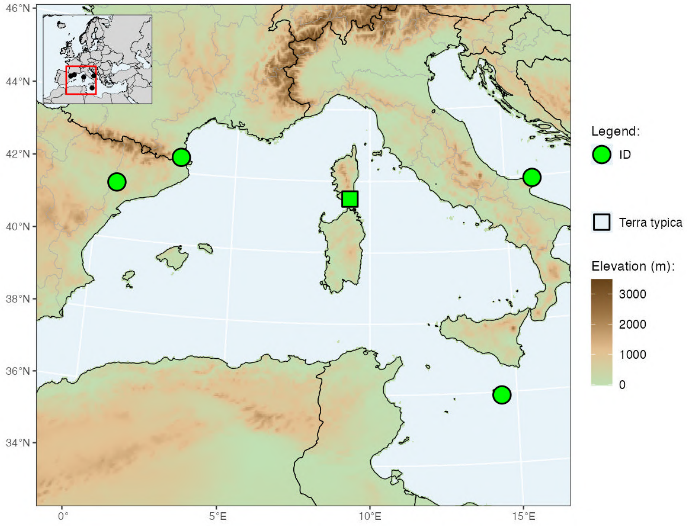

Figure 578: Genetic diversity map of *Solenopsis orbula* Emery, 1875. Nearby localities of sequenced specimens are merged in pies (left). Colours match the bidimensional colour space of the PCoA projection (Fig. 578 left) of p-dist between sequences (dots). Specimen identification (ID or cf.) and source (newly sequenced or retrieved) are represented by colours, while specimen attribute (terra typica, type locality, type specimen or faunistic novelty) is represented by the shape (right). Sequences: ID = 6, cf. = 0; maximum p-distance: strict = 0.2 %, less strict = 0.2 %.

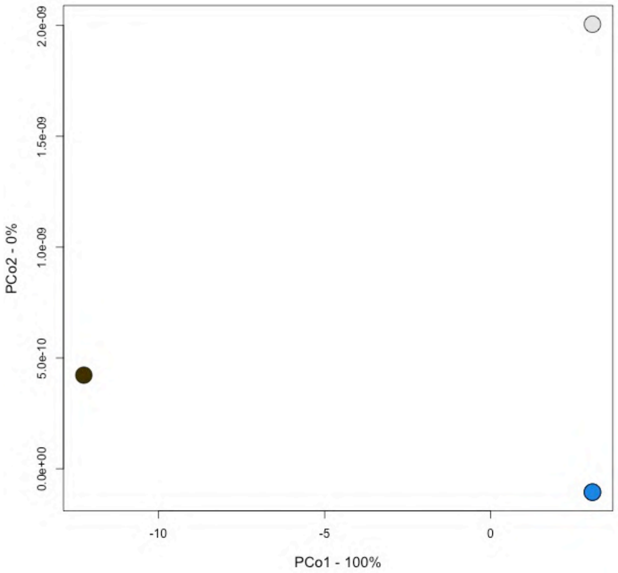

Figure 579: PCoA based on pairwise p-distances between *Solenopsis orbula* sequences (left). Colours match a bidimensional colour space. Haplotype network analysis of *Solenopsis orbula* was not possible.

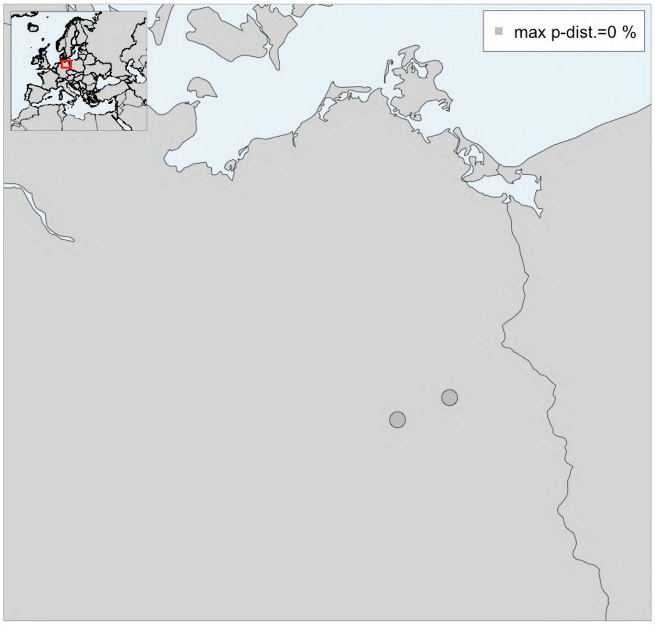

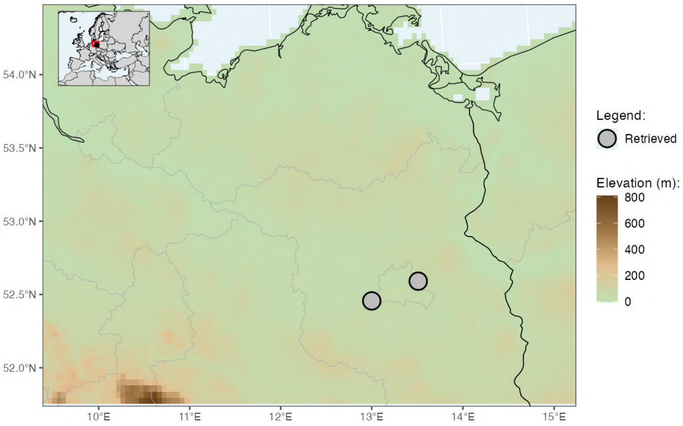

Figure 580: Genetic diversity map of *Solenopsis texana* Emery, 1895. PCoA projection was not done and therefore sequenced specimens in the genetic diversity map are coloured in gray (left). Specimen identification (ID or cf.) and source (newly sequenced or retrieved) are represented by colours, while specimen attribute (terra typica, type locality, type specimen or faunistic novelty) is represented by the shape (right). Sequences: ID = 2, cf. = 0; maximum p-distance: strict = NA, less strict = 0 %.

Haplotype network analysis of *Solenopsis texana* was not possible.

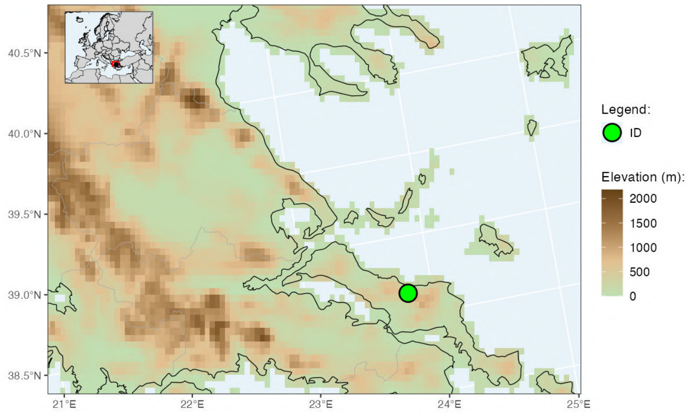

Figure 581: Map of *Solenopsis wolfi* Emery, 1915. Due to the presence of a single sequence, the genetic diversity map and the PCoA projection were not done. Specimen identification (ID or cf.) and source (newly sequenced or retrieved) are represented by colours, while specimen attribute (terra typica, type locality, type specimen or faunistic novelty) is represented by the shape. Sequences: ID = 1, cf. = 0; maximum p-distance: strict = NA, less strict = NA.

Haplotype network analysis of *Solenopsis wolfi* was not possible.

Stenamma

Stenamma debile

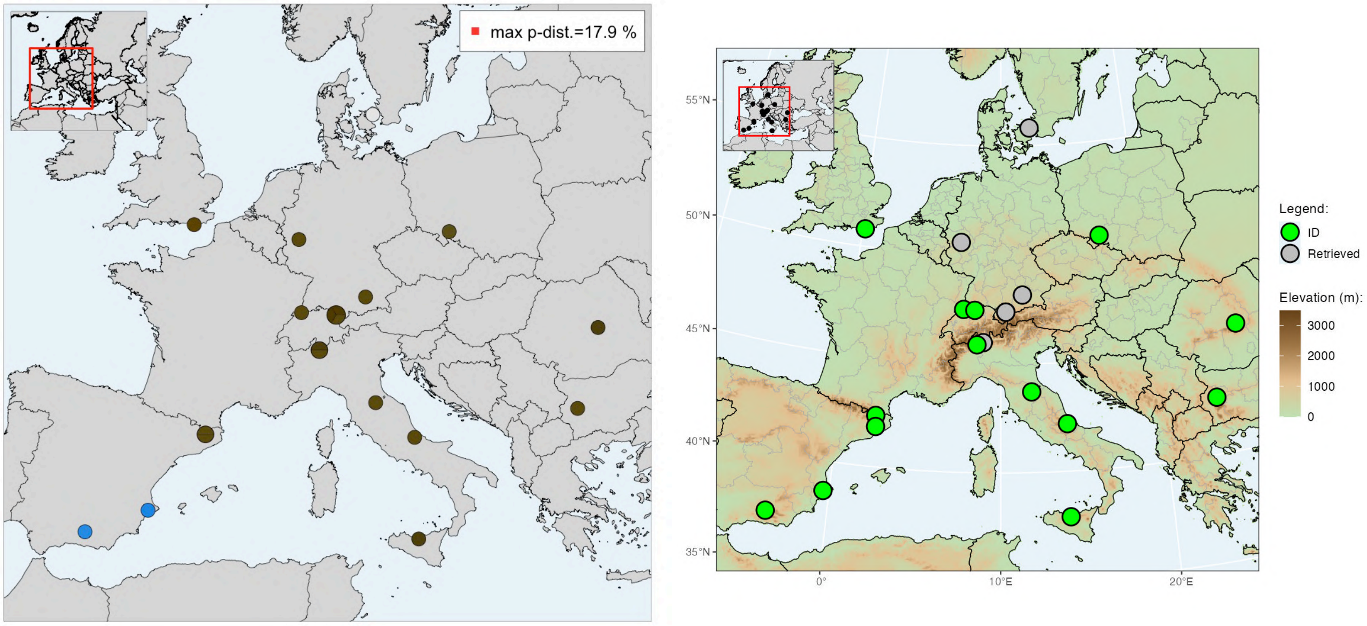

Figure 582: Genetic diversity map of *Stenamma debile* (Foerster, 1850). Nearby localities of sequenced specimens are merged in pies (left). Colours match the bidimensional colour space of the PCoA projection (Fig. 582 left) of p-dist between sequences (dots). Specimen identification (ID or cf.) and source (newly sequenced or retrieved) are represented by colours, while specimen attribute (terra typica, type locality, type specimen or faunistic novelty) is represented by the shape (right). Sequences: ID = 21, cf. = 0; maximum p-distance: strict = 17.9 %, less strict = 17.9 %.

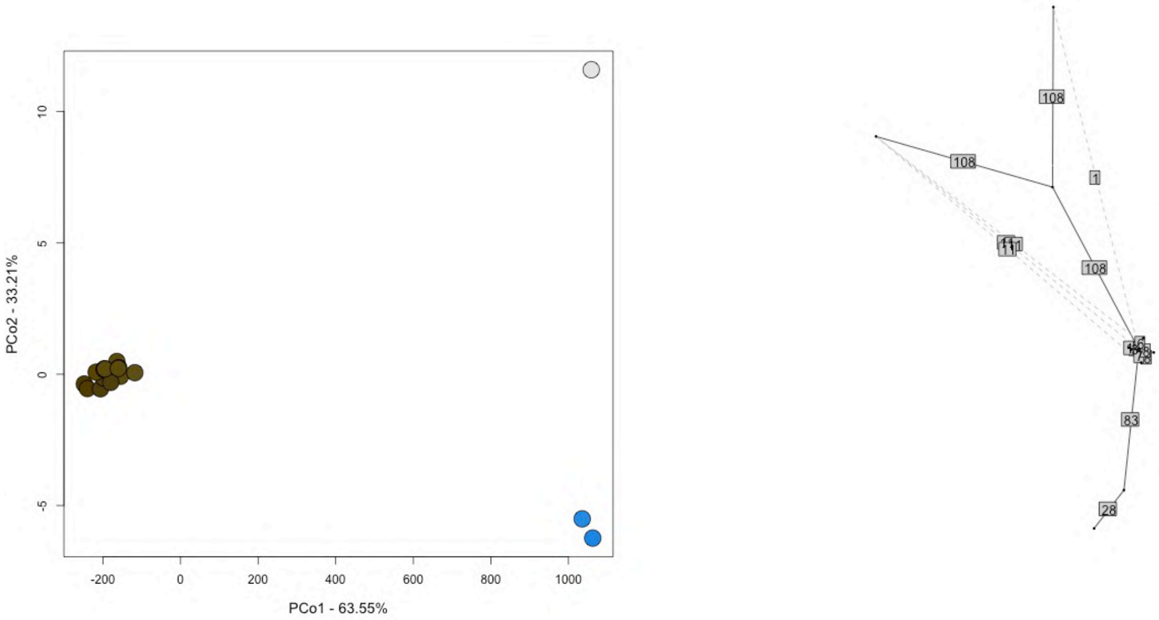

Figure 583: PCoA based on pairwise p-distances between *Stenamma debile* sequences (left). Colours match a bidimensional colour space. Haplotype network of *Stenamma debile* (right). Sequences > 599 bp: ID = 19, cf. = 0.

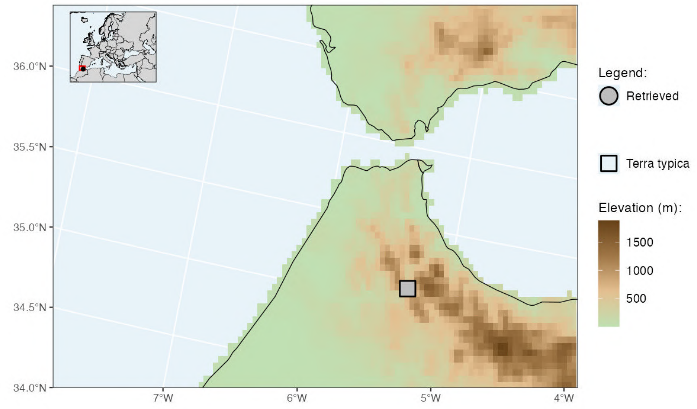

Figure 584: Map of *Stenamma punctiventre* Emery, 1908. Due to the presence of a single sequence, the genetic diversity map and the PCoA projection were not done. Specimen identification (ID or cf.) and source (newly sequenced or retrieved) are represented by colours, while specimen attribute (terra typica, type locality, type specimen or faunistic novelty) is represented by the shape. Sequences: ID = 1, cf. = 0; maximum p-distance: strict = NA, less strict = NA.

Haplotype network analysis of *Stenamma punctiventre* was not possible.

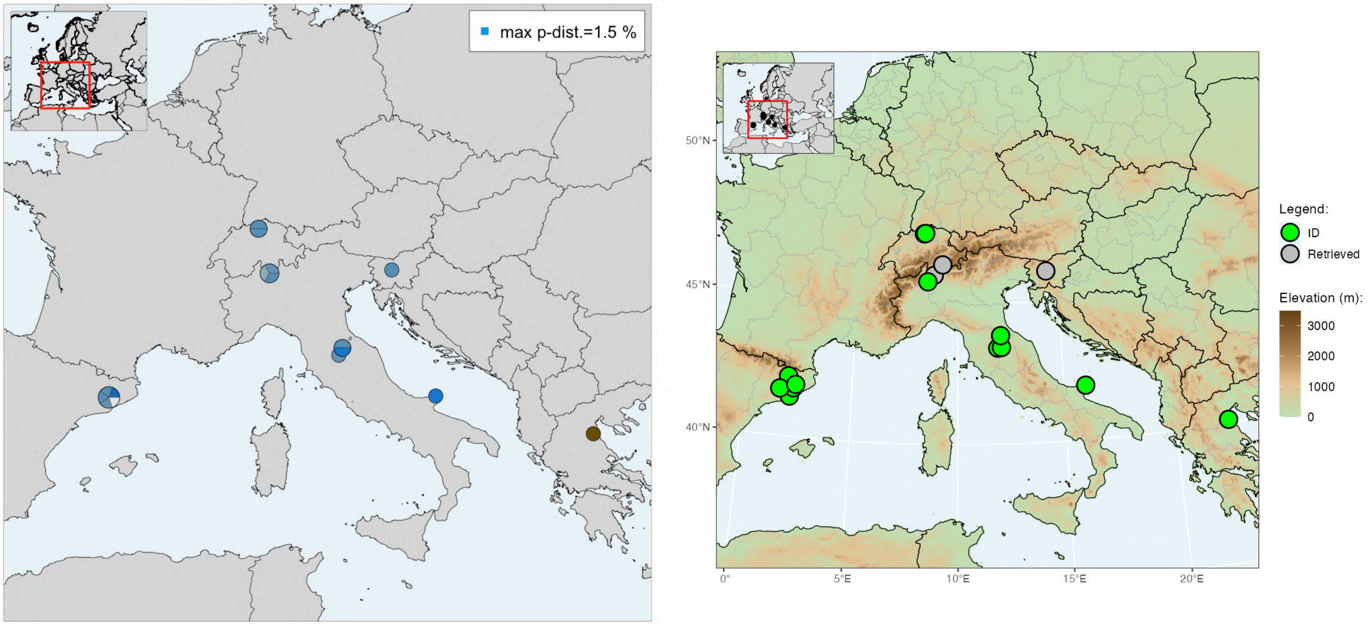

Figure 585: Genetic diversity map of *Stenamma striatulum* Emery, 1895. Nearby localities of sequenced specimens are merged in pies (left). Colours match the bidimensional colour space of the PCoA projection (Fig. 585 left) of p-dist between sequences (dots). Specimen identification (ID or cf.) and source (newly sequenced or retrieved) are represented by colours, while specimen attribute (terra typica, type locality, type specimen or faunistic novelty) is represented by the shape (right). Sequences: ID = 17, cf. = 0; maximum p-distance: strict = 1.5 %, less strict = 1.5 %.

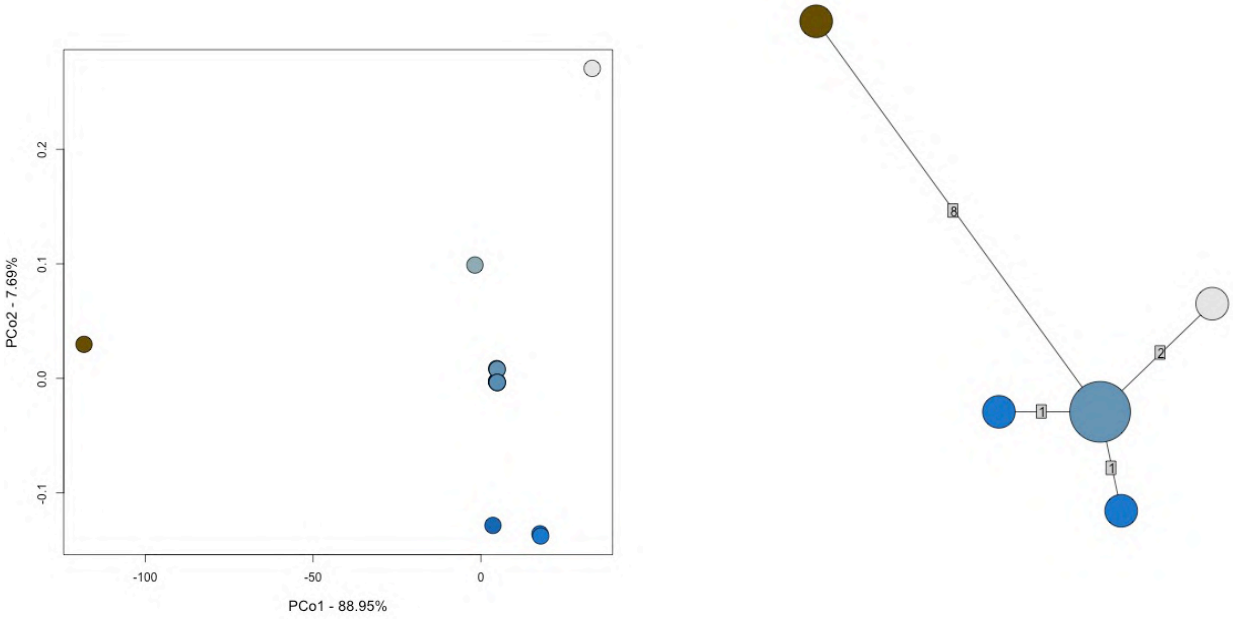

Figure 586: PCoA based on pairwise p-distances between *Stenamma striatulum* sequences (left). Colours match a bidimensional colour space. Haplotype network of *Stenamma striatulum* (right). Sequences > 599 bp: ID = 17, cf. = 0.

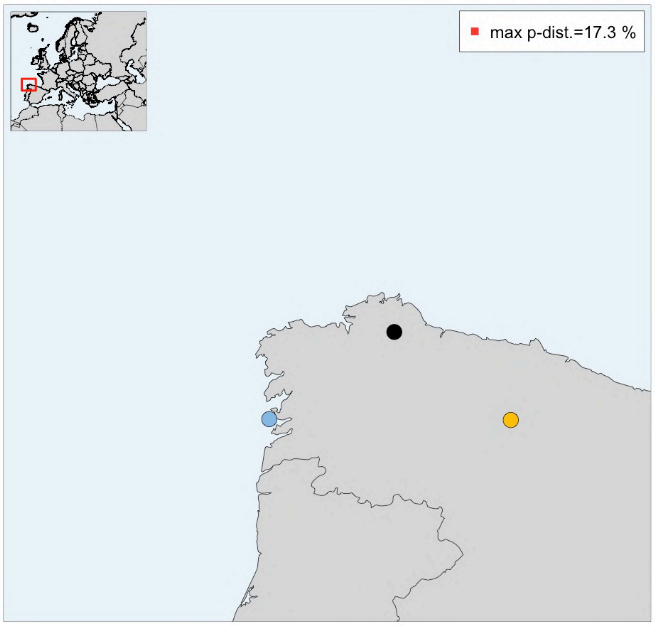

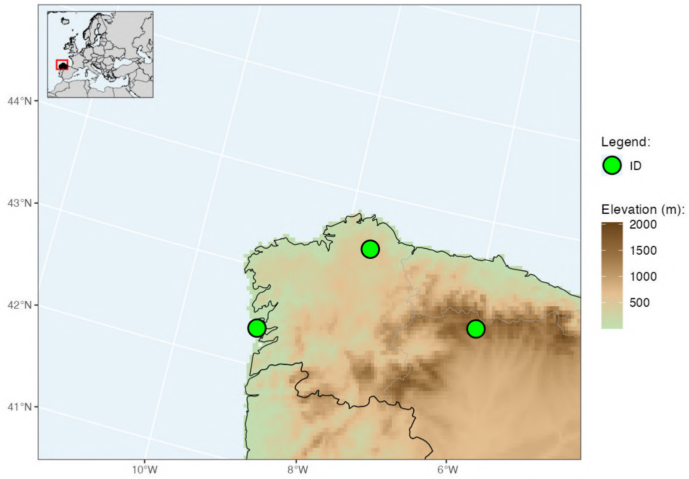

Figure 587: Genetic diversity map of *Stenamma westwoodii* Westwood, 1839. Nearby localities of sequenced specimens are merged in pies (left). Colours match the bidimensional colour space of the PCoA projection (Fig. 587 left) of p-dist between sequences (dots). Specimen identification (ID or cf.) and source (newly sequenced or retrieved) are represented by colours, while specimen attribute (terra typica, type locality, type specimen or faunistic novelty) is represented by the shape (right). Sequences: ID = 3, cf. = 0; maximum p-distance: strict = 17.3 %, less strict = 17.3 %.

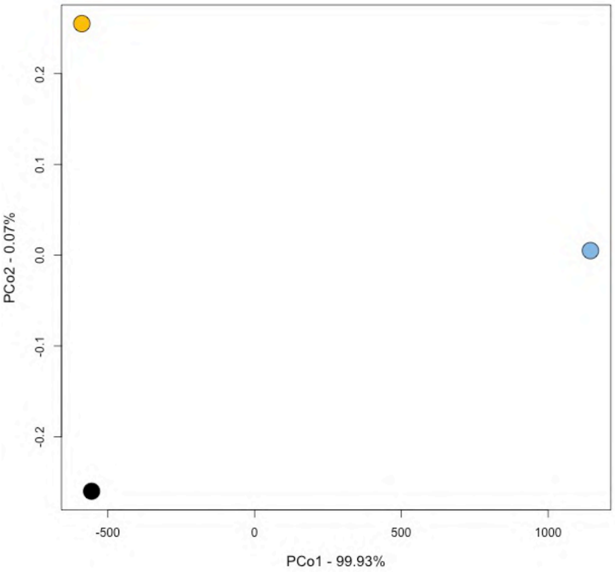

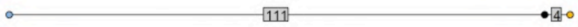

Figure 588: PCoA based on pairwise p-distances between *Stenamma westwoodii* sequences (left). Colours match a bidimensional colour space. Haplotype network of *Stenamma westwoodii* (right). Sequences > 599 bp: ID = 3, cf. = 0.

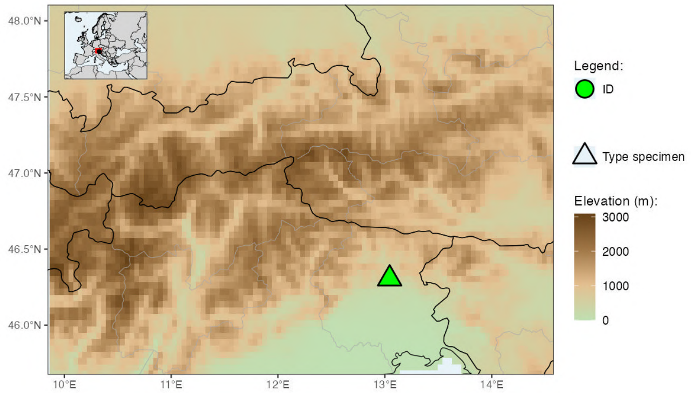

Figure 589: Map of *Stenamma zanoni* Rigato, 2011. Due to the presence of a single sequence, the genetic diversity map and the PCoA projection were not done. Specimen identification (ID or cf.) and source (newly sequenced or retrieved) are represented by colours, while specimen attribute (terra typica, type locality, type specimen or faunistic novelty) is represented by the shape. Sequences: ID = 1, cf. = 0; maximum p-distance: strict = NA, less strict = NA.

Haplotype network analysis of *Stenamma zanoni* was not possible.

Stigmatomma

Stigmatomma denticulatum

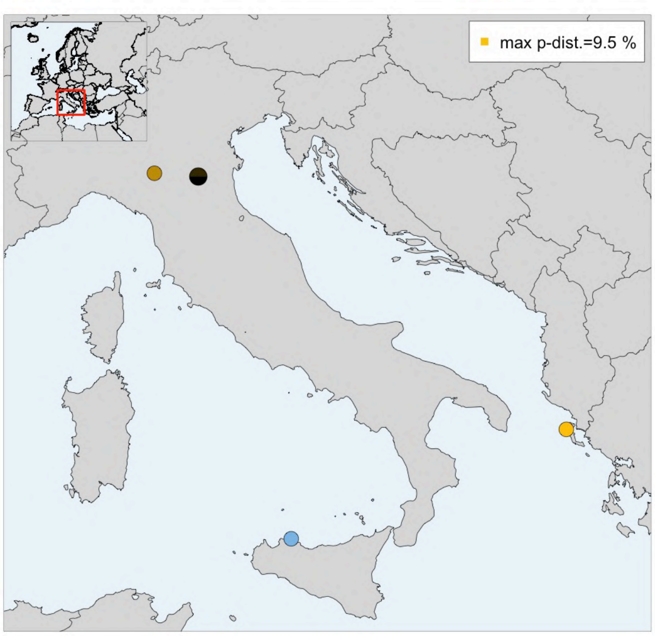

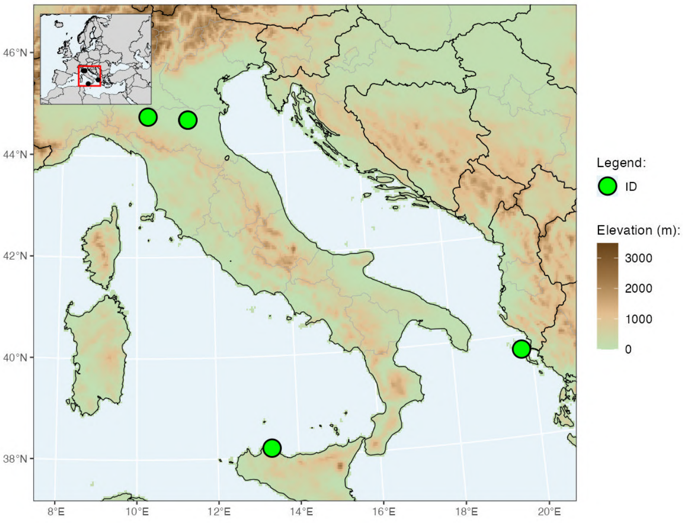

Figure 590: Genetic diversity map of *Stigmatomma denticulatum* Roger, 1859. Nearby localities of sequenced specimens are merged in pies (left). Colours match the bidimensional colour space of the PCoA projection (Fig. 590 left) of p-dist between sequences (dots). Specimen identification (ID or cf.) and source (newly sequenced or retrieved) are represented by colours, while specimen attribute (terra typica, type locality, type specimen or faunistic novelty) is represented by the shape (right). Sequences: ID = 5, cf. = 0; maximum p-distance: strict = 9.5 %, less strict = 9.5 %.

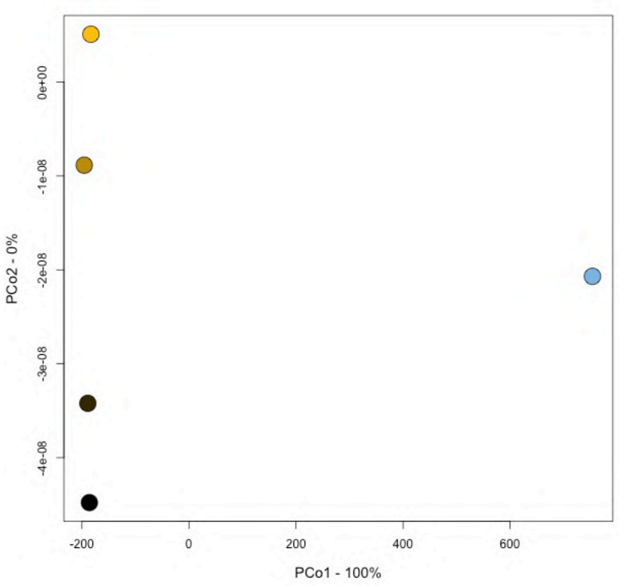

Figure 591: PCoA based on pairwise p-distances between *Stigmatomma denticulatum* sequences (left). Colours match a bidimensional colour space. Haplotype network analysis of *Stigmatomma denticulatum* was not possible.

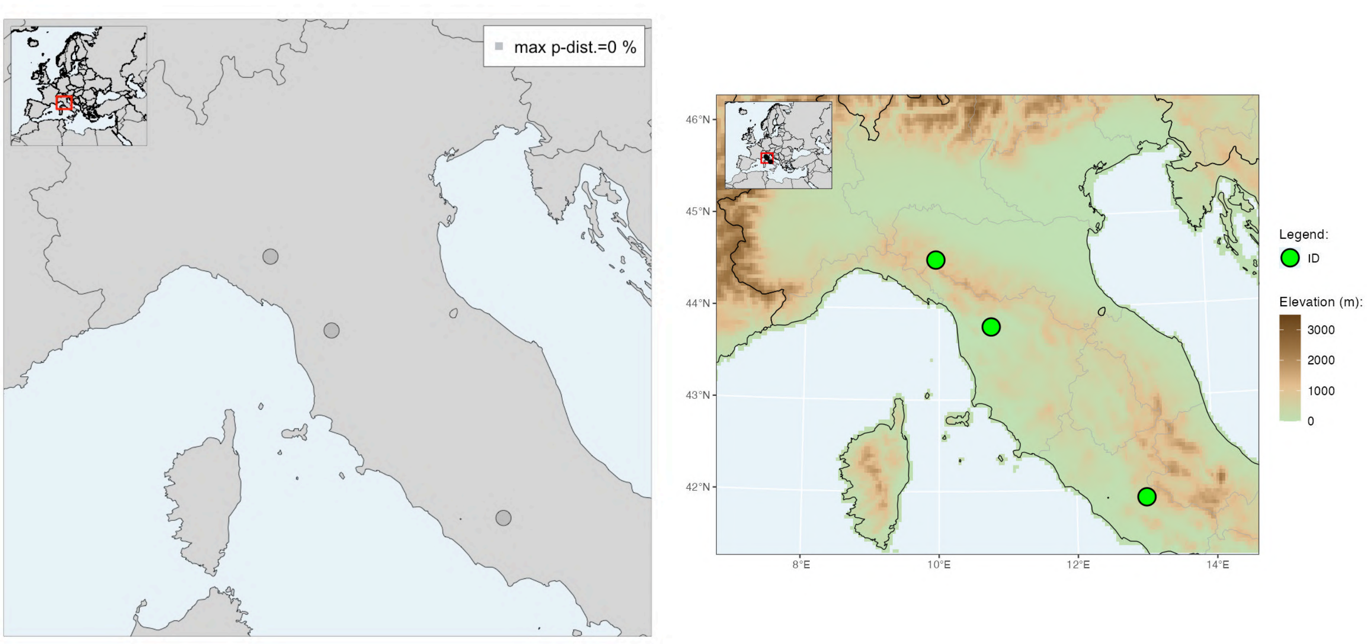

Figure 592: Genetic diversity map of *Stigmatomma impressifrons* Emery, 1869. PCoA projection was not done and therefore sequenced specimens in the genetic diversity map are coloured in gray (left). Specimen identification (ID or cf.) and source (newly sequenced or retrieved) are represented by colours, while specimen attribute (terra typica, type locality, type specimen or faunistic novelty) is represented by the shape (right). Sequences: ID = 3, cf. = 0; maximum p-distance: strict = 0 %, less strict = 0 %.

Haplotype network analysis of *Stigmatomma impressifrons* was not possible.

Strongylognathus

Strongylognathus alpinus

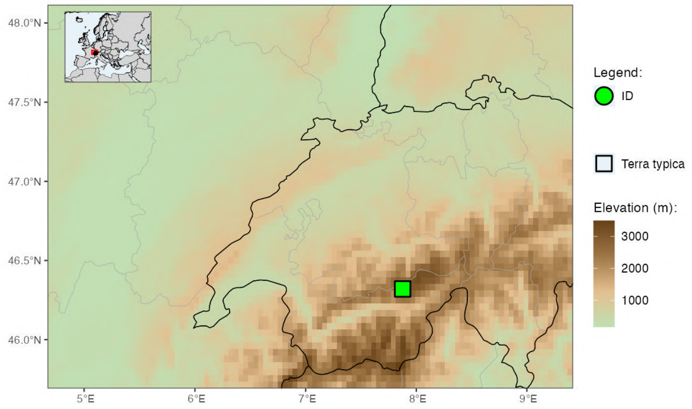

Figure 593: Map of *Strongylognathus alpinus* Wheeler, W.M., 1909. Due to the presence of a single sequence, the genetic diversity map and the PCoA projection were not done. Specimen identification (ID or cf.) and source (newly sequenced or retrieved) are represented by colours, while specimen attribute (terra typica, type locality, type specimen or faunistic novelty) is represented by the shape. Sequences: ID = 1, cf. = 0; maximum p-distance: strict = NA, less strict = NA.

Haplotype network analysis of *Strongylognathus alpinus* was not possible.

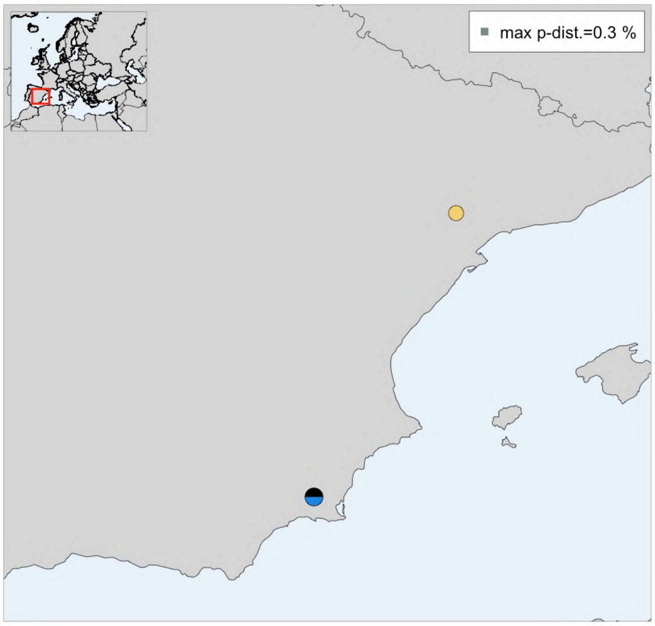

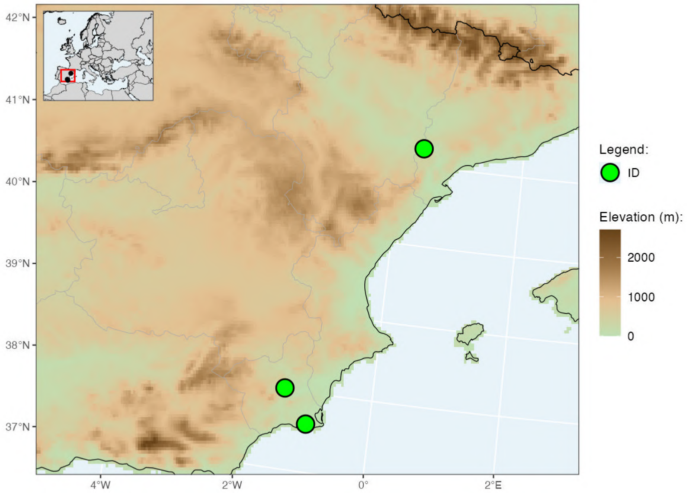

Figure 594: Genetic diversity map of *Strongylognathus caeciliae* Forel, 1897. Nearby localities of sequenced specimens are merged in pies (left). Colours match the bidimensional colour space of the PCoA projection (Fig. 594 left) of p-dist between sequences (dots). Specimen identification (ID or cf.) and source (newly sequenced or retrieved) are represented by colours, while specimen attribute (terra typica, type locality, type specimen or faunistic novelty) is represented by the shape (right). Sequences: ID = 3, cf. = 0; maximum p-distance: strict = 0.3 %, less strict = 0.3 %.

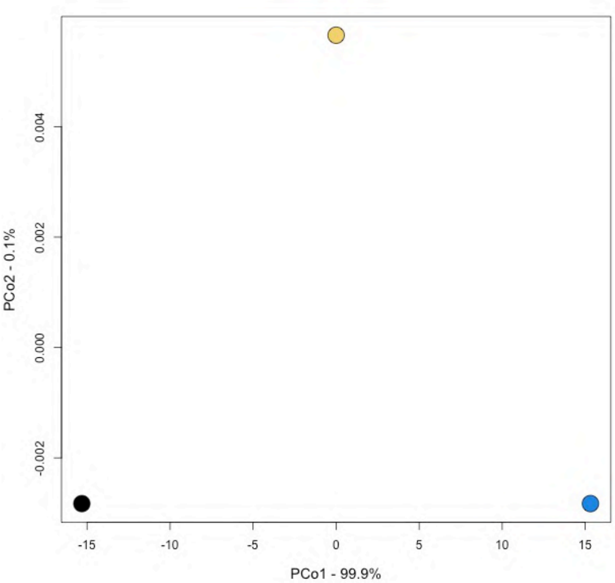

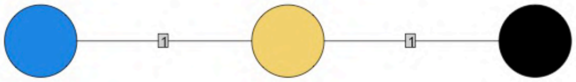

Figure 595: PCoA based on pairwise p-distances between *Strongylognathus caeciliae* sequences (left). Colours match a bidimensional colour space. Haplotype network of *Strongylognathus caeciliae* (right). Sequences > 599 bp: ID = 3, cf. = 0.

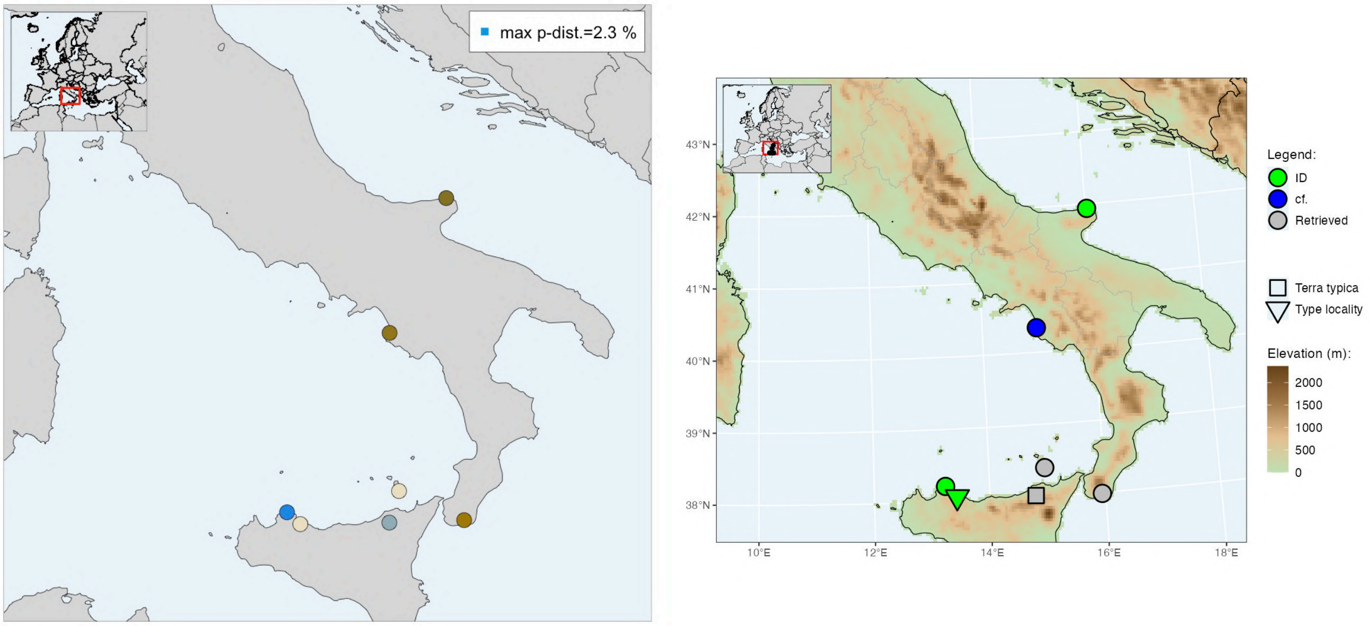

Figure 596: Genetic diversity map of *Strongylognathus destefanii* Emery, 1915. Nearby localities of sequenced specimens are merged in pies (left). Colours match the bidimensional colour space of the PCoA projection (Fig. 596 left) of p-dist between sequences (dots). Specimen identification (ID or cf.) and source (newly sequenced or retrieved) are represented by colours, while specimen attribute (terra typica, type locality, type specimen or faunistic novelty) is represented by the shape (right). Sequences: ID = 6, cf. = 1; maximum p-distance: strict = 2.3 %, less strict = 2.3 %.

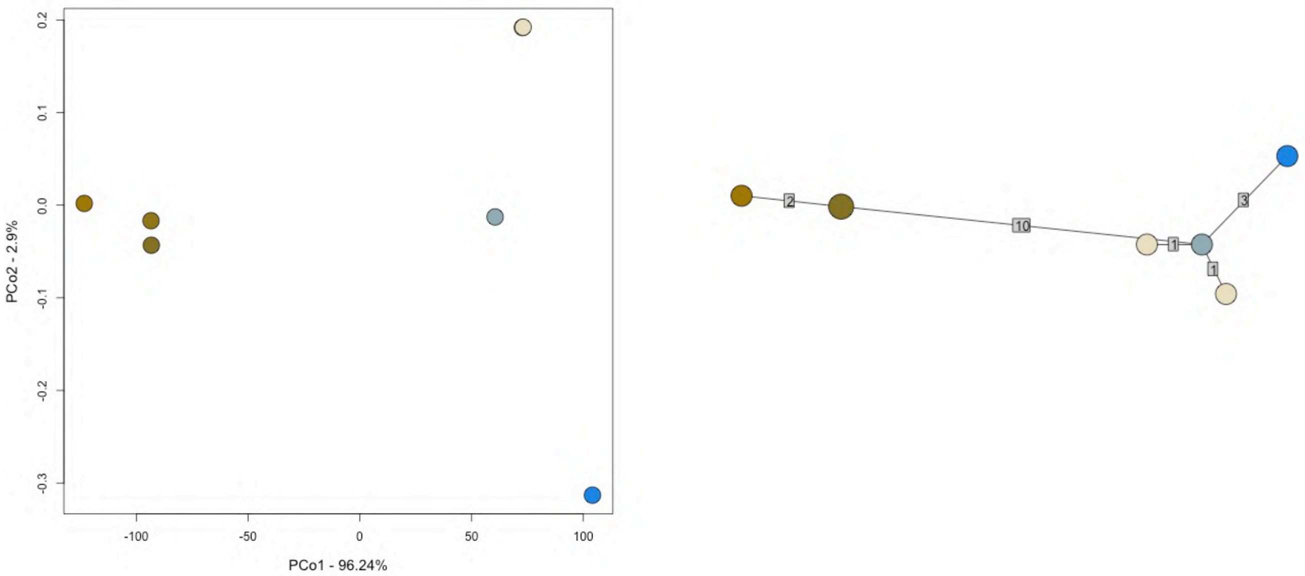

Figure 597: PCoA based on pairwise p-distances between *Strongylognathus destefanii* sequences (left). Colours match a bidimensional colour space. Haplotype network of *Strongylognathus destefanii* (right). Sequences > 599 bp: ID = 6, cf. = 1.

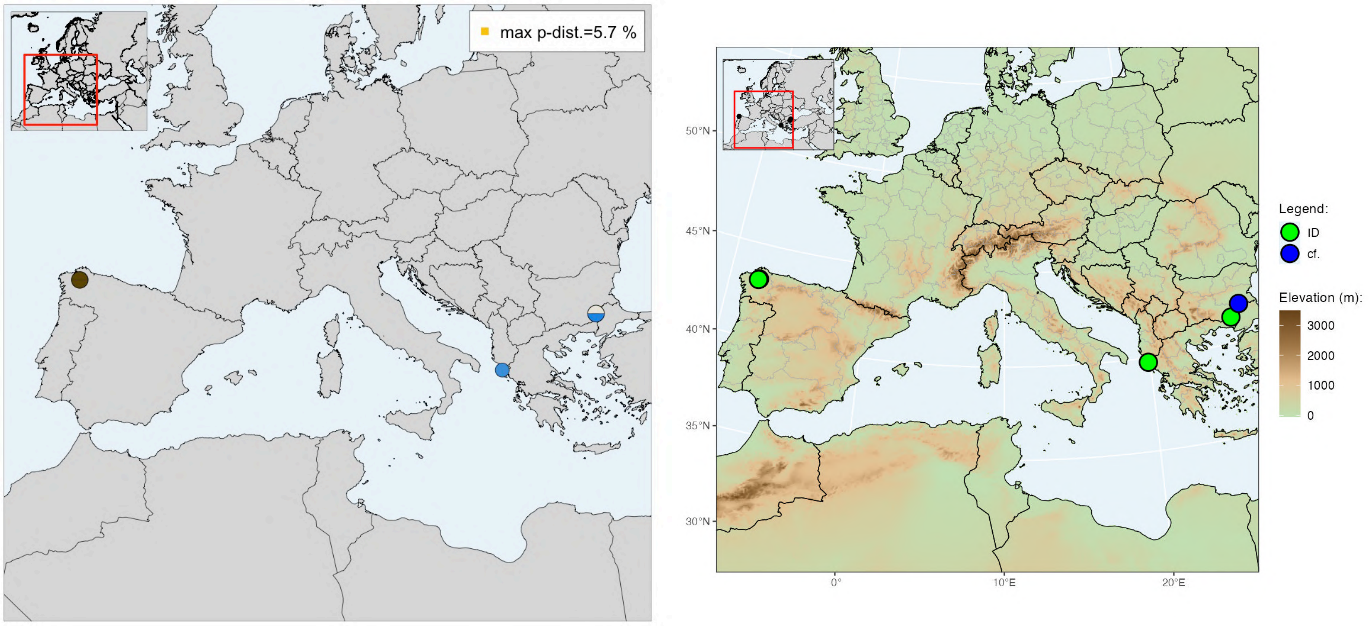

Figure 598: Genetic diversity map of *Strongylognathus huberi* Forel, 1874. Nearby localities of sequenced specimens are merged in pies (left). Colours match the bidimensional colour space of the PCoA projection (Fig. 598 left) of p-dist between sequences (dots). Specimen identification (ID or cf.) and source (newly sequenced or retrieved) are represented by colours, while specimen attribute (terra typica, type locality, type specimen or faunistic novelty) is represented by the shape (right). Sequences: ID = 4, cf. = 1; maximum p-distance: strict = 5.5 %, less strict = 5.7 %.

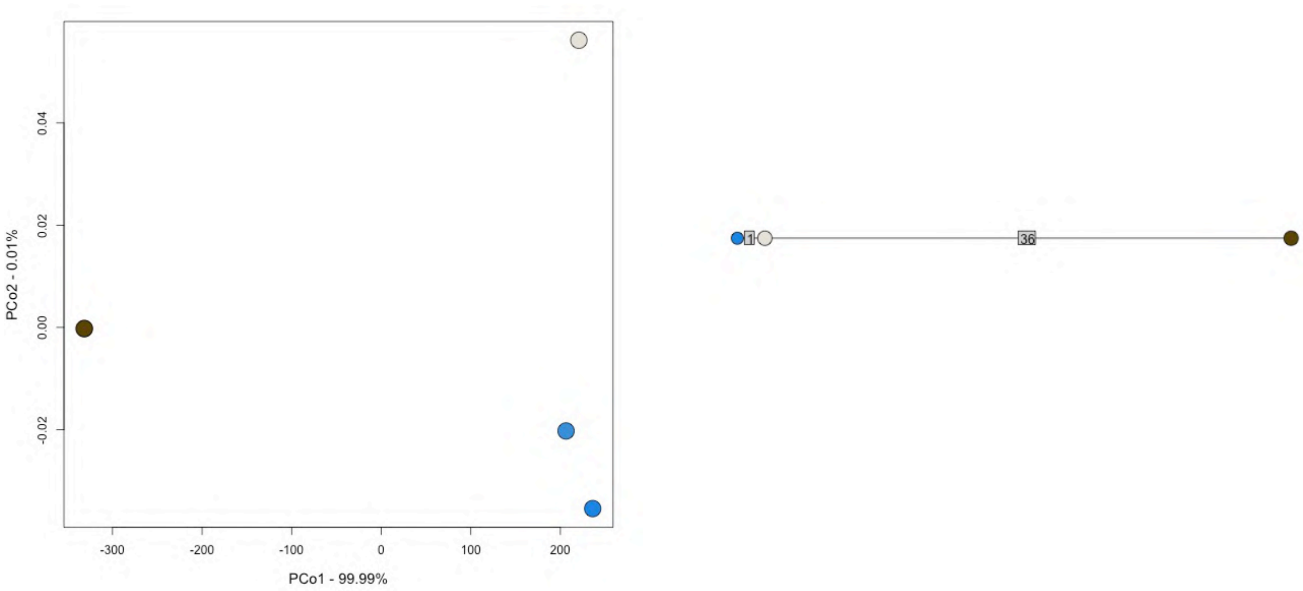

Figure 599: PCoA based on pairwise p-distances between *Strongylognathus huberi* sequences (left). Colours match a bidimensional colour space. Haplotype network of *Strongylognathus huberi* (right). Sequences > 599 bp: ID = 4, cf. = 1.

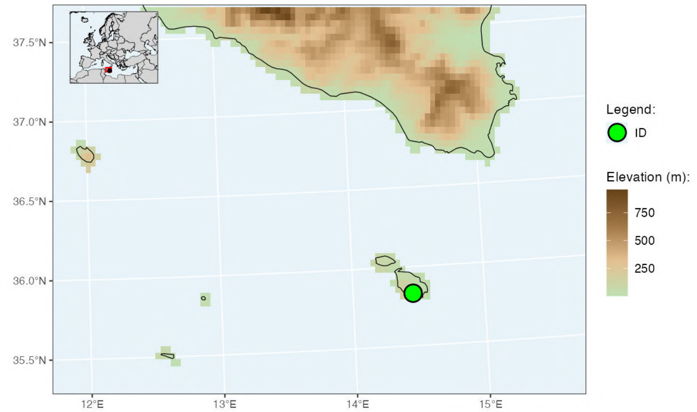

Figure 600: Map of *Strongylognathus insularis* Baroni Urbani, 1968. Due to the presence of a single sequence, the genetic diversity map and the PCoA projection were not done. Specimen identification (ID or cf.) and source (newly sequenced or retrieved) are represented by colours, while specimen attribute (terra typica, type locality, type specimen or faunistic novelty) is represented by the shape. Sequences: ID = 1, cf. = 0; maximum p-distance: strict = NA, less strict = NA.

Haplotype network analysis of *Strongylognathus insularis* was not possible.

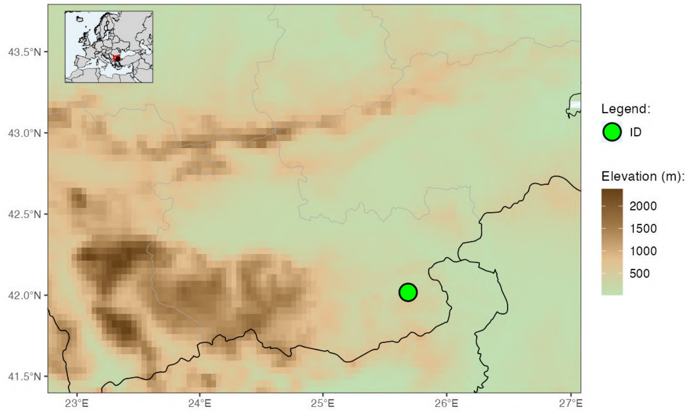

Figure 601: Map of *Strongylognathus karawajewi* Pisarski, 1966. Due to the presence of a single sequence, the genetic diversity map and the PCoA projection were not done. Specimen identification (ID or cf.) and source (newly sequenced or retrieved) are represented by colours, while specimen attribute (terra typica, type locality, type specimen or faunistic novelty) is represented by the shape. Sequences: ID = 1, cf. = 0; maximum p-distance: strict = NA, less strict = NA.

Haplotype network analysis of *Strongylognathus karawajewi* was not possible.

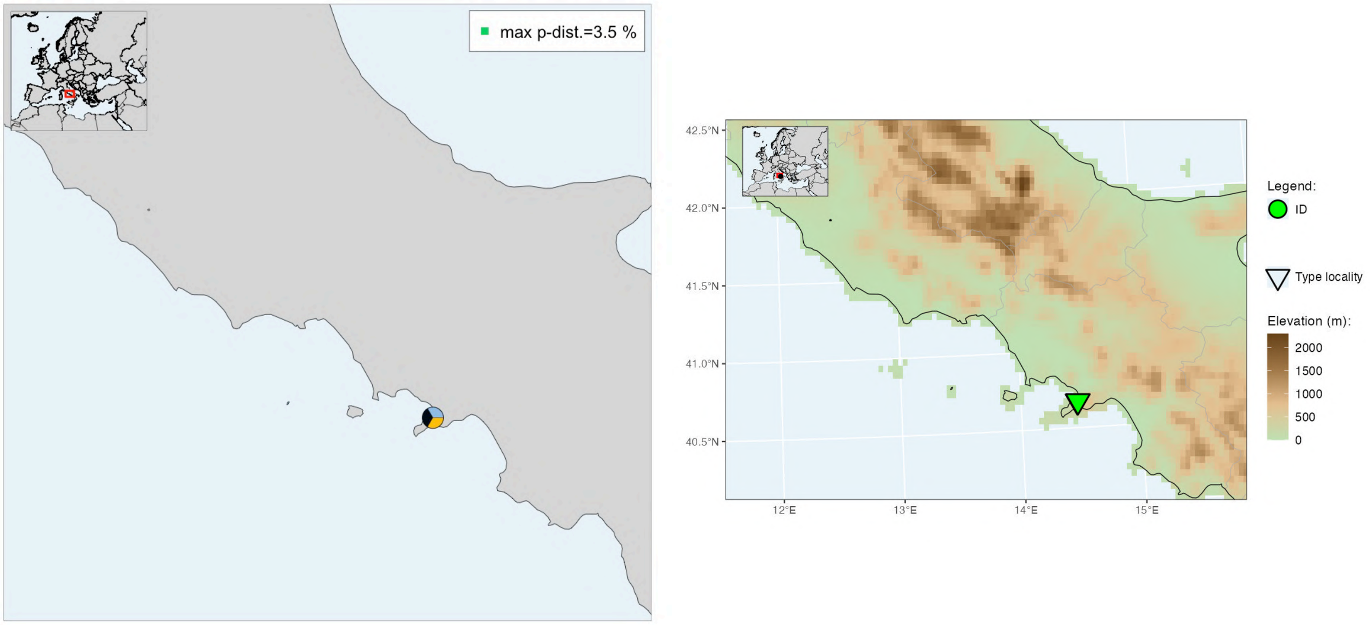

Figure 602: Genetic diversity map of *Strongylognathus pisarskii* Poldi, 1994. Nearby localities of sequenced specimens are merged in pies (left). Colours match the bidimensional colour space of the PCoA projection (Fig. 602 left) of p-dist between sequences (dots). Specimen identification (ID or cf.) and source (newly sequenced or retrieved) are represented by colours, while specimen attribute (terra typica, type locality, type specimen or faunistic novelty) is represented by the shape (right). Sequences: ID = 3, cf. = 0; maximum p-distance: strict = NA, less strict = 3.5 %.

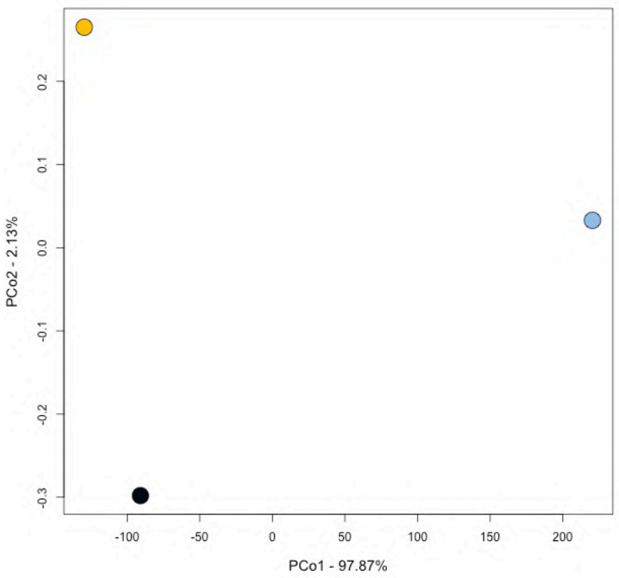

Figure 603: PCoA based on pairwise p-distances between *Strongylognathus pisarskii* sequences (left). Colours match a bidimensional colour space. Haplotype network analysis of *Strongylognathus pisarskii* was not possible.

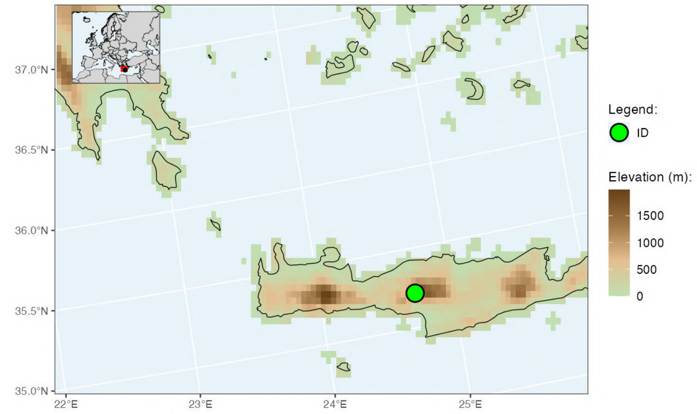

Figure 604: Map of *Strongylognathus silvestrii* Menozzi, 1936. Due to the presence of a single sequence, the genetic diversity map and the PCoA projection were not done. Specimen identification (ID or cf.) and source (newly sequenced or retrieved) are represented by colours, while specimen attribute (terra typica, type locality, type specimen or faunistic novelty) is represented by the shape. Sequences: ID = 1, cf. = 0; maximum p-distance: strict = NA, less strict = NA.

Haplotype network analysis of *Strongylognathus silvestrii* was not possible.

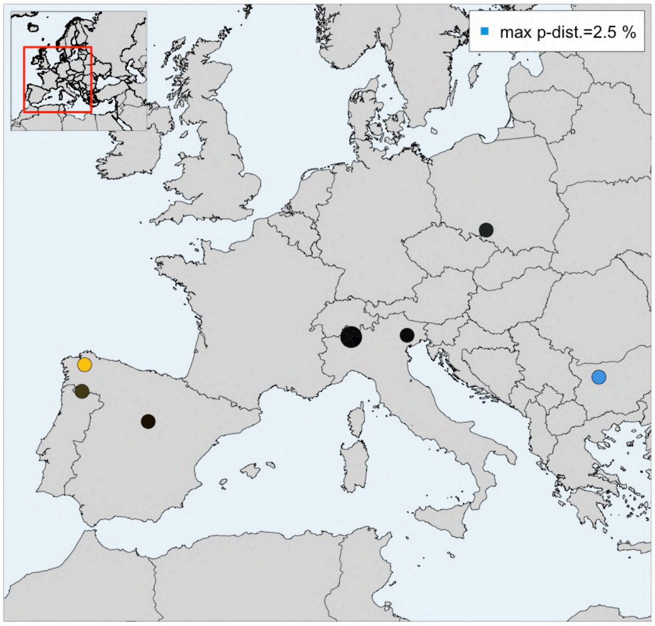

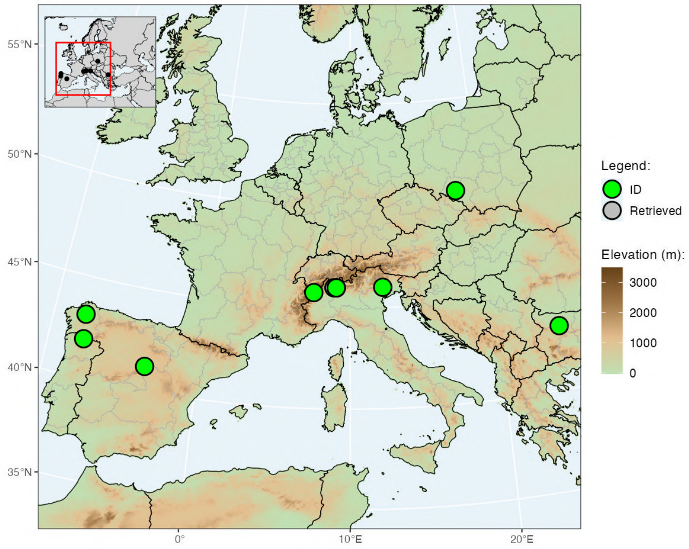

Figure 605: Genetic diversity map of *Strongylognathus testaceus* (Schenck, 1852). Nearby localities of sequenced specimens are merged in pies (left). Colours match the bidimensional colour space of the PCoA projection (Fig. 605 left) of p-dist between sequences (dots). Specimen identification (ID or cf.) and source (newly sequenced or retrieved) are represented by colours, while specimen attribute (terra typica, type locality, type specimen or faunistic novelty) is represented by the shape (right). Sequences: ID = 12, cf. = 0; maximum p-distance: strict = 2.5 %, less strict = 2.5 %.

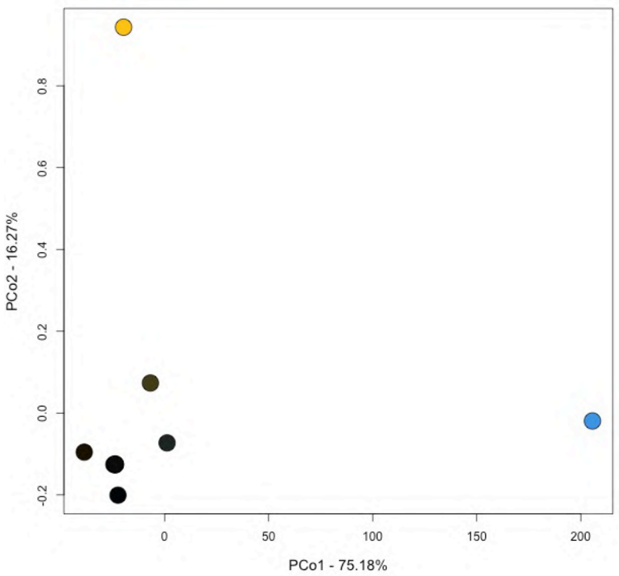

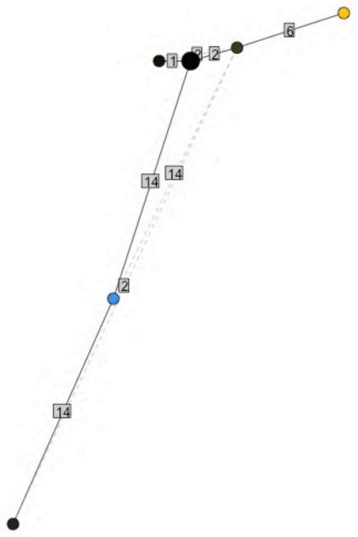

Figure 606: PCoA based on pairwise p-distances between *Strongylognathus testaceus* sequences (left). Colours match a bidimensional colour space. Haplotype network of *Strongylognathus testaceus* (right). Sequences > 599 bp: ID = 12, cf. = 0.

Strumigenys

Strumigenys argiola

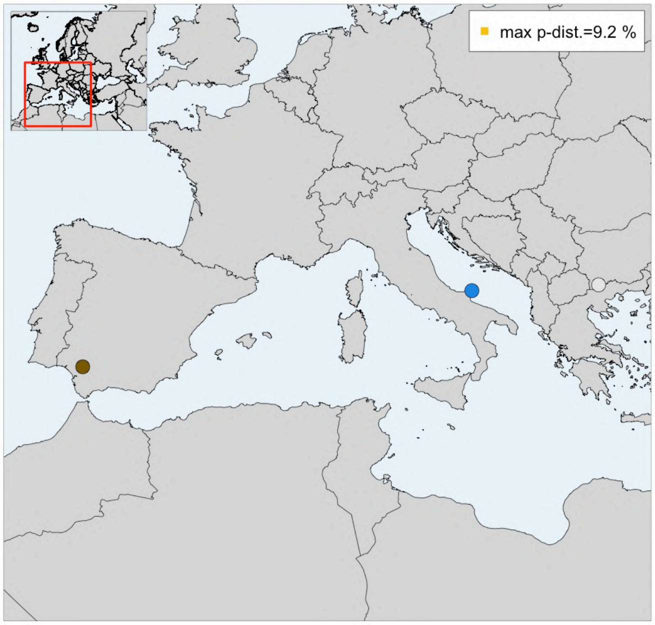

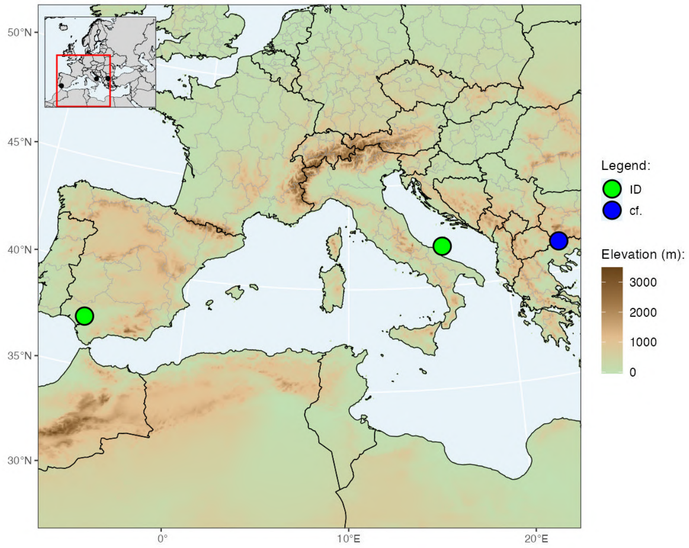

Figure 607: Genetic diversity map of *Strumigenys argiola* (Emery, 1869). Nearby localities of sequenced specimens are merged in pies (left). Colours match the bidimensional colour space of the PCoA projection (Fig. 607 left) of p-dist between sequences (dots). Specimen identification (ID or cf.) and source (newly sequenced or retrieved) are represented by colours, while specimen attribute (terra typica, type locality, type specimen or faunistic novelty) is represented by the shape (right). Sequences: ID = 2, cf. = 1; maximum p-distance: strict = NA, less strict = 9.2 %.

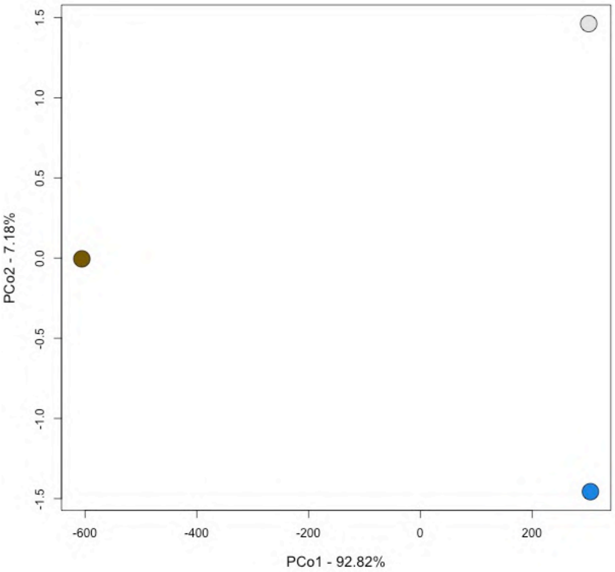

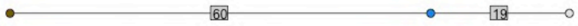

Figure 608: PCoA based on pairwise p-distances between *Strumigenys argiola* sequences (left). Colours match a bidimensional colour space. Haplotype network of *Strumigenys argiola* (right). Sequences > 599 bp: ID = 2, cf. = 1.

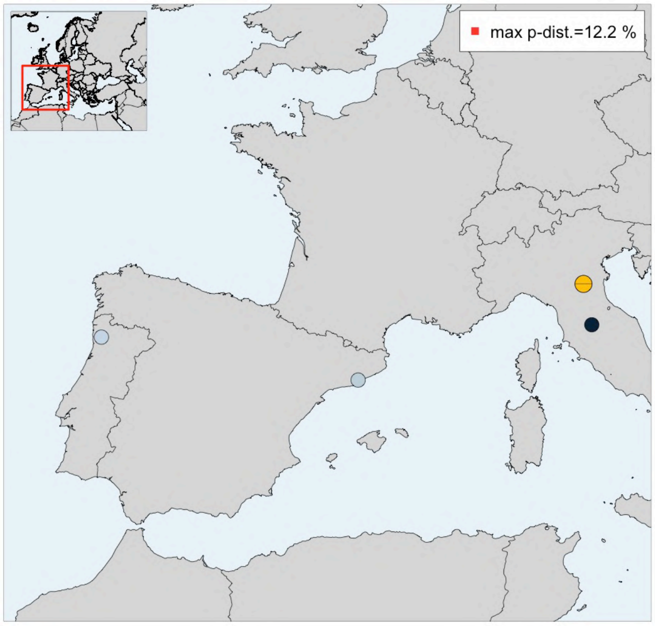

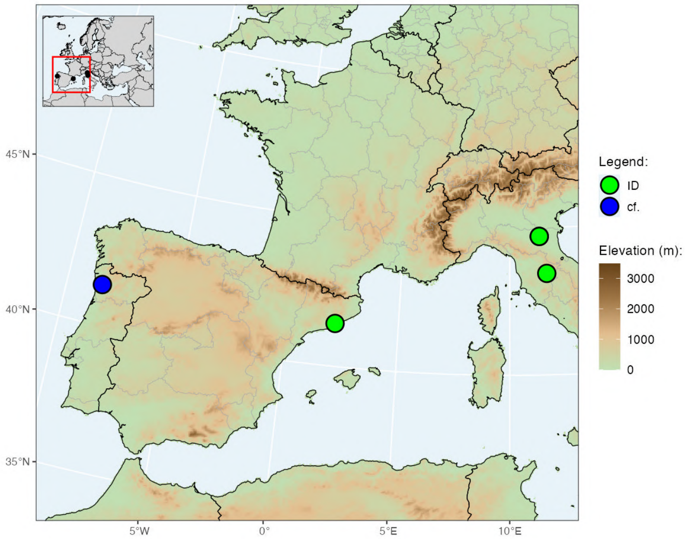

Figure 609: Genetic diversity map of *Strumigenys baudueri* (Emery, 1875). Nearby localities of sequenced specimens are merged in pies (left). Colours match the bidimensional colour space of the PCoA projection (Fig. 609 left) of p-dist between sequences (dots). Specimen identification (ID or cf.) and source (newly sequenced or retrieved) are represented by colours, while specimen attribute (terra typica, type locality, type specimen or faunistic novelty) is represented by the shape (right). Sequences: ID = 4, cf. = 1; maximum p-distance: strict = 11.7 %, less strict = 12.2 %.

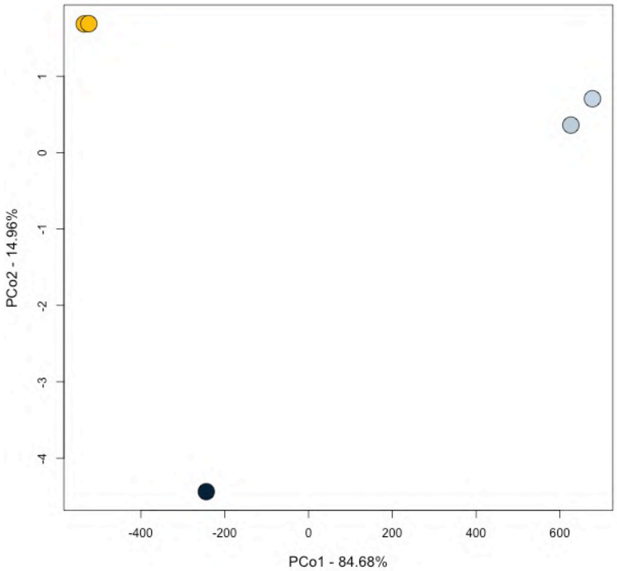

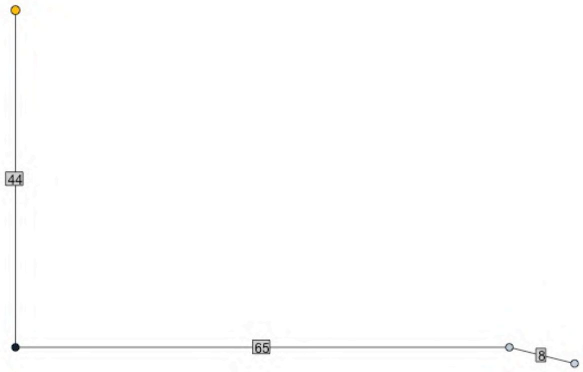

Figure 610: PCoA based on pairwise p-distances between *Strumigenys baudueri* sequences (left). Colours match a bidimensional colour space. Haplotype network of *Strumigenys baudueri* (right). Sequences > 599 bp: ID = 4, cf. = 1.

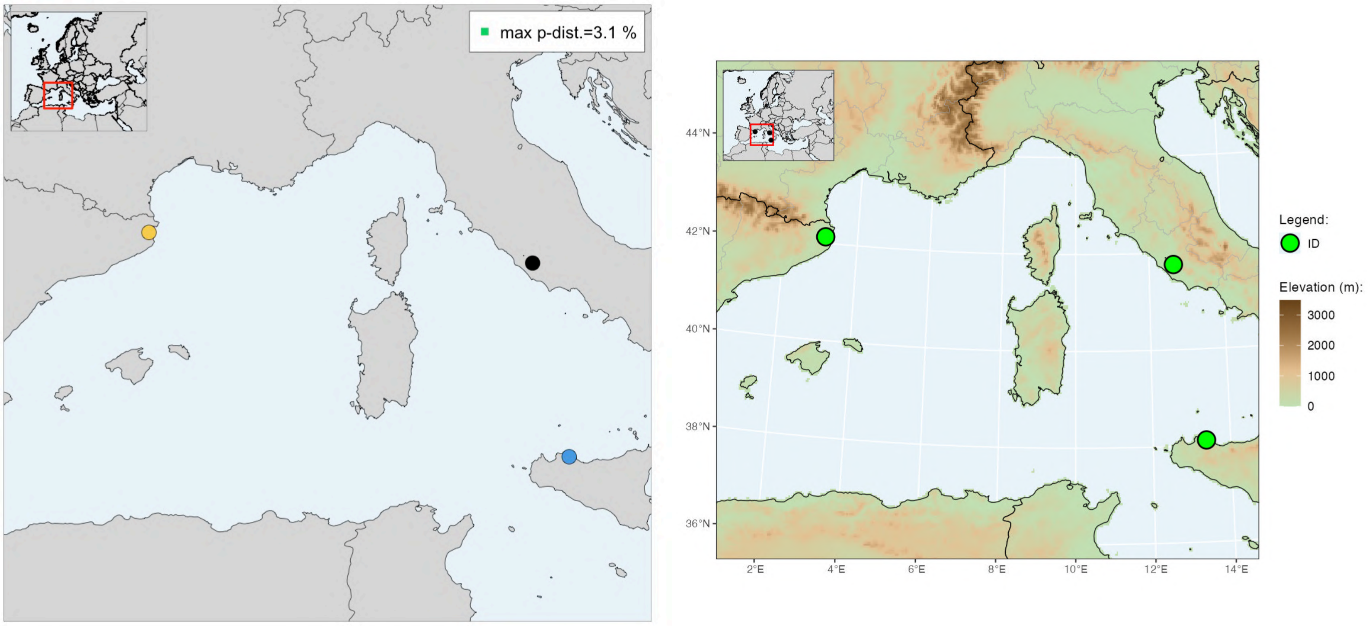

Figure 611: Genetic diversity map of *Strumigenys membranifera* Emery, 1869. Nearby localities of sequenced specimens are merged in pies (left). Colours match the bidimensional colour space of the PCoA projection (Fig. 611 left) of p-dist between sequences (dots). Specimen identification (ID or cf.) and source (newly sequenced or retrieved) are represented by colours, while specimen attribute (terra typica, type locality, type specimen or faunistic novelty) is represented by the shape (right). Sequences: ID = 3, cf. = 0; maximum p-distance: strict = 3.1 %, less strict = 3.1 %.

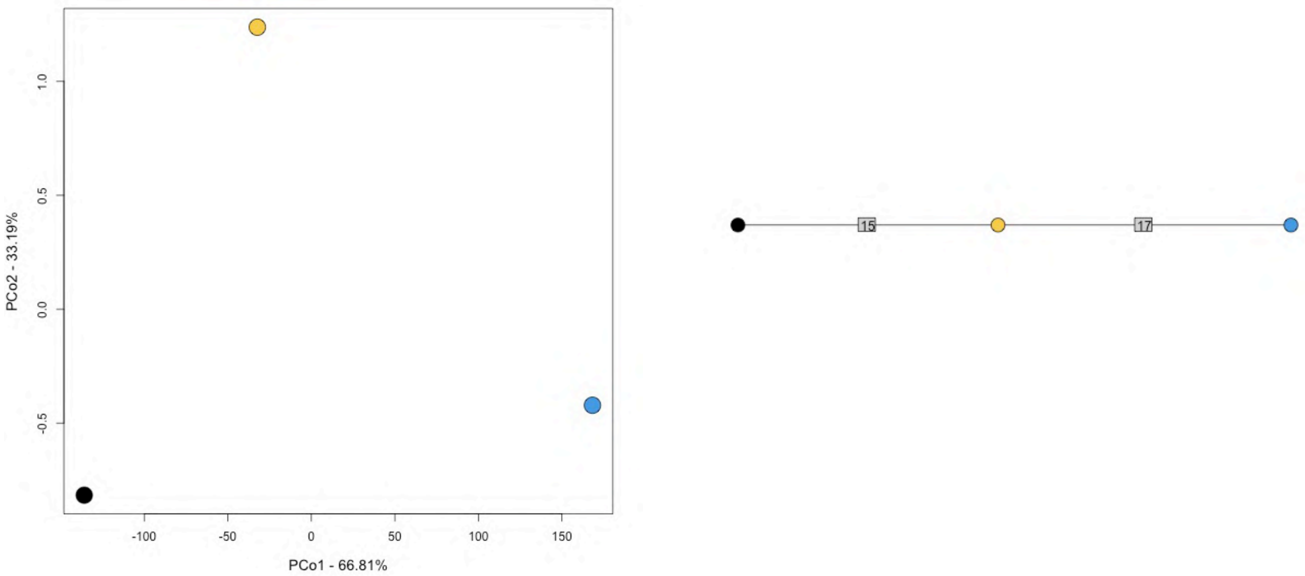

Figure 612: PCoA based on pairwise p-distances between *Strumigenys membranifera* sequences (left). Colours match a bidimensional colour space. Haplotype network of *Strumigenys membranifera* (right). Sequences > 599 bp: ID = 3, cf. = 0.

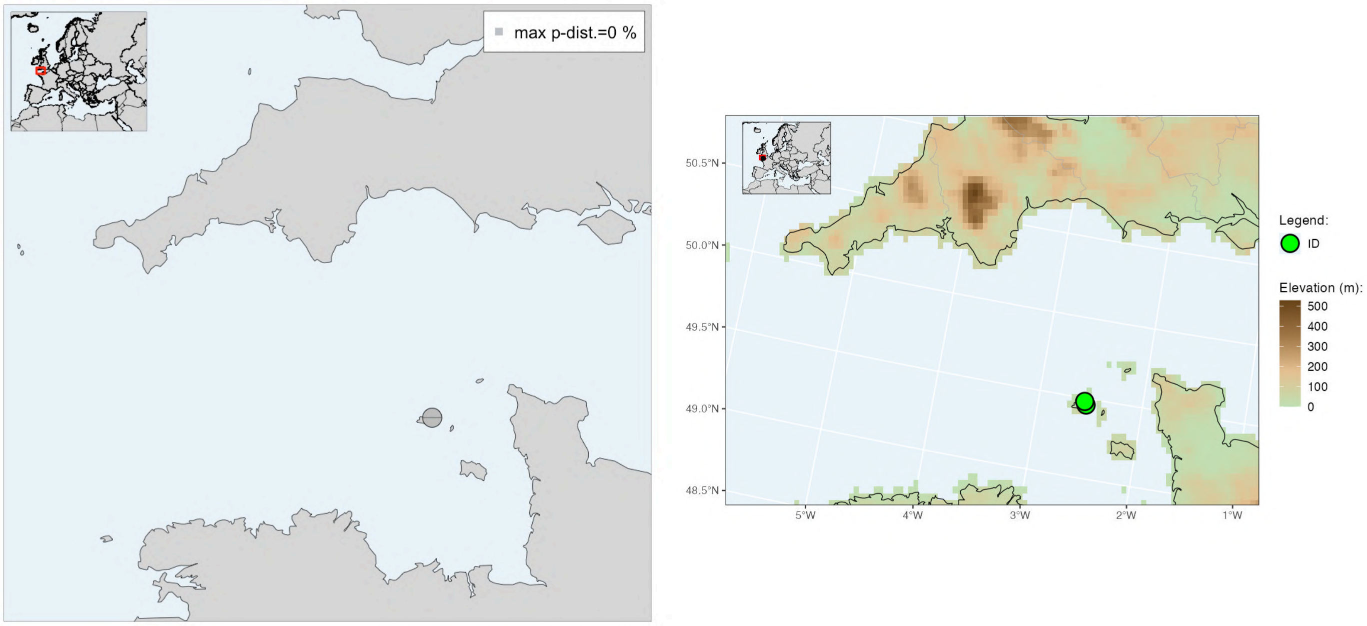

Figure 613: Genetic diversity map of *Strumigenys perplexa* (Smith 1876). PCoA projection was not done and therefore sequenced specimens in the genetic diversity map are coloured in gray (left). Specimen identification (ID or cf.) and source (newly sequenced or retrieved) are represented by colours, while specimen attribute (terra typica, type locality, type specimen or faunistic novelty) is represented by the shape (right). Sequences: ID = 6, cf. = 0; maximum p-distance: strict = NA, less strict = 0 %.

Haplotype network analysis of *Strumigenys perplexa* was not possible.

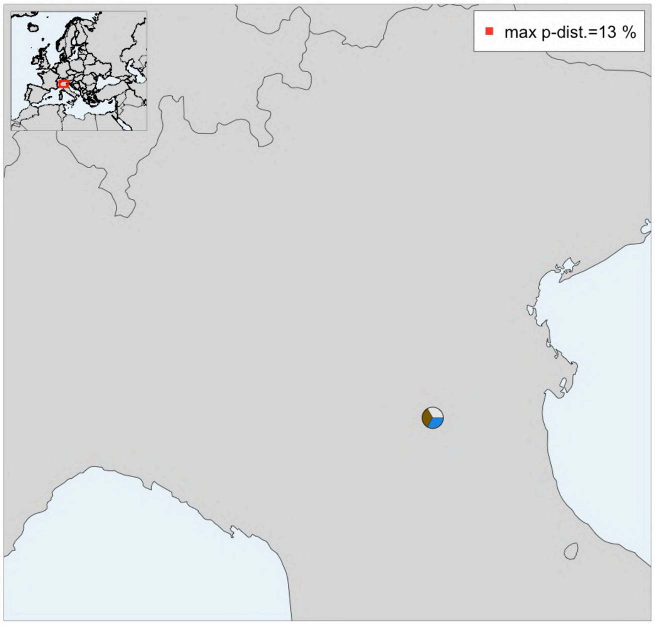

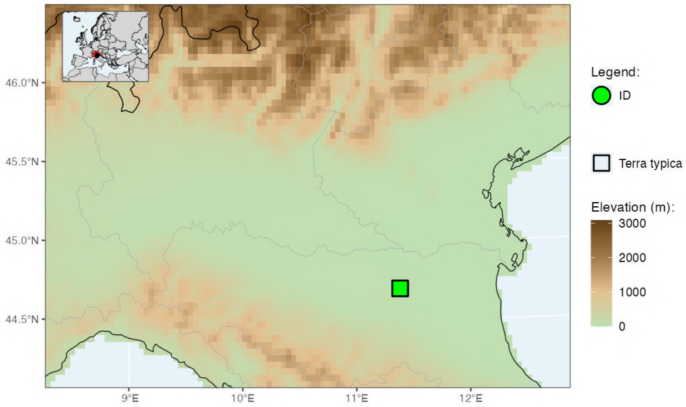

Figure 614: Genetic diversity map of *Strumigenys tenuipilis* Emery, 1915. Nearby localities of sequenced specimens are merged in pies (left). Colours match the bidimensional colour space of the PCoA projection (Fig. 614 left) of p-dist between sequences (dots). Specimen identification (ID or cf.) and source (newly sequenced or retrieved) are represented by colours, while specimen attribute (terra typica, type locality, type specimen or faunistic novelty) is represented by the shape (right). Sequences: ID = 3, cf. = 0; maximum p-distance: strict = 13 %, less strict = 13 %.

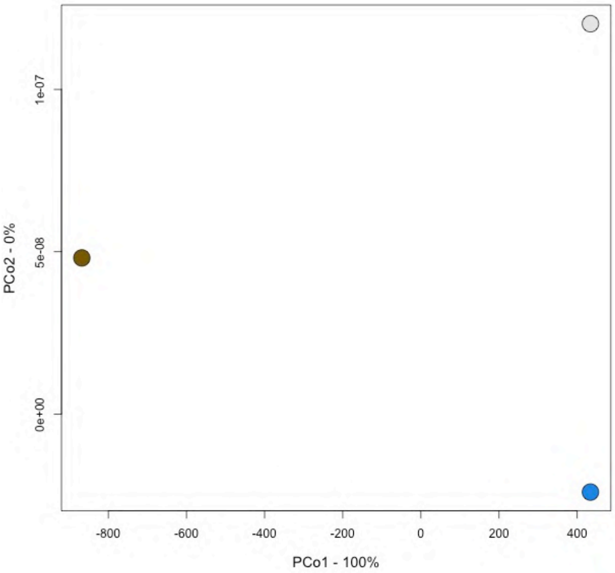

Figure 615: PCoA based on pairwise p-distances between *Strumigenys tenuipilis* sequences (left). Colours match a bidimensional colour space. Haplotype network analysis of *Strumigenys tenuipilis* was not possible.

Tapinoma

Tapinoma darioi

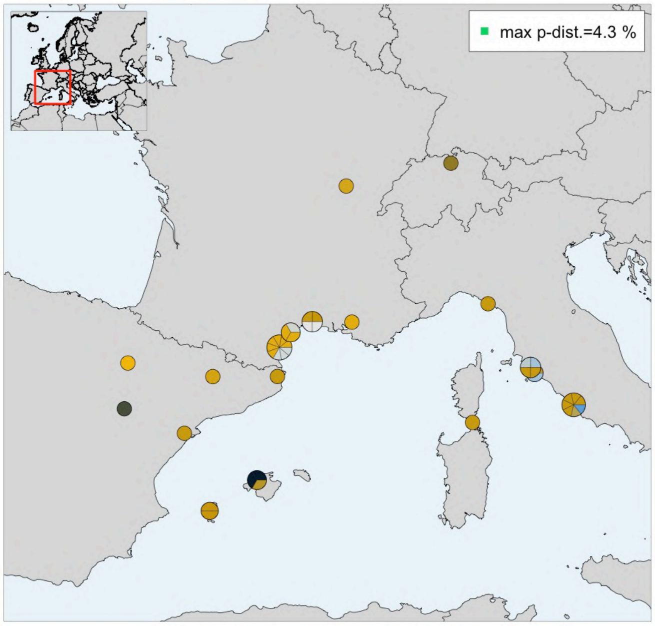

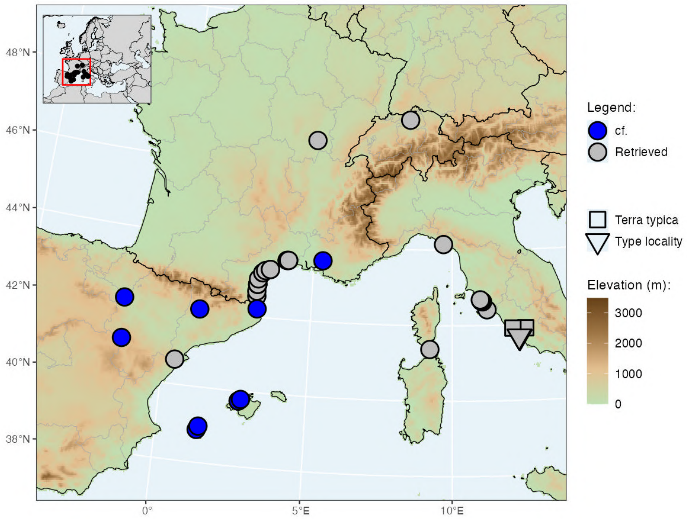

Figure 616: Genetic diversity map of *Tapinoma darioi* Seifert, D'Eustacchio, Kaufmann, Centorame & Modica, 2017. Nearby localities of sequenced specimens are merged in pies (left). Colours match the bidimensional colour space of the PCoA projection (Fig. 616 left) of p-dist between sequences (dots). Specimen identification (ID or cf.) and source (newly sequenced or retrieved) are represented by colours, while specimen attribute (terra typica, type locality, type specimen or faunistic novelty) is represented by the shape (right). Sequences: ID = 34, cf. = 10; maximum p-distance: strict = 4.1 %, less strict = 4.3 %.

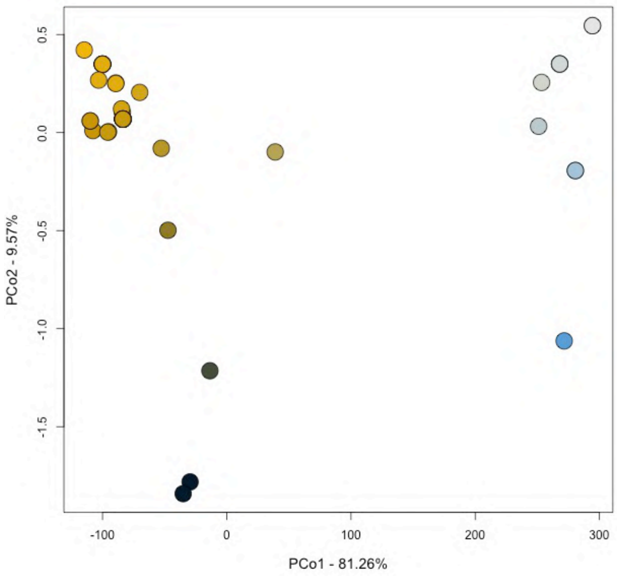

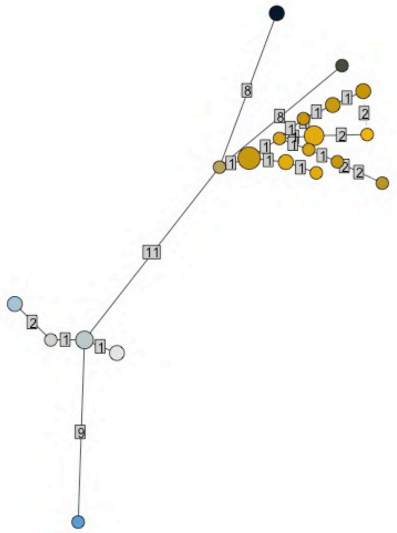

Figure 617: PCoA based on pairwise p-distances between *Tapinoma darioi* sequences (left). Colours match a bidimensional colour space. Haplotype network of *Tapinoma darioi* (right). Sequences > 599 bp: ID = 33, cf. = 10.

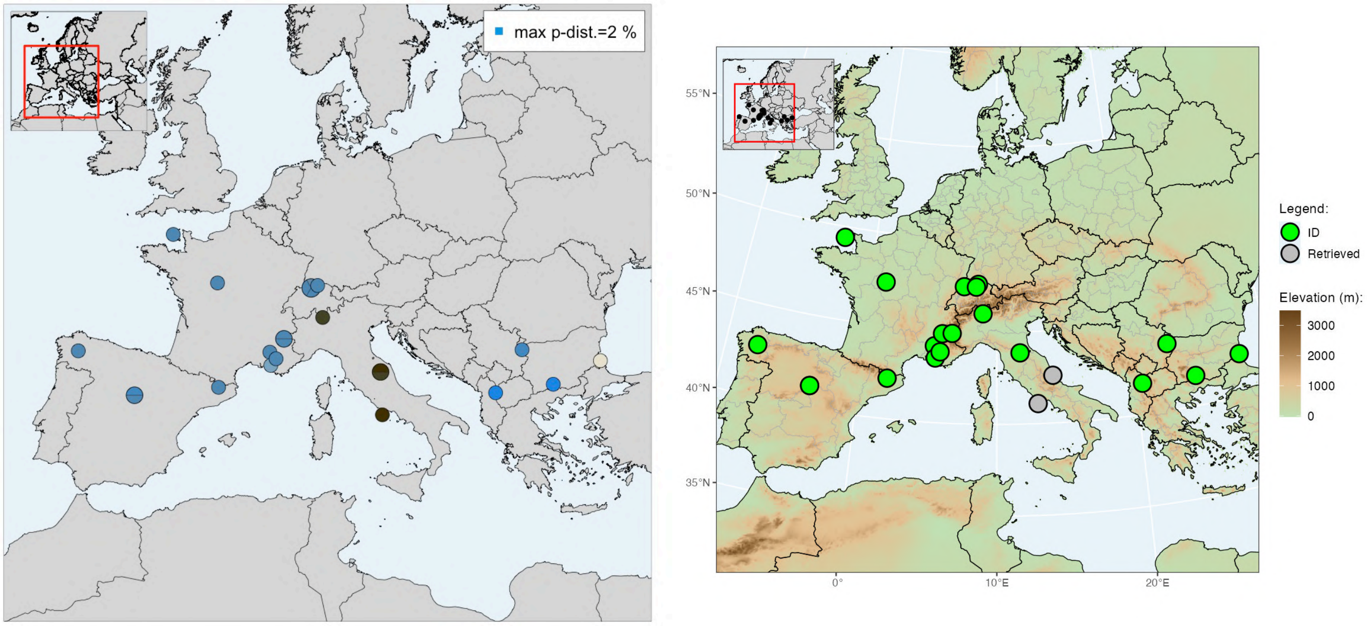

Figure 618: Genetic diversity map of *Tapinoma erraticum* (Latreille, 1798). Nearby localities of sequenced specimens are merged in pies (left). Colours match the bidimensional colour space of the PCoA projection (Fig. 618 left) of p-dist between sequences (dots). Specimen identification (ID or cf.) and source (newly sequenced or retrieved) are represented by colours, while specimen attribute (terra typica, type locality, type specimen or faunistic novelty) is represented by the shape (right). Sequences: ID = 23, cf. = 0; maximum p-distance: strict = 2 %, less strict = 2 %.

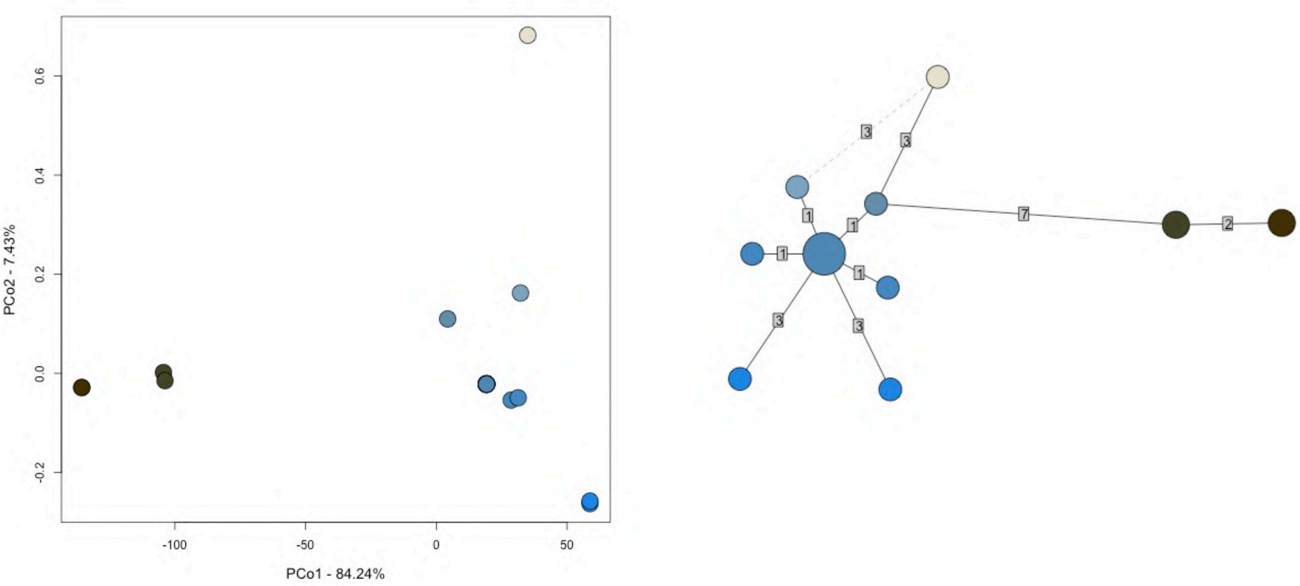

Figure 619: PCoA based on pairwise p-distances between *Tapinoma erraticum* sequences (left). Colours match a bidimensional colour space. Haplotype network of *Tapinoma erraticum* (right). Sequences > 599 bp: ID = 23, cf. = 0.

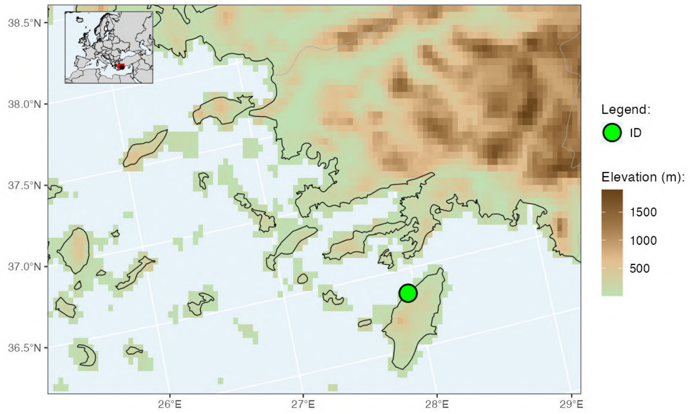

Figure 620: Map of *Tapinoma festae* Emery, 1925. Due to the presence of a single sequence, the genetic diversity map and the PCoA projection were not done. Specimen identification (ID or cf.) and source (newly sequenced or retrieved) are represented by colours, while specimen attribute (terra typica, type locality, type specimen or faunistic novelty) is represented by the shape. Sequences: ID = 1, cf. = 0; maximum p-distance: strict = NA, less strict = NA.

Haplotype network analysis of *Tapinoma festae* was not possible.

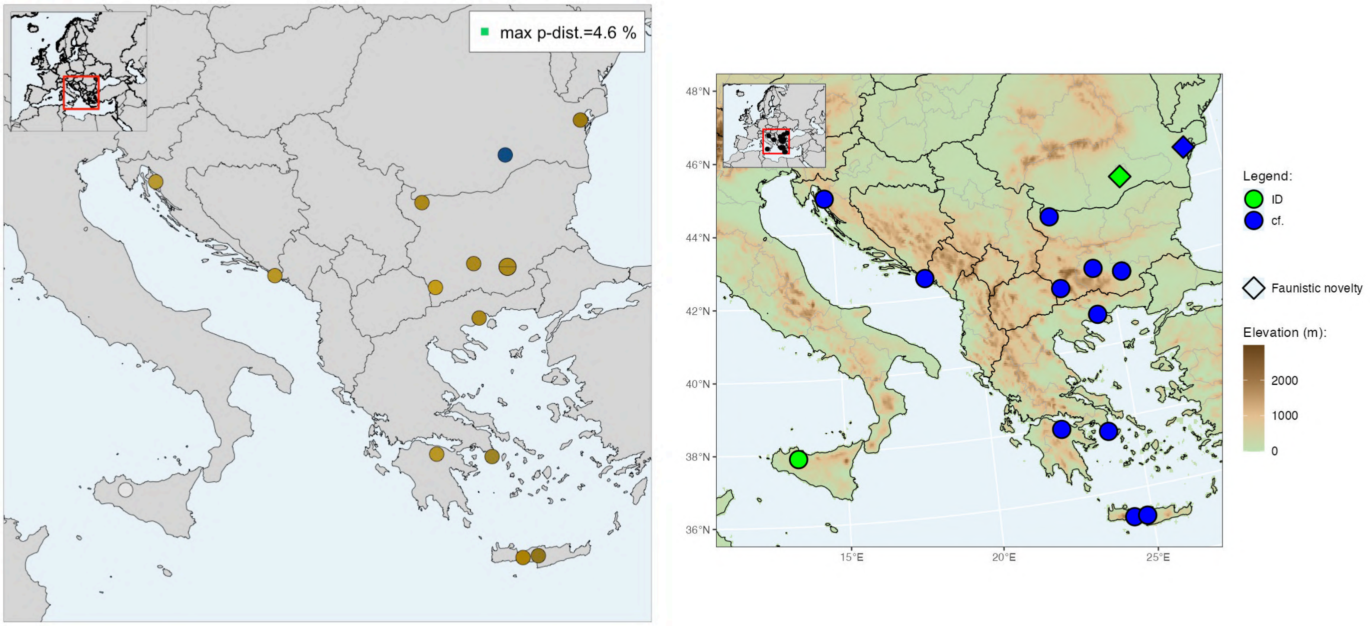

Figure 621: Genetic diversity map of *Tapinoma glabrella* Nylander, 1849. Nearby localities of sequenced specimens are merged in pies (left). Colours match the bidimensional colour space of the PCoA projection (Fig. 621 left) of p-dist between sequences (dots). Specimen identification (ID or cf.) and source (newly sequenced or retrieved) are represented by colours, while specimen attribute (terra typica, type locality, type specimen or faunistic novelty) is represented by the shape (right). Sequences: ID = 2, cf. = 13; maximum p-distance: strict = NA, less strict = 4.6 %.

The species is reported for the first time in Romania.

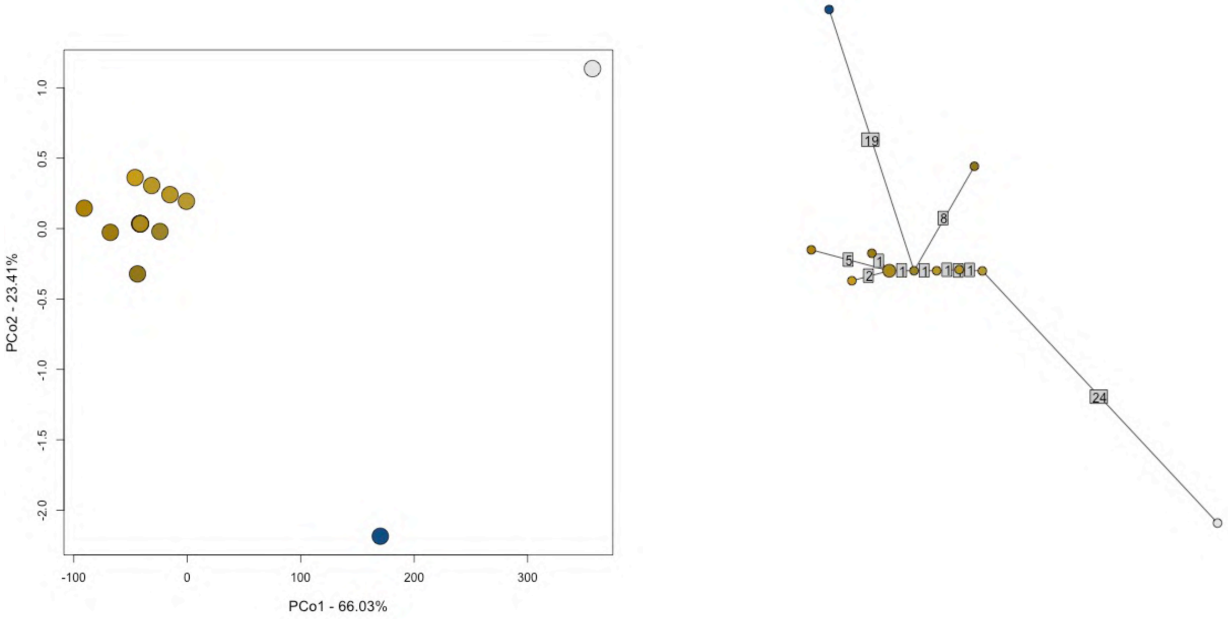

Figure 622: PCoA based on pairwise p-distances between *Tapinoma glabrella* sequences (left). Colours match a bidimensional colour space. Haplotype network of *Tapinoma glabrella* (right). Sequences > 599 bp: ID = 2, cf. = 13.

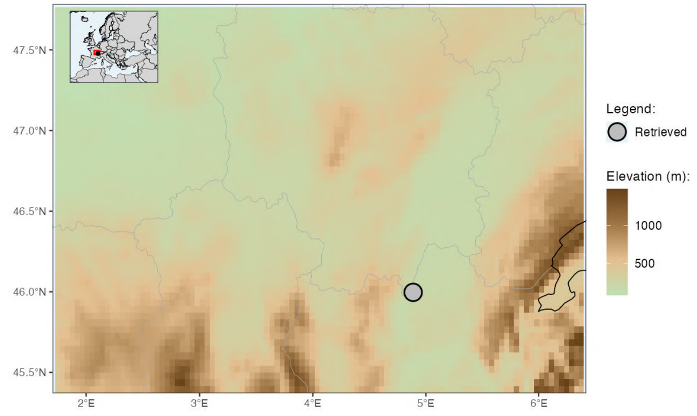

Figure 623: Map of *Tapinoma hispanicum* Seifert, Kaufmann & Fraysse, 2024. Due to the presence of a single sequence, the genetic diversity map and the PCoA projection were not done. Specimen identification (ID or cf.) and source (newly sequenced or retrieved) are represented by colours, while specimen attribute (terra typica, type locality, type specimen or faunistic novelty) is represented by the shape. Sequences: ID = 1, cf. = 0; maximum p-distance: strict = NA, less strict = NA.

Haplotype network analysis of *Tapinoma hispanicum* was not possible.

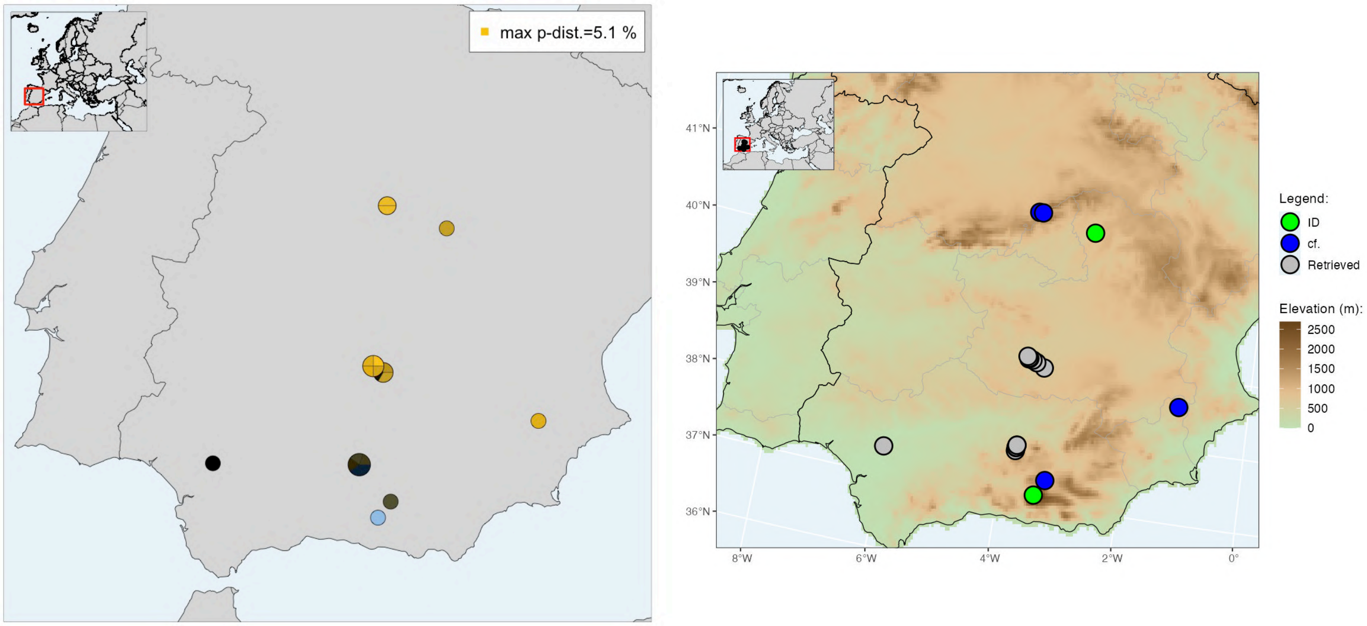

Figure 624: Genetic diversity map of *Tapinoma ibericum* Santschi, 1925. Nearby localities of sequenced specimens are merged in pies (left). Colours match the bidimensional colour space of the PCoA projection (Fig. 624 left) of p-dist between sequences (dots). Specimen identification (ID or cf.) and source (newly sequenced or retrieved) are represented by colours, while specimen attribute (terra typica, type locality, type specimen or faunistic novelty) is represented by the shape (right). Sequences: ID = 15, cf. = 4; maximum p-distance: strict = 5.1 %, less strict = 5.1 %.

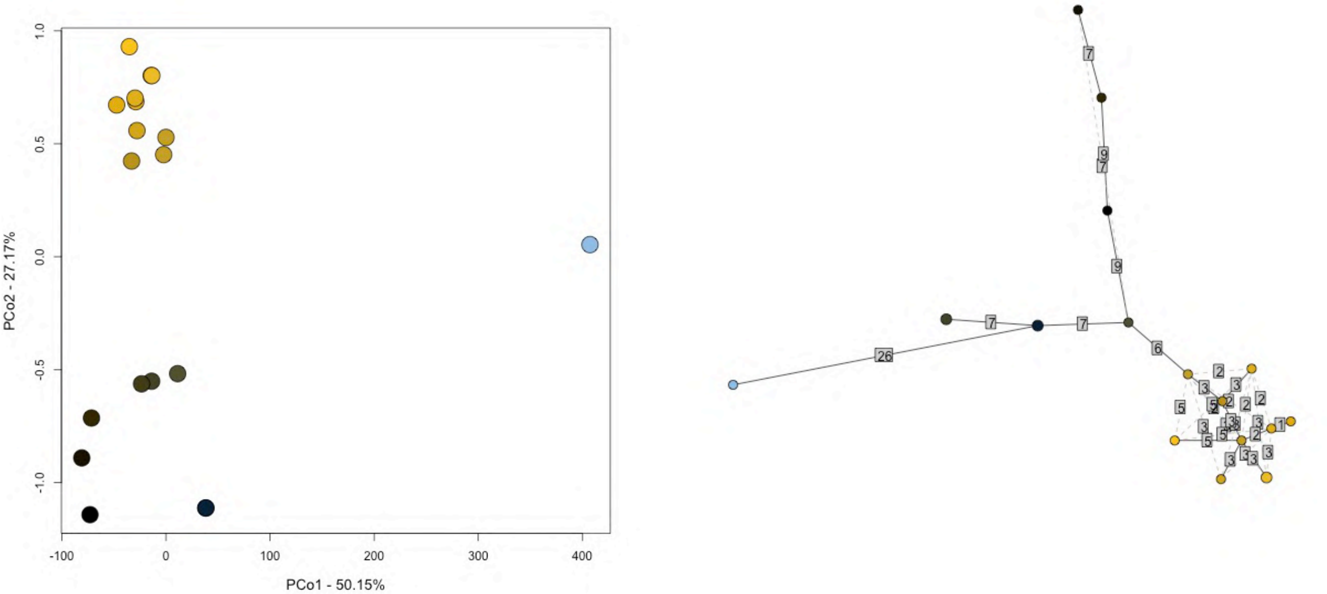

Figure 625: PCoA based on pairwise p-distances between *Tapinoma ibericum* sequences (left). Colours match a bidimensional colour space. Haplotype network of *Tapinoma ibericum* (right). Sequences > 599 bp: ID = 15, cf. = 4.

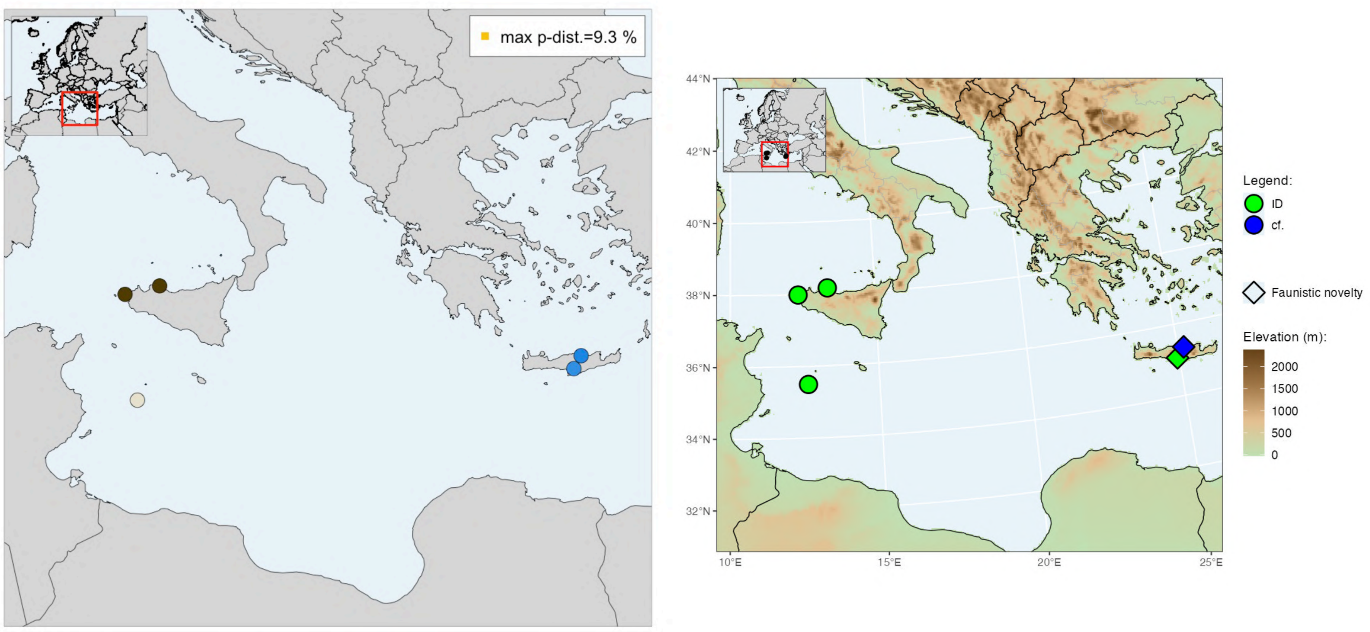

Figure 626: Genetic diversity map of *Tapinoma insularis* Seifert, Kaufmann & Fraysse, 2024. Nearby localities of sequenced specimens are merged in pies (left). Colours match the bidimensional colour space of the PCoA projection (Fig. 626 left) of p-dist between sequences (dots). Specimen identification (ID or cf.) and source (newly sequenced or retrieved) are represented by colours, while specimen attribute (terra typica, type locality, type specimen or faunistic novelty) is represented by the shape (right). Sequences: ID = 4, cf. = 1; maximum p-distance: strict = 9.2 %, less strict = 9.3 %.

The species is reported for the first time in Greece.

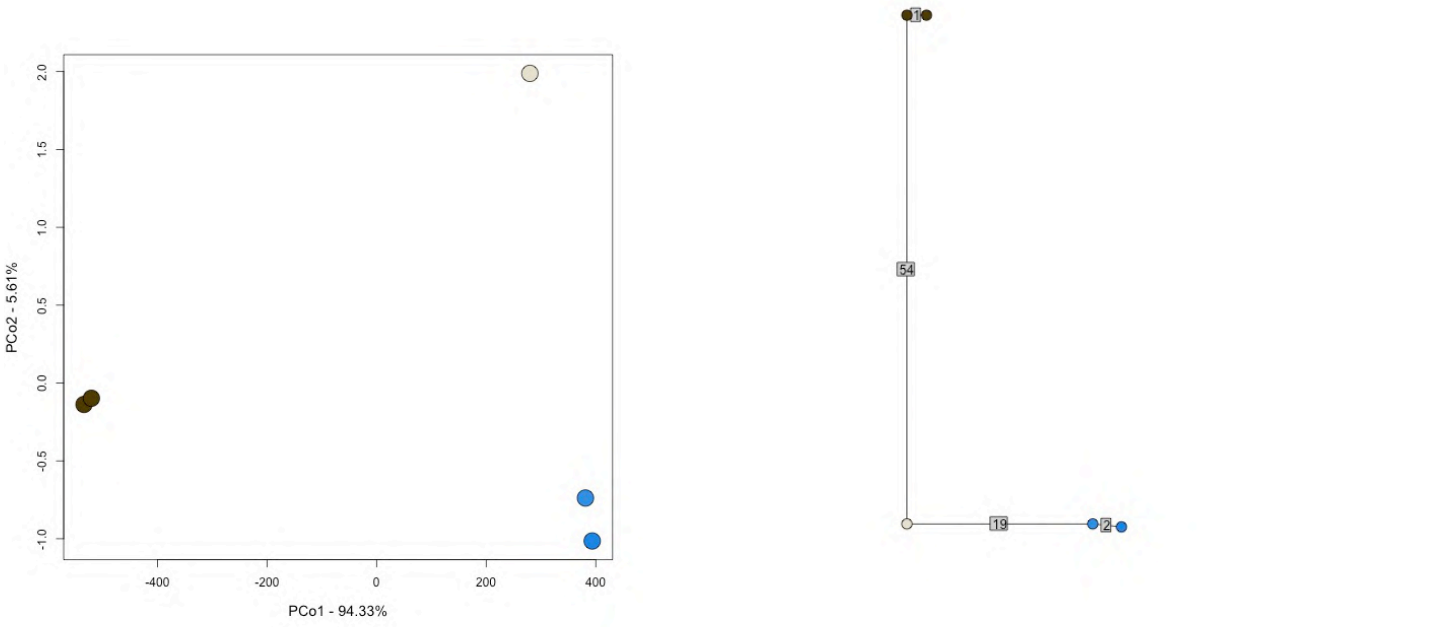

Figure 627: PCoA based on pairwise p-distances between *Tapinoma insularis* sequences (left). Colours match a bidimensional colour space. Haplotype network of *Tapinoma insularis* (right). Sequences > 599 bp: ID = 4, cf. = 1.

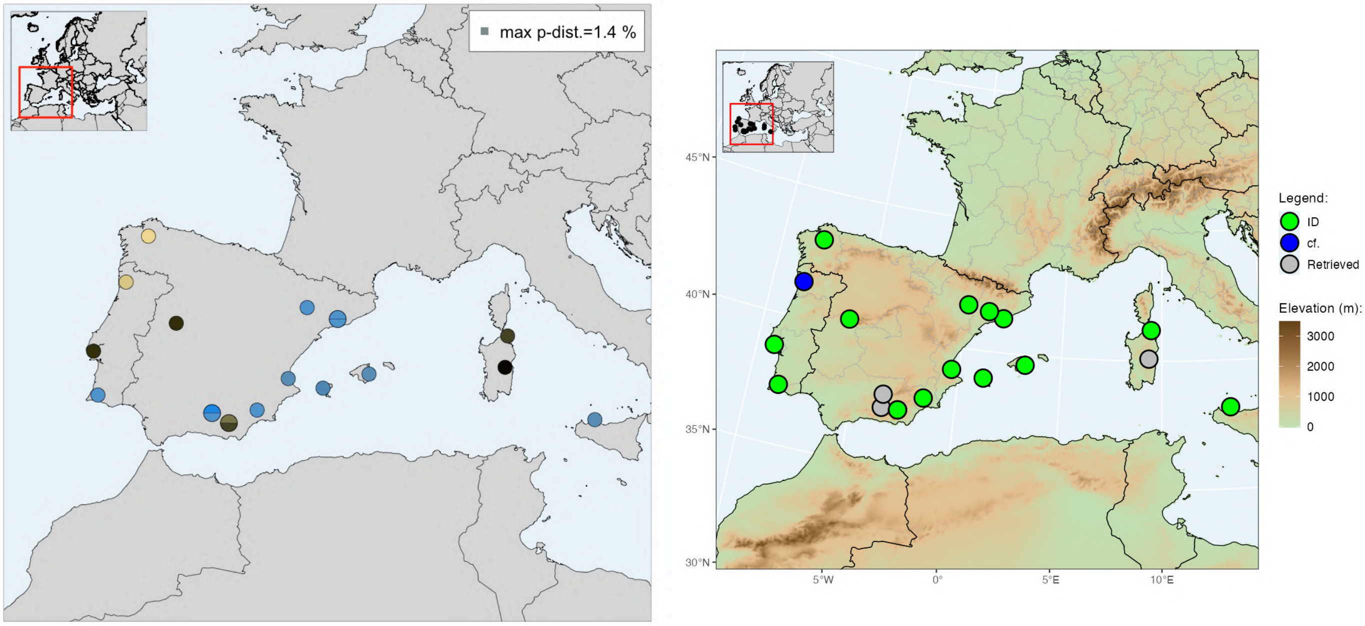

Figure 628: Genetic diversity map of *Tapinoma madeirense* Forel, 1895. Nearby localities of sequenced specimens are merged in pies (left). Colours match the bidimensional colour space of the PCoA projection (Fig. 628 left) of p-dist between sequences (dots). Specimen identification (ID or cf.) and source (newly sequenced or retrieved) are represented by colours, while specimen attribute (terra typica, type locality, type specimen or faunistic novelty) is represented by the shape (right). Sequences: ID = 18, cf. = 1; maximum p-distance: strict = 1.4 %, less strict = 1.4 %.

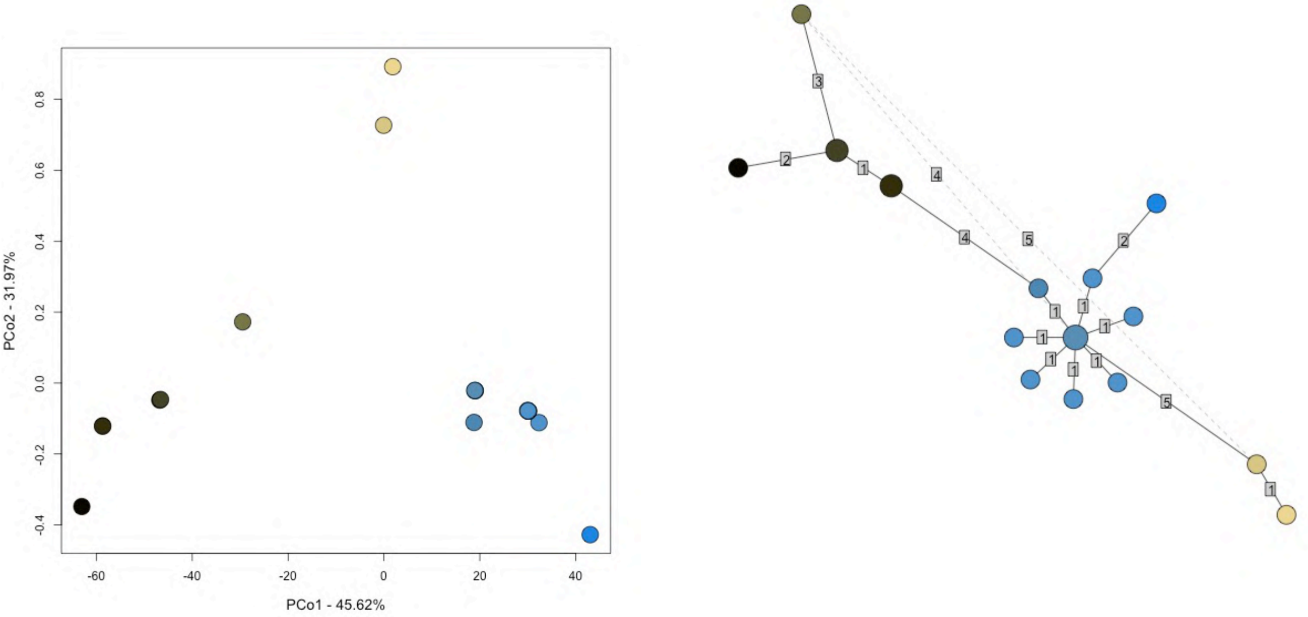

Figure 629: PCoA based on pairwise p-distances between *Tapinoma madeirense* sequences (left). Colours match a bidimensional colour space. Haplotype network of *Tapinoma madeirense* (right). Sequences > 599 bp: ID = 18, cf. = 1.

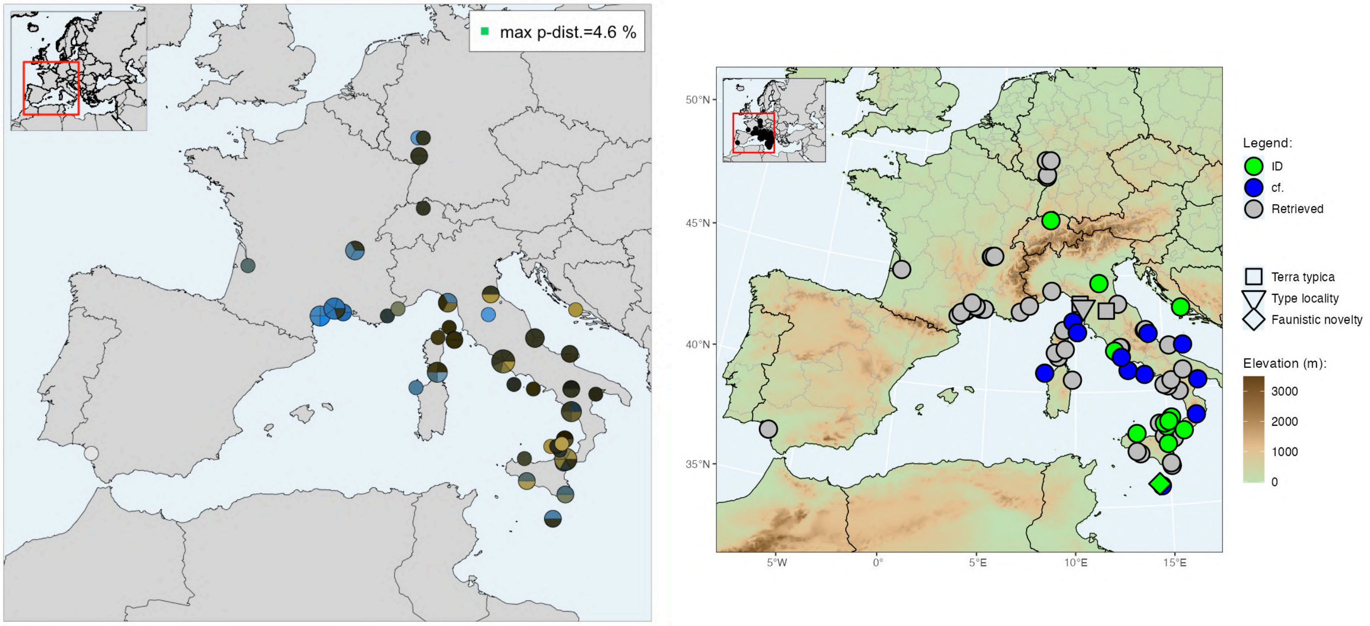

Figure 630: Genetic diversity map of *Tapinoma magnum* Mayr, 1861. Nearby localities of sequenced specimens are merged in pies (left). Colours match the bidimensional colour space of the PCoA projection (Fig. 630 left) of p-dist between sequences (dots). Specimen identification (ID or cf.) and source (newly sequenced or retrieved) are represented by colours, while specimen attribute (terra typica, type locality, type specimen or faunistic novelty) is represented by the shape (right). Sequences: ID = 70, cf. = 12; maximum p-distance: strict = 4.6 %, less strict = 4.6 %.

The species is reported for the first time in Malta.

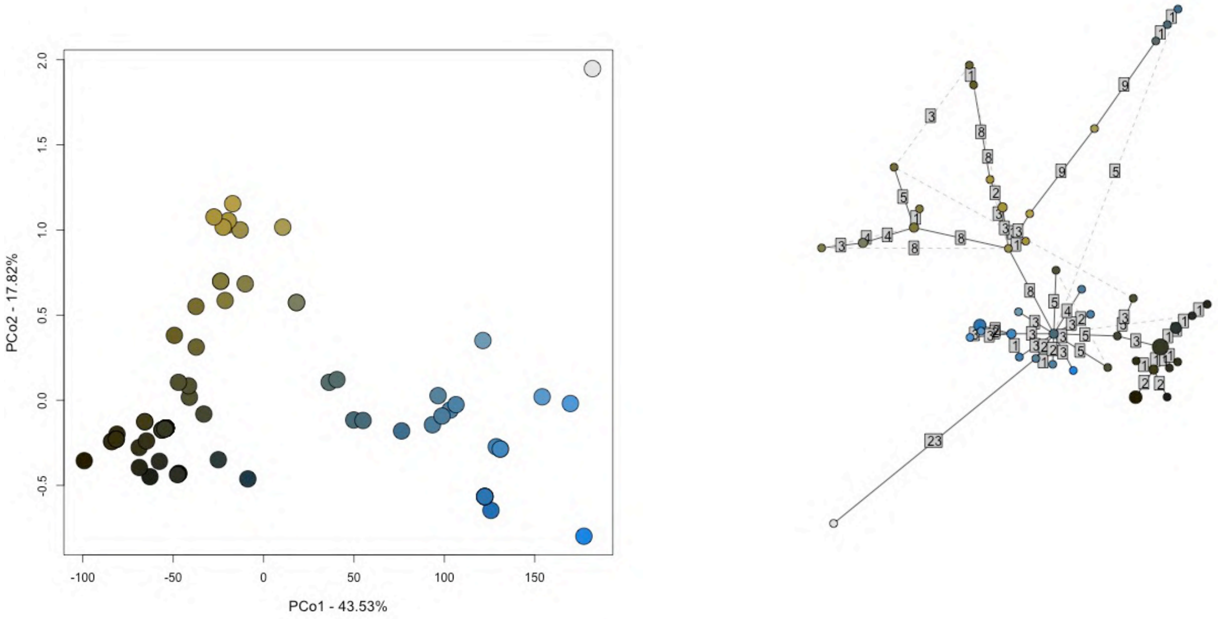

Figure 631: PCoA based on pairwise p-distances between *Tapinoma magnum* sequences (left). Colours match a bidimensional colour space. Haplotype network of *Tapinoma magnum* (right). Sequences > 599 bp: ID = 68, cf. = 12.

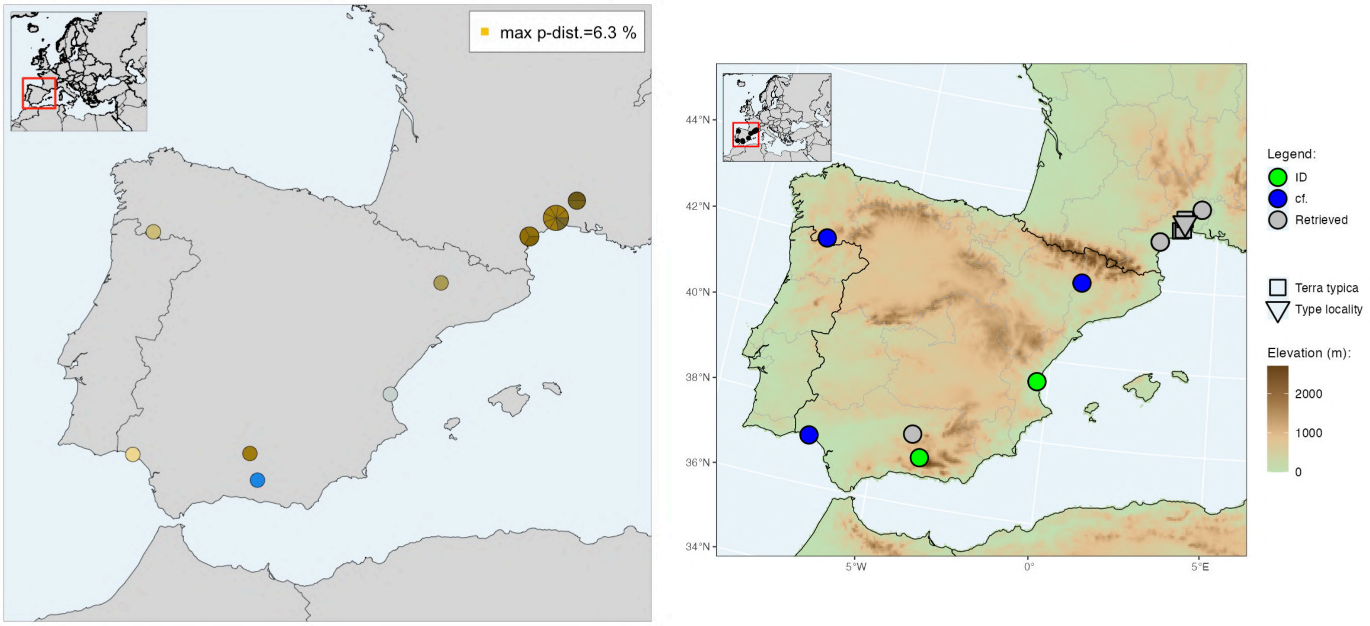

Figure 632: Genetic diversity map of *Tapinoma nigerrimum* Nylander, 1856. Nearby localities of sequenced specimens are merged in pies (left). Colours match the bidimensional colour space of the PCoA projection (Fig. 632 left) of p-dist between sequences (dots). Specimen identification (ID or cf.) and source (newly sequenced or retrieved) are represented by colours, while specimen attribute (terra typica, type locality, type specimen or faunistic novelty) is represented by the shape (right). Sequences: ID = 17, cf. = 3; maximum p-distance: strict = 1.5 %, less strict = 6.3 %.

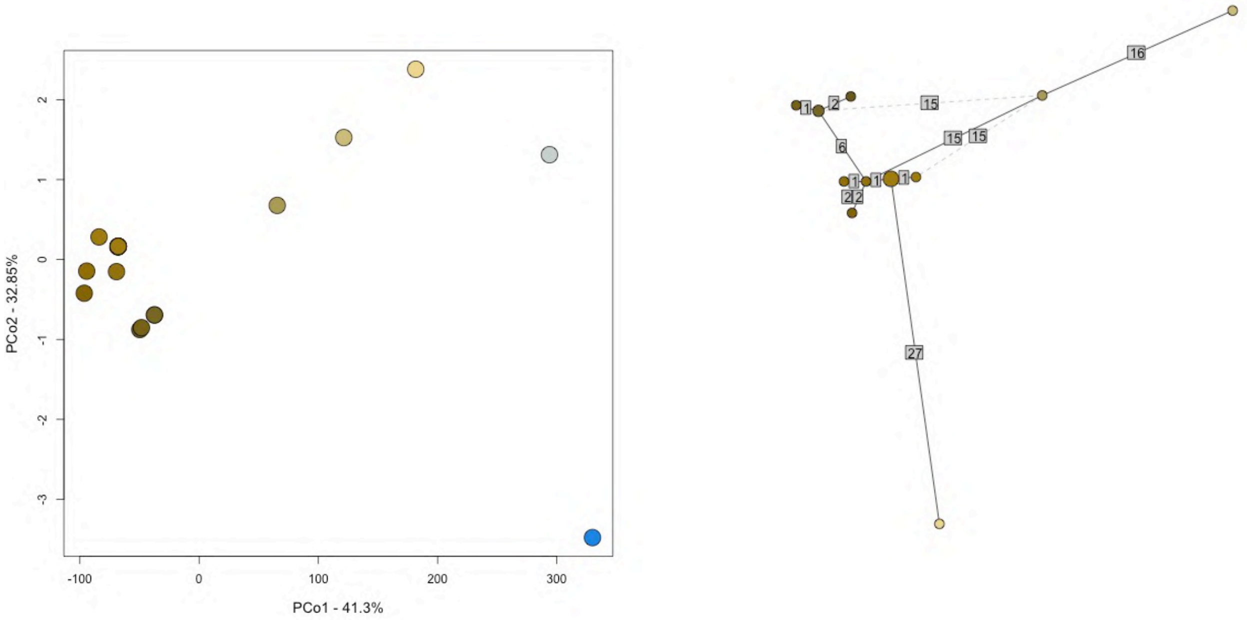

Figure 633: PCoA based on pairwise p-distances between *Tapinoma nigerrimum* sequences (left). Colours match a bidimensional colour space. Haplotype network of *Tapinoma nigerrimum* (right). Sequences > 599 bp: ID = 15, cf. = 3.

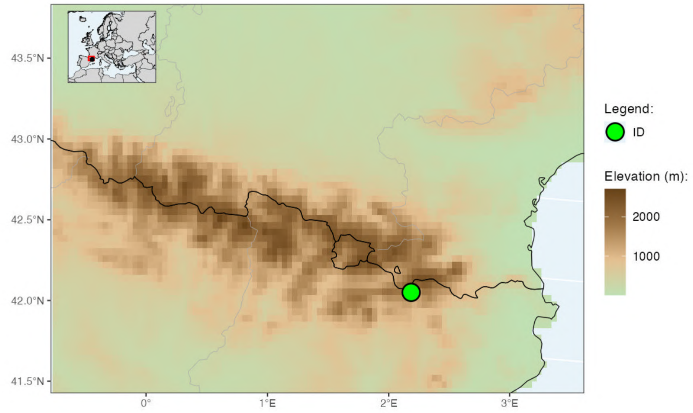

Figure 634: Map of *Tapinoma pygmaeum* (Dufour, 1857). Due to the presence of a single sequence, the genetic diversity map and the PCoA projection were not done. Specimen identification (ID or cf.) and source (newly sequenced or retrieved) are represented by colours, while specimen attribute (terra typica, type locality, type specimen or faunistic novelty) is represented by the shape. Sequences: ID = 1, cf. = 0; maximum p-distance: strict = NA, less strict = NA.

Haplotype network analysis of *Tapinoma pygmaeum* was not possible.

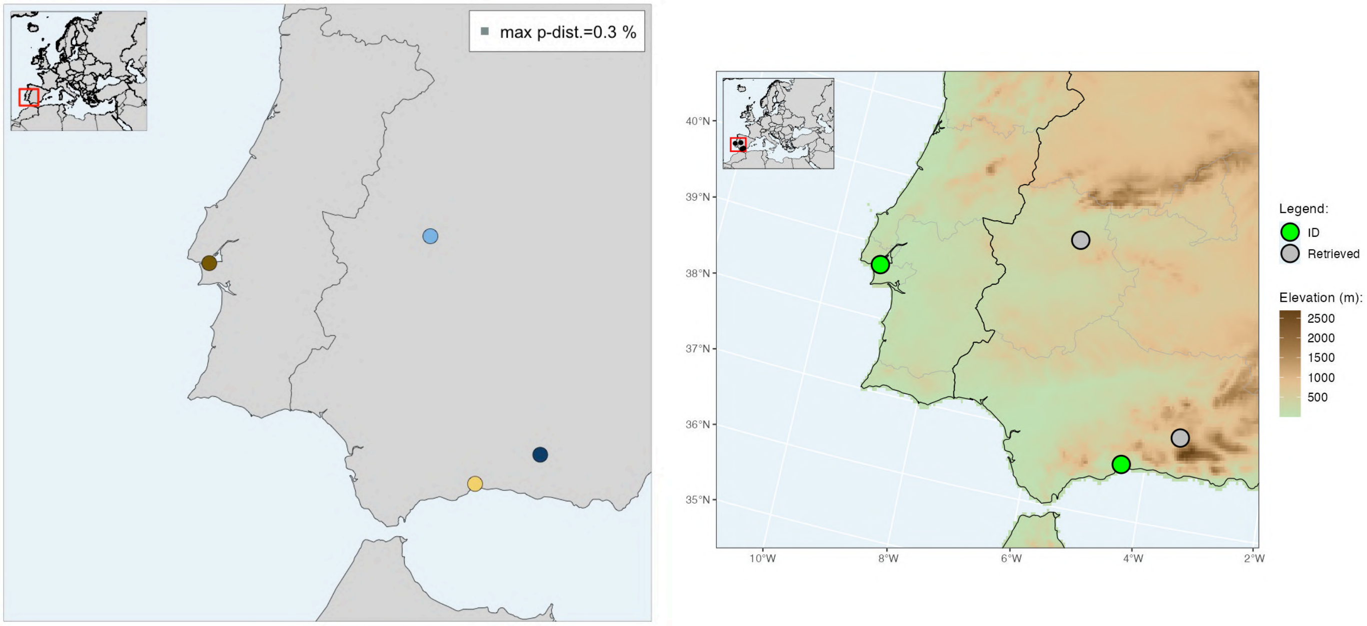

Figure 635: Genetic diversity map of *Tapinoma simrothi* Krausse, 1911. Nearby localities of sequenced specimens are merged in pies (left). Colours match the bidimensional colour space of the PCoA projection (Fig. 635 left) of p-dist between sequences (dots). Specimen identification (ID or cf.) and source (newly sequenced or retrieved) are represented by colours, while specimen attribute (terra typica, type locality, type specimen or faunistic novelty) is represented by the shape (right). Sequences: ID = 4, cf. = 0; maximum p-distance: strict = 0.3 %, less strict = 0.3 %.

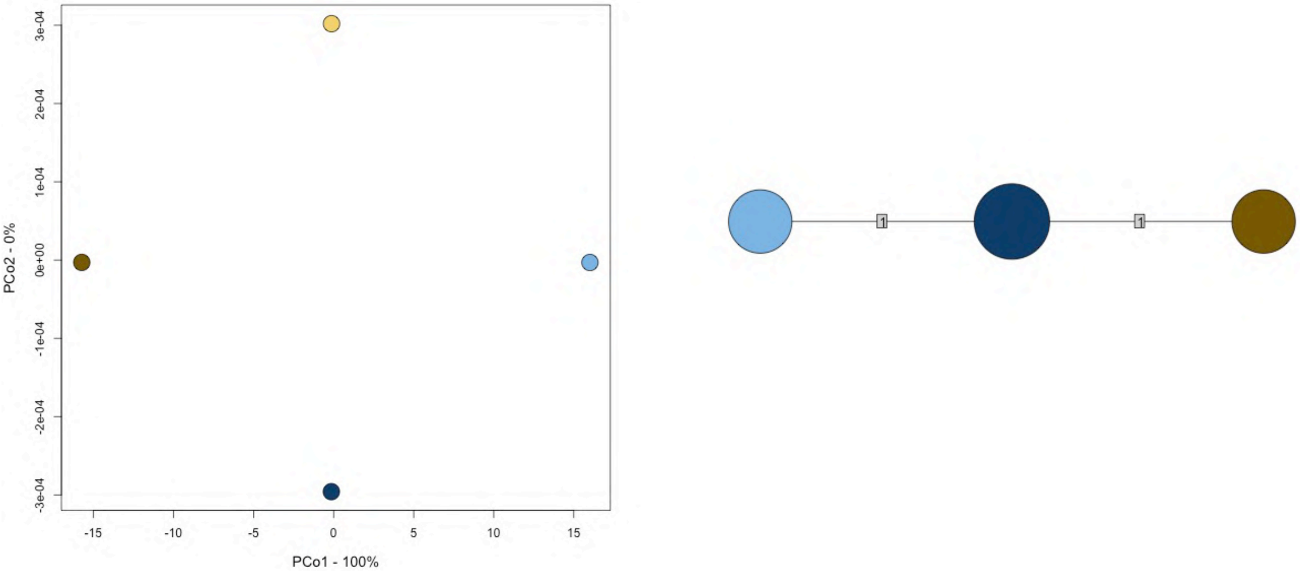

Figure 636: PCoA based on pairwise p-distances between *Tapinoma simrothi* sequences (left). Colours match a bidimensional colour space. Haplotype network of *Tapinoma simrothi* (right). Sequences > 599 bp: ID = 4, cf. = 0.

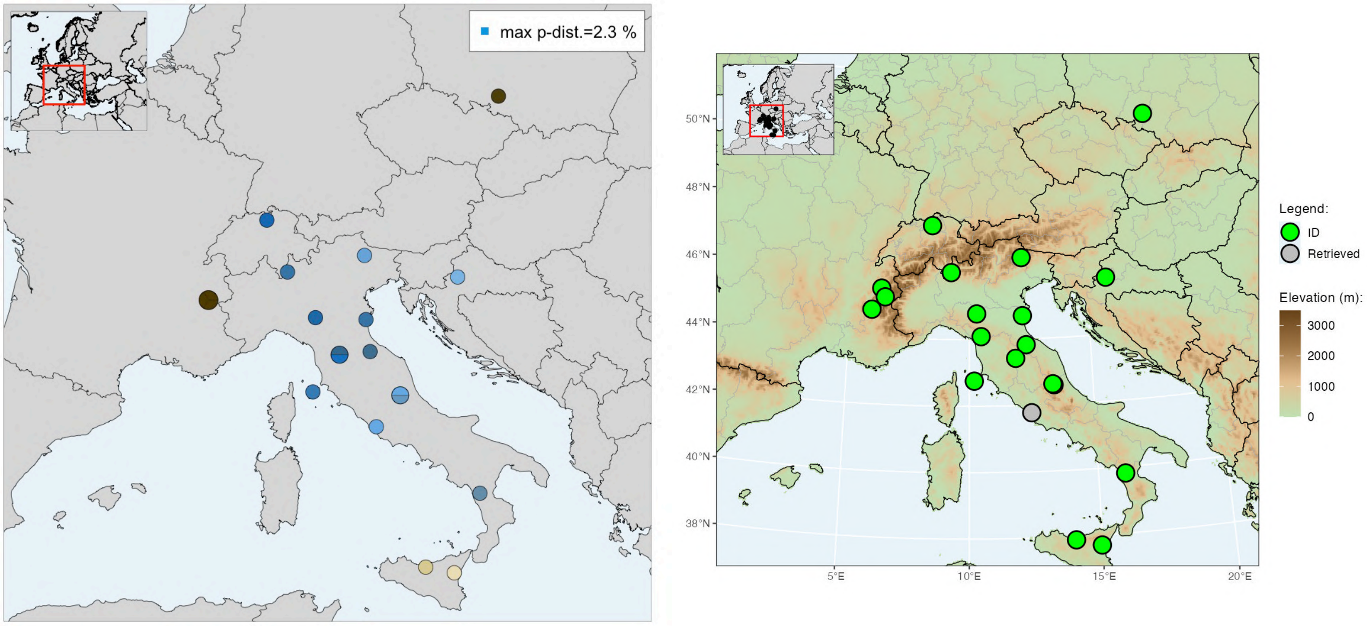

Figure 637: Genetic diversity map of *Tapinoma subboreale* Seifert, 2012. Nearby localities of sequenced specimens are merged in pies (left). Colours match the bidimensional colour space of the PCoA projection (Fig. 637 left) of p-dist between sequences (dots). Specimen identification (ID or cf.) and source (newly sequenced or retrieved) are represented by colours, while specimen attribute (terra typica, type locality, type specimen or faunistic novelty) is represented by the shape (right). Sequences: ID = 20, cf. = 0; maximum p-distance: strict = 2.3 %, less strict = 2.3 %.

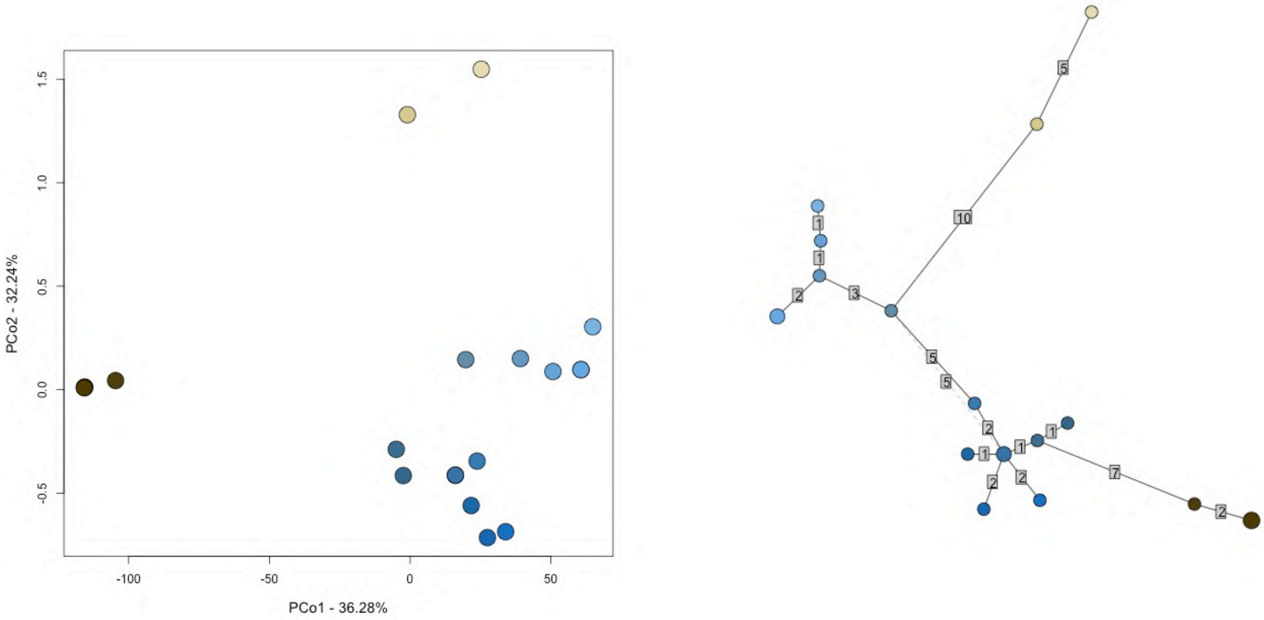

Figure 638: PCoA based on pairwise p-distances between *Tapinoma subboreale* sequences (left). Colours match a bidimensional colour space. Haplotype network of *Tapinoma subboreale* (right). Sequences > 599 bp: ID = 20, cf. = 0.

Technomyrmex

Technomyrmex albipes

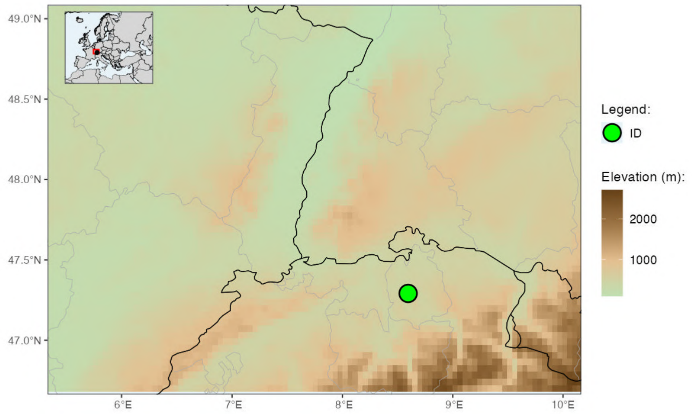

Figure 639: Map of *Technomyrmex albipes* (Smith, F., 1861). Due to the presence of a single sequence, the genetic diversity map and the PCoA projection were not done. Specimen identification (ID or cf.) and source (newly sequenced or retrieved) are represented by colours, while specimen attribute (terra typica, type locality, type specimen or faunistic novelty) is represented by the shape. Sequences: ID = 6, cf. = 0; maximum p-distance: strict = NA, less strict = NA.

Haplotype network analysis of *Technomyrmex albipes* was not possible.

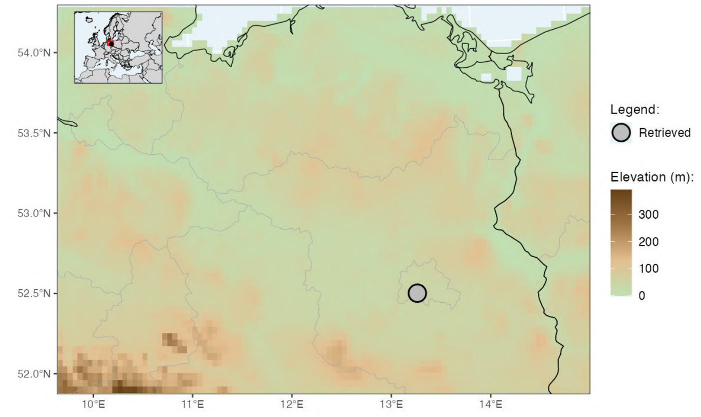

Figure 640: Map of *Technomyrmex difficilis* Forel, 1892. Due to the presence of a single sequence, the genetic diversity map and the PCoA projection were not done. Specimen identification (ID or cf.) and source (newly sequenced or retrieved) are represented by colours, while specimen attribute (terra typica, type locality, type specimen or faunistic novelty) is represented by the shape. Sequences: ID = 4, cf. = 0; maximum p-distance: strict = NA, less strict = NA.

Haplotype network analysis of *Technomyrmex difficilis* was not possible.

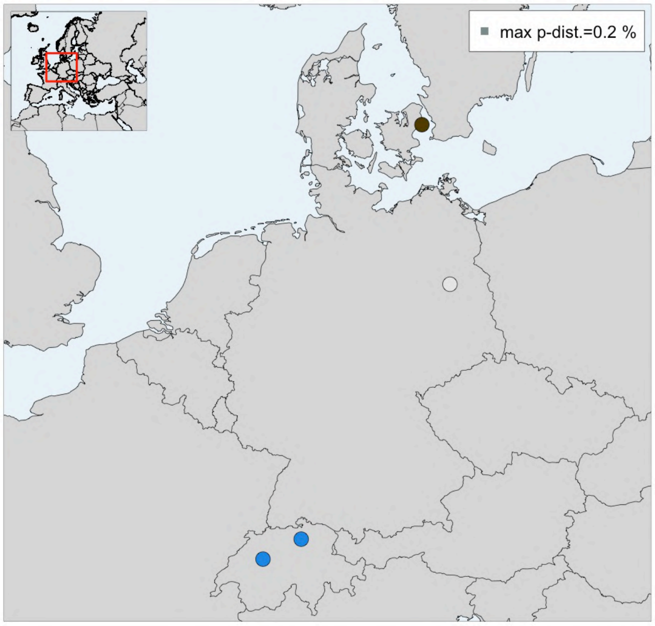

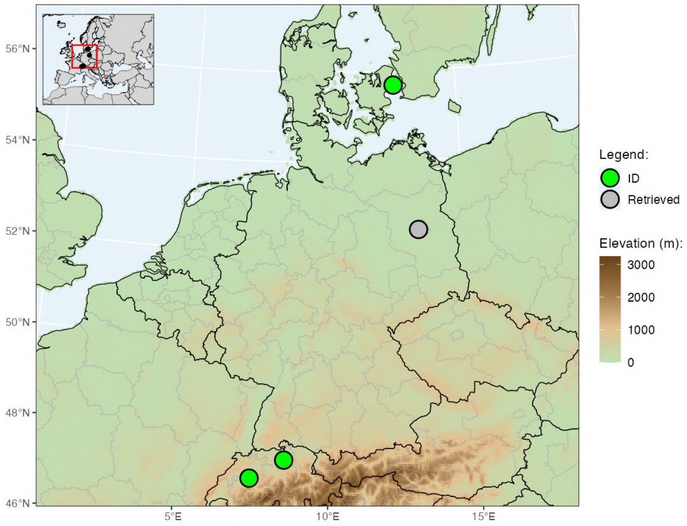

Figure 641: Genetic diversity map of *Technomyrmex vitiensis* Mann, 1921. Nearby localities of sequenced specimens are merged in pies (left). Colours match the bidimensional colour space of the PCoA projection (Fig. 641 left) of p-dist between sequences (dots). Specimen identification (ID or cf.) and source (newly sequenced or retrieved) are represented by colours, while specimen attribute (terra typica, type locality, type specimen or faunistic novelty) is represented by the shape (right). Sequences: ID = 4, cf. = 0; maximum p-distance: strict = 0.2 %, less strict = 0.2 %.

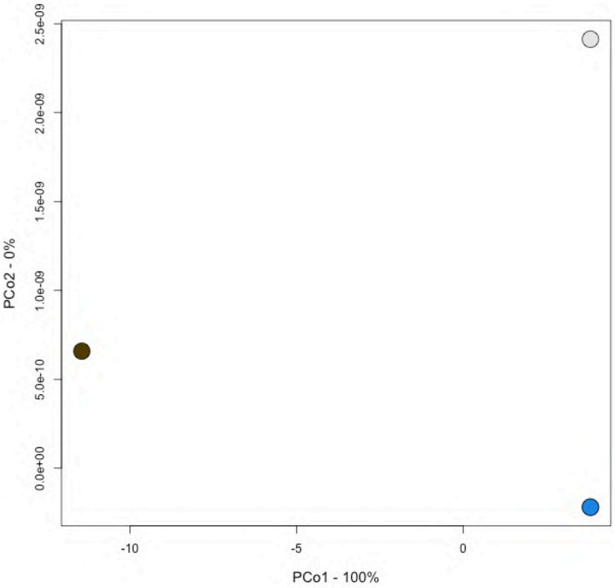

Figure 642: PCoA based on pairwise p-distances between *Technomyrmex vitiensis* sequences (left). Colours match a bidimensional colour space. Haplotype network analysis of *Technomyrmex vitiensis* was not possible.

Temnothorax

Temnothorax affinis

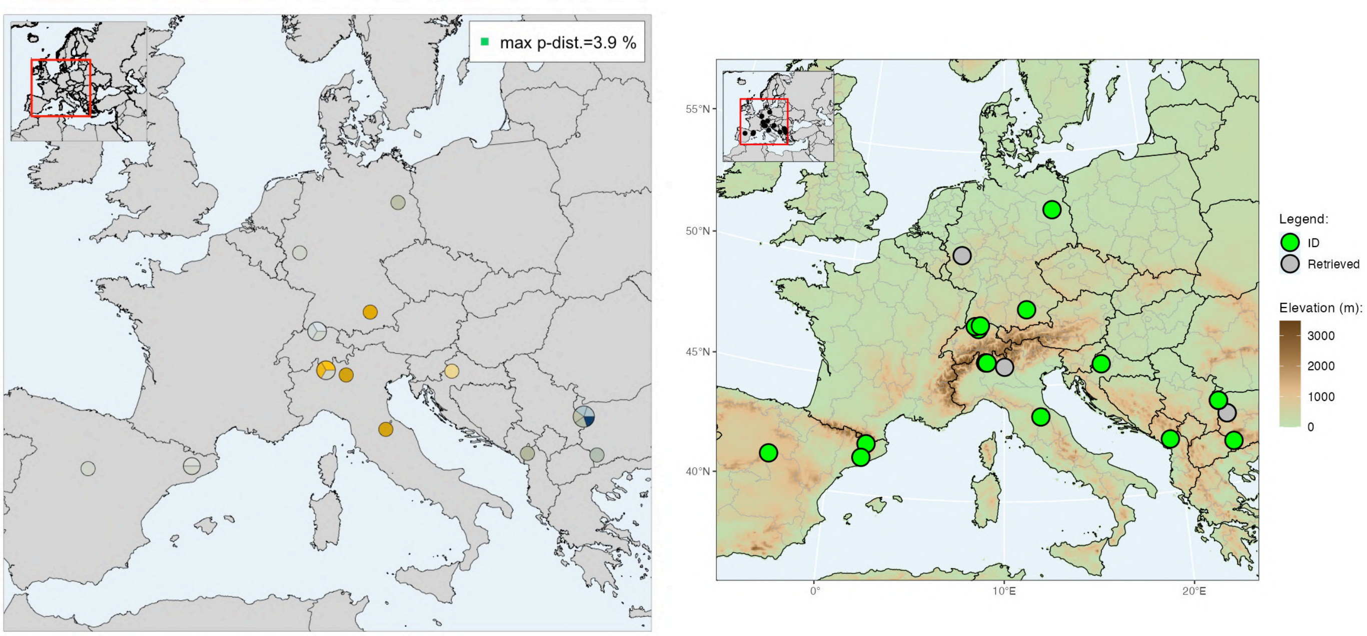

Figure 643: Genetic diversity map of *Temnothorax affinis* (Mayr, 1855). Nearby localities of sequenced specimens are merged in pies (left). Colours match the bidimensional colour space of the PCoA projection (Fig. 643 left) of p-dist between sequences (dots). Specimen identification (ID or cf.) and source (newly sequenced or retrieved) are represented by colours, while specimen attribute (terra typica, type locality, type specimen or faunistic novelty) is represented by the shape (right). Sequences: ID = 22, cf. = 0; maximum p-distance: strict = 3.9 %, less strict = 3.9 %.

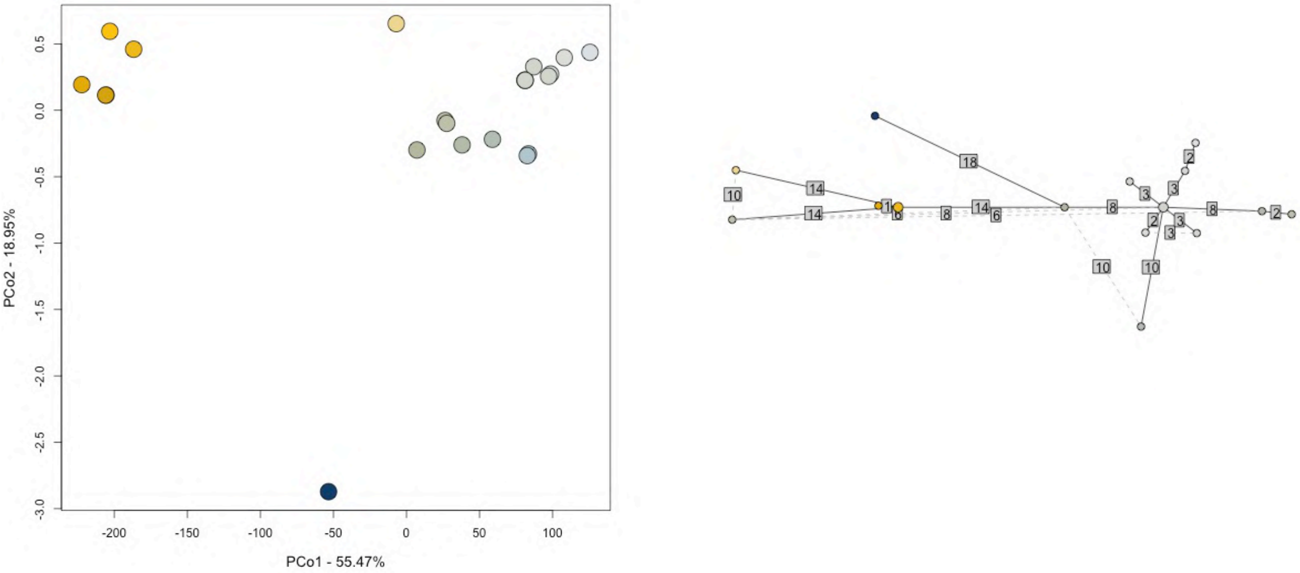

Figure 644: PCoA based on pairwise p-distances between *Temnothorax affinis* sequences (left). Colours match a bidimensional colour space. Haplotype network of *Temnothorax affinis* (right). Sequences > 599 bp: ID = 19, cf. = 0.

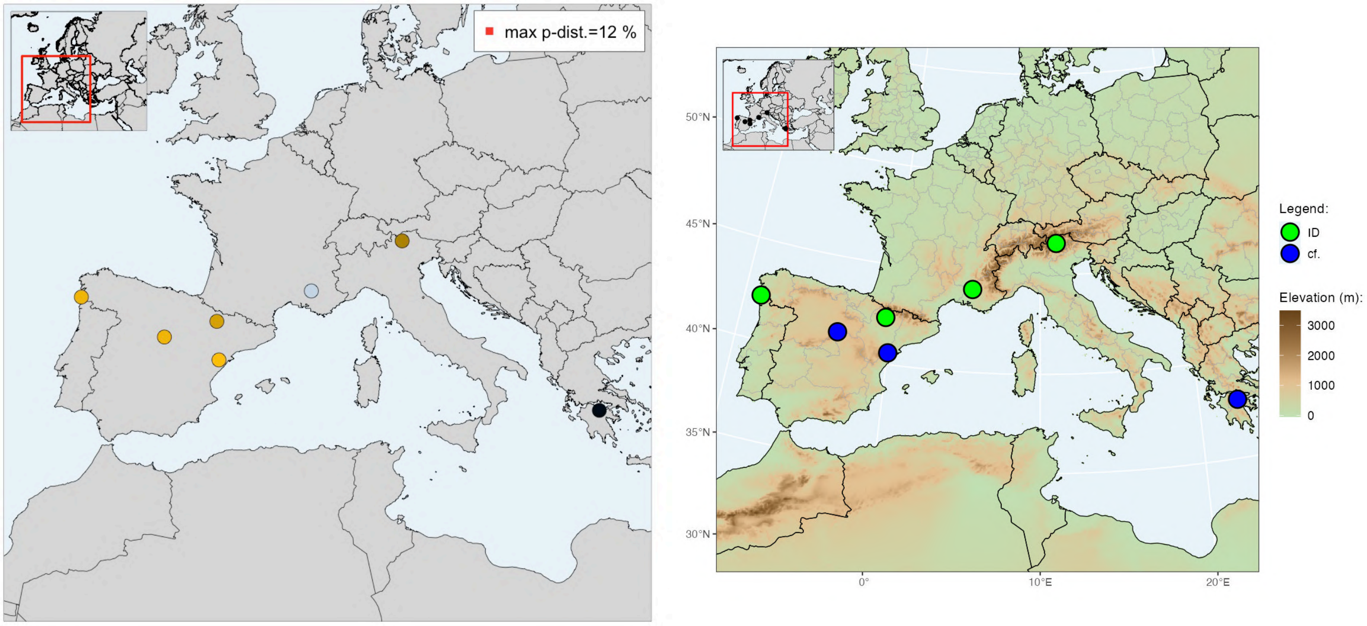

Figure 645: Genetic diversity map of *Temnothorax albipennis* (Curtis, 1854). Nearby localities of sequenced specimens are merged in pies (left). Colours match the bidimensional colour space of the PCoA projection (Fig. 645 left) of p-dist between sequences (dots). Specimen identification (ID or cf.) and source (newly sequenced or retrieved) are represented by colours, while specimen attribute (terra typica, type locality, type specimen or faunistic novelty) is represented by the shape (right). Sequences: ID = 4, cf. = 3; maximum p-distance: strict = 12 %, less strict = 12 %.

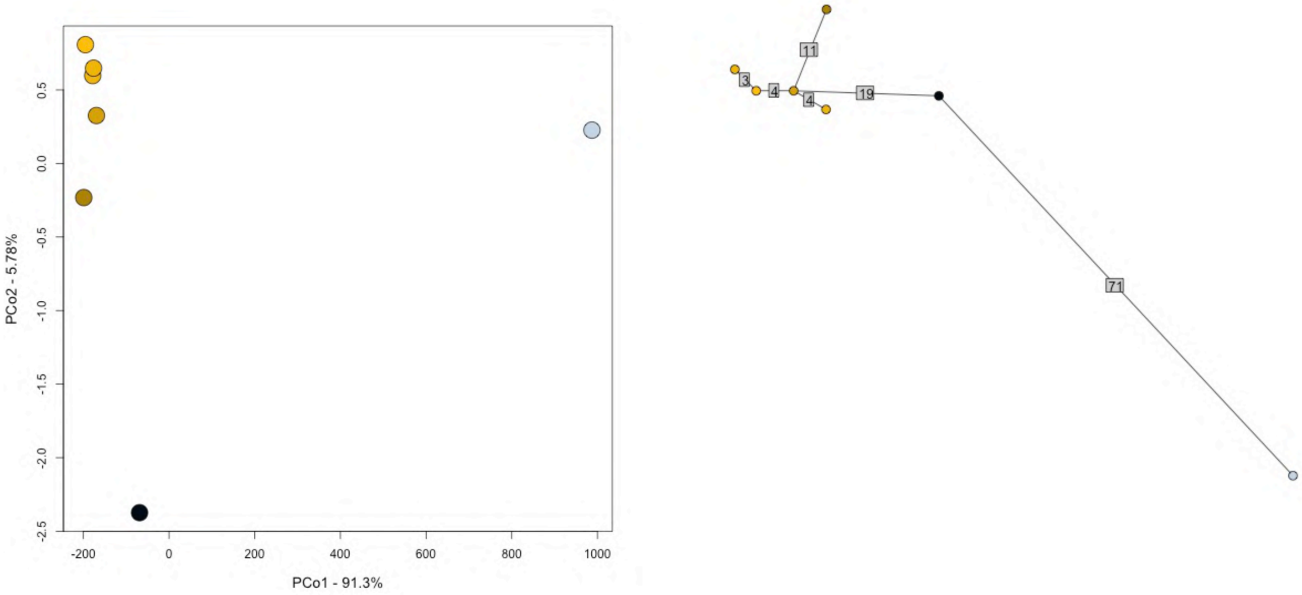

Figure 646: PCoA based on pairwise p-distances between *Temnothorax albipennis* sequences (left). Colours match a bidimensional colour space. Haplotype network of *Temnothorax albipennis* (right). Sequences > 599 bp: ID = 4, cf. = 3.

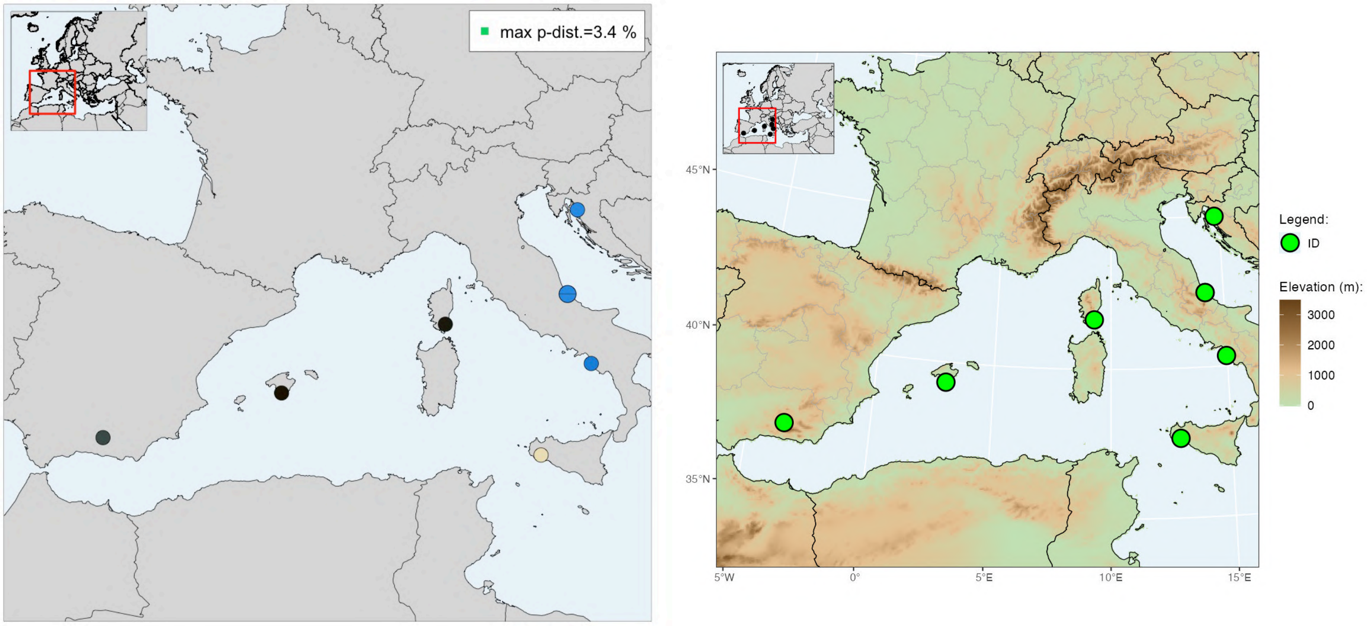

Figure 647: Genetic diversity map of *Temnothorax algiricus* (Forel, 1894). Nearby localities of sequenced specimens are merged in pies (left). Colours match the bidimensional colour space of the PCoA projection (Fig. 647 left) of p-dist between sequences (dots). Specimen identification (ID or cf.) and source (newly sequenced or retrieved) are represented by colours, while specimen attribute (terra typica, type locality, type specimen or faunistic novelty) is represented by the shape (right). Sequences: ID = 8, cf. = 0; maximum p-distance: strict = 3.4 %, less strict = 3.4 %.

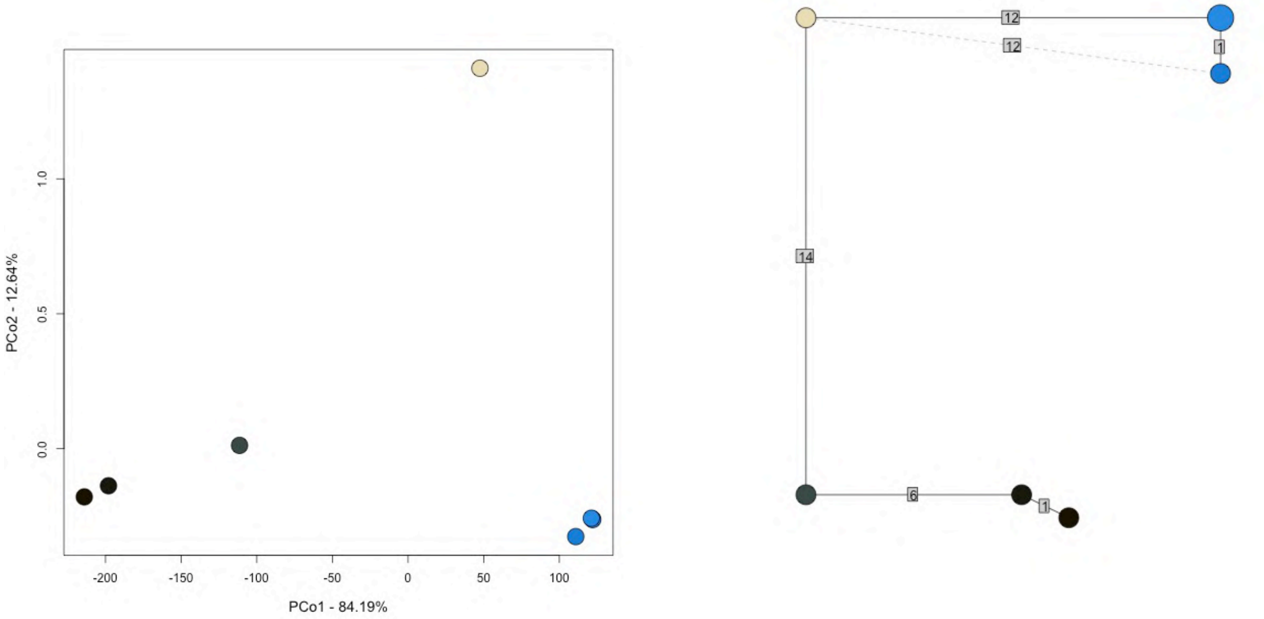

Figure 648: PCoA based on pairwise p-distances between *Temnothorax algiricus* sequences (left). Colours match a bidimensional colour space. Haplotype network of *Temnothorax algiricus* (right). Sequences > 599 bp: ID = 8, cf. = 0.

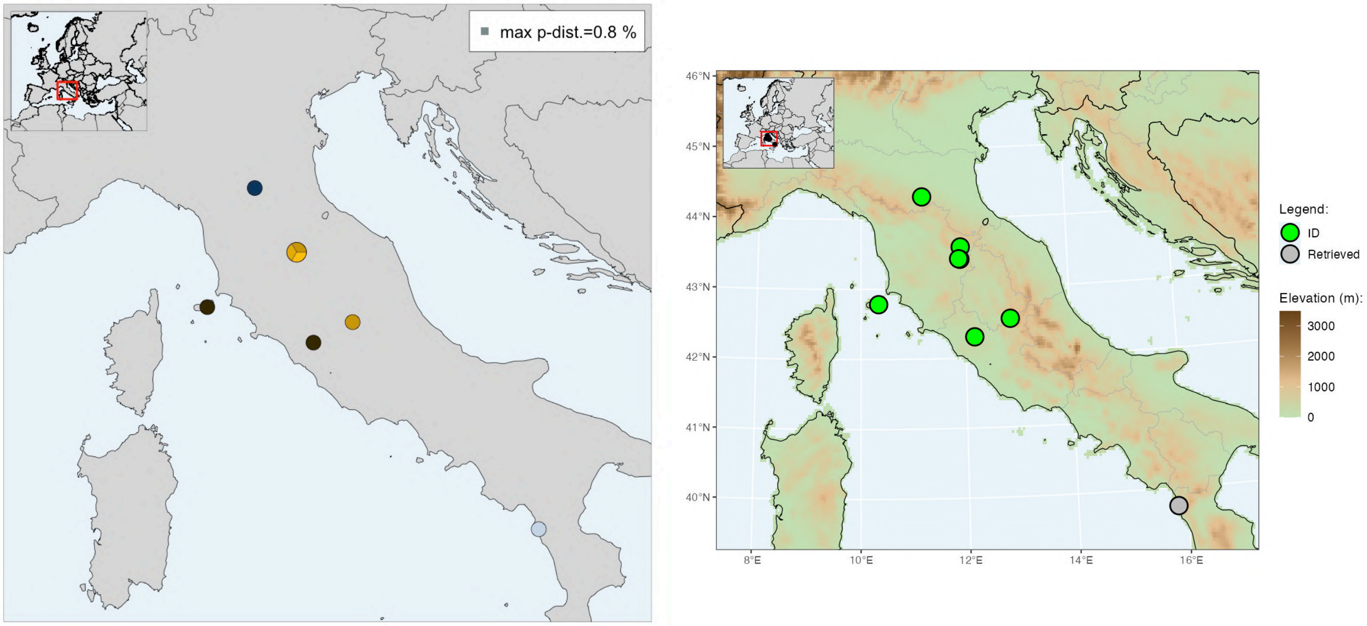

Figure 649: Genetic diversity map of *Temnothorax alienus* Schulz, Heinze & Pusch, 2007. Nearby localities of sequenced specimens are merged in pies (left). Colours match the bidimensional colour space of the PCoA projection (Fig. 649 left) of p-dist between sequences (dots). Specimen identification (ID or cf.) and source (newly sequenced or retrieved) are represented by colours, while specimen attribute (terra typica, type locality, type specimen or faunistic novelty) is represented by the shape (right). Sequences: ID = 8, cf. = 0; maximum p-distance: strict = 0.8 %, less strict = 0.8 %.

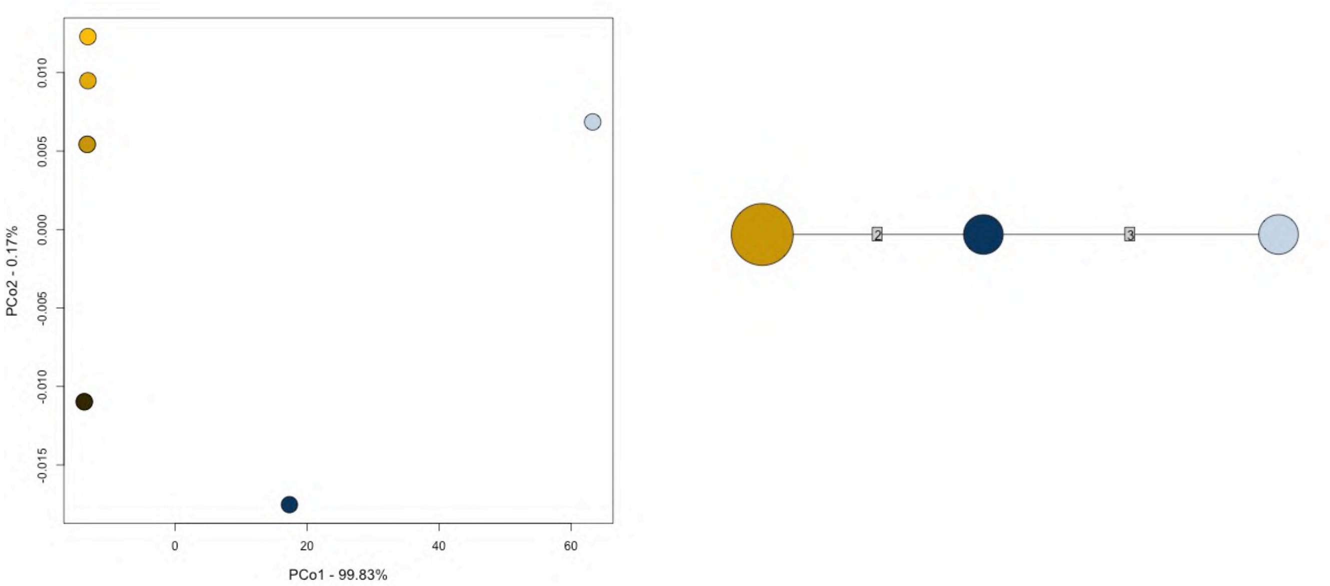

Figure 650: PCoA based on pairwise p-distances between *Temnothorax alienus* sequences (left). Colours match a bidimensional colour space. Haplotype network of *Temnothorax alienus* (right). Sequences > 599 bp: ID = 8, cf. = 0.

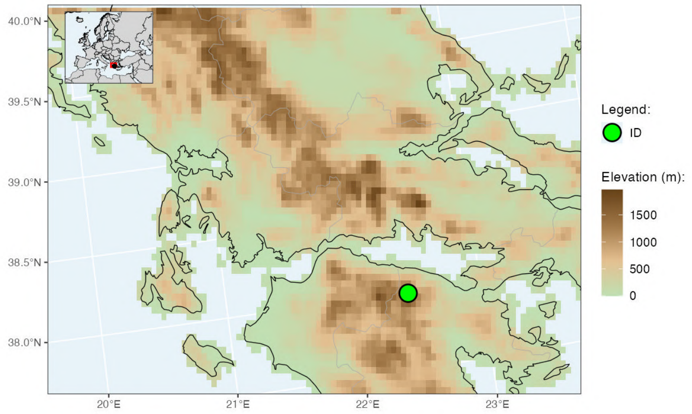

Figure 651: Map of *Temnothorax angulinodis* Csösz, Heinze & Mikó, 2015. Due to the presence of a single sequence, the genetic diversity map and the PCoA projection were not done. Specimen identification (ID or cf.) and source (newly sequenced or retrieved) are represented by colours, while specimen attribute (terra typica, type locality, type specimen or faunistic novelty) is represented by the shape. Sequences: ID = 1, cf. = 0; maximum p-distance: strict = NA, less strict = NA.

Haplotype network analysis of *Temnothorax angulinodis* was not possible.

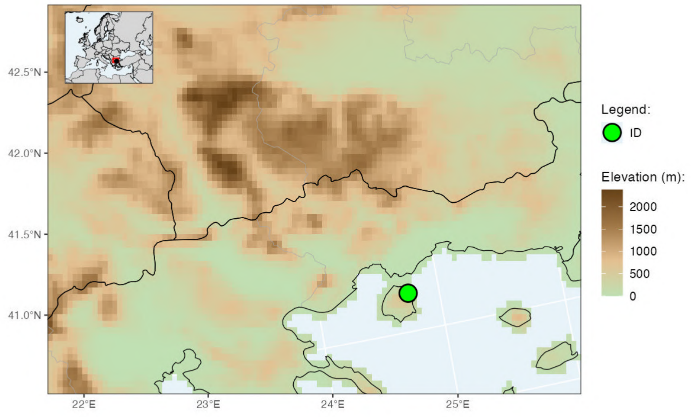

Figure 652: Map of *Temnothorax angustifrons* Csösz, Heinze & Mikó, 2015. Due to the presence of a single sequence, the genetic diversity map and the PCoA projection were not done. Specimen identification (ID or cf.) and source (newly sequenced or retrieved) are represented by colours, while specimen attribute (terra typica, type locality, type specimen or faunistic novelty) is represented by the shape. Sequences: ID = 1, cf. = 0; maximum p-distance: strict = NA, less strict = NA.

Haplotype network analysis of *Temnothorax angustifrons* was not possible.

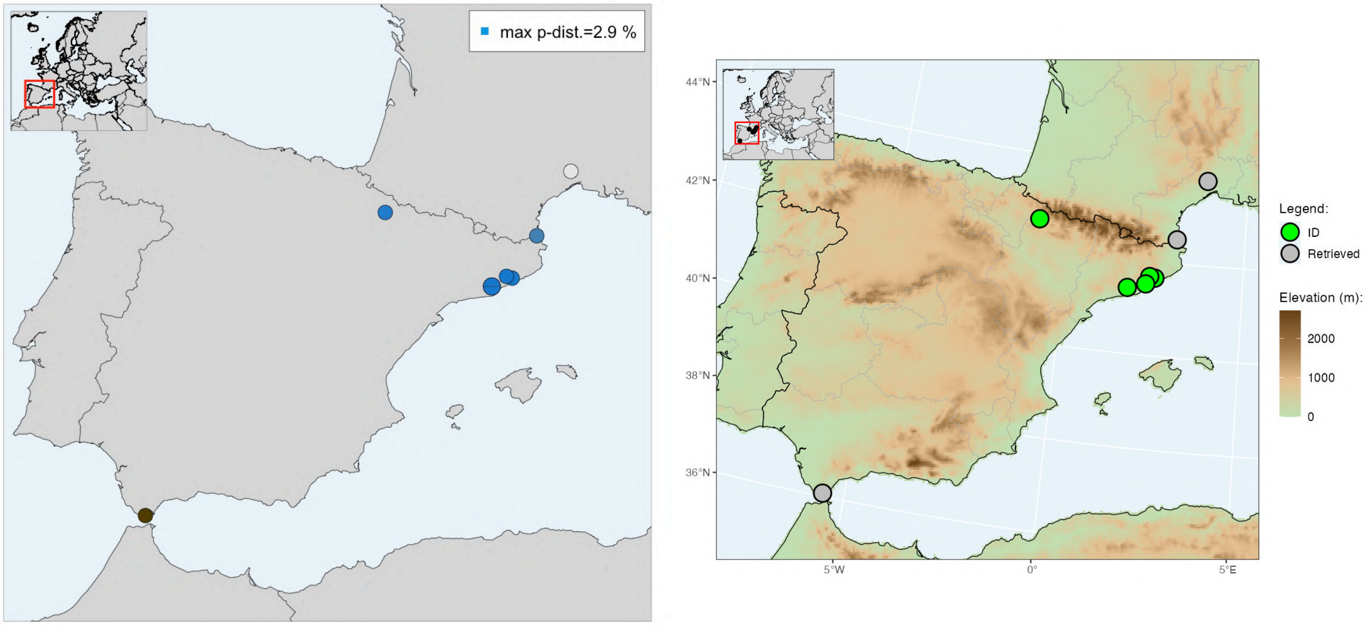

Figure 653: Genetic diversity map of *Temnothorax angustulus* (Nylander, 1856). Nearby localities of sequenced specimens are merged in pies (left). Colours match the bidimensional colour space of the PCoA projection (Fig. 653 left) of p-dist between sequences (dots). Specimen identification (ID or cf.) and source (newly sequenced or retrieved) are represented by colours, while specimen attribute (terra typica, type locality, type specimen or faunistic novelty) is represented by the shape (right). Sequences: ID = 8, cf. = 0; maximum p-distance: strict = 2.9 %, less strict = 2.9 %.

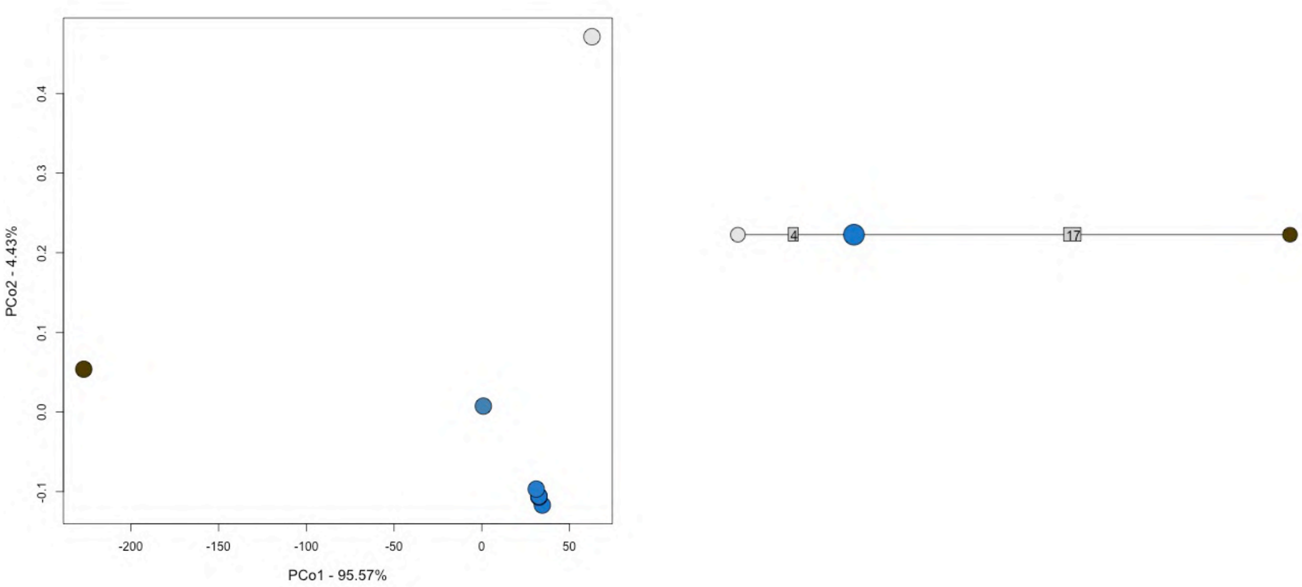

Figure 654: PCoA based on pairwise p-distances between *Temnothorax angustulus* sequences (left). Colours match a bidimensional colour space. Haplotype network of *Temnothorax angustulus* (right). Sequences > 599 bp: ID = 6, cf. = 0.

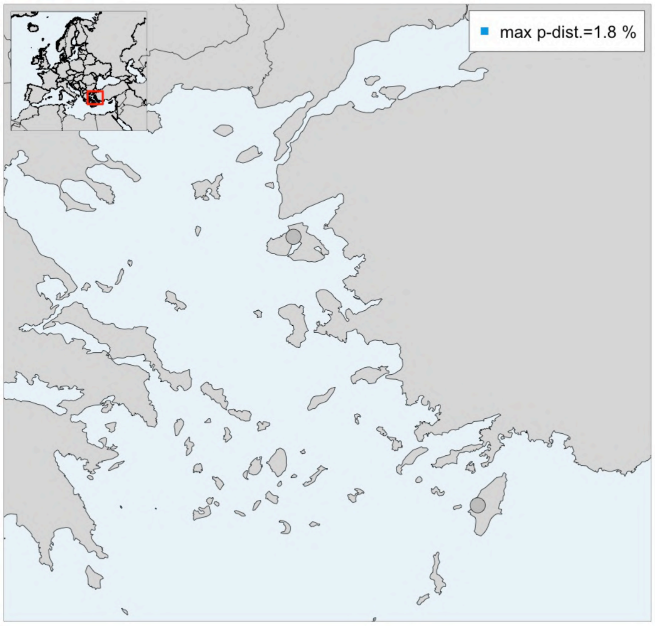

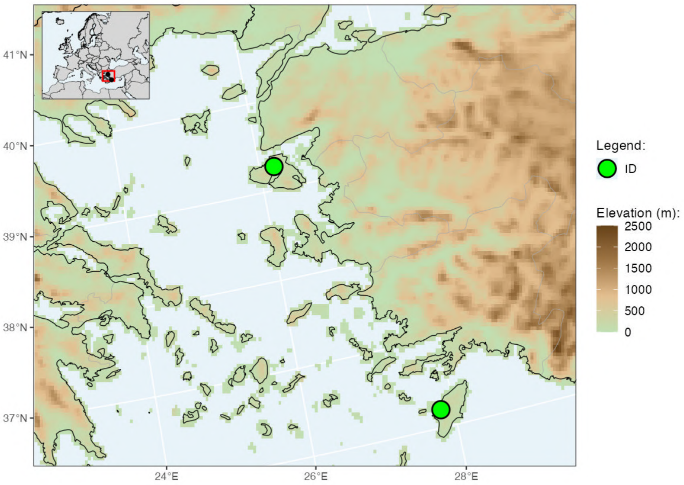

Figure 655: Genetic diversity map of *Temnothorax antigoni* (Forel, 1911). PCoA projection was not done and therefore sequenced specimens in the genetic diversity map are coloured in gray (left). Specimen identification (ID or cf.) and source (newly sequenced or retrieved) are represented by colours, while specimen attribute (terra typica, type locality, type specimen or faunistic novelty) is represented by the shape (right). Sequences: ID = 2, cf. = 0; maximum p-distance: strict = NA, less strict = 1.8 %.

Haplotype network analysis of *Temnothorax antigoni* was not possible.

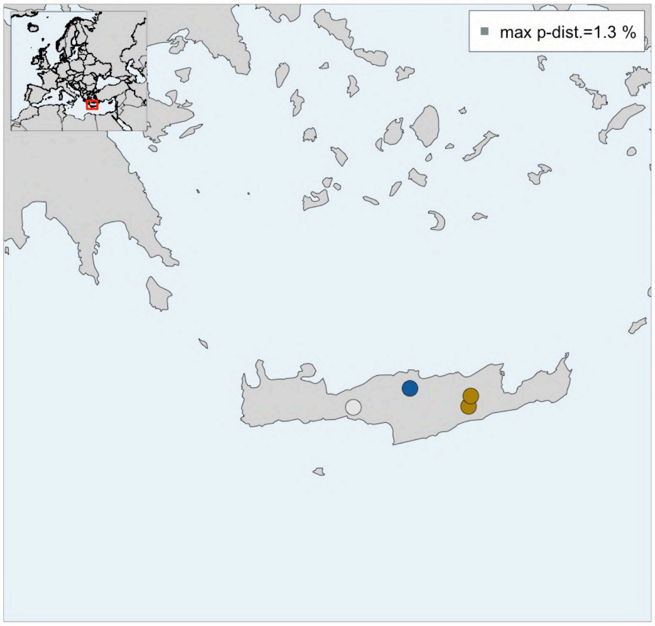

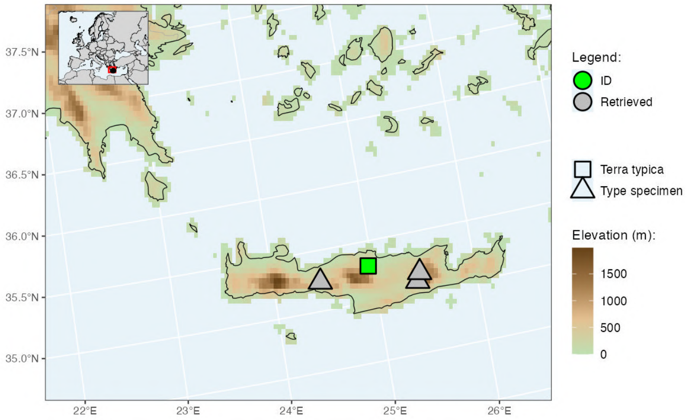

Figure 656: Genetic diversity map of *Temnothorax ariadnae* Csösz, Heinze & Mikó, 2015. Nearby localities of sequenced specimens are merged in pies (left). Colours match the bidimensional colour space of the PCoA projection (Fig. 656 left) of p-dist between sequences (dots). Specimen identification (ID or cf.) and source (newly sequenced or retrieved) are represented by colours, while specimen attribute (terra typica, type locality, type specimen or faunistic novelty) is represented by the shape (right). Sequences: ID = 4, cf. = 0; maximum p-distance: strict = 1.3 %, less strict = 1.3 %.

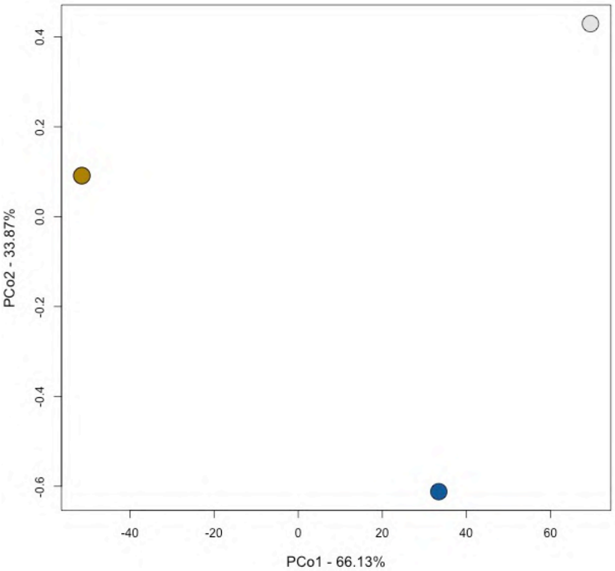

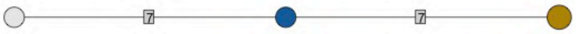

Figure 657: PCoA based on pairwise p-distances between *Temnothorax ariadnae* sequences (left). Colours match a bidimensional colour space. Haplotype network of *Temnothorax ariadnae* (right). Sequences > 599 bp: ID = 4, cf. = 0.

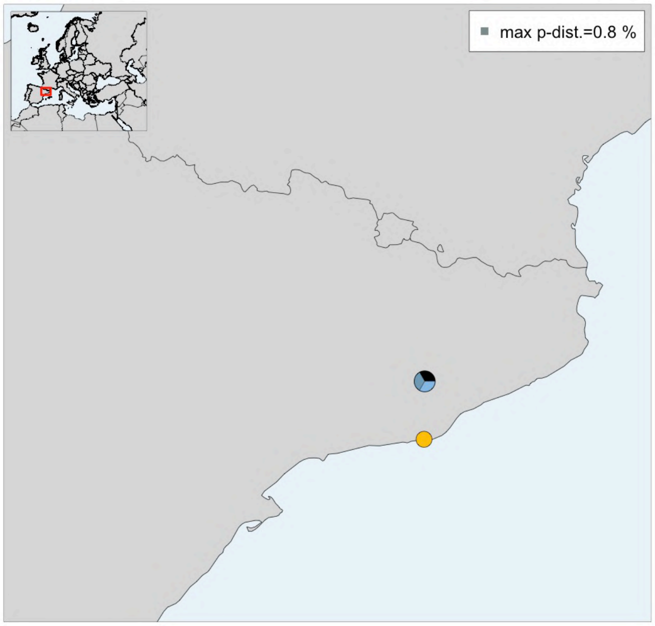

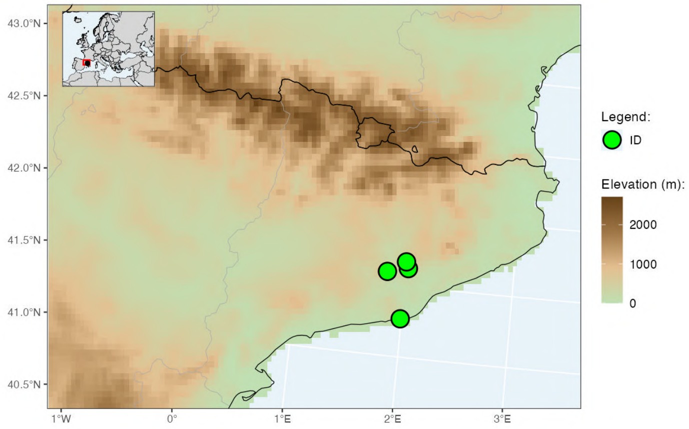

Figure 658: Genetic diversity map of *Temnothorax atlantis* (Santschi, 1911). Nearby localities of sequenced specimens are merged in pies (left). Colours match the bidimensional colour space of the PCoA projection (Fig. 658 left) of p-dist between sequences (dots). Specimen identification (ID or cf.) and source (newly sequenced or retrieved) are represented by colours, while specimen attribute (terra typica, type locality, type specimen or faunistic novelty) is represented by the shape (right). Sequences: ID = 4, cf. = 0; maximum p-distance: strict = 0.8 %, less strict = 0.8 %.

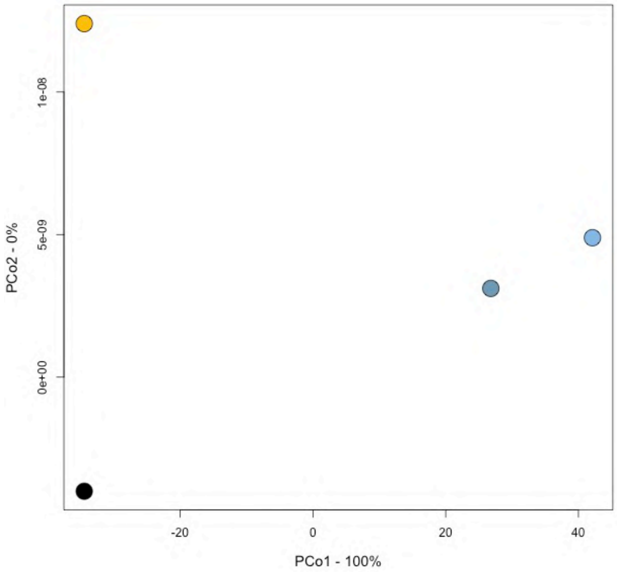

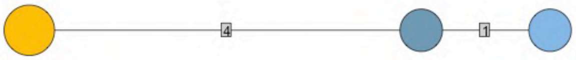

Figure 659: PCoA based on pairwise p-distances between *Temnothorax atlantis* sequences (left). Colours match a bidimensional colour space. Haplotype network of *Temnothorax atlantis* (right). Sequences > 599 bp: ID = 4, cf. = 0.

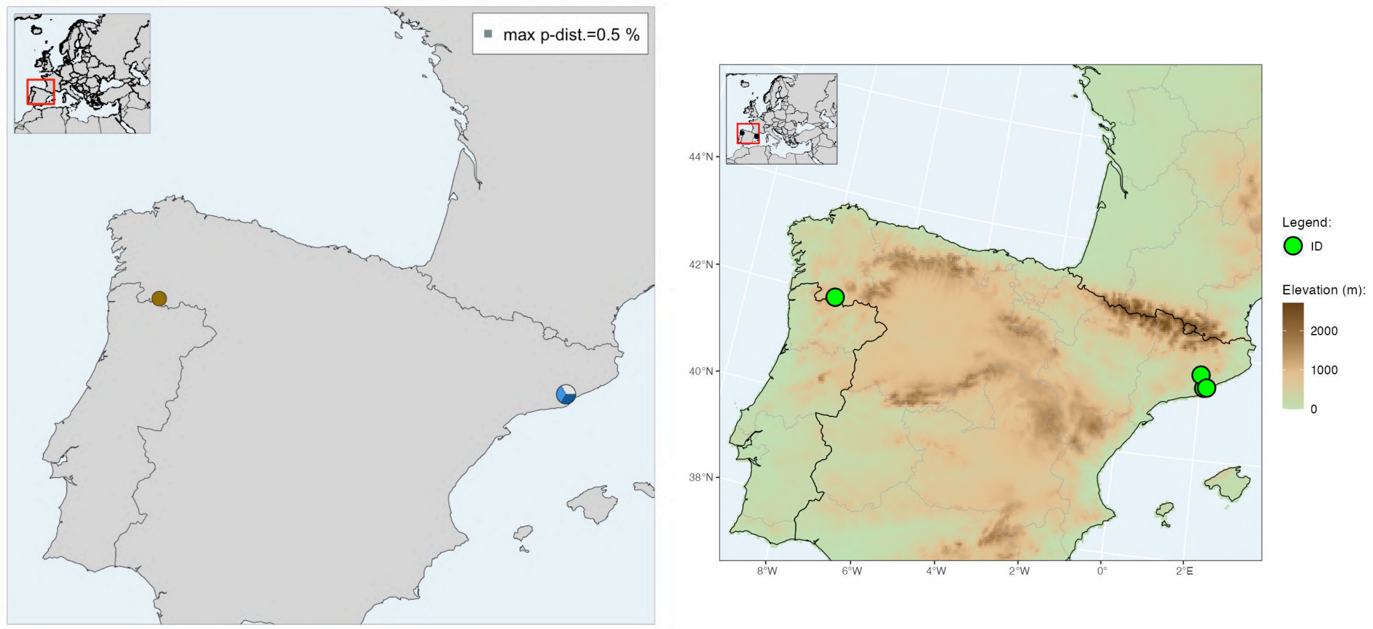

Figure 660: Genetic diversity map of *Temnothorax aveli* (Bondroit, 1918). Nearby localities of sequenced specimens are merged in pies (left). Colours match the bidimensional colour space of the PCoA projection (Fig. 660 left) of p-dist between sequences (dots). Specimen identification (ID or cf.) and source (newly sequenced or retrieved) are represented by colours, while specimen attribute (terra typica, type locality, type specimen or faunistic novelty) is represented by the shape (right). Sequences: ID = 4, cf. = 0; maximum p-distance: strict = 0.5 %, less strict = 0.5 %.

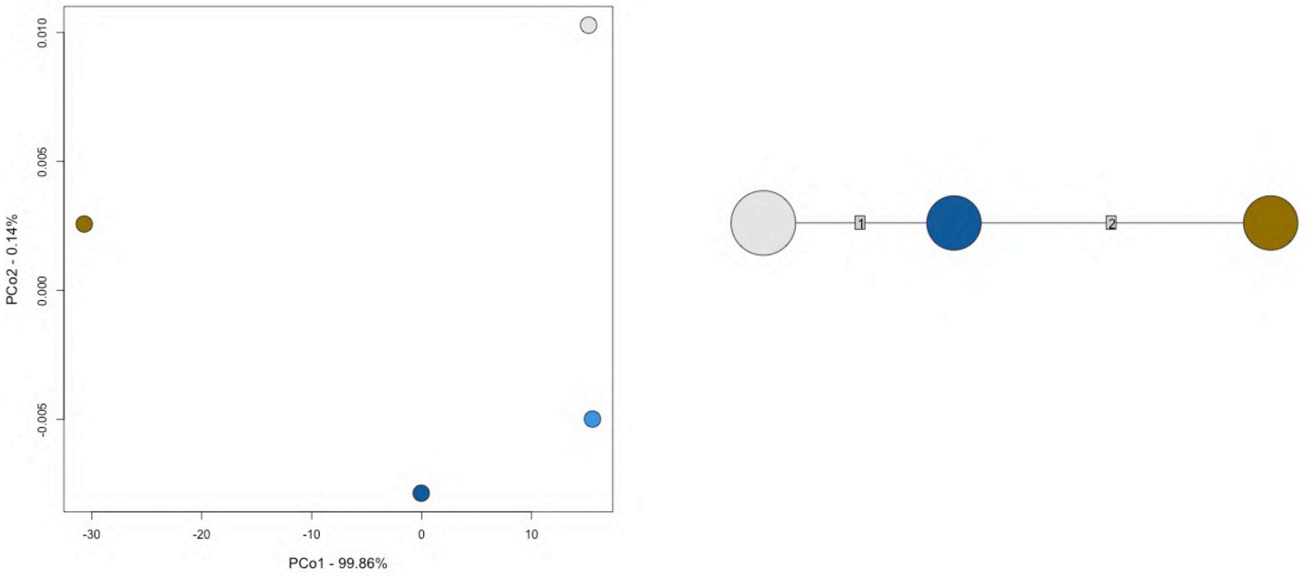

Figure 661: PCoA based on pairwise p-distances between *Temnothorax aveli* sequences (left). Colours match a bidimensional colour space. Haplotype network of *Temnothorax aveli* (right). Sequences > 599 bp: ID = 4, cf. = 0.

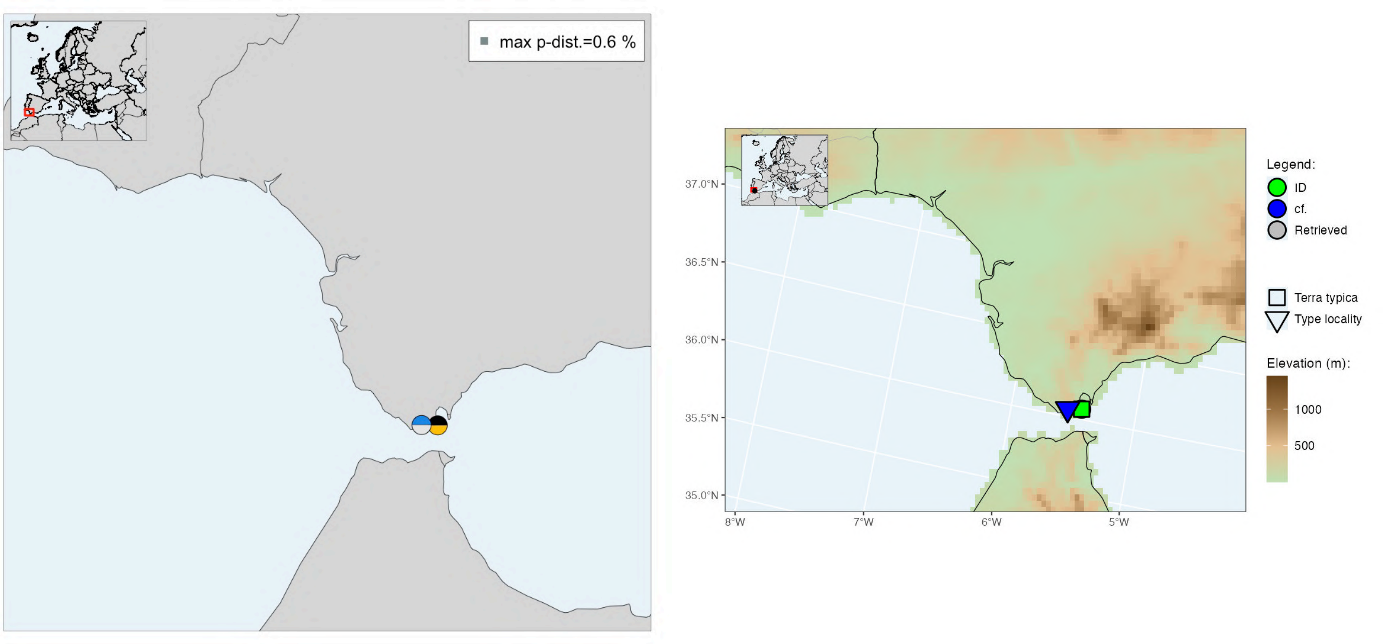

Figure 662: Genetic diversity map of *Temnothorax baeticus* (Emery, 1924). Nearby localities of sequenced specimens are merged in pies (left). Colours match the bidimensional colour space of the PCoA projection (Fig. 662 left) of p-dist between sequences (dots). Specimen identification (ID or cf.) and source (newly sequenced or retrieved) are represented by colours, while specimen attribute (terra typica, type locality, type specimen or faunistic novelty) is represented by the shape (right). Sequences: ID = 3, cf. = 1; maximum p-distance: strict = 0.6 %, less strict = 0.6 %.

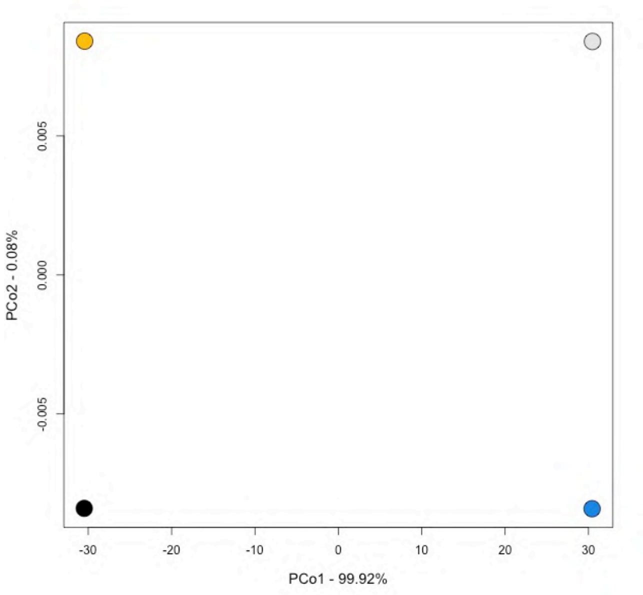

Figure 663: PCoA based on pairwise p-distances between *Temnothorax baeticus* sequences (left). Colours match a bidimensional colour space. Haplotype network analysis of *Temnothorax baeticus* was not possible.

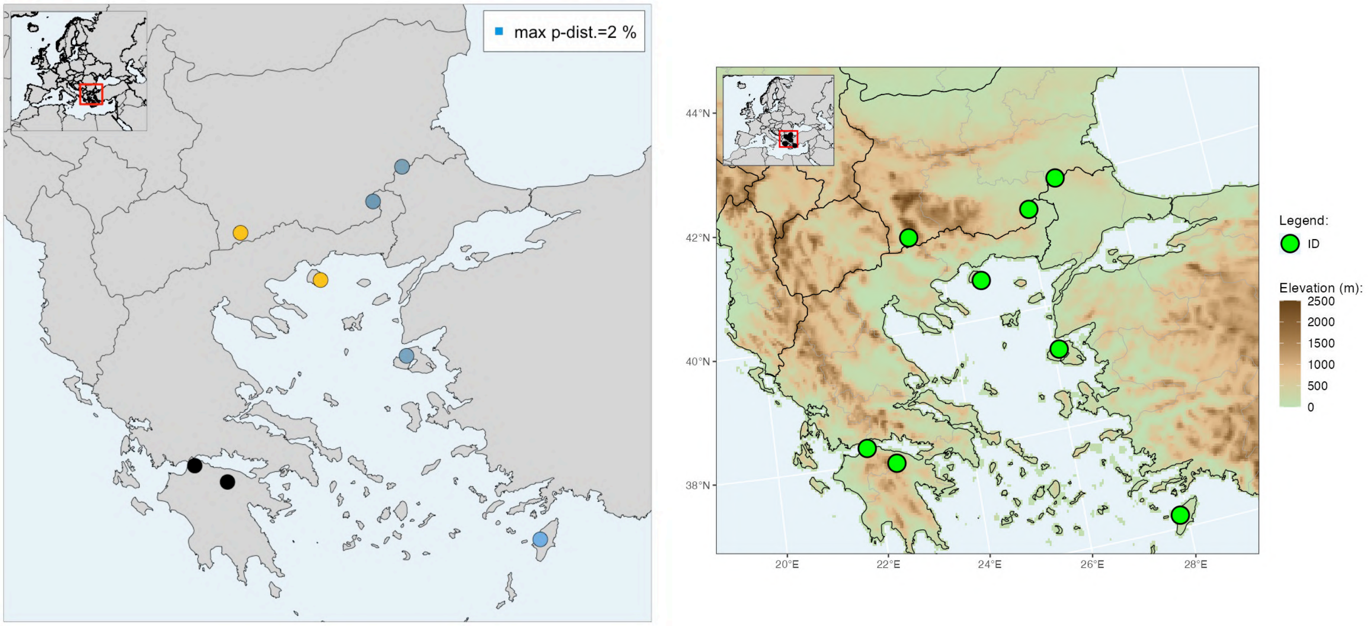

Figure 664: Genetic diversity map of *Temnothorax balcanicus* (André, 1881). Nearby localities of sequenced specimens are merged in pies (left). Colours match the bidimensional colour space of the PCoA projection (Fig. 664 left) of p-dist between sequences (dots). Specimen identification (ID or cf.) and source (newly sequenced or retrieved) are represented by colours, while specimen attribute (terra typica, type locality, type specimen or faunistic novelty) is represented by the shape (right). Sequences: ID = 8, cf. = 0; maximum p-distance: strict = 2 %, less strict = 2 %.

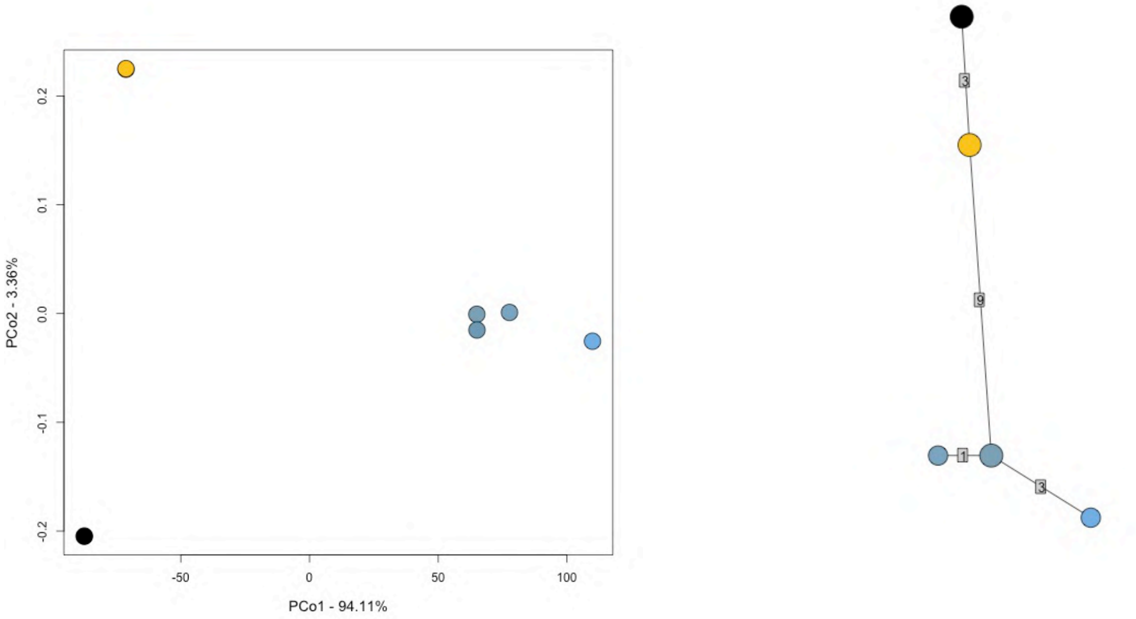

Figure 665: PCoA based on pairwise p-distances between *Temnothorax balcanicus* sequences (left). Colours match a bidimensional colour space. Haplotype network of *Temnothorax balcanicus* (right). Sequences > 599 bp: ID = 8, cf. = 0.

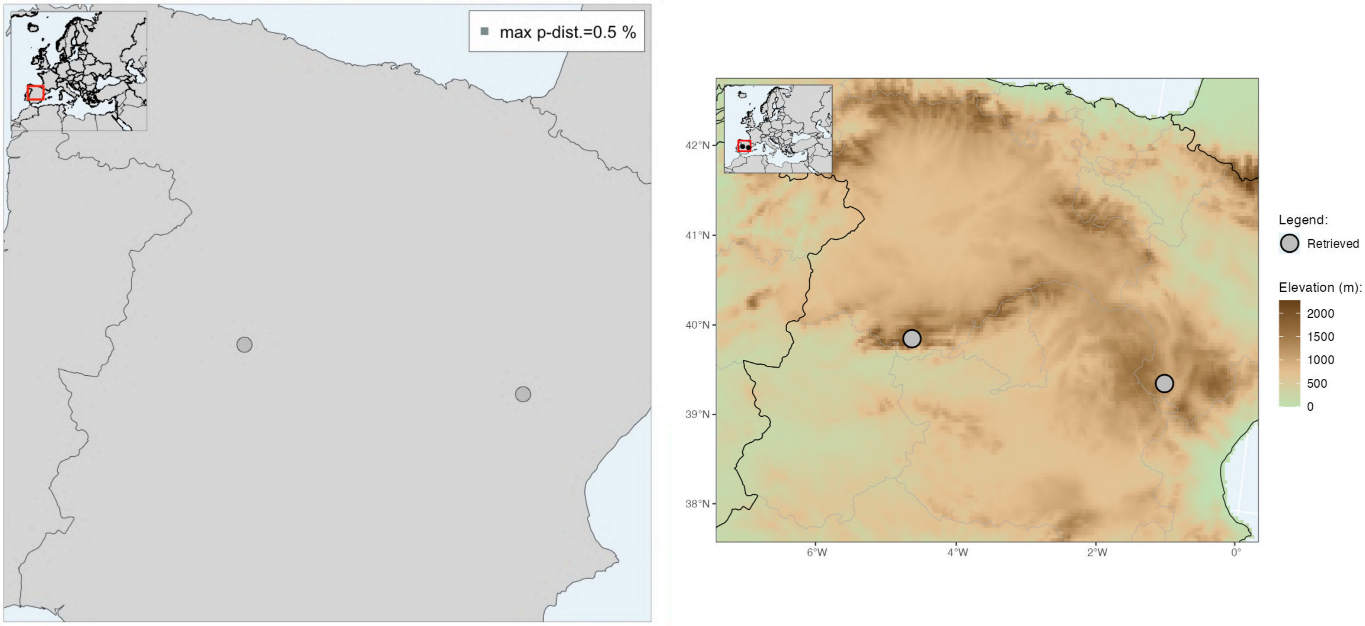

Figure 666: Genetic diversity map of *Temnothorax bernardi* (Espadaler, 1982). PCoA projection was not done and therefore sequenced specimens in the genetic diversity map are coloured in gray (left). Specimen identification (ID or cf.) and source (newly sequenced or retrieved) are represented by colours, while specimen attribute (terra typica, type locality, type specimen or faunistic novelty) is represented by the shape (right). Sequences: ID = 2, cf. = 0; maximum p-distance: strict = NA, less strict = 0.5 %.

Haplotype network analysis of *Temnothorax bernardi* was not possible.

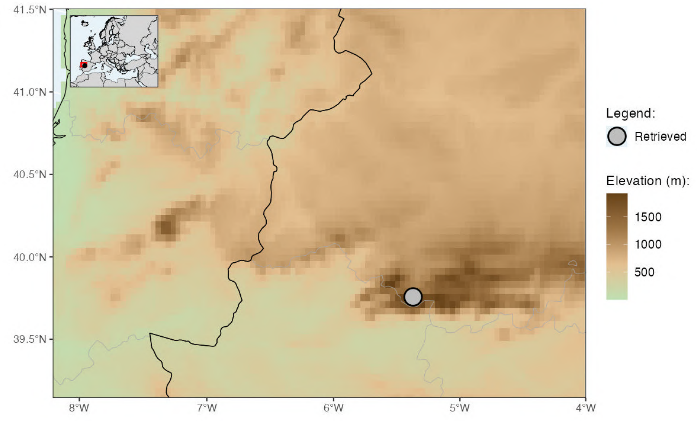

Figure 667: Map of *Temnothorax blascoi* (Espadaler, 1997). Due to the presence of a single sequence, the genetic diversity map and the PCoA projection were not done. Specimen identification (ID or cf.) and source (newly sequenced or retrieved) are represented by colours, while specimen attribute (terra typica, type locality, type specimen or faunistic novelty) is represented by the shape. Sequences: ID = 1, cf. = 0; maximum p-distance: strict = NA, less strict = NA.

Haplotype network analysis of *Temnothorax blascoi* was not possible.

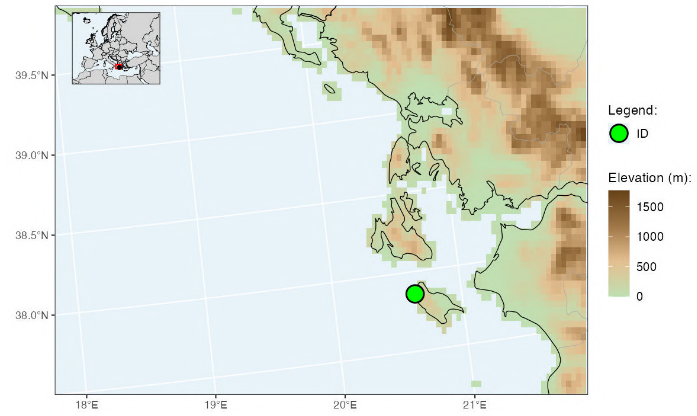

Figure 668: Map of *Temnothorax brackoi* Salata & Borowiec, 2019. Due to the presence of a single sequence, the genetic diversity map and the PCoA projection were not done. Specimen identification (ID or cf.) and source (newly sequenced or retrieved) are represented by colours, while specimen attribute (terra typica, type locality, type specimen or faunistic novelty) is represented by the shape. Sequences: ID = 1, cf. = 0; maximum p-distance: strict = NA, less strict = NA.

Haplotype network analysis of *Temnothorax brackoi* was not possible.

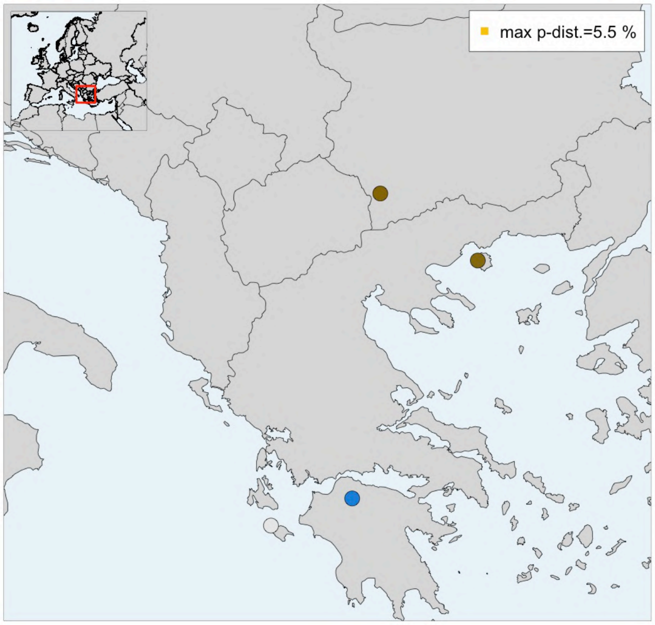

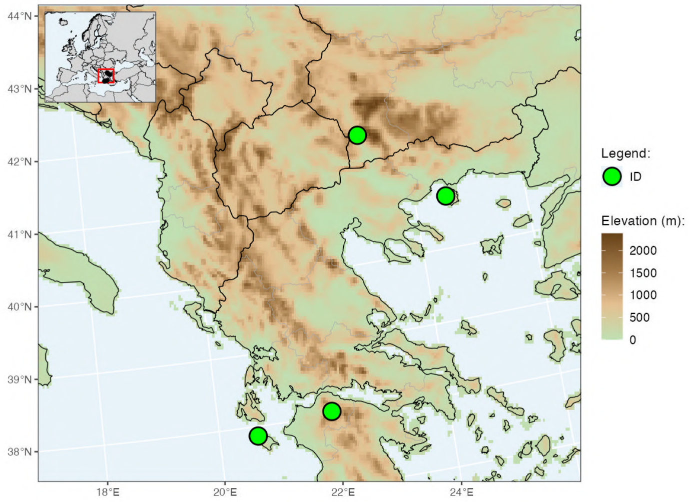

Figure 669: Genetic diversity map of *Temnothorax bulgaricus* (Forel, 1892). Nearby localities of sequenced specimens are merged in pies (left). Colours match the bidimensional colour space of the PCoA projection (Fig. 669 left) of p-dist between sequences (dots). Specimen identification (ID or cf.) and source (newly sequenced or retrieved) are represented by colours, while specimen attribute (terra typica, type locality, type specimen or faunistic novelty) is represented by the shape (right). Sequences: ID = 4, cf. = 0; maximum p-distance: strict = 5.5 %, less strict = 5.5 %.

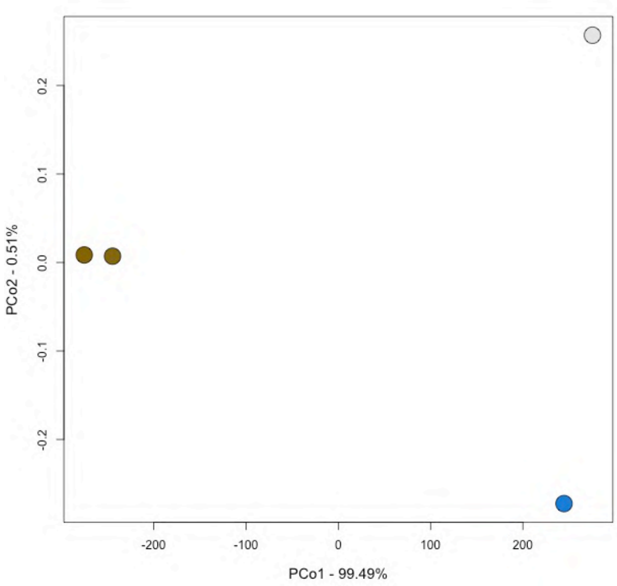

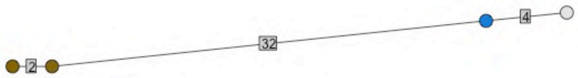

Figure 670: PCoA based on pairwise p-distances between *Temnothorax bulgaricus* sequences (left). Colours match a bidimensional colour space. Haplotype network of *Temnothorax bulgaricus* (right). Sequences > 599 bp: ID = 4, cf. = 0.

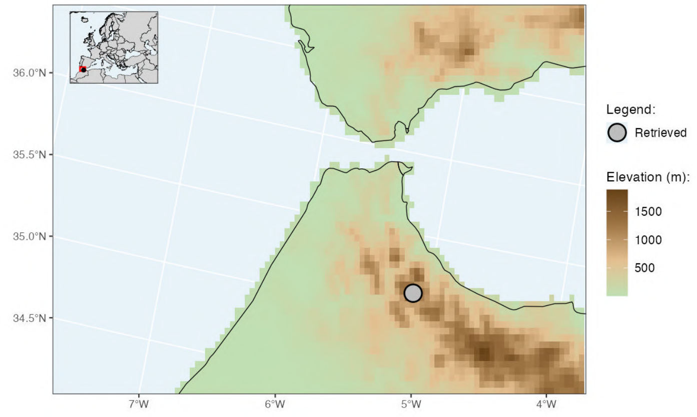

Figure 671: Map of *Temnothorax cagnianti* (Tinaut, 1983). Due to the presence of a single sequence, the genetic diversity map and the PCoA projection were not done. Specimen identification (ID or cf.) and source (newly sequenced or retrieved) are represented by colours, while specimen attribute (terra typica, type locality, type specimen or faunistic novelty) is represented by the shape. Sequences: ID = 1, cf. = 0; maximum p-distance: strict = NA, less strict = NA.

Haplotype network analysis of *Temnothorax cagnianti* was not possible.

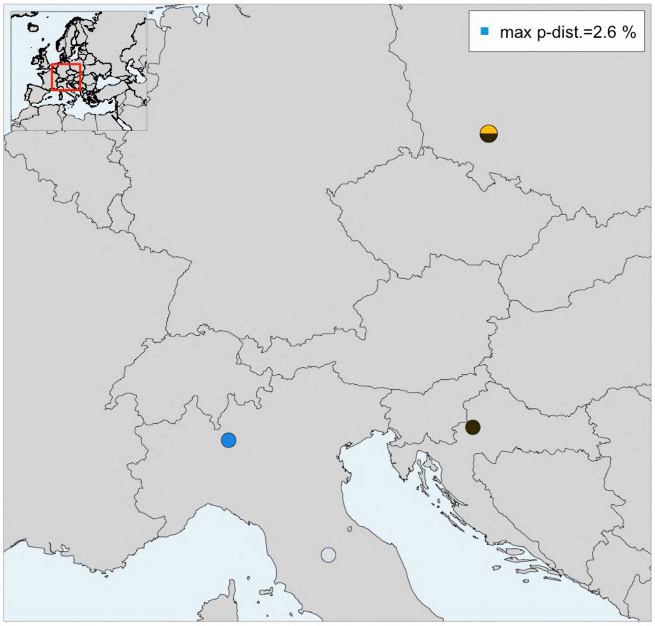

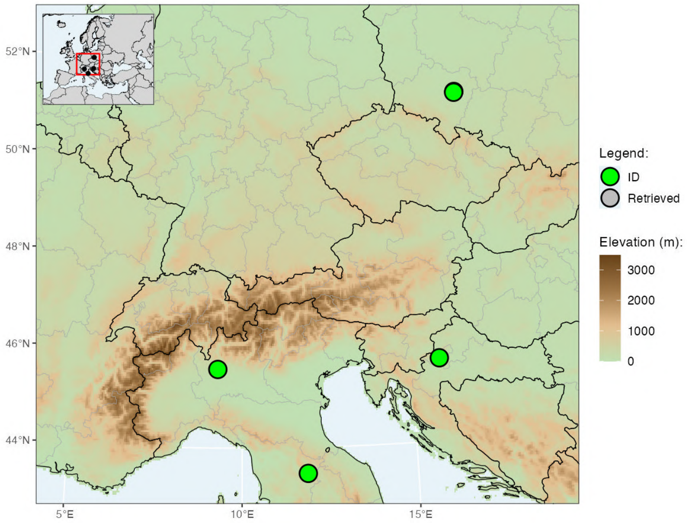

Figure 672: Genetic diversity map of *Temnothorax clypeatus* (Mayr, 1853). Nearby localities of sequenced specimens are merged in pies (left). Colours match the bidimensional colour space of the PCoA projection (Fig. 672 left) of p-dist between sequences (dots). Specimen identification (ID or cf.) and source (newly sequenced or retrieved) are represented by colours, while specimen attribute (terra typica, type locality, type specimen or faunistic novelty) is represented by the shape (right). Sequences: ID = 5, cf. = 0; maximum p-distance: strict = 2.6 %, less strict = 2.6 %.

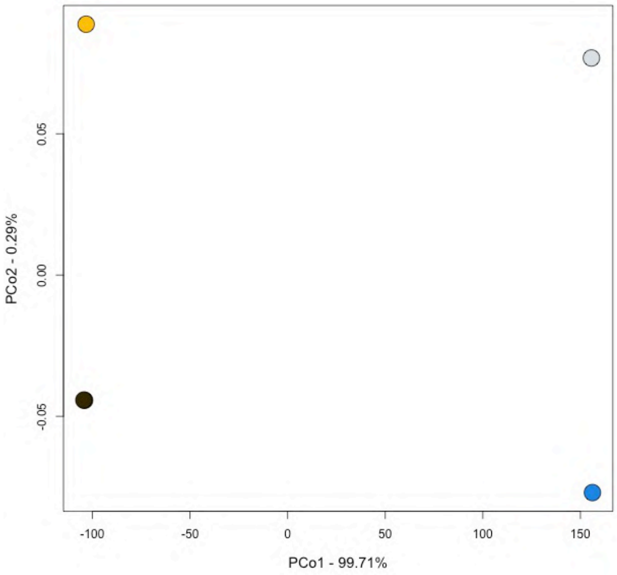

Figure 673: PCoA based on pairwise p-distances between *Temnothorax clypeatus* sequences (left). Colours match a bidimensional colour space. Haplotype network analysis of *Temnothorax clypeatus* was not possible.

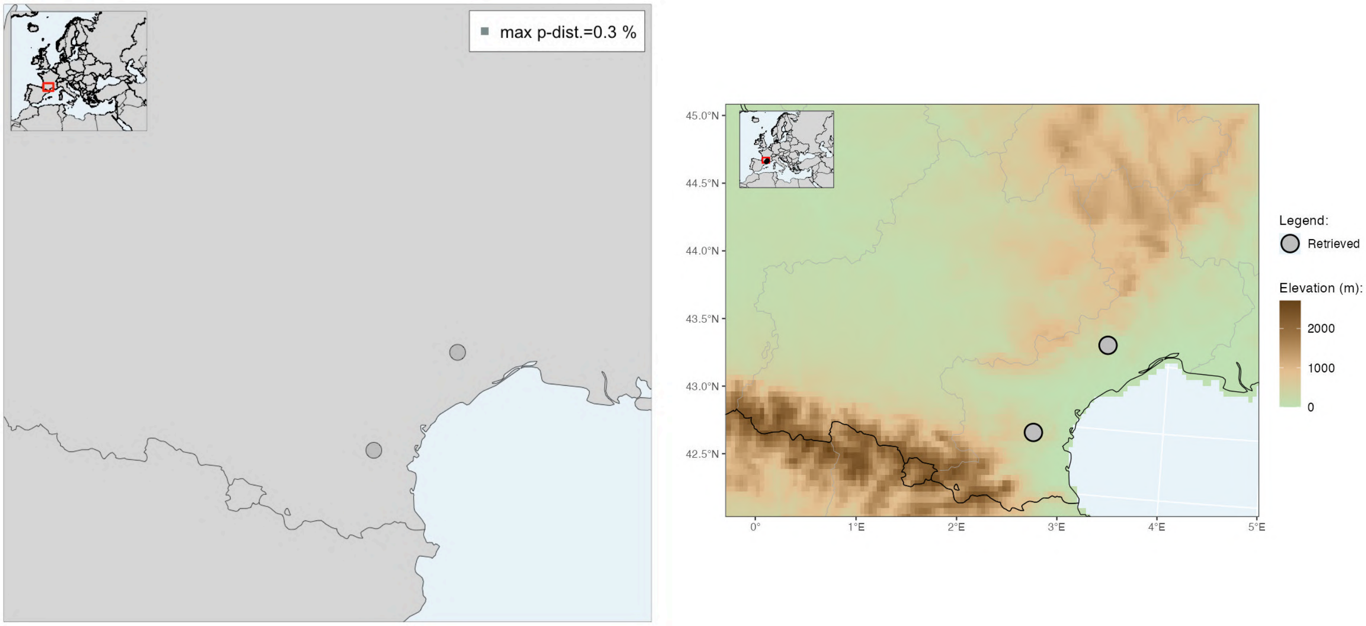

Figure 674: Genetic diversity map of *Temnothorax continentalis* Galkowski & Cagniant, 2017. PCoA projection was not done and therefore sequenced specimens in the genetic diversity map are coloured in gray (left). Specimen identification (ID or cf.) and source (newly sequenced or retrieved) are represented by colours, while specimen attribute (terra typica, type locality, type specimen or faunistic novelty) is represented by the shape (right). Sequences: ID = 2, cf. = 0; maximum p-distance: strict = NA, less strict = 0.3 %.

Haplotype network analysis of *Temnothorax continentalis* was not possible.

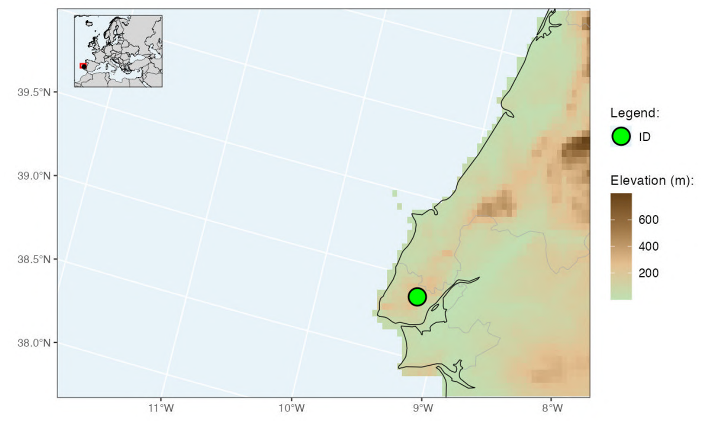

Figure 675: Map of *Temnothorax convexus* (Forel, 1894). Due to the presence of a single sequence, the genetic diversity map and the PCoA projection were not done. Specimen identification (ID or cf.) and source (newly sequenced or retrieved) are represented by colours, while specimen attribute (terra typica, type locality, type specimen or faunistic novelty) is represented by the shape. Sequences: ID = 1, cf. = 0; maximum p-distance: strict = NA, less strict = NA.

Haplotype network analysis of *Temnothorax convexus* was not possible.

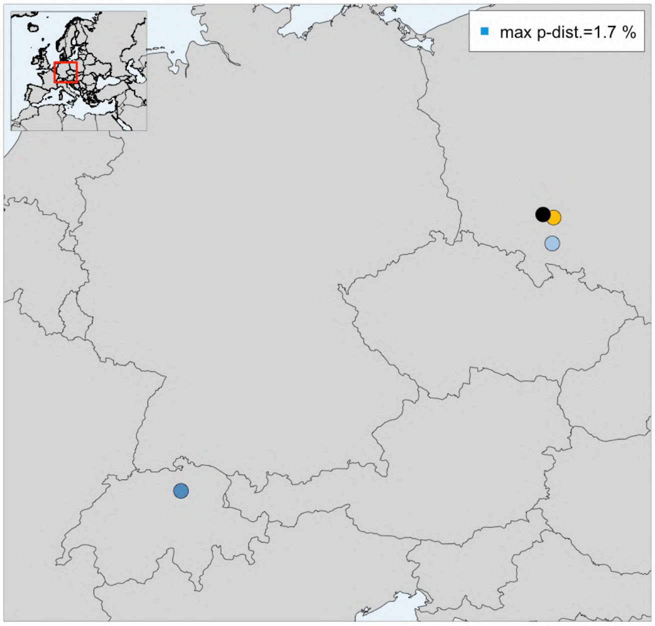

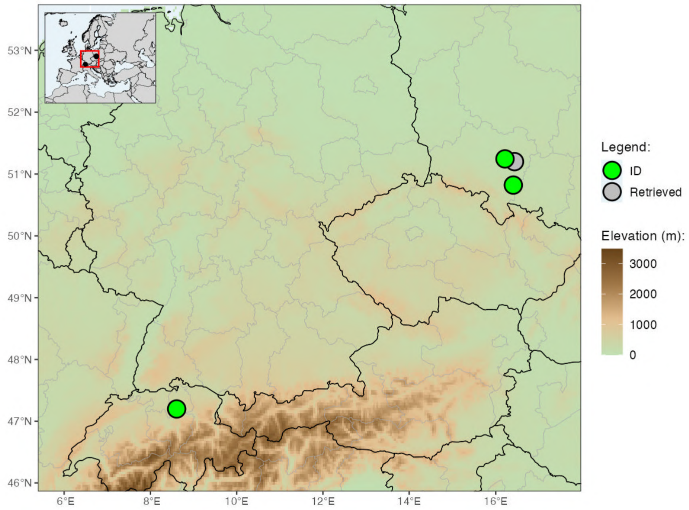

Figure 676: Genetic diversity map of *Temnothorax corticalis* (Schenck, 1852). Nearby localities of sequenced specimens are merged in pies (left). Colours match the bidimensional colour space of the PCoA projection (Fig. 676 left) of p-dist between sequences (dots). Specimen identification (ID or cf.) and source (newly sequenced or retrieved) are represented by colours, while specimen attribute (terra typica, type locality, type specimen or faunistic novelty) is represented by the shape (right). Sequences: ID = 4, cf. = 0; maximum p-distance: strict = 1.7 %, less strict = 1.7 %.

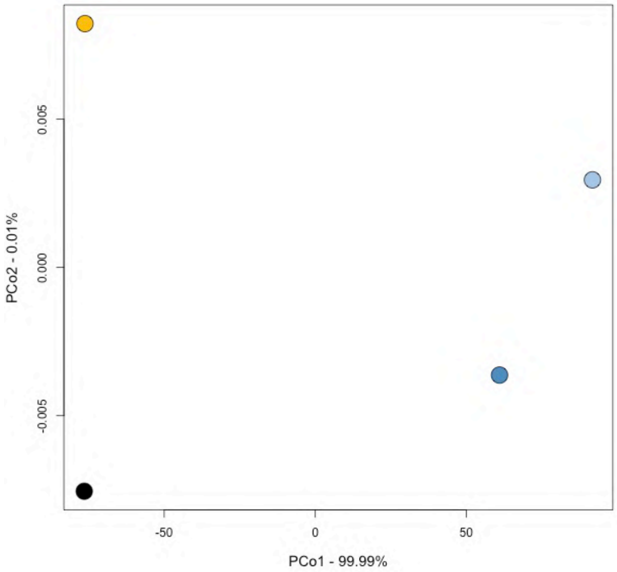

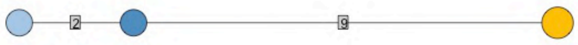

Figure 677: PCoA based on pairwise p-distances between *Temnothorax corticalis* sequences (left). Colours match a bidimensional colour space. Haplotype network of *Temnothorax corticalis* (right). Sequences > 599 bp: ID = 4, cf. = 0.

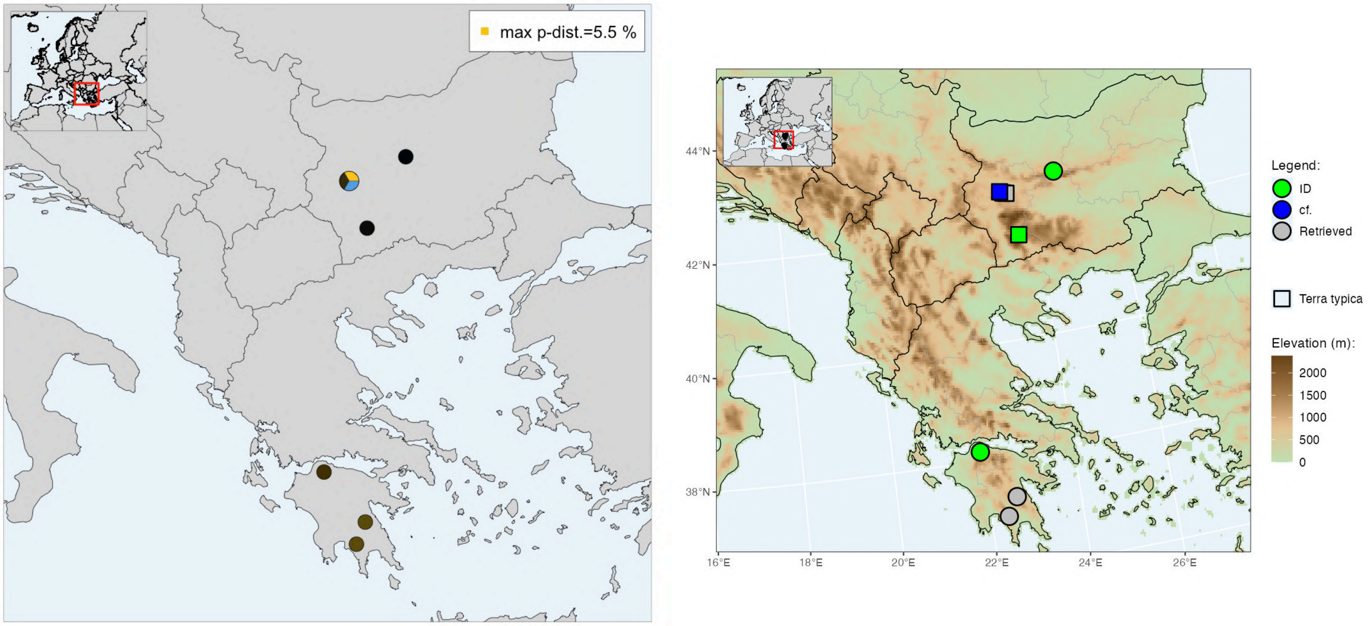

Figure 678: Genetic diversity map of *Temnothorax crasecundus* Seifert & Csősz, 2015. Nearby localities of sequenced specimens are merged in pies (left). Colours match the bidimensional colour space of the PCoA projection (Fig. 678 left) of p-dist between sequences (dots). Specimen identification (ID or cf.) and source (newly sequenced or retrieved) are represented by colours, while specimen attribute (terra typica, type locality, type specimen or faunistic novelty) is represented by the shape (right). Sequences: ID = 7, cf. = 1; maximum p-distance: strict = 2.7 %, less strict = 5.5 %.

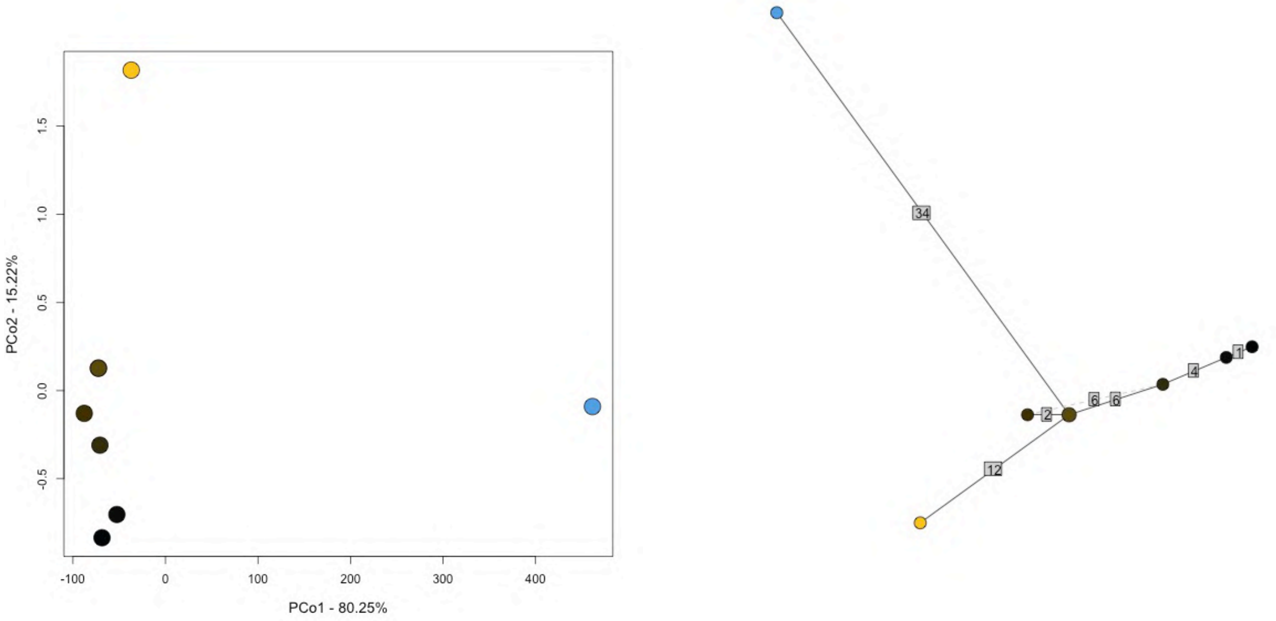

Figure 679: PCoA based on pairwise p-distances between *Temnothorax crasecundus* sequences (left). Colours match a bidimensional colour space. Haplotype network of *Temnothorax crasecundus* (right). Sequences > 599 bp: ID = 7, cf. = 1.

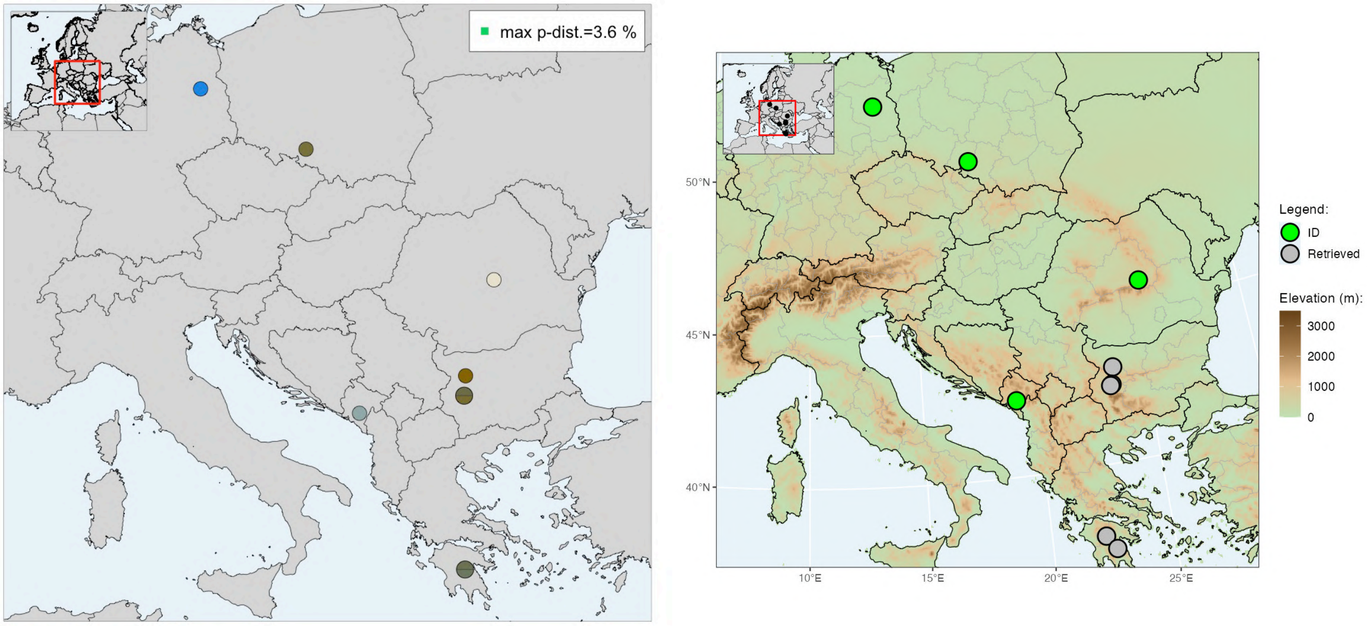

Figure 680: Genetic diversity map of *Temnothorax crassispinus* (Karavaiev, 1926). Nearby localities of sequenced specimens are merged in pies (left). Colours match the bidimensional colour space of the PCoA projection (Fig. 680 left) of p-dist between sequences (dots). Specimen identification (ID or cf.) and source (newly sequenced or retrieved) are represented by colours, while specimen attribute (terra typica, type locality, type specimen or faunistic novelty) is represented by the shape (right). Sequences: ID = 9, cf. = 0; maximum p-distance: strict = 3.6 %, less strict = 3.6 %.

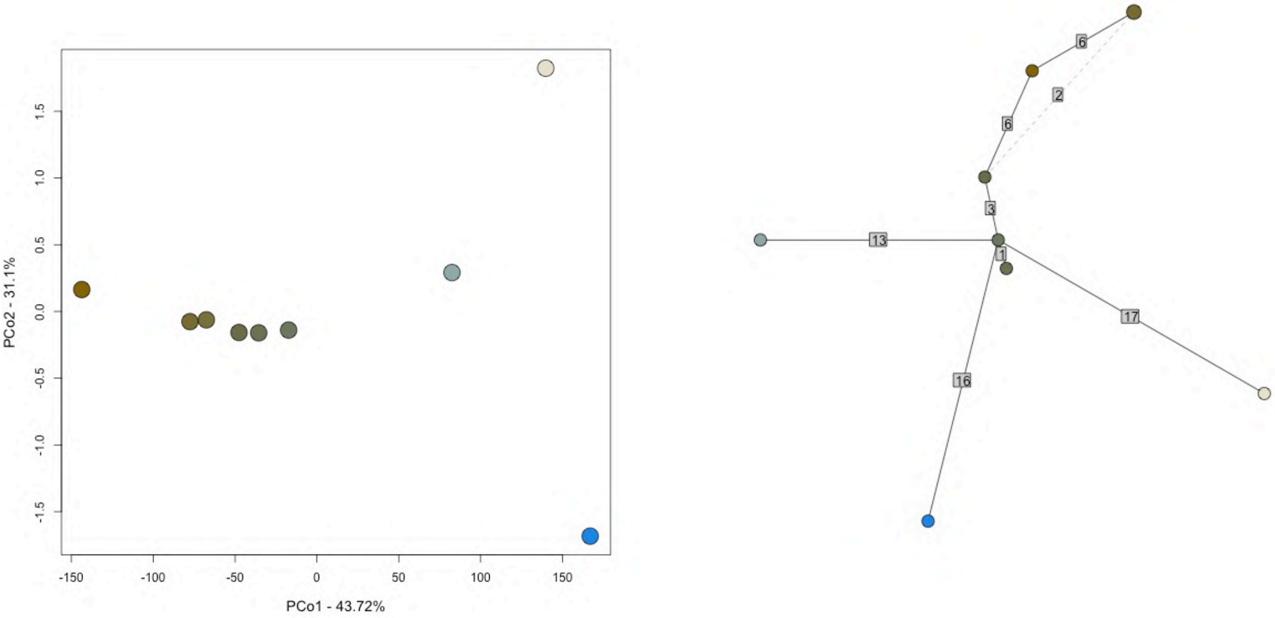

Figure 681: PCoA based on pairwise p-distances between *Temnothorax crassispinus* sequences (left). Colours match a bidimensional colour space. Haplotype network of *Temnothorax crassispinus* (right). Sequences > 599 bp: ID = 9, cf. = 0.

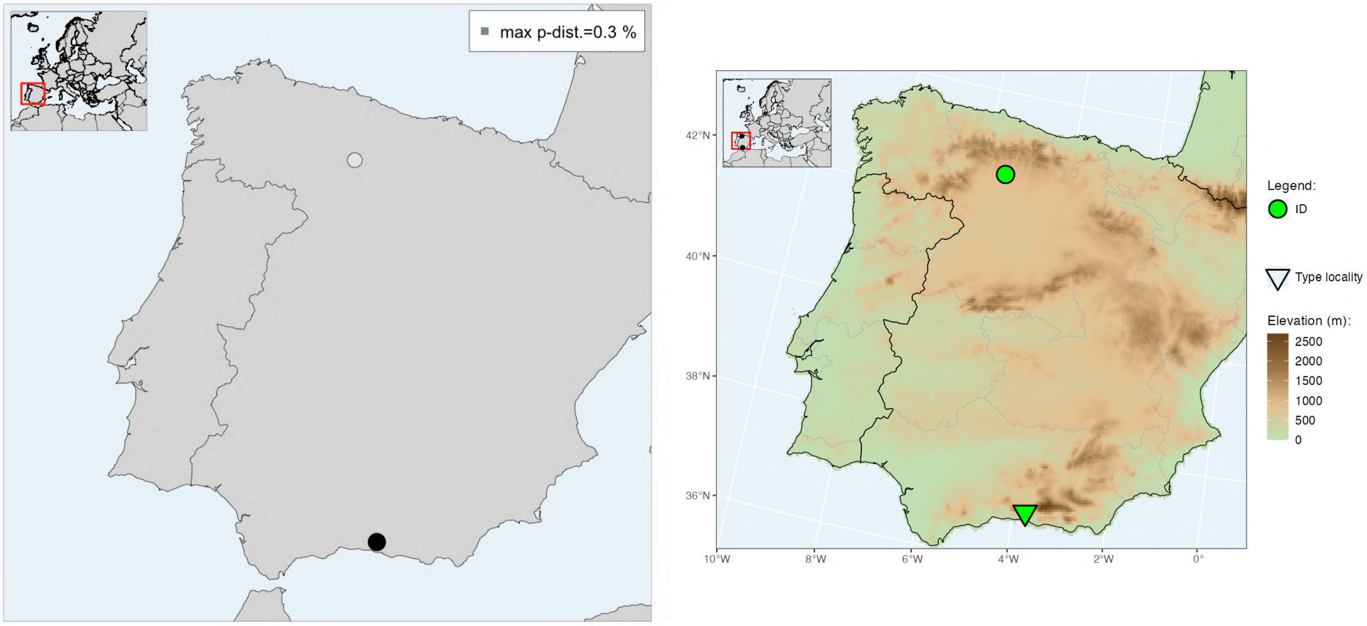

Figure 682: Genetic diversity map of *Temnothorax crepuscularis* (Tinaut, 1994). Nearby localities of sequenced specimens are merged in pies (left). Colours match the bidimensional colour space of the PCoA projection (Fig. 682 left) of p-dist between sequences (dots). Specimen identification (ID or cf.) and source (newly sequenced or retrieved) are represented by colours, while specimen attribute (terra typica, type locality, type specimen or faunistic novelty) is represented by the shape (right). Sequences: ID = 3, cf. = 0; maximum p-distance: strict = 0.3 %, less strict = 0.3 %.

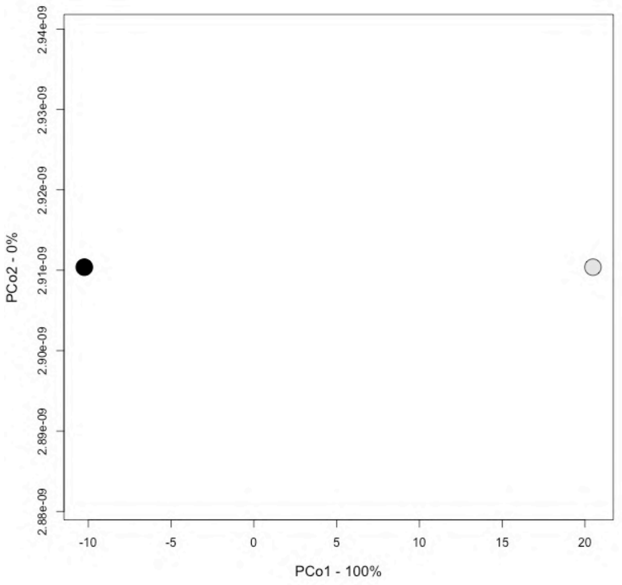

Figure 683: PCoA based on pairwise p-distances between *Temnothorax crepuscularis* sequences (left). Colours match a bidimensional colour space. Haplotype network analysis of *Temnothorax crepuscularis* was not possible.

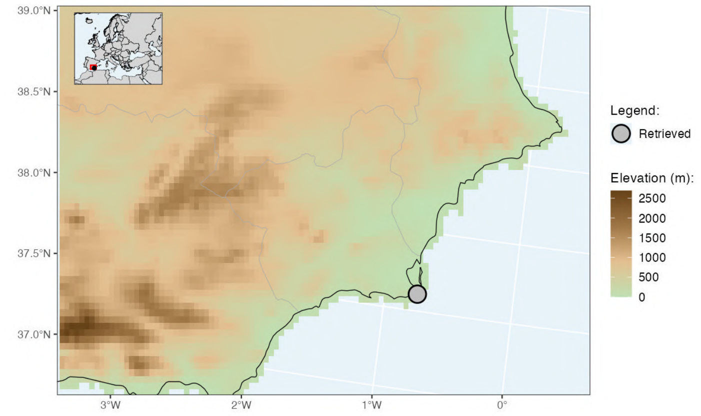

Figure 684: Map of *Temnothorax cristinae* (Espadaler, 1997). Due to the presence of a single sequence, the genetic diversity map and the PCoA projection were not done. Specimen identification (ID or cf.) and source (newly sequenced or retrieved) are represented by colours, while specimen attribute (terra typica, type locality, type specimen or faunistic novelty) is represented by the shape. Sequences: ID = 1, cf. = 0; maximum p-distance: strict = NA, less strict = NA.

Haplotype network analysis of *Temnothorax cristinae* was not possible.

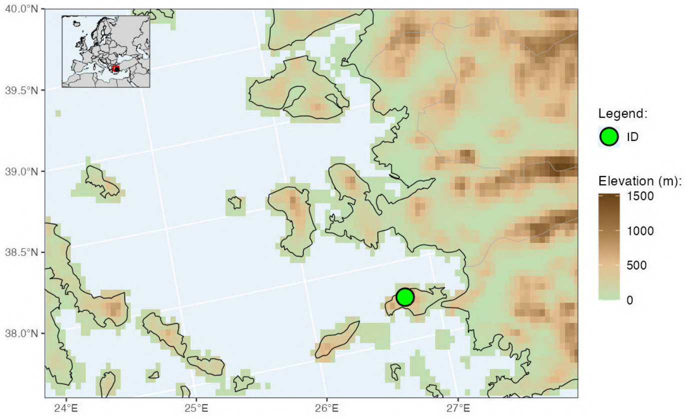

Figure 685: Map of *Temnothorax curtisetosus* Salata & Borowiec, 2015. Due to the presence of a single sequence, the genetic diversity map and the PCoA projection were not done. Specimen identification (ID or cf.) and source (newly sequenced or retrieved) are represented by colours, while specimen attribute (terra typica, type locality, type specimen or faunistic novelty) is represented by the shape. Sequences: ID = 1, cf. = 0; maximum p-distance: strict = NA, less strict = NA.

Haplotype network analysis of *Temnothorax curtisetosus* was not possible.

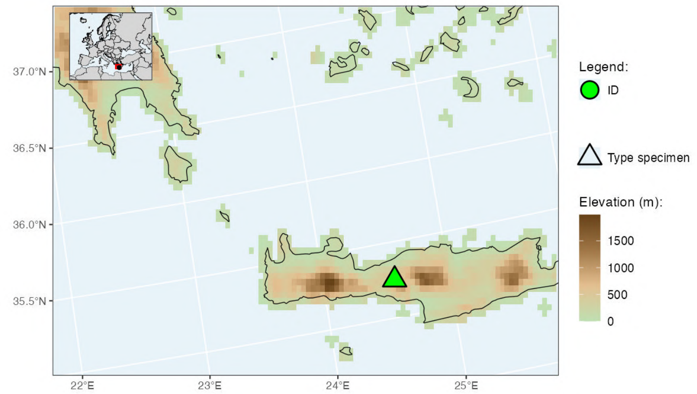

Figure 686: Map of *Temnothorax daidalosi* Salata, Borowiec & Trichas, 2018. Due to the presence of a single sequence, the genetic diversity map and the PCoA projection were not done. Specimen identification (ID or cf.) and source (newly sequenced or retrieved) are represented by colours, while specimen attribute (terra typica, type locality, type specimen or faunistic novelty) is represented by the shape. Sequences: ID = 1, cf. = 0; maximum p-distance: strict = NA, less strict = NA.

Haplotype network analysis of *Temnothorax daidalosi* was not possible.

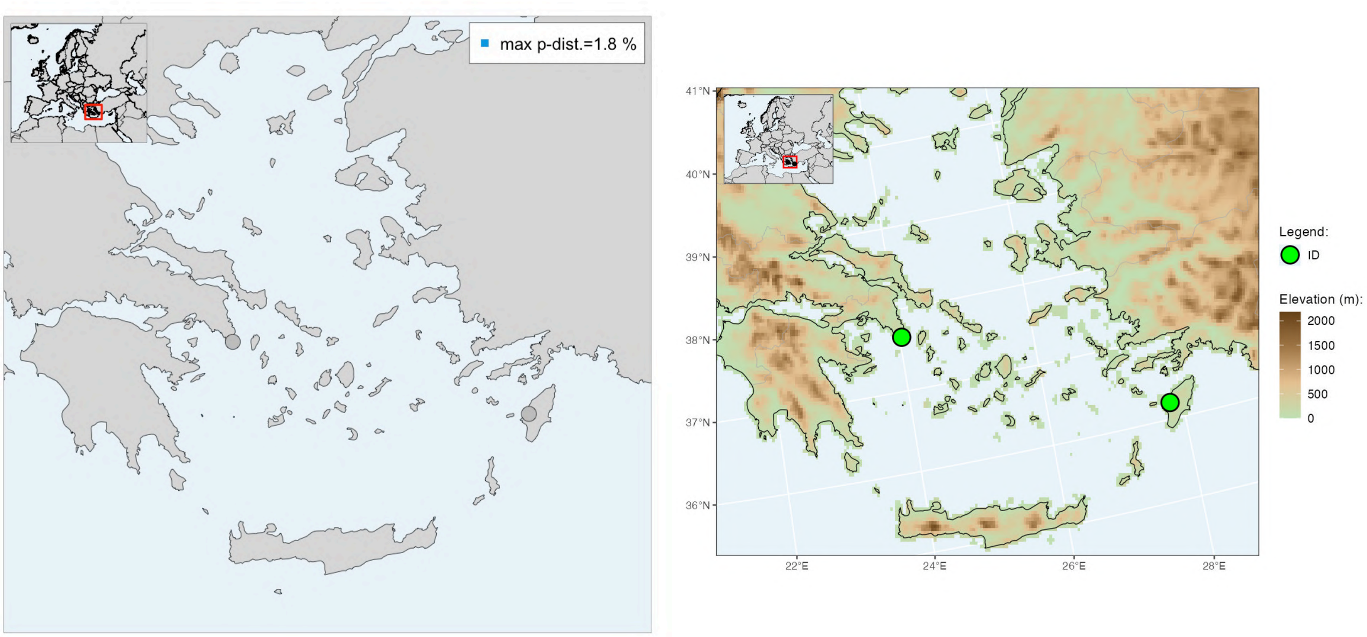

Figure 687: Genetic diversity map of *Temnothorax dessyi* (Menozzi, 1936). PCoA projection was not done and therefore sequenced specimens in the genetic diversity map are coloured in gray (left). Specimen identification (ID or cf.) and source (newly sequenced or retrieved) are represented by colours, while specimen attribute (terra typica, type locality, type specimen or faunistic novelty) is represented by the shape (right). Sequences: ID = 2, cf. = 0; maximum p-distance: strict = NA, less strict = 1.8 %.

Haplotype network analysis of *Temnothorax dessyi* was not possible.

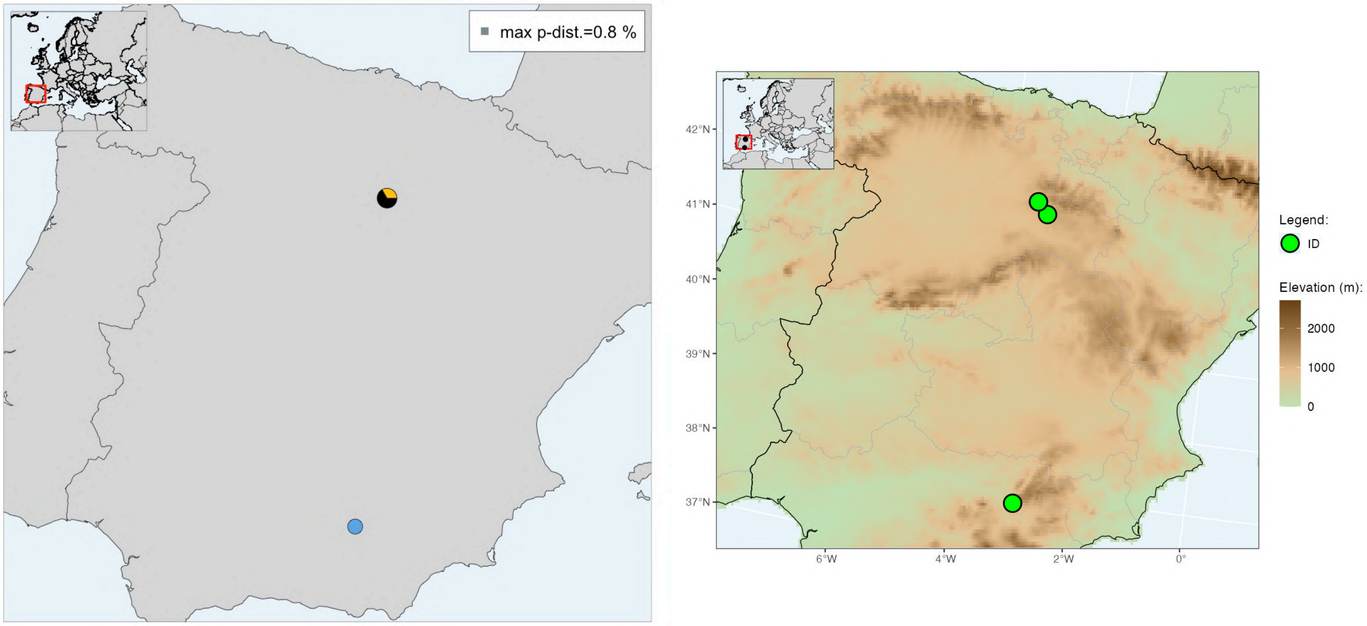

Figure 688: Genetic diversity map of *Temnothorax estel* Arcos González 2021. Nearby localities of sequenced specimens are merged in pies (left). Colours match the bidimensional colour space of the PCoA projection (Fig. 688 left) of p-dist between sequences (dots). Specimen identification (ID or cf.) and source (newly sequenced or retrieved) are represented by colours, while specimen attribute (terra typica, type locality, type specimen or faunistic novelty) is represented by the shape (right). Sequences: ID = 4, cf. = 0; maximum p-distance: strict = 0.8 %, less strict = 0.8 %.

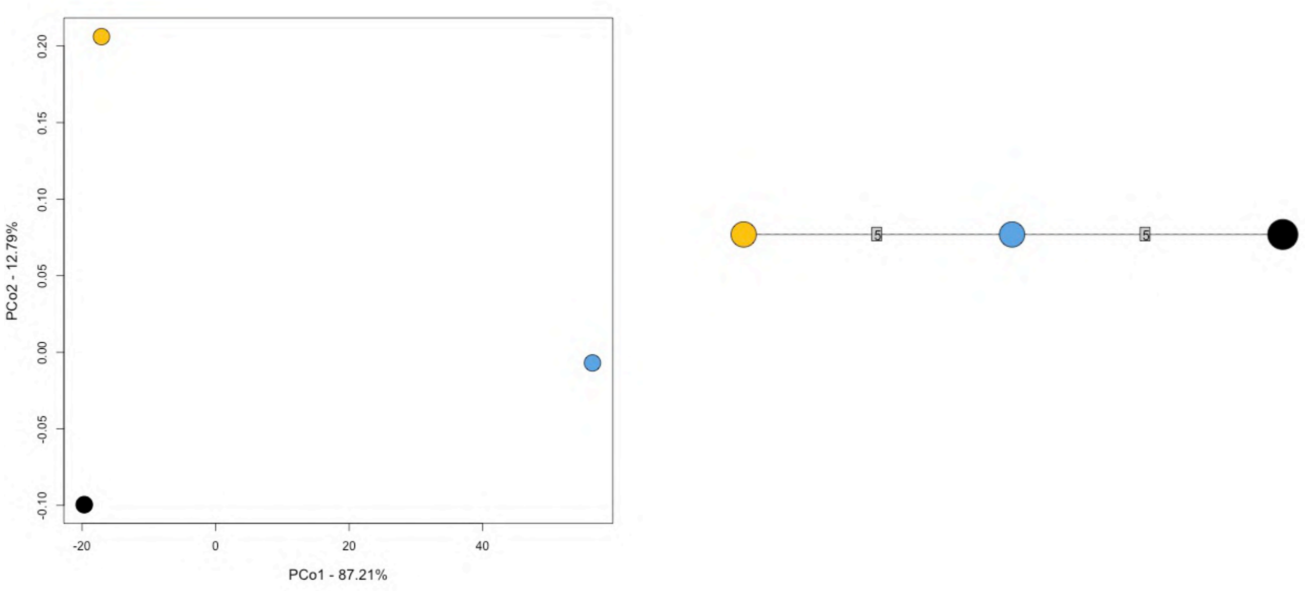

Figure 689: PCoA based on pairwise p-distances between *Temnothorax estel* sequences (left). Colours match a bidimensional colour space. Haplotype network of *Temnothorax estel* (right). Sequences > 599 bp: ID = 4, cf. = 0.

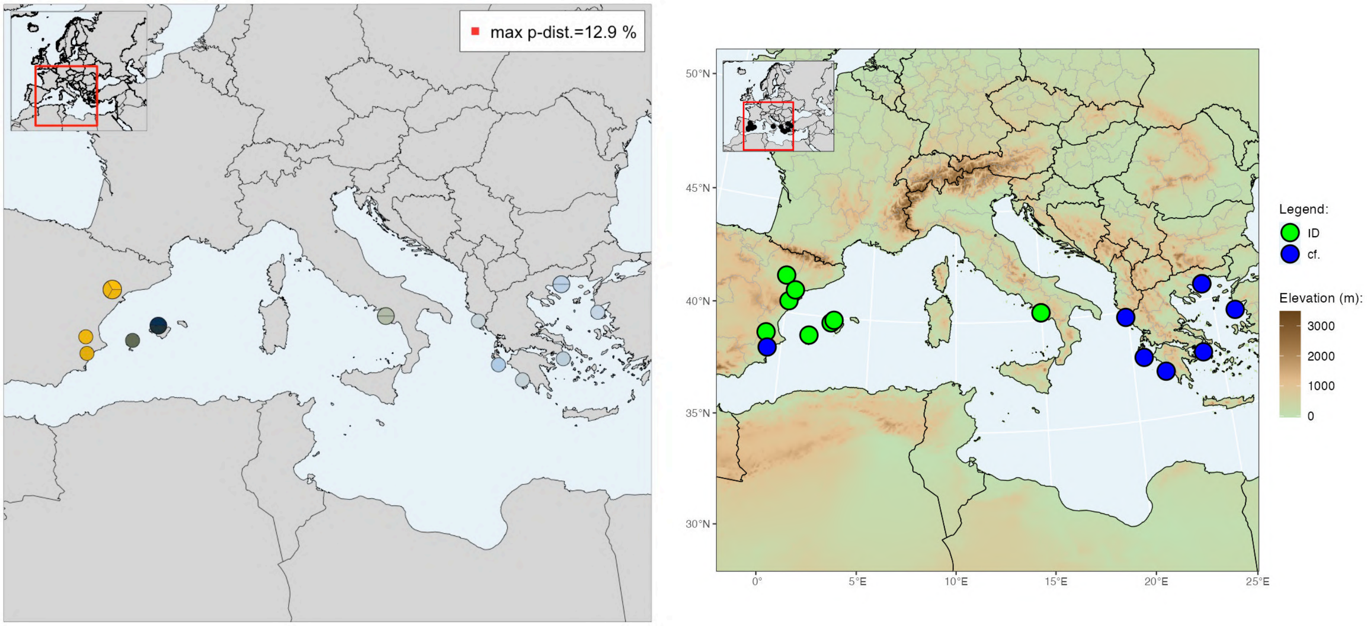

Figure 690: Genetic diversity map of *Temnothorax exilis* (Emery, 1869). Nearby localities of sequenced specimens are merged in pies (left). Colours match the bidimensional colour space of the PCoA projection (Fig. 690 left) of p-dist between sequences (dots). Specimen identification (ID or cf.) and source (newly sequenced or retrieved) are represented by colours, while specimen attribute (terra typica, type locality, type specimen or faunistic novelty) is represented by the shape (right). Sequences: ID = 9, cf. = 8; maximum p-distance: strict = 12 %, less strict = 12.9 %.

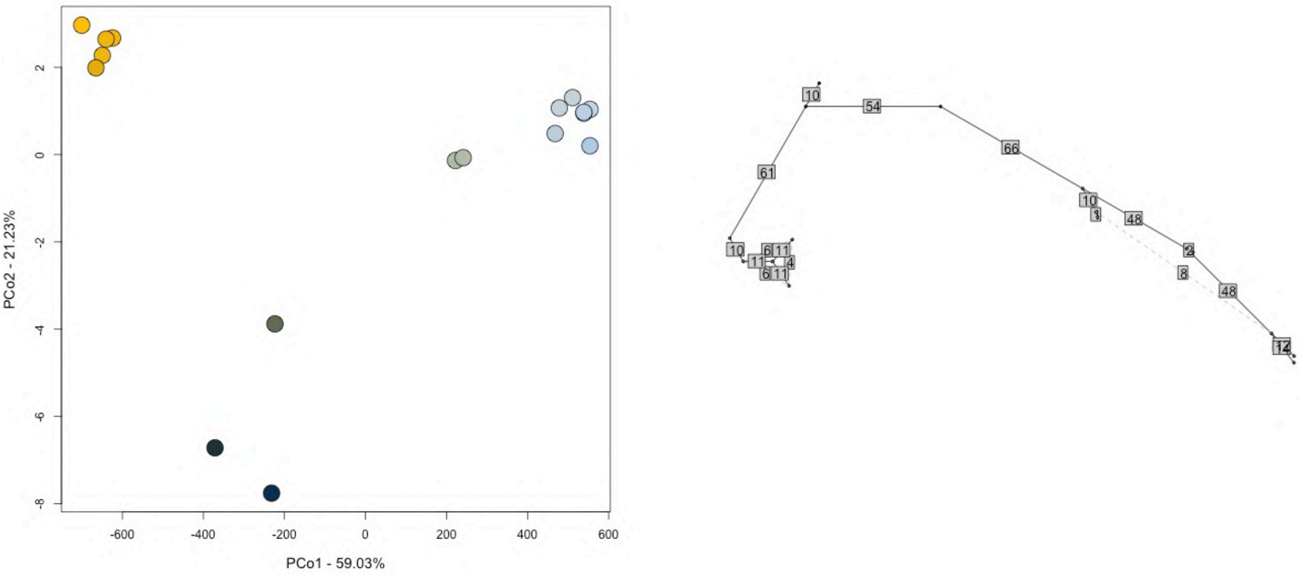

Figure 691: PCoA based on pairwise p-distances between *Temnothorax exilis* sequences (left). Colours match a bidimensional colour space. Haplotype network of *Temnothorax exilis* (right). Sequences > 599 bp: ID = 9, cf. = 8.

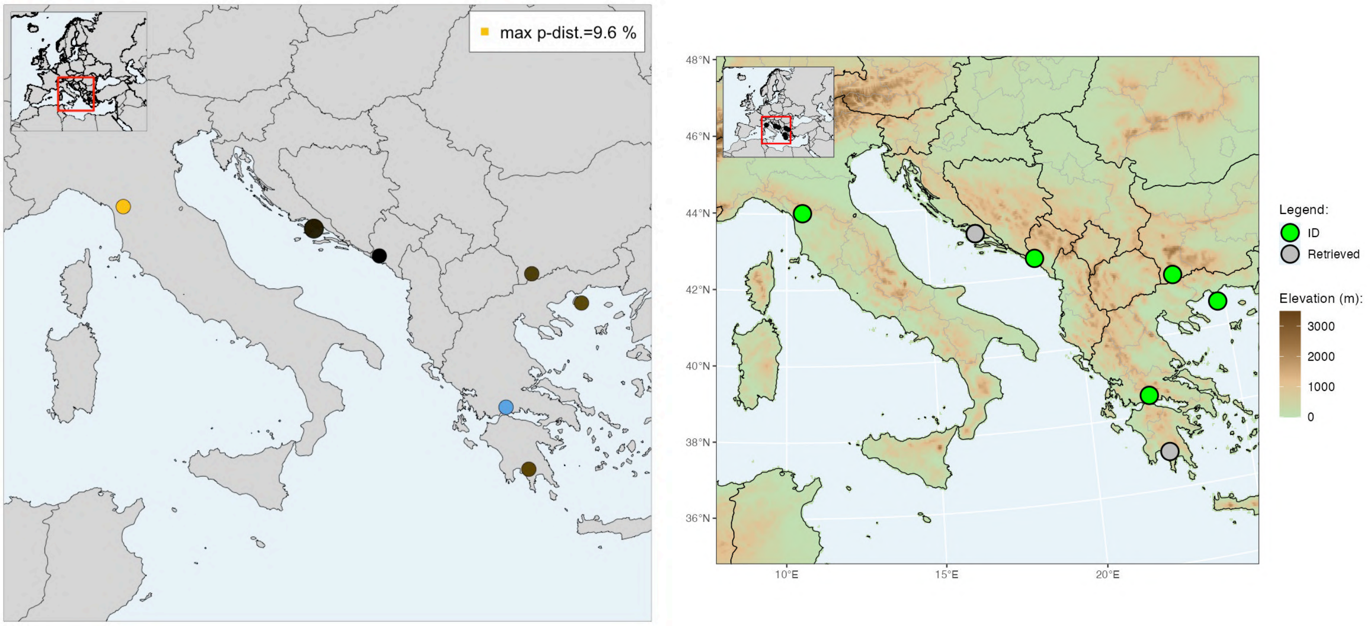

Figure 692: Genetic diversity map of *Temnothorax flavicornis* (Emery, 1870). Nearby localities of sequenced specimens are merged in pies (left). Colours match the bidimensional colour space of the PCoA projection (Fig. 692 left) of p-dist between sequences (dots). Specimen identification (ID or cf.) and source (newly sequenced or retrieved) are represented by colours, while specimen attribute (terra typica, type locality, type specimen or faunistic novelty) is represented by the shape (right). Sequences: ID = 9, cf. = 0; maximum p-distance: strict = 9.6 %, less strict = 9.6 %.

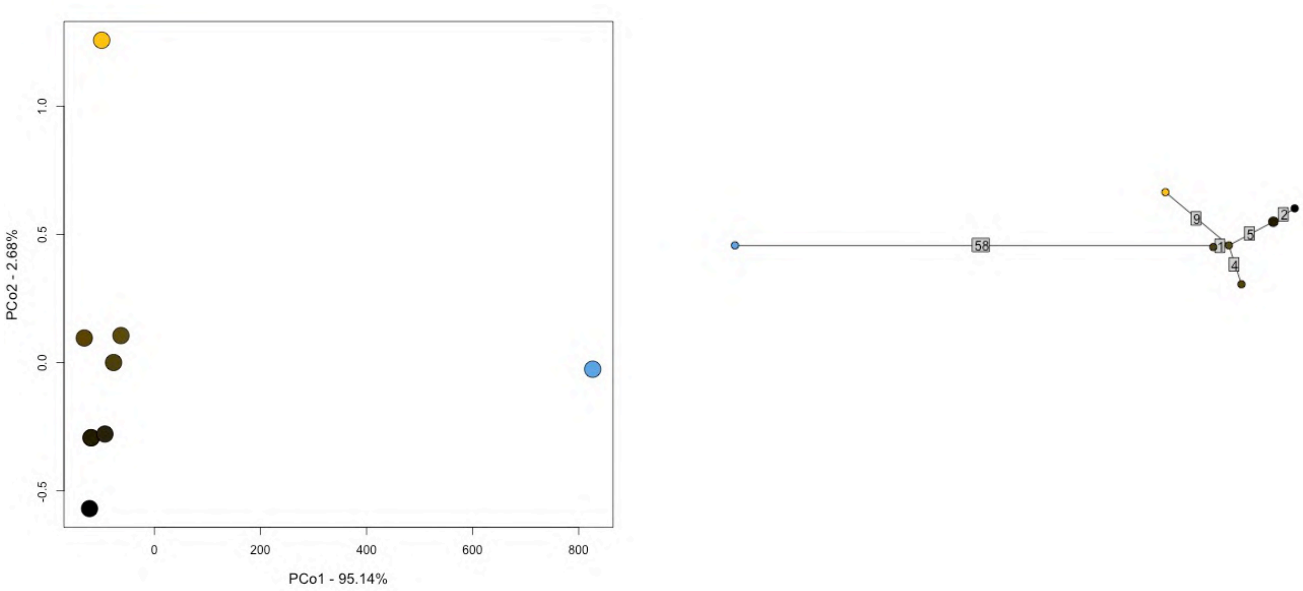

Figure 693: PCoA based on pairwise p-distances between *Temnothorax flavicornis* sequences (left). Colours match a bidimensional colour space. Haplotype network of *Temnothorax flavicornis* (right). Sequences > 599 bp: ID = 9, cf. = 0.

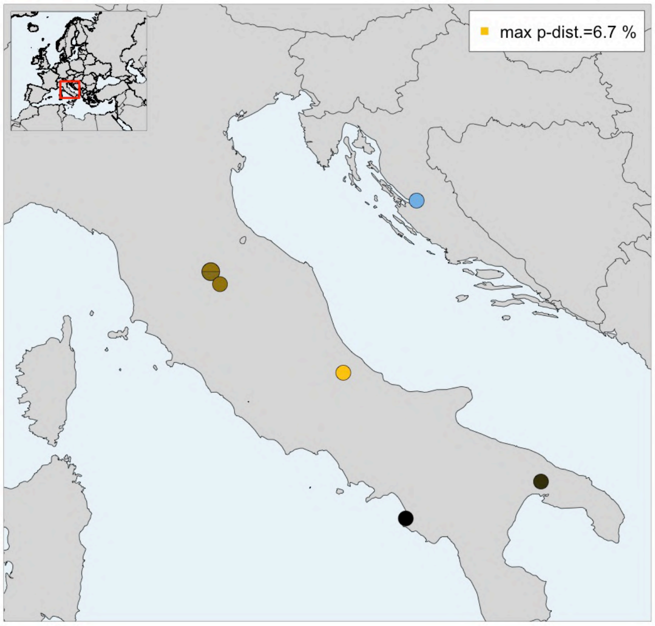

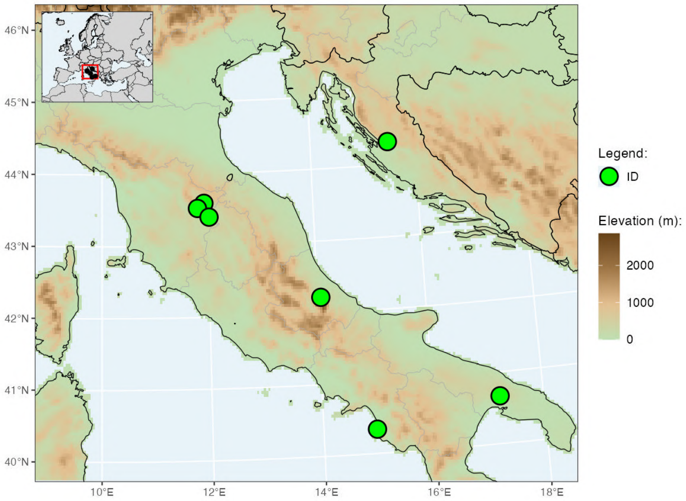

Figure 694: Genetic diversity map of *Temnothorax gallei* Csősz, Taheri, Schifani, Reyes-López, Alicata, Báthori & Prebus, 2025. Nearby localities of sequenced specimens are merged in pies (left). Colours match the bidimensional colour space of the PCoA projection (Fig. 694 left) of p-dist between sequences (dots). Specimen identification (ID or cf.) and source (newly sequenced or retrieved) are represented by colours, while specimen attribute (terra typica, type locality, type specimen or faunistic novelty) is represented by the shape (right). Sequences: ID = 7, cf. = 0; maximum p-distance: strict = 6.7 %, less strict = 6.7 %.

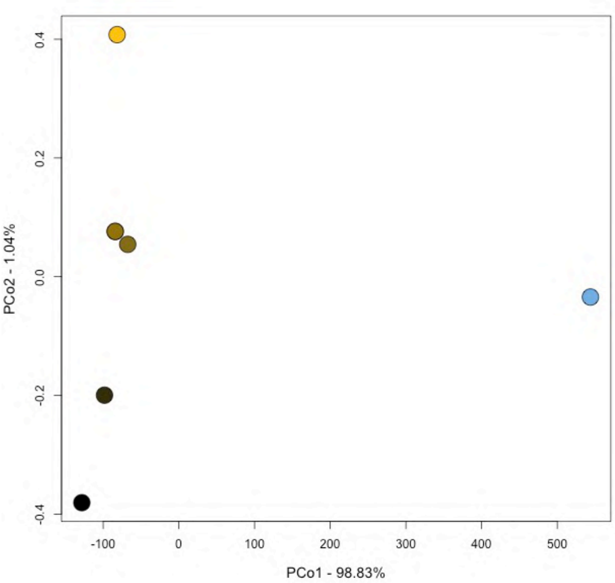

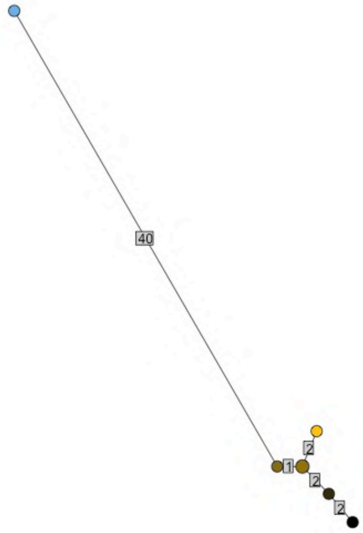

Figure 695: PCoA based on pairwise p-distances between *Temnothorax gallei* sequences (left). Colours match a bidimensional colour space. Haplotype network of *Temnothorax gallei* (right). Sequences > 599 bp: ID = 7, cf. = 0.

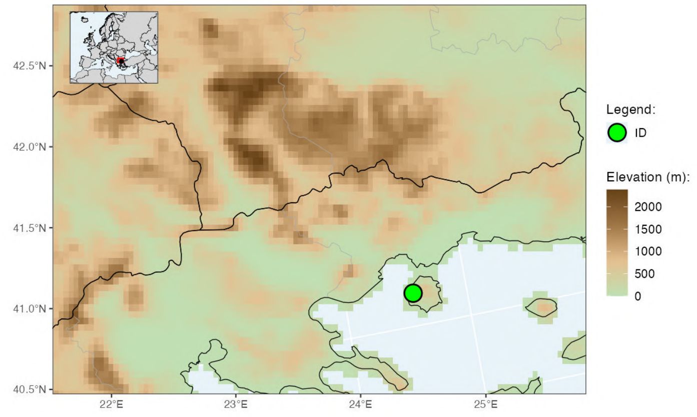

Figure 696: Map of *Temnothorax gordiagini* (Ruzsky, 1902). Due to the presence of a single sequence, the genetic diversity map and the PCoA projection were not done. Specimen identification (ID or cf.) and source (newly sequenced or retrieved) are represented by colours, while specimen attribute (terra typica, type locality, type specimen or faunistic novelty) is represented by the shape. Sequences: ID = 1, cf. = 0; maximum p-distance: strict = NA, less strict = NA.

Haplotype network analysis of *Temnothorax gordiagini* was not possible.

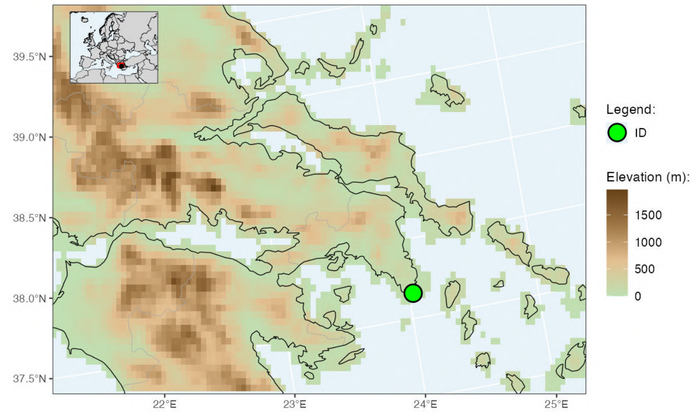

Figure 697: Map of *Temnothorax graecus* (Forel, 1911). Due to the presence of a single sequence, the genetic diversity map and the PCoA projection were not done. Specimen identification (ID or cf.) and source (newly sequenced or retrieved) are represented by colours, while specimen attribute (terra typica, type locality, type specimen or faunistic novelty) is represented by the shape. Sequences: ID = 1, cf. = 0; maximum p-distance: strict = NA, less strict = NA.

Haplotype network analysis of *Temnothorax graecus* was not possible.

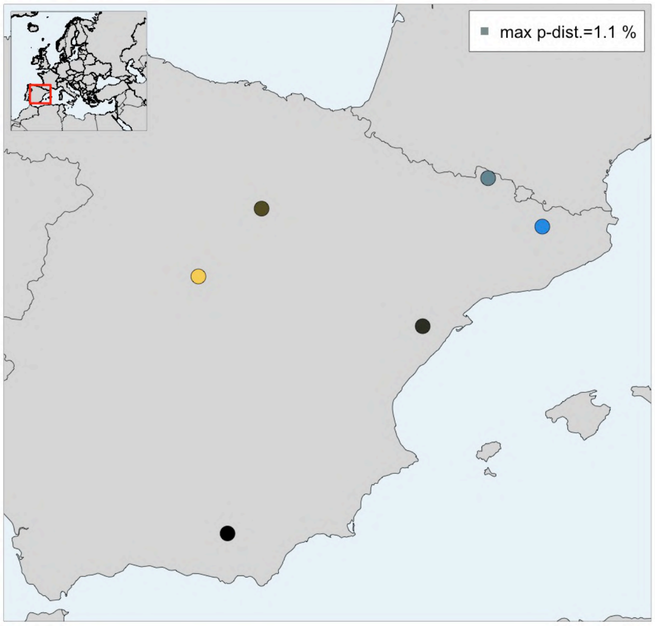

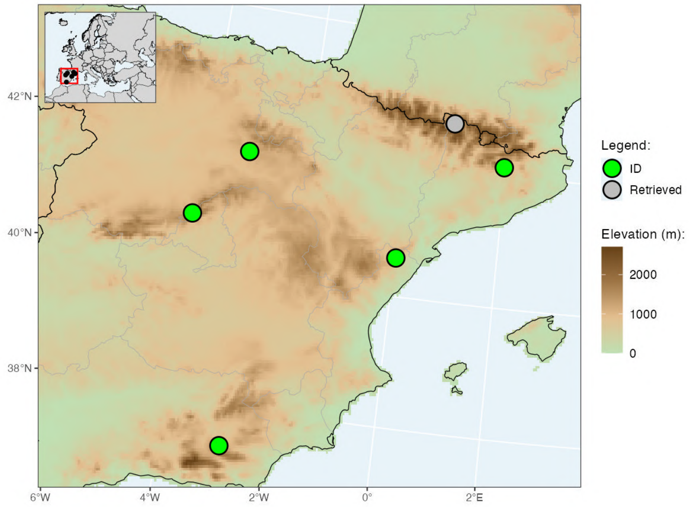

Figure 698: Genetic diversity map of *Temnothorax gredosi* (Espadaler & Collingwood, 1982). Nearby localities of sequenced specimens are merged in pies (left). Colours match the bidimensional colour space of the PCoA projection (Fig. 698 left) of p-dist between sequences (dots). Specimen identification (ID or cf.) and source (newly sequenced or retrieved) are represented by colours, while specimen attribute (terra typica, type locality, type specimen or faunistic novelty) is represented by the shape (right). Sequences: ID = 6, cf. = 0; maximum p-distance: strict = 1.1 %, less strict = 1.1 %.

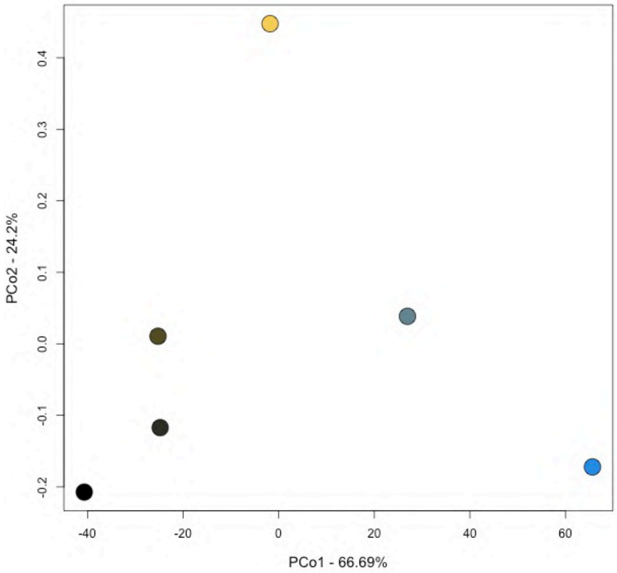

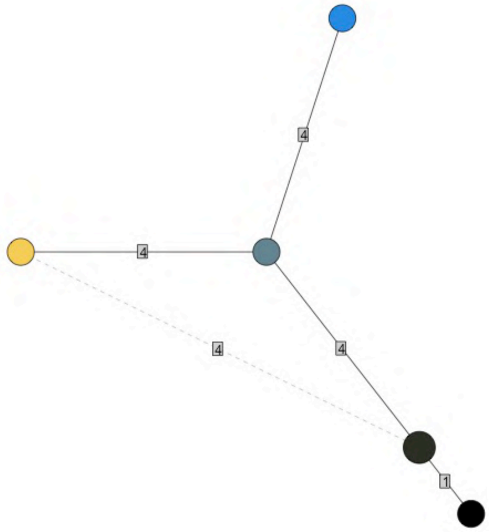

Figure 699: PCoA based on pairwise p-distances between *Temnothorax gredosi* sequences (left). Colours match a bidimensional colour space. Haplotype network of *Temnothorax gredosi* (right). Sequences > 599 bp: ID = 6, cf. = 0.

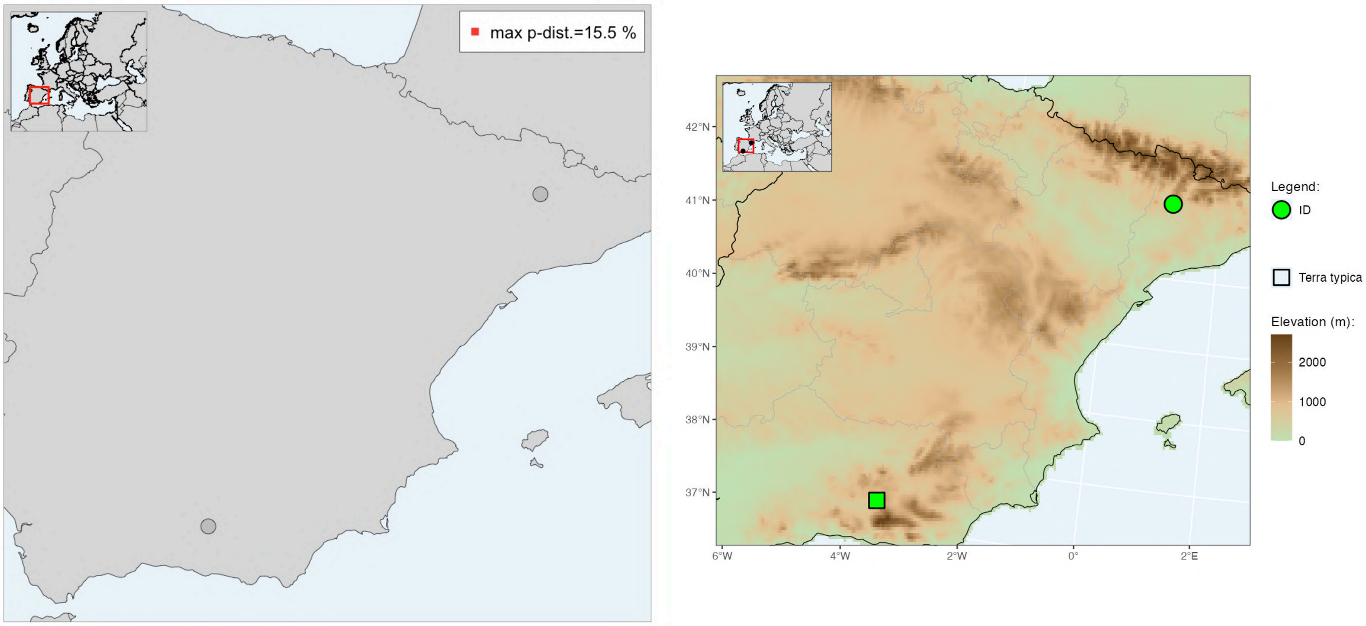

Figure 700: Genetic diversity map of *Temnothorax grouvellei* (Bondroit, 1918). PCoA projection was not done and therefore sequenced specimens in the genetic diversity map are coloured in gray (left). Specimen identification (ID or cf.) and source (newly sequenced or retrieved) are represented by colours, while specimen attribute (terra typica, type locality, type specimen or faunistic novelty) is represented by the shape (right). Sequences: ID = 2, cf. = 0; maximum p-distance: strict = NA, less strict = 15.5 %.

Haplotype network analysis of *Temnothorax grouvellei* was not possible.

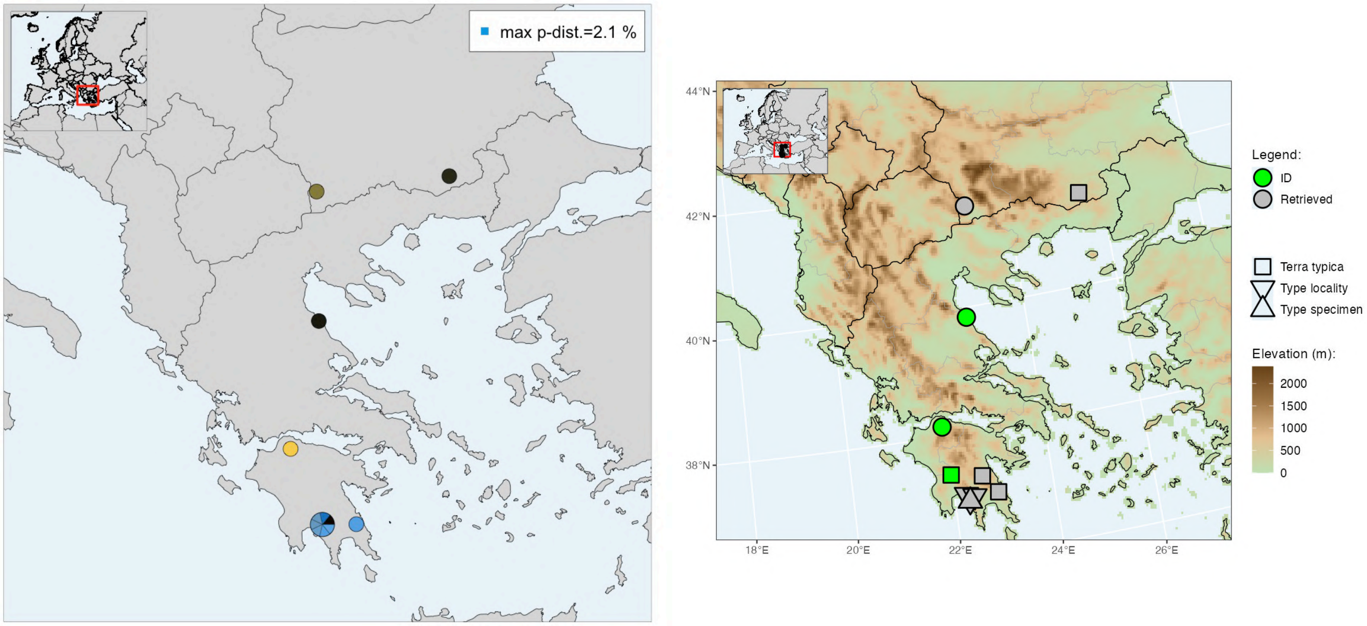

Figure 701: Genetic diversity map of *Temnothorax helenae* Csösz, Heinze & Mikó, 2015. Nearby localities of sequenced specimens are merged in pies (left). Colours match the bidimensional colour space of the PCoA projection (Fig. 701 left) of p-dist between sequences (dots). Specimen identification (ID or cf.) and source (newly sequenced or retrieved) are represented by colours, while specimen attribute (terra typica, type locality, type specimen or faunistic novelty) is represented by the shape (right). Sequences: ID = 12, cf. = 0; maximum p-distance: strict = 2.1 %, less strict = 2.1 %.

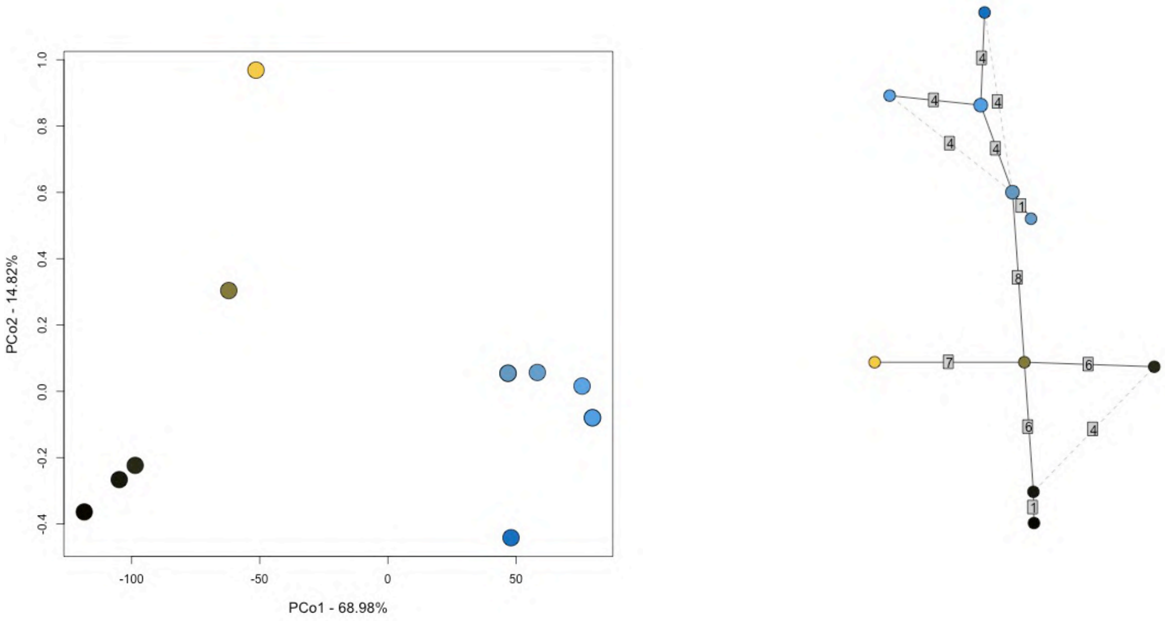

Figure 702: PCoA based on pairwise p-distances between *Temnothorax helenae* sequences (left). Colours match a bidimensional colour space. Haplotype network of *Temnothorax helenae* (right). Sequences > 599 bp: ID = 12, cf. = 0.

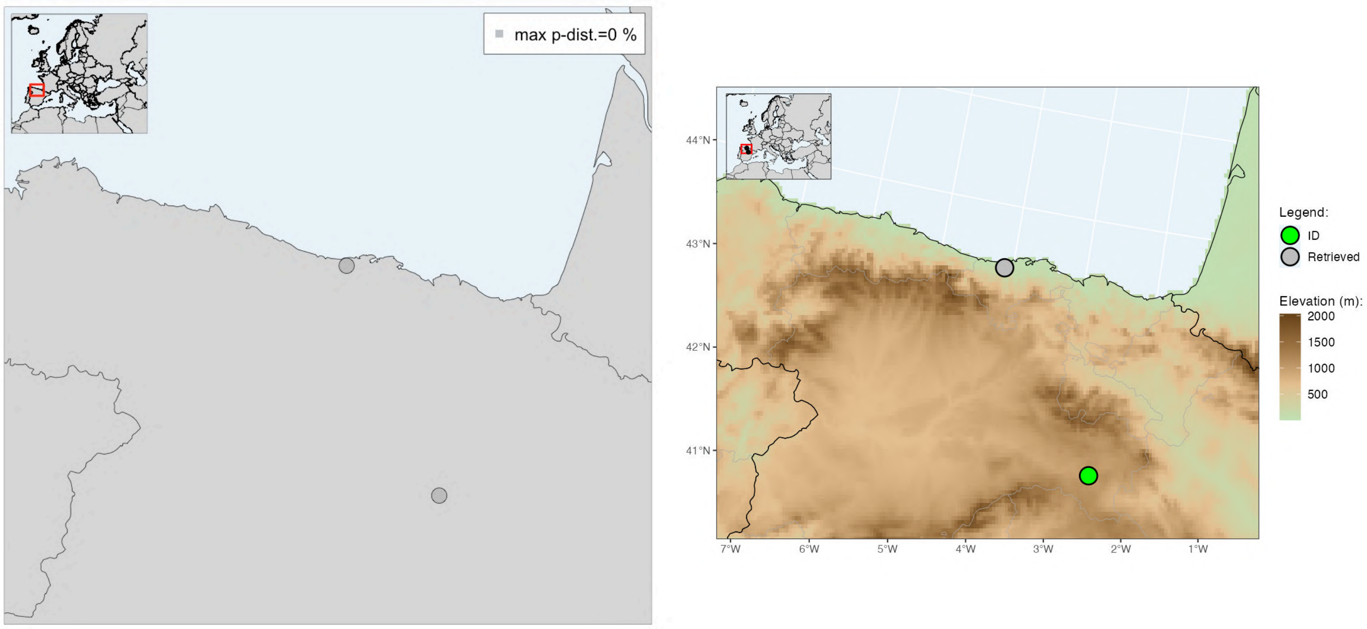

Figure 703: Genetic diversity map of *Temnothorax ibericus* (Menozzi, 1922). PCoA projection was not done and therefore sequenced specimens in the genetic diversity map are coloured in gray (left). Specimen identification (ID or cf.) and source (newly sequenced or retrieved) are represented by colours, while specimen attribute (terra typica, type locality, type specimen or faunistic novelty) is represented by the shape (right). Sequences: ID = 2, cf. = 0; maximum p-distance: strict = NA, less strict = 0 %.

Haplotype network analysis of *Temnothorax ibericus* was not possible.

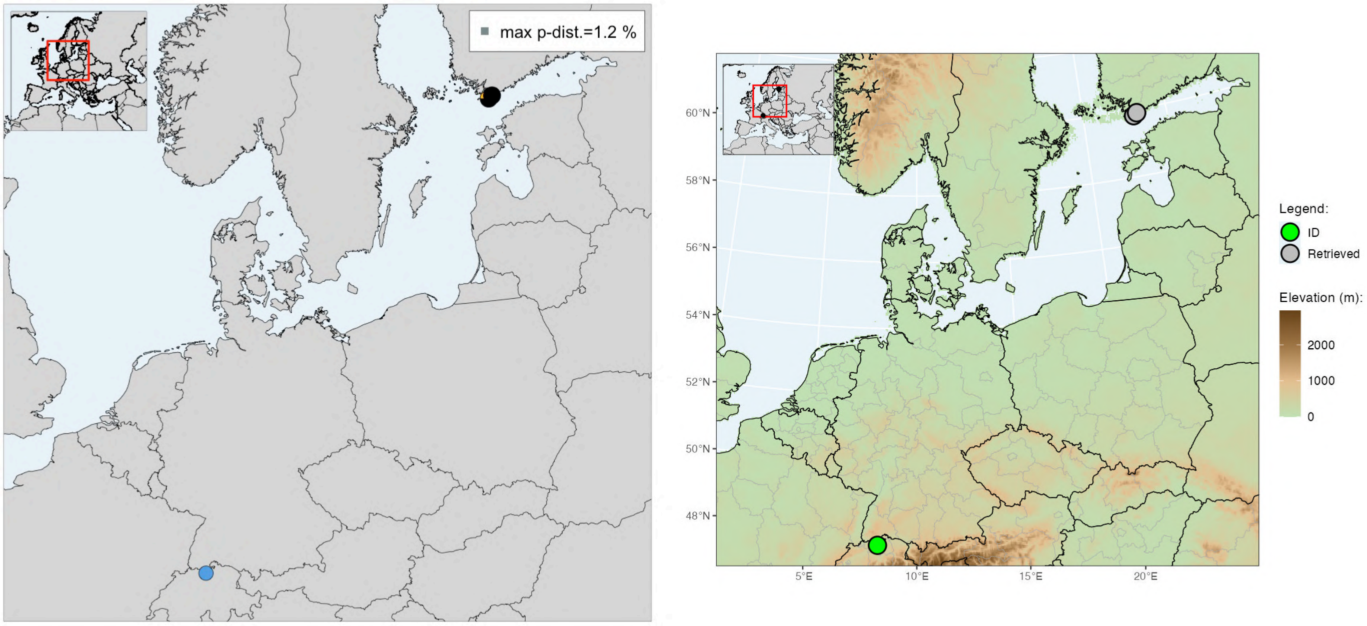

Figure 704: Genetic diversity map of *Temnothorax interruptus* (Schenck, 1852). Nearby localities of sequenced specimens are merged in pies (left). Colours match the bidimensional colour space of the PCoA projection (Fig. 704 left) of p-dist between sequences (dots). Specimen identification (ID or cf.) and source (newly sequenced or retrieved) are represented by colours, while specimen attribute (terra typica, type locality, type specimen or faunistic novelty) is represented by the shape (right). Sequences: ID = 5, cf. = 0; maximum p-distance: strict = 1.2 %, less strict = 1.2 %.

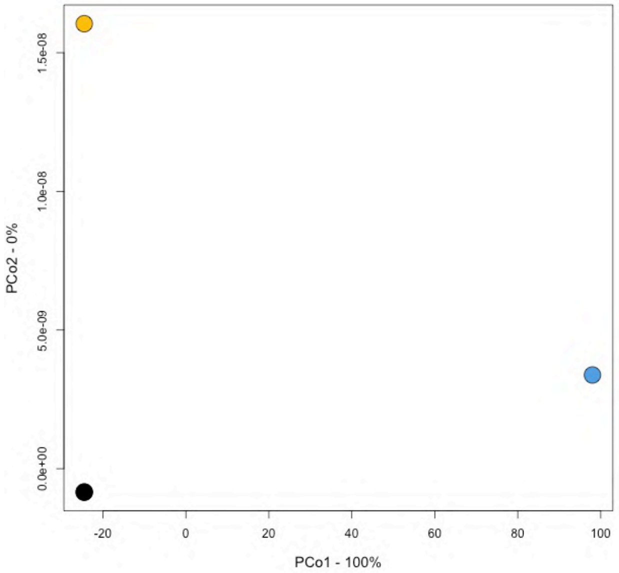

Figure 705: PCoA based on pairwise p-distances between *Temnothorax interruptus* sequences (left). Colours match a bidimensional colour space. Haplotype network analysis of *Temnothorax interruptus* was not possible.

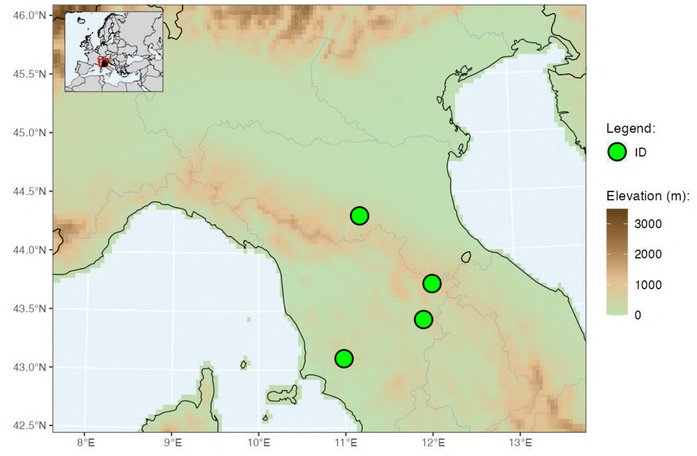

Figure 706: Map of *Temnothorax italicus* (Consani, 1952). Due to the presence of a single sequence, the genetic diversity map and the PCoA projection were not done. Specimen identification (ID or cf.) and source (newly sequenced or retrieved) are represented by colours, while specimen attribute (terra typica, type locality, type specimen or faunistic novelty) is represented by the shape. Sequences: ID = 4, cf. = 0; maximum p-distance: strict = 0.6 %, less strict = 0.6 %.

Haplotype network analysis of *Temnothorax italicus* was not possible.

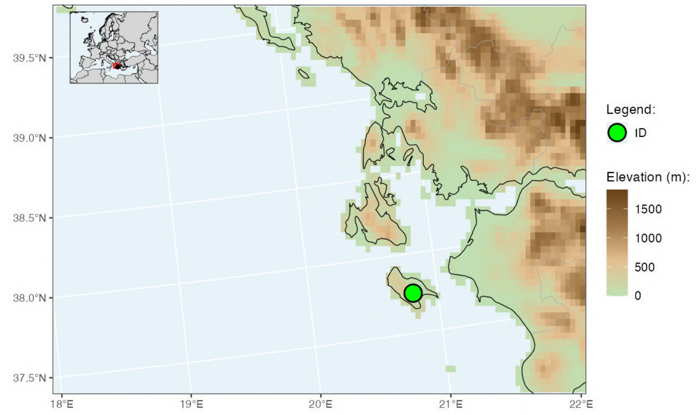

Figure 707: Map of *Temnothorax kemali* (Santschi, 1934). Due to the presence of a single sequence, the genetic diversity map and the PCoA projection were not done. Specimen identification (ID or cf.) and source (newly sequenced or retrieved) are represented by colours, while specimen attribute (terra typica, type locality, type specimen or faunistic novelty) is represented by the shape. Sequences: ID = 1, cf. = 0; maximum p-distance: strict = NA, less strict = NA.

Haplotype network analysis of *Temnothorax kemali* was not possible.

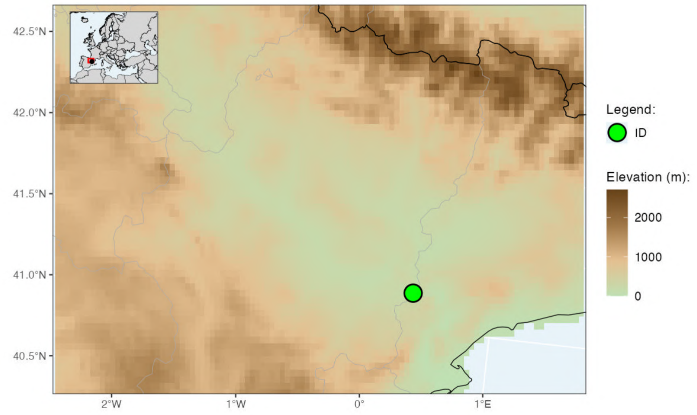

Figure 708: Map of *Temnothorax kraussei* (Emery, 1915). Due to the presence of a single sequence, the genetic diversity map and the PCoA projection were not done. Specimen identification (ID or cf.) and source (newly sequenced or retrieved) are represented by colours, while specimen attribute (terra typica, type locality, type specimen or faunistic novelty) is represented by the shape. Sequences: ID = 1, cf. = 0; maximum p-distance: strict = NA, less strict = NA.

Haplotype network analysis of *Temnothorax kraussei* was not possible.

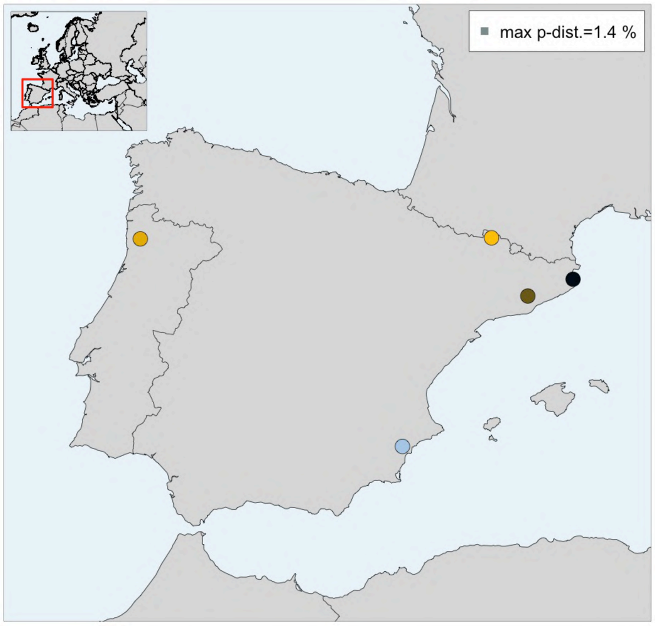

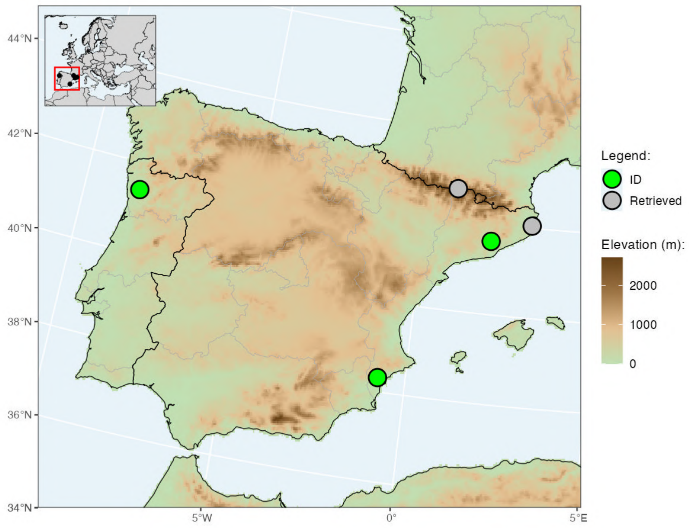

Figure 709: Genetic diversity map of *Temnothorax kutteri* (Cagniant, 1973). Nearby localities of sequenced specimens are merged in pies (left). Colours match the bidimensional colour space of the PCoA projection (Fig. 709 left) of p-dist between sequences (dots). Specimen identification (ID or cf.) and source (newly sequenced or retrieved) are represented by colours, while specimen attribute (terra typica, type locality, type specimen or faunistic novelty) is represented by the shape (right). Sequences: ID = 5, cf. = 0; maximum p-distance: strict = 1.4 %, less strict = 1.4 %.

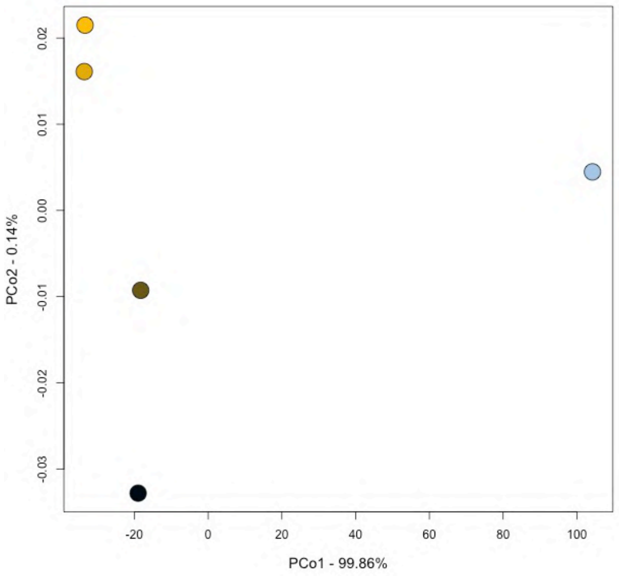

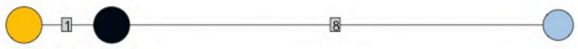

Figure 710: PCoA based on pairwise p-distances between *Temnothorax kutteri* sequences (left). Colours match a bidimensional colour space. Haplotype network of *Temnothorax kutteri* (right). Sequences > 599 bp: ID = 5, cf. = 0.

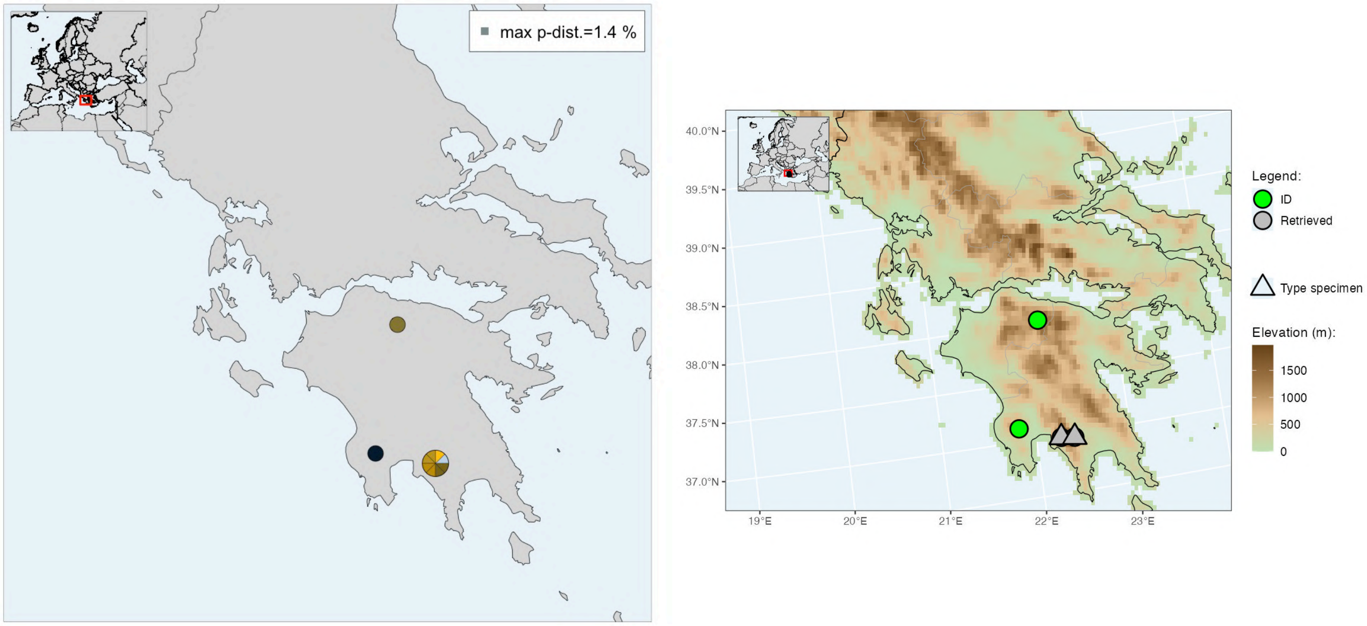

Figure 711: Genetic diversity map of *Temnothorax laconicus* Csősz, Seifert, Müller, Trindl, Schulz. & Heinze, 2013. Nearby localities of sequenced specimens are merged in pies (left). Colours match the bidimensional colour space of the PCoA projection (Fig. 711 left) of p-dist between sequences (dots). Specimen identification (ID or cf.) and source (newly sequenced or retrieved) are represented by colours, while specimen attribute (terra typica, type locality, type specimen or faunistic novelty) is represented by the shape (right). Sequences: ID = 10, cf. = 0; maximum p-distance: strict = 1.4 %, less strict = 1.4 %.

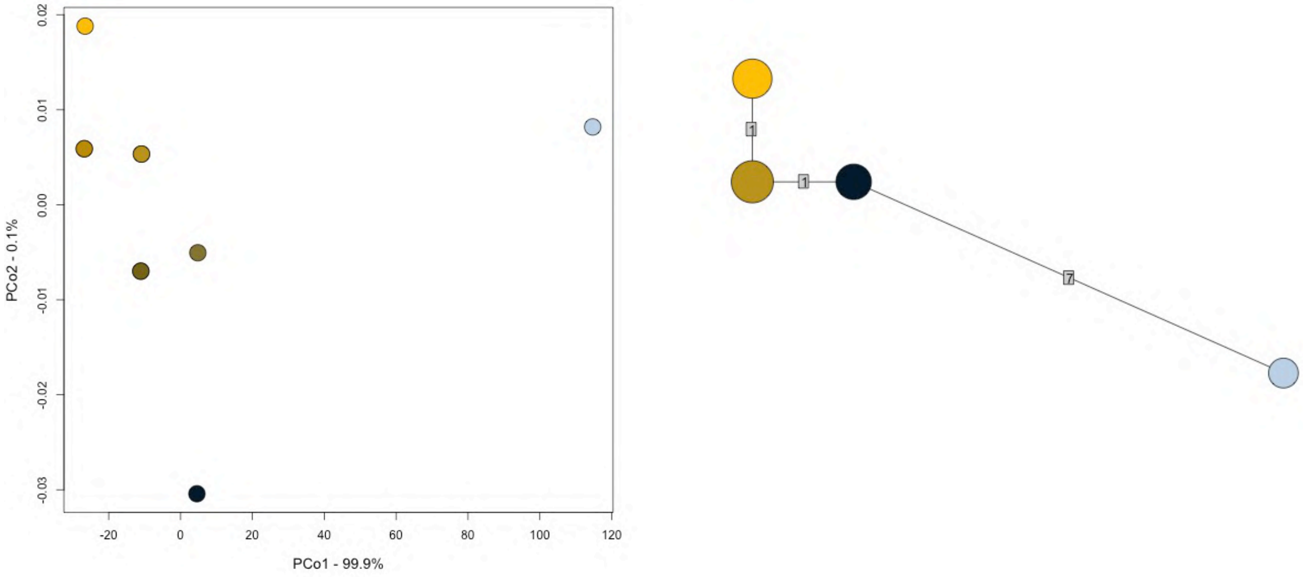

Figure 712: PCoA based on pairwise p-distances between *Temnothorax laconicus* sequences (left). Colours match a bidimensional colour space. Haplotype network of *Temnothorax laconicus* (right). Sequences > 599 bp: ID = 10, cf. = 0.

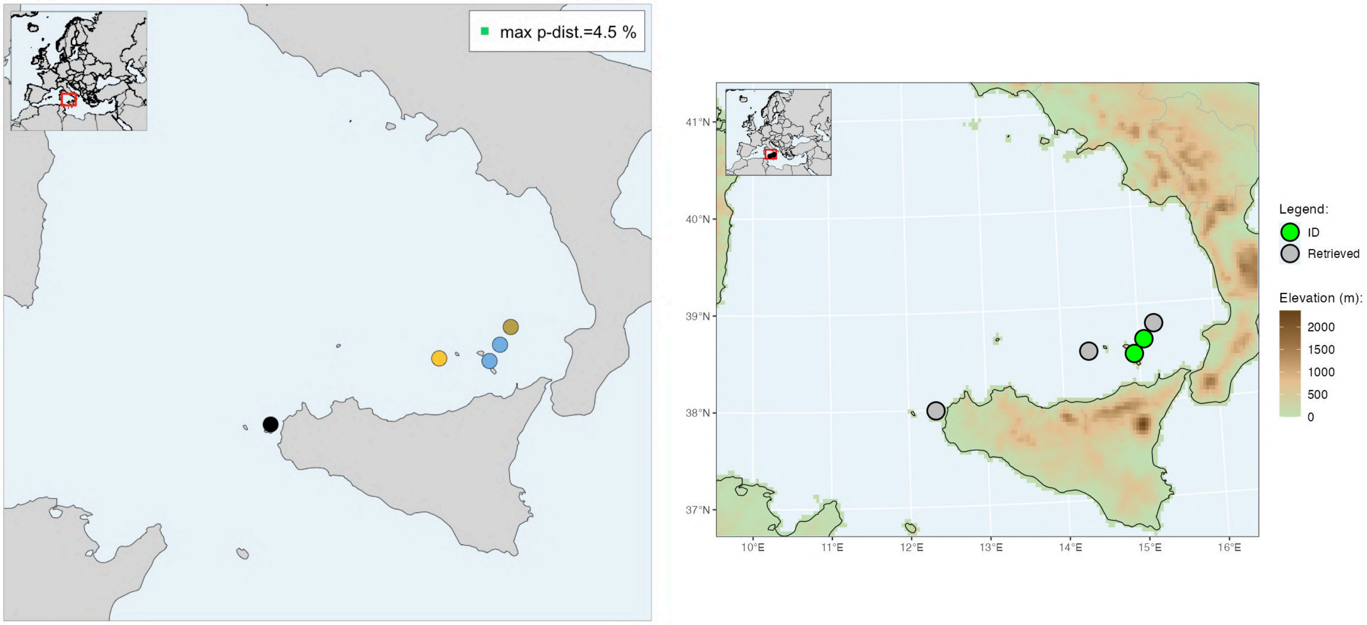

Figure 713: Genetic diversity map of *Temnothorax laestrygon* (Santschi, 1931). Nearby localities of sequenced specimens are merged in pies (left). Colours match the bidimensional colour space of the PCoA projection (Fig. 713 left) of p-dist between sequences (dots). Specimen identification (ID or cf.) and source (newly sequenced or retrieved) are represented by colours, while specimen attribute (terra typica, type locality, type specimen or faunistic novelty) is represented by the shape (right). Sequences: ID = 5, cf. = 0; maximum p-distance: strict = 4.5 %, less strict = 4.5 %.

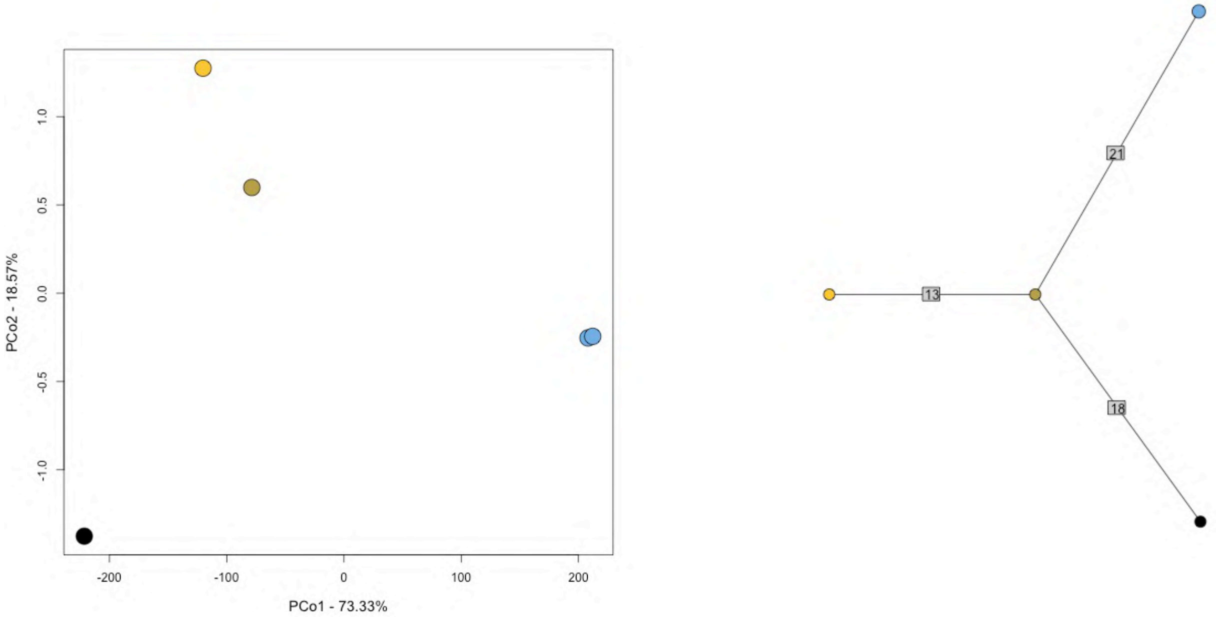

Figure 714: PCoA based on pairwise p-distances between *Temnothorax laestrygon* sequences (left). Colours match a bidimensional colour space. Haplotype network of *Temnothorax laestrygon* (right). Sequences > 599 bp: ID = 5, cf. = 0.

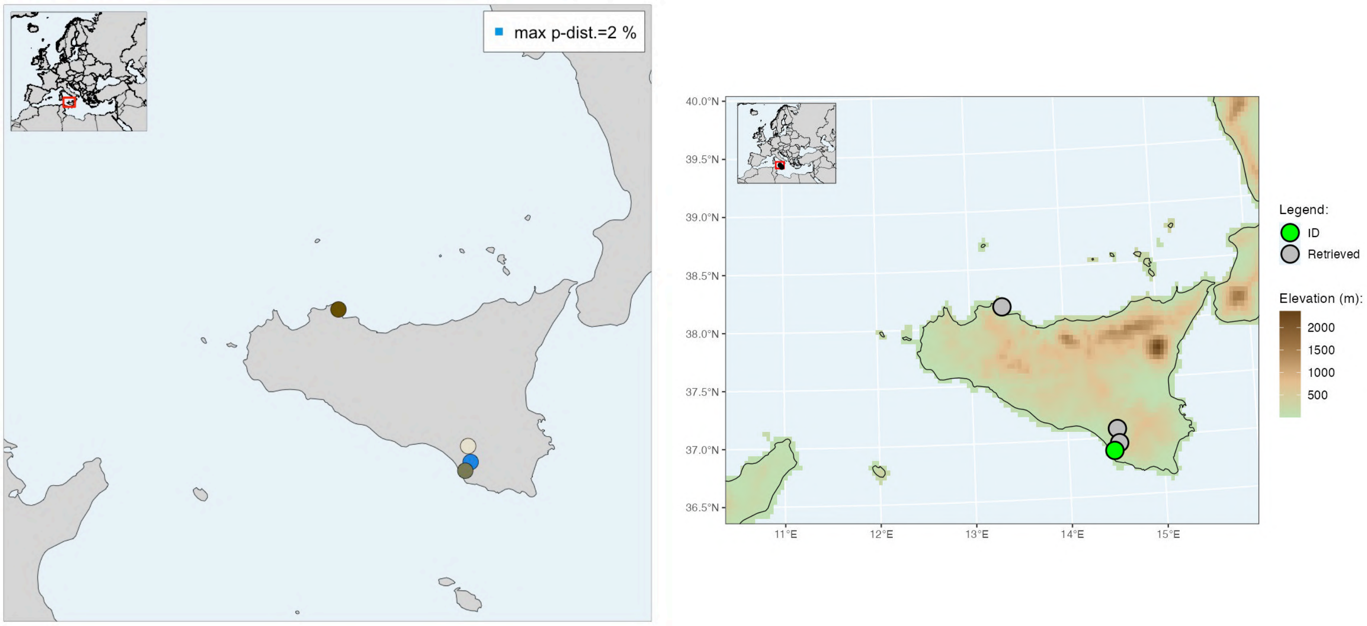

Figure 715: Genetic diversity map of *Temnothorax lagrecai* (Baroni Urbani, 1964). Nearby localities of sequenced specimens are merged in pies (left). Colours match the bidimensional colour space of the PCoA projection (Fig. 715 left) of p-dist between sequences (dots). Specimen identification (ID or cf.) and source (newly sequenced or retrieved) are represented by colours, while specimen attribute (terra typica, type locality, type specimen or faunistic novelty) is represented by the shape (right). Sequences: ID = 4, cf. = 0; maximum p-distance: strict = 2 %, less strict = 2 %.

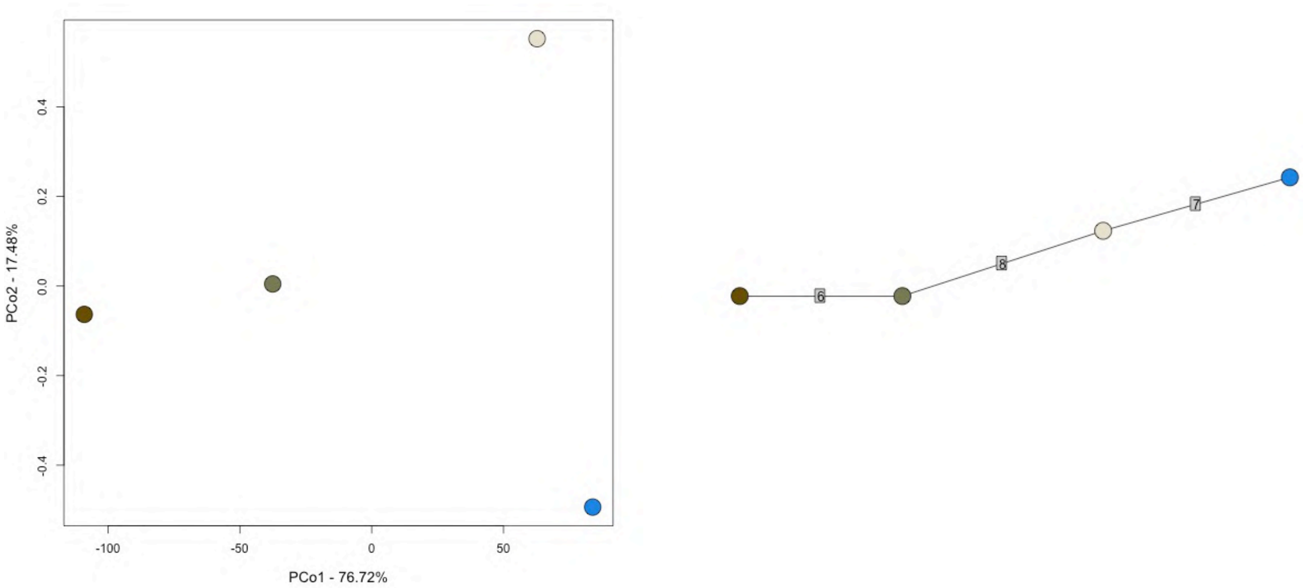

Figure 716: PCoA based on pairwise p-distances between *Temnothorax lagrecai* sequences (left). Colours match a bidimensional colour space. Haplotype network of *Temnothorax lagrecai* (right). Sequences > 599 bp: ID = 4, cf. = 0.

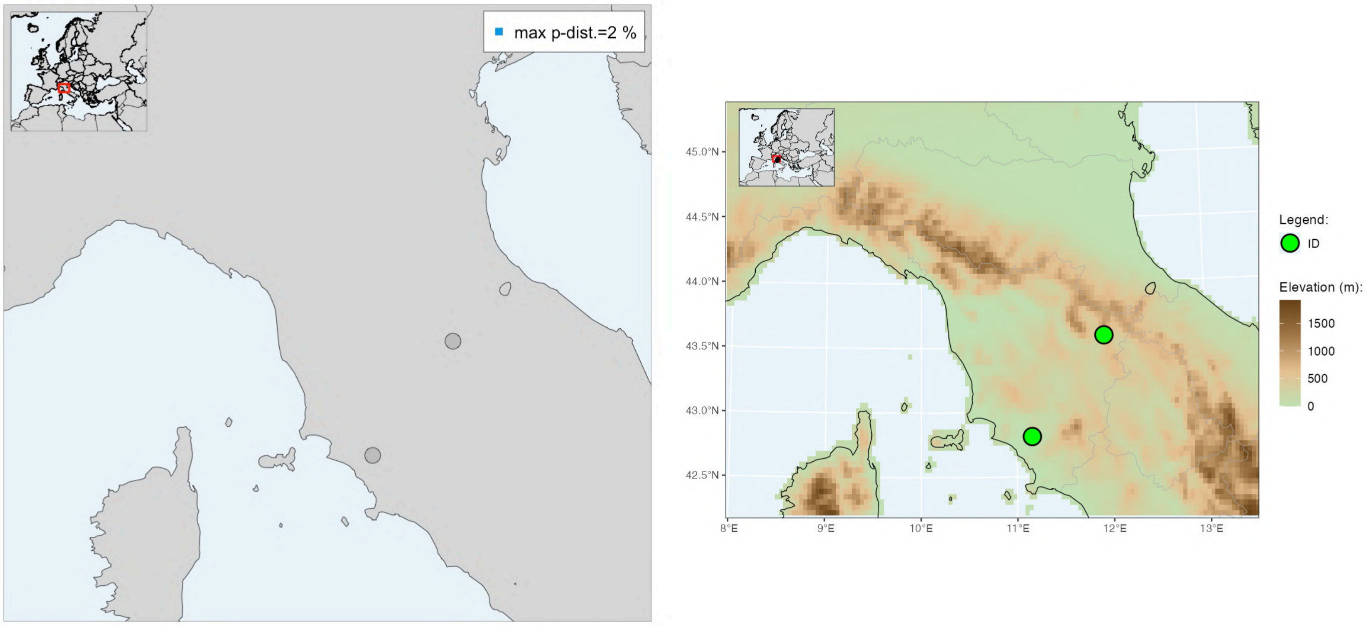

Figure 717: Genetic diversity map of *Temnothorax leviceps* (Emery, 1898). PCoA projection was not done and therefore sequenced specimens in the genetic diversity map are coloured in gray (left). Specimen identification (ID or cf.) and source (newly sequenced or retrieved) are represented by colours, while specimen attribute (terra typica, type locality, type specimen or faunistic novelty) is represented by the shape (right). Sequences: ID = 2, cf. = 0; maximum p-distance: strict = NA, less strict = 2 %.

Haplotype network analysis of *Temnothorax leviceps* was not possible.

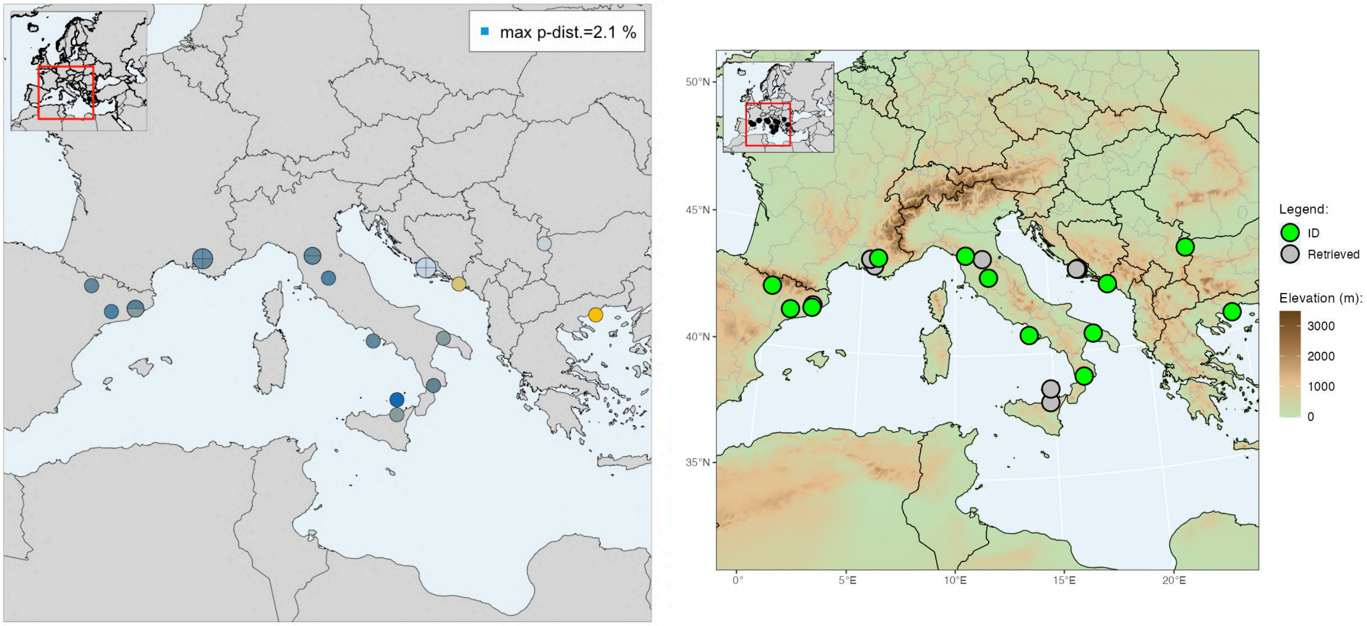

Figure 718: Genetic diversity map of *Temnothorax lichtensteini* (Bondroit, 1918). Nearby localities of sequenced specimens are merged in pies (left). Colours match the bidimensional colour space of the PCoA projection (Fig. 718 left) of p-dist between sequences (dots). Specimen identification (ID or cf.) and source (newly sequenced or retrieved) are represented by colours, while specimen attribute (terra typica, type locality, type specimen or faunistic novelty) is represented by the shape (right). Sequences: ID = 23, cf. = 0; maximum p-distance: strict = 2.1 %, less strict = 2.1 %.

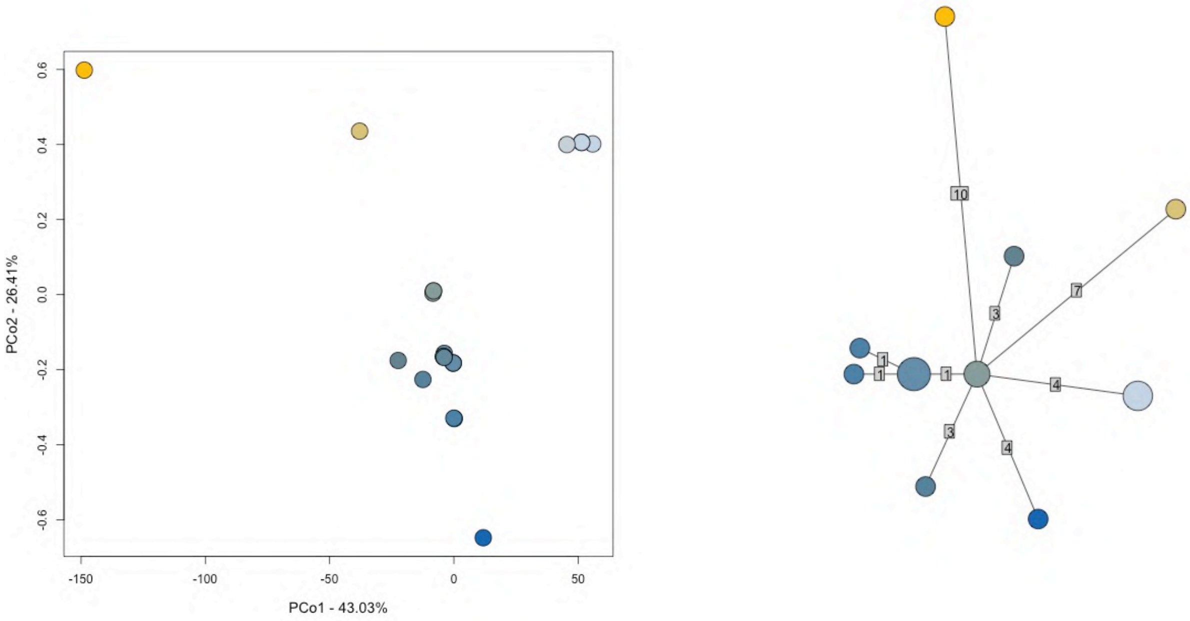

Figure 719: PCoA based on pairwise p-distances between *Temnothorax lichtensteini* sequences (left). Colours match a bidimensional colour space. Haplotype network of *Temnothorax lichtensteini* (right). Sequences > 599 bp: ID = 23, cf. = 0.

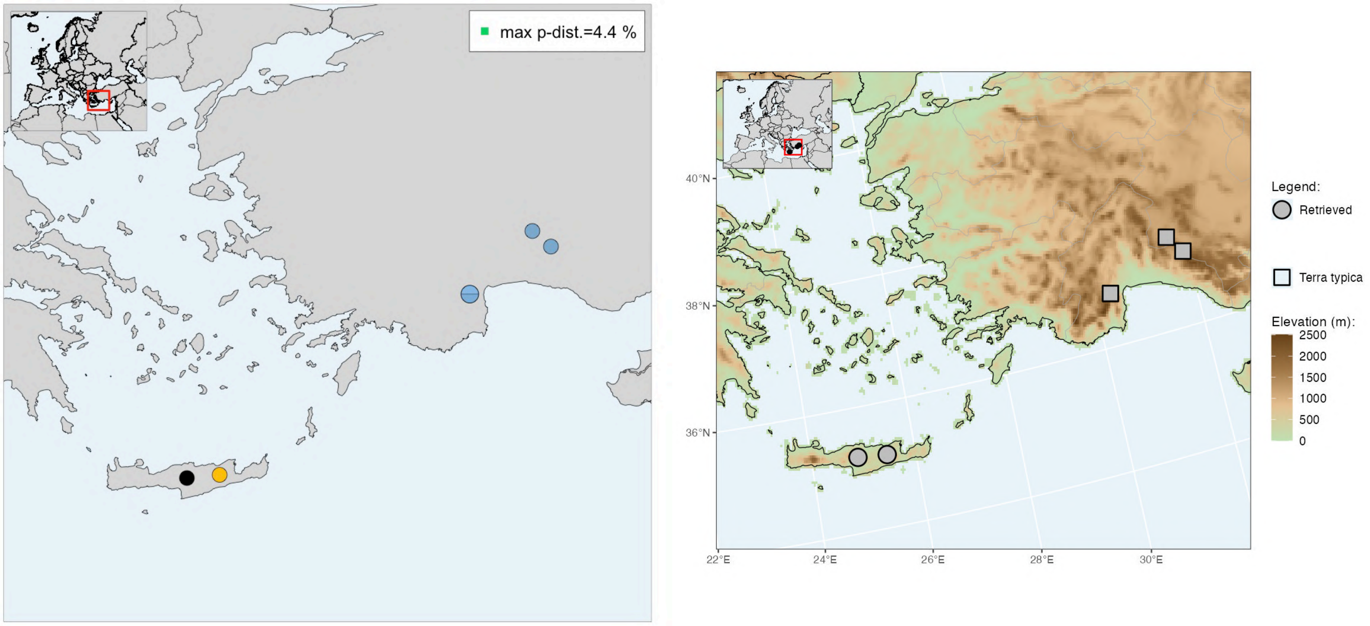

Figure 720: Genetic diversity map of *Temnothorax lucidus* Csösz, Heinze & Mikó, 2015. Nearby localities of sequenced specimens are merged in pies (left). Colours match the bidimensional colour space of the PCoA projection (Fig. 720 left) of p-dist between sequences (dots). Specimen identification (ID or cf.) and source (newly sequenced or retrieved) are represented by colours, while specimen attribute (terra typica, type locality, type specimen or faunistic novelty) is represented by the shape (right). Sequences: ID = 6, cf. = 0; maximum p-distance: strict = 4.4 %, less strict = 4.4 %.

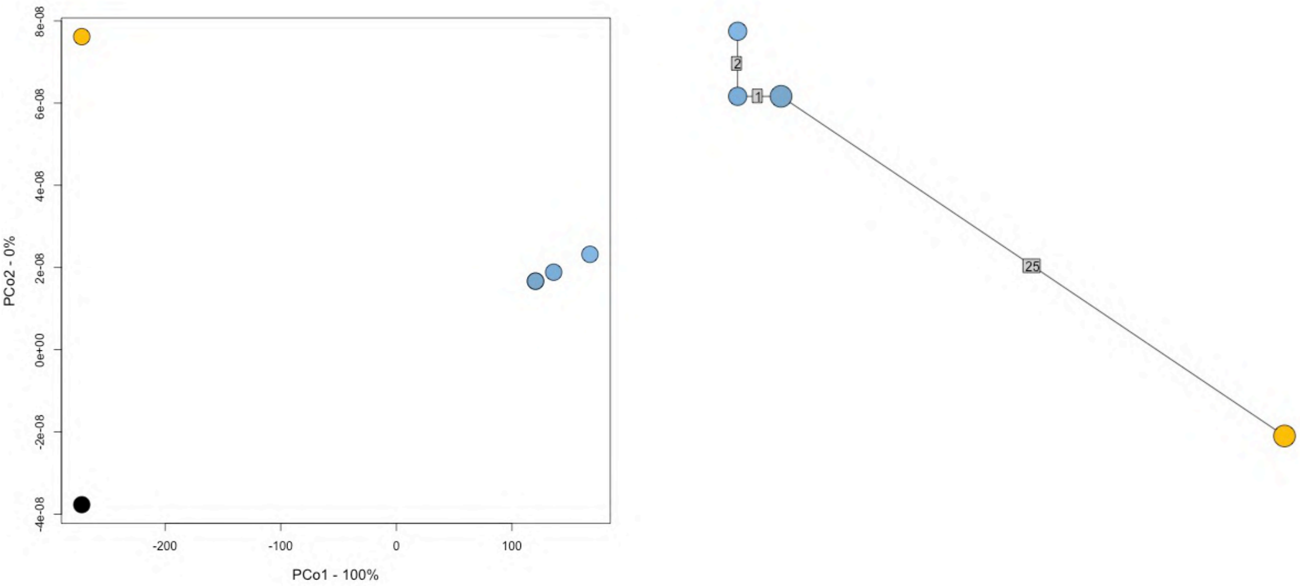

Figure 721: PCoA based on pairwise p-distances between *Temnothorax lucidus* sequences (left). Colours match a bidimensional colour space. Haplotype network of *Temnothorax lucidus* (right). Sequences > 599 bp: ID = 6, cf. = 0.

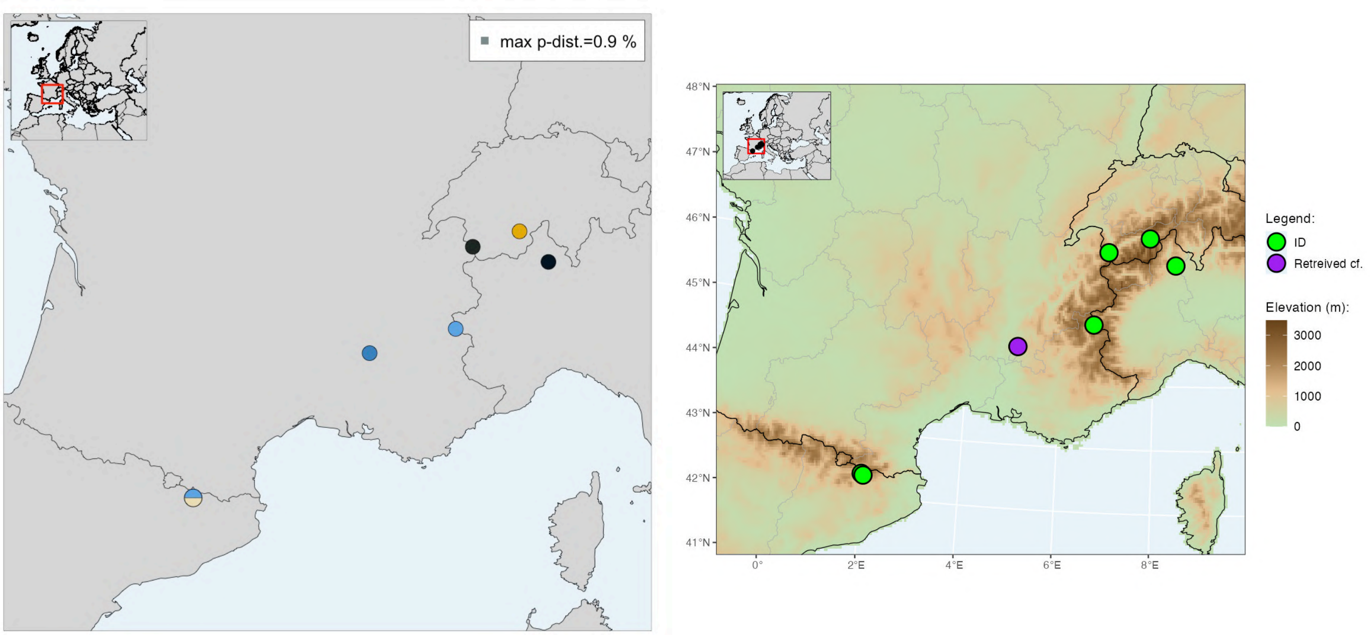

Figure 722: Genetic diversity map of *Temnothorax luteus* (Forel, 1874). Nearby localities of sequenced specimens are merged in pies (left). Colours match the bidimensional colour space of the PCoA projection (Fig. 722 left) of p-dist between sequences (dots). Specimen identification (ID or cf.) and source (newly sequenced or retrieved) are represented by colours, while specimen attribute (terra typica, type locality, type specimen or faunistic novelty) is represented by the shape (right). Sequences: ID = 6, cf. = 1; maximum p-distance: strict = 0.9 %, less strict = 0.9 %.

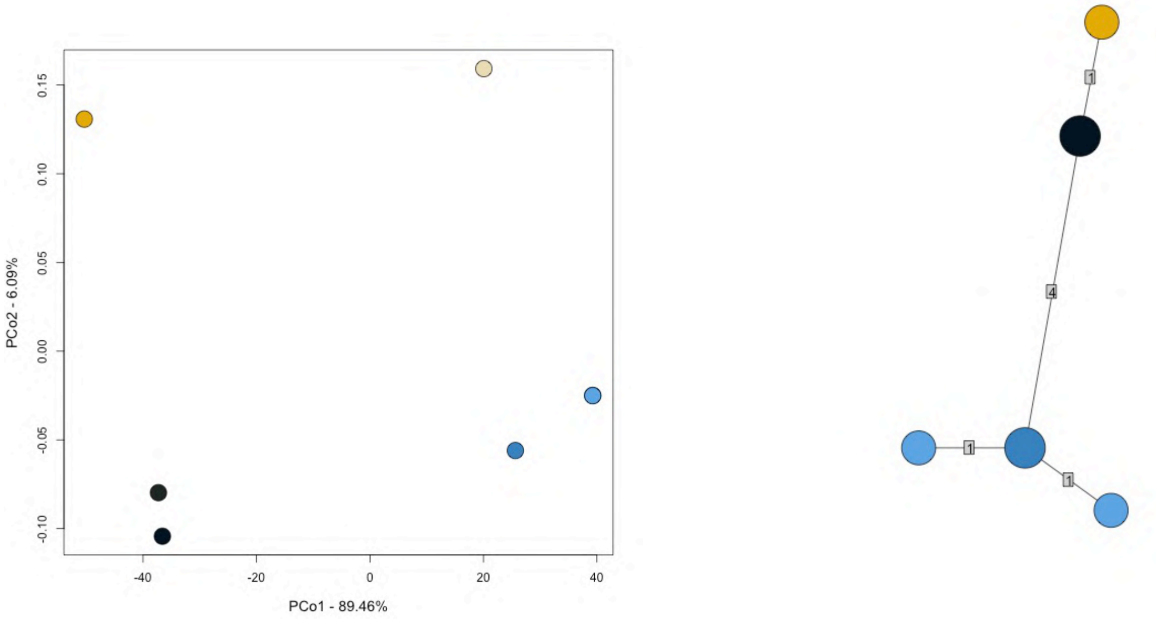

Figure 723: PCoA based on pairwise p-distances between *Temnothorax luteus* sequences (left). Colours match a bidimensional colour space. Haplotype network of *Temnothorax luteus* (right). Sequences > 599 bp: ID = 6, cf. = 1.

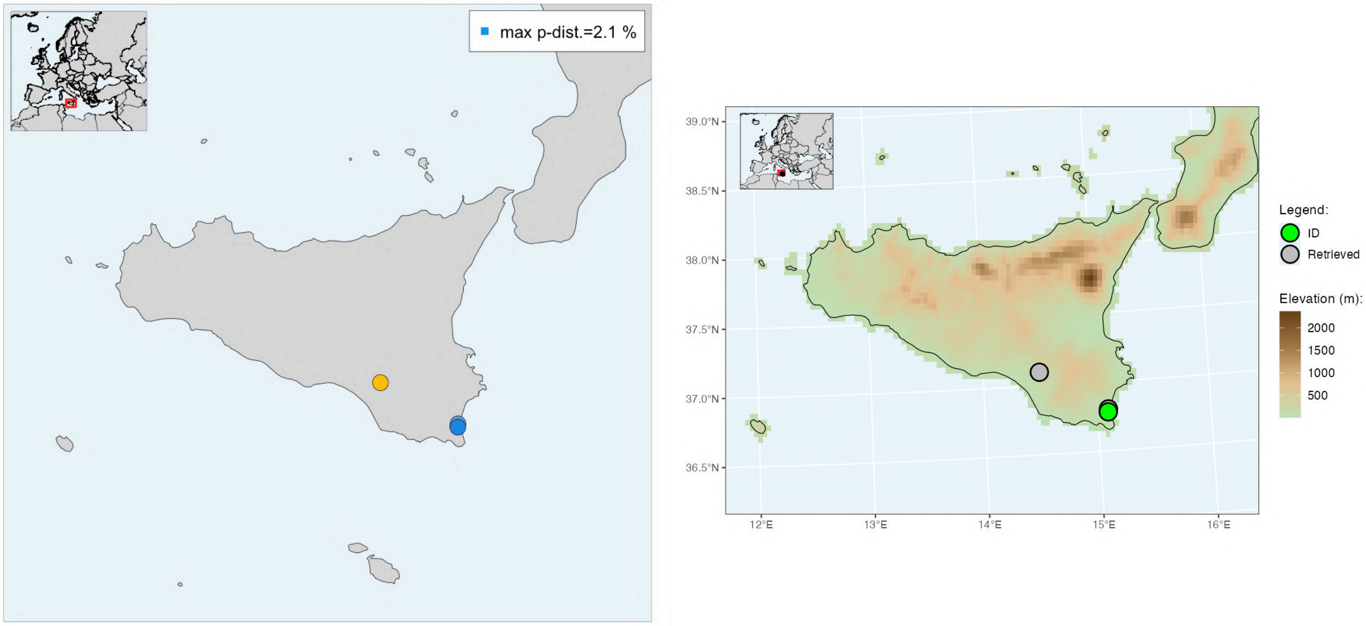

Figure 724: Genetic diversity map of *Temnothorax marae* Alicata, Schifani & Prebus, 2022. Nearby localities of sequenced specimens are merged in pies (left). Colours match the bidimensional colour space of the PCoA projection (Fig. 724 left) of p-dist between sequences (dots). Specimen identification (ID or cf.) and source (newly sequenced or retrieved) are represented by colours, while specimen attribute (terra typica, type locality, type specimen or faunistic novelty) is represented by the shape (right). Sequences: ID = 3, cf. = 0; maximum p-distance: strict = 2.1 %, less strict = 2.1 %.

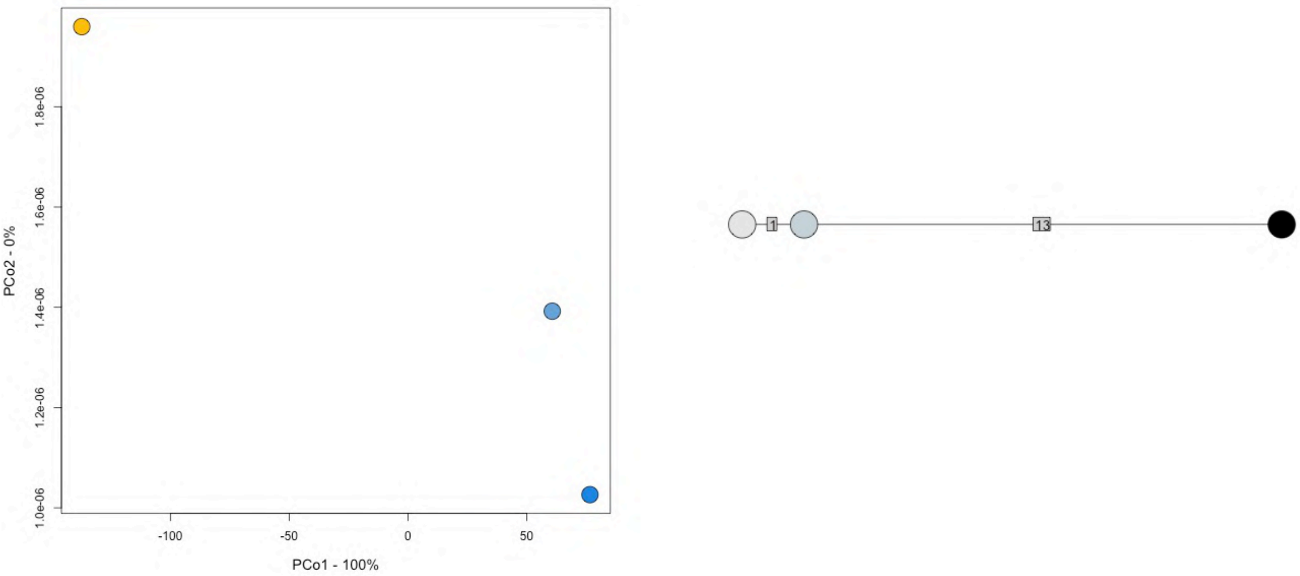

Figure 725: PCoA based on pairwise p-distances between *Temnothorax marae* sequences (left). Colours match a bidimensional colour space. Haplotype network of *Temnothorax marae* (right). Sequences > 599 bp: ID = 3, cf. = 0.

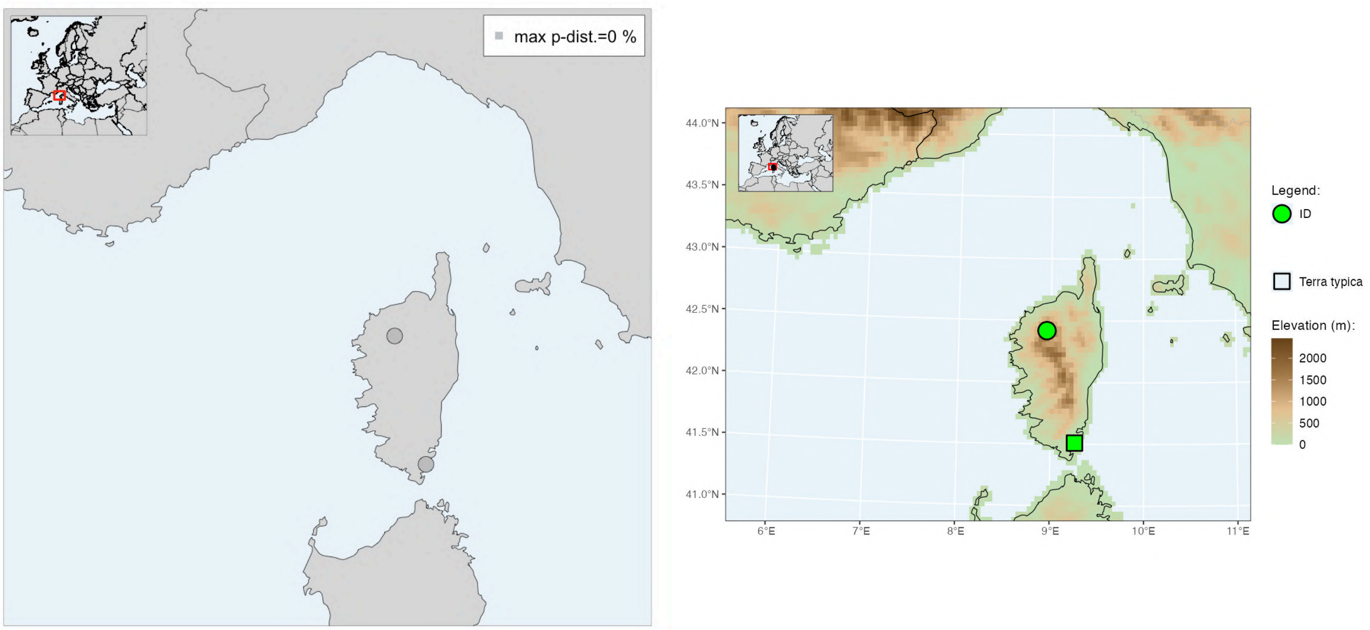

Figure 726: Genetic diversity map of *Temnothorax melas* (Espadaler, Plateaux & Casevitz-Weulersse, 1984). PCoA projection was not done and therefore sequenced specimens in the genetic diversity map are coloured in gray (left). Specimen identification (ID or cf.) and source (newly sequenced or retrieved) are represented by colours, while specimen attribute (terra typica, type locality, type specimen or faunistic novelty) is represented by the shape (right). Sequences: ID = 2, cf. = 0; maximum p-distance: strict = NA, less strict = 0 %.

Haplotype network analysis of *Temnothorax melas* was not possible.

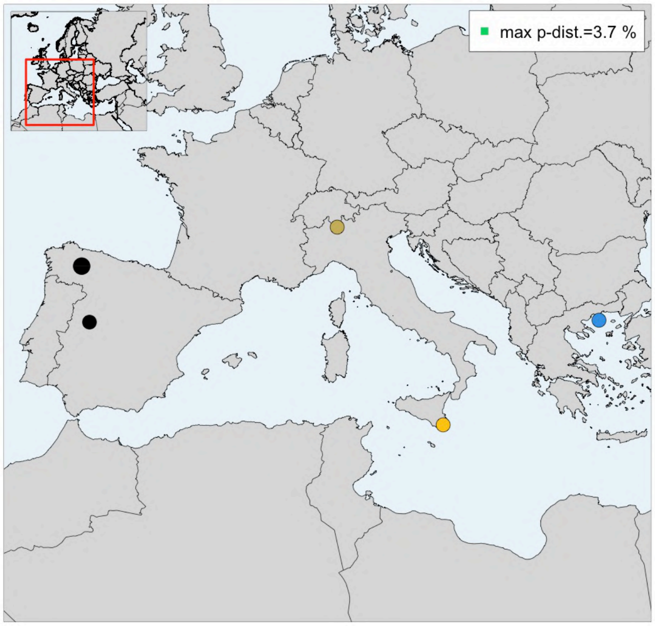

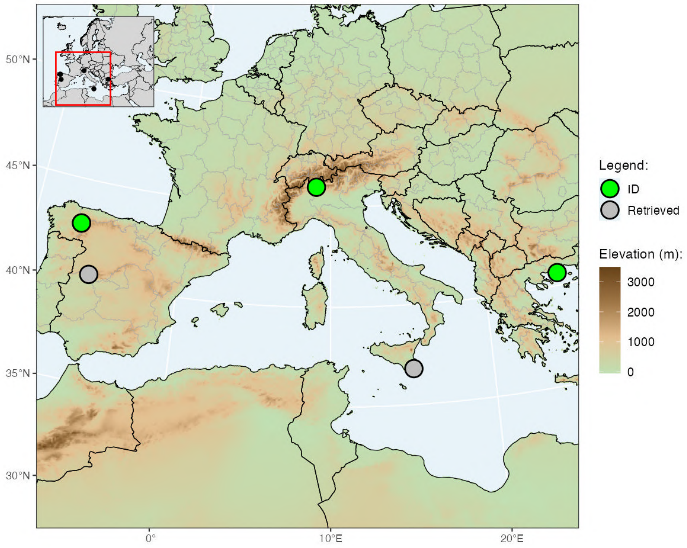

Figure 727: Genetic diversity map of *Temnothorax muellerianus* (Finzi, 1922). Nearby localities of sequenced specimens are merged in pies (left). Colours match the bidimensional colour space of the PCoA projection (Fig. 727 left) of p-dist between sequences (dots). Specimen identification (ID or cf.) and source (newly sequenced or retrieved) are represented by colours, while specimen attribute (terra typica, type locality, type specimen or faunistic novelty) is represented by the shape (right). Sequences: ID = 6, cf. = 0; maximum p-distance: strict = 3.7 %, less strict = 3.7 %.

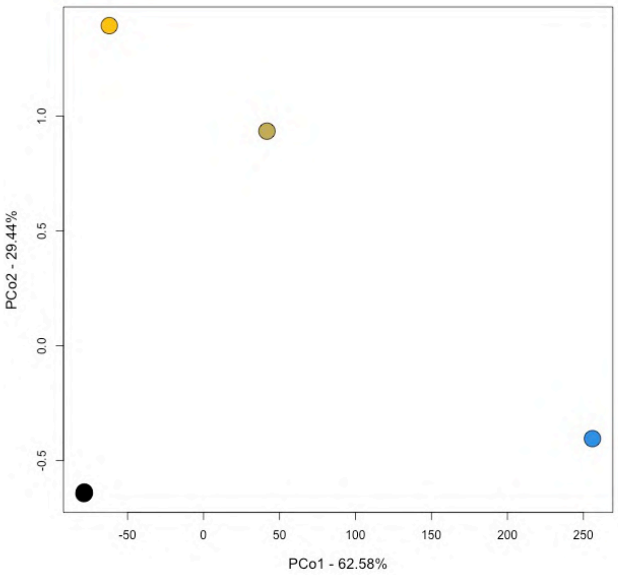

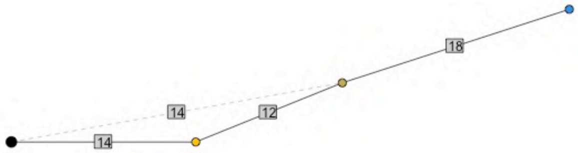

Figure 728: PCoA based on pairwise p-distances between *Temnothorax muellerianus* sequences (left). Colours match a bidimensional colour space. Haplotype network of *Temnothorax muellerianus* (right). Sequences > 599 bp: ID = 6, cf. = 0.

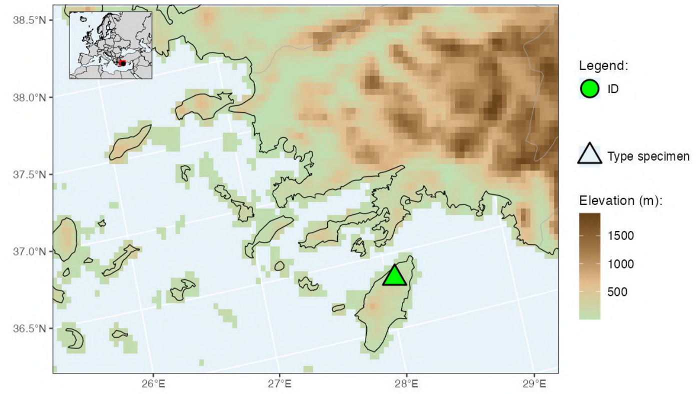

Figure 729: Map of *Temnothorax mytilenes* Salata, Srodon & Boroweic, 2023. Due to the presence of a single sequence, the genetic diversity map and the PCoA projection were not done. Specimen identification (ID or cf.) and source (newly sequenced or retrieved) are represented by colours, while specimen attribute (terra typica, type locality, type specimen or faunistic novelty) is represented by the shape. Sequences: ID = 1, cf. = 0; maximum p-distance: strict = NA, less strict = NA.

Haplotype network analysis of *Temnothorax mytilenes* was not possible.

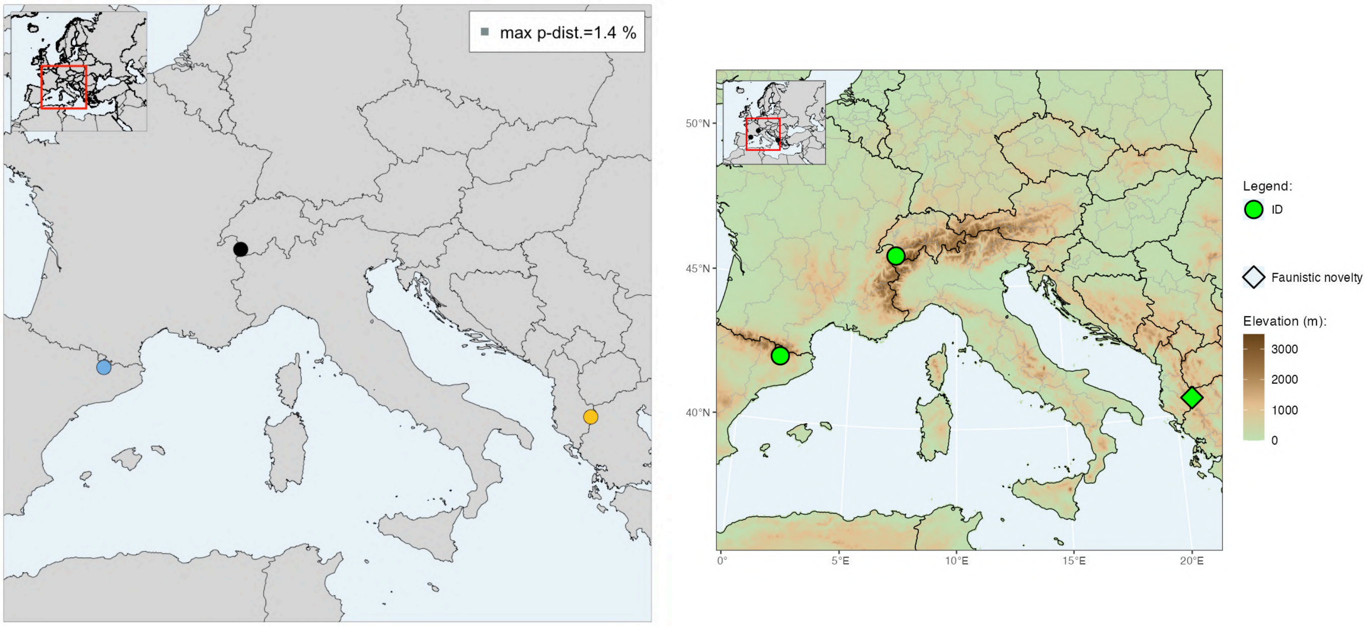

Figure 730: Genetic diversity map of *Temnothorax nadigi* (Kutter, 1925). Nearby localities of sequenced specimens are merged in pies (left). Colours match the bidimensional colour space of the PCoA projection (Fig. 730 left) of p-dist between sequences (dots). Specimen identification (ID or cf.) and source (newly sequenced or retrieved) are represented by colours, while specimen attribute (terra typica, type locality, type specimen or faunistic novelty) is represented by the shape (right). Sequences: ID = 3, cf. = 0; maximum p-distance: strict = 1.4 %, less strict = 1.4 %.

The species is reported for the first time in Albania.

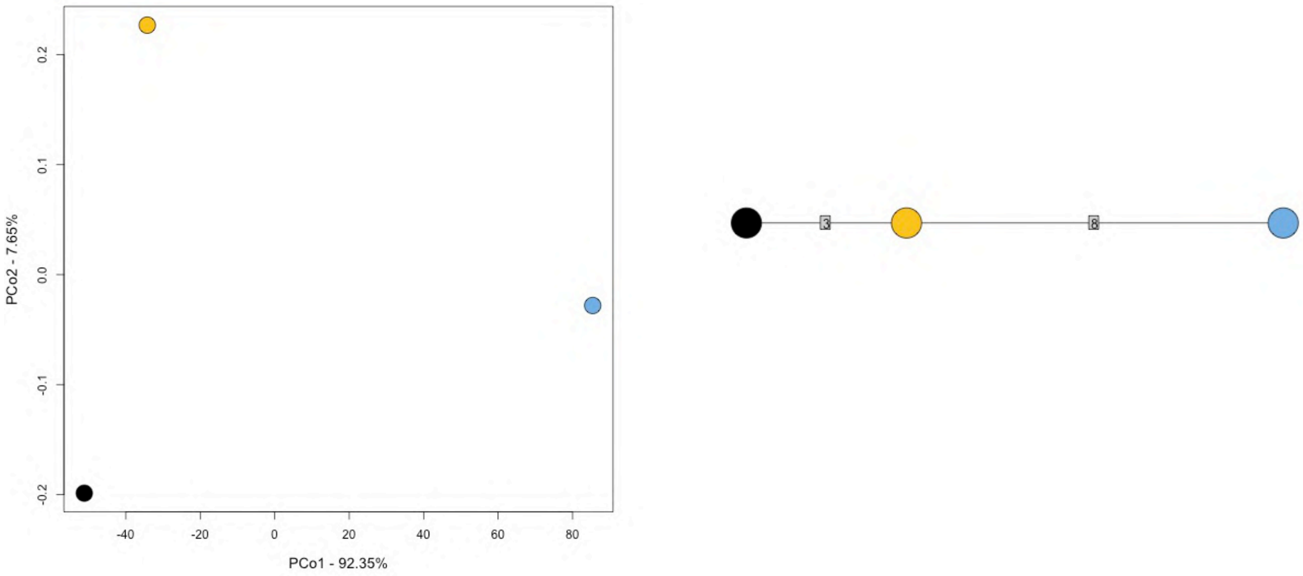

Figure 731: PCoA based on pairwise p-distances between *Temnothorax nadigi* sequences (left). Colours match a bidimensional colour space. Haplotype network of *Temnothorax nadigi* (right). Sequences > 599 bp: ID = 3, cf. = 0.

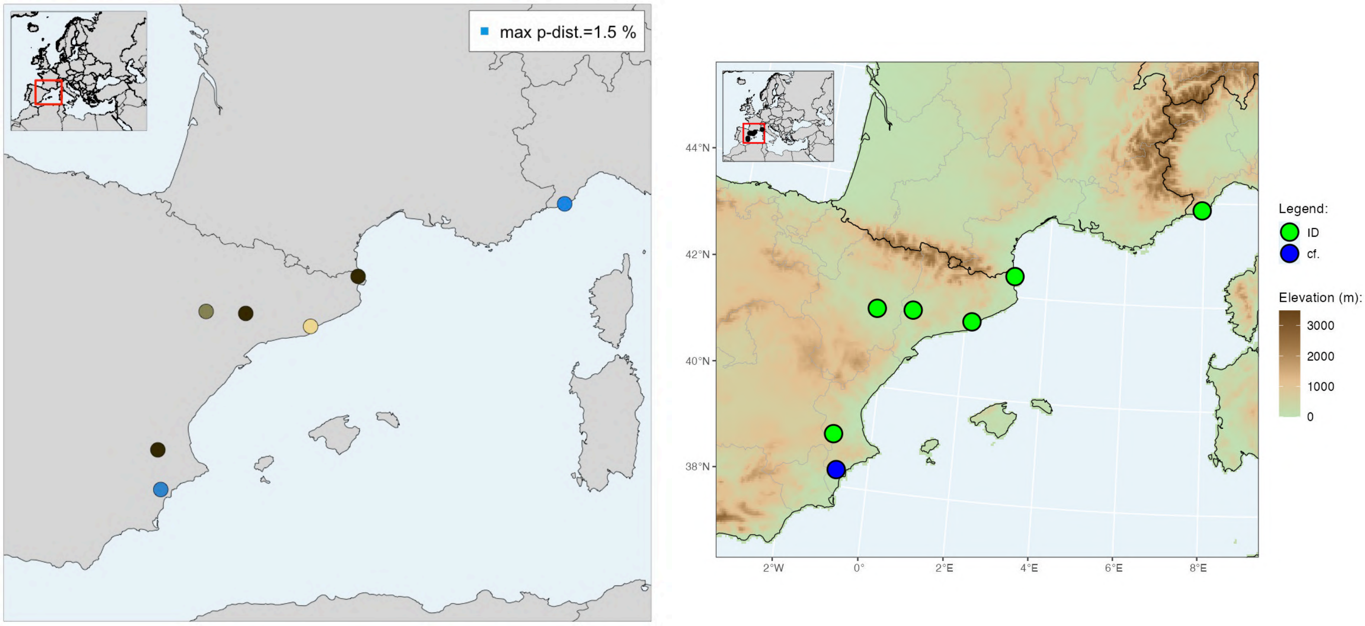

Figure 732: Genetic diversity map of *Temnothorax niger* (Forel, 1894). Nearby localities of sequenced specimens are merged in pies (left). Colours match the bidimensional colour space of the PCoA projection (Fig. 732 left) of p-dist between sequences (dots). Specimen identification (ID or cf.) and source (newly sequenced or retrieved) are represented by colours, while specimen attribute (terra typica, type locality, type specimen or faunistic novelty) is represented by the shape (right). Sequences: ID = 6, cf. = 1; maximum p-distance: strict = 1.5 %, less strict = 1.5 %.

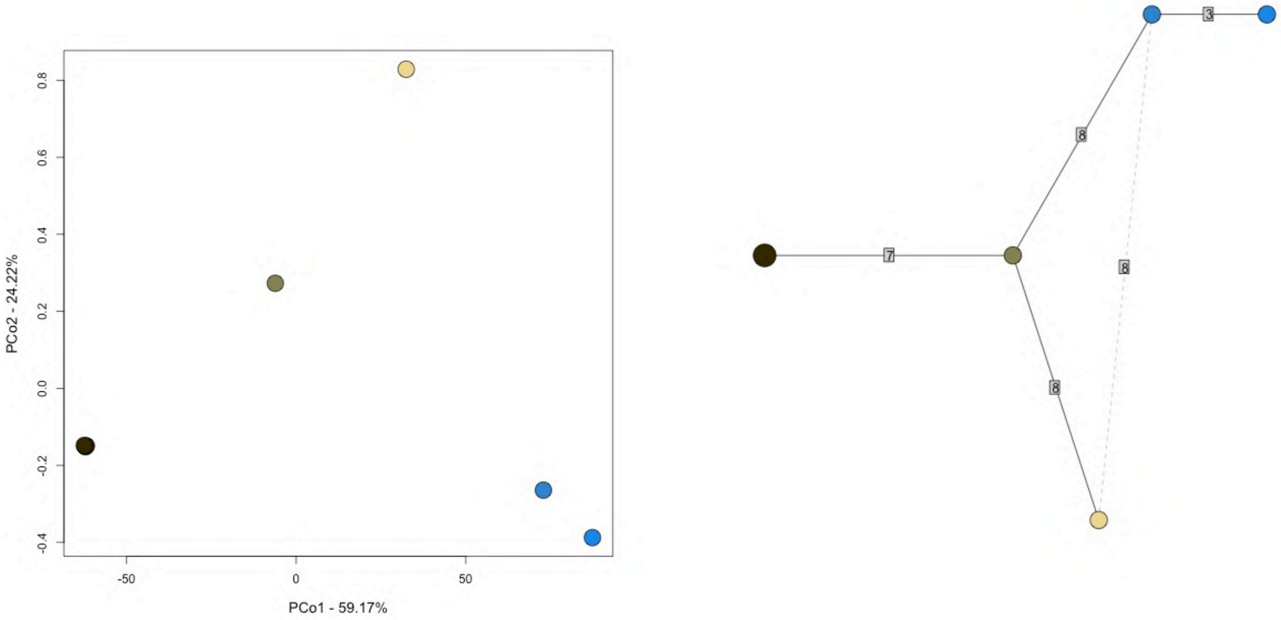

Figure 733: PCoA based on pairwise p-distances between *Temnothorax niger* sequences (left). Colours match a bidimensional colour space. Haplotype network of *Temnothorax niger* (right). Sequences > 599 bp: ID = 6, cf. = 1.

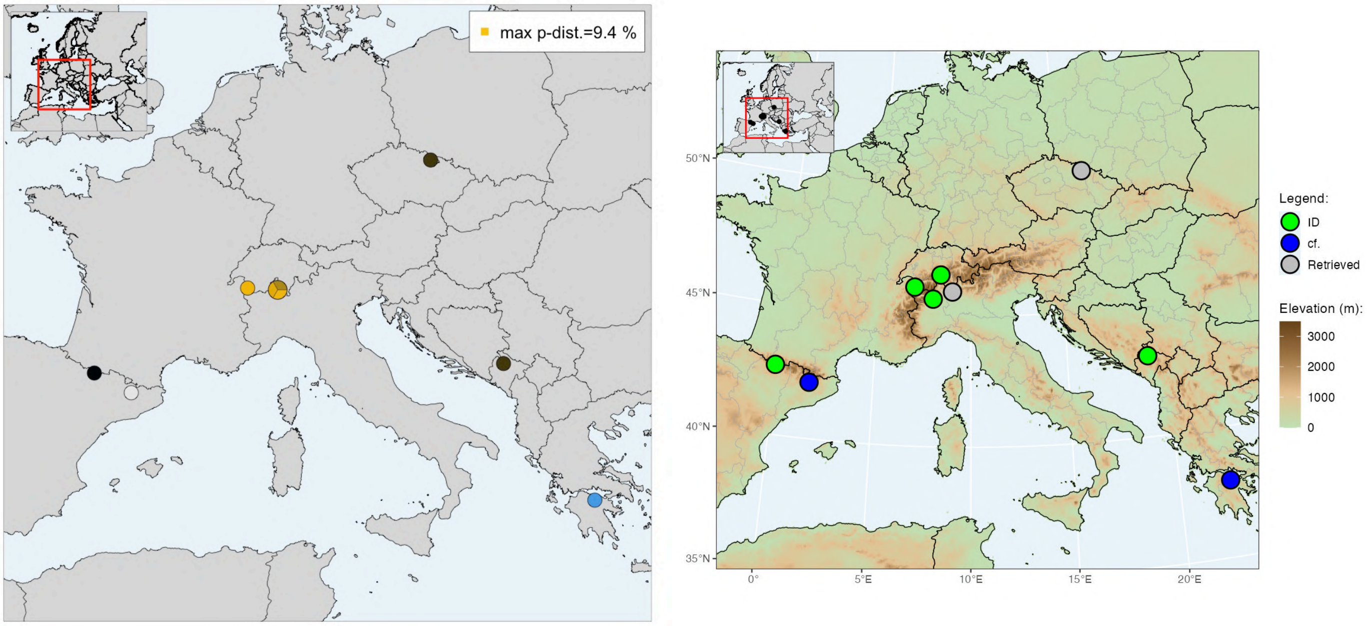

Figure 734: Genetic diversity map of *Temnothorax nigriceps* (Mayr, 1855). Nearby localities of sequenced specimens are merged in pies (left). Colours match the bidimensional colour space of the PCoA projection (Fig. 734 left) of p-dist between sequences (dots). Specimen identification (ID or cf.) and source (newly sequenced or retrieved) are represented by colours, while specimen attribute (terra typica, type locality, type specimen or faunistic novelty) is represented by the shape (right). Sequences: ID = 7, cf. = 2; maximum p-distance: strict = 3.7 %, less strict = 9.4 %.

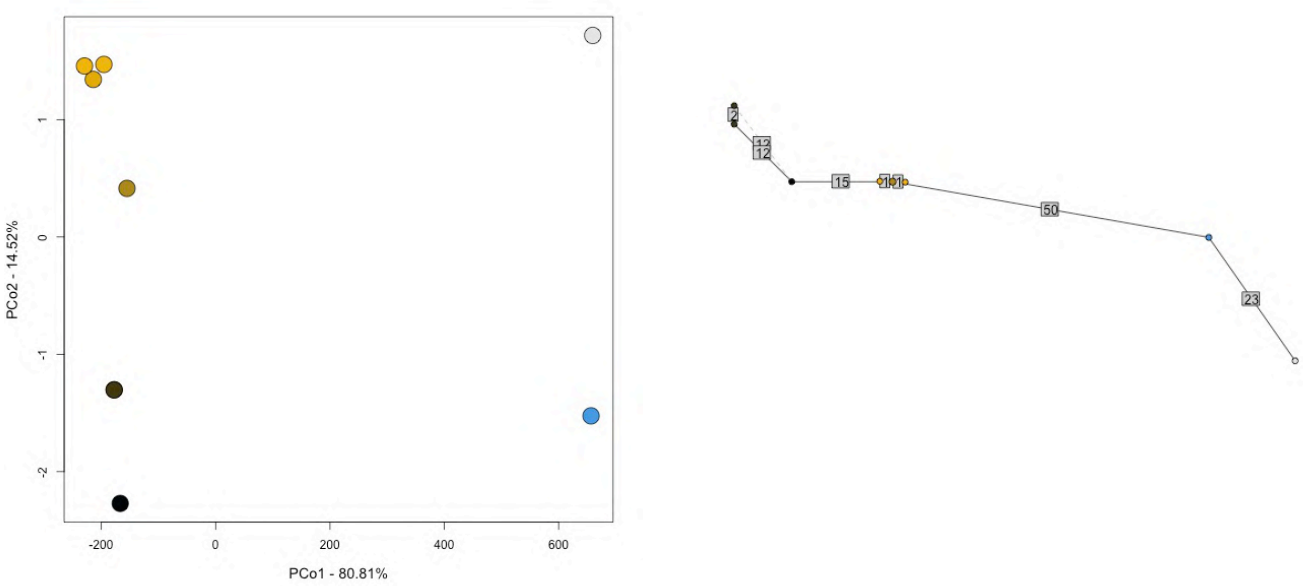

Figure 735: PCoA based on pairwise p-distances between *Temnothorax nigriceps* sequences (left). Colours match a bidimensional colour space. Haplotype network of *Temnothorax nigriceps* (right). Sequences > 599 bp: ID = 7, cf. = 2.

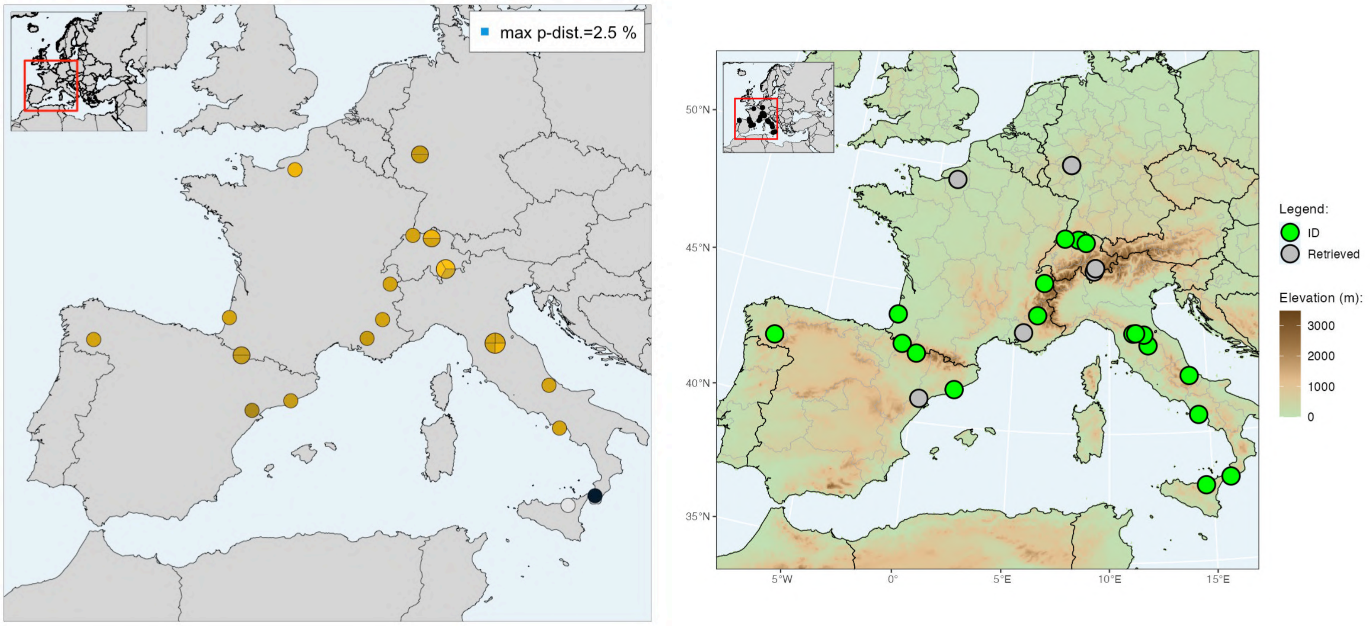

Figure 736: Genetic diversity map of *Temnothorax nylanderi* (Foerster, 1850). Nearby localities of sequenced specimens are merged in pies (left). Colours match the bidimensional colour space of the PCoA projection (Fig. 736 left) of p-dist between sequences (dots). Specimen identification (ID or cf.) and source (newly sequenced or retrieved) are represented by colours, while specimen attribute (terra typica, type locality, type specimen or faunistic novelty) is represented by the shape (right). Sequences: ID = 26, cf. = 0; maximum p-distance: strict = 2.5 %, less strict = 2.5 %.

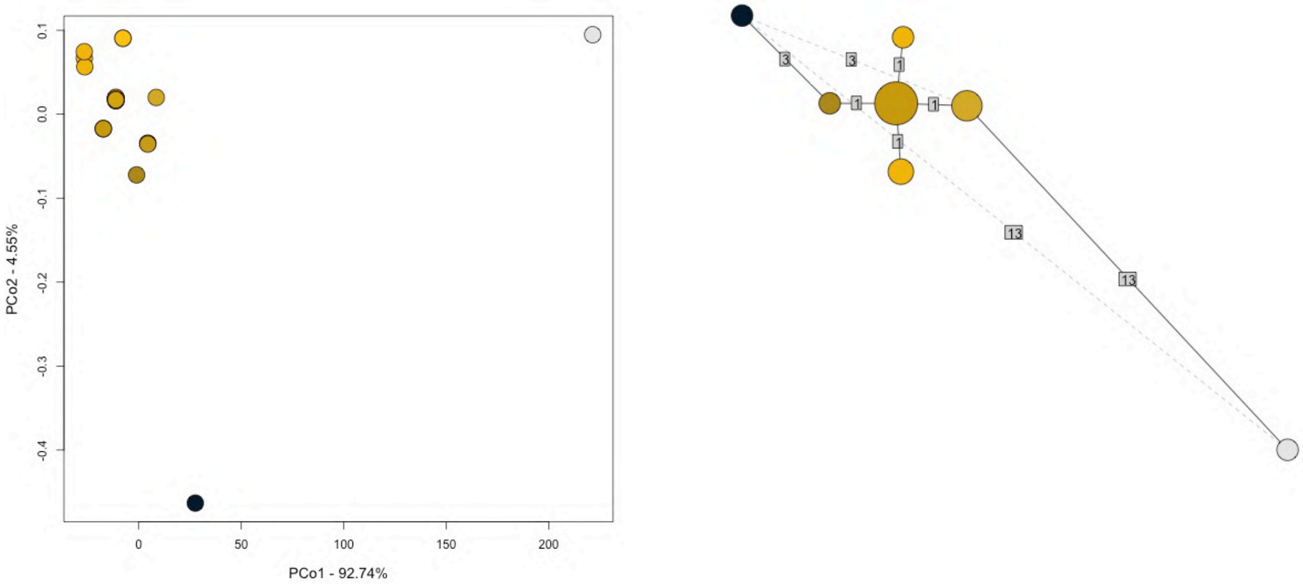

Figure 737: PCoA based on pairwise p-distances between *Temnothorax nylanderi* sequences (left). Colours match a bidimensional colour space. Haplotype network of *Temnothorax nylanderi* (right). Sequences > 599 bp: ID = 26, cf. = 0.

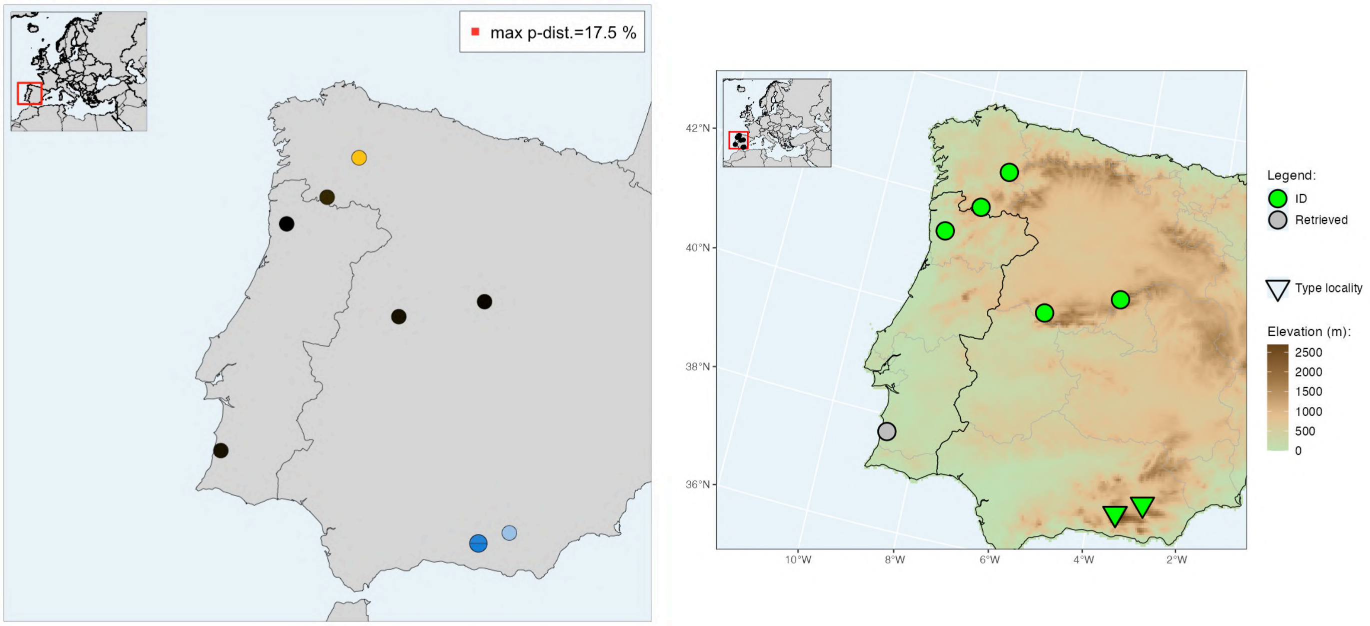

Figure 738: Genetic diversity map of *Temnothorax pardoi* (Tinaut, 1987). Nearby localities of sequenced specimens are merged in pies (left). Colours match the bidimensional colour space of the PCoA projection (Fig. 738 left) of p-dist between sequences (dots). Specimen identification (ID or cf.) and source (newly sequenced or retrieved) are represented by colours, while specimen attribute (terra typica, type locality, type specimen or faunistic novelty) is represented by the shape (right). Sequences: ID = 9, cf. = 0; maximum p-distance: strict = 17.5 %, less strict = 17.5 %.

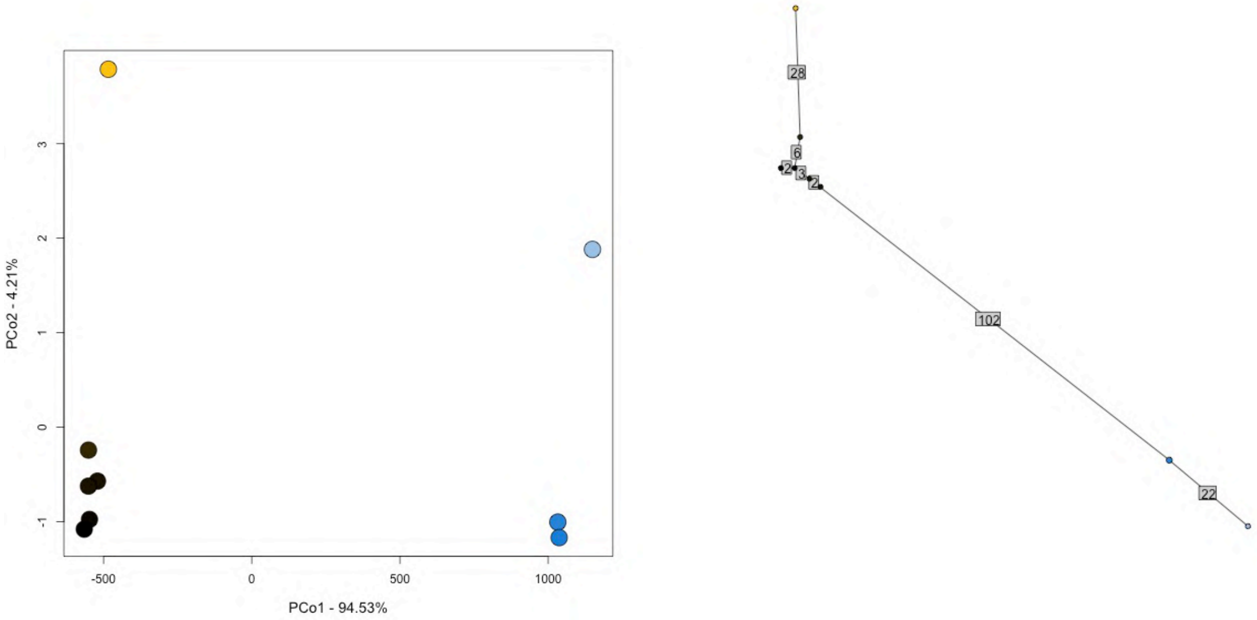

Figure 739: PCoA based on pairwise p-distances between *Temnothorax pardoi* sequences (left). Colours match a bidimensional colour space. Haplotype network of *Temnothorax pardoi* (right). Sequences > 599 bp: ID = 9, cf. = 0.

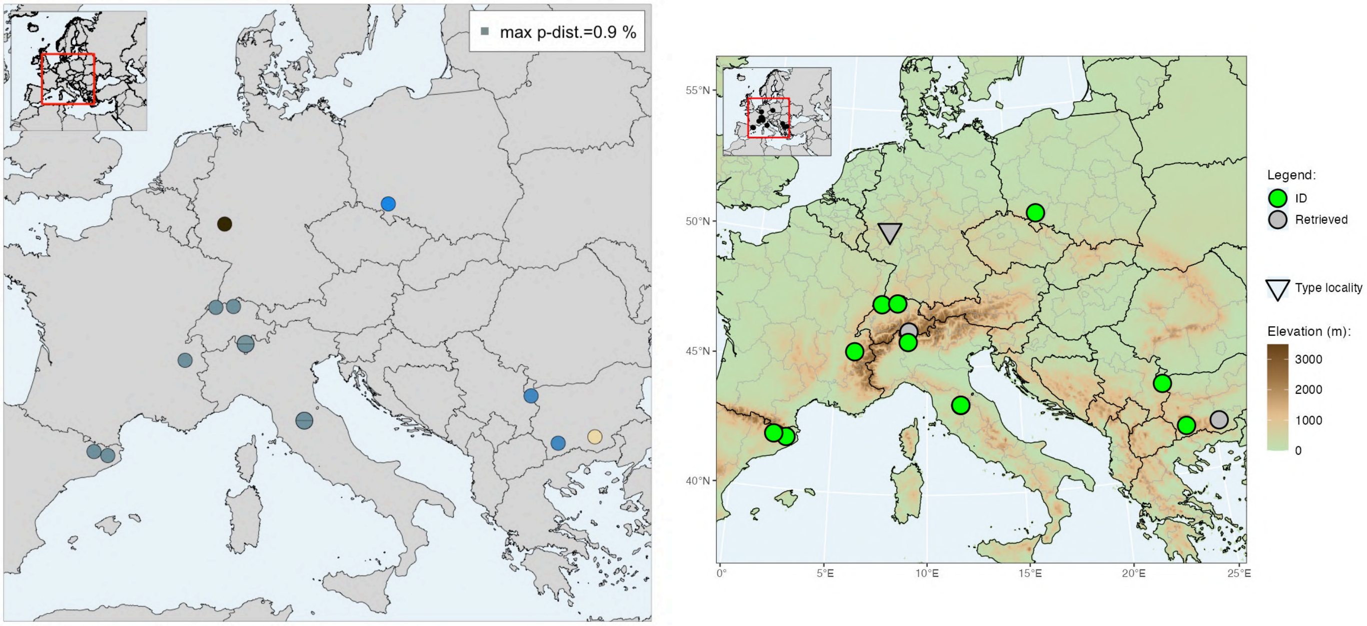

Figure 740: Genetic diversity map of *Temnothorax parvulus* (Schenck, 1852). Nearby localities of sequenced specimens are merged in pies (left). Colours match the bidimensional colour space of the PCoA projection (Fig. 740 left) of p-dist between sequences (dots). Specimen identification (ID or cf.) and source (newly sequenced or retrieved) are represented by colours, while specimen attribute (terra typica, type locality, type specimen or faunistic novelty) is represented by the shape (right). Sequences: ID = 14, cf. = 0; maximum p-distance: strict = 0.9 %, less strict = 0.9 %.

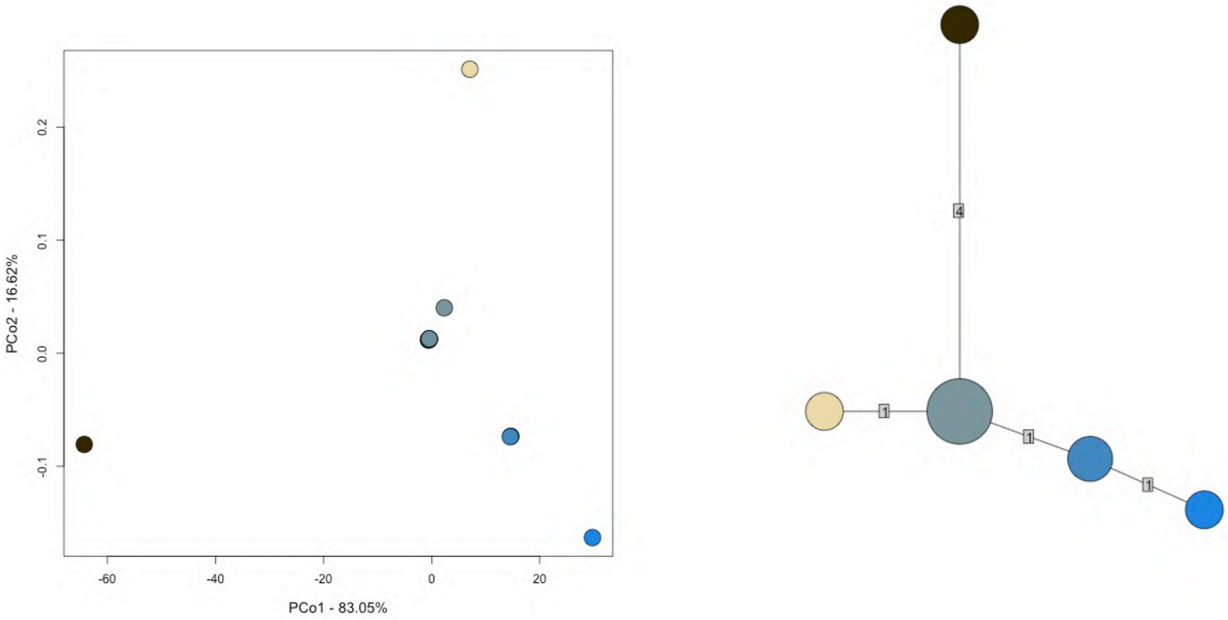

Figure 741: PCoA based on pairwise p-distances between *Temnothorax parvulus* sequences (left). Colours match a bidimensional colour space. Haplotype network of *Temnothorax parvulus* (right). Sequences > 599 bp: ID = 14, cf. = 0.

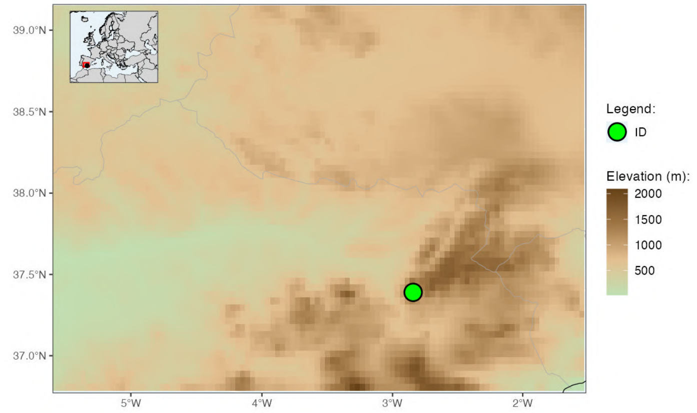

Figure 742: Map of *Temnothorax platycephalus* (Espadaler, 1997). Due to the presence of a single sequence, the genetic diversity map and the PCoA projection were not done. Specimen identification (ID or cf.) and source (newly sequenced or retrieved) are represented by colours, while specimen attribute (terra typica, type locality, type specimen or faunistic novelty) is represented by the shape. Sequences: ID = 1, cf. = 0; maximum p-distance: strict = NA, less strict = NA.

Haplotype network analysis of *Temnothorax platycephalus* was not possible.

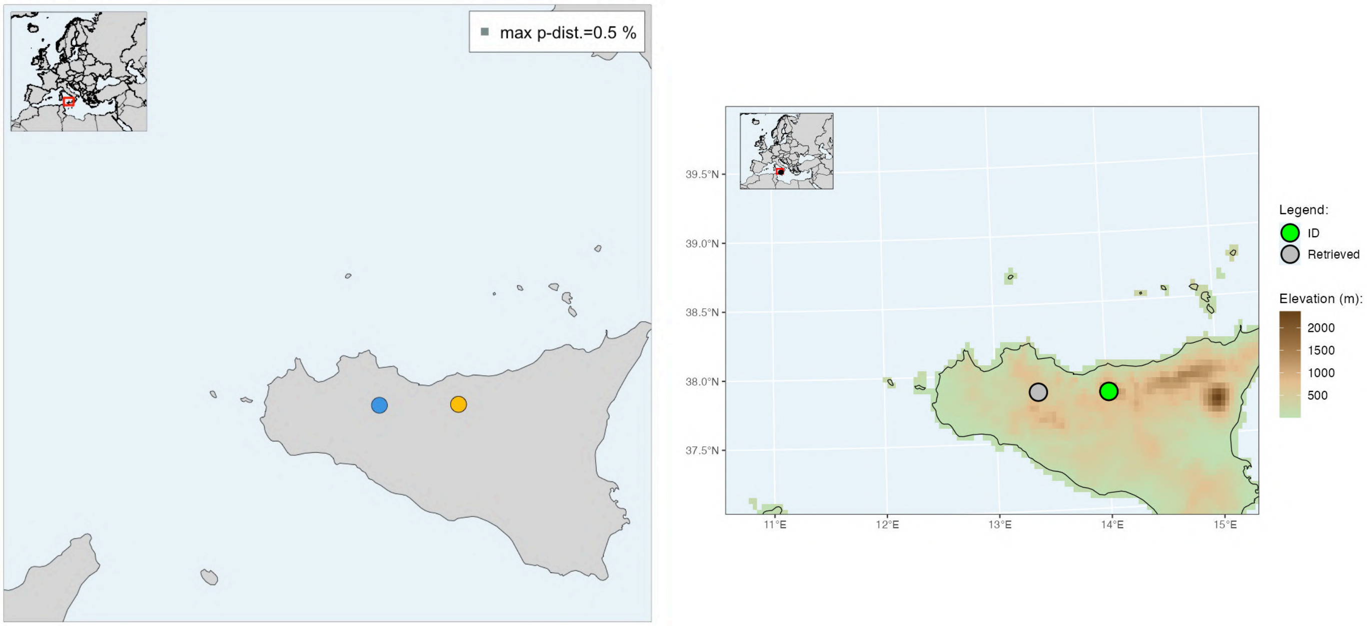

Figure 743: Genetic diversity map of *Temnothorax poldii* Alicata, Schifani & Prebus, 2022. Nearby localities of sequenced specimens are merged in pies (left). Colours match the bidimensional colour space of the PCoA projection (Fig. 743 left) of p-dist between sequences (dots). Specimen identification (ID or cf.) and source (newly sequenced or retrieved) are represented by colours, while specimen attribute (terra typica, type locality, type specimen or faunistic novelty) is represented by the shape (right). Sequences: ID = 3, cf. = 0; maximum p-distance: strict = 0.5 %, less strict = 0.5 %.

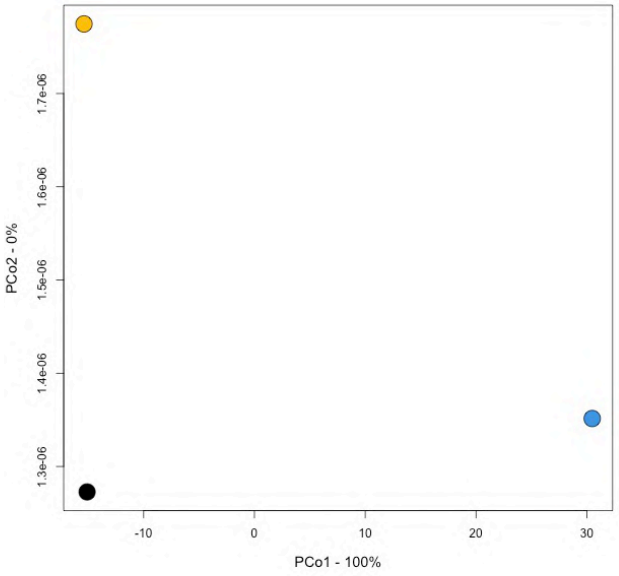

Figure 744: PCoA based on pairwise p-distances between *Temnothorax poldii* sequences (left). Colours match a bidimensional colour space. Haplotype network analysis of *Temnothorax poldii* was not possible.

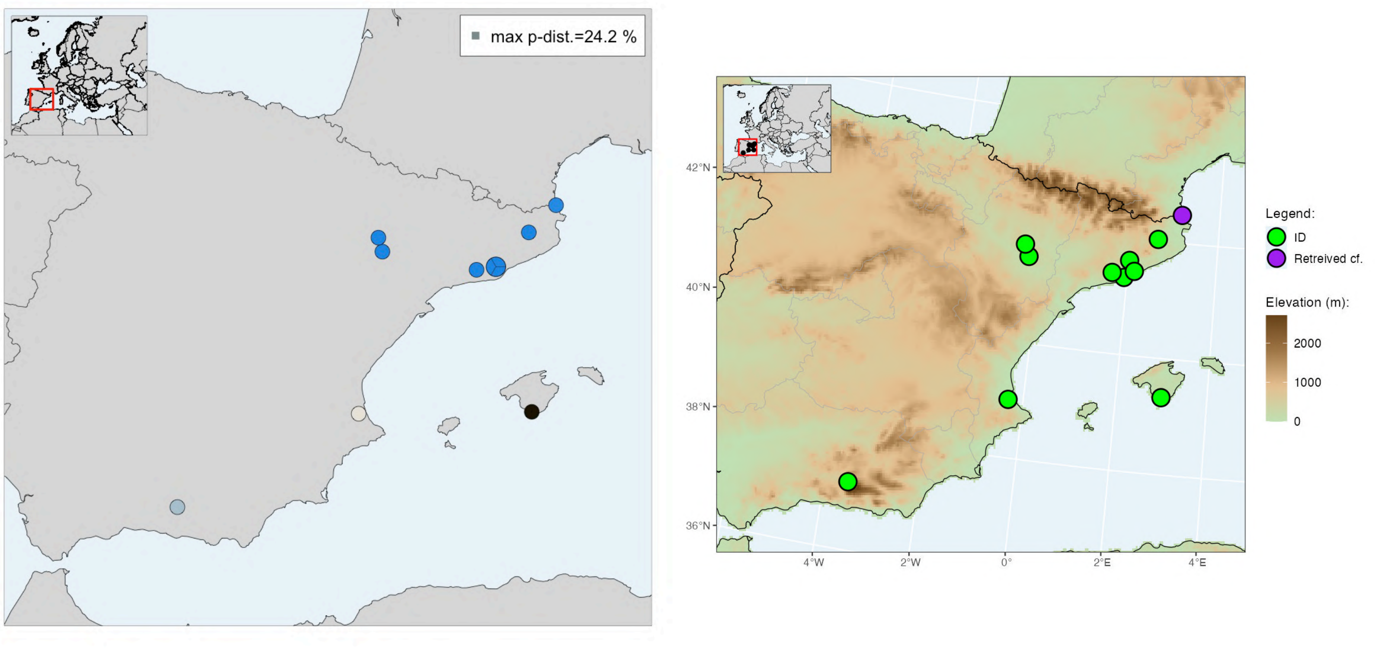

Figure 745: Genetic diversity map of *Temnothorax racovitzai* (Bondroit, 1918). Nearby localities of sequenced specimens are merged in pies (left). Colours match the bidimensional colour space of the PCoA projection (Fig. 745 left) of p-dist between sequences (dots). Specimen identification (ID or cf.) and source (newly sequenced or retrieved) are represented by colours, while specimen attribute (terra typica, type locality, type specimen or faunistic novelty) is represented by the shape (right). Sequences: ID = 10, cf. = 1; maximum p-distance: strict = 5.8 %, less strict = 24.2 %.

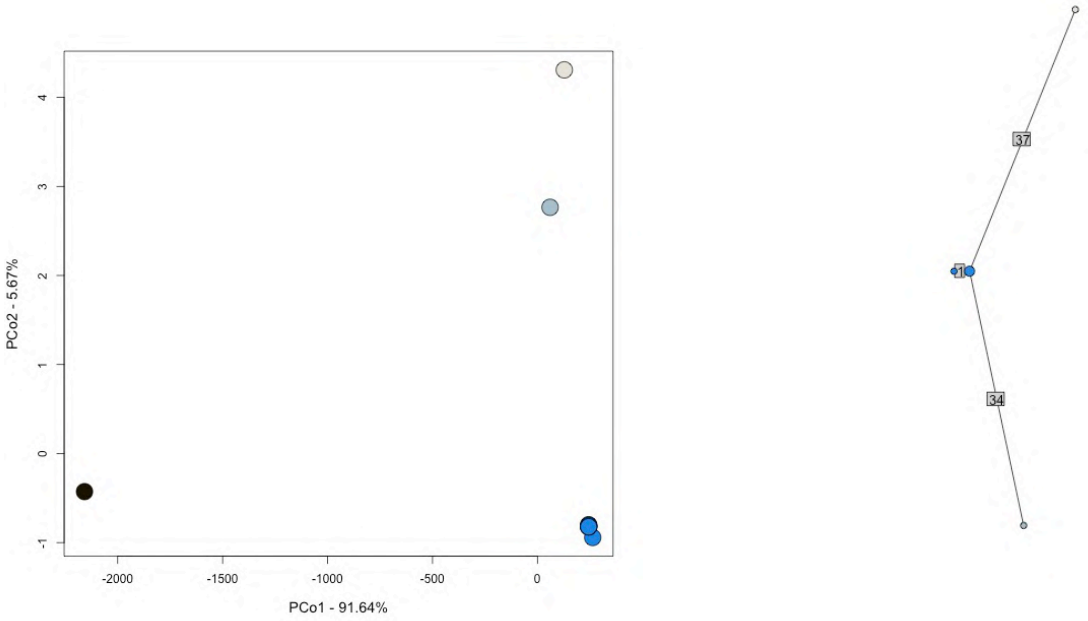

Figure 746: PCoA based on pairwise p-distances between *Temnothorax racovitzai* sequences (left). Colours match a bidimensional colour space. Haplotype network of *Temnothorax racovitzai* (right). Sequences > 599 bp: ID = 9, cf. = 1.

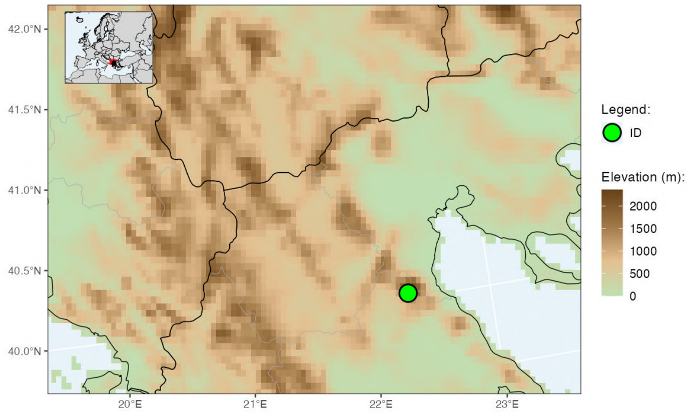

Figure 747: Map of *Temnothorax ravouxi* (André, 1896). Due to the presence of a single sequence, the genetic diversity map and the PCoA projection were not done. Specimen identification (ID or cf.) and source (newly sequenced or retrieved) are represented by colours, while specimen attribute (terra typica, type locality, type specimen or faunistic novelty) is represented by the shape. Sequences: ID = 1, cf. = 0; maximum p-distance: strict = NA, less strict = NA.

Haplotype network analysis of *Temnothorax ravouxi* was not possible.

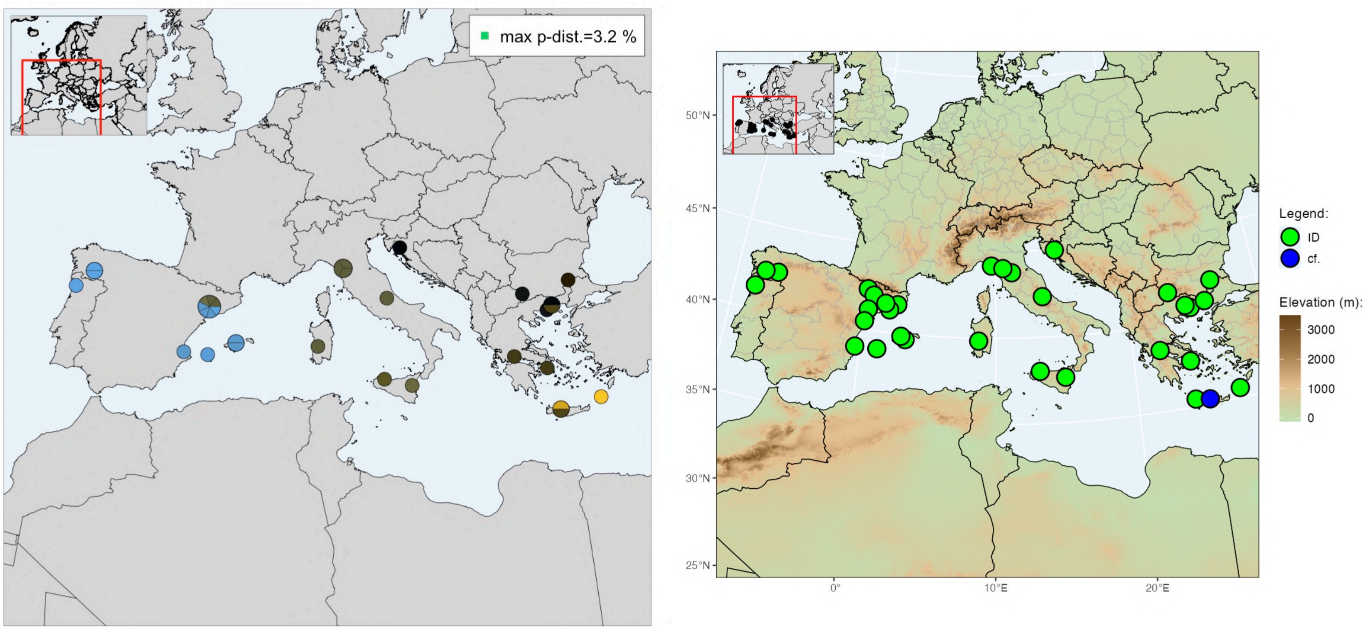

Figure 748: Genetic diversity map of *Temnothorax recedens* (Nylander, 1856). Nearby localities of sequenced specimens are merged in pies (left). Colours match the bidimensional colour space of the PCoA projection (Fig. 748 left) of p-dist between sequences (dots). Specimen identification (ID or cf.) and source (newly sequenced or retrieved) are represented by colours, while specimen attribute (terra typica, type locality, type specimen or faunistic novelty) is represented by the shape (right). Sequences: ID = 31, cf. = 1; maximum p-distance: strict = 3.2 %, less strict = 3.2 %.

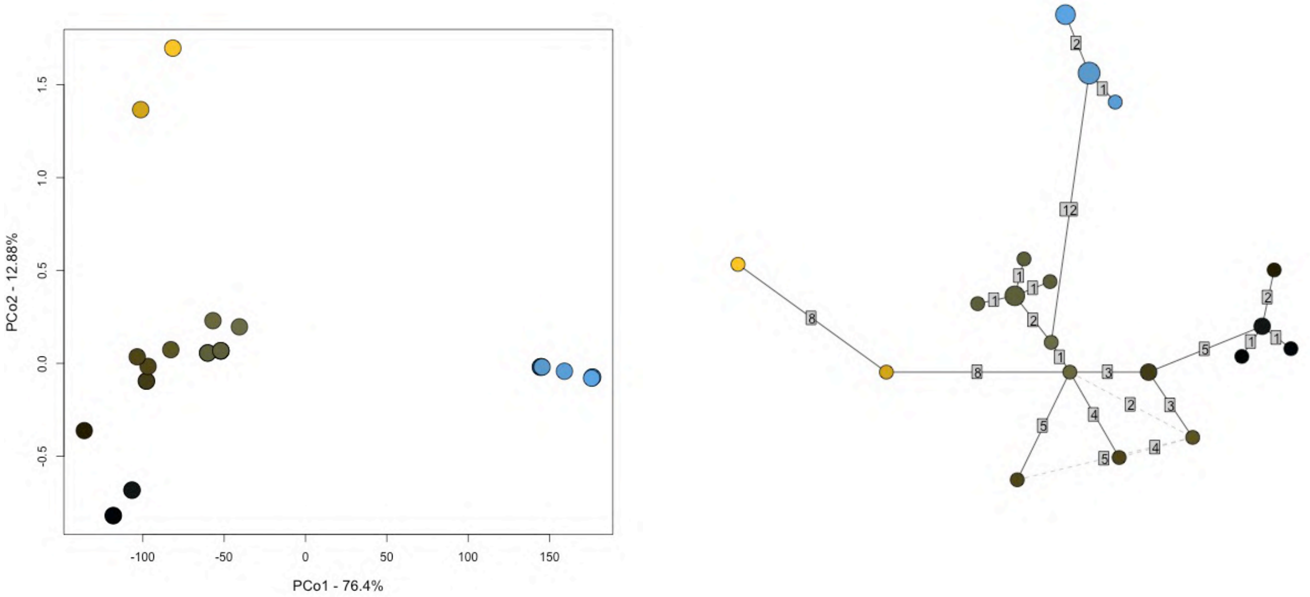

Figure 749: PCoA based on pairwise p-distances between *Temnothorax recedens* sequences (left). Colours match a bidimensional colour space. Haplotype network of *Temnothorax recedens* (right). Sequences > 599 bp: ID = 31, cf. = 1.

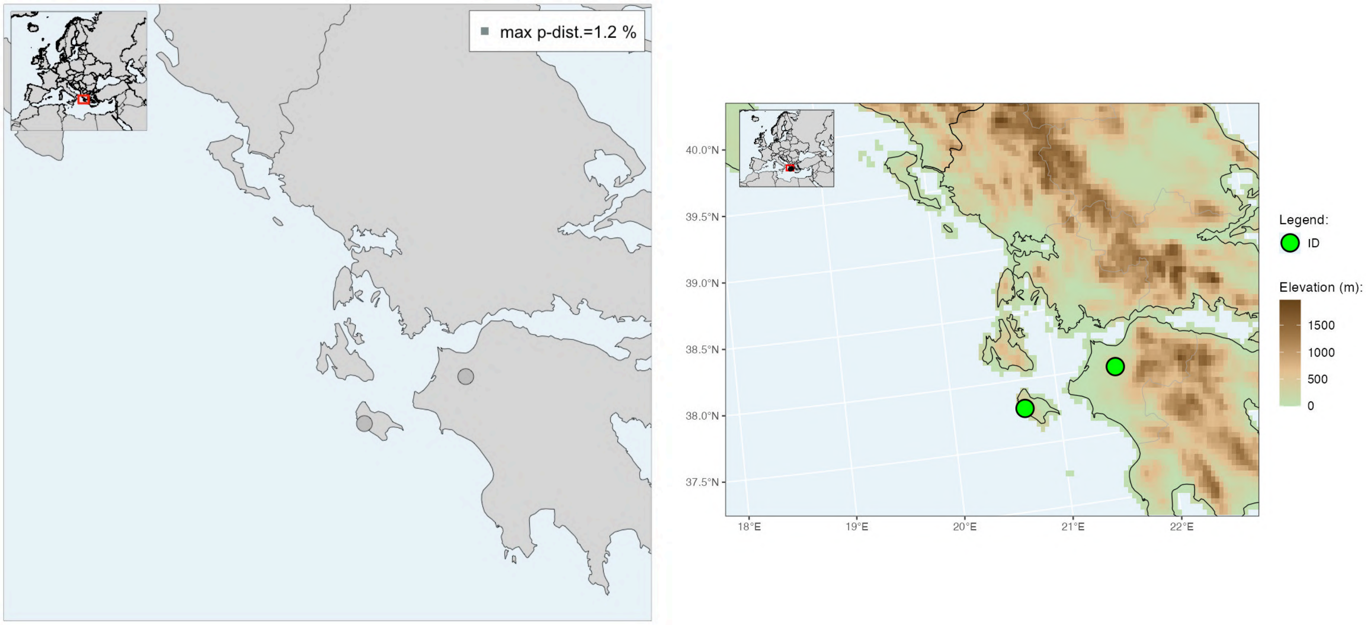

Figure 750: Genetic diversity map of *Temnothorax rogeri* Emery, 1869. PCoA projection was not done and therefore sequenced specimens in the genetic diversity map are coloured in gray (left). Specimen identification (ID or cf.) and source (newly sequenced or retrieved) are represented by colours, while specimen attribute (terra typica, type locality, type specimen or faunistic novelty) is represented by the shape (right). Sequences: ID = 2, cf. = 0; maximum p-distance: strict = NA, less strict = 1.2 %.

Haplotype network analysis of *Temnothorax rogeri* was not possible.

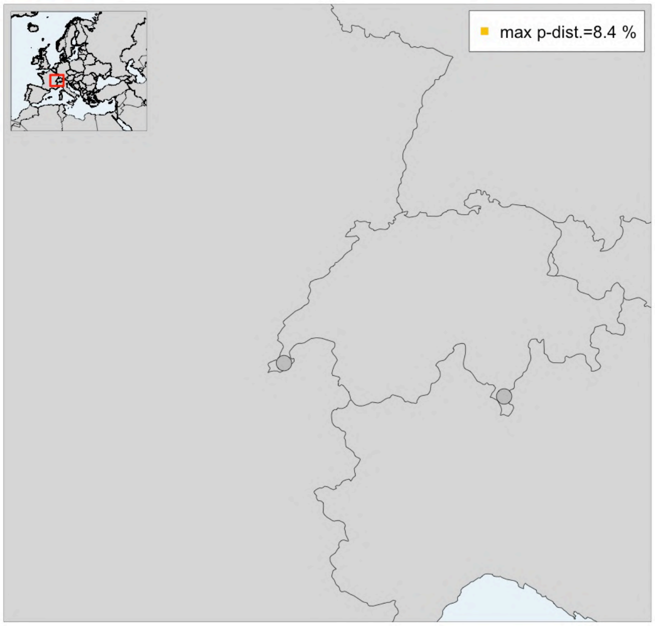

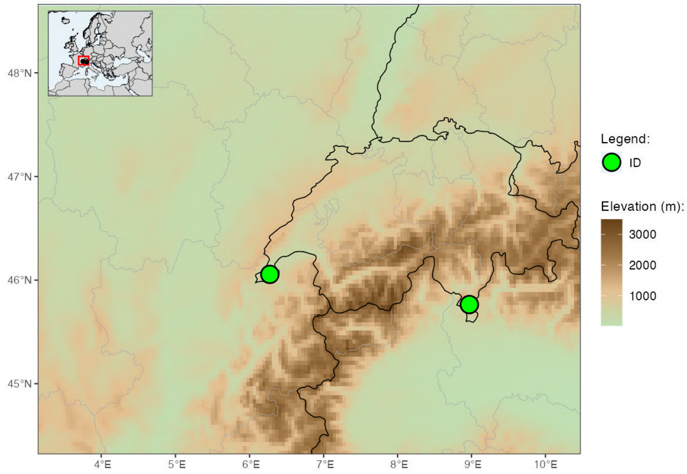

Figure 751: Genetic diversity map of *Temnothorax saxonicus* (Seifert, 1995). PCoA projection was not done and therefore sequenced specimens in the genetic diversity map are coloured in gray (left). Specimen identification (ID or cf.) and source (newly sequenced or retrieved) are represented by colours, while specimen attribute (terra typica, type locality, type specimen or faunistic novelty) is represented by the shape (right). Sequences: ID = 2, cf. = 0; maximum p-distance: strict = NA, less strict = 8.4 %.

Haplotype network analysis of *Temnothorax saxonicus* was not possible.

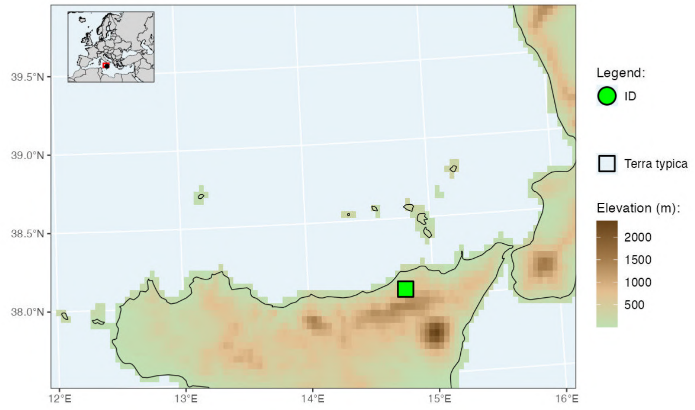

Figure 752: Map of *Temnothorax siculus* Schifani, Alicata, Prebus, Csősz, 2025. Due to the presence of a single sequence, the genetic diversity map and the PCoA projection were not done. Specimen identification (ID or cf.) and source (newly sequenced or retrieved) are represented by colours, while specimen attribute (terra typica, type locality, type specimen or faunistic novelty) is represented by the shape. Sequences: ID = 1, cf. = 0; maximum p-distance: strict = NA, less strict = NA.

Haplotype network analysis of *Temnothorax siculus* was not possible.

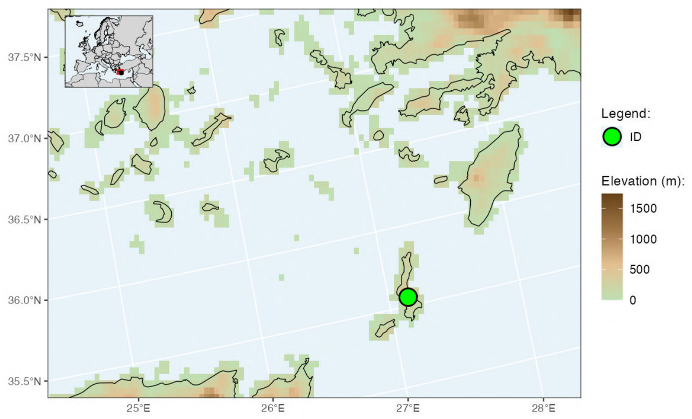

Figure 753: Map of *Temnothorax solerii* (Menozzi, 1936). Due to the presence of a single sequence, the genetic diversity map and the PCoA projection were not done. Specimen identification (ID or cf.) and source (newly sequenced or retrieved) are represented by colours, while specimen attribute (terra typica, type locality, type specimen or faunistic novelty) is represented by the shape. Sequences: ID = 1, cf. = 0; maximum p-distance: strict = NA, less strict = NA.

Haplotype network analysis of *Temnothorax solerii* was not possible.

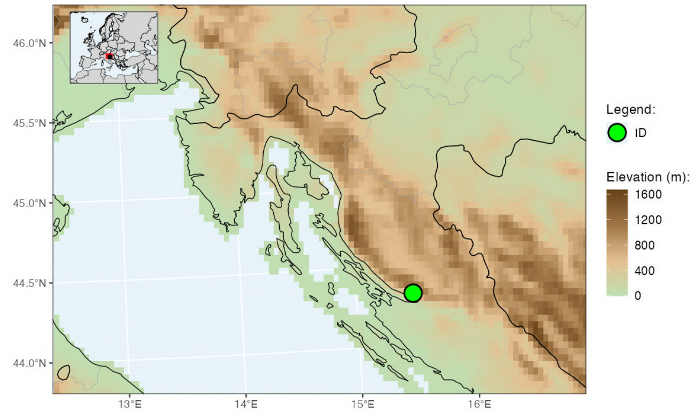

Figure 754: Map of *Temnothorax sordidulus* (Müller, 1923). Due to the presence of a single sequence, the genetic diversity map and the PCoA projection were not done. Specimen identification (ID or cf.) and source (newly sequenced or retrieved) are represented by colours, while specimen attribute (terra typica, type locality, type specimen or faunistic novelty) is represented by the shape. Sequences: ID = 1, cf. = 0; maximum p-distance: strict = NA, less strict = NA.

Haplotype network analysis of *Temnothorax sordidulus* was not possible.

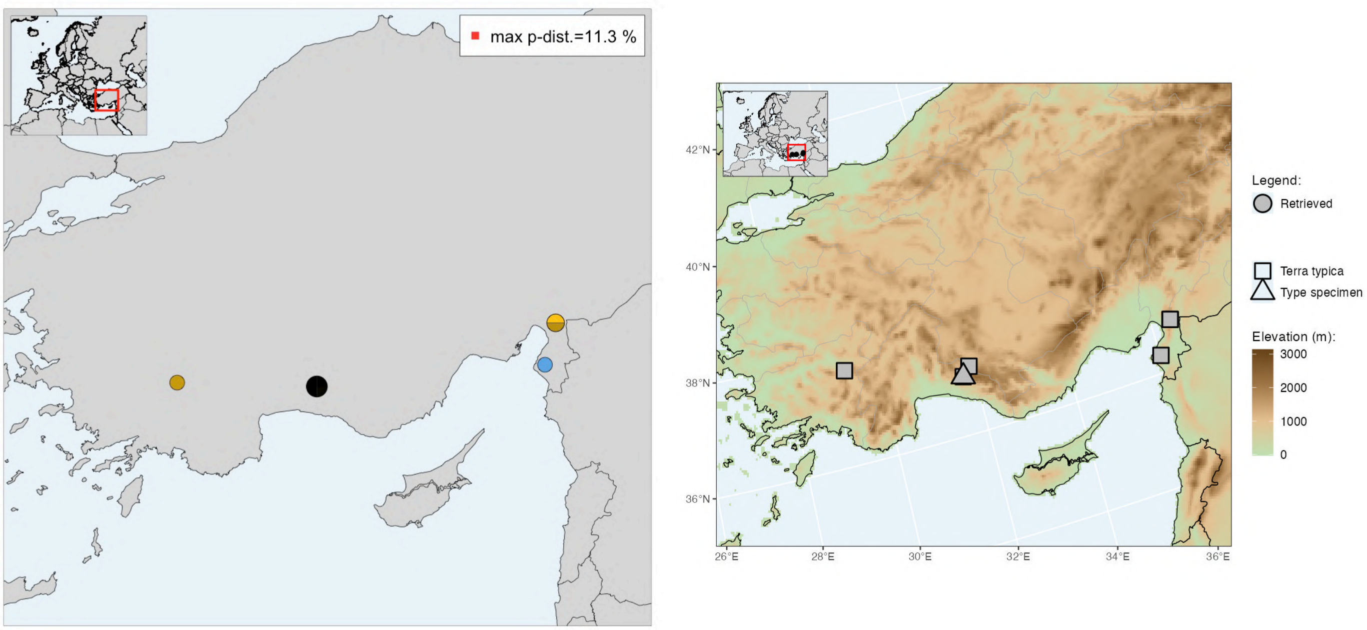

Figure 755: Genetic diversity map of *Temnothorax subtilis* Csösz, Heinze & Mikó, 2015. Nearby localities of sequenced specimens are merged in pies (left). Colours match the bidimensional colour space of the PCoA projection (Fig. 755 left) of p-dist between sequences (dots). Specimen identification (ID or cf.) and source (newly sequenced or retrieved) are represented by colours, while specimen attribute (terra typica, type locality, type specimen or faunistic novelty) is represented by the shape (right). Sequences: ID = 8, cf. = 0; maximum p-distance: strict = 11.3 %, less strict = 11.3 %.

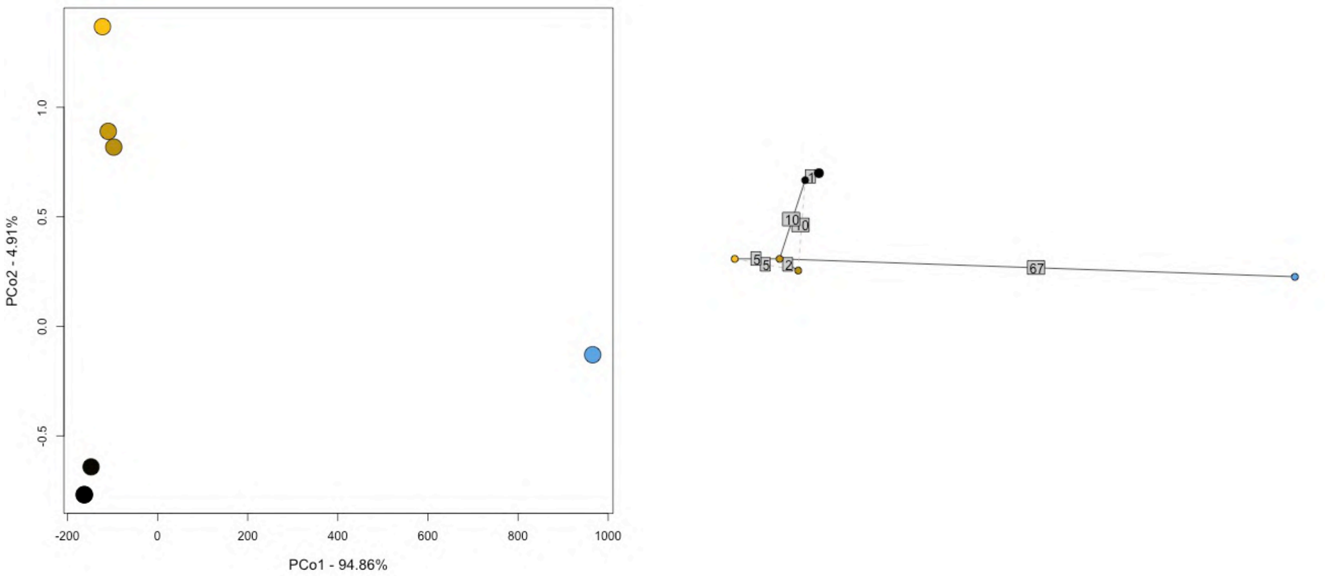

Figure 756: PCoA based on pairwise p-distances between *Temnothorax subtilis* sequences (left). Colours match a bidimensional colour space. Haplotype network of *Temnothorax subtilis* (right). Sequences > 599 bp: ID = 8, cf. = 0.

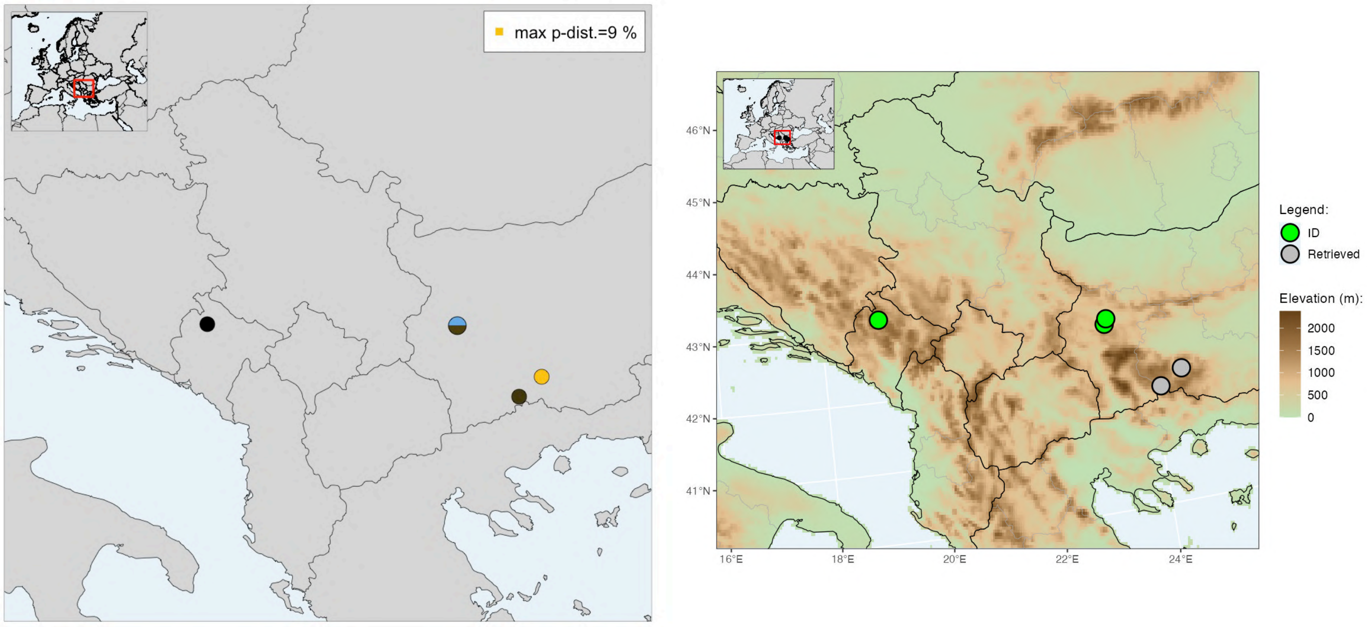

Figure 757: Genetic diversity map of *Temnothorax tergestinus* (Finzi, 1928). Nearby localities of sequenced specimens are merged in pies (left). Colours match the bidimensional colour space of the PCoA projection (Fig. 757 left) of p-dist between sequences (dots). Specimen identification (ID or cf.) and source (newly sequenced or retrieved) are represented by colours, while specimen attribute (terra typica, type locality, type specimen or faunistic novelty) is represented by the shape (right). Sequences: ID = 5, cf. = 0; maximum p-distance: strict = 9 %, less strict = 9 %.

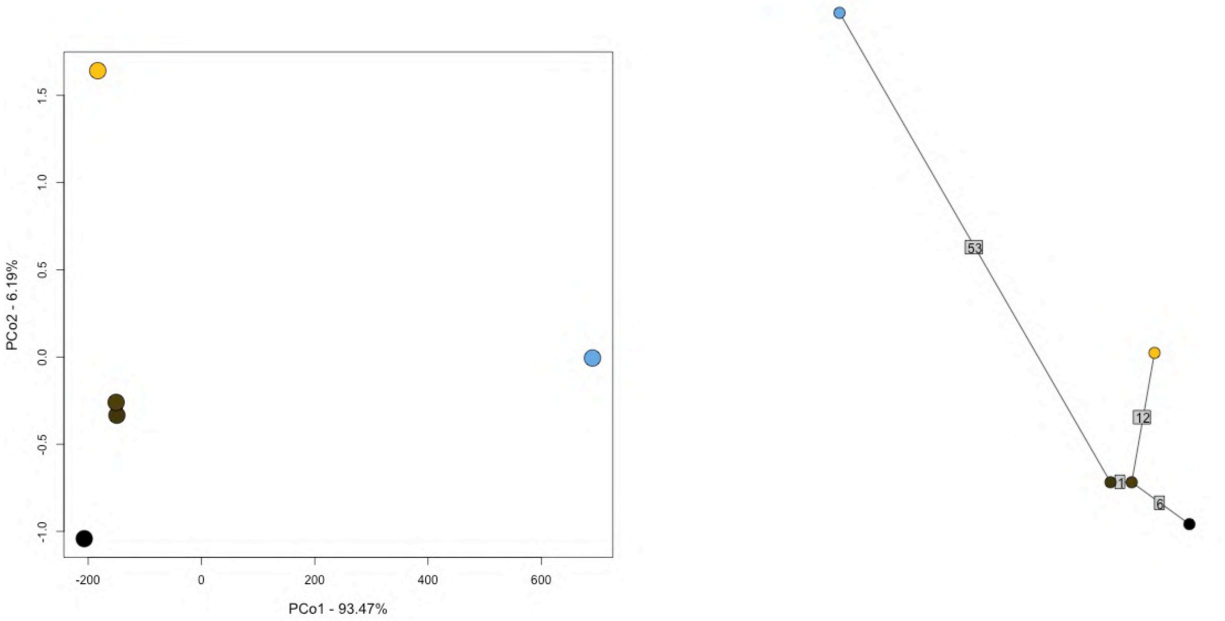

Figure 758: PCoA based on pairwise p-distances between *Temnothorax tergestinus* sequences (left). Colours match a bidimensional colour space. Haplotype network of *Temnothorax tergestinus* (right). Sequences > 599 bp: ID = 5, cf. = 0.

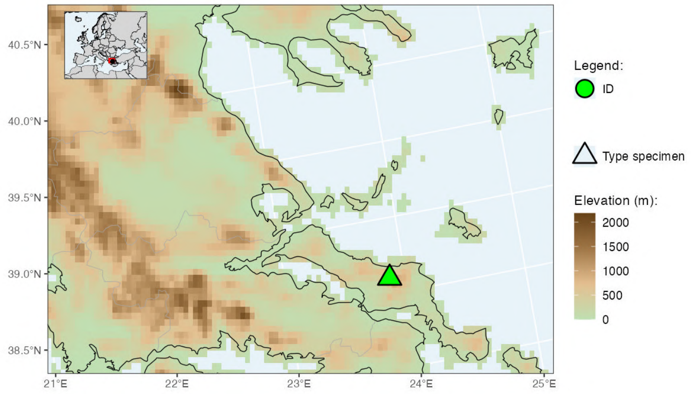

Figure 759: Map of *Temnothorax triangularis* Salata & Borowiec, 2019. Due to the presence of a single sequence, the genetic diversity map and the PCoA projection were not done. Specimen identification (ID or cf.) and source (newly sequenced or retrieved) are represented by colours, while specimen attribute (terra typica, type locality, type specimen or faunistic novelty) is represented by the shape. Sequences: ID = 1, cf. = 0; maximum p-distance: strict = NA, less strict = NA.

Haplotype network analysis of *Temnothorax triangularis* was not possible.

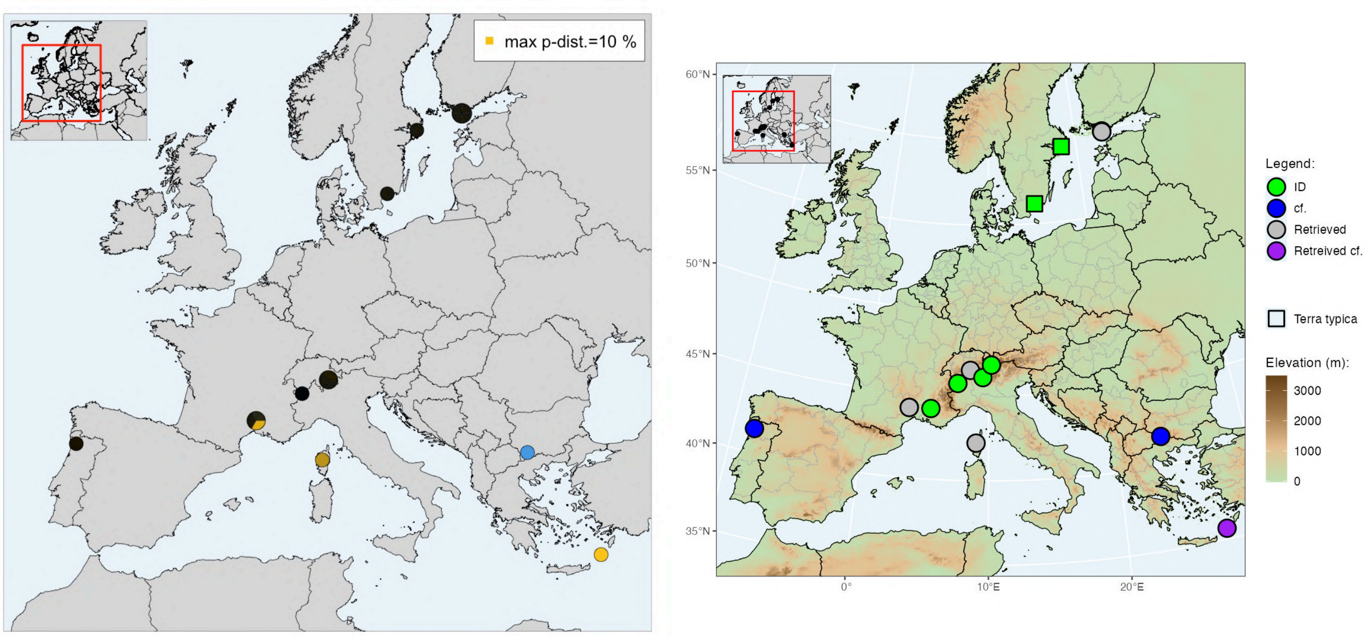

Figure 760: Genetic diversity map of *Temnothorax tuberum* (Fabricius, 1775). Nearby localities of sequenced specimens are merged in pies (left). Colours match the bidimensional colour space of the PCoA projection (Fig. 760 left) of p-dist between sequences (dots). Specimen identification (ID or cf.) and source (newly sequenced or retrieved) are represented by colours, while specimen attribute (terra typica, type locality, type specimen or faunistic novelty) is represented by the shape (right). Sequences: ID = 14, cf. = 3; maximum p-distance: strict = 3.8 %, less strict = 10 %.

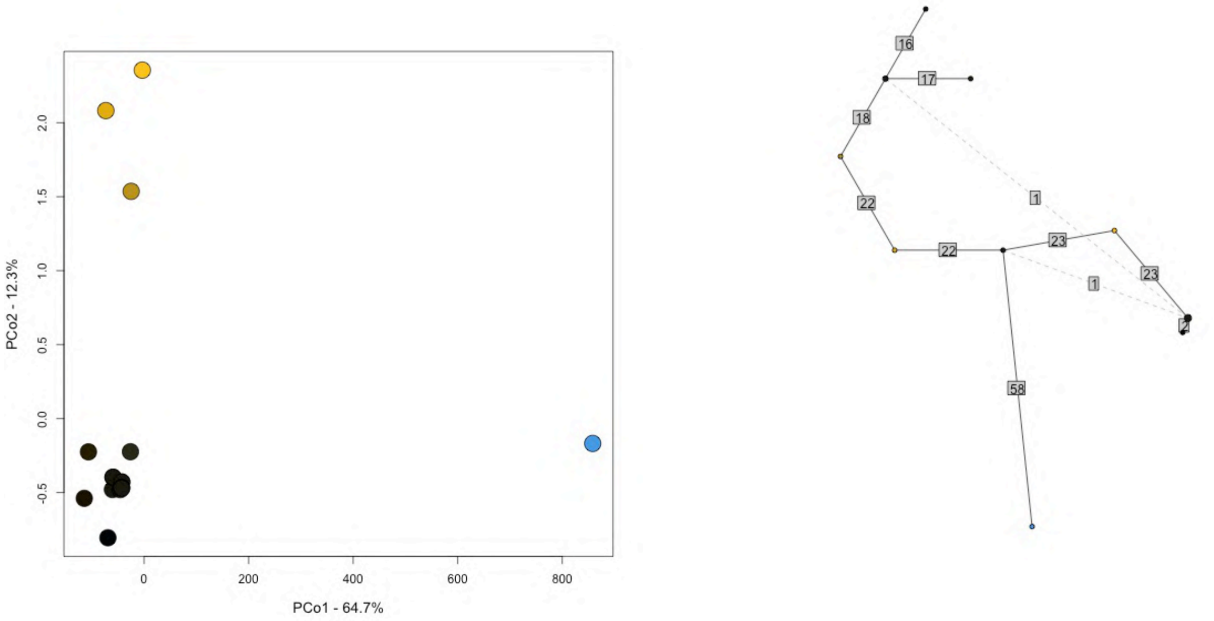

Figure 761: PCoA based on pairwise p-distances between *Temnothorax tuberum* sequences (left). Colours match a bidimensional colour space. Haplotype network of *Temnothorax tuberum* (right). Sequences > 599 bp: ID = 13, cf. = 3.

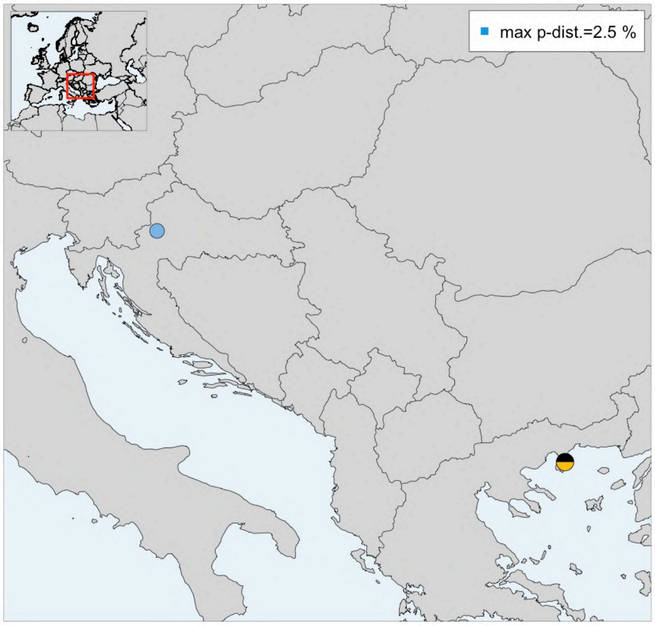

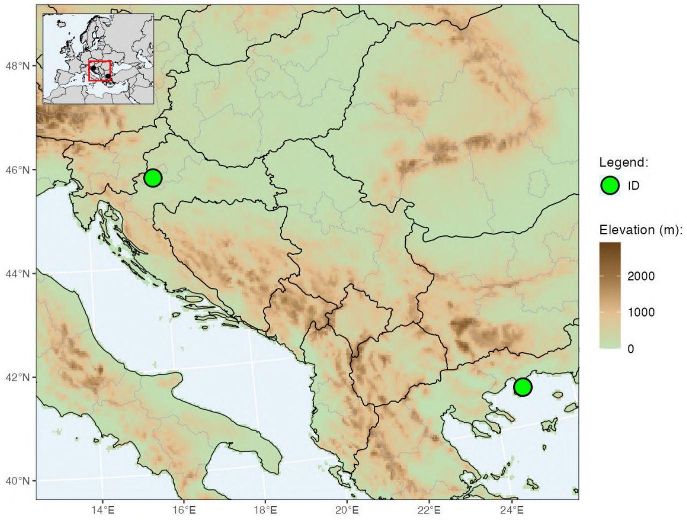

Figure 762: Genetic diversity map of *Temnothorax turcicus* (Santschi, 1934). Nearby localities of sequenced specimens are merged in pies (left). Colours match the bidimensional colour space of the PCoA projection (Fig. 762 left) of p-dist between sequences (dots). Specimen identification (ID or cf.) and source (newly sequenced or retrieved) are represented by colours, while specimen attribute (terra typica, type locality, type specimen or faunistic novelty) is represented by the shape (right). Sequences: ID = 3, cf. = 0; maximum p-distance: strict = 2.5 %, less strict = 2.5 %.

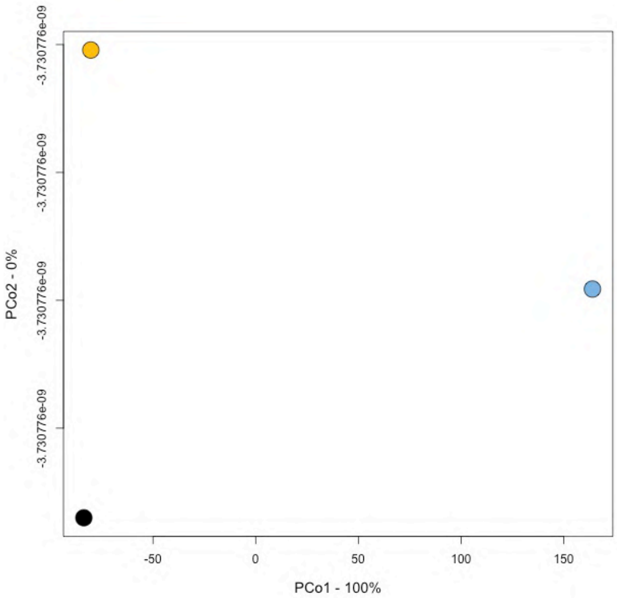

Figure 763: PCoA based on pairwise p-distances between *Temnothorax turcicus* sequences (left). Colours match a bidimensional colour space. Haplotype network analysis of *Temnothorax turcicus* was not possible.

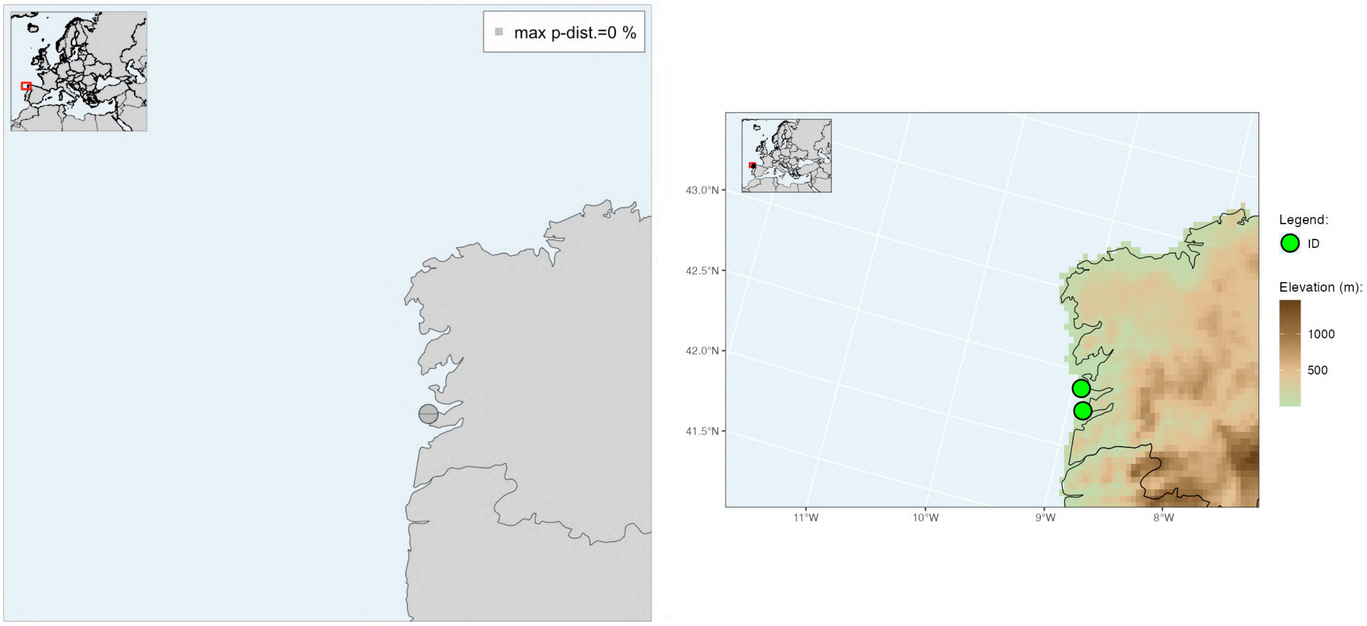

Figure 764: Genetic diversity map of *Temnothorax tyndalei* (Forel, 1909). PCoA projection was not done and therefore sequenced specimens in the genetic diversity map are coloured in gray (left). Specimen identification (ID or cf.) and source (newly sequenced or retrieved) are represented by colours, while specimen attribute (terra typica, type locality, type specimen or faunistic novelty) is represented by the shape (right). Sequences: ID = 2, cf. = 0; maximum p-distance: strict = NA, less strict = 0 %.

Haplotype network analysis of *Temnothorax tyndalei* was not possible.

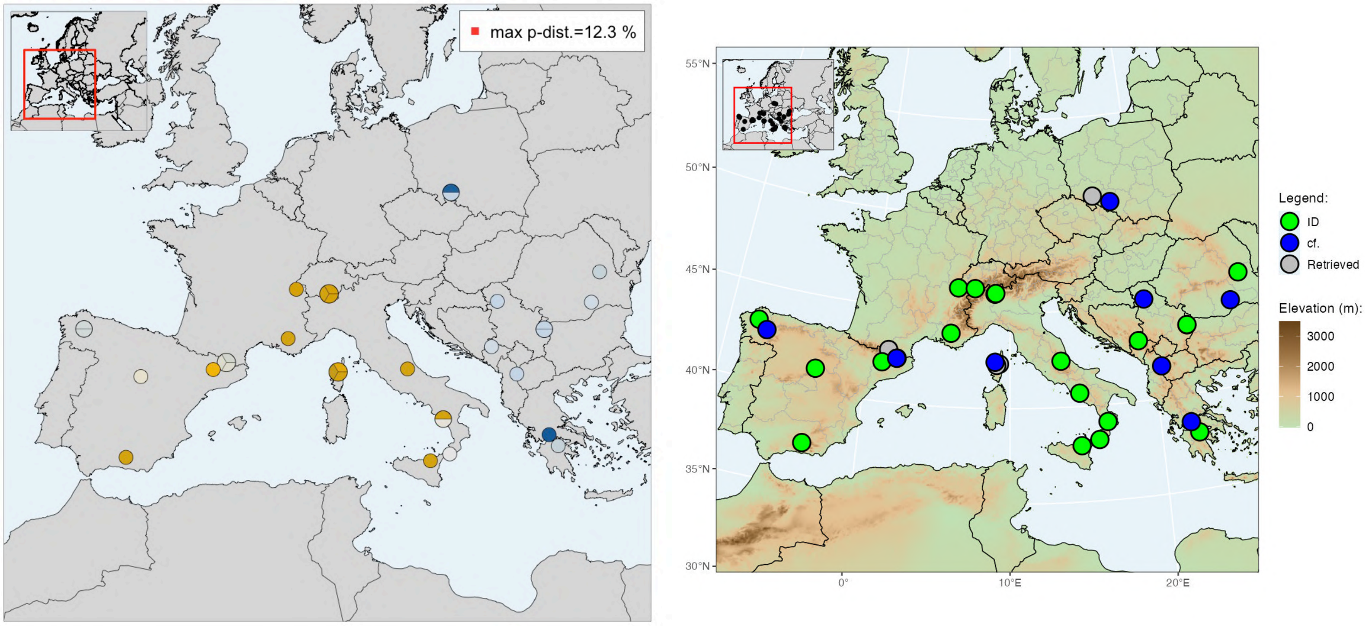

Figure 765: Genetic diversity map of *Temnothorax unifasciatus* (Latreille, 1798). Nearby localities of sequenced specimens are merged in pies (left). Colours match the bidimensional colour space of the PCoA projection (Fig. 765 left) of p-dist between sequences (dots). Specimen identification (ID or cf.) and source (newly sequenced or retrieved) are represented by colours, while specimen attribute (terra typica, type locality, type specimen or faunistic novelty) is represented by the shape (right). Sequences: ID = 23, cf. = 9; maximum p-distance: strict = 12.3 %, less strict = 12.3 %.

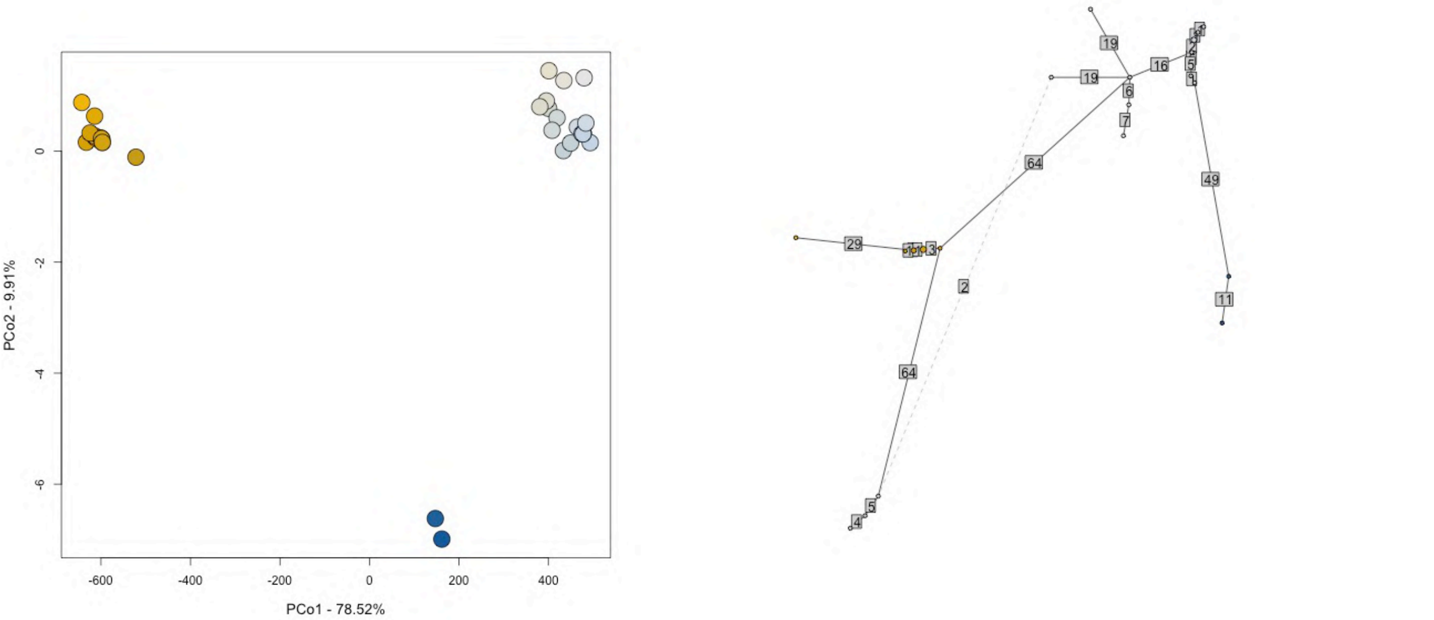

Figure 766: PCoA based on pairwise p-distances between *Temnothorax unifasciatus* sequences (left). Colours match a bidimensional colour space. Haplotype network of *Temnothorax unifasciatus* (right). Sequences > 599 bp: ID = 22, cf. = 9.

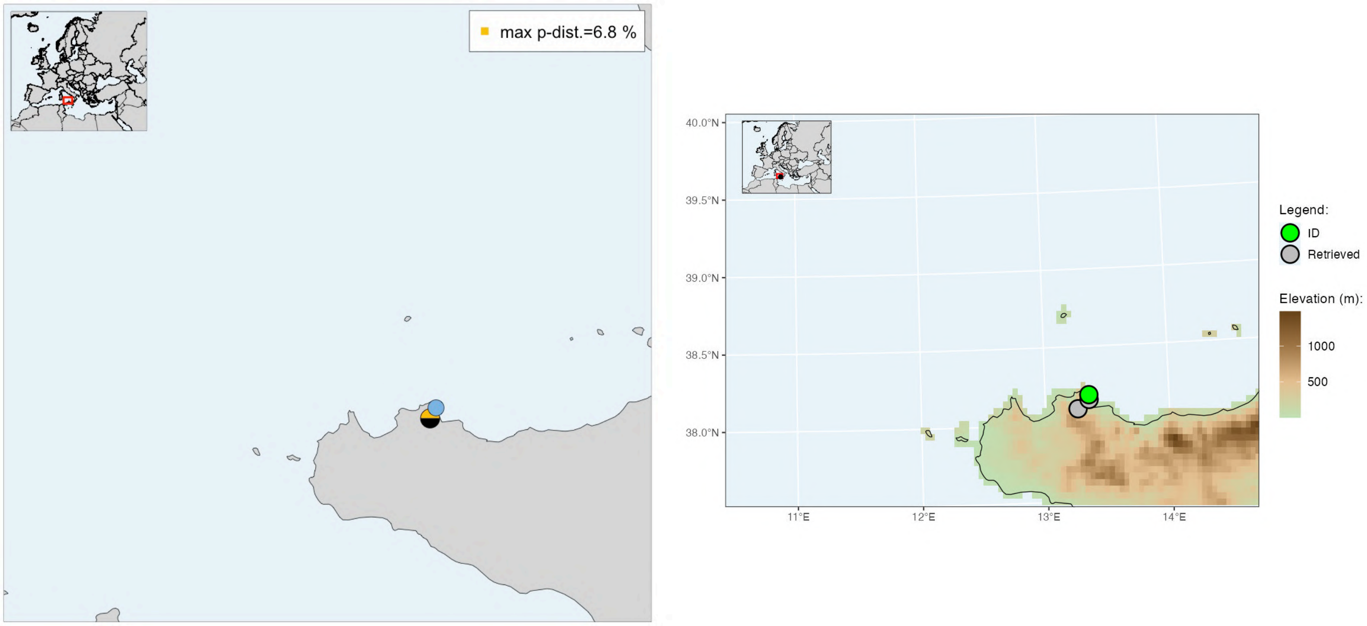

Figure 767: Genetic diversity map of *Temnothorax vivianoi* Schifani, Alicata & Prebus, 2022. Nearby localities of sequenced specimens are merged in pies (left). Colours match the bidimensional colour space of the PCoA projection (Fig. 767 left) of p-dist between sequences (dots). Specimen identification (ID or cf.) and source (newly sequenced or retrieved) are represented by colours, while specimen attribute (terra typica, type locality, type specimen or faunistic novelty) is represented by the shape (right). Sequences: ID = 3, cf. = 0; maximum p-distance: strict = NA, less strict = 6.8 %.

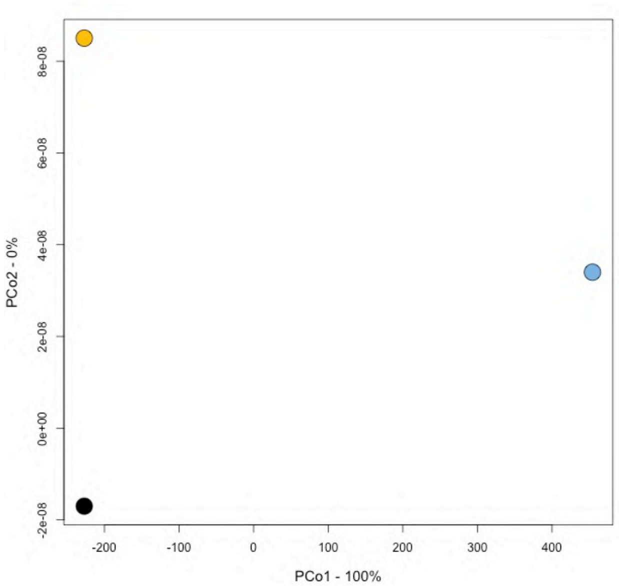

Figure 768: PCoA based on pairwise p-distances between *Temnothorax vivianoi* sequences (left). Colours match a bidimensional colour space. Haplotype network analysis of *Temnothorax vivianoi* was not possible.

Tetramorium

Tetramorium alpestre

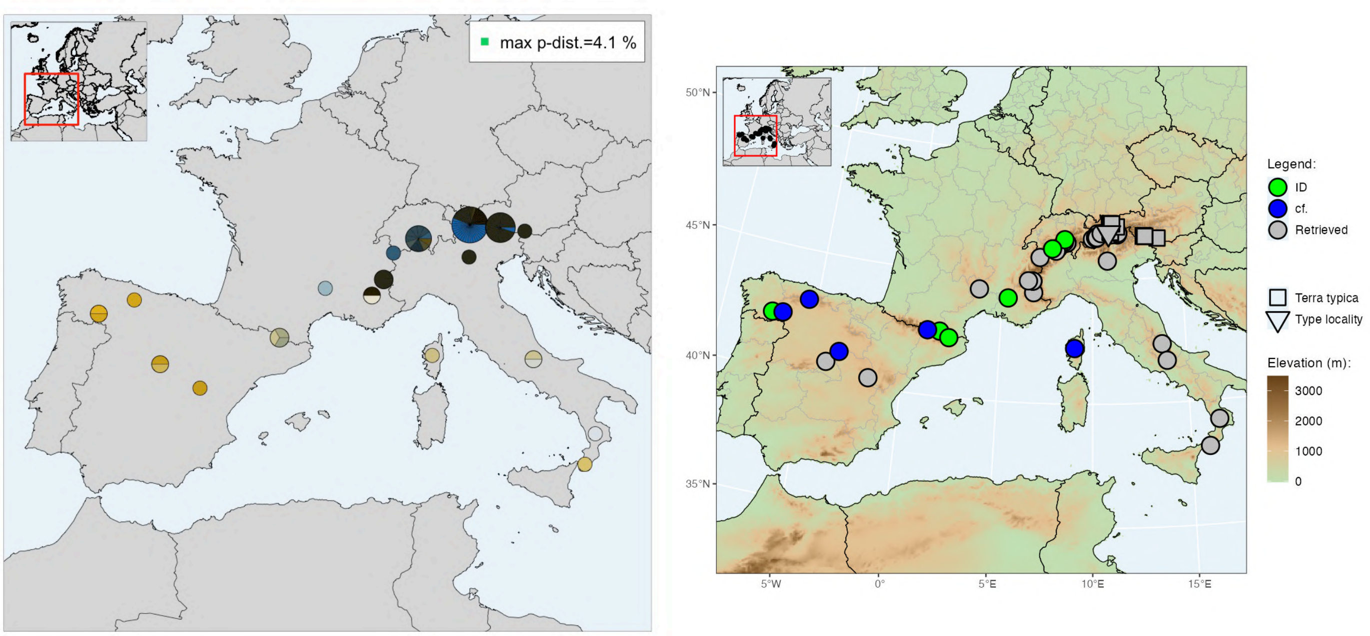

Figure 769: Genetic diversity map of *Tetramorium alpestre* Steiner, Schlick-Steiner & Seifert, 2010. Nearby localities of sequenced specimens are merged in pies (left). Colours match the bidimensional colour space of the PCoA projection (Fig. 769 left) of p-dist between sequences (dots). Specimen identification (ID or cf.) and source (newly sequenced or retrieved) are represented by colours, while specimen attribute (terra typica, type locality, type specimen or faunistic novelty) is represented by the shape (right). Sequences: ID = 89, cf. = 5; maximum p-distance: strict = 2.2 %, less strict = 4.1 %.

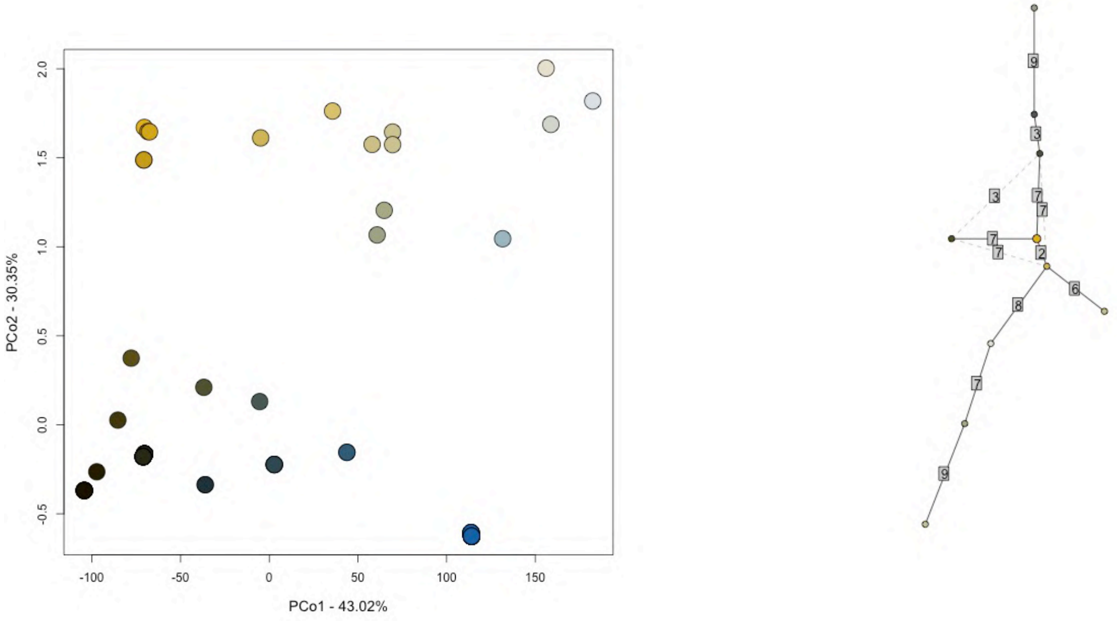

Figure 770: PCoA based on pairwise p-distances between *Tetramorium alpestre* sequences (left). Colours match a bidimensional colour space. Haplotype network of *Tetramorium alpestre* (right). Sequences > 599 bp: ID = 7, cf. = 5.

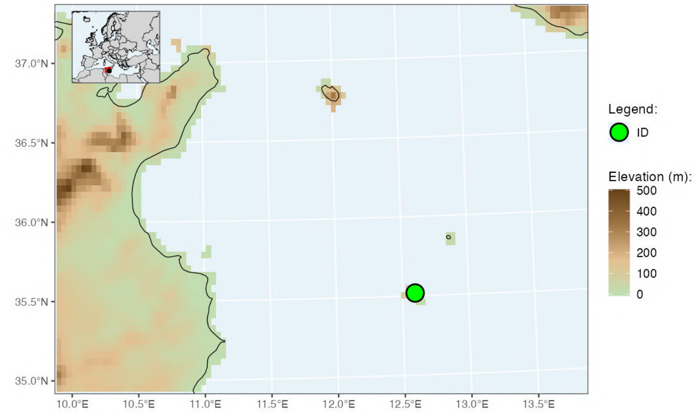

Figure 771: Map of *Tetramorium alternans* Santschi, 1929. Due to the presence of a single sequence, the genetic diversity map and the PCoA projection were not done. Specimen identification (ID or cf.) and source (newly sequenced or retrieved) are represented by colours, while specimen attribute (terra typica, type locality, type specimen or faunistic novelty) is represented by the shape. Sequences: ID = 1, cf. = 0; maximum p-distance: strict = NA, less strict = NA.

Haplotype network analysis of *Tetramorium alternans* was not possible.

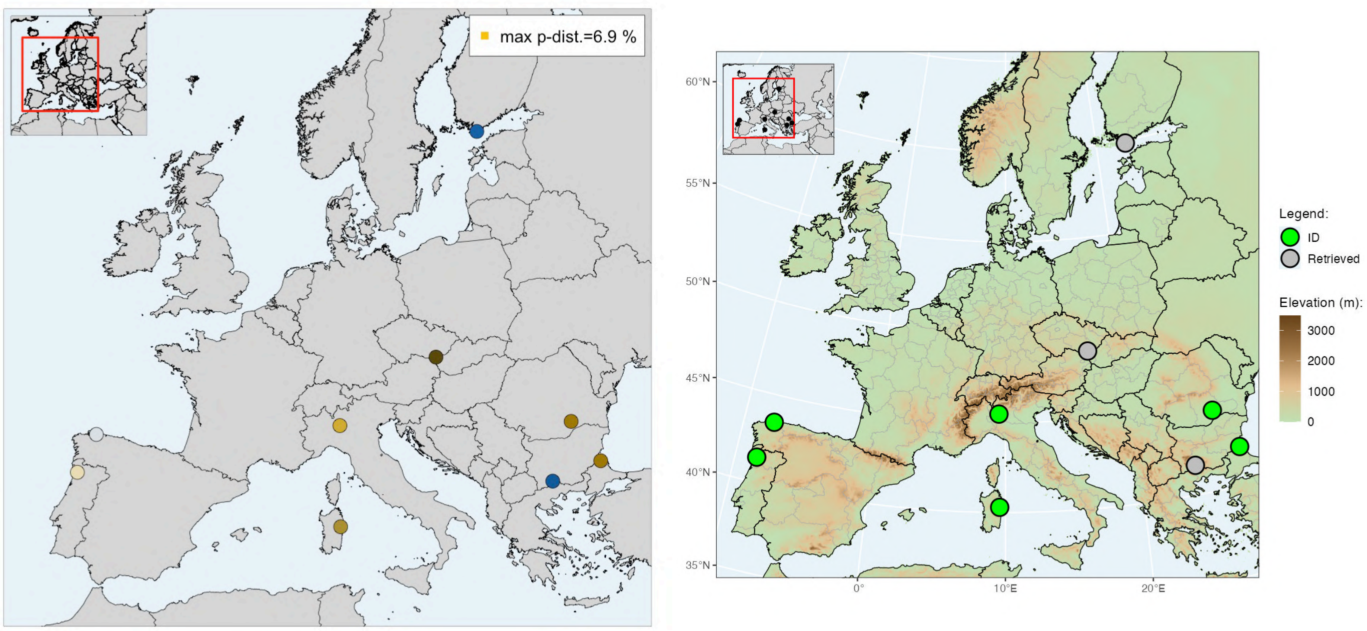

Figure 772: Genetic diversity map of *Tetramorium atratulum* (Schenck, 1852). Nearby localities of sequenced specimens are merged in pies (left). Colours match the bidimensional colour space of the PCoA projection (Fig. 772 left) of p-dist between sequences (dots). Specimen identification (ID or cf.) and source (newly sequenced or retrieved) are represented by colours, while specimen attribute (terra typica, type locality, type specimen or faunistic novelty) is represented by the shape (right). Sequences: ID = 9, cf. = 0; maximum p-distance: strict = 6.9 %, less strict = 6.9 %.

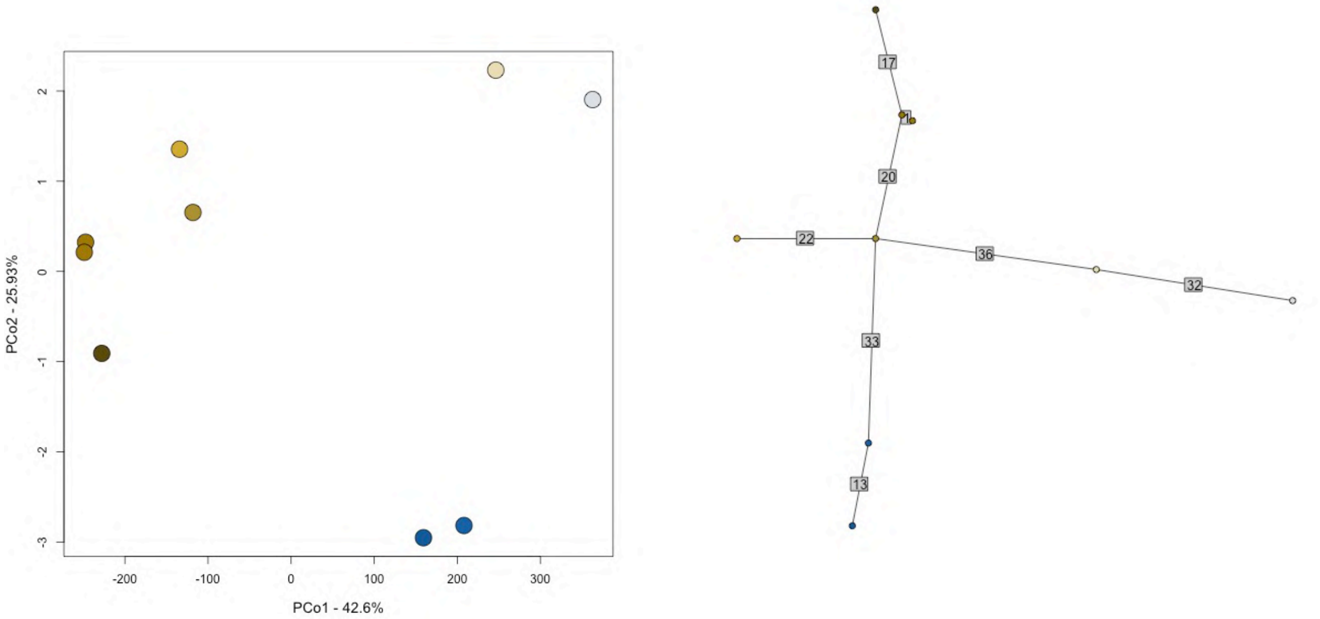

Figure 773: PCoA based on pairwise p-distances between *Tetramorium atratulum* sequences (left). Colours match a bidimensional colour space. Haplotype network of *Tetramorium atratulum* (right). Sequences > 599 bp: ID = 9, cf. = 0.

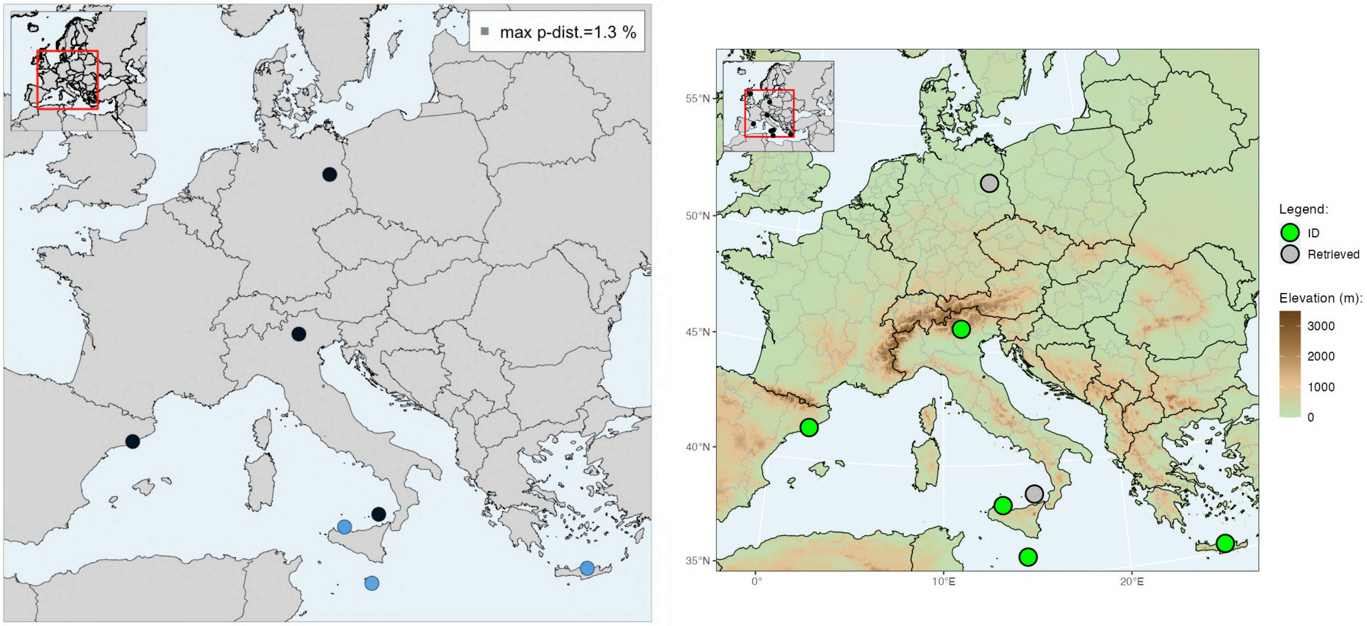

Figure 774: Genetic diversity map of *Tetramorium bicarinatum* (Nylander, 1846). Nearby localities of sequenced specimens are merged in pies (left). Colours match the bidimensional colour space of the PCoA projection (Fig. 774 left) of p-dist between sequences (dots). Specimen identification (ID or cf.) and source (newly sequenced or retrieved) are represented by colours, while specimen attribute (terra typica, type locality, type specimen or faunistic novelty) is represented by the shape (right). Sequences: ID = 8, cf. = 0; maximum p-distance: strict = 1.1 %, less strict = 1.3 %.

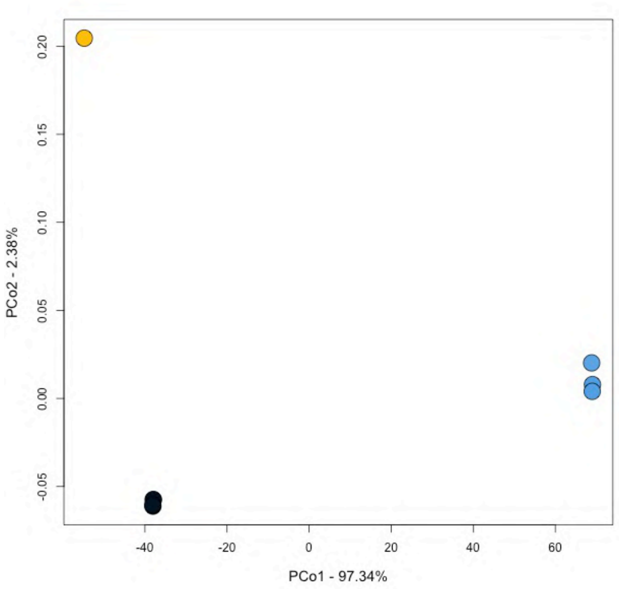

Figure 775: PCoA based on pairwise p-distances between *Tetramorium bicarinatum* sequences (left). Colours match a bidimensional colour space. Haplotype network analysis of *Tetramorium bicarinatum* was not possible.

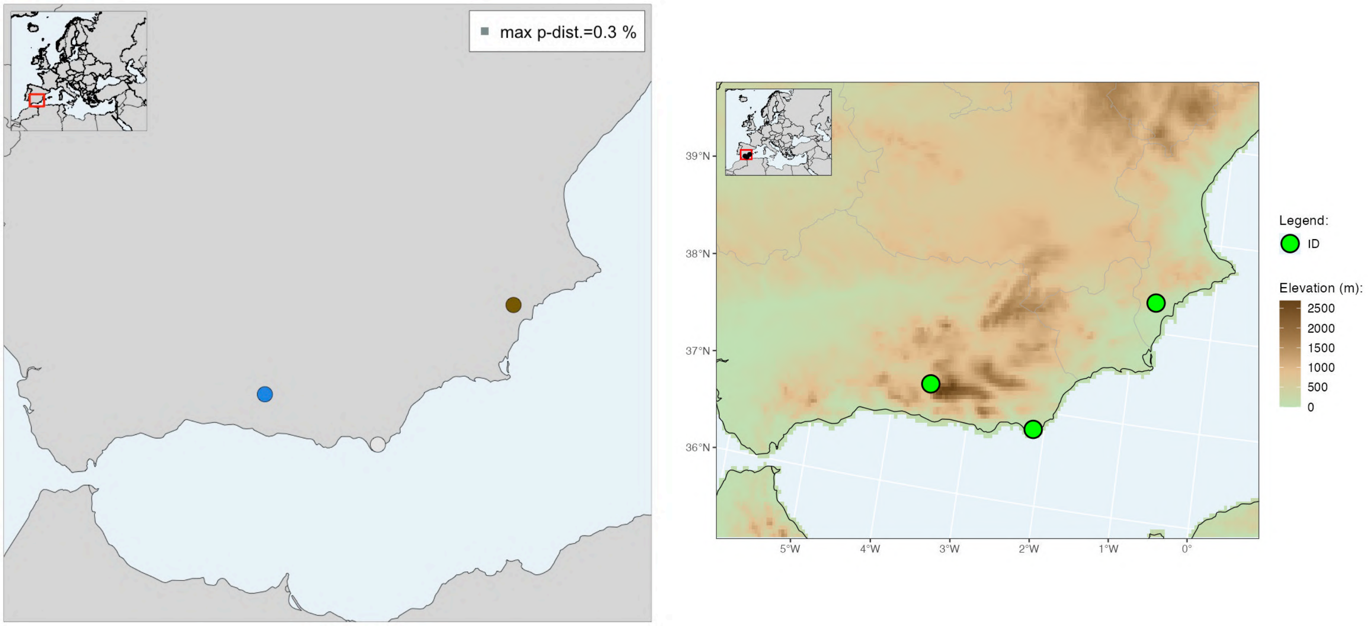

Figure 776: Genetic diversity map of *Tetramorium biskrense* Forel, 1904. Nearby localities of sequenced specimens are merged in pies (left). Colours match the bidimensional colour space of the PCoA projection (Fig. 776 left) of p-dist between sequences (dots). Specimen identification (ID or cf.) and source (newly sequenced or retrieved) are represented by colours, while specimen attribute (terra typica, type locality, type specimen or faunistic novelty) is represented by the shape (right). Sequences: ID = 3, cf. = 0; maximum p-distance: strict = 0.3 %, less strict = 0.3 %.

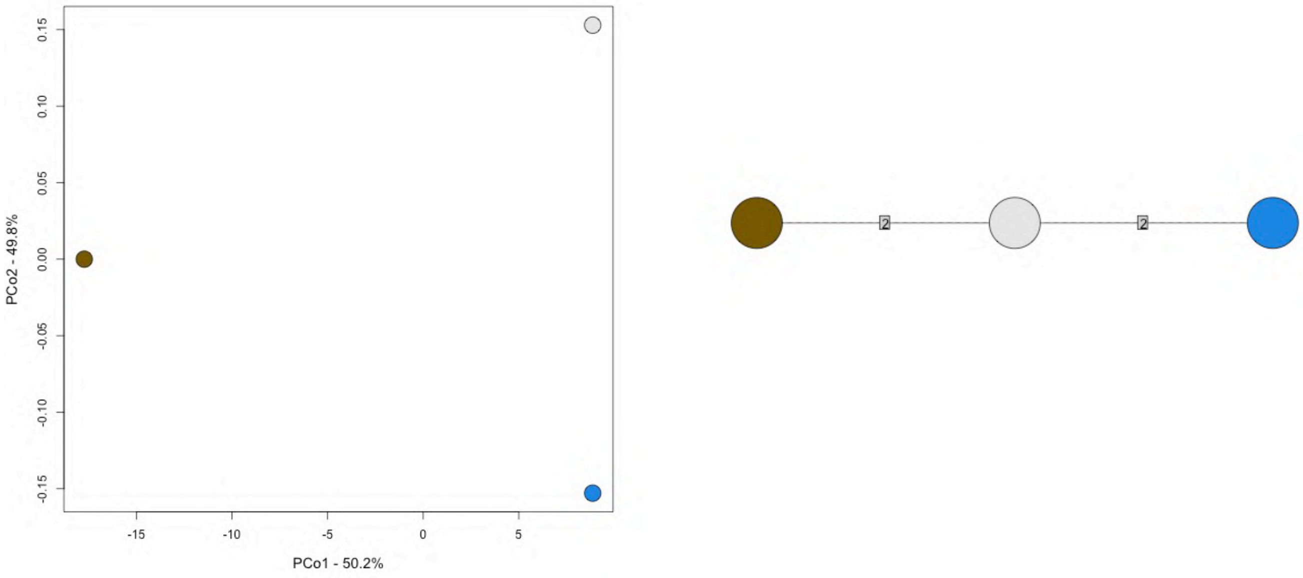

Figure 777: PCoA based on pairwise p-distances between *Tetramorium biskrense* sequences (left). Colours match a bidimensional colour space. Haplotype network of *Tetramorium biskrense* (right). Sequences > 599 bp: ID = 3, cf. = 0.

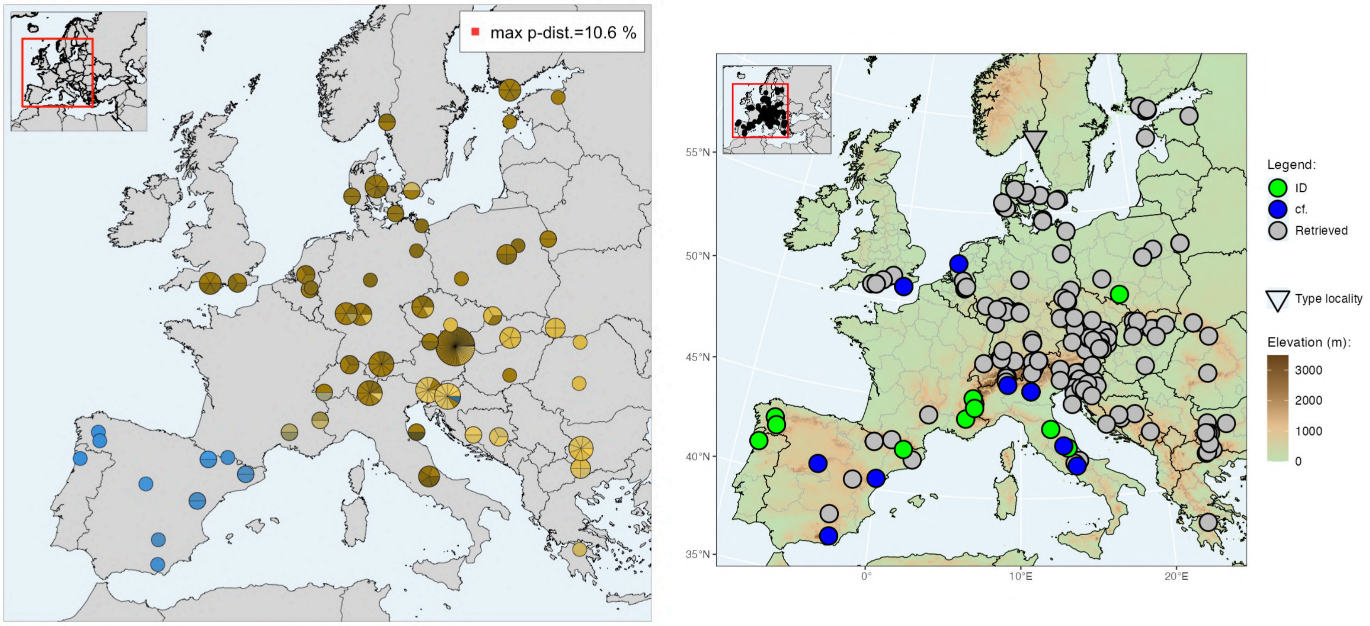

Figure 778: Genetic diversity map of *Tetramorium caespitum* (Linnaeus, 1758). Nearby localities of sequenced specimens are merged in pies (left). Colours match the bidimensional colour space of the PCoA projection (Fig. 778 left) of p-dist between sequences (dots). Specimen identification (ID or cf.) and source (newly sequenced or retrieved) are represented by colours, while specimen attribute (terra typica, type locality, type specimen or faunistic novelty) is represented by the shape (right). Sequences: ID = 225, cf. = 10; maximum p-distance: strict = 6.2 %, less strict = 10.6 %.

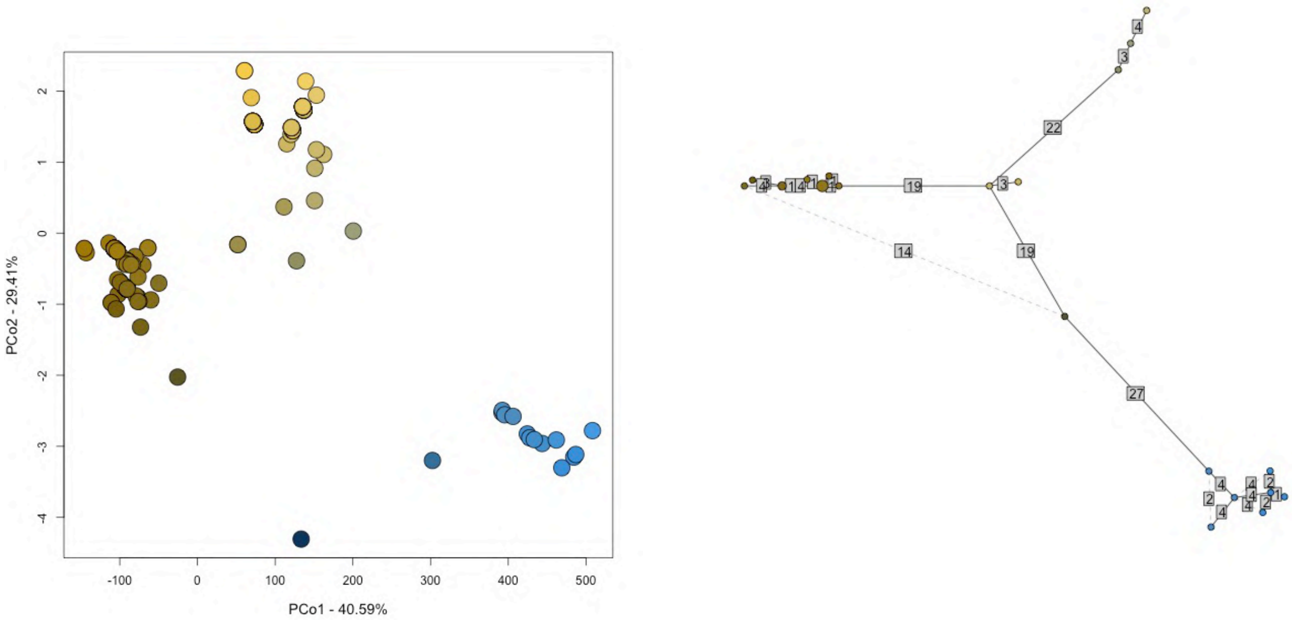

Figure 779: PCoA based on pairwise p-distances between *Tetramorium caespitum* sequences (left). Colours match a bidimensional colour space. Haplotype network of *Tetramorium caespitum* (right). Sequences > 599 bp: ID = 22, cf. = 10.

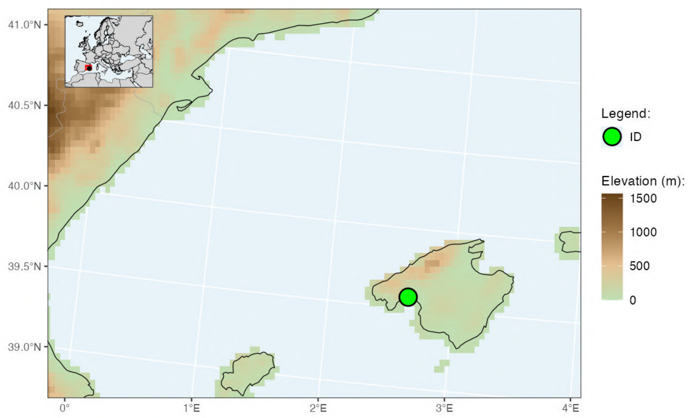

Figure 780: Map of *Tetramorium caldarium* (Roger, 1857). Due to the presence of a single sequence, the genetic diversity map and the PCoA projection were not done. Specimen identification (ID or cf.) and source (newly sequenced or retrieved) are represented by colours, while specimen attribute (terra typica, type locality, type specimen or faunistic novelty) is represented by the shape. Sequences: ID = 1, cf. = 0; maximum p-distance: strict = NA, less strict = NA.

Haplotype network analysis of *Tetramorium caldarium* was not possible.

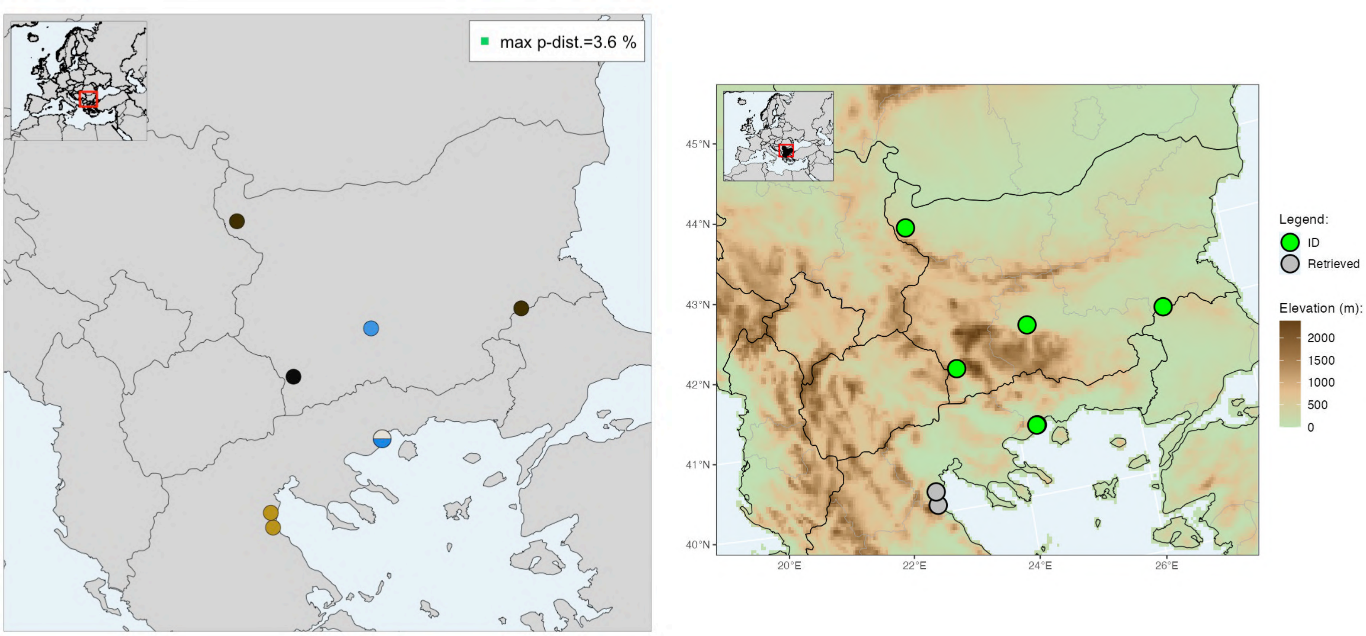

Figure 781: Genetic diversity map of *Tetramorium chefketi* Forel, 1911. Nearby localities of sequenced specimens are merged in pies (left). Colours match the bidimensional colour space of the PCoA projection (Fig. 781 left) of p-dist between sequences (dots). Specimen identification (ID or cf.) and source (newly sequenced or retrieved) are represented by colours, while specimen attribute (terra typica, type locality, type specimen or faunistic novelty) is represented by the shape (right). Sequences: ID = 8, cf. = 0; maximum p-distance: strict = 3.5 %, less strict = 3.6 %.

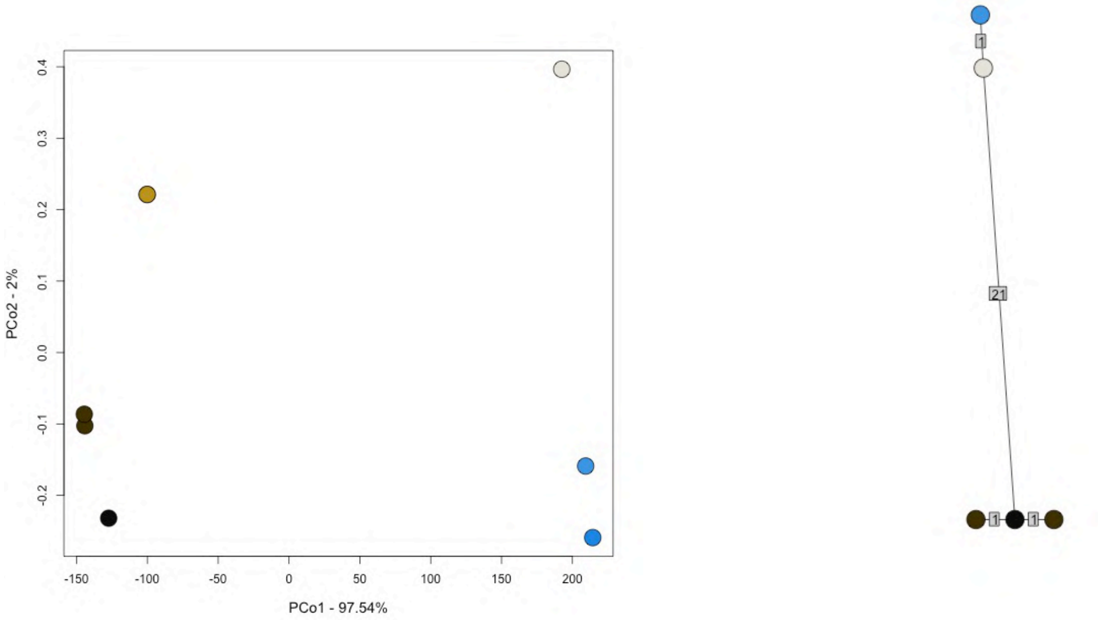

Figure 782: PCoA based on pairwise p-distances between *Tetramorium chefketi* sequences (left). Colours match a bidimensional colour space. Haplotype network of *Tetramorium chefketi* (right). Sequences > 599 bp: ID = 5, cf. = 0.

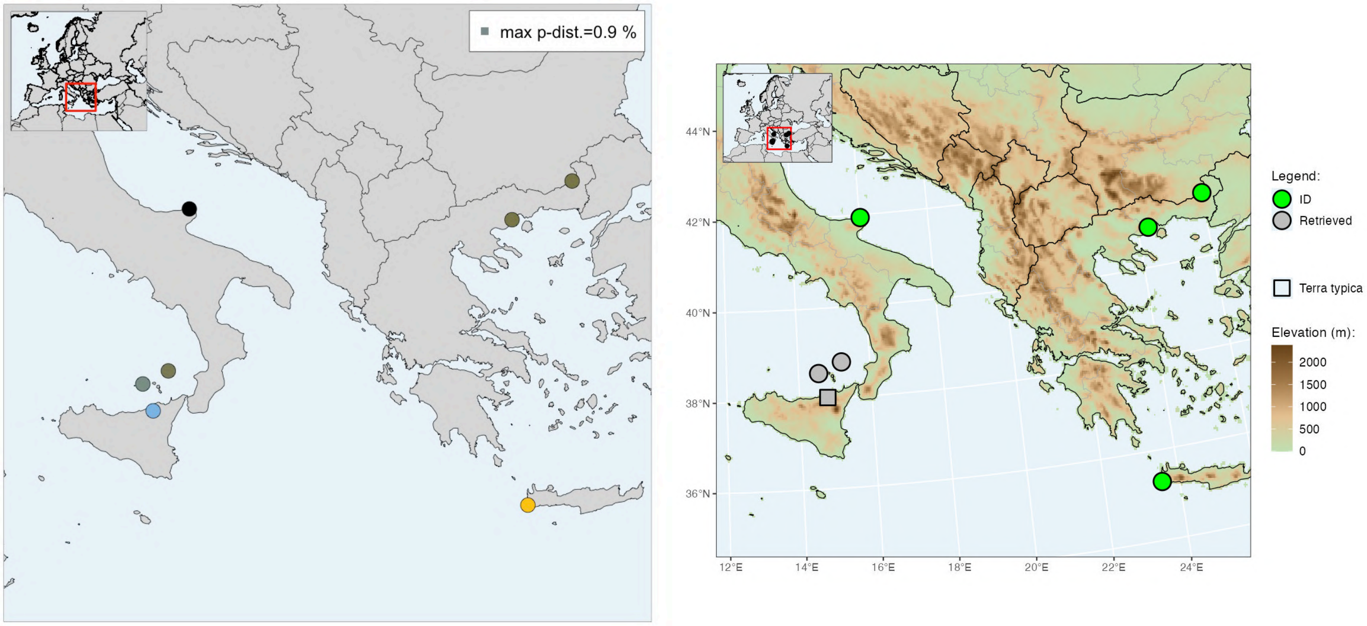

Figure 783: Genetic diversity map of *Tetramorium diomedeum* Emery, 1908. Nearby localities of sequenced specimens are merged in pies (left). Colours match the bidimensional colour space of the PCoA projection (Fig. 783 left) of p-dist between sequences (dots). Specimen identification (ID or cf.) and source (newly sequenced or retrieved) are represented by colours, while specimen attribute (terra typica, type locality, type specimen or faunistic novelty) is represented by the shape (right). Sequences: ID = 7, cf. = 0; maximum p-distance: strict = 0.9 %, less strict = 0.9 %.

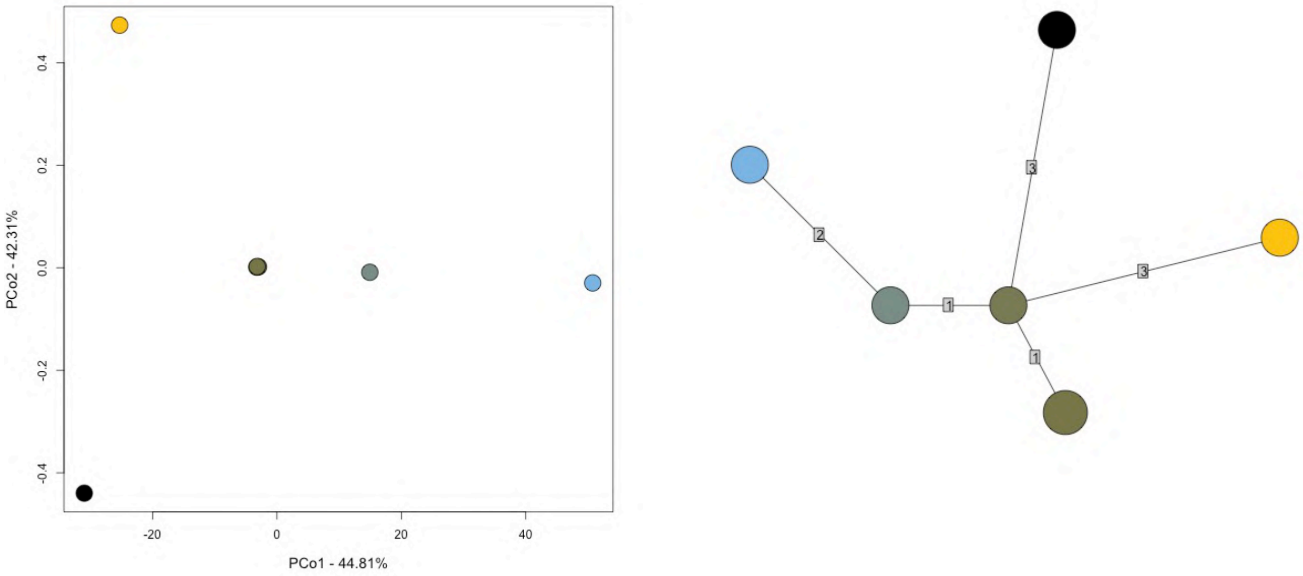

Figure 784: PCoA based on pairwise p-distances between *Tetramorium diomedeum* sequences (left). Colours match a bidimensional colour space. Haplotype network of *Tetramorium diomedeum* (right). Sequences > 599 bp: ID = 7, cf. = 0.

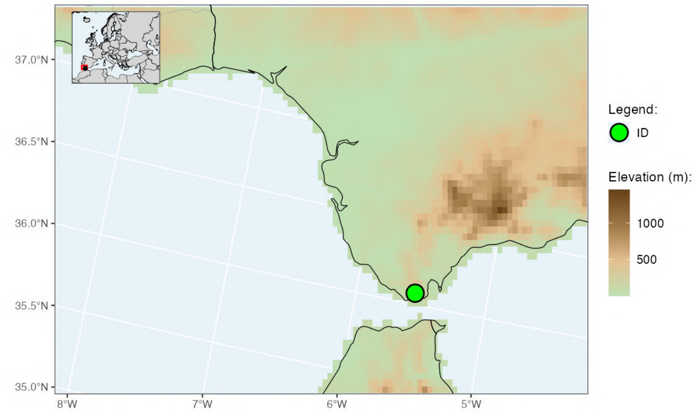

Figure 785: Map of *Tetramorium exasperatum* Emery, 1891. Due to the presence of a single sequence, the genetic diversity map and the PCoA projection were not done. Specimen identification (ID or cf.) and source (newly sequenced or retrieved) are represented by colours, while specimen attribute (terra typica, type locality, type specimen or faunistic novelty) is represented by the shape. Sequences: ID = 1, cf. = 0; maximum p-distance: strict = NA, less strict = NA.

Haplotype network analysis of *Tetramorium exasperatum* was not possible.

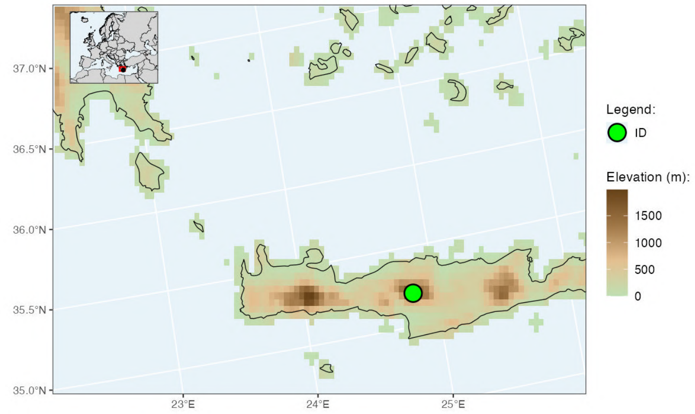

Figure 786: Map of *Tetramorium ferox* Ruzsky, 1903. Due to the presence of a single sequence, the genetic diversity map and the PCoA projection were not done. Specimen identification (ID or cf.) and source (newly sequenced or retrieved) are represented by colours, while specimen attribute (terra typica, type locality, type specimen or faunistic novelty) is represented by the shape. Sequences: ID = 1, cf. = 0; maximum p-distance: strict = NA, less strict = NA.

Haplotype network analysis of *Tetramorium ferox* was not possible.

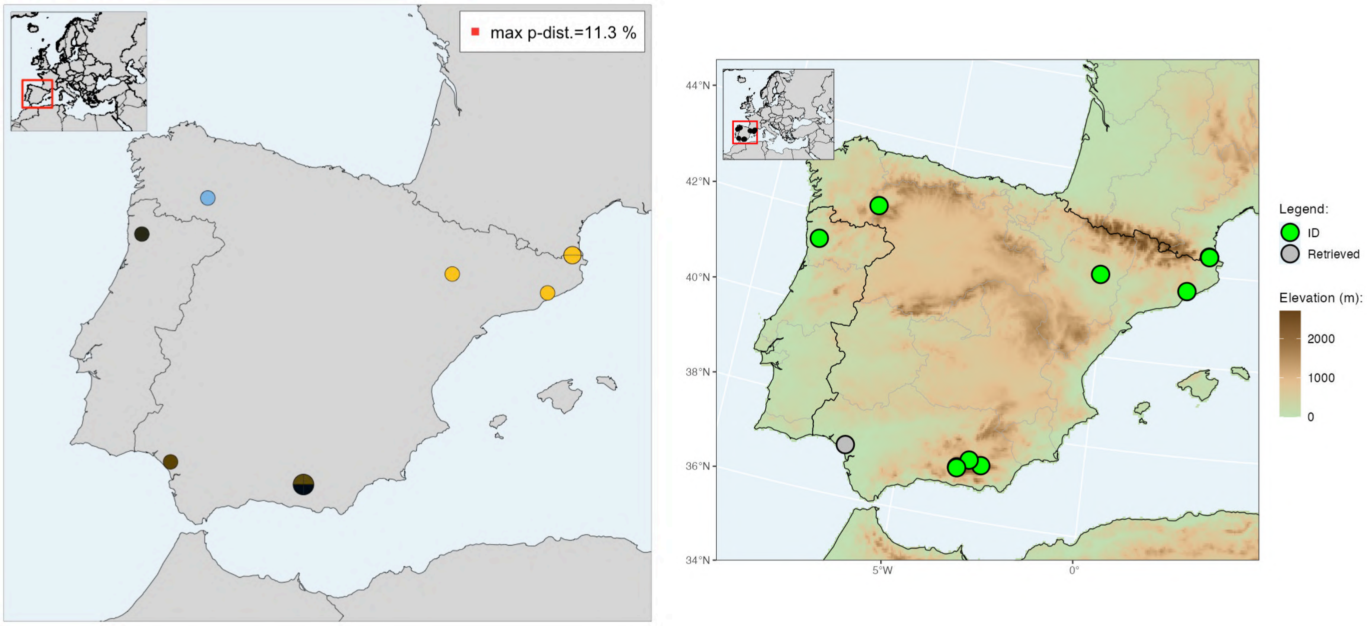

Figure 787: Genetic diversity map of *Tetramorium forte* Forel, 1904. Nearby localities of sequenced specimens are merged in pies (left). Colours match the bidimensional colour space of the PCoA projection (Fig. 787 left) of p-dist between sequences (dots). Specimen identification (ID or cf.) and source (newly sequenced or retrieved) are represented by colours, while specimen attribute (terra typica, type locality, type specimen or faunistic novelty) is represented by the shape (right). Sequences: ID = 11, cf. = 0; maximum p-distance: strict = 10.6 %, less strict = 11.3 %.

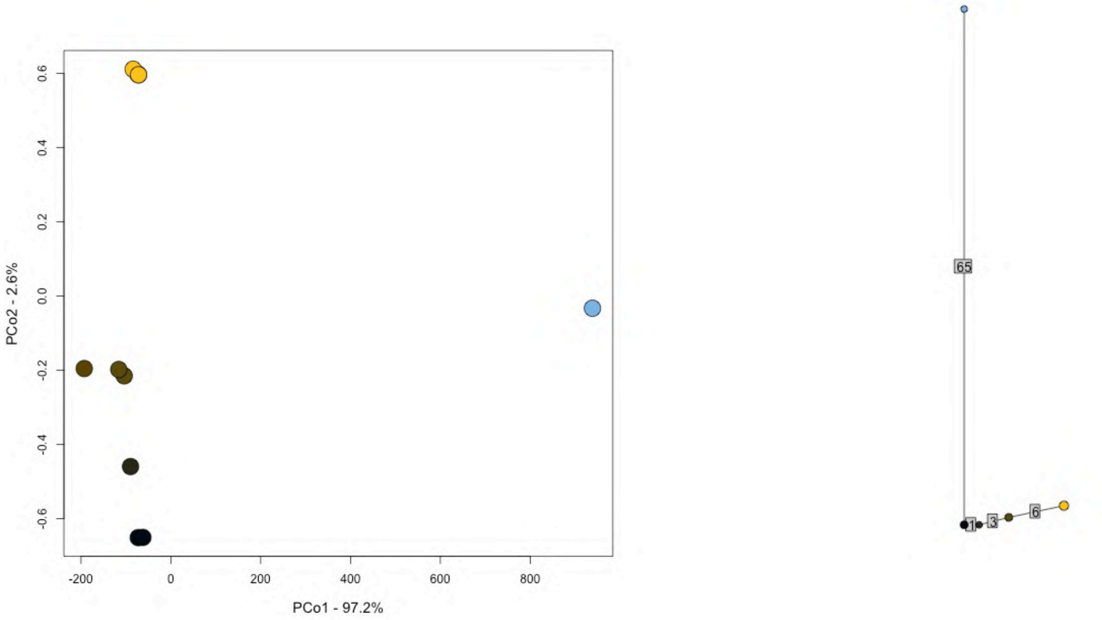

Figure 788: PCoA based on pairwise p-distances between *Tetramorium forte* sequences (left). Colours match a bidimensional colour space. Haplotype network of *Tetramorium forte* (right). Sequences > 599 bp: ID = 10, cf. = 0.

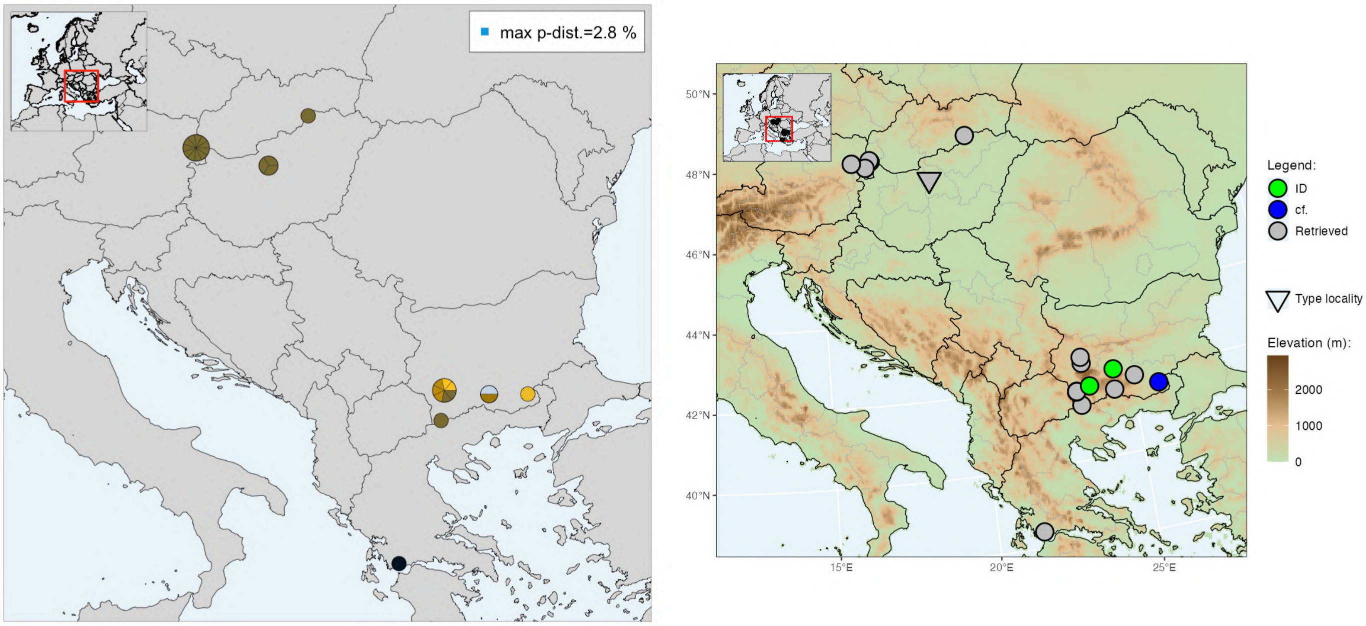

Figure 789: Genetic diversity map of *Tetramorium hungaricum* Röszler, 1935. Nearby localities of sequenced specimens are merged in pies (left). Colours match the bidimensional colour space of the PCoA projection (Fig. 789 left) of p-dist between sequences (dots). Specimen identification (ID or cf.) and source (newly sequenced or retrieved) are represented by colours, while specimen attribute (terra typica, type locality, type specimen or faunistic novelty) is represented by the shape (right). Sequences: ID = 26, cf. = 1; maximum p-distance: strict = 2.5 %, less strict = 2.8 %.

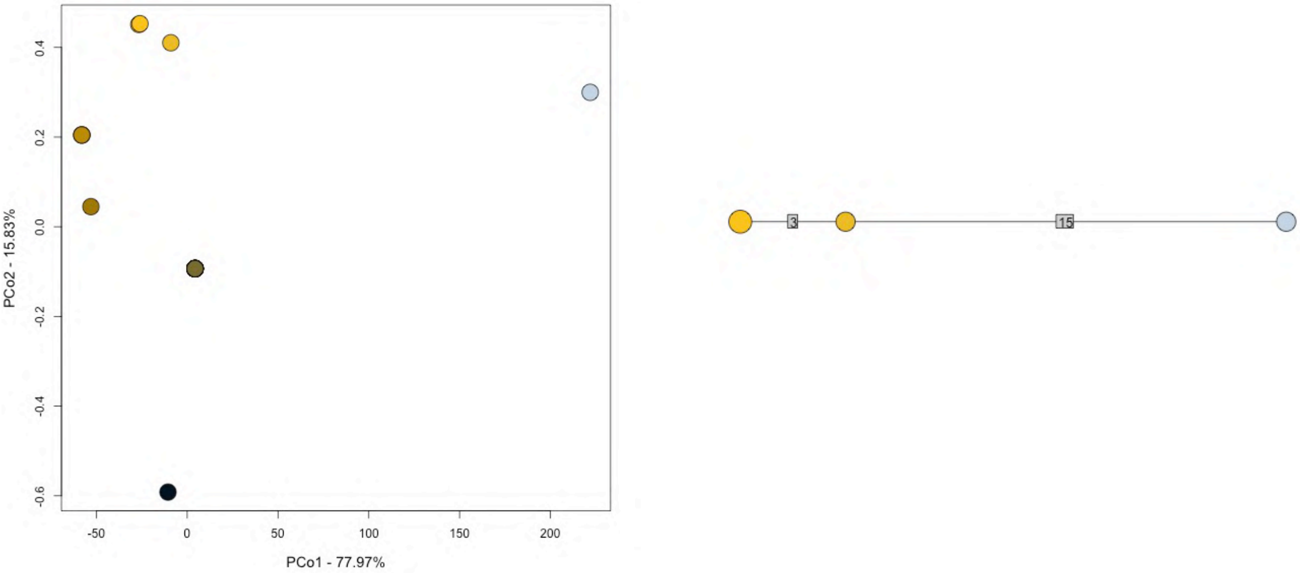

Figure 790: PCoA based on pairwise p-distances between *Tetramorium hungaricum* sequences (left). Colours match a bidimensional colour space. Haplotype network of *Tetramorium hungaricum* (right). Sequences > 599 bp: ID = 3, cf. = 1.

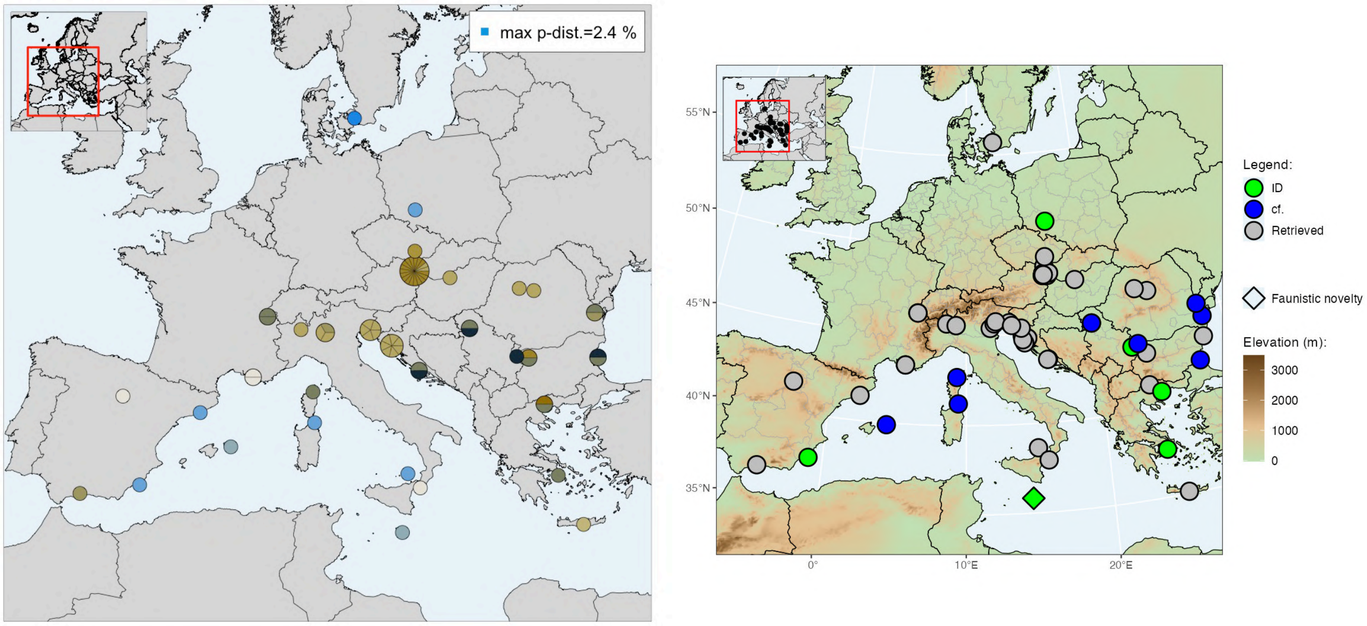

Figure 791: Genetic diversity map of *Tetramorium immigrans* Santschi, 1927. Nearby localities of sequenced specimens are merged in pies (left). Colours match the bidimensional colour space of the PCoA projection (Fig. 791 left) of p-dist between sequences (dots). Specimen identification (ID or cf.) and source (newly sequenced or retrieved) are represented by colours, while specimen attribute (terra typica, type locality, type specimen or faunistic novelty) is represented by the shape (right). Sequences: ID = 64, cf. = 9; maximum p-distance: strict = 1.4 %, less strict = 2.4 %.

The species is reported for the first time in Malta.

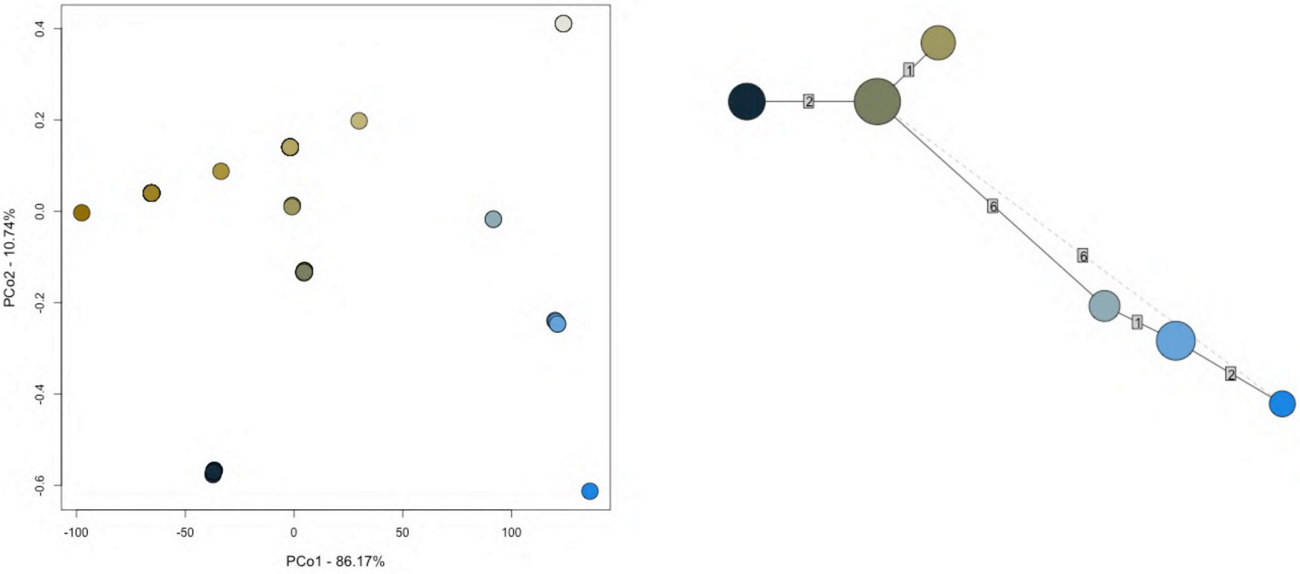

Figure 792: PCoA based on pairwise p-distances between *Tetramorium immigrans* sequences (left). Colours match a bidimensional colour space. Haplotype network of *Tetramorium immigrans* (right). Sequences > 599 bp: ID = 17, cf. = 9.

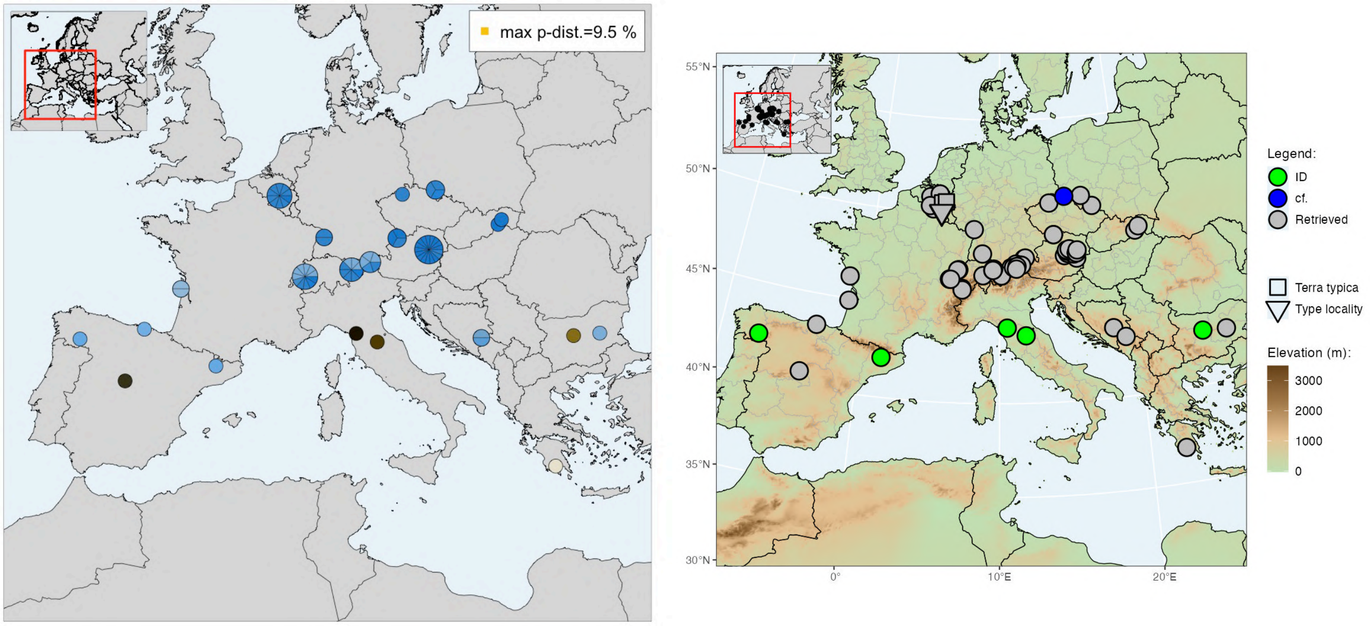

Figure 793: Genetic diversity map of *Tetramorium impurum* (Foerster, 1850). Nearby localities of sequenced specimens are merged in pies (left). Colours match the bidimensional colour space of the PCoA projection (Fig. 793 left) of p-dist between sequences (dots). Specimen identification (ID or cf.) and source (newly sequenced or retrieved) are represented by colours, while specimen attribute (terra typica, type locality, type specimen or faunistic novelty) is represented by the shape (right). Sequences: ID = 74, cf. = 1; maximum p-distance: strict = 9.5 %, less strict = 9.5 %.

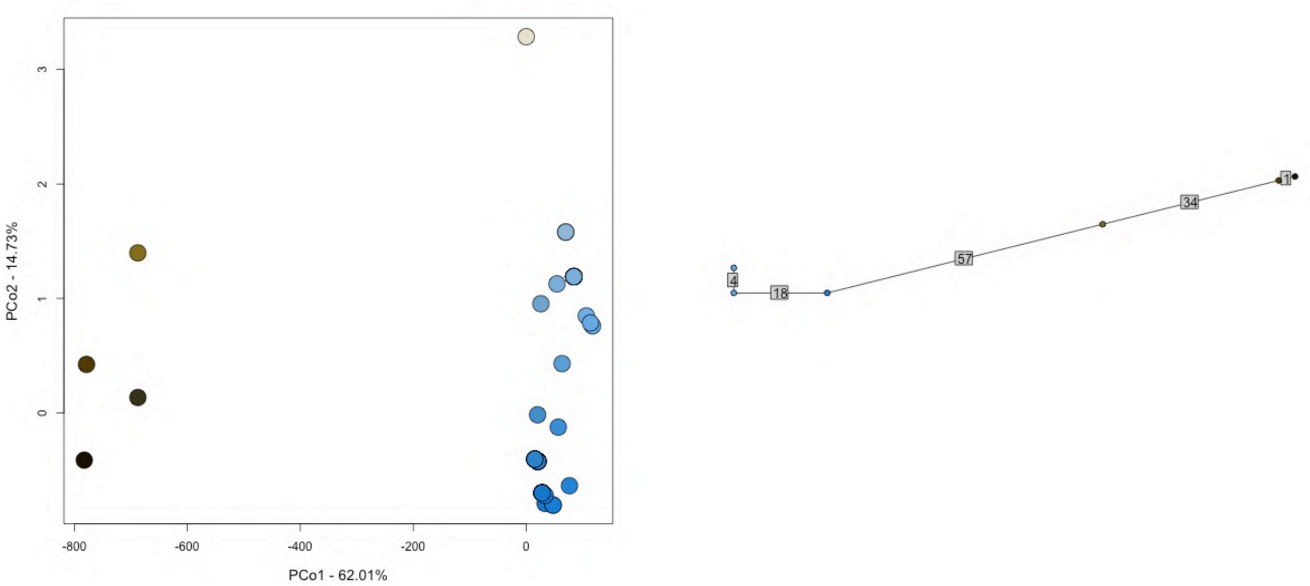

Figure 794: PCoA based on pairwise p-distances between *Tetramorium impurum* sequences (left). Colours match a bidimensional colour space. Haplotype network of *Tetramorium impurum* (right). Sequences > 599 bp: ID = 5, cf. = 1.

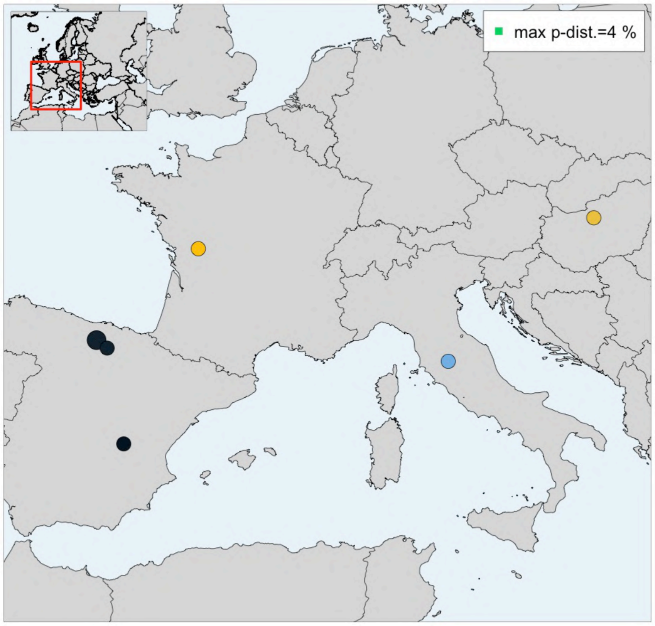

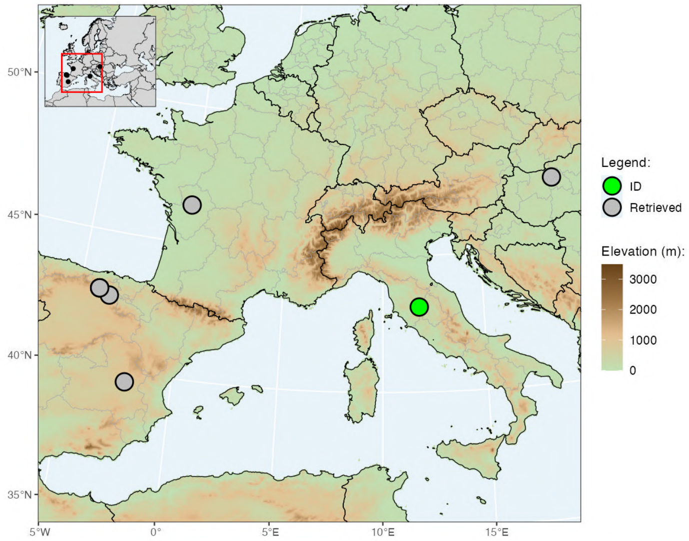

Figure 795: Genetic diversity map of *Tetramorium indocile* Santschi, 1927. Nearby localities of sequenced specimens are merged in pies (left). Colours match the bidimensional colour space of the PCoA projection (Fig. 795 left) of p-dist between sequences (dots). Specimen identification (ID or cf.) and source (newly sequenced or retrieved) are represented by colours, while specimen attribute (terra typica, type locality, type specimen or faunistic novelty) is represented by the shape (right). Sequences: ID = 8, cf. = 0; maximum p-distance: strict = NA, less strict = 4 %.

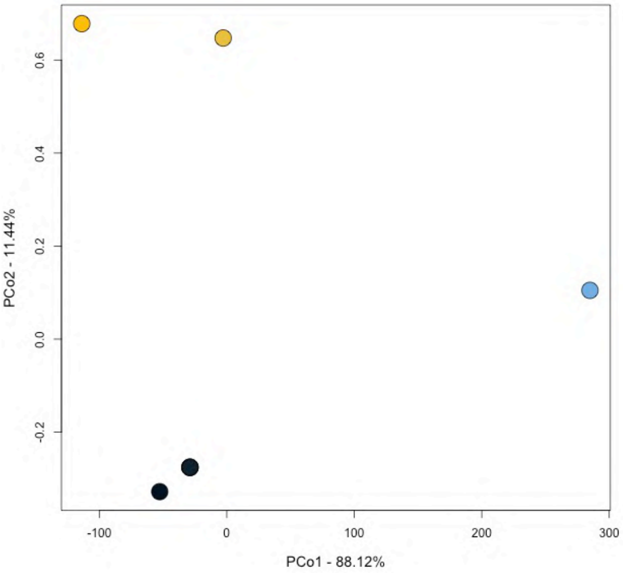

Figure 796: PCoA based on pairwise p-distances between *Tetramorium indocile* sequences (left). Colours match a bidimensional colour space. Haplotype network analysis of *Tetramorium indocile* was not possible.

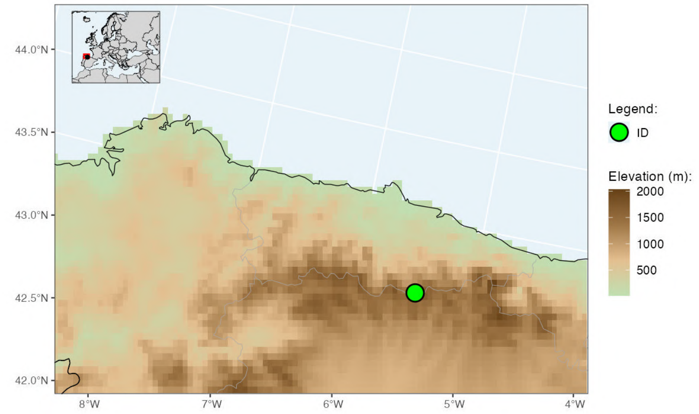

Figure 797: Map of *Tetramorium inquilinum* Ward, Brady, Fisher & Schultz, 2014. Due to the presence of a single sequence, the genetic diversity map and the PCoA projection were not done. Specimen identification (ID or cf.) and source (newly sequenced or retrieved) are represented by colours, while specimen attribute (terra typica, type locality, type specimen or faunistic novelty) is represented by the shape. Sequences: ID = 1, cf. = 0; maximum p-distance: strict = NA, less strict = NA.

Haplotype network analysis of *Tetramorium inquilinum* was not possible.

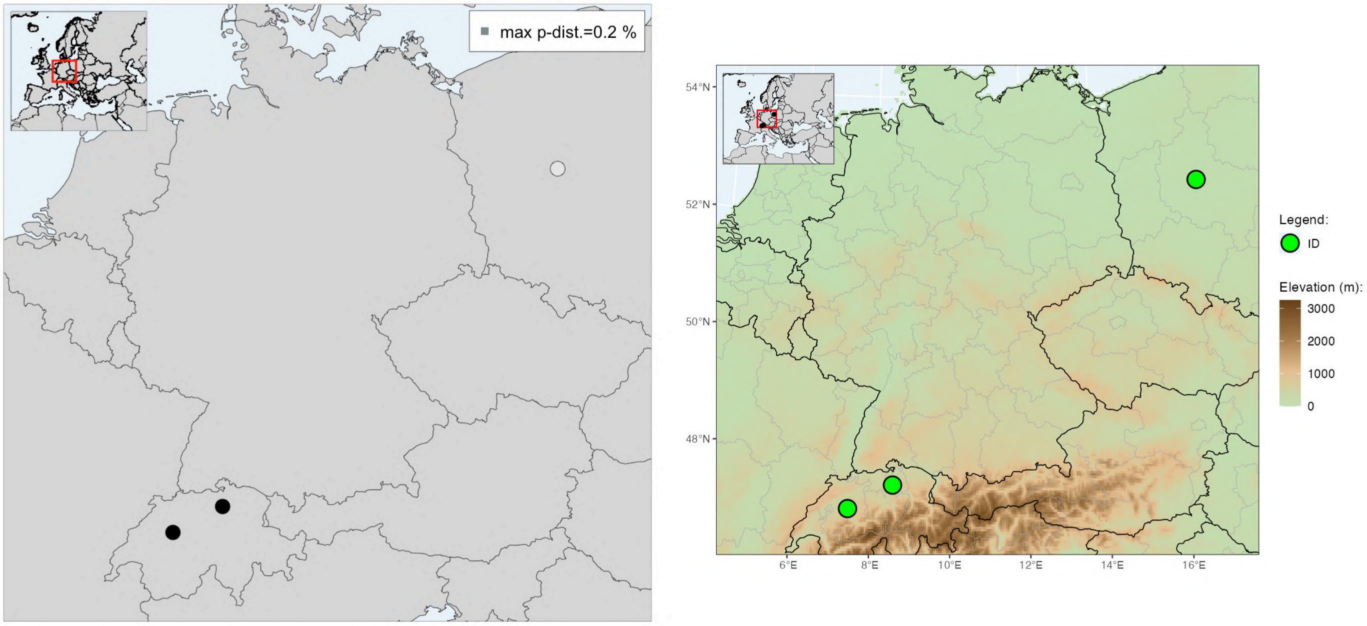

Figure 798: Genetic diversity map of *Tetramorium insolens* (Smith, F., 1861). Nearby localities of sequenced specimens are merged in pies (left). Colours match the bidimensional colour space of the PCoA projection (Fig. 798 left) of p-dist between sequences (dots). Specimen identification (ID or cf.) and source (newly sequenced or retrieved) are represented by colours, while specimen attribute (terra typica, type locality, type specimen or faunistic novelty) is represented by the shape (right). Sequences: ID = 3, cf. = 0; maximum p-distance: strict = 0.2 %, less strict = 0.2 %.

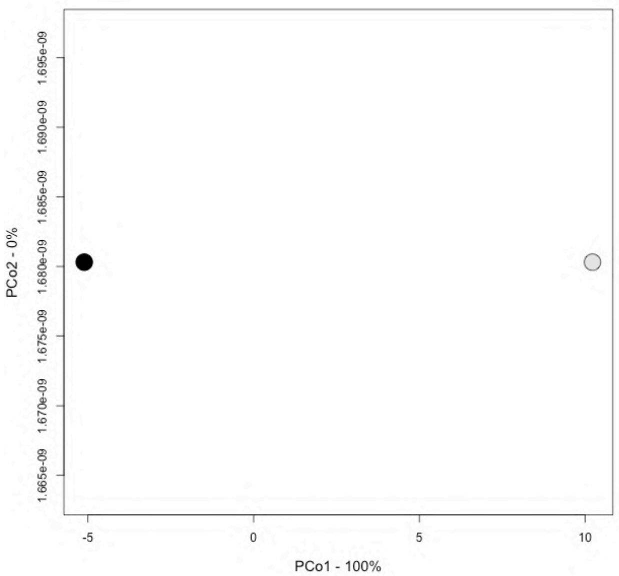

Figure 799: PCoA based on pairwise p-distances between *Tetramorium insolens* sequences (left). Colours match a bidimensional colour space. Haplotype network analysis of *Tetramorium insolens* was not possible.

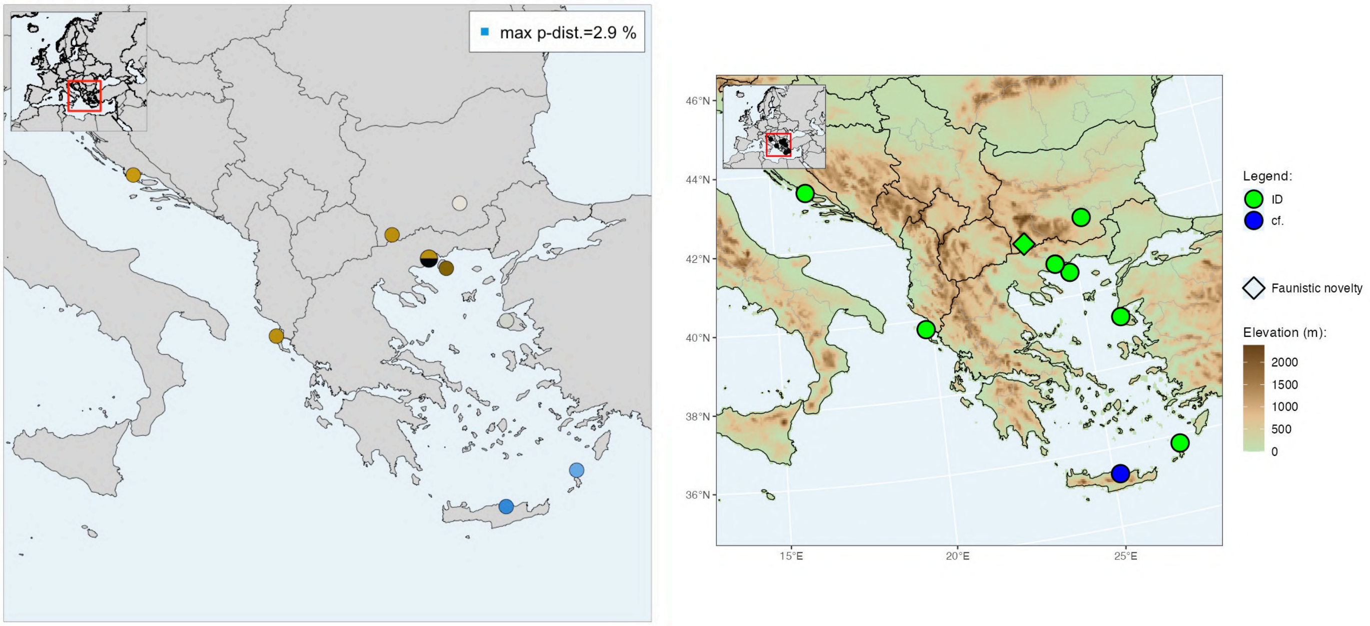

Figure 800: Genetic diversity map of *Tetramorium kephalosi* Salata & Borowiec, 2017. Nearby localities of sequenced specimens are merged in pies (left). Colours match the bidimensional colour space of the PCoA projection (Fig. 800 left) of p-dist between sequences (dots). Specimen identification (ID or cf.) and source (newly sequenced or retrieved) are represented by colours, while specimen attribute (terra typica, type locality, type specimen or faunistic novelty) is represented by the shape (right). Sequences: ID = 9, cf. = 1; maximum p-distance: strict = 2.9 %, less strict = 2.9 %.

The species is reported for the first time in Bulgaria.

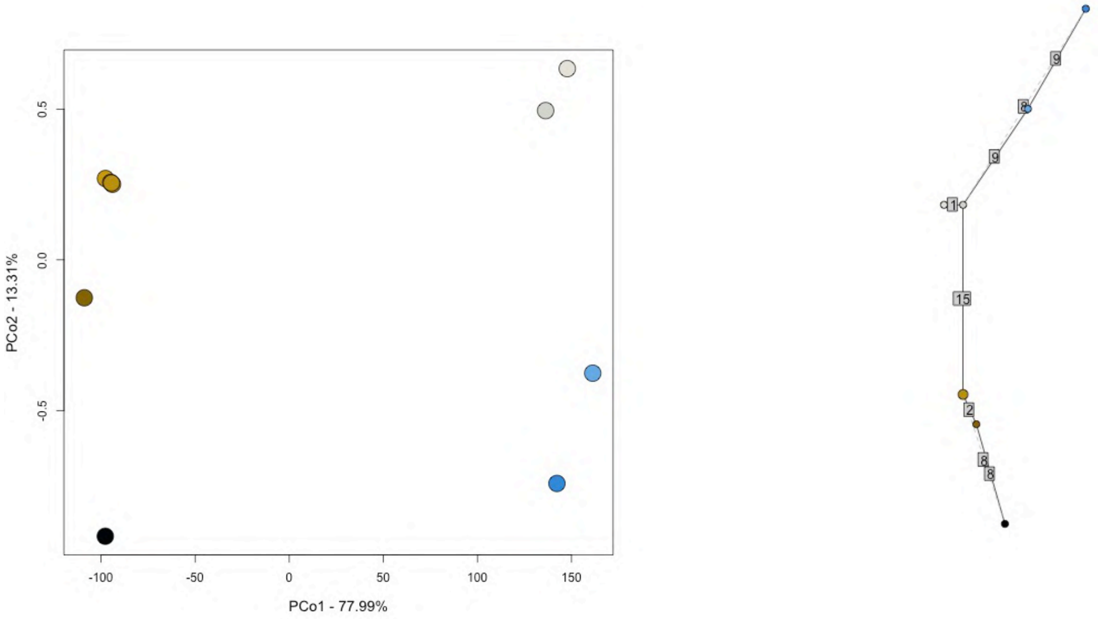

Figure 801: PCoA based on pairwise p-distances between *Tetramorium kephalosi* sequences (left). Colours match a bidimensional colour space. Haplotype network of *Tetramorium kephalosi* (right). Sequences > 599 bp: ID = 9, cf. = 1.

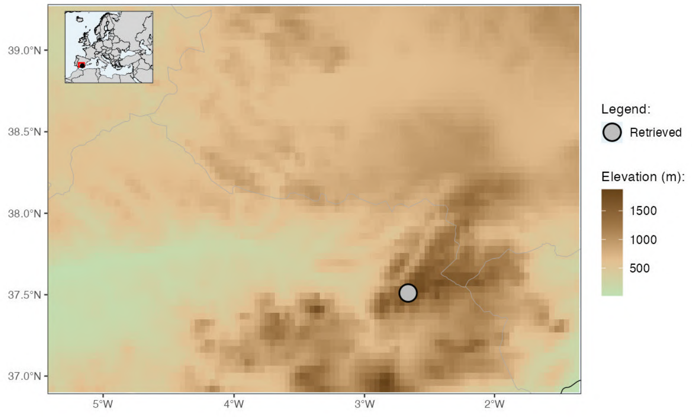

Figure 802: Map of *Tetramorium kutteri* (Tinaut, 1990). Due to the presence of a single sequence, the genetic diversity map and the PCoA projection were not done. Specimen identification (ID or cf.) and source (newly sequenced or retrieved) are represented by colours, while specimen attribute (terra typica, type locality, type specimen or faunistic novelty) is represented by the shape. Sequences: ID = 1, cf. = 0; maximum p-distance: strict = NA, less strict = NA.

Haplotype network analysis of *Tetramorium kutteri* was not possible.

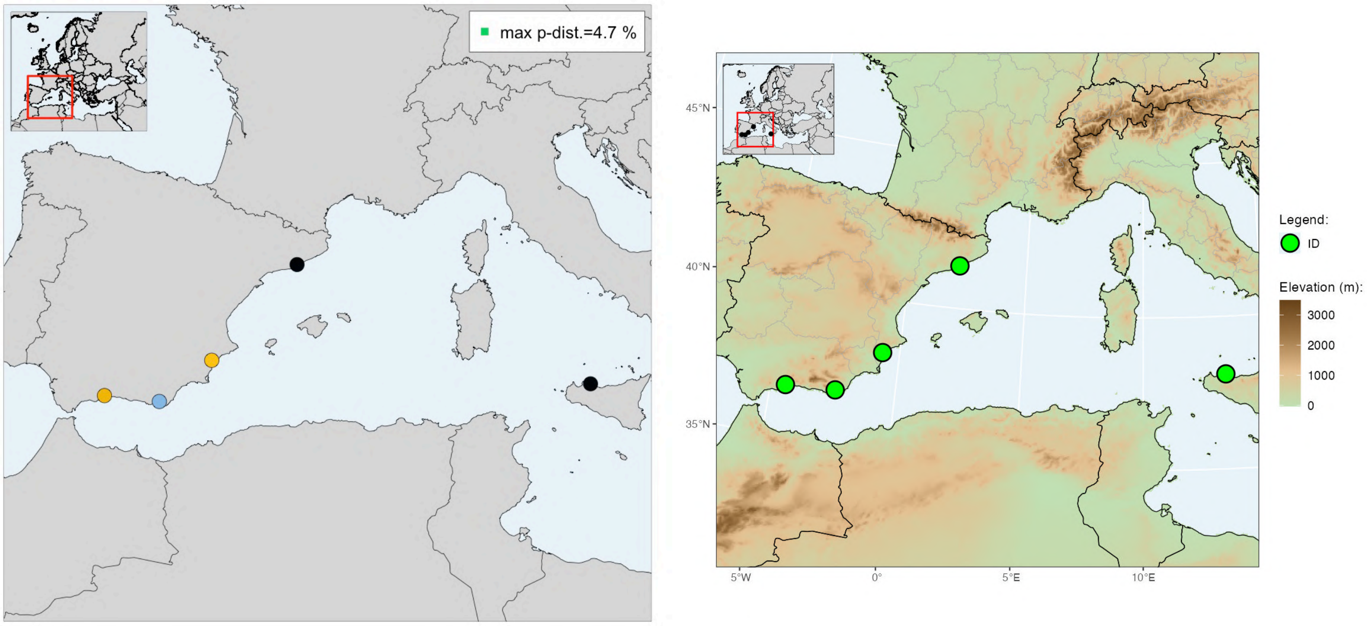

Figure 803: Genetic diversity map of *Tetramorium lanuginosum* Mayr, 1870. Nearby localities of sequenced specimens are merged in pies (left). Colours match the bidimensional colour space of the PCoA projection (Fig. 803 left) of p-dist between sequences (dots). Specimen identification (ID or cf.) and source (newly sequenced or retrieved) are represented by colours, while specimen attribute (terra typica, type locality, type specimen or faunistic novelty) is represented by the shape (right). Sequences: ID = 7, cf. = 0; maximum p-distance: strict = 4.7 %, less strict = 4.7 %.

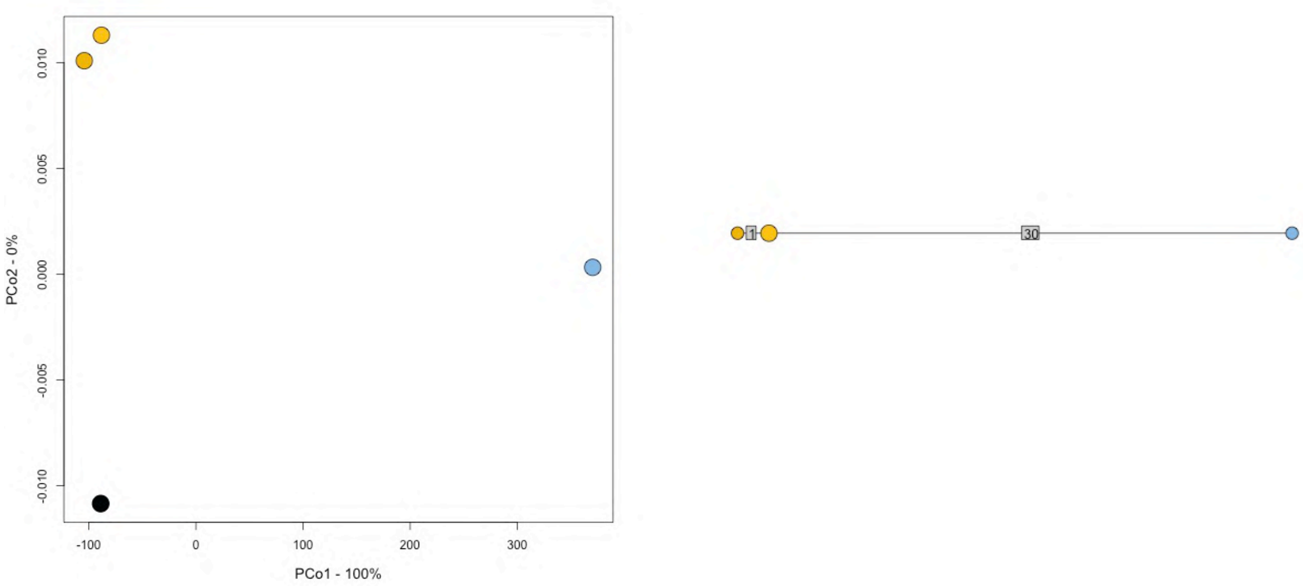

Figure 804: PCoA based on pairwise p-distances between *Tetramorium lanuginosum* sequences (left). Colours match a bidimensional colour space. Haplotype network of *Tetramorium lanuginosum* (right). Sequences > 599 bp: ID = 7, cf. = 0.

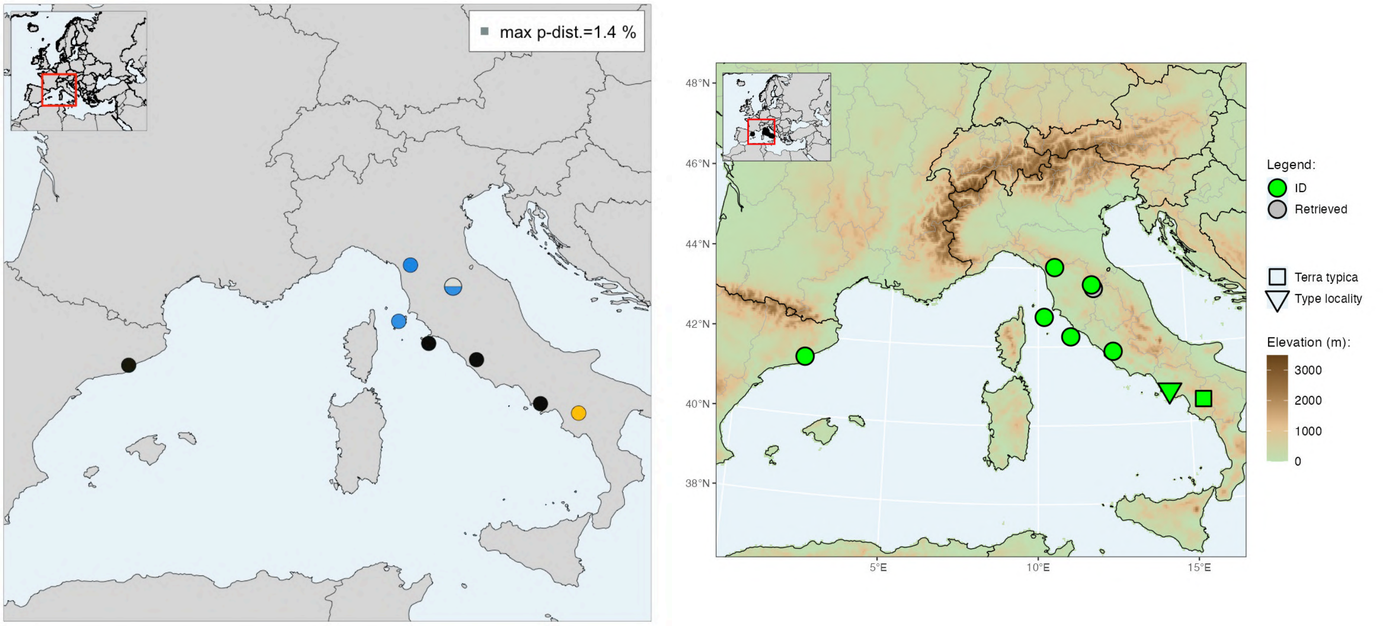

Figure 805: Genetic diversity map of *Tetramorium meridionale* Emery, 1870. Nearby localities of sequenced specimens are merged in pies (left). Colours match the bidimensional colour space of the PCoA projection (Fig. 805 left) of p-dist between sequences (dots). Specimen identification (ID or cf.) and source (newly sequenced or retrieved) are represented by colours, while specimen attribute (terra typica, type locality, type specimen or faunistic novelty) is represented by the shape (right). Sequences: ID = 9, cf. = 0; maximum p-distance: strict = 1.4 %, less strict = 1.4 %.

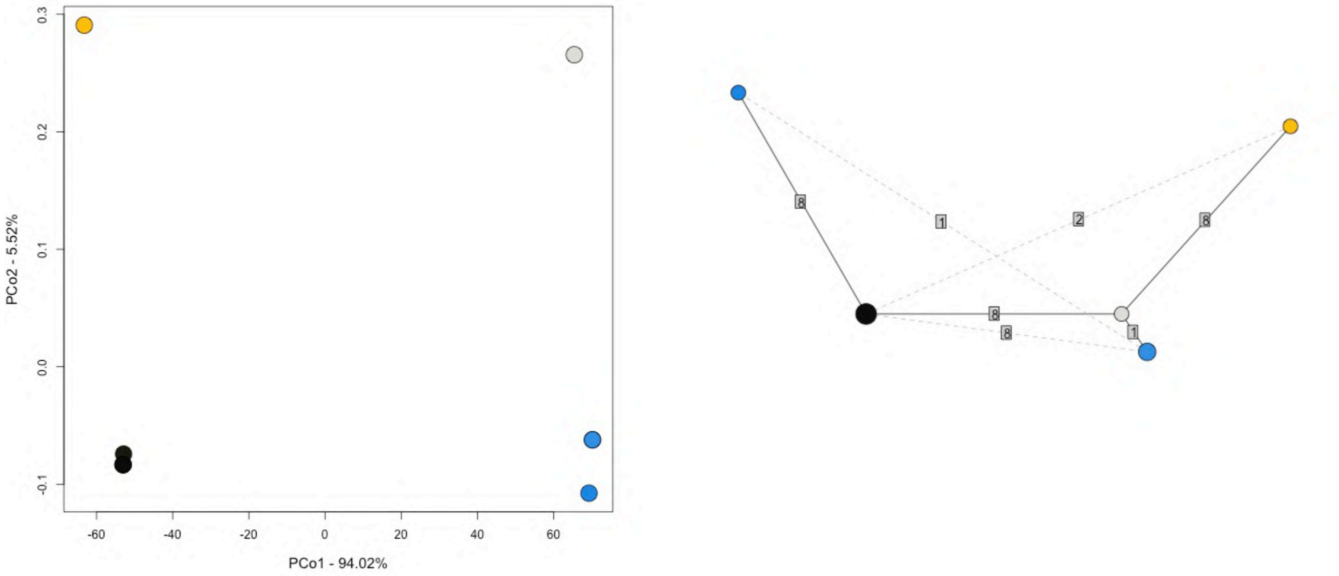

Figure 806: PCoA based on pairwise p-distances between *Tetramorium meridionale* sequences (left). Colours match a bidimensional colour space. Haplotype network of *Tetramorium meridionale* (right). Sequences > 599 bp: ID = 9, cf. = 0.

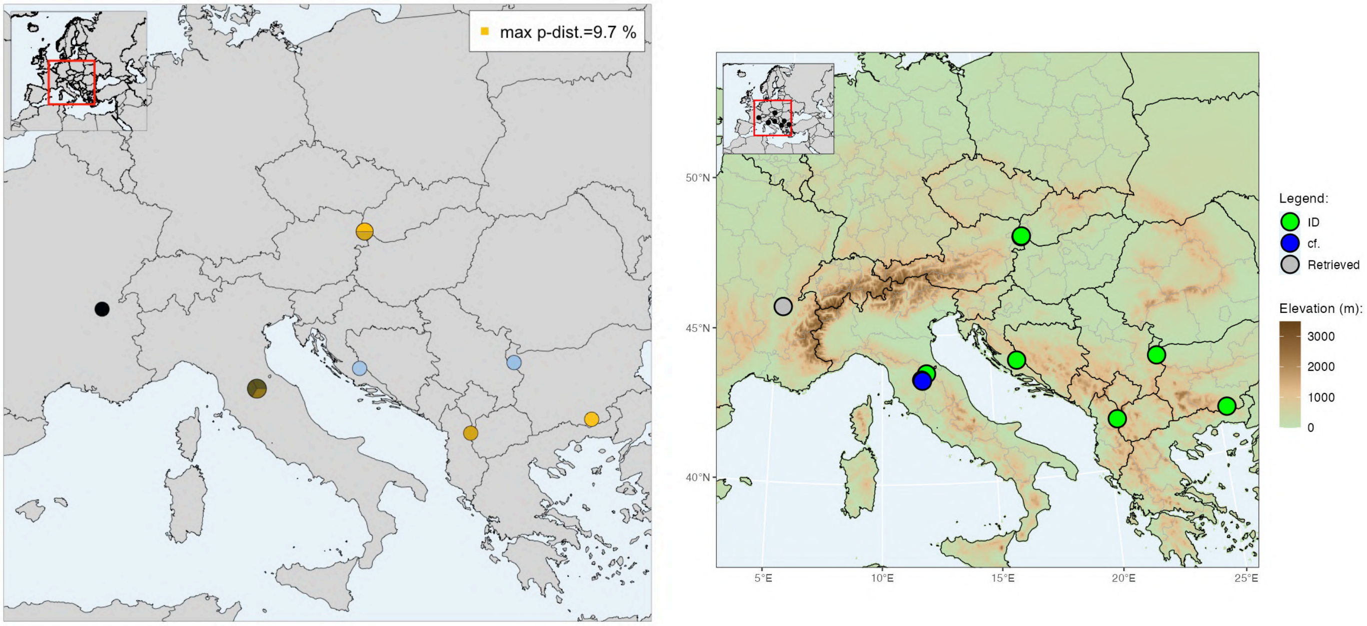

Figure 807: Genetic diversity map of *Tetramorium moravicum* Kratochvíl, 1941. Nearby localities of sequenced specimens are merged in pies (left). Colours match the bidimensional colour space of the PCoA projection (Fig. 807 left) of p-dist between sequences (dots). Specimen identification (ID or cf.) and source (newly sequenced or retrieved) are represented by colours, while specimen attribute (terra typica, type locality, type specimen or faunistic novelty) is represented by the shape (right). Sequences: ID = 9, cf. = 1; maximum p-distance: strict = 8.9 %, less strict = 9.7 %.

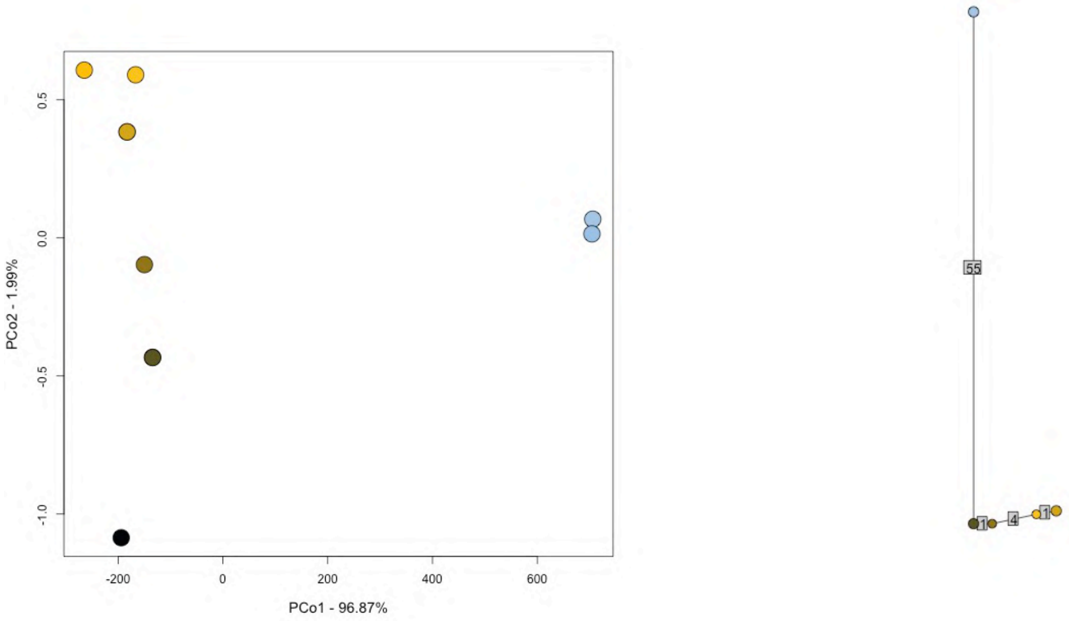

Figure 808: PCoA based on pairwise p-distances between *Tetramorium moravicum* sequences (left). Colours match a bidimensional colour space. Haplotype network of *Tetramorium moravicum* (right). Sequences > 599 bp: ID = 7, cf. = 1.

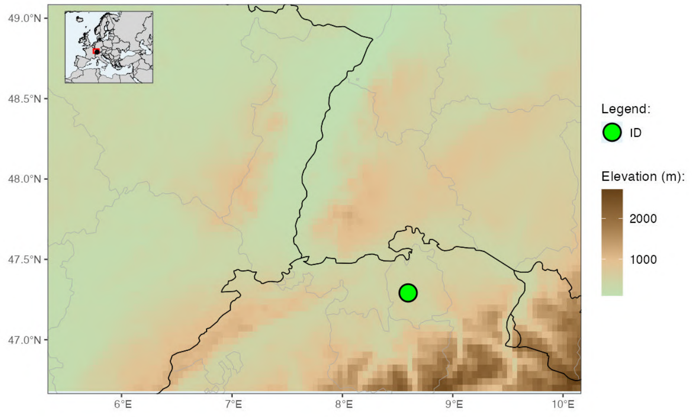

Figure 809: Map of *Tetramorium pacificum* Mayr, 1870. Due to the presence of a single sequence, the genetic diversity map and the PCoA projection were not done. Specimen identification (ID or cf.) and source (newly sequenced or retrieved) are represented by colours, while specimen attribute (terra typica, type locality, type specimen or faunistic novelty) is represented by the shape. Sequences: ID = 1, cf. = 0; maximum p-distance: strict = NA, less strict = NA.

Haplotype network analysis of *Tetramorium pacificum* was not possible.

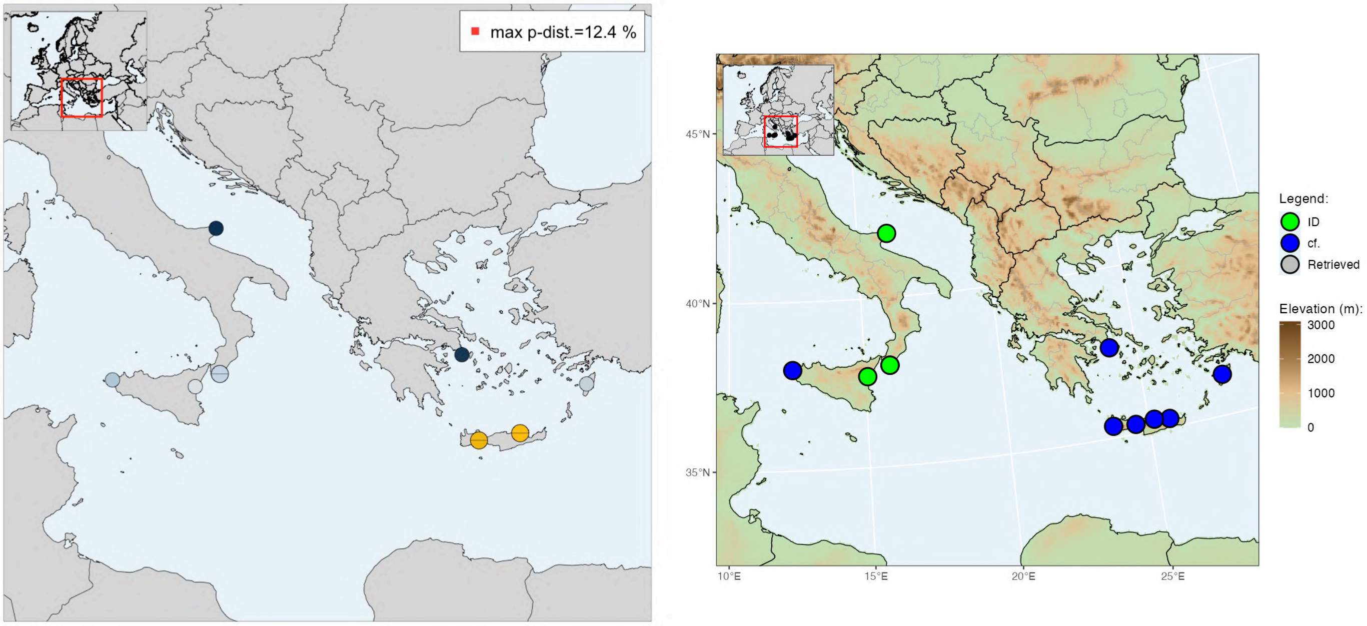

Figure 810: Genetic diversity map of *Tetramorium punctatum* Santschi, 1927. Nearby localities of sequenced specimens are merged in pies (left). Colours match the bidimensional colour space of the PCoA projection (Fig. 810 left) of p-dist between sequences (dots). Specimen identification (ID or cf.) and source (newly sequenced or retrieved) are represented by colours, while specimen attribute (terra typica, type locality, type specimen or faunistic novelty) is represented by the shape (right). Sequences: ID = 4, cf. = 7; maximum p-distance: strict = 10.9 %, less strict = 12.4 %.

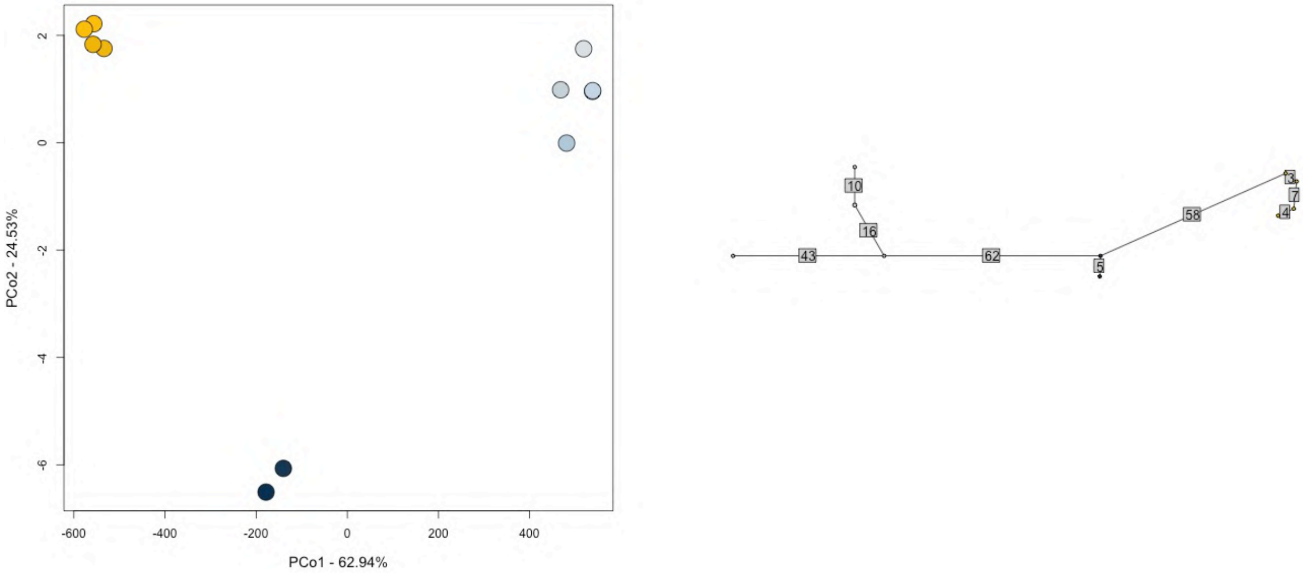

Figure 811: PCoA based on pairwise p-distances between *Tetramorium punctatum* sequences (left). Colours match a bidimensional colour space. Haplotype network of *Tetramorium punctatum* (right). Sequences > 599 bp: ID = 4, cf. = 7.

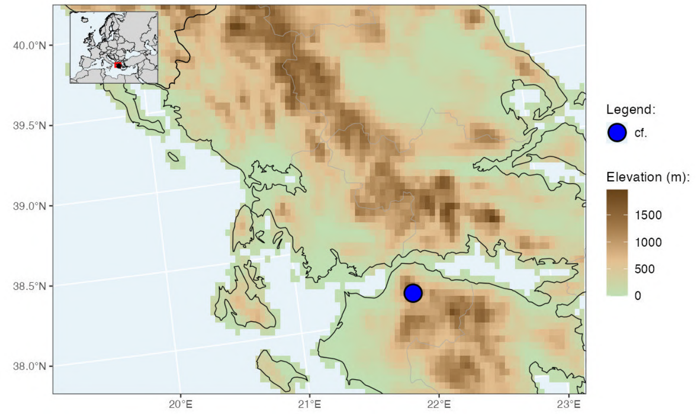

Figure 812: Map of *Tetramorium cf. punicum* (Smith, F., 1861). Due to the presence of a single sequence, the genetic diversity map and the PCoA projection were not done. Specimen identification (ID or cf.) and source (newly sequenced or retrieved) are represented by colours, while specimen attribute (terra typica, type locality, type specimen or faunistic novelty) is represented by the shape. Sequences: ID = 0, cf. = 1; maximum p-distance: strict = NA, less strict = NA.

Haplotype network analysis of *Tetramorium cf. punicum* was not possible.

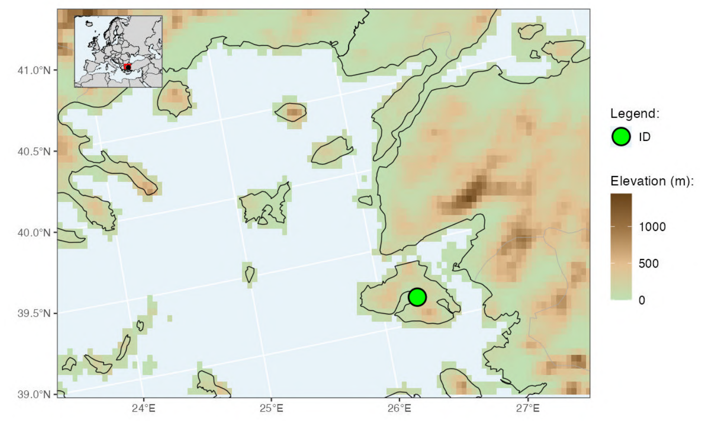

Figure 813: Map of *Tetramorium rhodium* Emery, 1924. Due to the presence of a single sequence, the genetic diversity map and the PCoA projection were not done. Specimen identification (ID or cf.) and source (newly sequenced or retrieved) are represented by colours, while specimen attribute (terra typica, type locality, type specimen or faunistic novelty) is represented by the shape. Sequences: ID = 1, cf. = 0; maximum p-distance: strict = NA, less strict = NA.

Haplotype network analysis of *Tetramorium rhodium* was not possible.

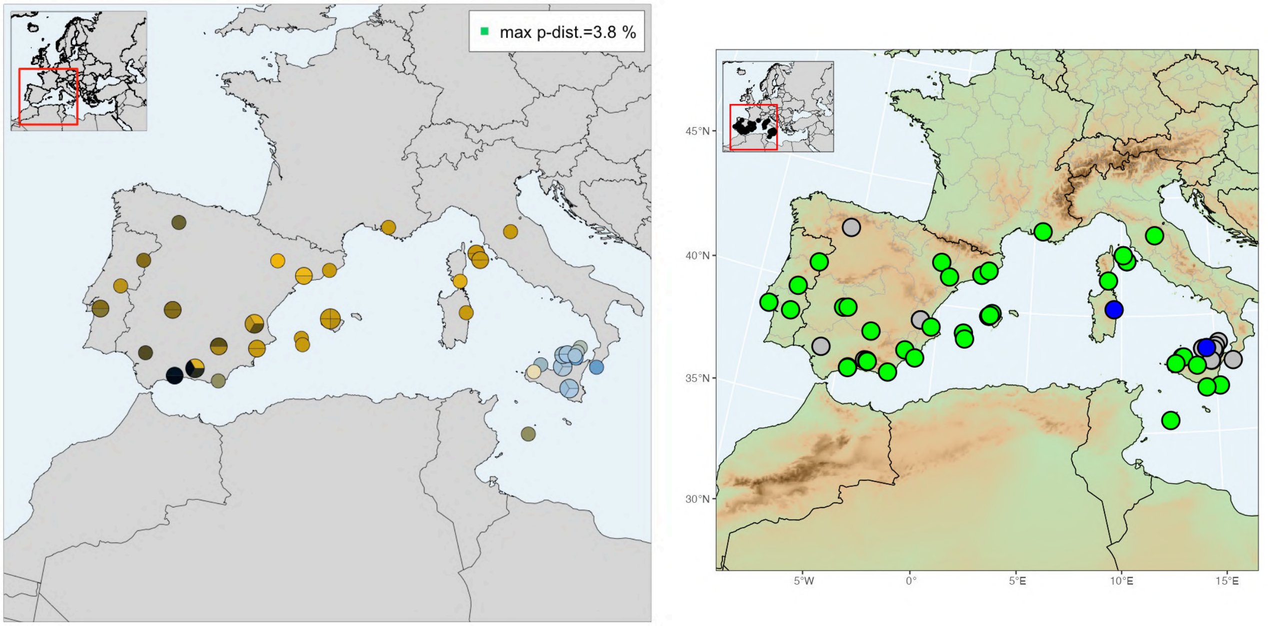

Figure 814: Genetic diversity map of *Tetramorium semilaeve* André, 1883. Nearby localities of sequenced specimens are merged in pies (left). Colours match the bidimensional colour space of the PCoA projection (Fig. 814 left) of p-dist between sequences (dots). Specimen identification (ID or cf.) and source (newly sequenced or retrieved) are represented by colours, while specimen attribute (terra typica, type locality, type specimen or faunistic novelty) is represented by the shape (right). Sequences: ID = 55, cf. = 2; maximum p-distance: strict = 3.8 %, less strict = 3.8 %.

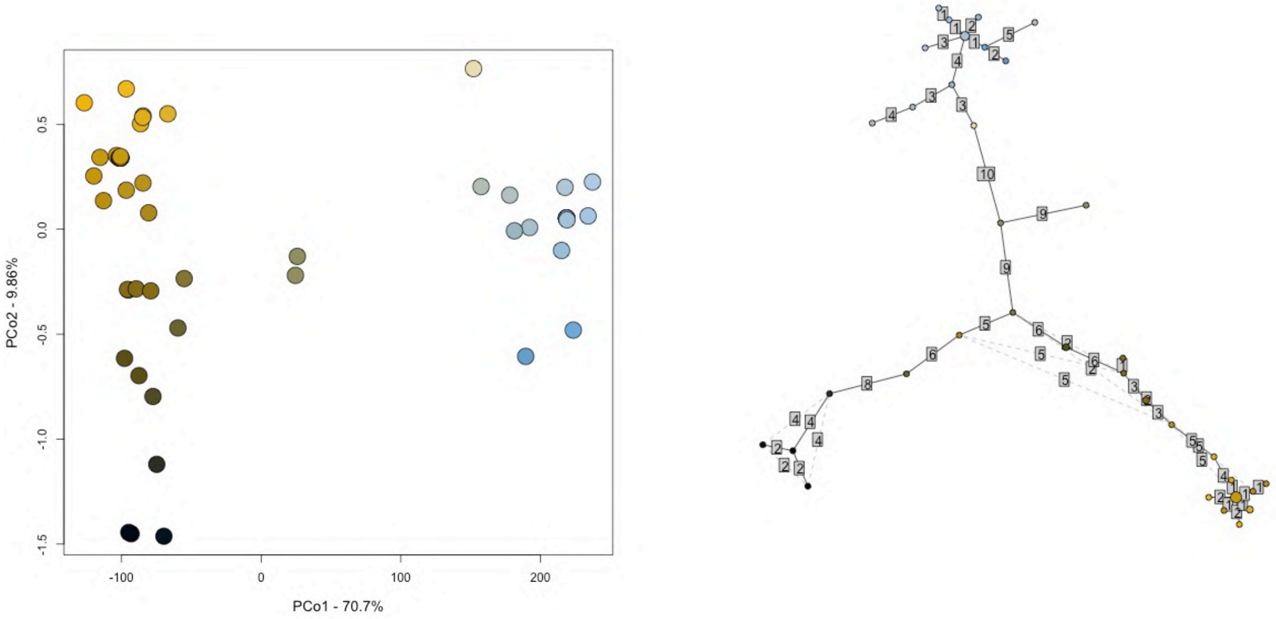

Figure 815: PCoA based on pairwise p-distances between *Tetramorium semilaeve* sequences (left). Colours match a bidimensional colour space. Haplotype network of *Tetramorium semilaeve* (right). Sequences > 599 bp: ID = 54, cf. = 2.

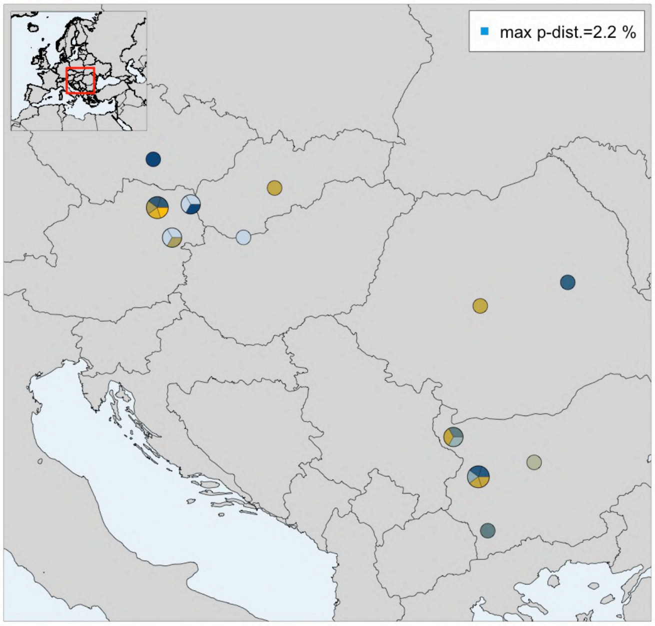

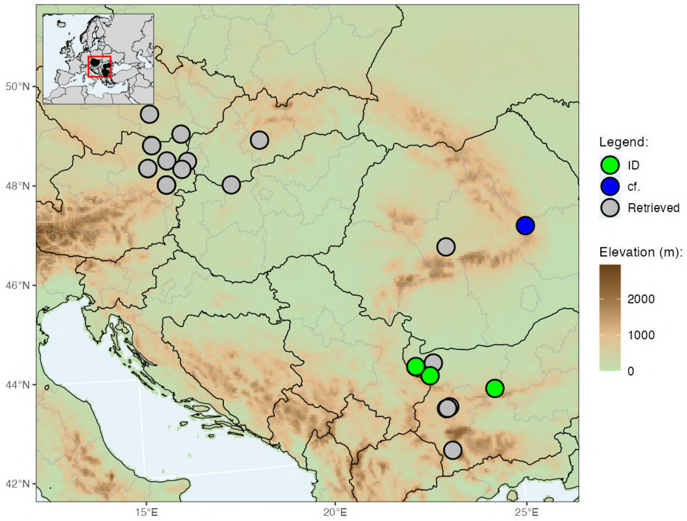

Figure 816: Genetic diversity map of *Tetramorium staerckei* Kratochvíl, 1944. Nearby localities of sequenced specimens are merged in pies (left). Colours match the bidimensional colour space of the PCoA projection (Fig. 816 left) of p-dist between sequences (dots). Specimen identification (ID or cf.) and source (newly sequenced or retrieved) are represented by colours, while specimen attribute (terra typica, type locality, type specimen or faunistic novelty) is represented by the shape (right). Sequences: ID = 25, cf. = 1; maximum p-distance: strict = 1.1 %, less strict = 2.2 %.

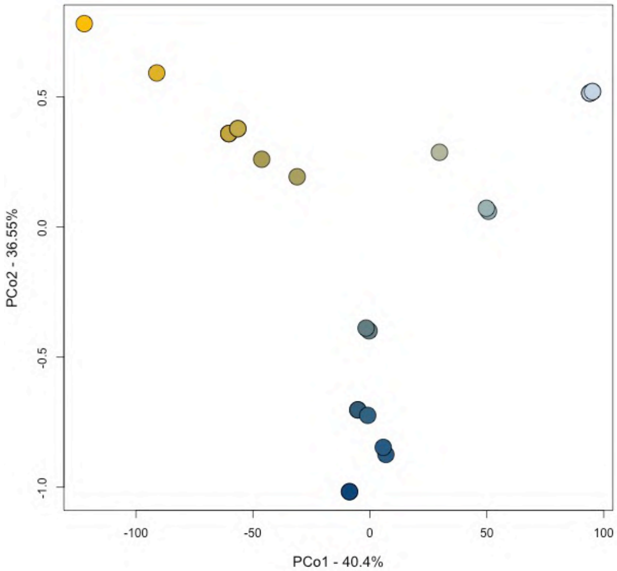

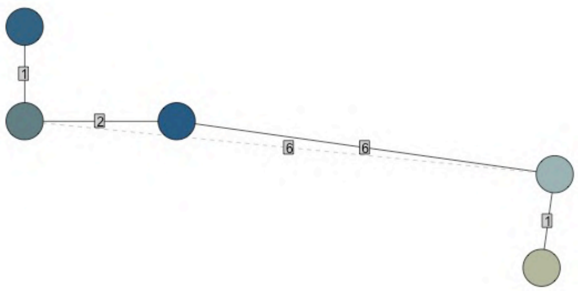

Figure 817: PCoA based on pairwise p-distances between *Tetramorium staerckei* sequences (left). Colours match a bidimensional colour space. Haplotype network of *Tetramorium staerckei* (right). Sequences > 599 bp: ID = 4, cf. = 1.

Trichomyrmex

Trichomyrmex perplexus

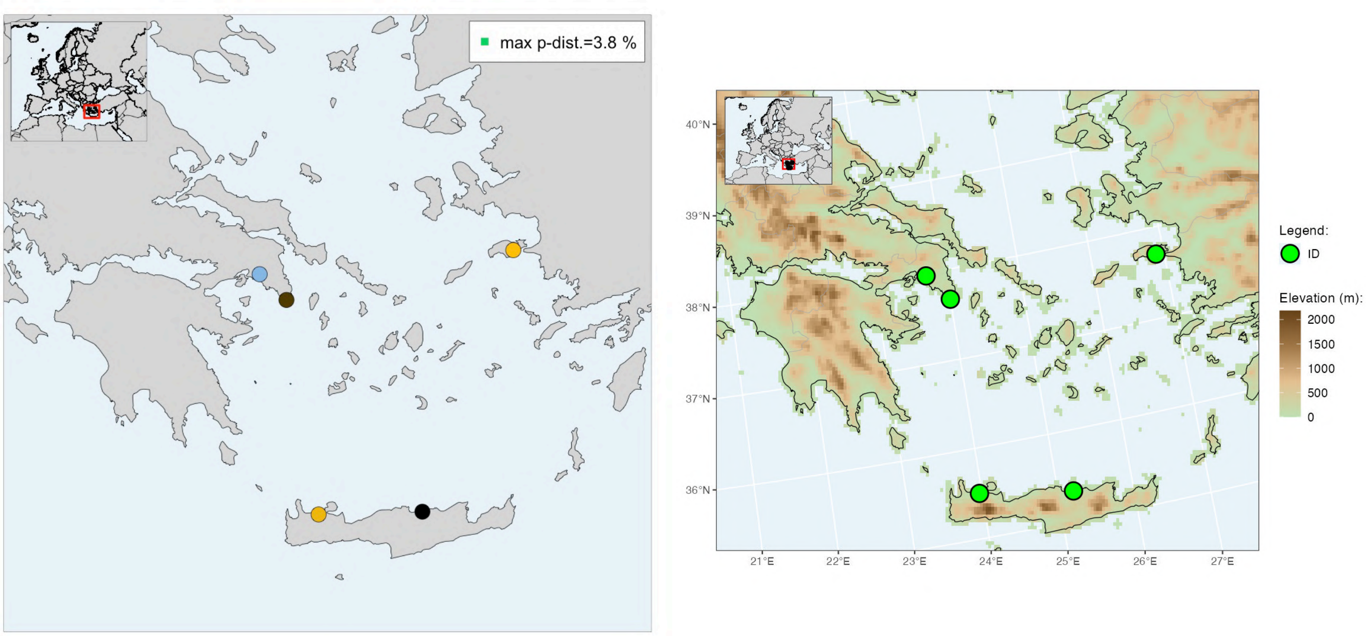

Figure 818: Genetic diversity map of *Trichomyrmex perplexus* (Radchenko, 1997). Nearby localities of sequenced specimens are merged in pies (left). Colours match the bidimensional colour space of the PCoA projection (Fig. 818 left) of p-dist between sequences (dots). Specimen identification (ID or cf.) and source (newly sequenced or retrieved) are represented by colours, while specimen attribute (terra typica, type locality, type specimen or faunistic novelty) is represented by the shape (right). Sequences: ID = 5, cf. = 0; maximum p-distance: strict = 3.8 %, less strict = 3.8 %.

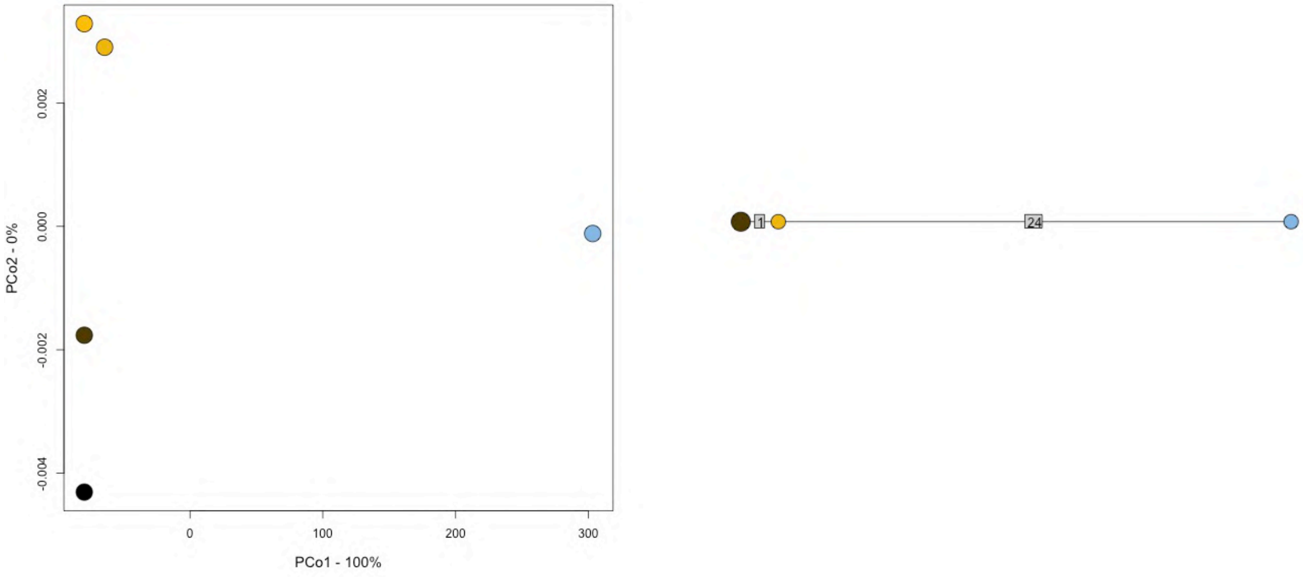

Figure 819: PCoA based on pairwise p-distances between *Trichomyrmex perplexus* sequences (left). Colours match a bidimensional colour space. Haplotype network of *Trichomyrmex perplexus* (right). Sequences > 599 bp: ID = 5, cf. = 0.

Wasmannia

Wasmannia auropunctata

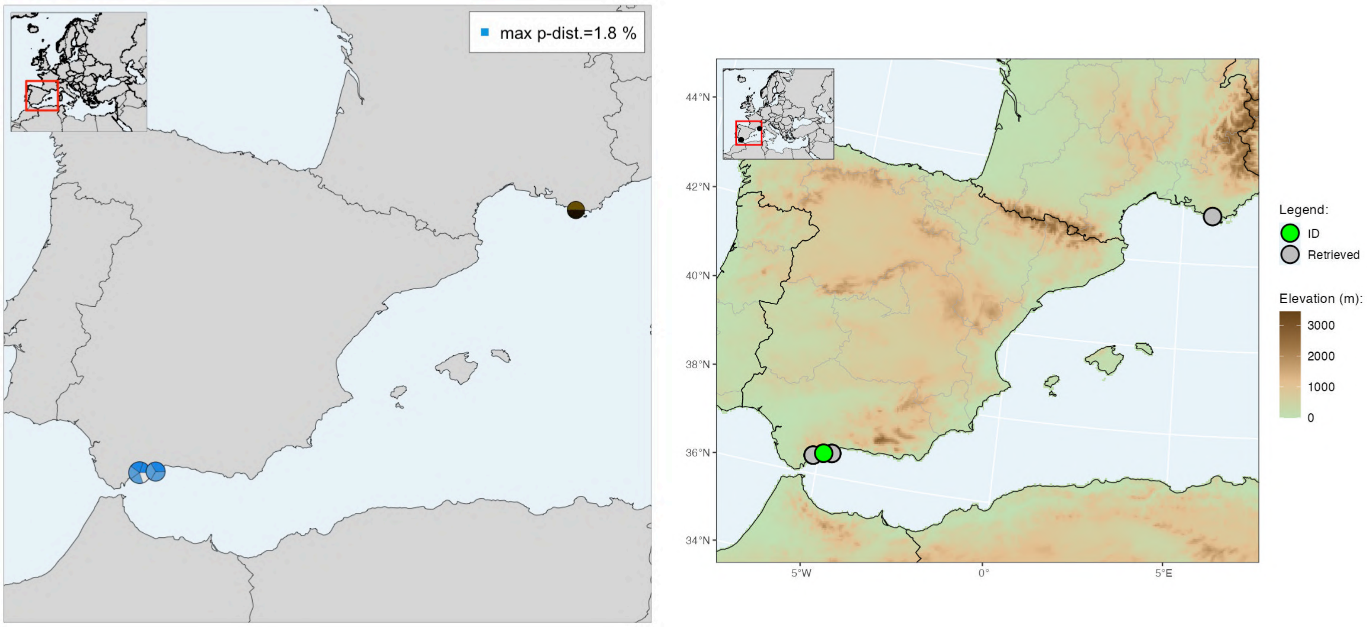

Figure 820: Genetic diversity map of *Wasmannia auropunctata* (Roger, 1863). Nearby localities of sequenced specimens are merged in pies (left). Colours match the bidimensional colour space of the PCoA projection (Fig. 820 left) of p-dist between sequences (dots). Specimen identification (ID or cf.) and source (newly sequenced or retrieved) are represented by colours, while specimen attribute (terra typica, type locality, type specimen or faunistic novelty) is represented by the shape (right). Sequences: ID = 12, cf. = 0; maximum p-distance: strict = 1.8 %, less strict = 1.8 %.

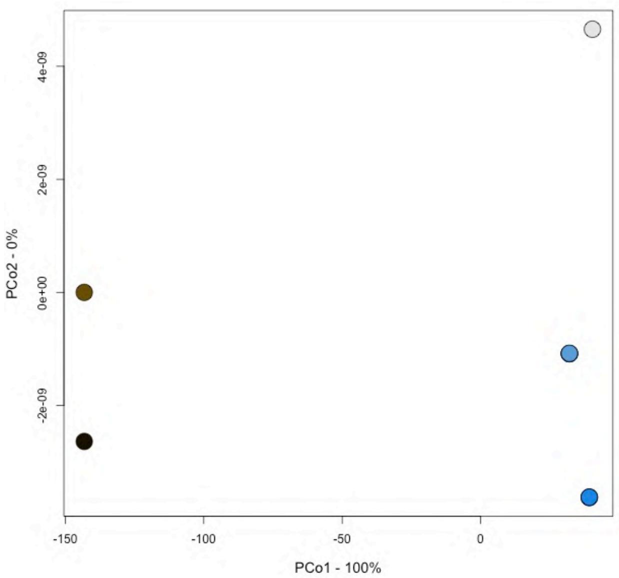

Figure 821: PCoA based on pairwise p-distances between *Wasmannia auropunctata* sequences (left). Colours match a bidimensional colour space. Haplotype network analysis of *Wasmannia auropunctata* was not possible.
