# Supplementary material for: DNA Barcode Reference Library for European Ants: A Roadmap for Phylogeography and Species Discovery
Source: Mol Ecol Resour. 2026 Apr 20;26(3):e70135. doi: 10.1111/1755-0998.70135 (PMC13093865; doi:10.1111/1755-0998.70135)
Supplement: Supplementary file 1 — Data S1: supinfo/men70135‐sup‐0001‐Supinfo.zip. [file MEN-26-e70135-s001.zip › men70135-sup-0001-Supinfo/Supporting Information 2.pdf]

Supplemental Information 2

Menchetti et al. DNA barcode reference library for European ants: a roadmap for phylogeography and species discovery

Table of Contents

Figure S1 . . . 2
Figure S2 . . . 2
Figure S3 . . . 3
Figure S4 . . . 4
Figure S5 . . . 4
Figure S6 . . . 5
Table S1 . . . 6

Figure S1

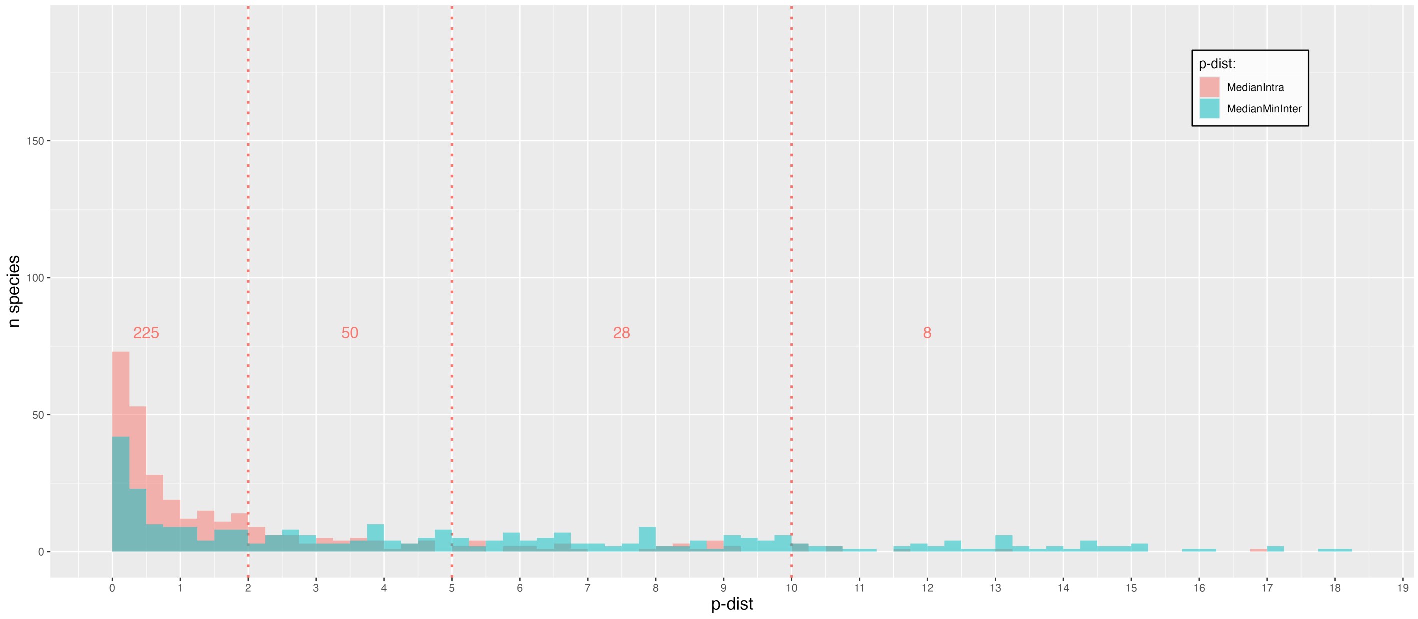

Figure S1: Frequency of pairwise distances (in % substitutions per site, grouped in ranges of 0.25 %) illustrating the distribution of median intraspecific (red) and the median minimum interspecific genetic (blue) values among congeneric species (n = 296 spp.). Vertical dashed lines delimit the thresholds of median intraspecific p-dist of 2% (conservative estimate of intraspecific variation), 5% (general cutoff for distinguishing between intra- and interspecific variation), and 10% (high level of intraspecific variation), while the numbers on top indicate the number of species falling within each cutoff.

Figure S2

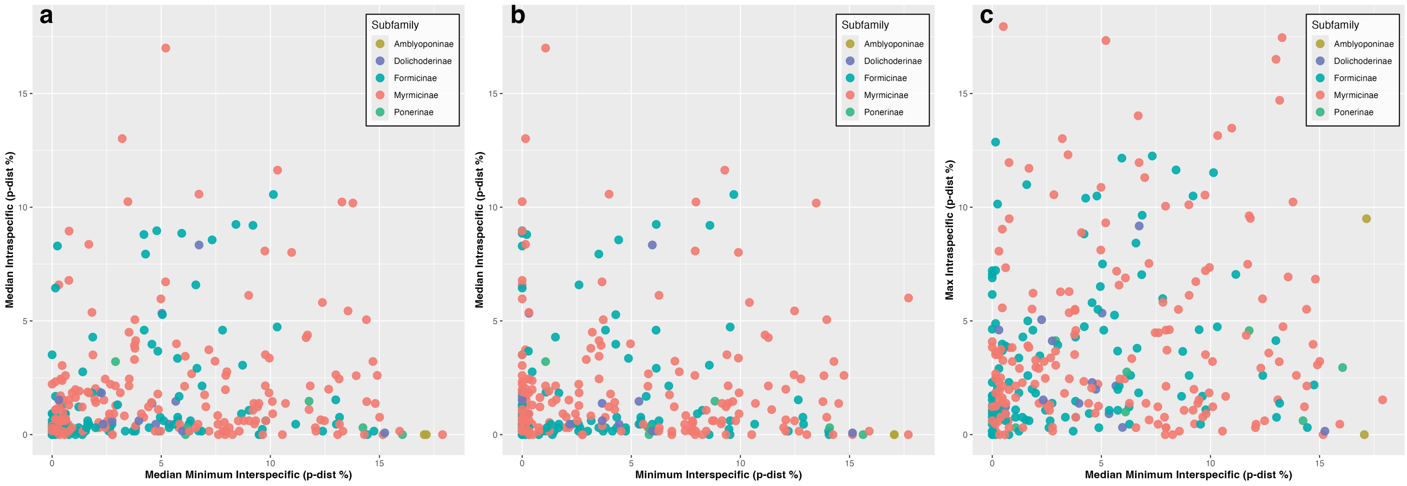

Figure S2: Graphs summarising the p- distances values (%) obtained for each species of median intraspecific p-dist vs the median of minimum interspecific p-dist **(a)**, the median intraspecific vs minimum intraspecific **(b)**, maximum intraspecific vs the median of minimum interspecific **(c)**. Colours represent subfamilies.

Figure S3

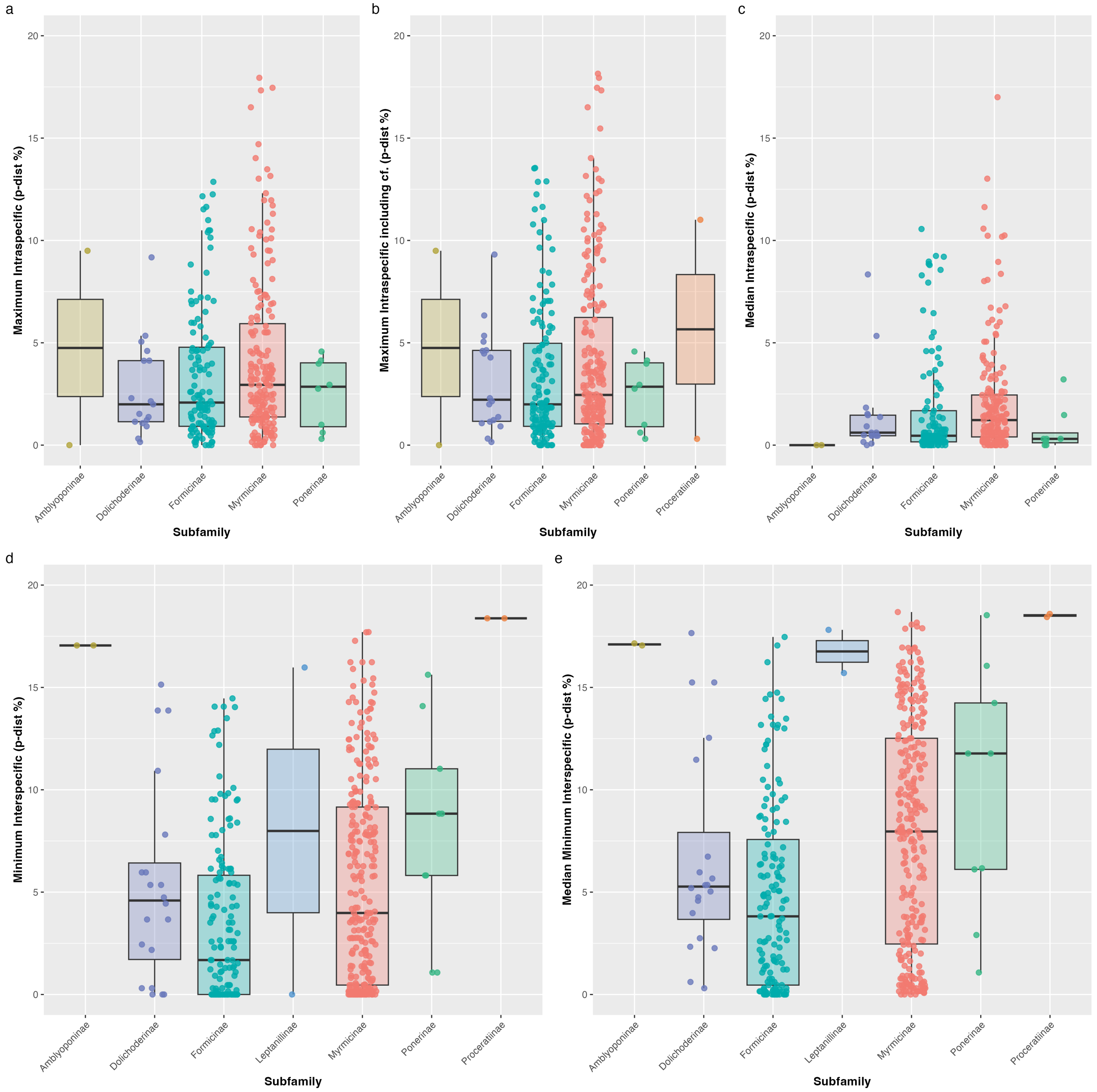

Figure S3: Distribution in each subfamily of the maximum intraspecific (a), maximum intraspecific including specimens identified as cf. and >1 specimens per species (b), median intraspecific (c), minimum interspecific (d), and median of the minimum interspecific (e) p-distance values (%).

Figure S4

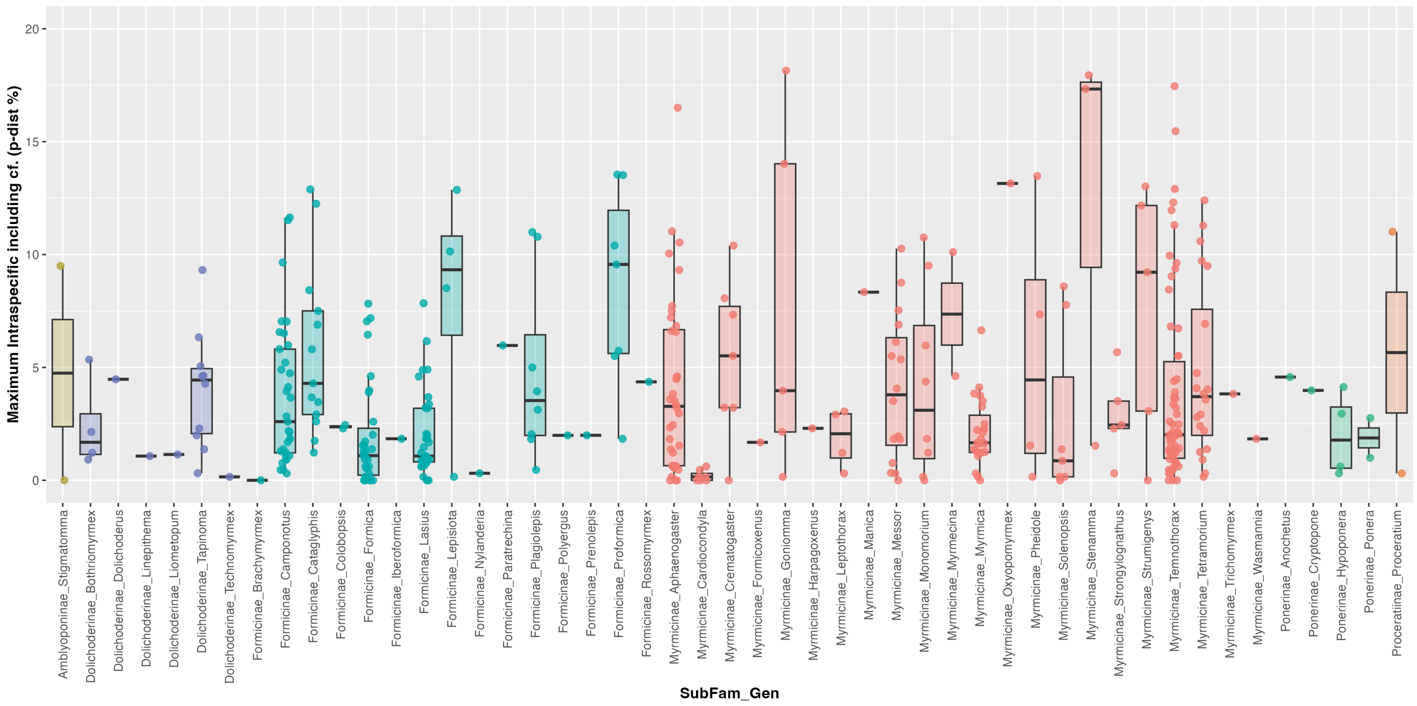

Figure S4: Box plot of the maximum intraspecific p-distance values (%), including specimens identified as cf. and >1 specimens per species, grouped by genus.

Figure S5

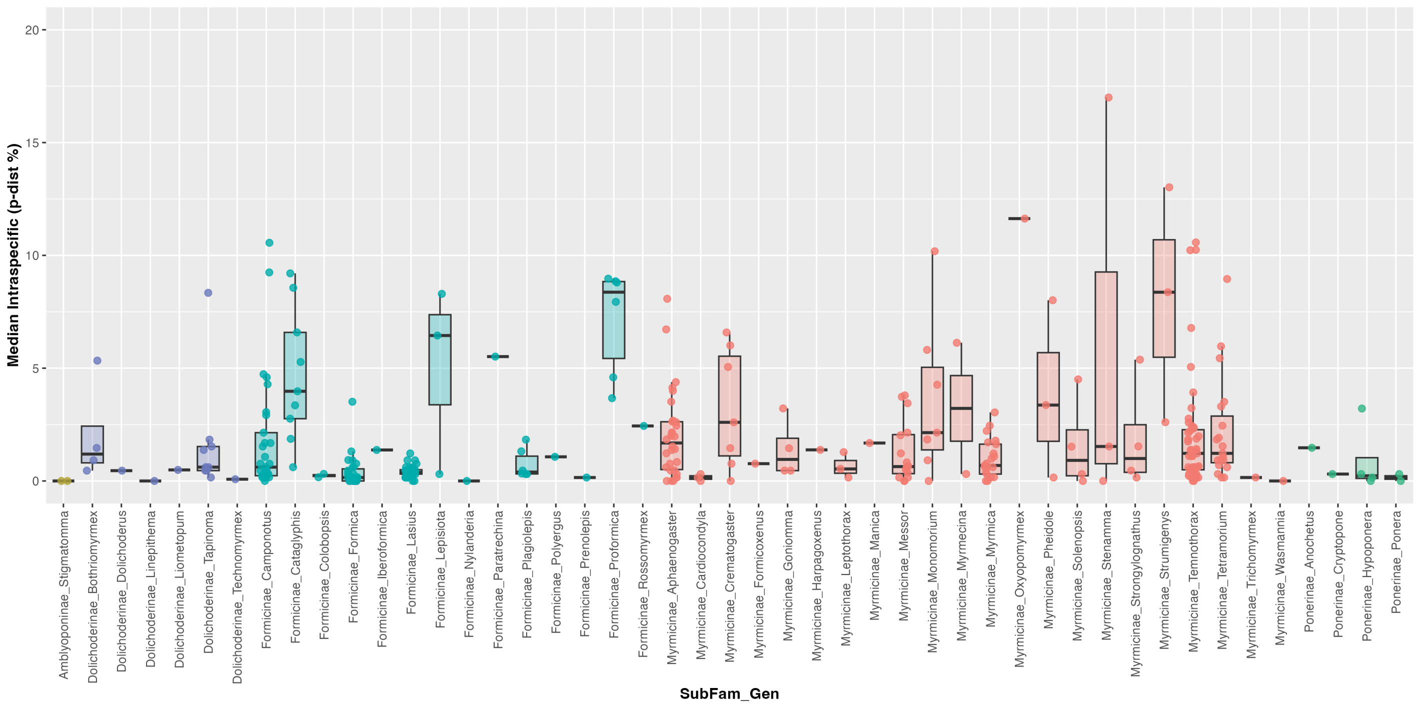

Figure S5: Box plot of the median intraspecific p-distance values (%) grouped by genus.

Figure S6

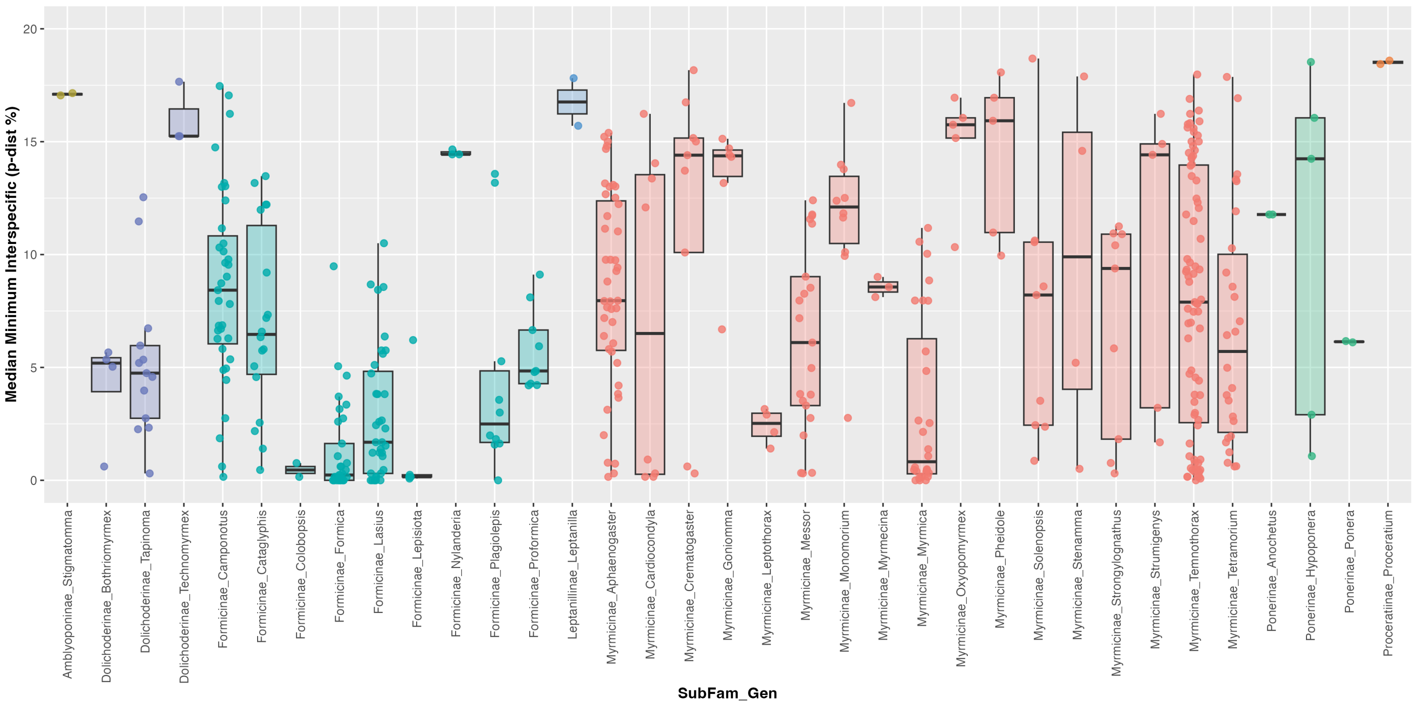

Figure S6: Box plot of the median of the minimum interspecific p-distance values (%) grouped by genus.

Table S1

Table S1: Best-fit substitution models selected with ModelFinder (Kalyaanamoorthy et al. 2017) for each genus-level phylogenetic dataset.

Tree	AIC	CAIC	BIC	Best_fit
Acropyga	TIM2+F+I	TIM2+F+I	TIM2+F+I	TIM2+F+I chosen according to BIC
Aenictus	TVM+F+I	TVM+F+I	K3Pu+F+I	K3Pu+F+I chosen according to BIC
Anochetus	TVM+F+G4	TVM+F+G4	K3Pu+F+G4	K3Pu+F+G4 chosen according to BIC
Aphaenogaster__Messor	TPM2+F+R5	JC+I+G4	TPM2+F+R5	TPM2+F+R5 chosen according to BIC
Bothriomyrmex	TIM2+F+G4	TIM2+F+G4	TPM2+F+G4	TPM2+F+G4 chosen according to BIC
Brachymyrmex	GTR+F+I	GTR+F+I	TPM2+F+I	TPM2+F+I chosen according to BIC
Brachyponera	GTR+F+I	GTR+F+I	TIM2+F+I	TIM2+F+I chosen according to BIC
Camponotus	TPM2u+F+R8	JC+I+G4	TPM2+F+R7	TPM2+F+R7 chosen according to BIC
Cardiocondyla	GTR+F+I+G4	GTR+F+I+G4	GTR+F+G4	GTR+F+G4 chosen according to BIC
Carebara	TIM2+F+I	TIM2+F+I	TIM2+F+I	TIM2+F+I chosen according to BIC
Cataglyphis	GTR+F+R7	TIM2+F+I+G4	TIM2+F+I+G4	TIM2+F+I+G4 chosen according to BIC
Colobopsis	TIM2+F+I	TIM2+F+I	TPM2+F+I	TPM2+F+I chosen according to BIC
Crematogaster	TPM2+F+I+G4	TPM2+F+I+G4	TPM2+F+I+G4	TPM2+F+I+G4 chosen according to BIC
Cryptopone	GTR+F+I	GTR+F+I	TIM2+F+I	TIM2+F+I chosen according to BIC
Dolichoderus	TVM+F+I	TPM2+F+I	TPM2+F+I	TPM2+F+I chosen according to BIC
Formica	TPM2+F+R4	JC+I+G4	HKY+F+R4	HKY+F+R4 chosen according to BIC
Formicoxenus	TIM2+F+I	TIM2+F+I	TIM2+F+I	TIM2+F+I chosen according to BIC
Goniomma__Oxyopomyrmex	GTR+F+R5	TIM2+F+I+G4	TIM2+F+I+G4	TIM2+F+I+G4 chosen according to BIC
Harpagoxenus	TIM2+F+I	TIM2+F+I	TIM2+F+I	TIM2+F+I chosen according to BIC
Hypoponera	GTR+F+I	GTR+F+I	TIM2+F+I	TIM2+F+I chosen according to BIC
Iberoformica	TN+F+I	TN+F+I	TN+F+I	TN+F+I chosen according to BIC
Lasius	GTR+F+R5	JC+I+G4	TPM2+F+I+G4	TPM2+F+I+G4 chosen according to BIC
Lepisiota	TIM2+F+G4	TIM2+F+G4	TIM2+F+G4	TIM2+F+G4 chosen according to BIC
Leptanilla	GTR+F+I+G4	GTR+F+I+G4	TIM2+F+I+G4	TIM2+F+I+G4 chosen according to BIC
Leptothorax	TIM3+F+I	TN+F+I	TN+F+I	TN+F+I chosen according to BIC
Linepithema	GTR+F+I	GTR+F+I	TIM2+F+I	TIM2+F+I chosen according to BIC
Liometopum	GTR+F+G4	GTR+F+G4	TPM2+F+G4	TPM2+F+G4 chosen according to BIC
Manica	K3Pu+F+G4	K3Pu+F+G4	HKY+F+G4	HKY+F+G4 chosen according to BIC
Metalasius	GTR+F+I	GTR+F+I	GTR+F+I	GTR+F+I chosen according to BIC
Monomorium	TIM2+F+I+G4	TIM2+F+I+G4	TIM2+F+I+G4	TIM2+F+I+G4 chosen according to BIC
Myrmecina	TIM2+F+I+G4	TIM2+F+I+G4	TN+F+G4	TN+F+G4 chosen according to BIC
Myrmica	TVM+F+I+G4	JC+I+G4	K3Pu+F+I+G4	K3Pu+F+I+G4 chosen according to BIC
Nylanderia	GTR+F+G4	GTR+F+G4	TIM+F+G4	TIM+F+G4 chosen according to BIC
Paratrechina	TVM+F+R2	TVM+F+R2	K3Pu+F+G4	K3Pu+F+G4 chosen according to BIC
Pheidole	TIM2+F+I+G4	TIM2+F+I+G4	TIM2+F+I+G4	TIM2+F+I+G4 chosen according to BIC
Plagiolepis	GTR+F+R4	TPM2+F+G4	TPM2+F+G4	TPM2+F+G4 chosen according to BIC
Polyergus	GTR+F+I	GTR+F+I	TIM+F+I	TIM+F+I chosen according to BIC
Ponera	TIM2+F+I	TIM2+F+I	TIM2+F+I	TIM2+F+I chosen according to BIC
Prenolepis	GTR+F+I	TIM+F+I	K3Pu+F+I	K3Pu+F+I chosen according to BIC
Proceratium	TIM2+F+G4	TIM2+F+G4	TIM2+F+G4	TIM2+F+G4 chosen according to BIC
Proformica	TIM2+F+R4	TPM2+F+R4	TPM2+F+R4	TPM2+F+R4 chosen according to BIC
Rossomyrmex	TIM2+F+G4	TIM2+F+G4	TPM2+F+G4	TPM2+F+G4 chosen according to BIC
Solenopsis	GTR+F+I+G4	TIM2+F+I+G4	TPM2+F+I+G4	TPM2+F+I+G4 chosen according to BIC
Stenamma	TIM2+F+I+G4	TIM2+F+I+G4	TIM2+F+I+G4	TIM2+F+I+G4 chosen according to BIC
Stigmatomma	GTR+F+I	GTR+F+I	TIM2+F+I	TIM2+F+I chosen according to BIC
Strongylognathus__Tetramorium	TPM2+F+R5	JC+R4	TPM2+F+R5	TPM2+F+R5 chosen according to BIC
Strumigenys	TIM2+F+I+G4	TIM2+F+I+G4	TIM2+F+I+G4	TIM2+F+I+G4 chosen according to BIC
Tapinoma	TPM2+F+R4	TPM2+F+R3	TPM2+F+R4	TPM2+F+R4 chosen according to BIC
Technomyrmex	GTR+F+I+G4	GTR+F+I+G4	TIM2+F+G4	TIM2+F+G4 chosen according to BIC
Temnothorax	TIM2+F+R6	GTR+F+G4	TIM2+F+R6	TIM2+F+R6 chosen according to BIC
Trichomyrmex	TIM2+F+G4	TIM2+F+G4	TIM2+F+G4	TIM2+F+G4 chosen according to BIC
Wasmannia	TIM2+F+I	TIM2+F+I	TIM2+F+I	TIM2+F+I chosen according to BIC
